# Re-examination of nepovirus polyprotein cleavage sites highlights the diverse specificities and evolutionary relationships of nepovirus 3C-like proteases

**DOI:** 10.1007/s00705-022-05564-x

**Published:** 2022-08-30

**Authors:** Hélène Sanfaçon

**Affiliations:** Summerland Research and Development Centre, Agriculture and Agri-Food Canada, 4200 Highway 97, V0H1Z0 Summerland, BC Canada

## Abstract

Plant-infecting viruses of the genus *Nepovirus* (subfamily *Comovirinae*, family *Secoviridae*, order *Picornavirales*) are bipartite positive-strand RNA viruses with each genomic RNA encoding a single large polyprotein. The RNA1-encoded 3C-like protease cleaves the RNA1 polyprotein at five sites and the RNA2 polyprotein at two or three sites, depending on the nepovirus. The specificity of nepovirus 3C-like proteases is notoriously diverse, making the prediction of cleavage sites difficult. In this study, the position of nepovirus cleavage sites was systematically re-evaluated using alignments of the RNA1 and RNA2 polyproteins, phylogenetic relationships of the proteases, and sequence logos to examine specific preferences for the P6 to P1’ positions of the cleavage sites. Based on these analyses, the positions of previously elusive cleavage sites, notably the 2a-MP cleavage sites of subgroup B nepoviruses, are now proposed. Distinct nepovirus protease clades were identified, each with different cleavage site specificities, mostly determined by the nature of the amino acid at the P1 and P1’ positions of the cleavage sites, as well as the P2 and P4 positions. The results will assist the prediction of cleavage sites for new nepoviruses and help refine the taxonomy of nepoviruses. An improved understanding of the specificity of nepovirus 3C-like proteases can also be used to investigate the cleavage of plant proteins by nepovirus proteases and to understand their adaptation to a broad range of hosts.

**Supplementary Information:**

The online version contains supplementary material available at 10.1007/s00705-022-05564-x.

## Introduction

Many viruses encode endoproteases that cleave viral polyproteins at specific cleavage sites and regulate the release of mature viral proteins and intermediate polyprotein precursors [44, 53, 61]. The archetype picornavirus 3C protease (3C-Pro) is thought to have ancient origins. Indeed, 3C-Pro probably evolved from an early symbiont membrane protease similar to the bacterial and mitochondrial HTrA serine proteases [42]. 3C-like proteases (3CL-Pros) are found in a wide variety of picorna-like viruses infecting hosts that range from algae, fungi, plants, insects, and mammals. Thus, 3CL-Pros have a long history of adaptation to virus infection cycles in a wide range of hosts [32, 53]. Plant viruses encoding 3CL-Pros include members of the families *Secoviridae* and *Potyviridae* [53, 81] as well as a proposed new family of plant picornaviruses exemplified by rice curl dwarf associated picornavirus [86]. Although very diverse in their primary sequence, all 3CL-Pros share a structural fold similar to that of chymotrypsin (a serine protease) and an active site composed of a catalytic triad that includes conserved histidine, aspartate (or glutamate), and cysteine (or occasionally serine) residues.

The specificity of 3CL-Pros is conferred by their substrate-binding pocket (SBP). Most 3CL-Pros recognize cleavage sites with a glutamine at the P1 position (immediately upstream of the scissile bond), in part due to a direct interaction with a conserved histidine in the SBP of the proteases [7]. The P1’ position (immediately downstream of the scissile bond) is usually occupied by a small amino acid such as glycine or serine. Thus, Q/G or Q/S (or alternatively E/G or E/S) cleavage sites are the most common. The specificity of 3CL-Pros is also directed by the nature of other amino acids around the cleavage sites, ranging from the P2 to P6 positions (2 to 6 amino acids upstream of the scissile bond), most commonly at the P2 or P4 positions [3, 11].

**Fig. 1:**
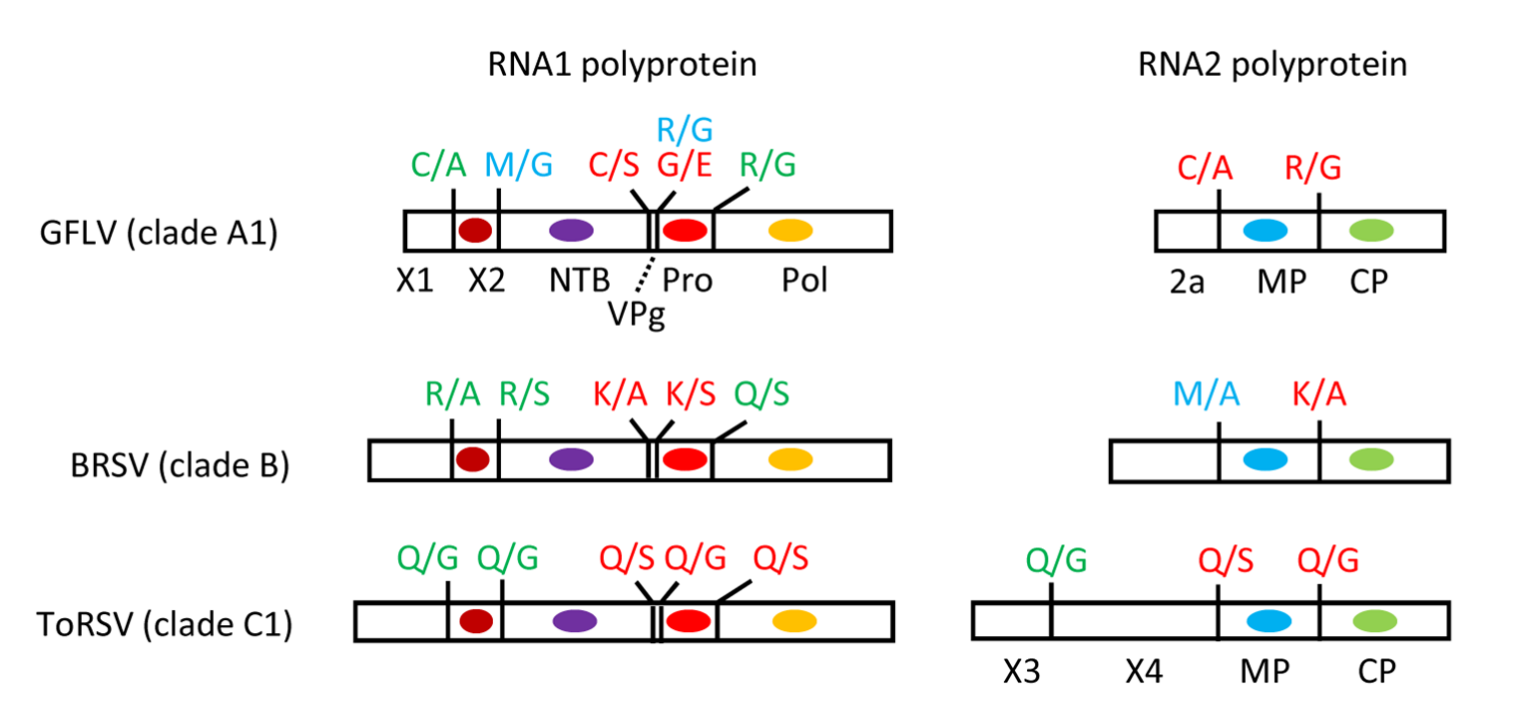
Genomic organization of representative nepoviruses. The RNA1 and RNA2 polyproteins are shown with conserved domains indicated (brown circles, hydrophobic sequences constituting the transmembrane domain of the X2 protein; purple ovals, NTP-binding motif in the NTB protein; red ovals, 3CL-Pro; yellow ovals, RNA-dependent RNA polymerase, Pol; blue ovals, movement protein, MP; green ovals, coat protein, CP). Cleavage sites are shown by vertical bars along with the sequence of the cleaved dipeptide. Experimentally confirmed cleavage sites are shown in red, confidently predicted cleavage sites in green, and tentatively predicted cleavage sites in blue. GFLV, grapevine fanleaf virus; BRSV, beet ringspot virus; ToRSV, tomato ringspot virus. For the GFLV Pro-Pol cleavage site, two possible adjacent dipeptides are shown: the G/E cleavage site (experimentally confirmed by C-terminal digestion of the cleaved product) and an alternative putative R/G cleavage site.

The genus *Nepovirus* was first recognized as the “nepovirus group” in 1971 [80] and is currently classified in the subfamily *Comovirinae*, family *Secoviridae*, order *Picornavirales* [71]. There are 46 recognized nepovirus species, 11 of which are not associated with significant genome sequence information (see https://talk.ictvonline.org/taxonomy/ for the latest taxonomy release of the International Committee for the Taxonomy of Viruses, ICTV). Nepoviruses have a bipartite genome, with each RNA encoding a single large polyprotein [65]. The RNA1 and RNA2 polyproteins are both cleaved by the RNA1-encoded 3CL-Pro. The RNA1 polyprotein contains six protein domains: the X1 protein of unknown function; the X2 protein, a trans-membrane protein probably associated with the viral replication complex; the NTP-binding protein (NTB), a putative helicase and a membrane anchor for the replication complex; the genome-linked VPg protein, a probable primer for viral RNA replication; the 3CL-Pro and the RNA-dependent RNA polymerase (Pol) [29] (Fig. 1). The RNA2 polyprotein contains either three or four protein domains (Fig. 1). The C-terminal region of the RNA2 polyprotein includes the domains for the movement protein (MP) and coat protein (CP). The N-terminal region of the polyprotein contains either the single domain for the 2a protein, a protein associated with the replication complex and referred to as the homing protein, or two domains for the X3 and X4 proteins of unknown function [29].

Nepoviruses branch together in phylogenetic trees based on deduced amino acid sequences of the CP or of the Pro-Pol domain, which is delineated by the catalytic cysteine (or serine) of the 3CL-Pro and the Pol GDD motif [65]. However, they also show more diversity than that observed for the other two genera (*Comovirus* and *Fabavirus*) in the subfamily *Comovirinae*. This diversity was recognized early on, and the genus *Nepovirus* was subdivided into three subgroups, based on the size of RNA2 and on immunological and phylogenetic relationships [29, 65]. However, it is becoming increasingly clear that the three subgroups do not adequately represent the diversity of nepoviruses.

Nepoviruses are notorious for the unusual and diverse specificities of their proteases [29, 53]. Indeed, it was noted already in the 1980s-1990s that the 3CL-Pros of subgroup A and B nepoviruses do not share the conserved SBP histidine, which is most often replaced by leucine. These proteases recognize atypical cleavage sites, including R/G, R/A, K/A, K/S, C/A, C/S, A/S, as confirmed by direct N-terminal sequencing (Edman degradation) of the CP or VPg proteins [8, 10, 13, 14, 24, 36, 54, 58, 67, 85]. More recently, the list of subgroup A and B nepovirus cleavage sites has expanded with an experimentally confirmed M/A cleavage site for melon mild mottle virus (MMMoV) [72]. Subgroup C nepovirus 3CL-Pros have the conserved SBP histidine, and early results with tomato ringspot virus (ToRSV) and cherry leaf roll virus (CLRV) proteases confirmed that they recognize the expected Q/G or Q/S cleavage sites [16, 17, 35, 66, 76, 77]. However, more recent results suggest that other subgroup C nepovirus 3CL-Pros recognize divergent cleavage sites in spite of the presence of the SBP histidine, with experimentally confirmed C/S, D/S and N/S cleavage sites for blueberry latent spherical virus (BLSV), blackcurrant reversion virus (BRV), and blueberry leaf mottle virus (BLMV), respectively [6, 38, 47].

Because of the diverse specificities of nepovirus 3CL-Pros, polyprotein cleavage sites are often difficult to predict. Annotations of cleavage sites in the literature or in the NCBI database are based on the nature of characterized nepovirus cleavage sites that were available at the time and are sometimes incorrect. In many other cases, cleavage sites are not annotated at all. Given the expanding diversity of confirmed nepovirus cleavage sites and the increasing number of nepovirus sequence available, a systematic re-evaluation of nepovirus polyprotein cleavage sites was undertaken, together with phylogenetic analysis of nepovirus proteases. The results highlight grouping of nepovirus proteases in clades, each sharing distinct cleavage site specificities.

## Materials and methods

Alignments of nepovirus polyproteins were generated using Clustal omega [68]. For phylogenetic studies, sequences were aligned with Clustal W, and trees were generated using the maximum-likelihood method as implemented in MEGA X, using default settings [43]. Bootstrap values for each node were calculated (1000 replicates). Sequence logos were generated using Weblogo3 [22], available at http://weblogo.threeplusone.com/.

## Results

### Retrieval of nepovirus polyprotein sequences and prediction/validation of cleavage sites

The sequence of the type isolate for each recognized nepovirus species was retrieved. Please note that new nepovirus species names that will conform to the binomial format are currently being considered by the ICTV but have not yet been formalized. To avoid unnecessary confusion, virus names (which, most of the time, correspond to the current species names) are used throughout this manuscript (see Table 1 for a list of virus abbreviations and full names).

**Table 1:** Nepovirus abbreviations, full names, and accession numbers

Abbreviation	Virus name	Pro	Size RNA2		Accession numbers	
		clade	polyprotein		RNA 1	RNA2
**Subgroup A**						
ArMV	arabis mosaic virus	A1	1100 aa		NC_006057	NC_006056
GFLV	grapevine fanleaf virus	A1	1109 aa		NC_003615	NC_003623
GDefV	grapevine deformation virus	A1	1107 aa		NC_017939	NC_017938
MMMoV	melon mild mottle virus		1120 aa		NC_038765	NC_038766
HoNV3	Hobart nepovirus 3		n/a		MG995737	n/a
PCMoV	petunia chlorotic mottle virus	A2	1199 aa		KX812815	KX812816
AVA	arracacha virus A	A2	1137 aa		KY569302	KX812816
PBRSV	potato black ringspot virus	A3	1082 aa		NC_022798	NC_022799
TRSV	tobacco ringspot virus	A3	1101 aa		NC_005097	NC_005096
AeRSV	aeonium ringspot virus	A3	1128 aa		NC_038762	NC_038761
HoNV1	Hobart nepovirus 1	A4	n/a		MG995736	n/a
HoNV2	Hobart nepovirus 2	A4	n/a		MG995735	n/a
MMLRaV	mulberry mosaic leaf roll associated virus		1093 aa		NC_038767	NC_038768
RpRSV	raspberry ringspot virus		1106 aa		NC_005266	NC_005267
OLRSV	olive latent ringspot virus		1145 aa		n/a	NC_038863
**Subgroup B**						
BRSV	beet ringspot virus ^a^	B	1357 aa		NC_003693	NC_003694
PoLNVA	poaceae Liege nepovirus A	B	1250 aa		MW289235	MW289236
TBRV	tomato black ring virus	B	1343 aa		NC_004439	NC_004440
AILV	artichoke Italian latent virus	B	1347 aa		LT608395	LT608396
RCNVA	red clover nepovirus A	B	1366 aa		MG253828	MG253829
GARSV	grapevine Anatolian ringspot virus	B	1350 aa		NC_018383	NC_018384
GCMV	grapevine chrome mosaic virus	B	1324 aa		NC_003622	NC_003621
PVB	potato virus B	B	1371 aa		KX656670	KX656671
CNSV	cycas necrotic stunt virus	B	1240 aa		NC_003791	NC_003792
**Subgroup C**						
ToRSV	tomato ringspot virus	C1	1822 aa		NC_003840	NC_003839
AnNVA	anemome nepovirus A	C1	incomplete		MH898479	MH898478
CLRV	cherry leaf roll virus	C1	1589 aa		NC_015414	NC_015415
SteNV	stenotaphrum nepovirus	C1	1602 aa		MZ325761	MZ325762
BaNV1	babaco nepovirus 1	C1	n/a		MN648672	n/a
BLSV	blueberry latent spherical virus	C2	1631 aa		NC_038764	NC_038763
PRMV	peach rosette mosaic virus	C2	1474 aa		NC_034214	NC_034215
CawYV	caraway yellows virus	C2	1673 aa		MK492273	MK492274
SLSV	soybean latent spherical virus	C2	1389 aa		NC_032270	NC_032271
PVU	potato virus U	C2	1544 aa		NC_040417	NC_040416
BRV	blackcurrant reversion virus	C3	1626 aa		NC_003509	NC_003502
GBLV	grapevine Bulgarian latent virus	C3	incomplete		NC_015492	NC_015493
BLMoV	blueberry leaf mottle virus	C3	1739 aa		MT591564	MT591565
AYRSV	artichoke yellow ringspot virus		n/a		NC_038862	n/a
GSPNeV	green Sichuan pepper nepovirus		1861 aa		MH323435	MH323434
GTRSV	grapevine Tunisian ringspot virus		1535 aa		n/a	MT303062
GNVA	grapevine nepovirus A		1330 aa		MT507290	MT507291

^a^ Beet ringspot virus was referred to as tomato black ring virus Scottish serotype in the early literature but classified as a member of a distinct species in 1998.

To identify non-classified nepovirus-related sequences, the 3CL-Pro sequences of representative nepoviruses (ToRSV, arabis mosaic virus [ArMV], BLSV, beet ringspot virus [BRSV], tobacco ringspot virus [TRSV], BRV, green Sichuan pepper nepovirus [GSPNeV], and grapevine nepovirus A [GNVA]) were used to search the NCBI database using BLASTp. This allowed the identification of six additional viruses that clearly branched with other nepoviruses in phylogenetic analysis but were distinct from the type isolates of existing nepovirus species, using the currently accepted species demarcation criteria (less than 80% amino acid sequence identity in Pro-Pol and/or less than 75% amino acid sequence identity in the CP) [71]. Three viruses were identified from plant samples: anemone nepovirus A (AnNVA, near-complete sequence of RNA1 and RNA2), stenotaphrum nepovirus (SteNV, complete sequence of RNA1 and RNA2) [73], and babaco nepovirus 1 (BaNV1, only RNA1 sequence available) [21]. Three additional viruses (Hobart nepovirus 1 to 3, HoNV1, HoNV2, HoNV3, with only the RNA1 sequence available) were identified by next-generation sequencing of Australian honey bee (*Apis mellifera*) samples but are probably plant viruses [60].

The polyprotein amino acid sequences were aligned (see Table 1 for accession numbers). A single alignment was generated for the RNA1 polyproteins (Supplementary Material 1). Given the diverse size of the RNA2 polyproteins (Table 1) and the sequence variability in their N-terminal regions, two separate alignments were generated. The first one included nepoviruses with a smaller RNA2 polyprotein (1082 to 1371 aa), i.e., nepoviruses currently assigned to subgroups A and B, as well as HoNV1, HoNV2, HoNV3, and GNVA (Supplementary Material 2). The second one included nepoviruses with a larger RNA2 polyprotein (1474 aa to 1861 aa), i.e., nepoviruses currently assigned to subgroup C, as well as AnNVA, SteNV, BaNV1, and GSPNeV (Supplementary Material 3).

The sequences of AnNVA RNAs are partial and do not include the first AUG of each polyprotein. In the NCBI database, the annotated translated polyproteins were initiated at internal AUGs. The two polyprotein translations were redone starting at the first available in-frame codon of the polyproteins. This allowed inclusion of a larger portion of the polyproteins, including the putative X1-X2 cleavage site for the RNA1 polyprotein (Supplementary Material 1 and 2). The sequence of grapevine Bulgarian latent virus (GBLV) RNA2 polyprotein showed a large gap in the MP domain compared to other subgroup C nepoviruses, but the regions of the polyprotein encompassing the cleavage sites upstream and downstream of MP were present (Supplementary Material 3).

Cleavage sites that have been confirmed experimentally by N-terminal Edman degradation or C-terminal carboxypeptidase A digestion of the cleaved products are shown in red in Table 2 and in the alignments (Supplementary Material 1–3) and were used to guide the prediction of non-annotated polyprotein cleavage sites and to assess the likelihood of previously predicted cleavage sites. Polyprotein cleavage sites are normally located in flexible linkers joining two protein domains. Because the length of these linkers can vary from one virus to another, a perfect alignment of the cleavage sites is not necessarily expected. However, in some cases, cleavage sites annotated in the NCBI database or previously predicted in the literature were clearly misaligned with experimentally confirmed cleavage sites and were likely incorrect. For example, the annotated GBLV VPg-Pro cleavage site (Q/A) [26] was located within the 3CL-Pro domain, downstream of the conserved His of the protease catalytic triad (Supplementary Material 1). An alternative N/S cleavage site was well aligned with other nepovirus VPg-Pro cleavage sites and contained an asparagine in the P1 position, which is also found in other predicted GBLV cleavage sites. In other cases, the nature of the amino acid at the P1 or P1’ positions deviated from dipeptides proposed for other cleavage sites of the same virus. For example, a G/S cleavage site was proposed at the VPg-Pro junction of the potato black ringspot virus (PBRSV) RNA1 polyprotein [70]. However, shifting the proposed cleavage site by one amino acid would result in a putative A/G cleavage site, which is more consistent with other proposed PBRSV cleavage sites that all have a cysteine or alanine at the P1 position. Cleavage sites that were confidently predicted based on these criteria are shown in green in Table 2 and in the alignments (Supplementary Material 1–3).

**Table 2:**
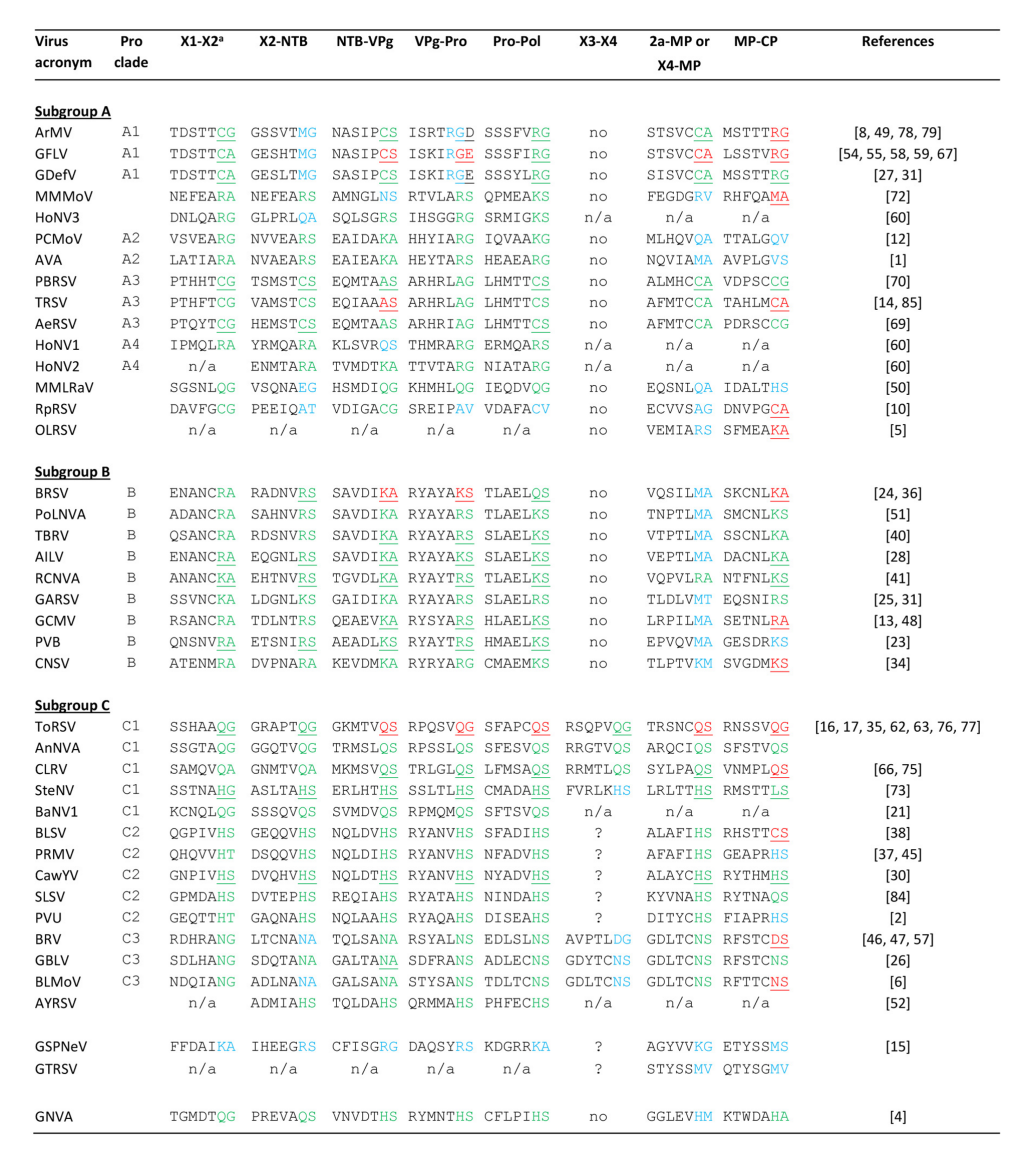
Confirmed and putative nepovirus polyprotein cleavage sites ^a^ Cleavage sites corresponding to each of the protein domain junctions. Amino acids in colour represent the P1 and P1’ position of the cleavage site. Experimentally confirmed cleavage sites (by Edman degradation or carboxypeptidase A digestion) are shown in red, cleavage sites predicted with a high degree of confidence are shown in green, and tentative cleavage sites are shown in blue. Cleavage sites that have already been proposed in the literature are underlined. Cleavage sites that are not underlined were either not predicted previously or are different from those previously predicted in the literature (see polyprotein alignments in Supplementary Material 1–3 for the position of previously predicted cleavage sites). no: the cleavage site is not present in these shorter RNA2 polyproteins, n/a: the genomic sequence is incomplete and does not include the region of the cleavage site, ?: a putative cleavage site could not be clearly identified at this position. See Table 1 for full virus names and accession numbers.

In some cases, cleavage sites were still difficult to predict because possible dipeptides did not align well with other nepovirus cleavage sites. For example, the polyproteins of the divergent GSPNeV did not align robustly with those of other nepoviruses. Thus, dipeptides with K, R, or M at the P1 position could only be tentatively proposed as putative GSPNeV cleavage sites (Table 1, Supplementary Material 1). In other cases, several putative cleavage sites were possible. For example, there are two adjacent N/A dipeptides at the X2-NTB junction of the BRV RNA1 polyprotein, each of which could correspond to the actual cleavage site. Tentatively predicted cleavage sites are shown in blue in Table 2 and in the alignments. When appropriate, alternative cleavage sites are also highlighted in pale blue in the alignments (Supplementary Material 1–3).

## Prediction of subgroup B nepovirus 2a-MP cleavage sites

**Fig. 2:**
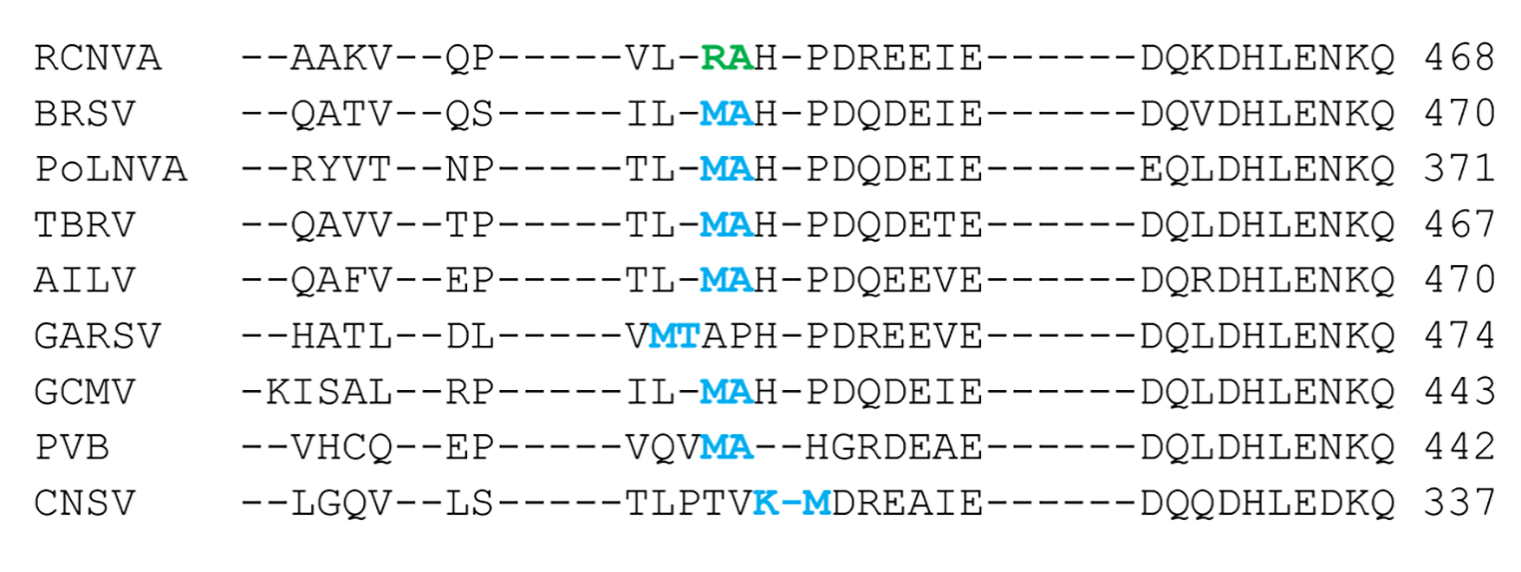
Alignment of the putative 2a-MP cleavage site of subgroup B nepoviruses (clade B). Confidently predicted and tentatively predicted cleavage sites are shown in green and blue, respectively. See Supplementary Material 2 for an alignment of the entire RNA2 polyproteins. See Table 1 for a list of virus abbreviations, full names, and accession numbers.

Almost all experimentally confirmed and previously predicted cleavage sites recognized by subgroup B nepovirus 3CL-Pros include a lysine or arginine at the P1 position. Cleavage sites at the 2a-MP junction could not be confidently predicted for subgroup B nepoviruses in the past, because K/S, K/A, R/S, or R/A dipeptides could not be found. However, the recently characterized red clover nepovirus A (RCNVA) has a possible R/A cleavage site at the 2a-MP junction (Fig. 2 and Supplementary Material 2) [41]. This allowed a re-evaluation of other subgroup B nepovirus MP-CP junctions. Cleavage sites with lysine or arginine at the P1 position could indeed not be identified, except for a putative K/M cleavage site for cycas necrotic stunt virus (CNSV). However, putative M/A or M/T cleavage sites could be predicted that aligned well with the proposed RCNVA R/A cleavage site (Fig. 2 and Supplementary Material 2). Although methionine is not positively charged like lysine or arginine, its overall conformation with a long non-branched side chain is similar to that of lysine. The suggestion that methionine can replace lysine or arginine at the P1 position of some nepovirus cleavage sites is consistent with the experimental confirmation of an M/A cleavage site for the MP-CP junction of the MMMoV RNA2 polyprotein together with confidently predicted R/A, R/S, or K/S cleavage sites in the MMMoV RNA1 polyprotein [72] (Table 2).

## Phylogenetic analysis highlights diverse clades of nepovirus 3CL-Pros

**Fig. 3:**
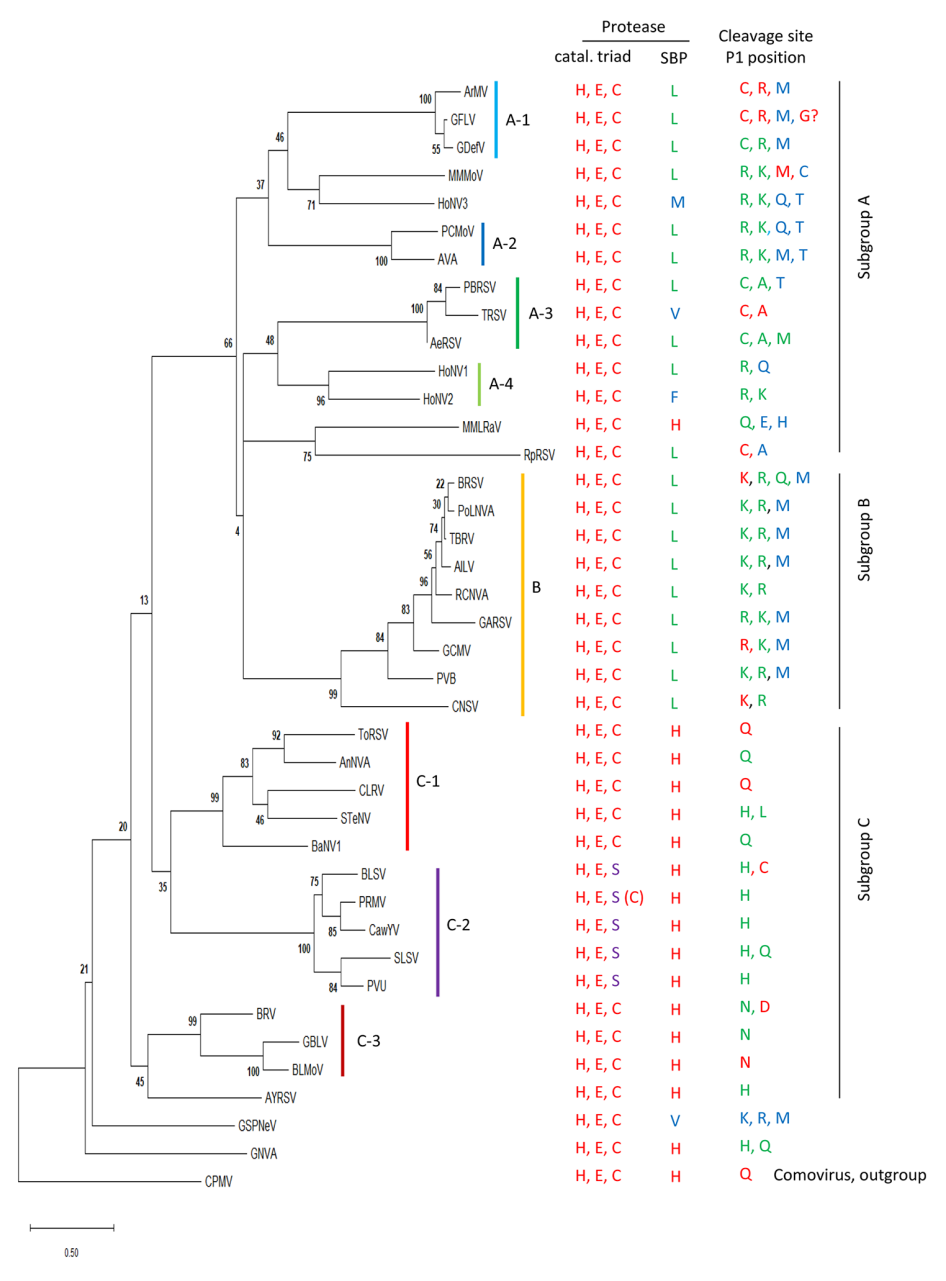
Phylogenetic relationships of nepovirus 3CL-Pros. The 3CL-Pro amino acid sequences were aligned and the phylogenetic tree was generated as described in Materials and methods. The number at each node indicates the bootstrap value (1000 replicates). See Table 1 for a list of virus abbreviations, full names, and accession numbers. Cowpea mosaic virus (CPMV), a comovirus, was used as an outgroup. Clades that were well supported by the bootstrap analysis are shown with coloured vertical bars and are labelled (A-1 to A-4, B, C-1 to C-3). Conserved amino acids of the protease catalytic triad or substrate-binding pocket are shown on the right (see Supplementary Material 1 for the positions of these amino acids in the RNA1 polyprotein alignment) as well as the amino acid found at the P1 position of experimentally confirmed (red), confidently predicted (green), or tentatively predicted (blue) cleavage sites (see Table 2).

The 3CL-Pros of nepoviruses are very diverse (as low as 16% amino acid sequence identity between type isolates of distinct species), much more so than those of comoviruses (36 to 46% amino acid sequence identity). A phylogenetic tree based on the deduced amino acid sequences of nepovirus 3CL-Pros highlights this diversity (Fig. 3). A number of strongly supported clades were identified (with bootstrap values of 95% or more). Please note that the same clades were identified when partial sequences of the 3CL-Pros were used (from the catalytic histidine to the catalytic cysteine or serine, or from the catalytic histidine to the histidine or leucine of the SBP) (Supplementary Material 4). Thus, elucidation of the VPg-Pro and Pro-Pol cleavage sites is not strictly required to evaluate the relationships of the nepovirus 3CL-Pros.

The 3CL-Pros of subgroup B nepoviruses branched together, forming clade B. However, the 3CL-Pros of subgroup C or subgroup A nepoviruses did not form two clear separate branches. Three separate clades were identified for the 3CL-Pros of subgroup C nepoviruses (clades C-1, C-2, and C-3). The 3CL-Pro of artichoke yellow ringspot virus (AYRSV) formed a possible separate fourth clade, although it showed some relationship to the 3CL-Pros from clade C-3. The phylogenetic relationships among the 3CL-Pros of subgroup A nepoviruses were even more complex, with at least three strongly supported clades (A-1, A-2, A-3) and a fourth clade (A-4) encompassing two bee-associated nepovirus-like sequences (HoNV1 and HoNV2). The 3CL-Pros of four other subgroup A nepoviruses (MMMoV, HoNV3, mulberry mosaic leaf roll associated virus [MMLRaV], and raspberry ringspot virus [RpRSV]) possibly formed four additional clades, although distant relationships were noted between the 3CL-Pros of MMMoV and HoNV3 and the 3CL-Pros of MMLRaV and RpRSV. Finally, the 3CL-Pros of two newly identified nepoviruses, GNVA and GSPNeV, branched separately from those of other nepoviruses (Fig. 3).

The catalytic triad of most nepovirus 3CL-Pros consists of histidine, glutamate, and cysteine. Proteases from clade C-2 are an exception, with a catalytic serine replacing the cysteine (Fig. 3, Supplementary Material 1), although the Pro of one partially sequenced isolate of peach rosette mosaic virus (PRMV) does have a catalytic cysteine rather than serine [45].

The 3CL-Pros from subgroup C nepoviruses, GNVA, and MMLRaV (a subgroup A nepovirus) include the conserved histidine in their substrate-binding pockets (Fig. 3, Supplementary Material 1). The SBP histidine was replaced by leucine in most other nepoviruses, although other amino acids were also present at this position (valine for TRSV and GSPNeV, methionine for HoNV3, and phenylalanine for HoNV2).

Interestingly, the strongly supported 3CL-Pro clades could also be identified in phylogenetic trees derived from the Pro-Pol or CP sequences (Supplementary Material 5), confirming that the evolution of nepovirus genomes is linked to that of their proteases.

## Cleavage site sequence logos highlight the diverse specificities of nepovirus 3CL-Pros

**Fig. 4:**
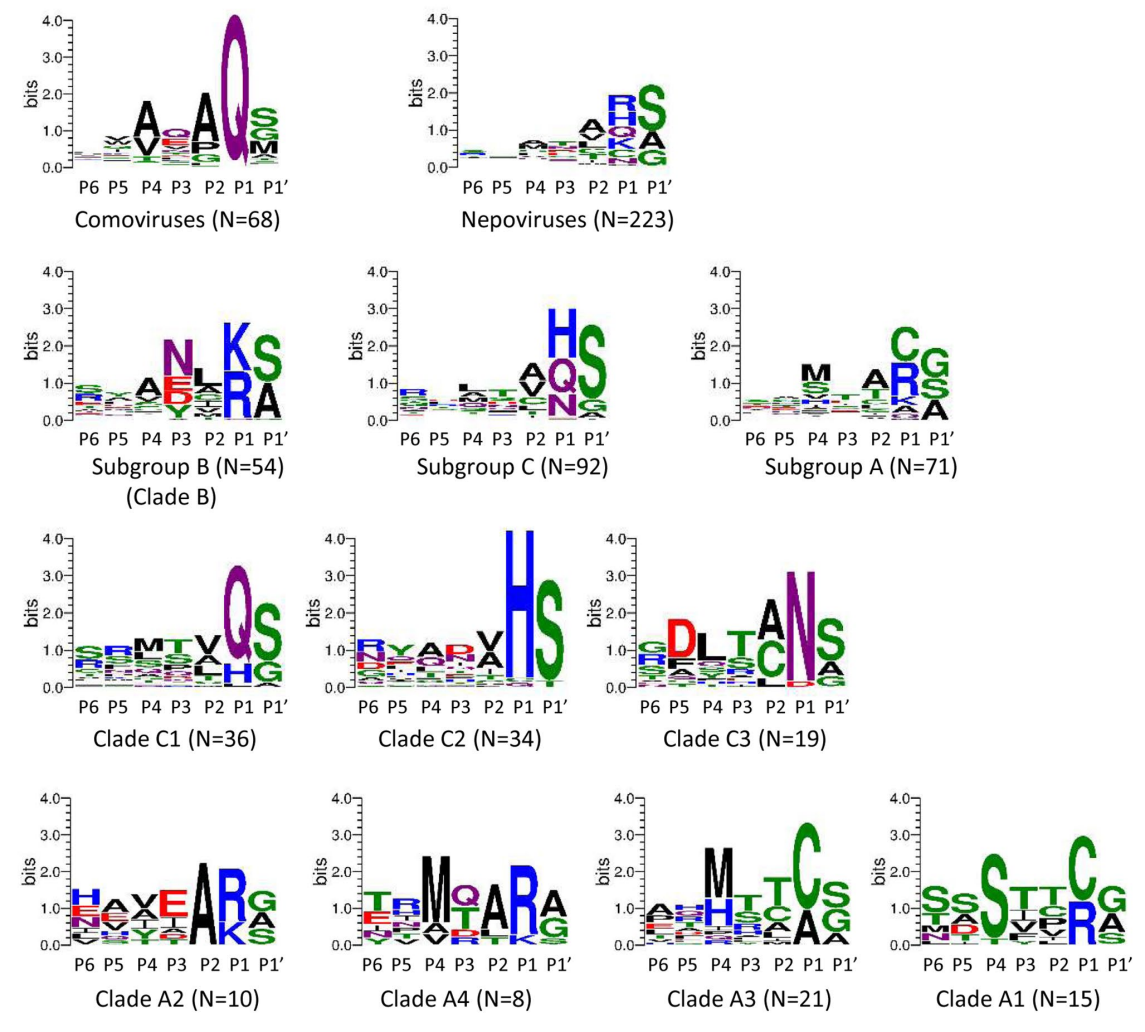
Sequence logos for the P6 to P1’ positions of cleavage sites corresponding to nepovirus subgroups or protease clades. Sequence logos were produced as described in Materials and methods, using confidently predicted or experimentally confirmed cleavage sites shown in Table 2. Cleavage sites that were only tentatively predicted were not included in the analysis. The number of cleavage sites included in each sequence logo is indicated (N = x). Amino acid colours correspond to the chemistry: polar amino acids (G, S, T, Y, C, green), neutral amino acids (Q, N, purple), basic amino acids (K, R, H, blue), acidic amino acids (D, E, red) and hydrophobic amino acids (A, V, L, I, P, W, F, M, black).

**Fig. 5:**
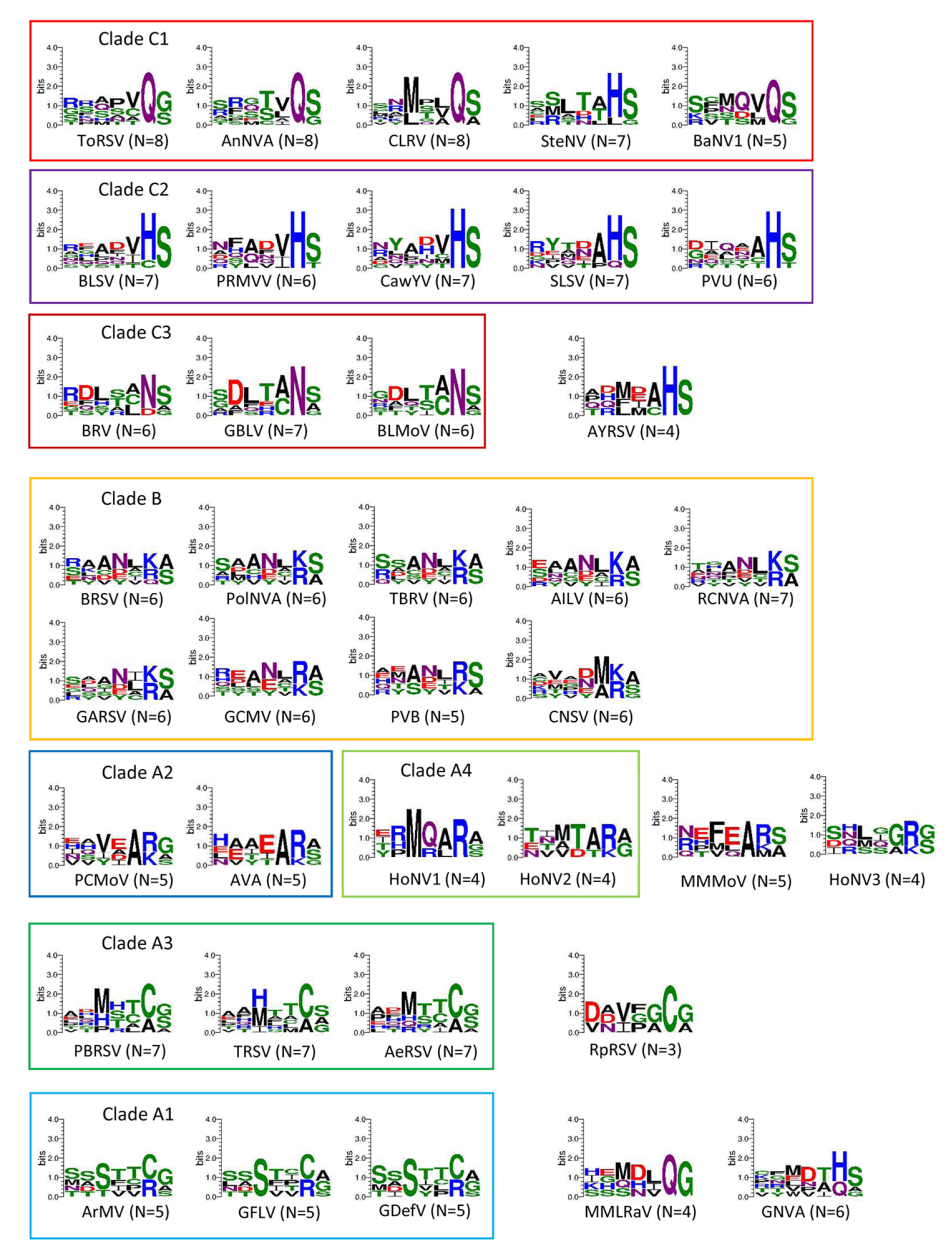
Sequence logos for the P6 to P1’ positions of cleavage sites corresponding to individual nepoviruses. Sequence logos were produced as described in Materials and methods, using confidently predicted or experimentally confirmed cleavage sites shown in Table 2. Cleavage sites that were only tentatively predicted were not included in the analysis. The number of cleavage sites included in each sequence logo is indicated (N = x, with a minimum value of 3). Amino acid colours are as described in Fig. 4.

Comovirus 3CL-Pros are known to share a cleavage site consensus sequence with a glutamine at the P1 position, a serine, glycine or methionine at the P1’ position, and an alanine at the P2 and P4 positions. Cleavage sites can be easily identified in comovirus polyproteins and were used to generate a sequence logo (from the P6 to P1’ positions) that confirmed this signature sequence (Fig. 4). In contrast, a sequence logo generated with all experimentally confirmed and confidently predicted nepovirus cleavage sites from Table 2 did not highlight a clear consensus sequence, except for an enrichment for small amino acids (serine, alanine, or glycine) at the P1’ position. Next, sequence logos were generated for the three nepovirus subgroups or for nepoviruses belonging to the same protease clades (Fig. 4). Nepovirus subgroups did not show clear cleavage site consensus sequences, with the exception of subgroup B, which also corresponds to 3CL-Pro clade B. Clearer signature sequences were obtained when considering individual 3CL-Pro clades (Fig. 4) or individual viruses (Fig. 5).

## Specificities of the 3CL-Pros of clades C-1, C-2, and C-3

All subgroup C nepovirus 3CL-Pros contain the conserved His in their SBP. However, cleavage site sequence logos derived from subgroup C nepoviruses did not highlight a strong consensus sequence (Fig. 4). Sequence logos derived from individual 3CL-Pro clades were more informative. Clade C-1 cleavage sites showed an enrichment for glutamine at the P1 position and a serine or glycine at the P1’ position, similar to the cleavage sites of comoviruses and picornaviruses (Fig. 4). A notable exception for this clade were the predicted cleavage sites of SteNV, with histidine or leucine at the P1 position (Table 2, Fig. 5). The majority of clade C-2 cleavage sites had a histidine at the P1 position and a marked preference for a serine at the P1’ position. Clade C-3 cleavage sites showed a strong bias for asparagine at the P1 position and preferences for serine, alanine, or glycine at the P1’ position and cysteine or alanine at the P2 position.

## Specificities of the 3CL-Pros of clades B, A2, and A4

The cleavage sites of clade B 3CL-Pros showed a preference for lysine or arginine at the P1 position and serine or alanine at the P1’ position (Fig. 4). Non-charged residues were often found at the P2 and P4 positions, and asparagine, aspartate, and glutamate were enriched at the P3 position. This consensus sequence was also observed when producing sequence logos for individual viruses within this clade (Fig. 5). It should be noted that the putative M/A or M/T 2a-MP cleavage sites were not included in the sequence logo analysis, but all contained a leucine or valine at the P2 position, consistent with the consensus sequence (Table 2).

Similar to the cleavage sites recognized by clade B proteases, the majority of cleavage sites proposed for viruses in clades A2 and A4 and for MMMoV and HoNV3 have a lysine or arginine at the P1 position (Figs. 4 and 5, Table 2). However, cleavage sites in the RNA2 polyproteins were diverse and often difficult to predict, with confirmed or putative M/A cleavage sites (MMMoV, arracacha virus A [AVA]), but also tentative Q/A and C/A cleavage sites for petunia chlorotic mottle virus (PCMoV) and MMMoV, respectively (Table 2, Supplementary Material 2).

The sequence logos were used to refine the prediction of cleavage sites. For example, a V/A cleavage site was proposed for the PCMoV MP-CP junction [12]. However, a valine at the P1 position has not been confirmed experimentally for nepovirus cleavage sites. In addition, the proposed V/A cleavage site would include a glutamine at the P2 position, which is not seen in other cleavage sites for this group of viruses (Fig. 5, Table 2). Shifting the proposed PCMoV by one amino acid results in a tentative Q/V cleavage site, which would include a glutamine at the P1 position and a glycine at the P2 position, both of which are more consistent with other cleavage sites (Table 2, Fig. 5). However, the position of the PCMoV MP-CP cleavage site remains uncertain, as valine is an uncommon amino acid at the P1’ position of nepovirus cleavage sites.

## Specificity of clade A3 3CL-Pros

Clade A3 had the most consistent cleavage sites, all including a cysteine or alanine at the P1 position, and a preference for methionine or histidine at the P4 position (Figs. 4 and 5). RpRSV showed a similar preference for a cysteine at the P1 position (Fig. 5).

## The surprising relaxed specificity of clade A1 3CL-Pros

Clade A1 3CL-Pros have diverse cleavage sites, including arginine or cysteine at the P1 position of confirmed cleavage sites (Table 2). The side chains of these amino acids are very different in size. A G/E cleavage site was also identified at the grapevine fanleaf virus (GFLV) VPg-Pro junction [58] based on digestion of the C-terminal residue of the VPg by carboxypeptidase A. This cleavage site is particularly surprising, because the glutamate at the P1’ position deviates from the glycine, alanine, or serine commonly found at this position in nepovirus cleavage sites. Also, a glycine at the P1 position has not been identified for any other nepovirus cleavage sites. A shift of one amino acid would identify an R/G dipeptide, which would be more consistent with another experimentally confirmed GFLV cleavage site (Table 2). It should be noted that carboxypeptidase A releases all C-terminal amino acids, with the exception of arginine, lysine, and proline [9]. Thus, cleavage at the alternate R/G site would not have been identified by this method. A G/V cleavage site was proposed for the X2-NTB junction in the ArMV RNA1 polyprotein, and support for this prediction was provided by site-directed mutagenesis (deletion of the GV dipeptide) [78]. However, re-examination of the sequence identifies a possible M/G dipeptide (shifted by one amino acid) that would also be affected by this mutation (Table 2).

To gain more insights in the specificity of clade A1 3CL-Pros, alignments of the polyproteins of completely sequenced isolates of GFLV and ArMV were generated (Supplementary Material 6), and a summary is presented in Table 3. The cysteine at the P1 position of the ArMV X1-X2 and NTB-VPg cleavage sites was replaced by arginine in two and three isolates, respectively. A substitution of arginine for methionine was observed at the Pro-Pol cleavage site for one ArMV isolate and two GFLV isolates. In addition, 11 ArMV isolates showed a replacement of the P1 methionine of the newly proposed X2-NTB M/G cleavage site by lysine. These results suggest that arginine, lysine, methionine, and cysteine at the P1 position are all recognized by clade A1 proteases. Sequence logos produced using confidently predicted or experimentally confirmed (by Edman degradation sequencing) cleavage sites, thus excluding the X2-NTB and VPg-Pro cleavage sites, showed a strong preference for serine or occasionally threonine at the P4 position (Figs. 4 and 5). This serine (or threonine) was conserved in all ArMV and GFLV isolates with very few exceptions (Table 3). A serine at the P4 position was not present in the previously proposed G/E or G/D VPg-Pro cleavage sites and G/V or G/A X2-NTB cleavage sites. However, the alternate R/G VPg-Pro and M/G X2-NTB cleavage sites would include a serine at the P4 position. N-terminal Edman degradation of the Pro and NTB cleaved products would be necessary to confirm the exact positioning of these cleavage sites.

**Table 3:**
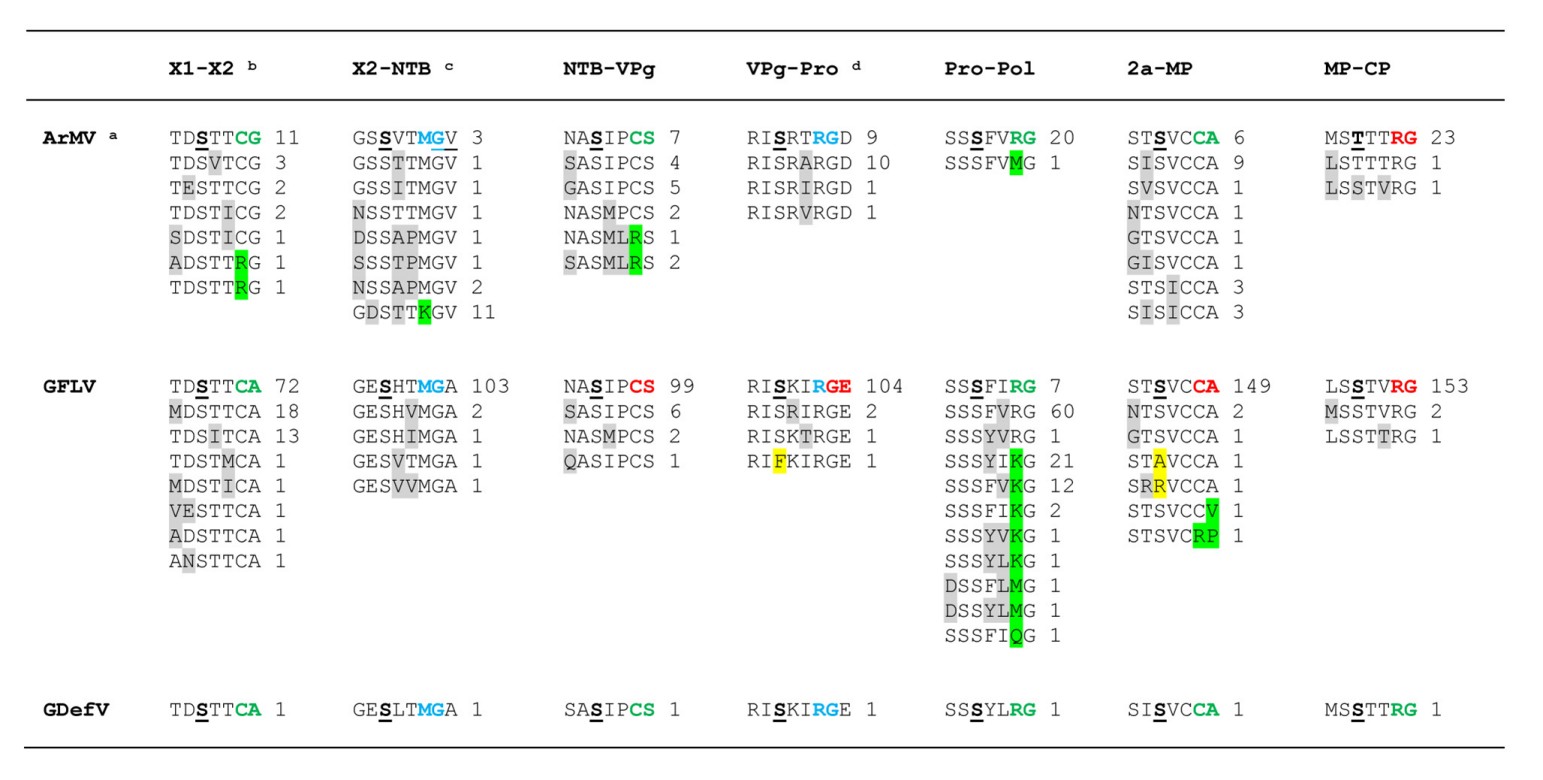
Variability in the P6 to P1’ sequence of cleavage sites in clade A1 virus isolates ^a^ Sequences of the polyproteins of ArMV and GFLV isolates were aligned (Supplementary Material 6) and variability in cleavage site sequences was analyzed. There were 21 ArMV RNA1 polyprotein sequences, 25 ArMV RNA2 polyprotein sequences, 106 GFLV RNA1 polyprotein sequences, and 156 GFLV RNA2 polyprotein sequences in total. ^b^ The top sequence for each cleavage site represents that of the type isolate for each virus (see Table 1 for accession numbers of the type isolate). Amino acids at positions P1 and P1’ of each cleavage site are highlighted in red (experimentally confirmed), green (confidently predicted), and blue (tentative). The conserved serine (or threonine in the case of the ArMV MP-CP cleavage site) at the P4 position is shown in bold and underlined. Sequences that deviated at one or more positions (positions P6 to P1’ were compared) are listed below the type sequence with the number of sequences associated with each variant indicated. Amino acids that differed in positions P6, P5, P3, and P2 are shaded in grey. Amino acids that differed in positions P1 and P1’ are shaded in green. Variation from the highly conserved serine or threonine at position P4 are shaded in yellow. ^c^ A previously predicted G/V cleavage site is underlined for the ArMV X2-NTB cleavage site. However, a more probable M/G cleavage site is indicated in blue. The underlined serine corresponds to the P4 position of the putative M/G cleavage site. ^d^ A G/E cleavage site at the GFLV VPg-Pro junction, which was previously identified by C-terminal sequencing of the VPg using carboxypeptidase A digestion, is shown in red. An adjacent alternative R/G cleavage site is shown with the blue letter for the P1 position. The underlined serine corresponds to the P4 position of the alternative R/G cleavage site.

## Discussion

This analysis provides new insights into the cleavage site specificity of nepovirus proteases and the genomic organization of nepoviruses. Cleavage sites delineating the X1 and X2 domains could be confidently identified in the P1 polyproteins of all nepoviruses (Table 2, Supplementary Material 1), thus distinguishing nepoviruses from members of the other two genera, *Comovirus* and *Fabavirus* in the subfamily *Comovirinae*, all of which have a single protein domain upstream of NTB. The RNA2 polyprotein was more diverse, with either one or two protein domains upstream of MP. Viruses with a shorter RNA2 had a single protein domain in this region of the polyprotein (Table 2, Supplementary Material 2). Two protein domains could be clearly delineated for viruses in clades C1 and C3 (Table 2, Supplementary Material 3) and have been supported by experimental evidence for ToRSV [17]. However, the presence of one or two protein domains upstream of MP could not be clearly established for viruses in clade C2, or for GSPNeV and GTRSV.

Sequence logos produced from experimentally confirmed and confidently predicted cleavage sites highlight the different specificities of nepovirus 3CL-Pros in correlation with the phylogenetic clades. It is anticipated that this analysis will be helpful in predicting cleavage sites of new nepoviruses. The results confirmed previous conclusions that the conserved histidine in the SBP of the protease is a major determinant of its specificity [7]. In the presence of the histidine, cleavage sites with glutamine, histidine, and asparagine at the P1 position were preferred, although there were some exceptions, including experimentally confirmed D/S and C/S cleavage sites at the MP-CP junction for BRV and BLSV, respectively. Thus, even in the presence of the conserved histidine, nepovirus 3CL-Pros show more-relaxed specificity than their picornavirus or comovirus counterparts.

In the absence of the conserved SBP histidine, three different types of protease specificities were observed. Proteases from clades B, A2, and A4, MMoV, HoNV3, and possibly GSPNeV prefer arginine, lysine, and methionine at the P1 position, all of which have long side chains. Proteases from clade A3 and RpRSV prefer the smaller cysteine or alanine at this position. The specificity of proteases from clade A1 is the most relaxed. Experimentally confirmed or predicted cleavage sites of GFLV and ArMV isolates had cysteine, arginine, lysine, methionine, and possibly glycine at the P1 position. It is possible that the strict requirement for serine (or threonine) at the P4 position of clade A1 cleavage sites compensates for the more-relaxed specificity at the P1 position.

Although sequence logos are informative, it should be acknowledged that not all cleavage sites are recognized with the same efficiency or specificity. Suboptimal cleavage sites regulate the accumulation of intermediate polyproteins and mature proteins that can differ in their biological properties. For example, the proteolytic activity of the ToRSV VPg-Pro intermediate polyprotein is different from that of the mature Pro [18]. Differential proteolytic activities of VPg-Pro-Pol, VPg-Pro, and Pro were also noted for BRSV and GFLV [36, 56]. The ToRSV NTB-VPg and VPg-Pro-Pol intermediate polyproteins both accumulate in infected cells and regulate the formation and activity of the replication complex [19, 33]. The ArMV X2-NTB junction is also inefficiently cleaved, leading to the accumulation of the NTB-VPg intermediate polyprotein [79]. Suboptimal cleavage sites often deviate from the consensus sequence, as shown for potyvirus VPg-Pro E/G cleavage sites, which diverge from the consensus Q/G [53, 83]. Similarly, the suboptimal BRSV Pro-Pol Q/S cleavage site deviates from other BRSV cleavage sites with a lysine or arginine at the P1 position (Table 2) [36]. Mutagenesis analyses have also shown that the specificity of the ToRSV and cowpea mosaic virus (comovirus) proteases is more stringent for sites cleaved *in trans* than for sites cleaved *in cis* [16, 20]. Finally, the specificity of nepovirus 3CL-Pros can also differ between isolates of the same virus species, as shown for the ArMV-NW and ArMV-Lv isolates [79].

Picornavirus 3C-Pros are known to cleave hundreds of host proteins to facilitate their infection cycles [39, 64]. The case has been made that evolution of 3C- and 3CL-Pros is driven at least in part by evolutionary pressures dictated by the cleavage of host proteins [74]. Recently, cleavage of several plant proteins by potyvirus NIa 3CL-Pros was confirmed experimentally [82]. It can be anticipated that the 3CL-Pros of nepoviruses also target plant proteins. A broad host range has been noted for the vast majority of nepoviruses based on the prevalence of individual nepoviruses in diverse crops in the field and on experimental host screening. These broad host ranges can include woody and herbaceous plants spanning diverse plant families [65]. It is possible that the relaxed and diverse specificities of nepovirus 3CL-Pros help facilitate the switch from one host to another. It will be particularly interesting to identify plant proteins cleaved by nepovirus 3CL-Pros with divergent specificities. Cleavage of similar families of plant proteins by nepoviruses could be anticipated, given the similar infection cycles and shared hosts and symptomatology of many nepoviruses [29]. The identification of plant proteins cleaved by potyvirus 3CL-Pros was facilitated by a bioinformatic interrogation of plant proteomes using the consensus cleavage site sequence of the plum pox virus 3CL-Pro [82]. It is hoped that clarification of nepovirus 3CL-Pros specificities will also help identify plant protein targets of these proteases.

Finally, the identification of nepovirus 3CL-Pro clades (Fig. 3), which are supported by phylogenetic analyses of the Pro-Pol and CP sequences (Supplementary Material 5), may help clarify the taxonomy of nepoviruses. With the exception of subgroup B, the existing subgroups are not supported by these phylogenetic analyses and do not adequately represent the diversity of nepoviruses. New subgenera may be created in the future that could be represented by the clades identified here, although other criteria may also be considered.

## Electronic Supplementary Material

Below is the link to the electronic supplementary material

Supplementary Material 1

Supplementary Material 2

Supplementary Material 3

Supplementary Material 4

Supplementary Material 5

Supplementary Material 6

## References

[1] Adams IP, Boonham N, Jones RAC (2017) First Complete Genome Sequence of Arracacha virus A Isolated from a 38-Year-Old Sample from Peru. Genome announcements 5 10.1128/genomeA.00141-17 PMC5477179 28473370

[2] Adams IP, Boonham N, Jones RAC (2018) A 33-Year-Old Plant Sample Contributes the First Complete Genomic Sequence of Potato Virus U. Microbiol Resour Announc 7 10.1128/MRA.01392-18 PMC6298553 30574586

[3] Adams MJ, Antoniw JF, Beaudoin F (2005). Overview and analysis of the polyprotein cleavage sites in the family *Potyviridae*. Mol Plant Pathol.

[4] Al Rwahnih M, Alabi OJ, Hwang MS, Tian T, Mollov D, Golino D (2021). Characterization of a New Nepovirus Infecting Grapevine. Plant Dis.

[5] Alkowni R, Grieco F, Martelli GP (2001). Complete nucleotide sequence of RNA-2 of Olive latent ringspot virus. Archive of Virology.

[6] Bacher JW, Warkentin D, Ramsdell D, Hancock JF (1994). Sequence analysis of the 3’ termini of RNA1 and RNA2 of blueberry leaf mottle virus. Virus Res.

[7] Bergmann EM, Mosimann SC, Chernaia MM, Malcolm BA, James MN (1997). The refined crystal structure of the 3C gene product from hepatitis A virus: specific proteinase activity and RNA recognition. J Virol.

[8] Bertioli DJ, Harris RD, Edwards ML, Cooper JI, Hawes WS (1991). Transgenic plants and insect cells expressing the coat protein of arabis mosaic virus produce empty virus-like particles. J Gen Virol.

[9] Bhagavan NV (2002) Chap. 3 - Protein isolation and determination of amino acid sequence. Medical Biochemistry (Fourth Edition). Academic Press, pp 35–50

[10] Blok VC, Wardell J, Jolly CA, Manoukian A, Robinson DJ, Edwards ML, Mayo MA (1992). The nucleotide sequence of RNA-2 of raspberry ringspot nepovirus. J Gen Virol.

[11] Blom N, Hansen J, Blaas D, Brunak S (1996). Cleavage site analysis in picornaviral polyproteins: discovering cellular targets by neural networks. Protein Sci.

[12] Bratsch S, Lockhart B, Mollov D (2017). Characterization of a New Nepovirus Causing a Leaf Mottling Disease in Petunia hybrida. Plant Dis.

[13] Brault V, Hibrand L, Candresse T, Le Gall O, Dunez J (1989). Nucleotide sequence and genetic organization of Hungarian grapevine chrome mosaic nepovirus RNA2. Nucleic Acids Res.

[14] Buckley B, Silva S, Singh S (1993). Nucleotide sequence and in vitro expression of the capsid protein gene of tobacco ringspot virus. Virus Res.

[15] Cao M, Zhang S, Li M, Liu Y, Dong P, Li S, Kuang M, Li R, Zhou Y (2019) Discovery of Four Novel Viruses Associated with Flower Yellowing Disease of Green Sichuan Pepper (Zanthoxylum Armatum) by Virome Analysis. Viruses 11 10.3390/v11080696 PMC6723833 31370205

[16] Carrier K, Hans F, Sanfacon H (1999). Mutagenesis of amino acids at two tomato ringspot nepovirus cleavage sites: effect on proteolytic processing in cis and in trans by the 3C-like protease. Virology.

[17] Carrier K, Xiang Y, Sanfacon H (2001). Genomic organization of RNA2 of Tomato ringspot virus: processing at a third cleavage site in the N-terminal region of the polyprotein in vitro. J Gen Virol.

[18] Chisholm J, Wieczorek A, Sanfaçon H (2001). Expression and partial purification of recombinant tomato ringspot nepovirus 3C-like proteinase: comparison of the activity of the mature proteinase and the VPg-proteinase precursor. Virus Res.

[19] Chisholm J, Zhang G, Wang A, Sanfacon H (2007). Peripheral association of a polyprotein precursor form of the RNA-dependent RNA polymerase of Tomato ringspot virus with the membrane-bound viral replication complex. Virology.

[20] Clark AJ, Bertens P, Wellink J, Shanks M, Lomonossoff GP (1999). Studies on hybrid comoviruses reveal the importance of three-dimensional structure for processing of the viral coat proteins and show that the specificity of cleavage is greater in trans than in cis. Virology.

[21] Cornejo-Franco JF, Medina-Salguero A, Flores F, Chica E, Grinstead S, Mollov D, Quito-Avila DF (2020). Exploring the virome of Vasconcellea x heilbornii: the first step towards a sustainable production program for babaco in Ecuador. Eur J Plant Pathol.

[22] Crooks GE, Hon G, Chandonia JM, Brenner SE (2004). WebLogo: a sequence logo generator. Genome Res.

[23] De Souza J, Muller G, Perez W, Cuellar W, Kreuze J (2017). Complete sequence and variability of a new subgroup B nepovirus infecting potato in central Peru. Arch Virol.

[24] Demangeat G, Hemmer O, Reinbolt J, Mayo MA, Fritsch C (1992). Virus-specific proteins in cells infected with tomato black ring nepovirus: evidence for proteolytic processing in vivo. J Gen Virol.

[25] Digiaro M, Nahdi S, Elbeaino T (2012). Complete sequence of RNA1 of grapevine Anatolian ringspot virus. Arch Virol.

[26] Elbeaino T, Digiaro M, Fallanaj F, Kuzmanovic S, Martelli GP (2011). Complete nucleotide sequence and genome organisation of grapevine Bulgarian latent virus. Archive of Virology.

[27] Elbeaino T, Digiaro M, Ghebremeskel S, Martelli GP (2012). Grapevine deformation virus: completion of the sequence and evidence on its origin from recombination events between Grapevine fanleaf virus and Arabis mosaic virus. Virus Res.

[28] Elbeaino T, Belghacem I, Mascia T, Gallitelli D, Digiaro M (2017). Next generation sequencing and molecular analysis of artichoke Italian latent virus. Arch Virol.

[29] Fuchs M, Schmitt-Keichinger C, Sanfaçon H (2017). A renaissance in nepovirus research provides new insights into their molecular interface with hosts and vectors. Adv Virus Res.

[30] Gaafar YZA, Richert-Poggeler KR, Sieg-Muller A, Luddecke P, Herz K, Hartrick J, Maass C, Ulrich R, Ziebell H (2019). Caraway yellows virus, a novel nepovirus from Carum carvi. Virol J.

[31] Ghanem-Sabanadzovic NA, Sabanadzovic S, Digiaro M, Martelli GP (2005). Complete nucleotide sequence of the RNA-2 of grapevine deformation and Grapevine Anatolian ringspot viruses. Virus Genes.

[32] Gorbalenya AE, Donchenko AP, Blinov VM, Koonin EV (1989). Cysteine proteases of positive strand RNA viruses and chymotrypsin-like serine proteases. A distinct protein superfamily with a common structural fold. FEBS Lett.

[33] Han S, Sanfacon H (2003). Tomato ringspot virus proteins containing the nucleoside triphosphate binding domain are transmembrane proteins that associate with the endoplasmic reticulum and cofractionate with replication complexes. J Virol.

[34] Han SS, Karasev AV, Ieki H, Iwanami T (2002). Nucleotide sequence and taxonomy of Cycas necrotic stunt virus. Brief report. Archive of Virology.

[35] Hans F, Sanfacon H (1995). Tomato ringspot nepovirus protease: characterization and cleavage site specificity. J Gen Virol.

[36] Hemmer O, Greif C, Dufourcq P, Reinbolt J, Fritsch C (1995). Functional characterization of the proteolytic activity of the tomato black ring nepovirus RNA-1-encoded polyprotein. Virology.

[37] Ho T, Harris A, Katsiani A, Khadgi A, Schilder A, Tzanetakis IE (2018). Genome sequence and detection of peach rosette mosaic virus. J Virol Methods.

[38] Isogai M, Tatuto N, Ujiie C, Watanabe M, Yoshikawa N (2012). Identification and characterization of blueberry latent spherical virus, a new member of subgroup C in the genus Nepovirus. Arch Virol.

[39] Jagdeo JM, Dufour A, Klein T, Solis N, Kleifeld O, Kizhakkedathu J, Luo H, Overall CM, Jan E (2018) N-Terminomics TAILS Identifies Host Cell Substrates of Poliovirus and Coxsackievirus B3 3C Proteinases That Modulate Virus Infection. J Virol 92 10.1128/JVI.02211-17 PMC5874412 29437971

[40] Jonczyk M, Le Gall O, Palucha A, Borodynko N, Pospieszny H (2004). Cloning and sequencing of full-length cDNAs of RNA1 and RNA2 of a Tomato black ring virus isolate from Poland. Archive of Virology.

[41] Koloniuk I, Pribylova J, Franova J (2018). Molecular characterization and complete genome of a novel nepovirus from red clover. Arch Virol.

[42] Koonin EV, Wolf YI, Nagasaki K, Dolja VV (2008). The Big Bang of picorna-like virus evolution antedates the radiation of eukaryotic supergroups. Nat Rev Microbiol.

[43] Kumar S, Stecher G, Li M, Knyaz C, Tamura K (2018). MEGA X: Molecular Evolutionary Genetics Analysis across Computing Platforms. Mol Biol Evol.

[44] Laitinen OH, Svedin E, Kapell S, Nurminen A, Hytönen VP, Flodström-Tullberg M (2016). Enteroviral proteases: structure, host interactions and pathogenicity. Rev Med Virol.

[45] Lammers AH, Allison RF, Ramsdell DC (1999). Cloning and sequencing of peach rosette mosaic virus RNA1. Virus Res.

[46] Latvala-Kilby S, Lehto K (1999). The complete nucleotide sequence of RNA2 of blackcurrant reversion nepovirus. Virus Res.

[47] Latvala S, Susi P, Kalkkinen N, Lehto K (1998). Characterization of the coat protein gene of mite-transmitted blackcurrant reversion associated nepovirus. Virus Res.

[48] Le Gall O, Candresse T, Brault V, Dunez J (1989). Nucleotide sequence of Hungarian grapevine chrome mosaic nepovirus RNA1. Nucleic Acids Res.

[49] Loudes AM, Ritzenthaler C, Pinck M, Serghini MA, Pinck L (1995). The 119 kDa and 124 kDa polyproteins of arabis mosaic nepovirus (isolate S) are encoded by two distinct RNA2 species. J Gen Virol.

[50] Lu QY, Wu ZJ, Xia ZS, Xie LH (2015) A new nepovirus identified in mulberry (Morus alba L.) in China. Arch Virol 10.1007/s00705-014-2330-x 25577167

[51] Maclot FJ, Debue V, Blouin AG, Fontdevila Pareta N, Tamisier L, Filloux D, Massart S (2021). Identification, molecular and biological characterization of two novel secovirids in wild grass species in Belgium. Virus Res.

[52] Maliogka VI, Dovas CI, Lesemann DE, Winter S, Katis NI (2006). Molecular Identification, Reverse Transcription-Polymerase Chain Reaction Detection, Host Reactions, and Specific Cytopathology of Artichoke yellow ringspot virus Infecting Onion Crops. Phytopathology.

[53] Mann KS, Sanfaçon H (2019). Expanding repertoire of plant positive-strand RNA virus proteases. Viruses.

[54] Margis R, Viry M, Pinck M, Pinck L (1991). Cloning and in vitro characterization of the grapevine fanleaf virus proteinase cistron. Virology.

[55] Margis R, Ritzenthaler C, Reinbolt J, Pinck M, Pinck L (1993). Genome organization of grapevine fanleaf nepovirus RNA2 deduced from the 122K polyprotein P2 in vitro cleavage products. J Gen Virol.

[56] Margis R, Viry M, Pinck M, Bardonnet N, Pinck L (1994). Differential proteolytic activities of precursor and mature forms of the 24K proteinase of grapevine fanleaf nepovirus. Virology.

[57] Pacot-Hiriart C, Latvala-Kilby S, Lehto K (2001). Nucleotide sequence of black currant reversion associated nepovirus RNA1. Virus Res.

[58] Pinck M, Reinbolt J, Loudes AM, Le Ret M, Pinck L (1991). Primary structure and location of the genome-linked protein (VPg) of grapevine fanleaf nepovirus. FEBS Lett.

[59] Ritzenthaler C, Viry M, Pinck M, Margis R, Fuchs M, Pinck L (1991). Complete nucleotide sequence and genetic organization of grapevine fanleaf nepovirus RNA1. J Gen Virol.

[60] Roberts JMK, Anderson DL, Durr PA (2018). Metagenomic analysis of Varroa-free Australian honey bees (Apis mellifera) shows a diverse Picornavirales virome. J Gen Virol.

[61] Rodamilans B, Shan H, Pasin F, Garcia JA (2018). Plant Viral Proteases: Beyond the Role of Peptide Cutters. Front Plant Sci.

[62] Rott ME, Tremaine JH, Rochon DM (1991). Nucleotide sequence of tomato ringspot virus RNA-2. J Gen Virol.

[63] Rott ME, Gilchrist A, Lee L, Rochon D (1995). Nucleotide sequence of tomato ringspot virus RNA1. J Gen Virol.

[64] Saeed M, Kapell S, Hertz NT, Wu X, Bell K, Ashbrook AW, Mark MT, Zebroski HA, Neal ML, Flodstrom-Tullberg M, MacDonald MR, Aitchison JD, Molina H, Rice CM (2020). Defining the proteolytic landscape during enterovirus infection. PLOS pathog.

[65] Sanfacon H (2021) Nepovirus (Secoviridae). In: Bamford DH, Zuckerman M (eds) Encyclopedia of Virology, 4th Edition. Academic Press, Elseviers, pp 486–494

[66] Scott NW, Cooper JI, Edwards ML (1993). The identification, cloning, and sequence analysis of the coat protein coding region of a birch isolate (I2) of cherry leaf roll nepovirus. Archive of Virology.

[67] Serghini MA, Fuchs M, Pinck M, Reinbolt J, Walter B, Pinck L (1990). RNA2 of grapevine fanleaf virus: sequence analysis and coat protein cistron location. J Gen Virol.

[68] Sievers F, Wilm A, Dineen D, Gibson TJ, Karplus K, Li W, Lopez R, McWilliam H, Remmert M, Soding J, Thompson JD, Higgins DG (2011). Fast, scalable generation of high-quality protein multiple sequence alignments using Clustal Omega. Mol Syst Biol.

[69] Sorrentino R, De Stradis A, Russo M, Alioto D, Rubino L (2013). Characterization of a putative novel nepovirus from Aeonium sp. Virus Res.

[70] Souza Richards R, Adams IP, Kreuze JF, De Souza J, Cuellar W, Dullemans AM, Van Der Vlugt RA, Glover R, Hany U, Dickinson M, Boonham N (2014). The complete genome sequences of two isolates of potato black ringspot virus and their relationship to other isolates and nepoviruses. Arch Virol.

[71] Thompson JR, Dasgupta I, Fuchs M, Iwanami T, Karasev AV, Petrzik K, Sanfaçon H, Tzanetakis I, van der Vlugt R, Wetzel T, Yoshikawa N, ICTV Report Consortium (2017). ICTV virus taxonomy profile: *Secoviridae*. J Gen Virol.

[72] Tomitaka Y, Usugi T, Yasuda F, Okayama H, Tsuda S (2011). A novel member of the genus Nepovirus isolated from Cucumis melo in Japan. Phytopathology.

[73] Tran NT, Teo AC, Crew KS, Campbell PR, Thomas JE, Geering ADW (2021) Genome sequence and geographic distribution of a new nepovirus infecting Stenotaphrum secundatum in Australia.Virus Res:198554 10.1016/j.virusres.2021.198554 34487768

[74] Tsu BV, Fay EJ, Nguyen KT, Corley MR, Hosuru B, Dominguez VA, Daugherty MD (2021). Running With Scissors: Evolutionary Conflicts Between Viral Proteases and the Host Immune System. Front Immunol.

[75] von Bargen S, Langer J, Robel J, Rumbou A, Buttner C (2012). Complete nucleotide sequence of Cherry leaf roll virus (CLRV), a subgroup C nepovirus. Virus Res.

[76] Wang A, Carrier K, Chisholm J, Wieczorek A, Huguenot C, Sanfacon H (1999). Proteolytic processing of tomato ringspot nepovirus 3C-like protease precursors: definition of the domains for the VPg, protease and putative RNA-dependent RNA polymerase. J Gen Virol.

[77] Wang A, Sanfacon H (2000). Proteolytic processing at a novel cleavage site in the N-terminal region of the tomato ringspot nepovirus RNA-1-encoded polyprotein in vitro. J Gen Virol.

[78] Wetzel T, Chisholm J, Bassler A, Sanfacon H (2008). Characterization of proteinase cleavage sites in the N-terminal region of the RNA1-encoded polyprotein from Arabis mosaic virus (subgroup A nepovirus). Virology.

[79] Wetzel T, Chisholm J, Dupuis-Maguiraga L, Bassler A, Sanfaçon H (2013). *In vitro* and *in vivo* evidence for differences in the protease activity of two arabis mosaic nepovirus isolates and their impact on the infectivity of chimeric cDNA clones. Virology.

[80] Wildy P (1971) Classification and Nomenclature of Viruses. 1st Report of the International Committee on Nomenclature of Viruses. S. Karger 6066183

[81] Wolf YI, Kazlauskas D, Iranzo J, Lucia-Sanz A, Kuhn JH, Krupovic M, Dolja VV, Koonin EV (2018) Origins and Evolution of the Global RNA Virome. MBio 9 10.1128/mBio.02329-18 PMC6282212 30482837

[82] Xiao H, Lord E, Sanfacon H (2022). Proteolytic Processing of Plant Proteins by Potyvirus NIa Proteases. J Virol.

[83] Yang X, Li Y, Wang A (2021) Research Advances in Potyviruses: From the Laboratory Bench to the Field. Annu Rev Phytopathol:in press 10.1146/annurev-phyto-020620-114550 33891829

[84] Yasmin T, Nelson BD, Hobbs HA, McCoppin NK, Lambert KN, Domier LL (2017). Molecular characterization of a new soybean-infecting member of the genus Nepovirus identified by high-throughput sequencing. Arch Virol.

[85] Zalloua PA, Buzayan JM, Bruening G (1996). Chemical cleavage of 5’-linked protein from tobacco ringspot virus genomic RNAs and characterization of the protein-RNA linkage. Virology.

[86] Zhang T, Li C, Cao M, Wang D, Wang Q, Xie Y, Gao S, Fu S, Zhou X, Wu J (2021). A Novel Rice Curl Dwarf-Associated Picornavirus Encodes a 3C Serine Protease Recognizing Uncommon EPT/S Cleavage Sites. Front Microbiol.

