# Supplementary material for: Re-examination of nepovirus polyprotein cleavage sites highlights the diverse specificities and evolutionary relationships of nepovirus 3C-like proteases
Source: Arch Virol. 2022 Aug 30;167(12):2529–43. doi: 10.1007/s00705-022-05564-x (PMC9741568; doi:10.1007/s00705-022-05564-x)
Supplement: Supplementary file 1 — Supplementary Material 1 [file 705_2022_5564_MOESM1_ESM.pdf]

Supplementary Material 1 – Alignment of nepovirus P1 polyproteins

Re-examination of nepovirus polyprotein cleavage sites highlights the diverse specificities and evolutionary relationships of nepovirus 3C-like proteases

Archives of Virology

Hélène Sanfaçon

Corresponding author: Hélène Sanfaçon Summerland Research and Development Centre, Agriculture and Agri-Food Canada, helene.sanfacon@agr.gc.ca

Polyprotein sequences were aligned using CLUSTAL O(1.2.4), see Table 1 in the manuscript for accession numbers. Color lines on the left side of the alignments represent the protease clades as shown in Fig. 3 of the manuscript. Cleavage sites (P1 and P1' positions) are annotated as follows. Yellow highlights: cleavage sites annotated in the NCBI accessions, underlines: annotated putative or confirmed cleavage sites from publications (see Table 2 for relevant list of publications for each virus), red letters: cleavage sites confirmed experimentally by N-terminal Edman degradation sequencing or by C-terminal carboxypeptidase A digestion (GFLV VPg-Pro cleavage site), green letters (cleavage sites confidently predicted), blue letters (cleavage sites only tentatively predicted, when more than one choice is possible, the darker blue is the one integrated in Table 2), grey highlights (cleavage sites supported by mutagenesis). Protease sequences are annotated as follows. Red letters: consensus amino acids from the catalytic triad (H, E, C) or in the substrate-binding pocket (H). Green, blue or purple letters: divergent amino acids from the catalytic triad (S instead of C) or in the substrate-binding pocket (L, V, M or F instead of H). The GDD motif of the RNA-dependent RNA polymerase is shown in brown letters.

```
ArMV      --------------------------MWQISEGSQCCCTGKTWSNAEAKE----------    24
GFLV      --------------------------MWQVPEGSQCCCTGKSFSNAEAKE----------    24
GDefV     --------------------------MWQVSEGSQCCCTGKTFSNAEAKE----------    24
MMMoV     -------------------------MAWACQA-KACLKFGLPVSNAEARE----------    24
HoNV3     ------------------------------------------------------------    0
PCMoV     -------------------------MGWNCPN-GLCMYFGVKYTNTQAKE----------    24
AVA       -------------------------MGWSCPV-KGCLQSELSNREFREEC----------    24
PBRSV     -------------------------MGFICPN-LDCLGNKTVFSRKDLRDEGLTF-----    29
TRSV      -------------------------MGFTCPN-SDCLYSRSEWSNRALREEGLSF-----    29
AeRSV     -------------------------MGFHCPV-LNCSFHNVDWSRKALKEEGLTF-----    29
HoNV1     ------------------------------------------------------------    0
HoNV2     ------------------------------------------------------------    0
MMLRaV    ------------------------------------------------------------    0
RpRSV     -------------------------MGWTCPV-QGCMYSRSTWSNRGLKEDGLSS-----    29
BRSV      ------------MSVTLSPSGDCFSFNHVKYN-NSLNKYLFYNSNLDIVLDDFDFYFNFY    47
PoLNVA    ---------------MDSAMLDEVCLPYCKYN-YSLNKNLYFNSNLDIDLDNFRFYSFFV    44
TBRV      ------------MSVTLSPPGDCFTFNHVKYN-NSLNKYLFYNNNLDIVLDDFDFYFNFY    47
AILV      ----------MRSFVTVSPDGDSFSFNFCKYN-NSLNKFLLSSSNLDVVLDDFDFYFSFF    49
RCNVA     -----------MTFVTFPPSGDSFSFNYVKYN-RSLNKYLFFNSNLDVVLDDYDFYFSFY    48
GARSV     ------------------MDRAMMTLPYCKYN-NSLNKYLFY-SNLDIDLDNFSFYYNYN    40
GCMV      ------------------MMDSTIILPYAKYN-YSLNKYLFYNSNLDIDLDSFNFYKKYN    41
PVB       MQIGQKTNPKQGIAPLPDTMDSAIVLPYCKYN-NSLNLFFYQNTNSTINLDLFSYYSTFS    59
CNSV      -------------------------MGWICPN-VSCLGHTSVLSNKEI------------    22
ToRSV     ------------------------------------------------------------    0
AnNVA     ------------------------------------------------------------    0
CLRV      ------------------------------------------------------------    0
STeNV     ------------------------------------------------------------    0
BaNV1     ------------------------------------------------------------    0
BLSV      -------------------------MEYL--------------SRSQLKPRGG-------    14
PRMV      -------------------------MDFS--------------FIEYLPARKR-------    14
CawYV     -------------------------MSFCCPV-SSCGYATRRISRKEMREDGD-------    27
SLSV      -------------------------MSFHCPS-STCLGYTQSFSRRDMREQGD-------    27
PVU       ------------------------------------------------------------    0
BRV       ------------------------------------------------------------    0
GBLV      ------------------------------------------------------------    0
BLMoV     ------------------------------------------------------------    0
AYRSV     ------------------------------------------------------------    0
GSPNeV    ------------------------------------------------------------    0
GNVA      -------------------------MSFHCPN-PRCIAFGYRLSRKDMREDGD-------    27
```

```
ArMV      -----------------ARYVCNCILSCRLV-KVEVVPQLPK-SRIAPAQDK------AE   59
GFLV      -----------------LRYVCSCWMSTRLV-KAEAPPQQSRKSGIAPTPLK------SK   60
GDefV     -----------------LRYVCSCWMSTRLV-KAEAPPQQSRRSGIAPTPLK------SK   60
MMMoV     -----------------LRYVCDAVMCGASLMKRSQIGGLA-------IPQ---------   51
HoNV3     -----------------------------------------KRVDSAPAPET------VD   13
PCMoV     -----------------EKYXCPSAMCNSLLQKQKEP-----------------------   44
AVA       ----------------GRAGRCP--RCDALMMRVDSAPASQRKSSDSAEPKI------KC   60
PBRSV     ------------------SMRCPRACCGALLRKQ--EDIE-VVDSATTSRKSLDSAKPKC   68
TRSV      ------------------SMRCPGACCGAMLVRKQQEPEA-VDQRHSPERVRIAPHQNVC   70
AeRSV     ------------------SMRCPGACCGALLQRREQQDVK-QVDSAPASRKSVDSATPKC   70
HoNV1     ------------------------------------------------------------   0
HoNV2     ------------------------------------------------------------   0
MMLRaV    ------------------------------------------------------------   0
RpRSV     ------------------TMRCPGAMCGAMLVRSEPVQAP-KATVVPQTPVT--------   62
BRSV      VKKYNVLLSFFS-------DRVLSAL-YTSMSVSEAAS----------------------   77
PoLNVA    RKLEFIQVRFLS-------TFCRVSS-SSPSAIVRRGLLS--------------------   76
TBRV      AKKYNVLSSFFS-------DRVLSAL-YTPMSVSEAAS----------------------   77
AILV      VKKYIILENFWH-------NRCLSAL-TTPAGVVVNTP----------------------   79
RCNVA     IKKFKILKKFWY-------DRCLSAL-HTPAPVEVSTP----------------------   78
GARSV     R-KLKSLKDFFN-------NRVLSAL-STPAPVSLSSQ----------------------   69
GCMV      NTLSLLKRKFLD-------LFCIPVSSSSPPAIQKNGLLS--------------------   74
PVB       NNLKKAKSFFYFLLDTVHPDFRNSTVKFSPTYIQGSSIFKKW------------------   101
CNSV      ----------------SREGRCERAMCGS-LLVKVAVPQQPA------------------   47
ToRSV     ------------------------------------------------------------   0
AnNVA     ------------------------------------------------------------   0
CLRV      ------------------------------------------------------------   0
STeNV     ----------------------------------------------------------MC   2
BaNV1     ------------------------------------------------------------   0
BLSV      --------------------KVPCGVVPRAVLEASRLAKT-ILAKPA----NF-------   42
PRMV      --------------------KNVVAIVPRAVLEATRIATS-ILAKPA----NF-------   42
CawYV     --------------------RCLYAGCGALLVKASAMASP-APIAPAKSNVTL-------   59
SLSV      --------------------RCPHAMCGALLVKKVPTLSP-QGARPVVSKTTV-------   59
PVU       ------------------------------------------------------------   0
BRV       ------------------------------------------------------------   0
GBLV      ------------------------------------------------------------   0
BLMoV     ------------------------------------------------------------   0
AYRSV     ------------------------------------------------------------   0
GSPNeV    ------------------------------------------------------------   0
GNVA      --------------------RCTLPLCGSLYRKVEVASTP-VAPPAAPAVASF-------   59
```

```
ArMV     R-ITP----------LCNSNGGA---------APT-------------------------   74
GFLV     GTIQV----------SLPKATGV---------KPS-------------------------   76
GDefV    GTVQV----------PFPKVVGV---------KPN-------------------------   76
MMMoV    ---------------VVPVVGPI---------RPQEAS---------------LAKK-LT   71
HoNV3    RAT-------------------V---------T----SS----------SSSPFSQK---   28
PCMoV    --VAVPIT-------KVPSKAPK---------VAVNNGP----------SASPKPKKV--   74
AVA      LCWRLPIS-------RCPKHKGI---------SPSFSS------------PSPLPKSSLP   92
PBRSV    LCWHKSYGLA-----RCPKHQ---------GVAPVQTVSFGDEEVFTLVTPTPV----AP   110
TRSV     -AGLPGVGPS-----KCPKHS---------QVPLAPKSK---------------------   94
AeRSV    LCWLASVGVS-----RCPKHS---------PVASATKSK----------SPPPC----VE   102
HoNV1    ---------------HRPAVQTA---------NPKRVTP----------QPSAKVNGARA   26
HoNV2    ------------------------------------------------------------   0
MMLRaV   ------------------------------------------------------------   0
RpRSV    -------------------------------------------------AVR--------   65
BRSV     ------------------------------------------------------------   77
PoLNVA   ------------------------------------------------------------   76
TBRV     ------------------------------------------------------------   77
AILV     ------------------------------------------------------------   79
RCNVA    ------------------------------------------------------------   78
GARSV    ------------------------------------------------------------   69
GCMV     ------------------------------------------------------------   74
PVB      ------------------------------------------------------------   101
CNSV     ------------------------------------------------------------   47
ToRSV    ---MSSICFAGGNHARLPSKAAYYRAISDRELDREGRFPCGCLAQYTVQAPPPA----KT   53
AnNVA    ------------------------------------------------------------   0
CLRV     --MVKPTVFSNGE----------------------SVLPKALISE---------------   21
STeNV    LDFSFSLMKIEGE----------------------HVLPYGYLVR---------------   25
BaNV1    ------------------------------------------------------------   0
BLSV     -------------------------------------------------SL---------   44
PRMV     -------------------------------------------------AI---------   44
CawYV    -------------------------------------------------EV---------   61
SLSV     -------------------------------------------------VVPPQ------   64
PVU      ------------------------------------------------------------   0
BRV      ------------------------------------MVKIGSD------TLAPK------   12
GBLV     ----------------------------------MATFKMGSV------VLTPC------   14
BLMoV    ----------------------------------MASRTIGSV------TLRPR------   14
AYRSV    ------------------------------------------------------------   0
GSPNeV   ------------------------------------------------------------   0
GNVA     -----------------------------------QTQKSGEVRTV--------------   70
```

```
ArMV       -----IPKSKRAFEPRTPLIKQRCDVVVRV-GPPADLDLVYPALVQEEVAIPPTE-----   123
GFLV       -----IHKSKGASVAPAPLLKQRCEVVVQY-GPPADIELVYPPLVREEEKS-SNI-----   124
GDefV      -----DPKSKGASVAPTPLLKQRCEVVVQY-GPPADIELVYPPLVREEERS-SKK-----   124
MMMoV      VS---GPVR--PNGDSAPIQKQKADVVVSL-GEPLLMEFVYPPLVKEAISSQEEVPAMLL   125
HoNV3      -------KSRIAPAQI--------------------------------PEP---KKVVGL   46
PCMoV      ---------------VRPPANYQMDVVVEV-GPPANLQLIYPSRAIGKEERKR-------   111
AVA        PS---GIVQKFEKEEAPLPRASLCDAVVFT-GPPVNLELVYPSLVDQTPSPKIQR-----   143
PBRSV      --ATK----SKSAPSAPLLKKQNCDVVVAM-GPPVDLEFVYPALCG-----KGSAPTTKA   158
TRSV       --SVP----ARATVSASPLKKQHCDVVVTV-GPPADLELVYPALVS-----KGSANPPKV   142
AeRSV      KSKSA----PRAAVSASPLKKQNCDIVVAI-GPPVDLELVYPALCG-----NGTATPKKV   152
HoNV1      TSEAKIKVARSAPAVQEKLVKQTCDVCVRI-DPP-GIDFIYPALVDQSPPPKIQR-----   79
HoNV2      ------------------------------------------------------------   0
MMLRaV     ------------------------------------------------------------   0
RpRSV      --------PKRTVVSATPLRKQDCVVVVEV-GFPALLSLEYPALAGRARHTGEFDSALL-   115
BRSV       -----------------LAM------------------EDFCELALDELKINP---FHQL   99
PoLNVA     -------------ATNADHL------------------FAFANFV-LNAGVNP---FLHI   101
TBRV       -----------------LAL------------------EDFCELALDKLKINP---FHQL   99
AILV       -----------------L-Y------------------VAFAELALDKLRINP---FHQL   100
RCNVA      ------------------LY------------------EAFCKLALDELSINP---FHSL   99
GARSV      ------------------FV------------------EDFSVLILDKLRINP---FLHI   90
GCMV       -------------ASNPKHL------------------FAFANFVLD-CGVNP---FLQI   99
PVB        ------KKSNGGITTISAYT------------------ALFSSFLVKGLGVNP---YQDE   134
CNSV       ------KKKK---QATPAPR------------------PTYPPCVVEKTAATPVT-VEKV   79
ToRSV      QEKAVGRSADLQKGNVAPLKKQRCDVVVAV-SGPPPLELVYPARVGQHRLDQPSKGPLAV   112
AnNVA      ------------------------------------------------------------   0
CLRV       --------------------AREVAAFLKSTRNPAGFWVTFVAQ-------GTSLSVSQV   54
STeNV      --------------------QRMCRALL---AKPVSFAATFLAQ-------GASLSPMAV   55
BaNV1      ------------------------------------------------------------   0
BLSV       -----------------------------------------TFLAQ-----GASLKPRSV   58
PRMV       -----------------------------------------SFLAQ-----GASLKPRSV   58
CawYV      -----------------------------------------CQLYA-----GPQVEPRYL   75
SLSV       --------------------A------------------TEEVLYY-----GCAPRPAFL   81
PVU        ------------------------------------------------------------   0
BRV        -----------------QLVLENKYIAACL-SKPANFAL--SFLAQ-----GASLKPSVV   47
GBLV       -----------------FLAKERELVHQLL-RSSSSFAV--TFLAQ-----GASLKPIMV   49
BLMoV      -----------------FLIEESRLIRYVL-TSSSTFAV--TFLAQ-----GASLKPTVV   49
AYRSV      ------------------------------------------------------------   0
GSPNeV     ------------------------------------------------------------   0
GNVA       -----------AHKQPQPPVLRQCSAVVVQVHSNPALELVYPPLVDRTSAPCEREEPSNE   119
```

```
ArMV       -----------KV--------------LQP--TLK--AEVRVPIF-CAPKRMVAFP----   149
GFLV       -----------VV--------------LPP--TQK--VEVRVPVC-CAPKWMVAIP----   150
GDefV      -----------VV--------------LPP--TPK--VEIRVPVC-CAPKWMVAIP----   150
MMMoV      ---ASGCKAMAPF--------------GAVDAKVV--ESAPLPAW-AAPAWLRAMP----   161
HoNV3      ---SSGCSGVPTVC-------MLPVYMRPLGGRAV--VKQLKTKK-VTPSWLLEIRSLLR   93
PCMoV      -----------GS--------------KSPRSDIG--TAVPIPAH-LAPDWLKHEP----   139
AVA        ----------APV--------------RPKVVSEG--SAVPLSAA-LAPEWLKREP----   172
PBRSV      ----------------VKKSSAE-VLLEKRAAYQA----KH-AVP-TPGPVRVVKTA--A   193
TRSV       ----------------GKKSFNE-ALLEKRAAYQV----RT-AVP-PPGPIRVVKTA--A   177
AeRSV      ----------------EKKSLNE-VVLEKRAEYQA----RT-VVP-PPGPIRVVKTA--A   187
HoNV1      ----------AVQ--------------RPKILAEG--KACPLSVA-EAPAWLVPQL----   108
HoNV2      ------------------------------------------------------------   0
MMLRaV     ----------------------------------------------MGLLWADV------   8
RpRSV      ----------------ARKPSNG-GTARAPTGWFKPRVVRTQPAKKALPSWVSEARRLIK   158
BRSV       ---WEETLANWPVY-------PGTSL-LDFCRTQ------------------YEIRRE--   128
PoLNVA     ---WEETLANWPLF-------PGTAL-LDCCRAQ------------------FERRAE--   130
TBRV       ---WEETLANWPVY-------PGTSL-LDFFRTQ------------------YEIRRE--   128
AILV       ---WEETLANWPCC-------PGTSL-LDFCRTQ------------------YEIRRE--   129
RCNVA      ---WEETLANWPLC-------PGDSL-LAFCRTQ------------------YEIRQE--   128
GARSV      ---WEETLANWPIF-------PGTSL-LDFCRTQ------------------YEVRRE--   119
GCMV       ---WEETLANWPLF-------QGQSL-LACCRTT------------------AEVRRE--   128
PVB        ---WEPVLASWPTF-------GGSLL-NA-CQNC------------------HERMTE--   162
CNSV       ---FVEVIPTVPSC-------LAPKWMLGIQRVEG--APSKAPKQ-AVPKWVWQMRQLLK   126
ToRSV      PSAKQTSTAMEVVLSVGEAALTA-PWLLCSYKSGV----SS-PPP-PMTQ----------   155
AnNVA      ------------------------------------------------------------   0
CLRV       ----------------ALCAING-IVCRQSVESHS----NG-PSA--VAYW-----S--A   83
STeNV      ----------------ASAMIDG-ALMLSTSDFGF----ET-PAG-VSLTW-----G--D   85
BaNV1      ------------------------------------------------------------   0
BLSV       ----------------ALACANG-VIA-TGPGFCI----YT-PEG-IPLSWGD-------   87
PRMV       ----------------ALAVANG-VIA-TGPGFCI----YT-PEG-VPLTWGD-------   87
CawYV      ----------------QIAQSAD-VKA-AELAAKRQQIAAL-AKE-LALERAHEALL--D   113
SLSV       ----------------EAVLAAP-KLKMAPPVVDAMEVAYL-RGV-AAMEAEEKKLL--R   120
PVU        ------------------------------------------------------------   0
BRV        ----------------ARAAING-VIACDTTTFQL----LA-MDG-TPLSF-----A--A   77
GBLV       ----------------ARAIING-VIAIDTENFGF----VD-------QTW-----S--E   74
BLMoV      ----------------ARAIING-VVAIDTVNFGF----TT-ANG-EPLTW-----A--Q   79
AYRSV      ------------------------------------------------------------   0
GSPNeV     -----MKLSLASVCSFANVALPGNVMAEMPSKFAFSKFILE--NG----HIVEVRAS---   46
GNVA       ----------EVTV-LVPIRLAP-CWMRPQS---------------RPQKKRVLKHP--A   150
```

```
ArMV      ---KPPTKIASKRDALQFPAGAVAFNG--INFID----------AKGKVVLSEGAK----   190
GFLV      ---KPPVKLAPKASKLRFPKGAVAYNG--VNFID----------TKGKVVLSEGAK----   191
GDefV     ---KPPVKLALKASKLRFPKRAVAYNG--VNFID----------SRGKVVLSEGAK----   191
MMMoV     ---KQCTPAV--RPKTLFPKRAVAFNG--INFID----------ARGRIVLSDGAR----   200
HoNV3     ATLKESSSFGARYVRSSFPKARTLWAF--KMLTR----------NSPS-SVEENKMWKAS   140
PCMoV     ---KNKKKVSETFTPLAFPKGAHYYDG--RNYRT----------KSGKVILSAAAR----   180
AVA       ---VKVRPTTTKVEKSPFPKGAHHYDG--VNYRS----------KSGKIILSAAAK----   213
PBRSV     ----------TPVKVEKIQFPRGAVVYNGTNFF----------DAKGNVVLSAAAL----   229
TRSV      ----------TPVKVEKVRFPKGAVAFNGANFI----------DAKGYVVLSAGAL----   213
AeRSV     ----------APVKAERTKFPRGAVVYNGINFF----------DAKGRVVLSAGAL----   223
HoNV1     ---PKS---KGQQARRFFPKGAHYFDG--TFFRT----------KRGEVVFSDGTRLSQF   150
HoNV2     ------------------------------------------------------------   0
MMLRaV    --------------RSSWHLEDEASCLENLRAY----------LS-----PEAKRA----   35
RpRSV     GALEGSNAFGPRYCREKFPKARLVWVLGMLSKSSPPSVAIGRQLKKSFLALQARIA----   214
BRSV      AAEA-----SAEILRLKEVARQRAFDDE-VEFLIKHGAKVHFAPSFAAQLWRAGKDQKKC   182
PoLNVA    QAAA-----QKEILHLKEVERQVAFNAE-VKLLIAHGAKPEYAPSFARHIWRTGTEQKKY   184
TBRV      VAEA-----SAEALRLKKATRADAFADE-VKFLIKNGVLPHLAGDFARKIWSTGKDQKKT   182
AILV      AAEA-----SAEILRLKEVQRQEAFDAE-VKFLEKNGVLPAYSREFARKIWSAGKDQKKA   183
RCNVA     AIET-----AAEALRLKEAKRQLAFDNE-VKFLIKHGAMPELAPSFAQHIWKAGKQQKEV   182
GARSV     AAEA-----SAEALRLKEAKRQEAFDNE-VKFLISEGALPAYAPGFARKIWAAGKEQKKV   173
GCMV      AAEA-----SAEILRLKQVEREHAFQAE-VKSLIAAGALPACAEGLARKIWSAGKDQRKV   182
PVB       ER---------EYRRLAEERKQLYL-KE-LA------AKKA---AAARK------AARAK   196
CNSV      AALT-----GANSFGPRY-VRAHFSRAR-ISW-----IYAQLCEGCPLPLWNRGRALKKS   174
ToRSV     ------------------RQQFAAIKRRLVQ-------------K---------GQ----   171
AnNVA     ------------------------------------------------------------   0
CLRV      ----------LRAKLRSFLKGHGRWVTSLLA-------------K---------CA----   107
STeNV     ----------AARVVRAHRRAYGPVERRMFN-------------K---------CC----   109
BaNV1     ------------------------------------------------------------   0
BLSV      ----------AARRVKPFCRALAKSESGLY------------------------------   107
PRMV      ----------AARRVRPFIRALAKAESGLY------------------------------   107
CawYV     A---------NLLASRAFVARQEKKAVAAA------------------------------   134
SLSV      K-------------EKAAVVARAK------------------------------------   131
PVU       ------------------------------------------------------------   0
BRV       ----------AAVVVRQFQLAHNRFVKIGLRQF----------YKE--CTRRCNAF----   111
GBLV      ----------AARVVRRYSLAHSKYCRK-----------------V--RERSARRF----   101
BLMoV     ----------TARVVRQFQLAHTKFVAAGLRQF----------NKK--VDLSAKRF----   113
AYRSV     ------------------------------------------------------------   0
GSPNeV    -------------TASQLAEAYKVLCAKMER-------------RATYLALPIKEK----   76
GNVA      ----------N-ATLKAFGKLKPAVKLALLQ-------------RH--------GF----   174
```

```
ArMV      --RILKG--I--RVAKQQRQRTARRSAACKKVRKARDLA---LFKRLSE-----------   230
GFLV      --RILRG--I--RVAAKQRLRAARRSAACKKVRAKRALA---EFEAIVQ-----------   231
GDefV     --RILKG--I--RVATKQRLRAARRIVACRKVRAARALA---KFEAIAQ-----------   231
MMMoV     --KILKG--V--RALEKRQRKAAAIARRVERKKRAAMKA---AAAQKKQ-----------   240
HoNV3     SIKLLAKIAK--FKEAKQTKRSARRAAACRQSRLERFQV---RGLAMLA-----------   184
PCMoV     --QIKEG--A--RKLQAQKLRAIRRQVACKKVRIARLKE---LGKLLSQ-----------   220
AVA       --KLLAG--A--IKLHKQKLRSARRAAACRRVRMARYKE---EYALLSK-----------   253
PBRSV     --KILRGVKRLRSQAVR----AERRQRICKKIRLAAF----------AAK-VPELLRRAS   272
TRSV      --KILRGVQKLRRQQAR----SARRMAACRRVRLAVF----------AAR-LPSLLRKAD   256
AeRSV     --KILRGIKRLRQQQAR----SARRLAACRKARLARF----------EVM-VPGLLKKAS   266
HoNV1     KKKLVAA--A--TKARAQKARSERRQKVCRKIRMAHLKR---SAAALFA-----------   192
HoNV2     ------------------------------------------------------------   0
MMLRaV    --RYLERVSR-RRAAYR----AAYKKKAKKALKLRALVPLV-KQAQAAMR-LESLREIRR   86
RpRSV     -----------RALAKKQSARAARRQEACRKIRLALQQARGIAA-------------LER   250
BRSV      RGILLGKLNK--AKALGEAHRSAVARAQ----AKAEVLR---EFEPSPQ-----------   222
PoLNVA    RGILLRKLQL--IQDLSNAHRAAISRAQ----AKAEVLR---EFEPSPA-----------   224
TBRV      RTAFLVKIKK--ANQLKQQWNSARSLAA----ARAEVLR---EFEPSPQ-----------   222
AILV      RSALLVKVKK--GKQLKREWEEACFRAR----VRAELAR---EFEPSPH-----------   223
RCNVA     RKGFLIKLTK--ARALGNAHRSAVCRAQ----ARAEVLR---EFEPSPA-----------   222
GARSV     RKNLVTKIRV--AKTLGQRFRSAQALAA----AKAEVLA---EKEPTPA-----------   213
GCMV      RVAIRTKLTK--ANALGAAHRSAVATAQ----AKAEVLR---EFEPSPA-----------   222
PVB       RLAYRKRFYA--VRKLVIE---ARVQLI----ARAYLQLQAKPVPPTSA-----------   236
CNSV      SLALLARIED--TKQQKRAAWEKKE---------AAPLKSKREYEQKRALLIPLIEKLRA   223
ToRSV     --QIIRELIRARKAAKYAAFAARKKAAAVAAQKA-------------RAE-APRLAAQKA   215
AnNVA     ------------------------------------------------------------   0
CLRV      --EI------------F---ESRDL---CRYQRQ---------------R-AAYLARGAA   131
STeNV     --TA------------Y---LAKCAEKKERLHQM---------------R-AEYIVRFNR   136
BaNV1     ------------------------------------------------------------   0
BLSV      -----------QKMTEELAFRKFQRQVPHMVAASKRLSA--------RKQ-A---SRAKG   144
PRMV      -----------AQMGEELAFRQFKRQVPHIRAASKRLEE--------KKA-Q---LRAKG   144
CawYV     -----------TKQARKLAFEKREAKRASRKAAAAVLEKRALIAQATRKR-L---VVLRR   179
SLSV      --------------ARRAARKAAKKQVAEMKATVA-------LKLATRQK-TARLSAAKR   169
PVU       ------------------------------------------------------------   0
BRV       ---------NLQKAARQ----SRERRAACKRI-LASL----------DAD-LPCSRSSRR   146
GBLV      ---------AERKAARK----AAGVKAACKRI-LASL----------DAD-LPCSGRLMW   136
BLMoV     ---------ALKKSARI----AAGVRRSCKTI-LASL----------DAD-LPCNGRQRR   148
AYRSV     ------------------------------------------------------------   0
GSPNeV    --KAIR------RDRRQ----KRARQLSKQRKLLAA-----------RQA-ASLLLKEKK   112
GNVA      --EAI--VSALRANERR---------VKAEAARAAALKVR--AKIRAKKA-LRWLKRSAA   218
```

```
ArMV      ----------E-----------C---TFQDLPGGFAGEIPAGH-ACYRKVAA-------P   258
GFLV      ----------S-----------E---RLDQLKTGFQVVLPAPKMSCSLKEAA-------P   260
GDefV     ----------S-----------E---RLDQLETGFQVVLPAPKMSCSPKGAA-------P   260
MMMoV     ----------Q-----------I---LWAQM-QRAQAKAILKAL--ESEVQL-------S   266
HoNV3     ----------RQ---------EGKLGLYASI-YGKRVSVLSPSSSCVF------------   212
PCMoV     ----------Q-----------C---TYPLP-GGFAI-PLPPAQGCMAKTSL-------G   247
AVA       ----------T-----------L---TWESI-GSFAY-ALPPSHGCMGRSAT-------R   280
PBRSV     ----------------------------EATSGG--------------------------   278
TRSV      ----------------------------EATSGG--------------------------   262
AeRSV     ----------------------------EAQFGG--------------------------   272
HoNV1     ----------E-----------A---RLEELSSGFAVVLPQPKMGGVRLARK-------A   221
HoNV2     ------------------------------------------------------------   0
MMLRaV    ELE----------K--------------EVSIGSYV-TPPQIQKAFDSL-----------   110
RpRSV     QQA----------R-----------QLSGA--G---------------------------   260
BRSV      --------------------------------------QIQRALEAQIFADRLSRKYAAL   244
PoLNVA    --------------------------------------QIQSAIEAHIFAEKLSRKYADL   246
TBRV      --------------------------------------QIQKAIEAQLFAEKLGRKYATL   244
AILV      --------------------------------------QIQRAIEAQAFADKLGRKYATL   245
RCNVA     --------------------------------------QIQKAIEAQVYADKLGRKYANL   244
GARSV     --------------------------------------QIQSAVKAQAYSQALSEKWATF   235
GCMV      --------------------------------------HIQIAVKAHIFAEKLSRKYADL   244
PVB       --------------------------------------ELEEARNLYKEFQAYSLKYAQF   258
CNSV      RLL------QDEAR-------ELREQLFPSGNGGTDTTKVAAASKAEIKAAAQLKAYQDV   270
ToRSV     AIA----------K-----------ILRDRQLVSLP-PPPPPSAARLAAEAELA----SK   249
AnNVA     -------------------------------------PPPPRSACFLQAEDSLR----AA   19
CLRV      LRA----------K-----------ALKKR----KV-ALQKERATQLAQRQLEGERRAAA   165
STeNV     AMA----------K-----------KISGF----FE-RPPKPSANVLRQREVWAQKFTHL   170
BaNV1     -MA-------------------------SVKAILYS-PP--L-----SFSPIWENPYYTF   26
BLSV      --A----------A-----------TVAAR-----------SA----AATAELEARRAAG   166
PRMV      --A----------A-----------ATASR-----------KA----AIRAALEARRSCG   166
CawYV     --E----------R-----------AEKAR-----------KQ----ELAADLAARRAAG   201
SLSV      AMK----------A-----------KRKAA-----------KE----AARLALEERRAAG   193
PVU       ------------------------------------------------------------   0
BRV       AIV-------------------------TAHYAGVA-TAARKSALFRQM-----------   169
GBLV      AIK-------------------------RAHREDFA-SRMKTAEICRMI-----------   159
BLMoV     AII-------------------------KAHREATV-LAAQKSEIFKKI-----------   171
AYRSV     ------------------------------------------------------------   0
GSPNeV    KMAEAKAILRSIEREPTRAQQKVAAAFWRKVEATLP-PRPTEAQCLLAKYRAWRKRQD--   169
GNVA      GRK--------------------------------A-----------RLQRQWEL-----   230
```

```
ArMV      TTSFKKEVS-KGKKAKKPSTPVLPA---QDFSCVDS-----FDWGEKSS---PVE-----     301
GFLV      STTSVVVVK-KRKLP--RLPKILPE---QDFSCLEG-----FDWGEKSH---PVEVD---     303
GDefV     STTSVVVVK-KRKLA--KLPKAVPE---QDFSCLVG-----FDWGEKSH---PIETD---     303
MMMoV     SAQESLVREEIRAMRMASETMACKV---RAFKAHRAALVAERAAKAAEVQLLEQEGC---     320
HoNV3     -------KKK--KKGVTKVTPKVLDEDLFDWSSLPV---NSAVPAVNGDLTFDDDDC---     257
PCMoV     HKVVCPTKRQ--K-KKKQPLKAVPV---QKWTEEIEQLLAQYAEERRTSPIVEQPT----     297
AVA       KVDPSLLRRKLKK-RVKAVTPQPIA---QEWTPEVEALVSQFIAERNESLVFDDEDC---     333
PBRSV     ---------------------------------FRYNDLNAPKCAD-SVARPRFSRKGKK     304
TRSV      ---------------------------------FKYVDLNAPRVVR-KAEKRP------K     282
AeRSV     ---------------------------------FRYVDLNAPKSAN-LACKMPKK---KM     295
HoNV1     S----PLRLKARKTKVMSTATKTVV---QDFSILDDE----EFCSPRENLVFDDDDC---     267
HoNV2     ------------------------------------------------------------     0
MMLRaV    ----A------ALVAQRRQKRQMAAAAREREISLMVLALEAGL-----------------     143
RpRSV     ---------------------------------LYSVRLCTKRALS-PIVEVPK------     280
BRSV      TARVRAKRAAARELREKEL------------FLETQDLLNAPLLPP--------------     278
PoLNVA    TARVRARRAAARALREKTL------------ELELIDLFNAPVVQT--------------     280
TBRV      TARVRAKRAAARELREKQL------------YQETVDLLNASLLPP--------------     278
AILV      TARVRAKRAAARALREKAI------------YLEVQDLLNAPLLPP--------------     279
RCNVA     TARARARRAAARELREKAL------------YLETRDLLGAPLLPP--------------     278
GARSV     VAQVRARRAAARLERENAL------------GKEVCALLDSPLLPP--------------     269
GCMV      TAQVRARRAAARDLRAKEI------------YLEIVDLLGAPLLSI--------------     278
PVB       VATKRYQRRMAQEERAEKL------------FREVEALLKEPLRTV--------------     292
CNSV      CAKVWRVKRQEKKAQQA---------------KLVEDLITSANCGKQD------------     303
ToRSV     SASLQ------RLKAFHRANRVRP------------VLNNSFP-------SPPL-AC---     280
AnNVA     ALSLP------RLKVFHKAKAAASRAA-RLAESFQPGFFSSPP-------SPVL-SE---     61
CLRV      RRERE------ARKGLQTLR------R-KLA-----ALFCPPP---------PFPTTEW-     197
STeNV     AHDLQ------RKKALKRQR------Q-QSV-----VEFQTPE-------VTPLKTCQV-     204
BaNV1     S----------------SV-------G-ERVPPHPYVLPTSPR-------SPPFPSRQY-     54
BLSV      ATKKSALRRALKKKATKKVVSASAAMQ-RGEPTLG--------------SIFPFSSSL--     209
PRMV      GTNKSALRRSLKKKATKRVVTAALATV-RREPTLG--------------SIFPFSSSL--     209
CawYV     ATRKSALRRCLKKKAVRAAFAPAPLKE-RREPKLG--------------SIFPFSASL--     244
SLSV      LTRKSAFRRSLKKKATKMAGRKVPKTS-------T--------------STFPKVESFS-     231
PVU       ------------------------------------------------------------     0
BRV       -------------RADKRASRALAAA-------MQPLVLAPPTCAR-IPYVCPLSSS---     205
GBLV      -------------RASKREERAY-RAQ------YAALEVRRPVSMR-VPYVCPLVAN---     195
BLMoV     -------------RADKRAERARARLQ------AEALYVPAVRSIR-TSYICPLKKE---     208
AYRSV     ------------------------------------------------------------     0
GSPNeV    ----------------LAAARAAAAE------------IEAPK-----------------     185
GNVA      ATDGK------QLV--------------EVKDGFQIVL-AEPKMV--PAVVPAVKVDRVL     267
```

```
ArMV      ---------------IEDDWVLI-----EKP-------VLQRQ-------AAHSA-----    322
GFLV      ---------------IEDDWILV-----EKP-------VLKRQ-------AVQTA-----    324
GDefV     ---------------IEDDWVLV-----EKP-------VLLRQ-------AAHTV-----    324
MMMoV     ---------------IEDDWEMV-----PKT-------LPVRRV-----VAKASP-----    343
HoNV3     ---------------VVVERRRP-----V----------FK---TG--N-ARSDS-----    276
PCMoV     ----------------SMAAPKI-------A-------LRPCVKVG--KVPSVGT-----    320
AVA       ---------------VFVDVPRP-------A-------LPPREKVG--RIPVVET-----    357
PBRSV     TKSVQCTARSASPVQEEINWDDW------------------II-PESERTASPVKEEK-T    344
TRSV      KKPAKKAVRPASPVEEEINWDDF------------------II-PESERTASPMKEEK-P    322
AeRSV     KKTTKKVAGSASPVEEEINWDDF------------------II-PDSERTASPMKEEKKS    336
HoNV1     ---------------VIIS-----------A-------VPRRR-LS--R--EHLK-----    284
HoNV2     ------------------------------------------------------------    0
MMLRaV    -----------------LVTVPS------------------RG-RSMRRSVSPR------    161
RpRSV     -RHKKKAISLPSPVSVQEFPEGLVGPNFWETSGLLNCVASPQR-REDLVFPVYGI--DSC    336
BRSV      -----------------MEKVGI-----ERK-------YRKVRPTG--SNVTSTP-----    302
PoLNVA    -----------------VLAEPL-----RRT-------RRRR--NL--RWRSTQP-----    302
TBRV      -----------------MEKVEI-----ERK-------YRKVRPTG--ANVVHQV-----    302
AILV      -----------------MEKVEM-----IRK-------HRKVRPTG--SNVVHTP-----    303
RCNVA     -----------------KEKVEC-----LRK-------YTRRY-AR--VDIEHAP-----    301
GARSV     -----------------QEKVDL-----VMK-------AKKRSRATFEKTPVMQT-----    295
GCMV      -----------------PQQIKI-----KGK-------YLRRSVA---AEVEVPH-----    301
PVB       -----------------EPAPKV-----ARA-------AAREGR----RQQLHGI-----    314
CNSV      -----------------VSEPAI-----EKA-------ARPKRR----IEIGDFV-----    325
ToRSV     -----------------------------KP--------DPAL-LERLRLATPSRCTV-A    301
AnNVA     -----------------------------ER--------RREL-REKLHMAIPGHTTP-P    82
CLRV      -----GWDFL------------P-----SSP--------LPAY-RDFFTS---------P    217
STeNV     -----SWTT-------------------EGP--------VP-------------------    213
BaNV1     -----YVARNGTPLVEGSLGSRL-----VGP--------APRY---FVRGSVSGT-IL-R    91
BLSV      ----FERVS-VRPL-------GV-----PFN--------PPQR-EDFLSSSSPIP-----    238
PRMV      ----FSLPPIPRDK-------GV-----SFN--------PPQR-EDFLLSPPP-------    237
CawYV     ----FGEVR-PTFV-------GT-----PFD--------PPSL-LEGDILPPPP------    272
SLSV      ---CFPSSPLPAFIREEQPWVAV-----NTP--------CPHP-EGFDLAVDPAF-----    269
PVU       ---------MP----------HF-----WEH--------FPHP-EGVELDG-SME-----    21
BRV       --------------VEESVSRRS------------------LE-RCSKRVPREHA-----    227
GBLV      --------------REEVAGEPL------------------RG-RAFERTPRS-------    215
BLMoV     --------------EDE--PCVV------------------RG-RSFERTARRSA-----    228
AYRSV     ------------------------------------------------------------    0
GSPNeV    ----------------------------------------PQG-VSFDSQFSPSSGG---    201
GNVA      LRSSKK--KASSPLVRKEQDFSC-----LAD--------FPPL-RESVSVE---------    302
```

```
ArMV     -QGRATEALTRFAASGGFTVKAH-----------------------QKVEEL----ASS-  353
GFLV     -QGRATEALTRFAATSGFSLGAH-----------------------QKVEDF----ASS-  355
GDefV    -QGRATEALTRFAATSGFSLDAH-----------------------QKVEDF----ASL-  355
MMMoV    -LEKAISHVERMWYSAEYSSVLK-----------------------ERMQTE----WDS-  374
HoNV3    -WHNALATLE--WS-FTIPSIVK-----------------------VSVEGV----FQN-  304
PCMoV    -LSEAEFALESFEKESPFTVALK-----------------------SRLREI----WDT-  351
AVA      -VPQAIASLEAFWNTGCFTPAIK-----------------------SRVQEL----WDS-  388
PBRSV    KQPLVPLSLGFGWWR-PASGKLW--------TQVVHCMRAVKGTPIAVPCEK--FLCAAG  393
TRSV     KRSLVPNSLGFGWWR-PASGNLW--------DVVSQCQRACEGTFLEASAEA--CLVRAG  371
AeRSV    RQPLVPNCLGFGWWR-PASGSLW--------SSVLHCQRVCRGTFLAASAEA--NLVLAG  385
HoNV1    -PTSRVQNMEAFWNLPEVSVECK-----------------------QALQKL----WDE-  315
HoNV2    ------------------------------------------------------------  0
MMLRaV   -------STTPIVER-KVCGTLRLKEANPDLEWEGICDLPRE-TLVAAEP----------  202
RpRSV    RTDPEYFVVGPKSPS-KIMGDTK--------PRLPTCPAEALNLL-----------WASN  376
BRSV     -KPNVLENLCPFMGLGAKTADVRCQATLMAGKIHP-QYPRLASAIYAWVLGP----SMK-  355
PoLNVA   -EQNAFEKLCRAAKVNANLDDVRCQATLSAGSIYP-TYPRLASAIYAWVIGP----STK-  355
TBRV     -VANPLGSLCPYMGLGAKTADVRCQATLMAGKIHA-QYPRLATAIYAWVIGP----AAH-  355
AILV     -KRNALEDLCPYQGLGAKSADVRCQATLMAGKIHA-TYPRLATAIYSWVLGP----SMR-  356
RCNVA    -NVNPYVGLCPYMGLGARSADVRCRAVLVAGKVHA-NYPSLASEIYSWVIGK----ACN-  354
GARSV    -VGTAMDPLCPYMGLGARDADVRCWAISKAGTIAV-SHPRLASAIYSWVVGS----TRS-  348
GCMV     -TRNPMAELVPYKGLGAVSADPRCWVVAQCGKFSA-THANLCSEIYSMVIGP----WVQ-  354
PVB      -RQGILESL----NVSPGSKESSERAIVAVIAVAP-HYPRLASSIVSFLQEH----QVL-  363
CNSV     -PQKTLWGLYPCVGLGANMADPVCRVLSACVSIAG-KRPDLVSTIYAFITGE----AQV-  378
ToRSV    TKRQRDFVVAPLATQ-IRVAKCA---------SHQEAYDSCRSILIEEWPESRYLFGPLS  351
AnNVA    LFPTCKPSLKAFSFR-ALESSCT---------SHQEVYDRARRIIFEYSPAGPKLFNHLN  132
CLRV     LLE--EASVAPPPVG-PVGFNKS---------TPQALYVSVRARLATFSKATGALFPLVD  265
STeNV    ------------RDG-TLG-ALS---------HPAACYERAVKFVAEFSQATHELFPQMS  250
BaNV1    KLTLSKRSLTPLPRS------PI---------CARDHYLAARELIGSYSSSASLLFPIHN  136
BLSV     ----ACPGMGIAS-L-PLMG-RILSLAPQVEQLCELCAPGAGSSFSSAVAD------LAS  285
PRMV     ------PLMGYAKAH-FLMG-PIVDLGASLGPILEAGAPGAQAALFAAIQK------L-P  282
CawYV    ------PFVGLGHPL-AKMGSTIVELAPSLGEIFELGNPGSRASFYSAIER------LSS  319
SLSV     ----VAPIVGPIVSV-PFMA-PVVKAFNSLVEILAAINYEAAVDLRAYILE------IAS  317
PVU      ----VAPILGYLPSR-PFYA-DIVESANRCFPYLSAVSLEAAQDLRTYVLE------LAY  69
BRV      -SPRVAS-------------EMQ--------PFADRPRLFSRVLLLPLSEITRAFFSRVF  265
GBLV     --TSFSASPLPRMRS-PYEAEIA--------PLFGACDRASRLEL---HPDIKFFFREAP  261
BLMoV    -TSTLSSSPPPRVRS-PYESELF--------VLESRCRQAASLDL---PPEVKHFFFSAA  275
AYRSV    ------------------------------------------------------------  0
GSPNeV   ----RTQLVGWEKST-PAGV--A---FGHVLAAFKACHVSTSVVETLETPE--GLFFQAP  249
GNVA     ----HSFAPMEGWMR-KASTGARMLG--QPVRVTAETLVSARLTLRGEKCSE-QLLKVAD  354
```

```
ArMV      ----GEAG----HLIAGE-F-----AELCLRSLVYNDAPVLSA--SIEEL--ITE--QDF    393
GFLV      ----GEAE----YLMAGE-F-----ADLCLLSLVYNDAPTLSA--TIEEL--RDS--KDF    395
GDefV     ----GEAE----YLMAGE-F-----ADLCLQSLVYNDAPVLSA--TIAEL--KED--TDF    395
MMMoV     ----YPFK----RLAVGR-L-----CSAFLVAAKFTVPEVLEE--NLLMI--DDLTVANL    416
HoNV3     ----PKTENRTRDILVSM-I-----SSLEKVSRVYE-SIFLED---MFLL--WDPTYVEV    348
PCMoV     ----RSSL----RLEVGN-F-----CAYMQELADPEIFMDLEA--NFFAL--SRSEQEEL    393
AVA       ----SSCD----LVEIGR-F-----CAYLTELGDPEIAPLFEI--NFFAL--SAEEQTQL    430
PBRSV     AD--DT-----ALSVWAR-I-----SSSVVDLAAHYSFDILLE--NYTAL-E-GCTMDVL    436
TRSV      VD--DV-----ALSVWAR-I-----SKSVVQLSAYYDVNTLLE--NYTAL-S-ECTMDEL    414
AeRSV     TD--DE-----LLSVWAR-I-----SASVVDLSAHYPIPTLLE--NYSAL-S-ECSLEEL    428
HoNV1     ----TSTR----NDAA---Y-----AAYLVL-VRATLPVGIEQCSTKWLE--SFATPLGL    356
HoNV2     ------------------------------------------------------------    0
MMLRaV    -------------------L-----IARAIKIAQQTGIPQYANE-LLLEV-KGDAFLEST    236
RpRSV     LFEDWELD--IIGQMVSDGLLTSQAFLDGCTLVSYYGQEQMVD--SFHCLLQDPVPAE-V    431
BRSV      ----F-EC----IAPVKT-F-----IKGLTFMVDYFPEEVLID--ELNKI--N--SEARC    394
PoLNVA    ----L-NC----VGAITR-F-----VKGLTFMVDFFPEEALIA--ELKGI--T--TDERC    394
TBRV      ----F-EC----ITPVRN-F-----VKGLTFMVDFFPEEALIH--ELNEI--T--TEAVC    394
AILV      ----F-EC----VAPIKS-F-----IKGLTFMVDFFPEEVLIS--ELNKI--E--TEAQC    395
RCNVA     ----I-EN----PEYIRR-F-----VSGLSFMSDFYPEEALIN--QLRKI--D--DQHKC    393
GARSV     ----F-EV----SEPIRK-F-----IGNLTFLVDFYPEDALIA--ELGRI--T--TEAQL    387
GCMV      ----L-TD----FSSIRQ-F-----VMNISFLKDFYPEDALIA--SLKEV--N--TEAQL    393
PVB       ----LSKG----FAPFGI-F-----IDNICKLVRFYDEDQLIQ--SMSEI--K--DEARL    403
CNSV      ----W-LS----APRVCM-L-----AKRIIELSDWYPHELLAE--ELKKI--S--DEENC    417
ToRSV     FVGDWEHV--PGMLMQYR-L-----CVLFSMVRDVMPALSLVAD-TLHALRSGTAPNIVF    402
AnNVA     FSDGWKSV--PGMMESYK-L-----CVRFAMVLEVFNCCSIVSD-TYSSLMNGENCLDVY    183
CLRV      YD---RFC--PGHLMMFQ-L-----MHRLVAAYMACPVLSLVED-GLGALLRGDDYLFVF    313
STeNV     FSGDWKHV--PGLLVQYR-L-----ALAFEQAYKVMPTSSLVED-TLFALREGGGMVA-F    300
BaNV1     FD-DFPSC--DSIFFSVK-M-----CMKFARAYKACALNSLVED-TLTRLFKGDNSMDVL    186
BLSV      SS--TLHE--RD--VFFR-A-----MVAISQLFSAFPNAYILGN-FVGF---HFYNCEAI    329
PRMV      LS--TFHE--RA--LFRD-T-----QIAVSQLFVLYPSVHILGD-LNSF---FLQDCHGM    326
CawYV     AS--TFAE--IF--AFHR-A-----VVNISQLFVTYPCVYTVGD-FCGF---WDGSADEI    363
SLSV      YE--NFFA--LRRIFVGK-A-----LSWMALILPLYPYAGIFDT-YLPLDMVEDKNCDAY    366
PVU       AS--PH-----QLSRVSE-A-----LSWLAMLFPLCPLQGVVDS-YFPLQDVHGQDMCAL    115
BRV       ----VPGS--AMYDCATD-L-----AHVVLDWWSITPMAHYLA--MLDNF-LGTPTEESV    310
GBLV      NH--ASSL--GDVESLAR-L-----ARCTIAYFKLFPCALYFE--PLRRL-KPQSTTDEI    308
BLMoV     DI--SSSV--AHFEAISC-L-----ASYVCKYYARFPIPAYFA--QLRTL-SSDSSIKNV    322
AYRSV     ------------------------------------------------------------    0
GSPNeV    CLKDWGIA--F-------------------EAILPLCGH--LYDD-QALFALTKQLPTDKV    286
GNVA      E---VYAT--PSMPCHGK-L-----VCLLDDIAVLIGDSAYWD--SLPNEWH-SYSVEGL    400
```

X1-X2 cleavage site

```
ArMV      KDAI---ELFNIELAELP-T-----DSTTCGQFNDWASAAKKMAKGVGSIVG----DF--    438
GFLV      LEAI---ELLKLELAEIP-T-----DSTTCAPFKQWASAAKQMAKGVGTMVG----DF--    440
GDefV     ADAI---ELLKLELAELP-T-----DSTTCAPFKQWASAAKQMAKGAGALVG----DF--    440
MMMoV     NEIS---EGLEEEAQHFGPN-----EFEARAGIVNWI---KETAVSLAKLPK----NL--    459
HoNV3     CELA---KILAADAEAVS-D-----NLQARGFLRDFGRCAIDIVK---------------    384
PCMoV     AQIN---GVLKEECQHLEGV-----SVEARGGLFQFA---KQSAITVASKIR----NG--    436
AVA       ADIN---AALLDECISLEGL-----ATIARANMFQFV---KETASGLASVVK----TG--    473
PBRSV     QSVA---VQLDSEY---QEMG---PPTHHTCGLSSWARGAGKLMADFITPS---------    478
TRSV      QSVA---VQLDSEY---QELG---PPTHFTCGLSNWARGAGKILYNFVAPT---------    456
AeRSV     KGVA---VQLDSEY---QELG---PPTQYTCGLSSWARGAGKLVADFISPS---------    470
HoNV1     ADFI---SILKREVEDVEGI-----PMQLRAPFGKFINSATSIATGWMK--G--------    398
HoNV2     ------------------------------------------------------------    0
MMLRaV    REIS---EALDREERALEIS---------GSNLQGWWSALGDFSSKAASLS----GMVSK    280
RpRSV     AEA----LAVDVQALDF--------DAVFGCGISDFLRGTRDAMKGWI------------    467
BRSV      FEAS---LVLEEERAKLEAH-----AENANCRANIFMK--------AMAGVK----NM--    432
PoLNVA    EEAS---NVLDEERAKLEAC-----AADANCRANVFMR--------AAAGVK----NM--    432
TBRV      IGAS---MVLDEERAKLEAH-----AQSANCRANVFMK--------AMAGVK----NM--    432
AILV      FEAS---LVVEEERAKLEAH-----AENANCRANVFMR--------AMAGVK----NM--    433
RCNVA     LDAC---LALEEEKEKLDNA-----AANANCKANVFYR--------IAAGVR----KM--    431
GARSV     FAAS---LVLEEEKQKLRAY-----ASSVNCKANIFYK--------LAAGVK----NA--    425
GCMV      AVAA---IVTQEEVSKMEAN-----ARSANCRANIFKR--------IASRIT----SA--    431
PVB       QEAT---VVTSGELQLIEDA-----AQNSNVRASVFKR--------IAGKIK----NC--    441
CNSV      KEAE---REINLK--YLEIS-----KATENMRANGLFNKLKGKAQDLWSGIV----DF--    461
ToRSV     KNAM---STANQILECSHSS-------HAAQGFGNFLSR-GKSAAINLASGL----SSFV    447
AnNVA     SRAM---SASADILDCLTSS-------GTAQGFGDFLSA-GSRIATKFAKQA----TK-V    227
CLRV      KNMM---VAATQVVNKLSTCGRSAMQVQAGFGISNFFRGVGNSLASGFF-----------    359
STeNV     NRAY---ATAQQVVDIFAGN-----SSTNAHGFTDYMRKCGNYVKSTL------------    340
BaNV1     RDAC---DAADAVYNKFKKC-------NQLQGVPAFFSRTREALNNSFTASV----DYFA    232
BLSV      LNLV---EQANSMALYYICLF---KQGPIVHSFLDSFSDVASHISGAARI----------    373
PRMV      RAAL---ESAKRIADGISSIL---PQHQVVHTFLDAVKRVGSHISGAVSI----------    370
CawYV     AFAI---RRSKEVTATYALLL---NGNPIVHSFLDSIKSMGHTIGQVVRHP---------    408
SLSV      LASL---QQ---TAVMVLEVT---QGPMDAHSIFDIVRSVGSKVASIKDK----------    407
PVU       VNSL---RQ---TAMVFCKLQ---TGEQQTHTIFECMRSVAATIKQKKDS----------    156
BRV       RQAS---TNLLEEVEAMRALC----RDHRANGVFAWVTETAGTIG-----------STLK    352
GBLV      DKCA---NGLEMELDRLREIS---VSDLHANGLGTFIAGIAAGAATATGAV---VGTTVA    359
BLMoV     IRLS---SSLHGELMAFDQIF---KNDQIANGMGAFVAGLATGAVAAVSAVKVGAGALAS    376
AYRSV     ------------------------------------------------------------    0
GSPNeV    AIQMMNARFLEEKREFFESFG----NTSQCYGFFDAIKAFASRFHSTAEEVM----DVTE    338
GNVA      VAYC---ESLEQQMRVYRDTG------MDTQGL---------------------------    424
```

```
ArMV      ------------ARMSGAGVLITFDR-------CIEYLQKKALTFCQKVFNATMAPYLSH   479
GFLV      ------------TRAAGAAVVISFDM-------AVEFLQDKALKFCKRIFDVTMAPYLQH   481
GDefV     ------------ARAAGAAAVISFDM-------AVEFLQDKALKFCKKIFDVTMAPYLQH   481
MMMoV     ------------FCSAK------------------EKIEDMVISTMQSVFEKTMTPFLGH   489
HoNV3     -----------------AK-----------VAAGTDAIVSGLLKALRSSFDKTFAVYLDK   416
PCMoV     ------------ISWTA------------------DTLVEMVMKQLRKIFDNVLGHWLSP   466
AVA       ------------IHSVASKILESTSS-------GFDAVTTFVTDCVRKIFDKILGPWLAP   514
PBRSV     ----------------LNAMKGAANAVIDRAYLLTKDVIDGIFSQLKKLFYDSFGHLLGH   522
TRSV      ----------------VEGIAGAGCRIVERAYELSKTVIDEIFSKMKSLFYDCFGNLFGH   500
AeRSV     ----------------FERIAGIANGVLDKAYTLSRAVVDQIFAKMKSLFYDCFGHLMGH   514
HoNV1     ------------GC---ARVSALFNSAYEFSKGEAKKLADYMVDQYVKIFTETMAPFLSK   443
HoNV2     ------------------------------------------------------------   0
MMLRaV    AS------------------RSLATKAVDGAKHCWQSTLDGIVKMAISVFDGVFAKYLEN   322
RpRSV     ---------------------------MDPVIAKSTTWCNTIIDKVRALFDQYFAPFHKI   500
BRSV      ------------AKCAYSGFLTG-------CEEAGRSLSEGVCSVMINSFRECIKMIHKE   473
PoLNVA    ------------AKCAYSGFLTG-------CEEAGRSLSEGVCSVMIASFRECIRMIHRE   473
TBRV      ------------AKCAYTGFKTG-------CEEAGRSLAEGICSVMMRSFRECIAQIKTE   473
AILV      ------------ARCAYSGFLSG-------CEEAGRSLSEGICSVMVDSFRKCVEMVSKE   474
RCNVA     ------------ASNAYRGFLDG-------CEEAGRSVAEGVCHIMIRGFRECLVMVRTE   472
GARSV     ------------ACKAYGGFLEG-------CEQAGQKLSEGICSVMIKHFRDALTAVKHE   466
GCMV      ------------ACSVKNAFLDG-------CELTGKRLSEGVFSVVIGHFREALTTIKFE   472
PVB       ------------VSNAYSGAKQF-------CYDIGEAAAEGVFSVVMKCFHEMLKCVKQE   482
CNSV      ------------ASHPFRKYLAT-------AAEFVEGFSHRVVDAVMSRVNAAIAQFAAQ   502
ToRSV     GEKVV-------SG--------ANHVVNKASEVIVDKLFVPFVKLLREHFDDTIGKWIPK   492
AnNVA     GVDVA-------FGAAEVTADCASKVTRSVCDTVVYGAFIPMVKILRKEFDETIGKFIPK   280
CLRV      ------------EGARQSAID-AKDFIIDGINWTADKTVGAFVRALRDEFSNSVGKYLNA   406
STeNV     ---------------------------CSTVEAIGESFFAPLVRQMKRLFYDCLGQYLPH   373
BaNV1     -DKCA--------SAAEA----VIDYAADKTQTTAFLVWGPIVNQLRQVFDDTVGKFLPH   279
BLSV      --------------------------VGNVVKNFSSSIFDSILNKCESIFFKILSPYMAT   407
PRMV      --------------------------VKDKVSNFTSSLFDSILDKCKSCFMSTFSPFLAS   404
CawYV     --------------------RVALSTCANAISQAWHGTFEFILNKCHEIFFKIFAPHLAT   448
SLSV      --------------------------VVGCIVEKSAALLDTVIDTAKGMFFGVLGPYLAR   441
PVU       --------------------------FVSSASEWWYGMLDSILAKVRAPFVSILAPYLAT   190
BRV       TAAVA-------PFHGAG---VALKAVLTPCASATLAWGEKFFQTLKSKFFEFLKPYIQH   402
GBLV      GGKYL-------IDHAAAAFDKSCNKVVETVELATRKFGETMLDMVRKTFDSCLGPYLAT   412
BLMoV     AAKSA-------IDYGEAAFDRCSLKFVGTVGQATQQFGDTLLNLVYAQFDKCFAHLLSP   429
AYRSV     ------------------------------------------------------------   0
GSPNeV    GADYENPVPSTYERFCE-AAKASGRMVGTAATIAVDKMKERTWRAFMRLFDSTIGSYASA   397
GNVA      ------------FSSCRLGFECIAKYVGDGIKKWTGDVTEHIFSVVKREFYKIFEKFLDK   472
```

```
ArMV            LAEASNIISKIWKKLAEWMESLKGKAGLALEVLAQHAIFALGAIVVGGVVVLVEKVLVAC    539
GFLV            LASAHSILKKIWEKLSEWMESLKSKASLALEVMRQHAIFALGAMVIGGVVVLVEKVLIAA    541
GDefV           LASAHSIVKKIWEKLSEWMESLKSKASLALEVMAQHAIFALGAMVIGGVVVLVEKVLVAA    541
MMMoV           LTSCAELFRTFWDKCKAWIQKIRENLSDALLALQEHALWALVVMMAGGIVVLAETILMKL    549
HoNV3           LCDASRYLNDMWAKAKEWLCALKETCFCALAVLEECALWAFAITIASGIVILAETFLKQI    476
PCMoV           FKHLTEQIDAMWNKIKGWIVKVKDAMCIGIKVLQEFALYALAGLFVGAVITMAESALFQL    526
AVA             FKHLTEQINALWTKIKSWVQKIKESMPLALRVMEEHALFAFSAMVVGGVVVLVETTLQSL    574
PBRSV           LNVLLSTVDSFWQRASTWVMNILEKTHDAIKVLRDASVWSLLLILVGGMILLSERFLVSV    582
TRSV            LNVLLSTIDSFWARASTWIMNILEKTHDCLKVLRDSAVWSLLLILVGGLILLSERFLQSI    560
AeRSV           LNVLLSTVESFWSRATTWIMNILEKTHDAIKVLRDASVWSLLLILVGGMILLSERFLCSL    574
HoNV1           LREITGILNSMWSSIRSWVQELIENTGIALRSLGQHALYAFLATVVGAIVLLAEKVLCLL    503
HoNV2           -----SVLKSIWDSIREWAHRLLETTSLALQALGKYSMFAFLTTLAGAVVLLAEKVLVTI    55
MMLRaV          IPLVSNFVSDFWDKVRKWAQDMSSALGTVFEVIHEAALWALCIIVGAAIVSMVESVLVSM    382
RpRSV           -IDGMSYVNSLWAKCKEWAQSVLKNGSQLFSVMWETHCVSFVIIITCACTLLVENVLKEL    559
BRSV            LGCAMELIEVMIKKVKDWYNSMLEKLHCGLATLGTYAMYALAILLGCGLTTLLERCIG--    531
PoLNVA          LGCAMELIEVMIKKVKDWYNSMLDKLHCGLAALGKYAMYALAILLGCGLTSLLERCIG--    531
TBRV            LGCAIEMVEVMIKKVKDWFYSMLEKLQCGLETLGSYAMYALAILLGCGLTSLLERCIG--    531
AILV            LGTAIELIETMIRKVKKWFNDLLEKLHEGLATLGKYAMYALAILLGCGLTTLLENCIG--    532
RCNVA           LSGAMEIIEILINRVKSWYESLIKKLSDGAVALGIYTLYAICLLMGCGLIAVIAKCCG--    530
GARSV           LGIAMELIEVLISRVKAWYNTLLIKMGDAVATLGKYSLYAIAILIGLGLCRLIESCIG--    524
GCMV            LGVAMELVEVLIARVKSWFDTLLAKIDHALASLGKWACYALGILLGIGLCNLIETIIG--    530
PVB             LGLAHEFIEMLIKKVRAWYDNLIKKIGDAMYTIGVSGIIALMFLLCQMLTYSINKLLG--    540
CNSV            LDIAKTLVDQLVIHVKRWYTSLCTSFDDSLKLLGKWAGYALGLIVGVGVCHLVEVICA--    560
ToRSV           LLGATQKIEELWRWSLEWAQNMSKKLDVSLRVLRGSALVGVGLLLVSGILYFAEQLLRSF    552
AnNVA           ILDATAQIENLWRKSLEWATNMQAKLDVSLRVLKGSAFVGAGLLLVSGIIYFVEQLLQGL    340
CLRV            VSEYKAQIENFWAWAVRWSNNLMNKVDVSLRALQGSAFFAAALVIVGGIVYLIENLLPPA    466
STeNV           IVEVKEQIENFWRHAVKWVQNICGALDCTLKVLQGSAVIACAIVFIGGITYFVESLLQSL    433
BaNV1           IEQAKKLIAGFWPRLMNWSANLQDRLDITLRILGASAFIGAGLLLICALVAFVEGLLECI    339
BLSV            LHSSKVEIVNFWKKCKDWATNLWANAHLALQGLGMYAIWALVLMILCGIVYLLETMFITA    467
PRMV            LQSAKAEIEKFWQNCMSWARNLWGKAHLALQALGLYAIWALVLTILCGIVYLLESLFITA    464
CawYV           IHRAGEEIGKFWQRCKEWATNLWNKSHLALQALGTYAIWALMLTIMCGIVYLVETILISA    508
SLSV            AQKIGEEIKKFWDNCVEWATKLWQGAHIALQALGIYAVWAIVAMVLVGVMYILETILICL    501
PVU             AQRFKDEIENFWDGCVLWAKNLWKQTHIAVQALGAYAIWATIVMILVGIMYIIETMLMAM    250
BRV             AIYASAEIEKYWAFIHGWATKMWNNVGVELQALGDAAWWAIGITMVCGIVTLVEKLLVYL    462
GBLV            VSHAREQIETYWRRVTQWIKTMWSKLSIEVQALFDSTWWALGLILCAGVVVLAEHMLVAL    472
BLMoV           FTHARDIIANYWRAVREWIKKMWGNLSIEIQALFDSTWWALALIVVSGLVMLTEKLLVSL    489
AYRSV           ------------------------------------MWWASAMLLSAGIVAIIEKILVKM    24
GSPNeV          TTEAVSVVKIYLNKAKKWLDDALNCCNLAVQAVKNHVFTVLCLIIFSGVITMTENILFNL    457
GNVA            IAPVRNFIEMLWGKVKEWIHSLSTAADMFVDVMAQELRWVVALFLVCGMCLVVERALVSV    532
                                                            .
```

```
ArMV      KVIP--NCGIVLGAFLTLFFASLGLTALECTAEEI------------FRMHQCCKGAIYS    585
GFLV      KIIP--NCGIILGAFLTLFFASLGLTALECTAEEI------------FRMHACCKSAIYS    587
GDefV     KIIP--NCGIILGAFLTLFFASLGLTALECTAEEI------------FRLHACCKSAIYS    587
MMMoV     GVLE--RVGNVLGLFLTLFLTSLGFSAISLGADKF------------IALNNSFKMAVCT    595
HoNV3     GVLD--RVGITIGLFLTLMVTALGLKCASAGASKL------------GELYEMLKLSVIS    522
PCMoV     GVLA--KVGTTLGIFLTLFLTSLNLNALSMCTEQI------------AQLNEAFKVGIRI    572
AVA       GVIN--KVGATLGLFLTLFLTSLGLSGILACTEQI------------AELHRAFKVGVCC    620
PBRSV     GVIT--QPGTILGIFLATFLGIFGYTFFKKDDTLV------------SDLLWVFKTAITG    628
TRSV      GIIS--KPGTILGIFLATFLGIFGYTFFRKDDTLV------------SDLLCAFKIAITN    606
AeRSV     GIIG--KPGTILGIFLATFLGIFGYTFFKKDDTLV------------SDLLFVFKTAITG    620
HoNV1     GVTT--QCGGLLNLFLLAFISSIGVSACFMATEEL------------IACTDAVRKTVAG    549
HoNV2     GVTL--EVGSLLNLFLFTLIGAIGFSAVGMTTNQL------------CEMQSAIRLSVIS    101
MMLRaV    GIIA--MAGGAVGLFLTLFFSYLGVKAFLGGADKL------------SQICEVIKGAVCT    428
RpRSV     RLIS--RVGTLTSCVISGALGILGCGYILAKCEDL------------AVVSASIRAFLGV    605
BRSV      ------GAGILTKLFVTGVFAAIGLH-AAGGFDGL--------QREMVQMCTALAAGIFD    576
PoLNVA    ------GAGVLTKLFVTGVLAAIGLK-AAGGWDNL--------QREMVQLCTALAAGIFG    576
TBRV      ------GQGILTKLFITGVLAAIGLQ-AAGGFDNL--------QREMVQLCTALAAGIFD    576
AILV      ------GRGILTSLFVSGVFAAIGLQ-CAGGWDNL--------QREMVQMCTALAVAVFD    577
RCNVA     ------GGTALMGIFCTAFIAYFFSSADDEALAEL--------NRLLILSCTGLCTRIFI    576
GARSV     ------GAGMLSSIFAAAIFASIGLK-CAGGWDNL--------QREMIGMCTTMARSLFG    569
GCMV      ------GHGMLVSLFCTGVFATMAIK-CAGGWDAA--------QREMVAMITTLAQSIFG    575
PVB       ------VPD---PFFLMQIFSGLFF--AYSMWDNKLLQ--GAMRGEFIALINEFMRNFFS    587
CNSV      ------HMGLPLGGVITGVFTT-----AYMGWLFVKTPVGSELVMNLRMQVARIARNIFD    609
ToRSV     GLLI--VAGSFISMFVGGCLLAYAGSMAGIFDEQM------------MRVRGILCEIPML    598
AnNVA     GLMT--GTGTLLSLFIGGVLIAYNGGLSGIFDAQA------------TRVRGILCEVAMR    386
CLRV      A--TGLPIGSLSSLFVGGALLFWCGSELFQN-EKV------------MNIRVAIVTMAER    511
STeNV     GVISATANGTLLGLFLGSVIMWFCGFRVGVT-GPM------------LEMRAAMIRMAQA    480
BaNV1     GLCA--HRGTLVLLCVGGAIALFCGGITHFLSGSV------------SLIRRSIVDLALL    385
BLSV      GAIS--SHGLLVSGFLCLVMAACGYTVFAVGKESA------------QMI-RVMRECILM    512
PRMV      GVIG--SHGIILSIFLSVVMAAAGFTIFTVGKESA------------QMI-RTMRECILM    509
CawYV     SAIS--THGVLVSIFLSITMAAAGYTIFTMGRESA------------QLI-RTMREAILM    553
SLSV      GVMS--GHGVLVVALTGAVMTFLGFTVFQMGREYI------------HFL-TALRQIIFL    546
PVU       GILS--THGVLVSIFTGVLFAVLGYTVYTMGKEYI------------SFL-QTLRQIILY    295
BRV       GALN--AGGILCSLMLTGLLGAAGLLATGKFAEAS------------STLVGAMRSLIFT    508
GBLV      GVLT--HAGALTSLFITAFVGYLGWNFAAPATEAE------------GTLLTTLRALVHI    518
BLMoV     GVLS--HVGAITTLFMTGFMAYLGWNLASEGDSAE------------STLLRTIHALVHA    535
AYRSV     EIIA--YPGVLVGCFLSSILAYMGYTALDDNGAV-------------KMLMRTIKQSIIS    69
GSPNeV    GFIP--TKGNAASIFLTLALAFFGISKMSQNPGIF------------CEMQAHFRNLFAQ    503
GNVA      GILT--SVGSLTNLLLVTLIGGSMLCATTMVDEEL------------AHMAKVFKETVYS    578
                              .
```

X2-NTB cleavage site

```
ArMV     MYSVKEPMNE-------------------AE----------------GSSVT-MGVLQ--   607
GFLV     MYSVAEPTMA-------------------DE----------------GESHT-MGATQ--   609
GDefV    MYSVNESTMV-------------------GE----------------GESLT-MGAAS--   609
MMMoV    MLKPPELDSI--------VPGD-------VQ----------------NEFEA-RSLVG--   621
HoNV3    LLCGVDKQTRDQNHHNFGTPTQPETATSTGL----------------PRLQARNSVFD--   564
PCMoV    FLNVPA----DV------LGTS-------SR----------------NVVEA-RSLI---   595
AVA      MVRPNTNNSQNL------DVPG-------TE----------------NVAEA-RSLV---   647
PBRSV    LFRTKPGPPGSPII----------------------I-DGDVVIPEPATSMSTCSLLG--   663
TRSV     LFRTKPGPPGSPII----------------------V-DGDVVIPESAVAMSTCSFMG--   641
AeRSV    LFRTKPGPPGSPII----------------------I-DGDVVIPEPHHEMSTCSFLG--   655
HoNV1    LIYKGADPPV--------LPGD-------DY-----------------RMQA-RAPL---   573
HoNV2    MIRPDSSDFT--------VPGE-------VE-----------------NMTA-RADV---   125
MMLRaV   VMTRKPQSTSNFRD----------------------A---EVS--------QNAEGLG--   453
RpRSV    LLCPPTMEAV---------------------------DLNQSLIP---EEIQATSW-T--   632
BRSV     VHHKGNG----------------KYSPMADILAEQRLEDRR--------ADNVRS-IP--   609
PoLNVA   VQYTGRNNP---------VTGNRIFSPETDIIVDQAMEEMGVPTRPRYSAHNVRS-IP--   624
TBRV     IQHAGNG----------------KYKPSWDITAEHAREDAR--------DSNVRS-IP--   609
AILV     CQNTGRRNA----------QGQPIVSPIMDIHNAHHLEDRRT-RRMTDEQGNLRS-IP--   623
RCNVA    DNP--------------------DF--------------ADVVQREATEHTNVRS-IP--   599
GARSV    SESRANK----------------------------------------GLDGNLKS-IP--   586
GCMV     RRKGLD-----------------------------------------STDLNTRS-IP--   591
PVB      HAQSSK-----------------KWRPTK-------ADQEVLQRIEQGETSNIRS-IP--   620
CNSV     VQRTGIPPD---------LPANPNVGFSVPYEAFGGI--DNQPFSMGADVPNARA-IP--   655
ToRSV    LY-LKAQPDPFFPK-------------------------------KSGGRAPTQGLTD--   624
AnNVA    LY-AKP---------------------------------------KSMGGQTVQGVGD--   404
CLRV     MF-SKNKAV------------------------------------QSGNMTVQAGIQD--   532
STeNV    LF-QKPRDS------------------------------------MNASLT-AHSIGS--   500
BaNV1    MF-GNKVSKD-----------------------------------LPSSSSQVQSLGD--   407
BLSV     AVIPDDVVK----D------------------------IAAIPAP---GEQQVHSLLD--   539
PRMV     IVIPDDAAK----A------------------------IAE-AEP---DSQQVHSLFD--   535
CawYV    TVLPDDVTR----E------------------------VAR-SDP---DVQHVHSLLD--   579
SLSV     AVIPQGGLT----Q------------------------VNIMDS--SVDVTEPHSLLD--   574
PVU      SVIPDASVN----E------------------------AAGVTSPEIDGAQNAHSFFD--   325
BRV      LF-GSWKPT----------------------------EASDGLTC----------NAN--   527
GBLV     VV-EKPMSV----------------------------FRSDQTAN----------APN--   537
BLMoV    VL-DRAIGSG---------------------------SGADLNAN----------APN--   555
AYRSV    LYVQKETSPAEVRA----------------------IVHGDADMI----------AHS--   95
GSPNeV   CFATPTAPI---------------------------------------HEEGRSAAFA--   522
GNVA     VLCPG----ANFAA-----------------------NASH----VPSPREVAQSILDTH   607
```

```
ArMV            --GLDNAISALTRVGQSMISFKLGSFSYYAKIAQGFDQLARGKKAIGELTGWLIDLVGGV     665
GFLV            --GLDNAIQALTRVGQSMISFKLGSFSYYAKIAQGFDQLARGKRAIGELTSWLIDLVGSI     667
GDefV           --GLDTAVSALTRIGQSMISFKLGSMLYYAKIAQGFRPTCKGKKAIGELTSWLIDLVGSI     667
MMMoV           --GLDVAISALSTVGRSLCSLKFGTLMYWGKIGSAFDQLWRGKKAVEELGSWLVEIIGNI     679
HoNV3           --VVDWTTNALASVGSSMIAFHGGSLSRLAKMGVTLDQISKGRKALCELGSMLIGGLGKI     622
PCMoV           --GLDTAISALTGFGRSLVSFKLGTLQYYAKLGQAFDQLARGKKAIAELAAWTIDIIGNI     653
AVA             --GLDNAIAALTGFGKTLVSFKLGTLSYYAKMGQSFDQLARGKKAIQELGAWTIDIIGTI     705
PBRSV           --GLDIAIAAIGSVGSSILTFKMGTLQYAAKIATCLDQLRKGKDVLKEMTCWLIETLGQL     721
TRSV            --GLDIAIAAIGNVGASILGFKVGALQYAAKIATCLDQLRKGKDVLKEMTCWIIETLGAL     699
AeRSV           --GLDIAIAAIGNVGASILSFKMGTLQYAAKIATCLDQLRKGKDVLKEMTCWLIETLGQL     713
HoNV1           --GIDHALTAMSTLGTSLISCKLNTLAYAGKIGQSFDQVLRGKRAITELGAWMIEILGSI     631
HoNV2           --GIDRVLMSMKTLGNSLVGAKFGALQYAAKVGQCFDQVARGKRAIGELGAWIVETLGSI     183
MMLRaV          --PLDTAIRVVSALGSGIVNFKMGTLTYWAKVGSALDQLRKGKDVLKELASWLIEVLGRI     511
RpRSV           --GVDRVLGALNAVGSGLTGFNTDTIIYWGRFAQSFDGMRRGKDAVCALAACLFEKLGTV     690
BRSV            --IISGIISAMQQFGTGLCSMHSISLIEIGKLGAACHSMRMGKEALKEFCATIMYYLGRI     667
PoLNVA          --VLSSVLAALTQFGTGLCSMQSASLIEIGKIGAACHSMRMGKEALKEFCSTLMYYLGRI     682
TBRV            --IISGVIEALAQFGTGLCSMQSATLIEIGKIGAACHSMRMGKEALKEFCATLMYYLGRI     667
AILV            --VISGIINAMSQFGTGLCSMQSASLIEIGKIGAACHSMRMGKEALKEFCSTIMYYLGRV     681
RCNVA           --VISGIISAMTQFGTSLCNFQSLSLVEIGKMGAACHSLKMGKEAIKEFCGTLMYYLGRI     657
GARSV           --IISSVVTAMTTFGTSLCSLQGQSVVEIGKIAGACHQIRMGKEAIKEFAATISYYLGRL     644
GCMV            --LLTNVITAMTTFGTGLCKFQSSSIIEIGKLAGACHQMRMGKEALKEFAAMIMHYLGRI     649
PVB             --VVSGLIEAISAFGMGLCKFQSMSLLEVGKMAAALHQLRLGKEALKEFVSTILQCVGTI     678
CNSV            --VVSPIINAMAGFGASMLSMKAMGLIEMGKLGAACHSLRMGKDALCEFVSTVLYYFGRL     713
ToRSV           --VFGVPLSIMNAIGDGLVHHSLDTLTLMGKFGAAMDNVRKGITCMRSFVSWLMEHLALA     682
AnNVA           --IFGVPLNILNCIGDGLVHHSLDTLQLMGKFGSAMDNVRKGLMCMRTFVSWLMEHLAIA     462
CLRV            --IFGVPLTLMETIGSGLCGSALSSMTYVGKFGQAMDNIRKGVMCMRQFLGWMLEQLAEL     590
STeNV           --VFGVPCAVVEALGTGLVRTSADFLHYGSRWGAAVDNIRKGVVTMRQFLGGLLEHITFL     558
BaNV1           --LFGVPLKIVECIGSALAATSLDTLTYMGKWGTAVDNIRKGVTTMRTFVSWLLDGVADA     465
BLSV            --AAMVPIKFLDSLASGLSLFSTSSVTVLGKLGNSLEGIRKGYNCLADFISIFLNYTGVC     597
PRMV            --CAMAPVNFLESIASGLSLFSTSSITVLGKLGNSLEGIRKGYNCLTDFISIFFEKMGGL     593
CawYV           --VAMAPVNFLETVASGLSLFSTSSITVLGKLGNSLEGIRKGYNCLTDFLSIFFEKMGGL     637
SLSV            --TAMAPVHFLETVASGLTFFSSTSVTVLGKLGNSLEGIRKGFNCLRDFTSVLLEQFGNA     632
PVU             --TAMAPVHFLESIASGLSFFSSNSVTVLGKLGNSLEGIRKGYRCLTDFLSMLFDAFGNA     383
BRV             --ALDFPLKVLETVGTGLISAPLGTLQYIGKYGQAMDQIRKGKDAIKEFVGFCMDRVADA     585
GBLV            --VLEFPLRVLETLGNGLVSAPLGHSNMLGNMELLW-INPQGKDAMKDLL-MCFDCIADS     593
BLMoV           --ILEFPLRILETLGTGLISAPLGTLQYMGKYGQAMDQIRKGKDAMKEFIGFIFDRMSDA     613
AYRSV           --VGEVPLRFLNAIGSGLIAAPLGTLQYAGKYGAALDQIRRGKDAMKEFMGWTIECIGDA     153
GSPNeV          --DIYTPINMMTSLGNSLFSINTGTIAYFGKLGQSMEGIRKGINCVKDAASFAIESLASI     580
GNVA            SFNIFYPIELLSTLGEGLCKFNVDTVKYFGSLGNAIDGCRKGIVCLRDLMAKVIECAGQI     667
                         :  ..  :            .           *   :
```

```
ArMV        YSKVSGQESTFFDELSTIVCLDVRSWLLKSKRVRLQVETMAIGDRITLDTISKPTGMQGH    725
GFLV        YSQVSGQESTFFDELSTIVCLDVRAWLLKSKRVRLQVETMAIGDRITLDTIAKLL-EEGH    726
GDefV       YSTVTGQESTFFDELSTIVCLDVRAWLLKSKRVRLQVETMAVGDRITLNTIAKLL-EDGH    726
MMMoV       ADTLTGRHIEFFDELAATVGGDPKLWLKRAHDVKLQCQTMDLSGRMVLETVENLL-AEGQ    738
HoNV3       ADAITGRRLAFYEELSAVVQVDVPSWLSKTKSVLLKSHTLSLGDRSLLDDVTKLL-EEGN    681
PCMoV       YNKLTGRCSQFFDELSALVCCDVRMWLRSSQRVRLDALV-TPGSRNVLEIVEKLL-EQGN    711
AVA         YNKLTGRCSQFFDELSALVCCDVRMWLRSSQRVRLDALI-TPGSRNVLEIVEKLL-ETGQ    763
PBRSV       WNKVTGREATFFDEVSAIVAVDIREWLQESQDLCLAAQTFSIGDKIVLEQCERLI-ADGH    780
TRSV        WNKMTGREATFFDEVSAIVAVDIREWLEESQNLCLAAQTFSIGDKIVLEQCERLI-ADGH    758
AeRSV       WNKITGREATFFDEVSAIIAVDIREWLEESQNLCLAAQTFSIGDKIVLEQCERLI-ADGH    772
HoNV1       FEKVSGRQAAFFNDLSALVSVDVRTWLIRSHRYQAEACTTSLSSRETLEAVELLL-EEGH    690
HoNV2       YDTVTGSKNTFFNDLSTLVAVDAKKWLQLSSRYQMEACTTSLSDRHTLEMVEKLL-EDGR    242
MMLRaV      YDSVTGKESQFFDELSALVQVDVKHWLTQAQQVLLEAQTMALTDKVLLLSVSRLV-EDGN    570
RpRSV       YNRVTGKEAAFFHELSSLVSIDVQGWLNSSRRVMAESIAFAKSDAVAFATVERLI-NDGE    749
BRSV        SDKVTGRETVFFDELSTLVSVDVRGWILCAQSCIRESFHTEIGNQFFRDMVAQLV-DDGQ    726
PoLNVA      ADKVTGRETVFFDELSTLVAVDVRGWILRSQSCVRESFHTEIGNQFFRDMVAQLV-DEGQ    741
TBRV        ADKVTGRETIFFDELSTLVHVDVRGWIKRAQSCMRESFHTEIGNQFFRDMVAQLV-DEGQ    726
AILV        ADKVTGRETVFFDELSSLVKVNVRSWILQAQSCVRESFHTEIGNAYFRDMVAKLV-EEGQ    740
RCNVA       ADKVTGRETVFFDELSTMVSIDVRGWIRRAQGCMRESFHTEIGNQFFHDMVAQLV-DEGQ    716
GARSV       VDKITGRETIFFDELSTLVAVDVRGWIRRAQGCILESYHTDPGCTAFAEVVARLV-DEGH    703
GCMV        ADKITGRETIFFDELSTLVSVDVRGWIRCAQGAILESYHTDPGCETFQDVIGRLV-QEGQ    708
PVB         ADKITGRETTFFDELSTLVSVDVRGWLNRARGILMEGNYTDPGNPVFATVVNKLV-VDGD    737
CNSV        ADKVTGRETEFFDELSILVQIDVKDWITRSRGVLLDSCYTSLNNMICSDVVNKLV-TDGE    772
ToRSV       LDKITGKRTSFFRELATLINFDVEKWVRDSQQYLLAAEIYVDGDTVVMDTCRHLL-DKGL    741
AnNVA       LDYITGKKTAFFKELATLIHVDVEKWVKDSQEFLLSAEIMADGDRVILDTCLHLL-SKGQ    521
CLRV        YDNVSGRKVAFFRELATLAQVDVEKWISDVQEFLLVAEVAPEGDRVILDTVLLLL-NKGH    649
STeNV       YDKVTGKQAAFFRELTSLVYVDVEKWVKDCQEFLLTAEILPEGDRVVMDTTLELL-EKGN    617
BaNV1       FDIYSGRRAAFFRELSATLQYDVSSWIEKAQAFVLQVDIMPEGDRIVLDTSRKLL-TVGQ    524
BLSV        WEAVSGKKTSFFRDLATTVKINVANWTEDARRLIEYHEMAGLLDKFEYEKVRTLI-YQGE    656
PRMV        WEGISGKQTTFFHDLTTAVKINISSWTQDARRLIEYHEMAGTLDKFEYEKVRTLI-YQGE    652
CawYV       WETCSGKRTSFFRDLTTTIKVNLTSWTQDARRLIEYHEMAGSLDKYEYEKVRTLI-YQGE    696
SLSV        FEYISGKRTSFFRDLACAVKVDIARWTQDARETIEYFEISGSLDRYEYYRARELI-YQGQ    691
PVU         FEHVSGKRTSFFRDLACAVKVDVKKWTEDARKLIEYFEIAGSLDRFEYYRVRELI-YQGQ    442
BRV         WDYMTGRKDSFLREIASAAKVDIVYWIKQTQSVLLQAQTIAVTDIVLLDTVTHLL-YKGQ    644
GBLV        WDYMTGRKDSFFREIASMTKVDIVPWIKSAQQIVLEAQTVAVTDPVLMDTITHLL-YKGH    652
BLMoV       WDYMSGRKDTFFREISSMTKVQIVPWIEKSQKLVLEAQTVAVTDPVLMDTVTHLL-YQGH    672
AYRSV       WDMYSGRRDTFFHEIARLTKVDIVKWIRDSQNIILQSQTAANTDPLLLEMCTRAL-SKGW    212
GSPNeV      YYSATGKDVEFFAEIAALTRHDLKSWIKRAEKALLEFEVMKIRDRGMLDAMTNMI-HEGQ    639
GNVA        WDKITGQKATFFNEVSNLLRVDIGKWIERSQRLLQEAVLLPTTDCVLKDVCEALV-DQGI    726
                :*    *  :::     :   *                                *
```

```
ArMV            KILITAAGV---PRKTSADFTMCIKEEVSKLEEVHQRTACAGINEGMRQFPFWVYIFGAS     782
GFLV            KILVTAAGV---PRKTSADFTMCIKEEVSKLEEVHARTACAGINEGMRAFPFWVYIFGAS     783
GDefV           KILVTAAGV---PRKMSADFTMCIKEEVSKLEEVHARTACAGINEGMRAFPFWVYIYGAS     783
MMMoV           NLLVGISGV---PRRTSTDFGMIIKKQVEELIELRSRCAKAGKFEGTRVFPFWVYVFGAS     795
HoNV3           KLVIDAAGV---KRQTSLDYSLQIKSVTDQLSDLHARCVRAGTYRGTRTFPFWLYVSGES     738
PCMoV           KLKIGAAGV---PRKFSLDFTNVLCKEVEKLEEVRNQLANAGAYKGTRFYPFWVYVVGPS     768
AVA             KLKIGASGV---ARKFSLDFTNTIAKEVEKLEEVRNQLANAGAYKGTRFYPFWVYVVGDS     820
PBRSV           KLLRGMGDA---DRKLSASFLSTIQRKVSDLEKIHTQSVRAGYFEGRRMEPFWVYIHGPS     837
TRSV            KLLRGMGDA---DRKLSSSFLSTVQRKVSDLEKIHTQSVRAGYFEGRRMEPFWVYIHGPS     815
AeRSV           KLLRGMGDT---DRKLSSSFLSTIQRKVTDLEKIHTQSVRAGYFEGRRMEPFWVYIHGPS     829
HoNV1           KLRDGLAGM--N-RSRSHDFLLVVSKEVQALEALVFQAARAGVYRGTRTYPFWVYLEGPS     747
HoNV2           KLQLGLAGG--NPRRKSHEYLALINREVDALSKLVQQAAHAGPSDPTRTYPFWVYLEGAS     300
MMLRaV          KLLLGVSGI---PRKLTMDFFTLINKVQTDLKKIHEQCVKAGRFEGRRHTPFWLYLYGPS     627
RpRSV           TIQLTAASA---PKSHSMQFGQILAERLRELRTLRNDMAHAGSFEGRRCVPFWLYIYGPP     806
BRSV            KLQVGVNGI---PRKISTDYSQLIGQIMKDLMELHKRTIRAGISEGRRCEPVWIYLFGQR     783
PoLNVA          KLQLGVGGI---PRKISADYGQLIGMIMKDLMELHKRTVRAGITEGRRCEPVWIYLFGPR     798
TBRV            KLQIGVNGI---PRKISADYSQLIGQIMKDLVELHKRTIRAGISEGRRCEPVWIYLFGQR     783
AILV            SIQMGVNGL---PRKISADYGQLVGQIMKDLLELHKRTIRAGISEGRRPEPVWIYLFGQR     797
RCNVA           RLQVGINGI---PRKISADYGQLIGSIMKDLLELHKRTIRAGIAEGRRREPVWIYIFGPR     773
GARSV           KLQVGVNGI---PRKISADYATLIGTIMKDLIELHKRTVRSGCTEGRRKEPCWIYLWGQR     760
GCMV            KLQVGVNGI---PRKISADYASLIGQIMKDLLELQKRMMRCGTVTGRRKEPVWIYLWGPS     765
PVB             KLQHGINGV---PRKISCDYASLVGSVMKELRELQKKICRSGCTEGRRREPAWIYVWGKR     794
CNSV            QIASNIAGT---PRRLSLDFGQLVSSIMKDLLDLQQRIVRHGVTVGRRKEPTWIYIFGPS     829
ToRSV           KLQRMMVSA---KSGCSFNYGRLVGDLVKRLSDLHKRYCASGRRVHYRLAPFWVYLYGGP     798
AnNVA           KVQSMMAAT---KSGTSFNYSRIVADLVKRLNDVYKRFKIAGRKVLYRQAPFWIYLYGGA     578
CLRV            TIQRLLCQT---KQGTSFNYGRLVATLVKSLDDVYTKYTKAGRRVMYRFVPFWAYFFGKA     706
STeNV           TIQRVLCEHTPATRGVSFNYGQLVAGLIRRLNDVYKIAQKCGRKALYRKVPFWVYFYGEA     677
BaNV1           DIQRCLAGT----KGGSIAYSRLVSDMVHKLTMIHQKFAIAGRNVRYRKTPFWVYIYGQA     580
BLSV            EMVDIANKG--RSSHTSTSFLRTVGSLLNDLRDVRAKCARSLRFDGWRRQPFWVYIFGAS     714
PRMV            EIVDTANKG--RHSYTSNQFLRVVGSLLTDLREVRAKCARSLRFDGWRRQPFWVYIFGAS     710
CawYV           EIVDTANKS--RASHTSNQFLRTVGALLTDLKEVRSKCARSLRFDGWRRQPFWVYIYGAS     754
SLSV            DLIDTANKA--RSSHTSVKFLRTIGKLVEELKEIRAKCARSLKFPGWRRQPFWVYCFGES     749
PVU             DMMDAADKG--RSSQTSSNFLRTVGKLVENLKDVRAKCARSLKFPGWRRQPFWVYTFGES     500
BRV             ILQLTLAKA---SRTTSLDYARIVSTLIGELTKIRATCARAGSFDGRRPEPFWCYIYGKS     701
GBLV            ILQRTLAGA---KRATSLDYGRVVSSLVQELTKIRAQCARAGIFEGRRCEPFWTYIYGPS     709
BLMoV           VLQSTLAGA---KRTTSLDYGRVVSALVVELTKVRAQCARAGIFEGRRCEPFWVYLYGPS     729
AYRSV           KLQQSLAKG---KRTTNVDYGFIVGKYVTELQSIRARCAMAGKMEGRRKEPFWVYIYGES     269
GSPNeV          ELRHSMLDH-AGKSALGANYMRITSDLLKKIEEKRAIVAKMGDPLGRRLAPFWLYIYGPS     698
GNVA            QLKVGLAGM---PRKTSMDYGTVVSGILDKLNVLLVQCQKNAAFVGRRKEPFWVYIWGPS     783
                 :                 :          :                *  * * *  *
```

```
ArMV      QSGKTTIANSVIIPSLLEEMNLP--KTSVYSRPKTGGFWSGYARQACVKVDDFYA-IE--   837
GFLV      QSGKTTIANSIIIPALLEEMNLP--KSSVYSRPKTGGFWSGYARQACVKVDDFYA-IE--   838
GDefV     QSGKTTIANSIIIPELLDEMNLP--KSSVYSRPKTGGFWSGYARQACVKVDDFYA-IE--   838
MMMoV     QSGKTNFANSMVAPELLAEMNLP--RDSIFTKGKSDAFWSGYCRQSCIMIDDMFA-VK--   850
HoNV3     QSGKTQFVNHFAIPELLEQLGLP--RHSQFSKSKVDPFWSGYARQSAVKIDDMYA-VH--   793
PCMoV     QCGKTNIVSQYLAPGLLDKMDCA--LDSQYSKGKQDAYWSDYKRQALVKVDDMFA-IK--   823
AVA       QCGKTNVVSQYLAPGLLDKLDCA--LDSQYSKGKQDAYWSDYKRQALVKVDDMFA-IK--   875
PBRSV     HCGKSLLMEPMS-RELLKAGGFS--EASIYTKNSCDKYWSRYRRQACVQIDDLSA-GK--   891
TRSV      HCGKSLLMEPMS-RELLRAGGFS--ESSIYTKNSCDKYWSRYRRQACVQIDDLSA-GK--   869
AeRSV     HCGKSLLMEPMS-RELLRAGGYS--EASIYTKNSCDKYWSRYRRQACVQIDDLSA-GK--   883
HoNV1     QCGKTNFMNNLLIPELLDEMRIA--KDSVYSRCARDEFWSGYRRQTCVKIDDMYA-TA--   802
HoNV2     QCGKTNFVNTLLIPQLLEELNCA--QSSVYSRNPNDAYMSGYRRQACVKIDDLYA-VV--   355
MMLRaV    HCGKSLLMEQAA-DVLLTEAGYPLSSNSLYTKAATDAFWSGYRREYCVMLDDLSAICS--   684
RpRSV     GVGKTTTMHEFS-QALLTAFEFP--SDSLTSKSATDKYWSLYRRQALVQIDDLGAI----   859
BRSV      HCGKSNFMATLD-NALAKHFGLP--NTTAYRNCKDSFF-SGYSGQTFFHVDDLSS-VK--   836
PoLNVA    HCGKSNFMANID-NVLAKHFHLP--NTTAFRNCKDDFF-SGYSGQTFFHIDDLSC-VK--   851
TBRV      HCGKSNFMSTLD-NALAKHFNLP--NTTAYRNCKDAFY-SGYSGQTFFHMDDLSS-VK--   836
AILV      HCGKSNFMSTLD-NELSKYFGLP--NTTAHRNGKDQFF-SGYNGQTFLHIDDLSC-VK--   850
RCNVA     HCGKSNFMSSID-NALAKYFGLP--NTVAYRNCRDNFF-SGYSGQTFFHIDDLSS-VS--   826
GARSV     HCGKSNFMSELD-NRLSAYFDLP--NTVANRNAKDQFF-SGYMGQTFLHVDDLSA-IR--   813
GCMV      HCGKSNFMDVLG-MALCKHFDLP--YTVCGRNVKDSFF-SGYMGQTIMEIDDLSS-IK--   818
PVB       HCGKSNFMSELG-IRLCQHFDLP--YTVANRNIKDSFY-SSYLGQTIMQIDDLSA-TK--   847
CNSV      HCGKSNMMDHLT-SEVCRYFDLP--YTYIARNGQDNFFTTGYKRQTVLQIDDLSC-VE--   883
ToRSV     RCGKSLFAQSFM-NAAVDFMGTT--VDNCYFKNARDDFWSGYRQEAICCVDDLSS-CE--   852
AnNVA     RCGKSLFAQRFL-NAATEYMGTT--VDNVYYKNARDDFWSQYRQEAIVCIDDLSS-VE--   632
CLRV      RTGKTIFANNFK-NTMVQYLGTT--SENIFYKNARDQFWPKYRQQAIVIVDDLSA-VE--   760
STeNV     GCGKTLFSQTFV-NALTAHLGTT--SDNVYSKNSRDQYWSKYRQQRVVIVDDLSA-TE--   731
BaNV1     GCGKSIFLNKLT-SFFCTHLGTV--EDDVYSKNARDQYWSRYARQSIVTIDDLSS-VDPG   636
BLSV      QCGKSTLANYLA-PLLLTHMGWD--AHDVYSKDPTESYWSGYYQQKCLKMNDLSA-VIPR   770
PRMV      QCGKSTLANYLC-PLLLAHMGWD--AHDVYSKDPTEGYWSGYYQQKCLKMNDLSA-VVPK   766
CawYV     QCGKSTLANYLA-PNLLTHMGWD--AHDVYSKDPTESYWSGYYQQKCLKMNDLSA-VVPK   810
SLSV      QCGKSTLANYLM-PLLMSHMGWD--PQDVYSKDPVDGYWSGYYQQKGLKINDLSA-VNSK   805
PVU       QCGKSTMANYLM-PMLLTHLGCD--PNDVYSKDPVDGYWSGYYQQKGLKMNDLSA-VVPK   556
BRV       HCGKSLFMEDVS-RALLKENGHA--PNDIYAKNARDSFWSGYLQHACVQVDDLSA-CV--   755
GBLV      HCGKSLFMEEVT-RRLLKENGHA--LNDIYAKNARDPYWSGYLRQAAVQIDDLSA-CV--   763
BLMoV     HCGKSLFMEDVS-RRLLKDNGHA--PNDIYAKNARDSYWSGYLRQACVQIDDLSA-CV--   783
AYRSV     HCGKSLLMEPII-DALTDELGLP--AGDTYAKNGRDNFWPGYTRQAVVTVDDLSA-TI--   323
GSPNeV    HCGKSNAMNAFA-TDLLAEIGAP--PTDILTVTPTDKFMSGYERQACIMIDDLGATEV--   753
GNVA      HCGKSNYMDTIT-RELLDDIGRP--RSSIYPKTAGDSYWSGYLQQAAIQIDDLHSVQI--   838
            **:                                :   *  .    ::*: .
```

```
ArMV        QTPSLASSMIDVVNSEPYPLDMAYLHEKGM-SMDSPLVVTTANTVKPPTNAGITDEASFF   896
GFLV        QTPSLASSMIDVVNSEPYPLDMAYIHEKGM-SMDSPLVVTTANTAVPPTNSQVVDLPSFY   897
GDefV       QTPSLASSMIDVVNSEPYPLDMAYIHEKGM-SMDSPLVVTTANTVVPPTNSQVVDLPSFY   897
MMMoV       VEPSIESQMIDVVNSQAFPLNMAYIEDKGM-LMDSPIVVTTCNEEKLPSDSGVRDEPSFY   909
HoNV3       TEPTLAAQMIDVVNSEEFSLNMAAIEDKGM-LFDSPLVFTTSNSLHPPSDSGIIDLPSFY   852
PCMoV       D-ADIEPMMIDMVNSEPYPLNMAALADKGR-LFDSPLVVTTCNDLHPPSDCNLRDAPSFY   881
AVA         T-AEIEPMMIDMVNSEPFPLNMAALADKGR-LFDSPLVITTCNDLHPPSDCDLRDKPSFY   933
PBRSV       TDPSLETQLINLVASKEVPLDMAEVEDKGI-LFDSSILVTSSNTSSVPTNANINHVSAYN   950
TRSV        TDPSLESQLINLVASKEVPLDMAEVEDKGI-LFDSAILVTSSNTAHVPTNANVNHAEAYK   928
AeRSV       TDPSLESQLINLVASKEVPLDMAEVEDKGI-LFDSAILVTSSNTAHVPTNANVNHMEAYK   942
HoNV1       NKPSQESEMMHIVNSEPYPLNMAHLEDKGM-LFDSPLIVTTANALHPPTDAGIRDDASFY   861
HoNV2       TQPSAESLMIGLVNSEPLRLNMSDNNEKGM-FFDSPLIVTTCNDLTLPTDANVRDAPSFY   414
MMLRaV      NGVSLESEMLNIVSSQEYKPNMPFEGEKGM-YFDSPIIISSSNVFTAPTSANLLDEAAYN   743
RpRSV       SESGMEQEMMNIVSSATYNPTMAVANEKTT-LFDSKFIVSTSNNYSAGTDAKIHDRKAFN   918
BRSV        LDPPMEAEMINLVSCQEVPLNMADLADKPI-YFRSPFIISSSNFEDVPAGCGVRDIEAYR   895
PoLNVA      LDPPMEAEMINLVSCQETPLNMADLADKPI-YFRSPFIISSSNFEDAPAGCGVRDAEAYR   910
TBRV        LEPPLEAELINLVSCQEVPLNMADLADKPI-YFRSPFIISSSNFEDVPAGCGVRDIEAYR   895
AILV        LDPPLEAELINLVSCQDVPLNMADLADKPI-YFRSPFIITSSNFEDLPAATGVRDVEAYR   909
RCNVA       LDPPMEAELINLVSCQEMPLNMADLADKPI-YFRSPFIISSSNFEDVPPGCGVRDIEAYR   885
GARSV       TDPPMEGELINLVSCQDYPLNMADVADKPI-YFRSPFIISSSNFEDVPAASGIRDVEAYR   872
GCMV        TDPPMEGELINLVSCKDYPLNMADVADKPI-YFKSQFVISSSNQEDVPAGAGVRDISSYR   877
PVB         TEPAFEGEMLNLVSCQEHPLNMADLADKPI-YFRSDFVITSSNLEDVPPSC-VRDVEAYR   905
CNSV        NVPPIERELINLVSCSEYPLKMADLSDKSI-SFQSPFIISTSNQRTCLPTCGITHCEAFN   942
ToRSV       TQPSIESEFIQLITTMRYGLNMAGVEEKGA-SFDSKMVITTSNFFTAPTTAKIASKAAYN   911
AnNVA       TTPSIESEFIQLITTMRYGLNMAGVEEKGA-QFNSQMCITTSNFFTAPTTAKIHDMNAYN   691
CLRV        NPPSLESEFIQLMSTMPYGLNMAAVDEKGS-EFNSKMVITTSNKYTAPSCAKIHDMDAFN   819
STeNV       GQPSLESEFIQLVSVTPYALNMAAVDEKGA-EFNSDVIVTTSNVFTAPTMAKITDKAAYN   790
BaNV1       SAPSLESEMIQLVSTNQYGLNVAACEGKGM-NFDSNILLTTSNFFDAPESAKIHNKEAYL   695
BLSV        NNVPLEQQLIPLISTEEKMVSAAECEGKGI-QFLSEIAISSSNVEDAPTAAELIDGNAYR   829
PRMV        QVSPLEQQLIPLISTEEKMVSAAEIDGKGI-QFLSELAISSSNVNDAPTSCEILDPEAYR   825
CawYV       NVTPLEQQLIPLISTEEKMVSAAEITGKGI-QFLSEIAISSSNVDDAPTACEILDADAYK   869
SLSV        DVAPLEQQLIPLISTEEKMVSSAEIEGKGM-QFASEIVVSSSNFNDAPTGAEILDADAYR   864
PVU         NVTCLEQQLIPLISTEEKLVSSAEIEGKGV-QFLSEVIVSSSNVSDAPTGAEILDKDAYR   615
BRV         TRPSLESEFLQLVGSKMYSLNMAAVEDKGM-SFNSSIIVTTANVYTAPTSAEITDKDAYG   814
GBLV        TEPSVESEFLQTIGSKDYKLNMAAVEDKGM-SFNSSLFITTSNVFTAPTDARILDKNAYN   822
BLMoV       TEPSVESEFLQTIGSKDYKLNMAAVEDKGM-SFNSSIFVTTSNVFTAPTDAKILDKMAYN   842
AYRSV       AQPSLESEFMQLVGSKPYALNMAAVEEKGM-LFSSRVIVTSSNFFDAPTKAEIQDRVAYQ   382
GSPNeV      AEGGIEREIINMVSCVDYRPLMADVESKGM-IYDSPLLITSSNFYTAQPG-IFNDRQAYE   811
GNVA        LENSIESQLLQIVSTKEAYLNMADLPSKGTTLYNTPLIITSSNVREPPTNCNLLDETAFN   898
                    ::  :              *      : . .::.*         .    ::
```

```
ArMV          NRRAAVIEVRRKDNT---------------------HFTPRAYDNCIEVRFLHNKCAYVD     935
GFLV          NRRAAVLEVRRKDGS---------------------FFTPRAYDSCIEVRFMHNKCPYVD     936
GDefV         NRRAAVLEVRRKDGG---------------------IFDPEAYDSCIEVRFMHSKCPYVD     936
MMMoV         NRRGVVVECRRVEGS---------------------KYNPGNLEACAEVRLLQPKHQ-AK     947
HoNV3         NRRGAILEIRIKEGT---------------------KYDPEDITTCAEGRLLHPKHPKKS     891
PCMoV         NRRAVVLQMRRKEGT---------------------EYDPADTNNCSECRMLQPKVTWVN     920
AVA           NRRAVVLEMRRKEGS---------------------VYNPADTNDCSECRLLNPKVSQVN     972
PBRSV         NRQGCIIMCRQKPEYSP-------LGMELEGT--FQPFDPRNPQASVECMLQRWEKDILI     1001
TRSV          NRMNVVIQCRRKPEYSP-------MGMELEGT--FQPFDPRNPQASIECMLQHRE--THA     977
AeRSV         NRQNIVIQCRRKPEYST-------LGVELEGT--FQAFDPRNPQASIECMIQHRE--THA     991
HoNV1         NRRAIIVQCRRKQGV---------------------LYDPSDPTASTETRLLAPLYKPQD     900
HoNV2         NRRAVVVRCRRKEGA---------------------YYDPDNPLSATECQLVNPKVATGS     453
MMLRaV        NRRGAVLECRRCVGEN--------------GF--EVDFDPDNPYASTECRFVDKQSQQPT     787
RpRSV         RRRRVVIKTRGKAGV---------------------AFNPHDSTAAAEFCVVERDDRAET     957
BRSV          SRKACLVEMRRKPGV---------------------LFDPDNPLLASQARFKDPMSQMLL     934
PoLNVA        ARKACLIEMRRKPDT---------------------IFDPDNPLAASQARFKDPMSQMLL     949
TBRV          ARKACLVEMRRKPGV---------------------IYDPENPLLASQARFKDPMYQTLI     934
AILV          ARKACLIEMRRKPGV---------------------VYDPDNALAASQARFKDPMSQMLM     948
RCNVA         SRKACLIEMRKKPGV---------------------VYDPNDPLKASQARFKDPMSQLLL     924
GARSV         ARKACLIEMRRKPGA---------------------VYDEINPLAASQARFMDPMTQLLM     911
GCMV          SRKAALIEVRRKPGV---------------------IFDPDNAMAASQARFKDPMTHMLL     916
PVB           ARKCCLIEMRRKPGV---------------------QFDPDNPMTASQMRLKDPMTQMCE     944
CNSV          NRRAVVVEMRRKPGV---------------------VFDPMDCHAAMQGRFLDKRDHTPL     981
ToRSV         DRRHACILVQRKEG---------------------VAYNPSDPAAAAEAMFVDSTTQHPL     950
AnNVA         DRRHAVVEVQRVPG---------------------TTYDAKRPDLSTQARYVDSRDQTPR     730
CLRV          LRRHACVEVRRIEG---------------------VLYDPKNPYLSSEARFVNNQDQAPE     858
STeNV         RRRHAVVQVQRKPD---------------------SKYDPAQPCASSQARLVERQDQAPL     829
BaNV1         RRRHVVVECRKAVDEN--------------GN--LLPFNAEFPELSTQARLVNPIDQTPM     739
BLSV          LRRKVFLRCRRAAVWVHDDKGNRTEAIDAEGQIINRDYNPSDALDCVEVQWLHSSTGLPL     889
PRMV          LRRKVLLRCRRAATYQHDEAGNRTEVVDAEGNIVHRQYDPSDALACTEVQWLHANSCTPV     885
CawYV         LRRKVLLQCRRAAVWQHDDKGNRTELIDAEGKMVSRPYDPSDALSCTEVRWLHPASNTPI     929
SLSV          QRRKVLMRCRRAAEWVHNADGTRTEKLDAEGKIVYKPYDPSNALSCTEVQWIHPVSFTAL     924
PVU           LRRKAVLRCRRAVEWVHDDKGKRTERLDVDGKVVTKQYDPSDALACTEVQWVHPVTFNAL     675
BRV           NRRNVVVQCRAAPDV---------------------EFDPRNPSASCEARLVHRTDESPL     853
GBLV          NRRGAVVQCRRAPGV---------------------DFDARNPSGSCEARLVDPNDETPK     861
BLMoV         NRRGAVVQCRRAPGV---------------------EFSARHPSLSCEGRLVNIQDETPI     881
AYRSV         NRRNVVVWCRRKPDS---------------------EFDPTNPHASCEAVFVDRKTEQQI     421
GSPNeV        NRRNVILECRRKTDKDVAN-----------G---ASPYYEGVPLSGMMCRALEKHSPAPL     857
GNVA          NRKAIVLRMDFVDP--------------------LAIFDPERPYANSKFIFEDRLAGVPC     938
               *    :                              :
```

```
ArMV     SEG---------------------------------------------------------     938
GFLV     SAG---------------------------------------------------------     939
GDefV    SAG---------------------------------------------------------     939
MMMoV    ATD---------------------------------------------------------     950
HoNV3    DTT---------------------------------------------------------     894
PCMoV    GKE---------------------------------------------------------     923
AVA      GKD---------------------------------------------------------     975
PBRSV    ------------------------------------------------------------     1001
TRSV     ------------------------------------------------------------     977
AeRSV    ------------------------------------------------------------     991
HoNV1    GVVMLEESPVLSTNLKEDHPIYQLLARRKRISEAGKNVPHCDVAVVSKSEVRAHLQCSTA     960
HoNV2    APA---RPTMVSSNTTWEDDLNDVILANFKLEDPRPQIPHIDVHKINDTETRCHLKCNTG     510
MMLRaV   ------------------------------------------------------------     787
RpRSV    PIWV--------------------------------------------------------     961
BRSV     E-----------------------------------------------------------     935
PoLNVA   P-----------------------------------------------------------     950
TBRV     N-----------------------------------------------------------     935
AILV     P-----------------------------------------------------------     949
RCNVA    D-----------------------------------------------------------     925
GARSV    P-----------------------------------------------------------     912
GCMV     N-----------------------------------------------------------     917
PVB      ------------------------------------------------------------     944
CNSV     F-----------------------------------------------------------     982
ToRSV    ------------------------------------------------------------     950
AnNVA    ------------------------------------------------------------     730
CLRV     ------------------------------------------------------------     858
STeNV    ------------------------------------------------------------     829
BaNV1    ------------------------------------------------------------     739
BLSV     P-----------------------------------------------------------     890
PRMV     P-----------------------------------------------------------     886
CawYV    P-----------------------------------------------------------     930
SLSV     P-----------------------------------------------------------     925
PVU      E-----------------------------------------------------------     676
BRV      ------------------------------------------------------------     853
GBLV     ------------------------------------------------------------     861
BLMoV    ------------------------------------------------------------     881
AYRSV    ------------------------------------------------------------     421
GSPNeV   --HT--------------------------------------------------------     859
GNVA     ------------------------------------------------------------     938
```

```
ArMV      --------------------------IPQGPAVNTPMEEGWISPSEAVATLKNLLGEHVL   972
GFLV      --------------------------VPQGPAVNTPMDEGWITPSEAVAVLKNLLGEHIL   973
GDefV     --------------------------VPQGPAVNTPMDEGWITPSEAVAVLKNLLGVHVL   973
MMMoV     --------------------------P----ARGAPL-TDWMTPSEAMAVIKTKMGEHMG   979
HoNV3     -----------------------------ALPLHTPL-TEWIDLKECLTMIKHKLAEHMC   924
PCMoV     --------------------------E----ALHEPL-TDWISVKEATAFIETALLEHKA   952
AVA       --------------------------E----ALHQAL-TDWISVKDATARIETLLLEHKV   1004
PBRSV     ------------------------------------PVTGWISAGAAMAEAVNKFRLHRE   1025
TRSV      ------------------------------------PITGWISAGAAMAEAVNQFRLHRE   1001
AeRSV     ------------------------------------PLTGWISAGAAMAEAVNQFRLHRE   1015
HoNV1     CKGHLSIFKDSEADEGL--YELFRQRCSAGHDLHEPL-TEWQSAEISLAQIKTKMVGHMA   1017
HoNV2     CMGHMVMVKSTTEDKTLLFDKLLKQACWNVTTFHQPL-TDWESPEDILSHIKTYLAAHIS   569
MMLRaV    --------------------------------------GPWMNCLAALEVVRGMMATHTM   809
RpRSV     --------------------------QS-G-EAPDDKELYWMDFRTTVAYVIEQARIHHN   993
BRSV      -------------------------------G--QTEENSWLEMEDVVTEIINISARHRA   962
PoLNVA    -------------------------------G--QNEENSWMEMDDVLTEIINISERHRS   977
TBRV      -------------------------------G--QTEETSWMEMDDVVTEIINISARHRS   962
AILV      -------------------------------G--MDEETSWMEMSDVITEVLNISARHRA   976
RCNVA     -------------------------------G--QTEETSWMEMEDVITEIINISARHRS   952
GARSV     -------------------------------G--MTEETSWMEMPDVVTEVINIAARHRA   939
GCMV      -------------------------------G--QNDENSWMEMDDVITECINISARHRA   944
PVB       ----------------------------------GPEAESWIDIEDGITEILNRVARHRD   970
CNSV      -------------------------------GVQGQPETFWKDVPEMTTILLNICVAHRQ   1011
ToRSV     --------------------------------------SEWMSMQELSAELLLRYQQHRE   972
AnNVA     --------------------------------------SDWMMLPELTADLLTKFQAHKV   752
CLRV      --------------------------------------GEWMAMEQMQEELIARYQRHHE   880
STeNV     --------------------------------------SEWVSMEEMSAILLDKQVQHER   851
BaNV1     --------------------------------------TSFSMISELADQITLKYRDHSV   761
BLSV      -----------------------------------GPPGQWHMATSTIPLIKDAMDAHFL   915
PRMV      -----------------------------------GPAGMWHAAHSTIPLIKDAMDAHFL   911
CawYV     -----------------------------------GPSGQWHMAHSTIPMIYEAMDAHFT   955
SLSV      -----------------------------------GPCGQWHLAEFTIPMVKEAMDAHFL   950
PVU       -----------------------------------GPRGEWHLADHTIQMVKEAMDSHFQ   701
BRV       ------------------------------------GNGQWRNCSAVLEDIIHHAAIHRN   877
GBLV      --------------------------------------SEWANCSKVLEEVVHLNNAHRL   883
BLMoV     --------------------------------------TEWMNCSKILEQVSQMNAVHRD   903
AYRSV     --------------------------------------GEWRNCEALIDEIKIKAAAHDE   443
GSPNeV    --------------------------KSKD-DVDQHDFGQWTEGHKMSSFVLTEMHKHFE   892
GNVA      --------------------------------------TGWMDVEAATDYILTARLAHSE   960
                                                  :                *
```

```
ArMV            AEEEKLLDYRERIG-NDHPIYNAAQEFIGNMHY-PG----QWLTTEQKNTYGIN------    1020
GFLV            AEEAKLLEYRERIG-NDHPIYNAAKEFIGNMHY-PG----QWLTAEQKSTYGIK------    1021
GDefV           NEEKKLIAYRERIG-HDHPIYNAAKEFIGNMYY-PG----QWLTAEQKLTYEID------    1021
MMMoV           DEMIRINTFRSQRG-IRHPIFEFAKDFLKDLDL-IG----HPLSESEIEECNIRMKEGGE    1033
HoNV3           SEAKRIDAYFERNG-QGNTVNTWTKRFLKDCKFSLG----EYMSDSTKASLGIT------    973
PCMoV           KEERRMAAVNEAQR-GATGIMCFSRNYILEQRL-TG----VFIDEPDKERFKIK------    1000
AVA             REEIRINAVNEAQR-GQTGIMCFSKQYIMEQRL-QG----VFLDEPTKSRYELT------    1052
PBRSV           KEMILQNNHLSSFR-PAHPIYTECATFLSMYSR----DASFVPPVDLGCKWN-V------    1073
TRSV            KEMILQSNHLSSFR-PAHPIYTECATFLSMYAR----DASFVPPVDLGCKWE-I------    1049
AeRSV           KEMILQSNHLSSFR-PAHPIYTECATFLSMYAR----DASFVPPVDLGCKWQ-V------    1063
HoNV1           AEIKRVENFKLRTS-GEGPVTKGMRDFLRAQKV-EG----KYLPPDEKSLKGL-------    1064
HoNV2           KEQRRMEAVKRNTG-NEGPITGGARSFLKDQTV-IG----YPASDDEKARHHIT------    617
MMLRaV          KEHTLQVNYLSRRA-GVHPTFDAAQSFLSCLSG----EVSMYYPHDLLQACG-I------    857
RpRSV           AEDIEQAQYSMKHS-RTRQLYQVCENYIGEVKM----SVANFVPGDMLGAWNLE------    1042
BRSV            AQEKLQARYMREKS-LLDPLALAAENFLKGEVQNAY----LDFSGLELEKAGIPKP----    1013
PoLNVA          AQAKLQARFLREKA-RMDPLALAAENFLKKDVQKAY----LDFSGLELEKAGIARP----    1028
TBRV            AQEKLQARYMREKA-LLDPLALASESFLVKEAQKVF----LDFDGVELEKAGVPRP----    1013
AILV            AQDKLQARFLREKA-MSDPLALASEKFLQGDVKNCY----LDFSGIELEKAGIPRP----    1027
RCNVA           AQEILQARFMREKA-MMDPLALAAENFLKGEVQKAY----LNFDGLELEKAGIPLT----    1003
GARSV           AQDKLQANFFAAYVHAMIQVALALQEFLRKGCPLLL----SRLPDLEVEKGWYP-S----    990
GCMV            AQEKLQNASLRAKA-SLDPVTLASQEFLRKEVNSVY----LEIPTLEIEKAGISGV----    995
PVB             EQERLQRAHLRKTA-AVDPMILAAENFLQRKVEEVY----LHFPKIELEKAGLTC-----    1020
CNSV            EQDILQEQHIRKHA-VNDPLILASERFLKQESRKAL----CYMPRVEMEICGVQSQ----    1062
ToRSV           AQHAEYSYWKSTSR-TSHDVFDILQKCVNGDTQWLSLPVDVIPPSIR-QK----------    1020
AnNVA           QQADEQAYLKSTMR-STHDVFDILKEHIEKDIFWLSQPDTYLPPTEK-CK----------    800
CLRV            AQVDEYNFWKKNAR-NTHDVFDILEEKIKEEGFWLSSAEFCMPDRAS-QG----------    928
STeNV           DQQAEYLYWKRSAG-NTHDVFDAIKMNLDRDAFVFSYDSSLLPP--S-LG----------    897
BaNV1           KEDKVFANWKATSG-VSHSYFNIVERALEKEAYKGSFSFCMFDP--T-LK----------    807
BLSV            QEDIKRDAWVQQTD-MKSKTGREVSSYLSGLMGALG----SYKAIQRSSETS-E------    963
PRMV            IEDAKREAWVQQTN-MHSRTGAEVSSYLQSLVCALG----SYKAIQRSSEVS-D------    959
CawYV           QEDFKREAWVQKTN-MHSRTGREVSTYLQSLVTALG----SYKAIQKASETS-S------    1003
SLSV            QEETKMASWKSSLG-MKSQTARKMGSYLQDMIKGIG----NFVTLK---GAK-D------    995
PVU             TEDAKMSAWKDSLG-MHSHTARLMTSYLQDMIKAIG----CWQNTS---GQC-G------    746
BRV             KENLLMENYRERSD-TQHPVFVGAKSFIHRLAK---------------------------    909
GBLV            KELMLMANYRERND-TLHPVHSGARNFLRSCTK---------------------------    915
BLMoV           KELTLMCNYRERNE-LLHPIHDGAKNFLKNCTK---------------------------    935
AYRSV           KEEKLQAHYLGSHR-RVHPIHNDIQDFLRAGIV----MTKHLADTNYDTY---R------    489
GSPNeV          KERGYQAEHMHRVR-GRHPIFIDSDAVLRLQNRL-T----MMEEFSR-Q----G------    935
GNVA            SQEKQMACYLRQNA-QDHPVQHRAKEIIQECIRDMPRMLRLDIPLPE----E-T------    1008
                 :                         :
```

```
ArMV        -EEGFSFLAVDGKMYKYNVLG-KLNPCETVPPH--------------PNVI-PWLEEKT-    1062
GFLV        -DDGFSFLAVDGKIYKYNVLG-KLNPCESEPPH--------------PNVI-PWLEKKT-    1063
GDefV       -DSEFSFLAVDGKMYKYNALG-KLHPCTSKPSH--------------PNVI-PWLEKKT-    1063
MMMoV       RPRGFSFLALDRQLFVYNALH-RLVECTF-DKM------------------PLHLEGIT-    1072
HoNV3       -TKSNHCFLVDDRVFFTNAIG-ECEEVTYKKK---------------PES---IYENVA-    1012
PCMoV       -SSCFMFVVVDHKMYQQDASG-MIFNLENQPDM--------------PEKEWERMESRC-    1043
AVA         -SKCFMFLVVDGVVYQQDAAG-HVFPLDHQPKI--------------PEAELKKMEDRC-    1095
PBRSV       PTGYLSIAAVDGRTFGFTQF-GVCEEISHILKY------------------TEEMEAYT-    1113
TRSV        PSGYMTIAAVDGRVFGFSQL-GVCTEISKQMKF------------------TEEMEQYT-    1089
AeRSV       PAGFSTIAAVDGRVFGFSQL-GVCSEIKTGLEF------------------SEEMEQYT-    1103
HoNV1       PNHRYAFVSVDERMFAYNEMG-YLEEVEHKCT---------------PEY-MQSIQDYC-    1106
HoNV2       DKDKFVYMAVDGHLYGYDVLR-RMQPVTNTAT---------------RNW-LDLVEART-    659
MMLRaV      ESRYFFYAQVDGKLYGYDVNK-SAHLVPGD--L------------------GEEFEKAC-    895
RpRSV       PKGRFFYSCVDGRVYSYDPEQKAHDEGPVDK--------------------ALDFEQIC-    1081
BRSV        -Q-GGRGLYVDGSLYLLDPTF-QFEEIPITDD----------------GY-KRLWDERM-    1052
PoLNVA      -Q-GGRGLYVDGHLYLLNMSF-ELEEIRVHDE----------------NY-QRLWESRM-    1067
TBRV        -E-GGHGLYVDGVLYLVNASF-EFDEIPIKDG----------------GY-QRLWDSRM-    1052
AILV        -Q-GGRGLYVDGKVYLLDDLC-QLEELEIAEE----------------GY-KRLWEERM-    1066
RCNVA       -Q-GGRGLYVDGNLYLLNQNF-TFDEFVVKDE----------------GY-QRLWERRM-    1042
GARSV       -Q-RRKGFFVDGVLYLQNAQF-SFDSFPVKDC----------------MY-GKLWEKRM-    1029
GCMV        -R-GGRCLYADGILYTLGTEF-QLIPHPVENE----------------GY-QKLWAQRM-    1034
PVB         -A-EGRGLCVDGSLLMLNSRF-EMDVHTVHDS----------------NY-ARLWEKRT-    1059
CNSV        -AAGCYYLCVDQKLYTCEDDG-NLVETPCLNP----------------SY-A-QWERNS-    1101
ToRSV       -HKGNRVFAIDGRIFMFDYMTLEYDEIKEKEN-----------------LDARHLEAR--    1060
AnNVA       -YKGNRLLAVDGKIYYFDYLNFDYQMVDLVPG-----------------IDAAHIEQC--    840
CLRV        VSAADRFLGVDGKIIKYDALSFEGAYFDGKHLA----------------KSAQDLEHL--    970
STeNV       SDPRQRYIVVDENIIEFSPATMAGEIKKIPPPK-----------EGEAKVDWGSLEKH--    944
BaNV1       VDTTYRYICVDNKVIYFKPMNLDGECGYLAPSR-----------PL-RENEIKELSDW--    853
BLSV        AGERKFLVAVDGQIYSLDSQGVATLEANDAYDN------------------VEALEAVS-    1004
PRMV        AGERKFLVAVDGTIYSINSLGIATKEAADAYDN------------------VEALESTT-    1000
CawYV       AGERKFLVAVDSKIYSIDSLGMATAESDDAYDN------------------VEALESTT-    1044
SLSV        SDPRYLLVAVDGKCYALDQQGKATP--YEDCDV------------------VEEIERLT-    1034
PVU         TNPRKILVAVDGKAYELDAAGNALE--LKQCDN------------------VEDIERQT-    785
BRV         -EKNFAHIVCDDVLYYHDSYIDSTRVSEGTI--------------------NIGMEDA--    946
GBLV        -SLALGNLVCDGVIYVIDRETATCEATKEKP--------------------TSGYEQT--    952
BLMoV       -SLIFDSIACDGKLYIVDKVTRTVEEAKEAP--------------------NPGLENS--    972
AYRSV       ENWLDANLAVDGKLYKVS-LKNVCDELLELP--------------------PDGFEEA--    526
GSPNeV      QTMENMAMAVDGKIVHFPVWQKDCPECIKSGFHSKVNEVPVYYGHFLKEKDHPEIDPICL    995
GNVA        RFGEYAFVTCDGESYAFHLD-GTCRKLNEYVAG------------------IEELEINRV    1049
                      *
```

```
ArMV            LSIVHWDAHKH---------IATGPRNALVSCFLQGLVQDQSRVQSVDL-MGKDSSPEQQ     1112
GFLV            LEIVHWDVHKH---------IATGPRNALVACFLQGLVQGQSKVESVER-MGKDSSPEQQ     1113
GDefV           LEIVHWDVHKH---------IATGPRNALVACFLQGFVQGQSKVESVER-MGKDSSPEQQ     1113
MMMoV           LDNVYNDIHVR---------LARDPKNGFVGSFLHEMVNLQHNIISVDK-MTTKCSAEQQ     1122
HoNV3           VGELANDVHKY---------MMTVPNNGFVSCMLHDMVDGTNKVISIEE-LVPDASSEVV     1062
PCMoV           IQNVVYNIQAY---------LADSPPNGLVGTFLAHIIDETCNVKSVDA-LSSAATAGEK     1093
AVA             VANVVYNIQAY---------LADGPPNGLVGTFLAHIIDETCNVKSVAK-LSSSATAGEL     1145
PBRSV           LEKFAPTITRT---------LASQSRYKLVGAFLKGMVRDEDNVMSLKS-LGPKSTSTQR     1163
TRSV            LDKFAPDITKT---------MASQSRFKLVGAFLKGMIREEDNVVSLTS-LGPKSTATQR     1139
AeRSV           LEKFAPNITRT---------LASQSRFKLVGAFLKGMVRDEDNVASLKS-LGPKSTATQR     1153
HoNV1           IQYLPQNMQLY---------FIEKPKSRMVADFLLAMVREGTCIQSVEE-LGGNPSPMQK     1156
HoNV2           LECLPHNIQEY---------LANGPKNVMVGSFLEDMIHDGMRVDSVX------------     698
MMLRaV          LQKLLPTMQQT---------IAQRCNNGLVAIFLKSLVSGSCSVVSVDK-LSDSASAVQR     945
RpRSV           LEKLSYTLQAD---------IQGGPKSATAGIFLRSMVSGECAVESVDK-LNRSASREHL     1131
BRSV            RKSFLSKIQTG---------EYLNSKSMVVTGFLRSLVNGDCAVLSKDT-LSSSASVAQQ     1102
PoLNVA          RQKFLPSIQAG---------GYLNTSSMVVTGFLRSLVNGDCAVLSKDD-LSSSATVAQL     1117
TBRV            RKKFLPAIQRD---------EHLNTKSMVVTGFLRSLVNGECAVLSKDT-LTASATTAQL     1102
AILV            RLKFLPRVQAK---------QILNTSSMVVTGFLRSLVNGDCAVLSVDS-LSTGATVGQK     1116
RCNVA           REHFISTVQTG---------NYLNTKSMVVTGFLRSLVNGDCAILSKDS-LSSTATCAQQ     1092
GARSV           QEKFLPAIKAG---------GVLDTRSMVVTGFLRSLVNGDCAILSVDS-LSSSATACQQ     1079
GCMV            KNMYLPAVTTG---------RYLNASSMIVTGFLRSLVNGDCAVLSVDA-LSTTATFTQK     1084
PVB             MEQFFPMVHGG---------TYLNGKSLIVTGFLRSLVNSECSVLGLGQ-LSSTATSCQQ     1109
CNSV            SENFVGGVQAL---------DALECRSILVSGILRNLVQGQCCVLSIDE-MSRL-PLCTQ     1150
ToRSV           ILEKYGDTRLL---------LEKWGANGVVAQFIEQLLEGPSNVASLEV-LSKDSLESHK     1110
AnNVA           SLAKYGETRLL---------LEQWGTNGVVTQFIEQLLEGPSYVASAET-LTADSLESHK     890
CLRV            AMVKYHEFSDL---------LRAWSVNGVVRQFMEQLLTGPTHVDSANA-LNLDALPSHR     1020
STeNV           GDTWYPDLAAM---------LQGWQCNGIARQFVAQLLQGPTHVDSIKS-LNVEASASHK     994
BaNV1           SLTHMGDLSSE---------FSLELVNGIVAQFVEQLLEGPSRVISVDV-LSQDALPSHK     903
BLSV            LNQYRLDFSKQVREHCL-LTCNSSFHSSLVRDILEDMLVNDACIISVNK-ISRDTKQIHR     1062
PRMV            LLQYRLDFAKQVREHSL-LTNDGSFHSSMVRDLLEDMLVNDACVVSVDK-ISRDSKQLHR     1058
CawYV           LLQYRLDFAKQVREHAL-LTNDGSFHSSMVRDLLEDMLVNDACVVSVDK-LSRDTKQIHR     1102
SLSV            LLQYRSYFADLVMEHADNTYYQDSFHSSTVRDFLLALMKDGSTVLSVDS-LGSSTQDIHK     1093
PVU             KLNYRIEFSRAVYSHTT-LTYEDSFHASMVRDFLDDMLENGASVSSVKE-ISSSSKPMHK     843
BRV             CIQSVVQWSEL---------VGGVKDLGLLYAFVHAFTEGPCHVDSVEA-LNSEATSCQR     996
GBLV            AQHFVASLRQE---------ISNRACDGMLNTFLNGLIEGPCEVKSVDA-LNEAATSSQK     1002
BLMoV           CLFIMDSMKTE---------IDRNARSGMLNTFLYSLLEGPCEVESVDK-LSAGATDGQK     1022
AYRSV           CLARIPTLSTA---------MADEGVSEFVQTVIEGMIEGPSFVESVDK-MSAETPADHR     576
GSPNeV          EDRIMATIRRQ----GSVERTMFGMANPIVDAFLKQLVQKDVRVEGVDKKLSGLVTPAQR     1051
GNVA            FNHIDSEWRKL---------GVYESGDDLVVDFTQTLLSGPCRVSSVGS-LNEDASVSQR     1099
                                                .   :      : .
```

```
ArMV      AFFKRLT--LSERIYLRLCQIRIDAVKKEQL-S--SVSRGALDVLRDCMYKSKAKLVENY   1167
GFLV      NFFKRLS--LSERIYLRLCQIRIDNIQKEEL-A--GSGRGPMAILRECLMKSKQVVVENY   1168
GDefV     SFFRKLS--LSERIYLRLCQIRIDNIKQEEL-V--GSSRGPMAVLRECLLRSKQVVVENY   1168
MMMoV     TFFHSLP--LTERVYLRLSQLKIDTIHREAG-A--SPGEFAYQKLMRAMKKTYAAVADNG   1177
HoNV3     AFYKSLG--LKERIYLRLMQKRIEIERSNTF-D--SPLKGVHAKILMAIAKSYAFAKEHG   1117
PCMoV     EFWDSMSEDLRGRVYLRLCQKRKDSIAGETR-D--SVIDKTMERLSRFTMSSYQYVKDNG   1150
AVA       EFWESLECDLRGRVYLRLCQKRKDSIAGETR-D--SALDKSVEKLCSLVGDSYQYVRAHG   1202
PBRSV     EFFETLG--IGERVYLRAVQKRINKINNEPAF--NVD--NLHAKLLNSVVSSYEYVKEKG   1217
TRSV      EFFETLG--LAERVYLRAVQKKVNKIRTDPAF--DVE--ALHARLLSTIATSYEYVRTYG   1193
AeRSV     EFYETLG--LAERVYLRAIQKRINKIRDDPGF--EVD--KLHAKLLSIVAQSYEFVKEKG   1207
HoNV1     EAFATLA--LTERVYLRLAQKRIETITGGPSLL--ENAFAAQGKLLGVMLKGREKIVEHG   1212
HoNV2     ------D--LNERVFLRLAQKRNDAICENKGLA--DHLDGFQMRMLSALLKGYEQVEKHG   748
MMLRaV    EFFARLS--MGERVYLRLVQKRMNQFLLDEVTHVPFL-TNALKTCVDTMRAGARVVWENS   1002
RpRSV     IFFKNLS--LADRVYLRLVQKRILQLAMVGDP----LGLRSYTVMMEGFQNSYNYVKENG   1185
BRSV      SIFKALG--IDERIYLRTLQHQLDLYSADIPEN--PYSNSAWIKILKAIGMGRTYLAENG   1158
PoLNVA    SIFKALG--VDERVYLRTLQHQLDAYSQEIPEN--PYSNLAWIRVLRAIGSGRAYLEENG   1173
TBRV      SIFKALR--LEERVYLRTLQHQLDLYSQDIPEN--PYCNSAWVKVLGAIGAGRDYLVQNG   1158
AILV      SIFHGLR--ADERIYLRVLQHQLDLYSMEIPEN--PYSNSAWLKILRAIGAGRDFLVNHG   1172
RCNVA     AIFKALG--EDERIYLRTLQHQLDLYTQDCAEN--PYSNTAWCKVLEALGAARNFLEKHG   1148
GARSV     RIFQNLQ--LEERVYLRTLQVQMDKFMQDLPEN--PYSNSAWVKVLTAIASGREYLANNG   1135
GCMV      RIFESLN--LAERVYLRALQCQIDAYTLDIPEN--PYSNVCWVKMLKALGQGREFIVSNG   1140
PVB       RIFQHLG--LCEQAYLRCLQKRIDMYNAEVECN--PYCNTAWAKVLKAMMATRDLIIDNG   1165
CNSV      RLFKALQ--LQERVYLRLIQKKISHILSVDESN--VYSKNAWMRCLEFAAASRDYLKEHG   1206
ToRSV     EFFSTLG--LIERATLRAVQKKIDAAREDLMHL---SGLKPGRSLTELFVEAYDWVYANG   1165
AnNVA     EFFSTLP--LVHRATLRLVQKKIDLAKEDLKQF---GSLKGKFDLAPYFLESYEWVYNNG   945
CLRV      EFFDSMT--LPKRAVLRLIQKKVDMIKAGPAFEF--T-PTKGFTLAKVLKDGYDYVYNNG   1075
STeNV     EFFGTMG--LVERAILRLMQKRIDALRKDPVHVQ--CNDFQMRGIASYFKEGYDYVAENS   1050
BaNV1     EFFNTLS--LPKRALLRLAQLRLDECRKQNIFVTMSEGPTKPSVMAGWFIESYDWVVKHG   961
BLSV      DLWKELK--LSERVFLRISQKALNTLREQPHFK-----VDLKSQILDSFAVFRDSIVDNR   1115
PRMV      DLWSELK--LAERIFLRVSQKALNQLRDQPHFK-----VDVQSVFLQHMADFRDAIVDNK   1111
CawYV     DLWGELK--LAERIFLRVSQKALNQFRDQPHMK-----APIDGLFLQHMKSFLDSVVNNR   1155
SLSV      ELWAELG--LSEKIFLRVSQIARNQLRDAPHFK-----EDVAHNFLETMRHLRDAIVDRK   1146
PVU       DLWKELT--LSNRIFLRVSQVALNDVRDAPHFK-----EHITNTYLESMRLVRDAIVDNK   896
BRV       DFFQSLS--LLERIYMRLVQKQLDRIRANPDF--LFS-VDIKTRILQSFRHGYDEMITHG   1051
GBLV      EFFLELP--LLERVYLRILQRQLSAIREMPDF--AFN-MNVRERILASLRVGYNTVCEHG   1057
BLMoV     TFFKELP--LFERVYLRLVQKRIEYVKGIPEL--AFK-VDIKAHILQSLATGYKEVCAHG   1077
AYRSV     EFFSRLP--LGERVYFRLLQKRFEQLKADKDF--NFQ-IDMKVRVLKSLKSSYDKVVENG   631
GSPNeV    DFWEQLP--LAEKFYLRLMQKRIDEIRKLPSAAE----HVYIPRCIAALRQTGDWLWSNG   1105
GNVA      RFFDGLS--LGERIYLRLLQKRLDNTLEHFKM---SDGTSYYDAIKNSLIEGYQFLKTHK   1154
                      :  :*  *
```

```
                                                        NTB-VPg cleavage site

ArMV      SL----LLTLVAILVLIATAYSLISTLIGLAGCS-SFAGGMVALNHVSN--------ASI    1214
GFLV      SL----LLTLVAILLLISAAYTLLSTVVALAGCS-SFAGGMVAVTAVNN--------ASI    1215
GDefV     SL----LLTLVAIILLISAAYTLLSTIVALAGCS-SFAGGMVAVTAVNS--------ASI    1215
MMMoV     RSI---FLLLAAVVCIVISFYGLFSILASCFGGA-SVASAAIAMNKVDA--------MNG    1225
HoNV3     GTL---LLVIAALISMSTVMAGFFALFRSFFSAK-SFVGGAVAMSQLSG--------RSV    1165
PCMoV     GAL---CLLLAGFVTVSVSCYGLFAFMSSFFNAP-SLTGGIAALEAIDA--------KAV    1198
AVA       GTL---CLLLAGFVTMTISFYGLFSFMTHFFSAP-SITTGIVALEAIEA--------KAV    1250
PBRSV     PKI---FPLLMGFIVVVFACYGFVMPLLSFASGGS-AVGGMVAMEQMTA--------ASV    1265
TRSV      PKI---FPILMGFVCVVFACYGFIMPLLSFASGGS-AVGGMVAMEQIAA--------ASV    1241
AeRSV     PKI---FPLLMGFIVVVFACYGFVMPLLSFASGGS-AVGGMVAMEQMTA--------ASV    1255
HoNV1     FTI---LLVLSAVVCLSITLCGFYSLIAGLSTAT-GLTAQAALISKLSV--------RQS    1260
HoNV2     MSI---LLFVAGLVCVSIALYGFYHAASALCGAA-SFAGGLAAVTVMDT--------KAV    796
MMLRaV    GKI---LMVCSAILALLVLAQGFVGALSLFAGSASLA-TGVGVLHSMDIQGASNASSSSY    1058
RpRSV     GRL---LLILCSCMLLGIACYTFFNALAILIGGTSVA-AGAAAMVDIGACGS-----TST    1236
BRSV      CGI---LMIAAALILILVSAWGFWKLFIGLFSGSMSLGAAIVGMSAVDI--------KAQ    1207
PoLNVA    GAV---LMIAAALVLVLIGAWGFWKLFVGLFSGSMSLGAAIVGMSAVDI--------KAQ    1222
TBRV      CGI---LMIAAALILILVSGWGFWKLFVGLFSGTMSLGAAITGMSAVDI--------KAQ    1207
AILV      CGI---LMVAAALCLILVAGFGFWKLFVGLFSGSMSLGSAIVGFSAVDI--------KAQ    1221
RCNVA     GTL---LLLAGALVVVLISIWGFWKLFIGLFTSSVTLGGVMTGLTGVDL--------KAQ    1197
GARSV     CRI---LLVAAALLIVLVSAWGFWKLFIGLFTGSMSLGAAIGGLGAIDI--------KAQ    1184
GCMV      GGI---LMIAAAIILVLVCGWGFWKAFVGLFTGSMSLGAALAGCQEAEV--------KAH    1189
PVB       GGM---LMIMAAILVIIVSAWGFWKALGLLFTGTISIGTFFSSAAEADL--------KSQ    1214
CNSV      LEV---LLLLAAMMILCVALYYFVGAFIGVMGGALSMGAAMAGLKEVDM--------KAQ    1255
ToRSV     GKL---LLVLAAVILILFFGSACIKLMQAIFCGAAGGTVSMAAVGKMTV--------QST    1214
AnNVA     GKL---LLVLAAVILILFFGSACIGLLKTIFVGGASASATVGAMTRMSL--------QST    994
CLRV      GKI---FLIFAAVVILWFLCGTAMHLLRQIFCGGVGAG-SAGAMMKMSV--------QST    1123
STeNV     GKI---FLVFAALILVFFFASTCINLVKAIFIGGGAIS-AGAAVERLHT--------HST    1098
BaNV1     GRI---FLIFAAIIIIYFFASTFMHLLLNIFCGATSAK-MAYHMSVMDV--------QSV    1009
BLSV      QKI---LLFLSAILLVGTLSWSFLSLMKAFLSGSVGFGGALALKNQLDV--------HSC    1164
PRMV      QKI---LLFLGAILLVGVASWSFLSLMKTFLSGSVGFGSALALKNQLDI--------HSS    1160
CawYV     QKI---LLFLAGILLVGVSAYSFFALLRTFLSGSIGFGSALALKNQLDT--------HSS    1204
SLSV      EQI---LLFFAAVALVGLFSWGFLSLIKQFACGSLGFGAGIALREQI-A--------HSS    1194
PVU       EKI---LLFLCAVTLVGLTAWGFFSAFKAFTSGSMGFGAGLALKNQLAA--------HSS    945
BRV       GKV---LAIFAALLLVLLLYSSFFALYQTFVAGTSSALVSAGMITQLSANAG---SVCTS    1105
GBLV      GKL---LTIVAALVLILVIYSSFFCIYRTSIAGPTDGVAALVAVGALTANAG---SVSSV    1111
BLMoV     GKI---LTVVAALVLILILYSTFFSIFSVFIRGDQSGMATLATMGALSANAG---SVSTV    1131
AYRSV     GRI---FLVCCAFIMIYFAYSTFFSIFNAFVGGSSAGMAGALI-TQLDA--------HSV    679
GSPNeV    AKI---LVLLLALIIIYTAAKAFLALFACFISGRG----AVEVLTKLDT--------FSV    1150
GNVA      STIARYMLYVVALVLLIGSCYTFFQLLAGLFGGGGTCLATTL--VNLDT-----------    1201
                           .   :
```

```
                 NTB-VPg cleavage site          VPg-Pro cleavage site

ArMV        P--CSE----PR-LEEGYIPRN-KFVSRI-SRTR-------GD-GPAQG-----------    1246
GFLV        P--CSE----PR-LEERYSPRN-RFVSRI-SKIR-------GE-GPSKG-----------    1247
GDefV       P--CSE----PR-MEERYPPRN-RFVSRI-SKIR-------GE-GPSQG-----------    1247
MMMoV       L--NSS----SA--SGPSVRRPNRYMPVQ-QRTV-------LA-RSMEG-----------    1257
HoNV3       A--ASS----DIGSVDAYSARNVPNVYRQSTQYRSEASSSSIHSGGRGGKLFARGSRTTY    1219
PCMoV       M--SSS----SY--GDAYAKRNM---RPL-HHYI-------AR-GPCET-----------    1227
AVA         M--SSS----SY--GDAYGKRNV---RPL-HEYT-------AR-SAFEG-----------    1279
PBRSV       I--S-S----GSSP-VHHRSRAPPIQPR----YARHRL--------AGS-----------    1294
TRSV        V--S-S----GSSP-VAHRNRAPPVQPR----YARHRL--------AGA-----------    1270
AeRSV       I--S-S----GSSP-VHHRSRAPPIQPR----YARHRI--------AGS-----------    1284
HoNV1       L--DSA----GQ--SESYRSRHP---PTR-HKYS-------THMRARGG-----------    1290
HoNV2       F--PSG----SD--AEPYRGKHI---PSR-HRYI-------TTVTARGG-----------    826
MMLRaV      Y--DST------------RGHNNRVNHK----HMHLQ-----------------------    1077
RpRSV       Y--ASEYGAKMGRRNMPHRSREIPAVWS------------------EET-----------    1265
BRSV        Q--KSS----SQ--EGGYRARNI---PIH-HRYA-------YA-KSQAG-----------    1236
PoLNVA      Q--SSS----SQ--EKGYRARNI---PVN-HRYA-------YA-RSQAG-----------    1251
TBRV        Q--SSA----SQ--EKGYRARNI---PIH-HRYA-------YA-RSQAG-----------    1236
AILV        H--KSS----SQ--EGGYRARNI---PIH-HRYA-------YA-KSQAG-----------    1250
RCNVA       Q--SSS----SQ--EKGYRARNI---PVH-HRYA-------YT-RSQDE-----------    1226
GARSV       H--SSQ----GQ--DKGYRSRNI---PVH-HRYA-------YA-RSQAG-----------    1213
GCMV        SVYSAD----GG--DRGYRSRNI---PIN-HRYS-------YA-RSQAG-----------    1220
PVB         S--NSS----GI--DRGYRARNI---PVN-HRYA-------YT-RSTES-----------    1243
CNSV        Y--SSG----AQ--EGRYRSRNI---PIR-QRYR-------YA-RGELD-----------    1284
ToRSV       I--P-S----GSYA-DVYNARNMTRVFR----PQSVQGSS--------L-----------    1243
AnNVA       I--P-S----SSDM-NVHMVRSMKTVFR----PSSLQSSS--------A-----------    1023
CLRV        I--P-S----GSDV-QSYASRNLRRVYR----PTRLGLQSAM------N-----------    1154
STeNV       I--P-S----SSMA-DSYSSRNMRRVYR----PSSLTLHSSG------G-----------    1129
BaNV1       S--S-S----SSMD-RVHAVRNMPRVYR----PMQMQSDR--------K-----------    1038
BLSV        A--AST----ASVATSVYSSNSIPIVWAQAARYANVHSCLEE----N-------------    1201
PRMV        V--ASS----GSIA-AQYSARSIPIVWAKAARYANVHSQIEE----S-------------    1196
CawYV       V--AAS----GSIV-QSFSSRNIPIVWAKSARYANVHSSLEE----Q-------------    1240
SLSV        V--MSS----GSVT-SAFVARNMPVVWGKAARYATAHSSKEE----V-G-----------    1231
PVU         V--MSS----GSVT-SAFAQRNVPIVWGKAARYAQAHSQLED----A-------------    981
BRV         A--SNP----SGA--ASYVSSNIPIHHR----WRSNYSERSY--A-LNS-----------    1139
GBLV        Y--SSS----DGGL-SKFS-RNTPINYR----SA---ASSDF--R-ANS-----------    1142
BLMoV       Y--SSS----EGSR-ASYSTRNPPIQYR----SV---GGSTY--S-ANS-----------    1163
AYRSV       Y--S-S----GASV-QSYRSRNLPTTYR----QRMMAHSQDDS-K-KLA-----------    714
GSPNeV      F--PSS----SVDA-QSYRSRNTPLCYRTHTYS-----------T-NNG-----------    1180
GNVA        ----HS----RGEV-RTQGRSNRPVVER----YMNTHSGGT-------------------    1229
```

```
                                                Catalytic triad
ArMV        -QG--DH-----EELVTELYYY-FDGVKRLISCCWFKGRSLLLTRHQAMAIPIGNEIQVI    1297
GFLV        -QG--EH-----EELVTELYYY-CDGVKKLISTCWFKGRSLLMTRHQALAVPIGNEIEVI    1298
GDefV       -QG--EH-----EELVTEFYYY-SDGVKKLISTCWFKGRSLLMTRHQALAVPVGTEVEII    1298
MMMoV       -EEVPEW-----HSLIVEIHSG---HASQFISACQYFGRSLLMTRHQALDLKRGQEVVIA    1308
HoNV3       FSDCEPW-----QRALVEIHTG---KGTTFTSACRGKGRDIILTKHQAEAIPQGCEVTFI    1271
PCMoV       -NYKELW-----QRLVVKIFPEAGVARGFLIQAAQFEGRSLLLTRHQAQSIPHGSQVFIE    1281
AVA         -ESKELW-----QRLCVKIFPEAGPSKGKLVLSCQFEGRSLLLTRHQAQSIPHGSQVFVE    1333
PBRSV       -APDESYA-YEELMVVLYVDST----IAPVVNAVRGPGRSIFITEHQAMAIPNNSTVVAH    1348
TRSV        -SAEDAYA-YEEMMVVLYVDST----VAPVVNAVRGPGRQFF-NSHQALMIPNNSTVVAH    1323
AeRSV       -TPDEAFQ-YEELMVVLYVDST----TAPVVNAIRGPGRSLFMTEHQAMAIPNNSTVVAH    1338
HoNV1       -GNIPAY-----ENLMVDMHVK--SAKPFMISALHYYGNSLCLTRHQALLIPEKAEVECA    1342
HoNV2       -NTEHPW-----DDMMVTLSVG--CT-NIYISAIRYRGRSLCLTRHQALAIPEGAEVRCQ    877
MMLRaV      -GGNPTLP-CH--GASLSLYGP----NGFFCPATWARGRSFWITRHQAFAIPDRASMALI    1129
RpRSV       -GHDEKWQLCGLL----ETCRS----DMPAVHVNLVPGNKFAITKHQAQAIPDGSSVGLS    1316
BRSV        -DGLLPA-----ARLCVAIYQP----GGGFVSAMQYKNKSVRMTRHQALRFQEGEQLTVI    1286
PoLNVA      -DGLLPA-----ARLCVAIYQP----GGGFVSAMQYKNKSVRMTRHQALRFKEGEQLTVI    1301
TBRV        -DGLLPA-----ARLCVAIYQP----GGGFVSAMQYKNKSVRMTRHQALRFKEGEQLTVI    1286
AILV        -DGLLPA-----ARLCVAIYQP----GGGFVSAMQYKNKSVRMTRHQALRFKEGEQLSVI    1300
RCNVA       -NGLLPA-----ARLCVAIYQP----GGGFVSAMQYKNKSVRMTRHQALRFEEGEQLTVI    1276
GARSV       -DGLLPA-----ARLCVAIYQP----GGGFVSAMQYKNKSIRMTRHQALRFQEGEQLTLV    1263
GCMV        -NGLLPA-----SRLCVAIYGP----RGVFISGMQYKNKCVMMTRHQAQSLNEGDELSVV    1270
PVB         -DGMLPA-----ARLCVAIYTS----AGDFVSAMQYKNKSIMLTRHQALRFREGERLTLI    1293
CNSV        -EEVPLG-----GQLAVALYGS----QGRFISALQYKGKSVMLTRHQMLMFAEKERVTCI    1334
ToRSV       -AEAQFNE-SHAVNMLVRIDLP----DGNIISACRFRGKSLALTKHQALTIPPGAKIHIV    1297
AnNVA       -HRDVFNA-SHAVHLLVRIDLP----NGNIISACRFKARSLALTYHQAKMILPGTRVGIN    1077
CLRV        -PVETV---SQAEQLMAWIDTP----QGNLISCCRFKARSLAMTYHQARAIAPGAKIFIT    1206
STeNV       -VQDVKDK-EYALHALVRLTLS----DGRIISAMRFKSRSIALTYHQALTIAPGSLVSIA    1183
BaNV1       -VEEEFNE-SHLTRLIVRLDLA----DGGLISGMRFQNRSICLTYHQARTIPEGSKVRVV    1092
BLSV        -HNFKYFE-DGLAHLLVRLVGS----SGISENAILYGPRSIALCAHQIRLFPDHDRVQIH    1255
PRMV        -SHFNFFE-DGLAHLLVRLVGT----SGQSETAILFGSRAIALCAHQVRMFPDHDRVTVH    1250
CawYV       -TTFNLFD-DGLAHLLVRLQGT----SGQSETAILFGPRAIALCCHQIRCFPDGDRVLVN    1294
SLSV        -ANFDYFE-DASAHLLARVVGS----SGQSETCLLFGHQAIALCAHQLRLFPDNDRVTIH    1285
PVU         -GSFDYIK-DGMAHMLCRVVGT----SGQSETGLLFGPRSVAICAHQLRMFPDGDRVTIH    1035
BRV         -NLEDKYL----LDLLVWLQIP----GDSIISCIRFKGRSLLLTKHQALAIPEGARVYCN    1190
GBLV        -GGDDEFL----MSLLLWLETP----GGGLISCIRGKGRYIYLTAHQAEQIPDGARVFAR    1193
BLMoV       -AGKDEFL----MNLLVWLETP----GGGLTSAIRGKGRFLYLTKHQAEAIPNGARVECV    1214
AYRSV       -DEDAKFK----TDLLVRLTIP----GGRVICAVRFYGRSLLMTKHQAMAMRKGDRIMCN    765
GSPNeV      -NPGKEYP----IGMLVGLHTP----QGQFVSCIRGKDRSILLTQHQARRIPDGAKVLLS    1231
GNVA        -PPSREDQLRKLSVIISFIAGG----QEYHFMGARFFGGSIVLTGHQAYYIPDGTRVSIN    1284
                                                    .    **   .    :
```

```
                                       Catalytic triad
                                                 ⬇
ArMV      YADG-TE----RKLVWPGR--QEDRSCKGYIEFPDNELVVFEHARLLTMPIKYEK--F-F   1347
GFLV      YADG-TT----KKLVWPGR--QEDGNCKGFVEFPENELVVFEHPHLLTLPIKYEK--Y-F   1348
GDefV     YADG-TV----KKLVWPGR--QEDGNCKGFVEFPENELVVFEHPHLLTMPIKYEK--Y-F   1348
MMMoV     YDKS-YP----LYHVWNP---------DNIVEFDDSELVVYTSGDLQVLSNARKKR-F-F   1352
HoNV3     FWDGALP----LIHNWNP---------NKIQSFQDNELVVYHSADIPEFRNAQDL--F-I   1315
PCMoV     YCDL-PG----IYIFWNH---------QNIMMQDDREVVLYRDPALHSLSAKSYK--F-F   1324
AVA       YTDL-PG----VYLFWDH---------DRLLTFDDRELVLYRDAALHQLPSKSKK--F-F   1376
PBRSV     LSTKDV-----VEIHWEHSVA-------MKGKVKETEIVQYRCPSIPELPARLRG--Y-F   1393
TRSV      FSTRDV-----VEIHWEHDVV-------RKGEKKDTEIIQYRCPSIPELPSRCKK--Y-F   1368
AeRSV     FAGREC-----VEIHWEHDTA-------RKGKREETEIIQYRCPSIPELPSKFRK--Y-F   1383
HoNV1     FADIERT----IIICWDR---------SKMVEYDDNELITYHDVMIPVLSKAKEDFLF-P   1388
HoNV2     YND--KP----VYVTWRR---------SDIVEFEDSELVVYINNGLLPTSEKDADLLF-C   921
MMLRaV    MSDGTR-----VTFLWEA--S-------RLHEYAESEICRYFSPAIPPLRESLAR--KWY   1173
RpRSV     VAGRSF-----RTFQWRAS---------ALTEYAESEICTYFDSRIPSLGKQAMK--MYS   1360
BRSV      FSSTGES----QLIRWHK---------YHMREEPGSEIVTWLAPSLPSLSPDLKD--L-F   1330
PoLNVA    FASTGES----QLIRWHS---------YHMREELGSEIVTWLAPSLPTLSPDLKD--L-F   1345
TBRV      FASTGES----QLIRWHK---------YHMREEHGSEIVTWLAPSLPALSPDLKD--L-F   1330
AILV      FASTGES----KLINWHK---------YHMREEPNSEIVTWLAPSLPALSPDLKD--L-F   1344
RCNVA     FLSTGES----KLIRWHR---------RHMREEPGSEIVNWLAPSLPSLPTDLKD--L-F   1320
GARSV     FASTGES----QMIRWHK---------YHMREEPGSEIVTLACTKSSPSFLVICD--L-F   1307
GCMV      FASTGES----MMIRFHA---------YHIRENVGSEVVCWLAPSLPQLPCDLKG--L-F   1314
PVB       YSTDGER----KMVNWHQ---------CHMTEIHQSEIVLWTAPSLSQLPHQYAK--L-F   1337
CNSV      YLATGES----VVLTFNR---------DDVQEFPNHETCMWQAAGMLQLPAKFKD--C-F   1378
ToRSV     YTDNNGNTKAPLTHFFQPTGPNG---EHFLRFFNGTEVCIYSHPQLSALPGAPQN--Y-F   1351
AnNVA     YVTNNGAFMPRIEHVWQTTNQFG---VPNLRQFDSTEVCVYTHPELSALPCAPGS--F-F   1131
CLRV      YMTAAGTPSVPLEHIWDPQETAP---TPNLRRFNDTEVCVYTHPQLSPLPGPLES--M-F   1260
STeNV     YTNNQGVSQTPISHYWEPSED-------HLLRFSDTEICVYKHPQLSALPAAMQG--L-F   1233
BaNV1     YTNNRGECCEPLLIFWKSS---------CLKQFEDTEACIYSHPLLAPLEDPPQS--V-F   1140
BLSV      YLDRNHIP-KCFNFTWHYG---------NALEQDDTEVCIYRDDQLTPLPVYSRN--L-Y   1302
PRMV      YLDKARIA-KCFPMTWHWV---------NAIEEKDTEVCVYRDDQLTPLPVYPDS--F-Y   1297
CawYV     YLDKNRVA-RNFPITWHWN---------NVLEQDDTEVCVYRDDQLTPLPVYPDS--L-Y   1341
SLSV      YFSKERRP-VCFSFTWHYI---------NAIEWPDCEVIVYRDAQLTPLPVYSDD--N-Y   1332
PVU       YLSHDRIP-QCFSFTWHYI---------NAIEWPDCEVMVYRDDQLTPLPVYNTN--N-Y   1082
BRV       YYGRGRA-VQTIPLSWSY--K-------KVREFADTEAVLFLDAQLSTMPAGREH--Y-F   1237
GBLV      TRSANGV-VHTVQLLWDA--K-------QVRRYSDTEAVTYHDARFTALPEPIKS--A-F   1240
BLMoV     SRLQDGS-VRAVRIIWDS--K-------NIREYAGTEAVTYHDATLSPLPEPHKA--A-F   1261
AYRSV     YTARGVQ-SGKIEFLYDP--S-------RLTEFPDTELVQYADNVLSPLPNPAHN--A-F   812
GSPNeV    YRNSEGT-INSFSCIWNPTVRVCGGSRYGLFFPGETEVAIYRDPCLSPLPSANQS--F-F   1287
GNVA      YIDNAGQ-ECTMSHVWRHA---------NIVRQHGNELIVYKSPQFSHLPDAMKS--L-I   1331
                              :                    *
```

```
ArMV      VDDPDHQIS-PNVAVKCCVARLE-DGIP---------------QFHFW-NKYASA---RS    1386
GFLV      VDDADRQIS-PNVAVKCCVARLE-DGIP---------------QFHFW-SKYATA---RS    1387
GDefV     VDDADRQIS-PNVAVKCCVARLE-DGIP---------------QFHFW-NKYATA---RS    1387
MMMoV     LEDFEADLP-SKFRAHCAGVRLASDGTP---------------KTNEW-KS--DASVLTE    1393
HoNV3     QDYEKELTG-RNFNVSLVGARLDDKGSV---------------YRIHLDNVYAMRRDQMQ    1359
PCMoV     LHDDESQLA-PSLLVSRFGYRLNPDNNE---------------ILEVN-QSNLVAETVRE    1367
AVA       LLDDETRLA-PTMLVSRFGYRFDGISGQ---------------VADVR-RERLTASTVRE    1419
PBRSV     EYDLERDLP-GPFTLRASVYRMKSP-GK--IDL----------ELVDWTNHD---AELKT    1436
TRSV      EYDLERDFP-GPFTLDASCYRMQSP-GK--IDI----------ELVSWTDHD---RELRT    1411
AeRSV     EYDLERDLP-GPFTIDASVYRMKSP-GS--VEL----------ELVNWTNHD---AELIT    1426
HoNV1     WEKDENKLP-RTFQMESAVHKMGEDGMP---------------YREEW-TVNAEI---VH    1428
HoNV2     WKDDENALP-NSALYEHCVFRF-DDGQE---------------KRKIW-TRQGEL---VK    960
MMLRaV    LNDYEKHINMTTCDIYGVTVRRTGK-------------SYEEREIQWWKV-P---GSIVY    1216
RpRSV     DSDLDA-LNVKYFST--------RTL--------HFRLVDDQVEKRHWDA-D---ACVIS    1399
BRSV      LEDKEVDLP-NHFKTIGYVLRVDNTAFH---------------YDLLD----TYAAVDK-    1369
PoLNVA    LEDKEVDLP-NHFKTVGYVLRVDNTAFH---------------YDVLD----TYGAVDK-    1384
TBRV      LEDKEVDLP-NHFKTIGYVLRVDSTAFH---------------YDTLD----TYGAVDK-    1369
AILV      LEDKEVELP-NHFKTIGYVLRVDSTAFH---------------YDMLD----TYAAVDK-    1383
RCNVA     LEDKEVDLP-NHFKTVGYVLRTDDTSFH---------------YDTLD----TYASVDK-    1359
GARSV     LEDKETELP-SHFQDDGLCVESCQNCIS---------------LCTLD----TYGAVNKY    1347
GCMV      LEDAEVELP-SNFKSMGYVLRQDSNAFH---------------YDTLD----TYAAVDK-    1353
PVB       LEDAEVEMP-LNFKAMGYVLRNDKDGYH---------------YDTLD----TYATVDR-    1376
CNSV      LEKGETELA-PAFELEGYVLRPDSTAFI---------------MTILK----TWARVQY-    1417
ToRSV     LKDVEK-IS-GDIAIKGCGIKLGRTSVG-----ECVGVKDNEPVLNHWRA-V---AKVRT    1400
AnNVA     VKDIEK-FT-GDIVVKGAVIKLGRESAF------TESLRANEPFLHHWEA-V---VSVRT    1179
CLRV      VEDMQA-GP-SVYHIEGRVMKLVRDSHE--FLPNDFVGAPEEIVPHVWSG-V---VHLNT    1312
STeNV     VKDVEL-LP-RMLNFNGCVVKMAGESAQ--FVGE--VKNANEPILHMFGG-I---ISLNT    1283
BaNV1     LADYER-LQSRTLFAKGCVVKLSENSLE--FLNEGSIFKPGQPLLHHWEG-F---MTLQT    1193
BLSV      IPGGEK-LA-SAVNINGVCIKKRKFFHADSLTQAERQLDGETPIIRSWSN-V---GALCT    1356
PRMV      LRGEIQ-LP-SAININGVSIKKRRYYEDASLTPDERLLDGDSPIIRSWSN-V---AALST    1351
CawYV     LRADTK-LP-TALNINGVSIKKRKFFEDSSLTPDERLLEPDSSILRTWSN-V---AALST    1395
SLSV      LCGEQT-LP-TVLTITGLSIKKKKFFDDSTLCPSEKILDGEMPIVRSWKD-T---ASLRT    1386
PVU       LRGEQK-LP-TVININGVAIKKRKFFDEKTLTAGEKTLDGETLLIRSWSD-V---ATLCT    1136
BRV       NVPVER-LP-SVFDMNGVVMKQKRYMTD--SDDSLAAFTPNQPVVNTWENSR---AKLNC    1290
GBLV      ECDIDQ-LP-TLFDMNVVLVKRKSAMRQ--VDPTLAALPAEQPIIDCWKS-N---GKLNR    1292
BLMoV     DVDIEA-LP-TLFDMNVVVVKRKSTLRH--VDPSLQALPAEQPIIDRWSS-N---GKLNR    1313
AYRSV     AFDHAK-LK-GTLQLFGAVIKLRRHCGD--HTIGLENVDGDVPTLHKWDA-I---GNITT    864
GSPNeV    CSDYEAQLT-PYPELKFVGLKLKQFGHA----NHISHASDTTPICHMWNG-S---GVVMY    1338
GNVA      CSDPDTQLS-THFEITVLAS--------------QISKATRTPTYRHWRT-T---AVAKE    1372
```

```
                                          Catalytic triad      Substrate-binding pocket
ArMV        DVHTIKD--EGGSAVYQNKIRRYIIYAHEAKRNDCGAIAVAEI----QRTPKVLAMLVSG    1440
GFLV        EVHTLKD--EGGGNVYQNKIRRYIVYAHEAKKYDCGALAVAVI----QGIPKVIAMLVSG    1441
GDefV       ETHTLKD--EGGGNVYQNKIRRFIVYAHEAKKYDCGALAVAVI----QGVPKVIAMLVSG    1441
MMMoV       KATIVRY--QGKQCVYQRQIPKYVVYPFEAKDHDCGALCMAFV----NKQWKVTSLLVGY    1447
HoNV3       EIVIQDP--QTGDVIYSQVIPKYIEFGMEAQDQDCGSILVSTI----NRKIKVVGIMVAG    1413
PCMoV       T-LSVAK--SSGGFHYKRIINKFIRVEGIAKDWDCGTLIASMI----NGQMRVVGMLVGG    1420
AVA         T-LAVRR--TNGGYTYERILNKFIRVDGLAQDDDCGTLVATLI----GGQPRIVGMLVGG    1472
PBRSV       KALVISD--PFGEDRYRREYPRYISYRRSAQLHDCGAICVAQI----GGQYRVVGLLIST    1490
TRSV        RPLVIAD--PFGEDRYRREIPQYIQYGRPDQLHDCGAICVAKI----GGQHRIVGLVIST    1465
AeRSV       KALVISD--PFGEDRYRREYPRYIRYRRQAQLHDCGAICVTKI----NGHHRVVGLLIST    1480
HoNV1       KPLTVEW--EGQSAFYQRKIPRWIRTDFQAVDYDCGAIGATVV----AGKKRIVSMLVAG    1482
HoNV2       TKVVVT------RSNYKREVPTWIRTNYLGIDNDCGAIAVTTI----RGKKRIVGLFIAS    1010
MMLRaV      KQLQIDD--AYMGGTYVHYVPKYIHYSAQTQLHDCGAYVCALI----AGDWRIIGFHISR    1270
RpRSV       TPKTIVS--TINGVIYRQEIPTAITYRRESVKHDCGALVFTEV----RGKPKAVGMLVGT    1453
BRSV        TPLPLKG--VVGNELYLHEIPEKITFHYESRNDDCGMIILCQI----KGKMRVVGMLVAG    1423
PoLNVA      TPLPLKG--VIGDELYLHEIPEKIVFHYESRNDDCGMIMTCQI----KGKMRVVGMLVAG    1438
TBRV        TPLPLKG--VVGNELYLHEIPEKIVFHYESRNDDCGMIMLCQI----RGKMRVVGMLVAG    1423
AILV        TPLPLKG--VLGSELYLHEIPEKLTFHYESRNDDCGMILTAQI----RGKMRVVGMLVAG    1437
RCNVA       TPLPLKG--VIGNDLYIHEIPEKIVFHYESRNDDCGMILTCQI----RGKMKVVGLLVAG    1413
GARSV       APLPLKG--VVNDDLYLHEIPEKIVFHYESRNDDCGMILTCQI----KGKMKVVGMLVAG    1401
GCMV        TPLVLKG--VNGDDLYIHEIPEKIVFHYESRNNDCGMLLTCQL----SGKMKVVGMLVAG    1407
PVB         TPLPLKD--FSRGNCYSHEIPEKISFHYEARNHDCGMLILARI----SERYKVVGLLVAG    1430
CNSV        EPFVVRG--SLAKEKYVNELPTSIWFQYQSRNNDCGMVCLAQV----GGKKKIVGLLVAG    1471
ToRSV       TKITIDN--YSEGGDYSNDLPTSIISEYVNSPEDCGALLVAHL----EGGYKIIGMHVAG    1454
AnNVA       SEFQIDN--YEYGGDYCKKLPNSLVGEYINEVEDCGGILVAKV----ADSYRVVGMHVSG    1233
CLRV        HALTIDN--YKWGGDYKINIPRSLVGSYPNAKEDCGGLLFAKI----HNSYKVIGMHVSG    1366
STeNV       TALTIDN--YQWGGDYSKMIPKSWTGNYPSFKEDCGAILVAKL----QDGYKIVGMHVAG    1337
BaNV1       SEHIISD--NAERDAYTRKLPRYWVGAYANANEDCGGILVSKI----EGKFKVIGMHVSG    1247
BLSV        SKQTISN--PKPGVAYSRTLNRYLNSTYASGVHDSGGLITTIK----DGVRKVVGLHVAG    1410
PRMV        SVQTISN--PAPGIAYKRDLNRYLTSSYAAGVHDSGGLISILH----QGRRKVVGLHVAG    1405
CawYV       TKQTISN--PAPGVAYSRDLNRYLTSSYAAGVHDSGGLISIMH----QGQRKVVGLHVAG    1449
SLSV        QVQTILS--E----THRRDINRYYTSSYPSGKHDSGGIITAVI----NGKRKVIGMHCAG    1436
PVU         SVQTITG--P----NYRRDINRYYTSTYPAGVHDSGGLITTMH----NGRRVVVGLHCAG    1186
BRV         ERQGINT--FAYGGNYRNELPRSISSNCNTSPEDCGAIMTMIF----EGRRVVVGMHVAS    1344
GBLV        EMVGFNT--YAYGGSYRNEIPVSIVSKCKTYAEDCGAILTTMW----KGKRRVIGMHVAS    1346
BLMoV       TMQSFNT--FAYGGMYRNEIPISICSKCPTYAEDCGAMLTTMW----KGQRRVIGMHVAS    1367
AYRSV       TRQTIST--FVDGAPYYNDIPKYLHSNSPTTVEDCGAIMTALV----DGEYRVVGIHVAG    918
GSPNeV      ARHRIQTTESDGSVSYYNDLPKYIASTTSVGPEDCGTIVTTDLVVNNQTRQVIVGMVVGG    1398
GNVA        EDLPISD--AYSSGTYKYIVKRYIHSMGAGKPDDCGALVCTNV----GGVLKVVGMHVAG    1426
                           :                 *.*                  .:  .
```

Pro-Pol cleavage site

```
ArMV      IG-------------NVTYSSVIPSY--SSSFVRG---DVP--YVPEDGIKTNGYRKVGY   1480
GFLV      NR-------------GVTYSSVIPNY--SSSFIRG---EVP--YVPEDGLVSRGYRKVGY   1481
GDefV     NR-------------GVTYSSVIPAY--SSSYLRG---DVP--YVPEDGIVTSGYRKVGY   1481
MMMoV     AD-------------GRTTCSLLPAW--QPMEAKS---VLS--YNEEIVEIAPGYCKVGW   1487
HoNV3     AG-------------KFSQAALIPDY--ASRMIGKSLQSVLDSVQPEFVPTSAGYQKVGY   1458
PCMoV     TN-------------TNFVADIIPKF--IQVAAKG---LAR--IQEEFGVQENGYAKIGY   1460
AVA       SG-------------NTFCADIIPQF--HEAEARG---SVR--INQEFGIQEPGYAKLGY   1512
PBRSV     DKYNT----G---------VGLLPSAL-HMTTCSL---TYV--P-EEWEDAPRGLKKLGW   1530
TRSV      DKHNT----G---------VGLLPSAL-HMTTCSL---SYV--P-EEWEEAPRGLKKLGW   1505
AeRSV     DKYNT----G---------VGLLPSAL-HMTTCSL---SYV--P-EEWEDAPRGLKKRGW   1520
HoNV1     TG-------------RNCGVCFLPYV--ERMQARS---SIYDFLVEEAGEYADGYAKRGY   1524
HoNV2     RTIKPD-------VDGISSVCPLPSL--NIATARG---SIYDYMEQEDGRNEMGFAKIGF   1058
MMLRaV    KNGQC----A---------ATLIPDVI-EQDVQGF---S---FV-PKDGVLTDGYLKLGF   1309
RpRSV     LGGTT----------Y---VCKFPHIEVDAFACVP---DIRGFN-LEAGVSTLGYSKLGW   1496
BRSV      KD-------------KTSWADIMPPN--TLAELQS---QIE--YIPKFGEAYDGFFKAGY   1463
PoLNVA    KE-------------KTSWADIMPPN--TLAELKS---QIE--YIPEFGEACDGFFKAGY   1478
TBRV      KD-------------KTSWADIMPPN--SLAELKS---QID--YIPEFGEACDGYFKAGY   1463
AILV      KE-------------KTSWADIMPPN--SLAELKS---QIE--YIPEFGEACDGHFKVGY   1477
RCNVA     KD-------------KTSWADILPPC--TLAELKS---QID--YIPEFGEAHDGFFKVGY   1453
GARSV     KD-------------KTSWGDILPAN--SLAELRS---QIE--HFPKFGEAENGYFKAGY   1441
GCMV      KD-------------KTSWACILPNP--HLAELKS---QIE--YIPEFGEAEEGYFKVGH   1447
PVB       KE-------------KTSWACLLPNP--HMAELKS---SLE--YLPEFGEAEEGFSKIGY   1470
CNSV      VD-------------QQTWADNLPNP--CMAEMKS---QIE--YEFKLGAHTEGYTKLGY   1511
ToRSV     SSYPVE-VDGVQMPRYISHASFFPDYS-SFAPCQS---SVIKSLIQEAGVEERGVSKVGH   1509
AnNVA     SSYTVEGPDGREVRKYVSTAALFPDFD-SFESVQS---RIRSLELEDDGNPTRGVHKVGH   1289
CLRV      EQLAD--------GSYLSAAALLPRPS-LFMSAQS---GLRTLTI-EAGKDTRGVSKVGF   1413
STeNV     TRCAD--------GSFVSSAALLPAWN-CMADAHS---GPSTLQL-EAGRDTPGVKKVGW   1384
BaNV1     AHHND--------GTFTSIATLLPYFK-SFTSVQS---NLSI--QREGGIVTNGVVKVGY   1293
BLSV      TQEGH---------LFKSTVAFLPTG--SFADIHS---GEDFFV-PESGIETAGYVKIGY   1455
PRMV      TRVGH---------LFKSTISFLPHG--NFADVHS---QDDFFI-PEVGDREAGYEKIGF   1450
CawYV     IRSGH---------LFKSTISFLPHG--NYADVHS---TDEFFK-PEFVARTNGFDKIGF   1494
SLSV      AQNGK---------IYVSTIGLLPTR--NINDAHS---AADYFV-PSIGREEKGFCKIGW   1481
PVU       KQEGY---------LYKSTIGLLPTG--DISEAHS---SPDFFV-PSSGREEKGFCKIGW   1231
BRV       GKNPQ----G----RYMSTACLLPDYH-EDLSLNS---MLQ-YT-PYDGICKEGYRQIGN   1390
GBLV      GYKNG----TQNPKDWTSTATLLPGI--ADLECNS---GLN-MV-EESGVSYEGYRKIGY   1395
BLMoV     GYKDS----S--MKDWTSTATLLPTT--TDLTCNS---GIS-MV-EEKGCDYPGYRKLGW   1414
AYRSV     GVTPT----G----QYTSMACLIPYV--PHFECHS---RMPPLE-ERVGIDTLGCTKIGF   964
GSPNeV    GDGKD--------GRRKATIALIPEL--DTSETFS---SIN-FE-EEAGIDTEGVTKIGF   1443
GNVA      RDGRN----G---------ACFLPIH--SDMDAHF---NLAY--IPKPGRVTEGYSEIGY   1466
                                :*                             *  : *
```

```
ArMV       LM-AKDAPHVPSKTAFMKVPDEICFPY-----------------PNPKQPAILSAEDERL    1522
GFLV       LH-ASDAPHVPSKTSFMKVPDELCFPY-----------------PDPKQPAILSAEDERL    1523
GDefV      LH-RSDAPHVPSRTAFMRVPDELCFPY-----------------PNPKQPAILSTEDERL    1523
MMMoV      LP-VDQTPHLPKKSQFVEVPLKYQPKN-----------------VEVKHPAILSKDDERL    1529
HoNV3      LK-VADVPIMPSKSQYTRVPENLRIPG-----------------VEVKQPAILSKDDPRI    1500
PCMoV      LE-AQDRPTMPTKSQYVKVPEEVRINV-----------------AECKEPSILHKDDPRL    1502
AVA        LD-NSQRPTMPTKTQYVRVPEEFCISV-----------------PECKEPAILCKDDPRL    1554
PBRSV      KH-VSELPHMPRKTQYELVNESIRIPF-----------------ENPKIPSVLVADDPRT    1572
TRSV       ND-RSELPHMPRKTQYVAVNEDLAIPF-----------------DNPKIPSVLVSDDPRT    1547
AeRSV      KH-ASELPHMPSKTQYVKVPEEFEIPY-----------------ENPKIPSVLVADDPRT    1562
HoNV1      LK-AEHRPRMPTKTTFVEVPEELRFS------------------NDTKEPSILTATDPRL    1565
HoNV2      LP-NEFRPKVVAKTQFQKVPEEFVMQG-----------------VETKEPAILSGSDERL    1100
MMLRaV     VE-PERAPRMPVKTQYVEVPRHVQLPAA---------------LQDCKVPSILSADDERI    1353
RpRSV      LD-RRHQPHNSEKTEFVPIPEKYHMD-----------------DVPCKIPAVLSAKDPRL    1538
BRSV       VP-MADAPTLPKKTNMVPVPQSLRVPC----------------DVPIKEPAVLTNADKRC    1506
PoLNVA     VH-KCDAPTLPKKTNMVPVPESLRVPC----------------DVPIKEPAVLTAEDVRC    1521
TBRV       VH-KSEAPTLPKKTNMVPVPESLRVPC----------------DVPVKEPAVLTKDDPRC    1506
AILV       VG-KADAPTLPKKTNMVPVPAHLRVPC----------------EVPIKEPAVLTKEDPRC    1520
RCNVA      VS-QAQAPTLPKKTNMVVVPESLRVPC----------------DVPIKEPAVLTKDDPRC    1496
GARSV      VA-PNLAPTMPKKTNMVRVPEELRVPC----------------DVPIKEPSIISPQDPRC    1484
GCMV       VS-PKEAPTMPKKTNSVQVPQALRVPC----------------DLPIKEPSIISKNDPRC    1490
PVB        VD-KKDAPGIPKKTALVAVPEALLIPV----------------NVPIKEPAVLCRDDPRC    1513
CNSV       LT-KDKTPHLPKKNNAVLVKPEYRIDS----------------PVPIKEPSIISAEDPRC    1554
ToRSV      IKDPAETPHVGGKTKLELVDEAFLVP--S--------------PVEVKIPSILSKDDPRI    1553
AnNVA      IKDPAESARCGGKTKLELVPEAFLIP--S--------------PVPVKIPSVLSGSDPRL    1333
CLRV       IKA-EEIPRAPRKSSFIEVEEELKVPVPP--------------GVPLKQIAILSNSDERL    1458
STeNV      IAA-KDIPRAPRKTQYQWVDKEYRVP--T--------------DEPIKEPAILSGDDPRL    1427
BaNV1      LDNPAQRPNCPSKTQYQKVGVELAIDPPK--------------GVEQKIPAILSGNDIRL    1339
BLSV       IKNPALRPHVSTTSQIGRVTPNLCIPLPTYNIDDDEHFVDADEVFEIKENAILSKKDERL    1515
PRMV       IDNPAKAPHTSTTTQLGRVPTNFEIPLPIFDEEEEENFVDAGETFEIKEPAILSKKDPRL    1510
CawYV      IEDPGKAPHISTSSQLGRVPKNFEIPLPVYDEDEEENFLDCGETFEIKEPAILSKKDSRL    1554
SLSV       IADPSKRPHAATKTALAPVPKELALPLPE--------------GVSVKIPSILSAADPRL    1527
PVU        ISNPAKRPHTGAKTALAPVDPLLALPLPH--------------GVQTKIPSILSKKDERL    1277
BRV        IENIGARPYTSGKTAFVAVPQHLLYSPPVLQEKLPGSAQPVTIQVEVKQPAILSKDDPRI    1450
GBLV       LPRMADRPYQSGKTMFVPVPEEMMYIPTNLSERFSDGT-ERKVEVEIKQPAILSASDPRI    1454
BLMoV      IPKMSDRPYITGRTMFEPVPEELKYQPTNLVETFSDGT-ERKVEVEIKQPAILTPTDSRI    1473
AYRSV      VSNPVERPYYATKTQFVEVPEMVKIPY-----------------HDVKIPSVLAKGDTRL    1007
GSPNeV     IRNKQDRPVLPKKTQYQRVPEDLQLDI-----------------EVKKQPAIISKGDERL    1486
GNVA       LE-PQESIRPPKNTKLVEVPRDIRFPVP-----------------TAKVPAIIHKDDSRA    1508
                        .    :                            *  :::   * *
```

```
ArMV        IGTV------------HEGYTPIREG-MKKFAEPMHLLD--------------AQL----    1551
GFLV        KGTI------------HEGYTPLRDG-MKKFAEPMYLLE--------------EKL----    1552
GDefV       KGTV------------HEGYDPVKDG-MKKFAEPMSLLD--------------EKL----    1552
MMMoV       PKILADLEKKEGLPKGSMSYDPLING-LEKFAEPMEAVD--------------EGL----    1570
HoNV3       PHSN------------NPKFDPYKDG-MTKYAHPMDVLD--------------DKV----    1529
PCMoV       KEM-------------GKVYDPLKDG-LKKYKEPMKTLE--------------QNV----    1530
AVA         AAM-------------GKQYDPLRDG-LKKYAQPMGRLE--------------EPL----    1582
PBRSV       I------------GTPVEGQDPVLKA-MEKFYEPMLNFD--------------ETPV-AN    1604
TRSV        V------------GTPVEGKDPVLVA-MEKFYEPMTDFT--------------EEEVRPG    1580
AeRSV       I------------GTPVEGQDPVLVA-MEKFYEPMLDFD--------------DTFV-AG    1594
HoNV1       AG---------------KKYDPQREG-YKKFANPMKYLDAHEEFVHEDGSVEEREV----    1605
HoNV2       KG---------------KPYDPLKNG-MDKFSDPMLDLDAMEDFEGEEGSVK-RSV----    1139
MMLRaV      --------------PQGVTYDPYIQG-MEKFATPMSELD--------------DEI----    1380
RpRSV       ADI-----------PQCMGYDPYKQG-MEKFAHPMQEID--------------EQL----    1568
BRSV        PAG----------------VNPPVTALKKKFEHPMKELE--------------QEI----    1532
PoLNVA      PPG----------------VDPPRAALRKKFTQPMEELE--------------QDI----    1547
TBRV        PIG----------------VDPPRAALKKKFTQPMMELE--------------QEI----    1532
AILV        PAG----------------VNPPIAALKKKFTQPMAELE--------------QEI----    1546
RCNVA       PPG----------------IDPPIAALRKKFSQPMDELE--------------GDL----    1522
GARSV       PSG----------------VDPPKAALKKKFSEPMAELE--------------QEV----    1510
GCMV        PAN----------------VDPPKAAMKKKFQQPMLDLD--------------QKC----    1516
PVB         PEG----------------VDPPMKAFKKKFTQPMLELE--------------QEL----    1539
CNSV        PKDA-----------EGKPIDPIVKAFEKKFTTPMDLLE--------------DDI----    1585
ToRSV       P-------------EAYKGYDPLGDA-MEKFYEPMLDLD--------------EDV----    1581
AnNVA       P-------------DHLKGTDPLAHA-MDKFHNPMRELD--------------DSV----    1361
CLRV        K------------GTQFEGYDPLRQA-TVKCEDPMFDLR--------------SDV----    1487
STeNV       S-------------MDHKGYDPLRNA-TNKCEQPMLEMD--------------SQI----    1455
BaNV1       I------------DTPHEGYDPLKSC-VDKFQEPIGLLD--------------SKV----    1368
BLSV        KDLI---------S-NPEAFNPLQDG-MVKFANPMLPLD--------------EEI----    1546
PRMV        E--------------DPDSFDPLRTG-MGKFANPMSVLD--------------ETL----    1537
CawYV       E--------------DPDNFRPLQDG-MMKFANPMKVMD--------------ETI----    1581
SLSV        RDEV---------NPEFKDYDPLRVG-MDKFANPMDPLD--------------DEV----    1559
PVU         KTEV---------NPEFKDYDPLKDG-MSKFANPMALLD--------------EDI----    1309
BRV         --------------PEGTSYDPLIDG-MAKFSHPMAVLD--------------ENV----    1477
GBLV        --------------PEGVSYDPLKNG-MEKFKKPMDLLD--------------DAL----    1481
BLMoV       --------------PAGMKYDPLVQG-MEKFKQPMDLLD--------------DGL----    1500
AYRSV       R------------GTEHEAYDPLKNG-MSKYEEPMSLVD--------------EKL----    1036
GSPNeV      I------------GTPHEKFDPIHEA-PNKYANPMKELP--------------QDL----    1515
GNVA        -----------------DGFNPYEAG-MTKFAQPMADLD--------------EEV----    1532
                                 *       *   *:  .
```

```
ArMV        ------LDEVAGDMVHTWFDAGE------------ILEDVPLSIAINGDV----------    1583
GFLV        ------LDEVAGDMVQTWYDPGE------------FLEDISLDQAINGDM----------    1584
GDefV       ------LDEVAGDMVHTWYDPGE------------FLEDISLDQAINGDE----------    1584
MMMoV       ------LRHVADEIVEEWFDCLEG-----------SLSDVSLDVAINGADG---------    1604
HoNV3       ------LQEVADEIVDSWFDCNT------------CLSDVGLDVAINGVPEEYFDALST-    1570
PCMoV       ------LKEVAGDIVTTWYDCQEG-----------DLEDVDIDIAINGDE----------    1563
AVA         ------LKEVAADIVNTWYDCQEG-----------FLEDVSLDVAINGDD----------    1615
PBRSV       STEMAIFESVCDDIVQTWYDAGAD------------FEDVDDDVVING------------    1640
TRSV        QTEVSLFEQVCDDIVQTWFDAGAE------------FEDVEDDVVING------------    1616
AeRSV       STERDLFEQVCDDIVQTWYDAGAC------------FEDVDDDVVING------------    1630
HoNV1       ------LDIIAEEMISEYFDAEP------------ALENVSLSVALNGIPFEE-------    1640
HoNV2       ------LDIITDEMIEEYFDAAD------------CLENVDLDTALNGSFG---------    1172
MMLRaV      ------LRRVADDIVEEWHECES-------------FEDVTLDVAINGVDHEEL------    1415
RpRSV       ------LATVCDEIAQEFHDVGVR------------GRMVSMDEAINGHHKYEIPSFYVE    1610
BRSV        ------LDEVATDILETWYDCEDH-----------VLNDIPLVVAINGIPADS-------    1568
PoLNVA      ------LDEVASDILDTWYDCEDH-----------VLTDIPLSVAINGIPADS-------    1583
TBRV        ------LDEVATDILETWYDCEDH-----------VLSDISLSVAINGIPADS-------    1568
AILV        ------LDEVATDILETWYDCEDH-----------VLSDIPLEVAINGIPADS-------    1582
RCNVA       ------LEQVADEILETWYDCEDH-----------VLSDIPLSVAINGIPAGG-------    1558
GARSV       ------LDEVATEILETWYDCEDH-----------ILEDIPLSVAINGIPAGG-------    1546
GCMV        ------LDEIAGDMLETWYDCEDS-----------ILSDIPLATAINGIPAGC-------    1552
PVB         ------LDEVAMDILETWYDCEDH-----------VLSDIPLEVAINGIPAGC-------    1575
CNSV        ------LESIAQEMVDEWQDCESE-----------PLCDVPLEVAINGIPGTQ-------    1621
ToRSV       ------LESVMADMYDEFYDCQTT------------LRIMSDDEVINGSDF---------    1614
AnNVA       ------VGDVIDDIYDTFYDCSPN------------LSLMDDEEVINGSPF---------    1394
CLRV        ------LEDVLEDVLETWFDCAPS------------LSLLSDEEMVNGNDE---------    1520
STeNV       ------LHEVATEMVERWYDCNPQ------------LSNLSDFEMINGNDE---------    1488
BaNV1       ------LDEVAAQMLEEWFDCPSD------------LRILSDEETINGDDN---------    1401
BLSV        ------LEKVCDDMFDTWYDMLPQTDMG----APQFLQKVDLDIALNGIPD---------    1587
PRMV        ------LEAVCEDIFTTWYDALPTVTDNQGNVSRVLLEKTSLDIALNGIPG---------    1582
CawYV       ------LVDVCEDIYISWYDSLPTKTAADGSTSKIFLEKVDLDVALNGVTG---------    1626
SLSV        ------LQRVCEDIYETWYDCLPILDD-----EKVFLQKTSLEVALNGIPG---------    1599
PVU         ------LSAVCEDVYQTWYDALPIFDD-----EKQFLEKTSLDVALNGIPG---------    1349
BRV         ------CNEVAQDIVESWHDCFQD------------LQDVSDEIAINGST----------    1509
GBLV        ------CAEIANDIAESWHDCFDS------------LEDCSDEVAINGAE----------    1513
BLMoV       ------CAEIASDIAESWHDCMDA------------LEDTTDDVAINGAE----------    1532
AYRSV       ------LREVAQEIVETWHDCEDG-----------TFGDADDDVVINGIDG---------    1070
GSPNeV      ------LEETANDILKDWKQFLGN----------QSLKIESLDNALNGIEG---------    1550
GNVA        ------LAFVANEIVEEWQDALDE-----------ALDDAELHEAINGVMDD--------    1567
                        ::   : :                         :**
```

```
ArMV        ------------------------------------------------------------    1583
GFLV        ------------------------------------------------------------    1584
GDefV       ------------------------------------------------------------    1584
MMMoV       ------------------------------------------------------------    1604
HoNV3       ------------------------------------------------------------    1570
PCMoV       ------------------------------------------------------------    1563
AVA         ------------------------------------------------------------    1615
PBRSV       ------------------------------------------------------------    1640
TRSV        ------------------------------------------------------------    1616
AeRSV       ------------------------------------------------------------    1630
HoNV1       ------------------------------------------------------------    1640
HoNV2       ------------------------------------------------------------    1172
MMLRaV      ------------------------------------------------------------    1415
RpRSV       GASTRELNELRTSCSAEVWCCDPRSDIEFEYPRIIPGPVESKWKCESTCCGCTFKSGGTE    1670
BRSV        ------------------------------------------------------------    1568
PoLNVA      ------------------------------------------------------------    1583
TBRV        ------------------------------------------------------------    1568
AILV        ------------------------------------------------------------    1582
RCNVA       ------------------------------------------------------------    1558
GARSV       ------------------------------------------------------------    1546
GCMV        ------------------------------------------------------------    1552
PVB         ------------------------------------------------------------    1575
CNSV        ------------------------------------------------------------    1621
ToRSV       ------------------------------------------------------------    1614
AnNVA       ------------------------------------------------------------    1394
CLRV        ------------------------------------------------------------    1520
STeNV       ------------------------------------------------------------    1488
BaNV1       ------------------------------------------------------------    1401
BLSV        ------------------------------------------------------------    1587
PRMV        ------------------------------------------------------------    1582
CawYV       ------------------------------------------------------------    1626
SLSV        ------------------------------------------------------------    1599
PVU         ------------------------------------------------------------    1349
BRV         ------------------------------------------------------------    1509
GBLV        ------------------------------------------------------------    1513
BLMoV       ------------------------------------------------------------    1532
AYRSV       ------------------------------------------------------------    1070
GSPNeV      ------------------------------------------------------------    1550
GNVA        ------------------------------------------------------------    1567
```

```
ArMV        -------------EEEYFDPIAMDTSEGYPEVLQRKN-G-----EKGKARFFV-GEPG--    1621
GFLV        -------------DEEYFDPLVMDTSEGYPDVLDRKP-G-----EKGKARFFV-GEPG--    1622
GDefV       -------------EEEYFDSLVMDTSEGYPDVLDRKP-G-----EKGKARFFV-GEPG--    1622
MMMoV       ------------EAEKFFDPLVMSTSEGYPFTLTRTG-G-----ETGKARFFE-GLPG--    1643
HoNV3       -----DAEGQPIFIGEFYEKMVESTSEGYPHVLTREH-G-----EKGKSRFLE-GPIG--    1616
PCMoV       -------------FEDFFDPMVMSTSEGYPFVLERKG-G-----EGGKERYFE-GLPR--    1601
AVA         -------------VEDFFDPMVMSTSEGYPFVLERKN-G-----ERGKERYFE-GLPR--    1653
PBRSV       --------------DDEFDKLIMDTSEGYPYVLERSR-G-----EKGKTRYFEGGPG---    1677
TRSV        --------------DDDFDKLIMDTSEGYPYVLERTH-G-----EKGKTRYFEGGPG---    1653
AeRSV       --------------NDDFDKLIMDTSEGYPYVLERSH-G-----DKGKTRYFEGGPG---    1667
HoNV1       -----------SGCDTYMDPIVQNTSEGYPYVLSRSN-G-----EKGKGRFLE-GLDG--    1680
HoNV2       ------------DDGEYMDPIVTSTSEGYPYVLHRGR-NEDGTAQKGKARFLN-GFPG--    1216
MMLRaV      ----------DEDEAEFLDPMVMNTSEGYPFVLERKN-Q-----ESGKARYFEGVPG---    1456
RpRSV       AIISFVKARSPCCEEIFFDGLDLTTSEGYPLFLDRPA-G-----AKGKERFFEGSENQ--    1722
BRSV        -------E------EAELENFVMKTSPGYPYFKNNRAEK-----LKGKSAYFEEAEDG--    1608
PoLNVA      -------E------EAELENFVMKTSPGYPYFKHNRKEK-----LKGKHAYFEEDEDG--    1623
TBRV        -------E------EAELENFVMKTSPGYPYFKNNRAEK-----LKGKHAYFEEAEDG--    1608
AILV        -------E------EAELENFVMKTSPGYPYFKNNRAEK-----LKGKSAYFEEAEDG--    1622
RCNVA       -------E------EAELENFVMKTSPGYPYFKNNRELG-----VKGKHAYFEELEDG--    1598
GARSV       -------E------EAELENFVMKTSPGYPYFREVKN-V-----GKGKHPYFEEAEDG--    1585
GCMV        -------E------DAELENFVMKTSPGYPYFKNKG--L-----GKGKHPYFEECEDG--    1590
PVB         -------E------EQELENFVLKTSPGYPYFKENRKTH-----TKGKHAYFQEEEDG--    1615
CNSV        -------IDDDDEFEDAVECLKMRTSPGYPYVLHKE-PG-----MKGKEAYFELAPDG--    1666
ToRSV       --------------GFNIEAVVKGTSEGYPFVLSRRP-G-----EKGKARFLEELEPQPG    1654
AnNVA       --------------GYQVEAIVQNTSEGWPHVNDRLP-G-----EKGKARFLEEYAVRAG    1434
CLRV        --------------EVFLDAVVHSTSEGYPYVLERGP-G-----EKGKERYLEQDPALP-    1559
STeNV       --------------EQFVDAVVHNTSEGYPYVLERQP-G-----DKGKERYLEQVPDAP-    1527
BaNV1       --------------DEFVDCLVFNTSEGYPFVLERGP-N-----DRGKARYFEPMPTNP-    1440
BLSV        --------------DACMEAMKLDTSEGYPHCVERAP-G-----ESGKRRFVEVDDTF--    1625
PRMV        --------------DAYLEPMKLDTSEGYPHCVKRGP-G-----ESGKRRFVEIDDNF--    1620
CawYV       --------------DAYMEAMKLDTSEGYPHCVRRAP-G-----ESGKRRFVDIDDDF--    1664
SLSV        --------------EACYEAMRLDTSEGYPFVLERKP-G-----ESGKLSYVHIDENG--    1637
PVU         --------------EACYDAMRMDTSEGYPYVLERQP-G-----ESGKARYINIDDDG--    1387
BRV         --------------EMDYEPFNLQSSEGYPYVTQRKP-G-----ESGKIRFFEMDPYT--    1547
GBLV        --------------DEFFDKFNMTTSEGYPWVKQRGI-G-----ESGKLRYFEETVNG--    1551
BLMoV       --------------DEFFDKFNMQTSEGYPWVKQRGI-G-----QSGKSRYFEEAGDG--    1570
AYRSV       --------------EDFFDALVMSTSEGYPYIKERNI-G-----EKGKARYFEPTGDG--    1108
GSPNeV      --------------EDYLDKMVENTSEGYPYVLSRTR-G-----EKGKGRFLQPNPSDPD    1590
GNVA        ------------EGVTQEEGMVLSTSPGYPYTLEPH--V-----LKGKADYFEEADIP--    1606
                              : .   :* *:*                **  :.
```

```
ArMV            ----AREFVPGCGPERAYLSLE-------EECKT--RIPSLVSIETPKDERLKRSKIE--    1666
GFLV            ----NRAFVAGCNPEKAYYQLE-------EDSKT--KIPSLVSIETPKDERLKRSKID--    1667
GDefV           ----NRIFVAGCKPEKAYYQLE-------EDSKT--RVPALVSIETPKDERLKRSKID--    1667
MMMoV           ----ERTLLPNTPVEIAYRELC-------EY--E--GIPELVCVECPKDECLVERKIL--    1686
HoNV3           ----ARKLKEGSSVHTEYLEFW-------DILQQ--EVPPLFTLACAKDERVSEAKVTT-    1662
PCMoV           ----ERSLKPGTSVARAYEELK-------LEAPH--TVPELVCMECPKDERLPKRKIE--    1646
AVA             ----ERELKSGTTVEVAYLDLC-------SVAPH--AVPELWCMECPKDERLPSRKIQ--    1698
PBRSV           ----AYTLKPGTSVYRDYHTLQ-------EEVSVEGGIPEMVCIECPKDELLVPRKVLE-    1725
TRSV            ----AYTLKPGTSVYNDYHKLQ-------EEVQVEGGIPEMVCIECPKDELLVERKVLQ-    1701
AeRSV           ----AYTLKPGTTVFHDYHALQ-------QEVSVEGGIPEMVCIECPKDELLPRRKVLE-    1715
HoNV1           ----ERSFIEGTQVHKDYLALK-------ETLYY--QVPELVVIECPKDECLPSRKIQ--    1725
HoNV2           ----EYTLKPDTEVSRDFEELVRCMEADQDPRLT--PVPEVVCIECPKDERLPKRKID--    1268
MMLRaV          ----SMQLKQGTTVYAAYAKLC-------AEIPH--SVPELVCIECVKDERLARRKVFE-    1502
RpRSV           -----KFLIPDCPLDVQLKKGI-------EETHL--GTPQLIIKESAKDELLKEGKVLPS    1768
BRSV            ----TLKLKEGGMAAKLHENLV-------EFTKN--EVPELVVIECTKDELLPERKIK--    1653
PoLNVA          ----SLKLKAGGLAAELHENLV-------EFTKS--EVPELVVIECTKDELLPARKIR--    1668
TBRV            ----SLQLKKGGMAAELHENLV-------EFTKN--EVPELVVIECTKDELLPERKIK--    1653
AILV            ----TLKLKEGGMAAELHENLV-------EFTKN--EVPELVVIECTKDELLPERKIK--    1667
RCNVA           ----TLGLKPGSVAEELHTNLV-------EFTKN--EVPELVVIECPKDELLPERKIK--    1643
GARSV           ----SLKLKEGTMASELHENLV-------EFTKS--EVPELVVVECPKDELLPERKIK--    1630
GCMV            ----SLKLKEGSQAAELYENMA-------QFAKE--EVPELVVIECPKDELLPARKIK--    1635
PVB             ----RMALKKNSLAEDLYLNLR-------DFTKQ--GVPELVVIECPKDELLPVRKVK--    1660
CNSV            ----TRALKEGSLAAELYENIV-------QYSKS--AIPELVVIECPKDELLKTEKVN--    1711
ToRSV           DTKPKYKLVVGTEVHSAMVAME-------QQART--EVPLLIGMDVPKDERLKPSKVLE-    1704
AnNVA           --KPLYRLIPGTSVHKAYVALE-------QQIAH--EVPMLIGMEVPKDERLKREKVLE-    1482
CLRV            --EGKLRVRPGTSVHRDLLALE-------KSIHF--TIPILVGMEIPKDERLKESKIL--    1606
STeNV           --EGHKRLKEGTSVHRDFLALQ-------KSIYC--EVPQLWCMEIPKDERLPRRKID--    1574
BaNV1           ---MKRQLIVGTSVQKEYVDLQ-------ERILE--EVPDLVTLQIPKDELLPPRKVTG-    1487
BLSV            ----HVSLKEGTKVYENYHNLS-------NTISK--NIPVLNCVECLKDECLKKRKVA--    1670
PRMV            ----HFSLKPDTDVFRNYQALS-------GTISQ--QVPVLNCVECLKDECLKKRKVA--    1665
CawYV           ----HVTLKEGTDVYDNYQKLK-------STVST--RVPTLNCVECLKDECLKKRKVA--    1709
SLSV            ----VRSLIPGTSVFRDYHELS-------ASILT--QVPVLNCIECPKDELLKPSKVLE-    1683
PVU             ----FRTLIPGTSVARDYEELA-------CNIFK--HVPVLNCVECPKDELLKPSKVLE-    1433
BRV             ---GLKSLIPNTLPAMRYEALQ-------RDCFT--SVPEMVCIETPKDECLPLRKICI-    1594
GBLV            ---Q-LALRKDTPVYKAYHDLQ-------ELSKV--EVPELICIETPKDECLPLRKITL-    1597
BLMoV           ---S-LIMKRDTPVYKAYNDLQ-------EISRM--QVPELICIETPKDECLPLRKITV-    1616
AYRSV           ---CRKQLIQGTMVAQDIEYLK-------ETVHE--IVPEIICVETPKDERLPVRKVTG-    1155
GSPNeV          G--IRKMLIPGCIIEKDLDILL-------NRAKS--HIPELVCIDTPKDELLPLRKIFD-    1638
GNVA            ---GELKLKVGSSAETMFKKLR-------KQSLG--DVPQLVCTACAKDERVDVKKVTT-    1653
                       .  .                           * :      *** :   *:
```

```
ArMV        --TPGTRLFSVLPLAYNLLLRVKFLSFSRLLMKKRSHLPCQVGINPYSREWTDLYHRLAE    1724
GFLV        --TPGTRLFSVLPLAYNLLLRVKFLSFSRLLMKKRGHLPCQVGINPYSREWTDLYHRLGE    1725
GDefV       --TPGTRLFSVLPLAYNLLLRVKFLSFSRLLMKKRGHLPCQVGINPYSREWTDLYHRLGE    1725
MMMoV       --KPKTRLFSILPLHYNLRLRQKFLSFSAFLMRNRHRLPCQVGINVYSREWLDIYTRLAQ    1744
HoNV3       --KVKTRLFDILPMSYNLILRQKFLAFSVHLMKNRHRLPCQVGINPFSSEWKGLYNRLKE    1720
PCMoV       --NPKTRLFAILPLHFNLRLREKYLAFGQFIMKNRNRLSCQVGINPYSREWKELYYRLAQ    1704
AVA         --NPKTRLFAILPLHFNLRLRVKYLAFARFIMKNRSRLACQVGINPYSREWKELYARLAH    1756
PBRSV       --KLGTRNFEILELPKNMLFRKKFLSWASFLSEMRWCLPCQVGIVVQGREWGLLLDRLSE    1783
TRSV        --KLGTRNFEILELPKNMLFRKKFLHWALFLSDMRWCLPCQVGIVVQGREWGLLMDRLAA    1759
AeRSV       --KLGTRNFEILELPKNMLFRKKYLSWALFLTEMRWCLPCQVGIVVQGREWGLLMDRIAS    1773
HoNV1       --NPKTRHFSILPMPYNIVLREYTLAFSAFLMRERKRLPSQVGIVPYSNEWGELLRRLYE    1783
HoNV2       --NPKTRLFSVLPMPYNIMLRMYYLKFSQHLMQQRHRLPCQVGINPYSREWTELYVRLCE    1326
MMLRaV      --KPKSRLFSILPLHFNLKLREKFLHFSKFIMQNRHRLPSQVGINVHSREWLQLYARLGE    1560
RpRSV       EGMPGTRLFSICPAWYNIVVRQHFVYIAESVRKRRRTLSSQVGIVVGSREWDDLAARLRS    1828
BRSV        --VGACRLFEIMPLHYNLFLRQKTCAFTQFLQHNRHVLPCQVGTNPYSREWGHMLNRLMR    1711
PoLNVA      --VGACRLFEIMPLHYNLFLRQKTCAFTQFLQHNRHKLPCQVGTNPYSREWGHLLNRLMR    1726
TBRV        --VGACRLFEIMPLHYNLFLRQKTCAFTQFLQHNRHRLPCQVGTNPYSREWGHMLNRLMR    1711
AILV        --VGACRLFEIMPLHYNLFLRQKTCAFTQFLQHNRHKLPCQVGTNPYSREWGHMLNRLMR    1725
RCNVA       --VGACRLFEIMPLHYNLFLRQKTCAFTQFLQANRHRLPCQVGTNPYSREWGHILARLMR    1701
GARSV       --VGACRLFEIMPLHYNLFLRQKTCAFTQFLQHNRHRLPCQVGTNAYSREWGHLLSRLMR    1688
GCMV        --VGPCRLFEIMPLHYNLLLRVKTCAFTAFLQHNRHRLPCQVGTNPYSREWGHLLNRLRR    1693
PVB         --EGACRLFEIMPLHYNLFLREKTCAFTQFLQNERHKLPCQVGTNPYSREWSHIFQRLAK    1718
CNSV        ---KACRPFEIMPLHYNLFLREKTLAFSLFQQRNRHKLACQVGTKAYSHDWTHMYQRLVA    1768
ToRSV       --KPKTRTFVVLPMHYNLLLRKYVGILCSSMQVNRHRLACAVGTNPYSRDWTDIYQRLAE    1762
AnNVA       --KVGTRTFTVLPMEYNLLLRKYVGKFASFIQTNRHRLACAVGTNPYSREWTDMFRRLAH    1540
CLRV        --TPATRTFTVLPMPYNLLLRKYFGRCVAFLQGNRHRLPCAVGINPYSNEWTRIFDGLAR    1664
STeNV       --NPKTRTFTVLPMPYNLLLRKYTGRFMAFLQGNRHRLASAVGVNPYSAEWTRLYDRLAA    1632
BaNV1       --KPKTRTFEILPMTHNLVLRKYTGKFCSFLQTNRHKLPCAVGTNAYSREWTNIYDRLAA    1545
BLSV        ----TPRLFDVLPFEHNILLREYFLSFSAFLQHCRIQLPCCIGVNVYSREWTTLYDRLRE    1726
PRMV        ----TPRLFDVMPFEHNILLREYFLNFSAFIQANRIYLPACIGTNVYSREWTTLYDRLAE    1721
CawYV       ----TPRLFDVMPFEHNILLREYFLSFSSFIQANRINLPACIGTNVYSREWTTLYDRLAE    1765
SLSV        --KPGTRLFDVLPFTHNILLREYFLNFCVFLQQNRVHLPCSVGVNPYSREWTWLFDRLAA    1741
PVU         --KPGTRLFDTLPFVHNLLLREYFLNFCVFLQHNRLRLPCSVGINPYSREWTWLFDRLAS    1491
BRV         --KPKTRLFSILPLEFNLLLRKKFLHFSSSLQMHRDTLPTQVGVNPYSREWGELLQRLRA    1652
GBLV        --KPKTRLFSILPLEMNLLLRKKFLSFAANLQQNRDKLPTQVGVDPYSREWGHIYSRLRS    1655
BLMoV       --KPKTRLFSILPLEFNLLLRKKFLSFAANLQQNRDKLPTQVGVDPYSREWGHIFSRLRS    1674
AYRSV       --KPKTRLFSALPLSYNFMLRKKFLYFVAFLQKNRGRLPCQVGINAYSREWQTLYNRLAE    1213
GSPNeV      --KPKTRMFSICPFPYNLALRQYTLRLVAFLQTNRRSLAAQVGIVPQSQDWDNLYQRMCS    1696
GNVA        --NKKTRLFEILPLHYNLLLRMKFMKFNSFMQRNRFRLSCQVGTNPYSRDWQDIYRRLRE    1711
                  * *       *: .*             *  *   :*    . :*  :   :
```

```
ArMV        K-SDVGYNCDYKGFDGLITEQILAVVATMINAGFRN----PV-----------SNQQRSN    1768
GFLV        L-SDVGYNCDYKAFDGLITEQILSTIADMINAGYRD----PV-----------GNRQRKN    1769
GDefV       L-SDVGYNCDYRAFDGLITGQILSVIADMINAGYRD----PI-----------GNQQRKN    1769
MMMoV       V-NDVACNCDYQSFDGLMTYQFLNVIGDMINRCYRDDSE--K-----------SLSQRKN    1790
HoNV3       V-STTAYNCDYSRFDGYLSFQILEVIANMINRGYKGDN--QQ-----------SKDVRAR    1766
PCMoV       Y-SDTAYNCDYSSFDGLMTHQVLDVIADMINAMYSKEET-PY-----------SKAERKN    1751
AVA         V-SSEAYNCDYSSFDGLMTHQVLNTIADMINLMFSADEE-PS-----------SKAERKN    1803
PBRSV       K-NSVAYNCDYSKFDGLMSCQVLDAIGKMVNKCYANTNPNKKGR--GEVPGSPPQLARHN    1840
TRSV        K-NSVAYNCDYSKFDGLMSCQVLDAIGKMVNKCYSNANPNLKKKGKGELPGSPPQLARYN    1818
AeRSV       K-NSVAYNCDYSKFDGLMSCQVLNAIGKMVNRCYSNENPNFRGRG-GEVPGSPPQLARHN    1831
HoNV1       H-NDRAYNCDYSSFDGLMSAQVMLRIARIINAGYKKDG--AR-----------RLKEREN    1829
HoNV2       T-NSEVYNCDYKAFDGLITQQCLKVIARIINAGYKKDS--PK-----------KKQIREN    1372
MMLRaV      K-NTRAINCDYERFDGLMTAQVLSVIGSMINRTYKDGKDLG-------------GKERHN    1606
RpRSV       KKNDKMYCCDYSKFDGLMTPQIVHAITNIYERMFSGNDGMS--------------QFRQN    1874
BRSV        PKTNEAINCDYSGFDGLLNAQVIECIAKMINRLYALSGESEV-----------QQAQRYN    1760
PoLNVA      PKTTEAINCDYSGFDGLLNPQLIECMARMINRLYALSGESEV-----------QQAQRFN    1775
TBRV        PKTNEAINCDYSGFDGLLNPQLIECIARMINRLYALSGESDV-----------QQAQRYN    1760
AILV        VKTNEAINCDYSGFDGLLNAQVIECIAKMINRLYALSGESEV-----------QQAQRYN    1774
RCNVA       PGTNEAINCDYSAFDGLLNAQVIECIANMINKLYALSGESEV-----------QQAQRYN    1750
GARSV       PKTTEAINCDYSGFDGLLTPQLVETIAKMINRLYALSGESEV-----------SQAQRYN    1737
GCMV        VKTNEAINCDYSGFDGLLTPQLVEMMAKMINRLYLRSGESEV-----------MQAQRLN    1742
PVB         R-NSVAINCDYSGFDGLLNAQLIETMAKMINRLYALSGDTEL-----------SQAQRYN    1766
CNSV        K-SDRAINCDYSSFDGLLNSQVVSCIANMINSMYHSPEETVV-----------SKRQRYN    1816
ToRSV       K-NSVALNCDYSRFDGLLNYQAYVHIVNFINKLYNDE-HSI---------------VRGN    1805
AnNVA       V-SNEAINCDYSKFDGLLNYQIYRHIVALINRTYGDN-HQF---------------KRHN    1583
CLRV        V-SPKALNGDYKGFDGKLNFQMYDAIARLLCCLHRDESTST---------------ARYN    1708
STeNV       K-SPMALNGDYASFDGLMNFQMYDIIARMINRCYRDDDHAT---------------ARYN    1676
BaNV1       I-SPTALNCDYSTFDGKLSGQMYQYIINLIDGRFNDE-HSV---------------ARKN    1588
BLSV        Y-SDTGLNCDYSKFDGYISHQIYGWLAATINRLYRDGVEAN--------------AARNN    1771
PRMV        Y-SDTGLNCDYSKFDGYISHQIYSWMAATINRIFRDGEEAN--------------SARRN    1766
CawYV       Y-SDTGLNCDYSKFDGYISHQVYSWMVATINRLFRDGEEAN--------------SARRN    1810
SLSV        K-SDKALNCDYSKFDGLISHQVYMHMVATINRLFRDGEEAN--------------CARKN    1786
PVU         K-SDRALNCDYSKFDGLISHQVYMQMVSIINRLFKDGEEAN--------------LARRN    1536
BRV         Q-SSVAINCDYASFDGLLTGQILEKIGTMINKMYIGSEASK--------------IQRLN    1697
GBLV        K-NSVAVNCDYASFDGLITAQILKHIGIAINSVYVGSPESK--------------RQRAN    1700
BLMoV       K-NSVAINCDYASFDGLITAQILRHIGNAINSMYKDDDASK--------------KQRHN    1719
AYRSV       R-SENALNCDYSSFDGLMTGQMLSCIGDMINTMYGDSQKSK--------------NGRKN    1258
GSPNeV      MNADEGYNCDYSGFDGYLTAQVVDVIANMFNSMFAGETQND-------------QAIRYN    1743
GNVA        K-NDVALNCDYARFDGILSYQVLNCIGNMINECFVDDKVSR--------------RQRKN    1756
                     **  *** :. *    :       .                       * .
```

```
ArMV            LLMAISGRLSICGSQVYETEAGIPSGCALTVVINSIFNELLMRYCYKKIVPP----I-YR    1823
GFLV            LLLAICGRLSICGNQVYATEAGIPSGCALTVVLNSIFKELLMRYCFKKIVPP----V-YK    1824
GDefV           LLLAISGRLSICGNQVYATEAGIPSGCALTVVLNSIFNELLMRYCFKKIVPP----L-YK    1824
MMMoV           LLLALYQRKSIAGNQVYSLRAGIPSGCALTVLLNSLFNELLVRIAYRSLVPG----V-NR    1845
HoNV3           CLYAIFGRYTIAGNQVYRVRSGMPSGFALTVVVNSIFNEILIRYTYRILARRIPACP-YV    1825
PCMoV           LLLAIWGRKCVAGNQVYQVNAGIPSGCALTVLLNSIFNEILVRYAFKIFVPK----V-HK    1806
AVA             LMMAIWGRRCIAGSQVYQVNAGIPSGCALTVLLNSIFNELLVRYAYKKFVPG----I-AR    1858
PBRSV           LLMSIFGRKCLARSQVFEVRGGIPSGCALTVLLNSVFNEILIRYVYKTVVPS----P-EF    1895
TRSV            LLMSIFGRKCLARSQVFEVRGGIRRG-ALTVLLNSVFNEILIRYVYKTVIPS----P-EF    1872
AeRSV           LLMSIFGRKCLARSQVFEVRGGIPSGCALTVLLNSVFNEILIRYVYKTVVPS----P-QY    1886
HoNV1           LIMACVGRYSICGNQVYQLFAGIPSGCALTVLYNGMFNEILIRYCFRTLVPA----P-ER    1884
HoNV2           LLLAISGRYSICGSQVYQVFAGIPSGCALTVLINSMLNEILIRYCYRVLVPK----V-CR    1427
MMLRaV          LLMALYCRKSIAQGDVFEVRCGIPSGCALTVLLNCIFNEILIRYCFAVLVPA----P-RK    1661
RpRSV           LLMGICNRISICGSQVYRVEAGMPSGFALTVDFNSIFNEILVRCAYRSLVPE----I-ER    1929
BRSV            MLMALVGRYAFVGPEVYKVNCGLPSGFALTVVVNSVFNEILIRYAYKKLAPK----P-ER    1815
PoLNVA          MLMALTGRYAFVGQQVYKVNCGLPSGFALTVVINSVFNEILIRYAHKKLAPQ----V-ER    1830
TBRV            MLMALVGRYAFVGQQVYKVNCGLPSGFALTVVVNSVFNEILIRYAYKKLAPA----P-ER    1815
AILV            MLMALVGRYAFVGQKVYKVNCGLPSGFALTVVVNSVFNEILIRYAYKKLAPT----P-ER    1829
RCNVA           MLMALVGRYAFVGQKVFKVNCGLPSGFALTVVVNSVFNEILIRYAYKKLAPK----P-ER    1805
GARSV           MIMALCGRYALVGADLYKVNCGLPSGFSLTVVVNSVFNEILIRYAYRKLAPA----P-QR    1792
GCMV            MIMALCGRYALVGTQVYKVNCGLPSGFALTVVMNSIFNEILIRYAYKTLAPT----P-EK    1797
PVB             MIMALHGRYAFLGQKIYKVNAGLPSGFALTVVMNSLFNEILIRYAFKVLAPK----P-QR    1821
CNSV            MINALFGRLAITGQEVMRVRAGLPSGFALTVVINSVFNEILMRYCFKVLVLG----P-QR    1871
ToRSV           LLMAMYGRWSVCGQRVFEVRAGMPSGCALTVIINSLFNEMLIRYVYRITVPR----P-LV    1860
AnNVA           LLMAMFGRWSICGQRVFEVRAGMPSGCALTVIINSLFNEILIRYVYRIVVPP----R-ER    1638
CLRV            LILAMYARYSLCGSQVYEVRAGLPSGCAITVIMNSIFNEILIRYAYRVSVGP----I-LR    1763
STeNV           LIVAMCGRFSICGSQVYELVAGMPSGCAMTVIINSIFNEILIRYVWKRSISG----I-PR    1731
BaNV1           ILWSLYHRFTIAGTQFYRISAGMPSGCALTVVINSIFNEILVRYVYRVSVPP----L-YR    1643
BLSV            LLLMFIGRRSICGGQVYMVNGGMPSGCAFTAMINSLFNEILIRYVFRKCTPA----P-MK    1826
PRMV            LLLMFIGRRSICGRQVYMVRGGMPSGCAFTALINSIFNEILIRYVYRKVTPA----P-AC    1821
CawYV           LMLMFIGRRSIAERQVYMVRGGMPSGCAFTALINSLFNEILIRYVFRKVVPR----P-SC    1865
SLSV            LFLMFTSRRSICYDQVYMVKGGMPSGCALTVIINSILNEILVRYVYRITVPM----P-AK    1841
PVU             LFLMFTSRRSICYDQVYMVTGGMPSGCALTVIINSVLNEILVRYVYRKVTPQ----P-AR    1591
BRV             LLMSIVNRKSICGARVYEVRAGIPSGCALTVLLNSIFNEFLIRFVWRTTIIG----V-PR    1752
GBLV            LLMAIVNRKSICGSQVYEVAAGIPSGCALTVLLNSIFNEMLIRYVWKISVGG----V-PR    1755
BLMoV           MLMAIVNRKSICGSQVYEVSAGIPSGCALTVLLNSIFNELLIRYVWKTTVVG----V-PR    1774
AYRSV           LLMAICNRKSLCGADAYEVRAGIPSGCALTVLLNSIFNEILVRMVYKTVVPG----V-PR    1313
GSPNeV          LIMALVNRKVIAGSQVYEVRAGLPSGLALTVTINSIFNELLVRMAFKQLAPP----V-YR    1798
GNVA            LLMGISNRLVICCGDVLEVRCGMPSGFALTVVVNSILNEILIRYVFRNTVAR----VRPS    1812
                 :     *  .          *:  * ::*.  * :::*:*:*
```

Pol GDD motif

```
ArMV        ECFDRCVVLITYGDDNVFTVSQS----------------IMT---------------SFT    1852
GFLV        ECFDRCVVLITYGDDNVFTVAQS----------------VMQ---------------YFT    1853
GDefV       ECFDRCVVLVTYGDDNVFTVAQS----------------VME---------------HFT    1853
MMMoV       DRFSKCVCLLVYGDDNLIACSQS----------------VIK---------------DFN    1874
HoNV3       GSFNKVVRFLVYGDDNLIAVAPS----------------VRN---------------FFF    1854
PCMoV       NEFTSLVCLLVYGDDNLIAVKDV----------------ISDIEVCRIESGVTIKERVGF    1850
AVA         EQFSTQVCLLVYGDDNLIAVKQG----------------ISQFTIKSD----GEDKVVSF    1898
PBRSV       NRFETFVTLAVYGDDNLIAVDPV----------------MAN---------------AFT    1924
TRSV        NRFETFVTLVVYGDDNLIAVDPS----------------MQK---------------IFT    1901
AeRSV       DRFESFVSLVTYGDDNLISVDAS----------------MAS---------------IFT    1915
HoNV1       NHFSKRVCLIVYGDDNLIAVNPI----------------IED---------------TFY    1913
HoNV2       SSFSRYVTLVTYGDDNLMAVSPA----------------IEG---------------TFY    1456
MMLRaV      SCFSQYVCLLVYGDDNLIAVAPS----------------IEN---------------FFN    1690
RpRSV       PFFSNNVVLIVYGDDNVLGIHPN----------------IES---------------AFN    1958
BRSV        NRFNQVVCLLVYGDDNLISVSPS----------------IAS---------------WFT    1844
PoLNVA      NRFGSTVCLLVYGDDNLISVAPS----------------CAK---------------WFT    1859
TBRV        NRFGSTVCLLVYGDDNLISVSPS----------------IAS---------------WFT    1844
AILV        NRFNSTVCLLVYGDDNLISVSPS----------------IAS---------------WFT    1858
RCNVA       CRFQQTVCLIVYGDDNLISVAPS----------------CAS---------------WFN    1834
GARSV       NLFGVNVCLLVYGDDNLISCAPS----------------ISS---------------WFT    1821
GCMV        NSFGINVCLLVYGDDNLISVSPA----------------VAS---------------WFT    1826
PVB         NFFGLHVTLLVYGDDNLISRTPT----------------V-E---------------WFT    1849
CNSV        NSFSTYVTLLVYGDDNLMSCTDK----------------IAI---------------YFN    1900
ToRSV       NNFKQEVCLIVYGDDNLISIKPD----------------TMK---------------YFN    1889
AnNVA       SRFNSNVCLIVYGDDNLVAIDRKLTTGIRILED--GQEVTRF---------------AFN    1681
CLRV        NRFSHFVKLIVYGDDNLIAVDPQLASGTFAGYEGGKM-VVTD---------------VFD    1807
STeNV       NQFHLHVALVVYGDDNLIAVKPEFYSGTLSGYDEKGMPLTIN---------------QFD    1776
BaNV1       NCFTTFVTLIVYGDDNLIAISPR----------------VEQ---------------YFN    1672
BLSV        NFFNSYVRLMVYGDDNLISIKEE----------------AIP---------------YFD    1855
PRMV        NFFNKYVRLMVYGDDNLLTIKEE----------------VIP---------------YFD    1850
CawYV       NYFNKYVRLMVYGDDNLITIKEE----------------IIP---------------YFD    1894
SLSV        NYFNKYVELVVYGDDNLIAIHDD----------------VVS---------------FFD    1870
PVU         NYFNKYVELVVYGDDNLIAIHDD----------------VVD---------------YFD    1620
BRV         ERFSQYVTLLIYGDDNLIAVHPD----------------YLP---------------HFN    1781
GBLV        EMFSTYVTLIVYGDDNLISVHPE----------------FLP---------------HFN    1784
BLMoV       EMFSSYVTLIVYGDDNLISVHPE----------------FLP---------------YFN    1803
AYRSV       NHFSEYVTLLVYGDDNLIAVDPS----------------ILE---------------VFN    1342
GSPNeV      NSFHAKIFLAVYGDDNIITVHPD-----------------CK---------------YFT    1826
GNVA        LRFRDNVELVVYGDDNLIAIRNE----------------LLP---------------IFN    1841
              *   : :  *****:.
```

```
ArMV            GDALKAEMA-NLGVTITDGKDKSLATIPARPLLELEFLKRGFK-KGN-----GGLIYAPL    1905
GFLV            GDALKMQMA-KLGVTITDGKDKSLSTIPARPLLELEFLKRGFV-RSS-----GGMINAPL    1906
GDefV           GDALKRQMA-KIGVTITDGKDKSLSTIPARPLLELEFLKRGFK-RSP-----GGHVGAPL    1906
MMMoV           GNALKDWLA-QYKVTITDGKDKTAPTIEERPLLELDFLKRGFK-LAV-----GGRVLAPL    1927
HoNV3           GNAIKSEMA-SFGVVITDGTNKDSLVLEDKPIESLDFLKRTFK-SIH-----GGHIVAPL    1907
PCMoV           GKLLKETLA-EVGVTITDGTDKLSATLEAKPLGSLDFLKRGFK-K-V-----DGYILAPL    1902
AVA             GKLLKDTLA-GVGVTITDGTDKLSPTLEPKPLESLDFLKRGFK-K-K-----DGYILAPL    1950
PBRSV           GEVIKRTLA-RKGITITDGSDKLSPTLEAKPLSQLDFLKRSFL-VSS-----SGQVMPAL    1977
TRSV            GEVIKKTLA-RKKITITDGSDKLSPVLEAKPLAQLDFLKRSFL-VSD-----SGQVMPAL    1954
AeRSV           GEVIKKTLA-RKGVTITDGSDKLSPTLEAKPIAQLDFLKRSFL-VE------GGQVFPAL    1967
HoNV1           GEAFKAKLA-EYGVTITDGSDKTSDRLEAKNLTSLDFLKRGFV-EHR-----GGYVTAPL    1966
HoNV2           GEAVRLEMA-KYRITITDGTDKDAKGLYKKPIHQADFLKRGFR-QVA-----GGQMAAPL    1509
MMLRaV          GNEIKRVLA-ELNVNITDGICKQSPTIEMRQLEDLNFLKRGFK-VTV-----GDRVRAPL    1743
RpRSV           GNAIKAYMKEELGIKITDGADKLSPVICARPLEQCEFLKRTWR-KDRQ----YGLYRAPL    2013
BRSV            GEAIRITLA-EKKVKITDGSDKDAPTIEAKSFWELDFLKRKFL-KLD-----NGIVQAPL    1897
PoLNVA          GEAIRIVLA-EKKVKITDGSDKDAPTIEAKSFWELDFLKRKFL-KLD-----NGIVQAPL    1912
TBRV            GEAIRITLA-EKKVKITDGSDKDAPTIEAKSFWELDFLKRKFL-KLD-----NGIVQAPL    1897
AILV            GEAIRITLK-EKNVKITDGSDKDAPTIEAKSFWELDFLKRKFL-KLD-----NGIVQAPL    1911
RCNVA           GEAIRVTLA-EKKVKITDGSDKDAPTIESKPFWELDFLKRKFL-KLD-----TGVIQAPL    1887
GARSV           GEAIRICLA-EKKIKITDGSDKDAPTIEPKPFGELDFLKRKFL-KLD-----NGIVQAPL    1874
GCMV            GEAIRVTLA-EKRIKITDGSDKDAPTIEAKPFSELDFLKRKFYVHPE-----HGQVWAPL    1880
PVB             GEAIRCTLA-KYKVKITDGSDKLALTIEEKPLSELDFLKRKFL-KTN-----TGVIQAPL    1902
CNSV            GETIKETLK-KKNVTITDGSDKTAPDIKWKTLGELDFLKRRFL-KLE-----TGVVQAPL    1953
ToRSV           GEQIKTILA-KYKVTITDGSDKNSPVLRAKPLKQLDFLKRGFR-VES-----DGRVLAPL    1942
AnNVA           GEVIKSTLA-NFGITITDGSDKNCPVLVSKPLTSLDFLKRGFS-LQP-----SGRVLAPL    1734
CLRV            GATIQKVLA-QVNITITDGSDKNAKEWHFKSLESLDFLKRGFK-RMA-----DGRVLAPL    1860
STeNV           GPTIQKELA-ALKVKITDGSDKLAAEFHQKPLGSLDFLKRGFK-RMG-----DGRVLAPL    1829
BaNV1           GGIIKTTMA-QFGMTITDGSDKLATSLQAKPLLDLDFLKRGFR-REV-----DGRVWAPL    1725
BLSV            GPIIKNELA-QVGVIITDGTDKTSPTLQRKPLESLDFLKRGFR-RLS-----TGIVIAPL    1908
PRMV            GPVIKKEMA-SVGIIITDGTDKSSLTLERKPLSSLEFLKRGFR-VQE-----NGLVVAPL    1903
CawYV           GPVIKEEMA-ELGITITDGTDKSSPTLQRKPLKSLEFLKRGFR-KQP-----SGVIVAPL    1947
SLSV            GPIIKEKLA-EVGVTITDGTDKSSPTLLRKPLESLDFLKRGFR-KIE-----GGMYSCPL    1923
PVU             GPIIKEEMA-KVGVTITDGTDKLSPTLVRKPLESLDFLKRGFR-KGA-----GGLYSAPL    1673
BRV             GEIIRTRLA-DVNVIITDGSDKTAEKIEEKPLVQLDFLKRRFR-KLN-----DGTVYAPL    1834
GBLV            GMVIQAKLK-EVGVTITDGSDKTAEGIYEKPFEKLDFLKRRFA-KQS-----DGTVLAPL    1837
BLMoV           GMVIQKKLK-DVGVTITDGSDKTAEGIYEKPFEKLDFLKRRFA-KQS-----DGTVLAPL    1856
AYRSV           GDVIKKTMA-KWRVTITDGSDKLSPTLTEKPLLSLDFLKRGFK-LAD-----NGQVYAPL    1395
GSPNeV          GTAMKKVMN-EWGVTITDGSDKTSLVLKPKPLSEIDFLKRSFS-LQKKDQNDGLGIVAPL    1884
GNVA            GRVIKSELA-KVHITITDGSDKMALDLREKPLSELDFLKRKFV-LLD-----NDRVLAPL    1894
                *  .:  :     : ****  *       : : . :**** :                 *
```

```
ArMV        EKLSIMSSLVYIRS---------------DGSDM--LQKLVDNVNTALVELYLHQ-DREY    1947
GFLV        EKLSIMSSLVYIRS---------------DGSDM--LQKLLDNVNTALVELYLHG-DRTY    1948
GDefV       EKLSIMSSLIYIRS---------------DGSDL--LQKLLDNVNTALVELYLHA-DREY    1948
MMMoV       DKKSIYSSLVHVRA---------------KDLDW--VPLLFDNYQNCLRELVMHD-DREE    1969
HoNV3       DKKAIYSQLDYVRS---------------KDGNT--LEVLFPNIQNSLREMFLHQ-DREA    1949
PCMoV       DKNAIYSSLVWVAA---------------RDQDV--LEKLRLNVSVALQELWLHQ-DKQE    1944
AVA         DKNAIYSSLVWVAS---------------RDEDV--MEKLRLNVNVALQEIWLWQ-DVQE    1992
PBRSV       DRTCIYSSLLYLSS---------------KGADP--IPLLHQNVQNALQEMYHRQ-DRAE    2019
TRSV        DRTCIYSSLLYLRS---------------ADCDP--IPLLHQNVQNALQELYYRQ-DREE    1996
AeRSV       DLSCIFSSLKHVRA---------------EGADV--IPILHQNVQNALQELYYRR-DKEQ    2009
HoNV1       DPLALRTQL---------------------------------------------------    1975
HoNV2       DKLAIYSSLHYVKPL--------------NGNDV--IEALKVNVVAALREIVLHP-GRQD    1552
MMLRaV      ELNSLYSSLIWVAS---------------RGEDV--FDKLFLNVQVVLRELWHHD-DRDL    1785
RpRSV       VETSIYSCLRYVRL---------------QNYDW--QAPLLQNVQGSLYEASLHG--PDM    2054
BRSV        DRSAIFSSLYWLTPDKSKFHASQRASDFQGTVDV--VEELILNVNVALMELYLHN-DPRE    1954
PoLNVA      DRAAIFSSLYWLTPDKNKFHESQKASDYQGEVDV--IEELVLNVNVALMELYLHN-DQIE    1969
TBRV        DRSAIFSSLYWLTPDKSKFHESQKPSDFQGEVDV--IEELLLNVNVALMELYLHN-DVAE    1954
AILV        DRSAIFSSLYWLTPDKSKFHESQKASDYQGEVDV--VEELILNVNVALMELYLHN-DPAE    1968
RCNVA       DMTAIFSSLYWLTPSKDKFHTHQKASQYSGEVDV--IEELVLNVNVALMELYLHN-DPKE    1944
GARSV       DRSAIFSSLHWLTPEKSKFHESQKPIDYLGDVDV--VKELILNVNVALVELYLHN-DRTE    1931
GCMV        DKSAIFSCLHWLTPQKSKFALQEKACDYLGEVDV--VEELIINVNVSLVELYLHN-DKEE    1937
PVB         DKSAIYSCLHWLVPSKGKKVD-------GGPLDV--VEELILNVNVSLMELYLHD-DRDE    1952
CNSV        DLTAIFSCLHWVTPHPQKMPKGGAQL-QVENVDT--LYELALNVQVALTELYLHG-NKEE    2009
ToRSV       DLQAIYSSLYYINP----------------QGNI--LKSLFLNAQVALRELYLHG-DVEQ    1983
AnNVA       DLQSIFSSLHYINF----------------QGDM--LHSLFLNVNVALHELWLHQ-DVER    1775
CLRV        DLSAIFSSLHVVRP---------------DQGST--AAAVNINARVALRELWLHQ-DQNL    1902
STeNV       DKSAIFSSLHVVVP---------------EQGST--IMAVWKNAAVALRELYLHQ-DEQL    1871
BaNV1       DRSAIYSCLNYVRA---------------VDAEE--CDAVLVNARVALLELYAHG-NVKE    1767
BLSV        DKTSLYTRLYYSTA---------------GDDGYCNLDVLRANVKSFLEEIVLHPNHVNE    1953
PRMV        DKTSMYTRLFYSTA---------------GIDGVYSLDILRDNVKSFLEEIVLHPNHHRE    1948
CawYV       DKTSMYTRLYYSTA---------------GIDGVYSLDILRDNVKSFLEEIVLHPNHTRE    1992
SLSV        DKGSLYSRLYYSAG---------------GKGGLFQLDILHDNVKSFLEELTHHADHYEE    1968
PVU         DKTSLYSRLFYTTG---------------GADGKYQNDILHDNIKSFLEEITLHG-EYSE    1717
BRV         DLASVYTSLQNVTM---------------GAGSI--HIALQNNVHNALLELYLHG-NETW    1876
GBLV        DLASIFTSLQNVTL---------------GAGSI--PEAVRINVHVALTELYLHQ-KREW    1879
BLMoV       DVASIFTSLQNVTL---------------GAGSI--PAAVKQNVHSALIELYLHQ-RPEW    1898
AYRSV       DKTAIYSSLHWVAG---------------RGQDV--MSALKDNARSALIEMWAHQ-DKAE    1437
GSPNeV      DNDAIYSCLHYYKP---------------VGSEI---TTLVDSVRAALFELRLHK-DRPK    1925
GNVA        DLNSILSSLVWVHA---------------KFRDV--HSALFDNVQNALLELSLHP--KEV    1935
               .: : *
```

```
ArMV      SESVRDFYLEKLPPGSYKE---L-TTWYEAQIFHECQLSGESGWKPQGLIEVSH-GAS-F   2001
GFLV      FESVRAFYFEKLPPGAYKE---L-TTWFQAESFHECQKSGESGYKPQGLIEISH-GAA-F   2002
GDefV     FDSVRDFYLEKLPPGSYKE---L-TTWFQAETFHECQRSGESGYKPQGLIEISH-GAA-F   2002
MMMoV     FEALRKYYETLFPS--WKG---SSLTWNEVQSWHTAQLTGNSGLSYNDRMDVLI-NPQ-F   2022
HoNV3     FEYLREFYYERIPQWKERD---LLLTWTEAESFHLGQLSGVAPWRPAKHLDFSI-RPT-L   2004
PCMoV     FNAVRDFFVRGIPA--WKD---V-PDWKRIEGFHMEQLSHVKPWSPAKNVDIVV-RPE-M   1996
AVA       FNKLRNFYVSAIPS--WSG---L-PTWNAIKAFHVEQLTHTKPWQPAKNIDILV-RPE-M   2044
PBRSV     FDTLRNFYLERCPT--WRSGPNRLLDYNQCHAHWISRYTGQPNSNPAGVVDMLV-DPR-H   2075
TRSV      FDNLRTFYLERLPM--WRNGQHRLLDWNQCAEHWRARYTGCPSDNPAGVLDMLV-DPR-C   2052
AeRSV     FDYVRTFYLERIPV--WGTGKNRLIDWSYAHRHWISRYTGDPSANPAGVVDILV-DPR-Y   2065
HoNV1     ------------------------------------------------------------   1975
HoNV2     YDNIVTHYKTVAPL--WKL-----PSYQSCLDFHEEQRTGTCPFNVQRFSDIMI-DST-Q   1603
MMLRaV    FDKLRGFYVSEVPS--WGS---KLLTWRQVEDFHHQQLIGMPRITAAQDLDLLI-RPE-V   1838
RpRSV     HARIYKHFATHFPK--WVE-EHELYTYEQCRTRFIAAKNGDFNFHPASAQMGHVFSQQTE   2111
BRSV      FSRVRDFYIKALPL--ATG---QFRTWAFCEAFHSAQQTGMLKYDPAKVLDHMS-GLD-F   2007
PoLNVA    FNRVRNFYVRSLPL--MVE---QFRTWAFCESFHSAQQTGMLKYDPARVMDHMT-GVD-F   2022
TBRV      FQRVRGFYAQRLPL--MVS---QLRTWAFCEAFHSAQQTGMQKYDPAVVLDHMS-GVD-F   2007
AILV      FQRVRGFYVKTLPL--LVS---QLRTWSFCEAFHSQQQTGMLKYDPAVILDHMS-GAD-F   2021
RCNVA     FQRVRSFFVARLPL--LVD---QFRTWAFCESFHSSQQTGMLRHDPASVLDHMS-GVD-F   1997
GARSV     FDRVRGFYVKKLPT--CVD---QFRTWAFCESFHSAQQTGMLRHDPAKVLDYMV-GPD-F   1984
GCMV      FNRVRSFYIARLPM--QVD---QFRTWAFCEAFHSAQQTGMLRHDPAKILDSLA-GPE-F   1990
PVB       FNSVRQFYLKRLPM--QAD---LFRTWAMCESFHSSQQTGFLKYDPAKVLDIHI-APG-M   2005
CNSV      FQRVRNFYTKKMNI--LPA---GYYTWADREAFHMSKQTGMEAYQPAKEIDLDV-GQE-F   2062
ToRSV     FTAVRNFYVNQIGG--NFL---SLPQWRHCASFHDEQYSQWKPWSPVKFLEVDV-PD--A   2035
AnNVA     YNFVRNFFVSKCGG--QFL---KLPSWRVAGLFHDDQYANTNSFSLVKLYTQDI-PD--N   1827
CLRV      FEMVREFYKR---H--NFV---DLPTWRECRDFHQSQYSEWQPFKEYKFLELPL-PEQEN   1953
STeNV     FHYVRNFYLEK-GE--MWA---QLPTWRECRDFHQHQYSGWQPWAPHKFIEIPL-PST-N   1923
BaNV1     FESVRQHYLTRIPA--WSK---CLPSWRAADEFHFKQYYQVTPWRPTQILEVSA-QRD-D   1820
BLSV      FFRVRSFFLKKIPA--WAD---FLPTYAAAQDFHYRQQQTQSPYLVPRILETRP-MGT-E   2006
PRMV      FFRVRNFYVSKVPH--WGD---ILPTYGAAIDFHYRQQTTNTPYQTQRIFETRP-HGG-E   2001
CawYV     FNRVRDFYLNKCPH--WAN---YLPTYNAAIDFHYRQQTTMSPYQTQRIFETRP-NGG-E   2045
SLSV      FQRVRSFYIKRIPS--WSA---TLPTYATCLNMMEGQRNSATPWQPHKFIETRP-SGC-E   2021
PVU       FQRVRNFYVARVPS--WSA---TLPSWSHCIDFMEGQLNAATPWQPHKFIETRP-HGG-E   1770
BRV       FNHLRDFYRK-SHA--WVN----LPSWREAFAFHQGQISGVTPWTPYQMFDVPV-DGGRL   1928
GBLV      YDDLRNHYQK-TQG--WEN----LPTWAQSHAFHREHLTGALPWAPHRVMDIPV-DKKKL   1931
BLMoV     FDDLRSFYVR-GQG--WTD----LFTWQQARAFHREHMTGVLPWAPHRMLDIPV-EKTKL   1950
AYRSV     FVELRSFYVSKIPS--WSD----LPTWDQARCFHEAQQHGAMPYRPQASMELLV-DMHTD   1490
GSPNeV    FESLRNFYLERRPY--LKE-EHVLPDWDEATALHMELKGKGTPYKPYQVLEFRL-DIPKL   1981
GNVA      FNEVRSFYVERLPL--WAP---KLRTYSQCQAMMEEFFYSTRPWCPIEVLDQLV-VPE-F   1988
```

```
ArMV       ASFVQQNGTELERHDICPGLAISGSKYIAREEEI-L--MSLSS--LLPGD--IN--AVKL   2052
GFLV       ASFTQQAGTELEKHDICPGLSIAGTKYIATENEI-V--LSLSS--VLPGD--RN--VFKL   2053
GDefV      ASFTQQAGTELEKHDICPGLSIAGAKYVANENEI-V--LTLSS--KLPGD--TN--TFKL   2053
MMMoV      STFMQQHGPADVINSVDNNFSIAGPKWCDRGGDY-F-VVSTFP--LFRGE--VG--I-HV   2073
HoNV3      RAFCISQGDSNCYEAITPNFAICGPKYKIPSGTD-VLLVSFTR--LSATE--EAARHLYV   2059
PCMoV      RAFMDCHGYADEKFVVCPKIFVAGPKYRFSSDEF-G--ISFTQ--LLVGE--SS--L-NC   2046
AVA        FSFIDCHGYADEKFVVCEGIFVAGPKYRFEPGEF-G--VSLTN--LLKGE--DS--L-NC   2094
PBRSV      KSFLLPAGPANWSMPVADGIFVCGPKFFPNAPS---FTICF--NRLAAGE--TG--I-EI   2125
TRSV       KSFMLPAGPANWSMPIADRIFVCGPKFSASGPS---YTLCF--NRLAAGE--TG--V-QI   2102
AeRSV      KSFLLPAGPADWCMPIADRFFVCGPKFYPQGHS---FTVCF--NRLAAGE--NG--V-QI   2115
HoNV1      ------------------------------------------------------------   1975
HoNV2      AGLY-YHTRPDTKIPIMRRVSIAAAKYRATDDEC-I--VSLTG--RLVGE--GI--E-VL   1652
MMLRaV     KAFCTSEGASDMTFSLAPGIKIAGCKFVQKRPED--IIVSFSPRQPCERED-PN--V-LT   1892
RpRSV      IQELSQSQNPKRCFQLHPKIHICGPGHNEQDC----FYVDVRVKGKITKG--KG--F-HH   2162
BRSV       KKFMHVSEQGNKAHFYTEMLGVAGPHYKPQENDF-I--VSTEP--LKMGV--CG--E-HV   2057
PoLNVA     KKFMHVSEQGNKAHFYTSVLGVAGPHYKPLENDF-V--VSTEK--LKPGM--SG--E-HV   2072
TBRV       KRFMHMSEQGNKAHFYTEMLGVSGPHYKPQEGDF-I--VSNQP--LKPGV--QG--E-YV   2057
AILV       KKFMHVSEQGNKAHFYTDILGVSGPHYKPQENDF-V--VSTLP--LKMGV--AG--E-HV   2071
RCNVA      KKFMHVSEQGNKAHFYTSVLGVCGPHYKPCEEDF-I--VSTEP--LKMGI--LG--E-HV   2047
GARSV      ARFMRVSEQGNKAHFYCRDLGVCGPHYKPKDDEF-L--VSTVP--LN-GR--SG--E-YI   2033
GCMV       PRFMRCSGEGDKAHFYTPILGVCGPHYKPKEQDF-L--VSTLP--IKQG---EG--V-HI   2039
PVB        SKFLHCSGEGKACHRYTPTLAVCGPHYHCNKDEF-C--VSTIP--LKPGE--DG--C-VI   2055
CNSV       ARFMHTSDIGNQVHFTRQCLVVAGPFYKPTPDQL-L--VSTTP--LKQGE--SG--Y-WV   2112
ToRSV      K-FLQHKAPATALSIVADRLAVAGPGWRNKDPDR-YLLVSLTS--LK-ANE-GG--L-YF   2086
AnNVA      TLFFENHAPKQVLSVVERRIYVAGAGWNNPDPDT-YLVVSLNA--RK-GNE-DC--I-HW   1879
CLRV       REFMENHATRKSVCVVADQTIVVGPAWKPSNPEG-FFVVDLLG--PTCGRG-GN--V-TN   2006
STeNV      LQFMKNQGGKTTMCIVADQVCVVGEGWKNPDPSM-YFVVDLVH--KSTRDE-HT--L-CV   1976
BaNV1      TKFMESHISKDYTLIIEDRVVLAAEGWRAPSTRG-YYITSSSP--CFIGEE-T-------   1869
BLSV       YKMMAGQDNHDNIVWLTSRVAICGPKYPVPSGPL-HFVVALSS--FVRTCE-RG--V-NY   2059
PRMV       HKMMAGQDCRDQTIWITNRLAIIGRNTSIPKGSQ-HFVVACGS--SLRGGE-RG--V-TI   2054
CawYV      YKMMAGQDKEQGIIHVTDRLAIIGMKTEIPTDRP-GFVVALDA--GVRACE-RG--V-VY   2098
SLSV       TKMMAGQDNHAQKICVTERIIICGEKYTPPILET-SFVVSIDN--PLYIGE-RG--E-YV   2074
PVU        EKMMAGQDKHDQRIVVTPRLIVCGEKYRPPINEA-SFIVSIDN--PLYPNE-RG--I-SV   1823
BRV        RAMMANQGEAAFSTHLGREIYICGPKWCVSDPEH-QFVVSTTPLRSADRG--SG--I-HR   1982
GBLV       TQAMQSQGGLDFVVQVAERIFVCGPAFRPDATEQGSFVVSHT-GSLPRGL--QG--C-LS   1985
BLMoV      TRAMANQGEADYKCQVAERIFVCGPKSNL-SQEPAHFVVSHT-GSLKRGE--TG--I-VA   2003
AYRSV      KKMMANHGEQDHLIYVQPRIAVSGPKWIIESCDK-QFVVSTLPLSRAEKF--SG--I-HI   1544
GSPNeV     KTDLTQHSGANWCHAVERRISLAGNNYYPADSSD-LFFISLGCNYP---KGVSG--I-RV   2034
GNVA       KRAMFNVGAPDIILPVHRGFYIAGPRWVSPSAQD-YLVVDLGHERPHRPLDLGD--I-HY   2044
```

```
ArMV     TLK-CGDGIGRLPSKASVLSQRKPGIV--------M-QLCARAIKEKKTLVIRDERPYIG   2102
GFLV     DLP-CGDGIGRLPSKCSILNLRKPGLV--------M-RLCKRAQDEKKTLVIRDERPYIG   2103
GDefV    DLP-CGDGIGRLPSKNSILALRKPGLV--------R-NLCCLAQSGKKTLVIRDERPYIG   2103
MMMoV    PIV-PGGGIGCMPTNNWVRNWASGSCE-------YS-QKISEAISDKKHVVFRDNQPYIG   2124
HoNV3    PID-VGEGVGGLPTVQWVKKWRSANFP-------PH-QAILHALKLKKSIVFRDQQPGLV   2110
PCMoV    VYD---RADFEVPTQLWVDRWGTPKNI-------HV-ARARAAYDSGKSLVFRGHAPYII   2095
AVA      VFV---KQESDLPSATWVQSFGSPLNP-------CV-ARMRAAHSAGKKLVFRGHAPYMA   2143
PBRSV    KPV-HAATQGAMPTGKFVKSFRSMKKR-------PELELALAAKDAGSAIYFKGCAPYND   2177
TRSV     KPV-HAATQGAMPTAKFVESFRSIKKR-------PELELAISAYESGSNLYFKGCAPYND   2154
AeRSV    KPV-HAATQGAMPTSKFVDSFRSVKRR-------EELNLVLSAYDTGANIYFKGCAPYND   2167
HoNV1    ------------------------------------------------------------   1975
HoNV2    ESK-PEMGSGNLPTLKWIKQWRSLNNL-------TF-SRAAAAYDAGKEIVFRDHSPFIN   1703
MMLRaV   ETVVYGAGVGGLPTRMWGRKFRSEKKW-------PEFARCMATLRDGGNVYFRDVQPLAG   1945
RpRSV    APV-FSAGSGQLGTVKWASSFRSSSAC-------PMRDLAVDAFKRGECVYFRDNGELIN   2214
BRSV     PIQ-YGSGVGGLPTKKWVLDFGRPSQLKNKLGYLIH-PILRAQIEAGKRLVFMSPAPYVA   2115
PoLNVA   PIQ-YGAGIGRLPTKRWVGDFGRPTALKNQNGFLIY-KLLREQIESGKRLIFMSPAPYVA   2130
TBRV     PIV-FGEGIGGLPTKKWVGDFGKPSQLKNSKGYLIT-GLLREQIEAGKRLIFMGPAPYVA   2115
AILV     PVQ-FGTGIGGLPTKKWVGDFGRPSRLKNAKGYLIY-NLLREQVEAGKRLIFMGPAPYVA   2129
RCNVA    PIK-YGGGIGRLPTKNWVMSFGKSSSLKNAKGYLIY-PLLREQMEAGKRIVFMSPAPYVA   2105
GARSV    PVK-FGDGVGGLPTKKWVTDFGRSSQLKNAGGFLCR-DLLVQVLESGKKLIFMSPAPYVA   2091
GCMV     PIK-IGGGVGGLPTHQWVKNFGRPSQLKNNDGYACY-KLLCEQIEQGKRLVFMSAAPYVA   2097
PVB      PVT-SGAGVGGLPTKSWVNAFRSPRKLKNQEGYQIY-SFMLGAIEAGKTLIFKSPAPYVA   2113
CNSV     PVE-TGMGIGNLPTIAWVHRFMRPTQLVDAYGYKIW-GNVRSHIESGKSLVFRSEAPYVA   2170
ToRSV    PVD-YGEGTGQQATEASIRAYRRLKDH-------RV-RHMRDSWNEGKTIVFRCEGPFVS   2137
AnNVA    PAD-FGEGAGQMATRTSIKAYGKKSAN-------T--VRMKQFWQDGGSIVFRCEAPFIS   1929
CLRV     IPT-YGDGSGRLGTATWVRNWRSAKKQ-------PIVQLAIQARKEGKIIAFRDAAPFLN   2058
STeNV    SPV-CGEGAGRLPTEAWVASVRSPKNG-------WF-TTVSSLRKQGKIICFRDMAPYIT   2027
BaNV1    ------------------------------------------------------------   1869
BLSV     KVD-IAEGSGQLPTQSWTDSFSSSKKR-------TA---LHTAYGDGATIYFRSVMPYYS   2108
PRMV     KVE-TAPGMGCLPTQAWVDSFSSPKKR-------PS---LFEAYNEGCTLYFRSEMPYYS   2103
CawYV    PVE-TSDGNGRLPTQRWVDSFSSFKKR-------AL---LHEAYGEGCTIYFRSPMPRYL   2147
SLSV     KTE-FGSGMGRLPTDAWIKAFSSPKKH-------PQ---LFEAYKMGATIYFRSCMPYLN   2123
PVU      RTE-VDRGSGHLPMDRWSKDFSSIKRY-------PT---LHQAYEDGATIYFKSRMPYFW   1872
BRV      AIEYPCNGVGRLPSQDWVTKFKSSAHR-------VT-AEIRKAHASGKAIYFRDDPPYVA   2034
GBLV     PVDFVSEGQGRLPTQLWVNKFRSERHH-------LT-CLIRDAYNRGCNVYFRAEQPYIV   2037
BLMoV    PVDFVSEGQGRLPTQMWVKKFRSESHT-------FR-VMINDAYTRGHSIYFRSDPPYIT   2055
AYRSV    PVEC-GDGLGRMPSQDWVRRFRRIRHV-------QI-RVIYDAYAAGKTIIFKDVAPYIA   1595
GSPNeV   DYAHSHEGAGQQPTRMWMKRFRSSSNA-------ET-QKMREAYDAGKHLVFMSKGPFTA   2086
GNVA     GLE-LAPGVGKIPTKRWAQSFKSGKNP-------VI-VRVREALDLDQKIVFRGAHPYID   2095
```

```
ArMV      GWAMACICGES--FGFSIKDTLALYANLMGPNRKN----GLATYFTDFDS---PVHVKKI    2153
GFLV      AWAVACICGES--FGFGQQSVLALYANLLGPNQRN----GLASYFSDFES---PIHIKKV    2154
GDefV     AWAVACICGES--FGFGTQSVLALYANLLGPNRRN----GLASYFSDFDS---PVHIKRV    2154
MMMoV     AWTALISFCSG--FGFRTVEESMILYRNICPKDPI----HLSTFFSWCNK---RTPAGHL    2175
HoNV3     AWTALISSAIG--HGLEQTDQLLALVNNVIKNPG-----ALNHYFQDITY---SRPYGPF    2160
PCMoV     CWVAMMRFCIS--ANVCDKDTLQAMFYNIGGKKSV----DIAPYFSKFDM---SKRMGFK    2146
AVA       CWLAMMKFCIS--AGICDQDSLLALFYNLKGNKQT----DLSSYFSKFDR---EKRMGHR    2194
PBRSV     IWACAIAFCSA--FGFADKQVLLAVHDNSKPIGAS----SLRSYFNGNLA---GDGCA-R    2227
TRSV      IWACAISFCSA--FGYAQKQVLLHMYDNCKRLGAS----SLRSYFNKSLV---GDGCA-R    2204
AeRSV     IWACAIAFCSA--FGIAQKEILLALHDNSKPIGAS----SLRSYFNHKIV---GDGCA-R    2217
HoNV1     ------------------------------------------------------------    1975
HoNV2     AWCAAIAFCVYRPKPICDIHTMRLVYQNVAGSSKQSAESAIFSMFGGFDR---TKPYGDC    1760
MMLRaV    AWIAAIYFADK--TNRCDLETGKLLCGNLAGKQGH----QICKWFDTELH---GDVTW-K    1995
RpRSV     AWLAAINFGMS--INADGLDGLLQVYRNQGPTHLD----DLSFYFEGGVV---GVPAP--    2263
BRSV      NNAALIAFGTG--GKMLIQKDALVHYRNVIPESTS----GLEQYFDA------PLPTATI    2163
PoLNVA    NNAALIAFGSA--SKLLVQKDALAHYRNCIPESTC----GLEQYFDA------PVPIAAV    2178
TBRV      NNAALISFGSA--HKMLIQKDALAHYRNVIPESTS----GLEQYFDA------PIPQASV    2163
AILV      NNAALISFGTA--AKMLVQKDALVYYRNVIPEATS----GLEQYFDA------PLPTATV    2177
RCNVA     NNAALIAFGSA--TKLLNQKDALVHYRNCIPESTT----GLEQYFDA------PIPQANI    2153
GARSV     GNAAAICFGAS--VKMLNEKDALAHYRNSIPESVD----GLERYFDA------PIPAAAV    2139
GCMV      GNAALISFGSS--RKILKEQMPLCHYRNSIPESVD----GLTGYFDA------PLPAATI    2145
PVB       GNAALIAFADG--AKLARQQDLLYHYRNSIPENMN----GLEQYFDA------PLPSATI    2161
CNSV      GNAALMAFGQA--AKLLEIKTALNLYRNVIPESTY----GLEQYFDA------AIPQASL    2218
ToRSV     GWAAAISFGTS--VGMNAQDLLINYGIQ-GGAHKE----YLGRYFVGARF---KELE---    2184
AnNVA     GWAAAITFANA--LGFQVELMEQSYILQ-GGRHSQ----FLHHYFANEQC---SLLQ---    1976
CLRV      GWNAAIAFCEG--LGMDSNDLIAVYSRS-GGLHRS----LIERNFKAASF---EPRK---    2105
STeNV     GWAVALSFCIG--NGMNIKDILPMYKLS-GGQHPG----MVENYFKNKVY---IELS---    2074
BaNV1     ------------------------------------------------------------    1869
BLSV      SWCAAGRFLKS--QGISTSSVIALFEQY-KPANAG----NIAPLLATKEY---RRYSH--    2156
PRMV      SWCALGRFGKS--QGLSTSSIIALFEEY-KPTKGG----DIAPLLAERTY---KKFAH--    2151
CawYV     SWCALGGFARS--LGIDRSSIIALFEEY-KPRNAG----DIAPLLASKEY---KRHVS--    2195
SLSV      AWCAVCKFALA--HGIDQSSIIAMYESC-KPRGAG----DIAPLVAVKAY---TKFVN--    2171
PVU       SWCALCKFAQS--KGINQSSVIALYEKY-KPSNAG----DIAPLVADKAY---TKYVA--    1920
BRV       NWCAAIGFAQG--LGYDYKSMINLYHDVSVP-GSD----ALYFYFEQRAR---R------    2078
GBLV      NWLSATSFAMG--LGKDYRAILHLYHNVCTP-NAQ----CLDPYFEARRF---Q------    2081
BLMoV     NWLSATSFALG--KGMDYKAILGLYHNVCTP-DAQ----CLDEYFVSARF---R------    2099
AYRSV     GWTAAISFACA--QQYSYRDMLNRYNNVCTP-NSS----GIEQYFAKDMA---M------    1639
GSPNeV    SWLALISFCDA--YNIAKKDQLLELFRRVKTPGCS----DLSYYFEEAKPRYVGIGEW--    2138
GNVA      AWCALTGVLLQ--LQLCDLSQLEHKYTNLMPGSRP----VLSEFFPCGVH---TAGHHYT    2146
```

```
ArMV      HAITNGE-----EGVAMLKDSFAF----CEPTTIAATSCD------------TRKEMVSH   2192
GFLV      HAKTNSY-----EGGEALKEIFTF----CETIFYEATEMD------------TRKVMLQN   2193
GDefV     HAKTNTK-----EGSEALKEIFPF----CEVELYDATNAD------------VGREVICN   2193
MMMoV     SYTGPALVSVEEPFVKYLQDEFRLVP--FDEVVYPHICVT--------------------   2213
HoNV3     SYKGSVACSTSMEVKEVIDEVFKS-----------HVREQIDENLSVHE-----------   2198
PCMoV     KMPTDIKCVSLPHHMLMVSNYFRTAELLPGVIRYPKEFC------GVKEWTLKVSKEIRK   2200
AVA       ILPRPIHFATTNSKKLEQS-----DSIFHGVVQVDISCRELDDQPSVKLVIGDKALKLKE   2249
PBRSV     RLEVHSKAGQFAAVQ---------------RLAPTMQCKQIVYDP---------------   2257
TRSV      RCEIHTTPAIAKQVE---------------RLLPQVQCKHCEYDP---------------   2234
AeRSV     RLEIYSTKPRADIVK---------------RVLPQVQCVHIDYEP---------------   2247
HoNV1     ------------------------------------------------------------   1975
HoNV2     VPNKPMDVDDVSL---------------FDALQKQQINCV--------------------   1785
MMLRaV    VRGDPSAFGK---TR---------------MMQLGWQTENVGPIP---------------   2022
RpRSV     AHLMVYGTDT-SILN---------------RLCPKTV-LESAPPP---------------   2291
BRSV      ---GTFYF--------------------ANGETYAALCEY-KE-----------GKVLNY   2188
PoLNVA    ---NTFHF--------------------ANGETYAALCEF-KE-----------GVVMQY   2203
TBRV      ---GTFYF--------------------GDGETYTALCEY-KD-----------GKVLQY   2188
AILV      ---GTFYF--------------------ANGETYAALCEF-KD-----------GKVLNY   2202
RCNVA     ---GQFYF--------------------VDGETYAALNDF-KE-----------AKVLGY   2178
GARSV     ---GKCYF--------------------ANGETYAALCQF-KE-----------AEVLNI   2164
GCMV      ---GKSYF--------------------ANGETYAALCEF-KN-----------GEVLDI   2170
PVB       ---GKFWF--------------------SNAETYANLCHR-KE-----------GEVADI   2186
CNSV      --PGTFYL--------------------ANAESESLLQEH-KT-----------GTVIGL   2244
ToRSV     RYDRPFQSRIIAS-----------------------------------------------   2197
AnNVA     LTKQPYTRVGAATVL---------------A-----------------------------   1992
CLRV      VAPGVFA-----------------------------------------------------   2112
STeNV     TTIDPWTRRSKG------------------------------------------------   2086
BaNV1     ------------------------------------------------------------   1869
BLSV      RPI--FDLSS--IKQ---------------HLAAS-------------------------   2172
PRMV      RPM--FDFSG--IKQ---------------RMSAS-------------------------   2167
CawYV     RPI--FSFGK--IHQ---------------QLADAKS-----------------------   2213
SLSV      RPV--FKFKE--TVH---------------KEVLEAHN----------------------   2190
PVU       RPM--FDFSG--IRE---------------KIVT--------------------------   1935
BRV       ALPEPYIPPH-------------------------LRTRVR-------------------   2094
GBLV      R-VDAYVAPP-------------------------FHHRR--------------------   2095
BLMoV     R-VDAYRPPH-------------------------ITNRY--------------------   2113
AYRSV     KCKLPFHPPG-------------------------EKCTCDVPSP---------------   1659
GSPNeV    RHEFGYVPPPL---R---------------KM----------------------------   2152
GNVA      HCGGAFSSVT--------------------G----FQ---LHEGP---------------   2164
```

```
ArMV      LPTSFPNIVLIGGISYPKEGGEPGAL--YSPTDVV------MSKKLQGVYVSEAVLKCC-   2243
GFLV      QPDVYPSISLVGGVCFPNEGGEPGAM--YSETDVT------MAREVQGVYVSEACVKCC-   2244
GDefV     QPSTYPSVCLVGGISFPKEGGEPGAL--YSSADTV------MAKQVPGVYESEVCLKCC-   2244
MMMoV     -------------TELVKGGRK-------HYAFKD-----NALVSGRASFAATAKLKCS-   2247
HoNV3     -----------------------------VKALRKSTSLWPVLQLGRTEKEVSARLICD-   2228
PCMoV     EVWQFPLLILCEGLSLQKEGNAPGAS--YH----GKTFAWEEHCGESAILTTCGMFYCD-   2253
AVA       KSRVFPLLVEDTGFSILSEGEEPGAR--IKHVLQHEPGFWEQHKGKCAVVTSYAALYCD-   2306
PBRSV     ------D-FASKPTTHLRKCTDHGTDGGKALYI-------VQGL-----GVTAAKLVCT-   2297
TRSV      ------E-FASKPTTQLRKCTDPGVDGGKAMYI-------VRGL-----GRTAAKLVCS-   2274
AeRSV     ------G-FSSKPTTHLRRCTDSGADGGKAMYI-------VQGL-----GKTAAKLVCS-   2287
HoNV1     ------------------------------------------------------------   1975
HoNV2     ---------LVEDFE-----TQHGPY--YCTLK--------RNERIEGMDYCTISLKCG-   1820
MMLRaV    ------Y-ALGGVKGSINRALAS-EEFLPLLAI-------HSGLNNSGQQEVRIGARCS-   2066
RpRSV     ------G-RSSNVSERSQVQTFLHMSPKPCF------------ITLKGSGKVCHGLRCN-   2331
BRSV      ---E-GF-----PTLILNEAAKDRKV--PCMVA------------TQAKTKFKVSLACDS   2225
PoLNVA    ---T-GV-----PAIVLNQAARDGRV--PCMVA------------QAKGSRFRVSMVCDN   2240
TBRV      ---E-GL-----PTAILNQAAKDRKV--PCMVA------------RQWKSKFTVRMACDS   2225
AILV      ---E-GL-----PTQCLNQAVKERKL--PCMAA------------GQVRNKFIVSLVCDN   2239
RCNVA     ---ERDL-----PTLMLNRAAKDGNV--PCMVA------------QMKGKRCAVYLACDN   2216
GARSV     ---DAPL-----GTQALNEAAKMGKT--PCLFA------------SQQRGKFHVYMVCDN   2202
GCMV      ---VGPT-----NVQILNGAVRQGKV--PCLAA------------HSVGTKFKVSLVCNK   2208
PVB       ---NTAT-----VTTDLNAMSKLGKV--PAMSA------------RSFRSKFTVALACNK   2224
CNSV      ---TTEKFNLNGARDLIMQGQKLGKL--PVMAA------------TQAPNKFYVGLCCQK   2287
ToRSV     ------------------------------------------------------------   2197
AnNVA     ------------------------------------------------------------   1992
CLRV      ------------------------------------------------------------   2112
STeNV     ------------------------------------------------------------   2086
BaNV1     ------------------------------------------------------------   1869
BLSV      ------------------------------------------------------------   2172
PRMV      ------------------------------------------------------------   2167
CawYV     ------------------------------------------------------------   2213
SLSV      ---------------SVRVA----------------------------------------   2195
PVU       ------------------------------------------------------------   1935
BRV       ------------------------------------------------------------   2094
GBLV      ------------------------------------------------------------   2095
BLMoV     ------------------------------------------------------------   2113
AYRSV     ------R-----------------------------------------------------   1660
GSPNeV    ------------------------------------------------------------   2152
GNVA      ------T-FTNEKEFKV----WAGEKC-SSFPV-------FFSSCNKKDGAERIMFKCC-   2204
```

ArMV	LRCPGAAVKTVLQTSSPGSSL--------------------------------SQAHFRS	2271
GFLV	RRCVGVATRVVTDTQLFGNNL--------------------------------LKTHLKA	2272
GDefV	ERCIGVTTKVVTSTSVFANSM--------------------------------VKTHLKA	2272
MMMoV	SMCAGHEAMGIV---LVDIGVGN-----KWEDRFLY-----DDCSFEAACRRSRLPHYAA	2294
HoNV3	LSCEGHVVHKQLGPLDKCLDE-----------------------------VNGANPVTKC	2259
PCMoV	KICLGHAWGKSVGCRIIEDPTQKR-----GEEFFHFNGMWRDDSSLKCIK---AWPECAI	2305
AVA	AKCPGHIHGKSSGCRIIDSQDMYRDDLHGFEKFITFDGMYADDSKLKLSK---ANPRLAV	2363
PBRSV	DLCDGHV--VSCSNN----FDRM----------------V------------KDVLSQSC	2323
TRSV	DICDGHL--MSCNTS----FDKM----------------V------------VNLFRRSC	2300
AeRSV	DLCDGHL--ASCTNS----FEKM----------------V------------IDLFKQSC	2313
HoNV1	--	1975
HoNV2	NYCDGHEITSRAGLTAKSSALTR-------------------------VC----------	1845
MMLRaV	RRCRGHHHVVHCDGI----KEVD-----------------------------TENLLRQC	2093
RpRSV	NSCRGHISCTDVVRNSAANQRAA----------------M------------LDVLRRGC	2363
BRSV	TMCPHHTAVCETYEKA-------------------FRHCWLAKCKTSAV-----------	2255
PoLNVA	KMCPQHHETSQTFEQA-------------------FRMCWQAKCKTSGN-----------	2270
TBRV	NMCPHHSATCANFELA-------------------FKQCWLSKCKCAGN-----------	2255
AILV	TMCPHHRSTRQSYEEA-------------------FRECWTSKCKTSAC-----------	2269
RCNVA	KMCPHHHTTALNYEEA-------------------FRKCWEARCKTSKT-----------	2246
GARSV	KMCPHHHATSPNYEAA-------------------FRHCWLAKCKTKAC-----------	2232
GCMV	TMCPHHHHTGPTFEQA-------------------FRTCWLSKCKTKET-----------	2238
PVB	NMCPHHKATQDTMDKA-------------------FDVVWSQKCKTSNC-----------	2254
CNSV	NFCPGHATSSDSIAKA-------------------FSQCWAMRCAPNSSS----------	2318
ToRSV	--	2197
AnNVA	--	1992
CLRV	--	2112
STeNV	--	2086
BaNV1	--	1869
BLSV	--	2172
PRMV	--	2167
CawYV	--	2213
SLSV	--	2195
PVU	--	1935
BRV	--	2094
GBLV	--	2095
BLMoV	--	2113
AYRSV	--	1660
GSPNeV	--	2152
GNVA	KECPTHIATDKLGPI----PMRH----------------------------------MRL	2226

```
ArMV      LRRVQSHRCMRKS---------    2284
GFLV      LRKIQNHTCLRK----------    2284
GDefV     LRRVQSHMCPRR----------    2284
MMMoV     INKVRAMTCLGKWCGNVTIN--    2314
HoNV3     LRRLYNLRCYGK----------    2271
PCMoV     LENFKKYNCYL-----------    2316
AVA       FDHLKRSNCFRIK---------    2376
PBRSV     F---------------------    2324
TRSV      FKCL------------------    2304
AeRSV     F---------------------    2314
HoNV1     ----------------------    1975
HoNV2     -QDIAQQWCYKKKAPFYQSS--    1864
MMLRaV    LKSIRTHIC-------------    2102
RpRSV     YNIQ------------------    2367
BRSV      --------KVSPWHGTKLS---    2266
PoLNVA    --------KVSTWYGTKLS---    2281
TBRV      --------NVSKWYGTKFS---    2266
AILV      --------VVSKWYGTKLS---    2280
RCNVA     --------VTGKWYGTKLS---    2257
GARSV     --------QVSPWFGTKIS---    2243
GCMV      --------QVSPWFGTKFLGIS    2252
PVB       --------QVSDKFGLKQ----    2264
CNSV      ----RKVTFEPEWRKNKFLGIS    2336
ToRSV     ----------------------    2197
AnNVA     ----------------------    1992
CLRV      ----------------------    2112
STeNV     ----------------------    2086
BaNV1     ----------------------    1869
BLSV      ----------------------    2172
PRMV      ----------------------    2167
CawYV     ----------------------    2213
SLSV      ----------------------    2195
PVU       ----------------------    1935
BRV       ----------------------    2094
GBLV      ----------------------    2095
BLMoV     ----------------------    2113
AYRSV     ----------------------    1660
GSPNeV    ----------------------    2152
GNVA      LVQMRKQLCVPIVFNNN-----    2243
```
