# Supplementary material for: Re-examination of nepovirus polyprotein cleavage sites highlights the diverse specificities and evolutionary relationships of nepovirus 3C-like proteases
Source: Arch Virol. 2022 Aug 30;167(12):2529–43. doi: 10.1007/s00705-022-05564-x (PMC9741568; doi:10.1007/s00705-022-05564-x)
Supplement: Supplementary file 2 — Supplementary Material 2 [file 705_2022_5564_MOESM2_ESM.pdf]

Supplementary Material 2 – Alignment of shorter nepovirus P2 polyproteins

Re-examination of nepovirus polyprotein cleavage sites highlights the diverse specificities and evolutionary relationships of nepovirus 3C-like proteases

Archives of Virology

Hélène Sanfaçon

Corresponding author: Hélène Sanfaçon Summerland Research and Development Centre, Agriculture and Agri-Food Canada, helene.sanfacon@agr.gc.ca

Polyprotein sequences were aligned using CLUSTAL O(1.2.4), see Table 1 in the manuscript for accession numbers. Nepoviruses listed as subgroup A and B in Table 1 as well as GNVA were included in the alignment. Color lines on the left side of the alignment represent the protease clades as shown in Fig. 3 of the manuscript. Cleavage sites are annotated as follows (P1 and P1' positions). Yellow highlights: cleavage sites annotated in the NCBI accessions, underlines: annotated putative or confirmed cleavage sites from publications (see Table 2 for relevant list of publications for each virus), red letters: cleavage sites confirmed experimentally by Edman sequencing, green letters (cleavage sites confidently predicted), blue letters (cleavage sites only tentatively predicted, when two choices are possible, the darker blue is the one integrated in Table 2).

```
ArMV      ------------------------------------------------------------   0
GFLV      ------------------------------------------------------------   0
GDefV     ------------------------------------------------------------   0
MMMoV     ------------------------------------------------------------   0
PCMoV     ------------------------------------------------------------   0
AVA       ------------------------------------------------------------   0
PBRSV     ------------------------------------------------------------   0
TRSV      ------------------------------------------------------------   0
AeRSV     ------------------------------------------------------------   0
MMLRaV    ------------------------------------------------------------   0
RpRSV     ------------------------------------------------------------   0
OLRSV     ------------------------------------------------------------   0
BRSV      MGFRELFASSLGDAARAKASLVRGMSGWLNTTLATVQAAGPEIRACAYSALWAEVDSVKE   60
PoLNVA    MGFAELFASPLGDAARAKAAFNRGLSGWLNATLTTIRAAGPAIQEMAYSALWAEVDSAKE   60
TBRV      MGFKELFSSSLGDAARAKVSLVRGMSGWLDTTLATVQAAGPEIRNYAYSALWAEVDSVKE   60
AILV      MGFKELFASSLGDAARAKASLVRGMSGWLNATLATVQAAGPVIREQAYTALWAEVDSTME   60
RCNVA     MGFEELFSSDLGHSAQLKATLCGGLASFLRKTITTVQAAGPEIRKIAYSALWGEIDSVKE   60
GARSV     MGFGELFSSHLGVAAKAKASLIGGMSGWLTATLATVQAAAPVMREAVYTHLWRVVDSEKT   60
GCMV      MGFSEFFASALGTVARAKATLQGGFARFLSETVVTLQAASPEMRKFAYSKLWEEVDSVKE   60
PVB       MGFLELFSSEAGRLARAKAEALGRTKAICNYAVAFAQSLAKGTRKNFYCSVWEQIDSTME   60
CNSV      ------------------------------------------------------------   0
GNVA      -----------------------------------------------MIGAWAFA-----   8


ArMV      ------------------------------------------------------------   0
GFLV      ------------------------------------------------------------   0
GDefV     ------------------------------------------------------------   0
MMMoV     ------------------------------------------------------------   0
PCMoV     ------------------------------------------------------------   0
AVA       --------------MSVV------------------------------------------   4
PBRSV     --------------MELLCWQADATL----PRNVQHIGT---------------------   21
TRSV      --------------MEPLLWHVDATT----PSHIQALQSG--------------------   22
AeRSV     --------------MEPLLWQIDATS----SWNNPLLFPG--------------------   22
MMLRaV    ------------------------------------------------------------   0
RpRSV     --------------MSQ-FWGEFPEAVINTFQRLQIALIG--------------------   25
OLRSV     ------------------------------------------------------------   0
BRSV      LVPLSATMLLNQLRADLKCAQVRAQKCT-PASTSRFCSCGGIPLPGENKWEDEL------   113
PoLNVA    LVPLTQKRLRLELLAKLTTAQVRAQQCT-PIPAHRYCSCGAHPGQIEFQD----------   109
TBRV      LMPVTPAMLCAQLRKELWCDQKRTQRCA-PSSTSCFCKCGGMPLPPSIEWVDEE------   113
AILV      LVPVKRDRVLNELRAKLSTARVRAQKCT-PATTSRFCECGGIPAPGETIEAEEL------   113
RCNVA     LTPLTERMLADQLKADLLCTRVRTQKCN-PVTTSRFCSCGGVQKEPSMALVDVI------   113
GARSV     LVPVTEATMIAVLRQQLSCDKVRQQRCT-PASTSRFCSCGGIPGEATPTKILVD------   113
GCMV      LKPLTAQELVATLRKELWCAQVRAQKCT-LASTSRFCTCGGIPGEATPTVIKET------   113
PVB       LVPLSEVVERV---VPSWVQPVKSHSFGSFSFGRNYCRCGGIKPTAEMVDLVSALTVSTL   117
CNSV      ----------------------------------MFAPI-GAPGMGERAS----------   15
GNVA      ----------QRLRERLECFSLLPN----LFPPSPLSAPG--------------------   34
```

```
ArMV      ------------------------------------------------------------   0
GFLV      ------------------------------------------------------------   0
GDefV     ------------------------------------------------------------   0
MMMoV     ------------------------------------------------------------   0
PCMoV     ------------------------------------------------------------   0
AVA       ------------------------------------------------------------   4
PBRSV     ------------------------------------------------------------   21
TRSV      -------------------------------------------------SLPPA------   27
AeRSV     -------------------------------------------------TRPEC------   27
MMLRaV    ------------------------------------------------------------   0
RpRSV     -------------------------------------------------DIKKC------   30
OLRSV     ------------------------------------------------------------   0
BRSV      -----------VPIPDCPVGRNFCRDGVFCKRHHGPGETL--ERVQVLVEAPKCPHCQGT   160
PoLNVA    --------------------------------------------GKQYDSRYKCCFCNGT   125
TBRV      -----------VIIENCPSGRNLCHNGVRCIRHHGPGVAL--ERVQVLVEAPKCPHCHGT   160
AILV      -----------VPFEDCPPGRNFCRGGVRCKRHHGPGETL--EKVRTLVEAPKCPHCHGT   160
RCNVA     -----------VDVDICPPGRNLCRNGTHCTRHGGLGQFS--ERRIIPIEEPVCPHCSGT   160
GARSV     -----------EPIPRCPNGRNLCRHGPHCERHGGDLNATVQVERDVLVEAPICPHCQGT   162
GCMV      -----------VHVDECPNGRNLCRHGTRCLRHGGPGSFQ--QEREVQVDAPKCPHCAGT   160
PVB       SSPLNKQERRKLRRKRCPFSTTVCRPNSIDACHLCDQIQR-YEVTQEAPQPEECPHCLGT   176
CNSV      --------------------QNFR-------SASGFRDLLVHAVKVLVLSAYQRRPCTS-   47
GNVA      -------------------------------------------------DVPPPLFARGS   45

ArMV      ------------------------------------MAKFYYSDRRLACWAAGKN-----   19
GFLV      ------------------------------------MGKFYYSNRRLACWAAGKN-----   19
GDefV     ------------------------------------MGKFYFSDRRLAAYCLGTD-----   19
MMMoV     --------------------------------MA--V--VRPSSFPLS----VEEL----   16
PCMoV     --------------------------------MK--LDRIAVKSFFLT----SPEGL---   19
AVA       -----------LIGL----VLLICFLFSFLFRMK--IDTDCVTSFFLY----SPEGQ---   40
PBRSV     ----------PRSRV----ASAMAFFKKAARQFS--KQADACDLAALA----M-ERV---   57
TRSV      ------SPAAALTRV----QRALSFFRTAARKYC--KQADAPDLFALA----M-TRV---   67
AeRSV     ---------TPLSRV----QTALAFFKDAARKFS--KQADACDLMALA----M-ERV---   64
MMLRaV    ------------------------------------------------------------   0
RpRSV     ------PLSSPLFP--------------ELSKLDAHSQHHLLASFELP------------   58
OLRSV     ------------------------------------MVK--FTTERL-------------   9
BRSV      G---IIPRSEP--------------MAYIRSEYERQTKTFARPSNPLHEWILEEG-----   198
PoLNVA    G---IIPRSEP--------------MAHIRAIYARQTCDSVQPTVVFDRWVLEDG-----   163
TBRV      G---KIPRSEP--------------MQYLRNRYEQQQKTFARPSNPLHEWVLEEG-----   198
AILV      G---IIPRSEP--------------MAYIRAEYERQTKTFARPSNPLHEWVLEEG-----   198
RCNVA     G---ILPRSAP--------------YEYLQKNYELQTKTFARPSSPLHEWVLEEG-----   198
GARSV     G---IIPRSEP--------------MAHIRRCWRAQRKTYARPTSPLHEWILEEGG----   201
GCMV      G---IVPASAS--------------WREIRRCWREQRKVHSLPSLPLHPDVLFEGT----   199
PVB       G---IVC-SKE--------------WDLLRTTVRENRKTFQVPSLPLHEHVLQEG-----   213
CNSV      M---LRPREQK--------------RIKMRLKWMCLMK-----------FCFMQN-----   74
GNVA      TAFSLSPPSPPKISKTFSVKARTLRWEMWKVRYLAHLKRFLPPGFPKAARLMSHELHFQL   105
```

```
ArMV      ---------------------------PHLGGSIESWLAAIK------SDSSFRQTVKEDV    47
GFLV      ---------------------------PHLGGSVEQWLAAIN------TDPSFRQTVKEDV    47
GDefV     --------------------------G--RGTFEQWLQCM-------EDPSFRKEVKERV    44
MMMoV     ----------------------------KLFTAEIYHQ-YFVR----ADA--R-------    34
PCMoV     ----VNLRN------IAD------GASTKAALEYIMSQ-FDSS----ADA--R-------    49
AVA       ----LNLRN------IAD------GASTTRALSYVLQE-FRTS----ADA--R-------    70
PBRSV     ----AEHNG------ITV------TAANIQQLFEFVGQ-HVAD----PDE--R-------    87
TRSV      ----AEHND------IAV------DARNVEQLFHFVGQ-HVAN----TAE--R-------    97
AeRSV     ----AEHNG------FAV------RADNVRQLFNFVGQ-HVAN----PAE--R-------    94
MMLRaV    ------------------------------------------------------------    0
RpRSV     -----RFGG------VTP------G---------VMEQ-LHDAESELAEA--R-------    82
OLRSV     ------------------------------------------------------------    9
BRSV      --RESNHARRCSNWYHMSMSQIDKR-DNDPCDASTWMA-AAQILFDNADAQVY-------    247
PoLNVA    --SESRHATRCRNWAFTD------H-DDDPVDAQTWMT-AAQILFDHPDMRIC-------    206
TBRV      --SAGNWGRRCQNWKWTSSSQIGFT-DEDLCDVSTWMA-AAQILLDEEDCAVC-------    247
AILV      --REGAFARRCYNWRYTSRPKTGKE-ETEYVDASTWMA-AAQILFDDLDASCH-------    247
RCNVA     --SASRFIRRCQEWKWKSRDCLGLE-DENPVDVSTWMA-AAQILFDNTDARVY-------    247
GARSV     --SVGRWRQRCFQWSCH--PTHRYT-DGELVDQSEWMA-AAQILFDNEHCRIY-------    248
GCMV      --N--AWQTRL---RWLKTWRHVLG-DVKPCTPEKWMQ-AAQIMRTCAVPSFE-------    243
PVB       --LPGAW-------RYRSRIGYVSK-KHPITDVNTWMQ-AAAIVRSEPDFWM--------    254
CNSV      -----------NYWDTQRRFTGGMHNVPELATFDAWLQ-HWRVVHWRLNIISE-------    115
GNVA      LDMESNFR-------YHD------GPRGNTKKFYSWDHPMRAILFDKLDGKVR-------    145
```

```
ArMV      QVNRLQPTAVRMFSWKVGSGPIDNPEKCNWHYV-LT-G-----EV---------------    85
GFLV      QENREQPTAVRMFSWKVGSGPIDNPEKCDWHFV-LT-G-----ER---------------    85
GDefV     QFDRAVPSVSRIFEYPVGRGPVEGPAGIAWHYI-VH-G-----HS---------------    82
MMMoV     ---RAARARVREIL----------RSVGGWFY--CP-AAGHHAPLYEEVYDLPRE-----    73
PCMoV     ---RRIRAAIR--------------TT-EWYKVVKN-AFAS-------------------    71
AVA       ---RRIRAAIR--------------ST-DFYKVMCR-VATQEAAA---------------    96
PBRSV     ---KALRGALRE--------------QRKAFRASLP-G----------------------    107
TRSV      ---KALRAALRE--------------QRALFKASLP-G----------------------    117
AeRSV     ---KALRKALRE--------------QREAFCASLP-GA--PFPLPPRIIQIPLRDLLET    134
MMLRaV    -MDTQVTSFFFLLIFWV--------GFCYWYFVSSF---VETFSS---------------    33
RpRSV     --ARLLRERLHAVANK-----ENIPYLGDCMYYDAP-GINQ-------------------    115
OLRSV     --------AQFALAWNLGNGTYPNPPPV-WLYPTLV-GYVSKGPG---------------    44
BRSV      ---------------YP--------GSN----YR----------------------KLVG    258
PoLNVA    ---------------YP--------GPI----YK----------------------TLTG    217
TBRV      ---------------FP--------GSV----YK----------------------RLNG    258
AILV      ---------------FP--------NNT----RE----------------------RLNG    258
RCNVA     ---------------YP--------GTN----YM----------------------VLNG    258
GARSV     ---------------YP--------RTESWRNPD----------------------HLDG    263
GCMV      ---------------NP--------IPGQ-FGYE----------------------RLYN    257
PVB       ---------------R---------TDDPYLQRD----------------------IISG    268
CNSV      -------------------------ILIDFQALV----------------------RMMK    128
GNVA      --QHALTCPDQAFFYK-----ERGPDLRVWHDVPRKVVYDDDFPCPPPVSMQ---D----    191
```

```
ArMV      --------------P--A------------------------------------------   87
GFLV      --------------P--A------------------------------------------   87
GDefV     --------------LGLV------------------------------------------   86
MMMoV     EIEALCRWARAH-PEVFPPI----EVEEAFEEEPEFVI--------------------PV   108
PCMoV     ---------SSS-VAATTPA----KVEEDWGPAWAYNE--------------------CV   97
AVA       --------SQET-TAAVPVK----TEEEDWGPSRYYLP--------------------WA   123
PBRSV     ---------------ACFPM----PRP----IPS--------------------------   118
TRSV      ---------------ACFPA----PAPPGWGIPK--------------------------   132
AeRSV     KLQMEREFCESL-PGAPFPE----PKPEGWGIPT--------------------------   163
MMLRaV    ------------------------------------------------------------   33
RpRSV     ---------EEL-LQAAFLE----APTPEWESGR--------------------------   135
OLRSV     --------------RALFPIAVKSAIKAGWEPVFLIFPE---------------------   69
BRSV      GSKGY--------------------------DDWCQLP---------PSKEMCNRLFDWW   283
PoLNVA    GSDR---------------------------LDWARLP---------PSKEMCRRAYLWW   241
TBRV      GGKGY--------------------------DDWCRLP---------PNKEMCSRLFDWW   283
AILV      GSKGY--------------------------DDWYHLP---------PNKEMCDQLFSYW   283
RCNVA     GSKGY--------------------------DDWCRLP---------PSKEMCDRLFDWW   283
GARSV     GNGAY--------------------------ADWHRLP---------PSKYHCSQVFSWW   288
GCMV      GEGKE--------------------------EYWLQIP---------ATDKYTDLIINWW   282
PVB       GDGGY--------------------------DPWCYIA---------PTKHICAKIFDWW   293
CNSV      NLAGQ--------------------------CHFDFYP---------VCTHCVQLVNNWY   153
GNVA      ----MWELSEAIRSGMEALI----EEDECWEDADDFTPEEWEYMCLNAWEGNCDVTYEWL   243

ArMV      ---------QPTEPVKAREVVVP------PVKVIPS---------------PPPVP-RPY   116
GFLV      ----------PSRPVKADEVVVVPQP---KKVVIPT---------------PPPPP-APY   118
GDefV     ---------PPTQPAKADEV-VVPQP---KKVVIPL---------------PPPAP-KPY   117
MMMoV     AK--------PEFV-NVAWRSVERST--T--------------VVVEDCET-VPGV---E   139
PCMoV     SC--------RMHI-TIDWEVVPMSE--VPHKE-PK-------TEPYTCEAVPPMS---T   135
AVA       -T--------PHVF-TIDWEEVPMHD--VPHHA-PR-------Q-VYSDDFCPDSL---S   159
PBRSV     PP--------PLPT-FVVVKRPRFRVVAPPPFI-PR---------------APPLP---H   150
TRSV      PP--------PLPP-PFVWKGCRYNVVAPPPRI-PQ---------------PPPLP---K   164
AeRSV     PP--------PLPP-PFVWKGCHYNVVAPPPLI-PK---------------PPPPP---T   195
MMLRaV    -----------------------------------------------------SLL-LSS   39
RpRSV     IR--------PLWP-KDDWFRDAKQG----------------------------------   152
OLRSV     -A-RPTSVPTAAAPVKADWGPL-EAY---EPLCFPL---------------PLPGA-GSA   107
BRSV      HRQNTPDYYVSED-TLADFVKPRMGS---CHLPIEHDV-HL-LPREWHGRVSA-------   330
PoLNVA    YRNYIPGYQEPEEITLGDFTNPRMGV---CTIPVAHKV-DEKTFREKHGHSPL-------   290
TBRV      HRKYTPGYHTPED-TLADFVKPRMGE---CTLPIERDV-YL-LPREWSGKVPR-------   330
AILV      HRKNTPGYYIPED-TLADFTKPRMGA---CYLPIERDV-HL-LPREWHGRIPV-------   330
RCNVA     HRKNTPGYTTPER-TLVDFIKPRMGK---CYLPIEKEK-HI-LPYEWSGRVPC-------   330
GARSV     HRQNTPGYEEPLD-TLADFVKPRMGA---CSLPIARES-RV-APYVWAGSVPE-------   335
GCMV      HAKNTPGWEEPSS-SLMDFKRNRMGP---CLHIVEKRV-RN--SYVAPPWKPW-------   328
PVB       HTKYTPGYDCPNP-SFDDFDRKKMGP---PRWPLYNYESARTTQREMVHTVPK-------   342
CNSV      IAEFKPHLEEETE-WWKDLVKPRMGN---PVHEIEPNRK---SQYVESRRIPPPLN----   202
GNVA      ET--------HHPARFEAWIACYRAMHG---KQ-TQ-------TVEESVTVAAPVIEIPP   284
```

```
ArMV      FRPVGAFAPT---------------RSGFIRAT------------VERLSRKREESRAAA   149
GFLV      FRAVGAFAPT---------------RSEFVRAI------------VERLTRLREESRAAA   151
GDefV     FRPVGAFAPT---------------RSGFIRAT------------VERLSREREESRAAA   150
MMMoV     WSEVAALAEAV----------------------DA------LA-----------VADVLE   160
PCMoV     WEDIIGLSEAI----------------------EA------LQ-----------CAERAE   156
AVA       WEEVSGLFWAV----------------------SS------LAESAEEERLVAEEALVAE   191
PBRSV     FKKVVRPNFVV----------------------VA------PP-------------PPPE   169
TRSV      FAPFVRNNFRV----------------------VA------PP-------------PLGE   183
AeRSV     WKVIPRPCWRV----------------------VA------PP-------------PL-V   213
MMLRaV    FRLVGLDSSRA----------------------------FDT---LLRLPLARYSSLDTV   68
RpRSV     --------------------------------------------------------PYPE   156
OLRSV     FTLLSAHCPRLLLKRRKRTMTSSSLRGLFPRSLVRRETPFMRVMALASEAKESEEAEIAS   167
BRSV      -GNTSHFMACL----------------------D---------------GLSSSMEEFLD   352
PoLNVA    -GNPKHYFWCL----------------------E---------------GLKESLEEFDD   312
TBRV      -GDTSNFHWCL----------------------D---------------GLKSSLEEFLD   352
AILV      -GDTSHFEACL----------------------D---------------GLHSSLEEFLD   352
RCNVA     -GDFTVFGQCV----------------------D---------------GLMSSMEEFLD   352
GARSV     -GDFNHFLECC----------------------N---------------GLQSSMEEFLE   357
GCMV      -GEDIDILSVM----------------------D---------------SLSSQLEDFLD   350
PVB       -GDTDYFNYCV----------------------G---------------ALSSELEFFLD   364
CNSV      GDEFANFLHVC----------------------QCAKLAFDVERAATEENFEDALDTLEE   240
GNVA      VSDIVDLSEIE----------------------KI------YSFFSTFKSEEVKCAPVAH   316

ArMV      LFAELPL-----------------EYPQGAPLVVPRGFAAMRWTYHATWRRWYDASDER-   191
GFLV      LFAELPL-----------------EYPQGAPLKLSLA-AKFAMLKHTTWRKWYDTSDER-   192
GDefV     LFAELPL-----------------EFPQGAPLRLSLA-VKFAMLKHTTWRKWYDTSDER-   191
MMMoV     AFEELPL-----------------EYPAPAPVKVGLAFFQARR-----------------   186
PCMoV     LFLALPE-----------------EYPHAPEKKNVAVGFSRLF-----------------   182
AVA       LFSELPL-----------------EYPHAPAKKNVAAGFHQLF-----------------   217
PBRSV     VYQSPGA-----------------PFPLGR-SDQALRFFKLAA-----------------   194
TRSV      VYQPVGA-----------------PFPQTR-ASAALSFFRTAS-----------------   208
AeRSV     VYQPVGA-----------------PFPRSR-SQSALAFFRVAA-----------------   238
MMLRaV    FFPEFSA---------YVSRS--ESYFGSTFVTRLFSRFGF----WTLFVHFYNLDLLFS   113
RpRSV     DYGDIPL-----------------------------GDFDNLY-----------------   170
OLRSV     AFDELPL-----------------EYPQEEFVPDLLARLRGAIQDGKRFRL--KEAAEL-   207
BRSV      VFYDCAA-QFNGDIEIFLDTNE-KPSRVVGNLGGVRVLLTTPA-----------------   393
PoLNVA    HFYDCPE-PEV-------------------------------------------------   322
TBRV      VFYDCAA-QFDGELEISLGAHN-KATRCHGKLGGVQVLFTTPA-----------------   393
AILV      VFYDCVA-QFDGHLELFLDTNE-KPSRVTGKFGGVQVLLTTPA-----------------   393
RCNVA     VFYDCAA-QFDGYLEFYLDASE-RPSHIEGKLGGVQVLFKTPA-----------------   393
GARSV     LFYDCAA-HFDGELEFSLDASE-RPSRVSGKLGGVQVLLTTPS-----------------   398
GCMV      VFYDCAA-QFDGELEFSLS-ND-RLSSVTGELGGVPISIGAPS-----------------   390
PVB       EFYDCAS-TFDGELNLSIGNVSQRPYRAYFETSRLKVS----------------------   401
CNSV      DFYDIDS-TIPKRDLLAADARV------------VKRAFTLRR-----------------   270
GNVA      AEISVPIFSFEGELEIENE--FIREFTPEEEMDIEENFLRSST-----------------   357
```

```
ArMV      ---ALRVHPGGPALPPPPPPPPIQK---------PPSFEERLQAALERQSCARAFALET-    238
GFLV      ---LLEAHPGGPCLPP---PPPIQN---------PPSFQERVREFCRMKSCTKAFALET-    236
GDefV     ---LLEAHPGGPCLPP---PPLIQN---------PPSFCERVREFCRMKSCARAFALET-    235
MMMoV     -----------KCVQVEH-KELL-----------RQSLPKAIAAASAPRCGYPVKG-IFE    222
PCMoV     -----------NCRRFER-LVEK-----------KGILNDALNFASVTKTEMPLTG-QML    218
AVA       -----------KLRSHER-KVEA-----------KGIIFDAISCASAARESFVLSA-PAV    253
PBRSV     -----------TCRQTL------------------------------------V--CAV    204
TRSV      -----------TCRQVL------------------------------------V--ESC    218
AeRSV     -----------TCRQAL------------------------------------V--CEL    248
MMLRaV    FVLFYLNLILDSCLLILTSFHRIVSSFYFNCTVFTSFYFMGFLDFCRQQAEKRAVAAAQ-    172
RpRSV     -----------RAFDALV-EEHW-----------MSVYSTTLNPFALLRCGSEFVEECVV    207
OLRSV     ---AVREAAAAARL---------------------KERQRIALKCAMQAASMEP---V-    238
BRSV      -----------VCSPAKL-LPEI-----------EESDFDQLE-------------DESI    417
PoLNVA    ----------------------------------TPRKVQSLM-----------------    331
TBRV      -----------VCSPAKL-LPEI-----------GESDFVELE-------------EEET    417
AILV      -----------VCSPAKL-LPEI-----------GESDFDQLE-------------DEAI    417
RCNVA     -----------VCSPAKL-LPEI-----------GESDFDALE-------------SDV-    416
GARSV     -----------VCIPENL-VSNI-----------TEDDFDSLE-------------DEE-    421
GCMV      -----------KISNTP------------------------------------------    396
PVB       -----------------------------------------------------------    401
CNSV      -----------KRRPN--------------------------R-------------TSVY    280
GNVA      ----------------SV-SDTD-----------QTLISDTVKQFCNAKAELAMVEEGAS    389
```

2a-MP cleavage site

```
ArMV      -SLGLNMAWLGTAAI----P----ST-SVCCA--DGRTTGGQTIA-QEADPINHRV----    281
GFLV      -SLGLNKAWVGLVDI----P----ST-SVCCA--DGKTTGGQTIA-QEADPLQHRI----    279
GDefV     -SLGLNKAWVGYVDI----P----SI-SVCCA--DGRTTGGQTIA-QEADPLQHRV----    278
MMMoV     GDGRVFV---------------------ACAP---------------------ASSEQAE    240
PCMoV     HQVQAF-------------------------A---------------------PVGDDSE    232
AVA       NQVIAM-------------------------A---------------------STSEAYE    267
PBRSV     AEQPALM---------------------HCCA---------------------STDEVQE    222
TRSV      IQQPAFM---------------------TCCA---------------------STGEVQE    236
AeRSV     VREPAFM---------------------TCCA---------------------STGEVQE    266
MMLRaV    RDAELYALLVERIALIEQSNLQATRAPASETS--DARASGSQTQVVTAAAPFQQPGG---    227
RpRSV     SAGSLIP---------------------ACMM----------------TDHHLQPTGDRQA    231
OLRSV     -YLSFSPRVVEMIAR---SP----VGQGSHIH--TLQSEGGQIPI-EPAPNFQGRSQMVG    287
BRSV      SCDSMIPPLFRDNGL---------SALYANLVLK----QATVQSIL-MAHPDQ---DEIE    460
PoLNVA    --------------DE---------TALYACLVLC----RYVTNPTL-MAHPDQ---DEIE    361
TBRV      ---VSIPSLFKDNGL---------SELYMNLILK----QAVVTPTL-MAHPDQ---DETE    457
AILV      NMESQIPPLFHDNGL---------SALYANLVLK----QAFVEPTL-MAHPDQ---EEVE    460
RCNVA     -HDSHIPALFRDNGL---------SALYSNLVLN----AAKVQPVL-RAHPDR---EEIE    458
GARSV     -EDSHIPPFFRDNGL---------SALYANLVLK----HATLDLVMTAPHPDR---EEVE    464
GCMV      -------P---KVNF---------AELYGNLVRHNHRKISALRPIL-MAHPDQ---DEIE    433
PVB       ---------IVDKIYL---------ADAYK-M-------VHCQEPVQVMA-HGR---DEAE    432
CNSV      SMKGQPPVTFSASD----------------LVSC----LGQVLSTLPTVKMDR---EAIE    317
GNVA      LLQDVFKSVVSEITH---------ATQKQCEIAS-LDYLGG--LEVHMNNEKPGISENQE    437
```

```
ArMV       --------------TSNTAPGRAQWISERRSALRRREQ-ANSLQSL-----A-AQTDMTFE   321
GFLV       --------------STSVAPGRAQWISERRQALRRREQ-ANSFEGL-----A-AQTDMTFE   319
GDefV      --------------SSNTAPGRAQWISERRQALRRREQ-ANSLQGL-----A-AQTDMTFE   318
MMMoV      SSAAQQQ-LRDEGGIPTVVQVAKRLRNRNVALTSTSKKSFFFKEAGESRTI-EGGGVELT   298
PCMoV      EASHQAE-MREEQGIGTIADLKEKLAKRKAALPRKRGRVEEFKEAGHSRYD-PGGGLKVT   290
AVA        ERLHQEE-TRESTGIGTLADLKQILAKRKAPLPRRKGKQEEFSGAGEGQFI-PGGGLKLS   325
PBRSV      MTAMLTE-ARQSGKILTPKEVTSALAL----------KRKEIKGAEENRIS-FDEGVHLS   270
TRSV       MTSMLTE-ARQTGKILTPKEVSQALAQ----------KRKEIKGAEENRIS-FDEGVHLT   284
AeRSV      MTAMLTE-ARSSGKLLTPKEVSSALAL----------KRKEIKGAEENRIS-FDEGVHLS   314
MMLRaV     ----------------VTGVAERWRRARAQEQNVREQ-ARRLSETGSG--T-ASATPTFT   267
RpRSV      DKEERQDYADSQDSIQSMGDFWKEFYS------KDSG--KKIPDSHKSRLANDPNKVGFT   283
OLRSV      SSSGAV------SAPYKQEARNKWLRNRRSQIDS--Q-EDNIRKY-----A-DQQGISFE   332
BRSV       DQVDHLE-NKQGGEIVTTPAFIKMLKEKRKEV-----RGKEFAEGSEGRLV-RASDLTLS   513
PoLNVA     EQLDHLE-NKQGGEIVSTPSFIKMLKEKRKEV-----RGKEFEQGSEGRLV-RSADLELS   414
TBRV       DQLDHLE-NKQGGEIVSTPSFIKMLKDKRKEV-----RGKEFVEGSEGRLV-RSADLELS   510
AILV       DQRDHLE-NKQGGEIVTTPAFIKMLKDKRKEV-----RGKEFAEGSEGRLV-RSSDLTLS   513
RCNVA      DQKDHLE-NKQGGEVFSTPSFIRMLKAKKKEV-----RGKEFKNGSEGRLV-RSADLELS   511
GARSV      DQLDHLE-NKQGGEIITTPAFVKMLKEKRKEV-----RGNEFVSGSEGRLV-RSSDLTLD   517
GCMV       DQLDHLE-NKQGGEIVSTPSFIKMLKEKRKEV-----RGKEFEEGSEGRLV-RSKDLELS   486
PVB        DQLDHLE-NKQGGEIFSTPNFINMLKSKKKEV-----RGKDFAGATEGRIV-RSADLELS   485
CNSV       DQQDHLE-DKQGGEILTTPQFIEVLRKKKREV-----REKEFDDSTQGKLL-PAEDFTLS   370
GNVA       TRAEIVE-A---FGSGQLSDIVTILDVARKKVEENRQNGLKIPDSAVGRRARDQNKIKLK   493
                                                     :                .

ArMV       QARNAYLGAADMVEQGLPLLPPLRNAYAPRGL----W-RGPSTRANYT------------   364
GFLV       QARNAYLGAADMIEQGLPLLPPLRSAYAPRGL----W-RGPSTRANYT------------   362
GDefV      QARNAYLGAADMIEQGLPLLPPLRSAYAPRGL----W-RGPSTRANYT------------   361
MMMoV      ---------E-KDVFHRM---------------GVVKSWRTPDHNKTF-----VACVRPIE   329
PCMoV      ---------A-DDIFTTG---------------SIVKRWLPKSQEPQKPDDVRIDTNHVIK   326
AVA        ---------E-DDVFHVG---------------SIVKRWMPQSQEPKKAEDIRVRVEHPVK   361
PBRSV      ---------E-SDVFHRL---------------SLTKRYLAPKRDRTL-----VDVLMPTE   301
TRSV       ---------E-ADVFHRL---------------SLAKRFMAHKRDRTL-----VDVLMPTE   315
AeRSV      ---------E-EDVFHRV---------------GLLKRFSAPKCDRTL-----VDVLMPTE   345
MMLRaV     QAGRAYLLADGATATDASLLPRRGAIYGSRVMGIPRLARSVDVRSLRT------------   315
RpRSV      ---------K-SALFHKQPLA------H-----SLAQTWANFRGTQDKADLVKVTMDMNIE   323
OLRSV      NARAAFLGATEAIPRQDPILPPLKLAYKRRGT----FAFGPSTRAQRT------------   376
BRSV       ---------R-EDVFLSGGLF------EKFRKSGIVQTFKGKD-----PKLTKVCVDLTNS   553
PoLNVA     ---------K-KDVFLAHSIM------DKFHNLGVVKKFSKSD-----PKLTKVCIDLTNQ   454
TBRV       ---------K-KDIFLAHTLM------DKFHGMSIVRKFGKSD-----PKLTKVCVDLTNQ   550
AILV       ---------K-DDVFLSGGLL------EKFRKSGIVQSFKGKD-----PKLTKVCVDLTNS   553
RCNVA      ---------K-NDVFIANTLM------ESLRAKNIVRTFMGND-----PRLSKTCVDLTNK   551
GARSV      ---------K-RDVFLSNSIL------ENLKKAGIVKSFVGKD-----PKLTKVCVDMTNQ   557
GCMV       ---------K-KDIFLAHTLM------DKFHGMSIVKKFGKSD-----PKLTKVCVDLTNQ   526
PVB        ---------K-QDIFLTNSLM------EKMRKSRIIKRFAGMS-----PLLTRMNMDITNK   525
CNSV       ---------K-HDVFLANSVL------DGLRKSKLIQRFAGKC-----ATSTKITVDLTNK   410
GNVA       ---------E-RDVFHGDVMS------D----NKLLSLWVDI------NLLNKVTAAQPIL   528
```

```
ArMV        -LDFRLNGIPT-GQNT------LEILYNPV-ADEEMDEYRD---RGMSAVVIDALEIAIN    412
GFLV        -LDFRLNGIPT-GTNT------LEILYNPV-SEEEMEEYRD---RGMSAVVIDALEIAIN    410
GDefV       -LDFRLNGIPT-GTNT------LEILYNPV-SEEEMEEFRD---RGMSAVVIDALEIAIN    409
MMMoV       QETVRMPKLRPDEKLE---CVPIFSPLPRC-LEETVRRLLEAGWKNTSSVCTDILVQSHL    385
PCMoV       HEMVKYLP-------S---EGPIFKSLPRM-TEEQLRKRLEQGWRGQTSVCCDLSIVSHV    375
AVA         AEMVRVPQ--GVDHSE---SIPIFKPLPRM-TEDSLRKLLEKGWKGQTSVCCDLSIQSHV    415
PBRSV       HETVRYPGTRPDGTLQ---M--CVSALPRM-SEEAARKLLEKGWKNSKNVSLDIGVTSYM    355
TRSV        HEVVRYPGTRPDGTLQ---M--CVSALPRM-SEEAARKLLEKGWKNSKNVSLDIGVTSYM    369
AeRSV       HEVVRYPGTRPDGGLQ---M--CVSALPRM-SEEAARKLLEKGWKNSKNVSLDIGVTSYM    399
MMLRaV      -LMTEAGAIRA-SDGG------VLINFNPC-PEEEMEAQRD---MGNNSYHLDLVELFVD    363
RpRSV       KYTVRLPDAVRTTAGP---LYIEWINLPRM-SENSARKLAEAGWNNADICGVDLAVKSHI    379
OLRSV       -LDVRLGQPTQ-NGQS------MVYYFNPV-SQQEIQDAQG---QGNDTVVIDAIEISIL    424
BRSV        QEIIKYPSKEMCSDSSGVHTGQVFTVLNRP-LYNELNKLAESGWKEAKSVCLNLHIRSYV    612
PoLNVA      EEVIRFPAKELQSTVDGVLSAQTFTVLNRP-QFNELNRLAEAGWKEAKSVCLNLHIRSYL    513
TBRV        EEVIRFPAKELQTTSEGVLSAQTFTVLNRP-QFKELNKLAEAGWKEAKSVCLNLHIRSYL    609
AILV        QEIIKYPVKEMCSDSSGIHTGQVFTVLNRP-LYNELNKLAESGWKEAKSVCLNLHIRSYV    612
RCNVA       AEIVKYPSRELTSTAEGVLTAQVFTVLNRP-QYQELNKLAERGWKEAKSVCLNLHIRSYL    610
GARSV       QEIVRYPSKEMCSDTSGVYTGQVFTVLNRP-QFKELNKLAEAGWKEAKSVCLNLHIRSYV    616
GCMV        EEVIKYPVKELQTTSEGVLSAQTFTVLNRP-QFKELNRLAEVGWKEAKSVCLNLHIRSYL    585
PVB         DEIVRYPVKELQCTIDGTLSAQTFTVLERP-RESLICKMSEEGWKEAKSVCLNLHVRSYL    584
CNSV        EEVVRYGPKELASEG----FRQTFNVLNRP-EYNALNKLAEAGWKEAKSVVLNLHIRSYL    465
GNVA        NKTVYYPGDTQGADCP---C--VYVTLPPGDLNDIRKQILEPGWSSTSRIALNMAYISAM    583
                                             .                  :

ArMV        PFGMPGNPTDLTVVATYGHERNMERAFIGSSSTFLGNGLARAIFFPGLQYSQEEP--RRE    470
GFLV        PFGMPGNPTDLTVVATYGHERDMTRAFIGSASTFLGNGLARAIFFPGLQYSQEEP--RRE    468
GDefV       PFGMPGNPTDLTVVATYGHERDMTRAFIGSASTFLGNGLARAIFFPGLQYSQEEP--RRE    467
MMMoV       GVGTP---VSALLTIVDSRTDDATEALLCGAFIKLGSDFAKFLSTPLINFPLS-------    435
PCMoV       GYGTP---IVVFVALLDTRTTNAEESYLAGSYIDIGRMKASVLSMPLINLPMG-------    425
AVA         GYGVP---IVVFVSLLDTRTTCAEEAYLTGAYLDIGRNKASMLSVPLINLPMG-------    465
PBRSV       PYGAP---IVAFMTIMDGRTTDPQEAALCANYMDLGREKSKVLSLPLVTIPLS-------    405
TRSV        PYGAP---IVAFMTIMDGRTDDPQEAALCANYMDLGREKSKVLSLPLVTIPLS-------    419
AeRSV       PYGAP---IVAFMTIMDGRTDDPEEAALCANYMDLGREKSKVLSLPLVTIPLS-------    449
MMLRaV      PSGLGGDDTDLSIVATWGGETEFTRAWLGTTAQYLGNGSSATVLAHGVRIFYESA--NHH    421
RpRSV       AVGTP---VRVIISLVDGACSDMPTATMCAFEVNLAAQNNRSLNLPLLSLPFS-------    429
OLRSV       PFGMPGDATDITGVVMWAQNSDPERAFIGSLSTFVGNGLARAIFFPQLHLSYEHCQVPDN    484
BRSV        PVHTP---LYAFCVIMWGHSSDAETASLCGAGVYLGDQEAAVLELPLVCSYLG-------    662
PoLNVA      PVHLP---VYAFCVIMWGHSSNAAQASLSGAYVYLGDQEASVLQLPLVCGYIG-------    563
TBRV        PVHLP---VYAFCVIMWGHSSNAEQASLSGAYVYLGDQEASVLQLPLLCGYIG-------    659
AILV        PVHTP---LYAFCVIMWGHSSDAETASLCGAGVYLGDQEAAVLELPLVCSYLG-------    662
RCNVA       PVHSP---VYAFCVIMWGHSSNADLASLCGAYCYLGDQEASVLELPLLCSHIG-------    660
GARSV       PVHTP---LYVFCVIMWGHSSDAETASLCGAGCYLGDQEAAVLELPLVCSHLG-------    666
GCMV        PVHLP---VYAFCVIMWGHSSNAEQASLSGAYVYLGDQEASVLQLPLLCGYIG-------    635
PVB         PTHVR---VYAFCVVMWGHSSNAEVASLSGSYLYLGDGEASMLQLPLICNPLG-------    634
CNSV        PQQMN---AYAFCVIMWGHSSDAQEAALSGSYVYLGDGEATMLQLPLLCEYVG-------    515
GNVA        PEGKP---IATYISLMWGGSSDLPTSFLSGALIDLGKRRADVVTLPRFSSPCT-------    633
                                     : :      :.      :    .
```

```
ArMV                SIIRLYVASTNATVDADSILAAISVGTLRQHIGS--------------L--------HN-    507
GFLV                SIIRLYVASTNATVDTDSVLAAISVGTLRQHVGS--------------M--------HY-    505
GDefV               SLIPLHVASTNATVDTDSLLAAINVGTLRQHVGS--------------M--------HY-    504
MMMoV               ----------KEIQDLDQYLGGLRLVTYLNNVQG--FYEGTPLFSYGTVEFQEHRP-VVS    482
PCMoV               ----------DTIEDHDNFLSGLCLVFYYQHVKG--FKSGMPLLSYGAIEFSELTA-AAN    472
AVA                 ----------ETIMDIDNFIGGLCLVFYYQHNRG--FRPGIPLLSYGAIEFSEITA-STN    512
PBRSV               ----------EI-EHDTTILDCLYIVTYFHGVQS--YQPGTLMLSYGTLEFQEYSNNSFT    452
TRSV                ----------EI-EHDQGILDCLYIVTYFHGVQS--YQPGTLMMSYGTLEFQEYSNNSFT    466
AeRSV               ----------EI-EHDVGILDCLYIVTYFHGIQS--YQPGTLMMSYGTLEFQEYSNNSFT    496
MMLRaV              RDLQLRVGSTNSTRDTDQVLAAIYVGTVRQHTGP--------------D--------SSL    459
RpRSV               ----------RLLADLHDFQHRVKIACQFRDPEG--FNVGTPMLSFSSLEFSELKQTAFE    477
OLRSV               RVLKVILSSTNSTMVGDLVQARVSLGTLRQHIGP--------------G--------HD-    521
BRSV                ----------NSLEDFDAYKRSLVLSTVFFGKSG--LFAGQNVFGITAVEFTEYMPTSYG    710
PoLNVA              ----------KSLEDMEAYQRSLVLSTCFFGTSG--LSAGQNMFGITAVEFTEYLPTSYG    611
TBRV                ----------NALEDMDAYKRSLVLSTCFFGTSG--LSAGQNMFGITAVEFTEYLPTSYG    707
AILV                ----------NSLEDFDAYKRSLVLSTVFFGKSG--LSAGQNVFGITAIEFTEYMPTSYG    710
RCNVA               ----------NSVDDFQAYERSLVLSTCFYGLSG--IKAGQQMFGITAVEFTEYLPSSFG    708
GARSV               ----------NSLEDFEAYQRSLVLSSVFFGKAG--LSGGQPVFGITAVEFTEYMPTSYG    714
GCMV                ----------NALEDMEAYKRSLVLSTCFFGTSG--LSPGQNMFGITAVEFTEYLPTSYG    683
PVB                 ----------NELQDQDAYRRSLVLSTSFFGPTG--FKPGQPIFGITAVEFTEYMPTSHG    682
CNSV                ----------HNLQDFEAYKRSLVLSTVFPEFSG--IADGKAMFGITSIEFTEYLPTSHA    563
GNVA                ----------NV-DDLNTMVDALYCLVSFPGLRKGMIHENQAMFSFGFVEFDEHKKSAMN    682
                                          :


ArMV                -RTV-----ASSVHAAQVQGTTLRATMMGNAVVVSPEGSLVTGTPE---ANVQIGSGS--    556
GFLV                -RTV-----ASTVHQAQVQGTTLRATMMGNTVVVSPEGSLVTGTPE---ARVEIGGGS--    554
GDefV               -RTV-----ASAEHQAQVQGTTLRATMMGNTVVVSPERSLFTGTPN---AHVEIGGGS--    553
MMMoV               SVTRTREGWDDLIANNE---RQGFRVQAGFNTIQPIEKDTQPMLPDFPDFDLVSVPRQVQ    539
PCMoV               YRTKARDSWEQVV-RDD---KHDRRIIAGLNALQVLEKDLNEPIPSLDAVQIAVPASAVQ    528
AVA                 YRTKARDSWAEITKRND---PQGKRIVAGLNAIQTLEKDFNEPLPSLGEVKITVPYAAPE    569
PBRSV               TATRVRESWDQILQRNS---NLGKRVHAGIGVLGTIEKEMDQALPDFPQIDLQVRPQPVV    509
TRSV                TATRVRESWDQILKRNE---NLGKRVHAGIGVLGTIEKEMDQQLEDFPAINLETRPRPVV    523
AeRSV               TATRVRESWEQILKRNE---NLGKRVHAGIGVLGTIEKEMDTQLAEFPALNLETRPRPVV    553
MMLRaV              RHVI-----SNTVARDQEAGRLIRAQQLGNVWAITPTGGFVPGIPD---VRMRLHGNS--    509
RpRSV               RNSLLRDSWSEIEKRAC---HGGGRCVASQGIVQTWEKEVNPPLKEYAPLVLPPVPQPKR    534
OLRSV               -RTI-----TRDLQNSQVLGLQLKATQLGSAVTTAPSGGEVIGIPD---ANVDLGGDT--    570
BRSV                GITHERDSWQAMLRNHQ--GKDKGRFIAGFNVVDALERDKEEPI-KMPNLDLEPVPRTQP    767
PoLNVA              GITHERESWNQMLKNHQ--GNEKRRFLAGFNVVDFVEAGKDKQL-VFPDFDLQPVPRSQP    668
TBRV                GITHERDSWNQMLRNHQ--GVDKQRFISGFNVVDFVEAGKEKQL-NFPEFDLQPVPKSQP    764
AILV                GITHERDSWQAMLRNHQ--GKDKGRFIAGFNVVDALERDKEEPI-KMPSFDLEPVPRTQP    767
RCNVA               GITHERDSWHALLRKHQ--GKEKSRFISGFNVVDVLEAGKERGM-KFPDFKLEPVDLTQP    765
GARSV               GITHERDSWQAMLRNHQ--GKDKGRFIAGFNVVDALERDKEEPI-QMPHLELEPVPRHRP    771
GCMV                GITHERDSWNQMLRNHQ--GVDKQRFISGFNVVDFVEAGKEKQL-HFPDFDLQPVPKHQP    740
PVB                 GISHEQDSWDKMLQAHQ--GPNKQRFISGYNVVDVIESGADKAV-KFPEFDMSAVPNHQP    739
CNSV                GITHERDSWDAMLRNHT---EEKRRFLAGFNVVDTIEKGNRKGF-SFPDFDLKAVPRHQA    619
GNVA                CATRLRSTWDDIFNHSS---NDPARIVAGYSVLDSVEADTSEPIPEMDLSGLRCRPSKKP    739
                                            .                      :
```

```
ArMV      ------SMRMVGPL-AWENVEEPGQTFTIRNRSRS-MRVDRNADV---------GV--AL     597
GFLV      ------SIRMVGPL-QWESVEEPGQTFSIRSRSRS-VRIDRNVDL---------PQLEAE     597
GDefV     ------SINMVGPL-QWESVEEPGQTFSIPSRSRS-VRIDRNADV---------GE--AL     594
MMMoV     RPILVGQDG-VQAP-LQRCSSVRVPGFRMAGG-APRFSVERGRVS--------RQTDVNI     588
PCMoV     MPAYLRGGKVIQPS-EIVSKKLHIPAMRAPNR-TGRLMFESGESS--------ARFNQQF     578
AVA       MPAYISQGRVVQPT-LGRVQSFTVPSSRVPNT-TGRLSFAGSRPP--------DE-----     614
PBRSV     RTF----QETAR-P-QLACRSMRIGTTSFTGN-TGRKSLDVV--H--------K-WQDV-     550
TRSV      RTF----QNAHQ-P-LHKTRSMRIGTTSFTGN-TGRTVLPPV--V--------KTYEEG-     565
AeRSV     RTF----QQPEQTG-LNKCRSMRIGATNFSGI-TGRKTLQTMPVV--------QHYEDP-     598
MMLRaV    ------VLEMVDPM-TYRAVSTPDQWFTVRGDGQGRLNRTRSNFVSLPREEVARRSIDAL     562
RpRSV     NFIDQQSGEVVRPL-IQKSRSMRFKSPSDL---WSRPSVDGGST----------------     574
OLRSV     ------TLEQTGTF-SFALRRAQSQRFEILGSRRSRPSVSREEDV---------RNLEAA     614
BRSV      IVRTFTG-EGKQPL-LNKSRSMRIQSFVSFRG--SN--IPVGRRI--------DNTAEAI     813
PoLNVA    VVRTF-N-DSKQPA-LGRTCSMRVRTFRPFMA--GN--IPIERRT--------DNTKTAV     713
TBRV      TVRTF-G-EEKQPL-LNKSRSMRVKTFSSFRA--GN--IPVGRRL--------DNTTEAI     809
AILV      IVRTFTG-EGKQPL-LNKSRSMRIQSFSSFKG--GN--IPVGRRV--------DNTTEAI     813
RCNVA     IVRNF-G-EEKQPL-LNKSSSLKVGTFERFRA--GN--ISIGRQL--------DNRVDAI     810
GARSV     VVRTFTG-EGKQPL--DKSRSMRIQSFAAFRG--GS--IPVGRRV--------DNTAEAI     816
GCMV      IVRTF-G-KEKQPL-LNKSRSMRVKTFTSFRA--GN--IPIGRQI--------DNTAEAI     785
PVB       VVRKFND-KVGVLG-IQRSTSMRVKNFSKYAG--GN--IPVVNHF--------DYTQNIA     785
CNSV      VVRTFED-QDVAPI-LSKAKSMRVKTFGSFRA--GN--IPVNFLG--------TPSNGQV     665
GNVA      EVRKMTSSGLVQPPILSRTASARISLFPSNVR-TGRRSIDCGSWA--------GTSADK-     789
```

MP-CP cleavage site

```
ArMV      P---------------------------------------------RMSTTTRGLAGRGS     612
GFLV      P---------------------------------------------RLSSTVRGLAGRGV     612
GDefV     P---------------------------------------------RMSSTTRGLAGRGS     609
MMMoV     Y-----------------DVNDNPRHFQA-----------MADCPIVDTVGNLAYVSG     618
PCMoV     KTELMPKIIESGVGSTRSNSEQETEPMHETT--------------ALGQVADHNFLFNKK     624
AVA       -----------------ATGKRDAVPRHTAV--------------PLGVSADPNYVYSER     643
PBRSV     ---------------PSGSSSIPPLPRHS-----------DHSFTAPTVVVDPSCCGHLS     584
TRSV      ---------------NANFDSLQSKPRHSSAST-------AHLMCAVTVVPDPTCCGTLS     603
AeRSV     --------------------PPIQSKPRHSV--S-------GHGFMAPTVVPDRSCCGVFS     630
MMLRaV    THSQIP-----------------GNPRDMVE--------EPRTSNLAGFSTPADILFSTS     597
RpRSV     ----------------------FTLSPSRGSLRC-------DNVPGCAYEVDPLHLLYYEL     606
OLRSV     RHSFM-------------------------E--------AKAGSTQQESERPVDLVGVKW     641
BRSV      N-----Y--EL----GRASTSS-VSSRLD------------ESKCNLKAGGSYAF--GET     847
PoLNVA    G-----Y--EL----GRASVSG-TAPRLD------------DSMCNLKSNGDYAF--GET     747
TBRV      N-----Y--EL----ARASTSN--QPRLD------------ESSCNLKADGDFAC--GET     842
AILV      N-----Y--EL----GRASTSG-LNPRLD--------------ACNLKANGDFAF--SQR     845
RCNVA     E-----Y--EL----GRASTSR-VGDLRSEIDGV----ERRLNTFNLKSLGDFAF--SAR     852
GARSV     K-----Y--EL----GRASTSE-LGTSLKGRLD--------EQSNIRSNDGDFVF--VHT     854
GCMV      N-----F--EL----GRASTSNAINPRLD------------TSETNLRAGGEFAF--IHT     820
PVB       S-----GVGES----DRKSASIVHDPILQTIPTVLLKDAGKDTNCFASDLGPMEYIGTEM     836
CNSV      A-----S---------KHSVSE----------------NAGYSVGDMKSAENFVF--TQL     693
GNVA      -------------------ASASDNPNTDQREK-------TWDAHADSLPGDMEIIALEF     823
```

```
ArMV        VQVPKDCQAGRYLKTLDLRDMVSGFSGIQYEKWITAGLVMPDFKVVIRYPANAFTGITWV    672
GFLV        IYIPKDCQANRYLGTLNIRDMISDFKGVQYEKWITAGLVMPTFKIVIRLPANAFTGLTWV    672
GDefV       VQVPKDCRKDAFLKTLDMRSMTAGFAGIQYEKWITSGLCMPKFEVVIRYPPNAFTGLTWV    669
MMMoV       FNIPKDAKQGTVLLTSNLRAIARADLMPCWWKWWEENLASLEFRFEFELAGSPYSGLALA    678
PCMoV       VKVQKDAKRGAILCSLDLYEEINRYQTARRLDWLRDGLIYPKFKVSIKTTSNQFIGMSIG    684
AVA         FKVDKDAKANTIVAAIDLRLEIETHRTRAWFKWWENNVDFPKFTFKVHTTRNGFIGAAFT    703
PBRSV       FKVPKDAKKGTHLGTLDMAAAINAYGGAHAQNWWAKGVLNPCFTVRLHAPKNAFAGLSIA    644
TRSV        FKVPKDAKKGKHLGTFDIRQAIMEYGGLHSQEWCAKGIVNPTFTVRMHAPRNAFAGLSIA    663
AeRSV       FKVPKDAKKGKHLHTESLADMMRTYGGIHVQEWWGSCAINPIFKIRMHAPRNAFAGLSIA    690
MMLRaV      FMVPKDAKEGAVLSSFNILEEAETFNSQFYNEWFASNLFLGGLNLVCEAPDSKYCGTALL    657
RpRSV       VNVPKDTLGGTLLTRIDVRAKAATFDSAVWRQWVRDGCLKPKIKMRITAATSCFSGIVLG    666
OLRSV       VSVPKDAKQSAFLGTHNFREIAKEYAGEFYNRWVYNRNISPCILVRCHLPKMPFLGLTLG    701
BRSV        IELPATVTPGTVLAVFNIFDKIQETNTKVCSKWLEQGYVSQNLTAISHLAPNAFSGIAIW    907
PoLNVA      IELPATVAAGTVLGSFNIFDSIQAAGTRVCNEWLRDGYVSHNLLMVSHLAPNSYSGVALW    807
TBRV        ITLPATSASGSILAKIDLISLIKNTNTRVCSEWLMDGYVSQNLRAVSHLAPNSFSGISIW    902
AILV        ITYPAAATVGTVIGTLDIFALITTTNSRVCAEWLERGYVDRNILMVSHLSTSPYLGMAIW    905
RCNVA       IKYPKAVSVGTVLSKIDLFASITGTNSRVCAEWLEMGYIDRNLRFISHLSAGPFLGAAVW    912
GARSV       IDLPNAVTVGTVLAKIDILEKIKTTHSAVCAEWVQMGYMDRNLKLISHLAPSQFCGVAIW    914
GCMV        IDLPTAVTEGQVLAKIDIFKKIQDAKSMVCVQWMQAGYVNKNLTFISHLAPSQFCGVAIW    880
PVB         CVMPPEARAGHIMRKIPLMDTFRRVQGSAYNRWLNLGYMDCDIALVSHLAGNVYSGVSVY    896
CNSV        ITVPAASTKGNVLAGVDILANARTTMSGFYMRWLQKGYIDTNLKLICHLPRAPFAGMSFF    753
GNVA        PQFKKDAKAGTLVSNFSLKTIVENFGSQTCEHWKRELSTYPTIALKFTCTGNMFCGMCVG    883
                     .  :    .              *         :          : *

ArMV        MSFDAYNRITSSIT-TTASPAYTLSVPHWLLHHK--NGTTSCDIDYGELCGHAMWFNATT    729
GFLV        MSFDAYNRITSRIT-ASADPVYTLSVPHWLIHHK--LGTFSCEIDYGELCGHAMWFKSTT    729
GDefV       MSFDAYNRITTSIT-TTASPVYTLSVPHWLLFHA--KGTTTCELDYGELCGHAMWFESTT    726
MMMoV       CTIDWYSRLDVGKMGNIMAPVVSTLLPTEVFSCR-AGGMQSYTFSTDEVANYAQATWSAA    737
PCMoV       IALDWFSKFPK--NTGLVPL-VANEFPSLGPIVL-KENEHSFVIDVKETFGTAMSLVVDS    740
AVA         LFVDWWGKLDPAKFTEIPPS-VANELPGPIPITF-RQDLHVFEWDTRKSGGCAFAAVGDF    761
PBRSV       CTFDNYKRIDLATLGNSCPVQEMFEFPTKVFVLK-DADVHEWTFTYGDLTGHGLVQWTNT    703
TRSV        CTFDDYKRIDLPALGNECPPSEMFELPTKVFMLK-DADVHEWQFNYGELTGHGLCNWANV    722
AeRSV       CTFDNFKRIDVAALGGSCPPSEMFEFPTRVFSLK-DADVHEWDIDYGALTGNSLICWDNV    749
MMLRaV      FVFDFYDRLDTTMTSLKLE--VGKHFPHTVHILR-NGDRHVFPLSLKEHFGHALHARGGG    714
RpRSV       ACLDAYRRIPATTKTDFTAS-LVTGLPNIVWATR-DTSEIEWDIDLAAVCGHTFFALEDT    724
OLRSV       MCADFYNRIKPSDMANELPPSVAFSLPHRLMHFNAIDGPVDYIYDVGESCGHSIYVDSAS    761
BRSV        YIFDAYGKIPGDVTTTFELE-MARSFDPHVQVLR-DVSTSTWVIDFHKICGQTLNFSGQG    965
PoLNVA      YIFDAYSKIPSDITTTMEPE-IARSFGPHIQILR-EPNTATWLVDFYKMCGQTLNFSGSG    865
TBRV        YIFDAYGKIPADISTTIELE-MAKCLSPHVQTLR-DATTSSWIIDFHKMCGQTLNFSGPG    960
AILV        YVFDAYGHIPTDVTTTVELE-SIRHLSPHVHILK-DNTTSTWTLNFHREGGQSLNFAGPG    963
RCNVA       FVFDAFGHMPTNVSTTIELE-SVRHLCPHVQILK-DSTTDTWVLDFHRSCGQSLSFSGTG    970
GARSV       YVFDAYGHIPSDITTTIELD-MVRNLSPHIHVLR-EPTSAVWTIDFHRYCGQSLNFAGRG    972
GCMV        YIFDAYGKIPSDVTTSLELE-IARSLCPHVHVLR-DSKTSVWTIDFHKICGQSLNFSGRG    938
PVB         FVLDCYNRLPESLSTETFMS-QITQFPLFIHMLS-DNKTSTHVIPLRKIVGHTLHVGGDA    954
CNSV        VLIDGTGYLAKDAPTSLNEE-EILSYPLHLVTTS-DVSSYEFVLDWHRYIGQVPFAEENA    811
GNVA        ITIDWYNRVDHTKLGGALPATVANQLGTFVCPLK-DGPEFDFRLDLQDICGHAFYWYDMG    942
               *    .                                         .
```

```
ArMV        F-ESPKLHFTCLTGNNKELAADWEFVVELYAEFEA-A-KTFLGRPNFVYSA-DAFNGSFK     785
GFLV        F-ESPRLHFTCLTGNNKELAADWQAVVELYAELEE-A-TSFLGKPTLVFDP-GVFNGKFQ     785
GDefV       F-ESPKLHFTCITGNNKELAADWEFVVELYAEMEP-V-SSFLGRPNFVHTA-NPTLGSFK     782
MMMoV       YDINPMVYVYVFTTNQMVMASDWVVDYRLYMKSQ-KT-TQFLQQPYHVWPP-ALPSTVKI     794
PCMoV       FSEAPKWIFYVLTTNQVVQAHDWFFFVEVSLDYD-IA-DEYRGSPLYQYPI-VEKNNLVV     797
AVA         FSRKPMIYLCVGSTNQIVQAHDWFVGIEVFMESN-PD-TTWLGTPFCRFPI-TYGDYFPI     818
PBRSV       V-TQPKLFFYVASTNQVTMAADWNCVTTLHMVDGDDE-PRFELEPTITWPI-TCRNSFNI     760
TRSV        V-TQPTLYFFVASTNQVTMAADWQCIVTMHVDMGPVI-DRFELVPTMTWPI-QLGDTFAI     779
AeRSV       I-TSPRLLFYVVTTNQVVMAADWNCVVTVHIDRTQQV-ESFELAPTVTWPL-SVGDSLSV     806
MMLRaV      F-CNPRVFVSVATGNQIESFDAWKCTINFMVSREAFG-DQFSLGPVATWPP-APIS---L     768
RpRSV       F-GYMDFLVYVLRGNEVTAVADWSIYVSFHVDWTQES-MSATLIPTFVWPP-EPADISYF     781
OLRSV       F-SEPLFHFFCATSNHLLGAGNWRFLVEYYVYRDSPD-VTFVDRPLCILPL-SPSVVHNL     818
BRSV        Y-CVPKIWVIAASTFQLARSTATKFRLEFYTRGEKLV-RGLAE-QPLSYPI-EARHLTDL     1021
PoLNVA      Y-CNPKIWVVAASSVQLARSTAVKFRIEFYVSGERLV-RGLAE-NPLVYPI-QATHLRDL     921
TBRV        F-CKPTLYIVVASEFQLARSAETKFRLEFYATGERLV-RGLSE-NPLTYPI-ESRHLEDL     1016
AILV        F-MKPKVWIIAASSAQMPCSADVQYVVEGYATGESFV-RGLATEKVLTYPV-ESTHLADL     1020
RCNVA       F-LKPTLWVISASSAQLECSADVTFVLEAYATGDRMV-KGLATDSVLTYPI-GPESLSDL     1027
GARSV       F-CNPYLWVIAATSAQLPWSAPVVYRLEALVTGERYV-QGLATEGVLEYPI-SPKHLRDL     1029
GCMV        F-SKPTLWVIAASTAQLPWSAQVTYRLEALAQGDEIA-HGLATRSIVTYPI-SLEHLKDI     995
PVB         F-ANPMLYVVCGSRASLPISVDGHFDIEFYTSGPLYDNAGFAPDSLLQYPI-TDKSLEDL     1012
CNSV        F-LRPTLFLVACVSSTLALSAKVEFYLEAQSVGEELP-RTLAPSPVLSYPF-QNSFLEDL     868
GNVA        F-SDPRIYVHVISTNEIPMNADWFSCLRLYSKPC-DR-PCYVDRPIFTYPVPTFPEIVKL     999
                    .

ArMV        FLTIPPLEYDLSTTSAYKSVSLLLGQTLIDGTH--KVYNYNNTLLSYYLGIGGVVKGRVH     843
GFLV        FLTCPPIFFDLTAVTALRSAGLTLGQVPMVGTT--KVYNLNSTLVSCVLGMGGTVRGRVH     843
GDefV       FFTLQPQYYALNTASAIKNVALDLGSTLRSGTN--LVYSYNNALLSYFLGFGGIIKGKIH     840
MMMoV       DRWFPPLSFKLGATTRSVQIPLNLARIEETGYG--KCINLISAFLSNFQSIGGRLRCRLL     852
PCMoV       NRVLGNFSVAGN-NPTGELIPLFMARPLETQLG-NKFISPWRVYLEHFMGYSGDFVFEFI     855
AVA         ERNFGPFSIKGS-NPGNSLVRLNWAEKVDTGRG--STISPWRALLEHCQGFSGVLEATFI     875
PBRSV       DRYYEAKEIKLDGTTTFLSIEYNFGGPIKASVK--SSISFSRAVMAQCLGWSGTISGSVK     818
TRSV        DRYYEAKEIKLDGSTSMLSISYNFGGPVKHSKK--HAISYSRAVMSRNLGWSGTISGSVK     837
AeRSV       DRYYEAKELKLDGTTKLMYIDYNFGGPIKLTQK--SSISFPRAVMARYIGFSGSIKGSIK     864
MMLRaV      SRYLGPFTVNSGTSLNDVDFSIALGSVSKFSGG--AIMSFPSAVFSCFQGSSGSLRFVLE     826
RpRSV       KEVWGPYHFTLDGTEAKESFSLMPGMAIPRGAQ--TVRTFPRVLAAHFRSWTGKVRMSIQ     839
OLRSV       DRTLGPFSISIDANSHETYHGIDLGSDLMTDKG--KIANFSSAWFSHWLGFQGILHGRIT     876
BRSV        NLMLAPKQIAVG-TYAMITFPVSLAAKLQSTSG-RTAYSYAAGLLSHFLGVGGTIHFVVR     1079
PoLNVA      NLVLQPRQFALG-TYAAINFPISLAEKQTTASG-RIVYSYAAGLLSHFLGIGGTIHFVID     979
TBRV        DLILKSGSIAVG-TYTMTKVPVSLAMRIDSAAK-RQAYSYAAGILSHFLGVGGEIIFSVH     1074
AILV        DLLLAPQQLAIG-TTATTNFPLSFAEKSITSTK-RETYSYAAGLLSHFLGIGGKLRFAVH     1078
RCNVA       DMVLSPTQLALG-THAATALPLTLAEKSVTTSG-IETYSYAMGILSHFLGVGGTVRFSVH     1085
GARSV       DLTLTPRTMAVG-TQATTNFPLSFATKQVSASK-RVSYSYAAGLLSHFLGVGGILHFKVQ     1087
GCMV        EIMLPPRQMAIG-NAGSINFPLSFAVQQKSSSG-RIAYSYAAGLLSHFLGIGGTIHFKIQ     1053
PVB         DIVLPTRIIGIG-SAAPTSFPLSWALPR-TDHG-FTSYSFGSAILSHFLGVAGTLDFTLY     1069
CNSV        DLFLPPKRLTLG-ERETTIIPLSFAKSK-KSGD-AVLYSHAAARLAHFQGIGGVLHGVVY     925
GNVA        KRWYGPFTLKQGGKDYYARVGLNLAVSSLTTVSKTYAYSTSAAILSHYQGGDGYLKGRVV     1059
                                    .             .          .  * .   .
```

```
ArMV        ICSPCTYGIVLRVVSEWNGV-TNNWNQLFKYPGCYIGEDG-NFEIEIRSPYHRTPLRLLD    901
GFLV        ICAPIFYSIVLWVVSEWNGT-TMDWNELFKYPGVYVEEDG-SFEVKIRSPYHRTPARLLA    901
GDefV       FCGPITYGAVIRIVSEWAGN-TATWNNVFKYPGVNVDSSG-EFEIEIRSPYHRTPLRLMD    898
MMMoV       PTSSIFVGAELAAAITSTGDPVPRK-DLWTMPHVRLDSLGCEFELEIRSSYLVTPLRSKD    911
PCMoV       PCSSAMINCGLRTCMWYNVDTFPSLSEMSFVEHEDLDQ-RKEFTLSVRAPRGKLA----T    910
AVA         PCSSVMVGCKLRAGAWHSATSVPTIGDLCLQEHEDLENESKDFSLRLRSSTNMNQ----T    931
PBRSV       SVSSLFCSASYILFPWAW-DAPPSLHDVLWGPHQIMHG-DGDFEIAIKTSYKSTP---TM    873
TRSV        SVSSLFCTASFVIFPWEH-EAPPTLRQVLWGPHQIMHG-DGQFEIAIKTRLHSAA---TT    892
AeRSV       CTSSIFCTASFVIFPWTW-DTNPSWTDIFWGPHQIVTG-DSDFEICIKTSLHSTS---TT    919
MMLRaV      PTCSLFCTAKFICVLVFGSF-VPSTAQMWKMHHVVVTGGE-VSTLPFDVPFGAVPNVGLK    884
RpRSV       EVSSIFLTGTYMVGVSWN--ATADLTDITTRKHWIVKS-GEVFELDLYCPYGENPTFTGL    896
OLRSV       VSSPITCTSIFLLCMCYKRN-ASAYTSIYRKRHITLEGGG-NFSFPLDSPFSSAPTRYVS    934
BRSV        TTSSAFVTSKLRIALWGT---VPETDQLAQMPHVDVEVNV-DASLQIQSPFFSTA---NF    1132
PoLNVA      VLSSTFVTSKLRVAIWNG---VPSTDQIAQMPHVDVTSGE-PASLVIQSPFYATA---NF    1032
TBRV        STASTFVSCSLRIALWGT---VPTTDELAQIPHVDVTLDT-KATLQIQSPFFATA---NF    1127
AILV        STSSCFVTCKLRVFLWGT---QPTAVQTAQIPHIDIDGAG-TGELLIQSAFYTTA---NF    1131
RCNVA       STSSKFVSCKLRIIIWGT---TPTIAQTGQMPHCDIIEGG-SGELKIQSPFYSTA---NF    1138
GARSV       CTSSAFVSSRLRVALWGA---QITMIQLCQMPHVDVDVGV-TASLKIQSPFYATA---NL    1140
GCMV        CTSSAFVTARLRVALWGD---TITLEQLSQMPHVDCDVDV-VSSLKIQSPFYATA---NF    1106
PVB         VVSSVFTSCKLRVLLWNS---LPSNLFLPRIPHIDVESRTQRMQLRIQDPFVSSS---TF    1123
CNSV        LVGSQLVASQSRISMWSK---EQHIQHQAVNVHVDTDTGV-AFDLPIKDAFYASS---VY    978
GNVA        KIGSGMVSCSILVAIVPTLN-QSDATYVMKAPHVELPMGEGDFMLRTTGFMNAVN---FH    1115
                                                        .

ArMV        AQAASAFTSTLNFYAISGPIAPSGETAKMPVVVQIDEIAL-PDLSVPSF-P--NDYFLWV    957
GFLV        GQSQRD-MSSLNFYAIAGPIAPSGETAQLPIVVQIDEIVR-PDLSLPSF-E--DDYFVWV    956
GDefV       TQVGTN-MSTLSLYAISGPIAPSGETADMPIILEIDEIKL-PDLSVPTF-P--NDYFLWV    953
MMMoV       PKL----HPHLQIYLVSGVTAPKDSSSDFEFAIKIESI-EQAHSPMRVLSD--ETWFCWF    964
PCMoV       SRN----VSKLAIVPLSGVTAPDTMTKDFEYLIKLKRI-DNLRMGPRIF-T--NEWYQLF    962
AVA         GNS----QCYLVFIPFSGVTAPDQHVKDFEFYVRIKGI-SDFVCGSPML-E--SDWMQFC    983
PBRSV       MAG----VGRLGILPLSGPVAPDAHVGAYEFFVHIREWVPDEQIHPPIFST--QDVYNWI    927
TRSV        EEG----FGRLGILPLSGPIAPDAHVGSYEFIVHIDTWRPDSQVHPPMFSS--AELYNWF    946
AeRSV       ETG----YGRLGIFPLGGPIAPEAHTGAFEFIVYISEWKPDAQVHTPMFAS--EEIYNWF    973
MMLRaV      G-------ARFICRPIGGVKAPKDFTGKYECLIHLLGIEG-ESLSRRVF-SDTDDFMSWF    935
RpRSV       VNG----IPYIIVHRLGGIIGPKDSVGTFGFMIHLHGLTGV-YKNPTLHSGDRSVGSAWF    951
OLRSV       TADSSS-GCELYVDTICGPIAPADSVTKLEYFLHFDRLEG-TMTAPNLV-A--EIGSLWC    989
BRSV        GNS----GSAFYVSTLCAPMAPETVETGSEYYIQIKGIEANPGLCREI-NY--KQRFAWC    1185
PoLNVA      GSS----GASFFVSTLSAPMAPEALETAFEYSIRILGVEAAPQLCREI-NY--KQKFAWF    1085
TBRV        GDD----GTAFYISTLCAPLAPETMETGFQYYIHIHGIKVKANLCREI-NY--NQHFAWF    1180
AILV        GDS----GARFWIMPLSAPAAPQTVETKFEFYIRILGIDVIPDLCRQI-NY--KQRFGWF    1184
RCNVA       GSE----GAQFWVIPLSSPMAPAKVESKFEFYIRIHGIDSQPDLCKQI-NY--KQRFGWF    1191
GARSV       GDS----DAAFWVTPMSSPMAPEAIESALEYYIQILGIEADTPLCRAI-NY--KQAFGWF    1193
GCMV        GDS----GARFWVTPMSSPMAPETMESKLEYYIQILGIDADPPMCRQI-NY--DQRFAWF    1159
PVB         GDT----GAQLIVVPLCTVYTPEHVESAFEFGITIHGIVPDSKLCRSI-NY--TNKFAWF    1176
CNSV        GDS----GAVIQVTCLCSPMSPNAIKAPFDMIFKIRGFTPDAPMCRTI-NF--TQRFGWF    1031
GNVA        GTG----AQHLCIYAVSSPTAADRMAAPYEAMIYFDEFIPQRE--LPLINSATESWWAYA    1169
                      :    .                . :
```

```
ArMV        DFSAFTVDA----EEYVIGSRFFDISS------TTS--TVHLGDNPFAHMIACHGLHHGI    1005
GFLV        DFSEFTLDK----EEIEIGSRFFDFTS------NTC--RVSMGENPFAAMIACHGLHSGV    1004
GDefV       DFSAFTVDE----EEYVIGSRFFDISS------STS--TVALGNNPFAHMIACHGLHCGT    1001
MMMoV       TLTDFTEDL----VSLKIPSRIADLEV------KSA--TIMHATNPFSLMVASAGLMAGD    1012
PCMoV       WIDDFKKDD----FKLLVNGYVAEIKS------QDV--KILMNQGIFSHMIACTGFHEGM    1010
AVA         LLTDFTADQ----IDFYFLSQINDNTS------SGC--KVTMAPSVISHLVSSTGLHGGI    1031
PBRSV       TISTVTPDAVSGVWETTIPGYIHDYAD------KNA--VVSLSSNPLSWLVAATGWHFGE    979
TRSV        TLTNLKPDANTGVVNFDIPGYIHDFAS------KDA--TVTLASNPLSWLVAATGWHYGE    998
AeRSV       TLLNLKPDATTGVTNFEIPGYIHDFAS------KDA--DVVLASNPLSWLVAATGWHFGE    1025
MMLRaV      SCGAITKDD----FTLEIPARLRDFST------KDA--TFTMYSNGFSQMVGAAGFHMGE    983
RpRSV       RVTNILDDN----LVFNIPGRIEDMVA------VAGKYEVTNYANPTSMLFSVTGLHGGF    1001
OLRSV       RVTSFLKED----VDIPIRARIYDIAA------KDC--TLELATNPFSKMIAATGFLEGQ    1037
BRSV        LLEC-LDNSKASPIKVKIPSRIGNLSS------KHV--KVTNFVNALAILCATTGMHHGN    1236
PoLNVA      MFEC-IDSTKSDPIDLRVSSRLVNVTS------TKV--NVTNFVNAFAIMCATTGMHHGN    1136
TBRV        MLEH-LDTNTTGMVSLKIPARMANLTS------KEV--QITNFVNALAIMCATTGMHFGK    1231
AILV        MISP-SDK-TTTELDFKIPSRIGNISV------KNT--KCVNFTNAFAIMCATTGMHWGR    1234
RCNVA       MVKP-EGT--DREFQMFVPSRVSNLVI------KGV--DCTNFVNAFAIMCATTGMHWGK    1240
GARSV       TLVR-RST-TNKEIALKIPSRIANIEY------KEA--EVINHVNAFSIMCATTGMQWGS    1243
GCMV        TLLRPPDPKLSKILKLTLPSRVCNIAY------KEA--TVTNYVNAFAIMCATTGMHAGK    1211
PVB         MMKV---NTPSGMTAVDIPARCVNLKH------TAA--TFQHFVNPFTTLCSATGLHGGD    1225
CNSV        AVEP---TTSTGAIKLKIWPVSNHLES------EDM--KVTGYTNAFLQMCQTSTMHFGS    1080
GNVA        TMSDFKSDK----FAISLPTRICDVFPPRTGEVDDC--VLLNHVNSFTRVASSTGFHGGR    1223
                             .     .                    .    :        *

ArMV        LDLKLMWDLEG---EFGKSSGGVTITKLCG-DKATGMDGASRVCALQ-NMGCETELYIGN    1060
GFLV        LDLKLQWSLNT---EFGKSSGSVTITKLVG-DKAMGLDGPSHVFAIQ-KLEGTTELLVGN    1059
GDefV       LELKFMWSLGTT--TYGGSSGSVIFTKLCG-DKATGLDGGSEVIALQ-ELSHTTSMYIGN    1057
MMMoV       CELEFTWDWSMK---FGEHVGAIEFTTGYG-ADEDFQSYGRNLTVPLSQLRWVERYTIGS    1068
PCMoV       LDVRITWSYANK---LGNVEGHITFLHGFC-S-PRKLTGAAAVIANSKGIYEAESINVGS    1065
AVA         VDISITWAHKVK---YGENEGCISFVHGYG-SSPPQISGRAEVINNANGAFQADSMRIGQ    1087
PBRSV       VDLCFSWARTGK---AADQESIVSIAVCYS-DYDKRIRGNTRT-FDVRNTSYEVPYFLGS    1034
TRSV        VDLCISWPRSKQ---AQAQEGSVSITTNYR-DWGAYWQGQARI-YDLRRTEAEIPIFLGS    1053
AeRSV       VDLLISWARSKK---AAEQEGSLSITTNHR-LATDRWQGQARI-FDLRQTSCEISLFLGS    1080
MMLRaV      VELEFTWSLDS---SIAEAKGWISLSTLFG-PVANNFRGHYTVSNCVLPTSKIVRLSVGT    1039
RpRSV       IRLHITWCPNTS---LGESKGTLKYMQYLYHTTTENFFGDQATRGIIDQNGFTVDLACGD    1058
OLRSV       VTLSVSWSTNV---EASKIKGHIIFVVYDA-DLVNSFDGASTVVPMA-KGSFKCLLNCKT    1092
BRSV        CTIHFSWLWHPA--ELGKQLGRLKFVQGMGI--NNEHIGDTMC-YNSLSNTHSVPFQFGS    1291
PoLNVA      CILHFSWKWHAT--EVGKQQGRLSFVTGMGKKTVQEHIGDTMI-FNYPQNSFQIPFQFGS    1193
TBRV        CTLHFSWNWFRG--KLADQSGVFSIQTGMGKSTVAEHFGGRHS-FNYPATNFSLPFQFGS    1288
AILV        CILHFTWSWHRNT-EAGKMKGDFAIQTGMGNSTATHHLGDTRV-FSVYDNAYSIPFEFGS    1292
RCNVA       CIVHFTWAWKHDI-EASKMKGNLSFGTGMGT--KDVHHGETRI-FSIYDNSYSIPFEFGS    1296
GARSV       PFALHL-----DS-H-MTQADDQKICESHSAGITTEHIGDIKM-CGVLATSQVIPFEIGS    1295
GCMV        CILHFSWTLNKGT-SFKDLQGHISFYSGMGDSTIGEHHGEFHL-GGPLSSSLAVPFEFGS    1269
PVB         VVLHFYWSLDKSRKKMSDLTGSVVISSGMGVPT-DFFRGGIQI-FNMLECKASIPIQFGT    1283
CNSV        VIIHFSWTLFGGTTNAATAGGVVTIAEGFGPEE-ENFRGHCRN-LSIYEGRATVPLELGT    1138
GNVA        VRMRIEWSTKEA---FANLKGSIICNYKFG---TSNWLSTSHITSPGVGHVESATVFFGN    1277
                                .
```

```
ArMV        FAGANPNSAL-SLYSRWLAIK-LDKARSMKMLRILCKPRGNFEFYGRTCFRV--------    1110
GFLV        FAGANPNTRF-SLYSRWMAIK-LDQAKSIKVLRVLCKPRPGFSFYGRTSFPV--------    1109
GDefV       FAGVNPNTAL-SLYSRWFAIK-LDKARSMKILRVLCRPIGDFNFYGRTSFRV--------    1107
MMMoV       FSGATTSKAL-TEYSNFIRFR-GSQLKSLRSVRVNVRPLAGFAFYGRSVSPLVK------    1120
PCMoV       MAGPVSGR-D-TDQNKWIEFH-FWKGTQIEQFHIMVRPHENFRFYGDSCIATTIPST---    1119
AVA         VGGANRSG-D-NQLNRWLRLH-FEKATQLSEMRISIRPHDGFSFYGDSCAVPV-------    1137
PBRSV       YAGATPSGPL--GDQNYIRIS-VINAKTLMAMRIGIRPR-SLSFWGRTATLF--------    1082
TRSV        YAGATPSGAL--GKQNYVRIS-IVNAKDIVALRVCLRPK-SIKFWGRSATLF--------    1101
AeRSV       FAGATPSALL--GNVNYVRLS-IVNSKDILSVRVAIRPK-SLKFWGRTATLF--------    1128
MMLRaV      FAGGTTANFK-TYDTNSVKFH-TNIGKYISQINVGIRPLPGFSFYGRSAIIRKNPP----    1093
RpRSV       FFGATRVG-L-KGEVERLGIY-SSNAKSIAEIRVSFEIL-SMKFYGSTIRVK--------    1106
OLRSV       SARGTTSADP-SLLTAWMMIK-VHHAKDIQELRVNVIPQTGFKFYGRSAEAIKVP-----    1145
BRSV        FAGPITSGGKADEAENWIEIQ-SPDFSWVASLHVSIEVHEGFKFYGRSAGPLTIPATVAD    1350
PoLNVA      FAGPVPSHGNPDDAENWIEIV-SPNFSWITSVHVTVEVLDGFKFYGRSAGPLTVPATITK    1252
TBRV        FAGPISCGGEPMAAENWVRLQ-IPNMKWITSLTVSIEVHDGFQFYGRSAGPLTIPA----    1343
AILV        FAGPVISGGTPNEAENWVRVQ-STSWQWIHAVTVSIEVLPGFRFYGRSAGPMTIPS----    1347
RCNVA       FAGPVTSGGKPFEAENWIKIH-TTSWNWIHSIMASIEVLPGFKFYGRSAGPMLKPPESSD    1355
GARSV       FAGPVTSGGTPFESENWIRVW-SKHWDWFTSLSVSIEVLEGFRFYGRSAGPMTIPS----    1350
GCMV        FAGPVTSGGTPFTSENWLRVE-TAHWDWLTSLTVDIQVLPGFRFYGRSAGPLTIPS----    1324
PVB         FSGVAPSTAPNHVHLNWVRFALDGDWEFFNTLHVSVEVLPGFSFYGRTAGPFSPISGAAD    1343
CNSV        FAGPTPLKKLDFKYRNWIRFT-TPKGRNISSIFCAIEVLPGFSFYGRTGSPRLSVVGTTV    1197
GNVA        HTGFTTFAPT--AEEGNFSFK-TTCATSLEWVRISIMPCDGFSFYGRSIAPLITIE----    1330
                             . .        .  .        .: *:* :

ArMV        -------------------------------------------    1110
GFLV        -------------------------------------------    1109
GDefV       -------------------------------------------    1107
MMMoV       -------------------------------------------    1120
PCMoV       -------------------------------------------    1119
AVA         -------------------------------------------    1137
PBRSV       -------------------------------------------    1082
TRSV        -------------------------------------------    1101
AeRSV       -------------------------------------------    1128
MMLRaV      -------------------------------------------    1093
RpRSV       -------------------------------------------    1106
OLRSV       -------------------------------------------    1145
BRSV        VSAVSGS------------------------------------    1357
PoLNVA      STSTSTS------------------------------------    1259
TBRV        -------------------------------------------    1343
AILV        -------------------------------------------    1347
RCNVA       EAVVTESSQKS--------------------------------    1366
GARSV       -------------------------------------------    1350
GCMV        -------------------------------------------    1324
PVB         TKALIKEEMDKRKSRTKKG-------------GSADDTSTS--    1371
CNSV        PPTADASTSNSQGGDEDIGDQYSAALGRGRGRGSRPGPSPIRG    1240
GNVA        -------------------------------------------    1330
```
