# Supplementary material for: Re-examination of nepovirus polyprotein cleavage sites highlights the diverse specificities and evolutionary relationships of nepovirus 3C-like proteases
Source: Arch Virol. 2022 Aug 30;167(12):2529–43. doi: 10.1007/s00705-022-05564-x (PMC9741568; doi:10.1007/s00705-022-05564-x)
Supplement: Supplementary file 3 — Supplementary Material 3 [file 705_2022_5564_MOESM3_ESM.pdf]

Supplementary Material 3 – Alignment of longer nepovirus P2 polyproteins

Re-examination of nepovirus polyprotein cleavage sites highlights the diverse specificities and evolutionary relationships of nepovirus 3C-like proteases

Archives of Virology

Hélène Sanfaçon

Corresponding author: Hélène Sanfaçon Summerland Research and Development Centre, Agriculture and Agri-Food Canada, helene.sanfacon@agr.gc.ca

Polyprotein sequences were aligned using CLUSTAL O(1.2.4), see Table 1 in the manuscript for accession numbers. Nepoviruses listed as subgroup C in Table 1 as well as GSPNeV and GTRSV were included in the alignment. Color lines on the left side of the alignment represent the protease clades as shown in Fig. 3 of the manuscript. Cleavage sites are annotated as follows (P1 and P1' positions). Yellow highlights: cleavage sites annotated in the NCBI accessions, underlines: annotated putative or confirmed cleavage sites from publications (see Table 2 for relevant list of publications for each virus), red letters: cleavage sites confirmed experimentally by Edman sequencing, green letters (cleavage sites confidently predicted), blue letters (cleavage sites only tentatively predicted, when two choices are possible, the darker blue is the one integrated in Table 2), grey highlights (cleavage sites supported by mutagenesis).

```
ToRSV     ---------------------M--------SSICF-AGGNHARLPSKAAYYRAISDRELD    30
AnNVA     ------------------------------------------------------------    0
CLRV      --------------------MV--------KPVVF-SNGESV-LPKA-LISEAREVAAFL    29
SteNV     --------------------MSGVLGGAATPAKCGPSCGVGERVFSKAAVLRACKDGDLT    40
BLSV      ------------------------------------------------------------    0
PRMV      ------------------------------------------------------------    0
CawYV     ------------------------------------------------------------    0
SLSV      ------------------------------------------------------------    0
PVU       ------------------------------------------------------------    0
BRV       ---------------------MSESGNTTSMPGCGRMCALR-STWSKRAFLVACKDGALT    38
GBLV      ---MCGGGTSTQ-----------------LPTGCGTLCGAK-TPYSKRGLAAAIRDGDLS    39
BLMoV     MSFYCPAETCCNYRVPVTRVDAKEDGWRCTAPCCGTLYQRF-AAAS--------SE--RV    49
GSPNeV    ------------------------------------------------------------    0
GTRSV     ------------------------------------------------------------    0


ToRSV     R---------------------------EGRFPCGCLAQYTVQAPPPAKTQEK-------    56
AnNVA     ------------------------------------------------------------    0
CLRV      K---------------------------STRNPAGFWVTFVAQG----------------    46
SteNV     RDA--RCHYCGAMAVVVKMQPQLSSVDARK-----------KQAPMKGVR----------    77
BLSV      ------------------------------------------------------------    0
PRMV      ------------------------------------------------------------    0
CawYV     ------------------------------------------------------------    0
SLSV      ------------------------------------------------------------    0
PVU       ------------------------------------------------------------    0
BRV       SDGRCPQYGCGALVSITKGVQQPKKTASAKVVKCLCWVQPARWCEKHSKGPASPNGSVTT    98
GBLV      GP-EGRCVYCGALAQIEKGAPQPKT---ATGPKCSCWRTAVARCPKHGAQPTP--ARVGK    93
BLMoV     AT-SPAKVGCGEKA-------SLVR---SKGPKCLCWRLPILQCPKHGQKSAP--ASVHG    96
GSPNeV    ------------------------------------------------------------    0
GTRSV     ------------------------------------------------------------    0


ToRSV     AVGRSADLQKGNVAPLKKQRCDVVVAVSGPPPLELVYPARVGQHRLDQPSKGPLAVPSAK    116
AnNVA     ------------------------------------------------------------    0
CLRV      ---TSLSVSQVALCAINGIVC---------------------RQSVESHSNGPSAVAFWS    82
SteNV     --VAGPKKTRSVVRHTPRKFKDLVQKV---ASLELVWPAPVLRQAEEPAKR---AAPLRK    129
BLSV      ------------------------MMTYDPDFAERMISA------MKTDPKGFMAF----    26
PRMV      ------------------------ML----PLHERMILVRQDLEKMGVPEMGILQV----    28
CawYV     ------------------------------------------------------------    0
SLSV      -------------------------MVWSPDKV--------------------LSL----    11
PVU       -------------------------MVYS-----------------STQVPGFLSF----    14
BRV       KRSNSARAAP-APLPYKKQTCDVVVTV---GPLELVYPALVSEELPT-PVA---ATPTK-    149
GBLV      QSAPELKKER-VELPYKKQTCAVVVQV---GPLELVYPALVSEEKEP-RSA---ASPLK-    144
BLMoV     KRQSTPEVDR-VVLPYKKQTCAVVVQV---GPLELVYPPLVTEEKEP-QSA---AVPLK-    147
GSPNeV    -----MIISLSSVCSFAGV----------------ALPGNVMAE---MPS----KFAFSK    32
GTRSV     ------------------------------------------------------------    0
```

```
ToRSV     QTSTAMEVVLSAEEAAITAPWLLRPCKG--EAPP---PPPLTQRQQFAALKKRL-----A    166
AnNVA     ---------------------------------F---PPPLTQRQEFALLKKRL-----V    19
CLRV      ALRARLRSFLKG-----HGRWVTSLLAK-TTEAFEAQDLCRYQRQRTAYLAR--------    128
SteNV     ---VVSEGQGSALPLWVAPSWLVPQ---EPKVAPPQVGRSLTQRQEFALLKKRL-----V    178
BLSV      -----------------------------------------VEKTKAAYAKGGL------    39
PRMV      -----------------------------------------LS------A----------    31
CawYV     -----MQ-------------------------------VSLSRAERHELIRKDL-----L    19
SLSV      -----------------------------------------HAQIRASFAQKNSFSSTVV    30
PVU       -----------------------------------------TGTHV-EFA-QHF------    25
BRV       ----VEEVPIPELPLWLAPAWMV-----EQPYAATPEVLCLTQREEFALLKKRL-----T    195
GBLV      ----EDEYVPMGLTLAEAPEWLAYPLECRPYTNAAPAAKPVSQREEFALIKRRL-----V    195
BLMoV     ----TEEYVPRGLTLEEAPEWLACPIECKPYSQPKKGMVVLTQDQEFALLKKRL-----T    198
GSPNeV    FI--LENGHVVEVR-ASTASRLA----------DAYKALCAKAE-------RRA-----K    67
GTRSV     ------------------------------------------------------------    0


ToRSV     VKG-----QQIIREHIRARKAAKYAAIAKAKKAAALAAVK---AAQEAPRLAA-----QK    213
AnNVA     ALG-----RQKIIAAIRQRKEAQWAKDKARRKAERIAAFK---AAQEAPRLAA-----QK    66
CLRV      --G-----AVLRAKALRKRKTALRK-----ERAAQLAQRQLEGERRAAARLVR-----ET    171
SteNV     LKG-----KRLIREAKGRRIRRSKKL--AAKEAVRVAQRENVRRAVFAVW---------R    222
BLSV      -----EATWAVVP----PTVTEP------------------------LPKVQVP-FVSRA    65
PRMV      -----MGDYAAFVAA---TDEEKEK-ISARQR------------AVKLPISLSSYSERYA    70
CawYV     SKGMPLEGVEKFLSLLRGVSEFREATPE--QQKEILQKED---LTSGEPDLKVIYAERYD    74
SLSV      VSPLPEGDNAISTAVLSPVVPATGALNALREK---LSAEGVVPSKVVLPIVGPSYAARRA    87
PVU       -----MATWGAEIKATMPVVASPEI-LALRAKVASVASTCDISADAHKCANAAA-ESHRL    78
BRV       RKG-----KLLQRRATHARFEARAAL--ARVRAATQRKVEEVTALVIKGRRI-----LAA    243
GBLV      KLG-----KEKMRLAYRKRQDKLVAE--KKARAAYQRKYDALLPLVREARAH-----IEC    243
BLMoV     LVG-----KQRNKLARRQRLDREYDQ--KRARAATQRKVDHLRELVLLGRKQ-----IAL    246
GSPNeV    YLALPAKEQKKIRRARRDRRARQLS---KQRKAAAKASL--IRENRIALKKR-----AER    117
GTRSV     ------------------------------------------------MK----------    2


ToRSV     AAISKILRDR---------------DVAALPPPPPP--SAAR-------LAA--------    241
AnNVA     AAICKILRDR---------------AVAALPPSPPP--SLCF-------TEA--------    94
CLRV      RKGLQILRCK---------------LAALFSPPPPF--PTTE--------WA--------    198
SteNV     RYLLAKLGHS---------------SNGNVAAAPSP--SVVKQWRAQEQRQALLTA----    261
BLSV      TVLRGIVERA---------------IQLASTKI-CS--SVLFQRRVGRAIWALAEG----    103
PRMV      KCMAGIVETA---------------IVRASTKSGPT--RFLFQRRVGRAIWALTTG----    109
CawYV     KVMAGIIGAA---------------IQAASRPSGPN--KFLFQRRVGRAIWAVTAS----    113
SLSV      TVLLGLVERA---------------MKLASTIV--Y--RTLFQRRSGRKVYALEPW----    124
PVU       RVLAGITERA---------------IKLASEKV-YT--GTLFQWRVGRAIYALGDK----    116
BRV       HQLLRELEE-----------------VAPLSQAQEQ--LVASSCAAAAARQEECAS----    280
GBLV      EQIIRDLRAT---------------ERQPLTPAQHE--LVSKSCRLAGEERASMEA----    282
BLMoV     HELLAEVRRR---------------AQAPLTAEQQR--YVTDSCKRAREEAASMQA----    285
GSPNeV    TQILRSLESEPSRAQQKKAIAFFKKVESSLPPKPTEAQCLLAKYRAWRNKQAASAAKAAA    177
GTRSV     --------------------------------ARSKGLKILAAYR---RHQAAATPA---    24
```

```
ToRSV      ----E---A-ELASKAESLRRLKAFKT---FSRVR---PALNTS----F---PPPPPPPP    280
AnNVA      ----H---A-ALKAASLSLPSLKAFHK---ARAAANKAAKLAESFQPNFYSSPPPAVLSP    143
CLRV       ----W---E-SLPS--SPLPTYWE------------------------FFSDATSVARTP    224
SteNV      -DLAVKKVL-SRQGQKE----------------CPHMRARE----------QPARVVPEP    293
BLSV       -VVKY---A-TLLGVRAALAMEEASQW---VPICSSGSYSNVVSSNSPFCTSNW-GVPPP    154
PRMV       -VLKY---A-LRWGITLALALEKGSTW---IPICSEGPYASLGCSNTPISLAPS-K----    156
CawYV      -VLKY---A-KLVGIAVAQRTQWDFVP---IVK---------GG----FIPS-TEVCRPL    151
SLSV       -RVPF---A-RLLGIVVAMRMENAPVA---GP----------VDREVKLLEEPV-E----    161
PVU        -VLQY---A-RRWGITFATRVVKDVEW---VPICGSQKVSGPLDRISTIVGKV----L--    162
BRV        -FLRR---A-KAWRKSI----------------SA--TPPV------AFATAVASKVVSA    311
GBLV       -FLLG---A-KARRHRV----------------CPHVAPRE------EIESPR--VVPSC    313
BLMoV      -FLLG---A-KARRHRV----------------CPHMAPSQ------EVCLPPVPTVRTC    318
GSPNeV     AALEVPKPPPTLDISRAAFPYGK--VEGVP-PP-------------TSPCSATFGSMPIP    221
GTRSV      ------KVEEVTKTSTPSFPMGWRDTEGLSSPPCSP--------LSKNFCSSTFGGCPE-    69
```

```
                                         X3-X4 cleavage site

ToRSV      -----------ARSSELLAAF-------EAAMNRSQPVQGGFSLPTRKGVYV--------    314
AnNVA      -----------ERRSELRKQL-------RQAIPVSFLRRGTVQ--------S--------    169
CLRV       -----------SRGATLLSAL---PEGVDSFLPSSFPRRMTLQS----------------    254
SteNV      TTPWASLGL--SVLAYRPT-PIDGTLGAVCAV----ALQQGFV------RLK--------    332
BLSV       ---SSKMAVVVSAT----TQI---PA--LQEQVEMIPPMGFVT-FKSQGAETPKNDALKA    201
PRMV       ------------VQ----DKI---PV--VQQQVALAPPMGLNL-EVQPQVAG--------    186
CawYV      ---SKNLGIQFSVAGEHDTQV---PLGAQSNLEIQFPVAGEDTQVPLGAQE--------    197
SLSV       --------------------G---PV--DHQFVDMFPHINNYF-----------------    179
PVU        ------------------TPL---ES--LSATSQKVDSVGLVS-QQ--------VDVLSN    190
BRV        TMPWAHLGL--SLGGLLAVPTLDGTLGAKQWN----AKTIATW----VLKPV--------    353
GBLV       TTPWSGLGM--TLTSLRDGGSSDATLGVKSLA----SRQGDYTCNSALGFPT--------    359
BLMoV      TVPWAGLDC--SLTSLRDSGVIDGTLGAKSR-----PMHGDLTCNSSLGLPT--------    363
GSPNeV     -----KDGETFSLPP-TKGWR---TLPMRQRVEKMAKAIGSYTFQL-VG-----------    260
GTRSV      -------GQMISLPSIPFGWR---IPSYKQRFAALDLKK--PARLL-KG-----------    105
```

```
                                X3-X4 cleavage site

ToRSV      ------------------------------APTVQGVVRAGLR-----------------    327
AnNVA      ------------------------------SP-LPSFF-PRSH-----------------    180
CLRV       ------------------------------AQTTKGSF----------------------    262
SteNV      ------------------------------HSCAVAMQR-------GVVHLKNKCA----    351
BLSV       LQIPAHQEQVEMIPPMGFVTFKSQGAETPKNDALKALQIPAHQEQVAVAPPTELVVLKPQ    261
PRMV       -----------------------------EHDTQVVCSFPGHTLEPGC------------    205
CawYV      ---------------------------------IPGVQFQ--------------------    204
SLSV       ------------------LFY-------------------EEEEQERV------------    190
PVU        IHSAVN-----------------------SNTAV------EHTVNV--------------    207
BRV        ------------------------------VSCVQSV----HAKVR-------DWL----    368
GBLV       ------------------------------KSYAVAIMASSLERARKVVRATINFA-RGV    388
BLMoV      ------------------------------PRMAVSVAATSLRRARRVVAATIKFV-KAS    392
GSPNeV     -----------------------------------GYKVLAILKQFRITKTLIE------    279
GTRSV      -----------------------------------GF-FIFLSTAFRVSAAVIK------    123
```

```
ToRSV     -------------------AQKGFLNA--------------VSTGIVAGAR---------   345
AnNVA     -------------------KRASFVAA--------------LKEACVVG-----------   196
CLRV      --------------------------------------------GLV-------------   265
SteNV     --VALQRGVAHLKSRSAPILENSAECQQ-QLVPGWVRDQQWSPAPLH-------------   395
BLSV      EVETSKSGTL--KVSQIPVLQEQVVMAP-PIELVVLKPQEVETSRIDAPKA---------   309
PRMV      --------------------QCSFCAVP-EAQLKL-SVQQGVAGELD--TQ---------   232
CawYV     -----------------------------------------VAEELD-------------   210
SLSV      ----------------TPSLEERYGKFR-PITPML-GPMKWT------YER---------   217
PVU       ---------------QVSILSDGY------------SEENSN------TSV---------   225
BRV       H-SQPEVGVT---------NTKVPLVLP-EVCLGVLSPPSLSEEIV-----DNPQETSQS   412
GBLV      K-TLPRK-MLEVAS---KIKTKVLSVQQ-KVAPEVVDKCTIVPDIGQVPQEVVVKQTNWN   442
BLMoV     L-ALPFLGLSEVVS---KIKTKVLSVQQ-GVAPAVVDNNTFIPE-------EEVKQTSWS   440
GSPNeV    ---------------SVTGLLDQFLSDIDQSSSFWIDYITPVQSTGTLKAV---------   315
GTRSV     ---------------GITSMADSFLDAMDCTDTLWLEFVTPQATSCVNKAC---------   159

ToRSV     ILKSKSQ------NWFRRSMGIA-HDYVE----GCMAS-TV-LGCAGPVVQRQEA----C   388
AnNVA     ---LRAH------QWVVEA--------------------------------PVRL----C   211
CLRV      ------------------------------------------------------------   265
SteNV     -----------IGRYRQISFATNVRTDEP----GVPFWVLN-GSWIPPCLHIGRYRQISF   439
BLSV      LDVVPPMAFGNSVSIQDETWGPE-EAYLP----FEIPI-SK-ANLCGPCIHVVGLPTT--   360
PRMV      LENLP---------VQQEVAGEL-------------DT-QN-SNLL-VQQEVAGEPDT--   265
CawYV     -TSVPNV------DWGP-----A-EAYLP----FEINF-SK-ANLCGPCVRAVGVPSFYG   251
SLSV      KELLPPPST-------------------------------I-EDLCT----LHGAFLS--   239
PVU       VEIPPPHSYILQYS-------------------------------C-----DDGIGDI--   247
BRV       GIWHPEMGVRNIYVFHDDSWETSPEEDEN----YTYTFS----RQCGIPYLLVEGRGAE-   463
GBLV      GIQLPEMGVRNIYAFHDDSWSASPEEDEK----YTIPWS----NRCGPCVHAVIPTTKV-   493
BLMoV     GIQLPEMGVRNIYVFHDDSWDASPEEDDK----YTIAWS----KRCGPCVHEVVVPKRQ-   491
GSPNeV    YNDFPAI-DCSSNG---------------IMYPKVIQPSDPEGCDCDKCL----------   349
GTRSV     VSIIPSS-NPSGLGWYGKYWGSFEDHNERPFSYYMARRSDKEGCDCKDCI----------   208

ToRSV     SVVAAPPIVEPVLWVPPLSEYANDFPKLTCSTFTEWQRPRKQ-----SIAIS-NLF--RK   440
AnNVA     SHIVGKGFEVLKSWWNKDRQPRSARRRPPCQTFKEAFAKAAADEADDPLVLHQEIK--KE   269
CLRV      ------------------------------------------------------------   265
SteNV     ASNVRTSEPRIPQWVLDGT----------WNP----------------------------   461
BLSV      ---------PLSGWSS--------------------------------------------   367
PRMV      ---------YIL------------------------------------------------   268
CawYV     RFDIGDGLIPPPTSWQ--------------------------------------------   267
SLSV      ---------MIQAWIHMESQG---------------------------------------   251
PVU       ---------PISGWACVNAIPASSL----CNTQR--------------------------   268
BRV       -----ERKNTILGWDFSLH----------NDG----------------------------   480
GBLV      -----PYD-D--CWYMASS----------CRTVR--------------------------   509
BLMoV     -----LYD-DSDSAYMAIS----------DRDLR--------------------------   509
GSPNeV    ----------SSDWISYITPTTSQS-----GTILGVF-------NDFPIIENKEIINYKI   387
GTRSV     ----------F-SWDNMISPV-QRT-----GSLRAVF-------NDFPVVSAN----YRK   240
```

```
ToRSV     LIDRALLVSGVSLI--ASVLLFEIAENFAVRQAVCPVEMPSCATSVSEKSLVSLDEGNFY   498
AnNVA     DLDPPTII-GV-----------------------APLVLH------QETVVEDLDSQQII   299
CLRV      -------------------------------------------------------LRRMI   270
SteNV     ------------------------------------------------------------   461
BLSV      ------------------FCG---------------------------------------   370
PRMV      ------------------------------------------------------------   268
CawYV     ------------------------------------------------------------   267
SLSV      ------------------------------------------------------------   251
PVU       ---------GNENY--SSLLG---------------------------------------   278
BRV       ------------------------------------------------------------   480
GBLV      ------------------------------------------------------------   509
BLMoV     ------------------------------------------------------------   509
GSPNeV    DCFFVLSIDGEDIHVTTEFLE------------------------------------EEI   411
GTRSV     GIMYPKA-----------------------------------------------------   247

ToRSV     LRKYLSPPPYPFGRESFYFQARPRFIGPMPSMVRAVPQIVQQ-PTMTEELEFEVPSSWS-   556
AnNVA     LDESLRWRPLA--LPAKWVGRYDKFVKPQRVCLPDEEMDLLD-LFVEQDPEPPVATWWS-   355
CLRV      LKAVAAQRSFLAAFYNYFFKR-EDVIAEEKSS-------ISE-VPAEDDPYAGFD-HWGA   320
SteNV     ----------------------------SYLHIGRYQQISYASN---VHS--SLPAWAKS   488
BLSV      -----------FENP-PLPVTDKECFSLYSACIALAMQVE---LSLDNCPAFDFCGWDEP   415
PRMV      --------------------------------------HG---LSVGHFPCSYTCG----   283
CawYV     -----------------------DILALSNLCISL-----------EEADIEEFDAWLEN   293
SLSV      --------------------------------------------IDSLEEYYADDGWETS   267
PVU       -----------FEPCAPWFVTTEEHTALEQAIIALVQSE-----QDSLASEIEACGWDAA   322
BRV       --------------------------EF------EFLPSPEEGYTKELVTPVALEEE-DK   507
GBLV      ----------------------QGIRELEKAIIGLYLGAKEERA----------EEL-AR   536
BLMoV     ----------------------RNLRSLELAMVGLFLEAKEARGAVERINTTKLEVV-EK   546
GSPNeV    CDKYGALRPCT---------------------------------------CEEC------   426
GTRSV     VSSAADEEGCT---------------------------------------CDEC------   262

ToRSV     ------SPLPLFANFKV-----NRGA--CFLQVLPQRVV------------LPDECMDLL   591
AnNVA     ------SPLPLFGDFRV-----NRGA--CALQVMPARPV------------LPNWQMLPA   390
CLRV      YSAFLSSVLP-FPTLRV-----GKFHSAGNYQYCARAST------------CAE--MDMH   360
SteNV     ----------------------KSLLQDCQLSGPSNHLG------------EE-------   507
BLSV      -------SLSFIPTA---------------------------------------------   423
PRMV      ------------------------------------------------------------   283
CawYV     KRAIESERLVVQPQVAAELDTVEFLDSNLNLVVQPQVAV------------ELN-TMESM   340
SLSV      -------SDQDYEED---------------------------------------------   275
PVU       -------PKSKSRTS---------------------------------------------   330
BRV       ----------------------YSTASSCGFF----------------------------   517
GBLV      ----------------------QTKEPEVQLEVQRETVV------------KQI------   556
BLMoV     ----------------------QTKDPEVQLQVQPEVAD------------DQT------   566
GSPNeV    ---CINTPCPIFGQFKGVCKCAYCSISWLNYASPVHSTGSLKAVYNDFPIFTPNIVEGFS   483
GTRSV     ---L----------------------SAFAWMSPVQRTGSLKAVFNDFPVVSANYRKGI-   296
```

```
ToRSV     S--------------------LFEDQLPEG-PLP------SFSWSSPL-PLFANFKVNRG   623
AnNVA     PRVLHSKVQEQRLAVVPTIPLVLHQEIPKEDLEPSFQY--PKWWSTPL-PLHSSFSVSTA   447
CLRV      LHSLFEECKE----------------CRMGAGPPSQGF--SPLF---------REPLNRG   393
SteNV     ----------------EEVA---------ELAE-GSET-FYD---AETPTEEEHLFLDQK   537
BLSV      -----------------------------------PSLEAYASFSYSFTPDFQEYYKNFL   448
PRMV      --------------------------------------------CSLYTSPFEEYYLN-F   298
CawYV     DSKLNPVVQPQVAAELNTVESMDSKLNPVVQPQVAAELNTVESLDSKLNPVVQS-QVA-A   398
SLSV      -----------------------------------EEDEEEE-ECV---VAHDEYY-ATA   295
PVU       -----------------------------------EKKDKYIRPVMALN-IFQSLPKEEI   354
BRV       --------------SLDDVSSAITIQCPGLLSADADVH----------------------   541
GBLV      --------------NEPCVQRVVLVESPEQIAERARQT-AYE---AEIAAQRRRQQID--   596
BLMoV     --------------KVPVVQQLVQGESPEQIAERARLS-HLE---AEMAAQRRRSMMS--   606
GSPNeV    SRLRK----PWSVHVIPDEEGCDCNDC------------LSSSWEEYVSPVHST------   521
GTRSV     ---LY----PKAVSSVVDEEGCTCDEC------------LSAF--AWMSPVQRT------   329

ToRSV     ACFLQVLPQ------------RVVLPDECMDLL---SL---FEDQ--LPEGP----LPSF   659
AnNVA     CLQLQVMPV------------ATVLPECEMPLE---SL---FTDS--VDIPH----IPAW   483
CLRV      RLFLAALPE------------RLVLPDLQMHLH---SF---FDEC--TCYAG----HVAD   429
SteNV     ECYVK--------------LREAFLEG-VAELT---------------------------   555
BLSV      FCGPFENPSFYEGPIGPLSFGATIMASH--------------------------------   476
PRMV      FCGPFENPRFYEVPIGPLTFGATTLAQH--------------------------------   326
CawYV     ELDTVESPD--------SKLNPVVQPQVAAELSTVESLDSKLNPVVLIDLPQ----GSGW   446
SLSV      FCGPFNNPQQYAYAIGPFKPNRMS------------------------------------   319
PVU       PV--VKQEVCILANVQPERVSNISIAHS--------------------------------   380
BRV       FFDGPG----YRCSSRPRDFRPPVVRGCDYESR---------------------------   570
GBLV      FWYIPL----S----MKMLLKEPEMQSEVQRVD---------------------------   621
BLMoV     DCFGF-----Y----PEMDINVPVVQQQVAPVK---------------------------   630
GSPNeV    -----------------------------------GSLKAVFNDFPEFQPSFVTKFGSRF   546
GTRSV     -----------------------------------GSLKAVFNDFPVVSANYRKG---IL   351

ToRSV     --SWSSP--LPLFASFKV----------------NRGACFLQVLPARKVVSDEFMDVLPF   699
AnNVA     --VWSFT--STPVLCVPRRE-TFYPLCDPWNKWLHEVHCLPSCMTVYELDSIE-MPTLDS   537
CLRV      TLNYRVP--STRDCAVV---------AKRTGVAIKHVHALLSCWDRIL----E----IPS   470
SteNV     -----------------------------------QNKCLQ----I-----------LGR   565
BLSV      ------------------------------------------------------------   476
PRMV      ------------------------------------------------------------   326
CawYV     NCYRAINTNSKFPFSLRGEENYFEFYSDPWPITTKEFTDLFISLDALS------------   494
SLSV      ------------------------------------------------------------   319
PVU       ------------------------------------------------------------   380
BRV       -----------------------------------VKA-------S-----------IQR   577
GBLV      -----------------------------------V-----------------------V   623
BLMoV     -----------------------------------EDK-------K-----------IPE   637
GSPNeV    YTKWVSNALS-----------------DGEG--CTCDECNSSLWI-------EYITPVQR   580
GTRSV     YPK-AVSSVV-----------------DEEG--CTCNECLSAF---------AWMSPVQR   382
```

```
ToRSV      L--------FSPLVSHQEEEPEMVPAVLEAADSVGDITEAFF----------DDLECESF     741
AnNVA      LFS-----DPPPLVSHQKVQQQVLDLAV------SDFDREVFDSSPHPYIQDDDEDPALF     586
CLRV       --------VRPPLFERGWRK-SVKSI-C--------TAHALWAQSQVSSCEPMDMDVHSL     512
SteNV      QL------CKGPALFPP-----R-----------------------SLFQRCSHRPVKVE     591
BLSV       ---------------------------------------------------SLDHDCDLS     485
PRMV       ---------------------------------------------------FLSHDCDSS     335
CawYV      ------------------------------------------------------------     494
SLSV       ------------------------------------------------------------     319
PVU        ---------------------------------------------------ALEVPTTLS     389
BRV        K-------IENPLQERF-----I-----------------------TVLREKRKKNKKKE     602
GBLV       KE------TKEPDVQHQ-----V-----------------------AP-TAEDKQPQPLD     648
BLMoV      VV------TKEPVVQPM-----V-----------------------AP-EQEDKFVSHEE     662
GSPNeV     TGRLHNNYIGVPLVVEGIYTSS---------------------CVRRVSADAEGCSCTIC     619
GTRSV      TGSLKAVFNDFPVVAANYCKGVL------------------YPKVVRPIADEEGCDCDDC     424
```

```
ToRSV      YDSYSDEEE-------AE-----------------------W---AEVPRCKTMSELC-A     767
AnNVA      FDSFDSDED-------CE-----------------------AASYEPNYSCSHILRVV-E     615
CLRV       ---FCECAE-------CE-----------------------INSSHPPLGSKSI------     533
SteNV      E--YKYLTR------FGDND-TLQH--------------------GTCAWCREKG-----     617
BLSV       FSSLSAT-----------AG-ILHSS--------------IEVSAKLGSWPKRDFSHSPA     519
PRMV       FRSYSPIGG------------LLFSS---------------IEERSLGSWPKAAFSFDDA     368
CawYV      ------EGK-------DE-----------------------WECVEKPTWPKHPFSFNIN     518
SLSV       RESFSPILS------LID--------------------------TIFPQIKEDDFIFRVS     347
PVU        VDSSELVSQ------ICDDQ-ILHQAPLCLKFEKVITTLTVDSSELVSQICDDQ------     436
BRV        FHSFSACFA------FKRK-----Q----------------------IQWPPTPNEMVNE     629
GBLV       EEELVDCES--------DD-----E--------------------PDFSWVMDKYGITRP     675
BLMoV      EDSFEECEN------FDEE-----E--------------------SDYEWVKQKYGVNRP     691
GSPNeV     SAPKVVEIVKQEPQQGCTADCLYRECRM---------------CDNAKICCDINARIQAE     664
GTRSV      TDQFEE----------CVED------------------------LEEESWLDYVSPVQRT     450
```

```
ToRSV      --SLTLAGDAEGLR--KSHGVFLK------------RL---------------------V     790
AnNVA      DAIRSIKFDTCGMR--KGHVALFQ------------SM--------------------LK     641
CLRV       ------------------------------------------------------------     533
SteNV      ------------------------------------------------------------     617
BLSV       LLMKGES--FCGFK-SLPWPITTKDFDL---------LMGALD--ALHETPQQGTWPCGG     565
PRMV       LLLKGSV--NFNYLNSDPLPMLDREFNS---------LFLALDSLSESEQPSQGHWPCGG     417
CawYV      LLSKKRKGDNSVLQ--KPKSIGIEAFE---------ALSYALNSLKKEEEPLTGKWHCYK     567
SLSV       V---------------------------------------------EKRIP---NW--GP     357
PVU        ILHQAPP--CLKFE-GVTTTLTVDSSELVSQICDDQILHQVVP--TLKETVSTTV-IVDG     490
BRV        ------------------------------------------------------------     629
GBLV       YLTMTP------------------------------------------------------     681
BLMoV      YFCSSQ------------------------------------------------------     697
GSPNeV     ------GG--EIFQ------AALKWYDLRH------LATEELKYHGKHAAWRARHWHSNS     704
GTRSV      ------GR---LI----------AVYNDYP------VLRAEYRYQGRFILPL--------     477
```

```
ToRSV     TYL-QSFEEPLY-SSRAFY-SVKVK----P---------VYR---------PKK----FE   821
AnNVA     HFS-DDVEVAGF-SSRSLF-ISP-----------------------------RR----AV   665
CLRV      --S-LSGPPTLF-SSRPRY-SLPKYAGNSL---------VYT---------PDK----CV   566
SteNV     ----------------TYYSLV--------------------------------------   623
BLSV      TTSFNPSYTPRFKRGDENY-SLPSNWKGKYSASNEALDRIYTRISEWGVRVNPK----SG   620
PRMV      VNSFKSSFTPRFKRGEENY-CLPKMP-----------PRILK-----KEAINSL----SG   456
CawYV     EIK-ATKPTPRYKRGNENY-ILPPK--------------VY----------ERD----CD   597
SLSV      FHGLPKTIPYHFSRGEENY-FFPARPKVIY--------------HEWELRNFEKCPWLHH   402
PVU       IAPVWQNLKPFV-SNSDLY-------LKRTNGSLPTMPRVLMSAKE------MR----DG   532
BRV       -----------------------------W-------E----------------------   631
GBLV      --------IKRE-SRGAYFANLRVGSHAFY-------IEQCR------RRNKLS----RE   715
BLMoV     --------PKRA-KGAKIFSCFPVNSHAFY-------LELYR------RRNKLR----CN   731
GSPNeV    ----------------ELFK-------------------------------QLKCPEASF   717
GTRSV     -------------------A-------------------------------PIPVPE--R   485


ToRSV     -GHIDCTC-------L--D---GNM-------------------GEWEW----------R   839
AnNVA     -GTVENITTW-MPRPT--W---DQF-------------------AAMRW----------Q   689
CLRV      -HCKNEAE---LPVSV--A---ERE-------------------AYLTI----------T   588
SteNV     --SNKCYCWYPSPSIVLAT---HSAPEFYI-----------APPLFRKYNPRSIIP----   663
BLSV      -HFIACKGACCIPRCV--S---YEH-------------------GWICGQD--------V   647
PRMV      -HFSFCECARCIPRCV--D---NTH-------------------AYLCGKA--------T   483
CawYV     -QHREIECRYCFPRCPIEM---HQM-------------------LRAKWNP--------I   626
SLSV      -QMMACDGFR--------------------------------------FVD--------R   415
PVU       ---ETCK--SALHECL--A---HRD-------------------IWT---E--------R   552
BRV       -----EYC------------------------------------IAQAWLPFEVVVTDEI   650
GBLV      MCMVKRTCALPQTFRYI----------IRP-----------KDPDRREYELFQTMVTKKI   754
BLMoV     QQALKRTCPKPIVNRTI----------FMA-----------KD---TEYIPYQKMVTDKV   767
GSPNeV    -----MQVYNLCL-YEESWIPTGGLIDFFPGYLSSMGVKGMPDLSTWCRQKRSTKYTFEP   771
GTRSV     -----CSCYNCYMVSLTDWV--------LPKFKSPIGDKRIG-NPDWEKQPSGRMVCIN-   530


ToRSV     ESVD-AMWRCPGRLLNTKR--------TFTRDDW-------------------------E   865
AnNVA     QKID-LKWSRQPLILP-LH--------NVHRMEW-------------------------D   714
CLRV      GKYG-VCLCGGPRIVKTLQ--PSSVYTYAKRCGW-------------------------K   620
SteNV     IEED-YTWFGIIYHPAGTW-------GDIS------------------------------   685
BLSV      LYAH-DCWICAIEALNATG--------KQDRKER-------------------------K   673
PRMV      LYAH-ECWICPILALSKVA--------KQERKER-------------------------A   509
CawYV     LLAS-DCWLCPIEALVDVA--------EMEQDER-------------------------D   652
SLSV      FFVF-QCYWCSFEALKHQG--------KVEQAVR-------------------------A   441
PVU       FYLS-QCLYCAFDALQQWG--------IEEAQLK-------------------------A   578
BRV       EDV------TPL-YPGGRD---------YNC--N-------------------------S   667
GBLV      AANE-LEVRVPRYNPDGIG-------TRLDI--D-------------------------N   779
BLMoV     AAHE-LEWRCVQYNPDGIG-------TRLDI--H-------------------------N   792
GSPNeV    YTKNLNGSFCKRLLVSQRKIKSNVVIGKPIVRTHEQIVADAAKAAEVAKGSQKPKPIVRT   831
GTRSV     -------KNCR--------IALNPVTGR-----REPFQEDKR-----------------T   553
```

```
ToRSV      RVQYLR--------------IGFN-EGRYRRNW-RVLNLEEMD---LSLHEYPEIS--S-   903
AnNVA      RAPYRA--------------LPCV-ETDHMLAFFRACDAL-DS---AGFVEEPQIE--H-   752
CLRV       FLNPVR--------------YHYATYEEDYLCFMRALDALQAS---FVI-EQPRSK----   658
SteNV      ----------------------------------QYCEVE-----------EPEIENSS-   699
BLSV       L--FA---------------ALL--E-HTS--------IVGSA----EIVKEPV----P-   696
PRMV       K--FE---------------ALA--KHLDELDFCDQLNSIDKA---AMKLETAA----T-   542
CawYV      D-QRFR--------------LQCL---------QKHIDKLPDL---PGSVVARKLETAT-   684
SLSV       H--RI---------------AHF--NKQFE--------EPPPA----EIKKGGP----S-   465
PVU        W--RV---------------ADF--NSQFE--------KLPAA----EIEKAGV----S-   602
BRV        QLLFPL--------------APLS--TVYC---DDSCFHP----------------NDG-   691
GBLV       HFIYRA----------------------EC---LEDCQWNES-----TV--QAPIENCT-   806
BLMoV      HFIYVA----------------------EF---LEGCDYGVR-----EAPAVAQIEQTA-   821
GSPNeV     HEQIVADAAKAAEVAKGLQRPKSIVRTHEQIVA----DAAEAAEAAKDVQRSKPIIRSHE   887
GTRSV      GKKYF-------------FDPLYV-------VW----ELPPTKEELKEIQRLNIIKR--E   587


ToRSV      -------AP--VQSSLFSRVVDR-------------GAT---L--ASSIPFV---TRSNC   933
AnNVA      -------PQLAASKTLYSGRIRR-------------PSM---ARSLAAIPLLS-VARQCI   788
CLRV       -------IE---IRGLLRYVVRA-------------PAP---PIEDE-------------   679
SteNV      -------FFSR----------VW----NRVVVRPDGPNT---VRIPKGTLR------LTT   729
BLSV       -------EK--PSGGF--------LGCFYATKHEVAPKK---NSLSTIATLFAAPALAFI   736
PRMV       -------PK-QTYGGLFSGVAKHI------------PSK---VSLVDVATFIAAPAFAFI   579
CawYV      -------PFKESFGGLFKAVSSHI------------PSP---YRLGGVAAFIAAPALAYC   722
SLSV       -------GTIAQVGMVFGGKA---------------------ITTIRNTVEFATCKYVNA   497
PVU        -------KS-SLFGGLFDASTRHNVWCEYVHAVDYVQWRA--SQAKKLLMDRNPNDITYC   652
BRV        -------WTTDGNGKHFRLSPQF---VLPDVPIPIVHRV---TRQLPQFLYDLGIGDLTC   738
GBLV       -------PMDDKKGGHP-FV-PF---SLPIQMRGDFRSG---GRLLRSLLNFTPFGDLTC   851
BLMoV      -------LTANSRGNMP-FVDPF---TIPIQERGDFRAK---GSALRSLLRYTPWGDLTC   867
GSPNeV     QIVADAAKAAEAAKSLPRLDISTSR-QTIMVKREVEPVVLKEITMDQALKMIRTEPTKWS   946
GTRSV      AILV------EQRKEVQRLQLERA---PLDRQRE------------EDL-RICLERARWN   625


                 X4-MP cleavage site

ToRSV      QSSLGTPGLNVHT--------------IHQ---EAPTTLRAPPFTGARNVMGS-SDAG--   973
AnNVA      QSRMTDPNQGVPSTVVAPPEVAQFSSVLRE---TEDSRPPGPYFSGLGRQIGSDVGAA--   843
CLRV       -----DLFLDAPSYLPAQSGIGANVQTLHQ---REPSGTEQTPFQGASRPLGASDGAV--   729
SteNV      HSGIVDTGRE--------------VSEVQ-------------------------------   744
BLSV       HSEREEAETSAKL---------------Q-------------------------DK----   752
PRMV       HSERESEERAADL---------------E-------------------------DH----   595
CawYV      HSD-----------LERNNEIGGATQTVVLPPPSNKVAGPSTSFPGGGRTLGMSDGAG--   769
SLSV       HSGREDEEMRSNL---------------H-------------------------DR----   513
PVU        HSEREDGETSAKL---------------Q-------------------------DK----   668
BRV        NSG-YQAENL--------------QEEIQ-------------------------------   752
GBLV       NSA-LGE-----------------TSEIQ-------------------------------   862
BLMoV      NSA-AGA-----------------TSEIQ-------------------------------   878
GSPNeV     FSKLDQAGYV---------------VKGEE---TFKRIDRYLPFSHTYSSMPIPN-SASE   987
GTRSV      NSST----YS---------------SMVPE---TLSNEHLAQGVSQTYSSMGAEKPSDQE   663
```

```
ToRSV      --AN-AAPYRSEARKRWLSRKQE--DSQEDNIKRYADK----------------------    1006
AnNVA      --ST-SAPYQQEARRKWWSSRQSNIEKQEDRIRRLADK----------------------    878
CLRV       --AT-QAPFRQEARQRWLGRRAHDLESQEDRIRKIADS----------------------    764
SteNV      --------ERMQDRDADAP--VGSVDQLVSVLQK-RSAKVAGAGENRVADKK-VLTEKMV    792
BLSV       --------ADEEHDKYWS-----SVESVKSKLKAKKGKKVPNSAEGMLGDKKTKLVERDV    799
PRMV       --------AANVHSEHGSLTIAQSITNLRKKKRKNKKGQILEYGEGRLADRNTKILEKDV    647
CawYV      --LN-VAPYQQDARSRWLSARQNNVESQRDRISRYADM----------------------    804
SLSV       --------ASSEIAEHGAPTISNAIEVLRTRKRS-KNNKTLVHGEGRFGDENTHITEKDV    564
PVU        --------AEEDHDKYWS-----SVESVKDKLKRKKGKKVPNSGEGMLGDKKTKLVEKDV    715
BRV        --------ERMEDRSEEKP--VPSLDTLISKLSK-RSTKVKGAGENRYADRH-SLTEKAI    800
GBLV       --------SGCKTVM-WMP---FHQYTLSRQLAT-RKSKVSGAGENRVADKK-QLTERMV    908
BLMoV      --------ERMQDRD-ADT--ISSIETLTAKLAS-RKSKVSGAGENRVADKK-QLTEKMV    925
GSPNeV     LHEEAQLASDMIERNYTLLTPQALIETALSK-----EVRSFKIGEGKILGKT-RLKPEDV    1041
GTRSV      AYENAEIAREVIERNYNLLTPETLIATAFGK-----EQRGFKVGEGKILEKA-TLKAQDV    717

ToRSV      -----HGISFEEARAVYKAPKEGVPTQRSILPDVRDAYSARSAGARVRSLFGGSPTTRAQ    1061
AnNVA      -----HGLTFEQARGAFSGAQSAIAAQSPLLPELRKIYSRR-------SRFNSAPSTRAQ    926
CLRV       -----QGISYASARAAYGAPDEAVPSQAPILPRLDEAYTRDSVVP--RFLLGRTASTRAQ    817
SteNV      FHHP---GIV----QKLRDKGGNTKIAANLNSDRVLVRMQP-------------------    826
BLSV       FAKVVHGSTMDRLKNRFISDGSMILTDQTFPLKDEEVRLGST------------------    841
PRMV       FVRVAHGGWADKAQKWLGSNSAGILTQQTFPTRAETVYMPSD------------------    689
CawYV      -----HGLSYEQARSAFNGATEAAPSQAPLLPKLSQAYRRR-------SIFGGGPSTRAQ    852
SLSV       FEKVVHNNMSRRVLSKLGAPDPKILTQNTFPTLHETVFMPQN------------------    606
PVU        FTKVVHGGHFDKLRHKFTSDGSMILTEQTFPLRDEEIRIGTT------------------    757
BRV        FHQP---GAL----SRMRSGKEKTIVAANHNSDQISVRMAE-------------------    834
GBLV       FHQP---GVL----SRMQNQ----------------------------------------    921
BLMoV      FHQP---GVL----SRMKSKGHKTITAANLNSDLETVRMQK-------------------    959
GSPNeV     FYIE---SFMEKLQRGLGN-GIKPES---------------------KI-------TFNQ    1069
GTRSV      LYIE---SFLDKAKRGLGIDHFKPES---------------------RY-------TFGE    746

ToRSV      RTE-DFVLTSPSAG-DASSFSFYFNPV---SEQ------EMAEQERGGNTMLSLDAVEVV    1110
AnNVA      RTV-DVVLSNPDLDEDGSTACFYFNPV---SQQ------ELSEMRAKGNTMLSLDAVEVS    976
CLRV       RTV-DVVLASPSVDKENHTATFYFNPV---SQQ------EIDRMKSSGNTMVSIDAVEIA    867
SteNV      ------------------QGKPTFTPLPRMTEDQLRKLAEKGFGST-A--SVALDLGIQS    865
BLSV       ------------------VGCPIYTRLPTFTEKELRKLTDKWEMSNTGVVALD--MAIQS    881
PRMV       ------------------IRQCIHTPLPQFTENQLRHLLDVQDMKSSGITAID--LAIQS    729
CawYV      RTV-DVVLQAPSADESTASSIFYFNPV---SSQ------ELAQMSSGGNTMLSLDALEIS    902
SLSV       ------------------RKKCIYSPLPVFKEDELRALLDKHEMKSSGITAID--LAIQS    646
PVU        ------------------KDCPIYTRLPTFTEAELRRLLDKNAMSNTGVVALD--MAIQS    797
BRV        ------------------CGKPVFTPLPRMSDEMLRKFLEKGLGST-S--TVALDIGIQS    873
GBLV       ------------------------------------------------------------    921
BLMoV      ------------------KGQPAYTPLPRMSEEMMRKLLEKGIGST-S--SVALDIGVQS    998
GSPNeV     TTNFEVVRHPGNKEN-GKLSQQIFSRLPMMQKNQARKLLEKGIVSSE---RTAFDLGLIS    1125
GTRSV      TTNFEVVRHPGNKDNTGNLSHQVFSRLPMMHKKQARKLLEKGVVTSE---RTAFDIGFLS    803
```

```
ToRSV     IDPVGMPGDDTDLTVMVLWCQNSDDQRALIGAMSTFVGNGLARAVF--YPGLKL--LYAN    1166
AnNVA     IDPVGMPGDDTDLTVIVMWNQNSDPQRALIGAMTTFVGNGLARCVF--FPGLTL--MHQH    1032
CLRV      IDPVGMPGDDTDLTVLVMWCQNTDPQRAILGALSTFVGNGLARCVF--YPGLKL--MHQH    923
SteNV     HIPQGMPT---VAFMNVMDTRSEKPEYASLCGSYV--DLGRDRAKTLCLPLANFPLNKSL    920
BLSV      HVPEGTPM---VAFATIMDGRTDDPHVAAQCGSYF--DLGRGRCQALSLPLVNFNLNDLC    936
PRMV      HAGEGIPL---SAFCTIMDGDNGDPNMAALSGSKF--DLGRDRCQVITLPLVNVNLNNLH    784
CawYV     IDPVGMPGDDTDITVVVMWNQDRDPQRALLGSMSTFVGNGLARCVF--FPGLKL--MHQH    958
SLSV      HIPEGHPA---VAFCTVMDGDCGNPNLAALSGSKF--DLGRDRCQVITLPLVNVNLNYAL    701
PVU       HVPEGTPM---VAFSTIMDGRTDDPHVAAQCGAYC--DLGRGRCQILSLPLVNFNLNELS    852
BRV       HIPQGMPT---VAFVNVMDTRIEDPLYSSLCGSYI--DLGRDRAKTLCLPLVNFPMSKLA    928
GBLV      ------------------------------------------------------------    921
BLMoV     HVPQGMPV---VAFLNVMDTRMEDPLYSSLCGSFV--DLGRDRAKTLCLPLANFPLSKHI    1053
GSPNeV    HMPQGKPY---IALVTVMDGRFDDTGEAILCSSYV--NLGQKQARLMVAPLVNFPLTKED    1180
GTRSV     YMPMGKPF---MCLVSVMDGRFADSGEALLCSSYI--NLGQKKARMMVAPLVNFPLTQES    858


ToRSV     CRVRDGRVLKVIVSSTNSTLTHGLPQAQVSIGTLRQHLGPGHDRTISGALYASQQQGFNI    1226
AnNVA     CSVPDGRVLKVLVSSTNTTVQHGLPQAQISIGTNRQHLGPGHDRTVSQDMVMAQQMGFRI    1092
CLRV      CSAPDGRVLKVMVSSTNSTLIGGLPQAQVSIGTLRQHIGPGHDRTISRALATAQVQGYNV    983
SteNV     EDS-DDVLHGLVLATYFQDPTGFAVGKPAFQYGSLEFQEYKPSAYSDYSRVRDRWDEIAK    979
BLSV      KRLGNDD-PELYLATYFNDLLGYRPGELVFTYGSSELLEHKPDAYTNKSLCKDTWDDILK    995
PRMV      KQVNEDNKTRLYLATLFNNSCGIYPNAPVFQYGTSQLLEHRPDAYTNATLCKDEWHDIER    844
CawYV     CSVPDGRVIKVIISSTNSTLLDDLPRAQISIGTLRQHLGPGHDRTINQDLVESQVRGYRI    1018
SLSV      NQLNLDNDVRLYLATLLNEGVDKLVGRPVFSYGTSILLEHRRDALTNQTLCKDDWLEIEK    761
PVU       KRLGNDD-PELYLATYFNDTLGYRPGELVCTYGSSELLEHKPDAYTNKSLCKDTWDDILK    911
BRV       EDV-DDVLNGLMLCTHFQDSTKFGVGKPAFQYGTLEFQEFKPSAYSDFSRVRDNWDAIAK    987
GBLV      ------------------------------------------------------------    921
BLMoV     EDV-DDALNGLVLTTYFEDSTGFSVGKPVFQYGTLEFQEFKESAYSDFSRVRDRWDEIAK    1112
GSPNeV    --I-TDFVDNLYICTIIYNIGSLRSGCPVFSYGVIEAAEHWERANWDASRFVGDWDKILS    1237
GTRSV     --L-DDFLDNLYLTFIFYNINSIRKNQEILSYGVVEAAEHWERANWDVSRFVGDWDKILS    915


ToRSV     RATEQGGAVT---------------------------FAPQGGHVEGIPSANVQMGAGEH    1259
AnNVA     RATQQGGATT---------------------------MAPVGGHVEGTPSANVNLGAGDV    1125
CLRV      RAVQQGNATV---------------------------FAPQGGHVEGTPSADLQMGAGET    1016
SteNV     RQDTPGDRVLAGFSVLGAVSQDYNQSLPEFESISLQC-PPKNKPVVSTY-----------    1027
BLSV      RNVQKGNRIVKGFNVIDSISQDYEQEIPDFGDVNFNT-KPRSI-VAPTK-AFT-----AK    1047
PRMV      RNAKKGCRILKGFNVEEHIAQDYDQELLPFSEGSLLTCRTTER-SAPTH-AFS-----AA    897
CawYV     GATQQGNAVI---------------------------LAPRGTAVEGTPSASVDLGIGRQ    1051
SLSV      RNVQKGNRILEGFNVEQHIAQDYNQELAPFAPDLKLECRPNVVAEGATH-AFS-----AK    815
PVU       RNVQKGNRIVKGFNVIDSISQDYDQEVPDFGEVSFKT-KPRSG-VAPTK-AFT-----AK    963
BRV       QQNTPNDRILAGFSVLGAVSQAYNQALPVFKSVELVA-PPKRKPVVATF-----------    1035
GBLV      ------------------------------------------------------------    921
BLMoV     RQNTPGDRVLAGFSVLGAVSQDYNQPLPEFGEVELKC-PPRNKPVVATY-----------    1160
GSPNeV    GPTKRGGRVLAGFNLEGIASAPLDEPIPDLNQKLEIVAKPPSLPICGQLTVNG-------    1290
GTRSV     GPTARGGRVLAGLNLAGVASAPLDEPIPELNQRLEIVARPPSVPEMGALYVSG-------    968
```

```
ToRSV       LIQAGPMQWRLQRSQSSRFVVSGHSRTRG---------SSLFT-GS-VDR----TQ---Q    1301
AnNVA       LIQSGPTQWQLQRSRSERFVVAGNSRPRG---------ADARFNST-VQR----------    1165
CLRV        LVQTGGTHWKLQRSASSRFVVEGTSRTVG---------PRRMADVR-VDR----GDFPDQ    1062
SteNV       --REPKRFDDLSRHASFRMPATGFGAATN--RALDD------GKV--YSRVIDRG---FT    1072
BLSV        GVKEHSDPSTLRRSFSLARTQFGRVPTRN---------PTPFGGLAMGQAKHDP-----M    1093
PRMV        GVVKNAESSRLQRSCSVRFLNDSSSRSRN---------PSQFGGLAMGSAKHDP-----K    943
CawYV       LVQSGPLSWELTRTQSSRMSITGTSRARG---------ASSFGLAH-VSKRHDRGKQTED    1101
SLSV        GIVKNSLNSRLGRSMSVQFLNDIGSSSRR--TEFRPPVVQDFGGIAAGFANHTE-----G    868
PVU         GVVEVPNERKLTRTFSLARTQFGRAPTRN---------PSQFGGLANVSTSHVD-----Y    1009
BRV         --QNPTTLGRSNTTRSFRMPTMDLPRSTG--RDAPI--------------PIVHR---RN    1074
GBLV        ---EPQKLNRIGSTRSFRMPAVGMPNMTG--RVTIP--------------VQARP---RA    959
BLMoV       --QEPNKLNSSRSTRSFRMPSVNMPNMTG--RASIP--------------VQTNI---RN    1199
GSPNeV      ---QTDCAPILQRAYSVAGPSRLAGRSTIFSQRFTPPVVSNWETT--------RG-----    1334
GTRSV       ---QQEKASDFKRSYSIAGPSSRSS-FSGFSQRFNPPVVKNWDET--------PG-----    1011
                           *


                  MP-CP cleavage site

ToRSV       GTGAFEDPGFLPPRNS-SVQGGSWQE---------GTEAAYLGKVTCAKDAKGGTLLHTL    1351
AnNVA       -PRQEPTPGFNPARHSFSTVQSAWVQ---------GTEAVYAAKVTCAADAKAGTLLHSV    1215
CLRV        GARGQQQPPVNMPLQSGILPGMHWSA---------------ATSFKCAAEAAENSILARW    1107
SteNV       EPNKMARGEVAPPRMSTTLSGYV-----------APLHYSTYTQFTVPKDAAGGKLLKRE    1121
BLSV        PSNLDSKSMFEAPRHSTTCSGLE-----------SPYEIAISETYNVPKDAREGTHIGTI    1142
PRMV        PNNTI---PGEAPRHSTCMSAPD-----------NPFIVACTSVVKVPKDAKEGHYLGTI    989
CawYV       GAINIE--DMTAPRYTHMHSGLEENETVATVVEANPFVVAMTKAFEVNKDAKRGTYIGKI    1159
SLSV        -----STSKFEAPRYTNAQSGLE-----------NPYVVAVTKPFAVKKDAKEGTLITSL    912
PVU         TGSNREHNSFIAPRHSTAQSELE-----------NPFVVATTSVFKVAKDAKEGTFIGTI    1058
BRV         NNDVHGFDEATPARFSTCDSGLV-----------ADTTLAFAKMYQCKKDAKAGHVLATI    1123
GBLV        TTLIRDGEEVTPARFSTCNSALL-----------ADTSIAYIEQGWCPKDATAGRVLEAI    1008
BLMoV       SPRIVDGEEITPPRFTTCNSGLI-----------ADTSIAHVVQGWVPKDATKGRVLEAI    1248
GSPNeV      -RN-----DF---DTSETYSSMSTEV----STSSSVGKIFSYQEFVVPKGTTAGKVLCDH    1381
GTRSV       -R-------F---STSQTYSGMVAEQ----NA-SSSREILSYQEVSIPKDTSAGKVLCDH    1055
                           :                                  :  .  :


ToRSV       DIIKECKSQNLLRYKEWQRQGFLHGKLRLRCFIPTNIFCGHSMMCSLDAFGRYDSN-VLG    1410
AnNVA       DIIAAAKEMAFPKYLEWQRGGMMKGILKVSVYLPTNVFCGHSLMSVFDAFGRYSHD-IHG    1274
CLRV        SLRSIISESGTDAWIKWQREQRSTFLVEGTIAMSVNIMAGTTLGLVCDAFNRAK----HL    1163
SteNV       RLRDMLSRVVSAAEPNWMKSVSANLKVSGRISMGSNLMAGTALALVCDAFNRSAEF--GD    1179
BLSV        DFYTSIASQHKMPYQKWLGLGLIDAEVMLNIYCGGNPFLGTTIGIVYDFYNKLDIATTLG    1202
PRMV        DFYALLAKQRKAPYLNWCGKGLIAPEIIFRFFSGSNSFVGTTIGVVHDFFNRLDVKTTLG    1049
CawYV       DFLREIKDANRLPFQQWVATGLIDPVIQIKIYTGSNPFVGTTIAGTLDMFRRIDVD-KLG    1218
SLSV        DFYNLIKQQHRSPFYDWAAKGLIDPEIVIKTYAGSNQFVGTTIAIAHDFFSRVDVQGKLG    972
PVU         DFYDLVAKQHKSPYQQWLGSGLVDPEIQIKIFSGANAFMGTTVAIVHDFYNRLDIATKLG    1118
BRV         DIQECVFEDNRRVALDWLAHGLASFKYDLQLTVDSNPFVGVTLGITVDAFDRLLPQIS-D    1182
GBLV        NLREEIATGDNLVKYDWLAKGMIEPDMSVRLTVGQNPFVGISIGVCCDFSGRLAQYYDGA    1068
BLMoV       NLRDDIATSDNLVKYEWLAKGLIHPDLKLRMTIGQNPFVGISIGVCCDYFGRLSKYYEGD    1308
GSPNeV      HLMEKAEEFSAEVWHQLLSMQNFTGKFSFEVDLNISPLMGISIGVCFDFYNSLNLE--DL    1439
GTRSV       QILPSMRSFGGKAYQSLLQYENFVADFDFEVSLNMSPFMGISIGVCFDFFNSINLT--QL    1113
             :             .                   . : * ::    *
```

```
ToRSV       ASFPVKLASLLPTEVISLADGPVVTWTFDIGRLCGHGLYYSEGAYARPKIYFLVLSDNDV     1470
AnNVA       TSFPVKLACLMPSEVFSLADGPLVTWDVDIETYCGHGLFYAGGGYSTPVLHFLVLSDNAV     1334
CLRV        DNFPSALGQNMPQKVFPLSNPLERNFSFSMSELLGYTMHPHASAYEDVQFILYVLNTNDV     1223
SteNV       TTFPVKLGNMMPLALFPMADATARTFSFDLGELIGYPQHFKDDNFANVYFILYVVDTNES     1239
BLSV        GKLPRILGNCLPQTLHPMALGGFGSYKVNMSQYLGHSLFVSAKSFADPRFHLYIYDDNTT     1262
PRMV        GKLPRQLANCMPLSLHPLCEGGISEQSVNIRQYAGHALYAKSKGFCDPKFHIFIYDDNAL     1109
CawYV       GKMPVKAAGILPNFVHPLSGKSIKTYSFDTTRLAGHSLYPHARGFADPLIHFYVFGDNAV     1278
SLSV        GKMSRVIGNCLPQTLHPLSEGAIGTHNVNIEKYVGHSFYFNSPTFADPQFHVYIYSTNAV     1032
PVU         GRLPRLVGNCMPQTLHPLSEGGVTIDTVNISRYLGHSLYISAKGFADPRFHVYIYDDNAV     1178
BRV         EVIAVPLAFQLPTYLFPISKKGTFTQTIDFAAIAGYNFFPHVAAFGRPKIIVYIVSDNDL     1242
GBLV        TAIPIEICNQLPNFVCPISERSVFVHKINM-LLAGYNLFQTQKHFADPYILVYIIDTNTL     1127
BLMoV       TALPIEVCNQLPNFVCPISEKSVFEFDLDM-SLAGYNLFLTSKGFADPVLLVYIIDTNSL     1367
GSPNeV      GDLPIKAAALLPNFVFSSGDSI--KGSFDIREQLGHSLSVHGNSFGSGRIIVYTTTNNSI     1497
GTRSV       TDVPILAAQLLPNFVFSPGDPI--KGTFSLERDVGHSWNARANAYGTGRILVYTMLNNHV     1171
              .       :*  :            ..     *:        :    : .     *

ToRSV       PAEADWQFTYQLLFEDHT-FSNSFGAVPF-ITLP-HIFNRLDIGYWRG-PTEIDLTSTPA     1526
AnNVA       PAAASWQFAYEIFFDGAIVVDNSFVARPF-FSVP-YSFDRINLGYWKG-VNEFALSDTPT     1391
CLRV        ACAAEWGGHILWQVKDDA-------AEPYELQLPVVPKDGARLDVWRG-PATMSQGLFPY     1275
SteNV       PAEADWSGTIMWRVEESN-SAPI-----V-TTLPFPEAATAKLDLWRG-PAQFKQNVPLE     1291
BLSV        EASAEWRCTVEILVRRCSEERENFNIPIL-TIPCSNFNSFINLDLFKG-FGTIPLGKDPA     1320
PRMV        EMSAAWRLTVELLMKEDVEVEE-VGETPF-LRVSDSHLDYINLDLYKG-FGTIPLTDDPA     1166
CawYV       PCAANWQLAVDILIRKAP-RDATFACVPF-VSFPYTPPDFLDLGITIG-EGSLSLKSSNI     1335
SLSV        EMESDWQLTIEILIRKRESEMS-ASLTPI-LTVPQNPSAYVDLDLYKG-FGEIKLASRPQ     1089
PVU         EAAEGWRLTVEILVRKATNTTEEVVDNAL-LTVPMAPSSFIELDIYKG-FGSIALADEPL     1236
BRV         PASDTWMCLVELHMT-RL-ESSTLACSPT-LVLPQAFGGDLPLDLWRG-PYTFPLGGGTK     1298
GBLV        SASDEWGYTIELCVHSSV-HTTQFARTPF-LTLPGTFDGTLPLDLWRG-PFSFKTGKSAP     1184
BLMoV       PASDEWVYTCEVCIKSAL-HATSVANKPI-LSLPHSFDGRLPLDLWRG-PFSFELGRGSK     1424
GSPNeV      LAAEDFTGLITTFLSDIN--PAEFLLQPT-ISMPEFTTDERHHQIALGRYFRKRQSDTSA     1554
GTRSV       PAAEAFKGVLKVFVNNIR--PSPFLLRPT-VSLPEFTTDETHSRIGIGRHFTLKQNPDTK     1228
                 :       .                                 *

ToRSV       PN-AYRLLFG-LSTVISGNMSTLNANQALLRFFQGSNGTLHGRIKKIGTALTTCSLLLSL     1584
AnNVA       NM-AFLMTAG-AKWHVADGITTYSANIAFLRLAQGACGELVGRIKKVGTGLVTGVLLVAL     1449
CLRV        TT-NANLGFAEPRSVLTGYAPITSFHQAALSYYISYGGTIHGRLVKIGSGLVQVDIALAM     1334
SteNV       QH-TVALNFTNKQKFLTNRQPGFNFNMAKLGMYAGYRGWLVGKIVRTGPAIVQADLYLTI     1350
BLSV        IM-PIGLDFA-VAKEYATGKTCLGTTQAIFRCFLGVGGTLEGQLIRTSTIMVSCNVRILI     1378
PRMV        LA-PLGLDFA-MQREYTDGKYCLGTTQAMYRQVLGAGGLLEGRLRKVGTAMVSCILRLVM     1224
CawYV       PV-NVRLDFA--SKVDTNTSTSLGMTQALYGLNCGIAGFLHGNITKIGTALVSCSVRLVM     1392
SLSV        EV-MIAMDIA-ESRAYSDSHYCLGLPQAMFRTMQGSSGILHGRLRRVGSCLVSCVARLVM     1147
PVU         FV-PIGMNFA-SEVTYATGKTCLGFTQAKYRVYQGAGGRLQGRLRRVGTTLTSCILRLVM     1294
BRV         RLS-TSLDIGTSTTTVSGWR-TVSFPAAYALFLQGHGGSLVGEVVHTGSAAVSCALHLCI     1356
GBLV        REERIGINFGSKRTYNSGAKEFYSLPAAHIQLLQSVGGILHGSVIQTGSRAISCELYMIL     1244
BLMoV       REEHIGINFGSARV-VSGTNTFYSFPAAYTQLLQSVGGILHGTVVQTGSRAISCEMFLIL     1483
GSPNeV      II-KFDLDFARIES-VLTGKSALCHTAAIQSILAYQTGELEMEFFKLGSAFIQGGFIVSS     1612
GTRSV       VV-AFSLDFAILYS-VLTDKTVLCSSAAIQRLLMYQEGDLELEFQKLGISLVQAGFIVSI     1286
                  :                    *         * :   . : .         :
```

```
ToRSV       RHKDASLTL-ETAYQRPH--YILAD-GQGAFSLPISTPHAATSFLEDMLRLEIFAIAGPF   1640
AnNVA       SNKGDPIDT-YSMRRRPH--VKLLS-GEGSFRLPISSAFNALSFLDDVMQLRVCCLAGPH   1505
CLRV        WHECADMVSYRGIIKIPH--VLLRG-GEGEFALPINAPFGYTSTRDRGPTLAVCLVSGVV   1391
SteNV       ASG-AA-QSYARLLQYEG--WELPG-GQGDFRLRLNNAYMATSAYEQDSLLYVTRISGPV   1405
BLSV        WYGLSL-PTLQETGSIPHEDLDFSR-SDGTFRLKIQSPFARIANRTIDARLLVYPLGGPI   1436
PRMV        WWDLEL-PTLEETSEFPHIDIDLQDPKQGDFSILMRSPFARVPNRETRGRLLMYPIGGPI   1283
CawYV       WWGSKF-PPFEETSSMPHEDMDLDV-GSQDFRIQFQTPFSTVGSYDSTARLFIYALGGPL   1450
SLSV        WWGSDP-PTLAETSMYPFVDIDFQV-SDGEFSLKIQSAFARFPNRERKAWLVVYPLGGPL   1205
PVU         WWGSTY-PTLEETSAIPHVDMDLAK-VDGSFDLEIRSPYGRVPNREMQGYLVIYPIGGPI   1352
BRV         SFGGAP-PTLEEALVFPG--FRLPS-GEGKFHIKVQTPYGRLSTLTPDCALYVYLAGGPI   1412
GBLV        QPDKTA-NNLEQAVKLPG--CRVPT-GGGPFSLRIQSAFLRSQIYETGVQLVIYALGGPL   1300
BLMoV       QPDKTA-HNLEQALRLPG--CRIPT-GGGPFSIRIQTPFQREQIFNTGVQLVVYAVGGPM   1539
GSPNeV      WWGNHD-HSLSEILRVHH--VRLNE-GIGKISIPISTPFGNIPMMEKRAQILLYFHNKPS   1668
GTRSV       WPDARA-RGISDVLKVHH--VSLPT-GVGRVTLPLASPFGRVSTTEVTDTVQLYFDSTPN   1342
                                  .       . : .   .           : :

ToRSV       SPKDNKAKYQFMCYFDHIELVEGVPR-TIAGEQQFNWCSFRNFKI----DDWKFEWPARL   1695
AnNVA       APKDVTSPFQFMVYFDHIDLKATVPR-VICADTVFNWCMMSKFTV----DNWSAQFPARL   1560
CLRV        APKDCSAPYRYMIYFDRVEFNAQLPP-VIANRLQFLWASFSEFKPVVPASNRTWMIPCRL   1450
SteNV       APASISASFCYMVYLDHIEFDVEIPP-IVTSSLAFDWCVITPETD-----DFGVRIPARL   1459
BLSV        ATKGCNAPFSFAIYVKGIHFDEAVQP-LLFPDREYHWFQLDSFAK----GALTIPLPNHI   1491
PRMV        AATGVNSPFNFVIYIKGIRPLQRVPI-ISLANAWYSWMQIDTFSK----GHIELTIPNHV   1338
CawYV       APTGCTASCDFCLKIQGVETVKPPIP-IELMGEEIAWCELQEIDS----SVTSISIPNHI   1505
SLSV        AAKGVGTSFQFAIYLKGIEVSEPMPP-VLLPDHEYVWMQVNPPNQ----GEFTFDLPNHI   1260
PVU         SPSGSTAPFNFSVYIGGIQATQQIPT-LLLPDKEYVWCQLDAFNP----GVVTFDLPNHI   1407
BRV         AVAPMSVPYQFCIHLERLVDDG-APPRTIGLIREFNWATINNFKS----DDITFAIPARL   1467
GBLV        GAATISAPYQYMVHFSHITEEEGFVPRPIGTILEFNWATLAQLTL-----KDRFQIPARL   1355
BLMoV       GAQAISAPYQYMVHFSHIQEEGDPPPRPIGNVLLFNWATISKMTN-----LTRFQIPARL   1594
GSPNeV      APSTYTGAYEGFVKFLRFKPKIFIPR-TISFEERYAWCSISGLTS----ELNQFYLPARL   1723
GTRSV       APDTINGAYQGYVRFLGFRPREFIPR-VVNAGTRFAWCQLSELKE----DFTSFFLPARL   1397
            .             .  .                  *  .                * ::

ToRSV       PDILDDK---SEVLLRQHPLSLLISSTGFFTGRAIFVFQWGLNTTAGNMKGSFSARLAFG   1752
AnNVA       SDIVNKE---CSVKMHTHPLSVLVASTGFMSGEAEFEFMWSLSAEFGKAHGIVSAHTMYG   1617
CLRV        SDYKVEG---ATIKMEAHPLARLVASAGMFQGTMRFILRWTFSNTLNTPTTYVQLTHKFG   1507
SteNV       SDIILKD--KAKVHMNNHPLAKMVATSGMMRGKLTMVIQWMYESAVTIPKGYVWAGANFG   1517
BLSV        CDFALDKQKVATVHLRSNPLSAIFGSCGFFKGNLTMIFRWTMERKISDGGSAIWIARCYG   1551
PRMV        CDVKFTG---AEARLRTNPLSVLFGTCGFFSGHIKMRFEWAQTGKITDGGTVIWLGKMYG   1395
CawYV       CDFDIAG---VGILLRDNPLSRLFSACGFFSGDIEFSFSWSSAKQITEGGASLTISRNFG   1562
SLSV        CDVKVSG---ATTLLRENPLACIFGACGFFAGTLTMTVGWSNRGGISTKESTLWFGKCYG   1317
PVU         CDFTVVG---AAVHLRSNPLSAIFGSCGFFKGSLSITLEWTQEGKLPDKGSVVWIAHKYG   1464
BRV         SDLVLTC---GDVTMSTNPLALLIGSCGFFRGNLTVVLEWATFLKAGDKEGTVQLTTCRG   1524
GBLV        SDLVIPG---VSVHMRSNPLASIIGACGFFRGHVTFILQWSLNVEHVKPKTYMQVQTCVG   1412
BLMoV       SDLVLPG---QTVTMRRNTLANLIGSCGFFRGRVTFVFQWTLNVAHIVPTATMQILTAVG   1651
GSPNeV      CEIKLTG---ALVVMWNNNHLHKLVATSGIFRGSITYHLSISYKKRLTELNGRIRIQSAYG   1780
GTRSV       CDLVHKS---AKVIMWSNHLHWLVGTSGFFRGNITLHVTLAYSKKVTELDKLFQVQGIHG   1454
             :            :  : *  :..: *:: *     .              .      *
```

```
ToRSV        KG-VEE----IEQTSTVQPLVG------ACEARIPVEFKTYTGYTTSGPPGS--MEPYIY     1799
AnNVA        PV-EDQ----FVLGVVTQPLTS------SNMLKVTVALSNYTGCITSGDSGF--QEPYIW     1664
CLRV         TA-TVN----ESYLTKLAHASQ------ATEISIDVVVAGLGGFMRSGVAES--RENFVA     1554
SteNV        KLVSGTEDENT---RFSVTRSKAF----PEPIRIPLIVGDVSGFIASGGRGA--FEAYVN     1568
BLSV         TT-DSH----EVLESQISNVYT------PGEIRLELNTGDFSGANIPGGTTS--PKQFPL     1598
PRMV         TM-ADR----EVLESVTNNTYS------PGLAEFVIDVADFSGYNIPGGSKQ--KTQFIV     1442
CawYV        AP-GKG----LSLSTKTYNLNI------PGVHRQILKVGDFSGFNIPGGTGH--ETQYSS     1609
SLSV         DRVGTR----EILDHCTFNAHQ------AGSCSYALEVGDFSGFQIPGSTGN--KHQYIS     1365
PVU          TP-ADS----EILDSCTSNSYL------PGKCTFLLHTGDYTNANKPGGSGN--KLQFIT     1511
BRV          MINNVKGVRN-AIQKKVVNLSL------VGSVSRYLNVGDFTGFAQSGGQ-VGYDEIFLE     1576
GBLV         TFIPAPVKHSQILQSWVVPISQ------RFELRVPFDLVDYPGFNSSGGIGLDHMQPFID     1466
BLMoV        RVGNAETNGSQILQSWIVPISQ------VFEKEVEMDLTDYPGFNTSGGIGADHDQPYID     1705
GSPNeV       RL--------NALDTWQRKSTRIFSCYDTHTITQQLLVGDFSGATTSSPREV--LENFMV     1830
GTRSV        EI--------KVPTTWQVRSSSLFTCFQHDRASYPVTVGDYSGVTTSAPTGH--RENFIH     1504
                                                .      .    .         :

ToRSV        VRLTQAKLVDRLSVNVILQEGFSFYGPSVKHFKKEVGTPSATLGTNNPVGRPPENVDTGG     1859
AnNVA        LNISQAKAIERLNVNVKLKPGLKLYGPTVMRMHKAVGSTSAQNTVGA-------------     1711
CLRV         VSLSKPGDLAKLDIIIELMPGFRFRGPTITPLVRV-------------------------     1589
SteNV        IYATAAKEIKRMVLSVELDSGFEFRGPTISPIVS--------------------------     1602
BLSV         IWIADGASVGNIQCSVLLHSGFAFYGRSCLAIK---------------------------     1631
PRMV         IWIDNGEVIHNIRVSVQLQSDFSFYGRSCLLT----------------------------     1474
CawYV        LWCQDWSLLETMTISARILPGFKFYGWSCIKPSA---TPAAFTTVEH---SPPSAA-T-K     1661
SLSV         VWMENAGSVSHFRISVRLHRGFSFYGRSCLPIK---------------------------     1398
PVU          VWLGEASAVDNIRVSVRLMPGFSFYGRSISFPT---------------------------     1544
BRV          FSTNKAKQIRYLNINVELDENFELYGRTIIPLKNTAP---AFASTSA---SAPNES----     1626
GBLV         IACGDFSQLEYFNINVELKPGFEIYGRSVTPLK---------------------------     1499
BLMoV        IACGNFPQIFYMNINVRVHPGFELYGRSITPLRT--------------------------     1739
GSPNeV       VGIDTPDMIDTIDVDVEVH-HLDFYGNKIVII----------------------------     1861
GTRSV        FFTNAAELISHIEVEVEVH-NLSFYGRKLIIK----------------------------     1535
             .       :  :     :   : : * .

ToRSV        PGGQYAAALQAAQQAGKNPFGRG     1882
AnNVA        -----------------------     1711
CLRV         -----------------------     1589
SteNV        -----------------------     1602
BLSV         -----------------------     1631
PRMV         -----------------------     1474
CawYV        PREERARKVRTV-----------     1673
SLSV         -----------------------     1398
PVU          -----------------------     1544
BRV          -----------------------     1626
GBLV         -----------------------     1499
BLMoV        -----------------------     1739
GSPNeV       -----------------------     1861
GTRSV        -----------------------     1535
```
