# Supplementary material for: Re-examination of nepovirus polyprotein cleavage sites highlights the diverse specificities and evolutionary relationships of nepovirus 3C-like proteases
Source: Arch Virol. 2022 Aug 30;167(12):2529–43. doi: 10.1007/s00705-022-05564-x (PMC9741568; doi:10.1007/s00705-022-05564-x)
Supplement: Supplementary file 4 — Supplementary Material 4 [file 705_2022_5564_MOESM4_ESM.pdf]

Supplementary Material 4 – Phylogenetic trees using partial protease sequences

Re-examination of nepovirus polyprotein cleavage sites highlights the diverse specificities and evolutionary relationships of nepovirus 3C-like proteases

Archives of Virology

Hélène Sanfaçon
Corresponding author: Hélène Sanfaçon Summerland Research and Development Centre, Agriculture and Agri-Food Canada, helene.sanfacon@agr.gc.ca

Partial protease sequences were aligned using Clustal W and trees were generated using the maximum likelihood method as implemented in MEGA X using default settings. Bootstrap values were calculated over 1000 replicates and the values are indicated at each node. The tree on the left used the sequence of the protease comprised between the conserved catalytic histidine and the conserved histidine (or leucine) of the substrate-binding pocket; the tree on the right used the sequence of the protease comprised between the conserved catalytic histidine and the conserved catalytic cysteine (or serine). Please see Online Resource 1 for labelling of these amino acids in the alignments. Identified clades are labelled with the same colors as those shown in Fig. 3.

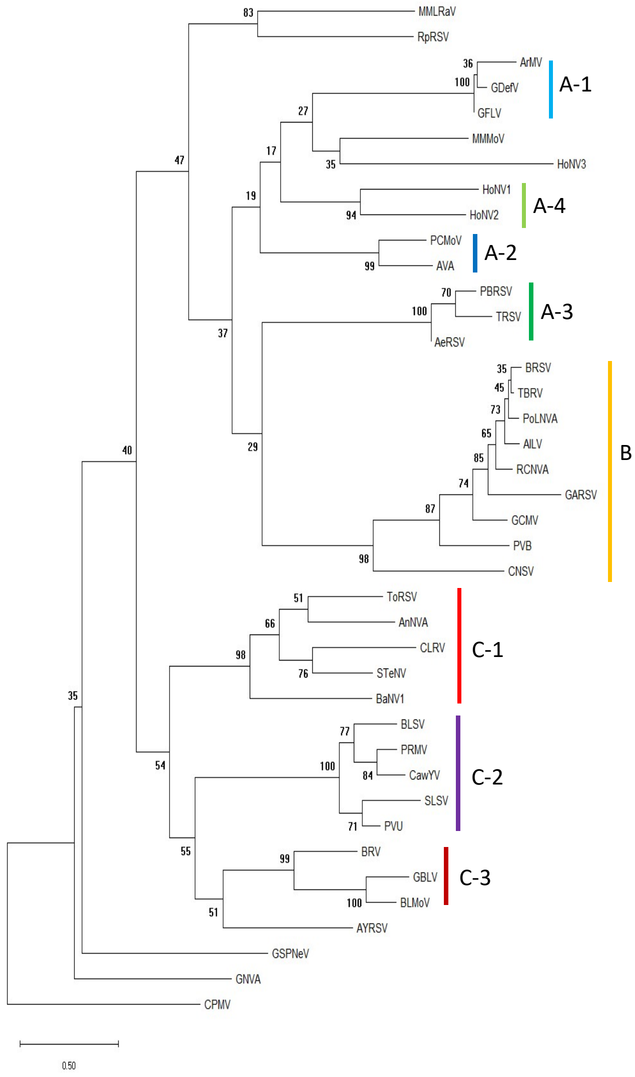

Protease catalytic His to SBP His (or Leu)

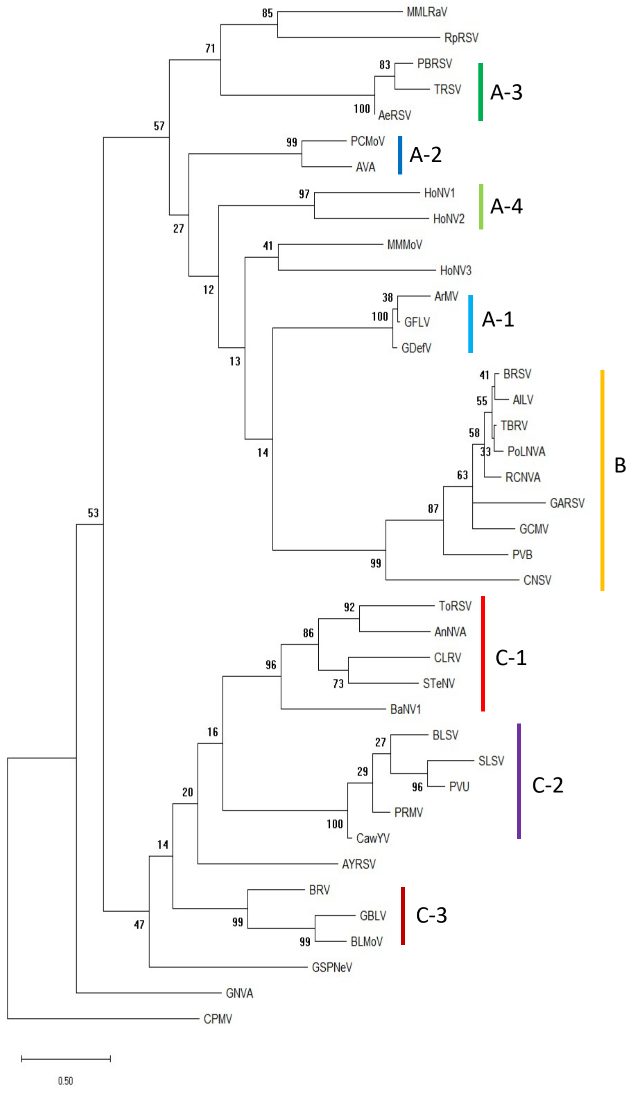

Protease catalytic His to catalytic Cys (or Ser)
