# Supplementary material for: Re-examination of nepovirus polyprotein cleavage sites highlights the diverse specificities and evolutionary relationships of nepovirus 3C-like proteases
Source: Arch Virol. 2022 Aug 30;167(12):2529–43. doi: 10.1007/s00705-022-05564-x (PMC9741568; doi:10.1007/s00705-022-05564-x)
Supplement: Supplementary file 5 — Supplementary Material 5 [file 705_2022_5564_MOESM5_ESM.pdf]

Supplementary Material 5 – Phylogenetic trees using the Pro-Pol or CP sequences

Re-examination of nepovirus polyprotein cleavage sites highlights the diverse specificities and evolutionary relationships of nepovirus 3C-like proteases

Archives of Virology

Hélène Sanfaçon
Corresponding author: Hélène Sanfaçon Summerland Research and Development Centre, Agriculture and Agri-Food Canada, helene.sanfacon@agr.gc.ca

Sequences were aligned using Clustal W and trees were generated using the maximum likelihood method as implemented in MEGA X using default settings. Bootstrap values were calculated over 1000 replicates and the values are indicated at each node. Sequences from CPMV (a comovirus) was used as an outgroup. The tree on the left used the Pro-Pol sequence comprised between the conserved catalytic cysteine (or serine) of the protease and the GDD motif of the polymerase. Please see Online Resource 1 for labelling of these amino acids in the alignments. The tree on the right used the CP sequence (for CPMV, both the two amalgamated large CP and small CP were amalgamated). Identified clades are labelled with the same colors as those shown in Fig. 3.

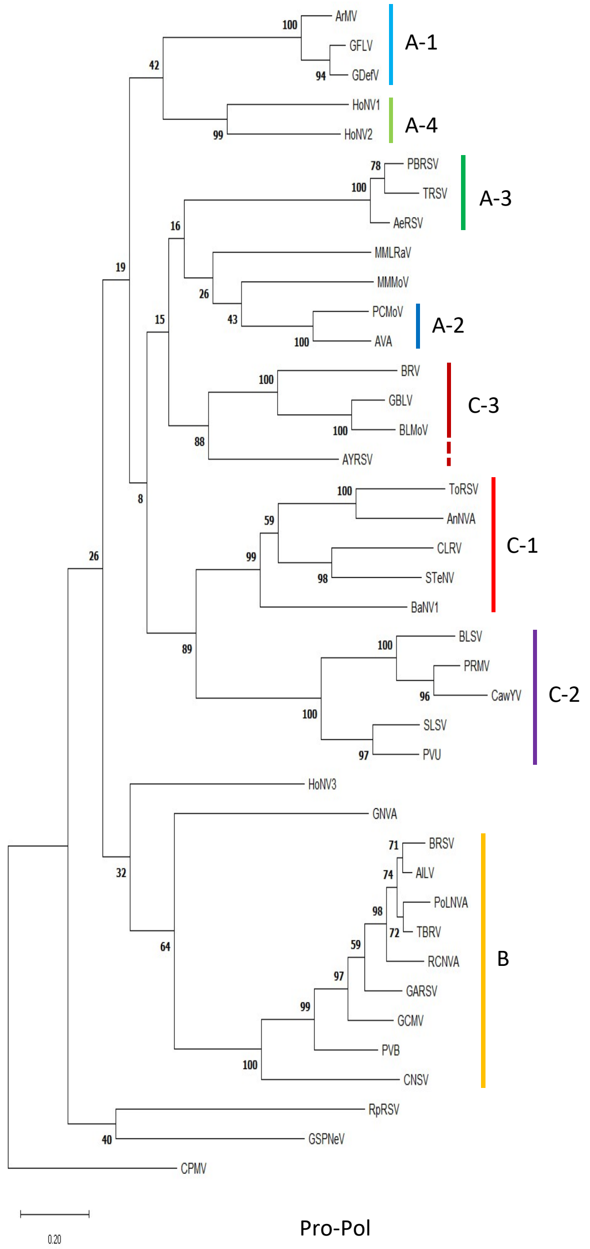

Pro-Pol

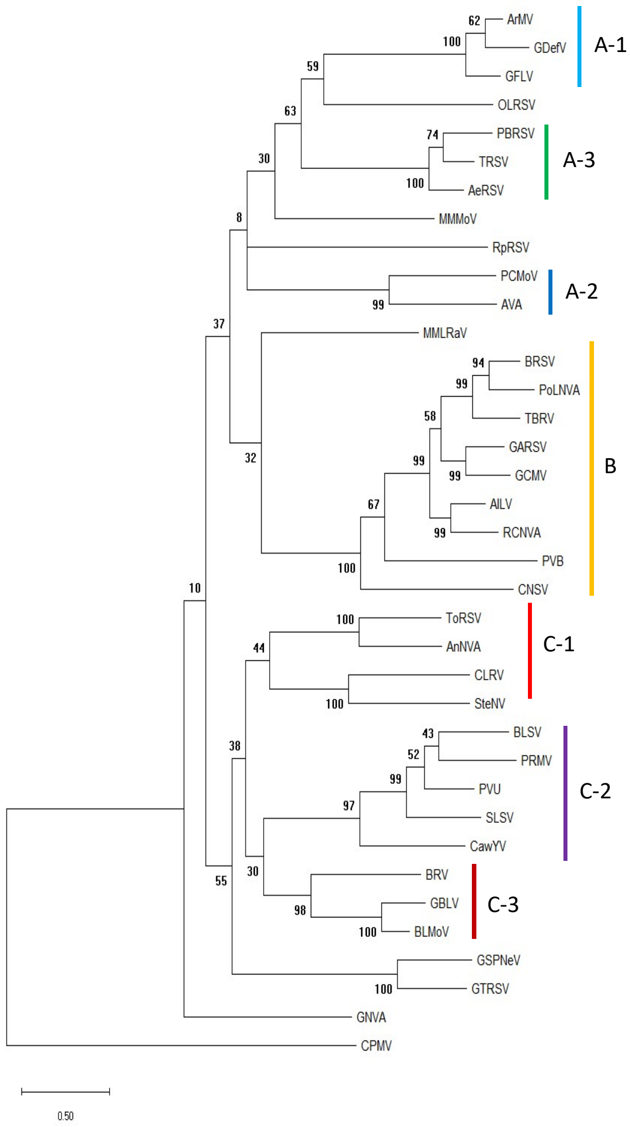

CP
