# Supplementary material for: Re-examination of nepovirus polyprotein cleavage sites highlights the diverse specificities and evolutionary relationships of nepovirus 3C-like proteases
Source: Arch Virol. 2022 Aug 30;167(12):2529–43. doi: 10.1007/s00705-022-05564-x (PMC9741568; doi:10.1007/s00705-022-05564-x)
Supplement: Supplementary file 6 — Supplementary Material 6 [file 705_2022_5564_MOESM6_ESM.pdf]

Supplementary Material 6 – Alignment of P1 and P2 polyproteins of ArMV and GFLV isolates

Re-examination of nepovirus polyprotein cleavage sites highlights the diverse specificities and evolutionary relationships of nepovirus 3C-like proteases

Archives of Virology

Hélène Sanfaçon

Corresponding author: Hélène Sanfaçon Summerland Research and Development Centre, Agriculture and Agri-Food Canada, helene.sanfacon@agr.gc.ca

The sequence of the type isolate for the RNA1 and RNA2 polyproteins of ArMV and GFLV were used to conduct a BLAST search against other isolates available in NCBI. Alignments of selected isolates were generated using the NCBI interface. Cleavage sites are indicated above the alignments (positions P6 to P1'). Amino acids that differ from the cleavage site sequence of the type isolates are highlighted in green (positions P1 and P1'), yellow (position P4) or grey (positions P6, P5, P3 and P2).

ArMV isolates RNA1 polyproteins

```
YP_053925.1  1     MWQISEGSQCCCTGKTWSNAEAKEARYVCNCILSCRLVKVEVVPQLPKSRIAPAQDKAERITPLCNSNGGAAPTIPKSKR  80
AGT56093.1   1     MWQISEGSQCCCTGKTWSNAEAKEARYVCNCILSCRLVKVEVVPQLPKSRIAPAQDKAERITPLCNSNGGAAPTIPKSKR  80
QBZ78358.1   1     MWQISEGSQCCCTGKTWSNAEAKEARYVCNCILSCRLVKVEAVPQLPKSRIAPAQNKAERITPLCNPSGEAVPTTPKSKR  80
AGD98722.1   1     MWQISEGSQCCCTGKTWSNAEAKEARYVCNCILSCRLVKVEVVPQLPKSRIAPAQSGAERVTPLCNLNGGIAPTTLKSKR  80
DAZ85702.1   1     MWQISEGSQCCCTGKTWSNAEAKEARYVCDCILSCRLVKVEAVPQLPKSRIAPAQNEAERVIPLCGSNGGIAPTTLKSKR  80
QGY99255.1   1     MWQISEGSQCCCTGKTWSNAEAKEARYVCNCILSCRLVKVEAVPQLPKSRIAPTQSGAERITPLCNLNGGIAPTIPKSKR  80
DAZ85701.1   1     MWQISEGSQCICTGKTWSNAEAKEARYVCNCFLSCRLVKVGVVPQLPVSRIAPAQNIAERVAPPCISNDGIAPITPKSKR  80
DAZ85704.1   1     MWQISEGSQCCCTGKSWSNAEAKEARYVCGCFFNCRLVKVEVAPQPPKSRIAPVHQKKERIAPLQNSNGRTAPTTPKSKR  80
QYU59218.1   1     MWQISEGSQCCCTGKSWSNAEAKEARYVCNCFIGCRLVKVEAVPQLPEGRIAPAQFKAEKLMPSCDVNGRIAPTTHQSKR  80
ADJ39329.1   1     MWQISEGSQCCCTGKSWSNAEAKEARYVCDCLYNCRLVKVEVAPQPPKSRIAPAQQKKERIAPLQNSNGRMAPTTPKFKR  80
QBZ78354.1   1     MWQISEGSQCCCTGKTWSNAEAKEARYVCNCILSCRLVKVGVAPQLPKSRIAPVQNVAEEVAPPCFLNGGTAPTFPKSKR  80
QBZ78356.1   1     MWQISEGSQCCCTGKTWSNAEAKEARYVCNCILSCRLVKVGVAPQLPKSRIAPVQNVAEEVAPPCFLNGGIAPTLPKSKR  80
QYA72350.1   1     MWQISEGSQCCCTGKSWSNAEAKEARYVCNCFLNCRLVKVEVAPQPPKSRIAPAHLMKERIAPLHNSNGRTAPTTPKSKR  80
ADJ39331.1   1     MWQISEGSQCCCTGKSWSNAEAKEARYVCNCLYNCRLVKVEVTPQPPKSRIAPAQQKKERIAPLQNSNGRMALTTPKSKR  80
DAZ85705.1   1     MWQISEGSQCCCTGKSWSNAEAKEARYVCDCHFNCRLVKVEVVPQPPKSRIAPAQQKKERIAPLQNSNGRTAPTTPKSKR  80
DAZ85709.1   1     MWQISEGSQCCCTGKSWSNAEAKEARYVCDCVYSCRLVKVEVAPQPPKSRIAPAQQKKERIAPLQNSNGRTAPTTPKSKR  80
DAZ85710.1   1     MWQISEGSQCCCTGKSWSNAEAKEARYVCDCVYSCRLVKVEVAPQPPKSRIAPAQQKKERIAPLQNSNGRTAPTTPKSKR  80
DAZ85703.1   1     MWQISKGSQCCCTGKSWSNAEAKEAQYVCDCHFNCRLVKVEVAPQPLKSRIAPAQQKKERIAPLQNSNGRIAPTTPKSKR  80
ADJ39330.1   1     MWQISEGSQCCCTGKSWSNAEAKEARYVCDCLYNCRLVKVEVAPQPPKSRIAPAQQKKERIAPLQNSNGRMAPTTPKSKR  80
ACF32434.1   1     MWQISEGSQCCCTGKSWSNAEAKEARYVCDCLFNCRLVKVEVAPQPPKSRIAPAQQKKERIAPLQNSNGRIAPTTPKSKR  80
QYU59216.1   1     MWQISEGSQCCCTGKSWSNAEAKEARYVCNCFYNCRLVKVEVAPQPLKSRIAPAHQKKERIAPLHNSNGRIAPTTPKSKR  80


YP_053925.1  81    AFEPRTPLIKQRCDVVVRVGPPADLDLVYPALVQEEVAIPPTEKVLQPTLKAEVRVPIFCAPKRMVAFPKPPTKIASKRD  160
AGT56093.1   81    AFEPRTPLIKQRCDVVVRVGPPADLDLVYPALVQEEVAIPPTEKVLQPTLKAEVRVPIFCAPKWMVAFPKPPTKIASKRD  160
QBZ78358.1   81    AFKPCTPLIKQRCDVVVRVGPPADLDLIYPALVQEEVVVPPTEKVPQPTLKAEVRVPVSCAPKWMVAFPRPPVKIAPRRE  160
AGD98722.1   81    VFVPNVPLFKQRCDVVVRVGPPANLDLVYPALVQDEVATPPTEKVLQPTLKADVRVPVSCAPKWMVVFPKPPIKIAPKGV  160
DAZ85702.1   81    ALVPSAPLLKQRCDVVVRVGPPADLDLVYPALVRDEVAIPPTGKVLQPTLKADVRVPVACAPKWMVAFPRPSTKIAPKGV  160
QGY99255.1   81    AFVPNAPLLKQRCDVIVRVGPPVDLDLVYPALVQEEVVIPPTEKVLQPTLKADARVPVSCAPKWMVAFPQPPVTVAPKRE  160
DAZ85701.1   81    AFVPGAPLLKQRCDVVVRVGPPANLDLVYPALVQEEVAIPPTEEVLQPTLKADTRVPVFCAPKWMVAFPKPSISVAPKGV  160
DAZ85704.1   81    APVPDTPLVKQRCDVVVRVGPPANLELIYPALVQEEVAIPPTEKVLQPTLKADVRVPVCCAPKWMVAVPKPPVKIAPRKE  160
QYU59218.1   81    ALVPKAPLFKQRCDVVVRVGPPADLDLIYPALVREEVAIPPPEEVLQPTVKADIRVPVSCAPHWLVAFPEPPAKKAPKRD  160
ADJ39329.1   81    ASVPDAPLVKQRCDVVVRVGPPANLELVYPALVQEEVATPPTEKVLQPTTKADVRVPVCCAPKWMVAIPKPPVKTAPRVE  160
QBZ78354.1   81    AFVPDTPLLKQRCDVVVRVGPPADLDLVYPALVQEEVVVPPTEKVLQPTLKADVRVPVSCAPKWMVAFPEPSIKIAPKGA  160
QBZ78356.1   81    AFVPGTPLLKQRCDVVVRVGPPADLDLIYPALVQEEVAVPPTEKVLQPTLKADVRVPVSCAPKWMVAFPEPSIKIAPKGA  160
QYA72350.1   81    AFKPDTPLVKQGCDVVVKIGPPANLELTYPALVREEVAAPPTERVLRPTLKADVRVPVSCAPKWMVAIPKPPVKIAPKRE  160
ADJ39331.1   81    AYVPDAPLVKQRCDVVVRVGPPASLELIYPALVQEEVATPPTKKVLQPTTKADVRVPVCCAPKWMVAIPKPPVKTAPRVE  160
DAZ85705.1   81    ASVPNVPLVKQRCDIVVRVGPPANLELIYPALVQEEVAIPLTEKVLQPTIKADVRVPVNCAPKWMVAVPRPPVKIAPKRE  160
DAZ85709.1   81    AFVPSAPLVKQRCDVVVRVGPPANLELIYPALVQEEVVVPPSERVLQPTQKADVRVPVSMAPKWMVAFPKPPVKTAPVVE  160
DAZ85710.1   81    AFVPSAPLVKQRCDVVVRVGPPANLELIYPALVQEEVVVPPSERVLQPTQKADVRVPVSMAPKWMVAFPKPPVKTAPVVE  160
DAZ85703.1   81    ASVPDAPLVKQRCDVVVRVGPPANLELIYPALVQEEVAIPPTEKVLQPTIKADVRVPVCCAPKWMVAIPRPPVKTAPKVE  160
ADJ39330.1   81    ASVPDAPLVKQRCDVVVRVGPPANLELVYPALVQEEVATPPTEKVLQPTTKADVRVPVCCAPKWMVAIPKPPVKTAPRVE  160
ACF32434.1   81    ASVPDVPLVKQRCDVVVRVGPPANLELIYPALVREEVATPPTEKVLQPTIKADIGVPVSMAPKWMVAIPKPPVKTAPEVE  160
QYU59216.1   81    ATVALPPLVKQRCDIVVKVGPPANLELIYPALVQEEVAIPPTKKVLRPTEKADIRVPVCCAPKWMVALPRPPIKTAPKRE  160


YP_053925.1  161   ALQFPAGAVAFNGINFIDAKGKVVLSEGAKRILKGIRVAKQQRQRTARRSAACKKVRKARDLALFKRLSEECTFQDLPGG  240
AGT56093.1   161   ALQFPAGAVAFNGINFIDAKGKVVLSEGAKRILKGIRVAKQQRQRTARRSAACKKVRKARDLALFKRLSEECTFQDLPGG  240
QBZ78358.1   161   KAQFPKGAVCYNGVNFIDVKGKVVLSEGAKRILKGIRVAEQQRRRAARRSAACRKIRKARDLALYLRLSEERTFQELPGG  240
AGD98722.1   161   ELQFPAKAVAFNGVNFVDAKGKVVLSEGAKRILRGIRVAKQQRQRAARRSAACKEVRKARDLALFKRLSEERTFQELPGG  240
DAZ85702.1   161   ELQFPTGAVAFNGVNFIDAKGKVVLSEGAKRILKGIRVAKQQRQRAARRSAACRKVRKARDLALFKRLSEERTFQELPGG  240
QGY99255.1   161   ALQFPTGAVAFNGTNFIDAKGKVVLSEGAKRILKGIRVAERQRQRAVRRSAACMKVRKARDLALFKRLSEERTFQELPGG  240
DAZ85701.1   161   ELQFPTGAVAYNGINFIDVKGKVVLSEGAKRILKGIRVAKQQRQRAARRSDACRKVRKARDLALFKRLFEERTFQELPGG  240
DAZ85704.1   161   KMQFPRGAVCFNGTNFFDAKGKVVLSEGAKRILKGIRVAKQQRLRAARRSAACKKIRKARALALYKRLSEERTFQELPGG  240
QYU59218.1   161   ELQFPAGAVCYNGVNFIDVKGEVVLSEGAKRILKGIRVAKQQRQRAARRIDACRKIRKARDLALFKRLSEERTFQELPGG  240
ADJ39329.1   161   QTQFPKGAVCYNGTNFLDAAGKVVLSEGAKRILKGIRVAKQQRQRAARRSAACKKVRKARALALYKRLSEERTFQELPGG  240
QBZ78354.1   161   KLQFPAGAVAYNGINFIDAKGKVVLSEGAKRILKGIRVAKQQRQRAVRRSAACRKVRKARDLALFERLSEERIFQELPGG  240
QBZ78356.1   161   KLQFPAGAVAYNGINFIDAKGKVVLSEGAKRILKGIRVAKQQRQRAARRSAACRKVRKARDLALFERLSEERIFQELPGG  240
QYA72350.1   161   KIRFPKGAVCYDGTNFFDAKGKVVLSEGAKRILKGIRVAAKQRLRAARRSAACKKIRKARALALYKRLSEERTFQELPGG  240
ADJ39331.1   161   QTQFPKGAVCYNGTNFLDAAGKVVLSEGAKRILKGIRVAKQQRQRAARRSAACKKIRKARALALYKRLSEERTFQELPGG  240
DAZ85705.1   161   KTQFPKGAVCYNGTNFLDAKGKVVLSEGAKRILKGIRVAEQQRQRAARRSAACKRIRKARALALYKRLSEERTFQELPGG  240
DAZ85709.1   161   KTQFPKGAVSYNGVNFLDAKGKVVLSEGAKRILRGIRVAKKQRLRAARRSAACKKIRKARALALYERLSEERTFQELPGG  240
DAZ85710.1   161   KTQFPKGAVCYNGINFLDAKGKVVLSEGAKRILRGIRVAKKQRLRAARRSAACKKIRKARALALYERLSEERTFQELPGG  240
DAZ85703.1   161   KMQFPKGAVCYDGTNFLDAKGKVVLSEGAKRILKGIRVAKRQRLRAARRSAACKKIRKARALALYKRLSEERTFQELPGG  240
ADJ39330.1   161   QTQFPKGAVCYNGTNFLDAAGKVVLSEGAKRILKGIRVAKQQRQRAARRSAACKKVRKARALALYKRLSEERTFQELPGG  240
ACF32434.1   161   KTQFPKGAVCYNGTNFLDAKGKVVLSEGAKRVLKGIRVAKQQRLRAARRSAACKKIRKARALALYERLSEERAFQELPGG  240
QYU59216.1   161   KRQFPKGAVAYNGTNFFDAKGKVVLSEGAKRILKGIRVAEKQRLRAARRSAACRKIRKVRALALYKRLSEERTFQELPGS  240
```

```
YP_053925.1   241   FAGEIPAGHACYRK-VAAPTTSFKKEVSKGKKAKKPSTPVLPAQDFSCVDSFDWGEKSSPVE--IEDDWVLIEKPVLQRQ  317
AGT56093.1    241   FAGEIPAGHACYRK-VAAPTTSFKKEVSKGKKAKKPSTPVLPAQDFSCVDSFDWGEKSSPVE--IEDDWVLIEKPVLQRQ  317
QBZ78358.1    241   FAGEVPAGHACYQK-VAAPAASLKKEVSKRKKAKKPSTPVLPAQDFSCVDSFDWGEKSSPVE--IEDDWVLIEKPVLQRQ  317
AGD98722.1    241   FMGEIPIGNMCYGKPIASPNATFKKVVSKGKKTVKSSKPALSVQDFSCIEDFDWGEKSSAVN--IEDDWILVEKPVLKRR  318
DAZ85702.1    241   FMGEIPIGNMCYGKAIASPNATLKKVVSKGKKALMSSKPVLPVQDFSCIEDFDWGDKSSAVD--IEDDWILVEKPVLKRQ  318
QGY99255.1    241   FMGEIPIGSMCYSKTVASPFATPEKVASKGKKAIKPSKPVLPVQDFSCIEDFDWGEKSLAVE--IEDDWVLIEKPVIKRQ  318
DAZ85701.1    241   FMGEIPIGHMCYGKTIASPTATFKKVVSKGKKAAKPSKPMLPVQDFSCIEDFDWGEKSLLVD--IEDDWVLVEKPVIKRQ  318
DAZ85704.1    241   FAGEIPAGFFSYGKTVASPSATPKKVVSKRKRASKPSPIAVPVQDFSCVDAFDWGEKSLPVEEAIEDDWVLIEKPVLKRQ  320
QYU59218.1    241   FMGEIPIGNMCYGNTIAPPVATPNKVASKGKKAVKPSKPLLPAQDFSCVEDFDWGEKSLAID--IEDDWVLIEKPVIKRQ  318
ADJ39329.1    241   FAGEIPAGFFSYGKTVASPIATPKKVVTKRKRVSKPSTIAVPVQDFSCIDAFDWGEKSFPNEEAIEDDWVLVEKPVLKRQ  320
QBZ78354.1    241   FMGEIPIGNMCYGKTVASPTATLKKVASKGKKATKPSKPILPVQDFSCIEGFDWGEKSLSVD--IEDDWVLIEKPVIKRQ  318
QBZ78356.1    241   FMGEIPIGNMCYGKTVASPTATLKKVASKGKKATKPSKPILPVQDFSCIEGFDWGEKSLSVD--IEDDWVLIEKPVIKRQ  318
QYA72350.1    241   FAGEIPAGYFSYGKAVASPAAAFMKVVSKRKRAVKPSTPVPPVQDFSCVDEFDWGEKSLPVD--IEDDWILVEKPVLKRQ  318
ADJ39331.1    241   FAGEIPAGFFSYGKTVASPIATPKKVVSKRKRVSKPSTIAVPVQDFSCIDAFDWREKSLPVEEAIEDDWVLVEKPVLKRQ  320
DAZ85705.1    241   FAGEIPAGFFSYGNTVASPIATPKKVVSKRKRASKPSPIAAPVQDFSCIDAFDWGEKSLPIEEAIEDDWVLIEKPVLKRQ  320
DAZ85709.1    241   FAGEIPAGFFSYGKTVATPTATPKKVASKRKRTAKSSTNVTPVQDFSCIDAFDWGEKSFPVGEAIEDDWVLVEKPVLKRQ  320
DAZ85710.1    241   FAGEIPAGFFSYGKTVATPTATPKKVASKRKRTAKSSTNVTPVQDFSCIDAFDWGEKSFPVGEAIEDDWVLVEKPVLKRQ  320
DAZ85703.1    241   FAGEIPAGFFSYGKTVASPSATPKKVVSKRKRASKPSPVAVPMQDFSCIDAFDWGEKSFPVEEAIEDDWILIEKPVLKRQ  320
ADJ39330.1    241   FAGEIPAGFFSYGKTVASPIATPKKVVSKRKRVSKPSTIAVPVQDFSCIDAFDWGEKSFPNEEAIEDDWVLVEKPVLKRQ  320
ACF32434.1    241   FAGEMPVGFFSYGKTVAIPTATPKKVVSKRKRASKPSTVAVPVQDFSCIDAFDWGEKSFSVKEVIEDDWILIEKPVLKRQ  320
QYU59216.1    241   FAGEIPAGHAFY-SFLYNPTTTFKKVVSKRKRVVKISSTPV-VQDFSCIDSFDWGEKSFPSEE-IEDDWVLIEKPVLRRQ  317

YP_053925.1   318   AAHSAQGRATEALTRFAASGGFTVKAHQKVEELASSGEAGHLIAGEFAELCLRSLVYNDAPVLSASIEELITEQDFKDAI  397
AGT56093.1    318   AAHSAQGRATEALTRFAASGGFTVKAHQKVEELASSGEAGHLIAGEFAELCLRSLVYNDAPVLSASIEELITEQDFKDAI  397
QBZ78358.1    318   AAHSAQGRATEALTRFAASGGFTIKAHQKVEELASSGEAGHLIAGEFAELCLXXXXXXXXXVLSASIEELVTEQDFKDAI  397
AGD98722.1    319   AAHSVQGRATEALTRFAASSGFTVKAHQKVEDLASTSEAGHLIAGEFAELCLRSLVYNDAPVLSASIEELVTEQDFKDAI  398
DAZ85702.1    319   AAHSVQGRATEALTRFAASSGFTVKAHQKVEDLASISEASHLIAGEFAELCLRSLVYNDAPVLSASIEDLVTEQDFKDAI  398
QGY99255.1    319   AAHSVQGRATEALTRFAASSGFTTGAHQRVEELASSGEAGHLIAGEFAELCLRSLVYNDALTLSASIEELATEQDFKDAI  398
DAZ85701.1    319   AAHSVQGRATEALTRFAATSGFTLEAHQRVEELASSGEAGYLIAGEFAELCLRSLVYNDAPVLSASIEGLVVEQDFKDAI  398
DAZ85704.1    321   AAHSVQGRATEALTRFAASSGFTMKAHQKVEELAAKDAAGHLIAGEFADLCLRSLVYNDAPTLSASIEELACEQDFKDAI  400
QYU59218.1    319   AAHSVQGRAVEALTRFAASSGFTIKAHQRVEELASSGEAGCLIAGEFAELCLKSLAYNDAPVLSASIEELITEQDFEDAI  398
ADJ39329.1    321   AAHSVQGRATEALTRFAASSGFTTKAHQKVEELASSGEAGHLLAGEFAELCLRSLVYNDAPILSASIEVLACEQDFKDAI  400
QBZ78354.1    319   AAHSVQGRATEALTRFAASSGFTIGAHQRVEELASSGEAGHLVAGEFAELCLRSLTYNDAFVLSASIEELGTEQDFKDAI  398
QBZ78356.1    319   AAHSVQGRATEALTRFAASSGFTIGAHQRVEELASSGEAGHLVAGEFAELCLRSLTYNDALVLSASIEELGTEQDFKDAI  398
QYA72350.1    319   AAHSVQGRATEALTRFAASSGFTIKAHQRVEELAASNAAGHLIAGEFAELCLRSLVYNDAPVLSASIEELACEQDFKDAI  398
ADJ39331.1    321   AAHSAQGRATEALTRFAASSGFTVKAHQKVEELAAIDAASHLIAGEFADLCLRSLVYNDAPTLSASIEELACTQDFRDAI  400
DAZ85705.1    321   AVHSAQGRATEALTRFAASSGFTIKAHQKVEELAASDAASHLLAGEFAELCLRSLVYNDAPTLSASIEELACEQDFKDAI  400
DAZ85709.1    321   AAHSAQGRATEALTRFAASSGFTAEAHQKVEELAARDAAGHLIAGEFADPCLRSLVYNDAPLLSASIEELATEQDFKDAI  400
DAZ85710.1    321   AAHSAQGRATEALTRFAASSGFTAEAHQKVEELAARDAAGHLIAGEFADPCLRSLVYNDAPLLSASIEELATEQDFKDAI  400
DAZ85703.1    321   AAHSVQGRATEALTRFAASSGFTIKAHRKVEELAARDAASHLIAGEFAELCLRSLVYNDAPTLSASIEELACEQDFKDAI  400
ADJ39330.1    321   AAHSVQGRATEALTRFAASSGFTTKAHQKVEELAASGAAGHLIAGEFAELCLRSLVYNDAPTLSASIEELACEQDFKDAI  400
ACF32434.1    321   AVHSVQGRATEALTRFAASSGFTVKAHQKVEELALSGEAGHLIAGEFADLCLRSLVYNDAPVLSASIEELACEQDFKDAI  400
QYU59216.1    318   AAHTVQGRASEALVRFAATSGFTAGAHQKVEEFAASGAANHLIAGEFVDLCLRSLVYNDAPQLSTSIEELATEQDFKDAI  397

                                X1-X2 cleavage site

YP_053925.1   398   ELFNIELAELPTDSTTCGQFNDWASAAKKMAKGVGSIVGDFARMSGAGVLITFDRCIEYLQKKALTFCQKVFNATMAPYL  477
AGT56093.1    398   ELFNIELAELPTDSTTCGQFNDWASAAKKMAKGVGSIVGDFARMSGAGVLITFDRCIEYLQKKALTFCQKVFNATMAPYL  477
QBZ78358.1    398   ELFSVELAELPTDSTTCGQFNDWASAAKKMAKGVGSMVGDFARMSGAGVLITFDKCIEYLQEKALILCKKVFNATMAPYL  477
AGD98722.1    399   ELFNVELAELPTESTTCGRFNDWASTAKTMAKGVGSMVGDFARMSGAGVLITFDKCIEYLQEKALTLCQRVFNATMAPYL  478
DAZ85702.1    399   ELFNVELAELPTESTTCGRFNDWASTAKTMAKGVGSMVGDFARMSGAGVLITFDKCIEYLQEKALTLCQRVFNATMAPYL  478
QGY99255.1    399   ELFHIELAELPADSTTRGHFNAWATSAKTMARGVGNLVGDFARMSGALTLITFDKCIEYLQEKALTLCQKVFNATMAPYL  478
DAZ85701.1    399   ELFNIELAELPTDSTTRGRFNDWASAAKSMAKGVGSVVGDFARMSGAGVLITFDKCIEYLQERALTLCQKVFNATMAPYL  478
DAZ85704.1    401   ELFNIELAELPTDSTTCGRFNDWASSAKIMAKGVGSLVGDYSRLVGSGLVITYDKAIEFLQEKALTLCQKVFNATMAPYL  480
QYU59218.1    399   ELFHIELAELPTDSVTCGRFNAWAASAKTMAKGVGSLMGDLARMSGALTLITFDKCIEYLQEKALTLCQKVFNATMAPYL  478
ADJ39329.1    401   ELFNIELAEIPTDSTTCGRFNEWASSVKTMAKGVGSLVGDFARGAGTLTLITFDKAIEYLQEKALTLCQKVFNATMVPYL  480
QBZ78354.1    399   ELFHIELAELPTDSVTCGRFNAWAASAKTMAKGVGNLVGDFARLSGALTLITFDKCIEYLQEKALTLCQKVFNATMAPYL  478
QBZ78356.1    399   ELFHIELAELPTDSVTCGRFNAWAASAKTMAKGVGNLVGDFARLSGALTLITFDKCIEYLQEKALTLCQKVFNATMAPYL  478
QYA72350.1    399   ELFNIELAEIPTDSTTCGRFNEWASSAKTMAKGVGTLVGDLARGAGALTLITFDKAIEYLQEKALTLCQKVFNATMAPYL  478
ADJ39331.1    401   ELFNIELAELPTDSTTCGRFSDWAASAKSMAKGVGSLVGDLARGAGAGALITFDKCIEYLQEKALTLCQKVFNATMAPYM  480
DAZ85705.1    401   ELFNIELAELPTDSTTCGRFNDWASSVKTMAKGVGSLVGDYSRLVGSGLVITFDKALEFLQEKALILCQKVFNATMAPYL  480
DAZ85709.1    401   ELFNIELAELPTDSTICGRFNDWASSAKIMAKGVGSLVGDLARGVGAGALITFDKCIEYLQEKALTLCQKVFNATMAPYL  480
DAZ85710.1    401   ELFNIELAELPTDSTICGRFNDWASSAKIMAKGVGSLVGDLARGVGAGALITFDKCIEYLQEKALTLCQKVFNATMAPYL  480
DAZ85703.1    401   ELFNIELAEIPTDSTTCGRFNEWASSAKTIARGVGSLVGDFARGAGAMTLITFDKAIEYLQEKALTLCQKVFNATMAPYL  480
ADJ39330.1    401   ELFNIELAELPTDSTTCGRFNDWASAAKTMAKGTGSLVGDLARLAGAGLVITFDKAIEYLQEKALTLCQKVFNATMAPYV  480
ACF32434.1    401   ELFNIELAELPTDSTTCGRFNDWAASAKTMAKGVGSLVGDLARATGAGALITFDKCLEYLQEKALTLCQKVFNATMAPYL  480
QYU59216.1    398   ELFNIELAELPSDSTICGRFNDWAATAKTMARGVGSMVGDLGRLAGAGALITFDKCIEYLQERALTLCQKVFNATMAPYL  477
```

```
YP_053925.1  478   SHLAEASNIISKIWKKLAEWMESLKGKAGLALEVLAQHAIFALGAIVVGGVVVLVEKVLVACKVIPNCGIVLGAFLTLFF  557
AGT56093.1   478   SHLAEASNIISKIWKKLAEWMESLKGKAGLALEVLAQHAIFALGAIVVGGVVVLVEKVLVACKVIPNCGIVLGAFLTLFF  557
QBZ78358.1   478   SHLAEASNIXNKIWKKLAEWMESLKGKAGLALEVLAQHAIFALGAIVVGGVVVLVEKVLVACKIIPSCGIVLGAFLTLFF  557
AGD98722.1   479   SHLAEASNIISKIWKKLAEWMESLKGKAGLALEVLAQHAIFALGAIVVGGVVVLVEKVLVACKIIPSCGIVLGAFLTLFF  558
DAZ85702.1   479   SHLAEASNIISKIWKKLAEWMESLKGKAGLALEVLAQHAIFALGAIVVGGVVVLVEKVLVACKIIPSCGIVLGAFLTLFF  558
QGY99255.1   479   SHLTEASNIINKIWKKLGEWMESLKGKAGLALEVLAQHAIFALGAIVVGGVVVLVEKVLVACKIIPSCGIVLGAFLTLFF  558
DAZ85701.1   479   SHLAEASNIINKIWKKLAEWMESLKGKAGLALEVLAQHAIFALGAIVVGGVVVLVEKVLVACKIIPSCGIVLGAFLTLFF  558
DAZ85704.1   481   SHLTEASNIIKKIWKKLSEWMESLKGKAGLALEVLAHHAIFALGAIVVGGVVVLVEKVLVACKIIPTCGIVLGAFLTLFF  560
QYU59218.1   479   SHLVEASNIINKIWKKLAEWMESLKGKAGLALEVLAQHAIFALGAIVVGGVVVLVEKVLVACKIIPSCGIVLGAFLTLFF  558
ADJ39329.1   481   SHLAEASDIIKKIWKKLSEWMESLKGKAGLALEVLAHHAIFALGAIVVGGVVVLVEKVLVACKVIPTCGIVLGAFLTLFF  560
QBZ78354.1   479   SHLAEASNIINKIWKKLAEWMESLKGKAGLALEVLAQHAIFALGAIVVGGVVVLVEKVLVACKIIPSCGIVLGAFLTLFF  558
QBZ78356.1   479   SHLAEASNIINKIWKKLAEWMESLKGKAGLALEVLAQHAIFALGAIVVGGVVVLVEKVLVACKIIPSCGIVLGAFLTLFF  558
QYA72350.1   479   SHLTEASNIIKKIWKKLSEWMESLKGKAGLALEVLAHHAIFALGAIVVGGVVVLVEKVLVACKVIPSCGIVLGAFLTLFF  558
ADJ39331.1   481   SHLTEASNIIKQIWKKLSEWMESLKGKAGLALEVLAHHAIFALGAIVVGGVVVLVERVLVYCKVIPCCGIVLGAFLTLFF  560
DAZ85705.1   481   HHLTEASNIIKKIWTKLGEWMESLKGKAGLALEVLAHHAIFALGAIVVGGVVVLVEKVLVACKIIPTCGIVLGAFLTLFF  560
DAZ85709.1   481   SHLTEASNIIKKIWKKLSEWMESLKGKAGLALEVLAHHAIFALGAIVVGGVVVLVEKVLVACKVIPSCGIVLGAFLTLFF  560
DAZ85710.1   481   SHLTEASNIIKKIWKKLSEWMESLKGKAGLALEVLAHHAIFALGAIVVGGVVVLVEKVLVACKVIPSCGIVLGAFLTLFF  560
DAZ85703.1   481   SHLTEASNIIKKIWRKLSEWMESLKGKAGLALEVLAHHAIFALGAIVVGGVVVLVEKVLVACKVIPTCGIVLGAFLTLFF  560
ADJ39330.1   481   SHLSEASDIIKKIWKKLSEWMESLKGKAGLALEVLAHHAIFALGAIVVGGVVVLVEKVLVATKVIPTCGIVLGAFLTLFF  560
ACF32434.1   481   SHLAEASNIIKKIWKKLGEWMESLKGKAGLALEVLAHHAIFALGAIVVGGVIVLVEKVLVVCKVIPSCGIVLGAFLTLFF  560
QYU59216.1   478   SHLSEAFHPFTELLPNFIILEASLKGKAGLALEVLAQHAIFALGAIVVGGAVVLVEKVLVACKIIPTCGIVLGAFLTLFF  557

                                                               X2-NTB cleavage site

YP_053925.1  558   ASLGLTALECTAEEIFRMHQCCKGAIYSMYSVKEPMNEAEGSSVTMGVLQGLDNAISALTRVGQSMISFKLGSFSYYAKI  637
AGT56093.1   558   ASLGLTALECTAEEIFRMHQCCKGAIYSMYSVKEPMNEAEGSSVTMGVLQGLDNAISALTRVGQSMISFKLGSFSYYAKI  637
QBZ78358.1   558   ASLGLTALECTAEEIFRMHQCCKGAIYNMYSVKEPMNEGEGSSTTMGVLQGLDNAVSALTRVGQSMISFKLGSFSYYAKI  637
AGD98722.1   559   ASLGLTALECTAEEIFRMHQCCKGAIYSMYSVKEPMSEAEGSSITMGVLQGLDNAVSALTRVGQSMISFKLGSFSYYAKI  638
DAZ85702.1   559   ASLGLTALECTAEEIFRMHQCCKGAIYSMYSVKEPTSETEGSSVTMGVLQGLDNAVSALTRVGQSMISFKLGSFSYYAKI  638
QGY99255.1   559   ASLGLTALECTAEEIFRMHQCCKGAIYSMYSVKEPTCEGEDSSAPMGVLQGLDNAVSALTRVGQSMISFKLGSFSYYAKI  638
DAZ85701.1   559   ASLGLTALECTAEEIFRMHQCCKGAIYSMYSVKEPVCEGESSSTPMGVLQGLDNAVSALTRVGQSMISFKLGSFSYYAKI  638
DAZ85704.1   561   ASLGLTALECTAEEIFKMHQCCKGAIYSMYSVKEPVCEGEGDSTTKGVLQGLDNAISALTRVGQSMISFKLGSFSYYAKI  640
QYU59218.1   559   ASLGLTALECTAEEIFRMHQCCKGAIYSMYSVKEPVCEGENSSTTMGVLQGLDNAISALTRVGQSMISFKLGSFSYYAKI  638
ADJ39329.1   561   ASLGLTALECTADEIFKMHQCCKGAIYSMYSVKEPVCEGEGDSTTKGVLQGLDNAISALTRVGQSMISFKLGSFSYYAKI  640
QBZ78354.1   559   ASLGLTALECTAEEIFRMHQCCKGAIYSMYSVKEPVCEGENSSAPMGVLQGLDNAVSALTRVGQSMISFKLGSFSYYAKI  638
QBZ78356.1   559   ASLGLTALECTAEEIFRMHQCCKGAIYSMYSVKEPVCEGENSSAPMGVLQGLDNAVSALTRVGQSMISFKLGSFSYYAKI  638
QYA72350.1   559   ASLGLTALECTADEIFKMHQCCKGAIYSMYSVKEPVCEGEGDSTTKGVLQGLDNAISALTRVGQSMISFKLGSFSYYAKI  638
ADJ39331.1   561   ASLGLTALECTADEIFKMHQCCKGAIYSMYSVKEPVCEGEGDSTTKGVLQGLDNAISALTRVGQSMISFKLGSFSYYAKI  640
DAZ85705.1   561   ASLGLTALECTADEIFKMHQCCKGAIYSMYSVKEPVCEGEGDSTTKGVLQGLDNAISALTRVGQSMISFKLGSFSYYAKI  640
DAZ85709.1   561   ASLGLTALECTADEIFKMHQCCKSAIYSMYSVKEPVCEGEGDSTTKGVLQGLDNAISALTRVGQSMISFKLGSFSYYAKI  640
DAZ85710.1   561   ASLGLTALECTADEIFKMHQCCKSAIYSMYSVKEPVCEGEGDSTTKGVLQGLDNAISALTRVGQSMISFKLGSFSYYAKI  640
DAZ85703.1   561   ASLGLTALECTADEIFKMHQCCKGAIYSMYSVKEPVCEGEGDSTTKGVLQGLDNAISALTRVGQSMISFKLGSFSYYAKI  640
ADJ39330.1   561   ASLGLTALECTADEIFKMHQCCKGAIYSMYSVKEPVCEGEGDSTTKGVLQGLDNAISALTRVGQSMISFKLGSFSYYAKI  640
ACF32434.1   561   ASLGLTALECTADEIFKMHQCCKGAIYSMYSVKEPVCEGEGDSTTRGVLQGLDNAISALTRVGQSMISFKLGSFSYYAKI  640
QYU59216.1   558   ASLGLTALEYTADEIFKMHQCCKGAIYSMYSVKEPVCEGEGDSTTRGVLQGLDNAISALTRVGQSMISFKLGSFSYYAKI  637

YP_053925.1  638   AQGFDQLARGKKAIGELTGWLIDLVGGVYSKVSGQESTFFDELSTIVCLDVRSWLLKSKRVRLQVETMAIGDRITLDTIS  717
AGT56093.1   638   AQGFDQLARGKKAIGELTGWLIDLVGSVYSKVSGQESTFFDELSTIVCLDVRSWLLKSKRVRLQVETMAIGDRITLDTIS  717
QBZ78358.1   638   AQGFDQLARGKKAIGELTGWLIDLIGSVYSQVSGQESTFFDELSTIVCLDVRSWLLKSKRVRLQVETMAIGDRITLDTIS  717
AGD98722.1   639   AQGFDQLARGKKAIGELTSWLIDLVGSVYSKVSGQESTFFDELSTIVCLDVRSWLLKSKRVRLQVETMAIGDRITLDTIS  718
DAZ85702.1   639   AQGFDQLARGKKAIGELTSWLIDLVGSVYSQVSGQESTFFDELSTIVCLDVRSWLLKSKRVRLQVETMAIGDRITLDTIS  718
QGY99255.1   639   AQGFDQLARGKKAIGELTSWLIDLVGSVYSKVSGQESTFFDELSTIVCLDVRSWLLKSKRVRLQVETMAIGDRITLDTIS  718
DAZ85701.1   639   AQGFDQLARGKRAIGELTSWLIDLIGSVYSKVSGQESTFFDELSTIVCLDVRSWLLKSKRVRLQVETMAIGDRITLDTIS  718
DAZ85704.1   641   AQGFDQLARGKKAIGELTSWLIDLVGSVYSKVSGQESTFFDELSTIVCLDVRSWLLKSKRVRLQVETMAIGDRITLDTIS  720
QYU59218.1   639   AQGFDQLARGKKAIGELTSWLIDLVGSVYSKVSGQESTFFDELSTIVCLDVRSWLLKSKRVRLQVETMAIGDRITLDTIS  718
ADJ39329.1   641   AQGFDQLARGKRAIGELTSWLIDLVGSVYSKVSGQESTFFDELSTIVCLDVRSWLLKSKRVRLQVETMAIGDRITLDTIS  720
QBZ78354.1   639   AQGFDQLARGKKAIGELTSWLIDLIGSVYSKVSGQESTFFDELSTIVCLDVRSWLLKSKRVRLQVETMAIGDRITLDTIS  718
QBZ78356.1   639   AQGFDQLARGKKAIGELTSWLIDLIGSVYSKVSGQESTFFDELSTIVCLDVRSWLLKSKRVRLQVETMAIGDRITLDTIS  718
QYA72350.1   639   AQGFDQLARGKRAIGELTSWLIDLVGSVYSKVSGQESTFFDELSTIVCLDVRSWLLKSKRVRLQVETMAIGDRITLDTIS  718
ADJ39331.1   641   AQGFDQLARGKRAIGELTSWLIDLVGSVYSKVSGQESTFFDELSTIVCLDVRSWLLKSKRVRLQVETMAIGDRITLDTIS  720
DAZ85705.1   641   AQGFDQLARGKRAIGELTSWLIDLVGSVYSKVSGQESTFFDELSTIVCLDVRSWLLKSKRVRLQVETMAIGDRITLDTIS  720
DAZ85709.1   641   AQGFDQLARGKRAIGELTSWLIDLVGSVYSKVSGQESTFFDELSTIVCLDVRSWLLKSKRVRLQVETMAIGDRITLDTIS  720
DAZ85710.1   641   AQGFDQLARGKRAIGELTSWLIDLVGSVYSKVSGQESTFFDELSTIVCLDVRSWLLKSKRVRLQVETMAIGDRITLDTIS  720
DAZ85703.1   641   AQGFDQLARGKRAIGELTSWLIDLVGSVYSKVSGQESTFFDELSTIVCLDVRSWLLKSKRVRLQVETMAIGDRITLDTIS  720
ADJ39330.1   641   AQGFDQLARGKRAIGELTSWLIDLVGSVYSKVSGQESTFFDELSTIVCLDVRSWLLKSKRVRLQVETMAIGDRITLDTIS  720
ACF32434.1   641   AQGFDQLARGKRAIGELASWLIDLVGSVYSKVSGQESTFFDELSTIVCLDVRSWLLKSKRVRLQVETMAIGDRITLDTIS  720
QYU59216.1   638   AQGFDQLARGKRAIGELTSWLIDLVGSVYSKVSGQESTFFDELSTIVCLDVRSWLLKSKRVRLQVETMAIGDRITLDTIS  717
```

```
YP_053925.1  718   KPTGMQGHKILITAAGVPRKTSADFTMCIKEEVSKLEEVHQRTACAGINEGMRQFPFWVYIFGASQSGKTTIANSVIIPS  797
AGT56093.1   718   KLLE-EGHKILVTAAGVPRKTSADFTMCIKEEVSKLEEVHQRTACAGINEGMRQFPFWVYIFGASQSGKTTIANSVIIPS  796
QBZ78358.1   718   KLLE-EGHKILVTAAGVPRKTSADFTMCIKEEVSKLEEVHQRTACAGINEGMRPFPFWVYIFGASXXXKTTIANSVIIPS  796
AGD98722.1   719   KLLE-EGHKILVTAAGVPRKTSADFTMCIKEEVSKLEEVHQRTACAGINEGMRQFPFWVYIFGASQSGKTTIANSVIIPS  797
DAZ85702.1   719   KLLE-EGHKILVTAAGVPRKTSADFTMCIKEEVSKLEEVHQRTACAGINEGMRQFPFWVYIFGASQSGKTTIANSVIIPS  797
QGY99255.1   719   KLLE-EGHKILVTAAGVPRKTSADFTMCIKEEVSKLEEVHQRTACAGINEGMRQFPFWVYIFGASQSGKTTIANSVIIPS  797
DAZ85701.1   719   KLLE-EGHKILVTAAGVPRKTSADFTMCIKEEVSKLEEVHQRTACAGINEGMRQFPFWVYIFGASQSGKTTIANSVIIPS  797
DAZ85704.1   721   KLLE-EGHKILVTAAGVPRKTSADFTMCIKEEVSKLEEVHQRTACAGINEGMRQFPFWVYIFGTSQSGKTTIANSVIIPS  799
QYU59218.1   719   KLLE-EGHKILVTAAGVPRKTSADFTMCIKEEVSKLEEVHQRTACAGINEGMRQFPFWVYIFGASQSGKTTIANSVIIPS  797
ADJ39329.1   721   KLLE-EGHKILVTAAGVPRKTSADFTMCIKEEVSKLEEVHQRTACAGINEGMRQFPFWVYIFGASQSGKTTIANSVIIPS  799
QBZ78354.1   719   KLLE-EGHKILVTAAGVPRKTSADFTMCIKEEVSKLEEVHQRTACAGINEGMRQFPFWVYIFGASQSGKTTIANSVIIPS  797
QBZ78356.1   719   KLLE-EGHKILVTAAGVPRKTSADFTMCIKEEVSKLEEVHQRTACAGINEGMRQFPFWVYIFGASQSGKTTIANSVIIPS  797
QYA72350.1   719   KLLE-EGHKILVTAAGVPRKTSADFTMCIKEEVSKLEEVHQRTACAGINEGMRQFPFWVYIFGASQSGKTTIANSVIIPS  797
ADJ39331.1   721   KLLE-EGHKILVTAAGVPRKTSADFTMCIKEEVSKLEEVHQRTACAGINEGMRQFPFWVYIFGASQSGKTTIANSVIIPS  799
DAZ85705.1   721   KLLE-EGHKILVTAAGVPRKTSADFTMCIKEEVSKLEEVHQRTACAGINEGMRQFPFWVYIFGTSQSGKTTIANSVIIPS  799
DAZ85709.1   721   KLLE-EGHKILVTAAGVPRKTSADFTMCIKEEVSKLEEVHQRTACAGINEGMRQFPFWVYIFGASQSGKTTLANSVIIPS  799
DAZ85710.1   721   KLLE-EGHKILVTAAGVPRKTSADFTMCIKEEVSKLEEVHQRTACAGINEGMRQFPFWVYIFGASQSGKTTLANSVIIPS  799
DAZ85703.1   721   KLLE-EGHKILVTAAGVPRKTSADFTMCIKEEVSKLEEVHQRTACAGINEGMRQFPFWVYIFGTSQSGKTTIANSVIIPS  799
ADJ39330.1   721   KLLE-EGHKILVTAAGVPRKTSADFTMCIKEEVSKLEEVHQRTACAGINEGMRQFPFWVYIFGASQSGKTTIANSVIIPS  799
ACF32434.1   721   KLLE-EGHKILVTAAGVPRKTSADFTMCIKEEVSKLEEVHQRTACAGINEGMRQFPFWVYIFGTSQSGKTTIANSVIIPS  799
QYU59216.1   718   KLLE-EGHKILVTAAGVPRKTSADFTMCIKEEVSKLEEVHQRTACAGINEGMRQFPFWVYIFGASQSGKTTIANSVIIPS  796

YP_053925.1  798   LLEEMNLPKTSVYSRPKTGGFWSGYARQACVKVDDFYAIEQTPSLASSMIDVVNSEPYPLDMAYLHEKGMSMDSPLVVTT  877
AGT56093.1   797   LLEEMNLPKTSVYSRPKTGGFWSGYARQACVKVDDFYAIEQTPSLASSMIDVVNSEPYPLDMAYLHEKGMSMDSPLVVTT  876
QBZ78358.1   797   LLEEMNLPKTSVYSRPKTGGFWSGYARQACVKVDDFYAIEQTPSLASSMIDVVNSEPYPLDMAYLHEKGMSMDSPLVVTT  876
AGD98722.1   798   LLEEMNLPKTSVYSRPKTGGIWSGYARQACVKVDDFYAIEQTPSLASSMIDVVNSEPYPLDMAYLHEKGMSMDSPLVVTT  877
DAZ85702.1   798   LLEEMNLPKTSVYSRPKTGGFWSGYARQACVKVDDFYAIEQTPSLASSMIDVVNSEPYPLDMAYLHEKGMSMDSPLVVTT  877
QGY99255.1   798   LLEEMNLPKTSVYSRPKTGGFWSGYARQACVKVDDFYAIEQTPSLASSMIDVVNSEPYPLDMAYLHEKGMSMDSPLVVTT  877
DAZ85701.1   798   LLEEMNLPKTSVYSRPKTGGFWSGYARQACVKIDDFYAIEQTPSLASSMIDVVNSEPYPLDMAYLHEKGMSMDSPLVVTT  877
DAZ85704.1   800   LLEEMNLPKTSVYSRPKTGGFWSGYARQACVKVDDFYAIEQTPSLASSMIDVVNSEPYPLDMAYLHEKGMSMDSPLVVTT  879
QYU59218.1   798   LLEEMNLPKTSVYSRPKTGGFWSGYARQACVKVDDFYAIEQTPSLASSMIDVVNSEPYPLDMAYLHEKGMSMDSPLVVTT  877
ADJ39329.1   800   LLEEMNLPKTSVYSRPKTGGFWSGYARQACVKVDDFYAIEQTPSLASSMIDVVNSEPYPLDMAYLHEKGMSMDSPLVVTT  879
QBZ78354.1   798   LLEEMNLPKTSVYSRPKTGGFWSGYARQACVKVDDFYAIEQTPSLASSMIDVVNSEPYPLDMAYLHEKGMSMDSPLVVTT  877
QBZ78356.1   798   LLEEMNLPKTSVYSRPKTGGFWSGYARQACVKVDDFYAIEQTPSLASSMIDVVNSEPYPLDMAYLHEKGMSMDSPLVVTT  877
QYA72350.1   798   LLEEMNLPKTSVYSRPKTGGFWSGYARQACVKVDDFYAIEQTPSLASSMIDVVNSEPYPLDMAYLHEKGMSMDSPLVVTT  877
ADJ39331.1   800   LLEEMNLPKTSVYSRPKTGGFWSGYARQACVKVDDFYAIEQTPSLASSMIDVVNSEPYPLDMAYLHEKGMAMDSPLVVTT  879
DAZ85705.1   800   LLEEMNLPKTSVYSRPKTGGFWSGYARQACVKVDDFYAIEQTPSLASSMIDVVNSEPYPLDMAYLHEKGMSMDSPLVVTT  879
DAZ85709.1   800   LLEEMNLPKTSVYSRPKTGGFWSGYARQACVKVDDFYAIEQTPSLASSMIDVVNSEPYPLDMAYLHEKGMSMDSPLVVTT  879
DAZ85710.1   800   LLEEMNLPKTSVYSRPKTGGFWSGYARQACVKVDDFYAIEQTPSLASSMIDVVNSEPYPLDMAYLHEKGMSMDSPLVVTT  879
DAZ85703.1   800   LLEEMNLPKTSVYSRPKTGGFWSGYARQACVKVDDFYAIEQTPSLASSMIDVVNSEPYPLDMAYLHEKGMSMDSPLVVTT  879
ADJ39330.1   800   LLEEMNLPKTSVYSRPKTGGFWSGYARQACVKVDDFYAIEQTPSLASSMIDVVNSEPYPLDMAYLHEKGMSMDSPLVVTT  879
ACF32434.1   800   LLEEMNLPKTSVYSRPKTGGFWSGYARQACVKVDDFYAIEQTPSLASSMIDVVNSEPYPLDMAYLHEKGMSMDSPLVVTT  879
QYU59216.1   797   LLEEMNLPKTSVYSRPKTGGFWSGYARQACVKVDDFYAIEQTPSLASSMIDVVNSEPYPLDMAYLHEKGMSMDSPLVVTT  876

YP_053925.1  878   ANTVKPPTNAGITDEASFFNRRAAVIEVRRKDNTHFTPRAYDNCIEVRFLHNKCAYVDSEGIPQGPAVNTPMEEGWISPS  957
AGT56093.1   877   ANTVKPPTNAGITDEASFFNRRAAVIEVRRKDNTHFTPRAYDNCIEVRFLHNKCAYVDSEGIPQGPAVNTPMEEGWISPS  956
QBZ78358.1   877   ANTVKPPTNAGITDEASFFNRRAAVIEVRRKDNTFFTPRAYDNCIEVRFFNNKCAYIDSEGVPQGPAVNTPMEEGWISPS  956
AGD98722.1   878   ANTVKPPTNAGITDEDSFFNRRAAVIEVRRKDNTHFTPRAYDNCIEVRFLHNKCAYVDSEGVPQGPAVNTPMEEGWVSPS  957
DAZ85702.1   878   ANTVKPPTNAGITDEASFFNRRAAVIEVRRKDNTHFTPRAYDNCIEVRFLHNKCAYVDSEGVPQGPAVNTPMGEGWISPS  957
QGY99255.1   878   ANTVKPPTNAGITDEASFFNRRAAVIEVRRKDNTHFTPRAYDNCIEVRFLHNKCAYVDSEGVPQGPAVNTPMEEGWISPS  957
DAZ85701.1   878   ANTVKPPTNAGITDEASFFNRRAAVIEVRRKDNTHFTPRAYDNCIEVRFLHNKCAYVDSEGIPQGPAVNTPMEEGWISPS  957
DAZ85704.1   880   ANTVKPPTNAGITDEASFFNRRAAVIEVRRKDNTHFTPRAYDNCIEVRFLHNKCAYVDSEGVPQGPAVNTPMEEGWVSPS  959
QYU59218.1   878   ANTVKPPTNAGITDEASFFNRRAAVIEVRRKDNTHFTPRAYDNCIEVRFLHNKCAYVDSEGVPQGPAVNTPLEEGWISPS  957
ADJ39329.1   880   ANTVKPPTNAGITDEASFFNRRAAVIEVRRKDNTHFTPRAYDNCIEVRFLHNKCAYVDSGGVPQGPAVNTPMEEGWVSPS  959
QBZ78354.1   878   ANTVKPPTNAGITDEASFFNRRAAVIEVRRKDNTHFTPRAYDNCIEVRFLHNKCAYVDSEGIPQGPAVNTPMEEGWISPS  957
QBZ78356.1   878   ANTVKPPTNAGITDEASFFNRRAAVIEVRRKDNTHFTPRAYDNCIEVRFLHNKCAYVDSEGIPQGPAVNTPMEEGWISPS  957
QYA72350.1   878   ANTVKPPTNAGITDEASFFNRRAAVIEVRRKDNTHFTPRAYDDCIEVRFLHNKCAYIDSEGVPQGPAVNTPMEEGWVSPS  957
ADJ39331.1   880   ANTVKPPTNAGITDEASFFNRRAAVIEVRRKDNTHFTPRAYDNCIEVRFLHNKCAYIDSEGVPQGPAVNTPMEEGWVSPS  959
DAZ85705.1   880   ANTVKPPTNAGITDEASFFNRRAAVIEVRRKDNTHFTPRAYDNCIEVRFLHNKCAYIDSEGVPQGPAVNTPMEEGWVSPS  959
DAZ85709.1   880   ANTVKPPTNAGITDEASFFNRRAAVIEVRRKDNTHFTPRAYDNCIEVRFLHNKCAYVDSEGVPQGPAVNTPMEEGWVSPS  959
DAZ85710.1   880   ANTVKPPTNAGITDEASFFNRRAAVIEVRRKDNTHFTPRAYDNCIEVRFLHNKCAYVDSEGVPQGPAVNTPMEEGWVSPS  959
DAZ85703.1   880   ANTVKPPTNAGITDEASFFNRRAAVIEVRRKDNTHFTPRAYDNCIEVRFLHNKCAYIDSEGVPQGPAVNTPMEEGWVSPS  959
ADJ39330.1   880   ANTVKPPTNAGITDEASFFNRRAAVIEVRRKDNTHFTPRAYDNCIEVRFLHNKCAYVNSEGVPQGPAVNTPMEEGWVSPS  959
ACF32434.1   880   ANTVKPPTNAGITDEASFFNRRAAVIEVRRKDNTHFTPRAYDNCIEVRFLHNKCAYVDSEGVPQGPTVNTPMEEGWISPS  959
QYU59216.1   877   ANTVKPPTNAGITDEASFFNRRAAVLEVRRKDNTHFTPRAYDNCIEVRFLHNKCAYVNAEGVPQGPAVNTPMEEGWVSPS  956
```

```
YP_053925.1  958   EAVATLKNLLGEHVLAEEEKLLDYRERIGNDHPIYNAAQEFIGNMHYPGQWLTTEQKNTYGINEEGFSFLAVDGKMYKYN  1037
AGT56093.1   957   EAVATLKNLLGEHVLAEEEKLLDYRERIGNDHPIYNAAQEFIGNMHYPGQWLTTEQKNTYGINEEGFSFLAVDGKMYKYN  1036
QBZ78358.1   957   EAVATLKNLLGEHVLAEEEKMLDYRERIGNDHPIYNAAQEFIGNMHYPGQWLTTEQKNTYGINEEGFSFLAVDGKMYKYN  1036
AGD98722.1   958   EAVAVLKNLLGEHVLAEEEKLLDYRERIGNDHPIYNAAQEFIGNMHYPGQWLTTEQKNTYGINEEGFSFLAVDGKMYKYN  1037
DAZ85702.1   958   EAVATLKNLLGEHVLAEEEKLLDYRERIGNDHPIYNAAQEFIGNMHYPGQWLTTEQKNTYGINEEGFSFLAVDGKMYKYN  1037
QGY99255.1   958   EAVATLKNLLGEHVLAEEEKLLDYRERIGNDHPIYNAAQEFIGNMHYPGQWLTTEQKNTYGINEEGFSFLAVDGKMYKYN  1037
DAZ85701.1   958   EAVASLKNLLGEHVLAEEEKLLDYRERIGNDHPIYNAAQEFIGNMHYPGQWLTTEQKNTYGINDEGFSFLAVDGRMYRYN  1037
DAZ85704.1   960   EAVATLKNLLGEHVLAEEEKLLDYRERIGNDHPIYNAAQEFIGNMHYPGQWLTTEQKNTYGINDEGFSFLAVDGKMYKYN  1039
QYU59218.1   958   EAVATLKNLLGEHVLAEEEKLLDYRERIGNDHPIYNAAQEFIGNMHYPGQWLTTEQKNTYGINEEGFSFLAVDGKMYKYN  1037
ADJ39329.1   960   EAVATLKNLLGEHVLAEEEKLLDYRERIGNDHPIYNAAQEFIGNMHYPGQWLTTEQKNTYGINEEGFSFLAVDGKMYKYN  1039
QBZ78354.1   958   EAVATLKNLLGEHVLAEEEKLLDYRERIGNDHPIYNAAQEFIGNMHYPGQWLTTEQKNTYGINDEGFSFLAVDGKMYRYN  1037
QBZ78356.1   958   EAVATLKNLLGEHVLAEEEKLLDYRERIGNDHPIYNAAQEFIGNMHYPGQWLTTEQKNTYGINDEGFSFLAVDGRMYRYN  1037
QYA72350.1   958   EAVATLKNLLGEHVLAEEEKLLDYRERIGNDHPIYNAAQEFIGNMHYPGQWLTTEQKNTYGINDEGFSFLAVDGKMYKYN  1037
ADJ39331.1   960   EAVATLKNLLGEHVLAEEEKLLDYRERIGNDHPIYNAAQEFIGNMHYPGQWLTTEQKNTYGINEEGFSFLAVDGKMYKYN  1039
DAZ85705.1   960   EAVATLKNLLGEHVLAEEEKLLDYRERIGNDHPIYNAAQEFIGNMHYPGHWITTEDKNTYGIKDEGFSFLAVDGKMYKYN  1039
DAZ85709.1   960   EAVATLKNLLGEHVLAEEEKLLDYRERIGNDHPIYNAAQEFIGNMHYPGQWLTTEQKNTYGINDDGFSFLAVDGKMYKYN  1039
DAZ85710.1   960   EAVATLKNLLGEHVLAEEEKLLDYRERIGNDHPIYNAAQEFIGNMHYPGQWLTTEQKNTYGINDDGFSFLAVDGKMYKYN  1039
DAZ85703.1   960   EAVATLKNLLGEHVLAEEEKLLDYRERIGNDHPIYNAAQEFIGNMHYPGHWITTEDKNTYGIKDEGFSFLAVDGKMYKYN  1039
ADJ39330.1   960   EAVATLKNLLGEHVLAEEEKLLDYRERIGNDHPIYNAAQEFIGNMHYPGHWITTEDKNTYGIKDEGFSFLAVDGKMYKYN  1039
ACF32434.1   960   EAVATLKNLLGEHVLAEEEKLMDYRERIGNDHPIYNAAQEFIGNMHYPGEWIDADRKKTYEIEDEGFSFLAVDGKMYKYN  1039
QYU59216.1   957   EAVATLKNLLGEHVLAGEEKLLDYRERIGNDHPIYNAAQEFIGNMHYPGQWLTTEQKNTYGINEEGFSFLAVDGKMYKYN  1036


YP_053925.1  1038  VLGKLNPCETVPPHPNVIPWLEEKTLSIVHWDAHKHIATGPRNALVSCFLQGLVQDQSRVQSVDLMGKDSSPEQQAFFKR  1117
AGT56093.1   1037  VLGKLNPCETVPPHPNVIPWLEEKTLSIVHWDAHKHIATGPRNALVSCFLQGLVQDQSRVQSVDLMGKDSSPEQQAFFKR  1116
QBZ78358.1   1037  VLGKLNPCETVPSHPNVVPWLEEKTLSIVHWDVHKHIATGPRNALVSCFLQGLVQDQSRVQSVDLMGKDSSPEQQAFFKR  1116
AGD98722.1   1038  VLGKLNPCETIPPHPNVVPWLEEKTLSIVHWDVHKHIATGPRNALVSCFLQGLVQDQSRVQSVDLMGKDSSPEQQAFFKR  1117
DAZ85702.1   1038  VLGKLNPCMTVPPHPNVIPWLEEKTLTIVHWDVHKHIATGPRNALVSCFLQGLVQDQSRVQSVDLMGKDSSPEQQAFFKR  1117
QGY99255.1   1038  VLGKLNPCETVPPHPNVIPWLEEKTLSIVHWDVHKHIATGPRNALVSCFLQGLVQDQSRVQSVELMGKDSSPEQQAFFKR  1117
DAZ85701.1   1038  VLGKLNPCETVPPHPNVIPWLEEKTLSIVHWDVHKHIATGPRNALVSCFLQGLVQDQSRVQSVDLMGKDSSPEQQAFFKR  1117
DAZ85704.1   1040  VLGKLNPCETVPPHPNVIPWLEEKTLTIVHWDVHKHIATGPRNALVSCFLQGLVQDQSRVQSVELMGKDSSPEQQAFFKR  1119
QYU59218.1   1038  VLGKLNPCETVPPHPNVIPWLEEKTLSIVHWDVHKHIATGPRNALVSCFLQGLVQDQSRVQSVDLMGKDSSPEQQAFFKR  1117
ADJ39329.1   1040  VLGKLNPCETTPPHPNVIPWLEEKTLTVVHWDVHKHIATGPRNALVSCFLQGLVQDQSRVQSVELMGKDSSPEQQAFFKR  1119
QBZ78354.1   1038  VLGKLNPCETVPPHPNVIPWLEEKTLSIVHWDVHKHIATGPRNALVSCFLQGLVQDQSRVQSVDLMGKDSSPEQQAFFKR  1117
QBZ78356.1   1038  VLGKLNPCETVPPHPNVIPWLEEKTLSIVHWDVHKHIATGPRNALVSCFLQGLVQDQSRVQSVDLMGKDSSPEQQAFFKR  1117
QYA72350.1   1038  VLGKLNPCEAIPPHPNVIPWLEQKTLTIVHWDVHKHIATGPRNALVSCFLQGLVQDQSRVQSVELMGKDSSPEQQAFFKR  1117
ADJ39331.1   1040  VLGKLNPCATEPPHPNVIPWLEEKTLTMVHWDVHKHIATGPRNALVSCFLQGLVQDQSRVQSVELMGKDSSPEQQAFFKR  1119
DAZ85705.1   1040  VLGKLNPCEVAPPHPNVIPWLEEKTLTMVHWDVHKHIATGPRNALVSCFLQGLVQDQSRVQSVELMGKDSSPEQQAFFKR  1119
DAZ85709.1   1040  VLGKLNPCETVPPHPNVIPWLEEKTLTIVHWDVHKHIATGPRNALVSCFLQGLVQDQSRVQSVELMGKDSSPEQQAFFKR  1119
DAZ85710.1   1040  VLGKLNPCETVPPHPNVIPWLEEKTLTIVHWDVHKHIATGPRNALVSCFLQGLVQDQSRVQSVELMGKDSSPEQQAFFKR  1119
DAZ85703.1   1040  VLGKLNPCEVAPPHPNVIPWLEEKTLTMVHWDVHKHIATGPRNALVSCFLQGLVQDQSRVQSVELMGKDSSPEQQAFFKR  1119
ADJ39330.1   1040  VLGKLNPCEVVPPHPNVIPWLEEKTLTMVHWDVHKHIATGPRNALVSCFLQGLVQDQSRVQSVELMGKDSSPEQQAFFKR  1119
ACF32434.1   1040  VLGKLNPCAVPPTHPNVIPWLEERTLDKVHWDVHKHIATGPRNALVACFLQGLVQDQSYVQSVEIMGKDSSPEQQAFFKR  1119
QYU59216.1   1037  VLGKLNPCETTPPHPNVVPWLEEKTLSIVHWDVHKHIATGPRNALVSCFLQGLVQDQSRVQSVELMGKDSSPEQQAFFKR  1116


YP_053925.1  1118  LTLSERIYLRLCQIRIDAVKKEQLSSVSRGALDVLRDCMYKSKAKLVENYSLLLTLVAILVLIATAYSLISTLIGLAGCS  1197
AGT56093.1   1117  LTLSERIYLRLCQIRIDAVKKEQLSSVSRGALDVLRDCMYKSKAKLVENYSLLLTLVAILVLIATAYSLISTLIGLAGCS  1196
QBZ78358.1   1117  LTLSERIYLRLCQIRIDAVKKEQLSGVSRGALDVLRDCMHKSKAKLAENYSLLLTLVAILVLIATAYSLISTLIGLAGCS  1196
AGD98722.1   1118  LSLSERIYLRLCQIRIDAVKKEQLNGVSRGVLDVLRDCMNKSKARLAENYSLLLTLVAILVLIATAYSLISTLVGLAGCS  1197
DAZ85702.1   1118  LSLSERIYLRLCQIRIDAVKREQLNGVSRGVLDVLRDCMNKSKARLAENYSLLLTLVAILVLIATAYSLISTLIGLAGCS  1197
QGY99255.1   1118  LSLSERIYLRLCQIRIDTVKKEQLNGVSRGVLDILRDCMNKSKAKLAENYSLLLTLVAILVLIATAYSLISTLVGLAGCT  1197
DAZ85701.1   1118  LSLSERIYLRLCQIRIDAVKKEQLNGVSRGVLDILRDCMNKSKAKLAENYSLLLTLTAILVLIATAYSLISTLVGLAGCT  1197
DAZ85704.1   1120  LSLSERIYLRLCQIRIDAVKKEQLSGPSRGTLDVLRDCMAKSKAKLCENYSLLLTLVAVLVLIATAYSLISTLVGLAGCT  1199
QYU59218.1   1118  LSLSERIYLRLCQIRIDAVKKEQLNGVSRGVLDILRDCMNKSKAKLAENYSLLLTLVAILVLIATAYSLISTLIGLAGCT  1197
ADJ39329.1   1120  LSLSERIYLRLCQIRIDAVKKEQLGGPSRGTLDVLRDCMAKSKAKLCENYSLLLTLVAVLVLIATAYSLISTLVGLAGCT  1199
QBZ78354.1   1118  LSLSERIYLRLCQIRIDAVKKEQLNGVSRGVLDILRDCMNKSKAKLAENYSLLLTLTAILVLIATAYSLISTLVGLAGCT  1197
QBZ78356.1   1118  LSLSERIYLRLCQIRIDAVKKEQLNGVSRGVLDILRDCMNKSKAKLAENYSLLLTLTAILVLIATAYSLISTLVGLAGCT  1197
QYA72350.1   1118  LSLSERIYLRLCQIRIDAVKKEQLSGPSRGTLDVLRDCMAKSKAKLCENYSLLLTLVAVLVLIATAYSLISTLVGLAGCT  1197
ADJ39331.1   1120  LSLSERIYLRLCQIRIDAIKKEQLSGPSRGTLDVLRECMAKSKAKLAENYSLLLTLVAVLVLIATAYSLISTLVGLSGCA  1199
DAZ85705.1   1120  LSLSERIYLRLCQIRIDAVKKEQLSGPSRGTLDVLRDCMAKSKAKLCENYSLLLTLVAVLVLIATAYSLISTLVGLAGCT  1199
DAZ85709.1   1120  LSLSERIYLRLCQIRIDAVKKEQLSGPSRGTLDVLRDCMAKSKAKLCENYSLLLTLVAVLVLIATAYSLISTLVGLAGCT  1199
DAZ85710.1   1120  LSLSERIYLRLCQIRIDAVKKEQLSGPSRGTLDVLRDCMAKSKAKLCENYSLLLTLVAVLVLIATAYSLISTLVGLAGCT  1199
DAZ85703.1   1120  LSLSERIYLRLCQIRIDAVKKEQLSGPSRGTLDVLRDCMAKSKAKLCENYSLLLTLVAVLVLIATAYSLISTLVGLAGCT  1199
ADJ39330.1   1120  LSLSERIYLRLCQIRIDAVKKEQLSGPSRGTLDILKDCMAKSKAKLVENYSLLLTLVAVLVLIATAYSLISTLVGLAGCT  1199
ACF32434.1   1120  LSLSERIYLRLCQIRIDKVKKEQLSGPSRGTLDVLRDCMAKSKAKLAENYSLLLTLVAVLVLIATAYSLISTLVGLAGCT  1199
QYU59216.1   1117  LSLSERIYLRLCQIRIDAVKKEQLSGPSRGTLDVLKDCMAKSKAKLVENYSLLLTLVAVLVLIATAYSLISTLVSLAGCT  1196
```

```
                              NTB-VPg cleavage site     VPg-Pro cleavage site

YP_053925.1  1198  SFAGGMVALNHVSNASIPCSEPRLEEGYIPRNKFVSRISRTRGDGPAQGQGDHEELVTELYYYFDGVKRLISCCWFKGRS  1277
AGT56093.1   1197  SFAGGMVALNHVSNASIPCSEPRLEEGYIPRNKFVSRISRTRGDGPAQGQGDHEELVTELYYYFDGVKRLISCCWFKGRS  1276
QBZ78358.1   1197  SFAGGMVALNHVSNASIPCSEPRLEEGYIPRNKFVSRISRTRGDGPAQGQGDHEELVTELYYYFDGVKRLVSCCWFKGRA  1276
AGD98722.1   1198  SFAGGLVALNHVSSASIPCSEPRLEEGYIPRNKFVSRISRTRGDGPAQGQGDHEDLVTELYYYFDGVKRLISCCWFRGRS  1277
DAZ85702.1   1198  SFAGGLVALNHVSSASIPCSEPRLEEGYIPRNKFVSRISRIRGDGPAQGQGDHEELVTELYYYFDGVKRLISCCWFKGRS  1277
QGY99255.1   1198  SFAGGMVALTHVSGASIPCSEPRLEDGYIPRNRFVSRISRTRGDGPAQGQGEHEELVTELYYYFDGVKRLISCCWFKGRS  1277
DAZ85701.1   1198  SFAGGMVALTHVSGASIPCSEPRLEERYIPRNHFVSRISRTRGDGPAQGQGEHEELVTELYYYFDGVKRLISCCWFKGRS  1277
DAZ85704.1   1200  SFAGGMVAMNQVSNASIPCSEPRMEERYSARNRFVSRISRARGDGPAKGQGEHEELVTELYYYFDGVKRLISCCWFKGRS  1279
QYU59218.1   1198  SFAGGMVALTHVSGASIPCSEPRLEEGYIPRNRFVSRISRTRGDGPAQGQGEHEELVTELYYYFDGVKRLISCCWFKGRS  1277
ADJ39329.1   1200  SFAGGIVAMKQVSNASIPCSEPRLEERYSARNRFVSRISRARGDGPAKGQGEHEELVTELYYYFDGVKRLISCCWFKGRS  1279
QBZ78354.1   1198  SFAGGMVALTHVSGASIPCSEPRLEERYIPRNHFVSRISRTRGDGPAQGQGEHEELVTELYYYFDGVKRLISCCWFKGRS  1277
QBZ78356.1   1198  SFAGGMVALTHVSGASIPCSEPRLEERYIPRNHFVSRISRTRGDGPAQGQGEHEELVTELYYYFDGVKRLISCCWFKGRS  1277
QYA72350.1   1198  SFAGGMVAMKEVSNASMLRSEPRLEERYSARNRFVSRISRARGDGPAKGQGEHEELVTELYYYFDGVKRLISCCWFKGRS  1277
ADJ39331.1   1200  SFAGGMVAMKDVRNASIPCSEPRLEERYSARNRFVSRISRARGDGPAKGQGEHEELVTELYYYFDGVKRLISCCWFKGRS  1279
DAZ85705.1   1200  SFAGGMVAMNQVSSASIPCSEPRLEERYSARNRFVSRISRARGDGPAKGQGEHEELVTELYYYFDGVKRLISCCWFKGRS  1279
DAZ85709.1   1200  SFAGGMVAMKEVSSASMLRSEPRLEERYSARNRFVSRISRARGDGPAKGQGEHEELVTELYYYFDGVKRLISCCWFKGRS  1279
DAZ85710.1   1200  SFAGGMVAMKEVSSASMLRSEPRLEERYSARNRFVSRISRARGDGPAKGQGEHEELVTELYYYFDGVKRLISCCWFKGRS  1279
DAZ85703.1   1200  SFAGGMVAMNQVSSASIPCSEPRLEERYSARNRFVSRISRARGDGPAKGQGEHEELVTELYYYFDGVKRLISCCWFKGRS  1279
ADJ39330.1   1200  SFAGGLVSMKQVSNASMPCSEPRLEDRYAARNRFVSRISRARGDGPAKGQGEHEELVTELYYYFDGVKRLVSCCWFKGRS  1279
ACF32434.1   1200  SFAGGMVAMNQVSNASMPCSEPRLEERYSARNRFVSRISRVRGDGPAKGQGEHEELVTELYYYFDGVKRLISCCWFKGRS  1279
QYU59216.1   1197  SFAGGMVAMNQVSNASIPCSEPRLEERYTARNRFVSRISRARGDGPAKGQGEHEELVTELYYYYDGVKRLISCCWFKGRS  1276

YP_053925.1  1278  LLLTRHQAMAIPIGNEIQVIYADGTERKLVWPGRQEDRSCKGYIEFPDNELVVFEHARLLTMPIKYEKFFVDDPDHQISP  1357
AGT56093.1   1277  LLLTRHQAMAIPIGNEIQVIYADGTERKLVWPGRQEDRSCKGYIEFPDNELVVFEHARLLTMPIKYEKYFVDDPDHQISP  1356
QBZ78358.1   1277  LLLTRHQAMAIPIGNEIQVIYADGTERKLVWPGKQENGSCKGYVEFPDNELVVFEHTRLLTMPIKYEKYFVDDPDYQISP  1356
AGD98722.1   1278  LLMTRHQAMAIPLGNEVQIIYADGTERKLVWPGRQEDGSCKGYIEFPDNELVVFEHARLLTMPVKYEKYFVDDPDHQISP  1357
DAZ85702.1   1278  LLMTRHQALAIPIGNEVQIIYADGTERKLVWPGRQENSSCKGYIEFPDNELVVFEHARLLTMPIKYEKYFVDDPDHQISP  1357
QGY99255.1   1278  LLLTRHQAMAIPIGNEVQIIYADGTERKLVWPGKQENGSCKGYIEFPDNELVVFEHSRLLTMPIKYEKYFVDDPDRQISP  1357
DAZ85701.1   1278  LLLTRHQAMAIPVGNEVQIIYADGTERKLVWPGKQENGSCKGYIEFPDNELVVFEHSRLLTMPIKYEKYFVDDPDRQISP  1357
DAZ85704.1   1280  LLMTRHQALAIPIGNEVQVIYADGTDRKLVWPGKQEDGSCKGYIEFPENELVVFEHARLLTMPIKYEKYFVDDPDRQISP  1359
QYU59218.1   1278  LLLTRHQAMAIPVGNEVQIIYADGTERKLVWPGRQENGSCKGYIEFPDNELVVFEHSRLLTMPIKYEKYFVDDPDRQISP  1357
ADJ39329.1   1280  LLMTRHQALAIPIGNEVQVIYADGTDRKLVWPGKQEDGSCKGYIEFPENELVVFEHARLLTMPVKYEKYFVDDPDRQISP  1359
QBZ78354.1   1278  LLLTRHQAMAIPIGNEVQIIYADGTERKLVWPGKQENGSNKGYVEFPDNELVVFEHSRLLTMPIKYEKYFVDDPDRQISP  1357
QBZ78356.1   1278  LLLTRHQAMAIPIGNEVQIIYADGTERKLVWPGKQENGSNKGYVEFPDNELVVFEHSRLLTMPIKYEKYFVDDPDRQISP  1357
QYA72350.1   1278  LLMTRHQALAIPIGNEVQVIYADGTDRKLVWPGKQEDGSCKGYIEFPENELVVFEHARLLTMPIKYEKYFVDDPDRQISP  1357
ADJ39331.1   1280  LLMTRHQALAIPVGNEVQVIYADGTDRKLVWPGKQENGSCKGYIEFPENELVVFEHVRLLTMPVKYEKYFVDDPDRQISP  1359
DAZ85705.1   1280  LLMTRHQALAIPVGNEVQVIYADGTERKLVWPGKQEDGSCKGYIEFPENELVVFEHARLLTMPIKYEKYFVDDPDRQISP  1359
DAZ85709.1   1280  LLMTRHQALAIPIGNEVQVIYADGTDRKLVWPGKQEDGSCKGYIEFPENELVVFEHARLLTMPIKYEKYFVDDPDRQISP  1359
DAZ85710.1   1280  LLMTRHQALAIPIGNEVQVIYADGTDRKLVWPGKQEDGSCKGYIEFPENELVVFEHARLLTMPIKYEKYFVDDPDRQISP  1359
DAZ85703.1   1280  LLMTRHQALAIPVGNEVQVIYADGTDRKLVWPGKQEDGSCKGYIEFPENELVVFEHARLLTMPIKYEKYFVDDPDRQISP  1359
ADJ39330.1   1280  LLMTRHQALAIPVGNEVQVIYADGTDRKLVWPGKQEDGTCKGYIEFPENELVVFEHARLLTMPIKYEKYFVDDPDRQISP  1359
ACF32434.1   1280  LLMTRHQALAIPVGNEVQVIYADGTDRKLIWPGKQEDGSCKGYIEFPENELVVFEHARLLTMPIKYEKYFVDDPDRQISP  1359
QYU59216.1   1277  LLMTRHQALAIPIGNEVQVIYADGTDRKLVWPGKQEDGNCKGYIEFPENELVVFEHARLLTMPVKYEKYFVEDPDRQISP  1356

YP_053925.1  1358  NVAVKCCVARLEDGIPQFHFWNKYASARSDVHTIKDEGGSAVYQNKIRRYIIYAHEAKRNDCGAIAVAEIQRTPKVLAML  1437
AGT56093.1   1357  NVAVKCCVARLEDGIPQFHFWNKYASARSDIHTIKDEGGSAVYQNKIRRYLIYAHEAKRNDCGAIAVAEIQRTPKVVAML  1436
QBZ78358.1   1357  NIAVKCCVARLEDGLPQFHFWNKYASARSDVHTIKDEGGSAVYQNKIRRYLIYAHEAKKNDCGAIAVAEIQRTPKVVXXX  1436
AGD98722.1   1358  NVAVKCCVARLEDGLPQFHFWNKYASARSDVHTIKDEGGSAVYQNKIRRYIIYAHEAKRNDCGAIAVAEIQRTPKVIAML  1437
DAZ85702.1   1358  NVAVKCCVARLEDGLPQFHFWNKYASARSDVHTIKDEGGSAVYQNKIRRYIIYAHEAKKNDCGAIAVAEIHRTPKVIAML  1437
QGY99255.1   1358  NVAVKCCVARLVDEIPQFHFWNKYASARSDVHTIRDEGGSAVYQNKIRRYIIYAHEAKKNDCGAIAVAEIQRTPKVIAML  1437
DAZ85701.1   1358  NVAVKCCVARLVDEIPQFHFWNKYASARNDVHTIKDEGGSAVYQNKIRRYIIYAHEAKKNDCGAIAVAEIQRTPKVIAML  1437
DAZ85704.1   1360  NVAVKCCVARLEEGLPQFHFWNKYATARSDVHTVVDEGGSAVYQNKIRRHIIYAHEAKRNDCGAIAVAEIQRTPKVIAML  1439
QYU59218.1   1358  NVAVKCCVARLVDEIPQFHFWNKYASTRNDVHTIKDEGGSAVYQNKIRRYIIYAHEAKKNDCGAIAVAEIQRTPKVIAML  1437
ADJ39329.1   1360  NVAVKCCVARLEEGLPQFHFWNKYASARSDVHTVVDEGGSAVYQNKIRRHIIYAHEAKRNDCGAIAVAEIQRVPKVIAML  1439
QBZ78354.1   1358  NVAVKCCVARLEDEIPQFHFWNKYASARNDVHTIKDEGGSAVYQNKIRRYIIYSHEAKKNDCGAIAVAEIQRTPKVIAML  1437
QBZ78356.1   1358  NVAVKCCVARLEDEIPQFHFWNKYASARNDVHTIKDEGGSAVYQNKIRRYIIYSHEAKKNDCGAIAVAEIQRTPKVIAML  1437
QYA72350.1   1358  NVAVKCCVARLEEGLPQFHFWNKYATARSDVHTVVDEGGSAVYQNKIRRHIIYAHEAKRNDCGAIAVAEIQRTPKVIAML  1437
ADJ39331.1   1360  NVAVKCCVARLEEGLPQFHFWNKYASARSDVHTVVDEGGSAVYQNKIRRHIIYAHEAKRNDCGAIAVAEIQRVPKVIAML  1439
DAZ85705.1   1360  NVAVKCCVARLEEGLPQFHFWNKYATARSDVHTVVDEGGSAVYQNKIRRHIIYAHEAKRNDCGAIAVAEIQRTPKVIAML  1439
DAZ85709.1   1360  NVAVKCCVARLEEGFPQFHFWNKYATARSDVHTVVDEGGSAVYQNKIRRHIIYAHEAKRNDCGAIAVAEIQRTPKVIAML  1439
DAZ85710.1   1360  NVAVKCCVARLEEGFPQFHFWNKYATARSDVHTVVDEGGSAVYQNKIRRHIIYAHEAKRNDCGAIAVAEIQRTPKVIAML  1439
DAZ85703.1   1360  NVAVKCCVARLEEGLPQFHFWNKYATARSDVHTVVDEGGSAVYQNKIRRHIIYAHEAKRNDCGAIAVAEIQRTPKVIAML  1439
ADJ39330.1   1360  NVAVKCCVARLEEGLPQFHFWNKYATARSDVHTVVDEGGSAVYQNKIRRHIIYAHEAKRNDCGAIAVAEIQRTPKVIAML  1439
ACF32434.1   1360  NVAVKCCVARLEEGLPQFHFWNKYATARSDVHTVVDEGGSAVYQNKIRRHIIYAHEAKRNDCGAIAVAEIQRTPKVIAML  1439
QYU59216.1   1357  NVAVKCCVARLEEGLPQFHFWNKYATARSDVHTIVDEGGSSVYQNKIRRHIIYAHEAKRNDCGAIAVAEIQRVPKVIAML  1436
```

```
                              Pro-Pol cleavage site

YP_053925.1  1438  VSGIGNVTYSSVIPSYSSSFVRGDVPYVPEDGIKTNGYRKVGYLMAKDAPHVPSKTAFMKVPDEICFPYPNPKQPAILSA  1517
AGT56093.1   1437  VSGIGNVTYSSVIPSYSSSFVRGDVPYVPEDGIKTNGYRKVGYLMAKDAPHVPSKTAFMKVPDEICFPYPNPKQPAILSA  1516
QBZ78358.1   1437  XSGIGNVTYSSVIPSYSSSFVVGDVPYVPEDGIKTNGYRKVGYLMAKDAPHVPSKTAFMRVPDEICFPYPNPKQPAILSA  1516
AGD98722.1   1438  VSGIGNVTYSSVIPNYSSSFVRGDVPYVPEDGIKTNGYRKIGYLMAKDAPHVPSKTAFMRVPDEICFPYPNPKQPAILSA  1517
DAZ85702.1   1438  VSGIGNVTYSSVIPNYSSSFVRGDVPYVPEDGIKTNGYRKVGYLMAKDAPHVPSKTAFMRVPDEICFPYPNPKQPAILSA  1517
QGY99255.1   1438  VSGSGNVTYSSVIPNYSSSFVRGDVPYVPEDGIKTNGYRKVGYLMQKDAPHVPSRTAFMRVPDEICFPYPNPKQPAILSA  1517
DAZ85701.1   1438  VSGFGNVTYSSVIPNYSSSFVRGDVPYVPEDGIKTSGYRKVGYLMMKDAPHVPSKTAFMRVPDEICFPYPNPKQPAILSS  1517
DAZ85704.1   1440  VSGSGNVTYSSVIPNYSSSFVRGDVPYVPEDGIKTNGYRKVGYLMRKDAPHVPSKTAFMKVPDEICFPYPNPKQPAILSD  1519
QYU59218.1   1438  VSGFGNVTYSSVIPNYSSSFVRGDVPYVPEDGIKTNGYRKVGYLLRKDAPHVPSKTAFTRVPDEICFPYPNPKQPAILSA  1517
ADJ39329.1   1440  VSGAGNVSYSSVIPNYSSSFVRGDVPYVPEDGIKTNGYRKVGYLMRKDAPHVPSKTAFMRVPDEICFPYPNPKQPAILSA  1519
QBZ78354.1   1438  VSGFGNVTYSSVIPNYSSSFVRGDVPYVPEDGIKTNGYRKVGYLMMKDAPHVPSKTAFTRVPDEICFPYPNPKQPAILSS  1517
QBZ78356.1   1438  VSGFGNVTYSSVIPNYSSSFVRGDVPYVPEDGIKTNGYRKVGYLMMKDAPHVPSKTAFTRVPDEICFPYPNPKQPAILSS  1517
QYA72350.1   1438  VSGSGNVTYSSVIPNYSSSFVRGDVPYVPEDGIKTNGYRKVGYLARKDAPHVPSKTAFMKVPDEICFPYPNPKQPAILSD  1517
ADJ39331.1   1440  VSGTGNVSYSSVIPNYSSSFVRGDVPYVPEDGIKTNGYRKVGYLMRKDAPHVPSKTAFMKVPDEICFPYPNPKQPAILSE  1519
DAZ85705.1   1440  VSGSGNVTYSSVIPNYSSSFVRGDVPYVPEDGIKTNGYRKVGYLMRKDAPHVPSKTAFMKVPDEICFPYPNPKQPAILSD  1519
DAZ85709.1   1440  VSGSGNVTYSSVIPNYSSSFVRGDVPYVPEDGIKTNGYRKVGYLMRKDAPHVPSKTAFMRVPDEICFPYPNPKQPAILSD  1519
DAZ85710.1   1440  VSGSGNVTYSSVIPNYSSSFVRGDVPYVPEDGIKTNGYRKVGYLMRKDAPHVPSKTAFMRVPDEICFPYPNPKQPAILSD  1519
DAZ85703.1   1440  VSGSGNVTYSSVIPNYSSSFVRGDVPYVPEDGIKTNGYRKVGYLMRKDAPHVPSKTAFMKLPDEICFPYPNPKQPAILSD  1519
ADJ39330.1   1440  VSGSGNITYSSVIPNYSSSFVRGDVPYVPEDGIKTNGYRKVGYLMRKDAPHVPSKTAFMRVPDEICFPYPNPKQPAILSD  1519
ACF32434.1   1440  VSGSGNVTYSSVIPNYSSSFVRGDVPYVPEDGIKTNGYRKVGYLMRKDAPHVPSKTAFMKVPDEICFPYPNPKQPAILSA  1519
QYU59216.1   1437  VSGSGNVTYSSVIPNYSSSFVRGDVPYVPEDGIKTEGYRKVGYLLRKDAPHVPSKTAFMKVPDEICFPYPNPKQPAILSA  1516

YP_053925.1  1518  EDERLIGTVHEGYTPIREGMKKFAEPMHLLDAQLLDEVAGDMVHTWFDAGEILEDVPLSIAINGDVEEEYFDPIAMDTSE  1597
AGT56093.1   1517  EDERLKGTVHEGYTPIREGMRKFAEPMHLLDAQLLDEVAGDMVHTWFDAGEYLEDVPLNIAINGDVEEEYFDPIAMDTSE  1596
QBZ78358.1   1517  EDERLIGTVHEGYTPVREGMRKFAEPMHLLDAQLLDEVAGDMVHTWFDAGEFLEDVPLHIAINGDVEEEYFDPIAMDTSE  1596
AGD98722.1   1518  EDERLKGTVHEGYTPVREGMKKFAEPMHLLDAQLLDEVAGDMVHTWFDAGEFLEDVPLSVAINGDVDEEYFDPIAMDTSE  1597
DAZ85702.1   1518  EDERLKGTVHEGYNPVRDGMKKFAEPMHLLDARLLDEVAGDMVHTWFDAGEFLEDVPLDVAINGDANEEYFDPIAMDTSE  1597
QGY99255.1   1518  EDERLKGTVHEGYTPVRDGMKKFAEPVHLLDAKLLDEVAGDMVHTWFDVGEFLEDVPLHVAINGDMEEEYFDPIAMDTSE  1597
DAZ85701.1   1518  EDERLKGTVHEGYTPIRDGMKKFAEPVHLLDAKLLDEVAGDMVHTWFDAGEFLEDVPLHVAINGDVEEEYFDPIAMDTSE  1597
DAZ85704.1   1520  EDERLKGTVHEGYTPIRDGMKKFAEPMHLLDEKLLDEVAGDMVHTWFDVGEILEDVPLNIAINGDVDEEYFDPMAMDTSE  1599
QYU59218.1   1518  EDVRLKGTVHEGYTPIRDGMKKFAEPVHLLDAKLLDEVAGDMVHTWFDVGEFLEDVPLHVAINGDVEEEYFDPIAMDTSE  1597
ADJ39329.1   1520  EDERLKGTVHEGYTPIRDGMKKFAEPMHLLDEKLLDEVAGDMVHTWFDVGEFLEDVPLNIAINGDVDEEYFDPMAMDTSE  1599
QBZ78354.1   1518  EDERLKGTVHEGYTPIRDGMKKFAEPVHLLDAKLLDEVAGDMVHTWFDAGEFLEDVPLHVAINGDVDEEYFDPIAMDTSE  1597
QBZ78356.1   1518  EDERLKGTVHEGYTPIRDGMKKFAEPVHLLDAKLLDEVAGDMVHTWFDAGEFLEDVPLHVAINGDVDEEYFDPIAMDTSE  1597
QYA72350.1   1518  EDERLKGTVHEGYTPIRDGMKKFAEPMHLLDEKLLDEVAGDMVHTWFDVGEFLEDVPLNVAINGDVDEEYFDPMAMDTSE  1597
ADJ39331.1   1520  EDVRLKGTVHEGYTPTRDGMKKFAEPMHLLDEKLLDEVAGDMVQTWFDVGEFLEDVPLNVAINGDVDEEYFDPMAMDTSE  1599
DAZ85705.1   1520  EDERLKGTVHEGYTPIRDGMKKFAEPMHLLDEKLLDEVAGDMVHTWFDVGEFLEDVPLNVAINGDVDEEYFDPMAMDTSE  1599
DAZ85709.1   1520  EDERLKGTVHEGYTPIRDGMKKFAEPMHLLDEKLLDEVAGDMVHTWFDVGEFLEDVPLNIAINGDVDEEYFDPMAMDTSE  1599
DAZ85710.1   1520  EDERLKGTVHEGYTPIRDGMKKFAEPMHLLDEKLLDEVAGDMVHTWFDVGEFLEDVPLNIAINGDVDEEYFDPMAMDTSE  1599
DAZ85703.1   1520  EDERLKGTVHEGYTPIRDGMKKFAEPMHLLDEKLLDEVAGDMVHTWFDVGEFLEDVPLNVAINGDVDEEYFDPMAMDTSE  1599
ADJ39330.1   1520  EDERLKGTIHEGYTPIRDGMKKFAEPMHLLDEKLLDEVAGDMVHTWFEVGEFLEDVPLNVAINGDVDEEYFDPMAMDTSE  1599
ACF32434.1   1520  EDDRLKGTVHEGYTPIRDGMKKFAEPMHLLDEKLLDEVAGDMVQTWFDVGEFLEDVPLNTAINGDVDEEYFDPMAMDTSE  1599
QYU59216.1   1517  DDERLKGTVHEGYTPIRDGMKKFAEPMHLLDEKLLDEVAGDMVQTWFDVGECLTDVPLDIAINGDIEEEYFDPMAMDTSE  1596

YP_053925.1  1598  GYPEVLQRKNGEKGKARFFVGEPGAREFVPGCGPERAYLSLEEECKTRIPSLVSIETPKDERLKRSKIETPGTRLFSVLP  1677
AGT56093.1   1597  GYPEVLQRKNGEKGKARFFVGEPGAREFVPGCGPERAYLSLEEECKTRIPSLVSIETPKDERLKRSKIETPGTRLFSVLP  1676
QBZ78358.1   1597  GYPEVLQRKNGEKGKARFFVGEPGAREFVPGCGPERAYLSLEEECKTRIPSLVSIETPKDERLKRSKIETPGTRLFSVLP  1676
AGD98722.1   1598  GYPEVLQRKPGEKGKARFFVGEPGAREFVPGCGPEKAYLVLEEECKTCIPSLISIETPKDERLKRSKIETPGTRLFSVLP  1677
DAZ85702.1   1598  GYPEVLQRKPGEKGKARFFVGEPGAREFVPGCGPEKAYLVLEEECKTRIPSLISIETPKDERLKRSKIETPGTRLFSVLP  1677
QGY99255.1   1598  GYPEVLQRKTGEKGKARFFVGEPGSREFVPGCGPERTYQSLEEECKTRVPSLVSIETPKDERLKRSKIETPGTRLFSVLP  1677
DAZ85701.1   1598  GYPEVLQRKNGEKGKARFFVGEPGSREFVPGCGPERAYQSLEEECKTRVPSLVSIETPKDERLKRSKIETPGTRLFSVLP  1677
DAZ85704.1   1600  GYPEVLQRKAGEKGKARFFVGEPGAREFVPGCGPEKAYLSLEEECKTRVPSLISIETPKDERLKRSKIETPGTRLFSVLP  1679
QYU59218.1   1598  GYPEVLQRKTGEKGKARFFVGEPGLREFVPGCGPEKAYQSLEEECKMRIPSLVSIETPKDERLKRSKIETPGTRLFSVLP  1677
ADJ39329.1   1600  GYPEVLQRKAGEKGKARFFVGEPGSREFVPGCGPEKAYLSLEEECKTRVPSLISIETPKDERLKRSKIETPGTRLFSVLP  1679
QBZ78354.1   1598  GYPEVLQRKNGERGKARFFVGEPGSREFVPGCGPERAYQSLEEECKTRVPSLVSIETPKDERLKRSKIETPGTRLFSVLP  1677
QBZ78356.1   1598  GYPEVLQRKNGERGKARFFVGEPGSREFVPGCGPERAYQSLEEECKTRVPSLVSIETPKDERLKRSKIETPGTRLFSVLP  1677
QYA72350.1   1598  GYPEVLQRKAGEKGKARFFVGEPGAREFVPGCGPEKAYLSLEEECKVRVPSLISIETPKDERLKRSKIETPGTRLFSVLP  1677
ADJ39331.1   1600  GYPEVLQRKAGEKGKARFFVGEPGAREFVPGCGPEKAYLSLEEECKTRVPSLISIETPKDERLKRSKIETPGTRLFSVLP  1679
DAZ85705.1   1600  GYPEVLQRKAGEKGKARFFVGEPGAREFVPGCGPEKAYLSLEEECKTRVPSLISIETPKDERLKRSKIETPGTRLFSVLP  1679
DAZ85709.1   1600  GYPEVLQRKAGEKGKARFFVGEPGAREFVPGCGPEKAYLSLEEECKMRVPSLISIETPKDERLKRSKIETPGTRLFSVLP  1679
DAZ85710.1   1600  GYPEVLQRKAGEKGKARFFVGEPGAREFVPGCGPEKAYLSLEEECKMRVPSLISIETPKDERLKRSKIETPGTRLFSVLP  1679
DAZ85703.1   1600  GYPEVLQRKAGEKGKARFFVGEPGAREFVPGCGPEKAYLSLEEECKMRVPSLISIETPKDERLKRSKIETPGTRLFSVLP  1679
ADJ39330.1   1600  GYPEVLQRKAGEKGKARFFIGEPGSREFVPGCGPEKAYLSLEEECKMRVPSLISIETPKDERLKRSKIETPGTRLFSVLP  1679
ACF32434.1   1600  GYPEVLQRKAGEKGKARFFVGEPGAREFVPGCGPERAYLSLEEECKVRVPSLISIETPKDERLKRSKIETPGTRLFSVLP  1679
QYU59216.1   1597  GYPEVLQRKAGEKGKARFFVGEPGARVFVAGCGPERAYLSLEEECKTRVPSLVSIETPKDERLKSSKIETPGTRLFSVLP  1676
```

```
YP_053925.1  1678  LAYNLLLRVKFLSFSRLLMKKRSHLPCQVGINPYSREWTDLYHRLAEKSDVGYNCDYKGFDGLITEQILAVVATMINAGF  1757
AGT56093.1   1677  LAYNLLLRVKFLSFSRLLMKKRSHLPCQVGINPYSREWTDVYHRLAEKSDVGYNCDYKGFDGLITEQVLAVVATMINAGF  1756
QBZ78358.1   1677  LAYNLLLRVKFLSFSRLLMKKRSHLPCQVGINPFSREWTDLYHRLAEKSDMGYNCDYKGFDGLITEQILAVIATMINAGF  1756
AGD98722.1   1678  LAYNLLLRVKFLSFSRLLMKKRGYLPCQVGINPYSREWTDLYHRLAEKSDVGYNCDYKGFDGLITEQILTVIAKMINAGF  1757
DAZ85702.1   1678  LAYNLLLRVKFLSFSRLLMKKRSYLPCQVGINPYSREWTDLYHRLAEKSDVGYNCDYKGFDGLITEQILTVIAKMINAGF  1757
QGY99255.1   1678  LAYNLLLRVKFLSFSRMLMKKRSHLPCQVGVNPYSREWTDLYHRLAEKSDVGYNCDYKGFDGLITEQILTVIAAMINAGF  1757
DAZ85701.1   1678  LAYNLLLRVKFLSFSRMLMKKRSHLPCQVGVNPYSREWTDLYHRLAEKSDVGYNCDYKGFDGLITEQILTVIAAMINSGF  1757
DAZ85704.1   1680  LAYNLLLRVKFLSFSRLLMKKRSHLPCQVGINPYSREWTDLYHRLAEKSDVGYNCDYKGFDGLITEQILAVVAKMINAGY  1759
QYU59218.1   1678  LAYNLLLRVKFLSFSRMLMKKRSHLPCQVGINPYSREWTDLYHRLAEKSDVGYNCDYKGFDGLITEQILTVIATMINAGF  1757
ADJ39329.1   1680  LAYNLLLRVKFLSFSRLLMKKRSHLPCQVGINPYSREWTDLYHRLAEMSDVGYNCDYKGFDGLITEQILAVVATMINAGY  1759
QBZ78354.1   1678  LAYNLLLRVKFLSFSRMLMRKRSHLPCQVGINPFSREWTDLYHRLAEKADVGYNCDYKGFDGLITEQILTVIAAMINAGF  1757
QBZ78356.1   1678  LAYNLLLRVKFLSFSRMLMRKRSHLPCQVGINPFSREWTDLYHRLAEKADVGYNCDYKGFDGLITEQILTVIAAMINAGF  1757
QYA72350.1   1678  LAYNLLLRVKFLSFSRLLMKKRSHLPCQVGINPYSREWTDLYHRLAEMSDVGYNCDYKGFDGLITEQILATVATMINTGY  1757
ADJ39331.1   1680  LAYNLLLRVKFLSFSRLLMKKRSHLPCQVGINPYSREWTDLYHRLAEMSDVGYNCDYKGFDGLITEQILAVVATMINAGY  1759
DAZ85705.1   1680  LAYNLLLRVKFLSFSRLLMKKRSHLPCQVGINPYSREWTDLYHRLAEKSDVGYNCDYKGFDGLITEQILAVVAKMINAGY  1759
DAZ85709.1   1680  LAYNLLLRVKFLSFSRLLMKKRSHLPCQVGINPYSREWTDLYHRLAEMSDVGYNCDYKGFDGLITEQILATVATMINAGY  1759
DAZ85710.1   1680  LAYNLLLRVKFLSFSRLLMKKRSHLPCQVGINPYSREWTDLYHRLAEMSDVGYNCDYKGFDGLITEQILATVATMINAGY  1759
DAZ85703.1   1680  LAYNLLLRVKFLSFSRLLMKKRSHLPCQVGINPYSREWTDLYHRLAEMSDAGYNCDYKGFDGLITEQILATVATMINTGY  1759
ADJ39330.1   1680  LAYNLLLRVKFLSFSRLLMKKRSYLPCQVGINPYSREWTDLYHRLAEMSDVGYNCDYKGFDGLITEQILATVATMINAGY  1759
ACF32434.1   1680  LAYNLLLRVKFLSFSRLLMKKRSHLPCQVGINPYSREWTDLYHRLAEQSDIGYNCDYKGFDGLITEQILAVVATMINTGY  1759
QYU59216.1   1677  LAYNLLLRVKFLAFSRLLMKKGGHLPCQVGINPYMREWTDLYHRLAEMSDVGYNCDYKGFDGLITEQVLAMIAKMINAGY  1756

YP_053925.1  1758  RNPVSNQQRSNLLMAISGRLSICGSQVYETEAGIPSGCALTVVINSIFNELLMRYCYKKIVPPIYRECFDRCVVLITYGD  1837
AGT56093.1   1757  RDPVSNQQRSNLLMAISRRLSICGSQVYETEAGIPSGCALTVVINSIFNELLMRYCYKKIVPPIYRECFDRCVVLITYGD  1836
QBZ78358.1   1757  RNPVSNQQRSNLLMAISGRLSICGNQVYETGAGIPSGCALTVVINSIFNELLMRYCYKKIVPPVYRECFDRCVVLITYGD  1836
AGD98722.1   1758  RNPVSNQQRSNLLLAISNRLSICGNQVYETESGIPSGCALTVVINSIFNELLMRYCYKKIVPPIYRECFDRCVVLITYGD  1837
DAZ85702.1   1758  RNPVSNQQRSNLLLAISSRLSICGNQVYETESGIPSGCALTVVINSIFNELLMRYCYKKIVPPIYRECFDRCVVLITYGD  1837
QGY99255.1   1758  RDPVSNLQRSNLLLAISGRLSICGNQVYETEAGIPSGCALTVVLNSIFNELLMRYCFKKIVPPVYRECFDRCVVLITYGD  1837
DAZ85701.1   1758  RNPVSNLQRSNLLLAISGRLSICGNQVYETEAGIPSGCALTVVLNSIFNELLMRYCFKKIVPPVYRECFDRCVVLITYGD  1837
DAZ85704.1   1760  QNPVSDQQRSNLLLAISGRLSICGNQVYETEAGIPSGCALTVVINSIFNELLMRYCFKKIVPPIYRECFDRCVVLITYGD  1839
QYU59218.1   1758  RDPVSNLQRSNLLLAISGRLSICGNQVYETEAGIPSGCALTVVLNSIFNELLMRYCFKKIVPPIYRECFDRCVVLITYGD  1837
ADJ39329.1   1760  QNPVANQQRSNLLLAISGRLSICGNQVYETEAGIPSGCALTVVINSIFNELLMRYCFKKIVPPVYRECFDRCVVLITYGD  1839
QBZ78354.1   1758  RDPVSNLQRSNLLLAISGRLSICGNQVYETEAGIPSGCALTVVLNSIFNELLMRYCFKKIVPPVYRECFDRCVVLITYGD  1837
QBZ78356.1   1758  RDPVSNLQRSNLLLAISGRLSICGNQVYETEAGIPSGCALTVVLNSIFNELLMRYCFKKIVPPVYRECFDRCVVLITYGD  1837
QYA72350.1   1758  RNPVGNQQRSNLLLAISGRLSICGNQVYETEAGIPSGCALTVVINSIFNELLMRYCFKKIVPPIYRECFDRCVVLITYGD  1837
ADJ39331.1   1760  QNPVANQQRSNLLLAISGRLSICGNQVYETEAGIPSGCALTVVINSIFNELLMRYCFKKIVPPVYRECFDRCVVLITYGD  1839
DAZ85705.1   1760  QNPVSDQQRSNLLLAISGRLSICGNQVYETEAGIPSGCALTVVINSIFNELLMRYCFKKIVPPVYRECFDRCIVLITYGD  1839
DAZ85709.1   1760  RNPIGNQQRSNLLLAISGRLSICGNQVYETEAGIPSGCALTVVINSIFNELLMRYCFKKIVPPIYRECFDRCVVLITYGD  1839
DAZ85710.1   1760  RNPIGNQQRSNLLLAISGRLSICGNQVYETEAGIPSGCALTVVINSIFNELLMRYCFKKIVPPIYRECFDRCVVLITYGD  1839
DAZ85703.1   1760  RNPVSNQQRSNLLLAISGRLSICGNQVYETEAGIPSGCALTVVINSIFNELLMRYCFKKIVPPVYRECFDRCVVLITYGD  1839
ADJ39330.1   1760  RNPVSNQQRSNLLLAISGRLSICGNQVYETEAGIPSGCALTVVINSIFNELLMRYCFKKIVPPIYRECFDRCVVLITYGD  1839
ACF32434.1   1760  RNPVSNQQRSNLLLAICGRLSICGNQVYETEAGIPSGCALTVVINSIFNELLMRYCFKKIVPPVYRECFDRCVVLITYGD  1839
QYU59216.1   1757  RDPIGDRQRTNLLLAISGRLSICGNQVYETEAGIPSGCALTVVINSIFNELLMRYCFKKIVPPVYRECFDRCVVLITYGD  1836

YP_053925.1  1838  DNVFTVSQSIMTSFTGDALKAEMANLGVTITDGKDKSLATIPARPLLELEFLKRGFKKGNGGLIYAPLEKLSIMSSLVYI  1917
AGT56093.1   1837  DNVFTVSQSIMTSFTGDALKAEMANLGVTITDGKDKSLATIPARPLLELEFLKRGFKIGNGGLIYAPLEKLSIMSSLVYI  1916
QBZ78358.1   1837  DNVFTVSQSIMTHFTGDTLKAEMAKLGVTITDGKDKSLATIPARPLLELEFLKRGFKKGNGGLIYAPLEKLSIMSSLVYI  1916
AGD98722.1   1838  DNVFTVSQSIMTHFTGDALKAEMAGLGVTITDGKDKSLATIPARPLLELEFLKRGFKKGNGGLIYAPLEKLSIMSSLVYI  1917
DAZ85702.1   1838  DNVFTVSQSVMAYFTGDALKAEMANLGVTITDGKDKSLATIPARPLLELEFLKRGFKKGDGGLIYAPLEKLSIMSSLVYI  1917
QGY99255.1   1838  DNVFTVSQSVMASFTGDALKAEMAKLGVTITDGKDKSLATIPARPLLELEFLKRGFKKGDGGLIYAPLEKLSIMSSLVYI  1917
DAZ85701.1   1838  DNVFTVSQSVMASFTGDALKAEMANLGVTITDGKDKSLATIPARPLLELEFLKRGFKKGGGGLIYAPLEKLSIMSSLVYI  1917
DAZ85704.1   1840  DNVFTVAQSIMESFTGDALKAEMANLGVTITDGKDKSLATIPARPLLELEFLKRGIKKGNGGMIYAPLEKLSIMSSLVYI  1919
QYU59218.1   1838  DNVFTVSQSVMASFTGDALKAEMASLGVTITDGKDKSLATIPARPLLELEFLKRGFKKGDGGLIYAPLEKLSIMSSLVYI  1917
ADJ39329.1   1840  DNVFTVSQSIMESFTGDALKAEMANLGVTITDGKDKSLATIPARPLLELEFLKRGFKRGNGGMIYAPLEKLSIMSSLVYI  1919
QBZ78354.1   1838  DNVFTVSQSVMASFTGDALKAEMANLGVTITDGKDKSLATIPARPLLELEFLKRGFKKGGGGLIYAPLEKLSIMSSLVYI  1917
QBZ78356.1   1838  DNVFTVSQSVMASFTGDALKAEMANLGVTITDGKDKSLATIPARPLLELEFLKRGFKKGGGGLIYAPLEKLSIMSSLVYI  1917
QYA72350.1   1838  DNVFTVSQSIMESFTGDALKAEMANLGVTITDGKDKSLATIPARPLLELEFLKRGFKKGNGGMIYAPLEKLSIMSSLVYI  1917
ADJ39331.1   1840  DNVFTVSQSIMESFTGDALKAEMANLGVTITDGKDKSLATIPARPLLELEFLKRGFKKGNGGMIYAPLEKLSIMSSLVYI  1919
DAZ85705.1   1840  DNVFTVSQSVMESYTGDALKAEMANLGVTITDGKDKSLATIPARPLLELEFLKRGIKKGNGGMIYAPLEKLSIMSSLVYI  1919
DAZ85709.1   1840  DNIFTVSQSIMESFTGDVLKAEMAKLGVTITDGKDKSLATIPARPLLELEFLKRGFKRGDGGMIYAPLEKLSIMSSLVYI  1919
DAZ85710.1   1840  DNIFTVSQSIMESFTGDVLKAEMAKLGVTITDGKDKSLATIPARPLLELEFLKRGFKRGDGGMIYAPLEKLSIMSSLVYI  1919
DAZ85703.1   1840  DNVFTVSQSIKESFTGDALKAEMANLGVTITDGKDKSLATIPSRPLLELEFLKRGIKKGNGGMIYAPLEKLSIMSSLVYI  1919
ADJ39330.1   1840  DNVFTVAQSIMESFTGDALKAEMATLGVTITDGKDKSLATIPARPLLELEFLKRGFKKGDGGMIYAPLEKLSIMSSLVYI  1919
ACF32434.1   1840  DNVFTVSQSIMESFTGDALKAEMANVGVTITDGKDKSLATIPARPLLELEFLKRGIKKGNGGMIYAPLEKLSIMSSLVYI  1919
QYU59216.1   1837  DNVFTVSQSIMESFTGGALKAEMANLGVTITDGKDKSLATIPARPLLGLEFLKRGFKKGNGGLIYGPLEKLSIMSSLVYI  1916
```

```
YP_053925.1  1918  RSDGSDMLQKLVDNVNTALVELYLHQDREYSESVRDFYLEKLPPGSYKELTTWYEAQIFHECQLSGESGWKPQGLIEVSH  1997
AGT56093.1   1917  RSDGSDMLQKLVDNVNTALVELYLHQDREYSESVRDFYLEKLPPGSYKELTTWYEAQIFHECQLSGESGWKPQGLIEVSH  1996
QBZ78358.1   1917  RSDGSDMLQKLVDNVNTALVELYLHQDKGYSDSVRNFYLKRLPPGSYKELTTWYEAQAFHECQLSGESGWKPQGLIEVSH  1996
AGD98722.1   1918  RSDGSDMLQKLVDNVNTALVELFLHQDREYLESVRNFYLEKLPPGSYKELTTWHEAQTFHECQLSGESGWKPQGLIEVSH  1997
DAZ85702.1   1918  RSDGSDMLQKLVDNVNTALVELFLHQDREYLESVRNFYLEKLPPGSYKELTTWYEAQTFHECQLSGESGWKPQGLIEVSH  1997
QGY99255.1   1918  RSDGSDMLQKLVDNVNTALIELYLHQDREYFESVRDFYLEKLPPGSFKELTTWYEAQTFHECQLSGESGWKPQGLIEVSH  1997
DAZ85701.1   1918  RSDGSDMLQKLVDNVETALVELYLHQDREYFESVRNFYLEKLPPGAFKELTTWYAAQTFHECQLSGESGWKPQGLIEVSH  1997
DAZ85704.1   1920  RSDGSDMLQKLVDNVNSALVELFLHQDKEYFESVRNFYLEKLPPGSYKELTTWYQAQTFHECQLSGESNWKPQGLIEISH  1999
QYU59218.1   1918  RSDGSDMLQKLVDNVNNALVELYLHQDREYFESVRNFYLGKLPPGSFKELTTWYAAQTFHECQLSGESGWKPQGLIEVSH  1997
ADJ39329.1   1920  RSDGSDMLQKLVDNVNSALVELFLHQDREYSESVRNFYLEKLPPGSYKELTTWYQAQTFHECQLSGESNWKPQGLIEISH  1999
QBZ78354.1   1918  RSDGSDMLQKLIDNVNTALVELYLHQDREYFESVRNFYLEKLPPGSFKELETWYAAQTFHECQLSGESGWKPQGLIEVTH  1997
QBZ78356.1   1918  RSDGSDMLQKLIDNVNTALVELYLHQDREYFESVRNFYLEKLPPGSFKELETWYAAQTFHECQLSGESGWKPQGLIEVTH  1997
QYA72350.1   1918  RSDGSDMLQKLVDNVNSALVELFLHQDREYFESVRNFYLEKLPPGSYKELTTWYQAQSFHESQLCGESNWKPQGLIEISH  1997
ADJ39331.1   1920  RSDGSDMLQKLVDNVNSALVELFLHQDREYSESVRNFYLEKLPPGSYKELTTWYQAQAFHECQLSGESNWKPQGLIEISH  1999
DAZ85705.1   1920  RSDGSDMLQKLVDNVNSALVELFLHQDKEYFESVRNFYLEKLPPGSYKELTTWYQAQTFHECQLSGESNWKPQGLIEISH  1999
DAZ85709.1   1920  RSDGSDMLQKLVDNVNSALVELFLHQDREYSESVRNFYLEKLPPGSYKELTTWYQAQSFHESQLCGESNWKPQGLIEISH  1999
DAZ85710.1   1920  RSDGSDMLQKLVDNVNSALVELFLHQDREYSESVRNFYLEKLPPGSYKELTTWYQAQSFHESQLCGESNWKPQGLIEISH  1999
DAZ85703.1   1920  RSDGSDMLQKLVDNVNSALVELFLHQDKEYFESVRNFYLEKLPPGSYKELTTWYQAQTFHECQLSGESNWKPQGLIEISH  1999
ADJ39330.1   1920  RSDGSDMLQKLVDNVNSALVELFLHQDREYLESVRNFYLEKLPPGSYKELTTWYQAQSFHESQLCGESNWKPQGLIEISH  1999
ACF32434.1   1920  RSDGSDMLQKLVDNVNTALVELFLHQDKEYFDSVRNFYLEKLPPGSYKELTTWYQAQTFHECQLSGESNWKPQGLIEISH  1999
QYU59216.1   1917  RSDGSDMLQKLLDNVNSALIELFLHQDRDYFESVRSFYLEKLPPGSYKELITWYQAQTFHECQLSGESNWKPQGLIEISH  1996

YP_053925.1  1998  GASFASFVQQNGTELERHDICPGLAISGSKYIAREEEILMSLSSLLPGDINAVKLTLKCGDGIGRLPSKASVLSQRKPGI  2077
AGT56093.1   1997  GASFASFVQQNGTELERHDICPGLAISGSKYIAREEEILMSLSSLLPGDINAVKLTLKCGDGIGRLPSKASVLSQRKPGI  2076
QBZ78358.1   1997  GACFASFVQQNGTELERHDICPGLAISGSRYIAREEEILMSLSSLLPGDVNAVKLTLKCGDGIGRLPSKASVLSQKRSGI  2076
AGD98722.1   1998  NASFASFVQQNGTELERHDICPGLAISGARYIAKEEEILLSLSSLLPGDVNAVKLTLKCGDGIGRLPSRASVLNQRKPGI  2077
DAZ85702.1   1998  NASFASFVQQNGTELERHDICPGLAISGARYIAKEEEILMSLSSLLPGDVNTVKLTLKCGDGIGRLPSKTSVLDQRKPGI  2077
QGY99255.1   1998  DASFASFVQQNGSELERHDICPGLAVSGARYIATEEEILLSLSSLLPGDVNATRLTLKCGDGIGRLPSKTSVLDQRKPGI  2077
DAZ85701.1   1998  NASFASFVQQSGSELERHDICPGLAICGARYIATNEEILISLSSLLPGDVNATQLTLNCGDGIGRLPSKASVLNQRKPGV  2077
DAZ85704.1   2000  GAGFASFVQQDGTELERHDICPGLAISGARYIAKNEEILMSLSSLLPGDVNATKLELKCGDGIGRLPSKASVLAQRKPGV  2079
QYU59218.1   1998  DASFASFVQQNGSELERHAICPGLAISGARYIATQEEILLSLSSLLPGDVNAVQLTLKCGDGIGRLPSKTSVLDQRKPGV  2077
ADJ39329.1   2000  GAGFASFVQQDGTELERHDICPGLAISGARYIAKKEEILMSLSSLLPGDVNATKLELKCGDGIGRLPSKASVLAQRKPGI  2079
QBZ78354.1   1998  NASFARFVQQSGSELERHDICPGLAISGARYIATNEEILISLSSLLPGDVNATQLTLNCGDGIGRLPSRTSVLDQRKPGV  2077
QBZ78356.1   1998  NASFARFVQQSGSELERHDICPGLAISGARYIATNEEILISLSSLLPGDVNATQLTLNCGDGIGRLPSRTSVLDQRKPGV  2077
QYA72350.1   1998  GAGFASFVQQDGTELERHDICPGLAISGARYIAKDQEILMSLSSLLPGDVNAHRLELKCGDGIGRLPSKASVLAQRKPGI  2077
ADJ39331.1   2000  GAGFASFVQQDGTELERHDICPGLAISGARYIAKNEEILMSLSSLLPGDVNATKLELKCGDGIGRLPSKASVLAQRKPGI  2079
DAZ85705.1   2000  GAGFASFVQQDGTELERHDICPGLAISGARYIAKNEEILMSLSSLLPGDVNATKLELKCGDGIGRLPSKASVLAQRKPGV  2079
DAZ85709.1   2000  GAGFASFVQQDGTELERHDICPGLAISGARYIAKNEEILMSLSSLLPGDANATKLELKCGDGIGRLPSKASVLAQRKPGV  2079
DAZ85710.1   2000  GAGFASFVQQDGTELERHDICPGLAISGARYIAKNEEILMSLSSLLPGDANATKLELKCGDGIGRLPSKASVLAQRKPGV  2079
DAZ85703.1   2000  GTGFASFVQQDGTELERHDICPGLAISGARYIAKNEEILMSLSSLLPGDVNATKLELKCGDGIGRLPSKASVLAQRKPGV  2079
ADJ39330.1   2000  GAGFASFVQQDGTELERHDICPGLAISGARYIAKSEEILLSLSSLLPGDVNVHKLDLKCGDGIGRLPSKASVLAQRKPGI  2079
ACF32434.1   2000  GVGFASFVQQDGTELERHDICPGLAISGARYIAKKEEILMSLSSLLPGDVNAHKLELKCGDGIGRLPSKASVLAQRNPGI  2079
QYU59216.1   1997  GSGFASFVQQDGTELERHDICPGLAISGARYIAKDDEILLSLSSLLPGDVGAHKLDLKCGDGIGRLPSKMSCLAQRKPGV  2076

YP_053925.1  2078  VMQLCARAIKEKKTLVIRDERPYIGGWAMACICGESFGFSIKDTLALYANLMGPNRKNGLATYFTDFDSPVHVKKIHAIT  2157
AGT56093.1   2077  VMQLCARAIKEKKTLVIRDERPYIGGWAMACICGESFGFSIKDTLALYANLMGPNRKNGLATYFTDFDSPVHVKKIHAIT  2156
QBZ78358.1   2077  AMQMCARALKEKKTLVIRDERPYIGGWAMACVCGESFGFSIKDTLALYANLMGPNRKNGLATYFTDFDSPVHVKKIHAIT  2156
AGD98722.1   2078  VMQLCARALKEKKTLVIRDERPYIGGWAMACICGESFGFSIKDTLALYANLMGPNRKNGLATYFTDFDSPIHVKRVHAVT  2157
DAZ85702.1   2078  VMQLCERALKEKKTLVIRDERPYIGGWAMACICGESFGFSIKDTLALYANLMGPDRKNGLATYFTDFDGPVHVKRVHAIT  2157
QGY99255.1   2078  VMQLCKRAREERKTLVIRDERPYIGGWAIACICGESFGFSTRDTLALYANLMGPNRTNGLASYFADFESPVCVKKVHAIT  2157
DAZ85701.1   2078  VMRLCKRARDEKKTLVIRDERPYIGGWAIACICGESFGFSTRDTLALYANLMGPNRTNGLASYFADFESPVCVKKVHAVT  2157
DAZ85704.1   2080  VMRLCKQAIDEKKTLVIRDERPYIGGWAMACICGESFGFSIRDTLALYANLLGPERKNGLAGYFSNFESPVHVKKVHAIT  2159
QYU59218.1   2078  VMRLCKRAREEKKTLVIRDERPYIGGWAIACICGESFGFSIRDTLALYANLMGPNRTNGLASYFTDFESPVHVKKVHAIT  2157
ADJ39329.1   2080  VMRLCKQALDEKKTLVIRDERPYIGGWAMACICGESFGFSIRDTLALYANLLGPERKNGLAGYFSNFESPVHVKKVHAIT  2159
QBZ78354.1   2078  VMRLCKRARDEKKTLVIRDERPYIGGWAIACICGESFGFSIRDTLALYANLMGPDRTNGLASYFANFESPVCVKKVHAIT  2157
QBZ78356.1   2078  VMRLCKRARDEKKTLVIRDERPYVGGWAIACICGESFGFSIRDTLALYANLMGPDRTNGLASYFANFESPVCVKKVHAIT  2157
QYA72350.1   2078  VMRLCKQALDERKTLVIRDERPYIGGWAMACICGESFGFSIRDTLALYANLLGPERKNGLAGYFSNFESPVHVKKVHAIT  2157
ADJ39331.1   2080  VMRLCKQALDEKKTLVIRDERPYIGGWAMACICGESFGFSIRDTLALYANLLGPKRKNGLAEYFSNFESPVHVKKVHAIT  2159
DAZ85705.1   2080  VMRLCKQAIDEKKTLVIRDERPYIGGWAMACICGESFGFSIEDTLALYANLLGPKRKNGLAGYFSNFESSVHVKKVHAIT  2159
DAZ85709.1   2080  VMRLCKQALDEKRTLVIRDERPYIGGWAMACICGESFGFSIRDTLALYANLLGPNRKNGLAAYFLNFESPVHVKKVHAIT  2159
DAZ85710.1   2080  VMRLCKQALDEKRTLVIRDERPYIGGWAMACICGESFGFSIRDTLALYANLLGPNRKNGLAAYFSNFESPVHVKKVHAIT  2159
DAZ85703.1   2080  VMRLCKQALDEKKTLVIRDERPYIGGWAMACICGESFGFSIRDTLALYANLLGPERKNGLAGYFSNFESPVHVKKVHAIA  2159
ADJ39330.1   2080  VMRLCKQALNEKKTLVIRDERPYIGGWAMACICGESFGFSIRDTLALYANLLGPNRKNGLAGYFSNFESPVHVKKVHAIT  2159
ACF32434.1   2080  VMRLCKQALEEKKTLVIRDERPYIGGWAMACICGESFGFSIRSTLALYANLLGPDRKNGLAGYFSNFESPVHVKKVHAIT  2159
QYU59216.1   2077  VMKLCRRALDEKKTLVIRDERPYIGGWAIACICGESFGFPIRSTLALYANLLGPDRKNGLAGYFTDFESPVNIKKVFAKT  2156
```

```
YP_053925.1  2158  NGEEGVAMLKDSFAFCEPTTIAATSCDTRKEMVSHLPTSFPNIVLIGGISYPKEGGEPGALYSPTDVVMSKKLQGVYVSE  2237
AGT56093.1   2157  NGEEGVAMLKDSFAFCEPTTIAATSCDTRKEMVSHLPTSFPNIVLIGGISYPKEGGEPGALYSPTDVVMSKKLQGVYVSE  2236
QBZ78358.1   2157  NGEEGVAMLKDSFAFCEPTIVEATLCDTRKEMLSHPSTSFPNIVLIGGISYPKEGGEPGALYSPTDVVMAKKVQGVYVSE  2236
AGD98722.1   2158  NGEEGVAMLKESFAFCEPTTVVATSCDTRREMVSHLPMSFPNVVLIGGISFPKEGGEPGALYSSTDVVMSKKLQGVYVSE  2237
DAZ85702.1   2158  NGEEGVAMLKDSFAFCEPTVVVATSCDTRREMVSHSPVSFPSVVLIGGISFPKEGGEPGALYSSTDVVMSKKIQGVYVSE  2237
QGY99255.1   2158  NGEEGATMLRDVFAFCEPRIVNATFSNTRKEMLAHQPELYPNVVLIGGISFPKESGEPGALYSHVDVAMSKEVKGVFVSE  2237
DAZ85701.1   2158  NGEEGATMLKDVFTFCEPRIVNATFSDTRKEMLIHQPESFPNIVLIGGISFPKEGGEPGALYSHVDVTMSKEVKGVFVSE  2237
DAZ85704.1   2160  NGEEGAAMLKEVFTFCEPKIIAATSRDTRKEMLTHPPESFPNVVLIGGVSFPKEGGEPGASYSHTDVIMSKKTKGVYVSE  2239
QYU59218.1   2158  NGEEGAITLKDVFAFCEPKVVNATFSNTREEMLTHRPESFPNVVLIGGISFPKENGEPGALYSHTDVAMSKEVKGVYVSE  2237
ADJ39329.1   2160  NGEEGAAMLKEVFTFCEPKIYAATSCDTRREMLTHPPESFPNIVLIGGVSFPKEGGEPGALYSHTDITMSKKTKGVYVSE  2239
QBZ78354.1   2158  NGEEGATILKDVFAFCEPKIVNATFSNTRKEMLIHHPESFPNVVLVGGISFPNESGEPGALYSHVDVTMSNKVKGVYVSE  2237
QBZ78356.1   2158  NGEEGATILKDVFAFCEPKIVNATFSNTRKEMLIHHPESFPNVVLVGGISFPNENGEPGALYSHVDVTMSNKVKGVYVSE  2237
QYA72350.1   2158  NGEEGAAMLKEVFTFCDPKIIAATSHETRKEMLTHPPESFPNVVLIGGISFPKEGGEPGALYSHTDIVMSKKTKGVYVSE  2237
ADJ39331.1   2160  NGEEGAAMLKEVFTFCEPKICAATSCDTRREMLAHPPELFPNIVLIGGVSFPKEGGEPGALYSHTDIVMSKKTKGVYVSE  2239
DAZ85705.1   2160  NGEEGAVMLKEVFTFCEPKIVAATFCDTRKEMLTHPPESFPNVVLIGGVSFPREGGEPGALYSHTDVAMSKKVKGVYVSE  2239
DAZ85709.1   2160  NGDEGVEALKDVFTFCEPKIVSASSHDTRKEMLTHPPESFPNVVLIGGVSFPKEGGEPGALYSHTDITMAKQVKGAYVSE  2239
DAZ85710.1   2160  NGDEGVEALKDVFTFCEPKIVSASSHDTRKEMLTHPPESFPNVVLIGGVSFPKEGGEPGALYSHTDITMAKQVKGAYVSE  2239
DAZ85703.1   2160  NGEEGAAMLKEVFTFCEPKIVAATSCDTRKEMLTHPPESFPNVVLIGGVSFPKEGGEPGALYSHTDITMSKKVKGVYVSE  2239
ADJ39330.1   2160  NGEEGAAELKEVFTFCEPRIVAASFGETRKEMLAHPPESFPNVVLVGGISFPKEGGEPGALYSHTDIVMSKKTKGVYVSE  2239
ACF32434.1   2160  NGEEGATELKEVFTLCEPKIVRASSCETRTEMLAHPSEAFPSVVLIGGISYPKEGGEPGALYSHTDVTMSKKNKGVYVSE  2239
QYU59216.1   2157  NCDEGVAMLKEVFTFCEPIKVQASKQETRSEMLSHPPEAFPNVVLIGGISFPNEGGEPGALYSQADKVMSKKVKGVYVSE  2236

YP_053925.1  2238  AVLKCCLRCPGAAVKTVLQTSSPGSSLSQAHFRSLRRVQSHRCMRKS  2284
AGT56093.1   2237  AVLKCCLRCPGAAVKTVLQTSSPGSSLSQAHFRSLRRVQSHRCMRKS  2283
QBZ78358.1   2237  AVLKCCLRCPGVAVKTVLQTSSPGSSLSQAHFRSIRQVQSHRCVRKS  2283
AGD98722.1   2238  AILKCCLRCPGVAVKTVLQTSSPGSSLSQAHFRSLRRIQSHRCVRKP  2284
DAZ85702.1   2238  AILKCCLRCPGAAVKTVIQTSSPGSSLSQAHYRSLCRIQSHRCVRRT  2284
QGY99255.1   2238  AVLKCCKRCPGVATKTVLHTSTFGTNLSKAHSRSLRQLQTHQCVRR-  2283
DAZ85701.1   2238  AVLKCCMRCPGVATKTVLHNSTFGANLSKAHSRSLRQLQTHRCVRR-  2283
DAZ85704.1   2240  AILKCCLRCPGVTTKTVLQTSTIGSSLSKAHLKSLRQLQTHQCVRR-  2285
QYU59218.1   2238  AVLKCCMRCPGVATRTVLHTSTFGTNLSKAHLRSLRQLQTHQCTRR-  2283
ADJ39329.1   2240  AILKCCLRCPGVSTKTVLQTSTVGSNLSKAHLKSLRQLQTHQCVRR-  2285
QBZ78354.1   2238  AVLKCCMRCPGVATKTVLHTSTFGANLSKAHLRSLRQLQTHQCVRR-  2283
QBZ78356.1   2238  AVLKCCMRCPGVATKTVLHTSTFGANLSKAHLRSLRQLQTHQCVRR-  2283
QYA72350.1   2238  AILKCCLRCPGVTTKTVLQTSTIGSNLPKAHLKSLRQLQTHQCVRR-  2283
ADJ39331.1   2240  AILKCCLRCPGVTTKTVLQTSTIGSSLSKAHLKSLRQLQTHQCVRR-  2285
DAZ85705.1   2240  AILKCCLRCPGVSTRTVLQTSTIGSNLSKAHLKSLRQLQTHRCVRK-  2285
DAZ85709.1   2240  AILKCCLRCPGVTTKTVLQTSTIGSNLCKAHLKSLRQLQTHQCVRR-  2285
DAZ85710.1   2240  AILKCCLRCPGVTTKTVLQTSTIGSNLCKAHLKSLRQLQTHQCVRR-  2285
DAZ85703.1   2240  AILKCCLRCPGVTTKTVLQTSTIGSNLSKAHLKSLRQLQTHQCVRR-  2285
ADJ39330.1   2240  AILKCCLRCPGVTTKTVLQTSTIGSSLSKAHLKSLRQLQTHQCVRR-  2285
ACF32434.1   2240  AILKCCLRCPGVATKTVLQTTEIGSSLSKAHLKSLRRLQTHQCVRR-  2285
QYU59216.1   2237  AILKCCLRCPGVTTKTILHTTDIGSSIAKAHLRSLRKIQTHQCVRK-  2282
```

ArMV isolates RNA2 polyprotein

```
YP_053924.1  1     MAKFYYSDRRLACWAAGKNPHLG-GSIESWLAAIKSDSSFRQTVKEDVQVNRLQPTAVRMFSWKVGSGPIDNPEKCNWHY  79
DAZ85714.1   1     MGKFYYSNRRLACWAAGKNPHLG-GSIEQWLAAINTDSSFRQTVKEDVQENRLQPTAVRMFSYKIGSGPIDNPDMSDWQF  79
QBZ78359.1   1     MTRFYYSNRRLACWAAGKNPHLG-GSIERWLAAITTDSSFRQTVKEDVQENRNEPTAIRMFSWKLGCGPIDNPAKCNWHY  79
QBZ78357.1   1     MGKFYYSNRRLACWAAGKNPHLG-GSVEQWLAAINTDSSFRQTVKEDVQENRLQPTAVRMFSYKIGSGPIDNPDMLDWQF  79
QBZ78355.1   1     MGKFYYSNRRLACWAAGKNPHLG-GSVEQWLAAINTDSSFRQTVKEDVQENRLQPTAVRMFSYKIGSGPIDNPDMLDWQF  79
ABR09918.1   1     MGKFYYSNRRLACWAAGKNPHLG-GSVESWLAAITTDSSFRQTVKEDVQENRDEPTAIRMFSWKIGYGPIDNPEKCNWHF  79
QGY99256.1   1     MGKFYYSNRRLACWAAGKNPHLG-GSVEQWLAAINTDSSFRQTVKEDVQENRLQPTAVRMFSYKVGSGPIDNPDVSDWQF  79
QYA72351.1   1     MGKFYYSSRRLACWAAGKNPHLG-GSIEQWLAAINTDSSFRQTVKEDVQANRAEPTAIRMFSWKVGSGPIDNPDMLDWQF  79
AEK20744.1   1     MGKFYYSNRRLACWAAGKSPDVR-GSIEQWLAAITTDSSFRQTVKEDVQANRSEPTAIRMFSWKVGTGPIDNPEMCNWHY  79
DAZ85717.1   1     MGKFYYSNRRLACWVAGKNPHLG-GSIEQWLAAINTDSSFRQTVKEDVQNNRNEPTAIRVFSWKVGYGPIDNPDSCNWDF  79
ADJ39333.1   1     MGKFYYSNRRLACYAQATNRHLG-GSFEQWLQCME-DSAFRAEVKARVQSEREEVRVKRLFSYPVGRGPAEDPRGVNWAY  78
DAZ85715.1   1     MGKFYYSNRRLACYAQATNRHLG-GSFEQWLQCME-DSAFRAEVKARVQSEREEVRVKRLFSCPIGRGPAEDPRGVNWAY  78
DAZ85721.1   1     MGKFYFSNRRLACYAQATNRHLG-GSFEQWLQCME-DSAFKAEVKARVQSEREEVRVKRLFSCPVGRGPAEDPRGVNWAY  78
ACF32435.1   1     MGKFYFSNRRLACYAQATNRHLG-GSFEQWLQCME-DSAFKAEVKARVQSEREEIRVKRLFSYPVGRGPAEDPRGVNWAY  78
ADJ39332.1   1     MGKFYYSNRRLACYAQATNRHLG-GSFEQWLQCME-DSAFRAEVKARVQTEREEVRVKRLFSHPVGRGPAEDPRGVNWAY  78
QYU59217.1   1     MGKFYYSNRRLACYAQATNRHLG-GSFAQWLQCME-DSAFRAEVKARVQTEREEVRVKRLFSRPVGLGPAEDPRGVNWAY  78
DAZ85724.1   1     MGKFYFSNRRLACYAQATNRHLG-GSFEQWLQCME-DSAFKAEVKARVQSEREEVRVKRLFSCPVGRGPAEDPRGVNWAY  78
DAZ85722.1   1     MGKFYFSNRRLACYAQATNRHLG-GSFEQWLQCME-DSAFKAEVKARVQSEREEVRVKRLFSCPVGRGPAEDPRGVNWAY  78
Q65030.1     1     MGKFYYSNRRLACYAQATNRHLG-GSFEQWLQCME-DSAFRAEVKARVQSEREEVRVRRLFSYPVGSGPAEDPRGVNWAY  78
DAZ85716.1   1     MGIFYSCYQRFAGPPKVEAPYV------RPMTFLE--------ARASMRMAQAEYAAAQLFK----ELPLEYPQGA----  58
BAF35853.1   1     MGILYSVYRWIDGPPPAPTYNIY-GNFREIQATLR------------------EAENARQNMYMFLELPLEYPQGA----  57
CAA57413.1   1     MGILYSCYRRFACQPVVDVPYI------RPTTFLE--------ARASMRIAQAEGAAAALFA----ELPLEYPQGV----  58
BAF35852.2   1     MGILYSIYRFIDGPPPERTFNLYQGNFRETQAILR------------------EAEEARQNMYRFLELPLEYPQGA----  58
Q91HK5.1     1     MGKFYYSNRRLACWAAGKNPHLG-GSVEQWLAAINTDSSFRQTVKEDVQDNREQPTAIRMFSWKVGFGPIDNPEKCDWHF  79
BAF35851.2   1     MVKFYYSNRRLVCWAVSKNLHLG-GSVEQWLQCVE-DSAFRAEVKSDVVANRDHPTAIRTFSYKVGYGPIDDPRYADWGY  78


YP_053924.1  80    VLTGEVP---------AQP-TEPVKAREVV-VP-------------PVK---VIPSPPPVPR-PYFRPVGAFAPTRSGFI  131
DAZ85714.1   80    VLTGKLS---------RQP-TEPVKAQEVV-VP-------------PVK---VIPSPPPVPR-PYFRPIGAFAPTRLGFI  131
QBZ78359.1   80    VLTGEVP---------VQP-TEPAKAQEVV-VP-------------PVK---VVPSPPPMPR-PYFRPIGAFAPTRSGFI  131
QBZ78357.1   80    VLTGKPS---------RQP-TEPVKAQEVV-VP-------------PVK---VIPSPPPVPR-PYFRPVGAFAPTRSGFI  131
QBZ78355.1   80    VLTGKPS---------RQP-TEPVKAQEVV-VP-------------PVK---VIPSPPPVPR-PYFRPVGAFAPTRSGFI  131
ABR09918.1   80    VLTGERP---------AQP-SELVEAQKVV-VP-------------PVK---VIPSPPPMPR-PYFRPVGAFAPTRSGFI  131
QGY99256.1   80    VLTGKSS---------RQP-IEPVKAQEVV-VS-------------PVK---VIPSPPPVPR-PYFRPIGAFAPTRSGFI  131
QYA72351.1   80    VLTGKLSR---------QP-TEPVKAQEVV-VP-------------PVK---VIPSPPPVPR-PYSRPIGAFAPTRSGFI  131
AEK20744.1   80    VLTGEEP---------AQP-TEPVKAQEVV-VP-------------PVK---VIPSPPPMPR-PHFRPIGAFAPTRSGYI  131
DAZ85717.1   80    VLTGRRPE---------QP-TGPVRAQEVV-VP-------------PVK---VIPSPPPMPR-PYFRPIGAFAPTRSGFI  131
ADJ39333.1   79    ICLGTTAHWAGVPGDMIAP-VEPVEVPKVV-VP-------------PVK---VIPSPPPMPR-PYFRSIGAFAPTRSGFI  139
DAZ85715.1   79    ICLGTTAFWAGVPGDMVPP-PEPVKAQEVV-VP-------------PVK---VIPSPPPVPR-PYFRPVGAFAPTRSGFI  139
DAZ85721.1   79    ICLGTTAHWAGVPGDMIPP-TEPVKAQEVV-VP-------------PVK---VIPSPPPVPR-PYSRPIGAFAPTRSGFI  139
ACF32435.1   79    ICLGTTAHWAGVPGDMVSI-PKPPVAPKVV-VP-------------PVK---VIPSPPPVPR-PHFRPIGAFAPTRSGFI  139
ADJ39332.1   79    ICLGTTAHWAGVPGDMVPP-PDPVEVIKGG-VP-------------PVK---VIPSPPPMPR-PYFRPIGAFAPTRSGFI  139
QYU59217.1   79    ICLGTTAYWAGVPGDMVPP-PEPVQAQEVV-VP-------------PVK---VIPSPPPVPR-PYFRPVGAFAPTRSGFI  139
DAZ85724.1   79    ICLGTTAHWAGVPGDMIPP-TEPVKAQEVV-VP-------------PVK---VIPSPPPVPR-PYSRPIGAFAPTRSGFI  139
DAZ85722.1   79    ICLGTTAHWAGVPGDMIPP-TEPVKAQEVV-VP-------------PVK---VIPSPPPVPR-PYSRPIGAFAPTRSGFI  139
Q65030.1     79    ICLGTTAHWAGVPGDMVPP-PEPVKAQEVV-VQRSVGEDGRTGYRRQCL---NIPNPPPMPK-PYSRPIGAFAPTRSGFI  152
DAZ85716.1   59    --------------PLVLP-TEPVKAQEVV-VP-------------PVK---VIPSPPPMPR-PYFRPIGAFAPTRSGFI  105
BAF35853.1   58    --------------PLVLP-TEPVKAQEVV-VP-------------PVK---VIPSPPPMPR-SYSRPIGAFAPTRSGFI  104
CAA57413.1   59    --------------PLVQP-TEPVKAQEVM-VP-------------PVR---VIPSPPPVPR-PYSRPIGAFAPTRSGFI  105
BAF35852.2   59    --------------PLVLP-TEPVKAQEVV-VP-------------SVK---VIPSPPPIPR-PYFRPIGAFAPTQSGFI  105
Q91HK5.1     80    VLTGERP---------AQP-TRPVKADEVV-VV-------------PQLKKVVIPSPPPPPA-VYFRAVGAFAPTRSGFI  134
BAF35851.2   79    VLTGVATP--GLRGGVVPPPTEPVKAHEVVEVP-------------QPRVE--IPSPPPVPTYTLYRSVGAFAPTRSGFI  141


YP_053924.1  132   RATVERLSRKREESRAAALFAELPLEYPQGAPLVVPRG--FAAMRWTYHATWRRWYDASDERALRVHPGGPALPPPPPPP  209
DAZ85714.1   132   RATVERLTREREESRAAALFAELPLEYPQGTPLVVPRG--FAAMRWTYHTTWRRWYDTSDERALRVHPGGPALPPPPPPP  209
QBZ78359.1   132   RATVERLSREREESRAAALFAELPLEYPQGAPLVVPRG--FAAMRWTYHTTWRRWYDASDERALRVHPGGPALPPPPPPP  209
QBZ78357.1   132   RATVERLSREREESRAAALFAELPLEYPQGAPLVVPRG--FAAMRWTYHTTWRRWYDTSDERALRAHPGGPALPPPPPPP  209
QBZ78355.1   132   RATVERLSREREESRAAALFAELPLEYPQGAPLVVPRG--FAAMRWTYHTTWRRWYDTSDERALRAHPGGPALPPPPPPP  209
ABR09918.1   132   RATVERLSREREESRAAALFAELPLEYPQGVPLVVPRG--FAAMRWTYHTTWRRWYDTSDERALRVHPGGPALPPPPPPP  209
QGY99256.1   132   RATVERLSREREESRAAALFAELPLEYPQGAPLVVPRG--FAAMRWTYHTTWRRWYDTSDERALRVHPGGPALPPPSLPP  209
QYA72351.1   132   RATVERLSRAREESRAAALFAELPLEYPQGAPLVVPRG--FAAMRWTYHATWRRWYDTSDERALRVHPGGPALPPPPPPP  209
AEK20744.1   132   RATVERLSQEREESRAAALFAELPLEYPQGAPLVVPRG--FAAMRWTYHTTWRRWYDTSDERALRVHPGGPALPPPPPPP  209
DAZ85717.1   132   RATVERLTREREESRAAALFAELPLEYPQGAPLVVPRG--FAAMRWNYHATWRRWYDTSDERALCVHPGGPALPPPPPPP  209
ADJ39333.1   140   RATVERLTREREESRAAALFAELPLEYPQGAPLVVPRG--FAAMRWNYHATWRRWYDTSDERALRVHPGGPALPPPPPPP  217
DAZ85715.1   140   RATVERLTREREESRAAALFAELPLEYPQGAPLVVPRG--FAAMRWNYHATWRRWYDTGDERALRVHPGGPALPPSPPPP  217
DAZ85721.1   140   RATVERLTREREESRAAALFAELPLEYPQGAPLVVPRG--FAAMRWTYHTTWRRWYDTSDERAQRVHPGGPALPPPPPPP  217
ACF32435.1   140   RATVERLTREREESRAAALFTELPLEYPQGAPLVVPCG--FAAMRWTYHTTWRRWYDASDERALRVHPGGPALPPPPPPP  217
ADJ39332.1   140   RATVERLTREREELRAAALFAELPLEYPQGAPLVVPRG--FAAMRWTYHTTWRRWYDTSDERALRVHPGGPALPPPPPPP  217
QYU59217.1   140   RATVERLSCAREESRAAALFAELPLEYPQGAPLVVPRG--FAAMRWTYHATWRRWYDTSDERALRVHPGGPALPPPPPPP  217
DAZ85724.1   140   RATVERLTREREESRAAALFAELPLEYPQGAPLVVPRG--FAAMRWTYHTTWRRWYDTSDERAQRVHPGGPALPPPPPPP  217
DAZ85722.1   140   RATVERLTREREESRAAALFAELPLEYPQGAPLVVPRG--FAAMRWTYHTTWRRWYDTSDERAQRVHPGGPALPPPPPPP  217
Q65030.1     153   RATVKRLTREREESRAAALFAELPLEYPQGAPLVVPRG--FAAMRWTYHATWSRWYDTSDERALRTHPGGPALPPLPPPP  230
DAZ85716.1   106   RATVERLTREQEESRAAALFAELPLEYPQGAPLVIPRG--FAAMRWNYHATWKRWYDTSDERALRVHPGGPALPPPPPPP  183
BAF35853.1   105   RATVERLTREREESRAAALFAELPLEYPQGAPLVVPRG--FAAMRWNYHATWRRWYDASDERVLCAHPGGPALPPPPPPL  182
CAA57413.1   106   RATMERLTREREESRAAALFAELPLEYPQGAPLVVPRG--FAAMRWTYHATWRRWYDTSDERPLRAHPGGPALPPPSPPP  183
BAF35852.2   106   RATVERLTRERDESRAAALFAELPLEYPQGVPLVVPRG--FVAMRWTYHATWRRWYDTSDERALHIHPGGPALPPSPPPP  183
Q91HK5.1     135   RATVERLSREREESRAAALFAELPLEYPQGAPLKLSLAMKFAMLK---HTTWRKWYDTSDERLSEAHPGGPCLPP---PP  208
BAF35851.2   142   RAIVERLRRKRAESTAAALFAELPLEYPQGAPLKLTVIQ---KMGMVRYQSWKLWYDTSDARISGHYKGVSPFPPPPPPP  218
```

```
                                                                      2a-MP cleavage site

YP_053924.1  210   PIQKPPSFEERLQAALERQSCARAFALETSLGLNMAWLGTAAIPSTSVCCA-----DGRTTGGQTIAQEADPINHRVTSN  284
DAZ85714.1   210   PIQKPPSFEERLQAALERQSCARAFALETSLGLNMAWVGTAAIPNTSVCCA-----DGRTTGGQTIAQEADPINHRVASN  284
QBZ78359.1   210   PIQKPPSFEERLQAALQRQSCARAFALETSLGLNMVWVGMAAIPSTSVCCA-----DGRTTGGQTIAQEADPINHRVASN  284
QBZ78357.1   210   PIQKPPSFEERLQAALERQSCARAFALETSLGLNMAWVGTAAIPSTSVCCA-----DGRTTGGQTIAQEADPINHRVASN  284
QBZ78355.1   210   PIQKPPSFEERLQAALERQSCARAFALETSLGLNMAWVGTAAIPGTSVCCA-----DSRTTGGQTIAQEADPINHRVASS  284
ABR09918.1   210   SIQKPPSFEERLRAALERQSCARAFALETSLGLNMAWVGMAAIPSTSICCA-----DGRTTGGQTIAQEADPINHRVASN  284
QGY99256.1   210   PIQKPPSFKERLRAFLERQSCARAFALETSLGLNMAWVGTATIPSTSICCA-----DGRTTGGQTIAQEADPISHRVASS  284
QYA72351.1   210   PIQKPPSFEERLQAALQRQSYARAFALETSLGLNMAWVGTATIPSISVCCA-----DGRTTGGQIIAQEADPINHRVASN  284
AEK20744.1   210   PIQKPPSFEERLRAALERQSCARAFALETSLGLNVAWVGMATIPSTSICCA-----DGRTTGGQTIAQEADPINHRVASN  284
DAZ85717.1   210   PIQKPPSFEERLQAALQRQSRARAFAPETSLGLNMAWVGKAVIPSISVCCA-----DGRTTGGQTIAQEADPINHRVASN  284
ADJ39333.1   218   PIQKPPSFEERLQAALQRQSCARAFALETSLGLNMAWVGTATVPGISVCCA-----DGRTTGGQTIAQEADPINHRVASN  292
DAZ85715.1   218   PIQKPLSFEERLQAALEKQSCARAFALETSLGLNMAWVGTAAIPSISICCA-----DGRTTGGQTIAQEADPINHRVASN  292
DAZ85721.1   218   PIQKPPSFEERLQAALQRQSCARAFALETSLGLNMAWVGKAAIPSISVCCA-----DGRTTGGQTIAQEADPINHRVASN  292
ACF32435.1   218   PIQKPPSFEERLQAVLQRQSCARAFALETSLGLNMAWVGTATIPSISVCCA-----DGRTTGGQTIAQEADPINHRVASN  292
ADJ39332.1   218   PIQKPPSFEERLQAALDRQSCARAFALETSLGLNMAWVGTAVVPSISVCCA-----DGRTTGGQTIAQEADPINHRVASN  292
QYU59217.1   218   PIQKPPSFEERLQAALKKQSCARAFALETSLGYNMAWVGITDIPSISICCA-----DGRTTGGQTIAQEADPISHRVTSN  292
DAZ85724.1   218   PIQKPPSFEERLQAALSRQSCARAFALETSLGLNMAWVGTAAIPSISVCCA-----DGRTTGGQTIAQEADPINHRVASN  292
DAZ85722.1   218   PIQKPPSFEERLQAALSRQSCARAFALETSLGLNMAWVGTAAIPSISVCCA-----DGRTTGGQTIAQEADPINHRVASN  292
Q65030.1     231   PIQKPPSFEERLQAALHRQSCARAFALETSLGLNMAWVGMATIPSTSVCCA-----DGRTTGGQTIAQEADPLTHRVSSN  305
DAZ85716.1   184   QIQKPPSFEERLQAALQRQSCARAFALETSLGLNMAWVGTATIPSVSVCCA-----DGRTTGGQTIAQEADPINHRVASN  258
BAF35853.1   183   KIQKLPSFEERLQTALLRQACARASALETSLGLNMAWVAKAIIPSISVCCA-----DGRTTGGQTIAQEADPINHRVASN  257
CAA57413.1   184   KIQKPPSFEERLQAALQRQSCARAFALETSLGPNMAWVGMAAIPSISVCCA-----DGRTTGGQTIAQEADPINHRVASN  258
BAF35852.2   184   PIQRPSSFEERLQAALQRQSCAKAFALETSLGFNMALVGKTTIPSTSVCCA-----GGRTTGGQTIAQEADPINHRVASN  258
Q91HK5.1     209   PIQNPPFFQERVREFCRMKSCARAFALETSLGLNRAWVGLVDIPSTSVCCA-----DGRTTGGQTIAQEADPLQHRVSTS  283
BAF35851.2   219   ---KPLTFREKLAAACSRQVSGRIFSFQTSLGLNMAQTCRVAIPSISICCASSRGTDGRTTGGQTVAQEADPLYHRVASN  295


YP_053924.1  285   TAPGRAQWISERRSALRRREQANSLQSLAAQTD--MTFEQARNAYLGAADMVEQGLPLLPPLRNAYAPRGLWRGPSTRAN  362
DAZ85714.1   285   TAPGRAAWISERRSALRRREQANSLQGLAAQTD--MTFEQARNAYLGAADMVEQGLPLLPPLRNAYAPRGLWRGPSTRAN  362
QBZ78359.1   285   TAPGRAAWISERRSALRRREQAHSLQGLAAQTD--MTFEQARNAYLGAADMIEQGLPLLPPLRNAYAPRGLWRGPSTRAN  362
QBZ78357.1   285   TAPGRAAWISERRSALRRREQANSLQGLAAQTD--MTFEQARNAYLGAADMIEQGLPLLPPLRNAYAPRSLWRGPSTRAN  362
QBZ78355.1   285   TAPGRAAWISERRSALRRREQANSLQGLAAQTD--MTFEQARNAYLGAADMIEQGLPLLPPLRNAYAPRSLWRGPSTRAN  362
ABR09918.1   285   TAPGRAAWISERRSALRRREQANSLQGLAAQTD--MTFEQARNAYLGAADMIEQGLPLLPPLRNAYAPRGLWRGPSTRAN  362
QGY99256.1   285   TAPGRAAWISERRSALRRREQANSLQGLAAQTD--MTFEQARNAYLGAADMIEQGLPLLPPLRNAYAPRGLWRGPSTRAN  362
QYA72351.1   285   TAPGRAQWISERRSALRRREQANSLQSLAAQTD--MTFEQARNAYLGAADMIEQGLPLLPPLKNAYAPRGLWRGPSTRAN  362
AEK20744.1   285   TAPGRATWISERRNALRRREQANSLQGLAAQTD--MTFEQARNAYLGAADMIEQGLPLLPPLRNAYAPRGLWRGPSTRAN  362
DAZ85717.1   285   TAPGRAQWISERRSALRRREQANSLQGLAAQTD--MTFEQARNAYLGAADMIEQGLPLLPPLRNAYAPRGLWRGPSTRAN  362
ADJ39333.1   293   TAPGRAQWISERRSALRRKEQANSLQGLAAQTD--MTFEQARNAYLGAADMIEQGLPLLPPLRNAYAPRGLWRGPSTRAN  370
DAZ85715.1   293   TAPGRAQWISERRSALRRREQANSLQGLAAQTD--MTFEQARNAYLGAADMIEQGLPLLPPLRNAYAPRGLWRGPSTRAN  370
DAZ85721.1   293   TAPGRAQWISERRSALRRREQANSLQGLAAQTD--MTFEQARNAYLGAADMIEQGLPLLPPLRNAYAPRGLWRGPSTRAN  370
ACF32435.1   293   TAPGRAQWISERRSALRRREQANSLQGLAAQTD--MTFEQARNAYLGAADMIEQGLPLLPPLRNAYAPRGLWRGPSTRAN  370
ADJ39332.1   293   TAPGRAQWISERRSALRRREQANSLQGLAAQTD--MTFEQARNAYLGAADMIEQGLPLLPPLRNAYAPRGLWRGPSTRAN  370
QYU59217.1   293   TAPGRAAWISERRSALRRREQANSLQGLAAQTD--MTFEQARNAYLGAADMIEQGLPLLPPLRNAYAPRGLWRGPSTRAN  370
DAZ85724.1   293   TAPGRAQWISERRSALRRREQANSLQGLAAQTD--MTFEQARNAYLGAADMIEQGLPLLPPLRNAYAPRGLWRGPSTRAN  370
DAZ85722.1   293   TAPGRAQWISERRSALRRREQANSLQGLAAQTD--MTFEQARNAYLGAADMIEQGLPLLPPLRNAYAPRGLWRGPSTRAN  370
Q65030.1     306   TAPGRAQWISERRSALRRREQANSLQGLAAQTD--MTFEQARNAYLGAADMIEQGLPLLPPLRNAYAPRGLWRGPSTRAN  383
DAZ85716.1   259   TAPGRAQWISERRSALRRREQANSLQGLAAQTD--MTFEQARNAYLGAADMIEQGLPLLPPLRNAYAPRGLWRGPSTRAN  336
BAF35853.1   258   TAPGRAQWISERRSALRRREQANSLQGLAAQTD--MTFEQARNAYLGAADMIEQGLPLLPSLRNAYAPRGLWRGPSTRAN  335
CAA57413.1   259   TAPGRAQWISERRSALRRREQANSLQGLAAQTD--MTFEQARNAYLGAADMIEQGLPLLPPLRNAYAPRGLWRGPSTRAN  336
BAF35852.2   259   TAPGRAQWISERRSALRRREQANSLQGLAAQTD--MTFEQARNAYLGAADMIEQGLPLLPPLRNAYAPRGLWRGPSTRAN  336
Q91HK5.1     284   VAPGRAQWISERRQALRRREQANSLQGLAAQTD--MTFEQARNAYLGAADMIEQGLPLLPPLRSAYAPRGLWRGPSTRAN  361
BAF35851.2   296   TAPARQAWISERRQAIRRREQANSLQSLAAQTDQPVTFEQARNAYLGAAEMIDQGLPLLPPLRNAYAPRGLWRGPSTRAN  375


YP_053924.1  363   YTLDFRLNGIPTGQNTLEILYNPVADEEMDEYRDRGMSAVVIDALEIAINPFGMPGNPTDLTVVATYGHERNMERAFIGS  442
DAZ85714.1   363   YTLDFRLNGIPTGQNTLEILYNPVADEEMDDYRDRGMSAVVIDALEIAINPFGMPGNPTDLTVVATYGHERNMERAFIGS  442
QBZ78359.1   363   YTLDFRLNGIPTGQNTLEILYNPVADEEMDDYRDRGMSAVVIDALEIAINPFGMPGNPTDLTVVATYGHERNMERAFIGS  442
QBZ78357.1   363   FTLDFRLNGIPTGQNTLEILYNPVADEEMDDYRDRGMSAVVIDALEIAINPFGMPGNPTDLTVVATYGHERNMERAFIGS  442
QBZ78355.1   363   FTLDFRLNGIPTGQNTLEILYNPVADEEMDDYRDRGMSAVVIDALEIAINPFGMPGNPTDLTVVATYGHERNMERAFIGS  442
ABR09918.1   363   FTLDFRLNGIPTGQNTLEILYNPVADEEMDDYRDRGMSAVVIDALEIAINPFGMPGNPTDLTVVATYGHERNMERAFIGS  442
QGY99256.1   363   FTLDFRLNGIPTGQNTLEILYNPVADEEMDDYRDRGMSAVVIDALEIAINPFGMPGNPTDLTVVATYGHERNMERAFIGS  442
QYA72351.1   363   YTLDFRLNGIPTGQNTLEILYNPVADEEMDDYRDRGMSAVVIDALEIAINPFGMPGNPTDLTVVATYGHERNMERAFIGS  442
AEK20744.1   363   FTLDFRLNGIPTGQNTLEILYNPVADEEMEEYRDRGMSAVVIDALEIAINPFGMPGNPTDLTVVATYGHERNMERAFIGS  442
DAZ85717.1   363   YTLDFRLNGIPTGQNTLEILYNPVADEEMDDYRDRGMSAVVIDALEIAINPFGMPGNPTDLTVVATYGHERNMERAFIGS  442
ADJ39333.1   371   YTLDFRLNGIPTGQNTLEILYNPVADEEMDDYRDRGMSAVVIDALEIAINPFGMPGNPTDLTVVATYGHERNMERAFIGS  450
DAZ85715.1   371   FTLDFRLNGIPTGQNTLEILYNPVADEEMDDYRDRGMSAVVIDALEIAINPFGMPGNPTDLTVVATYGHERNMERAFIGS  450
DAZ85721.1   371   YTLDFRLNGIPTGQNTLEILYNPVADEEMDDYRDRGMSAVVIDALEIAINPFGMPGNPTDLTVVATYGHERNMERAFIGS  450
ACF32435.1   371   YTLDFRLNGIPTGQNTLEILYNPVSDEEMDDYRDRGMSAVVIDALEIAINPFGMPGNPTDLTVVATYGHERNMERAFIGS  450
ADJ39332.1   371   YTLDFRLNGIPTGQNTLEILYNPVADEEMDDYRDRGMSAVVIDALEIAINPFGMPGNPTDLTVVATYGHERNMERAFIGS  450
QYU59217.1   371   YTLDFRLNGIPTGQNTLEILYNPVADEEMDDYRDRGMSAVVIDALEIAINPFGMPGNPTDLTVVATYGHERNMERAFIGS  450
DAZ85724.1   371   YTLDFRLNGIPTGQNTLEILYNPVADEEMDDYRDRGMSAVVIDALEIAINPFGMPGNPTDLTVVATYGHERNMERAFIGS  450
DAZ85722.1   371   YTLDFRLNGIPTGQNTLEILYNPVADEEMDDYRDRGMSAVVIDALEIAINPFGMPGNPTDLTVVATYGHERNMERAFIGS  450
Q65030.1     384   YTLDFRLNGIPTGENTLEILYNPVSDEEMEDYRDRGMSAVVIDALEIAINPFGMPGNPTDLTVVATYGHERNMERAFIGS  463
DAZ85716.1   337   YTLDFRLNGIPTGQNTLEILYNPVADEEMDDYRDRGMSAVVIDALEIAINPFGMPGNPTDLTVVATYGHERNMERAFIGS  416
BAF35853.1   336   YTLDFRLNGIPTGQNTLEILYNPVSDEEMDDYRDRGMSAVVIDALEIAINPFGMPGNPTDLTVVTTYGHERNMERAFIGS  415
CAA57413.1   337   YTLDFRLNGIPTGQNTLEILYNPVADEEMDDYRDRGMSAVVIDALEIAINPFGMPGNPTDLTVVATYGHERNMERAFIGS  416
BAF35852.2   337   YTLDFRLNGIPTGQNTLEILYNPVADEEMDDYRDRGMSAVVIDALEIAINPFGMPGNPTDLTVVATYGHERNMERAFIGS  416
Q91HK5.1     362   YTLDFRLNGIPTGTNTLEILYNPVSEEEMEEYRDRGMSAVVIDALEIAINPFGMPGNPTDLTVVATYGHERDMTRAFIGS  441
BAF35851.2   376   YTLDFRLSGIPTGENTLEILYNPVADEEMDEYRDRGMSAVVIDALEIAINPFGMPGNPTDLTVVATYGHERNVERAFIGS  455
```

```
YP_053924.1  443   SSTFLGNGLARAIFFPGLQYSQEEPRRESIIRLYVASTNATVDADSILAAISVGTLRQHIGSLHNRTVASSVHAAQVQGT  522
DAZ85714.1   443   SSTFLGNGLARAIFFPGLQYSQEEPRRESIIRLYVASTNATVDADSILAAISVGTLRQHIGSLHNRTVASSVHAAQVQGT  522
QBZ78359.1   443   SSTFLGNGLARAIFFPGLQYSQEEPRRESLIRLYVASTNATVDTDSILAAISVGTLRQHIGSLHNRTVASSVHAAQVQGT  522
QBZ78357.1   443   SSTFLGNGLARAIFFPGLQYSQEEPRRESLIRLYVASTNATVDADSILAAISVGTLRQHIGSLHNRTVASSVHAAQVQGT  522
QBZ78355.1   443   SSTFLGNGLARAIFFPGLQYSQEEPRRESLIRLYVASTNATVDADSILAAISVGTLRQHIGSLHNRTVASSVHAAQVQGT  522
ABR09918.1   443   SSTFLGNGLARAIFFPGLQYSQEEPRRESLIRLYVASTNATVDADSILAAISVGTLRQHIGSLHNRTVASTVYAAQVQGT  522
QGY99256.1   443   SSTFLGNGLARAIFFPGLQYSQEEPRRESIIRLYVASTNATVDADSILAAISVGTLRQHIGSLHNRTVASSVHAAQVQGT  522
QYA72351.1   443   SSTFLGNGLARAIFFPGLQYSQEEPRRESLIRLYVASTNATVDADSILAAISVGTLRQHIGSLHNRTVASSVHAAQVRGT  522
AEK20744.1   443   SSTFLGNGLARAIFFPGLQYSQEEPRRNSIIRLYVASTNATVDTDSILAAISVGTLRQHIGSLHNRTVASSVHAAQVQGT  522
DAZ85717.1   443   SSTFLGNGLARAIFFPGLQYSQEEPRRESLIRLYVASTNATVDADSILAAISVGTLRQHIGSLHNRTVASSVHAAQVQGT  522
ADJ39333.1   451   SSTFLGNGLARAIFFPGLQYSQEEPRRESLIRLYVASTNATVDADSILAAISVGTLRQHIGSLHNRTVASSVHAAQVQGT  530
DAZ85715.1   451   SSTFLGNGLARAIFFPGLQYSQEEPRRDSLIRLYVASTNATVDADSILAAISVGTLRQHIGSLHNRTVASSVHAAQVQGT  530
DAZ85721.1   451   SSTFLGNGLARAIFFPGLQYSQEEPRRESLIRLYVASTNATVDADSILAAISVGTLRQHIGSLHNRTVASSVHTAQVQGT  530
ACF32435.1   451   SSTFLGNGLARAIFFPGLQYSQEEPRRESIIRLYVASTNATVDADSILAAISVGTLRQHIGSLHNRTVASSVHAAQVQGT  530
ADJ39332.1   451   SSTFLGNGLARAIFFPGLQYSQEEPRRESLIRLYVASTNATVDADSILAAISVGTLRQHIGSLHNRTVASSVHAAQVQGT  530
QYU59217.1   451   ASTFLGNGLARAIFFPGLQYSQEEPRRESLIRLYVASTNATVDADSILAAISVGTLRQHIGSLHNRTVASSVHAAQVQGT  530
DAZ85724.1   451   SSTFLGNGLARAIFFPGLQYSQEEPRRESLIRLYVASTNATVDADSILAAISVGTLRQHIGSLHNRTVASSVHAAQVQGT  530
DAZ85722.1   451   SSTFLGNGLARAIFFPGLQYSQEEPRRESLIRLYVASTNATVDADSILAAISVGTLRQHIGSLHNRTVASSVHAAQVQGT  530
Q65030.1     464   SSTFLGNGLARAIFFPGLQYSQEEPRRESLIRLYVASTNATVDADSVLAAISVGTLRQHIGSLHSRTVASSVHAAQVQGT  543
DAZ85716.1   417   SSTFLGNGLARAIFFPGLQYSQEEPRRESLIRLYVASTNATVDADSILAAISVGTLRQHIGSLHNRTVASSVHAAQVQGT  496
BAF35853.1   416   SSTFLGNGLARAIFFPGLQYSQEEPRRESLIRLYVASTNATVDADSILAAISVGTLRQHIGSLHNRTVASSVHAAQVQGT  495
CAA57413.1   417   SSTFLGNGLARAIFFPGLQYSQEEPRRESLIRLYVASTNATVDADSILAAISVGTLRQHIGSLHNRTVASSVHRAQWQGT  496
BAF35852.2   417   SSTFLGNGLARAIFFPGLQYSQEEPRRESLIRLYVASTNATVDADSILAAISVGTLRQHIGSLHNRTVASSVHAAQVQGT  496
Q91HK5.1     442   ASTFLGNGLARAIFFPGLQYSQEEPRRESIIRLYVASTNATVDTDSVLAAISVGTLRQHVGSMHYRTVASTVHQAQVQGT  521
BAF35851.2   456   ASTFLGNGLSRAIFFPGLQYSQEEPRRESIIRLYVASTNATVDADSVLAAISVGTLRQHIGSLHNRTVANSVHNAQVRGT  535

                                                                                      MP-CP cleavage site

YP_053924.1  523   TLRATMMGNAVVVSPEGSLVTGTPEANVQIGSGSSMRMVGPLAWENVEEPGQTFTIRNRSRSMRVDRNADVG--VALPRM  600
DAZ85714.1   523   TLRATMMGNAVVVSPEGSLVTGTPEANVQIGGGSSMRMVGPLAWENVDEPGQTFTIRNRSRSMRVDRNADVG--VALPRM  600
QBZ78359.1   523   TLRATMMGNAVVVSPEGSLVAGTPEANVQIGGGSSMRMVGPLAWENVEEPGQTFTIRNRSRSMRVDRNADVG--VALPRM  600
QBZ78357.1   523   TLRATMMGNAVVVSPEGSLVTGTPEANVQIGGGSSMRMVGPLAWENVEEPGHTFTIRNRSRSMRVDRNADVG--IALPRM  600
QBZ78355.1   523   TLRATMMGNAVVVSPEGSLVTGTPEANVQIGGGSSMRMVGPLAWENVEEPGHTFTIRNRSRSMRVDRNADVG--IALPRM  600
ABR09918.1   523   TLRATMMGNAVVVSPEGSLVVGTPEANVQIGGGSSMRMVGPLAWENVEEPGQTFTIRNRSRSMRVDRNADVG--VALPRM  600
QGY99256.1   523   TLRATMMGNAVVVSPEGSLVTGTPEANVQIGGGSSMRMVGPLAWENVEEPGQTFTIRNRSRSMRVDRNADVG--VALPRM  600
QYA72351.1   523   TLRATMMGNAVVVSPEGSLVTGTPEANVQIGGGSSMRMVGPLAWENVEEPGQTFTIRNRSRSMRVDRNADVG--IALPRM  600
AEK20744.1   523   TLRATMMGNSVVVSPEGSLVAGTPEANVQIGGGSSMRMVGPLAWENVEEPGQTFTIRSRSRSMRVDRNADVG--VALPRM  600
DAZ85717.1   523   TLRATMMGNAVVVSPEGSLVTGTPEANVQIGGGSSMRVVGPLAWENVEEPGQTFTIRNRSRSMRVDRNADVG--IALPRM  600
ADJ39333.1   531   TLRATMMGNAVVVSPEGSLVTGTPEANVQIGGGSSMRMVGPLAWENVEEPGQTFTIRNRSRSMRVDRNADVG--IALPRM  608
DAZ85715.1   531   TLRATMMGNAVVVSPEGSLVTGTPEANVQIGGGSSMRMVGPLAWENVEEPGQTFTIRNRSRSMRVDRNADVG--VALPRM  608
DAZ85721.1   531   TLRATMMGNAVVVSPEGSLVTGTPEANVQIGGGSSMRMVGPLAWENVEEPGQTFTIRNRSRSVRVDRNADVG--VALPRM  608
ACF32435.1   531   TLRATMMGNAVVVSPEGSLVTGTPEANVQIGGVSSMRMVGPLAWENVDEPGQTFTIRNRSRPMRVNRNADVG--VALPRM  608
ADJ39332.1   531   TLRATMMGNAVVVSPEGSLVTGTPEANVQIGGGSSMRMVGPLAWENVEEPGQTFTIRNRSRSMRVDRNADVG--VALPRM  608
QYU59217.1   531   TLRATMMGNAVVVSPEGSLVTGTPEANVQIGGGSSMRMVGPLSWENVEEPGQTFTIRNRSRSMRVDRNADVG--VALPRM  608
DAZ85724.1   531   TLRATMMGNAVVVSPEGSLVTGTPEANVQIGGGSSMRMVGPLAWENVAEPGQTFTIRNRSRSMRVDRNADVG--NALPRM  608
DAZ85722.1   531   TLRATMMGNAVVVSPEGSLVTGTPEANVQIGGGSSMRMVGPLAWENVAEPGQTFTIRNRSRSMRVDRNADVG--NALPRM  608
Q65030.1     544   TLRATMMGNSVVVSPEGGLVSGVPEANVQIGGGSSMRMVGPLAWENVEEPGQTFSIRSRSRSVRVDRNADVG--IAHPRM  621
DAZ85716.1   497   TLRATMMGNAVVVSPEGSLVTGTPEANVQIGGGSSMRMVGPLVWENVEEPGQTFTIRNRSRSMRVDRNADVG--IALPRM  574
BAF35853.1   496   TLRATMMGNAVVVSPEGSLVTGTPEANVQMGGGSSMRMVGPLAWENVEEPGQTFTIRNRSRSMRVDRNADVG--VALPRM  573
CAA57413.1   497   TLRATMMGNAVVVSPEGSLVAGTPEANVQIGGGSSMRMVGPLAWENVEEPGQTFTIRNRSRSMRVDRNADVG--VALPRM  574
BAF35852.2   497   TLRATMMGNAVVVSPEGSLVTGTPEANVQIGGGSSMRMVGPLAWENVEEPGQTFTIRNRSRSMRVDRNADVG--VALPRM  574
Q91HK5.1     522   TLRATMMGNTVVVSPEGSLVTGTPEARVEIGGGSSIRMVGPLQWESVEEPGQTFSIRSRSRSVRIDRNVDLPQLEAEPRL  601
BAF35851.2   536   TLRAAMMGNTIVVSPEGGLVTGAPNANVTIGGSSSVRMVGPLAWENVQEPGRQFTIQSRSRSVRVDRNADLP--EAQPRT  613

               MP-CP cleavage site

YP_053924.1  601   STTTRGLAGRGSVQVPKDCQAGRYLKTLDLRDMVSGFSGIQYEKWITAGLVMPDFKVVIRYPANAFTGITWVMSFDAYNR  680
DAZ85714.1   601   STTTRGLAGRGSVQVPKDCQAGRYLKTLDLRDMVSGFSGIQYEKWITAGLVMPNFKVVIRYPANAFTGITWVMSFDAYNR  680
QBZ78359.1   601   STTTRGFAGRGSVQVPKDCQAGRYLKTLDLRDMVSGFSGIQYEKWITAGLVMPNFKVVVRYPANAFTGITWVMSFDAYNR  680
QBZ78357.1   601   STTTRGLAGRGSVQVPKDCQAGRYLKTLDLRDMISGFSGIQYEKWITAGLVMPNFKVVIRYPANAFTGITWVMSFDAYNR  680
QBZ78355.1   601   STTTRGLAGRGSVQVPKDCQAGRYLKTLDLRDMISGFSGIQYEKWITAGLVMPNFKVVIRYPANAFTGITWVMSFDAYNR  680
ABR09918.1   601   STTTRGLAGRGSVQVPKDCQAGKYLKTLDLRDMVSGFSGIQYEKWITAGLVMPEFKVVVRYPANAFTGITWVMSFDAYDR  680
QGY99256.1   601   STTTRGLAGRGSVQVPKDCQAGRYLKTLDLRDMISGFSGIQYEKWITAGLVMPNFKVVVRYPANAFTGITWVMSFDAYNR  680
QYA72351.1   601   STTTRGLAGRGSVQVPKDCQAGIYLKTLDLRDMVSGFSGIQYEKWITAGIVMPNFKVVIRYPANAFTGITWVMSFDAYNR  680
AEK20744.1   601   STTTRGLAGRGSVQVPKDCQAGRYLKTLDLRDMVSEFSGIQYEKWITAGLIMPEFKVVIRYPANAFTGITWVMSFDAYNR  680
DAZ85717.1   601   STTTRGLAGRGSVQVPKDCQAGKYLKTLDLRNMVSGFAGIQYEKWITAGIVMPDFKVVIRYPANAFTGITWVMSFDAYNR  680
ADJ39333.1   609   STTTRGLAGRGSVQVPKDCQAGKYLKTLDLRDMVSGFSGIQYEKWITAGLVMPDFKVVVRYPANAFTGITWVMSFDAYNR  688
DAZ85715.1   609   STTTRGLAGRGSVRVPKDCQAGRYLKTLDLRDMVSGFSGIQYEKWITAGLVMPEFKVVVRYPANAFTGITWVMSFDAYNR  688
DAZ85721.1   609   STTTRGLAGRGSVQVPKDCQAGKYLKTLDLRDMVSGFSGIQYEKWITAGIVMPDFKVVIRYPANAFTGITWVMSFDAYNR  688
ACF32435.1   609   STTTRGLAGRGSVQVPKDCQAGKYLKTLDLRDMVSGFSGIQYEKWITAGLVMPDFKVVIRYPANAFTGITWVMSFDAYNR  688
ADJ39332.1   609   STTTRGLAGRGSVQVPKDCQAGKYLKTLDLRDMVSGFSGIQYEKWITAGLVMPDFKVVIRYPANAFTGITWVMSFDAYNR  688
QYU59217.1   609   STTTRGLAGRGSVQVPKDCQAGRYLKTLDLRDMVSGFSGIQYEKWITAGLVMPEFKVVIRYPANAFTGITWVMSFDAYNR  688
DAZ85724.1   609   STTTRGLAGRGSVQVPKDCQAGKYLKTLDLRDMVSGFSGIQYEKWITAGIVMPDFKVVIRYPANAFTGITWVMSFDAYNR  688
DAZ85722.1   609   STTTRGLAGRGSVQVPKDCQAGKYLKTLDLRDMVSGFSGIQYEKWITAGIVMPDFKVVIRYPANAFTGITWVMSFDAYNR  688
Q65030.1     622   STTTRGLAGRGTVPVPKDCQAGKYLKTLDLRDMVSGFSGIQYEKWITAGLVMPDFKVVIRYPANAFTGITWVMSFDAYNR  701
DAZ85716.1   575   STTTRGLAGRGSVQVPKDCQAGKYLKTLDLRDMVSGFSGIQYEKWITAGIVMPDFKVVIRYPANAFTGITWVMSFDAYNR  654
BAF35853.1   574   STTTRGLAGRGSVQVPKDCQAGKYLKTLDLRDMISGFSGIQYEKWITAGLIMPDFKVVIRYPANAFTGITWVMSFDAYNR  653
CAA57413.1   575   STTTRGLAGRDSVQVPKDCQAGKYLKTLDLREMVSGFSGIQYEKWITAGIVMPDFKVVIRYPANAFTGITWVMSFDAYNR  654
BAF35852.2   575   STTTRGLAGRGSVQVPKDCQAGKYLKTLDLRDMISGFSGIQYEKWITAGLIMPDFKVVIRYPANAFTGITWVMSFDAYNR  654
Q91HK5.1     602   SSTVRGLAGRGVVYIPKDCQANRYLGTLNIRDMISDFKGVQYEKWITAGLVMPTFKIVIRLPANAFTGLTWVMSFDAYNR  681
BAF35851.2   614   STTTRGLAGRGSVQVPKDCKAGAFLKTLNLRDMISGFSGIQYEKWIAAGLVMPDFKVVIRYPANAFTGLTWVMSFDAYNR  693
```

```
YP_053924.1  681   ITSSITTTASPAYTLSVPHWLLHHKNGTTSCDIDYGELCGHAMWFNATTFESPKLHFTCLTGNNKELAADWEFVVELYAE  760
DAZ85714.1   681   ITSSISTTASPAYTLSVPHWLLHHKNGTTSCDIDYGELCGHAMWFNATTFESPKLHFTCLTGNNKELAADWEFVVELYAE  760
QBZ78359.1   681   ITSSISTTASPTYTLSVPHWLLHHKNGTTSCDIDYGELCGHAMWFSATTFESPKLHFTCLTGNNKELAADWEFVVELYAE  760
QBZ78357.1   681   ITSSISTTASPAYTLSVPHWLLHHKNGTTSCDVDYGELCGHAMWFSATTFESPKLHFTCLTGNNKELAADWEFVVELYAE  760
QBZ78355.1   681   ITSSISTTASPAYTLSVPHWLLHHKNGTTSCDVDYGELCGHAMWFSATTFESPKLHFTCLTGNNKELAADWEFVVELYAE  760
ABR09918.1   681   ITSSISTVASPTYTLSVPHWLLHHKNGTTSCEIDYGTLCGHAMWFNATTFESPKLHFTCLTGNNKELAADWEFVVELYAE  760
QGY99256.1   681   ITSSISTTASPTYTLSVPHWLLHHKKGTTSCDVDYGELCGHAMWFSATTFESPKLHFTCLTGNNKELAADWEFVVELYAE  760
QYA72351.1   681   ITSSISTTASPAYTLSVPHWLLHHKNGTTSCDLDYGELCGHAMWFGATTFESPKLHFTCLTGNNKELAADWEFVVELYAE  760
AEK20744.1   681   ITSSISTTANPVYTLSVPHWLLHHKNGTTSCDIDYGELCGHAMWFSATTFESPKLHFTCLTGNNKELAADWEFVVELYAE  760
DAZ85717.1   681   ITSSISTTASPAYTLSVPHWLLHHKNGTTSCDLDYGELCGHAMWFGATTFESPKLHFTCLTGNNKELAADWEFVVELYAE  760
ADJ39333.1   689   ITSSISTTASPAYTLSVPHWLLHHKNGTTSCDIDYGELCGHAMWFNATTFESPKLHFTCLTGNNKELAADWEFVVELYAE  768
DAZ85715.1   689   ITSSISTTASPTYTLSVPHWLLHHKNGTTSCDIDYGELCGHAMWFSATTFESPKLHFTCLTGNNKELAADWEFVVELYAE  768
DAZ85721.1   689   ITSSISTTASPAYTLSVPHWLLHHKNGTTSCDLDYGELCGHAMWFGATTFESPKLHFTCLTGNNKELAADWEFVVELYAE  768
ACF32435.1   689   ITSSISTTASPAYTLSVPHWLLHHKNGTTSCDIDYGELCGHAMWFSATTFESPKLHFTCLTGNNKELAADWEFVVELYAE  768
ADJ39332.1   689   ITSSISTTASPAYTLSVPHWLLHHKNGTTSCDIDYGELCGHAMWFNATTFESPKLHFTCLTGNNKELAADWEFVVELYAE  768
QYU59217.1   689   ITSSISTTANPVYTLSVPHWLLHHKNGTTSCDIDYGALCGHAMWFSATTFESPKLHFTCLTGNNKELAADWEFVVELYAE  768
DAZ85724.1   689   ITSSISTTASPAYTLSVPHWLLHHKNGTTSCDLDYGELCGHAMWFGATTFESPKLHFTCLTGNNKELAADWEFVVELYAE  768
DAZ85722.1   689   ITSSISTTASPAYTLSVPHWLLHHKNGTTSCDLDYGELCGHAMWFGATTFESPKLHFTCLTGNNKELAADWEFVVELYAE  768
Q65030.1     702   ITSSISTTASPAYTLSVPHWLLHHKNGTTSCDIDYGELCGHAMWFSATTFESPKLHFTCLTGNNKELAADWEFVVELYAE  781
DAZ85716.1   655   ITSSISTTASPAYTLSVPHWLLHHKNGTTSCDLDYGELCGHAMWFGATTFESPKLHFTCLTGNNKELAADWEFVVELYAE  734
BAF35853.1   654   ITSSISTTASPAYTLSVPHWLLHHKNGTTSCDIDYGELCGHALWFSATTFESPKLHFTCLTGNNKELAADWEFVVELYAE  733
CAA57413.1   655   ITSSISTTASPAYTLSVPHWLLHHKNGTTSCDLDYGELCGHAMWFGATTFESPKLHFTCLTGNNKELAADWEFVVELYAE  734
BAF35852.2   655   ITSSISTTASPAYTLSVPHWLLHHKNGTTSCDIDYGELCGHALWFSATTFESPKLHFTCLTGNNKELAADWEFVVELYAE  734
Q91HK5.1     682   ITSRITTSADPVYTLSVPHWLIHHKLGTFSCEIDYGELCGHAMWFKSTTFESPKLHFTCLTGNNKELAADWQAVVELYAE  761
BAF35851.2   694   ITSSITTTASPTYTLSVPHWLLHHKNGTTTCEVDYGKLCGHAMWFSTTTFETPKLHFTCLTGNNKALAADWEFVVELYAE  773


YP_053924.1  761   FEAAKTFLGRPNFVYSADAFNGSFKFLTIPPLEYDLSTTSAYKSVSLLLGQTLIDGTHKVYNYNNTLLSYYLGIGGVVKG  840
DAZ85714.1   761   FEAAKTFLGRPNFVYSADAFNGSFKFLTIPPLEYDLSTTSAYKSVSLLLGQTLVDGTHKVYNYNNTLLSYYLGIGGVVKG  840
QBZ78359.1   761   FEAAKTFLGRPNFIYSADAFDGSFKFLTIPPLEYDLSTTSAYKSVSLLLGQTLVDGTHKVYNYNNTLLSYYLGIGGVVKG  840
QBZ78357.1   761   FEAAKTFLGRPNFIYSADAFNGSFKFLTIPPLEYDLSTTSAYKSVSLLLGQTLVDGTHKVYNYNNTLLSYYLGIGGVVKG  840
QBZ78355.1   761   FEAAKTFLGRPNFIYSADAFNGSFKFLTIPPLEYDLSTTSAYKSVSLLLGQTLVDGTHKVYNYNNTLLSYYLGIGGVVKG  840
ABR09918.1   761   FEAAKTFLGKPNFIYSADAFNGSFKFLTIPPLEYDLGTTSAYKSVSLLLGQTLVDGAHKVYNYNNTLLSFYLGIGGVVKG  840
QGY99256.1   761   FEAAKTFLGRPNFIYSADAFNGSFKFLTIPPLEYDLSTTSAYKSVSLLLGQTLVDGTHKVYNYNNTLLSYYLGIGGVVKG  840
QYA72351.1   761   FEAAKSFLGKPNFIYSADAFNGSLKFLTIPPLEYDLSATSAYKSVSLLLGQTLVDGTHKVYNFNNTLLSYYLGIGGIVKG  840
AEK20744.1   761   FEAAKTFLGRPNFVYSAGAFSGSFNFLTVPPLEYDLSTTSAYKSVSLLLGQTLTDGTHKVYNYNNTLLSFFLGIGGVVKG  840
DAZ85717.1   761   FEAAKSFLGKPNFIYNADAFNGSLKFLTIPPLEYDLSSTSAYKSVSLLLGQTLVDGTHKVYNFNNTLLSYYLGIGGVVKG  840
ADJ39333.1   769   FEAAKTFLGRPNFIYSADAFNGSFKFLTIPPLEYDLSTTSAYKSVSLLLGQTLVDGTHKVYNYNNTLLSYYLGIGGVVKG  848
DAZ85715.1   769   FEAAKTFLGKPNFIYSADAFNGSFKFLTIPPLEYDLSTTSAYKSVSLLLGQTLVDGTHKVYNYNNTLLSFYLGIGGVVKG  848
DAZ85721.1   769   FEAAKSFLGKPNFIYNADAFNGSLKFLTIPPLEYDLSSTSAYKSVSLLLGQTLVDGTHKVYNFNNTLLSYYLGIGGVVKG  848
ACF32435.1   769   FEAAKTFLGRPNFIYSADAFNGSFKFLTIPPLEYDLSTTSAYKSVSLLLGQTLVDGTHKVYNYNNTLLSYYLGIGGVVKG  848
ADJ39332.1   769   FEAAKTFLGRPNFIYSADAFNGSFKFLTIPPLEYDLSTTSAYKSVSLLLGQTLVDGTHKVYNYNNTLLSYYLGIGGVVKG  848
QYU59217.1   769   FEAAKTFLGRPNFVYSADAFNGSFKFLTIPPLEYDLSTTSAYKSVSLLLGQTLIDGTHKVYNYNNTLLSYYLGIGGVVKG  848
DAZ85724.1   769   FEAAKSFLGKPNFIYNADAFNGSLKFLTIPPLEYDLSSTSAYKSVSLLLGQTLVDGTHKVYNFNNTLLSYYLGIGGVVKG  848
DAZ85722.1   769   FEAAKSFLGKPNFIYNADAFNGSLKFLTIPPQGDDLRSTRAHKRVSLLLGQTLVDGTHKEYNFNNTLLSYYLGIGGVVKG  848
Q65030.1     782   FEAAKSFLGKPNFIYSADAFNGSLKFLTIPPLEYDLSATSAYKSVSLLLGQTLVDGTHKVYNFNNTLLSYYLGIGGIVKG  861
DAZ85716.1   735   FEAAKSFLGKPNFIYNADAFNGSLKFLTIPPLEYDLSATSAYKSVSLLLGQTLVDGTHKVYNFNNTLLSYYLGIGGVVKG  814
BAF35853.1   734   FEAAKTFLGRPNFIYSAEAFNGSFKFLTIPPLEYDLSTTSAYKSVSLLLGQTLVDGIHKVYNYNNTLLSYYLGIGGVVKG  813
CAA57413.1   735   FEAAKSFLGKPNFIYSADAFNGSLKFLTIPPLEYDLSATSAYKSVSLLLGQTLVDGTHKVYNFNNTLLSYYLGIGGIVKG  814
BAF35852.2   735   FEAAKTFLGRPNFIYSADAFNGSFKFLTIPPLEYDLSTTSAYKSVSLLLGQTLVDGTHKVYNYNNTLLSYYLGVGGVVKG  814
Q91HK5.1     762   LEEASSFLGKPTLVFDPGVFNGKFQFLTCPPIFFDLTAVTALKSAGLTLGQVPMVGTTKVYNLNSALVSCVLGMGGTIRG  841
BAF35851.2   774   FEAADSFLGKPNFVHRNGAFEGSFEYFTLPPLNYALNTIAAYKSIPLNLGQTLSDGTSKVYNFNNTLLSYYLGLGGKLRG  853


YP_053924.1  841   RVHICSPCTYGIVLRVVSEWNGVTNNWNQLFKYPGCYIGEDGNFEIEIRSPYHRTPLRLLDAQAASAFTSTLNFYAISGP  920
DAZ85714.1   841   KVHICSPCTYGIVLRVVSEWNGVTNNWNQLFKYPGCYIDEDGNFEIEIRSPYHRTPLRLLSAQSASAFTSTLNFYAISGP  920
QBZ78359.1   841   KVHICSPCTYGIVLRVVSEWNGVTNNWNQLFKYPGCYIDEDGNFEIEIRSPYHRTPLRLLGGQAASSFTSTLNFYAISGP  920
QBZ78357.1   841   KVHICSPCTYGIVLRVVSEWNGVTNNWNQLFKYPGCYIDEDGNFEIEIRSPYHRTPLRLLNAQSASAFTSTLNFYAISGP  920
QBZ78355.1   841   KVHICSPCTYGIVLRVVSEWNGVTNNWNQLFKYPGCYIDEDGNFEIEIRSPYHRTPLRLLNAQSASAFTSTLNFYAISGP  920
ABR09918.1   841   RVHICSPCTYGIVLRVVSEWNGVTNNWNQLFKYPGCYVDENGNFEIEIRSPYHRTPLRLRDAQVASSFTSTLNFYAISGP  920
QGY99256.1   841   KVHICSPCTYGIVLRVVSEWNGVTNDWNQLFKYPGCYIDEDGNFEIEIRSPYHRTPLRLLSAQAASSFTSTLNFYAISGP  920
QYA72351.1   841   KVHVCSPCTYGIVLRVVSEWNGVTNNWNQLFKYPGCYIEEDGSFAIEIRSPYHRTPLRLIDAQSASSFTSTLNFYAISGP  920
AEK20744.1   841   RVHICSPCTYGIVLRVVSEWNGVTNNWNQLFKYPGCYVDEDGNFEIEIRSPYHRTPLRLLNAQAASSFTSSLNFYAISGP  920
DAZ85717.1   841   KVHICSPCTYGIVLRVVSEWNGVTNNWNQLFKYPGCYIEEDGSFAIEIRSPYHRTPLRLLDAQSASSFTSTLNFYAISGP  920
ADJ39333.1   849   KVHICSPCTYGIVLRVVSEWNGVTNNWNQLFKYPGCYIDEDGRFEIEIRSPYHRTPLRLLDGQAASSFTSTLNFYAISGP  928
DAZ85715.1   849   KVHICSPCTYGVVLRVVSEWNGATNNWNQLFKYPGCYIEEDGSFEIEIRSPYHRTPLRLMNAQAASSFVSTLNFYAISGP  928
DAZ85721.1   849   KVHICSPCTYGIILRVVSEWNGVTNNWNQLFKYPGCYIEEDGSFAIEIRSPYHRTPLRLLDAQSASSFTSTLNFYAISGP  928
ACF32435.1   849   KVHICSPCTYGIVLRVVSEWNGVTNNWNQLFKYPGCYIDEDGNFEIEIRSPYHRTPLRLLDAQAASSFTSTLNFYAISGP  928
ADJ39332.1   849   KVHICSPCTYGIVLRVVSEWNGVTNNWNQLFKYPGCYIDEDGRFEIEIRSPYHRTPLRLLDGQAASSFTGTLNFYAISGP  928
QYU59217.1   849   KVHICSPCTYGIVLRVVSEWNGVTNNWNQLFKYPGCYIDEDGNFEIEIRSPYHRTPLRLLSTQAVSAFTSTLNFYAISGP  928
DAZ85724.1   849   KVHICSPCTYGIILRVVSEWNGVTNNWNQLFKYPGCYIEEDGSFAIEIRSPYHRTPLRLLDAQSASSFTSTLNFYAISGP  928
DAZ85722.1   849   KVHICSPCTYGIILRVVSEWNGVTNNWNQLFKYPGCYIEEDGSFAIEIRSPYHRTPLRLLDAQSASSFTSTLNFYAISGP  928
Q65030.1     862   KVHVCSPCTYGIVLRVVSEWNGVTNNWNQLFKYPGCYIEEDGSFAIEIRSPYHRTPLRLIDAQSASSFTSTLNFYAISGP  941
DAZ85716.1   815   KVHVCSPCTYGIVLRVVSEWNGVTNNWNQLFKYPGCYIEEDGSFAIEIRSPYHRTPLRLIDAQSASSFTSTLNFYAISGP  894
BAF35853.1   814   RVHICSPCTYGIVLRVVSEWNGVTNNWNQLFKYPGCYIDEDGNFEIEIRSPYHRTPLRLLDGQAASSFTSTLNFYAISGP  893
CAA57413.1   815   KVHVCSPCTYGIVLRVVSEWNGVTNNWNQLFKYPGCYIEEDGSFAIEIRSPYHRTPLRLIDAQSASSFTSTLNFYAISGP  894
BAF35852.2   815   KVHICSPCTYGIVLRVVSEWNGVTNNWNQLFKYPGCYIDEDGNFEIEIRSPYHRTPLRLLDGQAASSFTSTLNFYAISGP  894
Q91HK5.1     842   RVHICAPIFYSIVLWVVSEWNGTTMDWNELFKYPGVYVEEDGSFEVKIRSPYHRTPARLLAGQSQRDM-SSLNFYAIAGP  920
BAF35851.2   854   RVLFCSPIFYGVVLRIVSEWNGVTNNWNQLFKYPGVNLEQEGPFEIEIRSPFHRTPMRLQGTQTASSM-SSINMYAVSGP  932
```

```
YP_053924.1  921   IAPSGETAKMPVVVQIDEIALPDLSVPSFPNDYFLWVDFSAFTVDAEEYVIGSRFFDISSTTSTVHLGDNPFAHMIACHG  1000
DAZ85714.1   921   IAPSGETARMPVVVQIDEIALPDLSVPSFPDDYFLWVDFSAFTVDAEEYVIGSRFFDISSTTSTVHLGDNPFAHMIACHG  1000
QBZ78359.1   921   IAPSGETAKMPVVVQIDEIALPDLSVPSFPNDYFLWVDFSAFTVDAEEYVIGSRFFDISSTTSTVHLGDNPFAHMIACHG  1000
QBZ78357.1   921   IAPSGETARMPVVVQIDEIALPDLSVPSFPDDYFLWVDFSAFTVDAEEYVIGSRFFDISSTTSTVHLGDNPFAHMIACHG  1000
QBZ78355.1   921   IAPSGETARMPVVVQIDEIALPDLSVPSFPDDYFLWVDFSAFTVDAEEYVIGSRFFDISSTTSTVHLGDNPFAHMIACHG  1000
ABR09918.1   921   IAPSGEIAKMPVVVQIDEIALPDLSVPSFPNDYFLWVDFSAFTVDVEEYVIGSRFFDITSTTSRVSLGDNPFAHMIACHG  1000
QGY99256.1   921   IAPSGETARMPVVVQINEIALPDLSVPSFPDDYFLWVDFSAFTVDTEEYVIGSRFFDITSTTSTVHLGDNPFAHMIACHG  1000
QYA72351.1   921   IAPSGETAKMPVVVQIEEIALPDLSVPSFPNDYFLWVDFSSFTVDVEEYVIGSRFFDISSTTSTVALGDNPFAHMIACHG  1000
AEK20744.1   921   IAPSGETARMSVVVQINEIALPDLSVPSFPDDYFLWVDFSAFTVDAEEYVIGSRFFDITSTTSTVHLGDNPFAHMIACHG  1000
DAZ85717.1   921   IAPSGETAKMPVVVQIEEIALPDLSVPSFPNDYFLWVDFSSFTVDVEEYVIGSRFFDISSTTSTVALGDNPFAHMIACHG  1000
ADJ39333.1   929   IAPSGETAKMPVVVQIEEVALPDLSVPTFPNDYFLWVDFSAFTVDTEEYVIGSRFFDISSTTSTVALGDNPFAHMIACHG  1008
DAZ85715.1   929   IAPSGEIAKMPVVVQIDEIALPDLSVPSFPDDYFLWVDFSAFTVDAEEYVIGSRFFDITSTTSTVHLGDNPFAHMIACHG  1008
DAZ85721.1   929   IAPSGETAKMPVVVQIEEIALPDLSVPSFPNDYFLWVDFSSFTVDVEEYVIGSRFFDISSTTSTVALGDNPFAHMIACHG  1008
ACF32435.1   929   IAPSGETAKMPVVVQINEIALPDLSVPSFPNDYFLWVDFSAFTVDTEEYVIGSRFFDISSTTSTVALGDNPFAHMIACHG  1008
ADJ39332.1   929   IAPSGETAKMPVVVQIEEIALRDLSVPTFPNDYFLWVDFSAFTVDTEEYVIGSRFFDISSTTSTVALGDNPFAYMIACHG  1008
QYU59217.1   929   IAPSGETAKMPVVVQINEIALPDLSVPSFPNDYFLWVDFSAFTVDAEEYVIGSRFFDITSATSTVHLGDNPFAHMIACHG  1008
DAZ85724.1   929   IAPSGETAKMPVVVQIEEIALPDLSVPSFPNDYFLWVDFSSFTVDVEEYVIGSRFFDISSTTSTVALGDNPFAHMIACHG  1008
DAZ85722.1   929   IAPSGETAKMPVVVQIEEIALPDLSVPSFPNDYFLWVDFSSFTVDVEEYVIGSRFFDISSTTSTVALGDNPFAHMIACHG  1008
Q65030.1     942   IAPSGETAKMPVVVQIEEIALPDLSVPSFPNDYFLWVDFSSFTVDVEEYVIGSRFFDISSTTSTVALGDNPFSHMIACHG  1021
DAZ85716.1   895   IAPSGETAKMPVVVQIEEIALPDLSVPSFPNDYFLWVDFSAFTVDAEEYVIGSRFFDISSTTSTVALGDNPFAHMIACHG  974
BAF35853.1   894   IAPSGETAKMPVVVQIDEIALPDLSVPTFPNDYFLWVDFSAFTVDVEEYVIGSRFFDISSTTSTVALGDNPFAHMIACHG  973
CAA57413.1   895   IAPSGETAKMPVVVQIEEIALPGLSVPSFPNDYFLWVDFSSFTVDVEEYVIGSRFFDISSTTSTVALGDNPFAHMIACHG  974
BAF35852.2   895   IAPSGETAKMPVVVQIDEIALPDLSVPTFPNDYFLWVDFSAFTVDTEEYVIGSRFFDISSTTSTVALGDNPFAHMIACHG  974
Q91HK5.1     921   IAPSGETARLPIVVQIDEIVRPDLALPSFEDDYFVWVDFSEFTLDKEELEIGSRFFDFTSSTCRVIMGENPFAAMIACHG  1000
BAF35851.2   933   IAPGGETADMAVVVQIDEIALPDLSAPAFPNDFFLWVDFSAFTIDQEEFVVGSRFFDFTSTTCTVALGTNPFAHMIACHG  1012

YP_053924.1  1001  LHHGILDLKLMWDLEGEFGKSSGGVTITKLCGDKATGMDGASRVCALQNMGCETELYIGNFAGANPNSALSLYSRWLAIK  1080
DAZ85714.1   1001  LHHGILDLKLMWDLEGEFGKSSGGVTITKLCGDKTTGMDGASRVCALQNMGCETELYIGNFAGANPNSALSLYSRWLAIK  1080
QBZ78359.1   1001  LHHGILDLKLMWDLEGEFGKSSGGVTITKLCGDKATGMDGASRVCALQTMGCETELYIGNFAGANPNTTLSLYSRWLAIK  1080
QBZ78357.1   1001  LHHGILDLKLMWDLEGEFGKSSGGVTITKLCGDKTTGMDGASRVCALQNMGCETELYIGNFAGANPNSALSLYSRWLAIK  1080
QBZ78355.1   1001  LHHGILDLKLMWDLEGEFGKSSGGVTITKLCGDKTTGMDGASRVCALQNMGCETELYIGNFAGANPNSALSLYSRWLAIK  1080
ABR09918.1   1001  LHHGILDLKLMWDLEGEFGKSSGGVTITKLCGDKATGMDGASRVCALQNMGCETELYVGNFAGANPNTALSLYSRWLAIK  1080
QGY99256.1   1001  LHHGILDLKLMWDLEGEFGKSSGGVTITKLCGDKTTGMDGASRVCALQNMGCETELYIGNFAGANPNTALSLYSRWLAIK  1080
QYA72351.1   1001  LHHGILDLKLMWDLEGEFGKSSGGVTITKLCGDKATGMDGASRVCALQNMGCETELYIGNYAGANPNTALSLYSRWLAIK  1080
AEK20744.1   1001  LHHGILDLKLMWDLEGEFGKSSGGVTITKLCGDKTTGMDGASRVCALQNMGCETELYIGNFAGANPNTALSLYSRWLAIK  1080
DAZ85717.1   1001  LHHGILDLKLMWDLEGEFGKSTGGVTITKLCGDKATGMDGASRVCALQNMGCETELYIGNYAGANPNTALSLYSRWLAIK  1080
ADJ39333.1   1009  LHHGILDLKLMWDLEGEFGKSSGGVTITKLCGDKATGMDGASRVCALQTMGCETELYIGNFAGANPNTALSLYSRWLAIK  1088
DAZ85715.1   1009  LHHGILDLKLMWDLEGEFGKSSGGVTITKLCGDKTTGMDGASRVCALQNMGCETELYIGNFAGANPNTALSLYSRWFAIK  1088
DAZ85721.1   1009  LHHGILDLKLMWDLEGEFGKSSGGVTITKLCGDKATGMDGASRVCALQSMGCETELYIGNYAGANPNTALSLYSRWLAIK  1088
ACF32435.1   1009  LHHGILDLKLMWDLEGEFGKSSGGVTITHLCGDKATGMDGASRVCALQNMGCETELYVGNFAGANPNTALNLYSRWLAIK  1088
ADJ39332.1   1009  LHHGILDLKLMWSLEGEFGKSSGGVTITRLCGDKATGMDGASRVCALQDMGCETELYVGNYAGANPNTALSLYSRWLAIK  1088
QYU59217.1   1009  LHHGVLDLKLMWDLEGEFGKSSGGVTITKLCGDKTTGMDGASRVCALQNMGCETELYIGNFAGANPNTALSLYSRWLAIK  1088
DAZ85724.1   1009  LHHGILDLKLMWDLEGEFGKSSGGVTITKLCGDKATGMDGASRVCALQSMGCETELYIGNYAGANPNTALSLYSRWLAIK  1088
DAZ85722.1   1009  LHHGILDLKLMWDLEGEFGKSSGGVTITKLCGDKATGMDGASRVCALQSMGCETELYIGNYAGANPNTALSLYSRWLAIK  1088
Q65030.1     1022  LHHGILDLKLMWDLEGEFGKSSGGVTITKLCGDKATGMDGASRVCALQNMGCETELYIGNYAGANPNTALSLYSRWLAIK  1101
DAZ85716.1   975   LHHGILDLKLMWDLEGEYGKSTGGVTITKLCGDKATGMDGASRVCALQNMGCETELYIGNYAGANPNTALSLYSRWLAIK  1054
BAF35853.1   974   LHHGILDLKLMWDLEGEFGKSSGGVTITRLCGDKATGMDGASRVCALQNMSCETELYVGNYAGANPNTALSLYSRWLAIK  1053
CAA57413.1   975   LHHGILDLKLMWDLEGEFGKSAGGVTITKLCGDKATGMDGASRVCALQNMGCETELYIGNYAGANPNTALSLYSRWLAIK  1054
BAF35852.2   975   LHCGVLDLKVMWDLEGEFGKSSGGVTITRLCGDKATGMDGASRVCALQDMSCETELYVGNYAGANPNTALSLYSRWLAIK  1054
Q91HK5.1     1001  LHSGVLDLKLQWSLNTDFGKSSGSVTVTKLVGDKATGLDGPSQVFAIQKLEGVTDLLIGNFAGANPNTHFSLYSRWMAIK  1080
BAF35851.2   1013  LHRGVLELKLEWSLVEEFGKSTGSVILTHLVGDKATGLDGGSRVCAIQDMGCLTELFVGNFAGPNPNTALDAYSRWLAIK  1092

YP_053924.1  1081  LDKARSMKMLRILCKPRGNFEFYGRTCFRV  1110
DAZ85714.1   1081  LDKARSMKMLRILCKPRGNFEFYGRTCFRV  1110
QBZ78359.1   1081  LDKARSMKMLRILCKPRGNFEFYGRTCFRV  1110
QBZ78357.1   1081  LDKARSMKMLRILCKPRGNFEFYGRTCFRV  1110
QBZ78355.1   1081  LDKARSMKMLRILCKPRGNFEFYGRTCFRV  1110
ABR09918.1   1081  LDKARSMKMLRILCKPRGNFEFYGRTCFKV  1110
QGY99256.1   1081  LDKARSMKMLRILCKPRGNFEFYGRTCFRV  1110
QYA72351.1   1081  LDKAKSMKMLRILCKPRGNFEFYGRTCFKV  1110
AEK20744.1   1081  LDKARSMKMLRILCKPRGNFEFYGRTCFRV  1110
DAZ85717.1   1081  LDKAKSMKMLRILCKPRGNFEFYGRTCFKV  1110
ADJ39333.1   1089  LDKAKSMKMLRVLCKPRGNFEFYGRTCFKV  1118
DAZ85715.1   1089  LDKARSMKMLRILCKPRGNFEFYGRTCFRV  1118
DAZ85721.1   1089  LDKAKSMKILRILCKPRGNFEFYGRTCFKV  1118
ACF32435.1   1089  LDKAKSMKMLRVLCKPRGNFEFYGRTCFKV  1118
ADJ39332.1   1089  LDKAKSMKMLRVLCKPRGNFEFYGRTCFKV  1118
QYU59217.1   1089  LDKARSMKMLRILCKPRGNFEFYGRTCFRV  1118
DAZ85724.1   1089  LDKAKSMKILRILCKPRGNFEFYGRTCFKV  1118
DAZ85722.1   1089  LDKAKSMKILRILCKPRGNFEFYGRTCFKV  1118
Q65030.1     1102  LDKAKSMKMLRILCKPRGNFEFYGRTCFKV  1131
DAZ85716.1   1055  LDKAKSMKMLRILCKPRGNFEFYGRTCFKV  1084
BAF35853.1   1054  LDKAKSMKMLRVLCKPRGNFEFYGRTCFKV  1083
CAA57413.1   1055  LDKAKSMKMLRILCKPRGNFEFYGRTCFKV  1084
BAF35852.2   1055  LDKAKSMKMLRVLCKPRGNFEFYGRTCFKV  1084
Q91HK5.1     1081  LDQAKSIKVLRVLCKPRPGFSFYGRTSFPV  1110
BAF35851.2   1093  LDKARSMKILRVLCRPKGDFEFYGRTCFKV  1122
```

GFLV isolates P1 polyprotein

```
NP_619689.1  1     MWQVPEGSQCCCTGKSFSNAEAKELRYVCSCWMSTRLVKAEAPPQQSRKSGIAPTPLKSKGTIQVSLPKATGVKPSIHKS  80
AWO68042.1   1     MWQVPEGSQCCCTGKSFSNAEAKELRYVCSCWMSTRLVKAEAPPQQSRKSGIAPTPLKSKGTIQVSLPKATGVKPSIHKS  80
QYU59223.1   1     MWQVPEGSQCCCTGKSYSNAEAKELRYVCSCWMSTRLVKAEAPPQQSRKSGIAPTPLKSKGTVQVPLPKAAGVKPSIQKS  80
ARO69887.1   1     MWQVPEGSQCCCAGKSFSNAEAKELRYVCSCWMGTRLVKAEAPPQQPRKSGIAPTPLKSKGAVSVPLPKAAGVKPSIPKS  80
ARO69840.1   1     MWQVPEGSQCCCTGKSFSNAEAKELRYVCSCWMGTRLVKAEAPPQQPRKSGIAPTPLKSKGAVSVPLPKAAGVKPSIPKS  80
ARO69892.1   1     MWQVPEGSQCCCTGKSFSNAEAKELRYVCSCWMGTRLVKAEAPPQQPRKSGIAPTPLKSKGAVSVPLPKAAGVKPSIPKS  80
ARO69862.1   1     MWQVPEGSQCCCTGKSFSNAEAKELRYVCSCWMGTRLVKAEAPPQQPRKSGIAPTPLKSKGAVSVPLPKAAGVKPSIPKS  80
ARO69844.1   1     MWQVPEGSQCCCTGKSFSNAEAKELRYVCSCWMGTRLVKAEAPPQQPRKSGIAPTPLKSKGAVSVPLPKAAGVKPSIPKS  80
ARO69896.1   1     MWQVPEGSQCCCTGKSFSNAEAKELRYVCSCWMGTRLVKAEAPPQQPRKSGIAPTPLKSKGAVSVPLPKAAGVKPSIPKS  80
QGP72541.1   1     MWQVPEGSQCCCTGKFFSNAEAKELRYACSCYMSTRLVKAEAPPQQPRKSGIAPAPLKSKGTVRVPLPKAAGVKPSIQKS  80
ARO69901.1   1     MWQVPEGSQCCCTGKSFSNAEAKELRYVCSCWMGTRLVKAEAPPQQPRKSGIAPTPLKSKGAVSVPLPKAAGVKPSIPKS  80
AYA22299.1   1     MWQVPAGSQCCCTGKSFSNAEAKELRYVCSCWMSTRLVKAEAPPQQPRKSGIAPTPLKSKGTVQVPLPKAAGVKPSVQKS  80
ARO69890.1   1     MWQVPEGSQCCCTGKSFSNAEAKELRYVCSCWMGTRLVKAEAPPQQPRKSGIAPTPLKSKGAVSVPLPKAAGVKPSIPKS  80
ARO69859.1   1     MWQVPEGSQCCCTGKSFSNAEAKELRYVCSCWMGTRLVKAEAPPQQPRKSGIAPTPLKSKGAVSVPLPKAAGVKPSIPKS  80
ARO69905.1   1     MWQVPEGSQCCCTGKSFSNAEAKELRYVCSCWMGTRLVKAEAPPQQPRKSGIAPTPLKSKGAVSVPLPKAAGVKPSIPKS  80
ARO69871.1   1     MWQVPEGSQCCCTGKSFSNAEAKELRYVCSCWMGTRLVKAEAPPQQPRKSGIVPTPQKSKGAVSVPLPKAAGVKPSIPKS  80
ARO69848.1   1     MWQVPEGSQCCCTGKSFSNAEAKELRYVCSCWMGTRLVKAEAPPQQPRKSGIAPTPLKSKGAVLVPLPKAAGVKPSIPKS  80
ARO69874.1   1     MWQVPEGSQCCCTGKSFSNAEAKELRYVCSCWMGTRLVKAEAPPQQPRKSGIAPTPLKSKGAVSVPLPKAAGVKPSIPKS  80
ARO69882.1   1     MWQVPEGSQCCCTGKSFSNAEAKELRYVCSCWMGTRLVKAEAPPQQPRKSGIVPTPQKSKGAVSVPLPKAAGVKPSIPKS  80
QYU59221.1   1     MWQVPEGSQCCCTGKSFSNAEAKELRYVCSCWMSTRLVKAEVPLQQLKKSGVAPTPLKSKGTVQVPFPKVAGIKPGIQKS  80
ARO69842.1   1     MWQVPEGSQCCCAGKSFSNAEAKELRYVCSCWMGTRLVKAEAPPQQPRKSGIAPTPLKSKGAVSVPLPKAAGVKPSIPKS  80
AFV34731.1   1     MWQVPEGSQCCCTGKSFSNAEAKELRYVCSCWMGTRLVKAEAPPQQSRKSGIAPTPLKSKGTAQVPFPKAAGVEPSIQKS  80
AND76470.1   1     MWQVPEGSQCCCTGKSFSNAEAKELRYVCSCWMGTRLVKAEAPPQQPRKSGIAPTPLKSKGAVSVPLPKAAGVKPSIPKS  80
ACZ58632.1   1     MWQVPEGSQCCCTGKSFSNAEAKELRYVCSCWMSTRLVKAEAPPRQPKKSGIAPTPQESKGTVQVPFPKAAGIKPSIQKS  80
QBZ78374.1   1     MWQVPEGSQCCCTGKSFSNAEAKELRYVCSCWMSTRLVKAEAPPQQPRKSGIAPTPLKSKGTVQVPLPKVAGVMPSIPKS  80
QGP72540.1   1     MWQVPEGSQCCCTGKFFSNAEAKELRYACSCYMSTRLVKAEAPPQQPRKSGIVPAPLKSKGTVRVPLPKAAGVKPSIQKS  80
QBZ78372.1   1     MWQVPEGSQCCCTGKSFSNAEAKELRYVCSCWMSTRLVKAEAPPQQPRKSGIAPTPLKSKGTVQVPLPKVAGVMPSIPKS  80
QYU59224.1   1     MWQVPEGSQCCCTGKSYSNVEAKELRYVCSCWMSTRLVKAEAPPQQSRKSGIAPTPLKSKGTFQVPLPRAAGIKPSDLKS  80
ARO69877.1   1     MWQVPEGSQCCCTGKSFSNAEAKELRYVCSCYMSTRLIKAEVPPQQPRKSGIAPTPLKSKGTAQVPLPKVAGVEPSIQKS  80
ARO69858.1   1     MWQVPEGSQCCCTGKSFSNAEAKELRYVCSCYMSTRLIKAEVPPQQPRKSGIAPTPLKSKGTAQVPLPKVAGVEPSIQKS  80
ARO69855.1   1     MWQVPEGSQCCCTGKSFSNAEAKELRYVCSCWMGTRLVKAEAPPQQPRKSGIAPTPLKSKGAVSVPLPKAAGVKPSIPKS  80
QGP72542.1   1     MWQVPGGSQCCCTGKSYSNAEAKELRYVCSCWMSTRLVKAEAPPQQSRKSGIAPTPSKSKGTVRVPLPKATGVSPSTQES  80
AYQ93055.1   1     MWQVPEGSQCCCTGKSFSNAEAKELRYVCSCYMSTRLVKAEAPPQQPRKSGIAPTPLKSKGTVQVPLPKVAGISPSIRES  80
AYQ93053.1   1     MWQVPEGSQCCCTGKSFSNAEAKELRYVCSCYMSTRLVKAEAPPQQPRKSGIAPTPLKSKGTVQVPLPKVAGISPSIRES  80
AYQ93051.1   1     MWQVPKGSQCCCTGKSFSNVEAKELRYVCSCFMSTRLVKAEAPPQQSRKSGIAPTPLKSKGTVQVPLPKVAGIIPSIQKS  80
AFI71813.1   1     MWQVPEGSQCCCTGKSFSNAEAKELRYVCSCFMCTRLVKAVAPPQQLRKSRIAPAPLKSKGTIEVPSQVAVGVKPVSPKS  80
QGP72539.1   1     MWQVPEGSQCHCTGKSYSNAEAKELRYVCSCWMSTRLVKAEAPPQQSRKSGIAPTPLKSKGTVRVPLPKATGVSPSTQES  80
QYU59226.1   1     MWQVPEGSQCCCTGKSFSNAEAKELRYVCSCWMSTRLVKADVPHQQPKKSGIAPIPLKSKGTAQVPFPKAAGIEPSIQKS  80
ARO69852.1   1     MWQVPEGSQCCCTGKPFSNAEAKELRYVCSCYMSTRLIKAEVPPQQPRKSGIAPTPLKSKGTAQVPLPKVAGVEPSIQKS  80
QGP72543.1   1     MWQVPEGSQCCCTGKSYSNAEAKELRYVCSCWMNTRLVKAEAPPQQPRKSGIAPTPLKSKGTVRVPLPKAARVKPSTLKS  80
QYU59219.1   1     MWQVPEGSQCCCTGKSFSNAEAKELRYVCSCWMSTRLVKAEVPPQQPRKSGIAPTPLKSKETIQVSLPKVAGVRPSIQKS  80
AGT42200.1   1     MWQVPEGSQCCCTGKSFSNVEAKELRYVCSCFMLTRLVKAVAPPQQLRKSRIAPAPLKSKGTIEVPSRVAVGVKPVSPKS  80
ACZ58633.1   1     MWQVPEGSQCCCTGKSFSNAEAKELRYVCSCWMSTRLVKAEAPPQPPRKSGIAPTPPKSKGTIRVPLPKAAGVVPSVQKS  80
QBZ78369.1   1     MWQVPEGSQCCCTGKSFSNAEAKELRYVCSCWMGTRLVKAEAPPQQPRKSRIAPAPLKSKGTVQVPLPTAAGVKPSIQKS  80
ARO69851.1   1     MWQVPEGSQCCCTGKSFSNAEAKELRYVCSCWMGTRLVKAEAPPQQPRKSGIAPTPLKSKGAVSVPLPKAAGVKPSIPKS  80
QYU59220.1   1     MWQVPEGSQCCCTGKSFSNAEAKELRYVCSCWMSTRLVKAEAPPQQLKKSGIAPTPLKSKGTVQVPLPKAAGVKPSIQKS  80
AFV34755.1   1     MWQVPEGSQCCCTGKSFSNAEAKELRYVCSCWMGTRLVKAEAPPQQPKQSRMAPALPKSKGTVQVPSQEVVGAEPIDPES  80
QGP72544.1   1     MWQVPEGSQCCCTGKSYSNAEAKELRYVCSCWMGTRLVRAEAPPQQSRKSGIAPTPLKSKGTAQVPLPKVAGVKPSIQKS  80
QYA72352.1   1     MWQVPEGSQCCCTGKSFSNAEAKELRYVCSCYMSTRLVKAEAPPQQPRKSGIAPTPLKSKGTVKVPSQVAAGVKPISPKS  80
ARO69854.1   1     MWQVPEGSQCCCTGKSFSNAEAKELRYVCSCYMSTRLIKAEVPPQQPRKSGIAPTPLKSKGTAQVPLPKVAGVEPSIQKS  80
ARO69860.1   1     MWQVPEGSQCCCTGKSFSNAEAKELRYVCSCYMSTRLIKAEVPPQQPRKSGIAPTPLKSKGTAQVPLPKVAGVEPSIQKS  80
ARO69863.1   1     MWQVPEGSQCCCTGKPFSNAEAKELRYVCSCYMSTRLIKAEVPPQQPRKSGIAPTPLKSKGTAQVPLPKVAGVEPSIQKS  80
DAZ85707.1   1     MWQVPEGSQCCCTGKSFSNAEAKELRYVCSCFMSTRLVKAEAPPQQPKKSRIAPAPQKSKGTVQVPLPQVAGVKPSIQKS  80
AWO67781.1   1     MWQVPEGSQCCCTGKSFSNAEAKELRYVCSCWMSTRLIKAEAPPQQSRKSGIAPTPLKSKGTVQVPLPKAAGVKPSIQKS  80
QBZ78367.1   1     MWQVPEGSQCCCTGKSFSNAEAKELRYVCSCWMSTRLVKAEAPPQSPRKSGIAPTPLKSKGTTRVPLPKAAGVVPGIQKS  80
ARO69873.1   1     MWQVPEGSQCCCTGKSFSNAEAKELRYVCSCYMSTRLIKAEVPPQQPRKSGIAPTPLKSKGTAQVPLPKVAGVEPSIQKS  80
AND76471.1   1     MWQVPEGSQCCCTGKSFSNAEAKELRYVCSCYMSTRLIKAEVPPQQPRKSGIAPTPLKSKGTAQVPLPKVAGVEPSIQKS  80
ARO69849.1   1     MWQVPEGSQCCCTGKSFSNAEAKELRYVCSCYMSTRLIKAEVPPQQPRKSGIAPTPLKSKGTAQVPLPKVAGVEPSIQKS  80
ARO69889.1   1     MWQVPEGSQCCCTGKSFSNAEAKELRYVCSCYMSTRLIKAEVPPQQPRKSGIAPTPLKSKGTAQVPLPKVAGVEPSIQKS  80
ARO69867.1   1     MWQVPEGSQCCCTGKSFSNAEAKELRYVCSCYMSTRLIKAEVPPQQPRKSGIAPTPLKSKGTAQVPLPKVAGVEPSIQKS  80
ARO69904.1   1     MWQVPEGSQCCCTGKSFSNAEAKELRYVCSCYMSTRLIKAEVPPQQPRKSGIAPTPLKSKGTAQVPLPKVAGVEPSIQKS  80
ARO69869.1   1     MWQVPEGSQCCCTGKSFSNAEAKELRYVCSCYMSTRLIKAEVPPQQPRKSGIAPTPLKSKGTAQVPLPKVAGVEPSIQKS  80
ARO69884.1   1     MWQVPEGSQCCCTGKSFSNAEAKELRYVCSCYMSTRLIKAEVPPQQPRKSGIAPTPLKSKGTAQVPLPKVAGVEPSIQKS  80
ARO69878.1   1     MWQVPEGSQCCCTGKPFSNAEAKELRYVCSCYMSTRLIKAEVPPQQPRKSGIAPTSLKSKGTAQVPLPKVAGVEPSIQKS  80
ARO69885.1   1     MWQVPEGSQCCCTGKSFSNAEAKELRYVCSCYMSTRLIKAEVPPQQPRKSGIAPTPLKSKGTAQVPLPKVAGVEPSIQKS  80
ARO69841.1   1     MWQVPEGSQCCCTGKSFSNAEAKELRYVCSCYMSTRLIKAEVPPQQPRKSGIAPTPLKSKGTAQVPLPKVAGVEPSIQKS  80
ARO69891.1   1     MWQVPEGSQCCCTGKSFSNAEAKELRYVCSCYMSTRLIKAEAPPQQPRKSGIAPTPLKSKGTAQVPLPKVAGVEPSIQKS  80
ARO69868.1   1     MWQVPEGSQCCCTGKSFSNAEAKELRYVCSCYMSTRLIKAEVPPQQPRKSGIAPTPLKSKGTAQVPLPKVAGVEPSIQKS  80
ARO69900.1   1     MWQVPEGSQCCCTGKSFSNAEAKELRYVCSCYMNTRLIKAEAPPQQPRKSGIAPTPLKSKGTAQVPLPKVAGVEPSIQKS  80
DAZ85706.1   1     MWQVPEGSQCCCTGKSFSNVEAKELRYVCSCWMSTRLVKAEAPPQQPRKSRIAPAPLKSKGTTTVPSQEAVGVKPVGPKS  80
ARO69899.1   1     MWQVPEGSQCCCTGKSFSNAEAKELRYVCSCYMSTRLIKAGAPPQQPRKSGIAPTPLKSKGTAQVPLPKVAGVEPSIQKS  80
ARO69898.1   1     MWQVPEGSQCCCTGKSFSNAEAKELRYVCSCYMSTRLIKAEVPPQQPRKSGIAPTPLKSKGTAQVPLLKVAGVEPSIQKS  80
AYA22297.1   1     MWQVPEGSQCCCTGKPFSNAEAKELRYVCSCFMSTRLVKAEAPPQQQRKSGIAPTPLKSKGAIQVPFPKATGVKPSTLKS  80
QYU59222.1   1     MWQVPEGTQCCCTGKSFSNAEAKELRYVCSCFMSTRLVKAEAPPQQPRKSGIAPTPLKSKGTVKVPSQVAAGVKPISPKS  80
ARO69845.1   1     MWQVPEGSQCCCTGKSFSNAEAKELRYVCSCYMSTRLIKAEAPPQQPRKSGIAPTPLKSKGTAQVPLPKVAGVEPSIQKS  80
AND76473.1   1     MWQVPEGSQCCCIGKSFSNAEAKELRYVCSCYMSTRLIKAEAPPQQPRKSGIAPTPLKSKGTAQVPLPKVAGVEPSIQKS  80
ARO69857.1   1     MWQVPEGSQCCCIGKSFSNAEAKELRYVCSCYMSTRLIKAEAPPQQPRKSGIAPTPLKSKGTAQVPLPKVAGVEPSIQKS  80
DAZ85708.1   1     MWQVPEGSQCCCTGKSFSNAEAKELRYVCSCFMSTRLVKAEAPPQQPRKSGIAPTPPKSKGTVQVPLPKATGVKPISQKS  80
ARO69881.1   1     MWQVPEGSQCCCTGKSFSNAEAKELRYVCSCFMSTRLVKAEAPPQQPRKSGIEPTPLKSKGTVKVPSQVAVGVKPISPKS  80
ARO69893.1   1     MWQVPEGSQCCCTGKSFSNAEAKELRYVCSCFMSTRLVKAEAPPQQPRKSGIEPTPLKSKGTVKVPSQVAVGVKSISPKS  80
ARO69897.1   1     MWQVPEGSQCCCTGKSFSNAEAKELRYVCSCFMSTRLVKAEAPPQQPRKSGIEPTPLKSKGTVKVPSQVAVGVKSISPES  80
ARO69872.1   1     MWQVPEGSQCCCTGKSFSNAEAKELRYVCSCFMSTRLVKAEAPPQQPRKSGIEPTPLKSKGTVKVPSQVAVGVKSISPES  80
ARO69843.1   1     MWQVPEGSQCCCTGKSFSNAEAKELRYVCSCFMSTRLVKAEAPPQQPRKSGIEPTPLKSKGTVKVPSQVAVGVKSISPKS  80
ARO69886.1   1     MWQVPEGSQCCCTGKSFSNAEAKELRYVCSCFMSTRLVKAEAPPQQPRKSGIEPTPLKSKGTVKVPSQVAVGVKSISPES  80
ARO69864.1   1     MWQVPEGSQCCCTGKSFSNAEAKELRYVCSCFMSTRLVKAEAPPQQPRKSGIEPTPLKSKGTVKVPSQVAVGVKPISPKS  80
ARO69876.1   1     MWQVPEGSQCCCTGKSFSNAEAKELRYVCSCFMSTRLVKAEAPPQQPRKSGIEPTPLKSKGTVKVPSQVAVGVKSISPES  80
```

```
ARO69888.1   1     MWQVPEGSQCCCTGKSFSNAEAKELRYVCSCFMSTRLVKAEAPPQQPRKSGIEPTPLKSKGTVKVPSQVAVGVKSISPES  80
ARO69853.1   1     MWQVPEGSQCCCTGKSFSNAEAKELRYVCSCFMSTRLVKAEAPPQQPRKSGIEPTPLKSKGTVKVPSQVAVGVKSISPES  80
ARO69903.1   1     MWQVPEGSQCCCTGKSFSNAEAKELRYVCSCFMSTRLVKAEAPPQQPRKSGIEPTPLKSKGTVKVPSQVAVGVKSISPES  80
ARO69865.1   1     MWQVPEGSQCCCTGKSFSNAEAKELRYVCSCFMSTRLVKAEAPPQQPRKSGIEPTPLKSKGTVKVPSQVAVGVKSISPES  80
ARO69902.1   1     MWQVPEGSQCCCTGKSFSNAEAKELRYVCSCFMSTRLVKAEAPPQQPRKSGIEPTPLKSKGTVKVPSQVAVGVKSISPES  80
ARO69861.1   1     MWQVPEGSQCCCTGKSFSNAEAKELRYVCSCFMSTRLVKAEAPPQQPRKSGIEPTPLKSKGTVKVPSQVAVGVKSISPES  80
ARO69850.1   1     MWQVPEGSQCCCTGKSFSNAEAKELRYVCSCFMSTRLVKAEAPPQQPRKSGIEPTPLKSKGTVKVPSQVAVGVKSISPES  80
QYU59227.1   1     MWQVPEGSQCCCTGKSFSNAEAKELRYVCSCWMNTRLVKAEAPPQQPRKSRTAPAPLKSKGTIEVPSQVAVGVMPVSLKS  80
AFM91094.1   1     MWQVPEGSQCCCTGKSFSNAEAKELRYVCSCYMSTRLVKAEAPPQQPRKSGIAPTPLKSKGTVQVPFPKAAGVKPSIQKS  80
ARO69883.1   1     MWQVPEGSQCCCTGKSFSNAEAKELRYVCSCYMSTRLVKAEAPPQQPRKSGIAPTPLKSKGTVKVPSQVAVGVKSISPES  80
ARO69879.1   1     MWQVPEGSQCCCTGKSFSNAEAKELRYVCSCFMSTRLVKAEAPPQQPRKSGIEPTPLKSKGTVKVPSQVAVGVKSISPES  80
AND76472.1   1     MWQVPEGSQCCCTGKSFSNAEAKELRYVCSCFMSTRLVKAEAPPQQPRKSGIEPTPLKSKGTVKVPSQVAVGVKSISPES  80
ARO69846.1   1     MWQVPEGSQCCCTGKSFSNAEAKELRYVCSCFMSTRLVKAEAPPQQPRKSGIAPTPLKSKGTVKVPSQVAVGVKSISPES  80
ARO69870.1   1     MWQVPEGSQCCCTGKSFSNAEAKELRYVCSCYMSTRLVKAEAPPQQPRKSGIAPTPLKSKGTVKVPSQVAVGVKSISPES  80
ARO69856.1   1     MWQVPEGSQCCCTGKSFSNAEAKELRYVCSCYMSTRLVKAEAPPQQPRKSGIAPTPLKSKGTVKVPSQVAVGVKSISPES  80
ARO69847.1   1     MWQVPEGSQCCCTGKSFSNAEAKELRYVCSCYMSTRLIKAEAPPQQPRKSGIAPTPLKSKGTTQAPLPKVAGVEPSILKS  80
ARO69875.1   1     MWQVPEGSQCCCTGKSFSNAEAKELRYVCSCYMSTRLIKAEAPPQQPRKSGIAPTPLKSKGTAQAPLPKVAGVEPSILKS  80
ARO69895.1   1     MWQVPEGSQCCCTGKSFSNAEAKELRYVCSCYMSTRLIKAEAPPQQPRKSGIAPTPLKSKGTAQAPLPKVAGVEPSILKS  80
ARO69880.1   1     MWQVPEGSQCCCTGKSFSNAEAKELRYVCSCYMSTRLIKAEAPPQQPRKSGIAPTPLKSKGTAQAPLPKVAGVEPSILKS  80
BCT87061.1   1     MWQVPEGSQCCCTGKSFSNAEAKELRYVCSCWMSTRLVKVEVPPQQPKKSRIAPAPLKSKEVVQTSFPKVAGVMPSSPKS  80
QYU59225.1   1     MWQISEGSQCCCTGKSWSNAEAKEARYVCSCYLSCRLVKVGVVPQPSRKSGIAPTPLKSKGTIQVPSQEVVGVKPINLKS  80
APD26306.1   1     MWQVPEGSQCCCTGKSFSNAEAKELRYVCSCWMSTRLIKAEAPPQQTRKSRIAPAPLKSKETVQVSFPNVVGIKPNNPKS  80


NP_619689.1  81    KGASVAPAPLLKQRCEVVVQYGPPADIELVYPPLVREEEKSSNIVVLPPTQKVEVRVPVCCAPKWMVAIPKPPVKLAPKA  160
AWO68042.1   81    KGASVAPAPLLKQRCEVVVQYGPPADIELVYPPLVREEEKSSNIVVLPPTQKVEVRVPVCCAPKWMVAIPKPPVKLAPKA  160
QYU59223.1   81    KGASVAPAPLLKQRCEVVVQYGPPADIELVYPPLVREEEKSSKKVVLPPTQKVDVRVPVCCAPKWMVAIPKPPVKLAPKA  160
ARO69887.1   81    KGASVAPAPLLKQRCEVVVQYGPPADIELVYPPLVRKEEKSSKEVVLPPTPKVEVRVPVCCAPKWMVAIPKPPVKLAPKA  160
ARO69840.1   81    KGASVAPAPLLKQRCEVVVQYGPPANIELVYPPLVRKEEKSSKEVVLPPTPKVEVRVPVCCAPKWMVAIPKPPVKLAPKA  160
ARO69892.1   81    KGASVAPAPLLKQRCEVVVQYGPPANIELVYPPLVRKEEKSSKEVVLPPTPKVEVRVPVCCAPKWMVAIPKPPVKLAPKA  160
ARO69862.1   81    KGASVAPAPLLKQRCEVVVQYGPPADIELVYPPLVRKEEKSSKEVVLPPTPKVEVRVPVCCAPKWMVAIPKPPVKLAPKA  160
ARO69844.1   81    KGASVAPAPLLKQRCEVVVQYGPPANIELVYPPLVRKEEKSSKEVVLPPTPKVEVRVPVCCAPKWMVAIPKPPVKLAPKA  160
ARO69896.1   81    KGASVAPAPLLKQRCEVVVQYGPPANIELVYPPLVRKEEKSSKEVVLPPTPKVEVRVPVCCAPKWMVAIPKPPVKLAPKA  160
QGP72541.1   81    KGASVAPTPLLKQRCEVVVQYGPPADIELVYPPLVREEEESSDKVVLPPAPKVEVRVPICCAPKWMVAIPKPPVKLAPKV  160
ARO69901.1   81    KGASVAPAPLLKQRCEVVVQYGPPANIELVYPPLVRKEEKSSKEVVLPPTPKVEVRVPVCCAPKWMVAIPKPPVKLAPKA  160
AYA22299.1   81    KGASVVPAPLLKQRCKVVVQYGPPANIELVYPPLVREEEKSSSKVVLPPTPKVEVRVPVCCAPKWMVAIPKPPVKLAPKA  160
ARO69890.1   81    KGASVAPAPLLKQRCEVVVQCGPPANIELVYPPLVRKEEKSSKEVVLPPTPKVEVRVPVCCAPKWMVAIPKPPVKLAPRA  160
ARO69859.1   81    KGASVAPAPLLKQRCEVVVQYGPPANIELVYPPLVRKEEKSSKEVVLPPTPKVEVRVPVCCAPKWMVAIPKPPVKLAPRA  160
ARO69905.1   81    KGASVAPAPLLKQRCEVVVQYGPPADIELVYPPLVRKEEKSSKEVVLPPTPKVEVRVPVCCAPKWMVAIPKPPVKLAPKA  160
ARO69871.1   81    KGASVAPAPLLKQRCEVVVQYGPPADIELVYPPLVRKEEKSSKEVVLPPTPKVEVRVPVCCAPKWMVAIPKPPVKLAPKA  160
ARO69848.1   81    KGASVAPAPLLKQRCEVVVQYGPPADIELVYPPLVRKEEKSSKEVVLPPTPKVEVRVPVCCAPKWMVAIPKPPVKLAPKA  160
ARO69874.1   81    KGASVAPAPLLKQRCEVVVQYGPPADIELVYPPLVRKEEKSSKEVVLPPTPKVEVRVPVCCAPKWMVAIPKPPVKLAPKA  160
ARO69882.1   81    KGASVAPAPLLKQRCEVVVQYGPPADIELVYPPLVRKEEKSSKEVVLPPTPKVEVRVPVCCAPKWMVAIPKPPVKLAPKA  160
QYU59221.1   81    KGASVAPTPPLKQRCEVIVQYGPPADIELIYPPLVRKEEETSNEVVLPPTQKVEVRVPVCCAPKWMVAIPKPPVKLAPKA  160
ARO69842.1   81    KGASVAPAPLLKQRCEVVVQYGPPADIELVYPPLVRKEEKSSKEVVLPPTPKVEVRVPVCCAPKWMVAIPKPPVKLAPKA  160
AFV34731.1   81    KGASVAPTPLLKQRCEVVVQYGPPADIELVYPPLVREEEKSSGKVVLPPTPKVEVRVPVCCAPKWMVAIPKPPVKLAPKA  160
AND76470.1   81    KGASVAPAPLLKQRCEVVVQYGPPADIELVYPPLVRKEEKSSKEVVLPPTPKVEVRVPVCCAPKWMVAIPKPQVKLAPKA  160
ACZ58632.1   81    KGASVAPAPLFKQRCEVVVQYGPPADIELVYPPLVREEEKSSKKVVLPPTPKVEARVPVCCAPKWMVALPRPPVKLAPKA  160
QBZ78374.1   81    KGALVAPTPLLKQRCEVVVQYGPPADIELVYPPLVREEEKSSNQVVLPPTPKVDVRVPICCAPKWMVAISKPPVKLAPKA  160
QGP72540.1   81    KGASVAPTPLLKQRCEVVVQYGPPADIELVYPPLVREEEESSDKVVLPPAPKVEVRVPICCAPKWMVAIPKPPVKLAPKV  160
QBZ78372.1   81    KGALVAPTPLLKQRCEVVVQYGPPADIELVYPPLVREEEKSSNQVVLPPTPKVDVRVPICCAPKWMVAISKPPVKLAPKA  160
QYU59224.1   81    KGTSVVPTPLLKQRCEVVVQYGPPADIELVYPPLVREEEKSSKKVVLPPTPKVEVRVPVCCAPKWMVAIPKPPVKLAPKA  160
ARO69877.1   81    KGASVAPAPLLKQRCEVVVQYGPPANIELVYSPLVREEEKSSEKVVLPPTPKVDVRVPVCCAPKWMVVIPKPPVKLAPKA  160
ARO69858.1   81    KGASVAPAPLLKQRCEVVVQYGPPANIELVYSPLVREEEKSSEKVVLPPTPKVDVRVPVCCAPKWMVVIPKPPVKLAPKA  160
ARO69855.1   81    KGASVAPAPLLKQRCEVVVQYGPPADIELVYPPLVRKEEKSSKEVVLPPTPKVEVRVPVCCAPKWMVAIPKPPVKLAPKA  160
QGP72542.1   81    KGASVAPAPLLKQRCEVIVQYGPPADIELVYPPLVREEGKSSNKVVLPPTQKVEVRVPVCCAPKWMVAIPKPPVKLASRA  160
AYQ93055.1   81    KGASVAPAPLLKQRCEVIVQYGPPASIELVYPPLVREEEKTSEKTALPPAKKVDVRVPVCCAPKWMVAIPKPPVKLAPKA  160
AYQ93053.1   81    KGASVAPAPLLKQRCEVIVQYGPPASIELVYPPLVREEEKTSEKTALPPAKKVDVRVPVCCAPKWMVAIPKPPVKLAPKA  160
AYQ93051.1   81    KGASEAPTPLLKQRCEVVVQYGPPADIELVYPPLVREEEKSSNTVVLPPTPKVEVRVPVCCAPKWMVAIPKPPVKLAPKA  160
AFI71813.1   81    KGTSVVPTPLLKQRCEVVVQYGPPADIELVYPPLVREEEKSSKEMALPPAKKVDVRVPVCCAPRWMVAISKPPKKLAVKA  160
QGP72539.1   81    KGASVAPAPLLKQRCEVIVQYGPPADIELVYPPLVREEGKSSNKVVLPPTQKVEVRVPVCCAPKWMVAIPKPPVKLAPRA  160
QYU59226.1   81    KGASVAPTPLLKQRCEVIVQYGPPADIELVYPPLVREEEKSSTKVVLPPTPKVEVRVPACCAPKWMVAIPKPPVKLAPKA  160
ARO69852.1   81    KGASVAPAPLLKQRCEVVVQYGPPANIELVYPPLVREEEKSSEKVVLPPTPKVDVRVPACCAPKWMVVIPKPPVRLAPKA  160
QGP72543.1   81    KGASVAPAPLLKQRCEVIVQYGPPADIELVYPPLVRGEEESSHEVVLPPTQKVEVRVPVCCAPKWMVAISKPPVKLAPKA  160
QYU59219.1   81    KGASVAPTPLLKQRCEVIVQYGPPADIELVYPPLVREEENSSKKVVLPPTKKVDVRVPVNCAPRWMVAIPKPPVKLAPKA  160
AGT42200.1   81    KGTSVVPTPLLKQRCEVVVQYGPPADIELVYPPLVREEEKSSKEMALPPAKKVDVRVPVCCAPKWMVAISKPPKKLAVKA  160
ACZ58633.1   81    KGASVAPAPLLKQRCEVVVQYGPPADIELVYPPLVREEEKSSDKVVLPPTLKAEARVPVCCAPKWMVAIPKPPVKLAPKA  160
QBZ78369.1   81    KGASVAPTPLLKQRCEVIVQYGPPADIELVYPPLVREEEKSSIKVVLPPTPKVEVRVPACCAPKWMVAIPKPPVKLAPKA  160
ARO69851.1   81    KGASVAPAPLLKQRREVVVQYGPPANIELVYPPLVRKEEKSSKEVVLPPTPKVEVRVPVCCAPKWMVAIPKPPVKLAPKA  160
QYU59220.1   81    KGASVAPVPLLKQRCEVVVRYGPPADIELVYPPIVREEVKSSNRVVLPPTPKVEVRVPACCAPKWMVAIPKPPMKPAPKA  160
AFV34755.1   81    KGASVAPTPLLKQRCEVVVQYGPPVDIELVYPPLVREEEKSSKEKALPPAAKVEIRVPVCCAPKWMVATPKPPVKLAPKA  160
QGP72544.1   81    KGAFVAPTPLLKQRCEVVVQYGPPADIELVYPPLVREEEESSKKVVLPPTPKVEVRVPACCAPKWMVAIPKPPVKLAPKA  160
QYA72352.1   81    KGASVAPAPLHKQRCEVIVQYGPPADIELIYPPLVREEEKSSKQMALPPARKVDIRVPVSCAPRWMVAIPKPLVKLAPKA  160
ARO69854.1   81    KGASVAPAPLLKQRCEVVVQYGPPANIELVYSPLVREEEKSSEKVVLPPTPKVDVRVPVCCAPKWMVVIPKPPVKLAPKA  160
ARO69860.1   81    KGASVAPAPLLKQRCEVVVQYGPPANIELVYSPLVREEEKSSEKVVLPPTPKVDVRVPVCCAPKWMVVIPKPPVKLAPKA  160
ARO69863.1   81    KGASVAPAPLLKQRCEVVVQYGPPANIELVYSPLVREEEKSSEKVVLPPTPKVDVRVPVCCAPKWMVVIPKPPVKLAPKA  160
DAZ85707.1   81    KGASVAPAPLLKQRCEVVVQYGPPADIELVYPPLVREEEKSSKKVVLPPTPKVEVRVPICCAPKWLVAIPKPPVRLAPKA  160
AWO67781.1   81    KGASVAPAPLLKQRCEVVVQYGPPADIELVYPPLVRKEEESSNKVVLPPAPKVEVRVPVCCAPKWMVAIPKPPVKLAPKA  160
QBZ78367.1   81    KGASMVPAPLLKQRCEVVVQYGPPADIELVYPPLVREEEKSSDKVVLPPTLKAEARVPVCCAPKWMVAIPKPPVKLAPKA  160
ARO69873.1   81    KGASVAPAPLLKQRCEVVVQYGPPANIELVYSPLVREEEKSSEKVVLPPTPKVDVRVPVCCAPKWMVVIPKPPVKLAPKA  160
AND76471.1   81    KGASVAPAPLLKQRCEVVVQYGPPANIELVYSPLVREEEKSSEKVVLPPTPKVDVRVPVCCAPKWMVVIPKPPVKLAPKA  160
ARO69849.1   81    KGASVAPAPLLKQRCEVVVQYGPPANIELVYSPLVREEEKSSEKVVLPPTPKVDVRVPVCCAPKWMVVIPKPPVKLAPKA  160
ARO69889.1   81    KGASVAPAPLLKQRCEVVVQYGPPANIELVYSPLVREEEKSSEKVVLPPTPKVDVRVPVCCAPKWMVVIPKPPVKLAPKA  160
ARO69867.1   81    KGASVAPAPLLKQRCEVVVQYGPPANIELVYSPLVREEEKSSKKVVLPPTPKVDVRVPVCCAPKWMVVIPKPPVKLAPKA  160
ARO69904.1   81    KGASVAPAPLLKQRCEVVVQYGPPANIELVYSPLVREEEKSSEKVVLPPTPKVDVRVPVCCAPKWMVVIPKPPVKLAPKA  160
ARO69869.1   81    KGASVAPAPLLKQRCEVVVQYGPPANIELVYSPLVREEEKSSEKVVLPPTPKVDVRVPVCCAPKWMVVIPKPPVKLAPKA  160
ARO69884.1   81    KGASVAPAPLLKQRCEVVVQYGPPANIELVYSPLVREEEKSSEKVVLPPTPKVDVRVPVCCAPKWMVVIPKPPVKLAPKA  160
ARO69878.1   81    KGASVAPAPLLKQRCEVVVQYGPPANIELVYSPLVREEEKSSEKVVLPPTPKVDVRVPVCCAPKWMVVIPKPPVKLAPKA  160
```

ARO69885.1	81	KGASVAPAPLLKQRCEVVVQYGPPANIELVYSPLVREEEKSSKKVVLPPTPKVDVRVPVCCAPKWMVVIPKPPVKLAPKA	160
ARO69841.1	81	KGASVAPAPLLKQRCEVVVQYGPPANIELVYSPLVREEEKSSEKVVLPPTPKVDVRVPVCCAPKWMVVIPKPPVKLAPKA	160
ARO69891.1	81	KGASAAPTPLLKQRCEVVVQYGPPANIELVYPPLVREEEKSSEKVVLPPTPKVDVRVPACCAPKWMVVIPKPPVKLAPKA	160
ARO69868.1	81	KGASAAPTPLLKQRCEVVVQYGPPANIELVYPPLVREEEKSSEKVVLPPTPKVDVRVPACCAPKWMVVIPKPPVKLAPKA	160
ARO69900.1	81	KGASVAPAPLLKQRCEVVVQYGPPANIELVYSPLVREEEKSSEKVVLPPTLKVDVRVPVCCAPKWMVVIPKPPVKLAPKA	160
DAZ85706.1	81	KGASVAPAPLLKQRCEVVVQYGPPADIELVYPPLVREEEKSSKKMALPPAKKVEVRVPVCCAPKWMVAIPKPPVKLAPKA	160
ARO69899.1	81	KGASAAPTPLLKQRCEVVVQYGPPANIELVYPPLVREEEKSSEKVVLPPTPKVDVRVPACCAPKWMVVIPKPPVKLAPKA	160
ARO69898.1	81	KGASVAPAPLLKQRCEVVVQYGPPANIELVYSPLVREEEKSSEKVVLPPTPKVDVRVPVCCAPKWMVVIPKPPVKLAPKA	160
AYA22297.1	81	KGASVAPAPLRKQRCEVVVQYGPPADIELVYPPLVREVEESSAKMVLPPTSKVEVRVPVCCAPKWMVAIPKPPVKLAPKA	160
QYU59222.1	81	KGASVAPAPLQKQRREVVVQYGPPADIELVYPPLVREEEKSSKRMALPPARKVDIRVPVSCAPKWMVAIPKPPVKLAPKA	160
ARO69845.1	81	KGASAAPTPLLKQRCEVVVQYGPPANIELVYPPLVREEEKSSEKVVLPPTPKVDVRVPACCAPKWMVVIPKPPVKLAPKA	160
AND76473.1	81	KGASVAPTPLLKQRCEVVVQYGPPANIELVYPPLVREEEKSSEKVVLPPTPKVDVRVPACCAPKWMVVIPKPPVKLAPKA	160
ARO69857.1	81	KGASVAPTPLLKQRCEVVVQYGPPANIELVYPPLVREEEKSSEKVVLPPTPKVDVRVPACCAPKWMVVIPKPPVKLAPKA	160
DAZ85708.1	81	KGASVAPTPLLKQRCEVVVQYGPPADIELVYPPLVREEEQSSDNVVLPPTQKVEVRVPVCCAPKWMVAIPKPPVKLAPKA	160
ARO69881.1	81	KGASVAPTPLQKQRCEVIVQYGPPADIELVYPPLVREEEKSSKQMALPPARKVDIRVPVSCAPRWMVAIPKPPVKLAPKA	160
ARO69893.1	81	KGASVAPTPLQKQRCEVIVQYGPPADIELVYPPLVREEEKSSKQMALPPARKVDIRVPVSCAPRWMVAIPKPPVKLAPKA	160
ARO69897.1	81	KGASVAPTPLQKQRCEVIVQYGPPADIELVYPPLVREEEKSSKQMALPPARKVDIRVPVSCAPRWMVAIPKPPVKLAPKA	160
ARO69872.1	81	KGASVAPTPLRKQRCEVIVQYGPPADIELVYPPLVREEEKSSKQMALPPARKVDIRVPVSCAPRWMVAIPKPPVKLAPKA	160
ARO69843.1	81	KGASVAPTPLQKQRCEVIVQYGPPADIELVYPPLVREEEKSSKQMALPPARKVDIRVPVSCAPRWMVAIPKPPVKLAPKA	160
ARO69886.1	81	KGASVAPTPLRKQRCEVIVQYGPPADIELVYPPLVREEEKSSKQMALPPARKVDIRVPVSCAPRWMVAIPKPPVKLAPKA	160
ARO69864.1	81	KGASVAPTPLQKQRCEVIVQYGPPADIELVYPPLVREEEKSSKQMALPPARKVDIRVPVSCAPRWMVAIPKPPVKLAPKA	160
ARO69876.1	81	KGASVAPTPLQKQRCEVIVQYGPPADIELVYPPLVREEEKSSKQMALPPARKVDIRVPVSCAPRWMVAIPKPPVKLAPKA	160
ARO69888.1	81	KGASVAPTPLQKQRCEVIVQYGPPADIELVYPPLVREEEKSSKQMALPPARKVDIRVPVSCAPRWMVAIPKPPVKLAPKA	160
ARO69853.1	81	KGASVAPTPLRKQRCEVIVQYGPPADIELVYPPLVREEEKSSKQMALPPARKVDIRVPVSCAPRWMVAIPKPPVKLAPKA	160
ARO69903.1	81	KGASVAPTPLQKQRCEVIVQYGPPADIELVYPPLVREEEKSSKQMALPPARKVDIRVPVSCAPRWMVAIPKPPVKLAPKA	160
ARO69865.1	81	KGASVAPTPLQKQRCEVIVQYGPPADIELVYPPLVREEEKSSKQMALPPARKVDIRVPVSCAPRWMVAIPKPPVKLAPKA	160
ARO69902.1	81	KGASVAPTPLQKQRCEVIVQYGPPADIELVYPPLVREEEKSSKQMALPPARKVDIRVPVSCAPRWMVAIPKPPVKLAPKA	160
ARO69861.1	81	KGASVAPTPLRKQRCEVIVQYGPPADIELVYPPLVREEEKSSKQMALPPARKVDIRVPVSCAPRWMVAIPKPPVKLAPKA	160
ARO69850.1	81	KGASVAPTPLQKQRCEVIVQYGPPADIELVYPPLVREEEKSSKQMALPPARKVDIRVPVSCAPRWMVAIPKPPVKLAPKA	160
QYU59227.1	81	KGASVAPTPLLKQRCEVVVQYGPPADIELIYPPLVREEEKSSTKVVLPPTPKVEVRVPVCCAPKWMVAIPKPPVKLAPKA	160
AFM91094.1	81	KGASVAPTPLLKQRCEVVVQYGPPADIELVYPPLVREEEKSSNKVVLPPTPKVEIGVPACCAPKWMVAIPKPPVRLAPKA	160
ARO69883.1	81	KGASVAPTPLRKQRCEVIVQYGPPADIELVYPPLVREEEKSSKQMALPPARKVDIRVPVSCAPRWMVAIPKPPVKLAPKA	160
ARO69879.1	81	KGASVAPTPLQKQRCEVIVQYGPPADIELVYPPLVREEEKSSKQMALPPARKVDIRVPVSCAPRWMVAIPKPPVKLAPKT	160
AND76472.1	81	KGASVAPTPLQKQRCEVIVQYGPPADIELVYPPLVREEEKSSKQMALPPARKVDIRVPVSCAPRWMVAIPKPPVKLAPKA	160
ARO69846.1	81	KGASVAPTPLQKQRCEVIVQYGPPADIELVYPPLVREEEKSSKQMALPPARKVDIRVPVSCAPRWMVAVPKPPVKLAPKA	160
ARO69870.1	81	KGASVAPTPLRKQRCEVIVQYGPPADIELVYPPLVREEEKSSKQMALPPARKVDIRVPVSCAPRWMVAIPKPPVKLAPKA	160
ARO69856.1	81	KGASVAPTPLRKQRCEVIVQYGPPADIELVYPPLVREEEKSSKQMALPPARKVDIRVPVSCAPRWMVAIPKPPVKLAPKA	160
ARO69847.1	81	KGASVAPAPLLKQRCEVVVQYGPPANIELIYPPFVREEEKSSEKVVLPPTPKVDVRVPACCAPKWMVVIPKPPVKLAPKA	160
ARO69875.1	81	KGASVAPAPLLKQRCEVVVQYGPPANIELIYPPFVREEEKSSEKVVLPPTPKVDVRVPACCAPKWMVVIPKPPVKLAPKA	160
ARO69895.1	81	KGASVAPAPLLKQRCEVVVQYGPPANIELIYPPFVREEEKSSEKVVLPPTPKVDVRVPACCAPKWMVVIPKPPVKLAPKA	160
ARO69880.1	81	KGASVAPAPLLKQRCEVVVQYGPPANIELIYPPFVREEEKSSEKVVLPPTPKVDVRVPACCAPKWMVVIPKPPVKLAPKA	160
BCT87061.1	81	KGASVAPVPLLKQRCEVVVQYGPPADIELVYPPLVREEEKSSQKKVLPPAKKVDVRVPASCAPKWMVAIPRPPVKLAPKA	160
QYU59225.1	81	KGVSMAPTPLLKQRCEVVVQYGPPASIELVYPPLVGEEEESSRKMALPPAKKVEVRVPVSCAPRWMVAIPKPPVKLAPKV	160
APD26306.1	81	KGTSVVPAPLLKQRCEVVVQYGPPADIELVYPPLVREEEKSSKRKVLPPTKKVEVRVPACCAPKWMVAIPKPPIKLAPRV	160

NP_619689.1	161	SKLRFPKGAVAYNGVNFIDTKGKVVLSEGAKRILRGIRVAAKQRLRAARRSAACKKVRAKRALAEFEAIVQSERLDQLKT	240
AWO68042.1	161	SKLRFPKGAVAYNGVNFIDTKGKVVLSEGAKRILRGIRVAAKQRLRAARRSAACKKVRAKRALAEFEAIVQSERLDQLKT	240
QYU59223.1	161	SKLRFPKGAIAYNGVNFIDTKGKVVLSEGAKRILKGIRVAARQRLRAARRAAACKKVRAKRALAEFEAIAQSERLDQLET	240
ARO69887.1	161	SKLRFPKGAVAYNGVNFIDTKGKVVLSEGAKRILKGIRVAIKQRLRAVRRSAACKKVRAARALANFEAIVQNERLDQLET	240
ARO69840.1	161	SKLRFPKGAVAYNGVNFIDTKGKVVLSEGAKRILKGIRVAIKQRLRAVRRSAACKKVRAARALANFEAIVQNERLDQLET	240
ARO69892.1	161	SKLRFPKGAVAYNGVNFIDTKGKVVLSEGAKRILKGIRVAIKQRLRAVRRSAACKKVRAARALANFEAIVQNERLDQLET	240
ARO69862.1	161	SKLRFPKGAVAYNGVNFIDTKGKVVLSEGAKRILKGIRVAIKQRLRAVRRSAACKKVRAARALANFEAIVQNERLDQLET	240
ARO69844.1	161	SKLRFPKGAVAYNGVNFIDTKGKVVLSEGAKRILKGIRVAIKQRLRAVRRSAACKKVRAARALANFEAIVQNERLDQLET	240
ARO69896.1	161	SKLRFPKGAVAYNGVNFIDTKGKVVLSEGAKRILKGIRVAIKQRLRAVRRSAACKKVRAARALANFEAIVQNERLDQLET	240
QGP72541.1	161	SKLQFPKGAVAYNGVNFIDTKGKVVLSEGAKRILKGIRVATKQRLRAVRRSAACKKVRAKRALAKFEAIVLSERLDQLET	240
ARO69901.1	161	SKLRFPKGAVAYNGVNFIDTKGKVVLSEGAKRILKGIRVAIKQRLRAVRRSAACKKVRAARALANFEAIVQNERLDQLET	240
AYA22299.1	161	SKLRFPRGAVAYNGVNFIDTKGKVVLSEGAKRILKGIRVATRQRLRAARRSAACKKVRAARALAKFEAIVQNGRLDQLKT	240
ARO69890.1	161	SKLRFPKGAVAYNGVNFIDTKGKVVLSEGAKRILKGIRVAIKQRLRAVRRSAACKKVRAARALANFEAIVQNERLDQLET	240
ARO69859.1	161	SKLRFPKGAVAYNGVNFIDTKGKVVLSEGAKRILKGIRVAIKQRLRAVRRSAACKKVRAARALANFEAIVQNERLDQLET	240
ARO69905.1	161	SKLRFPKGAVAYNGVNFIDTKGKVVLSEGAKRILKGIRVAIKQRLRAVRRSAACKKVRAARALANFEAIVQNERLDQLET	240
ARO69871.1	161	SKLRFPKGAVAYNGVNFIDTKGKVVLSEGAKRILKGIRVATKQRSRAVRRSAACKKVRAARALANFEAIVQNERLDQLET	240
ARO69848.1	161	SKLRFPKGAVAYNGVNFIDTKGKVVLSEGAKRILKGIRVAIKQRLRAVRRSAACKKVRAARALANFEAIVQNERLDQLET	240
ARO69874.1	161	SKLRFPKGAVAYNGVNFIDTKGKVVLSEGAKRILKGIRVAIKQRSRAVRRSAACKKVRAARALANFEAIVQNERLDQLET	240
ARO69882.1	161	SKLRFPKGAVAYNGVNFIDTKGKVVLSEGAKRILKGIRVAIKQRSRAVRRSAACKKVRAARALANFEAIVQNERLDQLET	240
QYU59221.1	161	SKLRFPKGAVAYNGVNFIDIKGTVVLSEGAKRILKGIRVATKQRLRAMRRSAACRKVRAKRALAEFEAIVQNERLDQLDT	240
ARO69842.1	161	SKLRFPKGAVAYNGVNFIDTKGKVVLSEGAKRILKGIRVAIKQRLRAVRRSAACKKVRAARALANFEAIVQNERLDQLET	240
AFV34731.1	161	NKLRFPKGAVAYNGVNFIDAKGKVVLSEGAKRILRGIRVAAKQRSRAARRSAACKKVRAARALAKFEAIVLNARLDQLKT	240
AND76470.1	161	SKLRFPKGAVAYNGVNFIDTKGKVVLSEGAKRILKGIRVAIKQRLRAVRRSAACKKVRAARALANFEAIVQNERLDQLET	240
ACZ58632.1	161	SKLQFPKGAVAYNGVNFIDIKGKVVLSEGAKRILKGIRVAAKQRHRAARRSAACKEVRAKRALAKFEAIVQSERLDQLET	240
QBZ78374.1	161	GKLRFPKGAVAYNGVNFIDTKGKVVLSEGAKRILKGIRVAIKQRLRAVRRSAACKKVRAKRALAEFEAIVQKERLDQLET	240
QGP72540.1	161	SKLQFPKGAVAYNGVNFIDTKGKVVLSEGAKRILKGIRVATKQRLRAVRRSAACKKVRAKRALAKFEAIVLSERLDQLET	240
QBZ78372.1	161	GKLRFPKGAVAYNGVNFIDTKGKVVLSEGAKRILKGIRVAIKQRLRAVRRSAACKKVRAKRALAEFEAIVQKERLDQLET	240
QYU59224.1	161	SKLRFPKGAVAYNGVNFIDIKGKVVLSEGAKRILKGIRVATKQRLRAARRSAACRKVRAARALAKFEAIVQSERLDQLET	240
ARO69877.1	161	SKLRFPKGAVAYNGVNFIDTKGKVVLSEGAKRILKGIRVATKQRLRAARRSTACKKVRAARALAKFEAIVQNERLDQLET	240
ARO69858.1	161	SRLRFPKGAVAYNGVNFIDTKGKVVLSEGAKRILKGIRVATKQRLRAARRSTACKKVRAARALAKFEAIVQNERLDQLET	240
ARO69855.1	161	SKLRFPKGAVAYNGVNFIDTKGKVVLSEGAKRILKGIRVAIKQRSRAVRRSAACKKVRAARALANFEAIVQNERLDQLET	240
QGP72542.1	161	SKLRFPKGAVAYNGVNFIDTKGKVVLSEGAKRILKGIRVAAKQRLRAARRSAACKKVRARRALAKFEAIVQSERLDQLET	240
AYQ93055.1	161	SKLRFPKGAVAYNGVNFIDTRGKVVLSEGAKRILKGIRVATKQRLRAARRSAACKKVRATCALATFEAIVQNERLDQLET	240
AYQ93053.1	161	SKLRFPKGAVAYNGVNFIDTRGKVVLSEGAKRILKGIRVATKQRLRAARRSAACKKVRAARALATFEAIVQNERLDQLET	240
AYQ93051.1	161	SKLRFPKGAVAYNGVNFIDTKGKVVLSEGAKRILKGIRVATKQRLRAARRSAACRKVRAARALAKFEAIVQSERLDQLET	240
AFI71813.1	161	SKLQFPKGAVAYNGVNFINTKGKVVLSEGAKRILKGIRVATKQRLRAARRSAACKRVRAVRALAKFEAIVQSERLDQLKT	240
QGP72539.1	161	SKLRFPKGAVAYNGVNFIDTKGKVVLSEGAKRILKGIRVAAKQRLRAARRSAACKKVRAGRALAKFEAIVQSERLDQLET	240
QYU59226.1	161	SKLRFPKGAVAYNGVNFIDTRGKVVLSEGAKRILKGIRVAVKQRLRAARRSAACKKVRAARALAKFEAIAQNERLDQLET	240
ARO69852.1	161	SKLRFPKGAVAYNGVNFIDTKGKVVLSEGAKRILKGIRVATKQRLRAARRSAACKKVRAARALAKFEAIVQSERLDQLDT	240
QGP72543.1	161	SGLRFPKGAVAYNGVNFIDTKGKVVLSEGAKRILRGIRVATKQRLRAARRSAACKKVRAARALAKFEAIVQSERLDQLET	240
QYU59219.1	161	NKLRFPKGAVAYNGVNFIDSRGKVVLSEGAKRILKGIRVATRQRLRAARRFAACKKVRAKRALAIFEAIALNERLDQLST	240
AGT42200.1	161	SKIQFPKGAVAYNGVNFINTKGKVVLSEGAKRILKGIRVATKQRLRAARRSAACKRVRAVRALAKFEAIVQSERLDQLKT	240

```
ACZ58633.1   161   SKLRFPKGAVAYNGVNFIDTRGKVVLSEGAKRILKGVRVAIKQRQRAVRRSAACKKVRAARALAKFEAIVQSERLDQLET  240
QBZ78369.1   161   NKLRFPKGAVAYNGVNFIDTKGKVVLSEGAKRILKGIRVATKQRLRAARRSAACKKVRATRALAKFEAIVQSERLDQLKT  240
ARO69851.1   161   SKLRFPKGAVAYNGVNFIDTKGKVVLSEGAKRILKGIRVAIKQRLRAVRRSAACKKVRAARALANFEAIVQNERLDQLET  240
QYU59220.1   161   GKLRFPKGAVAYNGVNFIDTKGKVVLSEGAKRILKGIRVAAKQRLRAARRSAACKKVRAKRALAKFEAIVQSERLDQLKT  240
AFV34755.1   161   SKLRFPKGAVAYNGVNFIDTKGKVVLSEGAKRILKGIRVATKQRLRAARRSAACKRVRAARALAKFEAIVQNERLDQLKT  240
QGP72544.1   161   SKLRFPKGAIAYNGVNFIDIKGKVVLSEGAKRILKGIRVVTRQRLRAARRTAACKKVRAKRALTQFEAIAQSERLDQLNT  240
QYA72352.1   161   SKLQFPKGAVAYNGVNFIDTKGKVVLSEGAKRILKGIRVATKQRLRATRRSAACKKVRAKRALAKFEAIVQHERLDQLAT  240
ARO69854.1   161   SKLRFPKGAVAYNGVNFIDTKGKVVLSEGAKRILKGIRVATKQRLRAARRSTACKKVRAARALAKFEAIVQSERLDQLET  240
ARO69860.1   161   SKLRFPKGAVAYNGVNFIDTKGKVVLSEGAKRILKGIRVATKQRLRAARRSTACKKVRAARALAKFEAIVQSERLDQLET  240
ARO69863.1   161   SKLRFPKGAVAYNGVNFIDTKGKVVLSEGAKRILKGIRVATKQRLRAARRSTACKKVRAARALAKFEAIVQSERLDQLET  240
DAZ85707.1   161   SKLRFPKGAVAYNGVNFIDTKGKVVLSEGAKRILKGIRVATKQRLRAARRSAACKKVRAARALAKFEAIVQNERLDQLQT  240
AWO67781.1   161   GKLRFPKGAIAYNGVNFIDTKGKVVLSEGAKRILKGIRVATKQRLRAARRSAACKKVRAMRALAEFEAIVQNERLDQLET  240
QBZ78367.1   161   SKLRFPKGAVAYNGVNFIDTRGKVVLSEGAKRILKGVRVAIKQRHRAARRSAACKKVRAARALAKFEAIVQSERLDQLKT  240
ARO69873.1   161   SKLRFPKGAVAYNGVNFIDTKGKVVLSEGAKRILKGIRVATKQRLRAARRSTACKKVRAARALAKFEAIVQSERLDQLET  240
AND76471.1   161   SKLRFPKGAVAYNGVNFIDTKGKVVLSEGAKRILKGIRVATKQRLRAARRSTACKKVRAARALAKFEAIVQNERLDQLET  240
ARO69849.1   161   SKLRFPKGAVAYNGVNFIDTKGKVVLSEGAKRILKGIRVATKQRLRAARRSTACKKVRAARALAKFEAIVQSERLDQLET  240
ARO69889.1   161   SKLRFPKGAVAYNGVNFIDTKGKVVLSEGAKRILKGIRVATKQRLRAARRSTACKKVRAARALAKFEAIVQSERLDQLET  240
ARO69867.1   161   SKLRFPKGAVAYNGVNFIDTKGKVVLSEGAKRILKGIRVATKQRLRAARRSTACKKVRAARALAKFEAIVQSERLDQLET  240
ARO69904.1   161   SKLRFPKGAVAYNGVNFIDTKGKVVLSEGAKRILKGIRVATKQRLRAARRSTACKKVRAARALAKFEAIVQNERLDQLET  240
ARO69869.1   161   SKLRFPKGAVAYNGVNFIDTKGKVVLSEGAKRILKGIRVATKQRLRAARRSTACKKVRAARALAKFEAIVQNERLDQLET  240
ARO69884.1   161   SKLRFPKGAVAYNGVNFIDTKGKVVLSEGAKRILKGIRVATKQRLRAARRSTACKKVRAARALAKFEAIVQSERLDQLET  240
ARO69878.1   161   SKLRFPKGAVAYNGVNFIDTKGKVVLSEGAKRILKGIRVATKQRLRAARRSTACKKVRAARALAKFEAIVQSERLDQLET  240
ARO69885.1   161   SKLRFPKGAVAYNGVNFIDTKGKVVLSEGAKRILKGIRVATKQRLRAARRSTACKKVRAARALAKFEAIVQSERLDQLET  240
ARO69841.1   161   SKLRFPKGAVAYNGVNFIDTKGKVVLSEGAKRILKGIRVATKQRLRAARRSTACKKVRAARALAKFEAIVQNERLDQLET  240
ARO69891.1   161   SKLRFPKGAVAYNGVNFIDTKGKVVLSEGAKRILKGIRVATKQRLRAARRSAACKKVRAARALAKFEAIVQSERLDQLET  240
ARO69868.1   161   SKLRFPKGAVAYNGVNFIDTKGKVVLSEGAKRILKGIRVATKQRLRAARRSAACKKVRAARALAKFEAIVQSERLDQLET  240
ARO69900.1   161   SKLRFPKGAVAYNGVNFIDTKGKVVLSEGAKRILKGIRVATKQRLRAARRSTACKKVRAARALAKFEAIVQNERLDQLET  240
DAZ85706.1   161   SKLRFPKGAVAYNGVNFIDTKGKVVLSEGAKRILKGIRVATKQRLRAARRSAACKKVRAMRALAKFEAIAQNERLDQLQT  240
ARO69899.1   161   SKLRFPKGAVAYNGVNFIDTKGKVVLSEGAKRILKGIRVATKQRLRAARRSAACKKVRAARALAKFEAIVQSERLDQLET  240
ARO69898.1   161   SKLRFPKGAVAYNGVNFIDTKGKVVLSEGAKRILKGIRVATKQRLRAARRSTACKKVRAACALAKFEAIVQSERLDQLET  240
AYA22297.1   161   SRLRFPKGAVAYNGVNFIDTKGKVVLSEGAKRILKGIRVATRQRLRAARRSAACKKVRAARALAKFEAIVQSERLDQLQT  240
QYU59222.1   161   SKLRFPKGAVAYNGVNFIDTNGKVVLSEGAKRILKGIRVATKQRLRAARRSAACKKVRAKRALAKFEAIVQCERLDQLAT  240
ARO69845.1   161   SKLRFPKGAVAYNGVNFIDTKGKVVLSEGAKRILKGIRVATKQRLRAARRSAACKKVRAARALAKFEAIVQSERLDQLET  240
AND76473.1   161   SKLRFPKGAVAYNGVNFIDTKGKVVLSEGAKRILKGIRVATKQRLRAARRSAACKKVRAARALAKFEAIVQSERLDQLET  240
ARO69857.1   161   SKLRFPRGAVAYNGVNFIDTKGKVVLSEGAKRILKGIRVATKQRLRAARRSAACKKVRAARALAKFEAIVQSERLDQLET  240
DAZ85708.1   161   SKLRFPKGAVAYNGVNFIDTRGKVVLSEGAKRILKGIRVATKQRLRAARRSAACKKVRAARALAKFEAIVQSERLDQLET  240
ARO69881.1   161   SKLQFPKGAVAYNGVNFIDTKGKVVLSEGAKRILKGIRVAAKQRLRAARRSAACRKVRAKRALAKFEAIVQHERLDQLMT  240
ARO69893.1   161   SKLQFPKGAVAYNGVNFIDTKGKVVLSEGAKRILKGIRVAAKQRLRAARRSAACRKVRAKRALAKFEAIVQHERLDQLMT  240
ARO69897.1   161   SKLQFPKGAVAYNGVNFIDTKGKVVLSEGAKRILKGIRVATKQRLRAARRSAACRKVRAKRALAKFEAIVQHERLDQLMT  240
ARO69872.1   161   SKLQFPKGAVAYNGVNFIDTKGKVVLSEGAKRILKGIRVATKQRLRAARRSAACRKVRAKRALAKFEAIVQHERLDQLMT  240
ARO69843.1   161   SKLQFPKGAVAYNGVNFIDTKGKVVLSEGAKRILKGIRVAAKQRLRAARRSAACRKVRAKRALAKFEAIVQHERLDQLMT  240
ARO69886.1   161   SKLQFPKGAVAYNGVNFIDTKGKVVLSEGAKRILKGIRVATKQRLRAARRSAACRKVRAKRALAKFEAIVQHERLDQLMT  240
ARO69864.1   161   SKLQFPKGAVAYNGVNFIDTKGKVVLSEGAKRILKGIRVAAKQRLRAARRSAACRKVRAKRALAKFEAIVQHERLDQLMT  240
ARO69876.1   161   SKLQFPKGAVAYNGVNFIDTKGKVVLSEGAKRILKGIRVATKQRLRAARRSAACRKVRAKRALAKFEAIVQHERLDQLMT  240
ARO69888.1   161   SKLQFPKGAVAYNGVNFIDTKGKVVLSEGAKRILKGIRVATKQRLRAARRSAACRKVRAKRALAKFEAIVQHERLDQLMT  240
ARO69853.1   161   SKLQFPKGAVAYNGVNFIDTKGKVVLSEGAKRILKGIRVATKQRLRAARRSAACRKVRAKRALAKFEAIVQHERLDQLMT  240
ARO69903.1   161   SKLQFPKGAVAYNGVNFIDTKGKVVLSEGAKRILKGIRVAAKQRLRAARRSAACRKVRAKRALAKFEAIVQHERLDQLMT  240
ARO69865.1   161   SKLQFPKGAVAYNGVNFIDTKGKVVLSEGAKRILKGIRVATKQRLRAARRSAACRKVRAKRALAKFEAIVQHERLDQLMT  240
ARO69902.1   161   SKLQFPKGAVAYNGVNFIDTKGKVVLSEGAKRILKGIRVATKQRLRAARRSAACRKVRAKRALAKFEAIVQHERLDQLTT  240
ARO69861.1   161   SKLQFPKGAVAYNGVNFIDTKGKVVLSEGAKRILKGIRVATKQRLRAARRSAACRKVRAKRALAKFEAIVQHERLDQLMT  240
ARO69850.1   161   SKLQFPKGAVAYNGVNFIDTKGKVVLSEGAKRILKGIRVATKQRLRAARRSAACRKVRAKRALAKFEAIVQHERLDQLMT  240
QYU59227.1   161   SRLRFPKGAVAYNGVNFIDAKGKVVLSEGAKKILKGIRVATKQRLRAARRSAACKKVRAKRALAIFEAIALNERLDQLST  240
AFM91094.1   161   SKLRFPKGAVAYNGVNYIDIKGKVVLSEGAKRILKGIRVATKQRLRAARRSAACKKVRAKRALATFEAIAQSERLDQLDT  240
ARO69883.1   161   SKLQFPKGAVAYNGVNFIDTKGKVVLSEGAKRILKGICVATKQRLRAARRSAACRKVRAKCALAKFEAIVQHERLDQLMT  240
ARO69879.1   161   SKLQFPKGAVAYNGVNFIDTKGKVVLSEGAKRILKGIRVATKQRLRAARRSAACRKVRAKRALAKFEAIVQHERLDQLMT  240
AND76472.1   161   SKLQFPKGAVAYNGVNFIDTKGKVVLSEGAKRILKGIRVATKQRLRAARRSAACRKVRAKRALAKFEAIVQHERLDQLMT  240
ARO69846.1   161   SKLQFPKGAVAYNGVNFIDTKGKVVLSEGAKRILKGIRVATKQRLRAARRSAACRKVRAKCALAKFEAIVQHERLDQLMT  240
ARO69870.1   161   SKLQFPKGAVAYNGVNFIDTKGKVVLSEGAKRILKGICVATKQRLRAARRSAACRKVRAKCALAKFEAIVQHERLDQLMT  240
ARO69856.1   161   SKLQFPKGAVAYNGVNFIDTKGKVVLSEGAKRILKGICVATKQRLRAARRSAACRKVRAKCALAKFEAIVQHERLDQLMT  240
ARO69847.1   161   SKPRFPKGAVAYNGVNFIDTKGKVVLSEGAKRILKGIRVATKQRLRAARRSAACKKVRAARALAKFEAIVQNERLDQLET  240
ARO69875.1   161   SKPRFPKGAVAYNGVNFIDTKGKVVLSEGAKRILKGIRVATKQRLRAARRSAACKKVRAARALAKFEAIVQSERLDQLDT  240
ARO69895.1   161   SKPRFPKGAVAYNGVNFIDTKGKVVLSEGAKRILKGIRVATKQRLRAARRSAACKKVRAARALAKFEAIVQSERLDQLDT  240
ARO69880.1   161   SKPRFPKGAVAYNGVNFIDTKGKVVLSEGAKRILKGIRVATKQRLRAARRSAACKKVRAARALAKFEAIVQSERLDQLDT  240
BCT87061.1   161   SKLQFPKRAVAYNGVNFVDSMGKVVLSEGAKRILRGIRVAKKQRLRAARRLAACKRVRAARALAKFEAIAQNERLDQLET  240
QYU59225.1   161   SKPQFPKGAVAYNGVNFIDLKGKVVLSEGAKRILKGIRVAKKQRLRAARRSAACRKVRAARALAKFEAIAQNERLDQLST  240
APD26306.1   161   SKLRFPKGAVAYNGVNFIDSRGKVVLSEGAKRILKGIRVATKQRLRAARRSAACKRVRAARALAQFEAIAQTERLDQLRT  240


NP_619689.1  241   GFQVVLPAPKMSCSLKEAAPSTTSVVVVKKRKLPRLPKILPEQDFSCLEGFDWGEKSHPVEVDIEDDWILVEKPVLKRQA  320
AWO68042.1   241   GFQVVLPAPKMSCSLKEAAPSTTSVVVVKKRKLPRLPKILPEQDFSCLEGFDWGEKSHPVEVDIEDDWILVEKPVLKRQA  320
QYU59223.1   241   GFQVVLPAPKMSCSLKEAAPSTTSRVVVKKRKPSKLPKILPEQDFSCLEDFDWGEKSHPVEVDIEDDWILVEKPVLKRQA  320
ARO69887.1   241   GFQVVLPAPKMSCSLKEAAPSTTPVVVVKKRKPLRLPRTLPEQDFSCLEDFDWGEKSHPVEINIEDDWVLVEKPVLSRQA  320
ARO69840.1   241   GFQVVLPAPKMSCSLKEAAPSTTSVVVVKKRRPLRLPRTLPEQDFSCLEDFDWGEKSHPVEINIEDDWVLVEKPVLSRQA  320
ARO69892.1   241   GFQVVLPAPKMSCSLKEAAPSTTPVVVVKKRRPLRLPRTLPEQDFSCLEDFDWGEKSHPVEINIEDDWVLVEKPVLSRQA  320
ARO69862.1   241   GFQVVLPAPKMSCSLKEAAPSTTPVVVVKKRKPLRLPRTLPEQDFSCLEDFDWGEKSHPVEINIEDDWVLVEKPVLSRQA  320
ARO69844.1   241   GFQVVLPAPKMSCSLKEAAPSTTPVVVVKKRRPLRLPRTLPEQDFSCLEDFDWGEKSHPVEINIEDDWVLVEKPVLSRQA  320
ARO69896.1   241   GFQVVLPAPKMSCSLKEAAPSTTPVVVVKKRRPLRLPRTLPEQDFSCLEDFDWGEKSHPVEINIEDDWVLVEKPVLSRQA  320
QGP72541.1   241   GFQVVLPAPKMSCSLKEAAPSTTSVVVVKKRKPSKLPKISPEQDFSCLEDFDWGEKSYPVEVDIEDDWVLVEKPVLKRQA  320
ARO69901.1   241   GFQVVLPAPKMSCSLKEAAPSTTPVVVVKKRRPLRLPRTLPEQDFSCLEDFDWGEKSHPVEINIEDDWVLVEKPVLSRQA  320
AYA22299.1   241   GFQVVLPAPKMGCSLKEAAPSTASVAVVKKRKPSKLPKTLPEQDFSCLEDFDWGEKSHPVEVDIEDDWVLVEKPVLVRRA  320
ARO69890.1   241   GFQVVLPAPKMSCSLKEAAPSTTPVVVVKKRRPLRLPRTLPEQDFSCLEDFDWGEKSHPVEINIEDDWVLVEKPVLSRQA  320
ARO69859.1   241   GFQVVLPAPKMSCSLKEAAPSTTPVVVVKKRRPLRLPRTLPEQDFSCLEDFDWGEKSHPVEINIEDDWVLVEKPVLSRQA  320
ARO69905.1   241   GFQVVLPAPKMSCSLKEAAPSTTPVVVVKKRKPLRLPRTLPEQDFSCLEDFDWGEKSHPVEINIEDDWVLVEKPVLSRQA  320
ARO69871.1   241   GFQVVLPAPKMSCSLKEAAPSTTSVVVVKKRKPTKLPNILPEQDFSCLEDFDWGEKSHPVEINIEDDWVLVEKPVLSRQA  320
ARO69848.1   241   GFQVVLPAPKMSCSLKEAAPSITPVVVVKKRKPLRLPRTLPEQDFSCLEDFDWGEKSHPVEINIEDDWVLVEKPVLSRQA  320
ARO69874.1   241   GFQVVLPAPKMSCSLKEAAPSTTPVVVVKKRKPLRLPRTLPEQDFSCLEDFDWGEKSHPVEINIEDDWVLVEKPVLSRQA  320
ARO69882.1   241   GFQVVLPAPKMSCSLKEAAPSTTSVVVVKKRKPLRLPRTLPEQDFSCLEDFDWGEKSHPVEINIEDDWVLVEKPVLSRQA  320
QYU59221.1   241   GFHVVLPAPKMSCSLKEAAPSTTSVVVVKKRKLPKLPKTLPEQDFSCLEDFDWGEKSHPVEVDVEDDWVFVEKPVLTRQA  320
```

ARO69842.1	241	GFQVVLPAPKMSCSLKEAAPSTTPVVVVKKRKPLRLPRTLPEQDFSCLEDFDWGEKSHPVEINIEDDWVLVEKPVLSRQA	320
AFV34731.1	241	GFQVVLPAPKMGCSLKEAAPSTTSAVVVKKRKPVKLPRVLPEQDFSCLDDFDWGEKSHPVEVDIEDDWVLVEKPVLKRQA	320
AND76470.1	241	GFQVVLPAPKMSCSLKEAAPSTTPVVVVKKRKPLRLPRTLPEQDFSCLEDFDWGEKSHPVEINIEDDWVLVEKPVLSRQA	320
ACZ58632.1	241	GFQVVLPAPKMSCSLKEAAPSTTSTVVVRKRKLSKLPKILPEQDFSCLEGFDWGEKSHPVEADIEDDWVLVEKPVLKRQA	320
QBZ78374.1	241	GFQVVLPAPKMSCGLKKAAPSTTSMVVVKKRKLSKLPKTLPEQDFSCLEGFDWGEKSLPVEANIEDDWVLVEKPVLKRQA	320
QGP72540.1	241	GFQVVLPAPKMSCSLKEAAPSTTSVVVVKKRKPSKLPKISPEQDFSCLEDFDWGEKSYPVEVDIEDDWVLVEKPVLKRQA	320
QBZ78372.1	241	GFQVVLPAPKMSCGLKKAAPSTTSMVVVKKRKLSKLPKTLPEQDFSCLEGFDWGEKSLPVEANIEDDWVLVEKPVLKRQA	320
QYU59224.1	241	GFQVVLPAPKMSCSLREAAPSAASVVAVKKRKLTRLPKILPEQDFSCLEDFDWGEKSHPVEVDIEDDWVLVEKPVLMRQA	320
ARO69877.1	241	GFQVVLPAPKMSCSLKEAAPSTTSVVVVKKRKPTKLPNILPEQDFSCLEGFDWGEKSHPAEVDIEDDWVLVEKPVLLRQA	320
ARO69858.1	241	GFQVVLPAPKMSCSLKEAAPSTTSVVVVKKRKPMKLPNILPEQDFSCLEGFDWGEKSHPAEVDIEDDWVLVEKPVLLRQA	320
ARO69855.1	241	GFQVVLPAPKMSCSLKEAAPSTTPVVVVKKRKPLRLPRTLPEQDFSCLEDFDWGEKSHPVEINIEDDWVLVEKPVLSRQA	320
QGP72542.1	241	GFQVVLPAPKMSCSLRVAAPSTTSAVVVKKRKLLRLPKILPEQDFSCLEGFDWGEKSQLVEADIEDDWVLVEKPVLKRQA	320
AYQ93055.1	241	GFQVVLPAPKMSCSLREAAPSITSVVVVKKRKLPKLPKILPEQDFSCLEDFDWGEKSHPAEVDIEDDWVLVEKPVLKRQA	320
AYQ93053.1	241	GFQVVLPAPKMSCSLREAAPSTTSVVVVKKRKLPKLPKIPPEQDFSCLEDFDWGEKSHPAEVDIEDDWVLVEKPVLKRQA	320
AYQ93051.1	241	GFQVVLPAPKMSCSLREAAPSAASVVAVKKRKLTRLPKILPEQDFSCLEDFDWGEKSHPVEVEIEDDWVLVEKPVLKRQA	320
AFI71813.1	241	GFQVVLPAPKMSCSLREAAPSTTSVVVVKKRKLSKLPKILPEQDFSCLEGFDWGEKSHPVEVDIEDDWVLVEKPVLKRQA	320
QGP72539.1	241	GFQVVLPAPKMSCSSRVAAPSTTSAVVVKKRKLLRLPKILPEQDFSCLEGFDWGEKSQLVEANIEDDWVLAEKPVLKRQA	320
QYU59226.1	241	GFQVVLPAPKMSCNLKEVAPSAASVAVVKKRKLIKLPKILPEQDFSCLEDFDWGEKSLPVEVDIEDDWVLVEKPVLVRQA	320
ARO69852.1	241	GFQVVLPAPKMSCSLKEAAPSTTSVVVVKKRKPMKLPNILPEQDFSCLEGFDWGEKSHPAEVDIEDDWVLVEKPVLLRQA	320
QGP72543.1	241	GFQVVLPAPKMSCSLKEAAPSTTSLVVVKKRKLSKLPKIPPEQDFSCLEGFNWGEKSHPIEVDIEDDWVLIEKPVLKRQA	320
QYU59219.1	241	GFQVVLPAPKMGCNLKEVAPSTASVAVVKKRKPAKLPKAVPEQDFSCLEGFDWGEKSHLVEADIEDDWVLVEKPVLKRQA	320
AGT42200.1	241	GFQVVLPAPKMSCSLREAAPSTTSVVVVKKRKLSKLPKILPEQDFSCLEGFDWGEKSHPVEVDIEDDWVLVEKPVLKRQA	320
ACZ58633.1	241	GFQVVLPAPKMSCSLKEAAPSTTSTVVVRKRKLSKLPKILPEQDFSCLEGFDWGEKSHLVEADIEDDWVLVEKPVLKRRA	320
QBZ78369.1	241	GFQVVLPAPKMSCSLKEAAPSTTSVVVVKKRKLLKLPKILPKQDFSCLEDFDWGEKSHPIEVDIEDDWVLVEKPVLKKQA	320
ARO69851.1	241	GFQVVLPAPKMSCSLKEAAPSTTPVVVVKKRRPLRLPRTLPEQDFSCLEDFDWGEKSHPVEINIEDDWVLVEKPVLSRQA	320
QYU59220.1	241	GFQVVLPAPKMSCSLKEAAPSTAFVAVVKKRKLSKLPKILPEQDFSCLEGFDWGEKSCPVEENIEDDWVLVEKPVLKRQA	320
AFV34755.1	241	GFQVVLPAPKMSCSLEKAAPSTTPMVVVKKRKLVKLPKILPEQDFSCLEDFDWGEKSLPVEVDIEDDWVLVEKPVLMRQA	320
QGP72544.1	241	GFQVVLPAPKMSCSLKEAAPSTAIVAVVKKRKLIKLPKILPEQDFSCLEGFDWGEKSRPVEVDIEDDWVLVEKPVLKRQA	320
QYA72352.1	241	GFQVVLPAPKMSCSLKEAAPSTTSEVVVKKRKLLRLPKILPEQDLSCLEGFDWGEKSHPVEVDIEDDWVLVEKPVLVRQA	320
ARO69854.1	241	GFQVVLPAPKMSCSLKEAAPSTTSVVVVKKRKPTKLPNILPEQDFSCLEGFDWGEKSHPAEVDIEDDWVLVEKPVLLRQA	320
ARO69860.1	241	GFQVVLPAPKMSCSLKEAAPSTTSVVVVKKRKPTKLLNILPEQDFSCLEGFDWGEKSHPAEVDIEDDWVLVEKPVLLRQA	320
ARO69863.1	241	GFQVVLPAPKMSCSLKEAAPSTTSVVVVKKRKPTKLLNILPEQDFSCLEGFDWGEKSHPAEVDIEDDWVLVEKPVLLRQA	320
DAZ85707.1	241	GFQVVLPAPKMSCSLKEAAPSTTSVVVVKKRKLSKLPKILPKQDFSCLEDFDWGEKSHPVEVDIEDDWVLVEKPVLKRQA	320
AWO67781.1	241	GFQVVLPAPKMSCSLKEAAPSTTPAVVVKKRKLLRLPKILPEQDFSCLEDFDWGEKLLPVEEDIEDDWVLIEKPVLKRQA	320
QBZ78367.1	241	GFQVVLPAPKMSCSLREAAPSTTSVVVVKRRKLVKLPKTLPEQDFSCLEDFDWGEKSCPVAVDIEDDWVLVEKPVLRRQA	320
ARO69873.1	241	GFQVVLPAPKMSCSLKEAAPSTTSVVVVKKRKPTKLPNILPEQDFSCLEGFDWGEKSHPAEVDIEDDWVLVEKPVLLRQA	320
AND76471.1	241	GFQVVLPAPKMSCSLKEAAPSTTSVVVVKKRKPTKLPNILPEQDFSCLEGFDWGEKSHPAEVDIEDDWVLVEKPVLLRQA	320
ARO69849.1	241	GFQVVLPAPKMSCSLKEAAPSTTSVVVVKKRKPTKLPNILPEQDFSCLEGFDWGEKSQPAEVDIEDDWVLVEKPVLLRQA	320
ARO69889.1	241	GFQVVLPAPKMSCSLKEAAPSTTSVVVVKKRKPTKLPNILPEQDFSCLEGFDWGEKSHPAEVDIEDDWVLVEKPVLLRQA	320
ARO69867.1	241	GFQVVLPAPKMSCSLKEAAPSTTSVVVVKKRKPTKLPNILPEQDFSCLEGFDWGEKSHPAEVDIEDDWVLVEKPVLLRQA	320
ARO69904.1	241	GFQVVLPAPKMSCSLKEAAPSTTSVVVVKKRKPTKLPNILPEQDFSCLEGFDWGEKSHPAEVDIEDDWVLVEKPVLLRQA	320
ARO69869.1	241	GFQVVLPAPKMSCSLKEAAPSTTSVVVVKKRKPTKLPNILPEQDFSCLEGFDWGEKSHPAEVDIEDDWVLVEKPVLLRQA	320
ARO69884.1	241	GFQVVLPAPKMSCSLKEAAPSTTSVVVVKKRKPTKLPNILPEQDFSCLEGFDWGEKSHPAEVDIEDDWVLVEKPVLLRQA	320
ARO69878.1	241	GFQVVLPAPKMSCSLKEAAPSTTSVVVVKKRKPTKLPNILPEQDFSCLEGFDWGEKSHPAEADIEDDWVLVEKPVLLRQA	320
ARO69885.1	241	GFQVVLPAPKMSCSLKEAAPSTTSVVVVKKRKPTKLPNILPEQDFSCLEGFDWGEKSHPAEVDIEDDWVLVEKPVLLRQA	320
ARO69841.1	241	GFQVVLPAPKMSCSLKEAAPSTTSVVVVKKRKPTKLPNILPEQDFSCLEGFDWGEKSHPAEVDIEDDWVLVEKPVLLRQA	320
ARO69891.1	241	GFQVVLPAPKMSCSLKEAAPSTTFVVVVKKRKPTKLPNILPEQDFSCLEGFDWGEKSHPAEVDIEDDWVLVEKPVLLRQA	320
ARO69868.1	241	GFQVVLPAPKMSCSLKEAAPSTTFVVVVKKRKPTKLPNILPEQDFSCLEGFDWGEKSHPAEVDIEDDWVLVEKPVLLRQA	320
ARO69900.1	241	GFQVVLPAPKMSCSLKEAAPSTTSVVVVKKRKLTKLPNILPEQDFSCLEGFDWGEKSHPAEVDIEDDWVLVEKPVLLRQA	320
DAZ85706.1	241	GFQVVLPAPKMSCSLKEAAPSTTSVVVVKKRKLSKLPKTLPEQDFSCLEDFDWGEKSHPVEVDIEDDWVLVEKPVLKRQA	320
ARO69899.1	241	GFQVVLPAPKMSCSLKEAAPSTTFVVVVKKRKPTKLPNILPEQDFSCLEGFDWGEKSHPAEVDIEDDWVLVEKPVLLRQA	320
ARO69898.1	241	GFQVVLPAPKMSCSLKEAAPSTTSVVVVKKRKPTKLPNILPEQDFSCLEGFDWGEKSHPAEVDIEDDWVLVEKPVLLRQA	320
AYA22297.1	241	GFQVVLPAPKMSCSLKEAAPSTTTVVVVKKRKPVKLPKILSEQDFSCLEDFDWGEKSHSVEVDIEDDWVLVEKPVLLRQA	320
QYU59222.1	241	GFQVVLPAPKMCCSLREAAPSTTFEVVVKKRKLSRLPKNLPEQDFSCLEGFDWGEKSHPVEVNIEDDWVLVEKPVLVRQA	320
ARO69845.1	241	GFQVVLPAPKMSCSLKEAAPSTTFVVVVKKRKPTKLPNILPEQDFSCLEGFDWGEKSHPAEVDIEDDWVLVEKPVLLRQA	320
AND76473.1	241	GFQVVLPAPKMSCSLKEAAPSTTFVVVVKKRKPTKLPNILPEQDFSCLEGFDWGEKSHPAEVDIEDDWVLVEKPVLLRQA	320
ARO69857.1	241	GFQVVLPAPKMSCSLKEAAPSTTFVVVVKKRKPTKLPNILPEQDFSCLEGFDWGEKSHPAEVDIEDDWVLVEKPVLLRQA	320
DAZ85708.1	241	GFQVVLPAPKMSCGLKEAAPSTTSVVVVKKRKLSKLPKTLPEQDFSCLEDFDWGEKSHPVEVDIEDDWILVEKPVLKRQA	320
ARO69881.1	241	GFQVVLPAPKMSCSLKEAAPSTTSEVVVKKRKLSRLPKILPEQDFSCLEGFDWGEKSHPVEVDIEDDWVLVEKPVLVRQA	320
ARO69893.1	241	GFQVVLPAPKMSCSLKEAAPSTTSEVVVKKRKLSRLPKILPEQDFSCLEGFDWGEKSHPVEVDIEDDWVLVEKPVLVRQA	320
ARO69897.1	241	GFQVVLPAPKMSCSLKEAAPSTTSEVVVKKRKLSRLPKILPEQDFSCLEGFDWGEKSHPVEVDIEDDWVLVEKPVLVRQA	320
ARO69872.1	241	GFQVVLPAPKMSCSLKEAAPSTTSEVVVKKRKLSRLPKILPEQDFSCLEGFDWGEKSHPVEVDIEDDWVLVEKPVLVRQA	320
ARO69843.1	241	GFQVVLPAPKMSCSLKEAAPSTTSEVVVKKRKLSRLPKILPEQDFSCLEGFDWGEKSHPVEVDIEDDWVLVEKPVLVRQA	320
ARO69886.1	241	GFQVVLPAPKMSCSLKEAAPSTTSEVVVKKRKLSRLPKILPEQDFSCLEGFDWGEKSHPVEVDIEDDWVLVEKPVLVRQA	320
ARO69864.1	241	GFQVVLPAPKMSCSLKEAAPSTTSEVVVKKRKLSRLPKILPEQDFSCLEGFDWGEKSHPVEVDIEDDWVLVEKPVLVRQA	320
ARO69876.1	241	GFQVVLPAPKMSCSLKEAAPSTTSEVVVKKRKLSRLPKILPEQDFSCLEGFDWGEKSHPVEVDIEDDWVLVEKPVLVRQA	320
ARO69888.1	241	GFQVVLPAPKMSCSLKEAAPSTTSEVVVKKRKLSRLPKILPEQDFSCLEGFDWGEKSHPVEVDIEDDWVLVEKPVLVRQA	320
ARO69853.1	241	GFQVVLPAPKMSCSLKEAAPSTTSEVVVKKRKLSRLPKILPEQDFSCLEGFDWGEKSHPVEVDIEDDWVLVEKPVLVRQA	320
ARO69903.1	241	GFQVVLPAPKMSCSLKEAAPSTTSEVVVKKRKLSRLPKILPEQDFSCLEGFDWGEKSHPVEVDIEDDWVLVEKPVLVRQA	320
ARO69865.1	241	GFQVVLPAPKMSCSLKEAAPSTTSEVVVKKRKLSRLPKILPEQDFSCLEGFDWGEKSHPVEVDIEDDWVLVEKPVLVRQA	320
ARO69902.1	241	GFQVVLPAPKMSCSLKEAAPSTTSEVVVKKRKLSRLPKILPEQDFSCLEGFDWGEKSHPVEVDIEDDWVLVEKPVLVRQA	320
ARO69861.1	241	GFQVVLPAPKMSCSLKEAAPSTTSEVVVKKRKLSRLPKILPEQDFSCLEGFDWGEKSHPVEVDIEDDWVLVEKPVLVRQA	320
ARO69850.1	241	GFQVVLPAPKMSCSLKEAAPSTTSEVVVKKRKLSRLPKILPEQDFSCLEGFDWGEKSHPVEVDIEDDWVLVEKPVLVRQA	320
QYU59227.1	241	GFQVVLPAPKMGCNLKEVAPSTASVAVVKKRKPVKLPKAVPEQDFSCLEGFDWGEKSHLVEADIEDDWVLVEKPVLKRQA	320
AFM91094.1	241	GFQVVLPAPKMSCFLREAAPSTTSVVVVKKRKLLKLPKILPEQDFSCLEDFDWGEKSHPIEVDIEDDWVLVEKPVLKRQA	320
ARO69883.1	241	GFQVVLPAPKMGCSLKEAAPSTTSGVVVKKRKLSRLPKILPEQDFSCLEGFDWGEKSHPVEVDIEDDWVLVEKPVLVRQA	320
ARO69879.1	241	GFQVVLPAPKMSCSLKEAAPSTTSEVVVKKRKLSRLPKILPEQDFSCLEGFDWGEKSHPVEVDIEDDWVLVEKPVLVRQA	320
AND76472.1	241	GFQVVLPAPKMSCSLKEAAPSTTSEVVVKKRKLSRLPKILPEQDFSCLEGFDWGEKSHPVEVDIEDDWVLVEKPVLVRQA	320
ARO69846.1	241	GFQVVLPAPKMSCSLKEAAPSTTSEVVVKKRKLSRLPKILPEQDFSCLEGFDWGEKSHPVEVDIEDDWVLVEKPVLVRQA	320
ARO69870.1	241	GFQVVLPAPKMGCSLKEAAPSTTSGVVVKKRKLSRLPKILPEQDFSCLEGFDWGEKSHPVEVDIEDDWVLVEKPVLVRQA	320
ARO69856.1	241	GFQVVLPAPKMGCSLKEAAPSTTSGVVVKKRKLSRLPKILPEQDFSCLEGFDWGEKSHPVEVDIEDDWVLVEKPVLVRQA	320
ARO69847.1	241	GFQVVLPAPKMSCSLKEAAPSTTSVVVVKKRKPTKLPNILPEQDFSCLEGFDWGEKSHPAEVDIEDDWVLVEKPVLLRQA	320
ARO69875.1	241	GFQVVLPAPKMSCNLKEVAPSTTSVVVVKKRKSMKLPNILPEQDFSCLEGFDWGEKFHPAEVDIEDDWVLVEKPVLLRQA	320
ARO69895.1	241	GFQVVLPAPKMSCNLKEVAPSTTSVVVVKKRKSMKLPNILPEQDFSCLEGFDWGEKFHPAEVDIEDDWVLVEKPVLLRQA	320
ARO69880.1	241	GFQVVLPAPKMSCNLKEVAPSTTSVVVVKKRKSMKLPNILPEQDFSCLEGFDWGEKFHPAEVDIEDDWVLVEKPVLLRQA	320
BCT87061.1	241	GFHVVLPAPKMCCNSKGVAPSTASVAVVKKRKLSKLPKAAPEQDFSCLEGFDWGEKSHPVEVAIEDDWVLVEKPVVIRQA	320
QYU59225.1	241	GFQVVLPAPKMSCNLKEVAPSTASATVVKKRKPTKLPIAVPEQDFSCLEGFDWREKSHPVERDIEDDWVLVEKPVLKRQA	320
APD26306.1	241	GFQVVLPAPKMSCNSKGVAPSTATKAVVKKRKLIKLPKAMPEQDFSCLEDFDWGEKSHSIEVDIEDDWVLVEKPAVTRQA	320

```
NP_619689.1  321  VQTAQGRATEALTRFAATSGFSLGAHQKVEDFASSGEAEYLMAGEFADLCLLSLVYNDAPTLSATIEELRDSKDFLEAIE  400
AWO68042.1   321  VQTAQGRATEALTRFAATSGFSLGAHQKVEDFASSGEAEYLMAGEFADLCLLSLVYNDAPTLSATIEELRDSKDFLEAIE  400
QYU59223.1   321  AQTAQGRATEALTRFAATSGFSLGAHQKVEDFASLGEVEYLMAGEFVDLCLLSLVYNDAPTLSATIGELKDSKDFLEAIE  400
ARO69887.1   321  AQTAQGRATEALTRFAATSGFSLGAHQRVEDFASLGEAEYLMAGEFADLCLLSLVYNDAPTLSATIEELRDNKDFLEAIE  400
ARO69840.1   321  AQTAQGRATEALTRFAATSGFSLGAHQRVEDFASLGEAEYLMAGEFADLCLLSLVYNDAPTLSATIEELRDNKDFLEAIE  400
ARO69892.1   321  AQTAQGRATEALTRFAATSGFSLGAHQRVEDFASLGEAEYLMAGEFADLCLLSLVYNDAPTLSATIEELRDNKDFLEAIE  400
ARO69862.1   321  AQTAQGRATEALTRFAATSGFSLGAHQRVEDFASLGEAEYLMAGEFADLCLLSLVYNDAPTLSATIEELRDNKDFLEAIE  400
ARO69844.1   321  AQTAQGRATEALTRFAATSGFSLGAHQRVEDFASLGEAEYLMAGEFADLCLLSLVYNDAPTLSATIEELRDNKDFLEAIE  400
ARO69896.1   321  AQTAQGRATEALTRFAATSGFSLGAHQRVEDFASLGEAEYLMAGEFADLCLLSLVYNDAPTLSATIEELRDNKDFLEAIE  400
QGP72541.1   321  VQTAQGRATEALTRFAATSGFSLSAHQKVEDFASLGEAEYLMAGEFADLCLLSLVYNDAPALSATIEELGDNKDFQEAIE  400
ARO69901.1   321  AQTAQGRATEALTRFAATSGFSLGAHQRVEDFASLGEAEYLMAGEFADLCLLSLVYNDAPTLSATIEELRDNKDFLEAIE  400
AYA22299.1   321  VQSAQGRATEALTRFAATSGFSLGAHQKVEDFASLGEAEHLMAGEFADLCLLSLVYNDAPTLSATIEELRDSKDFLEAIE  400
ARO69890.1   321  AQTAQGRATEALTRFAATSGFSLGAHQRVEDFASLGEAEYLMAGEFADLCLLSLVYNDAPTLSATIEELRDNKDFLEAIE  400
ARO69859.1   321  AQTAQGRATEALTRFAATSGFSLGAHQRVEDFASLGEAEYLMAGEFADLCLLSLVYNDAPTLSATIEELRDNKDFLEAIE  400
ARO69905.1   321  AQTAQGRATEALTRFAATSGFSLGAHQRVEDFASLGEAEYLMAGEFADLCLLSLVYNDAPTLSATIEELRDNKDFLEAIE  400
ARO69871.1   321  AQTAQGRATEALTRFAATSGFSLGAHQKVEDFASLGEAEYLMAGEFADLCLLSLVYNDAPVLSATIEELGDNKDFLEAIE  400
ARO69848.1   321  AQTAQGRATEALTRFAATSGFSLGAHQRVEDFASLGEAEYLMAGEFADLCLLSLVYNDAPTLSATIEELRDNKDFLEAIE  400
ARO69874.1   321  AQTAQGRATEALTRFAATSGFSLGAHQRVEDFASLGEAEYLMAGEFADLCLLSLVYNDAPTLSATIEELGDNKDFLEAIE  400
ARO69882.1   321  AQTAQGRATEALTRFAATSGFSLGAHQRVEDFASLGEAEYLMAGEFADLCLLSLVYNDAPTLSATIEELGDNKDFLEAIE  400
QYU59221.1   321  AQTVQGRATEALTRFAATSGFSLSAHQKVEDFASLGEAEYLMAGEFADLCLLSLVYNDAPVLSATIEELRDRKDFLEAIE  400
ARO69842.1   321  AQTAQGRATEALTRFAATSGFSLGAHQRVEDFASLGEAEYLMAGEFADLCLLSLVYNDAPTLSATIEELRDNKDFLEAIE  400
AFV34731.1   321  AQTVQGRATEALTRFAATSGFSLGAHQKVEDFASLGEVEYLMAGEFADLCLLSLVYNDAPTLSATIEELRDSKDFQEAIE  400
AND76470.1   321  AQTAQGRATEALTRFAATSGFSLGAHQRVEDFASLGEAEYLMAGEFADLCLLSLVYNDAPTLSATIEELRDNKDFLEAIE  400
ACZ58632.1   321  AQTAQGRATETLTRFAATSGFSLGAHQKVEDFASLGEVEYLMAGEFADLCLLSLVYNDAPTLSATIEELGTKESFLEAIE  400
QBZ78374.1   321  AQTVQGRATEALTRFAATSGFSLGAHQVVEEFASLGEAELLMAGEFADLCLLSLVYNDAPTLSATIEELRGRRDFLEAIE  400
QGP72540.1   321  VQTAQGRATEALTRFAATSGFSLSAHQKVEDFASLGEAEYLMAGEFADLCLLSLVYNDAPVLSATIEELGDNKDFQEAIE  400
QBZ78372.1   321  AQTVQGRATEALTRFAATSGFSLGAHQVVEEFASLGEAELLMAGEFADLCLLSLVYNDAPTLSATIEELRGRRDFLEAIE  400
QYU59224.1   321  AQTVQGRATEALTRFAATSGFSLGAHQKVEDFASLGEVEYLMAGEFADLCLLSLVYNDAPVLSATIEELGDSKDFLEAIE  400
ARO69877.1   321  AQTVQGRATEALTRFAATSGFSLGAHQKVEDFASLGEAEYLMAGEFADLCLLSLVYNDAPVLSATIEELGDSKDFLDAIE  400
ARO69858.1   321  AQTVQGRATEALTRFAATSGFSLGAHQKVEDFASLGEAEYLMAGEFADLCLLSLVYNDAPVLSATIEELGDSKDFLDAIE  400
ARO69855.1   321  AQTAQGRATEALTRFAATSGFSLGAHQRVEDFASLGEAEYLMAGEFADLCLLSLVYNDAPTLSATIEELGDNKDFLEAIE  400
QGP72542.1   321  AQTAQGRATEALTRFAATSGFSLGAHQKVEDFASLGEVEYLMAGEFADLCLLSLVYNDAPTLSATIEELEDIKDFQEAIE  400
AYQ93055.1   321  AQTAQGRATEALTRFAATSGFSLGAHQRVEDFASLGEVECLMAGEFADLCLLSLVYNDAPTLSATIEELRGKKDFLEAIE  400
AYQ93053.1   321  AQTAQGRATEALTRFAATSGFSLGAHQRVEDFASLGEVECLMAGEFADLCLLSLVYNDALTLSATIEELREEKDFLEAIE  400
AYQ93051.1   321  ARTAQGGATEALTRFAATSGFSLGAHQKVEDFASLGEAEYLMAGEFADLCLLSLVYNDAPTLSATIEELGDSKDFLEAIE  400
AFI71813.1   321  AQTAQGRATEALTRFAATSGFSLGAHQKVEGFASLGEEEHLMAGEFADLCLLSLVYNDAPTLSATIEELGESKDFLDAIE  400
QGP72539.1   321  AQTAQGRATEALTRFAATSGFSLGAHQKVEDFASLGEVEYLMAGEFADLCLLSLVYNDAPTLSATIEELEDIKDFQEAIE  400
QYU59226.1   321  AQTVQGRATEALTRFAAASGFSLGAHQKVEDFASLGEVEYQMAGEFADLCLLSLVYNDAPTLSATIEELRDSKDFSEAIE  400
ARO69852.1   321  AQTVQGRATEALTRFAATSGFSLGAHQKVEDFASLGEAEYLMAGEFADLCLLSLVYNDAPVLSATIEELGDSKDFLDAIE  400
QGP72543.1   321  AQTAQGRATEALTRFAATSGFTLDAHQKVEDFASLGEVEYLMAGEFVDLCLLSLVYNDASILSTTIEELRDSKDFLDAIE  400
QYU59219.1   321  AQTVQGRATEALTRFAATSGFSLGAHQKVEGFASLGEAEHLMAGEFADLCLSSLVYNDAPTLSATIEELSDSKDFLEAIE  400
AGT42200.1   321  AQTAQGRATEALTRFAATSDFSLGAHQKVEDFASLGEEEYLLAGEFADLCLLSLVYNDAPTLSATIEELGESKDFLEAIE  400
ACZ58633.1   321  AQTAQGRATETLTRFAATSGFSLGAHQKVEDFASLGQVEYLMAGEFADLCLLFLVYNDAPTLSATIEELGTKESFLEAIE  400
QBZ78369.1   321  AQTVQGRATEALTRFAATSGFSLGAHRKVEGFASLGEVEYLIAGEFADLCLLSLVYNDAPTLSATIEELGDRKDFLDAIE  400
ARO69851.1   321  AQTAQGRATEALTRFAATSGFSLGAHQRVEDFASLGEAEYLMAGEFADLCLLSLVYNDAPTLSATIEELRDNKDFLEAIE  400
QYU59220.1   321  AQTAQGRATEALTRFAATSGFSLGAHQKVEDFASLGEAEYLMAGEFVDLCLSSLVYNDAPVLSTTIEELRSSDDFLEAIE  400
AFV34755.1   321  AQTAQGRATEALTRFAATSGFSLGAHQRVEDFASLGEAEYVMAGEFADLCLLSLVYNDAPTLSATIEELKEIKDFLEAIE  400
QGP72544.1   321  AQTAQGRATEALTRFAATNGFSLGAHQKVEDFASLGEAEYLMAGEFADLCLLSLVYNDAPVLSATIEELRDSQNFLEAIE  400
QYA72352.1   321  AQTAQGRATEALTRFAATSGFSLGAHQKVEDFASLGEVEYLMAGEFADLCLQSLVYNDAPTLSVTIEELGDSKDFLEAVE  400
ARO69854.1   321  AQTVQGRATEALTRFAATSGFSLGAHQKVEDFASLGEAEYLMAGEFADLCLLSLVYNDAPVLSATIEELGGSKDFLDAIE  400
ARO69860.1   321  AQTVQGRATEALTRFAATSGFSLGAHQKVEDFASLGEAEYLMAGEFADLCLLSLVYNDAPVLSATIEELGDSKDFLDAIE  400
ARO69863.1   321  AQTVQGRATEALTRFAATSGFSLGAHQKVEDFASLGEAEYLMAGEFADLCLLSLVYNDAPVLSATIEELGDSKDFLDAIE  400
DAZ85707.1   321  AQTAQGRATEALTRFAATSGFSLGAHQKVEDFASSGEAEYLVAGEFADLCLSSLVYNDAPMLSATIEELSDHKDFLDAIE  400
AWO67781.1   321  AQTVQGRATEALTRFAATSGFSLGAHQKVEDFASLGEVEYLVAGEFADLCLQSLVYNDAPTLSATIEELGDSKDFLEAIE  400
QBZ78367.1   321  AQTVQGRATEALTRFAATSGFSLGAHQRVEDFASLGEVEYLMAGEFVDLCLLSLVYNDAPTLSTTIEDLRDSKDFLEAIE  400
ARO69873.1   321  AQTVQGRATEALTRFAATSGFSLGAHQKVEDFASLGEAEYLMAGEFADLCLLSLVYNDAPVLSATIEELGDSKDFLDAIE  400
AND76471.1   321  AQTVQGRATEALTRFAATSGFSLGAHQKVEDFASLGEAEYLMAGEFADLCLLSLVYNDAPVLSATIEELGNSKDFLDAIE  400
ARO69849.1   321  AQTVQGRATEALTRFAATSGFSLGAHQKVEDFASLGEAEYLMAGEFADLCLLSLVYNDAPVLSATIEELGDSKDFLDAIE  400
ARO69889.1   321  AQTVQGRATEALTRFAATSGFSLGAHQKVEDFASLGEAEYLMAGEFADLCLLSLVYNDAPVLSATIEELGGSKDFLDAIE  400
ARO69867.1   321  AQTVQGRATEALTRFAATSGFSLGAHQKVEDFASLGEAEYLMAGEFADLCLLSLVYNDAPVLSATIEELGGSKDFLDAIE  400
ARO69904.1   321  AQTVQGRATEALTRFAATSGFSLGAHQKVEDFASLGEAEYLMAGEFADLCLLSLVYNDAPVLSATIEELGDSKDFLDAIE  400
ARO69869.1   321  AQTVQGRATEALTRFAATSGFSLGAHQKVEDFASLGEAEYLMAGEFADLCLLSLVYNDAPVLSATIEELGDSKDFLDAIE  400
ARO69884.1   321  AQTVQGRATEALTRFAATSGFSLGAHQKVEDFASLGEAEYLMAGEFADLCLLSLVYNDAPVLSATIEELGGSRDFLDAIE  400
ARO69878.1   321  AQTVQGRATEALTRFAATSGFSLGAHQKVEDFASLGEAEYLMAGEFADLCLLSLVYNDAPVLSATIEELGDSKDFLDAIE  400
ARO69885.1   321  AQTVQGRATEALTRFAATSGFSLGAHQKVEDFASLGEAEYLMAGEFADLCLLSLVYNDAPVLSATIEELGGSKDFLDAIE  400
ARO69841.1   321  AQTVQGRATEALTRFAATSGFSLGAHQKVEDFASLGEAEYLMAGEFADLCLLSLVYNDAPVLSATIEELGGSKDFLDAIE  400
ARO69891.1   321  AQTVQGRATEALTRFAATSGFSLGAHRKVEDFASLGEAEYLMAGEFADLCLLSLVYNDAPVLSATIEELGDSKDFLDAIE  400
ARO69868.1   321  AQTVQGRATEALTRFAATSGFSLGAHQKVEDFASLGEAEYLMAGEFADLCLLSLVYNDAPVLSATIEELGDSKDFLDAIE  400
ARO69900.1   321  AQTVQGRATEALTRFAATSGFSLGAHQKVEDFASLGEAEYLMAGEFADLCLLSLVYNDAPVLSATIEELGGSKDFLDAIE  400
DAZ85706.1   321  AQTVQGRATEALTRFAATSGFSLGAHQKVEDFASLGEAEYLMAGEFADLCLLSLVYNDAPTLSATIEELRESNDFLEAIE  400
ARO69899.1   321  AQTVQGRATEALTRFAATSGFSLGAHRKVEDFASLGEAEYLMAGEFADLCLLSLVYNDAPVLSATIEELGDSKDFLDAIE  400
ARO69898.1   321  AQTVQGRATEALTRFAATSGFSLGAHQKVEDFASLGEAEYLMAGEFADLCLLSLVYNDAPVLSATIEELGGSKDFLDAIE  400
AYA22297.1   321  AQTAQGRATEALTRFAATSGFSLGAHQKVEGFASLGGAEYLMAGEFADLCLSSLVYNDAPTLSATIEELGDNKDFQEAIE  400
QYU59222.1   321  AQTVQGRATEALTRFAATSGFSLGAHQKVEDFASLGEAEYLMAGEFADLCLLSLVYNDAPTLSATIGELKESKDFLEAIE  400
ARO69845.1   321  AQTVQGRATEALTRFAATSGFSLGAHQKVEDFASLGEAEYLMAGEFADLCLLSLVYNDAPVLSATIEELGDSKDFLDAIE  400
AND76473.1   321  AQTVQGRATEALTRFAATSGFSLGAHRKVEDFASLGEAEYLMAGEFADLCLLSLVYNDAPVLSATIEELGDSKDFLDAIE  400
ARO69857.1   321  AQTVQGRATEALTRFAATSGFSLGAHRKVEDFASLGEAEYLMAGEFADLCLLSLVYNDAPVLSATIEELGDSKDFLDAIE  400
DAZ85708.1   321  AQTAQGRATEALTRFAATSGFSLGAHQKVEDFASLGEVEYLMAGEFADLCLLSLVYNDAPTLSATIEELKESKDFLEAIE  400
ARO69881.1   321  AQTAQGRATEALTRFAATSDFSLGAHQKVEDFASLGEVEYSIAGEFADLCLQSLVYNDAPTLSATIEELGDSKDFLEAVE  400
ARO69893.1   321  AQTAQGRATEALTRFAATSDFSLGAHQKVEDFASLGEVEYSIAGEFADLCLQSLVYNDAPTLSATIEELGDSKDFLEAVE  400
ARO69897.1   321  AQTAQGRATEALTRFAATSDFSLGAHQKVEDFASLGEVEYSIAGEFADLCLQSLVYNDAPTLSATIEELGDSKDFLEAVE  400
ARO69872.1   321  AQTAQGRATEALTRFAATSDFSLGAHQKVEDFASLGEVEYSIAGEFADLCLQSLVYNDAPTLSATIEELGDSKDFLEAVE  400
ARO69843.1   321  AQTAQGRATEALTRFAATSDFSLGAHQKVEDFASLGEVEYSIAGEFADLCLQSLVYNDAPTLSATIEELGDSKDFLEAVE  400
ARO69886.1   321  AQTAQGRATEALTRFAATSGFSLGAHQKVEDFASLGEVEYSIAGEFADLCLQSLVYNDAPTLSATIEELGDSKDFLEAVE  400
ARO69864.1   321  AQTAQGRATEALTRFAATSDFSLEAHQKVEDFASLGEVEYSIAGEFADLCLQSLVYNDAPTLSATIEELGDSKDFLEAVE  400
ARO69876.1   321  AQTAQGRATEALTRFAATSDFSLGAHQKVEDFASLGEVEYSIAGEFADLCLQSLVYNDAPTLSATIEELGDSKDFLEAVE  400
ARO69888.1   321  AQTAQGRATEALTRFAATSGFSLGAHQKVEDFASLGEVEHSIAGEFADLCLQSLVYNDAPTLSATIEELGDSKDFLEAVE  400
```

```
ARO69853.1   321   AQTAQGRATEALTRFAATSDFSLGAHQKVEDFASLGEVEYSIAGEFADLCLQSLVYNDAPTLSATIEELGDSKDFLEAVE  400
ARO69903.1   321   AQTAQGRATEALTRFAATSDFSLGAHQKVEDFASLGEVEYSIAGEFADLCLQSLVYNDAPTLSATIEELGDSKDFLEAVE  400
ARO69865.1   321   AQTAQGRATEALTRFAATSDFSLGAHQKVEDFASLGEVEYSIAGEFADLCLQSLVYNDAPTLSATIEELGDSKDFLEAVE  400
ARO69902.1   321   AQTAQGRATEALTRFAATSDFSLGAHQKVEDFASLGEVEYSIAGEFADLCLQSLVYNDAPTLSATIEELGDSKDFLEAVE  400
ARO69861.1   321   AQTAQGRATEALTRFAATSDFSLGAHQKVEDFASLGEVEYSIAGEFADLCLQSLVYNDAPTLSATIEELGDSKDFLEAVE  400
ARO69850.1   321   AQTAQGRATEALTRFAATSDFSLGAHQKVEDFASLGEVEYSIAGEFADLCLQSLVYNDAPTLSATIEELGDSKDFLEAVE  400
QYU59227.1   321   AQTVQGRATEALTRFAATSGFSLGAHQKVEGFASLGEAEHLMAGEFADLCLSSLVYNDAPTLSATIEELSDSKDFLEAIE  400
AFM91094.1   321   AQTARGRATEALTRFAATSGFSLGAHQKVEDFASLGEVEYLMAGEFADLCLQSLVYNDAPTLSATIEELGDSKDFLEAVE  400
ARO69883.1   321   AQTAQGRATEALTRFAATSDFSLGAHQKVEDFASLGEVEYSIAGEFADLCLQSLVYNDAPTLSATIEELGDSKDFLEAVE  400
ARO69879.1   321   AQTAQGRATEALTRFAATSDFSLGAHQKVEDFASLGEVEYSIAGEFADLCLQSLVYNDAPTLSATIEELGDSKDFLEAVE  400
AND76472.1   321   AQTAQGRATEALTRFAATSDFSLGAHQKVEDFASLGEVEYSIAGEFADLCLQSLVYNDAPTLSATIEELGDSKDFLEAVE  400
ARO69846.1   321   AQTAQGRATEALTRFAATSDFSLEAHQKVEDFASLGEVEYSIAGEFADLCLQSLVYNDAPTLSATIEELGDSKDFLEAVE  400
ARO69870.1   321   AQTAQGRATEALTRFAATSDFSLGAHQKVEDFASLGEVEYSIAGEFADLCLQSLVYNDAPTLSATIEELGDSKDFLEAVE  400
ARO69856.1   321   AQTAQGRATEALTRFAATSDFSLGAHQKVEDFASLGEVEYSIAGEFADLCLQSLVYNDAPTLSATIEELGDSKDFLEAVE  400
ARO69847.1   321   AQTVQGRATEALTRFAATSGFSLGAHQKVEDFASLGEAEYLMAGEFADLCLLSLVYNDAPVLSATIEELGDSKDFLDAIE  400
ARO69875.1   321   AQTVQGRATEALTRFAATSGFSLGAHQKVEDFASLGEAEYLMAGEFADLCLLSLVYNDAPVLSATIEELGDSKDFLDAIE  400
ARO69895.1   321   AQTVQGRATEALTRFAATSGFSLGAHQKVEDFASLGEAEYLMAGEFADLCLLSLVYNDAPVLSATIEELGDSKDFLDAIE  400
ARO69880.1   321   AQTVQGRATEALTRFAATSGFSLGAHQKVEDFASLGEAEYLMAGEFADLCLLSLVYNDAPVLSATIEELGDSKDFLDAIE  400
BCT87061.1   321   AQTVQGRAAEALTRFSATSGFSLGAHQKVENFASLGEAEFLVAGEFVDLCLKSLVYNDAPVLSTTIEELEGINDFHDAIE  400
QYU59225.1   321   AQTVQGRATEALTRFAATSGFSLGAHQKVEDFASLGEAESLVAGEFADLCLLSLVYNDAPTLSATIEELRDSKDFLEAIE  400
APD26306.1   321   AQSVQGRATEALTRFAATSGFSLDAHRKVEEFASLGEAERLMAGEFVDLCLKSLVYNDAPILSTTIEELTGKEDFQNAIE  400
```

X1-X2 cleavage site

```
NP_619689.1  401   LLKLELAEIPTDSTTCAPFKQWASAAKQMAKGVGTMVGDFTRAAGAAVVISFDMAVEFLQDKALKFCKRIFDVTMAPYLQ  480
AWO68042.1   401   LLKLELAEIPTDSTTCAPFKQWASAAKQMAKGVGTMVGDFTRAAGAAVVISFDMAVEFLQDKALKFCKRIFDVTMAPYLQ  480
QYU59223.1   401   LLKLELAEIPTDSTTCAPFRQWASAAKQMAKGVGTMVGDFTRAAGAAVIISFDMAVEFLQDKALKFCKRIFDVTMAPYLQ  480
ARO69887.1   401   LLKLELAEIPTDSTTCAPFRQWASAAKQMAKGVGTMVGDFTRAAGAAVVISFDMAVEFLQDKALKFCKKIFDVTMAPYLQ  480
ARO69840.1   401   LLKLELAEIPTDSTTCAPFRQWASAAKQMAKGVGTMVGDFTRAAGAAVVISFDMAVEFLQDKALKFCKKIFDVTMAPYLQ  480
ARO69892.1   401   LLKLELAEIPTDSTTCAPFRQWASAAKQMAKGVGTMVGDFTRAAGAAVVISFDMAVEFLQDKALKFCKKIFDVTMAPYLQ  480
ARO69862.1   401   LLKLELAEIPTDSTTCAPFRQWASAAKQMAKGVGTMVGDFTRAAGAAVVISFDMAVEFLQDKALKFCKKIFDVTMAPYLQ  480
ARO69844.1   401   LLKLELAEIPTDSTTCAPFRQWASAAKQMAKGVGTMVGDFTRAAGAAVVISFDMAVEFLQDKALKFCKKIFDVTMAPYLQ  480
ARO69896.1   401   LLKLELAEIPTDSTTCAPFRQWASAAKQMAKGVGTMVGDFTRAAGAAVVISFDMAVEFLQDKALKFCKKIFDVTMAPYLQ  480
QGP72541.1   401   LLKLELAEIPTDSITCAPFRQWASAAKQMAKGVGTMVGDFTRAAGAAVIISFDMAVEFLQDKALKFCKRIFDVTMAPYLQ  480
ARO69901.1   401   LLKLELAEIPTDSTTCAPFRQWASAAKQMAKGVGTMVGDFTRAAGAAVVISFDMAVEFLQDKALKFCKKIFDVTMAPYLQ  480
AYA22299.1   401   LLKLELAEIPTDSTTCAPFRQWASAAKQMAKGVGTMVGDFTRAAGAAVVISFDMAVEFLQDKALKFCKKIFDVTMAPYLQ  480
ARO69890.1   401   LLKLELAEIPTDSTTCAPFRQWASAAKQMAKGVGTMVGDFTRAAGAAVVISFDMAVEFLQDKALKFCKKIFDVTMAPYLQ  480
ARO69859.1   401   LLKLELAEIPTDSTTCAPFRQWASAAKQMAKGVGTMVGDFTRAAGAAVVISFDMAVEFLQDKALKFCKKIFDVTMAPYLQ  480
ARO69905.1   401   LLKLELAEIPTDSTTCAPFRQWASAAKQMAKGVGTMVGDFTRAAGAAVVISFDMAVEFLQDKALKFCKKIFDVTMAPYLQ  480
ARO69871.1   401   LLKLELAEIPTDSTTCAPFRQWASAAKQMAKGVGTMVGDFTRAAGAAVVISFDMAVEFLQDKALKFCKKIFDVTMAPYLQ  480
ARO69848.1   401   LLKLELAEIPTDSTTCAPFKQWASAAKQMAKGVGTMIGDFTRAAGAAAIISFDMAVEFLQDKALKFCKRIFDVTMAPYLQ  480
ARO69874.1   401   LLKLELAEIPTDSTTCAPFRQWASAAKQMAKGVGTMVGDFTRAAGAAVVISFDMAVEFLQDKALKFCKKIFDVTMAPYLQ  480
ARO69882.1   401   LLKLELAEIPTDSTTCAPFRQWASAAKQMAKGVGTMVGDFTRAAGAAVVISFDMAVEFLQDKALKFCKKIFDVTMAPYLQ  480
QYU59221.1   401   LLKLELAEIPTDSTTCAPFKQWAAAAKQMAKGVGAMVGDFTRAAGAAVIISFDMAVEFLQDKALKFCKKIFDVTMAPYLQ  480
ARO69842.1   401   LLKLELAEIPTDSTTCAPFRQWASAAKQMAKGVGTMVGDFTRAAGAAVVISFDMAVEFLQDKALKFCKKIFDVTMAPYLQ  480
AFV34731.1   401   LLKLELAEIPTDSTTCAPFKQWASAAKQMAKGVGTMVGDFTRAAGAAVIISFDMAVEFLQDKALKFCKRIFDVTMAPYLQ  480
AND76470.1   401   LLKLELAEIPTDSTTCAPFRQWASAAKQMAKGVGTMVGDFTRAAGAAVVISFDMAVEFLQDKALKFCKKIFDVTMAPYLQ  480
ACZ58632.1   401   LLKLELAEIPTDSTTCAPFKQWASAAKQMAKGVGTMVGDFTRAAGAAVIISFDMAVEFLQDKALKFCKKIFDVTMAPYLQ  480
QBZ78374.1   401   LLKLELAEIPTDSTTCAPFRQWASAAKQMAKGVGAMVGDFTRAAGAAVIISFDMAVEFLQDKALKFCKRIFDVTMAPYLQ  480
QGP72540.1   401   LLKLELAEIPTDSITCAPFRQWASAAKQMAKGVGTMVGDFTRAAGAAVVISFDMAVEFLQDKALKFCKRIFDVTMAPYLQ  480
QBZ78372.1   401   LLKLELAEIPTDSTTCAPFRQWASAAKQMAKGVGAMVGDFTRAAGAAVIISFDMAVEFLQDKALKFCKRIFDVTMAPYLQ  480
QYU59224.1   401   LLKLELAEIPTDSTTCAPFKQWASAAKQMAKGVGTMVGDFARAAGAAAIISFDMAVEFLQDKALKFCKKIFDVTMAPYLQ  480
ARO69877.1   401   LLKLELAEIPMDSTTCAPFKQWASAAKQMAKGVGTMVGDFARAAGAAAIISFDMAVEFLQDKALKFCKKIFDVTMAPYLQ  480
ARO69858.1   401   LLKLELAEIPMDSTTCAPFKQWASAAKQMAKGVGTMVGDFARAAGAAAIISFDMAVEFLQDKALKFCKKIFDVTMAPYLQ  480
ARO69855.1   401   LLKLELAEIPTDSTTCAPFRQWASAAKQMAKGVGAMVGDFTRAAGAAVVISFDMAVEFLQDKALKFCKKIFDVTMAPYLQ  480
QGP72542.1   401   LLKLELAEVPTDSTTCAPFKQWASAAKQMAKGVGTMVGDFTRAAGAAVVISFDMAVEFLQDKALKFCKRIFDVTMAPYLQ  480
AYQ93055.1   401   LLKLELAEIPTDSITCAPFKQWASAAKQMAKGVGTMVGDFARAAGAAAIISFDMAVEFLQDKALKFCKRIFDVTMAPYLQ  480
AYQ93053.1   401   LLKLELAEIPTDSTTCAPFKQWASAAKQMAKGVGAMVGDFTRAAGAAVIISFDMAVEFLQDKALKFCKKIFDVTMAPYLQ  480
AYQ93051.1   401   LLKLELAEIPTDSITCAPFKQWASAAKQMAKGVGTMIGDFTRAAGAAVVISFDMAVEFLQDKALKFCKKIFDVTMAPYLQ  480
AFI71813.1   401   LLKLELAEIPTDSTTCAPFRQWASAAKQMAKGVGTMVGDFTRAAGAAVVISFDMAVEFLQDKALKFCKRIFDVTMAPYLQ  480
QGP72539.1   401   LLKLELAEVPTDSTTCAPFKQWASAAKQMAKGVGTMVGDFTRAAGAAVVISFDMAVEFLQDKALKFCKRIFDVTMAPYLQ  480
QYU59226.1   401   LLKLELAEIPTDSTTCAPFRQWASAAKQMAKGVGTMVGNFTRAAGAAVVISFDMAVEFLQDKALKFCKKIFDVTMAPYLQ  480
ARO69852.1   401   LLRLELAEIPTDSTTCAPFKQWASATKQMAKGVGTMVGDFVRAAGAAAIISFDMAVEFLQDKALKFCKKIFDVTMAPYLQ  480
QGP72543.1   401   LLKLELAEIPTDSTTCAPFRQWASAAKQMAKGIGTMVGDFSRAAGAAAIITFDMAVEFLQDKALKFCKRIFDVTMAPYLQ  480
QYU59219.1   401   LLKLELAEIPMDSTTCAPFKQWASVAKQMAIGVGTMVGDFTRAAGAAAIISFDMAVEFLQDKALKFCKKIFDVTMAPYLQ  480
AGT42200.1   401   LLKLELAEIPTDSTTCAPFRQWASAAKQMAKGVGTMVGDFTRAAGAAVVISFDMAVEFLQDKALKFCKRIFDVTMAPYLQ  480
ACZ58633.1   401   LLKLELAEIPTDSTTCAPFKQWASAAKQMAKGVGTMVGDFTRAAGAAAIISFDMAIEFLQDKALKFCKKIFDVTMAPYLQ  480
QBZ78369.1   401   LLRLELAEIPTDSTMCAPFKQWASAAKQMARGVGTMVGDFTRAAGAAVIISFDMAVEFLQDKALKFCKRIFDVTMAPYLQ  480
ARO69851.1   401   LLKLELAEIPTDSTTCAPFRQWASAAKQMAKGVGTMVGDFTRAAGAAVVISFDMAVEFLQDKALKFCKKIFDVTMAPYLQ  480
QYU59220.1   401   LLKLELAEIPTDSTTCAPFKQWASAAKQMAKGVGTMVGDFTRAAGAAVIISFDMAVEFLQDKALKFCKKIFDVTMAPYLQ  480
AFV34755.1   401   LLKLELAEIPTDSITCAPFRQWASAAKQMAKGVGTMVGDFTRAAGAAVIISFDMAVEFLQDKALKFCKRIFDVTMAPYLQ  480
QGP72544.1   401   LLKLELAEIPTDSTTCAPFKQWASAAKQMAKGVGTMVGDFTRAAGAAVIISFDMAVEFLQDKALKFCKKIFDVTMAPYLQ  480
QYA72352.1   401   LLKLELAEIPTDSITCAPFRQWASAAKQMAKGVGAMVGDFTRAAGAAVVISFDMAVEFLQDKALKFCKRIFDVTMAPYLQ  480
ARO69854.1   401   LLKLELAEIPMDSTTCAPFKQWASAAKQMAKGVGTMVGDFARAAGAAAIISFDMAVEFLQDKALKFCKKIFDVTMAPYLQ  480
ARO69860.1   401   LLKLELAEIPMDSTTCAPFKQWASAAKQMAKGVGTMVGDFARAAGAAAIISFDMAVEFLQDKALKFCKKIFDVTMAPYLQ  480
ARO69863.1   401   LLKLELAEIPMDSTTCAPFKQWASAAKQMAKGVGTMVGDFARAAGAAAIISFDMAVEFLQDKALKFCKKIFDVTMAPYLQ  480
DAZ85707.1   401   LLKLELAEIPTDSTTCAPFRQWAFAAKQMAKGVGTMVGDFTRAAGAAVIISFDMAVEFLQDKALKFCKRIFDVTMAPYLQ  480
AWO67781.1   401   LLKLELAEIPTDSTTCAPFRQWASAAKQMAKGVGTMVGDFTRAAGAAVIISFDMAVEFLQDKALKFCKKIFDVTMAPYLQ  480
QBZ78367.1   401   LLKLELTEIPTDSITCAPFRQWASAAKQMAKGVGTMVGDFARAAGAAAIISFDMAIEFLQDKALKFCKKIFDVTMAPYLQ  480
ARO69873.1   401   LLKLELAEIPMDSTTCAPFKQWASAAKQMAKGVGTMVGDFARAAGAAAIISFDMAVEFLQDKALKFCKKIFDVTMAPYLQ  480
AND76471.1   401   LLKLELAEIPTDSTTCAPFKQWASAAKQMAKGVGTMVGDFARAAGAAAIISFDMAVEFLQDKALKFCKKIFDVTMAPYLQ  480
ARO69849.1   401   LLKLELAEIPMDSTTCAPFKQWASAAKQMAKGVGTMVGDFARAAGAAAIISFDMAVEFLQDKALKFCKKIFDVTMAPYLQ  480
ARO69889.1   401   LLKLELAEIPMDSTTCAPFKQWASAAKQMAKGVGTMVGDFARAAGAAAIISFDMAVEFLQDKALKFCKKIFDVTMAPYLQ  480
ARO69867.1   401   LLKLELAEIPMDSTTCAPFKQWASAAKQMAKGVGTMVGDFARAAGAAAIISFDMAVEFLQDKALKFCKKIFDITMAPYLQ  480
ARO69904.1   401   LLKLELAEIPMDSTTCAPFKQWASAAKQMAKGVGTMVGDFARAAGAAAIISFDMAVEFLQDKALKFCKKIFDVTMAPYLQ  480
ARO69869.1   401   LLKLELAEIPMDSTTCAPFKQWASAAKQMAKGVGTMVGDFARAAGAAAIISFDMAVEFLQDKALKFCKKIFDVTMAPYLQ  480
ARO69884.1   401   LLKLELAEIPMDSTTCAPFKQWASAAKQMAKGVGTMVGDFARAAGAAAIISFDMAVEFLQDKALKFCKKIFDVTMAPYLQ  480
```

```
ARO69878.1   401   LLKLELAEIPMDSTTCAPFKQWASAAKQMAKGVGTMVGDFARAAGAAAIISFDMAVEFLQDKALKFCKKIFDVTMAPYLQ  480
ARO69885.1   401   LLKLELAEIPMDSTTCAPFKQWASAAKQMAKGVGTMVGDFARAAGAAAIISFDMAVEFLQDKALKFCKKIFDITMAPYLQ  480
ARO69841.1   401   LLKLELAEIPMDSTICAPFKQWASAAKQMAKGVGTMVGDFARAAGAAAIISFDMAVEFLQDKALKFCKKIFDVTMAPYLQ  480
ARO69891.1   401   LLKLELAEIPTDSITCAPFKQWASAAKQMAKGVGTMIGDFTRAAGAAAIISFDMAVEFLQDKALKFCKKIFDVTMAPYLQ  480
ARO69868.1   401   LLKLELAEIPTDSITCAPFKQWASAAKQMAKGVGTMIGDFTRAAGAAAIISFDMAVEFLQDKALKFCKKIFDVTMAPYLQ  480
ARO69900.1   401   LLKLELAEIPMDSTTCAPFKQWASAAKQMAKGVGTMVGDFARAAGAAAIISFDMAVEFLQDKALKFCKKIFDVTMAPYLQ  480
DAZ85706.1   401   LLKLELAEIPTDSTTCAPFKQWASAAKQMAKGVGTMVGDFARAAGAAAIISFDMAVEFLQDKALKFCKKIFDITMAPYLQ  480
ARO69899.1   401   LLKLELAEIPTDSITCAPFKQWASAAKQMAKGVGTMIGDFTRAAGAAAIISFDMAVEFLQDKALKFCKKIFDVTMAPYLQ  480
ARO69898.1   401   LLKLELAEIPMDSTTCAPFKQWASAAKQMAKGVGTMVGDFARAAGAAAIISFDMAVEFLQDKALKFCKKIFDVTMAPYLQ  480
AYA22297.1   401   LLKLELAEIPTDSTTCAPFKQWASAAKQMAKGVGTMVGDFARAAGAAAIISFDMAVEFLQDKALKFCKKIFDVTMAPYLQ  480
QYU59222.1   401   LLKLELAEIPVESTTCAPFKQWASAAKQMAKGVGTMVGDFTRAAGAAVIISFDMAVEFLQDKALKFCKRIFDVTMAPYLQ  480
ARO69845.1   401   LLKLELAEIPTDSITCAPFKQWASAAKQMAKGVGTMVGDFARAAGAAAIISFDMAVEFLQDKALKFCKKIFDVTMAPYLQ  480
AND76473.1   401   LLKLELAEIPTDSITCAPFKQWASAAKQMAKGVGTMIGDFARAAGAAAIISFDMAVEFLQDKALKFCKKIFDVTMAPYLQ  480
ARO69857.1   401   LLKLELAEIPTDSITCAPFKQWASAAKQMAKGVGTMIGDFARAAGAAAIISFDMAVEFLQDKALKFCKKIFDVTMAPYLQ  480
DAZ85708.1   401   LLKLELAEIPTDSTTCAPFKRWASAAKQMAKGVGTMVGDFTRAAGAAVIISFDMAVEFLQDKALKFCKKIFDVTMAPYLQ  480
ARO69881.1   401   LLKLELAEIPTDSTTCAPFKQWASAAKQMAKGVGTMIGDFTRAAGAAAIISFDMAVEFLQDKALKFCKRIFDVTMAPYLQ  480
ARO69893.1   401   LLKLELAEIPTDSTTCAPFKQWASAAKQMAKGVGTMIGDFTRAAGAAAIISFDMAVEFLQDKALKFCKRIFDVTMAPYLQ  480
ARO69897.1   401   LLKLELAEIPTDSTTCAPFKQWASAAKQMAKGVGTMIGDFTRAAGAAAIISFDMAVEFLQDKALKFCKRIFDVTMAPYLQ  480
ARO69872.1   401   LLKLELAEIPTDSTTCAPFKQWASAAKQMAKGVGTMIGDFTRAAGAAAIISFDMAVEFLQDKALKFCKRIFDVTMAPYLQ  480
ARO69843.1   401   LLKLELAEIPTDSTTCAPFKQWASAAKQMAKGVGTMIGDFTRAAGAAAIISFDMAVEFLQDKALKFCKRIFDVTMAPYLQ  480
ARO69886.1   401   LLKLELAEIPTDSTTCAPFKQWASAAKQMAKGVGTMIGDFTRAAGAAAIISFDMAVEFLQDKALKFCKRIFDVTMAPYLQ  480
ARO69864.1   401   LLKLELAEIPTDSTTCAPFKQWASAAKQMAKGVGTMIGDFTRAAGAAAIISFDMAVEFLQDKALKFCKRIFDVTMAPYLQ  480
ARO69876.1   401   LLKLELAEIPTDSTTCAPFKQWASAAKQMAKGVGTMIGDFTRAAGAAAIISFDMAVEFLQDKALKFCKRIFDVTMAPYLQ  480
ARO69888.1   401   LLKLELAEIPTDSTTCAPFKQWASAAKQMAKGVGTMIGDFTRAAGAAAIISFDMAVEFLQDKALKFCKRIFDVTMAPYLQ  480
ARO69853.1   401   LLKLELAEIPTDSTTCAPFKQWASAAKQMAKGVGTMIGDFTRAAGAAAIISFDMAVEFLQDKALKFCKRIFDVTMAPYLQ  480
ARO69903.1   401   LLKLELAEIPTDSTTCAPFKQWASAAKQMAKGVGTMIGDFTRAAGAAAIISFDMAVEFLQDKALKFCKRIFDVTMAPYLQ  480
ARO69865.1   401   LLKLELAEIPTDSTTCAPFKQWASAAKQMAKGVGTMIGDFTRAAGAAAIISFDMAVEFLQDKALKFCKRIFDVTMAPYLQ  480
ARO69902.1   401   LLKLELAEIPTDSTTCAPFKQWASAAKQMAKGVGTMIGDFTRAAGAAAIISFDMAVEFLQDKALKFCKRIFDVTMAPYLQ  480
ARO69861.1   401   LLKLELAEIPTDSTTCAPFKQWASAAKQMAKGVGTMIGDFTRAAGAAAIISFDMAVEFLQDKALKFCKRIFDVTMAPYLQ  480
ARO69850.1   401   LLKLELAEIPTDSTTCAPFKQWASAAKQMAKGVGTMIGDFTRAAGAAAIISFDMAVEFLQDKALKFCKRIFDVTMAPYLQ  480
QYU59227.1   401   LLKLELAEIPTDSTTCAPFKQWASAAKQMAKGVGTMVGDFARAAGAAAIISFDMAVEFLQDKALKFCKKIFDVTMAPYLQ  480
AFM91094.1   401   LLKLELAEIPTDSITCAPFRQWASAAKQMAKGVGTMIGDFTRAAGAAVIISFDMAVEFLQDKALKFCKRIFDVTMAPYLQ  480
ARO69883.1   401   LLKLELAEIPTDSTTCAPFKQWASAAKQMAKGVGTMIGDFTRAAGAAAIISFDMAVEFLQDKALKFCKRIFDVTMAPYLQ  480
ARO69879.1   401   LLKLELAEIPTDSTTCAPFKQWASAAKQMAKGVGTMIGDFTRAAGAAAIISFDMAVEFLQDKALKFCKRIFDVTMAPYLQ  480
AND76472.1   401   LLKLELAEIPTDSTTCAPFKQWASAAKQMAKGVGTMIGDFTRAAGAAAIISFDMAVEFLQDKALKFCKRIFDVTMAPYLQ  480
ARO69846.1   401   LLKLELAEIPTDSTTCAPFKQWASAAKQMAKGVGTMIGDFTRAAGAAAIISFDMAVEFLQDKALKFCKRIFDVTMAPYLQ  480
ARO69870.1   401   LLKLELAEIPTDSTTCAPFKQWASAAKQMAKGVGTMIGDFTRAAGAAAIISFDMAVEFLQDKALKFCKRIFDVTMAPYLQ  480
ARO69856.1   401   LLKLELAEIPTDSTTCAPFKQWASAAKQMAKGVGTMIGDFTRAAGAAAIISFDMAVEFLQDKALKFCKRIFDVTMAPYLQ  480
ARO69847.1   401   LLKLELAEIPMDSTTCAPFKQWASAAKRMAKGVGTMVGDFARAAGAAAIISFDMAVEFLQDKALKFCKKIFDVTMAPYLQ  480
ARO69875.1   401   LLKLELAEIPTDSTTCAPFKQWASAAKQMAKGVGTMVGDFARAAGAAAIISFDMAVEFLQDKALKFCKKIFDVTMAPYLQ  480
ARO69895.1   401   LLKLELAEIPTDSTTCAPFKQWASAAKQMAKGVGTMVGDFARAAGAAAIISFDMAVEFLQDKALKFCKKIFDVTMAPYLQ  480
ARO69880.1   401   LLKLELAEIPTDSTTCAPFKQWASAAKQMAKGVGTMVGDFARAAGAAAIISFDMAVEFLQDKALKFCKKIFDVTMAPYLQ  480
BCT87061.1   401   LLKLELMELPADSTTCAPFKQWASAAKQMANGVGAMLGDYARAAGTAAIISFDMAVEFLQDKALKFCKKIFDVTMAPYMQ  480
QYU59225.1   401   LLKLELAEIPTDSTTCAPFKQWASAAKQMAKGVGTMVGDFTRAAGAAVIISFDMAVEFLQDKALKFCKKIFDVTMAPYLQ  480
APD26306.1   401   LLKLELGELPANSTTCAPFKQWASASKQMAKGVGTMVGNFVRAAGAAAVISFDIAVEFLQDKALKFCKKIFDVTMAPYLQ  480


NP_619689.1  481   HLASAHSILKKIWEKLSEWMESLKSKASLALEVMRQHAIFALGAMVIGGVVVLVEKVLIAAKIIPNCGIILGAFLTLFFA  560
AWO68042.1   481   HLASAHSILKKIWEKLSEWMESLKSKASLALEVMAQHAIFALGAMVIGGVVVLVEKVLIAAKIIPNCGIILGAFLTLFFA  560
QYU59223.1   481   HLASAHTILKKIWEKLSEWMESLKNKASLALEVMAQHAIFALGAMVIGGVVVLVEKVLIAAKIIPNCGIILGAFLTLFFA  560
ARO69887.1   481   HLASAHSILKKIWEKLSEWMESLKSKASLALEVMAQHAIFALGAMVIGGVVVLVEKVLIAAKIIPNCGIILGAFLTLFFA  560
ARO69840.1   481   HLASAHSILKKIWEKLSEWMESLKSKASLALEVMAQHAIFALGAMVIGGVVVLVEKVLIAAKIIPNCGIILGAFLTLFFA  560
ARO69892.1   481   HLASAHSILKKIWEKLSEWMESLKSKASLALEVMAQHAIFALGAMVIGGVVVLVEKVLIAAKIIPNCGIILGAFLTLFFA  560
ARO69862.1   481   HLASAHSILKKIWEKLSEWMESLKSKASLALEVMAQHAIFALGAMVIGGVVVLVEKVLIAAKIIPNCGIILGAFLTLFFA  560
ARO69844.1   481   HLASAHSILKKIWEKLSEWMESLKSKASLALEVMAQHAIFALGAMVIGGVVVLVEKVLIAAKIIPNCGIILGAFLTLFFA  560
ARO69896.1   481   HLASAHSILKKIWEKLSEWMESLKSKASLALEVMAQHAIFALGAMVIGGVVVLVEKVLIAAKIIPNCGIILGAFLTLFFA  560
QGP72541.1   481   HLASAHSILKKIWEKLSEWMESLKSKASLALEVMAQHAIFALGAMVIGGVVVLVEKVLIAAKIIPNCGIILGAFLTLFFA  560
ARO69901.1   481   HLASAHSILKKIWEKLSEWMESLKSKASLALEVMAQHAIFALGAMVIGGVVVLVEKVLIAAKIIPNCGIILGAFLTLFFA  560
AYA22299.1   481   HLASAHSILRKIWEKLSEWMESLKSKASLALEVMAQHAIFALGAMVIGGVVVLVEKVLIAAKIIPNCGIILGAFLTLFFA  560
ARO69890.1   481   HLASAHSILKKIWEKLSEWMESLKSKASLALEVMAQHAIFALGAMVIGGVVVLVEKVLIAAKIIPNCGIILGAFLTLFFA  560
ARO69859.1   481   HLASAHSILKKIWEKLSEWMESLKSKASLALEVMAQHAIFALGAMVIGGVVVLVEKVLIAAKIIPNCGIILGAFLTLFFA  560
ARO69905.1   481   HLASAHSILKKIWEKLSEWMESLKSKASLALEVMAQHAIFALGAMVIGGVVVLVEKVLIAAKIIPNCGIILGAFLTLFFA  560
ARO69871.1   481   HLASAHSILKKIWEKLSEWMESLKSKASLALEVMAQHAIFALGAMVIGGVVVLVEKVLIAAKIIPNCGIILGAFLTLFFA  560
ARO69848.1   481   HLASAHSILKKIWEKLSEWMESLKSKASLALEVMAQHAIFALGAMVIGGVVVLVEKVLIAAKIIPNCGIILGAFLTLFFA  560
ARO69874.1   481   HLASAHSILKKIWEKLSEWMESLKSKASLALEVMAQHAIFALGAMVIGGVVVLVEKVLIAAKIIPNCGIILGAFLTLFFA  560
ARO69882.1   481   HLASAHSILKKIWEKLSEWMESLKSKASLALEVMAQHAIFALGAMVIGGVVVLVEKVLIAAKIIPNCGIILGAFLTLFFA  560
QYU59221.1   481   HLASAHSILKKIWEKLSEWMEGLKNKASLALEVMAQHAIFALGAMVIGGVVVLVEKVLIAAKIIPNCGIILGAFLTLFFA  560
ARO69842.1   481   HLASAHSILKKIWEKLSEWMESLKSKASLALEVMAQHAVFALGAMVIGGVVVLVEKVLIAAKIIPNCGIILGAFLTLFFA  560
AFV34731.1   481   HLASAHSILKKIWEKLSEWMESLKSKASLALEVMAQHAIFALGAMVIGGVVVLVEKVLIAAKIIPNCGIILGAFLTLFFA  560
AND76470.1   481   HLASAHSILKKIWEKLSEWMESLKSKASLALEVMAQHAIFALGAMVIGGVVVLVEKVLIAAKIIPNCGIILGAFLTLFFA  560
ACZ58632.1   481   HLASAHSILKKIWEKLSEWMESLKNKASLALEVMAQHAIFALGAMVIGGVVVLVEKVLIAAKIIPNCGIILGAFLTLFFA  560
QBZ78374.1   481   HLASAHSILKKIWEKLSEWMESLKNKASLALEVMAQHAIFALGAMVIGGVVVLVEKVLIAAKIIPNCGIILGAFLTLFFA  560
QGP72540.1   481   HLASAHSILKKIWEKLSEWMESLKSKASLALEVMAQHAIFALGAMVIGGVVVLVEKVLIAAKIIPNCGIILGAFLTLFFA  560
QBZ78372.1   481   HLASAHSILKKIWDKLSEWMESLKNKASLALEVMAQHAIFALGAMVIGGVVVLVEKVLIAAKIIPNCGIILGAFLTLFFA  560
QYU59224.1   481   HLASAHSILKKIWEKLSEWMESLKNKASLALEVMAQHAIFALGAMVIGGVVVLVEKVLIAAKIIPNCGIILGAFLTLFFA  560
ARO69877.1   481   HLASAHSILKKIWEKLSEWMESLKSKASLALEVMAQHAIFALGAMVIGGVVVLVEKVLVAAKIIPNCGIILGAFLTLFFA  560
ARO69858.1   481   HLASAHSILKKIWEKLSEWMESLKSKASLALEVMAQHAIFALGAMVIGGVVVLVEKVLVAAKIIPNCGIILGAFLTLFFA  560
ARO69855.1   481   HLASAHSILKKIWEKLSEWMESLKSKASLALEVMAQHAIFALGAMVIGGVVVLVEKVLIAAKIIPNCGIILGAFLTLFFA  560
QGP72542.1   481   HLASAHSILKKIWEKLSEWMESLKSKASLALEVMAQHAIFALGAMVIGGVVVLVEKVLIAAKIIPNCGIILGAFLTLFFA  560
AYQ93055.1   481   HLASAHSILKKIWEKLSEWMESLKSKASLALEVMAQHAIFALGAMVIGGIVVLVEKVLIAAKIIPNCGIILGAFLTLFFA  560
AYQ93053.1   481   HLASAHSIIKKIWEKLSEWMESLKSKASLALEVMAQHAIFALGAMVIGGVVVLVEKVLVAAKIIPNCGIILGAFLTLFFA  560
AYQ93051.1   481   HLASAHSILKRIWEKLSEWMESLKSKASLALEVMAQHAIFALGAMVIGGVVVLVEKVLVAAKIIPNCGIILGAFLTLFFA  560
AFI71813.1   481   HLASAHSILKKIWEKLSEWMESLKNKASLALEVMAQHAIFALGAMVIGGVVVLVEKVLIAAKIIPNCGIILGAFLTLFFA  560
QGP72539.1   481   HLASAHSILKKIWEKLSEWMESLKSKASLALEVMAQHAIFALGAMVIGGVVVLVEKVLIAAKIIPNCGIILGAFLTLFFA  560
QYU59226.1   481   HLASAHSILKKIWEKLSEWMESLKSKASLALEVMAQHAIFALGAMVIGGVVVLVEKVLIAAKIIPNCGIILGAFLTLFFA  560
ARO69852.1   481   HLASAHSILKKIWEKLSEWMESLKNKASLALEVMAQHAIFALGAMVIGGVVVLVEKVLIAAKIIPNCGIILGAFLTLFFA  560
QGP72543.1   481   HLASAHSILKKIWEKLSEWMESLKSKASLALEVMAQHAIFALGAMVIGGVVVLVEKVLVAAKIIPNCGIVLGAFLTLFFA  560
QYU59219.1   481   HLASAHSILKKIWEKLSEWMESLKNKASLALEVMAQHAIFALGAMVIGGVVVLVEKVLIAAKIIPNCGIILGAFLTLFFA  560
```

```
AGT42200.1   481   HLASAHSILKKIWEKLSEWMESLKNKASLALEVMAQHAIFALGAMVIGGVVVLVEKVLVAAKIIPNCGIILGAFLTLFFA  560
ACZ58633.1   481   HLASAHSILKKIWEKLSEWMESLKNKASLALEVMAQHAIFALGAMVIGGVVVLVEKVLVAAKIIPNCGIILGAFLTLFFA  560
QBZ78369.1   481   HLASAHSILKKIWEKLSEWMESLKSKASLALEVMAQHAIFALGAMVIGGVVVLVEKVLIAAKIIPNCGIILGAFLTLFFA  560
ARO69851.1   481   HLASAHSILKKIWEKLSEWMESLKSKASLALEVMAQHAIFALGAMVIGGVVVLVEKVLIAAKIIPNCGIILGAFLTLFFA  560
QYU59220.1   481   HLASAHTILKKIWEKLSEWMESLKSKASLALEVMAQHAIFALGAMVIGGVVVLVEKVLIAAKIIPNCGIILGAFLTLFFA  560
AFV34755.1   481   HLASAHSILKKIWEKLSEWMESLKSKASLALEVMAQHAIFALGAMVIGGVVVLVEKVLIAAKIIPNCGIILGAFLTLFFA  560
QGP72544.1   481   HLASAHSILKKIWEKLSEWMESLKTKASLALEVMAQHAIFALGAMVIGGVVVLVEKVLIAAKIIPNCGIILGAFLTLFFA  560
QYA72352.1   481   HLASAHSILKKIWEKLSEWMESLKSKASLALEVMAQHAIFALGAMVIGGVVVLVEKVLIAAKIIPNCGIILGAFLTLFFA  560
ARO69854.1   481   HLASAHSILKKIWEKLSEWMESLKSKASLALEVMAQHAIFALGAMVIGGVVVLVEKVLVAAKIIPNCGIILGAFLTLFFA  560
ARO69860.1   481   HLTSAHSILKKIWEKLSEWMESLKSKASLALEVMAQHAIFALGAMVIGGVVVLVEKVLVAAKIIPNCGIILGAFLTLFFA  560
ARO69863.1   481   HLTSAHSILKKIWEKLSEWMESLKSKASLALEVMAQHAIFALGAMVIGGVVVLVEKVLVAAKIIPNCGIILGAFLTLFFA  560
DAZ85707.1   481   HLASAHSILKKIWEKLSEWMESLKSKASLALEVMAQHAIFALGAMVIGGVVVLVEKVLVAARIIPNCGIILGAFLTLFFA  560
AWO67781.1   481   HLASAHSILKKIWEKLSEWMESLKSKASLALEVMAQHAIFALGAMVIGGVVVLVEKVLIAAKIIPNCGIILGAFLTLFFA  560
QBZ78367.1   481   HLASAHSILKKIWEKLSEWMESLKNKASLALEVMAQHAIFALGAMVIGGVVVLVEKVLVAAKIIPNCGIILGAFLTLFFA  560
ARO69873.1   481   HLTSAHSILKKIWEKLSEWMESLKSKASLALEVMAQHAIFALGAMVIGGVVVLVEKVLVAAKIIPNCGIILGAFLTLFFA  560
AND76471.1   481   HLTSAHSILKKIWEKLSEWMESLKSKASLALEVMAQHAIFALGAMVIGGVVVLVEKVLVAAKIIPNCGIILGAFLTLFFA  560
ARO69849.1   481   HLTSAHSILKKIWEKLSEWMESLKSKASLALEVMAQHAIFALGAMVIGGVVVLVEKVLVAAKIIPNCGIILGAFLTLFFA  560
ARO69889.1   481   HLASAHSILKKIWEKLSEWMESLKSKASLALEVMAQHAIFALGAMVIGGVVVLVEKVLVAAKIIPNCGIILGAFLTLFFA  560
ARO69867.1   481   HLASAHSILKKIWEKLSEWMESLKSKASLALEVMAQHAIFALGAMVIGGVVVLVEKVLVAAKIIPNCGIILGAFLTLFFA  560
ARO69904.1   481   HLASAHSILKKIWEKLSEWMESLKSKASLALEVMAQHAIFALGAMVIGGVVVLVEKVLVAAKIIPNCGIILGAFLTLFFA  560
ARO69869.1   481   HLTSAHSILKKIWEKLSEWMESLKSKASLALEVMAQHAIFALGAMVIGGVVVLVEKVLVAAKIIPNCGIILGAFLTLFFA  560
ARO69884.1   481   HLASAHSILKKIWEKLSEWMESLKSKASLALEVMAQHAIFALGAMVIGGVVVLVEKVLVAAKIIPNCGIILGAFLTLFFA  560
ARO69878.1   481   HLTSAHSILKKIWEKLSEWMESLKSKASLALEVMAQHAIFALGAMVIGGVVVLVEKVLVAAKIIPNCGIILGAFLTLFFA  560
ARO69885.1   481   HLASAHSILKKIWEKLSEWMESLKSKASLALEVMAQHAIFALGAMVIGGVVVLVEKVLVAAKIIPNCGIILGAFLTLFFA  560
ARO69841.1   481   HLASAHSILKKIWEKLSEWMESLKSKASLALEVMAQHAIFALGAMVIGGVVVLVEKVLVAAKIIPNCGIILGAFLTLFFA  560
ARO69891.1   481   HLTSAHSILKKIWEKLSEWMESLKNKASLALEVMAQHAIFALGAMVIGGVVVLVEKVLVAAKIIPNCGIILGAFLTLFFA  560
ARO69868.1   481   HLTSAHSILKKIWEKLSEWMESLKNKASLALEVMAQHAIFALGAMVIGGVVVLVEKVLVAAKIIPNCGIILGAFLTLFFA  560
ARO69900.1   481   HLTSAHSILKKIWEKLSEWMESLKSKASLALEVMAQHAIFALGAMVIGGVVVLVEKVLVAAKIIPNCGIILGAFLTLFFA  560
DAZ85706.1   481   HLASAHSILKKIWEKLSEWMESLKSKASLALEVMAQHAIFALGAMVIGGVVVLVEKVLIAAKIIPNCGIILGAFLTLFFA  560
ARO69899.1   481   HLTSAHSILKKIWEKLSEWMESLKNKASLALEVMAQHAIFALGAMVIGGVVVLVEKVLVAAKIIPNCGIILGAFLTLFFA  560
ARO69898.1   481   HLASAHSILKKIWEKLSEWMESLKSKASLALEVMAQHAIFALGAMVIGGVVVLVEKVLVAAKIIPNCGIILGAFLTLFFA  560
AYA22297.1   481   HLASAHSILKKIWEKLSEWMESLKSKASLALEVMAQHAIFALGAMVIGGVVVLVEKVLVAAKIIPNCGIILGAFLTLFFA  560
QYU59222.1   481   HLASAHSILKKVWEKLSEWMESLKSKASLALEVMAQHAIFALGAIVIGGVVVLVEKVLIAAKIIPNCGIILGAFLTLFFA  560
ARO69845.1   481   HLASAHSILKKIWEKLSEWMESLKSKASLALEVMAQHAIFALGAMVIGGVVVLVEKVLVAAKIIPNCGIILGAFLTLFFA  560
AND76473.1   481   HLASAHSILKKIWEKLSEWMESLKNKASLALEVMAQHAIFALGAMVIGGVVVLVEKVLVAAKIIPNCGIILGAFLTLFFA  560
ARO69857.1   481   HLASAHSILKKIWEKLSEWMESLKNKASLALEVMAQHAIFALGAMVIGGVVVLVEKVLVAAKIIPNCGIILGAFLTLFFA  560
DAZ85708.1   481   HLASAHSILKKIWEKLSEWMESLKTKASLALEVMAQHAIFALGAMVIGGVVVLVEKVLVAAKIIPNCGIILGAFLTLFFA  560
ARO69881.1   481   HLASAHSILKKIWEKLSEWMESLKSKASLALEVMAQHAIFALGAMVIGGVVVLVEKVLIAAKIIPNCGIILGAFLTLFFA  560
ARO69893.1   481   HLASAHSILKKIWEKLSEWMESLKSKASLALEVMAQHAIFALGAMVIGGVVVLVEKVLIAAKIIPNCGIILGAFLTLFFA  560
ARO69897.1   481   HLASAHSILKKIWEKLSEWMESLKSKASLALEVMAQHAIFALGAMVIGGVVVLVEKVLIAAKIIPNCGIILGAFLTLFFA  560
ARO69872.1   481   HLASAHSILKKIWEKLSEWMESLKSKASLALEVMAQHAIFALGAMVIGGVVVLVEKVLIAAKIIPNCGIILGAFLTLFFA  560
ARO69843.1   481   HLASAHSILKKIWEKLSEWMESLKSKASLALEVMAQHAIFALGAMVIGGVVVLVEKVLIAAKIIPNCGIILGAFLTLFFA  560
ARO69886.1   481   HLASAHSILKKIWEKLSEWMESLKSKASLALEVMAQHAIFALGAMVIGGVVVLVEKVLIAAKIIPNCGIILGAFLTLFFA  560
ARO69864.1   481   HLASAHSILKKIWEKLSEWMESLKSKASLALEVMAQHAIFALGAMVIGGVVVLVEKVLIAAKIIPNCGIILGAFLTLFFA  560
ARO69876.1   481   HLASAHSILKKIWEKLSEWMESLKSKASLALEVMAQHAIFALGAMVIGGVVVLVEKVLIAAKIIPNCGIILGAFLTLFFA  560
ARO69888.1   481   HLASAHSILKKIWEKLSEWMESLKSKASLALEVMAQHAIFALGAMVIGGVVVLVEKVLIAAKIIPNCGIILGAFLTLFFA  560
ARO69853.1   481   HLASAHSILKKIWEKLSEWMESLKSKASLALEVMAQHAIFALGAMVIGGVVVLVEKVLIAAKIIPNCGIILGAFLTLFFA  560
ARO69903.1   481   HLASAHSILKKIWEKLSEWMESLKSKASLALEVMAQHAIFALGAMVIGGVVVLVEKVLIAAKIIPNCGIILGAFLTLFFA  560
ARO69865.1   481   HLASAHSILKKIWEKLSEWMESLKSKASLALEVMAQHAIFALGAMVIGGVVVLVEKVLIAAKIIPNCGIILGAFLTLFFA  560
ARO69902.1   481   HLASAHSILKKIWEKLSEWMESLKSKASLALEVMAQHAIFALGAMVIGGVVVLVEKVLIAAKIIPNCGIILGAFLTLFFA  560
ARO69861.1   481   HLASAHSILKKIWEKLSEWMESLKSKASLALEVMAQHAIFALGAMVIGGVVVLVEKVLIAAKIIPNCGIILGAFLTLFFA  560
ARO69850.1   481   HLASAHSILKKIWEKLSEWMESLKSKASLALEVMAQHAIFALGAMVIGGVVVLVEKVLIAAKIIPNCGIILGAFLTLFFA  560
QYU59227.1   481   HLASAHSILKKIWEKLSEWMESLKSKASLALEVMAQHAIFALGAMVIGGVVVLVEKVLIAAKIIPNCGIILGAFLTLFFA  560
AFM91094.1   481   HLASAHSILKKIWEKLSEWMESLKSKASLALEVMAQHAIFALGAMVIGGVVVLVEKVLIAAKIIPNCGIILGAFLTLFFA  560
ARO69883.1   481   HLASAHSILKKIWEKLSEWMESLKSKASLALEVMAQHAIFALGAMVIGGVVVLVEKVLIAAKIIPNCGIILGAFLTLFFA  560
ARO69879.1   481   HLASAHSILKKIWEKLSEWMESLKSKASLALEVMAQHAIFALGAIVIGGVVVLVEKVLIAAKIIPNCGIILGAFLTLFFA  560
AND76472.1   481   HLASAHSILKKIWEKLSEWMESLKSKASLALEVMAQHAIFALGAMVIGGVVVLVEKVLIAAKIIPNCGIILGAFLTLFFA  560
ARO69846.1   481   HLASAHSILKKIWEKLSEWMESLKSKASLALEVMAQHAIFALGAMVIGGVVVLVEKVLIAAKIIPNCGIILGAFLTLFFA  560
ARO69870.1   481   HLASAHSILKKIWEKLSEWMESLKSKASLALEVMAQHAIFALGAMVIGGVVVLVEKVLIAAKIIPNCGIILGAFLTLFFA  560
ARO69856.1   481   HLASAHSILKKIWEKLSEWMESLKSKASLALEVMAQHAIFALGAMVIGGVVVLVEKVLIAAKIIPNCGIILGAFLTLFFA  560
ARO69847.1   481   HLASAHSILKKIWEKLSEWMESLKNKASLALEVMAQHAIFALGAMVIGGVVVLVEKVLVAAKIIPNCGIILGAFLTLFFA  560
ARO69875.1   481   HLTSAHSILKKIWEKLSEWMESLKNKASLALEVMAQHAIFALGAMVIGGVVVLVEKVLVAAKIIPNCGIILGAFLTLFFA  560
ARO69895.1   481   HLTSAHSILKKIWEKLSEWMESLKNKASLALEVMAQHAIFALGAMVIGGVVVLVEKVLVAAKIIPNCGIILGAFLTLFFA  560
ARO69880.1   481   HLTSAHSILKKIWEKLSEWMESLKNKASLALEVMAQHAIFALGAMVIGGVVVLVEKVLVAAKIIPNCGIILGAFLTLFFA  560
BCT87061.1   481   HLASAHSIVKKIWEKLSEWMESLKSKASLALEVMAQHAIFALGAMVIGGVVVLVEKVLVAARIIPNCGVILGAFLTLFFA  560
QYU59225.1   481   HLASAHSIVKKIWEKLSEWMESLKSKASLALEVMAQHAIFALGAMVIGGVVVLVEKVLVAAKIIPNCGIILGAFLTLFFA  560
APD26306.1   481   HLASAHSIVKKIWEKLSEWMESLKSKASLALEVMAQHAIFALGAMVIGGVVVLVEKVLVAAKIIPNCGVILGAFLTLFFA  560
```

X2-NTB cleavage site

```
NP_619689.1  561   SLGLTALECTAEEIFRMHACCKSAIYSMYSVAEPTMADEGESHTMGATQGLDNAIQALTRVGQSMISFKLGSFSYYAKIA  640
AWO68042.1   561   SLGLTALECTAEEIFRMHACCKSAIYSMYSVAEPTIADEGESHTMGATQGLDNAIQALTRVGQSMISFKLGSFSYYAKIA  640
QYU59223.1   561   SLGLTALECTAEEIFRMHACCKSAIYSMYSVEEPTMAGEGESHTMGATQGLDNAIQALTRVGQSMISFKLGSFSYYAKIA  640
ARO69887.1   561   SLGLTALECTAEEIFRMHACCKSAIYSMYSVEEPTMVGEGESHTMGATQGLDNAIQALTRVGQSMISFKLGSFSYYAKIA  640
ARO69840.1   561   SLGLTALECTAEEIFRMHACCKSAIYSMYSVEEPTMVGEGESHTMGATQGLDNAIQALTRVGQSMISFKLGSFSYYAKIA  640
ARO69892.1   561   SLGLTALECTAEEIFRMHACCKSAIYSMYSVEEPTMVGEGESHTMGATQGLDNAIQALTRVGQSMISFKLGSFSYYAKIA  640
ARO69862.1   561   SLGLTALECTAEEIFRMHACCKSAIYSMYSVEEPTMVGEGESHTMGATQGLDNAIQALTRVGQSMISFKLGSFSYYAKIA  640
ARO69844.1   561   SLGLTALECTAEEIFRMHACCKSAIYSMYSVEEPTMVGEGESHTMGATQGLDNAIQALTRVGQSMISFKLGSFSYYAKIA  640
ARO69896.1   561   SLGLTALECTAEEIFRMHACCKSAIYSMYSVEEPTMVGEGESHTMGATQGLDNAIQALTRVGQSMISFKLGSFSYYAKIA  640
QGP72541.1   561   SLGLTALECTAEEIFRMHACCKSAIYSMYSVEEPTMAGEGESHTMGATQGLDNAIQALTRVGQSMISFRLGSFSYYAKIA  640
ARO69901.1   561   SLGLTALECTAEEIFRMHACCKSAIYSMYSVEEPTMVGEGESHTMGATQGLDNAIQALTRVGQSMISFKLGSFSYYAKIA  640
AYA22299.1   561   SLGLTALECTAEEIFRMHACCKSAIYSMYSVEEPTMVGEGESHTMGATQGLDNAIQALTRVGQSMISFKLGSFSYYAKIA  640
ARO69890.1   561   SLGLTALECTAEEIFRMHACCKSAIYSMYSVEEPTMVGEGESHTMGATQGLDNAIQALTRVGQSMISFKLGSFSYYAKIA  640
ARO69859.1   561   SLGLTALECTAEEIFRMHACCKSAIYSMYSVEEPTMVGEGESHTMGATQGLDNAIQALTRVGQSMISFKLGSFSYYAKIA  640
ARO69905.1   561   SLGLTALECTAEEIFRMHACCKSAIYSMYSVEEPTMVGEGESHTMGATQGLDNAIQALTRVGQSMISFKLGSFSYYAKIA  640
ARO69871.1   561   SLGLTALECTAEEIFRMHACCKSAIYSMYSVEEPTMAGEGESHTMGATQGLDNAIQALTRVGQSMISFKLGSFSYYAKIA  640
ARO69848.1   561   SLGLTALECTAEEIFRMHACCKSAIYSMYSVEEPTMAGEGESHTMGATQGLDNAIQALTRVGQSMISFKLGSFSYYAKIA  640
```

```
ARO69874.1   561   SLGLTALECTAEEIFRMHACCKSAIYSMYSVEEPTMVGEGESHTMGATQGLDNAIQALTRVGQSMISFKLGSFSYYAKIA  640
ARO69882.1   561   SLGLTALECTAEEIFRMHACCKSAIYSMYSVEEPTMVGEGESHTMGATQGLDNAIQALTRVGQSMISFKLGSFSYYAKIA  640
QYU59221.1   561   SLGLTALECTAEEIFRMHACCKSAIYSMYSVEEPTMAGEGESHTMGATQGLDNAIQALTRVGQSMISFKLGSFSYYAKIA  640
ARO69842.1   561   SLGLTALECTAEEIFRMHACCKSAIYSMYSVEEPTMVGEGESHTMGATQGLDNAIQALTRVGQSMISFKLGSFSYYAKIA  640
AFV34731.1   561   SLGLTALECTAEEIFRMHACCKSAIYSMYSVEEPTIVGEGESHTMGATQGLDNAIQALTRVGQSMISFKLGSFSYYAKIA  640
AND76470.1   561   SLGLTALECTAEEIFRMHACCKSAIYSMYSVEEPTMVGEGESHTMGATQGLDNAIQALTRVGQSMISFKLGSFSYYAKIA  640
ACZ58632.1   561   SLGLTALECTAEEIFRMHACCKSAIYSMYSVEEPTMAGEGESHTMGATQGLDNAIQALTRVGQSMISFKLGSFSYYAKIA  640
QBZ78374.1   561   SLGLTALECTAEEIFRMHACCKSAIYSMYSVEEPTMAGEGESHTMGATQGLDNAIQALTRVGQSMISFKLGSFSYYAKIA  640
QGP72540.1   561   SLGLTALECTAEEIFRMHACCKSAIYSMYSVEEPTMVGEGESHTMGATQGLDNAIQALTRVGQSMISFKLGSFSYYAKIA  640
QBZ78372.1   561   SLGLTALECTAEEIFRMHACCKSAIYSMYSVEEPTMAGEGESHTMGATQGLDNAIQALTRVGQSMISFKLGSFSYYAKIA  640
QYU59224.1   561   SLGLTALECTAEEIFRMHACCKSAIYSMYSVEEPTMAGEGESHTMGATQGLDNAIQALTRVGQSMISFKLGSFSYYAKIA  640
ARO69877.1   561   SLGLTALECTAEEIFRMHACCKSAIYSMYSVEEPTMAGEGESHTMGATQGLDNAIQALTRVGQSMISFKLGSFSYYAKIA  640
ARO69858.1   561   SLGLTALECTAEEIFRMHACCKSAIYSMYSVEEPTMAGEGESHTMGATQGLDNAIQALTRVGQSMISFKLGSFSYYAKIA  640
ARO69855.1   561   SLGLTALECTAEEIFRMHACCKSAIYSMYSVEEPTMVGEGESHTMGATQGLDNAIQALTRVGQSMISFKLGSFSYYAKIA  640
QGP72542.1   561   SLGLTALECTAEEIFRMHACCKSAIYSMYSVEEPTMVGEGESHTMGATQGLDNAIQALTRVGQSMISFKLGSFSYYAKIA  640
AYQ93055.1   561   SLGLTALECTAEEIFRMHACCKSAIYSMYSVEEPTMVGEGESHTMGATRGLDNAIQALTRVGQSMISFKLGSFSYYAKIA  640
AYQ93053.1   561   SLGLTALECTAEEIFRMHTCCKSAIYSMYSVEEPTMAGDGESHTMGATQGLDNAIQALTRIGQSMISFNLGSFSYYAKIA  640
AYQ93051.1   561   SLGLTALECTAEEIFRMHACCKSAIYSMYSVEEPTMAGEGESHTMGATQGLDNAIQALTRVGQSMISFKLGSFSYYAKIA  640
AFI71813.1   561   SLGLTALECTAEEIFRMHACCKSAIYSMYSVEEPTMVGEGESHTMGATQGLDNAIQALTRVGQSMISFKLGSFSYYAKIA  640
QGP72539.1   561   SLGLTALECTAEEIFRMHACCKSAIYSMYSVEEPTMVGEGESHTMGATQGLDNAIQALTRVGQSMISFKLGSFSYYAKIA  640
QYU59226.1   561   SLGLTALECTAEEIFRMHACCKSAIYSMYSVEEPTMAGEGESHTMGATQGLDNAIQALTRVGQSMISFKLGSFSYYAKIA  640
ARO69852.1   561   SLGLTALECTAEEIFRMHACCKSAIYSMYSVEEPTMAGEGESHTMGATQGLDNAIQALTRVGQSMISFKLGSFSYYAKIA  640
QGP72543.1   561   SLGLTALECTAEEIFRMHACCKSAIYSMYSVEEPIMAGEGESHTMGATQGLDNAIQALTRVGQSMISFKLGSFSYYAKIA  640
QYU59219.1   561   SLGLTALECTAEEIFRMHACCKSAIYSMYSVEEPTMVGEGESHTMGATQGLDNAIQALTRVGQSMISFKLGSFSYYAKIA  640
AGT42200.1   561   SLGLTALECTAEEIFRMHACCKSAIYSMYSVEEPTMVGEGESHTMGATQGLDNAIQALTRVGQSMISFKLGSFSYYAKIA  640
ACZ58633.1   561   SLGLTALECTAEEIFRMHACCKSAIYSMYSVEEPTMAGEGESHTMGATQGLDNAIQALTRVGQSMISFKLGSFSYYAKIA  640
QBZ78369.1   561   SLGLTALECTAEEIFRMHACCKSAIYSMYSVEEPTMAGEGESHTMGATQGLDNAIQALTRVGQSMISFKLGSFSYYAKIA  640
ARO69851.1   561   SLGLTALECTAEEIFRMHACCKSAIYSMYSVEEPTMVGEGESHTMGATQGLDNAIQALTRVGQSMISFKLGSFSYYAKIA  640
QYU59220.1   561   SLGLTALECTAEEIFRMHACCKSAIYSMYSVEEPTLAGEGESHTMGAAQGLDNAIQALTRIGQSMISFKLGSFSYYAKIA  640
AFV34755.1   561   SLGLTALECTAEEIFRMHACCKSAIYSMYSVEEPTMAGEGESHTMGATQGLDNAIQALTRVGQSMISFKLGSFSYYAKIA  640
QGP72544.1   561   SLGLTALECTAEEIFRMHACCKSAIYSMYSVEEPTMAGEGESHIMGATQGLDNAIQALTRVGQSMISFKLGSFSYYAKIA  640
QYA72352.1   561   SLGLTALECTAEEIFRMHACCKSAIYSMYSVEEPTIAGEGESHTMGATRGLDNAIQALTRVGQSMISFKLGSFAYYAKIA  640
ARO69854.1   561   SLGLTALECTAEEIFRMHACCKSAIYSMYSVEEPTMAGEGESHTMGATQGLDNAIQALTRVGQSMISFKLGSFSYYAKIA  640
ARO69860.1   561   SLGLTALECTAEEIFRMHACCKSAIYSMYSVEEPTMAGEGESHTMGATQGLDNAIQALTRVGQSMISFKLGSFSYYAKIA  640
ARO69863.1   561   SLGLTALECTAEEIFRMHACCKSAIYSMYSVEEPTMAGEGESHTMGATQGLDNAIQALTRVGQSMISFKLGSFSYYAKIA  640
DAZ85707.1   561   SLGLTALECTAEEIFRMHACCKSAIYSMYSVEEPTMAGEGESHVMGATQGLDNAIQALTRVGQSMISFKLGSFSYYAKIA  640
AWO67781.1   561   SLGLTALECTAEEIFRMHACCKSAIYSMYSVEEPTMVGEGESHTMGATQGLDNAIQALTRIGQSMISFKLGSFTYYAKIA  640
QBZ78367.1   561   SLGLTALECTAEEIFRMHACCKSAIYSMYSVEEPTMVGEGESHTMGATQGLDNAIQALTRVGQSMISFKLGSFSYYAKIA  640
ARO69873.1   561   SLGLTALECTAEEIFRMHACCKSAIYSMYSVEEPTMAGEGESHTMGATQGLDNAIQALTRVGQSMISFKLGSFSYYAKIA  640
AND76471.1   561   SLGLTALECTAEEIFRMHACCKSAIYSMYSVEEPTMAGEGESHTMGATQGLDNAIQALTRVGQSMISFKLGSFSYYAKIA  640
ARO69849.1   561   SLGLTALECTAEEIFRMHACCKSAIYSMYSVEEPTMAGEGESHTMGATQGLDNAIQALTRVGQSMISFKLGSFSYYAKIA  640
ARO69889.1   561   SLGLTALECTAEEIFRMHACCKSAIYSMYSVEEPTMAGEGESHTMGATQGLDNAIQALTRVGQSMISFKLGSFSYYAKIA  640
ARO69867.1   561   SLGLTALECTAEEIFRMHACCKSAIYSMYSVEEPTMAGEGESHTMGATQGLDNAIQALTRVGQSMISFKLGSFSYYAKIA  640
ARO69904.1   561   SLGLTALECTAEEIFRMHACCKSAIYSMYSVEEPTMAGEGESHTMGATQGLDNAIQALTRVGQSMISFKLGSFSYYAKIA  640
ARO69869.1   561   SLGLTALECTAEEIFRMHACCKSAIYSMYSVEEPTMAGEGESHTMGATQGLDNAIQALTRVGQSMISFKLGSFSYYAKIA  640
ARO69884.1   561   SLGLTALECTAEEIFRMHACCKSAIYSMYSVEEPTMAGEGESHTMGATQGLDNAIQALTRVGQSMISFKLGSFSYYAKIA  640
ARO69878.1   561   SLGLTALECTAEEIFRMHACCKSAIYSMYSVEEPTMAGEGESHTMGATQGLDNAIQALTRVGQSMISFKLGSFSYYAKIA  640
ARO69885.1   561   SLGLTALECTAEEIFRMHACCKSAIYSMYSVEEPTMAGEGESHTMGATQGLDNAIQALTRVGQSMISFKLGSFSYYAKIA  640
ARO69841.1   561   SLGLTALECTAEEIFRMHACCKSAIYSMYSVEEPTMAGEGESHTMGATQGLDNAIQALTRVGQSMISFKLGSFSYYAKIA  640
ARO69891.1   561   SLGLTALECTAEEIFRMHACCKSAIYSMYSVEEPTMAGEGESHTMGATQGLDNAIQALTRVGQSMISFKLGSFSYYAKIA  640
ARO69868.1   561   SLGLTALECTAEEIFRMHACCKSAIYSMYSVEEPTMAGEGESHTMGATQGLDNAIQALTRVGQSMISFKLGSFSYYAKIA  640
ARO69900.1   561   SLGLTALECTAEEIFRMHACCKSAIYSMYSVEEPTMAGEGESHTMGATQGLDNAIQALTRVGQSMISFKLGSFSYYAKIA  640
DAZ85706.1   561   SLGLTALECTAEEIFRMHACCKSAIYSMYSVEEPTMAGEGESHVMGATQGLDNAIQALTRVGQSMISFKLGSFSYYAKIA  640
ARO69899.1   561   SLGLTALECTAEEIFRMHACCKSAIYSMYSVEEPTMAGEGESHTMGATQGLDNAIQALTRVGQSMISFKLGSFSYYAKIA  640
ARO69898.1   561   SLGLTALECTAEEIFRMHACCKSAIYSMYSVEEPTMAGEGESHTMGATQGLDNAIQALTRVGQSMISFKLGSFSYYAKIA  640
AYA22297.1   561   SLGLTALECTAEEIFRMHACCKSAIYSMYSVEEPTMAGEGESHTMGATQGLDNAIQALTRVGQSMISFKLGSFSYYAKIA  640
QYU59222.1   561   SLGLTALECTAEEIFRMHACCKSAIYSMYSVEEPTMVGEGESHTMGATQGLDNAIQALTRIGQSMISFKLGSFSYYAKIA  640
ARO69845.1   561   SLGLTALECTAEEIFRMHACCKSAIYSMYSVEEPTMAGEGESHTMGATQGLDNAIQALTRVGQSMISFKLGSFSYYAKIA  640
AND76473.1   561   SLGLTALECTAEEIFRMHACCKSAIYSMYSVEEPTMAGEGESHTMGATQGLDNAIQALTRVGQSMISFKLGSFSYYAKIA  640
ARO69857.1   561   SLGLTALECTAEEIFRMHACCKSAIYSMYSVEEPTMAGEGESHTMGATQGLDNAIQALTRVGQSMISFKLGSFSYYAKIA  640
DAZ85708.1   561   SLGLTALECTAEEIFRMHACCKSAIYSMYSVEEPTMAGEGESHTMGATQGLDNAIQALTRVGQSMISFKLGSFSYYAKIA  640
ARO69881.1   561   SLGLTALECTAEEIFRMHTCCKSAIYSMYSVEEPTMAGEGESHTMGATQGLDNAIQALTRVGQSMISFKLGSFSYYAKIA  640
ARO69893.1   561   SLGLTALECTAEEIFRMHTCCKSAIYSMYSVEEPTMAGEGESHTMGATQGLDNAIQALTRVGQSMISFKLGSFSYYAKIA  640
ARO69897.1   561   SLGLTALECTAEEIFRMHTCCKSAIYSMYSVEEPTMVGEGESHTMGATQGLDNAIQALTRVGQSMISFKLGSFSYYAKIA  640
ARO69872.1   561   SLGLTALECTAEEIFRMHTCCKSAIYSMYSVEEPTMAGEGESHTMGATQGLDNAIQALTRVGQSMISFKLGSFSYYAKIA  640
ARO69843.1   561   SLGLTALECTAEEIFRMHTCCKSAIYSMYSVEEPTMAGEGESHTMGATQGLDNAIQALTRVGQSMISFKLGSFSYYAKIA  640
ARO69886.1   561   SLGLTALECTAEEIFRMHTCCKSAIYSMYSVEEPTMAGEGESHTMGATQGLDNAIQALTRVGQSMISFKLGSFSYYAKIA  640
ARO69864.1   561   SLGLTALECTAEEIFRMHTCCKSAIYSMYSVEEPTMAGEGESHTMGATQGLDNAIQALTRVGQSMISFKLGSFSYYAKIA  640
ARO69876.1   561   SLGLTALECTAEEIFRMHTCCKSAIYSMYSVEEPTMVGEGESHTMGATQGLDNAIQALTRVGQSMISFKLGSFSYYAKIA  640
ARO69888.1   561   SLGLTALECTAEEIFRMHTCCKSAIYSMYSVEEPTMAGEGESHTMGATQGLDNAIQALTRVGQSMISFKLGSFSYYAKIA  640
ARO69853.1   561   SLGLTALECTAEEIFRMHTCCKSAIYSMYSVEEPTMVGEGESHTMGATQGLDNAIQALTRVGQSMISFKLGSFSYYAKIA  640
ARO69903.1   561   SLGLTALECTAEEIFRMHTCCKSAIYSMYSVEEPTMAGEGESHTMGATQGLDNAIQALTRVGQSMISFKLGSFSYYAKIA  640
ARO69865.1   561   SLGLTALECTAEEIFRMHTCCKSAIYSMYSVEEPTMAGEGESHTMGATQGLDNAIQALTRVGQSMISFKLGSFSYYAKIA  640
ARO69902.1   561   SLGLTALECTAEEIFRMHTCCKSAIYSMYSVEEPTMAGEGESHTMGATQGLDNAIQALTRVGQSMISFKLGSFSYYAKIA  640
ARO69861.1   561   SLGLTALECTAEEIFRMHTCCKSAIYSMYSVEEPTMAGEGESHTMGATQGLDNAIQALTRVGQSMISFKLGSFSYYAKIA  640
ARO69850.1   561   SLGLTALECTAEEIFRMHTCCKSVIYSMYSVEEPTMAGEGESHTMGATQGLDNAIQALTRVGQSMISFKLGSFSYYAKIA  640
QYU59227.1   561   SLGLTALECTAEEIFRMHACCKSAIYSMYSVEEPTMAGEGESHTMGATQGLDNAIQALTRVGQSMISFKLGSFSYYAKIA  640
AFM91094.1   561   SLGLTALECTAEEIFRMHACCKSAIYSMYSVEEPTMVGEGESHTMGATQGLDNAIQALTRVGQSMISFKLGSFSYYAKIA  640
ARO69883.1   561   SLGLTALECTAEEIFRMHACCKSAIYSMYSVEEPTMAGEGESHTMGATQGLDNAIQALTRVGQSMISFKLGSFSYYAKIA  640
ARO69879.1   561   SLGLTALECTAEEIFRMHTCCKSAIYSMYSVEEPTMAGEGESHTMGATQGLDNAIQALTRVGQSMISFKLGSFSYYAKIA  640
AND76472.1   561   SLGLTALECTAEEIFRMHTCCKSAIYSMYSVEEPTMAGEGESHTMGATQGLDNAIQALTRVGQSMISFKLGSFSYYAKIA  640
ARO69846.1   561   SLGLTALECTAEEIFRMHTCCKSAIYSMYSVEEPTMAGEGESHTMGATQGLDNAIQALTRVGQSMISFKLGSFSYYAKIA  640
ARO69870.1   561   SLGLTALECTAEEIFRMHACCKSAIYSMYSVEEPTMAGEGESHTMGATQGLDNAIQALTRVGQSMISFKLGSFSYYAKIA  640
ARO69856.1   561   SLGLTALECTAEEIFRMHACCKSAIYSMYSVEEPTMAGEGESHTMGATQGLDNAIQALTRVGQSMISFKLGSFSYYAKIA  640
ARO69847.1   561   SLGLTALECTAEEIFRMHACCKSAIYSMYSVEEPTMAGEGESHTMGATQGLDNAIQALTRVGQSMISFKLGSFSYYAKIA  640
ARO69875.1   561   SLGLTALECTAEEIFRMHACCKSAIYSMYSVEEPTMAGEGESHTMGATQGLDNAIQALTRVGQSMISFKLGSFSYYAKIA  640
ARO69895.1   561   SLGLTALECTAEEIFRMHACCKSAIYSMYSVEEPTMAGEGESHTMGATQGLDNAIQALTRVGQSMISFKLGSFSYYAKIA  640
ARO69880.1   561   SLGLTALECTAEEIFRMHACCKSAIYSMYSVEEPTMAGEGESHTMGATQGLDNAIQALTRVGQSMISFKLGSFSYYAKIA  640
```

```
BCT87061.1   561   SLGLTALECTAEEIFRMHACCKSAIYSMYSVNEPTMVGEGESVTMGATRGLDNAISALTRVGQSMMSFKLGSFSYYAKIA  640
QYU59225.1   561   SLGLTALECTAEEIFRMHTCCKSAIYSMYSVNEPTMAGEGESHTMGATQGLDNAVQALTRVGQSMISFKLGSLSYYAKIG  640
APD26306.1   561   SLGLTALECTAEEIFRMHACCKSAIYSMYSVSEPTVVGEGESVVMGATQGLDNAIQALTRVGQSMISFKLGSFSYYAKIA  640


NP_619689.1  641   QGFDQLARGKRAIGELTSWLIDLVGSIYSQVSGQESTFFDELSTIVCLDVRAWLLKSKRVRLQVETMAIGDRITLDTIAK  720
AWO68042.1   641   QGFDQLARGKRAIGELTSWLIDLVGSIYSQVSGQESTFFDELSTIVCLDVRAWLLKSKRVRLQVETMAIGDRITLDTIAK  720
QYU59223.1   641   QGFDQLARGNCAIGELTSWLIDLVGSIYSQVSGQESTFFDELSTIVCLDVRAWLLKSKRVRLQVETMAIGDRVTLDTIAK  720
ARO69887.1   641   QGFDQLARGKRAIGELTSWLIDLVGSIYSQVSGQESTFFDELSTIVCLDVRAWLLKSKRVRLQVETMAIGDRITLDTIAK  720
ARO69840.1   641   QGFDQLARGKRAIGELTSWLIDLVGSIYSQVSGQESTFFDELSTIVCLDVRAWLLKSKRVRLQVETMAIGDRITLDTIAK  720
ARO69892.1   641   QGFDQLARGKRAIGELTSWLIDLVGSIYSQVSGQESTFFDELSTIVCLDVRAWLLKSKRVRLQVETMAIGDRITLDTIAK  720
ARO69862.1   641   QGFDQLARGKRAIGELTSWLIDLVGSIYSQVSGQESTFFDELSTIVCLDVRAWLLKSKRVRLQVETMAIGDRITLDTIAK  720
ARO69844.1   641   QGFDQLARGKRAIGELTSWLIDLVGSIYSQVSGQESTFFDELSTIVCLDVRAWLLKSKRVRLQVETMAIGDRITLDTIAK  720
ARO69896.1   641   QGFDQLARGKRAIGELTSWLIDLVGSIYSQVSGQESTFFDELSTIVCLDVRAWLLKSKRVRLQVETMAIGDRITLDTIAK  720
QGP72541.1   641   QGFDQLARGKRAIGELTSWLIDLVGSIYSQVSGQESTFFDELSTIVCLDVRAWLLKSKRVRLQVETMAIGDRITLDTIAK  720
ARO69901.1   641   QGFDQLARGKRAIGELTSWLIDLVGSIYSQVSGQESTFFDELSTIVCLDVRAWLLKSKRVRLQVETMAIGDRITLDTIAK  720
AYA22299.1   641   QGFDQLARGKRAIGELTSWLIDLVGSIYSQVSGQESTFFDELSTIVCLDVRAWLLKSKRVRLQVETMAIGDRITLDTIAK  720
ARO69890.1   641   QGFDQLARGKRAIGELTSWLIDLVGSIYSQVSGQESTFFDELSTIVCLDVRAWLLKSKRVRLQVETMAIGDRITLDTIAK  720
ARO69859.1   641   QGFDQLARGKRAIGELTSWLIDLVGSIYSQVSGQESTFFDELSTIVCLDVRAWLLKSKRVRLQVETMAIGDRITLDTIAK  720
ARO69905.1   641   QGFDQLARGKRAIGELTSWLIDLVGSIYSQVSGQESTFFDELSTIVCLDVRAWLLKSKRVRLQVETMAIGDRITLDTIAK  720
ARO69871.1   641   QGFDQLARGKRAIGELTSWLIDLVGSIYSQVSGQESTFFDELSTIVCLDVRAWLLKSKRVRLQVETMAIGDRITLDTIAK  720
ARO69848.1   641   QGFDQLARGKRAIGELTSWLIDLVGSIYSQVSGQESTFFDELSTIVCLDVRAWLLKSKRVRLQVETMAIGDRITLDTIAK  720
ARO69874.1   641   QGFDQLARGKRAIGELTSWLIDLVGSIYSQVSGQESTFFDELSTIVCLDVRAWLLKSKRVRLQVETMAIGDRITLDTIAK  720
ARO69882.1   641   QGFDQLARGKRAIGELTSWLIDLVGSIYSQVSGQESTFFDELSTIVCLDVRAWLLKSKRVRLQVETMAIGDRITLDTIAK  720
QYU59221.1   641   QGFDQLARGKRAIGELTSWLIDLVGSIYSQVSGQESTFFDELSTIVCLDVRAWLLKSKRVRLQVETMAIGDRITLDTIAK  720
ARO69842.1   641   QGFDQLARGKRAIGELTSWLIDLVGSIYSQVSGQESTFFDELSTIVCLDVRAWLLKSKRVRLQVETMAIGDRITLDTIAK  720
AFV34731.1   641   QGFDQLARGKRAIGELTSWLIDLVGSIYSQVSGQESTFFDELSTIVCLDVRAWLLKSKRVRLQVETMAIGDRITLDTIAK  720
AND76470.1   641   QGFDQLARGKRAIGELTSWLIDLVGSIYSQVSGQESTFFDELSTIVCLDVRAWLLKSKRVRLQVETMAIGDRITLDTIAK  720
ACZ58632.1   641   QGFDQLARGKRAIGELTSWLIDLVGSIYSQVSGQESTFFDELSTIVCLDVRAWLLKSKRVRLQVETMAIGDRITLDTIAK  720
QBZ78374.1   641   QGFDQLARGKRAIGELTSWLIDLVGSIYSQVSGQESTFFDELSTIVCLDVRAWLLKSKRVRLQVETMAIGDRITLDTIAK  720
QGP72540.1   641   QGFDQLARGKRAIGELTSWLIDLVGSIYSQVSGQESTFFDELSTIVCLDVRAWLLKSKRVRLQVETMAIGDRITLDTIAK  720
QBZ78372.1   641   QGFDQLARGKRAIGELTSWLIDLVGSIYSQVSGQESTFFDELSTIVCLDVRAWLLKSKRVRLQVETMAIGDRITLDTIAK  720
QYU59224.1   641   QGFDQLARGKRAIGELTSWLIDLVGSIYSQVSGQESTFFDELSTIVCLDVRAWLLKSKRVRLQVETMAIGDRITLDTIAK  720
ARO69877.1   641   QGFDQLARGKRAIGELTSWLIDLVGSIYSQVSGQESTFFDELSTIVCLDVRAWLLKSKRVRLQVETMAIGDRITLDTIAK  720
ARO69858.1   641   QGFDQLARGKRAIGELTSWLIDLVGSIYSQVSGQESTFFDELSTIVCLDVRAWLLKSKRVRLQVETMAIGDRITLDTIAK  720
ARO69855.1   641   QGFDQLARGKRAIGELTSWLIDLVGSIYSQVSGQESTFFDELSTIVCLDVRAWLLKSKRVRLQVETMAIGDRITLDTIAK  720
QGP72542.1   641   QGFDQLARGKRAIGELTSWLIDLVGSIYSQVSGQESTFFDELSTIVCLDVRAWLLKSKRVRLQVETMAIGDRITLDTIAK  720
AYQ93055.1   641   QGFDQLARGKRAIGELTSWLIDLVGSIYSQVSGQESIFFDELSTIVCLDVRAWLLKSKRVRLQVETMAIGDRITLDTIAK  720
AYQ93053.1   641   QGFDQLARGKRAIGELTSWLIDLVGSVYSQVSGQESTFFDELSTIVCLDVRAWLLKSKRVRLQVETMAIGDRITLDTIAK  720
AYQ93051.1   641   QGFDQLARGKRAIGELTSWLIDLVGSIYSQVSGQESTFFDELSTIVCLDVRAWLLKSKRVRLQVETMAIGDRITLDTIAK  720
AFI71813.1   641   QGFDQLARGKRAIGELTSWLIDLVGSIYSQVSGQESTFFDELSTIVCLDVRAWLLKSKRVRLQVETMAIGDRITLDTIAK  720
QGP72539.1   641   QGFDQLARGKRAIGELTSWLIDLVGSIYSQVSGQESTFFDELSTIVCLDVRAWLLKSKRVRLQVETMAIGDRITLDTIAK  720
QYU59226.1   641   QGFDQLARGKRAIGELTSWLIDLVGSIYSQVSGQESTFFDELSTIVCLDVRAWLLKSKRVRLQVETMAIGDRITLDTIAK  720
ARO69852.1   641   QGFDQLARGKRAIGELTSWLIDLVGSIYSQVSGQESTFFDELSTIVCLDVRAWLLKSKRVRLQVETMAIGDRITLDTIAK  720
QGP72543.1   641   QGFDQLARGKRAIGELTSWLIDLIGSIYSQVSGQESTFFDELSTIVCLDVRAWLLKSKRVRLQVETMAIGDRITLNTIAK  720
QYU59219.1   641   QGFDQLARGKRAIGELTSWLIDLVGSIYSQVSGQESTFFDELSTIVCLDVRAWLLKSKRVRLQVETMAIGDRITLDTIAK  720
AGT42200.1   641   QGFDQLARGKRAIGELTSWLIDLVGSIYSQVSGQESTFFDELSTIVCLDVRAWLLKSKRVRLQVETMAIGDRITLDTIAK  720
ACZ58633.1   641   QGFDQLARGKRAIGELTSWLIDLVGSIYSQVSGQESTFFDELSTIVCLDVRAWLLKSKRVRLQVETMAIGDRITLDTIAK  720
QBZ78369.1   641   QGFDQLARGKRAIGELTSWLIDLVGSIYSQVSGQESTFFDELSTIVCLDVRAWLLKSKRVRLQVETMAIGDRITLDTIAK  720
ARO69851.1   641   QGFDQLARGKRAIGELTSWLIDLVGSIYSQVSGQESTFFDELSTIVCLDVRAWLLKSKRVRLQVETMAIGDRITLDTIAK  720
QYU59220.1   641   QGFDQLARGKRAIGELTSWLIDLVGSIYSQVSGQESTFFDELSTIVCLDVRAWLLKSKRVRLQVETMAIGDRITLDTIAK  720
AFV34755.1   641   QGFDQLARGKRAIGELTSWLIDLVGSIYSQVSGQESTFFDELSTIVCLDVRAWLLKSKRVRLQVETMAIGDRITLDTIAK  720
QGP72544.1   641   QGFDQLARGKRAIGELTSWLIDLVGSIYSQVSGQESTFFDELSTIVCLDVRAWLLKSKRVRLQVETMAIGDRITLDTIAK  720
QYA72352.1   641   QGFDQLARGKRAIGELTSWLIDLVGSIYSQVSGQESTFFDELSTIVCLDVRAWLLKSKRVRLQVETMAIGDRITLNTIAK  720
ARO69854.1   641   QGFDQLARGKRAIGELTSWLIDLVGSIYSQVSGQESTFFDELSTIVCLDVRAWLLKSKRVRLQVETMAIGDRITLDTIAK  720
ARO69860.1   641   QGFDQLARGKRAIGELTSWLIDLVGSIYSQVSGQESTFFDELSTIVCLDVRAWLLKSKRVRLQVETMAIGDRITLDTIAK  720
ARO69863.1   641   QGFDQLARGKRAIGELTSWLIDLVGSIYSQVSGQESTFFDELSTIVCLDVRAWLLKSKRVRLQVETMAIGDRITLDTIAK  720
DAZ85707.1   641   QGFDQLARGKRAIGELTSWLIDLVGSIYSQVSGQESTFFDELSTIVCLDVRAWLLKSKRVRLQVETMAIGDRITLDTIAK  720
AWO67781.1   641   QGFDQLARGKRAIGELTSWLIDLVGSIYSQVSGQESTFFDELSTIVCLDVRAWLLKSKRVRLQVETMAIGDRITLNTIAK  720
QBZ78367.1   641   QGFDQLARGKRAIGELTSWLIDLVGSIYSQVSGQESTFFDELSTIVCLDVRAWLLKSKRVRLQVETMAIGDRITLDTIAK  720
ARO69873.1   641   QGFDQLARGKRAIGELTSWLIDLVGSIYSQVSGQESTFFDELSTIVCLDVRAWLLKSKRVRLQVETMAIGDRITLDTIAK  720
AND76471.1   641   QGFDQLARGKRAIGELTSWLIDLVGSIYSQVSGQESTFFDELSTIVCLDVRAWLLKSKRVRLQVETMAIGDRITLDTIAK  720
ARO69849.1   641   QGFDQLARGKRAIGELTSWLIDLVGSIYSQVSGQESTFFDELSTIVCLDVRAWLLKSKRVRLQVETMAIGDRITLDTIAK  720
ARO69889.1   641   QGFDQLARGKRAIGELTSWLIDLVGSIYSQVSGQESTFFDELSTIVCLDVRAWLLKSKRVRLQVETMAIGDRITLDTIAK  720
ARO69867.1   641   QGFDQLARGKRAIGELTSWLIDLVGSIYSQVSGQESTFFDELSTIVCLDVRAWLLKSKRVRLQVETMAIGDRITLDTIAK  720
ARO69904.1   641   QGFDQLARGKRAIGELTSWLIDLVGSIYSQVSGQESTFFDELSTIVCLDVRAWLLKSKRVRLQVETMAIGDRITLDTIAK  720
ARO69869.1   641   QGFDQLARGKRAIGELTSWLIDLVGSIYSQVSGQESTFFDELSTIVCLDVRAWLLKSKRVRLQVETMAIGDRITLDTIAK  720
ARO69884.1   641   QGFDQLARGKRAIGELTSWLIDLVGSIYSQVSGQESTFFDELSTIVCLDVRAWLLKSKRVRLQVETMAIGDRITLDTIAK  720
ARO69878.1   641   QGFDQLARGKRAIGELTSWLIDLVGSIYSQVSGQESTFFDELSTIVCLDVRAWLLKSKRVRLQVETMAIGDRITLDTIAK  720
ARO69885.1   641   QGFDQLARGKRAIGELTSWLIDLVGSIYSQVSGQESTFFDELSTIVCLDVRAWLLKSKRVRLQVETMAIGDRITLDTIAK  720
ARO69841.1   641   QGFDQLARGKRAIGELTSWLIDLVGSIYSQVSGQESTFFDELSTIVCLDVRAWLLKSKRVRLQVETMAIGDRITLDTIAK  720
ARO69891.1   641   QGFDQLARGKRAIGELTSWLIDLVGSIYSQVSGQESTFFDELSTIVCLDVRAWLLKSKRVRLQVETMAIGDRITLDTIAK  720
ARO69868.1   641   QGFDQLARGKRAIGELTSWLIDLVGSIYSQVSGQESTFFDELSTIVCLDVRAWLLKSKRVRLQVETMAIGDRITLDTIAK  720
ARO69900.1   641   QGFDQLARGKRAIGELTSWLIDLVGSIYSQVSGQESTFFDELSTIVCLDVRAWLLKSKRVRLQVETMAIGDRITLDTIAK  720
DAZ85706.1   641   QGFDQLARGKRAIGELTSWLIDLVGSIYSQVSGQESTFFDELSTIVCLDVRAWLLKSKRVRLQVETMAIGDRITLNTIAK  720
ARO69899.1   641   QGFDQLARGKRAIGELTSWLIDLVGSIYSQVSGQESTFFDELSTIVCLDVRAWLLKSKRVRLQVETMAIGDRITLDTIAK  720
ARO69898.1   641   QGFDQLARGKRAIGELTSWLIDLVGSIYSQVSGQESTFFDELSTIVCLDVRAWLLKSKRVRLQVETMAIGDRITLDTIAK  720
AYA22297.1   641   QGFDQLARGKRAIGELTSWLIDLVGSIYSQVSGQESTFFDELSTIVCLDVRAWLLKSKRVRLQVETMAIGDRITLDTIAK  720
QYU59222.1   641   QGFDQLARGKRAIGELTSWLIDLVGSIYSQVSGQESTFFDELSTIVCLDVRAWLLKSKRVRLQVETMAIGDRITLDTIAK  720
ARO69845.1   641   QGFDQLARGKRAIGELTSWLIDLVGSIYSQVSGQESTFFDELSTIVCLDVRAWLLKSKRVRLQVETMAIGDRITLDTIAK  720
AND76473.1   641   QGFDQLARGKRAIGELTSWLIDLVGSIYSQVSGQESTFFDELSTIVCLDVRAWLLKSKRVRLQVETMAIGDRITLDTIAK  720
ARO69857.1   641   QGFDQLARGKRAIGELTSWLIDLVGSIYSQVSGQESTFFDELSTIVCLDVRAWLLKSKRVRLQVETMAIGDRITLDTIAK  720
DAZ85708.1   641   QGFDQLARGKRAIGELTSWLIDLVGSIYSQVSGQESTFFDELSTIVCLDVRAWLLKSKRVRLQVETMAIGDRITLDTIAK  720
ARO69881.1   641   QGFDQLARGKRAIGELTSWLIDLVGSIYSQVSGQESTFFDELSTIVCLDVRAWLLKSKRVRLQVETMAIGDRITLDTIAK  720
ARO69893.1   641   QGFDQLARGKRAIGELTSWLIDLVGSIYSQVSGQESTFFDELSTIVCLDVRAWLLKSKRVRLQVETMAIGDRITLDTIAK  720
ARO69897.1   641   QGFDQLARGKRAIGELTSWLIDLVGSIYSQVSGQESTFFDELSTIVCLDVRAWLLKSKRVRLQVETMAIGDRITLDTIAK  720
ARO69872.1   641   QGFDQLARGKRAIGELTSWLIDLVGSIYSQVSGQESTFFDELSTIVCLDVRAWLLKSKRVRLQVETMAIGDRITLDTIAK  720
ARO69843.1   641   QGFDQLARGKRAIGELTSWLIDLVGSIYSQVSGQESTFFDELSTIVCLDVRAWLLKSKRVRLQVETMAIGDRITLDTIAK  720
```

```
ARO69886.1   641   QGFDQLARGKRAIGELTSWLIDLVGSIYSQVSGQESTFFDELSTIVCLDVRAWLLKSKRVRLQVETMAIGDRITLDTIAK  720
ARO69864.1   641   QGFDQLARGKRAIGELTSWLIDLVGSIYSQVSGQESTFFDELSTIVCLDVRAWLLKSKRVRLQVETMAIGDRITLDTIAK  720
ARO69876.1   641   QGFDQLARGKRAIGELTSWLIDLVGSIYSQVSGQESTFFDELSTIVCLDVRAWLLKSKRVRLQVETMAIGDRITLDTIAK  720
ARO69888.1   641   QGFDQLARGKRAIGELTSWLIDLVGSIYSQVSGQESTFFDELSTIVCLDVRAWLLKSKRVRLQVETMAIGDRITLDTIAK  720
ARO69853.1   641   QGFDQLARGKRAIGELTSWLIDLVGSIYSQVSGQESTFFDELSTIVCLDVRAWLLKSKRVRLQVETMAIGDRITLDTIAK  720
ARO69903.1   641   QGFDQLARGKRAIGELTSWLIDLVGSIYSQVSGQESTFFDELSTIVCLDVRAWLLKSKRVRLQVETMAIGDRITLDTIAK  720
ARO69865.1   641   QGFDQLARGKRAIGELTSWLIDLVGSIYSQVSGQESTFFDELSTIVCLDVRAWLLKSKRVRLQVETMAIGDRITLDTIAK  720
ARO69902.1   641   QGFDQLARGKRAIGELTSWLIDLVGSIYSQVSGQESTFFDELSTIVCLDVRAWLLKSKRVRLQVETMAIGDRITLDTIAK  720
ARO69861.1   641   QGFDQLARGKRAIGELTSWLIDLVGSIYSQVSGQESTFFDELSTIVCLDVRAWLLKSKRVRLQVETMAIGDRITLDTIAK  720
ARO69850.1   641   QGFDQLARGKRAIGELTSWLIDLVGSIYSQVSGQESTFFDELSTIVCLDVRAWLLKSKRVRLQVETMAIGDRITLDTIAK  720
QYU59227.1   641   QGFDQLARGKRAIGELTSWLIDLVGSIYSQVSGQESTFFDELSTVVCLDVRAWLLKSKRVRLQVETMAIGDRITLDTIAK  720
AFM91094.1   641   QGFDQLARGKRAIGELTSWLIDLVGSIYSQVSGQESTFFDELSTIVCLDVRAWLLKSKRVRLQVETMAIGDRITLDTIAK  720
ARO69883.1   641   QGFDQLARGKRAIGELTSWLIDLVGSIYSQVSGQESTFFDELSTIVCLDVRAWLLKSKRVRLQVETMAIGDRITLDTIAK  720
ARO69879.1   641   QGFDQLARGKRAIGELTSWLIDLVGSIYSQVSGQESTFFDELSTIVCLDVRAWLLKSKRVRLQVETMAIGDRITLDTIAK  720
AND76472.1   641   QGFDQLARGKRAIGELTSWLIDLVGSIYSQVSGQESTFFDELSTIVCLDVRAWLLKSKRVRLQVETMAIGDRITLDTIAK  720
ARO69846.1   641   QGFDQLARGKRAIGELTSWLIDLVGSIYSQVSGQESTFFDELSTIVCLDVRAWLLKSKRVRLQVETMAIGDRITLDTIAK  720
ARO69870.1   641   QGFDQLARGKRAIGELTSWLIDLVGSIYSQVSGQESTFFDELSTIVCLDVRAWLLKSKRVRLQVETMAIGDRITLDTIAK  720
ARO69856.1   641   QGFDQLARGKRAIGELTSWLIDLVGSIYSQVSGQESTFFDELSTIVCLDVRAWLLKSKRVRLQVETMAIGDRITLDTIAK  720
ARO69847.1   641   QGFDQLARGKRAIGELTSWLIDLVGSIYSQVSGQESTFFDELSTIVCLDVRAWLLKSKRVRLQVETMAIGDRITLDTIAK  720
ARO69875.1   641   QGFDQLARGKRAIGELTSWLIDLVGSIYSQVSGQESTFFDELSTIVCLDVRAWLLKSKRVRLQVETMAIGDRITLDTIAK  720
ARO69895.1   641   QGFDQLARGKRAIGELTSWLIDLVGSIYSQVSGQESTFFDELSTIVCLDVRAWLLKSKRVRLQVETMAIGDRITLDTIAK  720
ARO69880.1   641   QGFDQLARGKRAIGELTSWLIDLVGSIYSQVSGQESTFFDELSTIVCLDVRAWLLKSKRVRLQVETMAIGDRITLDTIAK  720
BCT87061.1   641   QGFDQLARGKRAIGELTSWLIDLVGSIYSKVSGQESTFFDELSTIVCLDVRAWLLRSKRVRLQVETMAIGDRITLDTISK  720
QYU59225.1   641   QGFDQLARGKKAIGELTSWLIDLVGSIYSTVSGQESTFFDELSTIVCLDVRAWLLKSKRVRLQVETMAIGDRITLDTISK  720
APD26306.1   641   QGFDQLARGKRAIGELTSWLIDLVGSIYSQVSGQESTFFDELSTIVCLDVRAWLLRSKRVRLQVETMAIGDRITLNTISK  720


NP_619689.1  721   LLEEGHKILVTAAGVPRKTSADFTMCIKEEVSKLEEVHARTACAGINEGMRAFPFWVYIFGASQSGKTTIANSIIIPALL  800
AWO68042.1   721   LLEEGHKILVTAAGVPRKTSADFTMCIKEEVSKLEEVHARTACAGINEGMRAFPFWVYIFGASQSGKTTIANSIIIPALL  800
QYU59223.1   721   LLEEGHKILVTAAGVPRKTSADFTMCIKEEVSKLEEVHARTACAGINEGMRAFPFWVYIFGASQSGKTTIANSVIIPALL  800
ARO69887.1   721   LLEEGHKILVTAAGVPRKTSADFTMCIKEEVSKLEEVHARTACAGINEGMRAFPFWVYIFGASQSGKTTIANSVIIPSLL  800
ARO69840.1   721   LLEEGHKILVTAAGVPRKTSADFTMCIKEEVSKLEEVHARTACAGINEGMRAFPFWVYIFGASQSGKTTIANSVIIPSLL  800
ARO69892.1   721   LLEEGHKILVTAAGVPRKTSADFTMCIKEEVSKLEEVHARTACAGINEGMRAFPFWVYIFGASQSGKTTIANSVIIPSLL  800
ARO69862.1   721   LLEEGHKILVTAAGVPRKTSADFTMCIKEEVSKLEEVHARTACAGINEGMRAFPFWVYIFGASQSGKTTIANSVIIPSLL  800
ARO69844.1   721   LLEEGHKILVTAAGVPRKTSADFTMCIKEEVSKLEEVHARTACAGINEGMRAFPFWVYIFGASQSGKTTIANSVIIPSLL  800
ARO69896.1   721   LLEEGHKILVTAAGVPRKTSADFTMCIKEEVSKLEEVHARTACAGINEGMRAFPFWVYIFGASQSGKTTIANSVIIPSLL  800
QGP72541.1   721   LLEEGHKILVTAAGVPRKTSADFTMCIKEEVSKLEEVHARTACAGINEGMRAFPFWVYIFGASQSGKTTIANSVIIPALL  800
ARO69901.1   721   LLEEGHKILVTAAGVPRKTSADFTMCIKEEVSKLEEVHARTACAGINEGMRAFPFWVYIFGASQSGKTTIANSVIIPSLL  800
AYA22299.1   721   LLEEGHKILVTAAGVPRKTSADFTMCIKEEVSKLEEVHARTACAGINEGMRAFPFWVYIFGASQSGKTTIANSVIIPALL  800
ARO69890.1   721   LLEEGHKILVTAAGVPRKTSADFTMCIKEEVSKLEEVHARTACAGINEGMRAFPFWVYIFGASQSGKTTIANSVIIPSLL  800
ARO69859.1   721   LLEEGHKILVTAAGVPRKTSADFTMCIKEEVSKLEEVHARTACAGINEGMRAFPFWVYIFGASQSGKTTIANSVIIPSLL  800
ARO69905.1   721   LLEEGHKILVTAAGVPRKTSADFTMCIKEEVSKLEEVHARTACAGINEGMRAFPFWVYIFGASQSGKTTIANSVIIPALL  800
ARO69871.1   721   LLEEGHKILVTAAGVPRKTSADFTMCIKEEVSKLEEVHARTACAGINEGMRAFPFWVYIFGASQSGKTTIANSIIIPSLL  800
ARO69848.1   721   LLEEGHKILVTAAGVPRKTSADFTMCIKEEVSKLEEVHARTACAGINEGMRAFPFWVYIFGASQSGKTIMANSVIIPALL  800
ARO69874.1   721   LLEEGHKILVTAAGVPRKTSADFTMCIKEEVSKLEEVHARTACAGINEGMRAFPFWVYIFGASQSGKTTIANSVIIPSLL  800
ARO69882.1   721   LLEEGHKILVTAAGVPRKTSADFTMCIKEEVSKLEEVHARTACAGINEGMRAFPFWVYIFGASQSGKTTIANSIIIPSLL  800
QYU59221.1   721   LLEEGHKILVTAAGVPRKTSADFTMCIKEEVSKLEEVHARTACAGINEGMRAFPFWVYIFGASQSGKTTIANSVIIPALL  800
ARO69842.1   721   LLEEGHKILVTAAGVPRKTSADFTMCIKEEVSKLEEVHARTACAGINEGMRAFPFWVYIFGASQSGKTIMANSVIIPALL  800
AFV34731.1   721   LLEEGHKILVTAAGVPRKTSADFTMCIKEEVSKLEEVHARTECAGINEGMRAFPFWVYIFGASQSGKTTIANSVIIPALL  800
AND76470.1   721   LLEEGHKILVTAAGVPRKTSADFTMCIKEEVSKLEEVHARTACAGINEGMRAFPFWVYIFGASQSGKTIMANSVIIPALL  800
ACZ58632.1   721   LLEEGHKILVTAAGVPRKTSADFTMCIKEEVSKLEEVHARTACAGINEGMRAFPFWVYIFGASQSGKTTIANSVIIPALL  800
QBZ78374.1   721   LLEEGHKILVTAAGVPRKTSADFTMCIKEEVSKLEEVHARTACAGINEGMRAFPFWVYIYGASQSGKTTIANSVIIPALL  800
QGP72540.1   721   LLEEGHKILVTAAGVPRKTSADFTMCIKEEVSKLEEVHARTACAGINEGMRAFPFWVYIFGASQSGKTTIANSVIIPALL  800
QBZ78372.1   721   LLEEGHKILVTAAGVPRKTSADFTMCIKEEVSKLEEVHARTACAGINEGMRAFPFWVYIYGASQSGKTTIANSVIIPALL  800
QYU59224.1   721   LLEEGHKILVTAAGVPRKTSADFTMCIKEEVSKLEEVHARTACAGINEGMRAFPFWVYIFGASQSGKTTIANSVIIPALL  800
ARO69877.1   721   LLEEGHKILVTAAGVPRKTSADFTMCIKEEVSKLEEVHARTACAGINEGMRAFPFWVYIFGASQSGKTTIANSVIIPSLL  800
ARO69858.1   721   LLEEGHKILVTAAGVPRKTSADFTMCIKEEVSKLEEVHARTACAGINEGMRAFPFWVYIFGASQSGKTTIANSVIIPSLL  800
ARO69855.1   721   LLEEGHKILVTAAGVPRKTSADFTMCIKEEVSKLEEVHARTACAGINEGMRAFPFWVYIFGASQSGKTTIANSIIIPALL  800
QGP72542.1   721   LLEEGHKILVTAAGVPRKTSADFTMCIKEEVSKLEEVHARTACAGINEGMRAFPFWVYIFGASQSGKTTIANSVIIPALL  800
AYQ93055.1   721   LLEEGHKILVTAAGVPRKTSADFTMCIKEEVSKLEEVHARTACAGINEGMRAFPFWVYIFGASQSGKTTIANSVIIPALL  800
AYQ93053.1   721   LLEQGHKILVTAAGVPRKTSADFTMCIKEEVSKLEEVHARTACAGINEGMRAFPFWVYIYGASQSGKTTIANSVIIPALL  800
AYQ93051.1   721   LLEEGHKILVTAAGVPRKTSADFTMCIKEEVSKLEEVHARTACAGINEGMRAFPFWVYIFGASQSGKTTIANSIIIPALL  800
AFI71813.1   721   LLEEGHKILVTAAGVPRKTSADFTMCIKEEVSKLEEVHARTACAGINEGMRAFPFWVYIFGASQSGKTTIANSIIIPALL  800
QGP72539.1   721   LLEEGHKILVTAAGVPRKTSADFTMCIKEEVSKLEEVHARTACAGINEGMRAFPFWVYIFGASQSGKTTIANSVIIPALL  800
QYU59226.1   721   LLEEGHKILVTAAGVPRKTSADFTMCIKEEVSKLEEVHARTACAGINEGMRAFPFWVYIFGASQSGKTTIANSVIIPALL  800
ARO69852.1   721   LLEEGHKILVTAAGVPRKTSADFTMCIKEEVSKLEEVHARTACAGINEGMRAFPFWVYIFGASQSGKTTIANSVIIPSLL  800
QGP72543.1   721   LLEEGHKILVTAAGVPRKMSADFTMCIKEEVSKLEEVHAKTACAGINEGMRAFPFWVYIFGASQSGKTTIANSIIIPALL  800
QYU59219.1   721   LLEEGHKILVTAAGVPRKTSADFTMCIKEEVSKLEEVHARTACAGINEGMRAFPFWVYIYGASQSGKTTIANSVIIPALL  800
AGT42200.1   721   LLEEGHKILVTAAGVPRKTSADFTMCIKEEVSKLEEVHARTACAGINEGMRAFPFWVYIFGASQSGKTTIANSIIIPALL  800
ACZ58633.1   721   LLEEGHKILVTAAGVPRKTSADFTMCIKEEVSKLEEVHARTACAGINEGMRAFPFWVYIFGASQSGKTTIANSVIIPALL  800
QBZ78369.1   721   LLEEGHKILVTAAGVPRKTSADFTMCIKEEVSKLEEVHARTACAGINEGMRAFPFWVYIFGASQSGKTTIANSVIIPALL  800
ARO69851.1   721   LLEEGHKILVTAAGVPRKTSADFTMCIKEEVSKLEEVHARTACAGINEGMRAFPFWVYIYGASQSGKTNIANSVIIPALL  800
QYU59220.1   721   LLEEGHKILVTAAGVPRKTSADFTMCIKEEVSKLEEVHARTACAGINEGMRAFPFWVYIFGASQSGKTTIANSVIIPALL  800
AFV34755.1   721   LLEEGHKILVTAAGVPRKTSADFTMCIKEEVSKLEEVHARTACAGINEGMRAFPFWVYIFGASQSGKTTIANSVIIPALL  800
QGP72544.1   721   LLEEGHKILVTAAGVPRKTSADFTMCIKEEVSKLEEVHARTACAGINEGMRAFPFWVYIHGASQSGKTTIANSVIIPALL  800
QYA72352.1   721   LLDEGHRILVTAAGVPRKMSADFTMCIKEEVSKLEEVHAKTACAGINEGMRAFPFWVYIFGASQSGKTTIANSIIIPALL  800
ARO69854.1   721   LLEEGHKILVTAAGVPRKTSADFTMCIKEEVSKLEEVHARTACAGINEGMRAFPFWVYIFGASQSGKTTIANSIIIPALL  800
ARO69860.1   721   LLEEGHKILVTAAGVPRKTSADFTMCIKEEVSKLEEVHARTACAGINEGMRAFPFWVYIFGASQSGKTTIANSIIIPALL  800
ARO69863.1   721   LLEEGHKILVTAAGVPRKTSADFTMCIKEEVSKLEEVHARTACAGINEGMRAFPFWVYIFGASQSGKTTIANSIIIPALL  800
DAZ85707.1   721   LLEEGHKILVTAAGVPRKTSADFTMCIKEEVSKLEEVHARTACAGINEGMRAFPFWVYIFGASQSGKTTIANSIIIPALL  800
AWO67781.1   721   LLEEGHKILVTAAGVPRKTSADFTMCIKEEVSKLEEVHARTACAGINEGMRAFPFWVYIFGASQSGKTTIANSIIIPALL  800
QBZ78367.1   721   LLEEGHKILVTAAGVPRKTSADFTMCIKEEVSKLEEVHARTACAGINEGMRAFPFWVYIFGASQSGKTTIANSVIIPALL  800
ARO69873.1   721   LLEEGHKILVTAAGVPRKTSADFTMCIKEEVSKLEEVHARTACAGINEGMRAFPFWVYIFGASQSGKTTIANSIIIPALL  800
AND76471.1   721   LLEEGHKILVTAAGVPRKTSADFTMCIKEEVSKLEEVHARTACAGINEGMRAFPFWVYIFGASQSGKTTIANSIIIPALL  800
ARO69849.1   721   LLEEGHKILVTAAGVPRKTSADFTMCIKEEVSKLEEVHARTACAGINEGMRAFPFWVYIFGASQSGKTTIANSIIIPALL  800
ARO69889.1   721   LLEEGHKILVTAAGVPRKTSADFTMCIKEEVSKLEEVHARTACAGINEGMRAFPFWVYIFGASQSGKTTIANSIIIPALL  800
ARO69867.1   721   LLEEGHKILVTAAGVPRKTSADFTMCIKEEVSKLEEVHARTACAGINEGMRAFPFWVYIFGASQSGKTTIANSIIIPALL  800
ARO69904.1   721   LLEEGHKILVTAAGVPRKTSADFTMCIKEEVSKLEEVHARTACAGINEGMRAFPFWVYIFGASQSGKTTIANSIIIPALL  800
```

```
ARO69869.1   721   LLEEGHKILVTAAGVPRKTSADFTMCIKEEVSKLEEVHARTACAGINEGMRAFPFWVYIFGASQSGKTTIANSIIIPALL  800
ARO69884.1   721   LLEEGHKILVTAAGVPRKTSADFTMCIKEEVSKLEEVHARTACAGINEGMRAFPFWVYIFGASQSGKTTIANSIIIPALL  800
ARO69878.1   721   LLEEGHKILVTAAGVPRKTSADFTMCIKEEVSKLEEVHARTACAGINEGMRAFPFWVYIFGASQSGKTTIANSIIIPALL  800
ARO69885.1   721   LLEEGHKILVTAAGVPRKTSADFTMCIKEEVSKLEEVHARTACAGINEGMRAFPFWVYIFGASQSGKTTIANSIIIPALL  800
ARO69841.1   721   LLEEGHKILVTAAGVPRKTSADFTMCIKEEVSKLEEVHARTACAGINEGMRAFPFWVYIFGASQSGKTTIANSIIIPALL  800
ARO69891.1   721   LLEEGHKILVTAAGVPRKTSADFTMCIKEEVSKLEEVHARTACAGINEGMRAFPFWVYIFGASQSGKTTIANSIIIPALL  800
ARO69868.1   721   LLEEGHKILVTAAGVPRKTSADFTMCIKEEVSKLEEVHARTACAGINEGMRAFPFWVYIFGASQSGKTTIANSIIIPALL  800
ARO69900.1   721   LLEEGHKILVTAAGVPRKTSADFTMCIKEEVSKLEEVHARTACAGINEGMRAFPFWVYIFGASQSGKTTIANSIIIPALL  800
DAZ85706.1   721   LLEEGHKILVTAAGVPRKMSADFTMCIKEEVSKLEEVHARTACAGINEGMRAFPFWVYIFGASQSGKTTIANSIIIPALL  800
ARO69899.1   721   LLEEGHKILVTAAGVPRKTSADFTMCIKEEVSKLEEVHARTACAGINEGMRAFPFWVYIFGASQSGKTTIANSIIIPALL  800
ARO69898.1   721   LLEEGHKILVTAAGVPRKTSADFTMCIKEEVSKLEEVHARTACAGINEGMRAFPFWVYIFGASQSGKTTIANSIIIPALL  800
AYA22297.1   721   LLEEGHKILVTAAGVPRKTSADFTMCIKEEVSKLEEVHARTACAGINEGMRAFPFWVYIYGASQSGKTTIANSVIIPALL  800
QYU59222.1   721   LLEEGHKILVTAAGVPRKTSADFTMCIKEEVSKLEEVHARTACAGINEGMRAFPFWVYIFGASQSGKTTIANSVIIPALL  800
ARO69845.1   721   LLEEGHKILVTAAGVPRKTSADFTMCIKEEVSKLEEVHARTACAGINEGMRAFPFWVYIFGASQSGKTTIANSIIIPALL  800
AND76473.1   721   LLEEGHKILVTAAGVPRKTSADFTMCIKEEVSKLEEVHARTACAGINEGMRAFPFWVYIFGASQSGKTTIANSIIIPALL  800
ARO69857.1   721   LLEEGHKILVTAAGVPRKTSADFTMCIKEEVSKLEEVHARTACAGINEGMRAFPFWVYIFGASQSGKTTIANSIIIPALL  800
DAZ85708.1   721   LLEEGHKILVTAAGVPRKTSADFTMCIKEEVSKLEEVHARTACAGINEGMRAFPFWVYIFGASQSGKTTIANSIIIPALL  800
ARO69881.1   721   LLEEGHKILVTAAGVPRKTSADFTMCIKEEVSKLEEVHARTACAGINEGMRAFPFWVYIFGASQSGKTIMANSVIIPALL  800
ARO69893.1   721   LLEEGHKILVTAAGVPRKTSADFTMCIKEEVSKLEEVHARTACAGINEGMRAFPFWVYIFGASQSGKTIMANSVIIPALL  800
ARO69897.1   721   LLEEGHKILVTAAGVPRKTSADFTMCIKEEVSKLEEVHARTACAGINEGMRAFPFWVYIFGASQSGKTIMANSVIIPALL  800
ARO69872.1   721   LLEEGHKILVTAAGVPRKTSADFTMCIKEEVSKLEEVHARTACAGINEGMRAFPFWVYIFGASQSGKTIMANSVIIPALL  800
ARO69843.1   721   LLEEGHKILVTAAGVPRKTSADFTMCIKEEVSKLEEVHARTACAGINEGMRAFPFWVYIFGASQSGKTIMANSVIIPALL  800
ARO69886.1   721   LLEEGHKILVTAAGVPRKTSADFTMCIKEEVSKLEEVHARTACAGINEGMRAFPFWVYIFGASQSGKTIMANSVIIPALL  800
ARO69864.1   721   LLEEGHKILVTAAGVPRKTSADFTMCIKEEVSKLEEVHARTACAGINEGMRAFPFWVYIFGASQSGKTIMANSVIIPALL  800
ARO69876.1   721   LLEEGHKILVTAAGVPRKTSADFTMCIKEEVSKLEEVHARTACAGINEGMRAFPFWVYIFGASQSGKTIMANSVIIPALL  800
ARO69888.1   721   LLEEGHKILVTAAGVPRKTSADFTMCIKEEVSKLEEVHARTACAGINEGMRAFPFWVYIFGASQSGKTIMANSVIIPALL  800
ARO69853.1   721   LLEEGHKILVTAAGVPRKTSADFTMCIKEEVSKLEEVHARTACAGINEGMRAFPFWVYIFGASQSGKTIMANSVIIPALL  800
ARO69903.1   721   LLEEGHKILVTAAGVPRKTSADFTMCIKEEVSKLEEVHARTACAGINEGMRAFPFWVYIFGASQSGKTIMANSVIIPALL  800
ARO69865.1   721   LLEEGHKILVTAAGVPRKTSADFTMCIKEEVSKLEEVHARTACAGINEGMRAFPFWVYIFGASQSGKTIMANSVIIPALL  800
ARO69902.1   721   LLEEGHKILVTAAGVPRKTSADFTMCIKEEVSKLEEVHARTACAGINEGMRAFPFWVYIFGASQSGKTIMANSVIIPALL  800
ARO69861.1   721   LLEEGHKILVTAAGVPRKTSADFTMCIKEEVSKLEEVHARTACAGINEGMRAFPFWVYIFGASQSGKTIMANSVIIPALL  800
ARO69850.1   721   LLEEGHKILVTAAGVPRKTSADFTMCIKEEVSKLEEVHARTACAGINEGMRAFPFWVYIFGASQSGKTIMANSVIIPALL  800
QYU59227.1   721   LLEEGHKILVTAAGVPRKTSADFTMCIKEEVSKLEEVHARTACAGINEGMRAFPFWVYIFGASQSGKTTIANSIIIPALL  800
AFM91094.1   721   LLEEGHKILVTAAGVPRKTSADFTMCIKEEVSKLEEVHARTACAGINEGMRTFPFWVYIFGASQSGKTIMANSVVIPALL  800
ARO69883.1   721   LLEEGHKILVTAAGVPRKTSADFTMCIKEEVSKLEEVHARTACAGINEGMRAFPFWVYIFGASQSGKTIMANSVVIPALL  800
ARO69879.1   721   LLEEGHKILVTAAGVPRKTSADFTMCIKEEVSKLEEVHARTACAGINEGMRAFPFWVYIFGASQSGKTIMANSVIIPALL  800
AND76472.1   721   LLEEGHKILVTAAGVPRKTSADFTMCIKEEVSKLEEVHARTACAGINEGMRAFPFWVYIFGASQSGKTIMANSVIIPALL  800
ARO69846.1   721   LLEEGHKILVTAAGVPRKTSADFTMCIKEEVSKLEEVHARTACAGINEGMRAFPFWVYIFGASQSGKTIMANSVIIPALL  800
ARO69870.1   721   LLEEGHKILVTAAGVPRKTSADFTMCIKEEVSKLEEVHARTACAGINEGMRAFPFWVYIFGASQSGKTIMANSVVIPALL  800
ARO69856.1   721   LLEEGHKILVTAAGVPRKTSADFTMCIKEEVSKLEEVHARTACAGINEGMRAFPFWVYIFGASQSGKTIMANSVVIPALL  800
ARO69847.1   721   LLEEGHKILVTAAGVPRKTSADFTMCIKEEVSKLEEVHARTACAGINEGMRAFPFWVYIFGASQSGKTTIANSIIIPALL  800
ARO69875.1   721   LLEEGHKILVTAAGVPRKTSADFTMCIKEEVSKLEEVHARTACAGINEGMRAFPFWVYIFGASQSGKTTIANSIIIPALL  800
ARO69895.1   721   LLEEGHKILVTAAGVPRKTSADFTMCIKEEVSKLEEVHARTACAGINEGMRAFPFWVYIFGASQSGKTTIANSMIIPALL  800
ARO69880.1   721   LLEEGHKILVTAAGVPRKTSADFTMCIKEEVSKLEEVHARTACAGINEGMRAFPFWVYIFGASQSGKTTIANSIIIPALL  800
BCT87061.1   721   LLEDGHRILVTAAGVPRKTSADFTMCIKEEVSKLEEVHARTACAGVNEGMRAFPFWVYISGASQSGKTTIANSVIIPALL  800
QYU59225.1   721   LLEEGHKILVTAAGVPRKTSADFTMCIKEEVSKLEEVHARTACAGINEGMRAFPFWVYIFGASQSGKTTIANSVIIPALL  800
APD26306.1   721   LLEEGHKILVTAAGVPRKTSADFTMCIKEEVSKLEEVHARTACAGVNEGMRAFPFWVYITGASQSGKTTIANSIIIPALL  800


NP_619689.1  801   EEMNLPKSSVYSRPKTGGFWSGYARQACVKVDDFYAIEQTPSLASSMIDVVNSEPYPLDMAYIHEKGMSMDSPLVVTTAN  880
AWO68042.1   801   EEMNLPKSSVYSRPKTGGFWSGYARQACVKVDDFYAIEQTPSLASSMIDVVNSEPYPLDMAYIHEKGMSMDSPLVVTTAN  880
QYU59223.1   801   EEMNLPKSSVYSRPKTGGFWSGYARQACVKVDDFYAIEQTPSLASSMIDVVNSEPYPLDMAYIHEKGMSMDSPLVVTTAN  880
ARO69887.1   801   EEMNLPKSSVYSRPKTGGFWSGYARQACVKVDDFYAIEQTPSLASSMIDVVNSEPYPLDMAYIHEKGMSMDSPLVVTTAN  880
ARO69840.1   801   EEMNLPKSSVYSRPKTGGFWSGYARQACVKVDDFYAIEQTPSLASSMIDVVNSEPYPLDMAYIHEKGMSMDSPLVVTTAN  880
ARO69892.1   801   EEMNLPKSSVYSRPKTGGFWSGYARQACVKVDDFYAIEQTPSLASSMIDVVNSEPYPLDMAYIHEKGMSMDSPLVVTTAN  880
ARO69862.1   801   EEMNLPKSSVYSRPKTGGFWSGYARQACVKVDDFYAIEQTPSLASSMIDVVNSEPYPLDMAYIHEKGMSMDSPLVVTTAN  880
ARO69844.1   801   EEMNLPKSSVYSRPKTGGFWSGYARQACVKVDDFYAIEQTPSLASSMIDVVNSEPYPLDMAYIHEKGMSMDSPLVVTTAN  880
ARO69896.1   801   EEMNLPKSSVYSRPKTGGFWSGYARQACVKVDDFYAIEQTPSLASSMIDVVNSEPYPLDMAYIHEKGMSMDSPLVVTTAN  880
QGP72541.1   801   DEMNLPKSSVYSRPKTGGFWSGYARQACVKIDDFYAIEQTPSLASSMIDVVNSEPYPLDMAYIHEKGMSMDSPLVVTTAN  880
ARO69901.1   801   EEMNLPKSSVYSRPKTGGFWSGYARQACVKVDDFYAIEQTPSLASSMIDVVNSEPYPLDMAYIHEKGMSMDSPLVVTTAN  880
AYA22299.1   801   EEMNLPKSSVYSRPKTGGFWSGYARQACVKVDDFYAIEQTPSLASSMIDVVNSEPYPLDMAYIHEKGMSMDSPLVVTTAN  880
ARO69890.1   801   EEMNLPKSSVYSRPKTGGFWSGYARQACVKVDDFYAIEQTPSLASSMIDVVNSEPYPLDMAYIHEKGMSMDSPLVVTTAN  880
ARO69859.1   801   EEMNLPKSSVYSRPKTGGFWSGYARQACVKVDDFYAIEQTPSLASSMIDVVNSEPYPLDMAYIHEKGMSMDSPLVVTTAN  880
ARO69905.1   801   EEMNLPKSSVYSRPKTGGFWSGYARQACVKVDDFYAIEQTPSLASSMIDVVNSEPYPLDMAYIHEKGMSMDSPLVVTTAN  880
ARO69871.1   801   EEMNLPKSSVYSRPKTGGFWSGYARQACVKVDDFYAIEQTPSLASSMIDVVNSEPYPLDMAYIHEKGMSMDSPLVVTTAN  880
ARO69848.1   801   EEMNLPKSSVYSRPKTGGFWSGYARQACVKVDDFYAIEQTPSLASSMIDVVNSEPYPLDMAYIHEKGMSMDSPLVVTTAN  880
ARO69874.1   801   EEMNLPKSSVYSRPKTGGFWSGYARQACVKVDDFYAIEQTPSLASSMIDVVNSEPYPLDMAYIHEKGMSMDSPLVVTTAN  880
ARO69882.1   801   EEMNLPKSSVYSRPKTGGFWSGYARQACVKVDDFYAIEQTPSLASSMIDVVNSEPYPLDMAYIHEKGMSMDSPLVVTTAN  880
QYU59221.1   801   EEMNLPKSSVYSRPKTGGFWSGYARQACVKVDDFYAIEQTPSLASSMIDVVNSEPYPLDMAYIHEKGMSMDSPLVVTTAN  880
ARO69842.1   801   EEMNLPKSSVYSRPKTGGFWSGYARQACVKVDDFYAIEQTPSLASSMIDVVNSEPYPLDMAYIHEKGMSMDSPLVVTTAN  880
AFV34731.1   801   EEMNLPKSSVYSRPKTGGFWSGYARQACVKVDDFYAIEQTPSLASSMIDVVNSEPYPLDMAYIHEKGMSMDSPLVVTTAN  880
AND76470.1   801   EEMNLPKSSVYSRPKTGGFWSGYARQACVKVDDFYAIEQTPSLASSMIDVVNSEPYPLDMAYIHEKGMSMDSPLVVTTAN  880
ACZ58632.1   801   EEMNLPKSSVYSRPKTGGFWSGYARQACVKVDDFYAIEQTPSLASSMIDVVNSEPYPLDMAYIHEKGMSMDSPLVVTTAN  880
QBZ78374.1   801   EEMNLPKSSVYSRPKTGGFWSGYARQACVKVDDFYAIEQTPSLASSMIDVVNSEPYPLDMAYIHEKGMSMDSPLVVTTAN  880
QGP72540.1   801   EEMNLPKSSVYSRPKTGGFWSGYARQACVKVDDFYAIEQTPSLASSMIDVVNSEPYPLDMAYIHEKGMSMDSPLVVTTAN  880
QBZ78372.1   801   EEMNLPKSSVYSRPKTGGFWSGYARQACVKVDDFYAIEQTPSLASSMIDVVNSEPYPLDMAYIHEKGMSMDSPLVVTTAN  880
QYU59224.1   801   EEMNLPKSSVYSRPKTGGFWSGYARQACVKVDDFYAIEQTPSLASSMIDVVNSEPYPLDMAYIHEKGMSMDSPLVVTTAN  880
ARO69877.1   801   EEMNLPKSSVYSRPKTGGFWSGYARQACVKVDDFYAIEQTPSLASSMIDVVNSEPYPLDMAYIHEKGMSMDSPLVVTTAN  880
ARO69858.1   801   EEMNLPKSSVYSRPKTGGFWSGYARQACVKVDDFYAIEQTPSLASSMIDVVNSEPYPLDMAYIHEKGMSMDSPLVVTTAN  880
ARO69855.1   801   EEMNLPKSSVYSRPKTGGFWSGYARQACVKVDDFYAIEQTPSLASSMIDVVNSEPYPLDMAYIHEKGMSMDSPLVVTTAN  880
QGP72542.1   801   EEMNLPKSSVYSRPKTGGFWSGYARQACVKVDDFYAIEQTPSLASSMIDVVNSEPYPLDMAYIHEKGMSMDSPLVVTTAN  880
AYQ93055.1   801   EEMNLPKSSVYSRPKTGGFWSGYARQACVKVDDFYAIEQTPSLASSMIDVVNSEPYPLDMAYIHEKGMSMDSPLVVTTAN  880
AYQ93053.1   801   EEMNLPKSSVYSRPKTGGFWSGYARQACVKVDDFYAIEQTPSLASSMIDVVNSEPYPLDMAYIHEKGMSMDSPLVVTTAN  880
AYQ93051.1   801   EEMNLPKSSVYSRPKTGGFWSGYARQACVKVDDFYAIEQTPSLASSMIDVVNSEPYPLDMAYIHEKGMSMDSPLVVTTAN  880
AFI71813.1   801   DEMNLPKSSVYSRPKTGGFWSGYARQACVKVDDFYAIEQTPSLASSMIDVVNSEPYPLDMAYIHEKGMSMDSPLVVTTAN  880
QGP72539.1   801   EEMNLPKSSVYSRPKTGGFWSGYARQACVKVDDFYAIEQTPSLASSMIDVVNSEPYPLDMAYIHEKGMSMDSPLVVTTAN  880
QYU59226.1   801   EEMNLPKSSVYSRPKTGGFWSGYARQACVKVDDFYAIEQTPSLASSMIDVVNSEPYPLDMAYIHEKGMSMDSPLVVTTAN  880
ARO69852.1   801   EEMNLPKSSVYSRPKTGGFWSGYARQACVKVDDFYAIEQTPSLASSMIDVVNSEPYPLDMAYIHEKGMSMDSPLVVTTAN  880
```

```
QGP72543.1   801   EEMSLPKSSVYSRPKTGGFWSGYARQACVKVDDFYAIEQTPSLASSMIDVVNSEPYPLDMAYIHEKGMSMDSPLVVTTAN  880
QYU59219.1   801   EEMNLPKSSVYSRPKTGGFWSGYARQACVKVDDFYAIEQTPSLASSMIDVVNSEPYPLDMAYIHEKGMSMDSPLVVTTAN  880
AGT42200.1   801   DEMNLPKSSVYSRPKTGGFWSGYARQACVKVDDFYAIEQTPSLASSMIDVVNSEPYPLDMAYIHEKGMSMDSPLVVTTAN  880
ACZ58633.1   801   EEMNLPKSSVYSRPKTGGFWSGYARQACVKVDDFYAIEQTPSLASSMIDVVNSEPYPLDMAYIHEKGMSMDSPLVVTTAN  880
QBZ78369.1   801   EEMNLPKSSVYSRPKTGGFWSGYARQACVKVDDFYAIEQTPSLASSMIDVVNSEPYPLDMAYIHEKGMSMDSPLVVTTAN  880
ARO69851.1   801   EEMNLPKSSVYSRPKTGGFWSGYARQACVKVDDFYAIEQTPSLASSMIDVVNSEPYPLDMAYIHEKGMSMDSPLVVTTAN  880
QYU59220.1   801   EEMNLPKSSVYSRPKTGGFWSGYARQACVKVDDFYAIEQTPSLASSMIDVVNSEPYPLDMAYIHEKGMSMDSPLVVTTAN  880
AFV34755.1   801   EEMNLPKSSVYSRPKTGGFWSGYARQACVKVDDFYAIEQTPSLASSMIDVVNSEPYPLDMAYIHEKGMSMDSPLVVTTAN  880
QGP72544.1   801   EEMSLPKSSVYSRPKTGGFWSGYARQACVKVDDFYAIEQTPSLASSMIDVVNSEPYPLDMAYIHEKGMSMDSPLVVTTAN  880
QYA72352.1   801   EEMNLPKSSVYSRPKTGGFWSGYARQACVKVDDFYAIEQTPSLASSMIDVVNSEPYPLDMAYIHEKGMSMDSPLVVTTAN  880
ARO69854.1   801   EEMNLPKSSVYSRPKTGGFWSGYARQACVKVDDFYAIEQTPSLASSMIDVVNSEPYPLDMAYIHEKGMSMDSPLVVTTAN  880
ARO69860.1   801   EEMNLPKSSVYSRPKTGGFWSGYARQACVKVDDFYAIEQTPSLASSMIDVVNSEPYPLDMAYIHEKGMSMDSPLVVTTAN  880
ARO69863.1   801   EEMNLPKSSVYSRPKTGGFWSGYARQACVKVDDFYAIEQTPSLASSMIDVVNSEPYPLDMAYIHEKGMSMDSPLVVTTAN  880
DAZ85707.1   801   EEMNLPKSSVYSRPKTGGFWSGYARQACVKVDDFYAIEQTPSLASSMIDVVNSEPYPLDMAYIHEKGMSMDSPLVVTTAN  880
AWO67781.1   801   DEMNLPKSSVYSRPKTGGFWSGYARQACVKVDDFYAIEQTPSLASSMIDVVNSEPYPLDMAYIHEKGMSMDSPLVVTTAN  880
QBZ78367.1   801   EEMNLPKSSVYSRPKTGGFWSGYARQACVKVDDFYAIEQTPSLASSMIDVVNSEPYPLDMAYIHEKGMSMDSPLVVTTAN  880
ARO69873.1   801   EEMNLPKSSVYSRPKTGGFWSGYARQACVKVDDFYAIEQTPSLASSMIDVVNSEPYPLDMAYIHEKGMSMDSPLVVTTAN  880
AND76471.1   801   EEMNLPKSSVYSRPKTGGFWSGYARQACVKVDDFYAIEQTPSLASSMIDVVNSEPYPLDMAYIHEKGMSMDSPLVVTTAN  880
ARO69849.1   801   EEMNLPKSSVYSRPKTGGFWSGYARQACVKVDDFYAIEQTPSLASSMIDVVNSEPYPLDMAYIHEKGMSMDSPLVVTTAN  880
ARO69889.1   801   EEMNLPKSSVYSRPKTGGFWSGYARQACVKVDDFYAIEQTPSLASSMIDVVNSEPYPLDMAYIHEKGMSMDSPLVVTTAN  880
ARO69867.1   801   EEMNLPKSSVYSRPKTGGFWSGYARQACVKVDDFYAIEQTPSLASSMIDVVNSEPYPLDMAYIHEKGMSMDSPLVVTTAN  880
ARO69904.1   801   EEMNLPKSSVYSRPKTGGFWSGYARQACVKVDDFYAIEQTPSLASSMIDVVNSEPYPLDMAYIHEKGMSMDSPLVVTTAN  880
ARO69869.1   801   EEMNLPKSSVYSRPKTGGFWSGYARQACVKVDDFYAIEQTPSLASSMIDVVNSEPYPLDMAYIHEKGMSMDSPLVVTTAN  880
ARO69884.1   801   EEMNLPKSSVYSRPKTGGFWSGYARQACVKVDDFYAIEQTPSLASSMIDVVNSEPYPLDMAYIHEKGMSMDSPLVVTTAN  880
ARO69878.1   801   EEMNLPKSSVYSRPKTGGFWSGYARQACVKVDDFYAIEQTPSLASSMIDVVNSEPYPLDMAYIHEKGMSMDSPLVVTTAN  880
ARO69885.1   801   EEMNLPKSSVYSRPKTGGFWSGYARQACVKVDDFYAIEQTPSLASSMIDVVNSEPYPLDMAYIHEKGMSMDSPLVVTTAN  880
ARO69841.1   801   EEMNLPKSSVYSRPKTGGFWSGYARQACVKVDDFYAIEQTPSLASSMIDVVNSEPYPLDMAYIHEKGMSMDSPLVVTTAN  880
ARO69891.1   801   EEMNLPKSSVYSRPKTGGFWSGYARQACVKVDDFYAIEQTPSLASSMIDVVNSEPYPLDMAYIHEKGMSMDSPLVVTTAN  880
ARO69868.1   801   EEMNLPKSSVYSRPKTGGFWSGYARQACVKVDDFYAIEQTPSLASSMIDVVNSEPYPLDMAYIHEKGMSMDSPLVVTTAN  880
ARO69900.1   801   EEMNLPKSSVYSRPKTGGFWSGYARQACVKVDDFYAIEQTPSLASSMIDVVNSEPYPLDMAYIHEKGMSMDSPLVVTTAN  880
DAZ85706.1   801   DEMNLPKSSVYSRPKTGGFWSGYARQACVKVDDFYAIEQTPSLASSMIDVVNSEPYPLDMAYLHEKGMSMDSPLVVTTAN  880
ARO69899.1   801   EEMNLPKSSVYSRPKTGGFWSGYARQACVKVDDFYAIEQTPSLASSMIDVVNSEPYPLDMAYIHEKGMSMDSPLVVTTAN  880
ARO69898.1   801   EEMNLPKSSVYSRPKTGGFWSGYARQACVKVDDFYAIEQTPSLASSMIDVVNSEPYPLDMAYIHEKGMSMDSPLVVTTAN  880
AYA22297.1   801   DEMNLPKSSVYSRPKTGGFWSGYARQACVKVDDFYAIEQTPSLASSMIDVVNSEPYPLDMAYIHEKGMSMDSPLVVTTAN  880
QYU59222.1   801   EEMNLPKSSVYSRPKTGGFWSGYARQACVKVDDFYAIEQTPSLASSMIDVVNSEPYPLDMAYIHEKGMSMDSPLVVTTAN  880
ARO69845.1   801   EEMNLPKSSVYSRPKTGGFWSGYARQACVKVDDFYAIEQTPSLASSMIDVVNSEPYPLDMAYIHEKGMSMDSPLVVTTAN  880
AND76473.1   801   EEMNLPKSSVYSRPKTGGFWSGYARQACVKVDDFYAIEQTPSLASSMIDVVNSEPYPLDMAYIHEKGMSMDSPLVVTTAN  880
ARO69857.1   801   EEMNLPKSSVYSRPKTGGFWSGYARQACVKVDDFYAIEQTPSLASSMIDVVNSEPYPLDMAYIHEKGMSMDSPLVVTTAN  880
DAZ85708.1   801   EEMNLPKSSVYSRPKTGGFWSGYARQACVKVDDFYAIEQTPSLASSMIDVVNSEPYPLDMAYIHEKGMSMDSPLVVTTAN  880
ARO69881.1   801   EEMNLPKSSVYSRPKTGGFWSGYARQACVKVDDFYAIEQTPSLASSMIDVVNSEPYPLDMAYIHEKGMSMDSPLVVTTAN  880
ARO69893.1   801   EEMNLPKSSVYSRPKTGGFWSGYARQACVKVDDFYAIEQTPSLASSMIDVVNSEPYPLDMAYIHEKGMSMDSPLVVTTAN  880
ARO69897.1   801   EEMNLPKSSVYSRPKTGGFWSGYARQACVKVDDFYAIEQTPSLASSMIDVVNSEPYPLDMAYIHEKGMSMDSPLVVTTAN  880
ARO69872.1   801   EEMNLPKSSVYSRPKTGGFWSGYARQACVKVDDFYAIEQTPSLASSMIDVVNSEPYPLDMAYIHEKGMSMDSPLVVTTAN  880
ARO69843.1   801   EEMNLPKSSVYSRPKTGGFWSGYARQACVKVDDFYAIEQTPSLASSMIDVVNSEPYPLDMAYIHEKGMSMDSPLVVTTAN  880
ARO69886.1   801   EEMNLPKSSVYSRPKTGGFWSGYARQACVKVDDFYAIEQTPSLASSMIDVVNSEPYPLDMAYIHEKGMSMDSPLVVTTAN  880
ARO69864.1   801   EEMNLPKSSVYSRPKTGGFWSGYARQACVKVDDFYAIEQTPSLASSMIDVVNSEPYPLDMAYIHEKGMSMDSPLVVTTAN  880
ARO69876.1   801   EEMNLPKSSVYSRPKTGGFWSGYARQACVKVDDFYAIEQTPSLASSMIDVVNSEPYPLDMAYIHEKGMSMDSPLVVTTAN  880
ARO69888.1   801   EEMNLPKSSVYSRPKTGGFWSGYARQACVKVDDFYAIEQTPSLASSMIDVVNSEPYPLDMAYIHEKGMSMDSPLVVTTAN  880
ARO69853.1   801   EEMNLPKSSVYSRPKTGGFWSGYARQACVKVDDFYAIEQTPSLASSMIDVVNSEPYPLDMAYIHEKGMSMDSPLVVTTAN  880
ARO69903.1   801   EEMNLPKSSVYSRPKTGGFWSGYARQACVKVDDFYAIEQTPSLASSMIDVVNSEPYPLDMAYIHEKGMSMDSPLVVTTAN  880
ARO69865.1   801   EEMNLPKSSVYSRPKTGGFWSGYARQACVKVDDFYAIEQTPSLASSMIDVVNSEPYPLDMAYIHEKGMSMDSPLVVTTAN  880
ARO69902.1   801   EEMNLPKSSVYSRPKTGGFWSGYARQACVKVDDFYAIEQTPSLASSMIDVVNSEPYPLDMAYIHEKGMSMDSPLVVTTAN  880
ARO69861.1   801   EEMNLPKSSVYSRPKTGGFWSGYARQACVKVDDFYAIEQTPSLASSMIDVVNSEPYPLDMAYIHEKGMSMDSPLVVTTAN  880
ARO69850.1   801   EEMNLPKSSVYSRPKTGGFWSGYARQACVKVDDFYAIEQTPSLASSMIDVVNSEPYPLDMAYIHEKGMSMDSPLVVTTAN  880
QYU59227.1   801   EEMNLPKSSVYSRPKTGGFWSGYARQACVKVDDFYAIEQTPSLASSMIDVVNSEPYPLDMAYIHEKGMSMDSPLVVTTAN  880
AFM91094.1   801   EEMNLPKSSVYSRPKTGGFWSGYARQACVKIDDFYAIEQTPSLASSMIDVVNSEPYPLDMAYIHEKGMSMDSPLVVTTAN  880
ARO69883.1   801   EEMNLPKSSVYSRPKTGGFWSGYARQACVKVDDFYAIEQTPSLASSMIDVVNSEPYPLDMAYIHEKGMSMDSPLVVTTAN  880
ARO69879.1   801   EEMNLPKSSVYSRPKTGGFWSGYARQACVKVDDFYAIEQTPSLASSMIDVVNSEPYPLDMAYIHEKGMSMDSPLVVTTAN  880
AND76472.1   801   EEMSLPKSSVYSRPKTGGFWSGYARQACVKVDDFYAIEQTPSLASSMIDVVNSEPYPLDMAYIHEKGMSMDSPLVVTTAN  880
ARO69846.1   801   EEMNLPKSSVYSRPKTGGFWSGYARQACVKVDDFYAIEQTPSLASSMIDVVNSEPYPLDMAYIHEKGMSMDSPLVVTTAN  880
ARO69870.1   801   EEMNLPKSSVYSRPKTGGFWSGYARQACVKVDDFYAIEQTPSLASSMIDVVNSEPYPLDMAYIHEKGMSMDSPLVVTTAN  880
ARO69856.1   801   EEMNLPKSSVYSRPKTGGFWSGYARQACVKVDDFYAIEQTPSLASSMIDVVNSEPYPLDMAYIHEKGMSMDSPLVVTTAN  880
ARO69847.1   801   EEMNLPKSSVYSRPKTGGFWSGYARQACVKVDDFYAIEQTPSLASSMIDVVNSEPYPLDMAYIHEKGMSMDSPLVVTTAN  880
ARO69875.1   801   EEMNLPKSSVYSRPKTGGFWSGYARQACVKVDDFYAIEQTPSLASSMIDVVNSEPYPLDMAYIHEKGMSMDSPLVVTTAN  880
ARO69895.1   801   EEMNLPKSSVYSRPKTGGFWSGYARQACVKVDDFYAIEQTPSLASSMIDVVNSEPYPLDMAYIHEKGMSMDSPLVVTTAN  880
ARO69880.1   801   EEMNLPKSSVYSRPKTGGFWSGYARQACVKVDDFYAIEQTPSLASSMIDVVNSEPYPLDMAYIHEKGMSMDSPLVVTTAN  880
BCT87061.1   801   EEMNLPKSSVYSRPKTGGFWSGYARQACVKIDDFYAIEQTPSLASSMIDVVNSEPYPLDMAYIHEKGMSMDSPLVVTTAN  880
QYU59225.1   801   EEMNLPKSSVYSRPKTGGFWSGYARQACVKVDDFYAIEQTPSLASSMIDVVNSEPYPLDMAYIHEKGMSMDSPLVVTTAN  880
APD26306.1   801   EEMNLPKSSVYSRPKTGGFWSGYARQACVKIDDFYAIEQTPSLASSMIDVVNSEPYPLDMAYLHEKGMSMDSPLVVTTAN  880


NP_619689.1  881   TAVPPTNSQVVDLPSFYNRRAAVLEVRRKDGSFFTPRAYDSCIEVRFMHNKCPYVDSAGVPQGPAVNTPMDEGWITPSEA  960
AWO68042.1   881   TAVPPTNSQVVDLPSFYNRRAAVLEVRRKDGSFFTPRAYDSCIEVRFMHNKCPYVDSAGVPQGPAVNTPMDEGWITPSEA  960
QYU59223.1   881   TVVPPTNSQVVDLPSFYNRRAAVLEVRRKDGSFFAPRAYDSCIEVRFMHNKCPYVDSAGLPQGPAVNTPMDEGWITPSEA  960
ARO69887.1   881   TVVPPTNSQVVDLPSFYNRRAAVLEVRRKDGSFFAPRAYDSCIEVRFMHNKCPYVDSAGLPQGPAVNTPMDEGWITPSEA  960
ARO69840.1   881   TVVPPTNSQVVDLPSFYNRRAAVLEVRRKDGSFFTPRAYDSCIEVRFMHNKCPYVDSAGLPQGPAVNTPMDEGWITPSEA  960
ARO69892.1   881   TVVPPTNSQVVDLPSFYNRRAAVLEVRRKDGSFFTPRAYDSCIEVRFMHNKCPYVDSAGLPQGPAVNTPMDEGWITPSEA  960
ARO69862.1   881   TVVPPTNSQVVDLPSFYNRRAAVLEVRRKDGSFFTPRAYDSCIEVRFMHNKCPYVDSAGLPQGPAVNTPMDEGWITPSEA  960
ARO69844.1   881   TVVPPTNSQVVDLPSFYNRRAAVLEVRRKDGSFFTPRAYDSCIEVRFMHNKCPYVDSAGLPQGPAVNTPMDEGWITPSEA  960
ARO69896.1   881   TVVPPTNSQVVDLPSFYNRRAAVLEVRRKDGSFFTPRAYDSCIEVRFMHNKCPYVDSAGLPQGPAVNTPMDEGWITPSEA  960
QGP72541.1   881   TVVPPTNSQVVDLPSFYNRRAAVLEVRRKDGSFFTPRAYDSCTEVRFMHNKCPYVDSAGVPQGPSVNTPMDEGWITPSEA  960
ARO69901.1   881   TVVPPTNSQVVDLPSFYNRRAAVLEVRRKDGSFFTPRAYDSCIEVRFMHNKCPYVDSAGLPQGPAVNTPMDEGWITPSEA  960
AYA22299.1   881   TVVPPTNSQVVDLPSFYNRRAAVLEIRRKDGSFFAPRAYDSCIEVRFMHNKCPYVDSAGIPQGPAVNTPMDEGWITPSEA  960
ARO69890.1   881   TVVPPTNSQVVDLPSFYNRRAAVLEVRRKDGSFFTPRAYDSCIEVRFMHNKCPYVDSAGLPQGPAVNTPMDEGWITPSEA  960
ARO69859.1   881   TVVPPTNSQVVDLPSFYNRRAAVLEVRRKDGSFFTPRAYDSCIEVRFMHNKCPYVDSAGLPQGPAVNTPMDEGWITPSEA  960
ARO69905.1   881   TVVPPTNSQVVDLPSFYNRRAAVLEVRRKDGSFFAPREHDSCTEVRFMHNKCPYVDSAGVPQGPAVNTPMDEGWITPSEA  960
ARO69871.1   881   TVVPPTNSQVVDLPSFYNRRAAVLEVRRKDGSFFTPRAYDSCIEVRFMHNKCPYVDSAGLPQGPAVNTPMDEGWITPSEA  960
ARO69848.1   881   TVVPPTNSQVVDLPSFYNRRAAVLEVRRKDGSFFAPREHDSCTEVRFMHNKCPYVDSAGVPQGPAVNTPMDEGWITPSEA  960
```

ARO69874.1	881	TVVPPTNSQVVDLPSFYNRRAAVLEVRRKDGSFFAPREHDSCTEVRFMHNKCPYVDSAGVPQGPAVNTPMDEGWITPSEA	960
ARO69882.1	881	TVVPPTNSQVVDLPSFYNRRAAVLEVRRKDGSFFTPRAYDSCIEVRFMHNKCPYVDSAGLPQGPAVNTPMDEGWITPSEA	960
QYU59221.1	881	TVVPPTNSQVVDLPSFYNRRAAVLEVRRKDGSFFAPRAYDSCIEVRFMHNKCPYVDSAGVPQGPSVNTPMDEGWITPSEA	960
ARO69842.1	881	TVVPPTNSQVVDLPSFYNRRAAVLEVRRKDGSFFAPREHDSCTEVRFMHNKCPYVDSAGVPQGPAVNTPMDEGWITPSEA	960
AFV34731.1	881	TVVPPTNSQVVDLPSFYNRRAAVLEVRRKDGSFFTPRAYDSCIEVRFMHNKCPYVDSAGVPQGPAVNTPMDEGWITPSEA	960
AND76470.1	881	TVVPPTNSQVVDLPSFYNRRAAVLEVRRKDGSFFAPREHDSCTEVRFMHNKCPYVDSAGVPQGPAVNTPMDEGWITPSEA	960
ACZ58632.1	881	TVVPPTNSQVVDLPSFYNRRAAVLEVRRKDGSFFAPRAYDSCIEVRFVHNKCPYVDSAGVPQGPAVNTPMDEGWITPSEA	960
QBZ78374.1	881	TVVPPTNSQVVDLPSFYNRRAAVLEVRRKDGSFFAPRAYDSCIEVRFMHNKCPYVDSAGVPQGPAVNTPMDEGWITPSEA	960
QGP72540.1	881	TVVPPTNSQVVDLPSFYNRRAAVLEVRRKDGSFFAPRAYDSCTEVRFMHNKCPYVDSAGVPQGPSVNTPMDEGWVTPSEA	960
QBZ78372.1	881	TVVPPTNSQVVDLPSFYNRRAAVLEVRRKDGSFFAPRAYDSCIEVRFMHNKCPYVDSAGVPQGPAVNTPMDEGWITPSEA	960
QYU59224.1	881	TVVPPTNSQVVDLPSFYNRRAAVLEVRRKDGSFFAPRAYDSCIEVRFMHNKCPYVDSVGTPQGPAVNTPMDEGWITPSEA	960
ARO69877.1	881	TVVPPTNSQVVDLPSFYNRRAAVLEVRRKDGSFFTPRAYDSCIEVRFMHNKCPYVDSAGLPQGPAVNTPMDEGWITPSEA	960
ARO69858.1	881	TVVPPTNSQVVDLPSFYNRRAAVLEVRRKDGSFFTPRAYDSCIEVRFMHNKCPYVDSAGLPQGPAVNTPMDEGWITPSEA	960
ARO69855.1	881	TVVPPTNSQVVDLPSFYNRRAAVLEVRRKDGGLFLPREYDSCIEVRFMHNKCPYVDSKGVPQGPAVNTPMDEGWITPSEA	960
QGP72542.1	881	TVVPPTNSQVVDLPSFYNRRAAVLEVRRKDGSFFAPRAYDSCTEVRFMHNKCPYVDSAGVPQGPSVNTPMDEGWVTPSEA	960
AYQ93055.1	881	TVVPPTNSQVVDLPSFYNRRAAVLEVRRKDGSFFAPRAYDSCIEVRFMHNKCPYVDSAGVPQGPSVNTPMDEGWITPSEA	960
AYQ93053.1	881	TVVPPTNSQVVDLPSFYNRRAAVLEVRRKDGSFFAPRAYDSCIEVRFMHNKCPYVDSAGTPQGPAVNTPMDEGWITPSEA	960
AYQ93051.1	881	TVVPPTNSQVVDLPSFYNRRAAVLEVRRRDGSIFAPREYDSCIEVRFMHNKCPYVDSAGVPQGPAVNTPMDEGWITPSEA	960
AFI71813.1	881	TVVPPTNSQVVDLPSFYNRRAAVLEVRRKDGSFFTPRAYDSCIEVRFMHNKCPYVDSAGTPQGPAVNTPMDEGWITPSEA	960
QGP72539.1	881	TVVPPTNSQVVDLPSFYNRRAAVLEVRRKDGSFFAPRAYDSCTEVRFMHNKCPYVDSAGVPQGPSVNTPMDEGWVTPSEA	960
QYU59226.1	881	TVVPPTNSQVVDLPSFYNRRAAVLEVRRKDGSFFTPRAYDSCIEVRFMHNKCPYVDSAGVPQGPSVNTPMDEGWITPSEA	960
ARO69852.1	881	TVVPPTNSQVVDLPSFYNRRAAVLEVRRKDGSFFTPRAYDSCTEVRFMHNKCPYVDSAGLPQGPAVNTPMDEGWITPSEA	960
QGP72543.1	881	TVVPPTNSQVVDLPSFYNRRAAVLEVRRKDGGVFVPREYDSCIEVRFMHNKCPYIDSAGVPQGPAVNTPMDEGWITPSEA	960
QYU59219.1	881	TVVPPTNSQVVDLPSFYNRRAAVLEVRRKDGSFFAPRAYDSCIEVRFMHNKCPYVDSAGVPQGPAVNTPMDEGWITPSEA	960
AGT42200.1	881	TVVPPTNSQVVDLPSFYNRRAAVLEVRRKDGSFFAPRAYDSCIEVRFMHNKCPYVDSAGTPQGPAVNTPMDEGWITPSEA	960
ACZ58633.1	881	TVVPPTNSQVVDLPSFYNRRAAVLEVRRKDGSFFTPRAYDSCIEVRFMHTKCPYVDSAGVPQGPAVNTPMDEGWITPSEA	960
QBZ78369.1	881	TVVPPTNSQVVDLPSFYNRRAAVLEVRRKDGSFFAPRAYDSCIEVRFMHNKCPYVDSAGVPQGPSVNTPMDEGWITPSEA	960
ARO69851.1	881	TVVPPTNSQVVDLPSFYNRRAAVLEVRRKDGSFYTPRAYDSCTEVRFMHNKCPYVDSAGVPQGPAVNTPMDEGWITPSEA	960
QYU59220.1	881	TVVPPTNSQVVDLPSFYNRRAAVLEVRRKDGSFFTPRAYDSCIEVRFMHNKCPYVDSAGVPQGPAVNTPMDEGWITPSEA	960
AFV34755.1	881	TVVPPTNSQVVDLPSFYNRRAAVLEVRRKDGSFFAPRAYDSCIEVRFMHNKCPYVDSAGVPQGPSVNTPMDEGWITPSEA	960
QGP72544.1	881	TVVPPTNSQVVDLPSFYNRRAAVLEVRRKDGSFFAPRAYDSCIEVRFMHNKCPYVDSAGVPQGPAVNTPMDEGWITPSEA	960
QYA72352.1	881	TVVPPTNSQVVDLPSFYNRRAAVLEVRRKDGSFFEPWAYDSCIEVRFMHNKCPYVDSAGTPQGPAVNTPMDEGWISPSEA	960
ARO69854.1	881	TAVPPTNSQVVDLPSFYNRRAAVLEVRRKDGGLFSPREYDSCIEVRFMHNKCPYVDSKGVPQGPAVNTPMDEGWITPSEA	960
ARO69860.1	881	TAVPPTNSQVVDLPSFYNRRAAVLEVRRKDGGLFLPREYDSCIEVRFMHNKCPYVDSKGVPQGPAVNTPMDEGWITPSEA	960
ARO69863.1	881	TAVPPTNSQVVDLPSFYNRRAAVLEVRRKDGGLFSPREYDSCIEVRFMHNKCPYVDSKGVPQGPAVNTPMDEGWITPSEA	960
DAZ85707.1	881	TVVPPTNSQVVDLPSFYNRRAAVLEVRRKDGSFFAPREYDSCIEVRFMHNKCPYVDSAGLPQGPAVNTPMDEGWITPSEA	960
AWO67781.1	881	TVVPPTNSQVVDLPSFYNRRAAVLEVRRKDGSVFSPREYDSCIEVRFMHNKCPYVDSAGVPQGPAVNTPMDEGWISTSEA	960
QBZ78367.1	881	TVVPPTNSQVVDLPSFYNRRAAVLEVRRKDGSFFTPRVYDSCIEVRFMHAKCPYVDSAGVPQGPAVNTPMDEGWITPSEA	960
ARO69873.1	881	TAVPPTNSQVVDLPSFYNRRAAVLEVRRKDGGLFLPREYDSCIEVRFMHNKCPYVDSKGVPQGPAVNTPMDEGWITPSEA	960
AND76471.1	881	TAVPPTNSQVVDLPSFYNRRAAVLEVRRKDGGLFLPREYDSCIEVRFMHNKCPYVDSKGVPQGPAVNTPMDEGWITPSEA	960
ARO69849.1	881	TAVPPTNSQVVDLPSFYNRRAAVLEVRRKDGGLFSPREYDSCIEVRFMHNKCPYVDSKGVPQGPAVNTPMDEGWITPSEA	960
ARO69889.1	881	TAVPPTNSQVVDLPSFYNRRAAVLEVRRKDGGLFSPREYDSCIEVRFMHNKCPYVDSKGVPQGPAVNTPMDEGWITPSEA	960
ARO69867.1	881	TAVPPTNSQVVDLPSFYNRRAAVLEVRRKDGGLFSPREYDSCIEVRFMHNKCPYVDSKGVPQGPAVNTPMDEGWITPSEA	960
ARO69904.1	881	TAVPPTNSQVVDLPSFYNRRAAVLEVRRKDGGLFLPREYDSCIEVRFMHNKCPYVDSKGVPQGPAVNTPMDEGWITPSEA	960
ARO69869.1	881	TAVPPTNSQVVDLPSFYNRRAAVLEVRRKDGGLFLPREYDSCIEVRFMHNKCPYVDSKGVPQGPAVNTPMDEGWITPSEA	960
ARO69884.1	881	TAVPPTNSQVVDLPSFYNRRAAVLEVRRKDGGLFSPREYDSCIEVRFMHNKCPYVDSKGVPQGPAVNTPMDEGWITPSEA	960
ARO69878.1	881	TAVPPTNSQVVDLPSFYNRRAAVLEVRRKDGGLFLPREYDSCIEVRFMHNKCPYVDSKGVPQGPAVNTPMDEGWITPSEA	960
ARO69885.1	881	TAVPPTNSQVVDLPSFYNRRAAVLEVRRKDGGLFSPREYDSCIEVRFMHNKCPYVDSKGVPQGPAVNTPMDEGWITPSEA	960
ARO69841.1	881	TAVPPTNSQVVDLPSFYNRRAAVLEVRRKDGGLFSPREYDSCIEVRFMHNKCPYVDSKGVPQGPAVNTPMDEGWITPSEA	960
ARO69891.1	881	TAVPPTNSQVVDLPSFYNRRAAVLEVRRKDGGLFLPREYDSCIEVRFMHNKCPYVDSKGVPQGPAVNTPMDEGWITPSEA	960
ARO69868.1	881	TAVPPTNSQVVDLPSFYNRRAAVLEVRRKDGGLFLPREYDSCIEVRFMHNKCPYVDSKGVPQGPAVNTPMDEGWITPSEA	960
ARO69900.1	881	TAVPPTNSQVVDLPSFYNRRAAVLEVRRKDGGLFLPREYDSCIEVRFMHNKCPYVDSKGVPQGPAVNTPMDEGWITPSEA	960
DAZ85706.1	881	TVVPPTNSQVVDLPSFYNRRAAVLETRRKDGSIFEPGAYDSCIEVRFMHNKCPYVDSAGVPQGPAVNTPMDEGWISPSEA	960
ARO69899.1	881	TAVPPTNSQVVDLPSFYNRRAAVLEVRRKDGGLFLPREYDSCIEVRFMHNKCPYVDSKGVPQGPAVNTPMDEGWITPSEA	960
ARO69898.1	881	TAVPPTNSQVVDLPSFYNRRAAVLEVRRKDGGLFSPREYDSCIEVRFMHNKCPYVDSKGVPQGPAVNTPMDEGWITPSEA	960
AYA22297.1	881	TVVPPTNSQVVDLPSFYNRRAAVLEVRRTDGSFFAPRAYDSCIEVRFMHNKCPYVDSAGVPQGPAVNTPMDEGWITPSEA	960
QYU59222.1	881	TVVPPTNSQVVDLPSFYNRRAAVLEVRRKDGSFFSPRAYDSCIEVRFMHNKCPYVDSAGVPQGPSVNTPMDEGWITPSEA	960
ARO69845.1	881	TAVPPTNSQVVDLPSFYNRRAAVLEVRRKDGGLFSPREYDSCIEVRFMHNKCPYVDSKGVPQGPAVNTPMDEGWITPSEA	960
AND76473.1	881	TAVPPTNSQVVDLPSFYNRRAAVLEVRRKDGGLFLPREYDSCIEVRFMHNKCPYVDSKGVPQGPAVNTPMDEGWITPSEA	960
ARO69857.1	881	TAVPPTNSQVVDLPSFYNRRAAVLEVRRKDGGLFLPREYDSCIEVRFMHNKCPYVDSKGVPQGPAVNTPMDEGWITPSEA	960
DAZ85708.1	881	TAVPPTNSQVVDLPSFYNRRAAVLEVRRKDGSVFTPWEYDSCIEVRFMHSKCPYIDSAGVPQGPAVNTPMDEGWISTSEA	960
ARO69881.1	881	TVVPPTNSQVVDLPSFYNRRAAVLEVRRKDGSFFAPREHDSCTEVRFMHNKCPYVDSAGVPQGPAVNTPMDEGWITPSEA	960
ARO69893.1	881	TVVPPTNSQVVDLPSFYNRRAAVLEVRRKDGSFFAPREHDSCTEVRFMHNKCPYVDSAGVPQGPAVNTPMDEGWITPSEA	960
ARO69897.1	881	TVVPPTNSQVVDLPSFYNRRAAVLEVRRKDGSFFAPREHDSCTEVRFMHNKCPYVDSAGVPQGPAVNTPMDEGWITPSEA	960
ARO69872.1	881	TVVPPTNSQVVDLPSFYNRRAAVLEVRRKDGSFFAPREHDSCTEVRFMHNKCPYVDSAGVPQGPAVNTPMDEGWITPSEA	960
ARO69843.1	881	TVVPPTNSQVVDLPSFYNRRAAVLEVRRKDGSFFAPREHDSCTEVRFMHNKCPYVDSAGVPQGPAVNTPMDEGWITPSEA	960
ARO69886.1	881	TVVPPTNSQVVDLPSFYNRRAAVLEVRRKDGSFFAPREHDSCTEVRFMHNKCPYVDSAGVPQGPAVNTPMDEGWITPSEA	960
ARO69864.1	881	TVVPPTNSQVVDLPSFYNRRAAVLEVRRKDGSFFAPREHDSCTEVRFMHNKCPYVDSAGVPQGPAVNTPMDEGWITPSEA	960
ARO69876.1	881	TVVPPTNSQVVDLPSFYNRRAAVLEVRRKDGSFFAPREHDSCTEVRFMHNKCPYVDSAGVPQGPAVNTPMDEGWITPSEA	960
ARO69888.1	881	TVVPPTNSQVVDLPSFYNRRAAVLEVRRKDGSFFAPREHDSCTEVRFMHNKCPYVDSAGVPQGPAVNTPMDEGWITPSEA	960
ARO69853.1	881	TVVPPTNSQVVDLPSFYNRRAAVLEVRRKDGSFFAPREHDSCTEVRFMHNKCPYVDSAGVPQGPAVNTPMDEGWITPSEA	960
ARO69903.1	881	TVVPPTNSQVVDLPSFYNRRAAVLEVRRKDGSFFAPREHDSCTEVRFMHNKCPYVDSAGVPQGPAVNTPMDEGWITPSEA	960
ARO69865.1	881	TVVPPTNSQVVDLPSFYNRRAAVLEVRRKDGSFFAPREHDSCTEVRFMHNKCPYVDSAGVPQGPAVNTPMDEGWITPSEA	960
ARO69902.1	881	TVVPPTNSQVVDLPSFYNRRAAVLEVRRKDGSFFAPREHDSCTEVRFMHNKCPYVDSAGVPQGPAVNTPMDEGWITPSEA	960
ARO69861.1	881	TVVPPTNSQVVDLPSFYNRRAAVLEVRRKDGSFFAPREHDSCTEVRFMHNKCPYVDSAGVPQGPAVNTPMDEGWITPSEA	960
ARO69850.1	881	TVVPPTNSQVVDLPSFYNRRAAVLEVRRKDGSFFAPREHDSCTEVRFMHNKCPYVDSAGVPQGPAVNTPMDEGWITPSEA	960
QYU59227.1	881	TAVPPTNSQVVDLPSFYNRRAAVLEVRRKDGSFFAPREYDSCIEVRFMHNKCPYVDSAGVPQGPAVNTPMDEGWITPSEA	960
AFM91094.1	881	TVVPPTNSQVVDLPSFYNRRAAVLEVRRKDGSFFAPRDHDSCTEVRFMHNKCPYVDSAGVPQGPAVNTPMDEGWITPSEA	960
ARO69883.1	881	TVVPPTNSQVVDLPSFYNRRAAVLEVRRKDGSFFAPREHDSCTEVRFMHNKCPYVDSAGVPQGPAVNTPMDEGWITPSEA	960
ARO69879.1	881	TVVPPTNSQVVDLPSFYNRRAAVLEVRRKDGSFFAPREHDSCTEVRFMHNKCPYVDSAGVPQGPAVNTPMDEGWITPSEA	960
AND76472.1	881	TVVPPTNSQVVDLPSFYNRRAAVLEVRRKDGSFFAPREHDSCTEVRFMHNKCPYVDSAGVPQGPAVNTPMDEGWITPSEA	960
ARO69846.1	881	TVVPPTNSQVVDLPSFYNRRAAVLEVRRKDGSFFAPREHDSCTEVRFMHNKCPYVDSAGVPQGPAVNTPMDEGWITPSEA	960
ARO69870.1	881	TVVPPTNSQVVDLPSFYNRRAAVLEVRRKDGSFFAPREHDSCTEVRFMHNKCPYVDSAGVPQGPAVNTPMDEGWITPSEA	960
ARO69856.1	881	TVVPPTNSQVVDLPSFYNRRAAVLEVRRKDGSFFAPREHDSCTEVRFMHNKCPYVDSAGVPQGPAVNTPMDEGWITPSEA	960
ARO69847.1	881	TVVPPTNSQVVDLPSFYNRRAAVLEVRRKDGGLFLPREYDSCIEVRFMHNKCPYVDSKGVPQGPAVNTPMDEGWITPSEA	960
ARO69875.1	881	TVVPPTNSQVVDLPSFYNRRAAVLEVRRKDGGLFLPREYDSCIEVRFMHNKCPYVDSKGVPQGPAVNTPMDEGWITPSEA	960
ARO69895.1	881	TVVPPTNSQVVDLPSFYNRRAAVLEVRRKDGGLFLPREYDSCIEVRFMHNKCPYVDSKGVPQGPAVNTPMDEGWITPSEA	960
ARO69880.1	881	TVVPPTNSQVVDLPSFYNRRAAVLEVRRKDGGLFLPREYDSCIEVRFMHNKCPYVDSKGVPQGPAVNTPMDEGWITPSEA	960

```
BCT87061.1   881   TVVPPTNSQVVDLPSFYNRRAAVLEVRRKDGSYFTPRAYDSCTEVRFLHNKCPYVDSMGIPQGPAVNTPMDEGWISPSEA  960
QYU59225.1   881   TVVPPTNSQVVDLPSFYNRRAAVLEVRRKDGSYFAPRAYDSCIEVRFMHNKCPYVDSTGLPQGPAVNTPMDEGWMSPSEA  960
APD26306.1   881   TAVPPTNSQVVDLPSFYNRRAAVLEVRRKDGSCFTPSDYDSCIEVRFLHSKCPYVDSAGVPQGPAVNTPMEEGWITPSEA  960


NP_619689.1  961   VAVLKNLLGEHILAEEAKLLEYRERIGNDHPIYNAAKEFIGNMHYPGQWLTAEQKSTYGIKDDGFSFLAVDGKIYKYNVL  1040
AWO68042.1   961   VAVLKNLLGEHILAEEAKLLEYRERIGNDHPIYNAAKEFIGNMHYPGQWLTAEQKSTYGIKDDGFSFLAVDGKIYKYNVL  1040
QYU59223.1   961   VAVLKNLLGEHILSEEEKLLEYRERIGNDHPIYNAAKEFIGNMHYPGQWLTAEQKSTYGIKDDGFSFLAVDGKMYKYNVL  1040
ARO69887.1   961   VAVLKNLLGEHILAEEEKLLEYRERIGNDHPIYNAAKEFIGNMHYPGQWLTAEQKSTYGIKDDGFSFLAVDGKMYKYNVL  1040
ARO69840.1   961   VAVLKNLLGEHILAEEEKLLEYRERIGNDHPIYNAAKEFIGNMHYPGQWLTAEQKSTYGIKDDGFSFLAVDGKMYKYNVL  1040
ARO69892.1   961   VAVLKNLLGEHILAEEEKLLEYRERIGNDHPIYNAAKEFIGNMHYPGQWLTAEQKSTYGIKDDGFSFLAVDGKMYKYNVL  1040
ARO69862.1   961   VAVLKNLLGEHILAEEEKLLEYRERIGNDHPIYNAAKEFIGNMHYPGQWLTAEQKSTYGIKDDGFSFLAVDGKMYKYNVL  1040
ARO69844.1   961   VAVLKNLLGEHILAEEEKLLEYRERIGNDHPIYNAAKEFIGNMHYPGQWLTAEQKSTYGIKDDGFSFLAVDGKMYKYNVL  1040
ARO69896.1   961   VAVLKNLLGEHILAEEEKLLEYRERIGNDHPIYNAAKEFIGNMHYPGQWLTAEQKSTYGIKDDGFSFLAVDGKMYKYNVL  1040
QGP72541.1   961   VAVLKNLLGEHILSEEEKLLEYRERIGNDHPIYNAAKEFIGNMHYPGQWLTTEQKNTYGIKDDGFSFLAVDGKMYKYNVL  1040
ARO69901.1   961   VAVLKNLLGEHILAEEEKLLEYRERIGNDHPIYNAAKEFIGNMHYPGQWLTAEQKSTYGIKDDGFSFLAVDGKMYKYNVL  1040
AYA22299.1   961   VAVLKNLLGEHILAEEEKLLEYRERIGNDHPIYNAAKEFIGNMHYPGQWLTAEQKSTYGIKDDGFSFLAVDGKMYKYNVL  1040
ARO69890.1   961   VAVLKNLLGEHILAEEEKLLEYRERIGNDHPIYNAAKEFIGNMHYPGQWLTAEQKSTYGIKDDGFSFLAVDGKMYKYNVL  1040
ARO69859.1   961   VAVLKNLLGEHILAEEEKLLEYRERIGNDHPIYNAAKEFIGNMHYPGQWLTAEQKSTYGIKDDGFSFLAVDGKMYKYNVL  1040
ARO69905.1   961   VAVLKNLLGEHILAEEEKLLEYRERIGNDHPIYNAAKEFIGNMHYPGQWLTAEQKSTYGIKDDGFSFLAVDGKMYKYNVL  1040
ARO69871.1   961   VAVLKNLLGEHILAEEEKLLEYRERIGNDHPIYNAAKEFIGNMHYPGQWLTAEQKSTYGIKDDGFSFLAVDGKMYKYNVL  1040
ARO69848.1   961   VAVLKNLLGEHILAEEEKLLEYRERIGNDHPIYNAAKEFIGNMHYPGQWLTAEQKSTYGIKDDGFSFLAVDGKMYKYNVL  1040
ARO69874.1   961   VAVLKNLLGEHILAEEEKLLEYRERIGNDHPIYNAAKEFIGNMHYPGQWLTAEQKSTYGIKDDGFSFLAVDGKMYKYNVL  1040
ARO69882.1   961   VAVLKNLLGEHILAEEEKLLEYRERIGNDHPIYNAAKEFIGNMHYPGQWLTAEQKSTYGIKDDGFSFLAVDGKMYKYNVL  1040
QYU59221.1   961   VAVLKNLLGEHILAEEEKLLEYRERIGNDHPIYNAAKEFIGNMHYPGQWLTAEQKSTYGIKDDGFSFLAVDGKMYKYNVL  1040
ARO69842.1   961   VAVLKNLLGEHILAEEEKLLEYRERIGNDHPIYNAAKEFIGNMHYPGQWLTAEQKSTYGIKDDGFSFLAVDGKMYKYNVL  1040
AFV34731.1   961   VAVLKNLLGEHILAEEEKLLEYRERIGNDHPIYNAAKEFIGNMHYPGQWLTAEQKNTYGIKDDGFSFLAVDGRMYKYNVL  1040
AND76470.1   961   VAVLKNLLGEHILAEEEKLLEYRERIGNDHPIYNAAKEFIGNMHYPGQWLTAEQKSTYGIKDDGFSFLAVDGKMYKYNVL  1040
ACZ58632.1   961   VAVLKNLLGEHILAEEEKLLEYRERIGNDHPIYNAAKEFIGNMHYPGQWLTAEQKSTYGIKDDGFSFLAVDGRMYKYNVL  1040
QBZ78374.1   961   VAVLKNLLGEHILSEEEKLLEYRERIGNDHPIYNAAKEFIGNMHYPGQWLTAEQKSTYGIKDDGFSFLAVDGKMYKYNVL  1040
QGP72540.1   961   VAVLKNLLGEHILAEEEKLLEYRERIGNDHPIYNAAKEFIGNMHYPGQWLTAEQKSTYGIKDDGFSFLAVDGKMYKYNVL  1040
QBZ78372.1   961   VAVLKNLLGEHILSEEEKLLEYRERIGNDHPIYNAAKEFIGNMHYPGQWLTAEQKSTYGIKDDGFSFLAVDGKMYKYNVL  1040
QYU59224.1   961   VAVLKNLLGEHILAEEEKLLEYRERIGNDHPIYNAAKEFIGNMHYPGQWLTAEQKSTYGIKDDGFSFLAVDGKMYKYNVL  1040
ARO69877.1   961   VAVLKNLLGEHILAEEEKLLEYRERIGNDHPIYNAAKEFIGNMHYPGQWLTAEQKSTYGIKDDGFSFLAVDGKMYKYNVL  1040
ARO69858.1   961   VAVLKNLLGEHILAEEEKLLEYRERIGNDHPIYNAAKEFIGNMHYPGQWLTAEQKSTYGIKDDGFSFLAVDGKMYKYNVL  1040
ARO69855.1   961   VSVLKNLLGDHILTEEEKLLEYRERIGNDHPIYNAAKEFIGNMHYPGQWLTAEQKSTYGIKDDGFSFLAVDGKMYKYNVL  1040
QGP72542.1   961   VAVLKNLLGEHILAEEEKLLEYRERIGNDHPIYNAAKEFIGNMHYPGQWLTAEQKSTYGIKDDGFSFLAVDGKMYKYNVL  1040
AYQ93055.1   961   VAVLKNLLGEHILAEEEKLLEYRERIGNDHPIYNAAKEFIGNMHYPGQWLTAEQKSTYGIKDDGFSFLAVDGKMYKYNVL  1040
AYQ93053.1   961   VAALKNLLGEHVLAEEEKLLEYRERIGNDHPIYNAAKEFIGNMHYPGQWLTAEQKNTYGIKDDGFSFLAVDGKMYKYNVL  1040
AYQ93051.1   961   VAVLKNLLGEHILAEEEKLLDYRERIGHDHPIYNAAKEFIGNMHYPGQWLTTEQKNTYGIKDDGFSFLAVDGKMYKYNVL  1040
AFI71813.1   961   VAVLKNLLGEHILAEEAKLLEYRERIGNDHPIYTAAKEFIGNMHYPGQWLTTEQKNTYGIKDDGFSFLAVDGKMYKYNVL  1040
QGP72539.1   961   VAVLKNLLGEHILAEEEKLLEYRERIGNDHPIYNAAKEFIGNMHYPGQWLTAEQKSTYGIKDDGFSFLAVDGKMYKYNVL  1040
QYU59226.1   961   VAVLKNLLGEHILAEEEKLLEYRERIGNDHPIYNAAKEFIGNMHYPGQWLTAEQKSTYGIKDDGFSFLAVDGKMYKYNVL  1040
ARO69852.1   961   VAVLKNLLGEHILAEEEKLLEYRERIGNDHPIYNAAKEFIGNMHYPGQWLTAEQKSTYGIKDDGFSFLAVDGKMYKYNVL  1040
QGP72543.1   961   VAVLKNLLGEHILIEEEKLLEYRERIGNDHPIYNAAKEFIGNMHYPGQWLTAEQKNTYGIKDDGFSFLAVDGKMYKYNVL  1040
QYU59219.1   961   VAVLKNLLGEHILAEEEKLLEYRERIGNDHPIYNAAKEFIGNMHYPGQWLTAEQKSTYGIKDDGFSFLAVDGKMYKYNVL  1040
AGT42200.1   961   VAVLKNLLGEHILAEEAKLLEYRERIGNDHPIYTAAKEFIGNMHYPGQWLTTEQKNTYGIKDDGLSFLAVDGKMYKYNVL  1040
ACZ58633.1   961   VAVLKNLLGEHILAEEEKLLEYRERIGNDHPIYNAAKEFIGNMHYPGQWLTAEQKSTYGIKDDGFSFLAVDGKMYKYNVL  1040
QBZ78369.1   961   VAVLKNLLGEHILAEEEKLLEYRERIGNDHPIYNAAKEFIGNMHYPGQWLTAEQKSTYGIKDDGFSFLAVDGKMYKYNVL  1040
ARO69851.1   961   VAVLKNLLGEHILAEEEKLLEYRERIGNDHPIYNAAKEFIGNMHYPGQWLTAEQKNTYGIKDDGFSFLAVDGKMYKYNVL  1040
QYU59220.1   961   VAALKNLLGEHILAEEEKLLEYRERIGNDHPIYNAAKEFIGNMHYPGQWLTAEQKNTYGIKDDGFSFLAVDGKMYRYNVL  1040
AFV34755.1   961   VAVLKNLLGEHILAEEEKLLEYRERIGNDHPIYNAAKEFIGNMHYPGQWLTAEQKSTYGIKDDGFSFLAVDGKMYKYNVL  1040
QGP72544.1   961   VAVLKNLLGEHILAEEEKLLEYRERIGNDHPIYNAAKEFIGNMHYPGQWLTTEQKGVYGIKDDGFSFLAVDGKMYKYNVL  1040
QYA72352.1   961   VAVLKNLLGEHILAEEAKLLEYRERIGNDHPIYNAAKEFIGNMHYPGQWLTTEQKSTYGIKDDGFSFLAVDGKMYKYNVL  1040
ARO69854.1   961   VSVLKNLLGDHILIEEEKLLEYRERIGNDHPIYNAAKEFIGNMHYPGQWLTAEQKNTYGIKDDGFSFLAVDGKMYKYNVL  1040
ARO69860.1   961   VSVLKNLLGDHILIEEEKLLEYRERIGNDHPIYNAAKEFIGNMHYPGQWLTAEQKNTYGIKDDGFSFLAVDGKMYKYNVL  1040
ARO69863.1   961   VSVLKNLLGDHILIEEEKLLEYRERIGNDHPIYNAAKEFIGNMHYPGQWLTAEQKNTYGIKDDGFSFLAVDGKMYKYNVL  1040
DAZ85707.1   961   VAVLKNLLGEHILAEEEKLLEYRERIGADHPIYNAAKEFIGNMHYPGQWLTTEQKSTYGIKDDGFSFLAVDGKMYKYNVL  1040
AWO67781.1   961   VAVLKNLLGEHILAEEAKLLEYRERIGNDHPIYNAAKEFIGNMHYPGQWLTAEQKSTYGIKDDGFSFLAVDGKMYKYNVL  1040
QBZ78367.1   961   VAALKNLLGEHILAEEEKLLEYRERIGNDHPIYNAAKEFIGNMHYPGQWLTAEQKSTYGIKDDGFSFLAVDGKMYKYNVL  1040
ARO69873.1   961   VSVLKNLLGDHILIEEEKLLEYRERIGNDHPIYNAAKEFIGNMHYPGQWLTAEQKNTYGIKDDGFSFLAVDGKMYKYNVL  1040
AND76471.1   961   VSVLKNLLGDHILIEEEKLLEYRERIGNDHPIYNAAKEFIGNMHYPGQWLTAEQKNTYGIKDDGFSFLAVDGKMYKYNVL  1040
ARO69849.1   961   VSVLKNLLGDHILIEEEKLLEYRERIGNDHPIYNAAKEFIGNMHYPGQWLTAEQKNTYGIKDDGFSFLAVDGKMYKYNVL  1040
ARO69889.1   961   VSVLKNLLGDHILIEEEKLLEYRERIGNDHPIYNAAKEFIGNMHYPGQWLTAEQKNTYGIKDDGFSFLAVDGKMYKYNVL  1040
ARO69867.1   961   VSVLKNLLGDHILIEEEKLLEYRERIGNDHPIYNAAKEFIGNMHYPGQWLTAEQKNTYGIKDDGFSFLAVDGKMYKYNVL  1040
ARO69904.1   961   VSVLKNLLGDHILIEEEKLLEYRERIGNDHPIYNAAKEFIGNMHYPGQWLTAEQKNTYGIKDDGFSFLAVDGKMYKYNVL  1040
ARO69869.1   961   VSVLKNLLGDHILIEEEKLLEYRERIGNDHPIYNAAKEFIGNMHYPGQWLTAEQKNTYGIKDDGFSFLAVDGKMYKYNVL  1040
ARO69884.1   961   VSVLKNLLGDHILIEEEKLLEYRERIGNDHPIYNAAKEFIGNMHYPGQWLTAEQKNTYGIKDDGFSFLAVDGKMYKYNVL  1040
ARO69878.1   961   VSVLKNLLGDHILIEEEKLLEYRERIGNDHPIYNAAKEFIGNMHYPGQWLTAEQKNTYGIKDDGFSFLAVDGKMYKYNVL  1040
ARO69885.1   961   VSVLKNLLGDHILIEEEKLLEYRERIGNDHPIYNAAKEFIGNMHYPGQWLTAEQKNTYGIKDDGFSFLAVDGKMYKYNVL  1040
ARO69841.1   961   VSVLKNLLGDHILIEEEKLLEYRERIGNDHPIYNAAKEFIGNMHYPGQWLTAEQKNTYGIKDDGFSFLAVDGKMYKYNVL  1040
ARO69891.1   961   VSVLKNLLGDHILIEEEKLLEYRERIGNDHPIYNAAKEFIGNMHYPGQWLTAEQKNTYGIKDDGFSFLAVDGKMFKYNVL  1040
ARO69868.1   961   VSVLKNLLGDHILIEEEKLLEYRERIGNDHPIYNAAKEFIGNMHYPGQWLTAEQKNTYGIKDDGFSFLAVDGKMFKYNVL  1040
ARO69900.1   961   VSVLKNLLGDHILIEEEKLLEYRERIGNDHPIYNAAKEFIGNMHYPGQWLTAEQKNTYGIKDDGFSFLAVDGKMYKYNVL  1040
DAZ85706.1   961   VAVLKNLLGEHILLEEQKLLEYRERIGNDHPIYNAAKEFIGNMHYPGQWLTAEQKNTYGIKDDGFSFLAVDGKMYKYNVL  1040
ARO69899.1   961   VSVLKNLLGDHILIEEEKLLEYRERIGNDHPIYNAAKEFIGNMHYPGQWLTAEQKNTYGIKDDGFSFLAVDGKMFKYNVL  1040
ARO69898.1   961   VSVLKNLLGDHILIEEEKLLEYRERIGNDHPIYNAAKEFIGNMHYPGQWLTAEQKNTYGIKDDGFSFLAVDGKMYKYNVL  1040
AYA22297.1   961   VAVLKNLLGDHILAEEEKLLEYRERIGNDHPIYNAAKEFIGNMHYPGQWLTTEQKNTYGIKDDGFSFLAVDGKMYKYNVL  1040
QYU59222.1   961   VAVLKNLLGEHILAEEEKLLEYRERIGNDHPIYNAAKEFIGNMHYPGQWLTAEQKSTYGIKDDGFSFLAVDGKMYKYNVL  1040
ARO69845.1   961   VSVLKNLLGDHILIEEEKLLEYRERIGNDHPIYNAAKEFIGNMHYPGQWLTAEQKNTYGIKDDGFSFLAVDGKMYKYNVL  1040
AND76473.1   961   VSVLKNLLGDHILIEEEKLLEYRERIGNDHPIYNAAKEFIGNMHYPGQWLTAEQKNTYGIKDDGFSFLAVDGKMFKYNVL  1040
ARO69857.1   961   VSVLKNLLGDHILIEEEKLLEYRERIGNDHPIYNAAKEFIGNMHYPGQWLTAEQKNTYGIKDDGFSFLAVDGKMFKYNVL  1040
DAZ85708.1   961   VAVLKNLLGEHILAEEAKLLEYRERIGSDHPIYNAAKEFIGNMHYPGQWLTTEQKNTYGIKDDGFSFLAVDGKMYKYNVL  1040
ARO69881.1   961   VAVLKNLLGEHILAEEEKLLEYRERIGNDHPIYNAAKEFIGNMHYPGQWLTAEQKSTYGIKDDGFSFLAVDGKMYKYNVL  1040
ARO69893.1   961   VAVLKNLLGEHILAEEEKLLEYRERIGNDHPIYNAAKEFIGNMHYPGQWLTAEQKSTYGIKDDGFSFLAVDGKMYKYNVL  1040
ARO69897.1   961   VAVLKNLLGEHILAEEEKLLEYRERIGNDHPIYNAAKEFIGNMHYPGQWLTAEQKSTYGIKDDGFSFLAVDGKMYKYNVL  1040
ARO69872.1   961   VAVLKNLLGEHILAEEEKLLEYRERIGNDHPIYNAAKEFIGNMHYPGQWLTAEQKSTYGIKDDGFSFLAVDGKMYKYNVL  1040
ARO69843.1   961   VAVLKNLLGEHILAEEEKLLEYRERIGNDHPIYNAAKEFIGNMHYPGQWLTAEQKSTYGIKDDGFSFLAVDGKMYKYNVL  1040
```

```
ARO69886.1   961   VAVLKNLLGEHILAEEEKLLEYRERIGNDHPIYNAAKEFIGNMHYPGQWLTAEQKSTYGIKDDGFSFLAVDGKMYKYNVL  1040
ARO69864.1   961   VAVLKNLLGEHILAEEEKLLEYRERIGNDHPIYNAAKEFIGNMHYPGQWLTAEQKSTYGIKDDGFSFLAVDGKMYKYNVL  1040
ARO69876.1   961   VAVLKNLLGEHILAEEEKLLEYRERIGNDHPIYNAAKEFIGNMHYPGQWLTAEQKSTYGIKDDGFSFLAVDGKMYKYNVL  1040
ARO69888.1   961   VAVLKNLLGEHILAEEEKLLEYRERIGNDHPIYNAAKEFIGNMHYPGQWLTAEQKSTYGIKDDGFSFLAVDGKMYKYNVL  1040
ARO69853.1   961   VAVLKNLLGEHILAEEEKLLEYRERIGNDHPIYNAAKEFIGNMHYPGQWLTAEQKSTYGIKDDGFSFLAVDGKMYKYNVL  1040
ARO69903.1   961   VAVLKNLLGEHILAEEEKLLEYRERIGNDHPIYNAAKEFIGNMHYPGQWLTAEQKSTYGIKDDGFSFLAVDGKMYKYNVL  1040
ARO69865.1   961   VAVLKNLLGEHILAEEEKLLEYRERIGNDHPIYNAAKEFIGNMHYPGQWLTAEQKSTYGIKDDGFSFLAVDGKMYKYNVL  1040
ARO69902.1   961   VAVLKNLLGEHILAEEEKLLEYRERIGNDHPIYNAAKEFIGNMHYPGQWLTAEQKSTYGIKDDGFSFLAVDGKMYKYNVL  1040
ARO69861.1   961   VAVLKNLLGEHILAEEEKLLEYRERIGNDHPIYNAAKEFIGNMHYPGQWLTAEQKSTYGIKDDGFSFLAVDGKMYKYNVL  1040
ARO69850.1   961   VAVLKNLLGEHILAEEEKLLEYRERIGNDHPIYNAAKEFIGNMHYPGQWLTAEQKSTYGIKDDGFSFLAVDGKMYKYNVL  1040
QYU59227.1   961   VAVLKNLLGEHILAEEEKLLEYRERIGNDHPIYNAAKEFIGNMHYPGQWLTAEQKSTYGIKDDGFSFLAVDGKMYKYNVL  1040
AFM91094.1   961   VAVLKNLLGEHILAEEEKLLEYRERIGNDHPIYNAAKEFIGNMHYPGQWLTAEQKSTYGIKDDGFSFLAVDGKMYKYNVL  1040
ARO69883.1   961   VAVLKNLLGEHILAEEEKLLEYRERIGNDHPIYNAAKEFIGNMHYPGQWLTAEQKSTYGIKDDGFSFLAVDGKMYKYNVL  1040
ARO69879.1   961   VAVLKNLLGEHILAEEEKLLEYRERIGNDHPIYNAAKEFIGNMHYPGQWLTAEQKSTYGIKDDGFSFLAVDGKMYKYNVL  1040
AND76472.1   961   VAVLKNLLGEHILAEEEKLLEYRERIGNDHPIYNAAKEFIGNMHYPGQWLTAEQKSTYGIKDDGFSFLAVDGKMYKYNVL  1040
ARO69846.1   961   VAVLKNLLGEHILAEEEKLLEYRERIGNDHPIYNAAKEFIGNMHYPGQWLTAEQKSTYGIKDDGFSFLAVDGKMYKYNVL  1040
ARO69870.1   961   VAVLKNLLGEHILAEEEKLLEYRERIGNDHPIYNAAKEFIGNMHYPGQWLTAEQKSTYGIKDDGFSFLAVDGKMYKYNVL  1040
ARO69856.1   961   VAVLKNLLGEHILAEEEKLLEYRERIGNDHPIYNAAKEFIGNMHYPGQWLTAEQKSTYGIKDDGFSFLAVDGKMYKYNVL  1040
ARO69847.1   961   VSVLKNLLGDHILTEEEKLLEYRERIGNDHPIYNAAKEFIGNMHYPGQWLTAEQKNTYGIKDDGFSFLAVDGKMYKYNVL  1040
ARO69875.1   961   VSVLKNLLGDHILTEEEKLLEYRERIGNDHPIYNAAKEFIGNMHYPGQWLTAEQKNTYGIKDDGFSFLAVDGKMYKYNVL  1040
ARO69895.1   961   VSVLKNLLGDHILTEEEKLLEYRERIGNDHPIYNAAKEFIGNMHYPGQWLTAEQKNTYGIKDDGFSFLAVDGKMYKYNVL  1040
ARO69880.1   961   VSVLKNLLGDHILTEEEKLLEYRERIGNDHPIYNAAKEFIGNMHYPGQWLTAEQKNTYGIKDDGFSFLAVDGKMYKYNVL  1040
BCT87061.1   961   VAVLKNLLGEHILAEEEKLLEYRERIGNDHPIYNAAKEFIGNMHYPGSWLTTDQKATYGIKDEGFSFLAVDGKMYKYNVL  1040
QYU59225.1   961   VAVLKNLLGEHVLAEEEKLLEYRERIGNDHPIYTAAKEFIGNMHYPGQWLTTEQKNTYGIKDEGFSFLAVDGKMYKYNVL  1040
APD26306.1   961   VMVLKNLLGVHVLAEEEKLLEYRERIGNDHPIYNAAKEFIGNMHYPGQWLTTEQKNTYGIKDDGFSFLAVDGKMYKYNVL  1040


NP_619689.1  1041  GKLNPCESEPPHPNVIPWLEKKTLEIVHWDVHKHIATGPRNALVACFLQGLVQGQSKVESVERMGKDSSPEQQNFFKRLS  1120
AWO68042.1   1041  GKLNPCESEPPHPNVIPWLEKKTLEIVHWDVHKHIATGPRNALVACFLQGLVQGQSKVESVERMGKDSSPEQQNFFKRLS  1120
QYU59223.1   1041  GKLNPCESEPPHPNVIPWLEKKTLEIVHWDVHKHIATGPRNALVACFLQGLVQGQSKVESVERMGKDSSPEQQSFFKRLS  1120
ARO69887.1   1041  GKLNPCESEPPHPNVIPWLEKKTLEIVHWDVHKHIATGPRNALVACFLQGLVQGQSKVESVERMGKDSSPEQQSFFKRLS  1120
ARO69840.1   1041  GKLNPCESEPPHPNVIPWLEKKTLEIVHWDVHKHIATGPRNALVACFLQGLVQGQSKVESVERMGKDSSPEQQSFFKRLS  1120
ARO69892.1   1041  GKLNPCESEPPHPNVIPWLEKKTLEIVHWDVHKHIATGPRNALVACFLQGLVQGQSKVESVERMGKDSSPEQQSFFKRLS  1120
ARO69862.1   1041  GKLNPCESEPPHPNVIPWLEKKTLEIVHWDVHKHIATGPRNALVACFLQGLVQGQSKVESVERMGKDSSPEQQSFFKRLS  1120
ARO69844.1   1041  GKLNPCESEPPHPNVIPWLEKKTLEIVHWDVHKHIATGPRNALVACFLQGLVQGQSKVESVERMGKDSSPEQQSFFKRLS  1120
ARO69896.1   1041  GKLNPCESEPPHPNVIPWLEKKTLEIVHWDVHKHIATGPRNALVACFLQGLVQGQSKVESVERMGKDSSPEQQSFFKRLS  1120
QGP72541.1   1041  GKLNPCESEPPHPNVIPWLEKKTLEIVHWDVHKHIATGPRNALVACFLQGLVQGQSKVESVERMGKDSSPEQQSFFKRLS  1120
ARO69901.1   1041  GKLNPCESEPPHPNVIPWLEKKTLEIVHWDVHKHIATGPRNALVACFLQGLVQGQSKVESVERMGKDSSPEQQSFFKRLS  1120
AYA22299.1   1041  GKLNPCESEPPHPNVIPWLEKKTLEIVHWDVHKHIATGPRNALVACFLQGLVQGQSKVESVERMGKDSSPEQQSFFKRLS  1120
ARO69890.1   1041  GKLNPCESEPPHPNVIPWLEKKTLEIVHWDVHKHIATGPRNALVACFLQGLVQGQSKVESVERMGKDSSPEQQSFFKRLS  1120
ARO69859.1   1041  GKLNPCESEPPHPNVIPWLEKKTLEIVHWDVHKHIATGPRNALVACFLQGLVQGQSKVESVERMGKDSSPEQQSFFKRLS  1120
ARO69905.1   1041  GKLNPCESEPPHPNVIPWLEKKTLEIVHWDVHKHIATGPRNALVACFLQGLVQGQSKVESVERMGKDSSPEQQSFFKRLS  1120
ARO69871.1   1041  GKLNPCESEPPHPNVIPWLEKKTLEIVHWDVHKHIATGPRNALVACFLQGLVQGQSKVESVERMGKDSSPEQQSFFKRLS  1120
ARO69848.1   1041  GKLNPCESEPPHPNVIPWLEKKTLEIVHWDVHKHIATGPRNALVACFLQGLVQGQSKVESVEHMGKDSSPEQQSFFKRLS  1120
ARO69874.1   1041  GKLNPCESEPPHPNVIPWLEKKTLEIVHWDVHKHIATGPRNALVACFLQGLVQGQSKVESVERMGKDSSPEQQSFFKRLS  1120
ARO69882.1   1041  GKLNPCESEPPHPNVIPWLEKKTLEIVHWDVHKHIATGPRNALVACFLQGLVQGQSKVESVERMGKDSSPEQQSFFKRLS  1120
QYU59221.1   1041  GKLNPCESEPPHPNVIPWLEKKTLEIVHWDVHKHIATGPRNALVACFLQGLVQGQSKVESVERMGKDSSPEQQSFYKRLS  1120
ARO69842.1   1041  GKLNPCESEPPHPNVIPWLEKKTLEIVHWDVHKHIATGPRNALVACFLQGLVQGQSKVESVEHMGKDSSPEQQSFFKRLS  1120
AFV34731.1   1041  GKLNPCESVPPHPNVVPWLEKKTLEIVHWDVHKHIATGPRNALVACFLQGLVQGQSKVESVERMGKDSSPEQQNFFKRLS  1120
AND76470.1   1041  GKLNPCESEPPHPNVIPWLEKKTLEIVHWDVHKHIATGPRNALVACFLQGLVQGQSKVESVEHMGKDSSPEQQSFFKRLS  1120
ACZ58632.1   1041  GKLNPCESDPPHPNVIPWLEKKTLEIVHWDVHKHIATGPRNALVACFLQGLVQGQSKVESVERMGKDSSPEQQSFFKRLS  1120
QBZ78374.1   1041  GKLNPCESEPPHPNVIPWLEKKTLEIVHWDVHKHIATGPRNALVACFLQGLVQGQSKVESVEHMGKDSSPEQQSFFKRLS  1120
QGP72540.1   1041  GKLNPCESEPPHPNVIPWLEKKTLEIVHWDVHKHIATGPRNALVACFLQGLVQGQSKVESVERMGKDSSPEQQSFFKRLS  1120
QBZ78372.1   1041  GKLNPCESEPPHPNVIPWLEKKTLEIVHWDVHKHIATGPRNALVACFLQGLVQGQSKVESVEHMGKDSSPEQQSFFKRLS  1120
QYU59224.1   1041  GKLNPCESEPPHPNVIPWLEKKTLEIVHWDVHKHIATGPRNALVACFLQGLVQGQSKVESVERMGKDSSPEQQSFYKRLS  1120
ARO69877.1   1041  GKLNPCESEPPHPNVIPWLEKKTLEIVHWDVHKHIATGPRNALVACFLQGLVQGQSKVESVERMGKDSSPEQQSFFKRLS  1120
ARO69858.1   1041  GKLNPCESEPPHPNVIPWLEKKTLEIVHWDVHKHIATGPRNALVACFLQGLVQGQSKVESVERMGKDSSPEQQSFFKRLS  1120
ARO69855.1   1041  GKLNPCESEPPHPNVIPWLEKKTLEIVHWDVHKHIATGPRNALVACFLQGLVQGQSKVESVERMGKDSSPEQQSFFKRLS  1120
QGP72542.1   1041  GKLNPCESEPPHPNVIPWLEKKTLEIVHWDVHKHIATGPRNALVACFLQGLVQGQSKVESVERMGKDSSPEQQSFFKRLS  1120
AYQ93055.1   1041  GKLNPCESEPPHPNVIPWLEKKTLEIVHWDVHKHIATGPRNALVACFLQGLVQGQSKVESVEHMGKDSSPEQQSFFKRLS  1120
AYQ93053.1   1041  GKLNPCESEPPHPNVIPWLEKKTLEIVHWDVHKHIATGPRNALVACFLQGLVQGQSKVESVERMGKDSSPEQQSFFKRLS  1120
AYQ93051.1   1041  GKLNPCESEPPHPNVIPWLEKKTLEIVHWDVHKHIVTGPRNALVACFLQGLVQGQSKVESVERMGKDSSPEQQNFFKRLS  1120
AFI71813.1   1041  GKLNPCESEPPHPNVIPWLEKKTLEIVHWDVHKHIATGPRNALVACFLQGLVQGQSKVESVERMGKDSSPEQQSFFKRLS  1120
QGP72539.1   1041  GKLNPCESEPPHPNVIPWLEKKTLEIVHWDVHKHIATGPRNALVACFLQGLVQGQSKVESVERMGKDSSPEQQSFFKRLS  1120
QYU59226.1   1041  GKLNPCESEPPHPNVIPWLEKKTLEIVHWDVHKHIATGPRNALVACFLQGLVQGQSKVESVERMGKDSSPEQQSFYKRLS  1120
ARO69852.1   1041  GKLNPCESEPPHPNVIPWLEKKTLEIVHWDVHKHIATGPRNALVACFLQGLVQGQSKVESVERMGKDSSPEQQSFFKRLS  1120
QGP72543.1   1041  GKLNSCESEPPHPNVIPWLEKKTLEIVHWDVHKHIATGPRNALVACFLQGLVQGQSKVESVERMGKDSSPEQQSFYKRLS  1120
QYU59219.1   1041  GKLNPCESEPPHPNVIPWLEKKTLEIVHWDVHKHIATGPRNALVACFLQGLVQGQSKVESVERMGKDSSPEQQSFYKRLS  1120
AGT42200.1   1041  GKLNPCESEPPHPNVIPWLEKKTLEIVHWDVHKHIATGPRNALVACFLQGLVQGQSKVESVERMGKDSSPEQQSFFKRLS  1120
ACZ58633.1   1041  GKLNPCESEPPHPNVIPWLEKKTLEIVHWDVHKHIATGPRNALVACFLQGLVQGQSKVESVERMGKDSSPEQQSFYKRLS  1120
QBZ78369.1   1041  GKLNPCESEPPHPNVIPWLEKKTLEIVHWDVHKHIATGPRNALVACFLQGLVQGQSKVESVERMGKDSSPEQQSFYKRLS  1120
ARO69851.1   1041  GKLNPCESEPPHPNVIPWLEKKTLEIVHWDVHKHIATGPRNALVACFLQGLVQGQSKVESVERMGKDSSPEQQSFFKRLS  1120
QYU59220.1   1041  GKLNPCESEPPHPNVIPWLEKKTLEIVHWDVHKHIATGPRNALVACFLQGLVQGQSKVESVERMGKDSSPEQQNFFRRLS  1120
AFV34755.1   1041  GKLNPCESEPPHPNVIPWLEKKTLEIVHWDVHKHIATGPRNALVACFLQGLVQGQSKVESVERMGKDSSPEQQSFFKRLS  1120
QGP72544.1   1041  GKLNPCESEPPHPNVIPWLERKTLEIVHWDVHKHIATGPRNALVACFLQGLVQGQSKVESVERMGKDSSPEQQSFFKRLS  1120
QYA72352.1   1041  GKLNPCESEPPHPNVIPWLEKKTLEIVHWDVHKHIVTGPRNALVACFLQGLVQGQSKVESVERMGKDSSPEQQSFFKRLS  1120
ARO69854.1   1041  GKLNPCESEPPHPNVIPWLEKKTLEIVHWDVHKHIATGPRNALVACFLQGLVQGQSKVESVERMGKDSSPEQQSFFKRLS  1120
ARO69860.1   1041  GKLNPCESEPPHPNVIPWLEKKTLEIVHWDVHKHIATGPRNALVACFLQGLVQGQSKVESVERMGKDSSPEQQSFFKRLS  1120
ARO69863.1   1041  GKLNPCESEPPHPNVIPWLEKKTLEIVHWDVHKHIATGPRNALVACFLQGLVQGQSKVESVERMGKDSSPEQQSFFKRLS  1120
DAZ85707.1   1041  GKLNPCESEPPHPNVIPWLEKKTLEIVHWDVHKHIATGPRNALVACFLQGLVQGQSKVESVERMGKDSSPEQQNFFKRLS  1120
AWO67781.1   1041  GKLNPCESEPPHPNVVPWLEKKTLEIVHWDVHKHIATGPRNALVACFLQGLVQGQSKVESVERMGKDSSPEQQSFFKRLS  1120
QBZ78367.1   1041  GKLNPCESEPPHPNVIPWLEKKTLEIVHWDVHKHIATGPRNALVACFLQGLVQGQSKVESVERMGKDSSPEQQSFYKRLS  1120
ARO69873.1   1041  GKLNPCESEPPHPNVIPWLEKKTLEIVHWDVHKHIATGPRNALVACFLQGLVQGQSKVESVERMGKDSSPEQQSFFKRLS  1120
AND76471.1   1041  GKLNPCESEPPHPNVIPWLEKKTLEIVHWDVHKHIATGPRNALVACFLQGLVQGQSKVESVERMGKDSSPEQQSFFKRLS  1120
ARO69849.1   1041  GKLNPCESEPPHPNVIPWLEKKTLEIVHWDVHKHIATGPRNALVACFLQGLVQGQSKVESVERMGKDSSPEQQSFFKRLS  1120
ARO69889.1   1041  GKLNPCESEPPHPNVIPWLEKKTLEIVHWDVHKHIATGPRNALVACFLQGLVQGQSKVESVERMGKDSSPEQQSFFKRLS  1120
ARO69867.1   1041  GKLNPCESEPPHPNVIPWLEKKTLEIVHWDVHKHIATGPRNALVACFLQGLVQGQSKVESVERMGKDSSPEQQSFFKRLS  1120
ARO69904.1   1041  GKLNPCESEPPHPNVIPWLEKKTLEIVHWDVHKHIATGPRNALVACFLQGLVQGQSKVESVERMGKDSSPEQQSFFKRLS  1120
```

```
ARO69869.1   1041  GKLNPCESEPPHPNVIPWLEKKTLEIVHWDVHKHIATGPRNALVACFLQGLVQGQSKVESVERMGKDSSPEQQSFFKRLS  1120
ARO69884.1   1041  GKLNPCESEPPHPNVIPWLEKKTLEIVHWDVHKHIATGPRNALVACFLQGLVQGQSKVESVERMGKDSSPEQQSFFKRLS  1120
ARO69878.1   1041  GKLNPCESEPPHPNVIPWLEKKTLEIVHWDVHKHIATGPRNALVACFLQGLVQGQSKVESVERMGKDSSPEQQSFFKRLS  1120
ARO69885.1   1041  GKLNPCESEPPHPNVIPWLEKKTLEIVHWDVHKHIATGPRNALVACFLQGLVQGQSKVESVERMGKDSSPEQQSFFKRLS  1120
ARO69841.1   1041  GKLNPCESEPPHPNVIPWLEKKTLEIVHWDVHKHIATGPRNALVACFLQGLVQGQSKVESVERMGKDSSPEQQSFFKRLS  1120
ARO69891.1   1041  GKLNPCESEPPHPNVIPWLEKKTLEIVHWDVHKHIATGPRNALVACFLQGLVQGQSKVESVERMGKDSSPEQQSFFKRLS  1120
ARO69868.1   1041  GKLNPCESEPPHPNVIPWLEKKTLEIVHWDVHKHIATGPRNALVACFLQGLVQGQSKVESVERMGKDSSPEQQSFFKRLS  1120
ARO69900.1   1041  GKLNPCESEPPHPNVIPWLEKKTLEIVHWDVHKHIATGPRNALVACFLQGLVQGQSKVESVERMGKDSSPEQQSFFKRLS  1120
DAZ85706.1   1041  GKLNPCESEPPHPNVIPWLERKTLEIVHWDVHKHIATGPRNALVACFLQGLVQGQSKVESVERMGKDSSPEQQSFFKRLS  1120
ARO69899.1   1041  GKLNPCESEPPHPNVIPWLEKKTLEIVHWDVHKHIATGPRNALVACFLQGLVQGQSKVESVERMGKDSSPEQQSFFKRLS  1120
ARO69898.1   1041  GKLNPCESEPPHPNVIPWLEKKTLEIVHWDVHKHIATGPRNALVACFLQGLVQGQSKVESVERMGKDSSPEQQSFFKRLS  1120
AYA22297.1   1041  GKLNPCESEPPHPNVIPWLEKKTLEIVHWDVHKHIATGPRNALVACFLQGLVQGQSKVESVERMGKDSSPEQQSFFKRLS  1120
QYU59222.1   1041  GKLNPCESEPPHPNVIPWLEKKTLEIVHWDVHKHIATGPRNALVACFLQGLVQGQSKVESVERMGKDSSPEQQSFYKRLS  1120
ARO69845.1   1041  GKLNPCESEPPHPNVIPWLEKKTLEIVHWDVHKHIATGPRNALVACFLQGLVQGQSKVESVERMGKDSSPEQQSFFKRLS  1120
AND76473.1   1041  GKLNPCESEPPHPNVIPWLEKKTLEIVHWDVHKHIATGPRNALVACFLQGLVQGQSKVESVERMGKDSSPEQQSFFKRLS  1120
ARO69857.1   1041  GKLNPCESEPPHPNVIPWLEKKTLEIVHWDVHKHIATGPRNALVACFLQGLVQGQSKVESVERMGKDSSPEQQSFFKRLS  1120
DAZ85708.1   1041  GKLNPCESEPPHPNVIPWLEKKTLEIVHWDVHKHIATGPRNALVACFLQGLVQGQSKVESVERMGKDSSPEQQSFFKRLS  1120
ARO69881.1   1041  GKLNPCESEPPHPNVIPWLEKKTLEIVHWDVHKHIATGPRNALVACFLQGLVQGQSKVESVEHMGKDSSPEQQSFFKRLS  1120
ARO69893.1   1041  GKLNPCESEPPHPNVIPWLEKKTLEIVHWDVHKHIATGPRNALVACFLQGLVQGQSKVESVEHMGKDSSPEQQSFFKRLS  1120
ARO69897.1   1041  GKLNPCESEPPHPNVIPWLEKKTLEIVHWDVHKHIATGPRNALVACFLQGLVQGQSKVESVEHMGKDSSPEQQSFFKRLS  1120
ARO69872.1   1041  GKLNPCESEPPHPNVIPWLEKKTLEIVHWDVHKHIATGPRNALVACFLQGLVQGQSKVESVEHMGKDSSPEQQSFFKRLS  1120
ARO69843.1   1041  GKLNPCESEPPHPNVIPWLEKKTLEIVHWDVHKHIATGPRNALVACFLQGLVQGQSKVESVEHMGKDSSPEQQSFFKRLS  1120
ARO69886.1   1041  GKLNPCESEPPHPNVIPWLEKKTLEIVHWDVHKHIATGPRNALVACFLQGLVQGQSKVESVEHMGKDSSPEQQSFFKRLS  1120
ARO69864.1   1041  GKLNPCESEPPHPNVIPWLEKKTLEIVHWDVHKHIATGPRNALVACFLQGLVQGQSKVESVEHMGKDSSPEQQSFFKRLS  1120
ARO69876.1   1041  GKLNPCESEPPHPNVIPWLEKKTLEIVHWDVHKHIATGPRNALVACFLQGLVQGQSKVESVEHMGKDSSPEQQSFFKRLS  1120
ARO69888.1   1041  GKLNPCESEPPHPNVIPWLEKKTLEIVHWDVHKHIATGPRNALVACFLQGLVQGQSKVESVEHMGKDSSPEQQSFFKRLS  1120
ARO69853.1   1041  GKLNPCESEPPHPNVIPWLEKKTLEIVHWDVHKHIATGPRNALVACFLQGLVQGQSKVESVEHMGKDSSPEQQSFFKRLS  1120
ARO69903.1   1041  GKLNPCESEPPHPNVIPWLEKKTLEIVHWDVHKHIATGPRNALVACFLQGLVQGQSKVESVEHMGKDSSPEQQSFFKRLS  1120
ARO69865.1   1041  GKLNPCESEPPHPNVIPWLEKKTLEIVHWDVHKHIATGPRNALVACFLQGLVQGQSKVESVEHMGKDSSPEQQSFFKRLS  1120
ARO69902.1   1041  GKLNPCESEPPHPNVIPWLEKKTLEIVHWDVHKHIATGPRNALVACFLQGLVQGQSKVESVEHMGKDSSPEQQSFFKRLS  1120
ARO69861.1   1041  GKLNPCESEPPHPNVIPWLEKKTLEIVHWDVHKHIATGPRNALVACFLQGLVQGQSKVESVEHMGKDSSPEQQSFFKRLS  1120
ARO69850.1   1041  GKLNPCESEPPHPNVIPWLEKKTLEIVHWDVHKHIATGPRNALVACFLQGLVQGQSKVESVEHMGKDSSPEQQSFFKRLS  1120
QYU59227.1   1041  GKLNPCESEPPHPNVIPWLEKKTLEIVHWDVHKHIATGPRNALVACFLQGLVQGQSKVESVERMGKDSSPEQQSFFRRLS  1120
AFM91094.1   1041  GKLNPCESEPPHPNVIPWLEKKTLEIVHWDVHKHIATGPRNALVACFLQGLVQGQSKVESVEHMGKDSSPEQQNFFKRLS  1120
ARO69883.1   1041  GKLNPCESEPPHPNVIPWLEKKTLEIVHWDVHKHIATGPRNALVACFLQGLVQGQSKVESVEHMGKDSSPEQQSFFKRLS  1120
ARO69879.1   1041  GKLNPCESEPPHPNVIPWLEKKTLEIVHWDVHKHIATGPRNALVACFLQGLVQGQSKVESVEHMGKDSSPEQQSFFKRLS  1120
AND76472.1   1041  GKLNPCESEPPHPNVIPWLEKKTLEIVHWDVHKHIATGPRNALVACFLQGLVQGQSKVESVEHMGKDSSPEQQSFFKRLS  1120
ARO69846.1   1041  GKLNPCESEPPHPNVIPWLEKKTLEIVHWDVHKHIATGPRNALVACFLQGLVQGQSKVESVEHMGKDSSPEQQSFFKRLS  1120
ARO69870.1   1041  GKLNPCESEPPHPNVIPWLEKKTLEIVHWDVHKHIATGPRNALVACFLQGLVQGQSKVESVEHMGKDSSPEQQSFFKRLS  1120
ARO69856.1   1041  GKLNPCESEPPHPNVIPWLEKKTLEIVHWDVHKHIATGPRNALVACFLQGLVQGQSKVESVEHMGKDSSPEQQSFFKRLS  1120
ARO69847.1   1041  GKLNPCESEPPHPNVIPWLEKKTLEIVHWDVHKHIATGPRNALVACFLQGLVQGQSKVESVERMGKDSSPEQQSFFKRLS  1120
ARO69875.1   1041  GKLNPCESEPPHPNVIPWLEKKTLEIVHWDVHKHIATGPRNALVACFLQGLVQGQSKVESVERMGKDSSPEQQSFFKRLS  1120
ARO69895.1   1041  GKLNPCESEPPHPNVIPWLEKKTLEIVHWDVHKHIATGPRNALVACFLQGLVQGQSKVESVERMGKDSSPEQQSFFKRLS  1120
ARO69880.1   1041  GKLNPCESEPPHPNVIPWLEKKTLEIVHWDVHKHIATGPRNALVACFLQGLVQGQSKVESVERMGKDSSPEQQSFFKRLS  1120
BCT87061.1   1041  GKLNPCESVPPHPNVIPWLEQKTLEIVHWDVHKHIATGPRNALVACFLQGLVQGQSKVESVERMGKDSSPEQQSFFKRLS  1120
QYU59225.1   1041  GKLNLCESVPPHPNVIPWLEKKTLEIVHWDVHKHIATGPRNALVACFLQGLVQGQSKVESVEQMGKDSSPEQQAFFKRLS  1120
APD26306.1   1041  GKLNPCESVPPHPNVIPWLEKKTLEIVHWDVHKHIATGPRNALVACFLQGLVQGQSKVESVERMGQDSSPEQQAFFKRLS  1120


NP_619689.1  1121  LSERIYLRLCQIRIDNIQKEELAGSGRGPMAILRECLMKSKQVVVENYSLLLTLVAILLLISAAYTLLSTVVALAGCSSF  1200
AWO68042.1   1121  LSERIYLRLCQIRIDNIQKEELAGSGRGPMAILRECLMKSKQVVVENYSLLLTLVAILLLISAAYTLLSTVVALAGCSSF  1200
QYU59223.1   1121  LSERIYLRLCQIRIDNIQKEELAGSGRGPMAILRECLLKSKQVVVENYSLLLTLVAILLLISAAYTLLSTVVALASCSSF  1200
ARO69887.1   1121  LSERIYLRLCQIRIDNIQKEELAGSGRGPMAILRECLLKSKQVVVENYSLLLTLVAILLLISAAYTLLSTVVALASCSSF  1200
ARO69840.1   1121  LSERIYLRLCQIRIDNIQKEELAGSGRGPMAILRECLLKSKQVVVENYSLLLTLVAILLLISAAYTLLSTVVALASCSSF  1200
ARO69892.1   1121  LSERIYLRLCQIRIDNIQKEELAGSGRGPMAILRECLLKSKQVVVENYSLLLTLVAILLLISAAYTLLSTVVALASCSSF  1200
ARO69862.1   1121  LSERIYLRLCQIRIDNIQKEELAGSGRGPMAILRECLLKSKQVVVENYSLLLTLVAILLLISAAYTLLSTVVALASCSSF  1200
ARO69844.1   1121  LSERIYLRLCQIRIDNIQKEELAGSGRGPMAILRECLLKSKQVVVENYSLLLTLVAILLLISAAYTLLSTVVALASCSSF  1200
ARO69896.1   1121  LSERIYLRLCQIRIDNIQKEELAGSGRGPMAILRECLLKSKQVVVENYSLLLTLVAILLLISAAYTLLSTVVALASCSSF  1200
QGP72541.1   1121  LSERIYLRLCQIRIDNIQKEELAGTGRGPMAILRECLLKSKQMVVENYSLLLTLVAILLLISAAYTLLSTVVALAGCSSF  1200
ARO69901.1   1121  LSERIYLRLCQIRIDNIQKEELAGSGRGPMAILRECLLKSKQVVVENYSLLLTLVAILLLISAAYTLLSTVVALASCSSF  1200
AYA22299.1   1121  LSERIYLRLCQIRIDNIQKEELAGSGRGPMAILRECLLKSKQVVVENYSLLLTLVAILLLISAVYTLLSTVVALASCSSF  1200
ARO69890.1   1121  LSERIYLRLCQIRIDNIQKEELAGSGRGPMAILRECLLKSKQVVVENYSLLLTLVAILLLISAAYTLLSTVVALASCSSF  1200
ARO69859.1   1121  LSERIYLRLCQIRIDNIQKEELAGSGRGPMAILRECLLKSKQVVVENYSLLLTLVAILLLISAAYTLLSTVVALASCSSF  1200
ARO69905.1   1121  LSERIYLRLCQIRIDNIQKEELAGSGRGPMAILRECLLKSKQVVVENYSLLLTLVAILLLISAAYTLLSTVVALASCSSF  1200
ARO69871.1   1121  LSERIYLRLCQIRIDNIQKEELAGSGRGPMAILRECLLKSKQVVVENYSLLLTLVAILLLISAAYTLLSTVVALASCSSF  1200
ARO69848.1   1121  LSERIYLRLCQIRIDNIQKEELAGSGRGPMAILRECLLKSKQVVVENYSLLLTLVAILLLISAAYTLLSTVVALASCSSF  1200
ARO69874.1   1121  LSERIYLRLCQIRIDNIQKEELAGSGRGPMAILRECLLKSKQVVVENYSLLLTLVAILLLISAAYTLLSTVVALASCSSF  1200
ARO69882.1   1121  LSERIYLRLCQIRIDNIQKEELAGSGRGPMAILRECLLKSKQVVVENYSLLLTLVAILLLISAAYTLLSTVVALASCSSF  1200
QYU59221.1   1121  LSERIYLRLCQIRIDNIQKEELAGSGRGPMAILRECLLKSKQVVVENYSLLLTLVAILLLISAAYTLLSTVVALASCSSF  1200
ARO69842.1   1121  LSERIYLRLCQIRIDNIQKEELAGSGRGPMAILRECLLKSKQVVVENYSLLLTLVAILLLISAAYTLLSTVVALASCSSF  1200
AFV34731.1   1121  LSERIYLRLCQIRIDNIQKEELVGSGRGPMAILRECLLKSKQVVVENYSLLLTLVAILLLISAAYTLLSTVVALASCSSF  1200
AND76470.1   1121  LSERIYLRLCQIRIDNIQKEELAGSGRGPMAILRECLLKSKQVVVENYSLLLTLVAILLLISAAYTLLSTVVALASCSSF  1200
ACZ58632.1   1121  LSERIYLRLCQIRIDNTQKEELAGSGRGPMAILRECLLKSKQVVVENYSLLLTLVAILLLISAAYTLLSTVVALASCSSF  1200
QBZ78374.1   1121  LSERIYLRLCQIRIDNIQKEELAGSGRGPMAILRECLLKSKQVVVENYSLLLTLVAILLLISAAYTLLSTVVALASCSSF  1200
QGP72540.1   1121  LSERIYLRLCQIRIDNIHKEGLAGSGRGPMAILRECLLKSKQVVVENYSLLLTLVAILLLISAAYTLLSTVVALASCSSF  1200
QBZ78372.1   1121  LSERIYLRLCQIRIDNIQKEELAGSGRGPMAILRECLLKSKQVVVENYSLLLTLVAILLLISAAYTLLSTVVALASCSSF  1200
QYU59224.1   1121  LSERIYLRLCQIRIDNIQKEELAGSGRGPMAILRECLLKSKQVVVENYSLLLTLVAILLLISAAYTLLSTVVALASCSSF  1200
ARO69877.1   1121  LSERIYLRLCQIRIDNIQKEELAGSGRGPMAILRECLLKSKQVVVENYSLLLTLVAILLLISAAYTLLSTVVALASCSSF  1200
ARO69858.1   1121  LSERIYLRLCQIRIDNIQKEELAGSGRGPMAILRECLLKSKQVVVENYSLLLTLVAILLLISAAYTLLSTVVALASCSSF  1200
ARO69855.1   1121  LSERIYLRLCQIRIDNIQKEELAGSGRGPMAILRECLLKSKQVVVENYSLLLTLVAILLLISAAYTLLSTVVALASCSSF  1200
QGP72542.1   1121  LSERIYLRLCQIRIDNIHKEGLAGSGRGPMAILRECLLKSKQVVVENYSLLLTLVAILLLISAAYTLLSTVVALASCSSF  1200
AYQ93055.1   1121  LSERIYLRLCQIRIDNIQKEELAGSGRGPMAILRDCLLKSKQVVVENYSLLLTLVAILLLISAAYTLLSTVVALASCSSF  1200
AYQ93053.1   1121  LSERIYLRLCQIRIDNIQKEELAGAGRDPMAILRECLLKSKQVVVENYSLLLTLVAILLLISAAYTLLSTVVALASCSSF  1200
AYQ93051.1   1121  LSERIYLRLCQIRIDNIQKEDLAGSGRGPMAVLRECLLKSKQIVVENYSLLLTLVAILLLISAAYTLLSTVVALASCSSF  1200
AFI71813.1   1121  LSERIYLRLCQIRIDNIQKEELAGSGRGPMAILRECLLKSKQVVVENYSLLLTLVAILLLISAAYTLLSTVVALASCSSF  1200
QGP72539.1   1121  LSERIYLRLCQIRIDNIHKEGLAGSGRGPMAILRECLLKSKQVVVENYSLLLTLVAILLLISAAYTLLSTVVALASCSSF  1200
QYU59226.1   1121  LSERIYLRLCQIRIDNIQKEELAGSGRGPMAILRECLLKSKQVVVENYSLLLTLVAILLLISAAYTLLSTVVALASCSSF  1200
ARO69852.1   1121  LSERIYLRLCQIRIDNIQKEELAGSGRGPMAILRECLLKSKQVVVENYSLLLTLVAILLLISAAYTLLSTVVALASCSSF  1200
```

QGP72543.1	1121	LSERIYLRLCQIRIDNIQKEELAGSGRGPMAILRECLLKSKQVVVENYSLLLTLVAILLLISAAYTLLSTVVALASCSSF	1200
QYU59219.1	1121	LSERIYLRLCQIRIDNIQKEELAGSGRGPMAILRECLLKSKQVVVENYSLLLTLVAILLLISAAYTLLSTVVALASCSSF	1200
AGT42200.1	1121	LSERIYLRLCQIRIDNIQKEELAGSGRGPMAILRECLLKSKQVVVENYSLLLTLVAILLLISAAYTLLSTVVALASCSSF	1200
ACZ58633.1	1121	LSERIYLRLCQIRIDNIQKEELAGSGRGPMAILRECLLKSKQVVIDNYSLLLTLVAIILLISAAYTLLSTVVALASCSSF	1200
QBZ78369.1	1121	LSERIYLRLCQIRIDNIQKEELAGSGRGPMAILRECLLKSKQVVVENYSLLLTLVAILLLISAAYTLLSTVVALASCSSF	1200
ARO69851.1	1121	LSERIYLRLCQIRIDNIQKEELAGSGRGPMAILRECLLKSKQVVVENYSLLLTLVAILLLISAAYTLLSTVVALASCSSF	1200
QYU59220.1	1121	LSERIYLRLCQIRIDNIQKEELAGSGRGPMAILKECLLKSKQVVVENYSLLLTLVAILLLISAAYTLLSTVVALASCSSF	1200
AFV34755.1	1121	LSERIYLRLCQIRIDNIQKEELAGSGRGPMAILRECLLKSKQVVVENYSLLLTLVAILLLISAAYTLLSTVVALASCSSF	1200
QGP72544.1	1121	LSERIYLRLCQIRIDNIQKEELAGSGRGPMAILRECLLKSKQVVVENYSLLLTLVAILLLISAAYTLLSTVVALASCSSF	1200
QYA72352.1	1121	LSERIYLRLCQIRIDNIQKEELAGSGRGPMAILKECLLKSKQVVVENYSLLLTLIAILLLISAAYTLLSTVVALASCSSF	1200
ARO69854.1	1121	LSERIYLRLCQIRIDNIQKEELAGSGRGPMEILRECLLKSKQVVVENYSLLLTLVAILLLISAAYTLLSTVVALASCSSF	1200
ARO69860.1	1121	LSERIYLRLCQIRIDNIQKEELAGSGRGPMEILRECLLKSKQVVVENYSLLLTLVAILLLISAAYTLLSTVVALASCSSF	1200
ARO69863.1	1121	LSERIYLRLCQIRIDNIQKEELAGSGRGPMEILRECLLKSKQVVVENYSLLLTLVAILLLISAAYTLLSTVVALASCSSF	1200
DAZ85707.1	1121	LSERIYLRLCQIRIDNIQKEELAGSGRGPMAILKECLLKSKQVVVENYSLLLTLVAILLLISAAYTLLSTVVALASCSSF	1200
AWO67781.1	1121	LSERIYLRLCQIRIDNIQKEGLAGCGRGPMEILRECLLKSKQKVVENYSLLLTLVAILLLISAAYTLLSTVVALASCSSF	1200
QBZ78367.1	1121	LSERIYLRLCQIRIDNIQKEELAGSGRGPMAILRECLLKSKQVVIDNYSLLLTLVAIVLLISAAYTLLSTVVALASCSSF	1200
ARO69873.1	1121	LSERIYLRLCQIRIDNIQKEELAGSGRGPMEILRECLLKSKQVVVENYSLLLTLVAILLLISAAYTLLSTVVALASCSSF	1200
AND76471.1	1121	LSERIYLRLCQIRIDNIQKEELAGSGRGPMEILRECLLKSKQVVVENYSLLLTLVAILLLISAAYTLLSTVVALASCSSF	1200
ARO69849.1	1121	LSERIYLRLCQIRIDNIQKEELAGSGRGPMEILRECLLKSKQVVVENYSLLLTLVAILLLISAAYTLLSTVVALASCSSF	1200
ARO69889.1	1121	LSERIYLRLCQIRIDNIQKEELAGSGRGPMEILRECLLKSKQVVVENYSLLLTLVAILLLISAAYTLLSTVVALASCSSF	1200
ARO69867.1	1121	LSERIYLRLCQIRIDNIQKEELAGSGRGPMEILRECLLKSKQVVVENYSLLLTLVAILLLISAAYTLLSTVVALASCSSF	1200
ARO69904.1	1121	LSERIYLRLCQIRIDNIQKEELAGSGRGPMEILRECLLKSKQVVVENYSLLLTLVAILLLISAAYTLLSTVVALASCSSF	1200
ARO69869.1	1121	LSERIYLRLCQIRIDNIQKEELAGSGRGPMEILRECLLKSKQVVVENYSLLLTLVAILLLISAAYTLLSTVVALASCSSF	1200
ARO69884.1	1121	LSERIYLRLCQIRIDNIQKEELAGSGRGPMEILRECLLKSKQVVVENYSLLLTLVAILLLISAAYTLLSTVVALASCSSF	1200
ARO69878.1	1121	LSERIYLRLCQIRIDNIQKEELAGSGRGPMEILRECLLKSKQVVVENYSLLLTLVAILLLISAAYTLLSTVVALASCSSF	1200
ARO69885.1	1121	LSERIYLRLCQIRIDNIQKEELAGSGRGPMEILRECLLKSKQVVVENYSLLLTLVAILLLISAAYTLLSTVVALASCSSF	1200
ARO69841.1	1121	LSERIYLRLCQIRIDNIQKEELAGSGRGPMEILRECLLKSKQVVVENYSLLLTLVAILLLISAAYTLLSTVVALASCSSF	1200
ARO69891.1	1121	LSERIYLRLCQIRIDNIQKEELAGSGRGPMEILRECLLKSKQVVVENYSLLLTLVAILLLISAAYTLLSTVVALASCSSF	1200
ARO69868.1	1121	LSERIYLRLCQIRIDNIQKEELAGSGRGPMEILRECLLKSKQVVVENYSLLLTLVAILLLISAAYTLLSTVVALASCSSF	1200
ARO69900.1	1121	LSERIYLRLCQIRIDNIQKEELAGSGRGPMEILRECLLKSKQVVVENYSLLLTLVAILLLISAAYTLLSTVVALASCSSF	1200
DAZ85706.1	1121	LSERIYLRLCQIRIDNIQKEELAGSGRGPMVILRECLLKSKQVVVENYSLLLTLVAILLLISAAYTLLSTVVALASCSSF	1200
ARO69899.1	1121	LSERIYLRLCQIRIDNIQKEELAGSGRGPMEILRECLLKSKQVVVENYSLLLTLVAILLLISAAYTLLSTVVALASCSSF	1200
ARO69898.1	1121	LSERIYLRLCQIRIDNIQKEELAGSGRGPMEILRECLLKSKQVVVENYSLLLTLVAILLLISAAYTLLSTVVALASCSSF	1200
AYA22297.1	1121	LSERIYLRLCQIRIDNIQKEELAGSGRGPMAILRECLLKSKQVVVENYSLLLTLVAILLLISAAYTLLSTVVALASCSSF	1200
QYU59222.1	1121	LSERIYLRLCQIRIDNIQKEELAGSGRGPMAILKECLLKSKQVVVENYSLLLTLVAILLLISAAYTLLSTVVALASCSSF	1200
ARO69845.1	1121	LSERIYLRLCQIRIDNIQKEELAGSGRGPMEILRECLLKSKQVVVENYSLLLTLVAILLLISAAYTLLSTVVALASCSSF	1200
AND76473.1	1121	LSERIYLRLCQIRIDNIQKEELAGSGRGPMEILRECLLKSKQVVVENYSLLLTLVAILLLISAAYTLLSTVVALASCSSF	1200
ARO69857.1	1121	LSERIYLRLCQIRIDNIQKEELAGSGRGPMEILRECLLKSKQVVVENYSLLLTLVAILLLISAAYTLLSTVVALASCSSF	1200
DAZ85708.1	1121	LSERIYLRLCQIRIDNIQKEELAGCGRGPMEILKECLLKSKQVVVENYSLLLTLVAILLLISAAYTLLSTVVALASCSSF	1200
ARO69881.1	1121	LSERIYLRLCQIRIDNIQKEELAGSGRGPMAILRECLLKSKQVVVENYSLLLTLVAILLLISAAYTLLSTVVALAGCSSF	1200
ARO69893.1	1121	LSERIYLRLCQIRIDNIQKEELAGSGRGPMAILRECLLKSKQVVVENYSLLLTLVAILLLISAAYTLLSTVVALAGCSSF	1200
ARO69897.1	1121	LSERIYLRLCQIRIDNIQKEELAGSGRGPMAILRECLLKSKQVVVENYSLLLTLVAILLLISAAYTLLSTVVALAGCSSF	1200
ARO69872.1	1121	LSERIYLRLCQIRIDNIQKEELAGSGRGPMAILRECLLKSKQVVVENYSLLLTLVAILLLISAAYTLLSTVVALAGCSSF	1200
ARO69843.1	1121	LSERIYLRLCQIRIDNIQKEELAGSGRGPMAILRECLLKSKQVVVENYSLLLTLVAILLLISAAYTLLSTVVALAGCSSF	1200
ARO69886.1	1121	LSERIYLRLCQIRIDNIQKEELAGSGRGPMAILRECLLKSKQVVVENYSLLLTLVAILLLISAAYTLLSTVVALAGCSSF	1200
ARO69864.1	1121	LSERIYLRLCQIRIDNIQKEELAGSGRGPMAILRECLLKSKQVVVENYSLLLTLVAILLLISAAYTLLSTVVALAGCSSF	1200
ARO69876.1	1121	LSERIYLRLCQIRIDNIQKEELAGSGRGPMAILRECLLKSKQVVVENYSLLLTLVAILLLISAAYTLLSTVVALAGCSSF	1200
ARO69888.1	1121	LSERIYLRLCQIRIDNIQKEELAGSGRGPMAILRECLLKSKQVVVENYSLLLTLVAILLLISAAYTLLSTVVALAGCSSF	1200
ARO69853.1	1121	LSERIYLRLCQIRIDNIQKEELAGSGRGPMAILRECLLKSKQVVVENYSLLLTLVAILLLISAAYTLLSTVVALAGCSSF	1200
ARO69903.1	1121	LSERIYLRLCQIRIDNIQKEELAGSGRGPMAILRECLLKSKQVVVENYSLLLTLVAILLLISAAYTLLSTVVALAGCSSF	1200
ARO69865.1	1121	LSERIYLRLCQIRIDNIQKEELAGSGRGPMAILRECLLKSKQVVVENYSLLLTLVAILLLISAAYTLLSTVVALAGCSSF	1200
ARO69902.1	1121	LSERIYLRLCQIRIDNIQKEELAGSGRGPMAILRECLLKSKQVVVENYSLLLTLVAILLLISAAYTLLSTVVALAGCSSF	1200
ARO69861.1	1121	LSERIYLRLCQIRIDNIQKEELAGSGRGPMAILRECLLKSKQVVVENYSLLLTLVAILLLISAAYTLLSTVVALAGCSSF	1200
ARO69850.1	1121	LSERIYLRLCQIRIDNIQKEELAGSGRGPMAILRECLLKSKQVVVENYSLLLTLVAILLLISAAYTLLSTVVALAGCSSF	1200
QYU59227.1	1121	LSERIYLRLCQIRIDNIQKEELAGSGRGPMAILRECLLKSKQVVVENYSLLLTLVAILLLISAAYTLLSTVVALASCSSF	1200
AFM91094.1	1121	LSERIYLRLCQIRIDNIQKEELAGSGRGPMEILRECLLKSKQVVVENYSLLLTLVAILLLISAAYTLLSTVVALASCSSF	1200
ARO69883.1	1121	LSERIYLRLCQIRIDNIQKEELAGSGRGPMAILRECLLKSKQVVVENYSLLLTLVAILLLISAAYTLLSTVVALAGCSSF	1200
ARO69879.1	1121	LSERIYLRLCQIRIDNIQKEELAGSGRGPMAILRECLLKSKQVVVENYSLLLTLVAILLLISAAYTLLSTVVALAGCSSF	1200
AND76472.1	1121	LSERIYLRLCQIRIDNIQKEELAGSGRGPMAILRECLLKSKQVVVENYSLLLTLVAILLLISAAYTLLSTVVALAGCSSF	1200
ARO69846.1	1121	LSERIYLRLCQIRIDNIQKEELAGSGRGPMAILRECLLKSKQVVVENYSLLLTLVAILLLISAAYTLLSTVVALAGCSSF	1200
ARO69870.1	1121	LSERIYLRLCQIRIDNIQKEELAGSGRGPMAILRECLLKSKQVVVENYSLLLTLVAILLLISAAYTLLSTVVALAGCSSF	1200
ARO69856.1	1121	LSERIYLRLCQIRIDNIQKEELAGSGRGPMAILRECLLKSKQVVVENYSLLLTLVAILLLISAAYTLLSTVVALAGCSSF	1200
ARO69847.1	1121	LSERIYLRLCQIRIDNIQKEELAGSGRGPMEILRECLLKSKQVVVENYSLLLTLVAILLLISAAYTLLSTVVALASCSSF	1200
ARO69875.1	1121	LSERIYLRLCQIRIDNIQKEELAGSGRGPMEILRECLLKSKQVVVENYSLLLTLVAILLLISAAYTLLSTVVALASCSSF	1200
ARO69895.1	1121	LSERIYLRLCQIRIDNIQKEELAGSGRGPMEILRECLLKSKQVVVENYSLLLTLVAILLLISAAYTLLSTVVALASCSSF	1200
ARO69880.1	1121	LSERIYLRLCQIRIDNIQKEELAGSGRGPMEILRECLLKSKQVVVENYSLLLTLVAILLLISAAYTLLSTVVALASCSSF	1200
BCT87061.1	1121	LSERIYLRLCQIRIDNIQKEELSGAGRGPMAILRECLLKSKQVVVENYSLLLTLVAILLLISAAYTLLSTVVALAGCSSF	1200
QYU59225.1	1121	LSERIYLRLCQIRIDNVQKEGLAGVGRDPMAILRECLLKSKQVIVENYSLLLTLVAILLLISAAYTLLSTVVALAGCSSF	1200
APD26306.1	1121	LSERIYLRLCQIRIDGIQNEELAGMGRGPMVILRECLLKSKQAVVENYSLLLTLVAILLLISAAYTLLSTVVALAGCSSF	1200

```
                         NTB-VPg cleavage site     VPg-Pro cleavage site

NP_619689.1   1201   AGGMVAVTAVNNASIPCSEPRLEERYSPRNRFVSRISKIRGEGPSKGQGEHEELVTELYYYCDGVKKLISTCWFKGRSLL   1280
AWO68042.1    1201   AGGMVAVTAVNNASIPCSEPRLEERYSPRNRFVSRISKIRGEGPSKGQGEHEELVTELYYYCDGVKKLISTCWFKGRSLL   1280
QYU59223.1    1201   AGGMVAVTAVNSASIPCSEPRLEERYSPRNRFLSRISKIRGEGPSKGQGEHEELVTELYYYCDGVKKLISTCWFKGRSLL   1280
ARO69887.1    1201   AGGVVAVTAVNNASIPCSEPRLEERYSPRNRFVSRISKIRGEGPSKGQGEHEELVTELYYFCDGVKKLISTCWFKGRSLL   1280
ARO69840.1    1201   AGGVVAVTAVNNASIPCSEPRLEERYSPRNRFVSRISKIRGEGPSKGQGEHEELVTELYYFCDGVKKLISTCWFKGRSLL   1280
ARO69892.1    1201   AGGVVAVTAVNNASIPCSEPRLEERYSPRNRFVSRISKIRGEGPSKGQGEHEELVTELYYFCDGVKKLISTCWFKGRSLL   1280
ARO69862.1    1201   AGGVVAVTAVNNASIPCSEPRLEERYSPRNRFVSRISKIRGEGPSKGQGEHEELVTELYYFCDGVKKLISTCWFKGRSLL   1280
ARO69844.1    1201   AGGVVAVTAVNNASIPCSEPRLEERYSPRNRFVSRISKIRGEGPSKGQGEHEELVTELYYFCDGVKKLISTCWFKGRSLL   1280
ARO69896.1    1201   AGGVVAVTAVNNASIPCSEPRLEERYSPRNRFVSRISKIRGEGPSKGQGEHEELVTELYYFCDGVKKLISTCWFKGRSLL   1280
QGP72541.1    1201   AGGMVAVTAVNNASIPCSEPRLEERYSPRNRFVSRISKIRGEGPSKGQGEHEELVTELYYFCDGVKKLISTCWFKGRSLL   1280
ARO69901.1    1201   AGGVVAVTAVNNASIPCSEPRLEERYSPRNRFVSRISKIRGEGPSKGQGEHEELVTELYYFCDGVKKLISTCWFKGRSLL   1280
AYA22299.1    1201   AGGMVAVTAVNNASIPCSEPRLEERYSPRNRFVSRISKIRGEGPSMGQGEHEELVTELYYYCDGVKKLISTCWFKGRSLL   1280
ARO69890.1    1201   AGGVVAVTAVNNASIPCSEPRLEERYSPRNRFVSRISKIRGEGPSKGQGEHEELVTELYYFCDGVKKLISTCWFKGRSLL   1280
ARO69859.1    1201   AGGVVAVTAVNNASIPCSEPRLEERYSPRNRFVSRISKIRGEGPSKGQGEHEELVTELYYFCDGVKKLISTCWFKGRSLL   1280
ARO69905.1    1201   AGGVVAVTAVNNASIPCSEPRLEERYSPRNRFVSRISKIRGEGPSKGQGEHEELVTELYYFCDGVKKLISTCWFKGRSLL   1280
ARO69871.1    1201   AGGVVAVTAVNNASIPCSEPRLEERYSPRNRFVSRISKIRGEGPSKGQGEHEELVTELYYFCDGVKKLISTCWFKGRSLL   1280
ARO69848.1    1201   AGGVVAVTAVNNASIPCSEPRLEERYSPRNRFVSRISKIRGEGPSKGQGEHEELVTELYYFCDGVKKLISTCWFKGRSLL   1280
ARO69874.1    1201   AGGVVAVTAVNNASIPCSEPRLEERYSPRNRFVSRISKIRGEGPSKGQGEHEELVTELYYFCDGVKKLISTCWFKGRSLL   1280
ARO69882.1    1201   AGGVVAVTAVNNASIPCSEPRLEERYSPRNRFVSRISKIRGEGPSKGQGEHEELVTELYYFCDGVKKLISTCWFKGRSLL   1280
QYU59221.1    1201   AGGMVAVTAVNNASIPCSEPRLEERYSPRNRFVSRISKIRGEGPSKGQGEHEELVTELYYYCDGVKKLISTCWFKGRSLL   1280
ARO69842.1    1201   AGGVVAVTAVNNASIPCSEPRLEERYSPRNRFVSRISKIRGEGPSKGQGEHEELVTELYYFCDGVKKLISTCWFKGRSLL   1280
AFV34731.1    1201   AGGVVAVTAVNNASIPCSEPRLEERYSPRNRFVSRISKIRGEGPSKGQGEHEELVTELYYFCDGVKKLISTCWFKGRSLL   1280
AND76470.1    1201   AGGVVAVTAVNNASIPCSEPRLEERYSPRNRFVSRISKIRGEGPSKGQGEHEELVTELYYFCDGVKKLISTCWFKGRSLL   1280
ACZ58632.1    1201   AGGMVAVTAVNNASIPCSEPRQEERYSPRNRFVSRISKIRGEGPSKGQGEHEELVTELYYYCDGVKKLISTCWFKGRSLL   1280
QBZ78374.1    1201   AGGMVAVTAVNNASIPCSEPRLEERYSPRNRFVSRISKIRGEGPSKGQGEHEELVTELYYFCDGVKKLISTCWFKGRSLL   1280
QGP72540.1    1201   AGGVVAVTAVNNASIPCSEPRLEERYSPRNRFASRISKIRGEGPSKGQGDHEELVTELYYYCDGVKKLISTCWFKGRSLL   1280
QBZ78372.1    1201   AGGMVAVTAVNNASIPCSEPRLEERYSPRNRFVSRISKIRGEGPSKGQGEHEELVTELYYFCDGVKKLISTCWFKGRSLL   1280
QYU59224.1    1201   AGGMVAVTAVNNASIPCSEPRLEERYSPRNRFLSRISKTRGEGPSKGQGEHEELVTELYYYCDGVKKLISTCWFKGRSLL   1280
ARO69877.1    1201   AGGVVAVTAVNNASMPCSEPRLEERYSPRNRFVSRISKIRGEGPSKGQGEHEELVTELYYFCDGVKKLISTCWFKGRSLL   1280
ARO69858.1    1201   AGGVVAVTAVNNASMPCSEPRLEERYSPRNRFVSRISKIRGEGPSKGQGEHEELVTELYYFCDGVKKLISTCWFKGRSLL   1280
ARO69855.1    1201   AGGVVAVTAVNNASIPCSEPRLEERYSPRNRFVSRISKIRGEGPSKGQGEHEELVTELYYFCDGVKKLISTCWFKGRSLL   1280
QGP72542.1    1201   AGGVVAVTAVNNASIPCSEPRLEERYSPRNRFASRISKIRGEGPSKGQGDHEELVTELYYYCDGVKKLISTCWFKGRSLL   1280
AYQ93055.1    1201   AGGMVAVTAVNSASIPCSEPRLEERYSPRNRFISRISKIRGEGPSKGQGEHEELVTELYYYCDGVKKLISTCWFKGRSLL   1280
AYQ93053.1    1201   AGGVVAVTAVNNASIPCSEPRMEERYSPRNRFISRISKIRGEGPSKGQGDHEELVTELYYYCDGVKRLISTCWFKGRSLL   1280
AYQ93051.1    1201   AGGMVAVTAVNNASIPCSEPRLEERYSPRNRFLSRISKIRGEGPSKGQGEHEELVAELYYYCDGVKKLISTCWFKGRSLL   1280
AFI71813.1    1201   AGGMVAVTAVNSASIPCSEPRMEERYSPRNRFVSRISKIRGEGPSKGQGEHEELVTELYYYCDGVKKLISTCWFKGRSLL   1280
QGP72539.1    1201   AGGVVAVTAVNNASIPCSEPRLEERYSPRNRFASRISKIRGEGPSKGQGDHEELVTELYYYCDGVKKLISTCWFKGRSLL   1280
QYU59226.1    1201   AGGMVAVTAVNNASIPCSEPRLEERYSPRNRFVSRISKIRGEGPSKGQGEHEELVTELYYYCDGVKKLISTCWFKGRSLL   1280
ARO69852.1    1201   AGGVVAVTAVNNASIPCSEPRLEERYSPRNRFVSRISKIRGEGPSKGQGEHEELVTELYYFCDGVKKLISTCWFKGRSLL   1280
QGP72543.1    1201   AGGMVAVTAVNNASIPCSEPRLEERYSPRNRFVSRISKIRGEGPSKGQGEHEELVTELYYYCDGVKKLISTCWFKGRSLL   1280
QYU59219.1    1201   AGGMVAVTAVNNASIPCSEPRLEERYSPRNRFVSRISKIRGEGPSKGQGEHEELVTELYYFCDGVKKLISTCWFKGRSLL   1280
AGT42200.1    1201   AGGMVAVTAVNSASIPCSEPRMEERYSPRNRFISRISKIRGEGPSKGQGEHEELVTELYYYCDGVKKLISTCWFKGRSLL   1280
ACZ58633.1    1201   AGGMVAVTAVNNASIPCSEPRLEERYSPRNRFVSRISKIRGEGPSKGQGEHEELVTELYYYCDGVKRLISTCWFKGRSLL   1280
QBZ78369.1    1201   AGGMVAVTAVNNASIPCSEPRLEERYSPRNRFISRISKIRGEGPSKGQGDHEELVTELYYYCDGVKKLISTCWFKGRSLL   1280
ARO69851.1    1201   AGGMVAVTAVNNASIPCSEPRLEERYSPRNRFVSRISKIRGEGPSKGQGDHEELVTELYYYCDGVKKLISTCWFKGRSLL   1280
QYU59220.1    1201   AGGMVAVTAVNNASIPCSEPRLEERYSPRNRFVSRISKIRGEGPSKGQGDHEELVTELYYYCDGVKKLISTCWFKGRSLL   1280
AFV34755.1    1201   AGGMVAVTAVNSASIPCSEPRLEERYSPRNRFVSRISKIRGEGPSKGQGEHEELVTELYYYCDGVKKLISTCWFKGRSLL   1280
QGP72544.1    1201   AGGMVAVTAVNSASIPCSEPRLEERYSPRNRFISRISKIRGEGPSMGQGEHEELVTELYYYCDGVKKLISTCWFKGRSLL   1280
QYA72352.1    1201   AGGMVAVTAVNNASIPCSEPRMEERYSPRNRFISRISKIRGEGPSKGQGDHEELVTELYYFCDGAKKLISTCWFKGRSLL   1280
ARO69854.1    1201   AGGMVAVTAVNNASIPCSEPRLEERYSPRNRFVSRISKIRGEGPSKGQGDHEELVTELYYYCDGVKKLISTCWFKGRSLL   1280
ARO69860.1    1201   AGGMVAVTAVNNASIPCSEPRLEERYSPRNRFVSRISKIRGEGPSKGQGDHEELVTELYYYCDGVKKLISTCWFKGRSLL   1280
ARO69863.1    1201   AGGMVAVTAVNNASIPCSEPRLEERYSPRNRFVSRISKIRGEGPSKGQGDHEELVTELYYYCDGVKKLISTCWFKGRSLL   1280
DAZ85707.1    1201   AGGMVAVTAVNNASIPCSEPRFEERYSPRNKYVSRISRIRGEGPSKGQGDHEELVTELYYYCDGVKKLISTCWFKGRSLL   1280
AWO67781.1    1201   AGGMVAVTAVNNASIPCSEPRLEERYSPRNRFVSRISKIRGEGPSKGQGEHEELVTELYYYCEGVKKLISTCWFKGRSLL   1280
QBZ78367.1    1201   AGGMVAVTAVNNASIPCSEPRLEERYSPRNRFISRISKIRGEGPSKGQGEHEELVTELYYYCDGVKRLISTCWFKGRSLL   1280
ARO69873.1    1201   AGGMVAVTAVNNASIPCSEPRLEERYSPRNRFVSRISKIRGEGPSKGQGDHEELVTELYYYCDGVKKLISTCWFKGRSLL   1280
AND76471.1    1201   AGGMVAVTAVNNASIPCSEPRLEERYSPRNRFVSRISKIRGEGPSKGQGDHEELVTELYYYCDGVKKLISTCWFKGRSLL   1280
ARO69849.1    1201   AGGMVAVTAVNNASIPCSEPRLEERYSPRNRFVSRISKIRGEGPSKGQGDHEELVTELYYYCDGVKKLISTCWFKGRSLL   1280
ARO69889.1    1201   AGGMVAVTAVNNASIPCSEPRLEERYSPRNRFVSRISKIRGEGPSKGQGDHEELVTELYYYCDGVKKLISTCWFKGRSLL   1280
ARO69867.1    1201   AGGMVAVTAVNNASIPCSEPRLEERYSPRNRFVSRISKIRGEGPSKGQGDHEELVTELYYYCDGVKKLISTCWFKGRSLL   1280
ARO69904.1    1201   AGGMVAVTAVNNASIPCSEPRLEERYSPRNRFVSRISKIRGEGPSKGQGDHEELVTELYYYCDGVKKLISTCWFKGRSLL   1280
ARO69869.1    1201   AGGMVAVTAVNNASIPCSEPRLEERYSPRNRFVSRISKIRGEGPSKGQGDHEELVTELYYYCDGVKKLISTCWFKGRSLL   1280
ARO69884.1    1201   AGGMVAVTAVNNASIPCSEPRLEERYSPRNRFVSRISKIRGEGPSKGQGDHEELVTELYYYCDGVKKLISTCWFKGRSLL   1280
ARO69878.1    1201   AGGMVAVTAVNNASIPCSEPRLEERYSPRNRFVSRISKIRGEGPSKGQGDHEELVTELYYYCDGVKKLISTCWFKGRSLL   1280
ARO69885.1    1201   AGGMVAVTAVNNASIPCSEPRLEERYSPRNRFVSRISKIRGEGPSKGQGDHEELVTELYYYCDGVKKLISTCWFKGRSLL   1280
ARO69841.1    1201   AGGMVAVTAVNNASIPCSEPRLEERYSPRNRFVSRISKIRGEGPSKGQGDHEELVTELYYYCDGVKKLISTCWFKGRSLL   1280
ARO69891.1    1201   AGGMVAVSAVNNASIPCSEPRLEERYSPRNRFVSRISKIRGEGPSKGQGDHEELVTELYYYCDGVKKLISTCWFKGRSLL   1280
ARO69868.1    1201   AGGMVAVSAVNNASIPCSEPRLEERYSPRNRFVSRISKIRGEGPSKGQGDHEELVTELYYYCDGVKKLISTCWFKGRSLL   1280
ARO69900.1    1201   AGGMVAVTAVNNASIPCSEPRLEERYSPRNRFVSRISKIRGEGPSKGQGDHEELVTELYYYCDGVKKLISTCWFKGRSLL   1280
DAZ85706.1    1201   AGGMVAVTAVNNASIPCSEPRLEERYSPRNRFVSRISKIRGEGPSKGQGEHEELVTELYYYCDGVKKLISTCWFKGRSLL   1280
ARO69899.1    1201   AGGMVAVSAVNNASIPCSEPRLEERYSPRNRFVSRISKIRGEGPSKGQGDHEELVTELYYYCDGVKKLISTCWFKGRSLL   1280
ARO69898.1    1201   AGGMVAVTAVNNASIPCSEPRLEERYSPRNRFVSRISKIRGEGPSKGQGDHEELVTELYYYCDGVKKLISTCWFKGRSLL   1280
AYA22297.1    1201   AGGMVAVTAVNQASIPCSEPRLEERYSPRNRFVSRISKIRGEGPSKGQGEHEELVTELYYYCDGVKKLISTCWFKGRSLL   1280
QYU59222.1    1201   AGGVVAVTAVNNASIPCSEPRLEERYSPRNRFISRISKIRGEGPSMGQGEHEELVTELYYYCDGVKKLISTCWFKGRSLL   1280
ARO69845.1    1201   AGGMVAVTAVNNASIPCSEPRLEERYSPRNRFVSRISKIRGEGPSKGQGDHEELVTELYYYCDGVKKLISTCWFKGRSLL   1280
AND76473.1    1201   AGGMVAVSAVNNASIPCSEPRLEERYSPRNRFVSRISKIRGEGPSKGQGDHEELVTELYYYCDGVKKLISTCWFKGRSLL   1280
ARO69857.1    1201   AGGMVAVSAVNNASIPCSEPRLEERYSPRNRFVSRISKIRGEGPSKGQGDHEELVTELYYYCDGVKKLISTCWFKGRSLL   1280
DAZ85708.1    1201   AGGMVAVTAVNNASIPCSEPRLEERYSPRNRFVSRISKIRGEGPSKGQGEHEELVTELYYFCDGVKKLVSTCWFKGRSLL   1280
ARO69881.1    1201   AGGVVAVTAVNNASIPCSEPRLEERYSPRNRFVSRISKIRGEGPSKGQGEHEELVTELYYFGDGVKKLISTCWFKGRSLL   1280
ARO69893.1    1201   AGGVVAVTAVNNASIPCSEPRLEERYSPRNRFVSRISKIRGEGPSKGQGEHEELVTELYYFGDGVKKLISTCWFKGRSLL   1280
ARO69897.1    1201   AGGVVAVTAVNNASIPCSEPRLEERYSPRNRFVSRISKIRGEGPSKGQGEHEELVTELYYFGDGVKKLISTCWFKGRSLL   1280
ARO69872.1    1201   AGGVVAVTAVNNASIPCSEPRLEERYSPRNRFVSRISKIRGEGPSKGQGEHEELVTELYYFGDGVKKLISTCWFKGRSLL   1280
```

```
ARO69843.1   1201  AGGVVAVTAVNNASIPCSEPRLEERYSPRNRFVSRISKIRGEGPSKGQGEHEELVTELYYFGDGVKKLISTCWFKGRSLL  1280
ARO69886.1   1201  AGGVVAVTAVNNASIPCSEPRLEERYSPRNRFVSRISKIRGEGPSKGQGEHEELVTELYYFGDGVKKLISTCWFKGRSLL  1280
ARO69864.1   1201  AGGVVAVTAVNNASIPCSEPRLEERYSPRNRFVSRISKIRGEGPSKGQGEHEELVTELYYFGDGVKKLISTCWFKGRSLL  1280
ARO69876.1   1201  AGGVVAVTAVNNASIPCSEPRLEERYSPRNRFVSRISKIRGEGPSKGQGEHEELVTELYYFGDGVKKLISTCWFKGRSLL  1280
ARO69888.1   1201  AGGVVAVTAVNNASIPCSEPRLEERYSPRNRFVSRISKIRGEGPSKGQGEHEELVTELYYFGDGVKKLISTCWFKGRSLL  1280
ARO69853.1   1201  AGGVVAVTAVNNASIPCSEPRLEERYSPRNRFVSRISKIRGEGPSKGQGEHEELVTELYYFGDGVKKLISTCWFKGRSLL  1280
ARO69903.1   1201  AGGVVAVTAVNNASIPCSEPRLEERYSPRNRFVSRISKIRGEGPSKGQGEHEELVTELYYFGDGVKKLISTCWFKGRSLL  1280
ARO69865.1   1201  AGGVVAVTAVNNASIPCSEPRLEERYSPRNRFVSRISKIRGEGPSKGQGEHEELVTELYYFGDGVKKLISTCWFKGRSLL  1280
ARO69902.1   1201  AGGVVAVTAVNNASIPCSEPRLEERYSPRNRFVSRISKIRGEGPSKGQGEHEELVTELYYFGDGVKKLISTCWFKGRSLL  1280
ARO69861.1   1201  AGGVVAVTAVNNASIPCSEPRLEERYSPRNRFVSRISKIRGEGPSKGQGEHEELVTELYYFGDGVKKLISTCWFKGRSLL  1280
ARO69850.1   1201  AGGVVAVTAVNNASIPCSEPRLEERYSPRNRFVSRISKIRGEGPSKGQGEHEELVTELYYFGDGVKKLISTCWFKGRSLL  1280
QYU59227.1   1201  AGGMVAVTAVNNASIPCSEPRMEERYSPRNRFVSRIFKIRGEGPSKGQGEHEELVTELYYYCDGVKKLISTCWFKGRSLL  1280
AFM91094.1   1201  AGGVVAVTAVNNASIPCSEPRLEERYSPRNRFVSRISKIRGEGPSKGQGEHEELVTELYYYCDGVKKLISTCWFKGRSLL  1280
ARO69883.1   1201  AGGVVAVTAVNNASIPCSEPRLEERYSPRNRFVSRISKIRGEGPSKGQGEHEELVTELYYFGDGVKKLISTCWFKGRSLL  1280
ARO69879.1   1201  AGGVVAVTAVNNASIPCSEPRLEERYSPRNRFVSRISKIRGEGPSKGQGEHEELVTELYYFGDGVKKLISTCWFKGRSLL  1280
AND76472.1   1201  AGGVVAVTAVNNASIPCSEPRLEERYSPRNRFVSRISKIRGEGPSKGQGEHEELVTELYYFGDGVKKLISTCWFKGRSLL  1280
ARO69846.1   1201  AGGVVAVTAVNNASIPCSEPRLEERYSPRNRFVSRISKIRGEGPSKGQGEHEELVTELYYFGDGVKKLISTCWFKGRSLL  1280
ARO69870.1   1201  AGGVVAVTAVNNASIPCSEPRLEERYSPRNRFVSRISKIRGEGPSKGQGEHEELVTELYYFGDGVKKLISTCWFKGRSLL  1280
ARO69856.1   1201  AGGVVAVTAVNNASIPCSEPRLEERYSPRNRFVSRISKIRGEGPSKGQGEHEELVTELYYFGDGVKKLISTCWFKGRSLL  1280
ARO69847.1   1201  AGGMVAVTAVNNASIPCSEPRLEERYSPRNRFVSRISKIRGEGPSKGQGDHEELVTELYYYCDGVKKLISTCWFKGRSLL  1280
ARO69875.1   1201  AGGMVAVTAVNNASIPCSEPRLEERYSPRNRFVSRISKIRGEGPSKGQGDHEELVTELYYYCDGVKKLISTCWFKGRSLL  1280
ARO69895.1   1201  AGGMVAVTAVNNASIPCSEPRLEERYSPRNRFVSRISKIRGEGPSKGQGDHEELVTELYYYCDGVKKLISTCWFKGRSLL  1280
ARO69880.1   1201  AGGMVAVTAVNNASIPCSEPRLEERYSPRNRFVSRISKIRGEGPSKGQGDHEELVTELYYYCDGVKKLISTCWFKGRSLL  1280
BCT87061.1   1201  AGGMVAVTAINNASIPCSEPRMEERYSPRNKYVSRISRIRGEGPSMGQGEHEELVTELYYYCDGVKKLISTCWFKGRSLL  1280
QYU59225.1   1201  AGGMVAVTAVSNASIPCSEPRLEERYSPRNRFVSRISKIRGEGPSQGQGEHEELVTELYYYSDGVKKLISTCWFKGRSLL  1280
APD26306.1   1201  AGGMLAVTAVNNASIPCSEPRMEERYSPRNRFVSRISKIRGEGPSMGQGEHEELVTELYYFSDGVKKLISTCWFKGRSLL  1280


NP_619689.1  1281  MTRHQALAVPIGNEIEVIYADGTTKKLVWPGRQEDGNCKGFVEFPENELVVFEHPHLLTLPIKYEKYFVDDADRQISPNV  1360
AWO68042.1   1281  MTRHQALAVPIGNEIEVIYADGTTKKLVWPGRQEDGNCKGFVEFPENELVVFEHPHLLTLPIKYEKYFVDDADRQISPNV  1360
QYU59223.1   1281  MTRHQALAVPIGNEIEVIYADGTTKKLVWPGRQEDGNCKGFVEFPENELVVFEHPHLLTLPIKYEKYFVDDADRQISPNV  1360
ARO69887.1   1281  MTRHQALAVPIGNEIEVIYADGTTKKLVWPGRQEDGNCKGFVEFPENELVVFEHPHLLTLPIKYEKYFVDDADRQISPNV  1360
ARO69840.1   1281  MTRHQALAVPIGNEIEVIYADGTTKKLVWPGRQEDGNCKGFVEFPENELVVFEHPHLLTLPIKYEKYFVDDADRQISPNV  1360
ARO69892.1   1281  MTRHQALAVPIGNEIEVIYADGTTKKLVWPGRQEDGNCKGFVEFPENELVVFEHPHLLTLPIKYEKYFVDDADRQISPNV  1360
ARO69862.1   1281  MTRHQALAVPIGNEIEVIYADGTTKKLVWPGRQEDGNCKGFVEFPENELVVFEHPHLLTLPIKYEKYFVDDADRQISPNV  1360
ARO69844.1   1281  MTRHQALAVPIGNEIEVIYADGTTKKLVWPGRQEDGNCKGFVEFPENELVVFEHPHLLTLPIKYEKYFVDDADRQISPNV  1360
ARO69896.1   1281  MTRHQALAVPIGNEIEVIYADGTTKKLVWPGRQEDGNCKGFVEFPENELVVFEHPHLLTLPIKYEKYFVDDADRQISPNV  1360
QGP72541.1   1281  MTRHQALAVPIGNEIEVIYADGTIKKLVWPGRQEDGNCKGFVEFPENELVVFEHPHLLTLPIKYEKYFIDDADRQISPNV  1360
ARO69901.1   1281  MTRHQALAVPIGNEIEVIYADGTTKKLVWPGRQEDGNCKGFVEFPENELVVFEHPHLLTLPIKYEKYFVDDADRQISPNV  1360
AYA22299.1   1281  MTRHQALAVPVGNEIEVIYADGTIKKLVWPGRQEDGNCKGFVEFPENELVVFEHPHLLTLPIKYEKYFVDDADRQISPNV  1360
ARO69890.1   1281  MTRHQALAVPIGNEIEVIYADGTTKKLVWPGRQEDGNCKGFVEFPENELVVFEHPHLLTLPIKYEKYFVDDADRQISPNV  1360
ARO69859.1   1281  MTRHQALAVPIGNEIEVIYADGTTKKLVWPGRQEDGNCKGFVEFPENELVVFEHPHLLTLPIKYEKYFVDDADRQISPNV  1360
ARO69905.1   1281  MTRHQALAVPIGNEIEVIYADGTTKKLVWPGRQEDGNCKGFVEFPENELVVFEHPHLLTLPIKYEKYFVDDADRQISPNV  1360
ARO69871.1   1281  MTRHQALAVPIGNEIEVIYADGTTKKLVWPGRQEDGNCKGFVEFPENELVVFEHPHLLTLPVKYEKYFVDDADRQISPNV  1360
ARO69848.1   1281  MTRHQALAVPIGNEVEVIYADGTTKKLVWPGRQEDGNCKGFVEFPENELVVFEHPHLLTLPIKYEKYFVDDADRQISPNV  1360
ARO69874.1   1281  MTRHQALAVPIGNEIEVIYADGTTKKLVWPGRQEDGNCKGFVEFPENELVVFEHPHLLTLPIKYEKYFVDDADRQISPNV  1360
ARO69882.1   1281  MTRHQALAVPIGNEIEVIYADGTTKKLVWPGRQEDGNCKGFVEFPENELVVFEHPHLLTLPIKYEKYFVDDADRQISPNV  1360
QYU59221.1   1281  MTRHQALAVPIGNEIEVIYADGTTKKLIWPGRQEDGNCKGFVEFFENELVVFEHPHLLTLPIKYEKYFVDDADRQISPNV  1360
ARO69842.1   1281  MTRHQALAVPIGNEVEVIYADGTTKKLVWPGRQEDGNCKGFVEFPENELVVFEHPHLLTLPIKYEKYFVDDADRQISPNV  1360
AFV34731.1   1281  MTRHQALAVPIGNEIEVIYADGTIKKLVWPGRQEDGNCKGFVEFPENELVVFEHPHLLTLPIKYEKFFVDDADRQISPNV  1360
AND76470.1   1281  MTRHQALAVPIGNEVEVIYADGTTKKLVWPGRQEDGNCKGFVEFPENELVVFEHPHLLTLPIKYEKYFVDDADRQISPNV  1360
ACZ58632.1   1281  MTRHQALAVPIGNEIEVIYADGTIKKLVWPGRQEDGNCKGFVEFPENELVVFEHPHLLTLPIKYEKFFVDDADRQISPNV  1360
QBZ78374.1   1281  MTRHQALAVPIGNEVEVIYADGTIKKLVWPGRQEDGNCKGFVEFPENELVVFEHPHLLTLPIKYEKYFVDDADRQISPNV  1360
QGP72540.1   1281  MTRHQALAIPLGNEIEVIYADGTTKKLIWPGRQEDGNCKGFVEFPENELVVFEHPHLSTLPIKFEKYFVDDADRQISPNV  1360
QBZ78372.1   1281  MTRHQALAVPIGNEVEVIYADGTIKKLVWPGRQEDGNCKGFVEFPENELVVFEHPHLLTLPIKYEKYFVDDADRQISPNV  1360
QYU59224.1   1281  MTRHQALAVPVGNEIEVIYADGTIKKLVWPGRQEDGNCKGFVEFFENELVVFEHPHLLTLPIKYEKYFVDDADKQISPNV  1360
ARO69877.1   1281  MTRHQALAVPIGNEIEVIYADGTTKKLVWPGRQEDGNCKGFVEFPENELVVFEHPHLLTLPIKYEKYFVDDADRQISPNV  1360
ARO69858.1   1281  MTRHQALAVPIGNEIEVIYADGTTKKLVWPGRQEDGNCKGFVEFPENELVVFEHPHLLTLPIKYEKYFVDDADRQISPNV  1360
ARO69855.1   1281  MTRHQALAVPIGNEIEVIYADGTTKKLVWPGRQVDGNCKGFVEFPENELVVFEHPHLLTLPIKYEKYFVDDADRQISPNV  1360
QGP72542.1   1281  MTRHQALAIPLGNEIEVIYADGTTKKLIWPGRQEDGNCKGFVEFPENELVVFEHPHLSTLPIKFEKYFVDDADRQISPNV  1360
AYQ93055.1   1281  MTRHQALAVPIGNEIEVIYADGTTKKLVWPGRQEDGNCKGFVEFFENELVVFEHPHLLTLPIKYEKYFVDDADRQISPNV  1360
AYQ93053.1   1281  MTRHQALAVPIGNEIEVIYADGTTKKLVWPGRQVDGNCKGFIEFPENELVVFEHPHLLTLPIKYEKYFVDDADRLISPNV  1360
AYQ93051.1   1281  MTRHQALAVPVGNEIEVIYADGTTKKMVWPGRQEDGNCKGFVEFPENELVVFEHPHLLTLPIKYEKYFVDDADRQISPNV  1360
AFI71813.1   1281  MTRHQALAIPIGSEIEVIYADGTIKKLVWPGRQEDGNCKGFVEFPENELVVFEHPHLLTQPIKYEKYFVDDADRQISPNV  1360
QGP72539.1   1281  MTRHQALAIPLGNEIEVIYADGTTKKLIWPGRQEDGNCKGFVEFPENELVVFEHPHLSTLPIKFEKYFVDDADRQISPNV  1360
QYU59226.1   1281  MTRHQALAVPIGNEIEVIYADGTTKKLVWPGRQEDGNCKGFVEFPENELVVFEHPHLLTLPIKYEKYFVDDADRQISPNV  1360
ARO69852.1   1281  MTRHQALAVPIGNEIEVIYADGTTKKLVWPGRQEDGNCKGFVEFPENELVVFEHPHLLTLPIKYEKYFVDDADRQISPNV  1360
QGP72543.1   1281  MTRHQALAVPIGNEIEVIYADGTTKKLVWPGRQEDGNCKGFVEFPENELVVFEHPHLLTLPIKYEKYFVDDADRQISPNV  1360
QYU59219.1   1281  MTRHQALAIPVGNEIEVIYADGTTKKLVWPGRQEDGNCKGFVEFPENELVVFEHPHLLTLPIKYEKYFVDDADRQISPNV  1360
AGT42200.1   1281  MTRHQALAIPIGSEIEVIYADGTTKKLVWPGRQEDGNCKGFVEFPENELVVFEHPHLLTQPIKYEKYFVDDADRQISPNV  1360
ACZ58633.1   1281  MTRHQALAIPIGNEIEVIYADGTIKKLVWPGRQEDGNCKGFVEFPENELVVFEHPHLLTLPIKYEKYFVDDADRQISPNV  1360
QBZ78369.1   1281  MTRHQALAVPIGNEIEVIYADGTTKKLIWPGRQEDGNCKGFVEFFENELVVFEHPHLLTLPIKYEKYFVDDADRQISPNV  1360
ARO69851.1   1281  MTRHQALAVPIGNEIEVIYADGTTKKLVWPGRQEDGNCKGFVEFPENELVVFEHPHLLTLPIKYEKYFVDDADRQISPNV  1360
QYU59220.1   1281  MTRHQALAVPIGNEIEVIYADGTTKKLVWPGRQEDGNCKGFVEFPENELVVFEHPHLLTLPIKYEKFFVDDADRQISPNV  1360
AFV34755.1   1281  MTRHQALAVPIGNEIEVIYADGTTKKLVWPGRQEDGNCKGFVEFPENELVVFEHPHLLTLPIKYEKYFVDDADRQISPNV  1360
QGP72544.1   1281  MTRHQALAVPIGNEIEVIYADGTTKKLVWPGRQEDGNCKGFVEFPENELVVFEHPHLLTLPIKYEKYFVDDADRQISPNV  1360
QYA72352.1   1281  MTRHQALAVPIGNEIEVIYADGTTKKLVWPGRQEDGNCKGFVEFPENELVVFEHPHLLTLPIKYEKYFVDDADRQISPNV  1360
ARO69854.1   1281  MTRHQALAIPIGNEIEVIYADGTTKKLVWPGRQEDGNCKGFVEFPENELVVFEHPHLLTLPIKYEKFFVDDADRQISPNV  1360
ARO69860.1   1281  MTRHQALAIPIGNEIEVIYADGTTKKLVWPGRQEDGNCKGFVEFPENELVVFEHPHLLTLPIKYEKFFVDDADRQISPNV  1360
ARO69863.1   1281  MTRHQALAIPIGNEIEVIYADGTTKKLVWPGRQEDGNCKGFVEFPENELVVFEHPHLLTLPIKYEKFFVDDADRQISPNV  1360
DAZ85707.1   1281  MTRHQALAVPIGNEIEVIYANGMTKKLIWPGRQVDGNCKGFVEFPENELVVFEHPHLLTLPIKYEKYFVDDADRQISPNV  1360
AWO67781.1   1281  MTRHQALAIPIGNEIEVIYADGTTKKLVWPGRQEDGNCKGFVEFPENELVVFEHPHLLTMPIKYEKYFVDDADRQISPNV  1360
QBZ78367.1   1281  MTRHQALAIPIGNEVEVIYADGTIKKLVWPGRQEDGNCKGFVEFPENELVVFEHPHLLTLPIKYEKYFVDDADRQISPNV  1360
ARO69873.1   1281  MTRHQALAIPIGNEIEVIYADGTTKKLVWPGRQEDGNCKGFVEFPENELVVFEHPHLLTLPIKYEKFFVDDADRQISPNV  1360
AND76471.1   1281  MTRHQALAIPIGNEIEVIYADGTTKKLVWPGRQEDGNCKGFVEFPENELVVFEHPHLLTLPIKYEKFFVDDADRQISPNV  1360
ARO69849.1   1281  MTRHQALAIPIGNEIEVIYADGTTKKLVWPGRQEDGNCKGFVEFLENELVVFEHPHLLTLPIKYEKFFVDDADRQISPNV  1360
ARO69889.1   1281  MTRHQALAIPIGNEIEVIYADGTTKKLVWPGRQEDGNCKGFVEFPENELVVFEHPHLLTLPIKYEKFFVDDADRQISPNV  1360
ARO69867.1   1281  MTRHQALAIPIGNEIEVIYADGTTKKLVWPGRQEDGNCKGFVEFPENELVVFEHPHLLTLPIKYEKFFVDDADRQISPNV  1360
```

```
ARO69904.1   1281  MTRHQALAIPIGNEIEVIYADGTTKKLVWPGRQEDGNCKGFVEFPENELVVFEHPHLLTLPIKYEKFFVDDADRQISPNV  1360
ARO69869.1   1281  MTRHQALAIPIGNEIEVIYADGTTKKLVWPGRQEDGNCKGFVEFPENELVVFEHPHLLTLPIKYEKFFVDDADRQISPNV  1360
ARO69884.1   1281  MTRHQALAIPIGNEIEVIYADGTTKKLVWPGRQEDGNCKGFVEFPENELVVFEHPHLLTLPIKYEKFFVDDADRQISPNV  1360
ARO69878.1   1281  MTRHQALAIPIGNEIEVIYADGTTKKLVWPGRQEDGNCKGFVEFPENELVVFEHPHLLTLPIKYEKFFVDDADRQISPNV  1360
ARO69885.1   1281  MTRHQALAIPIGNEIEVIYADGTTKKLVWPGRQEDGNCKGFVEFPENELVVFEHPHLLTLPIKYEKFFVDDADRQISPNV  1360
ARO69841.1   1281  MTRHQALAIPIGNEIEVIYADGTTKKLVWPGRQEDGNCKGFVEFPENELVVFEHPHLLTLPIKYEKFFVDDADRQISPNV  1360
ARO69891.1   1281  MTRHQALAIPIGNEIEVIYADGTIKKLVWPGRQEDGNCKGFVEFPENELVVFEHPHLLTLPIKYEKFFVDDADRQISPNV  1360
ARO69868.1   1281  MTRHQALAIPIGNEIEVIYADGTIKKLVWPGRQEDGNCKGFVEFPENELVVFEHPHLLTLPIKYEKFFVDDADRQISPNV  1360
ARO69900.1   1281  MTRHQALAIPIGNEIEVIYADGTTKKLVWPGRQEDGNCKGFVEFPENELVVFEHPHLLTLPIKYEKFFVDDADRQISPNV  1360
DAZ85706.1   1281  MTRHQALAVPVGNEIEVIYADGTTKKLVWPGRQKDGNCKGFVEFPENELVVFEHPHLLTLPIKYEKYFVDDADRQISPNV  1360
ARO69899.1   1281  MTRHQALAIPIGNEIEVIYADGTIKKLVWPGRQEDGNCKGFVEFPENELVVFEHPHLLTLPIKYEKFFVDDADRQISPNV  1360
ARO69898.1   1281  MTRHQALAIPIGNEIEVIYADGTTKKLVWPGRQEDGNCKGFVEFPENELVVFEHPHLLTLPIKYEKFFVDDADRQISPNV  1360
AYA22297.1   1281  MTRHQALAVPIGNEIEVIYADGTTKKLVWPGRQEDGNCKGFVEFPENELVVFEHPHLLTLPIKYEKYFVDDADRQISPNV  1360
QYU59222.1   1281  MTRHQALAVPIGNEVEVIYADGTMKKLVWPGRQEDGNCKGFVEFLENELVVFEHPHLLTLPLKYEKYFVDDADRQISPNV  1360
ARO69845.1   1281  MTRHQALAIPIGNEIEVIYADGTTKKLVWPGRQEDGNCKGFVEFPENELVVFEHPHLLTLPIKYEKFFVDDADRQISPNV  1360
AND76473.1   1281  MTRHQALAIPIGNEIEVIYADGTIKKLVWPGRQEDGNCKGFVEFPENELVVFEHPHLLTLPIKYEKFFVDDADRQISPNV  1360
ARO69857.1   1281  MTRHQALAIPIGNEIEVIYADGTIKKLVWPGRQEDGNCKGFVEFPENELVVFEHPHLLTLPIKYEKFFVDDADRQISPNV  1360
DAZ85708.1   1281  MTRHQALAIPIGNEIEVIYADGTTKKIVWPGRQKDGNCKGFVEFLENELVVFEHPHLMTLPIKYEKYFVDDADRQISPTV  1360
ARO69881.1   1281  MTRHQALAVPIGNEVEVIYADGTTKKLVWPGRQEDGNCKGFVEFPENELVVFEHPHLLTLPVKYEKYFVDDADRQISPNV  1360
ARO69893.1   1281  MTRHQALAVPIGNEVEVIYADGTTKKLVWPGRQEDGNCKGFVEFPENELVVFEHPHLLTLPVKYEKYFVDDADRQISPNV  1360
ARO69897.1   1281  MTRHQALAVPIGNEVEVIYADGTTKKLVWPGRQEDGNCKGFVEFPENELVVFEHPHLLTLPVKYEKYFVDDADRQISPNV  1360
ARO69872.1   1281  MTRHQALAVPIGNEVEVIYADGTTKKLVWPGRQEDGNCKGFVEFPENELVVFEHPHLLTLPVKYEKYFVDDADRQISPNV  1360
ARO69843.1   1281  MTRHQALAVPIGNEVEVIYADGTTKKLVWPGRQEDGNCKGFVEFPENELVVFEHPHLLTLPVKYEKYFVDDADRQISPNV  1360
ARO69886.1   1281  MTRHQALAVPIGNEVEVIYADGTTKKLVWPGRQEDGNCKGFVEFPENELVVFEHPHLLTLPVKYEKYFVDDADRQISPNV  1360
ARO69864.1   1281  MTRHQALAVPIGNEVEVIYADGTTKKLVWPGRQEDGNCKGFVEFPENELVVFEHPHLLTLPVKYEKYFVDDADRQISPNV  1360
ARO69876.1   1281  MTRHQALAVPIGNEVEVIYADGTTKKLVWPGRQEDGNCKGFVEFPENELVVFEHPHLLTLPVKYEKYFVDDADRQISPNV  1360
ARO69888.1   1281  MTRHQALAVPIGNEVEVIYADGTTKKLVWPGRQEDGNCKGFVEFPENELVVFEHPHLLTLPVKYEKYFVDDADRQISPNV  1360
ARO69853.1   1281  MTRHQALAVPIGNEVEVIYADGTTKKLVWPGRQEDGNCKGFVEFPENELVVFEHPHLLTLPVKYEKYFVDDADRQISPNV  1360
ARO69903.1   1281  MTRHQALAVPIGNEVEVIYADGTTKKLVWPGRQEDGNCKGFVEFPENELVVFEHPHLLTLPVKYEKYFVDDADRQISPNV  1360
ARO69865.1   1281  MTRHQALAVPIGNEVEVIYADGTTKKLVWPGRQEDGNCKGFVEFPENELVVFEHPHLLTLPVKYEKYFVDDADRQISPNV  1360
ARO69902.1   1281  MTRHQALAVPIGNEVEVIYADGTTKKLVWPGRQEDGNCKGFVEFPENELVVFEHPHLLTLPVKYEKYFVDDADRQISPNV  1360
ARO69861.1   1281  MTRHQALAVPIGNEVEVIYADGTTKKLVWPGRQEDGNCKGFVEFPENELVVFEHPHLLTLPVKYEKYFVDDADRQISPNV  1360
ARO69850.1   1281  MTRHQALAVPIGNEVEVIYADGTTKKLVWPGRQEDGNCKGFVEFPENELVVFEHPHLLTLPVKYEKYFVDDADRQISPNV  1360
QYU59227.1   1281  MTRHQALAVPIGNEVEVIYADGTTKKLVWPGRQEDGNCKGFVEFPENELVVFEHPHLLTLPIKYEKFFVDDADRQISPNV  1360
AFM91094.1   1281  MTRHQALAVPIGNEIEVIYADGTTKKLVWPGRQEDGNCKGFVEFPENELVVFEHPHLLTLPIKYEKYFVDDADRQISPNV  1360
ARO69883.1   1281  MTRHQALAVPIGNEVEVIYADGTTKKLVWPGRQEDGNCKGFVEFPENELVVFEHPHLLTLPVKYEKYFVDDADRQISPNV  1360
ARO69879.1   1281  MTRHQALAVPIGNEVEVIYADGTTKKLVWPGRQEDGNCKGFVEFPENELVVFEHPHLLTLPVKYEKYFVDDADRQISPNV  1360
AND76472.1   1281  MTRHQALAVPIGNEVEVIYADGTTKKLVWPGRQEDGNCKGFVEFPENELVVFEHPHLLTLPVKYEKYFVDDADRQISPNV  1360
ARO69846.1   1281  MTRHQALAVPIGNEVEVIYADGTTKKLVWPGRQEDGNCKGFVEFPENELVVFEHPHLLTLPVKYEKYFVDDADRQISPNV  1360
ARO69870.1   1281  MTRHQALAVPIGNEVEVIYADGTTKKLVWPGRQEDGNCKGFVEFPENELVVFEHPHLLTLPVKYEKYFVDDADRQISPNV  1360
ARO69856.1   1281  MTRHQALAVPIGNEVEVIYADGTTKKLVWPGRQEDGNCKGFVEFPENELVVFEHPHLLTLPVKYEKYFVDDADRQISPNV  1360
ARO69847.1   1281  MTRHQALAIPIGNEIEVIYADGTTKKLVWPGRQEDGNCKGFVEFSENELVVFEHPHLSTLPVKYEKFFVDDADRQISPNV  1360
ARO69875.1   1281  MTRHQALAIPIGNEIEVIYADGTTKKLVWPGRQEDGNCKGFVEFSENELVVFEHPHLSTLPVKYEKFFVDDADRQISPNV  1360
ARO69895.1   1281  MTRHQALAIPIGNEIEVIYADGTTKKLVWPGRQEDGNCKGFVEFSENELVVFEHPHLSTLPVKYEKFFVDDADRQISPNV  1360
ARO69880.1   1281  MTRHQALAIPIGNEIEVIYADGTTKKLVWPGRQEDGNCKGFVEFSENELVVFEHPHLSTLPVKYEKFFVDDADRQISPNV  1360
BCT87061.1   1281  MTRHQALAVPIGTEVQVIYADGTNKKLVWPGRQENGNCKGFIEFPENELVVFEHPHLLTMPIKYEKYFVDDADRQISPNV  1360
QYU59225.1   1281  MTRHQALAVPVGVEVEIIYADGTKKTLVWPGRQEDGNCKGFVEFPENELVVFEHPRLLTMPIKYEKYFVDDADKQISPNV  1360
APD26306.1   1281  MTRHQALAVPIGNEVEIIYADGTSRKLVWPGRQEDGNCKGFIEFPENELVVFEHPRLLTLPIKYEKFFVDDADRQISPNV  1360


NP_619689.1  1361  AVKCCVARLEDGIPQFHFWSKYATARSEVHTLKDEGGGNVYQNKIRRYIVYAHEAKKYDCGALAVAVIQGIPKVIAMLVS  1440
AWO68042.1   1361  AVKCCVARLEDGIPQFHFWSKYATARSEVHTLKDEGGGNVYQNKIRRYIVYAHEAKKYDCGALAVAVIQGIPKVIAMLVS  1440
QYU59223.1   1361  AVKCCVARLEDGIPQFHYWNKYAMARSEVHTLKDEGGGNVYQNKIRRYIVYPHEAKKYDCGALAVAVVQGIPKVIAMLVS  1440
ARO69887.1   1361  AVKCCVARLEDGIPQFHFWNKYAMARSEVHTLKDEGGGNVYQNKIRRYIVYAHEAKKYDCGALAVAVIQGIPKVIAMLVS  1440
ARO69840.1   1361  AVKCCVARLEDGIPQFHFWNKYAMARSEVHTLKDEGGGNVYQNKIRRYIVYAHEAKKYDCGALAVAVIQGIPKVIAMLVS  1440
ARO69892.1   1361  AVKCCVARLEDGIPQFHFWNKYAMARSEVHTLKDEGGGNVYQNKIRRYIVYAHEAKKYDCGALAVAVIQGIPKVIAMLVS  1440
ARO69862.1   1361  AVKCCVARLEDGIPQFHFWNKYAMARSEVHTLKDEGGGNVYQNKIRRYIVYAHEAKKYDCGALAVAVIQGIPKVIAMLVS  1440
ARO69844.1   1361  AVKCCVARLEDGIPQFHFWNKYAMARSEVHTLKDEGGGNVYQNKIRRYIVYAHEAKKYDCGALAVAVIQGIPKVIAMLVS  1440
ARO69896.1   1361  AVKCCVARLEDGIPQFHFWNKYAMARSEVHTLKDEGGGNVYQNKIRRYIVYAHEAKKYDCGALAVAVIQGIPKVIAMLVS  1440
QGP72541.1   1361  AVKCCVARLEDGIPQFHFWNKYASARSEVHTLKDEGGGNVYQNKIRRYIVYSHEAKKYDCGALAVAVIQGIPKVIAMLVS  1440
ARO69901.1   1361  AVKCCVARLEDGIPQFHFWNKYAMARSEVHTLKDEGGGNVYQNKIRRYIVYAHEAKKYDCGALAVAVIQGIPKVIAMLVS  1440
AYA22299.1   1361  AVKCCVARLEDGIPQFHFWNKYAMARSEVHTLKDEGGGNVYQNKIRRYIVYSHEAKKYDCGALAVAVIQGIPKVIAMLVS  1440
ARO69890.1   1361  AVKCCVARLEDGIPQFHFWNKYAMARSEVHTLKDEGGGNVYQNKIRRYIVYAHEAKKYDCGALAVAVIQGIPKVIAMLVS  1440
ARO69859.1   1361  AVKCCVARLEDGIPQFHFWNKYAMARSEVHTLKDEGGGNVYQNKIRRYIVYAHEAKKYDCGALAVAVIQGIPKVIAMLVS  1440
ARO69905.1   1361  AVKCCVARLEDGIPQFHFWNKYAMARSEVHTLKDEGGGNVYQNKIRRYIVYAHEAKKYDCGALAVAVIQGIPKVIAMLVS  1440
ARO69871.1   1361  AVKCCVARLEDGIPQFHFWNKYAMARSEVHTLKDEGGGNVYQNKIRRYIVYAHEAKKYDCGALAVAVIQGIPKVIAMLVS  1440
ARO69848.1   1361  AVKCCVARLEDGIPQFHFWNKYAMARSEVHTLKDEGGGNVYQNKIRRYIVYAHEAKKYDCGALAVAVIQGIPKVIAMLVS  1440
ARO69874.1   1361  AVKCCVARLEDGIPQFHFWNKYAMARSEVHTLKDEGGGNVYQNKIRRYIVYAHEAKKYDCGALAVAVIQGIPKVIAMLVS  1440
ARO69882.1   1361  AVKCCVARLEDGIPQFHFWNKYAMARSEVHTLKDEGGGNVYQNKIRRYIVYAHEAKKYDCGALAVAVIQGIPKVIAMLVS  1440
QYU59221.1   1361  AVKCCVARLEDGIPQFHFWNKYAMARSEVHTLKDEGGGNVYQNKIRRYIVYPHEAKKYDCGALAVAVVQGIPKVIAMLVS  1440
ARO69842.1   1361  AVKCCVARLEDGIPQFHFWNKYAMARSEVHTLKDEGGGNVYQNKIRRYIVYAHEAKKYDCGALAVAVIQGIPKVIAMLVS  1440
AFV34731.1   1361  AVKCCVARLEDGIPQFHFWNKYATARSEVHTLKDEGGGNVYQNKIRRYIVYAHEAKKYDCGALAVAVIQGIPKVIAMLVS  1440
AND76470.1   1361  AVKCCVARLEDGIPQFHFWNKYAMARSEVHTLKDEGGGNVYQNKIRRYIVYAHEAKKYDCGALAVAVIQGIPKVIAMLVS  1440
ACZ58632.1   1361  AVKCCVARLEDGIPQFHFWNKYAMARSEVHTLKDEGGGNVYQNKIRRYIVYPHEAKKYDCGALAVAVIQGIPKVIAMLVS  1440
QBZ78374.1   1361  AVKCCVARLEDGIPQFHFWNKYAMARSEVHTLKDEGGGNVYQNKIRRYIVYPHEAKKYDCGALAVAVIQGTPKVIAMLVS  1440
QGP72540.1   1361  AVKCCVARLEDGIPQFHFWNKYASARSEVHTLKDEGGGNVYQNKIRRYIVYAHEAKKYDCGALAVAVIQGIPKVIAMLVS  1440
QBZ78372.1   1361  AVKCCVARLEDGIPQFHFWNKYAMARSEVHTLKDEGGGNVYQNKIRRYIVYPHEAKKYDCGALAVAVIQGTPKVIAMLVS  1440
QYU59224.1   1361  AVKCCVARLEDGIPQFHFWNKYAMARSEVHTLKDEGGGNVYQNKIRRYIVYSHEAKKYDCGALAVAVVQGVPKVIAMLVS  1440
ARO69877.1   1361  AVKCCVARLEDGIPQFHFWNKYAMARSEVHTLKDEGGGNVYQNKIRRYIVYAHEAKKYDCGALAVAVIQGIPKVIAMLVS  1440
ARO69858.1   1361  AVKCCVARLEDGIPQFHFWNKYAMARSEVHTLKDEGGGNVYQNKIRRYIVYAHEAKKYDCGALAVAVIQGIPKVIAMLVS  1440
ARO69855.1   1361  AVKCCVARLEDGIPQFHFWDKYAMARSEVHTLKDEGGGNVYQNKIRRYIVYAHEAKKYDCGALAVAVIQGIPKVIAMLVS  1440
QGP72542.1   1361  AVKCCVARLEDGIPQFHFWNKYATARSEVHTLKDEGGGNVYQNKIRRYIVYAHEAKKYDCGALAVAVIQGIPKVIAMLVS  1440
AYQ93055.1   1361  AVKCCVARLEDGIPQFHFWDKYAMARSEVHTLKDEGGGNVYQNKIRRYIVYPHEAKKYDCGALAVAVVQGIPKVIAMLVS  1440
AYQ93053.1   1361  AVKCCVARLEDGIPQFHFWNKYAMARSEVHTLKDEGGGDVYQNKIRRYIVYSHEAKKYDCGALAVAVIQGTSKVIAMLVS  1440
AYQ93051.1   1361  AVKCCVARLEDGIPQFHFWNKYAMARSEVHTLKDEGGGNVYQNKIRRYIVYSHEARKYDCGALAVAVIQGIPKVIAMLVS  1440
AFI71813.1   1361  AVKCCVARLEDGIPQFHFWNKYATARSEVHTLKDEGGGNVYQNKIRRYIVYAHEAKKYDCGALAVAVIQGTPKVIAMLVS  1440
QGP72539.1   1361  AVKCCVARLEDGIPQFHFWNKYASARSEVHTLKDEGGGNVYQNKIRRYIVYAHEAKKYDCGALAVAVIQGIPKVIAMLVS  1440
QYU59226.1   1361  AVKCCVARLEDGVPQFHFWNKYAMARSEVHTLKDEGGGNVYQNKIRRYIVYSHEAKKYDCGALAVAVIQGVPKVIAMLVS  1440
```

```
ARO69852.1   1361  AVKCCVARLEDGIPQFHFWNKYAMARSEVHTLKDEGGGNVYQNKIRRYIVYAHEAKKYDCGALAVAVIQGIPKVIAMLVS  1440
QGP72543.1   1361  AVKCCVARLEDGIPQFHFWNKYATARSETHTLKDEGGGNVYQNKIRRYIVYPHEAKKYDCGALAVAVIQGIPKVIAMLVS  1440
QYU59219.1   1361  AVKCCVARLEDGIPQFHFWNKYAMARSEVHTLKDEGGGNVYQNKIRRYIVYSHEAKKYDCGALAVAVIQGTPKVIAMLVS  1440
AGT42200.1   1361  AVKCCVARLEDGIPQFHFWNKYATARSEVHTLKDEGGGNVYQNKIRRYIVYAHEAKKYDCGALAVAVIQGTPKVVAMLVS  1440
ACZ58633.1   1361  AVKCCVARLEDGIPQFHFWNKYATARSETHTLKDEGGGNVYQNKIRRYIVYSHEAKKYDCGALAVAVVQGIPKVIAMLVS  1440
QBZ78369.1   1361  AVKCCVARLEDGIPQFHFWNKYAMARSEVHTLKDEGGGNVYQNKIRRYIVYPHEAKKYDCGALAVAVVQGVPKVIAMLVS  1440
ARO69851.1   1361  AVKCCVARLEDGIPQFHYWNKYAMARSEVHTLKDEGGGNVYQNKIRRYIVYSHEAKKYDCGALAVAVIQGTPKVIAMLVS  1440
QYU59220.1   1361  AVKCCVARLEDGIPQFHFWNKYASARSEVHTLKDEGGGNVYQNKIRRYIVYAHEAKKYDCGALAVAVIQGTPKVIAMLVS  1440
AFV34755.1   1361  AVKCCVARLEDGIPQFHFWNKYAMARSEVHTLKDEGGGNVYQNKIRRYIVYSHEAKKYDCGALAVAVIQGIPKVIAMLVS  1440
QGP72544.1   1361  AVKCCVARLEDGIPQFHFWNKYAMARSEVHTLKDEGGGNVYQNKIRRYIVYSHEAKKYDCGALAVAVVQGIPKVIAMLVS  1440
QYA72352.1   1361  AVKCCVARLEEGIPQFHFWNKYATARSEVHTLKDEGGGNVYQNKIRRYIVYSHEAKKYDCGALAVAVIQGIPKVIAMLVS  1440
ARO69854.1   1361  AVKCCVARLEDGIPQFHFWNKYATARSEVHTLKDEGGGNVYQNKIRRYIVYVHEARKYDCGALAVAVVQGIPKVIAMLVS  1440
ARO69860.1   1361  AVKCCVARLEDGIPQFHFWNKYATARSEVHTLKDEGGGNVYQNKIRRYIVYAHEARKYDCGALAVAVVQGIPKVIAMLVS  1440
ARO69863.1   1361  AVKCCVARLEDGIPQFHFWNKYATARSEVHTLKDEGGGNVYQNKIRRYIVYAHEARKYDCGALAVAVVQGIPKVIAMLVS  1440
DAZ85707.1   1361  AVKCCVARLEDGIPQFHFWNKYAMARNEVHTLKDEGGGNVYQNKIRRYIVYAHDAKKYDCGALAVAVIQGTPKVIAMLVS  1440
AWO67781.1   1361  AVKCCVARLEEGIPQFHFWNKYATARSEVHTLKDEGGGNVYQNKIRRYIVYAHEAKKYDCGALAVAVIQGIPKVIAMLVS  1440
QBZ78367.1   1361  AVKCCVARLEDGIPQFHFWNKYATARSETHTLKDEGGGNVYQNKIRRYIVYSHEAKKYDCGALAVAVVQGIPKVIAMLVS  1440
ARO69873.1   1361  AVKCCVARLEDGIPQFHFWNKYATARSEVHTLKDEGGGNVYQNKIRRYIVYAHEARKYDCGALAVAVVQGIPKVIAMLVS  1440
AND76471.1   1361  AVKCCVARLEDGIPQFHFWNKYATARSEVHTLKDEGGGNVYQNKIRRYIVYVHEARKYDCGALAVAVIQGIPKVIAMLVS  1440
ARO69849.1   1361  AVKCCVARLEDGIPQFHFWNKYATARSEVHTLKDEGGGNVYQNKIRRYIVYAHEARKYDCGALAVAVVQGIPKVIAMLVS  1440
ARO69889.1   1361  AVKCCVARLEDGIPQFHFWNKYATARSEVHTLKDEGGGNVYQNKIRRYIVYVHEARKYDCGALAVAVIQGIPKVIAMLVS  1440
ARO69867.1   1361  AVKCCVARLEDGIPQFHFWNKYATARSEVHTLKDEGGGNVYQNKIRRYIVYVHEARKYDCGALAVAVVQGIPKVIAMLVS  1440
ARO69904.1   1361  AVKCCVARLEDGIPQFHFWNKYATARSEVHTLKDEGGGNVYQNKIRRYIVYVHEARKYDCGALAVAVIQGIPKVIAMLVS  1440
ARO69869.1   1361  AVKCCVARLEDGIPQFHFWNKYATARSEVHTLKDEGGGNVYQNKIRRYIVYAHEARKYDCGALAVAVVQGIPKVIAMLVS  1440
ARO69884.1   1361  AVKCCVARLEDGIPQFHFWNKYATARSEVHTLKDEGGGNVYQNKIRRYIVYVHEARKYDCGALAVAVVQGIPKVIAMLVS  1440
ARO69878.1   1361  AVKCCVARLEDGIPQFHFWNKYATARSEVHTLKDEGGGNVYQNKIRRYIVYAHEARKYDCGALAVAVVQGIPKVIAMLVS  1440
ARO69885.1   1361  AVKCCVARLEDGIPQFHFWNKYATARSEVHTLKDEGGGNVYQNKIRRYIVYVHEARKYDCGALAVAVVQGIPKVIAMLVS  1440
ARO69841.1   1361  AVKCCVARLEDGIPQFHFWNKYATARSEVHTLKDEGGGNVYQNKIRRYIVYVHEARKYDCGALAVAVVQGIPKVIAMLVS  1440
ARO69891.1   1361  AVKCCVARLEDGIPQFHFWNKYATARSEVHTLKDEGGGNVYQNKIRRYIVYAHEARKYDCGALAVAVIQGIPKVIAMLVS  1440
ARO69868.1   1361  AVKCCVARLEDGIPQFHFWNKYATARSEVHTLKDEGGGNVYQNKIRRYIVYAHEARKYDCGALAVAVIQGIPKVIAMLVS  1440
ARO69900.1   1361  AVKCCVARLEDGIPQFHFWNKYATARSEVHTLKDEGGGNVYQNKIRRYIVYVHEARKYDCGALAVAVIQGIPKVIAMLVS  1440
DAZ85706.1   1361  AVKCCVARLEDGIPQFHFWNKYAMARSEVHTLKDEGGGNVYQNKIRRYIVYSHEAKKYDCGALAVAEIQGTPKVIAMLVS  1440
ARO69899.1   1361  AVKCCVARLEDGIPQFHFWNKYATARSEVHTLKDEGGGNVYQNKIRRYIVYAHEARKYDCGALAVAVIQGIPKVIAMLVS  1440
ARO69898.1   1361  AVKCCVARLEDGIPQFHFWNKYATARSEVHTLKDEGGGNVYQNKIRRYIVYVHEARKYDCGALAVAVVQGIPKVIAMLVS  1440
AYA22297.1   1361  AVKCCVARLEDGIPQFHFWNKYATARSEVHTLKDEGGGNVYQNKIRRYIVYSHEAKKYDCGALAVAVIQGVPKVIAMLVS  1440
QYU59222.1   1361  AVKCCVARLEDGIPQFHFWNKYAMARSEVHTLKDEGGGNVYQNKIRRYIVYPHEAKKYDCGALAVAVIQGVPKVIAMLVS  1440
ARO69845.1   1361  AVKCCVARLEDGIPQFHFWNKYATARSEVHTLKDEGGGNVYQNKIRRYIVYVHEARKYDCGALAVAVVQGIPKVIAMLVS  1440
AND76473.1   1361  AVKCCVARLEDGIPQFHFWNKYATARSEVHTLKDEGGGNVYQNKIRRYIVYSHEARKYDCGALAVAVIQGIPKVIAMLVS  1440
ARO69857.1   1361  AVKCCVARLEDGIPQFHFWNKYATARSEVHTLKDEGGGNVYQNKIRRYIVYSHEARKYDCGALAVAVIQGIPKVIAMLVS  1440
DAZ85708.1   1361  AVKCCVARLEDGIPQFHFWDKYAMVRSEVHTLKDEGGGNVYQNKIRRYIVYTHEAKMNDCGALAVAVIQGIPKVIAMLVS  1440
ARO69881.1   1361  AVKCCVARLEDGIPQFHYWNKYAMARSEVHTLKDEGGGNVYQNKIRRYIVYAHEAKKYDCGALAVAVIQGIPKVIAMLVS  1440
ARO69893.1   1361  AVKCCVARLEDGIPQFHYWNKYAMARSEVHTLKDEGGGNVYQNKIRRYIVYAHEAKKYDCGALAVAVIQGIPKVIAMLVS  1440
ARO69897.1   1361  AVKCCVARLEDGIPQFHYWNKYAMARSEVHTLKDEGGGNVYQNKIRRYIVYAHEAKKYDCGALAVAVIQGIPKVIAMLVS  1440
ARO69872.1   1361  AVKCCVARLEDGIPQFHYWNKYAMARSEVHTLKDEGGGNVYQNKIRRYIVYAHEAKKYDCGALAVAVIQGIPKVIAMLVS  1440
ARO69843.1   1361  AVKCCVARLEDGIPQFHYWNKYAMARSEVHTLKDEGGGNVYQNKIRRYIVYAHEAKKYDCGALAVAVIQGIPKVIAMLVS  1440
ARO69886.1   1361  AVKCCVARLEDGIPQFHYWNKYAMARSEVHTLKDEGGGNVYQNKIRRYIVYAHEAKKYDCGALAVAVIQGIPKVIAMLVS  1440
ARO69864.1   1361  AVKCCVARLEDGIPQFHYWNKYAMARSEVHTLKDEGGGNVYQNKIRRYIVYAHEAKKYDCGALAVAVIQGIPKVIAMLVS  1440
ARO69876.1   1361  AVKCCVARLEDGIPQFHYWNKYAMARSEVHTLKDEGGGNVYQNKIRRYIVYAHEAKKYDCGALAVAVIQGIPKVIAMLVS  1440
ARO69888.1   1361  AVKCCVARLEDGIPQFHYWNKYAMARSEVHTLKDEGGGNVYQNKIRRYIVYAHEAKKYDCGALAVAVIQGIPKVIAMLVS  1440
ARO69853.1   1361  AVKCCVARLEDGIPQFHYWNKYAMARSEVHTLKDEGGGNVYQNKIRRYIVYAHEAKKYDCGALAVAVIQGIPKVIAMLVS  1440
ARO69903.1   1361  AVKCCVARLEDGIPQFHYWNKYAMARSEVHTLKDEGGGNVYQNKIRRYIVYAHEAKKYDCGALAVAVIQGIPKVIAMLVS  1440
ARO69865.1   1361  AVKCCVARLEDGIPQFHYWNKYAMARSEVHTLKDEGGGNVYQNKIRRYIVYAHEAKKYDCGALAVAVIQGIPKVIAMLVS  1440
ARO69902.1   1361  AVKCCVARLEDGIPQFHYWNKYAMARSEVHTLKDEGGGNVYQNKIRRYIVYAHEAKKYDCGALAVAVIQGIPKVIAMLVS  1440
ARO69861.1   1361  AVKCCVARLEDGIPQFHYWNKYAMARSEVHTLKDEGGGNVYQNKIRRYIVYAHEAKKYDCGALAVAVIQGIPKVIAMLVS  1440
ARO69850.1   1361  AVKCCVARLEDGIPQFHYWNKYAMARSEVHTLKDEGGGNVYQNKIRRYIVYAHEAKKYDCGALAVAVIQGIPKVIAMLVS  1440
QYU59227.1   1361  AVKCCVARLEDGIPQFHFWNKYAMARSEVHTLKDEGGGNVYQNKIRRYIVYSHEAKRNDCGALAVAVIQGVPKVIAMLVS  1440
AFM91094.1   1361  AVKCCVARLEDGIPQFHFWNKYAMARSEVHTLKDEGGGNVYQNKIRRYIVYAHEAKKYDCGALAVAVIQGTPKVIAMLVS  1440
ARO69883.1   1361  AVKCCVARLEDGIPQFHYWNKYAMARSEVHTLKDEGGGNVYQNKIRRYIVYAHEAKKYDCGALAVAVIQGIPKVIAMLVS  1440
ARO69879.1   1361  AVKCCVARLEDGIPQFHYWNKYAMARSEVHTLKDEGGGNVYQNKIRRYIVYAHEAKKYDCGALAVAVIQGIPKVIAMLVS  1440
AND76472.1   1361  AVKCCVARLEDGIPQFHYWNKYAMARSEVHTLKDEGGGNVYQNKIRRYIVYAHEAKKYDCGALAVAVIQGIPKVIAMLVS  1440
ARO69846.1   1361  AVKCCVARLEDGIPQFHYWNKYAMARSEVHTLKDEGGGNVYQNKIRRYIVYAHEAKKYDCGALAVAVIQGIPKVIAMLVS  1440
ARO69870.1   1361  AVKCCVARLEDGIPQFHYWNKYAMARSEVHTLKDEGGGNVYQNKIRRYIVYAHEAKKYDCGALAVAVIQGIPKVIAMLVS  1440
ARO69856.1   1361  AVKCCVARLEDGIPQFHYWNKYAMARSEVHTLKDEGGGNVYQNKIRRYIVYAHEAKKYDCGALAVAVIQGIPKVIAMLVS  1440
ARO69847.1   1361  AVKCCVARLEDGIPQFHFWNKYATARSEVHTLKDEGGGNVYQNKIRRYIVYAHEAKKYDCGALAVAVIQGVPKVIAMLVS  1440
ARO69875.1   1361  AVKCCVARLEDGIPQFHFWNKYATARSEVHTLKDEGGGNVYQNKIRRYIVYAHEAKKYDCGALAVAVIQGVPKVIAMLVS  1440
ARO69895.1   1361  AVKCCVARLEDGIPQFHFWNKYATARSEVHTLKDEGGGNVYQNKIRRYIVYAHEAKKYDCGALAVAVIQGVPKVIAMLVS  1440
ARO69880.1   1361  AVKCCVARLEDGIPQFHFWNKYATARSEVHTLKDEGGGNVYQNKIRRYIVYAHEAKKYDCGALAVAVIQGVPKVIAMLVS  1440
BCT87061.1   1361  AVKCCVARLEEGILQFHYWNKYATVRNEVHTLKDEGGGEVYQNVIRRHIIYAHEARKYDCGALAVAEIQGVPKVIAMLVS  1440
QYU59225.1   1361  AVKCCVARLEDGIPQFHFWNKYATVRSEVHTLKDEGGGNVYQNKIRRYVVYSHEAKKYDCGALAVAVIQGVPKVIAMLVS  1440
APD26306.1   1361  AVKCCVARLDDGIPQFHFWNKYATARSEVHTLKDEGGGDVYQNKIRRYIVYAHEAKKYDCGALAVAVIQGVPKVIAMLVS  1440
```

Pro-Pol cleavage site

```
NP_619689.1  1441  GNRGVTYSSVIPNYSSSFIRGEVPYVPEDGLVSRGYRKVGYLHASDAPHVPSKTSFMKVPDELCFPYPDPKQPAILSAED  1520
AWO68042.1   1441  GNRGVTYSSVIPNYSSSFIRGEVPYVPEDGLVSRGYRKVGYLHASDAPHVPSKTSFMKVPDELCFPYPDPKQPAILSAED  1520
QYU59223.1   1441  GNRGVTYSSVIPNYSSSFVRGEVPFVPEDGLVTRGYRKVGYLHASDAPHVPSKTSFMKVPDELCFPYPDPKQPAILSAED  1520
ARO69887.1   1441  GNRGVTYSSVIPNYSSSFVRGEVPYVPEDGLVTRGYRKVGYLRAPDAPHVPSKTAFMKVPDELCFPYPDPKQPAILSAED  1520
ARO69840.1   1441  GNRGVTYSSVIPNYSSSFVRGEVPYVPEDGLVTRGYRKVGYLRAPDAPHVPSKTAFMKVPDELCFPYPDPKQPAILSAED  1520
ARO69892.1   1441  GNRGVTYSSVIPNYSSSFVRGEVPYVPEDGLVTRGYRKVGYLRAPDAPHVPSKTAFMKVPDELCFPYPDPKQPAILSAED  1520
ARO69862.1   1441  GNRGVTYSSVIPNYSSSFVRGEVPYVPEDGLVTRGYRKVGYLRAPDAPHVPSKTAFMKVPDELCFPYPDPKQPAILSAED  1520
ARO69844.1   1441  GNRGVTYSSVIPNYSSSFVRGEVPYVPEDGLVTRGYRKVGYLRAPDAPHVPSKTAFMKVPDELCFPYPDPKQPAILSAED  1520
ARO69896.1   1441  GNRGVTYSSVIPNYSSSFVRGEVPYVPEDGLVTRGYRKVGYLRAPDAPHVPSKTAFMKVPDELCFPYPDPKQPAILSAED  1520
QGP72541.1   1441  GNRGVTYSSVIPNYSSSFVRGEVPYVPEDGLVTRGYRKAGYLRAPDAPHVPSKTSFMKVPDELCFPYPDPKQPAILSAED  1520
ARO69901.1   1441  GNRGVTYSSVIPNYSSSFVRGEVPYVPEDGLVTRGYRKVGYLRAPDAPHVPSKTAFMKVPDELCFPYPDPKQPAILSAED  1520
AYA22299.1   1441  GNRGVTYSSVIPNYSSSFIKGEVPYVPEDGLVTKGYRKVGYLHASDAPHVPSKTSFMKVPDELCFPYPDPKQPAILSAED  1520
ARO69890.1   1441  GNRGVTYSSVIPNYSSSFVRGEVPYVPEDGLVTRGYRKVGYLRAPDAPHVPSKTAFMKVPDELCFPYPDPKQPAILSAED  1520
ARO69859.1   1441  GNRGVTYSSVIPNYSSSFVRGEVPYVPEDGLVTRGYRKVGYLRAPDAPHVPSKTAFMKVPDELCFPYPDPKQPAILSAED  1520
```

```
ARO69905.1   1441  GNRGVTYSSVIPNYSSSFVRGEVPYVPEDGLVTRGYRKVGYLRAPDAPHVPSKTAFMKVPDELCFPYPDPKQPAILSAED  1520
ARO69871.1   1441  GNRGVTYSSVIPNYSSSFVRGEVPYVPEDGLVTRGYRKVGYLRAPDAPHVPSKTAFMKVPDELCFPYPDPKQPAILSAED  1520
ARO69848.1   1441  GNRGVTYSSVIPNYSSSFIRGEVPYVPEDGLVTRGYRKVGYLRAPDAPHVPSKTAFMKVPDELCFPYPDPKQPAILSAED  1520
ARO69874.1   1441  GNRGVTYSSVIPNYSSSFVRGEVPYVPEDGLVTRGYRKVGYLRAPDAPHVPSKTAFMKVPDELCFPYPDPKQPAILSAED  1520
ARO69882.1   1441  GNRGVTYSSVIPNYSSSFVRGEVPYVPEDGLVTRGYRKVGYLRAPDAPHVPSKTAFMKVPDELCFPYPDPKQPAILSAED  1520
QYU59221.1   1441  GNRGVTYSSVIPNYSSSFVRGEVPYVPEDGLVTRGYRKVGYLHASDAPHVPSKTSFMKVPDELCFPYPDPKQPAILSAED  1520
ARO69842.1   1441  GNRGVTYSSVIPNYSSSFIRGEVPYVPEDGLVTRGYRKVGYLRAPDAPHVPSKTAFMKVPDELCFPYPDPKQPAILSAED  1520
AFV34731.1   1441  GNRGVTYSSVIPNYSSSFVRGEVPYVPEDGLVTRGYRKAGYLRASDAPHVPSRTSFMKVPDELCFPYPDPKQPAILSAED  1520
AND76470.1   1441  GNRGVTYSSVIPNYSSSFIRGEVPYVPEDGLVTRGYRKVGYLRAPDAPHVPSKTAFMKVPDELCFPYPDPKQPAILSAED  1520
ACZ58632.1   1441  GNRGITYSSVIPNYSSSFVRGEVPYVPEDGLVTRGYRKVGYLRMSDAPHVPSKTSFMKVPDELCFPYPDPKQPAILSAED  1520
QBZ78374.1   1441  GNRGVTYSSVIPNYSSSFVRGEVPYVPEDGLVTRGYRKVGYLHASDAPHVPSKTSFMKVPDELCFPYPDPKQPAILSAAD  1520
QGP72540.1   1441  GNRGVTYSSVIPNYSSSFVRGEVPYVPEDGLVTRGYRKVGYLHASNAPHVPSKTSFMKVPDELCFPYPDPKQPAILSAED  1520
QBZ78372.1   1441  GNRGVTYSSVIPNYSSSFVRGEVPYVPEDGLVTRGYRKVGYLHASDAPHVPSKTSFMKVPDELCFPYPDPKQPAILSAAD  1520
QYU59224.1   1441  GNRGVTYSSVIPNYSSSFVRGEVPYVPEDGLVTRGYRKVGYLHASDAPHAPTKTSFMKVPDELCFPYPDPKQPAILSVED  1520
ARO69877.1   1441  GNRGVTYSSVIPNYSSSFVRGEVPYVPEDGLVTRGYRKVGYLRAPDAPHVPSKTAFMKVPDELCFPYPDPKQPAILSAED  1520
ARO69858.1   1441  GNRGVTYSSVIPNYSSSFVRGEVPYVPEDGLVTRGYRKVGYLRAPDAPHVPSKTAFMKVPDELCFPYPDPKQPAILSAED  1520
ARO69855.1   1441  GNRGVTYSSVIPNYSSSFVRGEVPYVPEDGLVTRGYRKVGYLRAPDAPHVPSKTAFMKVPDELCFPYPDPKQPAILSAED  1520
QGP72542.1   1441  GNRGVTYSSVIPNYSSSFVRGEVPYVPEDGLVTRGYRKVGYLHASNAPHVPSKTSFMKVPDELCFPYPDPKQPAILSAED  1520
AYQ93055.1   1441  GNRGVTYSSVIPNYSSSFVRGEVPYVPEDGLVTRGYRKAGYLHASDAPHVPSKTSFMKVPDELCFPYPDPKQPAILSAED  1520
AYQ93053.1   1441  GNRGVTYSSVIPNYSSSFVRGEVPYVPEDGLVTRGYRKAGYLHASNAPHAPSKTSFMKVPDELCFPYPDPKQPAILSAED  1520
AYQ93051.1   1441  GNRGVTYSSIIPNYSSSFVRGEVPYVPEDGLVTRGYRKVGYLHASDAPHAPSKTSFTKVPDELCFPFPDPKQPAILSAED  1520
AFI71813.1   1441  GNRGVTYSSVIPNYSSSFVRGEVPYVPEDGLVTRGYRKVGYLRASDAPHVPSKTSFLKVPDELCFPYPDPKQPAILSAED  1520
QGP72539.1   1441  GNRGVTYSSVIPNYSSSFVRGEVPYVPEDGLVTRGYRKVGYLHASNAPHVPSKTSFMKVPDELCFPYPDPKQPAILSAED  1520
QYU59226.1   1441  GNRGVTYSSVIPNYSSSFVRGEVPYVPEDGLVTRGYRKVGYLHASDAPHVPSKTSFIKVPDELCFPYPDPKQPAILNAED  1520
ARO69852.1   1441  GNRGVTYSSVIPNYSSSFVKGEVPYVPEDGLVTRGYRKVGYLRAPDAPHVPSKTAFMKVPDELCFPYPDPKQPAILSAED  1520
QGP72543.1   1441  GNRGVTYSSVIPNYSSSFVKGEVPYVPEDGLVTRGYRKVGYLYASNAPHVPSKTSFVKVPDELCFPYPDPKQPAILSAED  1520
QYU59219.1   1441  GNRGVTYSSVIPNYSSSFVRGEVPYVPEDGLVTRGYRKVGYLHAANAPHVPSKTSFMKVPDELCFPYPDPKQPAILSTED  1520
AGT42200.1   1441  GNRGVTYSSVIPNYSSSFVRGEVPYVPEDGLVTRGYRKVGYLRASDAPHVPSKTSFLKVPDELCFPYPDPKQPAILSAED  1520
ACZ58633.1   1441  GNRGVTYSSVIPNYSSSFVRGEVPYVPEDGIVTRGYRKVGYLHASDAPHVPSKTSFMKVPDELCFPYPDPKQPAILSVED  1520
QBZ78369.1   1441  GNRGVTYSSVIPNYSSSFVRGEVPYVPEDGLVTRGYRKVGYLHASDAPHVPSRTSFMKVPDELCFPYPDSKQPAILSAED  1520
ARO69851.1   1441  GNRGVTYSSVIPNYSSSFVRGEVPYVPEDGLVTRGYRKVGYLHAPDALHVPSKTAFMKVPDELCFPYPNPKQPAILSAED  1520
QYU59220.1   1441  GNRGVTYSSVIPNYSSSFVRGEVPYVPEDGLVARGYRKVGYLHASDAPHVPSKTSFMKVPDELCFPYPDPKQPAILSAED  1520
AFV34755.1   1441  GNRGVTYSSVIPNYSSSFVRGEVPYVPEDGLVTRGYRKVGYLHASDAPHVPSKTSFMKVPDELCFPYPDPKQPAILSAED  1520
QGP72544.1   1441  GNRGVTYSSIIPNYSSSFVRGEVPYVPEDGLVTRGYRKVGYLHVSDAPHVPSRTSFMKVPDELCFPYPEPKQPAILSAED  1520
QYA72352.1   1441  GNRGVTYSSVIPSYSSSFIKGEVPYVPEDGLVTRGYRKVGYLHVVNAPHVPSKTSFMRVPDELCFPYPDPKQPAILSAED  1520
ARO69854.1   1441  GNRGVTYSSVIPNYSSSFVRGEVPYVPEDGLVTRGYRKVGYLHASDAPHVPSKTSFMRVPDELCFPYPDPKQPAILSAED  1520
ARO69860.1   1441  GNRGVTYSSVIPNYSSSFVRGEVPYVPEDGLVTRGYRKVGYLHASDAPHVPSKTSFMRVPDELCFPYPDPKQPAILSAED  1520
ARO69863.1   1441  GNRGVTYSSVIPNYSSSFVRGEVPYVPEDGLVTRGYRKVGYLHASDAPHVPSKTSFMRVPDELCFPYPDPKQPAILSAED  1520
DAZ85707.1   1441  GNRGVTYSSVIPSYDSSFLMGEVPYVPEDGLVTRGYRKVGYLHASDAPHAPSKTSFMKVPDELCFPYPDPKQPAILSAED  1520
AWO67781.1   1441  GNRGVTYSSVIPNYSSSYVKGEVPYVPEDGLVTRGYRKVGYLHASDAPHAPSKTSFMRVPDELCFPYPDPKQPAILSAED  1520
QBZ78367.1   1441  GNRGVTYSSVIPNYSSSFVRGEVPYVPEDGIVTRGYRKVGYLHASDAPHVPSKTSFMKVPDELCFPYPDPKQPAILSVED  1520
ARO69873.1   1441  GNRGVTYSSVIPNYSSSFVRGEVPYVPEDGLVTRGYRKVGYLHASDAPHVPSKTSFMRVPDELCFPYPDPKQPAILSAED  1520
AND76471.1   1441  GNRGVTYSSVIPNYSSSFVRGEVPYVPEDGLVTRGYRKVGYLHASDAPHVPSKTSFMRVPDELCFPYPDPKQPAILSAED  1520
ARO69849.1   1441  GNRGVTYSSVIPNYSSSFVRGEVPYVPEDGLVTRGYRKVGYLHASDAPHVPSKTSFMRVPDELCFPYPDPKQPAILSAED  1520
ARO69889.1   1441  GNRGVTYSSVIPNYSSSFVRGEVPYVPEDGLVTRGYRKVGYLHASDAPHVPSKTSFMRVPDELCFPYPDPKQPAILSAED  1520
ARO69867.1   1441  GNRGVTYSSVIPNYSSSFVRGEVPYVPEDGLVTRGYRKVGYLHASDAPHVPSKTSFMRVPDELCFPYPDPKQPAILSAED  1520
ARO69904.1   1441  GNRGVTYSSVIPNYSSSFVRGEVPYVPEDGLVTRGYRKVGYLHASDAPHVPSKTSFMRVPDELCFPYPDPKQPAILSAED  1520
ARO69869.1   1441  GNRGVTYSSVIPNYSSSFVRGEVPYVPEDGLVTRGYRKVGYLHASDAPHVPSKTSFMRVPDELCFPYPDPKQPAILSAED  1520
ARO69884.1   1441  GNRGVTYSSVIPNYSSSFVRGEVPYVPEDGLVTRGYRKVGYLHASDAPHVPSKTSFMRVPDELCFPYPDPKQPAILSAED  1520
ARO69878.1   1441  GNRGVTYSSVIPNYSSSFVRGEVPYVPEDGLVTRGYRKVGYLHASDAPHVPSKTSFMRVPDELCFPYPDPKQPAILSAED  1520
ARO69885.1   1441  GNRGVTYSSVIPNYSSSFVRGEVPYVPEDGLVTRGYRKVGYLHASDAPHVPSKTSFMRVPDELCFPYPDPKQPAILSAED  1520
ARO69841.1   1441  GNRGVTYSSVIPNYSSSFVRGEVPYVPEDGLVTRGYRKVGYLHASDAPHVPSKTSFMRVPDELCFPYPDPKQPAILSAED  1520
ARO69891.1   1441  GNRGVTYSSVIPNYSSSFVKGEVPYVPEDGLVTRGYRKVGYLHASDAPHVPSKTSFMRVPDELCFPYPDPKQPAILSAED  1520
ARO69868.1   1441  GNRGVTYSSVIPNYSSSFVKGEVPYVPEDGLVTRGYRKVGYLHASDAPHVPSKTSFMRVPDELCFPYPDPKQPAILSAED  1520
ARO69900.1   1441  GNRGVTYSSVIPNYSSSFVRGEVPYVPEDGLVTRGYRKVGYLHASDAPHVPSKTSFMRVPDELCFPYPDPKQPAILSAED  1520
DAZ85706.1   1441  GNRGVTYSSVIPTYDSSYLMGEVPYVPEDGLVTRGYRKVGYLHASDAPHVPSKTSFMKVPDEICFPYPDPKQPAILSAED  1520
ARO69899.1   1441  GNRGVTYSSVIPNYSSSFVKGEVPYVPEDGLVTRGYRKVGYLHASDAPHVPSKTSFMRVPDELCFPYPDPKQPAILSAED  1520
ARO69898.1   1441  GNRGVTYSSVIPNYSSSFIRGEVPYVPEDGLVTRGYRKVGYLHASDAPHVPSKTSFMRVPDELCFPYPDPKQPAILSAED  1520
AYA22297.1   1441  GNRGVTYSSVIPNYSSSFVRGDVPYVPEDGLVTRGYRKVGYLHASDAPHVPSKTAFMKVPDELCFPYPDPKQPAILSAED  1520
QYU59222.1   1441  GNRGVTYSSVIPNYSSSFVRGDVPYVPEDGLVTRGYRKVGYLHASNAPHVPSRTSFMKVPDELCFPYPDPKQPAILSADD  1520
ARO69845.1   1441  GNRGVTYSSVIPNYSSSFVRGEVPYVPEDGLVTRGYRKVGYLHASDAPHVPSKTSFMRVPDELCFPYPDPKQPAILSAED  1520
AND76473.1   1441  GNRGVTYSSVIPNYSSSFVKGEVPYVPEDGLVTRGYRKVGYLHASDAPHVPSKTSFMRVPDELCFPYPDPKQPAILSAED  1520
ARO69857.1   1441  GNRGVTYSSVIPNYSSSFVKGEVPYVPEDGLVTRGYRKVGYLHASDAPHVPSKTSFMRVPDELCFPYPDPKQPAILSAED  1520
DAZ85708.1   1441  GNRGVTYSSVIPSYSSSYLKGEVPYVPEDGLVTRGYRKIGYLHASDAPHVPSRTSFMKVPDELCFPYPDPKQPAILSAED  1520
ARO69881.1   1441  GNRGVTYSSVIPSYSSSYIKGEVPFVPEDGLVTRGYRKVGYLRAPDAPHVPSKTAFMKVPDELCFPYPDPKQPAILSAED  1520
ARO69893.1   1441  GNRGVTYSSVIPSYSSSYIKGEVPFVPEDGLVTRGYRKVGYLRAPDAPHVPSKTAFMKVPDELCFPYPDPKQPAILSAED  1520
ARO69897.1   1441  GNRGVTYSSVIPSYSSSYIKGEVPFVPEDGLVTRGYRKVGYLHAPDAPHVPSKTAFMKVPDELCFPYPDPKQPAILSAED  1520
ARO69872.1   1441  GNRGVTYSSVIPSYSSSYIKGEVPFVPEDGLVTRGYRKVGYLHAPDAPHVPSKTAFMKVPDELCFPYPDPKQPAILSAED  1520
ARO69843.1   1441  GNRGVTYSSVIPSYSSSYIKGEVPFVPEDGLVTRGYRKVGYLRAPDAPHVPSKTAFMKVPDELCFPYPDPKQPAILSAED  1520
ARO69886.1   1441  GNRGVTYSSVIPSYSSSYIKGEVPFVPEDGLVTRGYRKVGYLRAPDAPHVPSKTAFMKVPDELCFPYPDPKQPAILSAED  1520
ARO69864.1   1441  GNRGVTYSSVIPSYSSSYIKGEVPFVPEDGLVTRGYRKVGYLRAPDAPHVPSKTAFMKVPDELCFPYPDPKQPAILSAED  1520
ARO69876.1   1441  GNRGVTYSSVIPSYSSSYIKGEVPFVPEDGLVTRGYRKVGYLHAPDAPHVPSKTAFMKVPDELCFPYPDPKQPAILSAED  1520
ARO69888.1   1441  GNRGVTYSSVIPSYSSSYIKGEVPFVPEDGLVTRGYRKVGYLRAPDAPHVPSKTAFMKVPDELCFPYPDPKQPAILSAED  1520
ARO69853.1   1441  GNRGVTYSSVIPSYSSSYIKGEVPFVPEDGLVTRGYRKVGYLHAPDAPHVPSKTAFMKVPDELCFPYPDPKQPAILSAED  1520
ARO69903.1   1441  GNRGVTYSSVIPSYSSSYIKGEVPFVPEDGLVTRGYRKVGYLRAPDAPHVPSKTAFMKVPDELCFPYPDPKQPAILSAED  1520
ARO69865.1   1441  GNRGVTYSSVIPSYSSSYIKGEVPFVPEDGLVTRGYRKVGYLRAPDAPHVPSKTAFMKVPDELCFPYPDPKQPAILSAED  1520
ARO69902.1   1441  GNRGVTYSSVIPSYSSSYIKGEVPFVPEDGLVTRGYRKVGYLRAPDAPHVPSKTAFMKVPDELCFPYPDPKQPAILSAED  1520
ARO69861.1   1441  GNRGVTYSSVIPSYSSSYIKGEVPFVPEDGLVTRGYRKVGYLRAPDAPHVPSKTAFMKVPDELCFPYPDPKQPAILSADD  1520
ARO69850.1   1441  GNRGVTYSSVIPSYSSSYIKGEVPFVPEDGLVTRGYRKVGYLRAPDAPHVPSKTAFMKVPDELCFPYPDPKQPAILSAED  1520
QYU59227.1   1441  GNRGVTYSSVIPSYSSSFVKGEVPYVPEDGLVARGYRKVGYLHASDAPHVPSRTSFVKVPDELCFPYPNPKQPAILSVED  1520
AFM91094.1   1441  GNRGVTYSSVIPSYSSSYVRGEVPYVPEDGLVTRGYRKVGYLRAPDAPHVPSKTSFMKVPDELCFPYPDPKQPAILSAED  1520
ARO69883.1   1441  GNRGVTYSSVIPSYSSSYIKGEVPFVPEDGLVTRGYRKVGYLHAPDAPHVPSKTAFMKVPDELCFPYPDPKQPAILSAED  1520
ARO69879.1   1441  GNRGVTYSSVIPSYSSSYIKGEVPFVPEDGLVTRGYRKVGYLRAPDAPHVPSKTAFMKVPDELCFPYPDPKQPAILSAED  1520
AND76472.1   1441  GNRGVTYSSVIPSYSSSYIKGEVPFVPEDGLVTRGYRKVGYLRAPDAPHVPSKTAFMKVPDELCFPYPDPKQPAILSAED  1520
ARO69846.1   1441  GNRGVTYSSVIPSYSSSYIKGEVPFVPEDGLVTRGYRKVGYLRAPDAPHVPSKTAFMKVPDELCFPYPDPKQPAILSAED  1520
ARO69870.1   1441  GNRGVTYSSVIPSYSSSYIKGEVPFVPEDGLVTRGYRKVGYLHAPDAPHVPSKTAFMKVPDELCFPYPDPKQPAILSAED  1520
ARO69856.1   1441  GNRGVTYSSVIPSYSSSYIKGEVPFVPEDGLVTRGYRKVGYLHAPDAPHVPSKTAFMKVPDELCFPYPDPKQPAILSAED  1520
ARO69847.1   1441  GNRGVTYSSVIPSYSSSFVKGEVPYVPEDGLVTRGYRKVGYLHASDAPHVPSKTSFMRVPDELCFPYPDPKQPAILSAED  1520
```

```
ARO69875.1   1441  GNRGVTYSSVIPSYSSSFVKGEVPYVPEDGLVTRGYRKVGYLHASDAPHVPSKTSFMRVPDELCFPYPDPKQPAILSAED  1520
ARO69895.1   1441  GNRGVTYSSVIPSYSSSFVKGEVPYVPEDGLVTRGYRKVGYLHASDAPHVPSKTSFMRVPDELCFPYPDPKQPAILSAED  1520
ARO69880.1   1441  GNRGVTYSSVIPSYSSSFVKGEVPYVPEDGLVTRGYRKVGYLHASDAPHVPSKTSFMRVPDELCFPYPDPKQPAILSAED  1520
BCT87061.1   1441  GDRNVTFSSVIPNYSSSFIRGEVPYVPEDGIVTNGYRKVGYLHISDAPHVPSKTAFMRVPDELCFPYPNPKQPAILSAED  1520
QYU59225.1   1441  GNRDVTYSSVIPNYSSSFIQGEVPYVPEDGLVTNGYRKVGYLHRSDAPHVPSKTAFIRVPDELCFPYPNPKQPAILSAED  1520
APD26306.1   1441  VDRNITISSVIPNYSSSFVRGDVPYVPEDGITTNGYRKVGYLHRPDAPHVPTKTAFMRVPDELCFPYPNPKQPAILSAED  1520


NP_619689.1  1521  ERLKGTIHEGYTPLRDGMKKFAEPMYLLEEKLLDEVAGDMVQTWYDPGEFLEDISLDQAINGDMDEEYFDPLVMDTSEGY  1600
AWO68042.1   1521  ERLKGTIHEGYTPLRDGMKKFAEPMYLLEEKLLDEVAGDMVQTWYDPGEFLEDISLDQAINGDMDEEYFDPLVMDTSEGY  1600
QYU59223.1   1521  ERLKGTIHEGYTPLRDGMRKFAEPMHLLEEKLLDEVAGDMVQTWYDPGEFLEDISLDQAINGDMDEEYFDPLVMDTSEGY  1600
ARO69887.1   1521  ERLKGTIHEGYTPIRDGMKKFAEPMHLLEEKLLDEVAGDMVQTWYDPGEFLEDISLDQAINGDMDEEYFDPLVMDTSEGY  1600
ARO69840.1   1521  ERLKGTIHEGYTPIRDGMKKFAEPMHLLEEKLLDEVAGDMVQTWYDPGEFLEDISLDQAINGDMDEEYFDPLVMDTSEGY  1600
ARO69892.1   1521  ERLKGTIHEGYTPIRDGMKKFAEPMHLLEEKLLDEVAGDMVQTWYDPGEFLEDISLDQAINGDMDEEYFDPLVMDTSEGY  1600
ARO69862.1   1521  ERLKGTIHEGYTPIRDGMKKFAEPMHLLEEKLLDEVAGDMVQTWYDPGEFLEDISLDQAINGDMDEEYFDPLVMDTSEGY  1600
ARO69844.1   1521  ERLKGTIHEGYTPIRDGMKKFAEPMHLLEEKLLDEVAGDMVQTWYDPGEFLEDISLDQAINGDMDEEYFDPLVMDTSEGY  1600
ARO69896.1   1521  ERLKGTIHEGYTPIRDGMKKFAEPMHLLEEKLLDEVAGDMVQTWYDPGEFLEDISLDQAINGDMDEEYFDPLVMDTSEGY  1600
QGP72541.1   1521  ERLKGTIHEGYTPIRDGMRKFAEPMYLLEEKLLDEVAGDMVQTWYDPGEFLEDISLDQAINGDVDEEYFDPLVMDTSEGY  1600
ARO69901.1   1521  ERLKGTIHEGYTPIRDGMKKFAEPMHLLEEKLLDEVAGDMVQTWYDPGEFLEDISLDQAINGDMDEEYFDPLVMDTSEGY  1600
AYA22299.1   1521  ERLKGTIHEGYTPLKDGMKKFAEPMRLLDEKLLDEVAGDMVHTWYDPGEFLEDISLEQAINGDMDEEYFDPLVMDTSEGY  1600
ARO69890.1   1521  ERLKGTIHEGYTPIRDGMKKFAEPMHLLEEKLLDEVAGDMVQTWYDPGEFLEDISLDQAINGDTDEEYFDPLVMDTSEGY  1600
ARO69859.1   1521  ERLKGTIHEGYTPIRDGMKKFAEPMHLLEEKLLDEVAGDMVQTWYDPGEFLEDISLDQAINGDTDEEYFDPLVMDTSEGY  1600
ARO69905.1   1521  ERLKGTIHEGYTPIRDGMKKFAEPMHLLEEKLLDEVAGDMVQTWYDPGEFLEDISLDQAINGDMDEEYFDPLVMDTSEGY  1600
ARO69871.1   1521  ERLKGTIHEGYTPIKDGMRKFAEPMHLLEEKLLDEVAGDMVQTWYDPGEFLEDISLDQAINGDMDEEYFDPLVMDTSEGY  1600
ARO69848.1   1521  ERLKGTIHEGYTPIRDGMKKFAEPMHLLEEKLLDEVAGDMVQTWYDPGEFLEDISLDQAINGDMDEEYFDPLVMDTSEGY  1600
ARO69874.1   1521  ERLKGTIHEGYTPIRDGMKKFAEPMHLLEEKLLDEVAGDMVQTWYDPGEFLEDISLDQAINGDMDEEYFDPLVMDTSEGY  1600
ARO69882.1   1521  ERLKGTIHEGYTPIRDGMKKFAEPMHLLEEKLLDEVAGDMVQTWYDPGEFLEDISLDQAINGDMDEEYFDPLVMDTSEGY  1600
QYU59221.1   1521  ERLKGTIHEGYTPLRDGMRKFAEPMHLLEEKLLDEVAGDMVQTWYDPGEFLEDISLDQAINGDVNEEFFDPLVMDTSEGY  1600
ARO69842.1   1521  ERLKGTIHEGYTPIRDGMKKFAEPMHLLEEKLLDEVAGDMVQTWYDPGEFLEDISLDQAINGDMDEEYFDPLVMDTSEGY  1600
AFV34731.1   1521  ERLKGTIHEGYTPLKDGMRKFAEPMHLLEEKLLDEVAGDMVQTWYDPGEFLEDISLDQAINGDVDEEYFDPLVMDTSEGY  1600
AND76470.1   1521  ERLKGTIHEGYTPIRDGMKKFAEPMHLLEEKLLDEVAGDMVQTWYDPGEFLEDISLDQAINGDMDEEYFDPLVMDTSEGY  1600
ACZ58632.1   1521  ERLKGTVHEGYTPIRDGMKKFAEPMHLLEEKLLDEVAGDMVQTWYDPGEFLEDISLEQAINGDSEEEYFDPLVMDTSEGY  1600
QBZ78374.1   1521  ERLRGTAHEGYTPLRDGMKKFAEPMHLLDEKLLDEVAGDMVQTWYDPGEFLEDISLDQAINGDMDEEYFDPLVMDTSEGY  1600
QGP72540.1   1521  ERLKGTVHEGYTPIRDGMRKFAEPMHLLEEKLLDEVAGDMVQTWYDPGEFLEDISLDQAINGDMDEEYFDPLVMDTSEGY  1600
QBZ78372.1   1521  ERLRGTAHEGYTPLRDGMKKFAEPMHLLDEKLLDEVAGDMVQTWYDPGEFLEDISLDQAINGDMDEEYFDPLVMDTSEGY  1600
QYU59224.1   1521  ERLKGTIYEGYTPLRDGMKKFAEPMHLLEEKLLDEVAGDMVQTWYDPGEFLEDISLDQAINGDMDEEYFDPLVMDTSEGY  1600
ARO69877.1   1521  ERLKGTIHEGYTPIRDGMKKFAEPMHLLEEKLLDEVAGDMVQTWYDPGEFLEDISLDQAINGDMDEEYFDPLVMDTSEGY  1600
ARO69858.1   1521  ERLKGTIHEGYTPIRDGMKKFAEPMHLLEEKLLDEVAGDMVQTWYDPGEFLEDISLDQAINGDMDEEYFDPLVMDTSEGY  1600
ARO69855.1   1521  ERLKGTIHEGYTPIRDGMKKFAEPMHLLEEKLLDEVAGDMVQTWYDPGEFLEDISLDQAINGDVDEEYFDPLVMDTSEGY  1600
QGP72542.1   1521  ERLKGTVHEGYTPIRDGMRKFAEPMHLLEEKLLDEVAGDMVQTWYDPGEFLEDISLDQAINGDMDEEYFDPLVMDTSEGY  1600
AYQ93055.1   1521  ERLKGTIHEGYTPLRDGMKKFAEPMHLLEEKLLDEVAGDMVQTWYDPGEFLEDISLDQAINGDVDEEYFDPLMMDTSEGY  1600
AYQ93053.1   1521  ERLKGTIHEGYTPLRDGMKKFAEPMHLLEEKLLDEVAGDMVQTWYDPGEFLEDITLDQAINGDMNEEYFDPLVMDTSEGY  1600
AYQ93051.1   1521  ERLKGTIHEGYTPLRDGMRKFAEPMHLLEEKLLDEVAGDMVQTWYDPGEFLEDISLDQAINGDMEEEYFDPLVMDTSEGY  1600
AFI71813.1   1521  ERLRGTVHEGYTPLRDGMRKFAEPMHLLEEKLLDEVAGDMVQTWYDPNEFLEDISLDQAINGDMDEEYFDPLVMDTSEGY  1600
QGP72539.1   1521  ERLKGTVHEGYTPIRDGMRKFAEPMHLLEEKLLDEVAGDMVQTWYDPGEFLEDISLDQAINGDMDEEYFDPLVMDTSEGY  1600
QYU59226.1   1521  ERLKGTIHEGYTPLKDGMRKFAEPMHLLEEKLLDEVAGDMVQTWYDPGEFLEDISLDQAINGDMDEEYFDPLVMDTSEGY  1600
ARO69852.1   1521  ERLKGTIHEGYTPIRDGMKKFAEPMHLLEEKLLDEVAGDMVQTWYDPGEFLEDISLDQAINGDMDEEYFDPLVMDTSEGY  1600
QGP72543.1   1521  ERLKGTIHEGYTPLRDGMKKFAEPMHLLEEKLLDEVAGDMVQTWYDPGEFLEDISLDQAINGDMDEEYFDPLVMDTSEGY  1600
QYU59219.1   1521  ERLKGTIHEGYTPIRDGMKKFAEPMQLLEEKLLDEVAGDMVQTWYDPGEFLEDISLDQAINGDMDEEFFDPLVMDTSEGY  1600
AGT42200.1   1521  ERLRGTVHEGYAPLRDGMRKFAEPMHLLEEKLLDEVAGDMVQTWYDPNEFLEDISLDQAINGDMDEEYFDPLVMDTSEGY  1600
ACZ58633.1   1521  ERLKGTIHEGYTPLRNGMRKFAEPMHLLEEKLLDEVAGDMVQTWYDSGEFLEDISLDQAINGDMDEEYFDPLVMDTSEGY  1600
QBZ78369.1   1521  ERLKGTIHEGYTPLRDGMRKFAEPMHLLDEKLLDEVAGDMVQTWYDPGEFLEDISLDQAINGDMDEEYFDPLVMDTSEGY  1600
ARO69851.1   1521  ERLKGTIHEGYTPLRDGMKKFAEPIHLLEEKLLDEVAGDMVQTWYDPGEFLEDISLDQAINGDVEEEYFDPLVMDTSEGY  1600
QYU59220.1   1521  ERLKGTSHEGYTPLRDGMKKFAEPMHLLEEKLLDEVAGDMVQTWYDPGEFLEDISLDLAINGDIDEEYFDPLVMDTSEGY  1600
AFV34755.1   1521  ERLKGTIHEGYTPLRDGMRKFAEPMHLLEEKLLDEVAGDMVQTWYDPGEFLEDISLDQAINGDINEEYFDPLVMDTSEGY  1600
QGP72544.1   1521  ERLKGTFHEGYTPIKDGMKKFAEPMHLLEEKLLDEVAGDMVHTWYDPGEFLEDISLDQAINGDLDEEYFDPLVMDTSEGY  1600
QYA72352.1   1521  ERLKGTIHEGYTPIKDGMKKFAEPLQLLEEKLLDEVAGDMVQTWYDPGEFLEDISLDQAINGDMDEEYFDPLVMDTSEGY  1600
ARO69854.1   1521  ERLKGTIHEGYTPIRDGMKKFAEPMHLLDEKLLDEVAGDMVQTWYDPGEFLEDISLDQAINGDTEEEFFDPLVMDTSEGY  1600
ARO69860.1   1521  ERLKGTIHEGYTPVRDGMKKFAEPMHLLDEKLLDEVAGDMVQTWYDPGEFLEDISLDQAINGDTEEEFFDPLVMDTSEGY  1600
ARO69863.1   1521  ERLKGTIHEGYTPVRDGMKKFAEPMHLLDEKLLDEVAGDMVQTWYDPGEFLEDISLDQAINGDTEEEFFDPLVMDTSEGY  1600
DAZ85707.1   1521  ERLKGTIHEGYTPLRDGMKKFAEPMHLLDEKLLDEVAGDMVQTWYDPDEFLEDISLDQAINGDMNEEYFDPLVMDTSEGY  1600
AWO67781.1   1521  ERLKGTTHEGYTPLRDGMRKFAEPMHLLEEKLLDEVAGDMVQTWYDPGEFLEDITLDQAINGDINEEYFDPLMMDTSEGY  1600
QBZ78367.1   1521  ERLKGTIHEGYTPLRNGMRKFAEPMHLLEEKLLDEVAGDMVQTWYDPGEFLEDISLDQAINGDMEEEYFDPLVMDTSEGY  1600
ARO69873.1   1521  ERLKGTIHEGYTPVRDGMKKFAEPMHLLDEKLLDEVAGDMVQTWYDPGEFLEDISLDQAINGDTEEEFFDPLVMDTSEGY  1600
AND76471.1   1521  ERLKGTIHEGYTPIRDGMKKFAEPMHLLDEKLLDEVAGDMVQTWYDPGEFLEDISLDQAINGDTEEEFFDPLVMDTSEGY  1600
ARO69849.1   1521  ERLKGTIHEGYTPVRDGMKKFAEPMHLLDEKLLDEVAGDMVQTWYDPGEFLEDISLDQAINGDTEEDFFDPLVMDTSEGY  1600
ARO69889.1   1521  ERLKGTIHEGYTPVRDGMKKFAEPMHLLDEKLLDEVAGDMVQTWYDPGEFLEDISLDQAINGDAEEEFFDPLVMDTSEGY  1600
ARO69867.1   1521  ERLKGTIHEGYTPVRDGMKKFAEPMHLLDEKLLDEVAGDMVQTWYDPGEFLEDISLDQAINGDTEEEFFDPLVMDTSEGY  1600
ARO69904.1   1521  ERLKGTIHEGYTPVRDGMKKFAEPMHLLDEKLLDEVAGDMVQTWYDPGEFLEDISLDQAINGDTEEEFFDPLVMDTSEGY  1600
ARO69869.1   1521  ERLKGTIHEGYTPVRDGMKKFAEPMHLLDEKLLDEVAGDMVQTWYDPGEFLEDISLDQAINGDTEEEFFDPLVMDTSEGY  1600
ARO69884.1   1521  ERLKGTIHEGYTPVRDGMKKFAEPMHLLDEKLLDEVAGDMVQTWYDPGEFLEDISLDQAINGDTEEEFFDPLVMDTSEGY  1600
ARO69878.1   1521  ERLKGTIHEGYTPIRDGMKKFAEPMHLLDEKLLDEVAGDMVQTWYDPGEFLEDISLDQAINGDTEEEFFDPLVMDTSEGY  1600
ARO69885.1   1521  ERLKGTIHEGYTPVRDGMKKFAEPMHLLDEKLLDEVAGDMVQTWYDPGEFLEDISLDQAINGDTEEEFFDPLVMDTSEGY  1600
ARO69841.1   1521  ERLKGTIHEGYTPVRDGMKKFAEPMHLLDEKLLDEVAGDMVQTWYDPGEFLEDISLDQAINGDTEEEFFDPLVMDTSEGY  1600
ARO69891.1   1521  ERLKGTIHEGYTPVRDGMKKFAEPMHLLDEKLLDEVAGDMVQTWYDPGEFLEDISLDQAINGDTEEEFFDPLVMDTSEGY  1600
ARO69868.1   1521  ERLKGTIHEGYTPVRDGMKKFAEPMHLLDEKLLDEVAGDMVQTWYDPGEFLEDISLDQAINGDTEEEFFDPLVMDTSEGY  1600
ARO69900.1   1521  ERLKGTIHEGYTPVRDGMKKFAEPMHLLDEKLLDEVAGDMVQTWYDPGEFLEDISLDQAINGDTEEEFFDPLVMDTSEGY  1600
DAZ85706.1   1521  ERLKGTIHEGYTPLRDGMKKFAEPMHLLEEKLLDEVAGDMVQTWYDPGEFLEDISLDQAINGDMDEEYFDPLVMDTSEGY  1600
ARO69899.1   1521  ERLKGTIHEGYTPVRDGMKKFAEPMHLLDEKLLDEVAGDMVQTWYDPGEFLEDISLDQAINGDTEEEFFDPLVMDTSEGY  1600
ARO69898.1   1521  ERLKGTIHEGYTPVRDGMKKFAEPMHLLDEKLLDEVAGDMVQTWYDPGEFLEDISLDQAINGDTEEEFFDPLVMDTSEGY  1600
AYA22297.1   1521  ERLKGTIHEGYTPLKDGMRKFAEPMHFLEEKLLDEVAGDMVQTWYDPGEFLEDISLDQAINGDMDEEYFDPLVMDTSEGY  1600
QYU59222.1   1521  ERLKGTIHEGYTPLKDGMRKFAEPMHLLDEKLLDEVAGDMVHTWYDPGEFLEDISLDQAINGDMDEEYFDPLVMDTSEGY  1600
ARO69845.1   1521  ERLKGTIHEGYTPVRDGMKKFAEPMHLLDEKLLDEVAGDMVQTWYDPGEFLEDISLDQAINGDTEEEFFDPLVMDTSEGY  1600
AND76473.1   1521  ERLKGTIHEGYTPVRDGMKKFAEPMHLLDEKLLDEVAGDMVQTWYDPGEFLEDISLDQAINGDTEEEFFDPLVMDTSEGY  1600
ARO69857.1   1521  ERLKGTIHEGYTPVRDGMKKFAEPMHLLDEKLLDEVAGDMVQTWYDPGEFLEDISLDQAINGDTEEEFFDPLVMDTSEGY  1600
DAZ85708.1   1521  ERLKGTSHEGYTPLRDGMRKFAEPMHLLDEKLLDEVAGDMVQTWYDPGEFLEDISLDQAINGDTEEEYFDPLVMDTSEGY  1600
ARO69881.1   1521  ERLKGTIHEGYTPIKDGMRKFAEPMHLLEEKLLDEVAGDMVQTWYDPGEFLEDISLDQAINGDMDEEYFDPLVMDTSEGY  1600
ARO69893.1   1521  ERLKGTIHEGYTPIKDGMRKFAEPMHLLEEKLLDEVAGDMVQTWYDPGEFLEDISLDQAINGDMDEEYFDPLVMDTSEGY  1600
```

```
ARO69897.1   1521  ERLKGTIHEGYTPIKDGMRKFAEPMHLLEEKLLDEVAGDMVQTWYDPGEFLEDISLDQAINGDMDEEYFDPLVMDTSEGY  1600
ARO69872.1   1521  ERLKGTIHEGYTPIKDGMRKFAEPMHLLEEKLLDEVAGDMVQTWYDPGEFLEDISLDQAINGDMDEEYFDPLVMDTSEGY  1600
ARO69843.1   1521  ERLKGTIHEGYTPIKDGMRKFAEPMHLLEEKLLDEVAGDMVQTWYDPGEFLEDISLDQAINGDMDEEYFDPLVMDTSEGY  1600
ARO69886.1   1521  ERLKGTIHEGYTPIKDGMRKFAEPMHLLEEKLLDEVAGDMVQTWYDPGEFLEDISLDQAINGDMDEEYFDPLVMDTSEGY  1600
ARO69864.1   1521  ERLKGTIHEGYTPIKDGMRKFAEPMHLLEEKLLDEVAGDMVQTWYDPGEFLEDISLDQAINGDMDEEYFDPLVMDTSEGY  1600
ARO69876.1   1521  ERLKGTIHEGYTPIKDGMRKFAEPMHLLEEKLLDEVAGDMVQTWYDPGEFLEDISLDQAINGDMDEEYFDPLVMDTSEGY  1600
ARO69888.1   1521  ERLKGTIHEGYTPIKDGMRKFAEPMHLLEEKLLDEVAGDMVQTWYDPGEFLEDISLDQAINGDMDEEYFDPLVMDTSEGY  1600
ARO69853.1   1521  ERLKGTIHEGYTPIKDGMRKFAEPMHLLEEKLLDEVAGDMVQTWYDPGEFLEDISLDQAINGDMDEEYFDPLVMDTSEGY  1600
ARO69903.1   1521  ERLKGTIHEGYTPIKDGMRKFAEPMHLLEEKLLDEVAGDMVQTWYDPGEFLEDISLDQAINGDMDEEYFDPLVMDTSEGY  1600
ARO69865.1   1521  ERLKGTIHEGYTPIKDGMRKFAEPMHLLEEKLLDEVAGDMVQTWYDPGEFLEDISLDQAINGDMDEEYFDPLVMDTSEGY  1600
ARO69902.1   1521  ERLKGTIHEGYTPIKDGMRKFAEPMHLLEEKLLDEVAGDMVQTWYDPGEFLEDISLDQAINGDMDEEYFDPLVMDTSEGY  1600
ARO69861.1   1521  ERLKGTIHEGYTPIKDGMRKFAEPMHLLEEKLLDEVAGDMVQTWYDPGEFLEDISLDQAINGDMDEEYFDPLVMDTSEGY  1600
ARO69850.1   1521  ERLKGTIHEGYTPIKDGMRKFAEPMHLLEEKLLDEVAGDMVQTWYDPGEFLEDISLDQAINGDMDEEYFDPLVMDTSEGY  1600
QYU59227.1   1521  ERLKGTIHEGYTPLKDGMKKFAEPMHLLEEKLLDEVAGDMVQTWYDPGEFLEDISLDQAINGDVDEEYFDPLVMDTSEGY  1600
AFM91094.1   1521  VRLKGTFHEGYTPLKEGMRKFSEPIHLLEEKLLDEVAGDMVQTWYDPGEFLEDISLEQAINGDMDEEYFDPLVMDTSEGY  1600
ARO69883.1   1521  ERLKGTIHEGYTPIKDGMRKFAEPMHLLEEKLLDEVAGDMVQTWYDPGEFLEDISLDQAINGDMDEEYFDPLVMDTSEGY  1600
ARO69879.1   1521  ERLKGTIHEGYTPIKDGMRKFAEPMHLLEEKLLDEVAGDMVQTWYDPGEFLEDISLDQAINGDMDEEYFDPLVMDTSEGY  1600
AND76472.1   1521  ERLKGTIHEGYTPIKDGMRKFAEPMHLLEEKLLDEVAGDMVQTWYDPGEFLEDISLDQAINGDMDEEYFDPLVMDTSEGY  1600
ARO69846.1   1521  ERLKGTIHEGYTPIKDGMRKFAEPMHLLEEKLLDEVAGDMVQTWYDPGEFLEDISLDQAINGDMDEEYFDPLVMDTSEGY  1600
ARO69870.1   1521  ERLKGTIHEGYTPIKDGMRKFAEPMHLLEEKLLDEVAGDMVQTWYDPGEFLEDISLDQAINGDMDEEYFDPLVMDTSEGY  1600
ARO69856.1   1521  ERLKGTIHEGYTPIKDGMRKFAEPMHLLEEKLLDEVAGDMVQTWYDPGEFLEDISLDQAINGDMDEEYFDPLVMDTSEGY  1600
ARO69847.1   1521  ERLKGTIHEGYTPVRDGMRKFAEPMHLLDEKLLDEVAGDMVQTWYDPGEFLEDISLDQAINGDAEEEFFDPLVMDTSEGY  1600
ARO69875.1   1521  ERLKGTIHEGYTPVRDGMRKFAEPMHLLDEKLLDEVAGDMVQTWYDPGEFLEDISLDQAINGDAEEEFFDPLVMDTSEGY  1600
ARO69895.1   1521  ERLKGTIHEGYTPVRDGMRKFAEPMHLLDEKLLDEVAGDMVQTWYDPGEFLEDISLDQAINGDAEEEFFDPLVMDTSEGY  1600
ARO69880.1   1521  ERLKGTIHEGYTPVRDGMRKFAEPMHLLDEKLLDEVAGDMVQTWYDPGEFLEDISLDQAINGDAEEEFFDPLVMDTSEGY  1600
BCT87061.1   1521  ERLKGTIHEGYTPLKEGMKKFAEPMGLLDEKLLDEVAGDMVHTWYDPGEFLEDISLDQAINGDEDEEYFDPLVMDTSEGY  1600
QYU59225.1   1521  ERLQGTSHEGYTPVRDGMKKFAEPMHLLEEKLLDEVAGDMVHTWYDPGEFLEDISLDQAINGDDDEEFFDPLVMDTSEGY  1600
APD26306.1   1521  ERLKGTIHEGYEPVKDGMKKFAEPMGLLDEKLLDEVAGDMVHTWYDPGEFLEDISLDLAINGDDNEEYFDPLVMDTSEGY  1600


NP_619689.1  1601  PDVLDRKPGEKGKARFFVGEPGNRAFVAGCNPEKAYYQLEEDSKTKIPSLVSIETPKDERLKRSKIDTPGTRLFSVLPLA  1680
AWO68042.1   1601  PDVLDRKPGEKGKARFFVGEPGNRAFVAGCNPEKAYYQLEEDSKTKIPSLVSIETPKDERLKRSKIDTPGTRLFSVLPLA  1680
QYU59223.1   1601  PDVLDRKPGEKGKARFFVGEPGNRVFVAGCKPEKAYYQLEEDSKTKVPSLVSIETPKDERLKRSKIDTPGTRLFSVLPLA  1680
ARO69887.1   1601  PDVLYRKPGEKGKARFFVGEPGSRIFVAGCNPEKAYYQLEEDSKTKVPSLVSIETPKDERLKRSKIDTPSTRLFSVLPLA  1680
ARO69840.1   1601  PDVLYRKPGEKGKARFFVGEPGSRIFVAGCNPEKAYYQLEEDSKTKVPSLVSIETPKDERLKRSKIDTPSTRLFSVLPLA  1680
ARO69892.1   1601  PDVLYRKPGEKGKARFFVGEPGSRIFVAGCNPEKAYYQLEEDSKTKVPSLVSIETPKDERLKRSKIDTPSTRLFSVLPLA  1680
ARO69862.1   1601  PDVLYRKPGEKGKARFFVGEPGSRIFVAGCNPEKAYYQLEEDSKTKVPSLVSIETPKDERLKRSKIDTPSTRLFSVLPLA  1680
ARO69844.1   1601  PDVLYRKPGEKGKARFFVGEPGSRIFVAGCNPEKAYYQLEEDSKTKVPSLVSIETPKDERLKRSKIDTPSTRLFSVLPLA  1680
ARO69896.1   1601  PDVLYRKPGEKGKARFFVGEPGSRIFVAGCNPEKAYYQLEEDSKTKVPSLVSIETPKDERLKRSKIDTPSTRLFSVLPLA  1680
QGP72541.1   1601  PDVLDRKPGEKGKARFFVGEPGSRVFVAGCNPEKAYYQLEEDSKTKVPSLVSIETPKDERLKRSKIDTPGTRLFSVLPLA  1680
ARO69901.1   1601  PDVLYRKPGEKGKARFFVGEPGSRIFVAGCNPEKAYYQLEEDSKTKVPSLVSIETPKDERLKRSKIDTPSTRLFSVLPLA  1680
AYA22299.1   1601  PDVLDRKPGEKGKARFFVGEPGSRIFVAGCNPEKAYYQLEEDSKTKIPSLVSIETPKDERLKRSKIDTPGTRLFSVLPLA  1680
ARO69890.1   1601  PDVLYRKPGEKGKARFFVGEPGSRIFVAGCNPEKAYYQLEEDSKTKVPSLVSIETPKDERLKRSKIDTPSTRLFSVLPLA  1680
ARO69859.1   1601  PDVLYRKPGEKGKARFFVGEPGSRIFVAGCNPEKAYCQLEEDSKTKVPSLVSIETPKDERLKRSKIDTPSTRLFSVLPLA  1680
ARO69905.1   1601  PDVLYRKPGEKGKARFFVGEPGSRIFVAGCNPEKAYYQLEEDSKTKVPSLVSIETPKDERLKRSKIDTPSTRLFSVLPLA  1680
ARO69871.1   1601  PDVLCRKPGEKGKARFFVGEPGSRIFVAGCNPEKAYYQLEEDSKTKVPSLVSIETPKDERLKRSKIDTPSTRLFSVLPLA  1680
ARO69848.1   1601  PDVLYRKPGEKGKARFFVGEPGSRIFVAGCNPEKAYYQLEEDSKTKVPSLVSIETPKDERLKRSKIDTPSTRLFSVLPLA  1680
ARO69874.1   1601  PDVLYRKPGEKGKARFFVGEPGSRIFVAGCNPEKAYYQLEEDSKTKVPSLVSIETPKDERLKRSKIDTPSTRLFSVLPLA  1680
ARO69882.1   1601  PDVLYRKPGEKGKARFFVGEPGSRIFVAGCNPEKAYYQLEEDSKTKVPSLVSIETPKDERLKRSKIDTPSTRLFSVLPLA  1680
QYU59221.1   1601  PDVLDRKPGEKGKARFFVGEPGNRVFVAGCNPEKAYYQLEEDSKTKVPSLVSIETPKDERLKRSKIDTPGTRLFSVLPLA  1680
ARO69842.1   1601  PDVLYRKPGEKGKARFFVGEPGSRIFVAGCNPEKAYYQLEEDSKTKVPSLVSIETPKDERLKRSKIDTPSTRLFSVLPLA  1680
AFV34731.1   1601  PDVLDRKPGEKGKARFFVGEPGNRIFVAGCNPEKAYYQLDEDSKTKVPSLVSIETPKDERLKRSKIDTPGTRLFSVLPLA  1680
AND76470.1   1601  PDVLYRKPGEKGKARFFVGEPGSRIFVAGCNPEKAYYQLEEDSKTKVPSLVSIETPKDERLKRSKIDTPSTRLFSVLPLA  1680
ACZ58632.1   1601  PDVLGRKPGEKGKARFFVGEPGSRVFVAGCNPEKAYYQLVEDSKTKVPSLVSIETPKDERLKRSKIDTPGTRLFSVLPLA  1680
QBZ78374.1   1601  PDVLDRKPGEKGKARFFVGEPGSRIFVAGCNPEKAYYQLEEDSKTKVPSLVSIETPKDERLKRSKIDTPGTRLFSVLPLA  1680
QGP72540.1   1601  PDVLDRKPGEKGKARFFVGEPGSRVFVAGCNPEKAYYQLEEDSKTKVPSLVSIETPKDERLKRSKIDTPGTRLFSVLPLA  1680
QBZ78372.1   1601  PDVLDRKPGEKGKARFFVGEPGSRIFVAGCNPEKAYYQLEEDSKTKVPSLVSIETPKDERLKRSKIDTPGTRLFSVLPLA  1680
QYU59224.1   1601  PDVLDRKPGEKGKARFFVGEPGNRVFVAGCNPEKAYYQLDEDSKTKVPSLVSIETPKDERLKRSKIDIPGTRLFSVLPLA  1680
ARO69877.1   1601  PDVLYRKPGEKGKARFFVGEPGSRIFVAGCNPEKAYYQLEEDSKTKVPSLVSIETPKDERLKRSKIDTPSTRLFSVLPLA  1680
ARO69858.1   1601  PDVLYRKPGEKGKARFFVGEPGSRIFVAGCNPEKAYYQLEEDSKTKVPSLVSIETPKDERLKRSKIDTPSTRLFSVLPLA  1680
ARO69855.1   1601  PDVLHRKPGEKGKARFFVGEPGSRIFVAGCNPEKAYYQLEEDSKTKVPSLVSIETPKDERLKRSKIDTPSTRLFSVLPLA  1680
QGP72542.1   1601  PDVLDRKPGEKGKARFFVGEPGSRVFVAGCNPEKAYYQLEEDSKTKVPSLVSIETPKDERLKRSKIDTPGTRLFSVLPLA  1680
AYQ93055.1   1601  PDVLDRKPGEKGKARFFVGEPGSRVFVAGCNPEKAYYRLEEDSKTKVPALVSIETPKDERLKRSKIDTPGTRLFSVLPLA  1680
AYQ93053.1   1601  PDVLDRKPGEKGKARFFVGEPGSRVFVAGCNPEKAYYQLEEDSKTKVPSLVSIETPKDERLKRSKIDTPGTRLFSVLPLA  1680
AYQ93051.1   1601  PDVLDRKPGEKGKARFFVGEPGSRVFVAGCNPERAYYQLEEDSKTKVPSLVSIETPKDERLKRSKIDTPGTRLFSVLPLA  1680
AFI71813.1   1601  PDVLDRKPGEKGKARFFVGEPGNRVFVAGCSPEKAYYQLEEDSKTKVPSLISIETPKDERLKRSKIDTPGTRLFSVLPLA  1680
QGP72539.1   1601  PDVLDRKPGEKGKARFFVGEPGSRVFVAGCNPEKAYYQLEEDSKTKVPSLVSIETPKDERLKRSKIDTPGTRLFSVLPLA  1680
QYU59226.1   1601  PEVLKRKPGEKGKARFFVGEPGNRVFVTGCNPEKAYYQLEEDSKTKVPSLVSIETPKDERLKRSKIDTPGTRLFSVLPLA  1680
ARO69852.1   1601  PDVLYRKPGEKGKARFFVGEPGSRIFVAGCNPEKAYYQLEEDSKTKVPSLVSIETPKDERLKRSKIDTPSTRLFSVLPLA  1680
QGP72543.1   1601  PDVLDRKPGEKGKARFFVGEPGTRVFVAGCSPEKAYYQLEEDSKTKVPSLVSIETPKDERLKRSKIDTPGTRLFSVLPLA  1680
QYU59219.1   1601  PDVLDRKPGEKGKARFFVGEPGSRVFVAGCNPEKAYYQLEEDSKTKVPSLVSIETPKDERLKRSKIETPGTRLFSVLPLS  1680
AGT42200.1   1601  PDVLDRKPGEKGKARFFVGEPGNRVFVAGCSPEKAYYQLEEDSKTKVPSLVSIETPKDERLKRSKIDTPGTRLFSVLPLA  1680
ACZ58633.1   1601  PDVLDRKPGEKGKARFFVGEPGNRVFVAGCNPEKAYYQLEEESKTKVPSLVSIETPKDERLKRSKIDTPSTRLFSVLPLA  1680
QBZ78369.1   1601  PDVIDRKPGEKGKARFFVGEPGNRVFVAGCNPEKAYYQLDEDCKTKVPALVSIETPKDERLKRSKIDTPGTRLFSVLPLA  1680
ARO69851.1   1601  PDVLDRKPGEKGKARFFVGEPGSRVFVSGCNPEKAYYQLEEDSKTKVPALVSIETPKDERLKRSKIDTPSTRLFSVLPLA  1680
QYU59220.1   1601  PDVLDRKPGEKGKARFFVGEPGSRVFVAGCNPEKAYYQLEEDSKTKVPSLVSIETPKDERLKRSKIETPGTRLFSVLPLA  1680
AFV34755.1   1601  PDVLDRKPGEKGKARFFVGEPGNRVFVAGCNPEKAYFQLDEDSKTKVPSLVSIETPKDERLKRSKIDTPGTRLFSVLPLA  1680
QGP72544.1   1601  PDVLDRKPGEKGKARFFVGEPGSRVFVAGCNPEKAYYQLEEDSKIKVPSLVSIETPKDERLKRSKIDTPGTRLFSVLPLA  1680
QYA72352.1   1601  PDVLDRKPGERGKARFFVGEPGSRVFVAGCNPEKAYYQLEEDSKTKVPSLVSIETPKDERLKRSKIDTPGTRLFSVLPLA  1680
ARO69854.1   1601  PDVLDRKPGEKGKARFFVGESGNRIFVAGCNPEKAYYQLEEDSKTKVPSLVSIETPKDERLKRSKIDTPGTRLFSVLPLA  1680
ARO69860.1   1601  PDVLDRKPGEKGKARFFVGEPGNRIFVAGCNPEKAYYQLEEDSKTKVPSLVSIETPKDERLKRSKIDTPGTRLFSVLPLA  1680
ARO69863.1   1601  PDVLDRKPGEKGKARFFVGEPGNRIFVAGCNPEKAYYQLEEDSKTKVPSLVSIETPKDERLKRSKIDTPGTRLFSVLPLA  1680
DAZ85707.1   1601  PDVLDRKPGEKGKARFFVGEPGSRVFVAGSGPEKAYYQLEEDSKTKVPSLVSIETPKDERLKRSKIDTPGTRLFSVLPLA  1680
AWO67781.1   1601  PDVLDRKPGEKGKARFFVGEPGSRVFVTGCNPEKAYHQLGEDSKTKIPSLVSIETPKDERLKRSKIDTPGTRLFSVLPLA  1680
QBZ78367.1   1601  PDVLDRKPGEKGKARFFVGEPGNRVFVAGCKPEKAYYQLEEDSKTKVPSLVSIETPKDERLKRSKIDTPNTRLFSVLPLA  1680
ARO69873.1   1601  PDVLDRKPGEKGKARFFVGEPGNRIFVAGCNPEKAYYQLEEDSKTKVPSLVSIETPKDERLKRSKIDTPGTRLFSVLPLA  1680
AND76471.1   1601  PDVLDRKPGEKGKARFFVGEPGNRIFVAGCNPEKAYYQLEEDSKTKVPSLVSIETPKDERLKRSKIDTPGTRLFSVLPLA  1680
ARO69849.1   1601  PDVLDRKPGEKGKARFFVGEPGNRIFVAGCNPEKAYYQLEEDSKTKVPSLVSIETPKDERLKRSKIDTPGTRLFSVLPLA  1680
```

```
ARO69889.1   1601  PDVLDRKPGEKGKARFFVGEPGNRIFVAGCNPEKAYYQLEEDSKTKVPSLVSIETPKDERLKRSKIDTPGTRLFSVLPLA  1680
ARO69867.1   1601  PDVLDRKPGEKGKARFFVGEPGNRIFVAGCNPEKAYYQLEEDSKTKVPSLVSIETPKDERLKRSKIDTPGTRLFSVLPLA  1680
ARO69904.1   1601  PDVLDRKPGEKGKARFFVGEPGNRIFVAGCNPEKAYYQLEEDSKTKVPSLVSIETPKDERLKRSKIDTPGTRLFSVLPLA  1680
ARO69869.1   1601  PDVLDRKPGEKGKARFFVGEPGNRIFVAGCNPEKAYYQLEEDSKTKVPSLVSIETPKDERLKRSKIDTPGTRLFSVLPLA  1680
ARO69884.1   1601  PDVLDRKPGEKGKARFFVGEPGNRIFVAGCNPEKAYYQLEEDSKTKVPSLVSIETPKDERLKRSKIDTPGTRLFSVLPLA  1680
ARO69878.1   1601  PDVLDRKPGEKGKARFFVGEPGNRIFVAGCNPEKAYYQLEEDSKTKVPSLVSIETPKDERLKRSKIDTPGTRLFSVLPLA  1680
ARO69885.1   1601  PDVLDRKPGEKGKARFFVGEPGNRIFVAGCNPEKAYYQLEEDSKTKVPSLVSIETPKDERLKRSKIDTPGTRLFSVLPLA  1680
ARO69841.1   1601  PDVLDRKPGEKGKARFFVGEPGNRIFVAGCNPEKAYYQLEEDSKTKVPSLVSIETPKDERLKRSKIDTPGTRLFSVLPLA  1680
ARO69891.1   1601  PDVLDRKPGEKGKARFFVGEPGNRIFVAGCNPEKAYYQLEEDSKTKVPSLVSIETPKDERLKRSKIDTPGTRLFSVLPLA  1680
ARO69868.1   1601  PDVLDRKPGEKGKARFFVGEPGNRIFVAGCNPEKAYYQLEEDSKTKVPSLVSIETPKDERLKRSKIDTPGTRLFSVLPLA  1680
ARO69900.1   1601  PDVLDRKPGEKGKARFFVGESGNRIFVAGCNPEKAYYQLEEDSKTKVPSLVSIETPKDERLKRSKIDTPGTRLFSVLPLA  1680
DAZ85706.1   1601  PDVLDRKPGEKGKVRFFVGEPGSRVFVAGCNPEKAYYQLEEDSKTKVPSLVSIETPKDERLKRSKIDTPGTRLFSVLPLA  1680
ARO69899.1   1601  PDVLDRKPGEKGKARFFVGEPGNRIFVAGCNPEKAYYQLEEDSKTKVPSLVSIETPKDERLKRSKIDTPGTRLFSVLPLA  1680
ARO69898.1   1601  PDVLDRKPGEKGKARFFVGEPGNRIFVAGCNPEKAYYQLEEDSKTKVPSLVSIETPKDERLKRSKIDTPGTRLFSVLPLA  1680
AYA22297.1   1601  PDVLGRAPGEKGKARFFVGEPGNRVFVAGCNPEKAYYQLEEDSKTKVPSLVSIETPKDERLKRSKIDTPGTRLFSVLPLA  1680
QYU59222.1   1601  PDVLDRKPGERGKARFFVGEPGNRVFVAGCNPEKAYYQLEEDSKAKVPSLVSIETPKDERLKRSKIDTPGTRLFSVLPLA  1680
ARO69845.1   1601  PDVLDRKPGEKGKARFFVGEPGNRIFVAGCNPEKAYYQLEEDSKTKVPSLVSIETPKDERLKRSKIDTPGTRLFSVLPLA  1680
AND76473.1   1601  PDVLDRKPGEKGKARFFVGEPGNRIFVAGCNPEKAYYQLEEDSKTKVPSLVSIETPKDERLKRSKIDTPGTRLFSVLPLA  1680
ARO69857.1   1601  PDVLDRKPGEKGKARFFVGEPGNRIFVAGCNPEKAYYQLEEDSKTKVPSLVSIETPKDERLKRSKIDTPGTRLFSVLPLA  1680
DAZ85708.1   1601  PDVLDRKPGDKGKARFFVGEPGSRVFVAGCNPERAYYQLEEDSKTKVPSLVSIETPKDERLKRSKIETPGTRLFSVLPLA  1680
ARO69881.1   1601  PDVLCRKPGEKGKARFFVGEPGSRIFVAGCNPEKAYYQLEEDSKTKVPSLVSIETPKDERLKRSKIDTPSTRLFSVLPLA  1680
ARO69893.1   1601  PDVLCRKPGEKGKARFFVGEPGSRIFVAGCNPEKAYYQLEEDSKTKVPSLVSIETPKDERLKRSKIDTPSTRLFSVLPLA  1680
ARO69897.1   1601  PDVLCRKPGEKGKARFFVGEPGSRIFVAGCNPEKAYYQLEEDSKTKVPSLVSIETPKDERLKRSKIDTPSTRLFSVLPLA  1680
ARO69872.1   1601  PDVLCRKPGEKGKARFFVGEPGSRIFVAGCNPEKAYYQLEEDSKTKVPSLVSIETPKDERLKRSKIDTPSTRLFSVLPLA  1680
ARO69843.1   1601  PDVLCRKPGEKGKARFFVGEPGSRIFVAGCNPEKAYYQLEEDSKTKVPSLVSIETPKDERLKRSKIDTPSTRLFSVLPLA  1680
ARO69886.1   1601  PDVLCRKPGEKGKARFFVGEPGSRIFVAGCNPEKAYYQLEEDSKTKVPSLVSIETPKDERLKRSKIDTPSTRLFSVLPLA  1680
ARO69864.1   1601  PDVLCRKPGEKGKARFFVGEPGSRIFVAGCNPEKAYYQLEEDSKTKVPSLVSIETPKDERLKRSKIDTPSTRLFSVLPLA  1680
ARO69876.1   1601  PDVLCRKPGEKGKARFFVGEPGSRIFVAGCNPEKAYYQLEEDSKTKVPSLVSIETPKDERLKRSKIDTPSTRLFSVLPLA  1680
ARO69888.1   1601  PDVLCRKPGEKGKARFFVGEPGSRIFVAGCNPEKAYYQLEEDSKTKVPSLVSIETPKDERLKRSKIDTPSTRLFSVLPLA  1680
ARO69853.1   1601  PDVLCRKPGEKGKARFFVGEPGSRIFVAGCNPEKAYYQLEEDSKTKVPSLVSIETPKDERLKRSKIDTPSTRLFSVLPLA  1680
ARO69903.1   1601  PDVLCRKPGEKGKARFFVGEPGSRIFVAGCNPEKAYYQLEEDSKTKVPSLVSIETPKDERLKRSKIDTPSTRLFSVLPLA  1680
ARO69865.1   1601  PDVLCRKPGEKGKARFFVGEPGSRIFVAGCNPEKAYYQLEEDSKTKVPSLVSIETPKDERLKRSKIDTPSTRLFSVLPLA  1680
ARO69902.1   1601  PDVLCRKPGEKGKARFFVGEPGSRIFVAGCNPEKAYYQLEEDSKTKVPSLVSIETPKDERLKRSKIDTPSTRLFSVLPLA  1680
ARO69861.1   1601  PDVLCRKPGEKGKARFFVGEPGSRIFVAGCNPEKAYYQLEEDSKTKVPSLVSIETPKDERLKRSKIDTPSTRLFSVLPLA  1680
ARO69850.1   1601  PDVLCRKPGEKGKARFFVGEPGSRIFVAGCNPEKAYYQLEEDSKTKVPSLVSIETPKDERLKRSKIDTPSTRLFSVLPLA  1680
QYU59227.1   1601  PDVLDRKPGERGKARFFVGEPGSRVFVAGCNPEKAYYQLEEDSKTKVPSLVSIETPKDERLKRSKIDTPGTRLFSVLPLA  1680
AFM91094.1   1601  PDVLDRKPGEKGKARFFVGEPGSRVFVAGCNPEKAYYRLEEDSKTKVPSLVSIETPKDERLKRSKIDTPGTRLFSVLPLA  1680
ARO69883.1   1601  PDVLCRKPGEKGKARFFVGEPGSRIFVAGCNPEKAYYQLEEDSKTKVPSLVSIETPKDERLKRSKIDTPSTRLFSVLPLA  1680
ARO69879.1   1601  PDVLCRKPGEKGKARFFVGEPGSRIFVAGCNPEKAYYQLEEDSKTKVPSLVSIETPKDERLKRSKIDTPSTRLFSVLPLA  1680
AND76472.1   1601  PDVLCRKPGEKGKARFFVGEPGSRIFVAGCNPEKAYYQLEEDSKTKVPSLVSIETPKDERLKRSKIDTPSTRLFSVLPLA  1680
ARO69846.1   1601  PDVLCRKPGEKGKARFFVGEPGSRIFVAGCNPEKAYYQLEEDSKTKVPSLVSIETPKDERLKRSKIDTPSTRLFSVLPLA  1680
ARO69870.1   1601  PDVLCRKPGEKGKARFFVGEPGSRIFVAGCNPEKAYYQLEEDSKTKVPSLVSIETPKDERLKRSKIDTPSTRLFSVLPLA  1680
ARO69856.1   1601  PDVLCRKPGEKGKARFFVGEPGSRIFVAGCNPEKAYYQLEEDSKTKVPSLVSIETPKDERLKRSKIDTPSTRLFSVLPLA  1680
ARO69847.1   1601  PDVLDRKPGEKGKARFFVGEPGNRIFVAGCNPEKAYYQLEEDSKTKVPSLVSIETPKDERLKRSKIDTPGTRLFSVLPLA  1680
ARO69875.1   1601  PDVLDRKPGEKGKARFFVGEPGNRIFVAGCNPEKAYYQLEEDSKTKVPSLVSIETPKDERLKRSKIDTPGTRLFSVLPLA  1680
ARO69895.1   1601  PDVLDRKPGEKGKARFFVGEPGNRIFVAGCNPEKAYYQLEEDSKTKVPSLVSIETPKDERLKRSKIDTPGTRLFSVLPLA  1680
ARO69880.1   1601  PDVLDRKPGEKGKARFFVGEPGNRIFVAGCNPEKAYYQLEEDSKTKVPSLVSIETPKDERLKRSKIDTPGTRLFSVLPLA  1680
BCT87061.1   1601  PDVLSRKPGERGKARFFVGEPGSRTFLAGCNPEKAYYQLEEDSKTKVPALVSIETPKDERLKRSKIDTPGTRLFSVLPLA  1680
QYU59225.1   1601  PDVLYRKPGEKGKARFFVGEPGNRVFVADCGPEKAYYQLEEDSKTKVPSLVSIETPKDERLKRSKIDTPGTRLFSVLPLA  1680
APD26306.1   1601  PDVLERKPGEKGKARFFVGEPGNRVFVAGCKPERAYYQLEEDSKTKVPSLVSIETPKDERLKKSKIDTPGTRLFSVLPLA  1680


NP_619689.1  1681  YNLLLRVKFLSFSRLLMKKRGHLPCQVGINPYSREWTDLYHRLGELSDVGYNCDYKAFDGLITEQILSTIADMINAGYRD  1760
AWO68042.1   1681  YNLLLRVKFLSFSRLLMKKRGHLPCQVGINPYSREWTDLYHRLGELSDVGYNCDYKAFDGLITEQILSTIADMINAGYRD  1760
QYU59223.1   1681  YNLLLRVKFLSFSRLLMKKRGHLPCQVGINPYSREWTELYHRLGELSDVGYNCDYKAFDGLITDQILSVIADMINAGYRD  1760
ARO69887.1   1681  YNLLLRVKFLSFSRLLMKKRGHLPCQVGINPYSREWTDLYHRLGELSDTGYNCDYKAFDGLITEQVLGVIADMINAGYRD  1760
ARO69840.1   1681  YNLLLRVKFLSFSRLLMKKRGHLPCQVGINPYSREWTDLYHRLGELSDTGYNCDYKAFDGLITEQVLGVIADMINAGYRD  1760
ARO69892.1   1681  YNLLLRVKFLSFSRLLMKKRGHLPCQVGINPYSREWTDLYHRLGELSDTGYNCDYKAFDGLITEQVLGVIADMINAGYRD  1760
ARO69862.1   1681  YNLLLRVKFLSFSRLLMKKRGHLPCQVGINPYSREWTDLYHRLGELSDTGYNCDYKAFDGLITEQVLGVIADMINAGYRD  1760
ARO69844.1   1681  YNLLLRVKFLSFSRLLMKKRGHLPCQVGINPYSREWTDLYHRLGELSDTGYNCDYKAFDGLITEQVLGVIADMINAGYRD  1760
ARO69896.1   1681  YNLLLRVKFLSFSRLLMKKRGHLPCQVGINPYSREWTDLYHRLGELSDTGYNCDYKAFDGLITEQVLGVIADMINAGYRD  1760
QGP72541.1   1681  YNLLLRVKFLSFSRLLMKKRGHLPCQVGINPYSREWTDLYHRLGELSDIGYNCDYKAFDGLITEQILSTIADMINAGYRD  1760
ARO69901.1   1681  YNLLLRVKFLSFSRLLMKKRGHLPCQVGINPYSREWTDLYHRLGELSDTGYNCDYKAFDGLITEQVLGVIADMINAGYRD  1760
AYA22299.1   1681  YNLLLRVKFLSFSRLLMKKRGHLPCQVGINPYSREWTDLYHRLGELSDIGYNCDYKAFDGLITDQILGTIADMINAGYRD  1760
ARO69890.1   1681  YNLLLRVKFLSFSRLLMKKRGHLPCQVGINPYSREWTDLYHRLGELSDTGYNCDYKAFDGLITEQVLGVIADMINAGYRD  1760
ARO69859.1   1681  YNLLLRVKFLSFSRLLMKKRGHLPCQVGINPYSREWTDLYHRLGELSDTGYNCDYKAFDGLITEQVLGVIADMINAGYRD  1760
ARO69905.1   1681  YNLLLRVKFLSFSRLLMKKRGHLPCQVGINPYSREWTDLYHRLGELSDTGYNCDYKAFDGLITEQVLGVIADMINAGYRD  1760
ARO69871.1   1681  YNLLLRVKFLSFSRLLMKKRGHLPCQVGINPYSREWTDLYHRLGELSDTGYNCDYKAFDGLITEQVLGVIADMINAGYRD  1760
ARO69848.1   1681  YNLLLRVKFLSFSRLLMKKRGHLPCQVGINPYSREWTDLYHRLGELSDTGYNCDYKAFDGLITEQVLGVIADMINAGYRD  1760
ARO69874.1   1681  YNLLLRVKFLSFSRLLMKKRGHLPCQVGINPYSREWTDLYHRLGELSDTGYNCDYKAFDGLITEQVLGVIADMINAGYRD  1760
ARO69882.1   1681  YNLLLRVKFLSFSRLLMKKRGHLPCQVGINPYSREWTDLYHRLGELSDTGYNCDYKAFDGLITEQVLGVIADMINAGYRD  1760
QYU59221.1   1681  YNLLLRVKFLSFSRLLMKKRGHLPCQVGINPYSREWTDLYHRLGELSDVGYNCDYRAFDGLITDQILSTIADMINAGYRD  1760
ARO69842.1   1681  YNLLLRVKFLSFSRLLMKKRGHLPCQVGINPYSREWTDLYHRLGELSDTGYNCDYKAFDGLITEQVLGVIADMINAGYRD  1760
AFV34731.1   1681  YNLLLRVKFLSFSRLLMKKRGHLPCQVGINPYSREWTDLYHRLGELSDIGYNCDYKAFDGLITDQILSTIADMINAGYRD  1760
AND76470.1   1681  YNLLLRVKFLSFSRLLMKKRGHLPCQVGINPYSREWTDLYHRLGELSDTGYNCDYKAFDGLITEQVLGVIADMINAGYRD  1760
ACZ58632.1   1681  YNLLLRVKFLSFSRLLMKKRGHLPCQVGINPYSREWTDLYHRLGELSDIGYNCDYKAFDGLITEQILSTIADMINTGYRD  1760
QBZ78374.1   1681  YNLLLRVKFLSFSRLLMKKRGHLPCQVGINPYSREWTDLYHRLGELSDVGYNCDYRAFDGLITDQILSTIADMINAGYRD  1760
QGP72540.1   1681  YNLLLRVKFLSFSRLLMKKRGHLPCQVGINPYSREWTDLYHRLGELSDIGYNCDYKAFDGLITEQILSTIADMINVGYRD  1760
QBZ78372.1   1681  YNLLLRVKFLSFSRLLMKKRGHLPCQVGINPYSREWTDLYHRLGELSDVGYNCDYRAFDGLITDQILSTIADMINAGYRD  1760
QYU59224.1   1681  YNLLLRVKFLSFSRLLMKKRGHLPCQVGINPYSREWGDLYHRLGELSDVGYNCDYKAFDGLITDQILSTIADMINAGYRD  1760
ARO69877.1   1681  YNLLLRVKFLSFSRLLMKKRGHLPCQVGINPYSREWTDLYHRLGELSDTGYNCDYKAFDGLITEQVLGVIADMINAGYRD  1760
ARO69858.1   1681  YNLLLRVKFLSFSRLLMKKRGHLPCQVGINPYSREWTDLYHRLGELSDTGYNCDYKAFDGLITEQVLGVIADMINAGYRD  1760
ARO69855.1   1681  YNLLLRVKFLSFSRLLMKKRGHLPCQVGINPYSREWTDLYHRLGELSDTGYNCDYKAFDGLITEQVLGVIADMINAGYRD  1760
QGP72542.1   1681  YNLLLRVKFLSFSRLLMKKRGHLPCQVGINPYSREWADLYHRLGELSDIGYNCDYKAFDGLITEQILSTIADMINVGYRD  1760
AYQ93055.1   1681  YNLLLRVKFLSFSRLLMKKRGHLPCQVGINPYSREWADLYHRLGELSDVGYNCDYKAFDGLITDQILSTIADMINAGYRD  1760
AYQ93053.1   1681  YNLLLRVKFLSFSRLLMKKRGHLPCQVGINPYSREWADLYHRLGELSDVGYNCDYKAFDGLITDQILSTIADMINAGYRD  1760
AYQ93051.1   1681  YNLLLRVKFLSFSRLLMKKRGHLPCQVGINPYSREWTDLYHRLGELSDVGYNCDYKAFDGLITEQILSIIADMINAGYRD  1760
AFI71813.1   1681  YNLLLRVKFLSFSRLLMKKRGHLPCQVGINPYSREWTDLYHRLGELSDVGYNCDYRAFDGLITDQILGVIADMINAGYRD  1760
```

```
QGP72539.1   1681  YNLLLRVKFLSFSRLLMKKRGHLPCQVGINPYSREWTDLYHRLGELSDIGYNCDYKAFDGLITEQILSTIADMINVGYRD  1760
QYU59226.1   1681  YNLLLRVKFLSFSRLLMKKRGHLPCQVGINPYSREWTDLYHRLGELSDVGYNCDYRAFDGLITDQILSTIADMINAGYRD  1760
ARO69852.1   1681  YNLLLRVKFLSFSRLLMKKRGHLPCQVGINPYSREWTDLYHRLGELSDTGYNCDYKAFDGLITEQVLGVIADMINAGYRD  1760
QGP72543.1   1681  YNLLLRVKFLSFSRLLMKKRGHLPCQVGINPYSREWTDLYHRLGELSDIGYNCDYKAFDGLITDQILSTIADMINAGYRD  1760
QYU59219.1   1681  YNLLLRVKFLSFSRLLMKKRGHLPCQVGINPYSREWTDLYHRLGELSDVGYNCDYKAFDGLITDQILGVIADMINAGYRD  1760
AGT42200.1   1681  YNLLLRVKFLSFSRLLMKKRGHLPCQVGINPYSREWTDLYHRLGELSDVGYNCDYRAFDGLITDQILGVIADMINAGYRD  1760
ACZ58633.1   1681  YNLLLRVKFLSFSRLLMKKRGHLPCQVGINPYSREWTDLYHRLGELSDVGYNCDYKAFDGLITDQILSVIADMINAGYRD  1760
QBZ78369.1   1681  YNLLLRVKFLSFSRLLMKKRGHLPCQVGINPYSREWNDLYHRLGELSDVGYNCDYKAFDGLITDQILSIIADMINAGYRD  1760
ARO69851.1   1681  YNLILRVKFLSFSRLLMKKRGHLPCQVGINPYSREWTDLYHRLGELSDIGYNCDYKAFDGLITDQILSVIADMINAGYRD  1760
QYU59220.1   1681  YNLLLRVKFLSFSRLLMKKRGHLPCQVGINPYSREWTDLYHRLGELSDIGYNCDYKAFDGLITDQILSVIADMINTGYRD  1760
AFV34755.1   1681  YNLLLRVKFLSFSRLLMKKRGHLPCQVGINPYSREWTDLYHRLGELSDTGYNCDYKAFDGLITEQILSTIADMINVGYRD  1760
QGP72544.1   1681  YNLLLRVKFLSFSRLLMKKRGHLPCQVGINPYSREWTDLYHRLGELSDTGYNCDYKAFDGLITDQILSVIADMINAGYRD  1760
QYA72352.1   1681  YNLLLRVKFLSFSRLLMKKRGHLPCQVGINPYSREWTDLYHRLCELSDTGYNCDYKAFDGLITDQILSVIADMINAGYRD  1760
ARO69854.1   1681  YNLLLRVKFLSFSRLLMKKRGHLPCQVGINPYSREWTDLYHRLGELSDVGYNCDYKAFDGLITDQILSIIADMINAGYRD  1760
ARO69860.1   1681  YNLLLRVKFLSFSRLLMKKRGHLPCQVGINPYSREWTDLYHRLGELSDVGYNCDYKAFDGLITDQILSIIADMINAGYRD  1760
ARO69863.1   1681  YNLLLRVKFLSFSRLLMKKRGHLPCQVGINPYSREWTDLYHRLGELSDVGYNCDYKAFDGLITDQILSIIADMINAGYRD  1760
DAZ85707.1   1681  YNLLLRVKFLSFSRLLMKKRGHLPCQVGINPYSREWTELYLRLGELSDTGYNCDYKAFDGLITDQILGTIADMINAGYRD  1760
AWO67781.1   1681  YNLLLRVKFLSFSRLLMKKRGHLPCQVGINPYSREWTDLYHRLGELSDVGYNCDYKAFDGLITDQVLGTIADMINAGYRD  1760
QBZ78367.1   1681  YNLLLRVKFLSFSRLLMKKRGHLPCQVGINPYSREWTDLYHRLGELSDVGYNCDYKAFDGLITDQILSVIADMINAGYRD  1760
ARO69873.1   1681  YNLLLRVKFLSFSRLLMKKRGHLPCQVGINPYSREWTDLYHRLGELSDVGYNCDYKAFDGLITDQILSIIADMINAGYRD  1760
AND76471.1   1681  YNLLLRVKFLSFSRLLMKKRGHLPCQVGINPYSREWTDLYHRLGELSDVGYNCDYKAFDGLITDQILSIIADMINAGYRD  1760
ARO69849.1   1681  YNLLLRVKFLSFSRLLMKKRGHLPCQVGINPYSREWTDLYHRLGELSDVGYNCDYKAFDGLITDQILSIIADMINAGYRD  1760
ARO69889.1   1681  YNLLLRVKFLSFSRLLMKKRGHLPCQVGINPYSREWTDLYHRLGELSDVGYNCDYKAFDGLITDQILSIIADMINAGYRD  1760
ARO69867.1   1681  YNLLLRVKFLSFSRLLMKKRGHLPCQVGINPYSREWTDLYHRLGELSDVGYNCDYKAFDGLITDQILSIIADMINAGYRD  1760
ARO69904.1   1681  YNLLLRVKFLSFSRLLMKKRGHLPCQVGINPYSREWTDLYHRLGELSDVGYNCDYKAFDGLITDQILSIIADMINAGYRD  1760
ARO69869.1   1681  YNLLLRVKFLSFSRLLMKKRGHLPCQVGINPYSREWTDLYHRLGELSDVGYNCDYKAFDGLITDQILSIIADMINAGYRD  1760
ARO69884.1   1681  YNLLLRVKFLSFSRLLMKKRGHLPCQVGINPYSREWTDLYHRLGELSDVGYNCDYKAFDGLITDQILSIIADMINAGYRD  1760
ARO69878.1   1681  YNLLLRVKFLSFSRLLMKKRGHLPCQVGINPYSREWTDLYHRLGELSDVGYNCDYKAFDGLITDQILSIIADMINAGYRD  1760
ARO69885.1   1681  YNLLLRVKFLSFSRLLMKKRGHLPCQVGINPYSREWTDLYHRLGELSDVGYNCDYKAFDGLITDQILSIIADMINAGYRD  1760
ARO69841.1   1681  YNLLLRVKFLSFSRLLMKKRGHLPCQVGINPYSREWTDLYHRLGELSDVGYNCDYKAFDGLITDQILSIIADMINAGYRD  1760
ARO69891.1   1681  YNLLLRVKFLSFSRLLMKKRGHLPCQVGINPYSREWTDLYHRLGELSDVGYNCDYKAFDGLITDQILSVIADMINAGYRD  1760
ARO69868.1   1681  YNLLLRVKFLSFSRLLMKKRGHLPCQVGINPYSREWTDLYHRLGELSDVGYNCDYKAFDGLITDQILSVIADMINAGYRD  1760
ARO69900.1   1681  YNLLLRVKFLSFSRLLMKKRGHLPCQVGINPYSREWTDLYHRLGELSDVGYNCDYKAFDGLITDQILSIIADMINAGYRD  1760
DAZ85706.1   1681  YNLLLRVKFLSFSRLLMKKRGHLPCQVGINPYSREWTDLYHRLGELSDTGYNCDYKAFDGLITDQILSVIADMINAGYRD  1760
ARO69899.1   1681  YNLLLRVKFLSFSRLLMKKRGHLPCQVGINPYSREWTDLYHRLGELSDVGYNCDYKAFDGLITDQILSVIADMINAGYRD  1760
ARO69898.1   1681  YNLLLRVKFLSFSRLLMKKRGHLPCQVGINPYSREWTDLYHRLGELSDVGYNCDYKAFDGLITDQILSIIADMINAGYRD  1760
AYA22297.1   1681  YNLLLRVKFLSFSRLLMKKRGHLPCQVGINPYSREWTELYHRLGELSDVGYNCDYKAFDGLITEQILSVIADMINAGYRD  1760
QYU59222.1   1681  YNLLLRVKFLSFSRLLMKKRGHLPCQVGINPYSREWTDLYHRLGELSDVGYNCDYKAFDGLITDQILSVIADMINAGYRD  1760
ARO69845.1   1681  YNLLLRVKFLSFSRLLMKKRGHLPCQVGINPYSREWTDLYHRLGELSDVGYNCDYKAFDGLITDQILSIIADMINAGYRD  1760
AND76473.1   1681  YNLLLRVKFLSFSRLLMKKRGHLPCQVGINPYSREWTDLYHRLGELSDVGYNCDYKAFDGLITDQILSVIADMINAGYRD  1760
ARO69857.1   1681  YNLLLRVKFLSFSRLLMKKRGHLPCQVGINPYSREWTDLYHRLGELSDVGYNCDYKAFDGLITDQILSVIADMINAGYRD  1760
DAZ85708.1   1681  YNLLLRVKFLSFSRLLMKKRGHLPCQVGINPYSREWTNLYHRLGDLSDVGYNCDYKAFDGLITDQILSTIADMINAGYRD  1760
ARO69881.1   1681  YNLLLRVKFLSFSRLLMKKRGHLPCQVGINPYSREWTDLYHRLGELSDTGYNCDYKAFDGLITEQILGVIADMINAGYRD  1760
ARO69893.1   1681  YNLLLRVKFLSFSRLLMKKRGHLPCQVGINPYSREWTDLYHRLGELSDTGYNCDYKAFDGLITEQILGVIADMINAGYRD  1760
ARO69897.1   1681  YNLLLRVKFLSFSRLLMKKRGHLPCQVGINPYSREWTDLYHRLGELSDTGYNCDYKAFDGLITEQILGVIADMINAGYRD  1760
ARO69872.1   1681  YNLLLRVKFLSFSRLLMKKRGHLPCQVGINPYSREWTDLYHRLGELSDTGYNCDYKAFDGLITEQILGVIADMINAGYRD  1760
ARO69843.1   1681  YNLLLRVKFLSFSRLLMKKRGHLPCQVGINPYSREWTDLYHRLGELSDTGYNCDYKAFDGLITEQILGVIADMINAGYRD  1760
ARO69886.1   1681  YNLLLRVKFLSFSRLLMKKRGHLPCQVGINPYSREWTDLYHRLGELSDTGYNCDYKAFDGLITEQILGVIADMINAGYRD  1760
ARO69864.1   1681  YNLLLRVKFLSFSRLLMKKRGHLPCQVGINPYSREWTDLYHRLGELSDTGYNCDYKAFDGLITEQILGVIADMINAGYRD  1760
ARO69876.1   1681  YNLLLRVKFLSFSRLLMKKRGHLPCQVGINPYSREWTDLYHRLGELSDTGYNCDYKAFDGLITEQILGVIADMINAGYRD  1760
ARO69888.1   1681  YNLLLRVKFLSFSRLLMKKRGHLPCQVGINPYSREWTDLYHRLGELSDTGYNCDYKAFDGLITEQILGVIADMINAGYRD  1760
ARO69853.1   1681  YNLLLRVKFLSFSRLLMKKRGHLPCQVGINPYSREWTDLYHRLGELSDTGYNCDYKAFDGLITEQILGVIADMINAGYRD  1760
ARO69903.1   1681  YNLLLRVKFLSFSRLLMKKRGHLPCQVGINPYSREWTDLYHRLGELSDTGYNCDYKAFDGLITEQILGVIADMINAGYRD  1760
ARO69865.1   1681  YNLLLRVKFLSFSRLLMKKRGHLPCQVGINPYSREWTDLYHRLGELSDTGYNCDYKAFDGLITEQILGVIADMINAGYRD  1760
ARO69902.1   1681  YNLLLRVKFLSFSRLLMKKRGHLPCQVGINPYSREWTDLYHRLGELSDTGYNCDYKAFDGLITEQILGVIADMINAGYRD  1760
ARO69861.1   1681  YNLLLRVKFLSFSRLLMKKRGHLPCQVGINPYSREWTDLYHRLGELSDTGYNCDYKAFDGLITEQILGVIADMINAGYRD  1760
ARO69850.1   1681  YNLLLRVKFLSFSRLLMKKRGHLPCQVGINPYSREWTDLYHRLGELSDTGYNCDYKAFDGLITEQILGVIADMINAGYRD  1760
QYU59227.1   1681  YNLLLRVKFLSFSRLLMKKRGHLPCQVGINPYSREWTDLYHRLGELSDTGYNCDYKAFDGLITDQILGIIADMINAGYRD  1760
AFM91094.1   1681  YNLLLRVKFLSFSRLLMKKRGHLPCQVGINPYSREWTDLYHRLGELSDTGYNCDYKAFDGLITDQILSVIADMINAGYRD  1760
ARO69883.1   1681  YNLLLRVKFLSFSRLLMKKRGHLPCQVGINPYSREWTDLYHRLGELSDTGYNCDYKAFDGLITEQILGVIADMINAGYRD  1760
ARO69879.1   1681  YNLLLRVKFLSFSRLLMKKRGHLPCQVGINPYSREWTDLYHRLGELSDTGYNCDYKAFDGLITEQILGVIADMINAGYRD  1760
AND76472.1   1681  YNLLLRVKFLSFSRLLMKKRGHLPCQVGINPYSREWTDLYHRLGELSDTGYNCDYKAFDGLITEQILGVIADMINAGYRD  1760
ARO69846.1   1681  YNLLLRVKFLSFSRLLMKKRGHLPCQVGINPYSREWTDLYHRLGELSDTGYNCDYKAFDGLITEQILGVIADMINAGYRD  1760
ARO69870.1   1681  YNLLLRVKFLSFSRLLMKKRGHLPCQVGINPYSREWTDLYHRLGELSDTGYNCDYKAFDGLITEQILGVIADMINAGYRD  1760
ARO69856.1   1681  YNLLLRVKFLSFSRLLMKKRGHLPCQVGINPYSREWTDLYHRLGELSDTGYNCDYKAFDGLITEQILGVIADMINAGYRD  1760
ARO69847.1   1681  YNLLLRVKFLSFSRLLMKKRGHLPCQVGINPYSREWTDLYHRLGELSDVGYNCDYKAFDGLITDQILSIIADMINAGYRD  1760
ARO69875.1   1681  YNLLLRVKFLSFSRLLMKKRGHLPCQVGINPYSREWTDLYHRLGELSDVGYNCDYKAFDGLITDQILSIIADMINAGYRD  1760
ARO69895.1   1681  YNLLLRVKFLSFSRLLMKKRGHLPCQVGINPYSREWTDLYHRLGELSDVGYNCDYKAFDGLITDQILSIIADMINAGYRD  1760
ARO69880.1   1681  YNLLLRVKFLSFSRLLMKKRGHLPCQVGINPYSREWTDLYHRLGELSDVGYNCDYKAFDGLITDQILSIIADMINAGYRD  1760
BCT87061.1   1681  YNLLLRVKFLSFSRLLMKKRGHLPCQVGINPYSREWTDIYHRLGELSDVGYNCDYKAFDGLITVQVLSVIADMINAGYRD  1760
QYU59225.1   1681  YNLLLRVKFLSFSRLLMKKRGHLPCQVGINPYSREWTDIYHRLGELSDVGYNCDYRAFDGLITEQILGVIADMINAGYRD  1760
APD26306.1   1681  YNLLLRVKFLSFSRLLMKKRGHLPCQVGINPYSREWTDIYHRLGELSDVGYNCDYKAFDGLITEQVLSVIADMINAGYRD  1760


NP_619689.1  1761  PVGNRQRKNLLLAICGRLSICGNQVYATEAGIPSGCALTVVLNSIFKELLMRYCFKKIVPPVYKECFDRCVVLITYGDDN  1840
AWO68042.1   1761  PVGNRQRKNLLLAICGRLSICGNQVYATEAGIPSGCALTVVLNSIFNELLMRYCFKKIVPPVYKECFDRCVVLITYGDDN  1840
QYU59223.1   1761  PVGNVQRKNLLLAISGRLSVCGNQVYVTEAGIPSGCALTVVLNSIFNELLMRYCFKKIVPPMYKECFDRCVVLITYGDDN  1840
ARO69887.1   1761  PVGNRQRKNLLMAINGRLSVCGNQVYATEAGIPSGCALTVVLNSIFNELLMRYCFKKIVPPVYKECFDRCVVLITYGDDN  1840
ARO69840.1   1761  PVGNRQRKNLLMAINGRLSVCGNQVYATEAGIPSGCALTVVLNSIFNELLMRYCFKKIVPPVYKECFDRCVVLITYGDDN  1840
ARO69892.1   1761  PVGNRQRKNLLMAINGRLSVCGNQVYATEAGIPSGCALTVVLNSIFNELLMRYCFKKIVPPVYKECFDRCVVLITYGDDN  1840
ARO69862.1   1761  PVGNRQRKNLLMAINGRLSVCGNQVYATEAGIPSGCALTVVLNSIFNELLMRYCFKKIVPPVYKECFDRCVVLITYGDDN  1840
ARO69844.1   1761  PVGNRQRKNLLMAISGRLSVCGNQVYATEAGIPSGCALTVVLNSIFNELLMRYCFKKIVPPVYKECFDRCVVLITYGDDN  1840
ARO69896.1   1761  PVGNRQRKNLLMAINGRLSVCGNQVYATEAGIPSGCALTVVLNSIFNELLMRYCFKKIVPPVYKECFDRCVVLITYGDDN  1840
QGP72541.1   1761  PVGNKQRRNLLLAISGRLSICGNQVYVTEAGIPSGCALTVVLNSIFNELLMRYCFKKIVPPVYKECFDRCVVLITYGDDN  1840
ARO69901.1   1761  PVGNRQRKNLLMAINGRLSICGNQVYATEAGIPSGCALTVVLNSIFNELLMRYCFKKIVPPVYKECFDRCVVLITYGDDN  1840
AYA22299.1   1761  PVGNRQRKNLLLAISGRLSICGNQVYATEAGIPSGCALTVVLNSIFNELLMRYCFKKIVPPVYKECFDRCVVLITYGDDN  1840
ARO69890.1   1761  PVGNRQRKNLLMAINGRLSVCGNQVYATEAGIPSGCALTVVLNSIFNELLMRYCFKKIVPPVYKECFDRCVVLITYGDDN  1840
ARO69859.1   1761  PVGNRQRKNLLMAINGRLSVCGNQVYATEAGIPSGCALTVVLNSIFNELLMRYCFKKIVPPVYKECFDRCVVLITYGDDN  1840
```

```
ARO69905.1   1761  PVGNRQRKNLLMAINGRLSVCGNQVYATEAGIPSGCALTVVLNSIFNELLMRYCFKKIVPPVYKECFDRCVVLITYGDDN  1840
ARO69871.1   1761  PVGNRQRKNLLMAINGRLSVCGNQVYATEAGIPSGCALTVVLNSIFNELLMRYCFKKIVPPVYKECFDRCVVLITYGDDN  1840
ARO69848.1   1761  PVGNRQRKNLLMAINGRLSVCGNQVYATEAGIPSGCALTVVLNSIFNELLMRYCFKKIVPPVYKECFDRCVVLITYGDDN  1840
ARO69874.1   1761  PVGNRQRKNLLMAINGRLSVCGNQVYATEAGIPSGCALTVVLNSIFNELLMRYCFKKIVPPVYKECFDRCVVLITYGDDN  1840
ARO69882.1   1761  PVGNRQRKNLLMAINGRLSVCGNQVYATEAGIPSGCALTVVLNSIFNELLMRYCFKKIVPPVYKECFDRCVVLITYGDDN  1840
QYU59221.1   1761  PVGNRQRKNLLLAISGRLSVCGNQVYVTEAGIPSGCALTVVLNSIFNELLMRYCFKKIVPPVYKECFDRCVVLITYGDDN  1840
ARO69842.1   1761  PVGNRQRKNLLMAINGRLSVCGNQVYATEAGIPSGCALTVVLNSIFNELLMRYCFKKIVPPVYKECFDRCVVLITYGDDN  1840
AFV34731.1   1761  PVGNRQRKNLLLAISGRLSICGNQVYATEAGIPSGCALTVVLNSIFNELLMRYCFKKIVPPVYKECFDRCVVLITYGDDN  1840
AND76470.1   1761  PVGNRQRKNLLMAINGRLSVCGNQVYATEAGIPSGCALTVVLNSIFNELLMRYCFKKIVPPVYKECFDRCVVLITYGDDN  1840
ACZ58632.1   1761  PIGNRQRKNLLLAICGRLSICGNQVYATEAGIPSGCALTVVLNSIFKELLMRYCFKKIVPPMYKECFDRCVVLITYGDDN  1840
QBZ78374.1   1761  PVGNRQRKNLLLAISGRLSICGNQVYVTEAGIPSGCALTVVLNSIFNELLMRYCFKKIVPPMYKECFDRCVVLITYGDDN  1840
QGP72540.1   1761  PVGNRQRKNLLLAICGRLSICGNQVYVTEAGIPSGCALTVVLNSIFNELLMRYCFKKIVPPMYKECFDRCVVLITYGDDN  1840
QBZ78372.1   1761  PVGNRQRKNLLLAISGRLSICGNQVYVTEAGIPSGCALTVVLNSIFNELLMRYCFKKIVPPMYKECFDRCVVLITYGDDN  1840
QYU59224.1   1761  PVGNRQRKNLLLAISGRLSICGNQVYVTEAGIPSGCALTVVLNSIFNELLMRYCFKKIVPPVYKECFDRCVVLITYGDDN  1840
ARO69877.1   1761  PVGNRQRKNLLMAINGRLSVCGNQVYATEAGIPSGCALTVVLNSIFNELLMRYCFKKIVPPVYKECFDRCVVLITYGDDN  1840
ARO69858.1   1761  PVGNRQRKNLLMAINGRLSVCGNQVYATEAGIPSGCALTVVLNSIFNELLMRYCFKKIVPPVYKECFDRCVVLITYGDDN  1840
ARO69855.1   1761  PVGNRQRKNLLMAINGRLSVCGNQVYATEAGIPSGCALTVVLNSIFNELLMRYCFKKIVPPVYKECFDRCVVLITYGDDN  1840
QGP72542.1   1761  PVGNRQRKNLLLAICGRLSICGNQVYVTEAGIPSGCALTVVLNSIFNELLMRYCFKKIVPPMYKECFDRCVVLITYGDDN  1840
AYQ93055.1   1761  PVGNKQRKNLLLAISGRLSICGNQVYVTEAGIPSGCALTVVLNSIFNELLMRYCFKKIVPPVYKECFDRCVVLITYGDDN  1840
AYQ93053.1   1761  PVGNKQRKNLLLAISGRLSICGNQVYVTEAGIPSGCALTVVLNSIFNELLMRYCFKKIVPPVYKECFDRCVVLITYGDDN  1840
AYQ93051.1   1761  PVGNRQRKNLLLAISGRLSICGNQVYATEAGIPSGCALTVVLNSIFNELLMRYCFKKIVPPVYRECFDRCVVLITYGDDN  1840
AFI71813.1   1761  PVGNRQRKNLLLAISGRLSICGNQVYATEAGIPSGCALTVVLNSIFNELLMRYCFKKIVPPVYKECFDRCVVLITYGDDN  1840
QGP72539.1   1761  PVGNRQRKNLLLAICGRLSICGNQVYVTEAGIPSGCALTVVLNSIFNELLMRYCFKKIVPPMYKECFDRCVVLITYGDDN  1840
QYU59226.1   1761  PVGNRQRKNLLLAISGRLSICGNQVYVTEAGIPSGCALTVVLNSIFNELLMRYCFKKIVPPVYKECFDRCVVLITYGDDN  1840
ARO69852.1   1761  PVGNRQRKNLLMAINGRLSVCGNQVYATEAGIPSGCALTVVLNSIFNELLMRYCFKKIVPPVYKECFDRCVVLITYGDDN  1840
QGP72543.1   1761  PVGNRQRKNLLLAISGRLSICGNQVYATEAGIPSGCALTVVLNSIFNELLMRYCFKKIVPPVYKECFDRCVVLITYGDDN  1840
QYU59219.1   1761  PVGNNQRKNLLLAISGRLSICGNQVYATEAGIPSGCALTVVLNSIFNELLMRYCFKKIVPPLYKECFDRCVVLITYGDDN  1840
AGT42200.1   1761  PVGNRQRKNLLLAISGRLSICGNQVYATEAGIPSGCALTVVLNSIFNELLMRYCFKKIVPPVYKECFDRCVVLITYGDDN  1840
ACZ58633.1   1761  PVGNIQRKNLLLAISGRLSVCGNQVYVTEAGIPSGCALTVVLNSIFNELLMIFCFKKIVPPIYKECFDRCVVLITYGDDN  1840
QBZ78369.1   1761  PVGNRQRKNLLLAINGRLSICGNQVYATEAGIPSGCALTVVLNSIFNELLMRYCFKKIVPPVYKECFDRCVVLITYGDDN  1840
ARO69851.1   1761  PVGNRQRKNLLLAISGRLSICGNQVYATEAGIPSGCALTVVLNSIFNELLMRYCFKKIVPPLYKECFDRCVVLITYGDDN  1840
QYU59220.1   1761  PVGNRQRKNLLLAMSGRLSICGNQVYVTEAGIPSGCALTVVLNSIFNELLMRYCFKKIVPPVYKECFDRCVVLVTYGDDN  1840
AFV34755.1   1761  PVGNRQRKNLLLAISGRLSICGNQVYATEAGIPSGCALTVVLNSIFNELLMRYCFKKIVPPVYKECFDRCVVLITYGDDN  1840
QGP72544.1   1761  PVGNRQRKNLLLAISGRLSICGNQVYATEAGIPSGCALTVVLNSIFNELLMRYCFKKIVPPMYKECFDRCVVLITYGDDN  1840
QYA72352.1   1761  PVGNKQRKNLLLAISGRLSICGNQVYATEAGIPSGCALTVVLNSIFNELLMRYCFKKIVPPVYRECFDRCVVLITYGDDN  1840
ARO69854.1   1761  PVGNRQRKNLLLAISGRLSICGSQVYVTEAGIPSGCALTVVLNSIFNELLMRYCFKKIVPPVYKECFDRCVVLVTYGDDN  1840
ARO69860.1   1761  PVGNRQRKNLLLAISGRLSICGSQVYVTEAGIPSGCALTVVLNSIFNELLMRYCFKKIVPPVYKECFDRCVVLVTYGDDN  1840
ARO69863.1   1761  PVGNRQRKNLLLAINGRLSICGNQVYVTEAGIPSGCALTVVLNSIFNELLMRYCFKKIVPPVYKECFDRCVVLVTYGDDN  1840
DAZ85707.1   1761  PVGNKQRKNLLLAISGRLSICGNQVYATEAGIPSGCALTVVLNSIFNELLMRYCFKKIVPPVYKECFDRCVVLITYGDDN  1840
AWO67781.1   1761  PVGNRQRKNLLLAISGRLSICGNQVYATEAGIPSGCALTVVLNSIFNELLMRYCFKKIVPPMYKECFDRCVVLITYGDDN  1840
QBZ78367.1   1761  PVGNIQRKNLLLAISGRLSVCGNQVYVTEAGIPSGCALTVVLNSIFNELLMRYCFKKIVPPIYKECFDRCVVLITYGDDN  1840
ARO69873.1   1761  PVGNRQRKNLLLAISGRLSICGSQVYVTEAGIPSGCALTVVLNSIFNELLMRYCFKKIVPPVYKECFDRCVVLVTYGDDN  1840
AND76471.1   1761  PVGNRQRKNLLLAISGRLSICGSQVYVTEAGIPSGCALTVVLNSIFNELLMRYCFKKIVPPVYKECFDRCVVLVTYGDDN  1840
ARO69849.1   1761  PVGNRQRKNLLLAISGRLSICGSQVYVTEAGIPSGCALTVVLNSIFNELLMRYCFKKIVPPVYKECFDRCVVLVTYGDDN  1840
ARO69889.1   1761  PVGNRQRKNLLLAISGRLSICGSQVYVTEAGIPSGCALTVVLNSIFNELLMRYCFKKIVPPVYKECFDRCVVLVTYGDDN  1840
ARO69867.1   1761  PVGNRQRKNLLLAISGRLSICGSQVYVTEAGIPSGCALTVVLNSIFNELLMRYCFKKIVPPVYKECFDRCVVLVTYGDDN  1840
ARO69904.1   1761  PVGNRQRKNLLLAISGRLSICGSQVYVTEAGIPSGCALTVVLNSIFNELLMRYCFKKIVPPVYKECFDRCVVLVTYGDDN  1840
ARO69869.1   1761  PVGNRQRKNLLLAISGRLSICGSQVYVTEAGIPSGCALTVVLNSIFNELLMRYCFKKIVPPVYKECFDRCVVLVTYGDDN  1840
ARO69884.1   1761  PVGNRQRKNLLLAISGRLSICGSQVYVTEAGIPSGCALTVVLNSIFNELLMRYCFKKIVPPVYKECFDRCVVLVTYGDDN  1840
ARO69878.1   1761  PVGNRQRKNLLLAISGRLSICGSQVYVTEAGIPSGCALTVVLNSIFNELLMRYCFKKIVPPVYKECFDRCVVLVTYGDDN  1840
ARO69885.1   1761  PVGNRQRKNLLLAISGRLSICGSQVYVTEAGIPSGCALTVVLNSIFNELLMRYCFKKIVPPVYKECFDRCVVLVTYGDDN  1840
ARO69841.1   1761  PVGNRQRKNLLLAISGRLSICGSQVYVTEAGIPSGCALTVVLNSIFNELLMRYCFKKIVPPVYKECFDRCVVLVTYGDDN  1840
ARO69891.1   1761  PVGNRQRKNLLLAISGRLSICGSQVYVTEAGIPSGCALTVVLNSIFNELLMRYCFKKIVPPVYKECFDRCVVLVTYGDDN  1840
ARO69868.1   1761  PVGNRQRKNLLLAISGRLSICGSQVYVTEAGIPSGCALTVVLNSIFNELLMRYCFKKIVPPVYKECFDRCVVLVTYGDDN  1840
ARO69900.1   1761  PVGNRQRKNLLLAISGRLSICGSQVYVTEAGIPSGCALTVVLNSIFNELLMRYCFKKIVPPVYKECFDRCVVLVTYGDDN  1840
DAZ85706.1   1761  PVGNRQRKNLLLAISGRLSVCGNQVYVTEAGIPSGCALTVVLNSIFNELLMRYCFKKIVPPVYKECFDRCVVLITYGDDN  1840
ARO69899.1   1761  PVGNRQRKNLLLAISGRLSICGSQVYVTEAGIPSGCALTVVLNSIFNELLMRYCFKKIVPPVYKECFDRCVVLVTYGDDN  1840
ARO69898.1   1761  PVGNRQRKNLLLAISGRLSICGSQVYVTEAGIPSGCALTVVLNSIFNELLMRYCFKKIVPPVYKECFDRCVVLVTYGDDN  1840
AYA22297.1   1761  PVGNMQRKNLLLAISGRLSICGSQVYATEAGIPSGCALTVVLNSIFNELLMRYCFKKIVPPIYKECFDRCVVLITYGDDN  1840
QYU59222.1   1761  PVSNRQRKNLLLAISGRLSICGNQVYVTEAGIPSGCALTVVLNSIFNELLMRYCFKKIVPPVYRECFDRCVVLITYGDDN  1840
ARO69845.1   1761  PVGNRQRKNLLLAISGRLSICGSQVYVTEAGIPSGCALTVVLNSIFNELLMRYCFKKIVPPVYKECFDRCVVLVTYGDDN  1840
AND76473.1   1761  PVGNRQRKNLLLAISGRLSICGSQVYVTEAGIPSGCALTVVLNSIFNELLMRYCFKKIVPPVYKECFDRCVVLVTYGDDN  1840
ARO69857.1   1761  PVGNRQRKNLLLAISGRLSICGSQVYVTEAGIPSGCALTVVLNSIFNELLMRYCFKKIVPPVYKECFDRCVVLVTYGDDN  1840
DAZ85708.1   1761  PVGNKQRKNLLLAISGRLSICGNQVYAVEAGIPSGCALTVVLNSIFNELLMRYCFKKIVPPLYKECFDRCVVLITYGDDN  1840
ARO69881.1   1761  PVGNRQRKNLLMAICGRLSVCGNQVYATEAGIPSGCALTVVLNSIFNELLMRYCFKKIVPPLYKECFDRCVVLITYGDDN  1840
ARO69893.1   1761  PVGNRQRKNLLMAICGRLSVCGNQVYATEAGIPSGCALTVVLNSIFNELLMRYCFKKIVPPLYKECFDRCVVLITYGDDN  1840
ARO69897.1   1761  PVGNRQRKNLLMAICGRLSVCGNQVYATEAGIPSGCALTVVLNSIFNELLMRYCFKKIVPPLYKECFDRCVVLITYGDDN  1840
ARO69872.1   1761  PVGNRQRKNLLMAICGRLSVCGNQVYATEAGIPSGCALTVVLNSIFNELLMRYCFKKIVPPLYKECFDRCVVLITYGDDN  1840
ARO69843.1   1761  PVGNRQRKNLLMAICGRLSVCGNQVYATEAGIPSGCALTVVLNSIFNELLMRYCFKKIVPPLYKECFDRCVVLITYGDDN  1840
ARO69886.1   1761  PVGNRQRKNLLMAICGRLSVCGNQVYATEAGIPSGCALTVVLNSIFNELLMRYCFKKIVPPLYKECFDRCVVLITYGDDN  1840
ARO69864.1   1761  PVGNRQRKNLLMAICGRLSVCGNQVYATEAGIPSGCALTVVLNSIFNELLMRYCFKKIVPPLYKECFDRCVVLITYGDDN  1840
ARO69876.1   1761  PVGNRQRKNLLMAICGRLSVCGNQVYATEAGIPSGCALTVVLNSIFNELLMRYCFKKIVPPLYKECFDRCVVLITYGDDN  1840
ARO69888.1   1761  PVGNRQRKNLLMAICGRLSVCGNQVYATEAGIPSGCALTVVLNSIFNELLMRYCFKKIVPPLYKECFDRCVVLITYGDDN  1840
ARO69853.1   1761  PVGNRQRKNLLMAICGRLSVCGNQVYATEAGIPSGCALTVVLNSIFNELLMRYCFKKIVPPLYKECFDRCVVLITYGDDN  1840
ARO69903.1   1761  PVGNRQRKNLLMAICGRLSVCGNQVYATEAGIPSGCALTVVLNSIFNELLMRYCFKKIVPPLYKECFDRCVVLITYGDDN  1840
ARO69865.1   1761  PVGNRQRKNLLMAICGRLSVCGNQVYATEAGIPSGCALTVVLNSIFNELLMRYCFKKIVPPLYKECFDRCVVLITYGDDN  1840
ARO69902.1   1761  PVGNRQRKNLLMAICGRLSVCGNQVYATEAGIPSGCALTVVLNSIFNELLMRYCFKKIVPPLYKECFDRCVVLITYGDDN  1840
ARO69861.1   1761  PVGNRQRKNLLMAICGRLSVCGNQVYATEAGIPSGCALTVVLNSIFNELLMRYCFKKIVPPLYKECFDRCVVLITYGDDN  1840
ARO69850.1   1761  PVGNRQRKNLLMAICGRLSVCGNQVYATEAGIPSGCALTVVLNSIFNELLMRYCFKKIVPPLYKECFDRCVVLITYGDDN  1840
QYU59227.1   1761  PVGNRQRKNLLLAISGRLSICGNQVYVTEAGIPSGCALTVVLNSIFNELLMRYCFKKIVPPVYKECFDRCVVLITYGDDN  1840
AFM91094.1   1761  PVGNRQRKNLLLAISGRLSVCGNQVYVTEAGIPSGCALTVVLNSVFNELLMRYCFKKIVPPLYKECFDRCVVLITYGDDN  1840
ARO69883.1   1761  PVGNRQRKNLLMAICGRLSVCGNQVYATEAGIPSGCALTVVLNSIFNELLMRYCFKKIVPPLYKECFDRCVVLITYGDDN  1840
ARO69879.1   1761  PVGNRQRKNLLMAICGRLSVCGNQVYATEAGIPSGCALTVVLNSIFNELLMRYCFKKIVPPLYKECFDRCVVLITYGDDN  1840
AND76472.1   1761  PVGNRQRKNLLMAICGRLSVCGNQVYATEAGIPSGCALTVVLNSIFNELLMRYCFKKIVPPLYKECFDRCVVLITYGDDN  1840
ARO69846.1   1761  PVGNRQRKNLLMAICGRLSVCGNQVYATEAGIPSGCALTVVLNSIFNELLMRYCFKKIVPPLYKECFDRCVVLITYGDDN  1840
ARO69870.1   1761  PVGNRQRKNLLMAICGRLSVCGNQVYATEAGIPSGCALTVVLNSIFNELLMRYCFKKIVPPLYKECFDRCVVLITYGDDN  1840
ARO69856.1   1761  PVGNRQRKNLLMAICGRLSVCGNQVYATEAGIPSGCALTVVLNSIFNELLMRYCFKKIVPPLYKECFDRCVVLITYGDDN  1840
ARO69847.1   1761  PVGNRQRKNLLLAINGRLSICGSQVYVTEAGIPSGCALTVVLNSIFNELLVRYCFKKIVPPVYKECFDRCVVLVTYGDDN  1840
```

```
ARO69875.1   1761  PVGNRQRKNLLLAINGRLSICGSQVYVTEAGIPSGCALTVVLNSIFNELLVRYCFKKIVPPVYKECFDRCVVLVTYGDDN  1840
ARO69895.1   1761  PVGNRQRKNLLLAINGRLSICGSQVYVTEAGIPSGCALTVVLNSIFNELLVRYCFKKIVPPVYKECFDRCVVLVTYGDDN  1840
ARO69880.1   1761  PVGNRQRKNLLLAINGRLSICGSQVYVTEAGIPSGCALTVVLNSIFNELLVRYCFKKIVPPVYKECFDRCVVLVTYGDDN  1840
BCT87061.1   1761  PVGNKQRKNLLLAISGRLSICGNQVYATEAGIPSGCALTVVLNSIFNELLMRYCFKKIVPPVYKECFDRCVVLITYGDDN  1840
QYU59225.1   1761  PVGNRQRKNLLLAISGRLSICGNQVYVTEAGIPSGCALTVVLNSIFNELLMRYCFKKIVPPVYKECFDRCVVLVTYGDDN  1840
APD26306.1   1761  PIGNRQRKNLLLAISGRLSICGNQVYVTEAGIPSGCALTVILNSIFNELLMRYCFKKIVPPLYKECFDRCVVLITYGDDN  1840


NP_619689.1  1841  VFTVAQSVMQYFTGDALKMQMAKLGVTITDGKDKSLSTIPARPLLELEFLKRGFVRSSGGMINAPLEKLSIMSSLVYIRS  1920
AWO68042.1   1841  VFTVAQSVMQYFTGDALKMQMAKLGVTITDGKDKSLSTIPARPLLELEFLKRGFVRSSGGMINAPLEKLSIMSSLVYIRS  1920
QYU59223.1   1841  VFTVAQSVMQHFTGDALKLQMAKLGVTITDGKDKSLSTIPARPLLELEFLKRGFVRSTGGLINAPLEKLSIMSSLIHIRS  1920
ARO69887.1   1841  VFTVAQSVMQHFTGDALKLQMAKLGVTITDGKDKSLSTIPARPLLELEFLKRGFVKSSGGLVNAPLEKLSIMSSLIHIRS  1920
ARO69840.1   1841  VFTVAQSVMQHFTGDALKLQMAKLGVTITDGKDKSLSTIPARPLLELEFLKRGFVKSSGGLVNAPLEKLSIMSSLIYIRS  1920
ARO69892.1   1841  VFTVAQSVMQHFTGDALKLQMAKLGVTITDGKDKSLSTIPARPLLELEFLKRGFVKSSGGLVNAPLEKLSIMSSLIYIRS  1920
ARO69862.1   1841  VFTVAQSVMQHFTGDALKLQMAKLGVTITDGKDKSLSTIPARPLLELEFLKRGFVKSSGGLVNAPLEKLSIMSSLIHIRS  1920
ARO69844.1   1841  VFTVAQSVMQHFTGDALKLQMAKLGVTITDGKDKSLSTIPARPLLELEFLKRGFVKSSGGLVNAPLEKLSIMSSLIHIRS  1920
ARO69896.1   1841  VFTVAQSVMQHFTGDALKLQMAKLGVTITDGKDKSLSTIPARPLLELEFLKRGFVKSSGGLVNAPLEKLSIMSSLIHIRS  1920
QGP72541.1   1841  VFTVAQSVMQYFTGDALKLQMSKLGVTITDGKDKSLSTIPARPLLELEFLKRGFVKSSGGLINAPLEKLSIMSSLIHIRS  1920
ARO69901.1   1841  VFTVAQSVMQHFTGDALKLQMAKLGVTITDGKDKSLSTIPARPLLELEFLKRGFVKSSGGLVNAPLEKLSIMSSLIHIRS  1920
AYA22299.1   1841  VFTVAQSVMQHFTGDALKLQMAELGVTITDGKDKSLSSIPARPLLELEFLKRGFGKSSGGLINAPLEKLSIMSSLVYIRS  1920
ARO69890.1   1841  VFTVAQSVMQHFTGDALKLQMAKLGVTITDGKDKSLSTIPARPLLELEFLKRGFVKSSGGLVNAPLEKLSIMSSLIYIRS  1920
ARO69859.1   1841  VFTVAQSVMQHFTGDALKLQMAKLGVTITDGKDKSLSTIPARPLLELEFLKRGFVKSSGGLVNAPLEKLSIMSSLIYIRS  1920
ARO69905.1   1841  VFTVAQSVMQHFTGDALKLQMAKLGVTITDGKDKSLSTIPARPLLELEFLKRGFVKSSGGLVNAPLEKLSIMSSLIHIRS  1920
ARO69871.1   1841  VFTVAQSVMQHFTGDALKLQMAKLGVTITDGKDKSLSTIPARPLLELEFLKRGFVKSSGGLVNAPLEKLSIMSSLIYIRS  1920
ARO69848.1   1841  VFTVAQSVMQHFTGDALKLQMAKLGVTITDGKDKSLSTIPARPLLELEFLKRGFVKSSGGLVNAPLEKLSIMSSLIHIRS  1920
ARO69874.1   1841  VFTVAQSVMQHFTGDALKLQMAKLGVTITDGKDKSLSTIPARPLLELEFLKRGFVKSSGGLVNAPLEKLSIMSSLIYIRS  1920
ARO69882.1   1841  VFTVAQSVMQHFTGDALKLQMAKLGVTITDGKDKSLSTIPARPLLELEFLKRGFVKSSGGLVNAPLEKLSIMSSLIHIRS  1920
QYU59221.1   1841  VFTVAQSVMQYFTGDALKLQMAKLGVTITDGKDKSLSTIPARPLLELEFLKRGFKKSMGGLINAPLEKLSIMSSLIYIRS  1920
ARO69842.1   1841  VFTVAQSVMQHFTGDALKLQMAKLGVTITDGKDKSLSTIPARPLLELEFLKRGFVKSSGGLVNAPLEKLSIMSSLIHIRS  1920
AFV34731.1   1841  VFTVAQSVMQHFTGDALKLQMAKLGVTITDGKDKSLSTIPARPLLELEFLKRGFVRSSGGMINAPLEKLSIMSSLIHIRS  1920
AND76470.1   1841  VFTVAQSVMQHFTGDALKLQMAKLGVTITDGKDKSLSTIPARPLLELEFLKRGFVKSSGGLVNAPLEKLSIMSSLIHIRS  1920
ACZ58632.1   1841  VFTVAQSVMQHFTGDALKLQMAKIGVTITDGKDKSLSTIPARPLLELEFLKRGFVRSSGGMINAPLEKLSIMSSLIHIRS  1920
QBZ78374.1   1841  VFTVAQSVMQHFTGDALKLQMARLGVTITDGKDKSLSTIPARPLLELEFLKRGFARSSGGLINAPLEKLSIMSSLIHIRS  1920
QGP72540.1   1841  VFTVAQSVMQHFTGDALKMQMAKLGVTITDGKDKSLSTIPARPLLELEFLKRGFVRSSGGLINAPLEKLSIMSSLIHIRS  1920
QBZ78372.1   1841  VFTVAQSVMQHFTGDALKLQMARLGVTITDGKDKSLSTIPARPLLELEFLKRGFARSSGGLINAPLEKLSIMSSLIHIRS  1920
QYU59224.1   1841  VFTVAQSVMQYFTGDALKLQMAKLGVTITDGKDKSLSTIPARPLLELEFLKRGFKKSTGGLVNAPLEKLSIMSSLIHIRS  1920
ARO69877.1   1841  VFTVAQSVMQHFTGDALKLQMAKLGVTITDGKDKSLSTIPARPLLELEFLKRGFVKSSGGLVNAPLEKLSIMSSLIHIRS  1920
ARO69858.1   1841  VFTVAQSVMQHFTGDALKLQMAKLGVTITDGKDKSLSTIPARPLLELEFLKRGFVKSSGGLVNAPLEKLSIMSSLIHIRS  1920
ARO69855.1   1841  VFTVAQSVMQHFTGDALKLQMAKLGVTITDGKDKSLSTIPARPLLELEFLKRGFVKSSGGLVNAPLEKLSIMSSLIYIRS  1920
QGP72542.1   1841  VFTVAQSVMQHFTGDALKMQMAKLGVTITDGKDKSLSTIPARPLLELEFLKRGFVRSSGGLINAPLEKLSIMSSLIHIRS  1920
AYQ93055.1   1841  VFTVAQSVMQHFTGDALKLQMAKLGVTITDGKDKSLPTIPARPLLELEFLKRGFIKGSGGLISAPLEKLSIMSSLIYIRS  1920
AYQ93053.1   1841  VFTVAQSVMQHFTGDALKLQMAKLGVTITDGKDKSLPTIPARPLLELEFLKRGFIKGSGGLISAPLEKLSIMSSLIYIRS  1920
AYQ93051.1   1841  VFTVAQSVMQYFTGDALKSQMAKLGVTITDGKDKSLSTIPARPLLELEFLKRGFVKSSGGLINAPLEKLSIMSSLIYIRS  1920
AFI71813.1   1841  VFTVAQSVMQHFTGDALKLQMAKLGVTITDGKDKSLSSIPARPLLELEFLKRGFVRSSGGLINAPLEKLSIMSSLVYIRS  1920
QGP72539.1   1841  VFTVAQSVMQHFTGDALKMQMAKLGVTITDGKDKSLSTIPARPLLELEFLKRGFVRSSGGLINAPLEKLSIMSSLIHIRS  1920
QYU59226.1   1841  VFTVAQSVMQHFTGDALKLQMAKLGVTITDGKDKSLSTIPARPLLELEFLKRGFVKSMGGLINAPLEKLSIMSSLIYIRS  1920
ARO69852.1   1841  VFTVAQSVMQHFTGDALKLQMAKLGVTITDGKDKSLSTIPARPLLELEFLKRGFVKSSGGLVNAPLEKLSIMSSLIHIRS  1920
QGP72543.1   1841  VFTVAQSVMQHFTGDALKLQMAKLGVTITDGKDKSLSTIPARPLLELEFLKRGFAKSSGGLINAPLDKLSIMSSLIHIRS  1920
QYU59219.1   1841  VFTVAQSVMQYFTGDALKLQMAKLGVTITDGKDKSLSTIPARPLLELEFLKRGFTRSSGGMINAPLEKLSIMSSLVYIRS  1920
AGT42200.1   1841  VFTVAQSVMQHFTGDALKLQMAKLGVTITDGKDKSLSSIPARPLLELEFLKRGFVRSSGGLINAPLEKLSIMSSLVYIRS  1920
ACZ58633.1   1841  VFTVAQSIMQHFTGDALKSHMAKLGVTITDGKDKSLSTIPARPLLELEFLKRGFMKSVGGLINAPLEKLSIMSSLIHIRS  1920
QBZ78369.1   1841  VFTVAQSVMQYFTGDALKLQMAKLGVTITDGKDKSLSTIPARPLLELEFLKRGFKKSMGGLMNAPLEKLSIMSSLIYIRS  1920
ARO69851.1   1841  VFTVAQSVMQYFTGDALKMQMAKLGVTITDGKDKSLSTIPARPLLELEFLKRGFARSTGGLISAPLEKLSIMSSLIYIRS  1920
QYU59220.1   1841  VFTVAQSVMQYFTGDALKLQMAKLGVTITDGKDKSLSTIPARPLQELEFLKRGLVKSSGGKINAPLEKLSIMSSLIYIRS  1920
AFV34755.1   1841  VFTVAQSVMQYFTGDALKLQMAKLGVTITDGKDKSLSTIPARPLLELEFLKRGFMRSTGGLINAPLEKLSIMSSLIHIRS  1920
QGP72544.1   1841  VFTVAQSVMQHFTGDALKLQMAKLGVTITDGKDKSLSTIPARPLLELEFLKRGFIKSSGGTIGAPLEKLSIMSSLIYIRS  1920
QYA72352.1   1841  VFTVAQSVMQYFTGDALKLQMAKLGVTITDGKDKSLSTIPARPLLELEFLKRGFVKSSGGTLNAPLEKLSIMSSLIYIRS  1920
ARO69854.1   1841  VFTVAQSVMQYFTGDALKSQMAKLGVTITDGKDKSLSTIPARPLLELEFLKRGFAKGSGGMINAPLEKLSILSSLIYIRS  1920
ARO69860.1   1841  VFTVAQSVMQYFTGDALKSQMAKLGVTITDGKDKSLSTIPARPLLELEFLKRGFAKGSGGMINAPLEKLSILSSLIYIRS  1920
ARO69863.1   1841  VFTVAQSVMQYFTGDALKSQMAKLGVTITDGKDKSLSTIPARPLLELEFLKRGFAKGSGGMINAPLEKLSILSSLIYIRS  1920
DAZ85707.1   1841  VFTVAQSVMQHFTGDALKLQMAKLGVTITDGKDKSLSTIPARPLLELEFLKRGFVKSSGGQINAPLEKLSIMSSLIYIRS  1920
AWO67781.1   1841  VFTVAQSVMQHFTGDALKLQMAKLGVTITDGKDKSLPTIPARPLLELEFLKRGFVRSSGGMINAPLEKLSIISSLIYIRS  1920
QBZ78367.1   1841  VFTVAQSIMQHFTGDALKLHMAKLGVTITDGKDKSLSTIPARPLLELEFLKRGFMKSVGGLMNAPLEKLSIMSSLIHIRS  1920
ARO69873.1   1841  VFTVAQSVMQYFTGDALKSQMAKLGVTITDGKDKSLSTIPARPLLELEFLKRGFAKGSGGMINAPLEKLSILSSLIYIRS  1920
AND76471.1   1841  VFTVAQSVMQYFTGDALKSQMAKLGVTITDGKDKSLSTIPARPLLELEFLKRGFAKGSGGMINAPLEKLSILSSLIYIRS  1920
ARO69849.1   1841  VFTVAQSVMQYFTGDALKSQMAKLGVTITDGKDKSLSTIPARPLLELEFLKRGFAKGSGGMINAPLEKLSILSSLIYIRS  1920
ARO69889.1   1841  VFTVAQSVMQYFTGDALKSQMAKLGVTITDGKDKSLSTIPARPLLELEFLKRGFAKGSGGMINAPLEKLSILSSLIYIRS  1920
ARO69867.1   1841  VFTVAQSVMQYFTGDALKSQMAKLGVTITDGKDKSLSTIPARPLLELEFLKRGFAKGSGGMINAPLEKLSILSSLIYIRS  1920
ARO69904.1   1841  VFTVAQSVMQYFTGDALKSQMAKLGVTITDGKDKSLSTIPARPLLELEFLKRGFAKGSGGMINAPLEKLSILSSLIYIRS  1920
ARO69869.1   1841  VFTVAQSVMQYFTGDALKSQMAKLGVTITDGKDKSLSTIPARPLLELEFLKRGFAKGSGGMINAPLEKLSILSSLIYIRS  1920
ARO69884.1   1841  VFTVAQSVMQYFTGDALKSQMAKLGVTITDGKDKSLSTIPARPLLELEFLKRGFAKGSGGMINAPLEKLSILSSLIYIRS  1920
ARO69878.1   1841  VFTVAQSVMQYFTGDALKLQMAKLGVTITDGKDKSLSTIPARPLLELEFLKRGFAKGSGGMINAPLEKLSILSSLIYIRS  1920
ARO69885.1   1841  VFTVAQSVMQYFTGDALKSQMAKLGVTITDGKDKSLSTIPARPLLELEFLKRGFAKGSGGMINAPLEKLSILSSLIYIRS  1920
ARO69841.1   1841  VFTVAQSVMQYFTGDALKSQMAKLGVTITDGKDKSLSTIPARPLLELEFLKRGFAKGSGGMINAPLEKLSILSSLIYIRS  1920
ARO69891.1   1841  VFTVAQSVMQYFTGDALKSQMAKLGVTITDGKDKSLSTIPARPLLELEFLKRGFAKGSGGMINAPLEKLSILSSLIYIRS  1920
ARO69868.1   1841  VFTVAQSVMQYFTGDALKSQMAKLGVTITDGKDKSLSTIPARPLLELEFLKRGFAKGSGGMINAPLEKLSILSSLIYIRS  1920
ARO69900.1   1841  VFTVAQSVMQYFTGDALKSQMAKLGVTITDGKDKSLSTIPARPLLELEFLKRGFAKGSGGMINAPLEKLSILSSLIYIRS  1920
DAZ85706.1   1841  VFTVAQSVMQYFTGDALKLQMAKLGVTITDGKDKSLSTIPARPLLELEFLKRGFVKSSGGQINAPLEKLSIMSSLIYIRS  1920
ARO69899.1   1841  VFTVAQSVMQYFTGDALKSQMAKLGVTITDGKDKSLSTIPARPLLELEFLKRGFAKGSGGMINAPLEKLSILSSLIYIRS  1920
ARO69898.1   1841  VFTVAQSVMQYFTGDALKSQMAKLGVTITDGKDKSLSTIPARPLLELEFLKRGFAKGSGGMINAPLEKLSILSSLIYIRS  1920
AYA22297.1   1841  VFTVAQSVMQHFTGDALKLQMAKLGVTITDGKDKSLSTIPARPLLELEFLKRGFVKSSGGLINAPLEKLSIMSSLIYIRS  1920
QYU59222.1   1841  VFTVAQSVMQHFTGEALKLQMARLGVTITDGKDKSLPTIPARPLLELEFLKRGFRRSTGGRINAPLEKLSIMSSLIHIRS  1920
ARO69845.1   1841  VFTVAQSVMQYFTGDALKSQMAKLGVTITDGKDKSLSTIPARPLLELEFLKRGFAKGSGGMINAPLEKLSILSSLIYIRS  1920
AND76473.1   1841  VFTVAQSVMQYFTGDALKSQMAKLGVTITDGKDKSLSTIPARPLLELEFLKRGFAKGSGGMINAPLEKLSIMSSLIYIRS  1920
ARO69857.1   1841  VFTVAQSVMQYFTGDALKSQMAKLGVTITDGKDKSLSTIPARPLLELEFLKRGFAKGSGGMINAPLEKLSILSSLIYIRS  1920
DAZ85708.1   1841  IFTVAQSVMQHFTGDALKLQMAKLGVTITDGKDKSLSTIPARPLLELEFLKRGFVKSSGGQINGPLEKLSIMSSLVYIRS  1920
ARO69881.1   1841  VFTVAQSVMQYFTGDALKMQMAKLGVTITDGKDKSLSTIPARPLLELEFLKRGFVRSTGGLISAPLEKLSIMSSLIYIRS  1920
ARO69893.1   1841  VFTVAQSVMQYFTGDALKMQMAKLGVTITDGKDKSLSTIPARPLLELEFLKRGFVRSTGGLINAPLEKLSIMSSLIYIRS  1920
```

```
ARO69897.1   1841  VFTVAQSVMQYFTGDALKMQMAKLGVTITDGKDKSLSTIPARPLLELEFLKRGFVRSTGGLINAPLEKLSIMSSLIYIRS  1920
ARO69872.1   1841  VFTVAQSVMQYFTGDALKMQMAKLGVTITDGKDKSLSTIPARPLLELEFLKRGFVRSTGGLISAPLEKLSIMSSLIYIRS  1920
ARO69843.1   1841  VFTVAQSVMQYFTGDALKMQMAKLGVTITDGKDKSLSTIPARPLLELEFLKRGFVRSTGGLISAPLEKLSIMSSLIYIRS  1920
ARO69886.1   1841  VFTVAQSVMQYFTGDALKMQMAKLGVTITDGKDKSLSTIPARPLLELEFLKRGFVRSTGGLISAPLEKLSIMSSLIYIRS  1920
ARO69864.1   1841  VFTVAQSVMQYFTGDALKMQMAKLGVTITDGKDKSLSTIPARPLLELEFLKRGFVRSTGGLISAPLEKLSIMSSLIYIRS  1920
ARO69876.1   1841  VFTVAQSVMQYFTGDALKMQMAKLGVTITDGKDKSLSTIPARPLLELEFLKRGFVRSTGGLISAPLEKLSIMSSLIYIRS  1920
ARO69888.1   1841  VFTVAQSVMQYFTGDALKMQMAKLGVTITDGKDKSLSTIPARPLLELEFLKRGFVRSTGGLISAPLEKLSIMSSLIYIRS  1920
ARO69853.1   1841  VFTVAQSVMQYFTGDALKMQMAKLGVTITDGKDKSLSTIPARPLLELEFLKRGFVRSTGGLISAPLEKLSIMSSLIYIRS  1920
ARO69903.1   1841  VFTVAQSVMQYFTGDALKMQMAKLGVTITDGKDKSLSTIPARPLLELEFLKRGFVRSTGGLISAPLEKLSIMSSLIYIRS  1920
ARO69865.1   1841  VFTVAQSVMQYFTGDALKMQMAKLGVTITDGKDKSLSTIPARPLLELEFLKRGFVRSTGGLISAPLEKLSIMSSLIYIRS  1920
ARO69902.1   1841  VFTVAQSVMQYFTGDALKMQMAKLGVTITDGKDKSLSTIPARPLLELEFLKRGFVRSTGGLISAPLEKLSIMSSLIYIRS  1920
ARO69861.1   1841  VFTVAQSVMQYFTGDALKMQMAKLGVTITDGKDKSLSTIPARPLLELEFLKRGFVRSTGGLISAPLEKLSIMSSLIYIRS  1920
ARO69850.1   1841  VFTVAQSVMQYFTGDALKMQMAKLGVTITDGKDKSLSTIPARPLLELEFLKRGFVRSTGGLISAPLEKLSIMSSLIYIRS  1920
QYU59227.1   1841  VFTVAQSVMQYFTGDALKLQMAKLGVTITDGKDKSLSTIPARPLLELEFLKRGFVKSSGGMINAALEKLSIMSSLIYIRS  1920
AFM91094.1   1841  VFTVAQSVMQYFTGDALKLQMAKLGVTITDGKDKSLSTIPARPLLELEFLKRGFVRSSGGLISAPLDKLSIMSSLVYIRS  1920
ARO69883.1   1841  VFTVAQSVMQYFTGDALKMQMAKLGVTITDGKDKSLSTIPARPLLELEFLKRGFVRSTGGLISAPLEKLSIMSSLIYIRS  1920
ARO69879.1   1841  VFTVAQSVMQYFTGDALKMQMAKLGVTITDGKDKSLSTIPARPLLELEFLKRGFVRSTGGLISAPLEKLSIMSSLIYIRS  1920
AND76472.1   1841  VFTVAQSVMQYFTGDALKMQMAKLGVTITDGKDKSLSTIPARPLLELEFLKRGFVRSTGGLISAPLEKLSIMSSLIYIRS  1920
ARO69846.1   1841  VFTVAQSVMQYFTGDALKMQMAKLGVTITDGKDKSLSTIPARPLLELEFLKRGFVRSTGGLISAPLEKLSIMSSLIYIRS  1920
ARO69870.1   1841  VFTVAQSVMQYFTGDALKMQMAKLGVTITDGKDKSLSTIPARPLLELEFLKRGFVRSIGGLISAPLEKLSIMSSLIYIRS  1920
ARO69856.1   1841  VFTVAQSVMQYFTGDALKLQMAKLGVTITDGKDKSLSTIPARPLLELEFLKRGFVRSTGGLISAPLEKLSIMSSLIYIRS  1920
ARO69847.1   1841  IFTVAQSVMQYFTGDALKSQMAKLGVTITDGKDKSLSTIPARPLLELEFLKRGFAKGSGGMINAPLEKLSIMSSLIYIRS  1920
ARO69875.1   1841  IFTVAQSVMQYFTGDALKSQMAKLGVTITDGKDKSLSTIPARPLLELEFLKRGFAKGSGGMINAPLEKLSIMSSLIYIRS  1920
ARO69895.1   1841  IFTVAQSVMQYFTGDALKSQMAKLGVTITDGKDKSLSTIPARPLLELEFLKRGFAKGSGGMINAPLEKLSIMSSLIYIRS  1920
ARO69880.1   1841  IFTVAQSVMQYFTGDALKSQMAKLGVTITDGKDKSLSTIPAGPLLELEFLKRGFAKGSGGMINAPLEKLSIMSSLIYIRS  1920
BCT87061.1   1841  VFTVAQSVAAHFTGDALKGQMAKLGVTITDGKDKSLSTIPARPLLEMEFLKRGFSKGSGGLIHAPLEKLSIMSSLVYIRS  1920
QYU59225.1   1841  VFTVAQSVMEHFTGEALKLQMAKLGVTITDGKDKSLSTIPARPLLELEFLKRGFMKGPGGHIYSPLEKLSIMSSLIYIRS  1920
APD26306.1   1841  VFTVAQSVMGYFTGDALKRQMAMLGVTITDGKDKSLSTIPARPLLELEFLKRGFYKGLGGQIYAPLEKLSIMSSLVYIRS  1920


NP_619689.1  1921  DGSDMLQKLLDNVNTALVELYLHGDRTYFESVRAFYFEKLPPGAYKELTTWFQAESFHECQKSGESGYKPQGLIEISHGA  2000
AWO68042.1   1921  DGSDMLQKLLDNVNTALVELYLHGDRTYFESVRAFYFEKLPPGAYKELTTWFQAESFHECQKSGESGYKPQGLIEISHGA  2000
QYU59223.1   1921  DGSDMLQKLLDNVNTALVELYLHGDRAYFESVRAFYMEKLPPGAYKELTTWYQAESFHECQKSGESGYKPQGLIEISHDT  2000
ARO69887.1   1921  DGSDMLQKLLDNVNTALVELYLHGDRTYFESVRTFYMEKLPPGAYKELMTWFQAESFHECQKSGESGYKPQGLIEVSHGT  2000
ARO69840.1   1921  DGSDMLQKLLDNVNTALVELYLHGDRTYFESVRTFYTEKLPPGAYKELMTWFQAESFHECQKSGESGYKPQGLIEVSHGT  2000
ARO69892.1   1921  DGSDMLQKLLDNVNTALVELYLHGDRTYFESVRTFYMEKLPPGAYKELMTWFQAESFHECQKSGESGYKPQGLIEVSHGT  2000
ARO69862.1   1921  DGSDMLQKLLDNVNTALVELYLHGDRTYFESVRTFYTEKLPPGAYKELMTWFQAESFHECQKSGESGYKPQGLIEVSHGT  2000
ARO69844.1   1921  DGSDMLQKLLDNVNTALVELYLHGDRTYFESVRTFYTEKLPPGAYKELMTWFQAESFHECQKSGESGYKPQGLIEVSHGT  2000
ARO69896.1   1921  DGSDMLQKLLDNVNTALVELYLHGDRTYFESVRTFYTEKLPPGAYKELMTWFQAESFHECQKSGESGYKPQGLIEVSHGT  2000
QGP72541.1   1921  DGSDMLQKLLDNVNTALVELYLHGDRTYFESVRAFYTEKLPPGSYRELTTWFQAESFHECQKSGESGYKPQGLIEISHGA  2000
ARO69901.1   1921  DGSDMLQKLLDNVNTALVELYLHGDRTYFESVRTFYTEKLPPGAYKELMTWFQAESFHECQKSGESGYKPQGLIEVSHGT  2000
AYA22299.1   1921  DGSDMLQKLLDNVNTALVELYLHGDRAYFESVRAFYMEKLPPGAYKELTTWFQAESFHECQKCGESGYKPQGLIEVSHGT  2000
ARO69890.1   1921  DGSDMLQKLLDNVNTALVELYLHGDRTYFESVRTFYTEKLPPGAYKELMTWFQAESFHECQKSGESGYKPQGLIEVSHGT  2000
ARO69859.1   1921  DGSDMLQKLLDNVNTALVELYLHGDRTYFESVRTFYTEKLPPGAYKELMTWFQAESFHECQKSGESGYKPQGLIEVSHGT  2000
ARO69905.1   1921  DGSDMLQKLLDNVNTALVELYLHGDRTYFESVRTFYTEKLPPGAYKELMTWFQAESFHECQKSGESGYKPQGLIEVSHGT  2000
ARO69871.1   1921  DGSDMLQKLLDNVNTALVELYLHGDRTYFESVRTFYMEKLPPGAYKELMTWFQAESFHECQKSGESGYKPQGLIEVSHGT  2000
ARO69848.1   1921  DGSDMLQKLLDNVNTALVELYLHGDRTYFESVRTFYTEKLPPGAYKELMTWFQAESFHECQKSGESGYKPQGLIEVSHGT  2000
ARO69874.1   1921  DGSDMLQKLLDNVNTALVELYLHGDRTYFESVRTFYTEKLPPGAYKELMTWFQAESFHECQKSGESGYKPQGLIEVSHGT  2000
ARO69882.1   1921  DGSDMLQKLLDNVNTALVELYLHGDRTYFESVRTFYMEKLPPGAYKELMTWFQAESFHECQKSGESGYKPQGLIEVSHGT  2000
QYU59221.1   1921  DGSDMLQKLLDNVNTALVELYLHGDRAYFESVRAFYMEKLPPGAYKELTTWYQAESFHECQKSGESGYKPQGLIEISHGT  2000
ARO69842.1   1921  DGSDMLQKLLDNVNTALVELYLHGDRTYFESVRTFYTEKLPPGAYKELMTWFQAESFHECQKSGESGYKPQGLIEVSHGT  2000
AFV34731.1   1921  DGSDMLQKLLDNVNTALVELYLHGDRAYFESVRAFYMEKLPPGAYKELTTWFQAESFHECQKSGESGYKPQGLIEISHGT  2000
AND76470.1   1921  DGSDMLQKLLDNVNTALVELYLHGDRTYFESVRTFYTEKLPPGAYKELMTWFQAESFHECQKSGESGYKPQGLIEVSHGT  2000
ACZ58632.1   1921  DGSDMLQKLLDNVNTALVELYLHGDRAYFESVRAFYMEKLPPGAYKELTTWFQAESFHECQNPRESGYKPQGLIEKSHGA  2000
QBZ78374.1   1921  DGSDMLQKLLDNVNTALVELYLHGDRAYFESVRAFYVEKLPPGAYKELTTWFQAESFHECQKSGESGYKPHGLIEISHGT  2000
QGP72540.1   1921  DGSDMLQKLLDNVNTALVELYLHGDRAYFESVRAFYAEKLPPGAYKELTTWFQAESFHECQKSGESGYKPQGLIEISHGA  2000
QBZ78372.1   1921  DGSDMLQKLLDNVNTALVELYLHGDRAYFESVRAFYVEKLPPGAYKELTTWFQAESFHECQKSGESGYKPHGLIEISHGT  2000
QYU59224.1   1921  DGSDMLQKLLDNVNTALVELYLHGDEAYFESVRAFYMEKLPPGAYKELTTWYQAESFHEYQKSGESGYKPQGLIEISHGT  2000
ARO69877.1   1921  DGSDMLQKLLDNVNTALVELYLHGDRTYFESVRTFYTEKLPPGAYKELMTWFQAESFHECQKSGESGYKPQGLIEVSHGT  2000
ARO69858.1   1921  DGSDMLQKLLDNVNTALVELYLHGDRTYFESVRTFYTEKLPPGAYKELMTWFQAESFHECQKSGESGYKPQGLIEVSHGT  2000
ARO69855.1   1921  DGSDMLQKLLDNVNTALVELYLHGDRTYFESVRTFYKEKLPPGAYKELMTWFQAESFHECQKSGESGYKPQGLIEVSHGT  2000
QGP72542.1   1921  DGSDMLQKLLDNVNTALVELYLHGDRAYFESVRAFYTEKLPPGAYKELTTWFQAESFHECQKSGESGYKPQGLIEISHGA  2000
AYQ93055.1   1921  DGSDMLQKLLDNVNTALVELFLHGDRTYFESVRAFYMEKLPPGAYKELTTWFQAESFHECQKSGESGYKPQGLIEISHGA  2000
AYQ93053.1   1921  DGSDMLQKLLDNVNTALVELFLHGDRTYFESVRAFYMEKLPPGAYKELTTWFQAESFHECQKSGESGYKPQGLIEISHGA  2000
AYQ93051.1   1921  DGSDMLQKLLDNVNTALVELYLHGDRTHFESVRAFYMEKLPPGAYKELTTWFQAESFHECQKSGESGYKPQGLIEISHGA  2000
AFI71813.1   1921  DGSDMLQKLLDNVNTALVELYLHGDRAYFESVRTFYMEKLPPGAYKELTTWFQAETFHECQKSGESGYKPQGLIEVSHGA  2000
QGP72539.1   1921  DGSDMLQKLLDNVNTALVELYLHGDRAYFESVRAFYAEKLPPGAYKELTTWFQAESFHECQKSGESGYKPQGLIEISHGA  2000
QYU59226.1   1921  DGSDMLQKLLDNVNTALVEIFLHGDRAYFESVRAFYLEKLPSGAYKELTTWYQAESFHECQKSGESGYKPQGLIEISHGT  2000
ARO69852.1   1921  DGSDMLQKLLDNVNTALVELYLHGDRTYFESVRTFYTEKLPPGAYKELMTWFQAESFHECQKSGESGYKPQGLIEVSHGT  2000
QGP72543.1   1921  DGSDMLQKLLDNVNTALVELYLHGDRTYFESVRAFYMEKLPPGAYKELTTWYQAESFHECQKSGESGYKPQGLIEISHGA  2000
QYU59219.1   1921  DGSDMLQKLLDNVNTALVELYLHDDRAYFESVRAFYMDRLPPGAYKELTTWYQAETFHECQKSGESSYKPQGLIEISHGA  2000
AGT42200.1   1921  DGSDMLQKLLDNVNTALVELYLHGDRAYFESVRTFYMEKLPPGAYKELTTWFQAETFHECQKSGESGYKPQGLIEVSHGA  2000
ACZ58633.1   1921  DGSDMLQKLLDNVNTALVELYLHGDKAYFESVRAFYMEKLPPGAYKELTTWYQAGSFHECQKRGASGHKPQGLIEVSHGT  2000
QBZ78369.1   1921  DGSDMLQKLLDNVNTALVELYLHGDRAYFESVRAFYMEKLPPGAYKELTTWYQAESFHECQKSGESGYKPQGLIEISHGT  2000
ARO69851.1   1921  EGSDMLQKLLDNVNTALVELYLHGDRAHFESVRAFYMGKLPPGAYKELTTWFQAEAFHECQRSGESGYKPQGLIEISHGA  2000
QYU59220.1   1921  DGSDMLQKLLDNVNTALVELYLHGDRTYFESVRAFYMEKLPPGAYKELTTWFQAEAFHECQKSGESGYKPQGLIEISHGA  2000
AFV34755.1   1921  DGSDMLQKLLDNVNTALVELYLHGDRAYFESVRAFYMGKLPPGSYKELTTWFQAESFHECQKSGESGYKPQGLIEVSHGT  2000
QGP72544.1   1921  DGSDMLQKLLDNVNTALVELYLHGNRAYFESVRAFYTEKLPPGAYKELTTWYQAESFHECQKSGESGYKPQGLIEISHGA  2000
QYA72352.1   1921  DGSDMLQKLLDNVNTALVELYLHGDRAYFESVRVFYMGRLPPGAYKELTTWYQAETFHEYQKSGESGYKPQGLIEISHGA  2000
ARO69854.1   1921  DGSDLLQKLLDNVNTALVELYLHGNRAYFESVRAFYTEKLPPGAYRELTTWFQAESFHECHKSGESGYKPQGLIEVSHGT  2000
ARO69860.1   1921  DGSDLLQKLLDNVNTALVELYLHGNRAYFESVRAFYTEKLPPGAYRELTTWFQAESFHECQKSGESGYKPQGLIEVSHGT  2000
ARO69863.1   1921  DGSDLLQKLLDNVNTALVELYLHGNRAYFESVRAFYTEKLPPGAYRELTTWFQAESFHECQKSGESGYKPQGLIEVSHGT  2000
DAZ85707.1   1921  DGSDMLQKLLDNVNTALVELYLHGDRAYFDSVRAFYMEKLPPGAYKELKTWYEVESFHECHKSGESGYKPQGLIEICHGA  2000
AWO67781.1   1921  DGSDMLQKLLDNVNTALVELYLHGDRAYFESVRTFYLEKLPPGAYKELRTWFEAESFHECQKSGESGYKPQGLIEVSHGA  2000
QBZ78367.1   1921  DGSDMLQKLLDNVNTALVELYLHGDKAYFESVRAFYMEKLPPGAYKELTTWYQAESFHECQKSGESGYKPQGLIEVSHGT  2000
ARO69873.1   1921  DGSDLLQKLLDNVNTALVELYLHGNRAYFESVRAFYTEKLPPGAYRELTTWFQAESFHECQKSGESGYKPQGLIEVSHGT  2000
AND76471.1   1921  DGSDLLQKLLDNVNTALVELYLHGNRAYFESVRAFYTEKLPPGAYRELTTWFQAESFHECHKSGESGYKPQGLIEVSHGT  2000
ARO69849.1   1921  DGSDLLQKLLDNVNTALVELYLHGDRAYFESVRAFYTEKLPPGAYKELTTWFQAESFHECHKSGESGYKPQGLIEVSHGT  2000
```

```
ARO69889.1   1921  DGSDLLQKLLDNVNTALVELYLHGNRAYFESVRAFYTEKLPPGAYRELTTWFQAESFHECHKSGESGYKPQGLIEVSHGT  2000
ARO69867.1   1921  DGSDLLQKLLDNVNTALVELYLHGNRAYFESVRAFYTEKLPPGAYRELTTWFQAESFHECHKSGESGYKPQGLIEVSHGT  2000
ARO69904.1   1921  DGSDLLQKLLDNVNTALVELYLHGNRAYFESVRAFYTEKLPPGAYRELTTWFQAESFHECHKSGESGYKPQGLIEVSHGT  2000
ARO69869.1   1921  DGSDLLQKLLDNVNTALVELYLHGNRAYFESVRAFYTEKLPPGAYRELTTWFQAESFHECQKSGESGYKPQGLIEVSHGT  2000
ARO69884.1   1921  DGSDLLQKLLDNVNTALVELYLHGNRAYFESVRAFYTEKLPPGAYRELTTWFQAESFHECHKSGESGYKPQGLIEVSHGT  2000
ARO69878.1   1921  DGSDLLQKLLDNVNTALVELYLHGDRAYFESVRAFYTEKLPPGAYRELTTWFQAESFHECHKSGESGYKPQGLIEVSHGT  2000
ARO69885.1   1921  DGSDLLQKLLDNVNTALVELYLHGNRAYFESVRAFYTEKLPPGAYRELTTWFQAESFHECHKSGESGYKPQGLIEVSHGT  2000
ARO69841.1   1921  DGSDLLQKLLDNVNTALVELYLHGDRAYFESVRAFYTEKLPPGAYKELTTWFQAESFHECHKSGESGYKPQGLIEVSHGT  2000
ARO69891.1   1921  DGSDMLQKLLDNVNTALDELYLHGDRVYFESVRAFYSEKLPPGAYKELTTWFQAESFHEYQKSGESGYKPQGLIEVSHGT  2000
ARO69868.1   1921  DGSDMLQKLLDNVNTALDELYLHGDRVYFESVRAFYSEKLPPGAYKELTTWFQAESFHEYQKSGESGYKPQGLIEVSHGT  2000
ARO69900.1   1921  DGSDLLQKLLDNVNTALVELYLHGNRAYFESVRAFYIEKLPPGAYKELTTWFQAESFHEYHKSGESGYKPQGLIEVSHGT  2000
DAZ85706.1   1921  DGSDMLQKLLDNVNTALVELYLHGDRACFDSVRAFYMEKLPPGAYKELRTWYEAESFHECHKSGESGYKPQGLIEISHGA  2000
ARO69899.1   1921  DGSDMLQKLLDNVNTALDELYLHGDRVYFESVRAFYSEKLPPGAYKELTTWFQAESFHEHQKSGESGYKPQGLIEVSHGT  2000
ARO69898.1   1921  DGSDLLQKLLDNVNTALVELYLHGNRAYFESVRAFYTEKLPPGAYRELTTWFQAESFHECHKSGESGYKPQGLIEVSHGT  2000
AYA22297.1   1921  NGSDMLQKLLDNVNTALVELFLHGDRAYFESVRTFYIEKLPPGAYKELTTWYQAESFHECQKSGESGYKPQGLIEVSHGT  2000
QYU59222.1   1921  DGSDMLQKLLDNVNTALVELYLHGDKAYFESVRAFYMDRLPPGAYKELTTWYQAESFHECQKSGESGYKPQGLIEISHGA  2000
ARO69845.1   1921  DGSDLLQKLLDNVNTALVELYLHGNRAYFESVRAFYTEKLPPGAYRELTTWFQAESFHECHKSGESGYKPQGLIEVSHGT  2000
AND76473.1   1921  DGSDMLQKLLDNVNTALDELYLHGDRVYFESVRAFYSEKLPPGAYKELTTWFQAESFHEYQKSGESGYKPQGLIEVSHGT  2000
ARO69857.1   1921  DGSDMLQKLLDNVNTALDELYLHGDRVYFESVRAFYSEKLPPGAYKELTTWFQAESFHEYQKSGESGYKPQGLIEVSHGT  2000
DAZ85708.1   1921  DGSDMLQKLLDNVNTALVELYLYGDRVHFESVRAFYMEKLPPGAYKELTTWFQAESFHECQKSGESGYKPQGLIEISHGT  2000
ARO69881.1   1921  DGSDMLQKLLDNVNTALVELYLHGDRAYFESVRAFYMEKLPPGAYKELRTWFQAESFHECQRSGESGYKPQGLVEISHGA  2000
ARO69893.1   1921  DGSDMLQKLLDNVNTALVELYLHGDRAYFESVRAFYMEKLPPGAYKELRTWFQAESFHECQRSGESGYKPQGLVEISHGA  2000
ARO69897.1   1921  DGSDMLQKLLDNVNTALVELYLHGDRAYFESVRAFYMEKLPPGAYKELRTWFQAESFHECQRSGESGYKPQGLVEISHGA  2000
ARO69872.1   1921  DGSDMLQKLLDNVNTALVELYLHGDRAYFESVRAFYMEKLPPGAYKELRTWFQAESFHECQRSGESGYKPQGLVEISHGA  2000
ARO69843.1   1921  DGSDMLQKLLDNVNTALVELYLHGDRAYFESVRAFYMEKLPPGAYKELRTWFQAESFHECQRSGESGYKPQGLVEISHGA  2000
ARO69886.1   1921  DGSDMLQKLLDNVNTALVELYLHGDRAYFESVRAFYMEKLPPGAYKELRTWFQAESFHECQRSGESGYKPQGLVEISHGA  2000
ARO69864.1   1921  DGSDMLQKLLDNVNTALVELYLHGDRAYFESVRAFYMEKLPPGAYKELRTWFQAESFHECQRSGESGYKPQGLVEISHGA  2000
ARO69876.1   1921  DGSDMLQKLLDNVNTALVELYLHGDRAYFESVRAFYMEKLPPGAYKELRTWFQAESFHECQRSGESGYKPQGLVEISHGA  2000
ARO69888.1   1921  DGSDMLQKLLDNVNTALVELYLHGDRAYFESVRAFYMEKLPPGAYKELRTWFQAESFHECQRSGESGYKPQGLVEISHGA  2000
ARO69853.1   1921  DGSDMLQKLLDNVNTALVELYLHGDRAYFESVRAFYMERLPPGAYKELRTWFQAESFHECQRSGESGYKPQGLVEISHGA  2000
ARO69903.1   1921  DGSDMLQKLLDNVNTALVELYLHGDRAYFESVRAFYMEKLPPGAYKELRTWFQAESFHECQRSGESGYKPQGLVEISHGA  2000
ARO69865.1   1921  DGSDMLQKLLDNVNTALVELYLHGDRAYFESVRAFYMEKLPPGAYKELRTWFQAESFHECQRSGESGYKPQGLVEISHGA  2000
ARO69902.1   1921  DGSDMLQKLLDNVNTALVELYLHGDRAYFESVRAFYMEKLPPGAYKELRTWFQAESFHECQRSGESGYKPQGLVEISHGA  2000
ARO69861.1   1921  DGSDMLQKLLDNVNTALVELYLHGDRAYFESVRAFYMEKLPPGAYKELRTWFQAESFHECQRSGESGYKPQGLVEISHGA  2000
ARO69850.1   1921  DGSDMLQKLLDNVNTALVELYLHGDRAYFESVRAFYMEKLPPGAYKELRTWFQAESFHECQRSGESGYKPQGLVEISHGA  2000
QYU59227.1   1921  DGSDMLQKLLDNVNTALVELYLHGDRTYFESVRTFYMEKLPPGAYKELLTWFQAESFHECQKSGESGYKPQGLIEVSHGT  2000
AFM91094.1   1921  DGSDMLQKLLDNVNTALVELYLHGDRAYFDSVRAFYMEKLPPGAYKELRTWFEAESIHECHKSGESGYKPQGLIEVSHGA  2000
ARO69883.1   1921  DGSDMLQKLLDNVNTALVELYLHGDRAYFESVRAFYMEKLPPGAYKELRTWFQAESFHECQRSGESGYKPQGLVEISHGA  2000
ARO69879.1   1921  DGSDMLQKLLDNVNTALVELYLHGDRAYFESVRAFYMEKLPPGAYKELRTWFQAESFHECQRSGESGYKPQGLVEISHGA  2000
AND76472.1   1921  DGSDMLQKLLDNVNTALVELYLHGDRAYFESVRAFYMEKLPPGAYKELRTWFQAESFHECQRSGESGYKPQGLVEISHGA  2000
ARO69846.1   1921  DGSDMLQKLLDNVNTALVELYLHGDRAYFESVRAFYMEKLPPGAYKELRTWFQAESFHECQRSGESGYKPQGLVEISHGA  2000
ARO69870.1   1921  DGSDMLQKLLDNVNTALVELYLHGDRAYFESVRAFYMEKLPPGAYKELKTWFQAESFHECQRSGESGYKPQGLVEISHGA  2000
ARO69856.1   1921  DGSDMLQKLLDNVNTALVELYLHGDRAYFESVRAFYMEKLPPGAYKELRTWFQAESFHECQRSGESGYKPQGLVEISHGA  2000
ARO69847.1   1921  DGSDMLQKLLDNVNTALGELYLHGNRAYFESVRAFYTEKLPPGAYKELMTWFQAESFHECQKSGESGYKPQGLIEVSHGT  2000
ARO69875.1   1921  DGSDMLQKLLDNVNTALGELYLHGNRAYFESVRAFYTEKLPPGAYKELMTWFQAESFHECQKSGESGYKPQGLIEVSHGT  2000
ARO69895.1   1921  DGSDMLQKLLDNVNTALGELYLHGNRAYFESVRAFYTEKLPPGAYKELMTWFQAESFHECQKSGESGYKPQGLIEVSHGT  2000
ARO69880.1   1921  DGSDMLQKLLDNVNTALGELYLHGNRAYFESVRAFYTEKLPPGAYKELMTWFQAESFHECQKSGESGYKPQGLIEVSHGT  2000
BCT87061.1   1921  DGSDLLQKLLDNVNTALVELYLHADRDYFESVRAFYMEKLPPGAYKELTTWYQAESFHECQKSGESGYKPQGLIEVSHGT  2000
QYU59225.1   1921  DGSDLLQKLLDNVNTALIELYLHADREYFESVRTFYLEKLPPGAYKELRTWYEAESFHEAHKSGESGYKPQGLIEISHGA  2000
APD26306.1   1921  DGSDLLQKLLDNVNTALVELYLHGDREYYESVMAFYMEKLPPGAYKQLTTWHQAESFHECQKSGESGYKPQGLIEVSHGA  2000


NP_619689.1  2001  AFASFTQQAGTELEKHDICPGLSIAGTKYIATENEIVLSLSSVLPGDRNVFKLDLPCGDGIGRLPSKCSILNLRKPGLVM  2080
AWO68042.1   2001  AFASFTQQAGTELEKHDICPGLSIAGTKYIATENEIVLSLSSVLPGDRNVFKLDLPCGDGIGRLPSKCSILNLRKPGLVM  2080
QYU59223.1   2001  AFASFTQQAGTELEKHDICPGLSIAGTKYIATENEIVISLSSILPGDRNVFKLDLPCGDGIGRLPSKPSILNLRKPGLVM  2080
ARO69887.1   2001  AFASFTQQAGTELEKHDICPGLSIAGTKYIATEDEIVLSLSSILPGDRNVFKLDLPCGDGIGRLPSKGSILNLRKPGLVK  2080
ARO69840.1   2001  AFASFTQQAGTELEKHDICPGLSIAGTKYIATEDEIVLSLSSILPGDRNVFKLDLPCGDGIGRLPSKGSILNLRKPGLVK  2080
ARO69892.1   2001  AFASFTQQAGTELEKHDICPGLSIAGTKYIATEDEIVLSLSSILPGDRNVFKLDLPCGDGIGRLPSKGSILNLRKPGLVK  2080
ARO69862.1   2001  AFASFTQQAGTELEKHDICPGLSIAGTKYIATEDEIVLSLSSIQPGDRNVFKLDLPCGDGIGRLPSKGSILNLRKPGLVK  2080
ARO69844.1   2001  AFASFTQQAGTELEKHDICPGLSIAGTKYIATEDEIVLSLSSILPGDRNVFKLDLPCGDGIGRLPSKGSILNLRKPGLVK  2080
ARO69896.1   2001  AFASFTQQAGTELEKHDICPGLSIAGTKYIATEDEIVLSLSSILPGDRNVFKLDLPCGDGIGRLPSKGSILNLRKPGLVK  2080
QGP72541.1   2001  AFASFVQQAGTELEKHDICPGLSVAGTKYIATENEIVLSLSSILPGDRNVLKLDLPCGDGIGRLPSKSSILNLRKPGPVM  2080
ARO69901.1   2001  AFASFTQQAGTELEKHDICPGLSIAGTKYIATEDEIVLSLSSILPGDRNVFKLDLPCGDGIGRLPSKGSILNLRKPGLVK  2080
AYA22299.1   2001  AFASFTQQAGTELEKHDICPGLSIAGTKYIATENEIVLSLSSILPGDRNVFKLDLPCGDGIGRLPSKCSVLNLRKPGLVM  2080
ARO69890.1   2001  AFASFTQQAGTELEKHDICPGLSIAGTKYIATEDEIVLSLSSILPGDRNVFKLDLPCGDGIGRLPSKGSILNLRKPGLVK  2080
ARO69859.1   2001  AFASFTQQAGTELEKHDICPGLSIAGTKYIATEDEIVLSLSSILPGDRNVFKLDLPCGDGIGRLPSKGSILNLRKPGLVK  2080
ARO69905.1   2001  AFASFTQQAGTELEKHDICPGLSIAGTKYIATEDEIVLSLSSILPGDRNVFKLDLPCGDGIGRLPSKGSILNLRKPGLVK  2080
ARO69871.1   2001  AFASFTQQAGTELEKHDICPGLSIAGTKYIATEDEIVLSLSSILPGDRNVFKLDLPCGDGIGRLPSKGSILNLRKPGLVK  2080
ARO69848.1   2001  AFASFTQQAGTELEKHDICPGLSIAGTKYIATEDEIVLSLSSILPGDRNVFKLDLPCGDGIGRLPSKGSILNLRKPGLVK  2080
ARO69874.1   2001  AFASFTQQAGTELEKHDICPGLSIAGTKYIATEDEIVLSLSSILPGDRNVFKLDLPCGDGIGRLPSKGSILNLRKPGLVK  2080
ARO69882.1   2001  AFASFTQQAGTELEKHDICPGLSIAGTKYIATEDEIVLSLSSILPGDRNVFKLDLPCGDGIGRLPSKGSILNLRKPGLVK  2080
QYU59221.1   2001  AFASFTQQAGTELEKHDICPGLSIAGTKYIAAENEIVLSLSSILPGDRNTFKLDLPCGDGIGRLPSKGSIWNLRRPGLVM  2080
ARO69842.1   2001  AFASFTQQAGTELEKHDICPGLSIAGTKYIATEDEIVLSLSSILPGDRNVFKLDLPCGDGIGRLPSKGSILNLRKPGLVK  2080
AFV34731.1   2001  AFASFTQQAGTELEKHDICPGLSIAGTKYIAAENEIVLSLSSILPGDRNVFKLDLPCGDGIGRLPSKSSILNLRKPGLVM  2080
AND76470.1   2001  AFASFTQQAGTELEKHDICPGLSIAGTKYIATEDEIVLSLSSILPGDRNVFKLDLPCGDGIGRLPSKGSILNLRKPGLVK  2080
ACZ58632.1   2001  AFASFTQQAGTELEKHDICPGLSIAGTKYIATENEIVLSLSSFLPGDRNAFKLDLPCGDGIGRLPSKCSILNLRKPGLVM  2080
QBZ78374.1   2001  AFASFTQQAGTELEKHDICPGLSIAGTKYIATENEIVISLSSILPGDINVYKLDLPCGDGIGRLPSKSSILNLRKPGLVM  2080
QGP72540.1   2001  AFASFAQQAGTELEKHDICPGLSIAGTKYIATEDEIVLSLSSILPGDRNVYKLDLPCGDGIGRLPSKSSILNLRKPGLVM  2080
QBZ78372.1   2001  AFASFTQQAGTELEKHDICPGLSIAGTKYIATENEIVISLSSILPGDINVYKLDLPCGDGIGRLPSKSSILNLRKPGLVM  2080
QYU59224.1   2001  AFASFTQQAGTELEKHAICPGLSIAGTKYIATENEIVLSLSSILPGDRNAFKLDLPCGDGIGRLPSKGSILNLRRPGLVM  2080
ARO69877.1   2001  AFASFTQQAGTELEKHDICPGLSIAGTKYIATEDEIVLSLSSILPGDRNVFKLDLPCGDGIGRLPSKGSILNLRKPGLVK  2080
ARO69858.1   2001  AFASFTQQAGTELEKHDICPGLSIAGTKYIATEDEIVLSLSSILPGDRNVFKLDLPCGDGIGRLPSKGSILNLRKPGLVK  2080
ARO69855.1   2001  AFASFTQQAGTELEKHDICPGLSIAGTKYIATEDEIVLSLSSILPGDRNVFKLDLPCGDGIGRLPSKGSILNLRKPGLVK  2080
QGP72542.1   2001  AFASFAQQAGTELEKHDICPGLSIAGTKYIATEDEIVLSLSSILPGDRNVFKLDLPCGDGIGRLPSKSSILNLRKPGLVM  2080
AYQ93055.1   2001  AFASFTQQAGTELEKHDICPGLSIAGTKYIATESEIVLSLSSILPGDRNVFKLDLPCGDGIGRLPSKCSILNLRKPGLVL  2080
AYQ93053.1   2001  AFASFTQQAGTELEKHDICPGLSIAGTKYIATESEIVLSLSSILPGDRNVFKLDLPCGDGIGRLPSKCSILNLRKPGLVQ  2080
AYQ93051.1   2001  AFASFTQQAGTELEKHDICPGLSIAGTKYIATENEIVLSLSSVLPGDRNVFKLDLPCGDGIGRLPSKSSILGLRKPGLVM  2080
AFI71813.1   2001  AFASFTQQAGTELEKHDICPGLSIAGTKYIASENEIILSLSSILPGDRNVFKLDLPCGDGIGRLPSKSSILKLREPGLVL  2080
```

```
QGP72539.1   2001  AFASFAQQAGTELEKHDICPGLSIAGTKYIATEDEIVLSLSSILPGDRNVFKLDLPCGDGIGRLPSKSSILNLRKPGLVM  2080
QYU59226.1   2001  AFASFTQQAGTELEKHDICPGLSIAGTKYIATENEIVLSLSSTLPGDRNVFKLDLPCGDGIGRLPSKGSILNLRAPGLVM  2080
ARO69852.1   2001  AFASFTQQAGTELEKHDICPGLSIAGTKYIATEDEIVLSLSSILPGDRNVFKLDLPCGDGIGRLPSKGSILNLRKPGLVK  2080
QGP72543.1   2001  AFASFVQQAGTELEKHDICPGLSIAGTKYVATESEIVLSLSSILPGDRNVFKLDLPCGDGIGRLPSKSSIINLRRPGLVM  2080
QYU59219.1   2001  AFASFTQQAGTELEKHAICPGLSIAGARYIATENEIVLSLSSILPGDRNVSKLDLPCGDGIGRLPSKSSILNLRKKGLVM  2080
AGT42200.1   2001  AFASFTQQAGTELEKHDICPGLSIAGTKYIANENEIILSLSSILPGDKNVFKLDLPCGDGIGRLPSKSSILKLREPGLVL  2080
ACZ58633.1   2001  AFASFTQQAGTELEKHDICPGLSIAGTKYIATENEIVLSLSSILPGDRNVFKLDLPCGDGIGRLPSKGSILNLRKPGLVM  2080
QBZ78369.1   2001  AFASFTQQAGTELEKHDICPGLSIAGTKYIAIENEIVLSLSSILPGDRNVFKLDLPCGDGIGRLPSKSSILNLRRPGLVM  2080
ARO69851.1   2001  AFASFTQQAGTELEKHDICPGLSIAGTKYIATEDEIVLSLSSILPGDKNVFKLDLPCGDGIGRLPSKGSILNLRKPGLVT  2080
QYU59220.1   2001  AFASFTQQAGTELEKHDICPGLSIAGTKYIATEDEIVLSLSSILPGDRNVFKLDLPCGDGIGRLPSKGSILNLRKPGLVS  2080
AFV34755.1   2001  AFASFTQQAGTELEKHDICPGLSIAGTKYIATEDEIVLSLSSILPGDRNVFKLDLPCGDGIGRLPSKGSILGLRKPGLVM  2080
QGP72544.1   2001  AFASFTQQAGSELEKHDICPGLSIAGTKYIATDDEIVLSLSSILPGDRNVFKLDLPCGDGIGRLPSKGSILNLRKPGLVS  2080
QYA72352.1   2001  AFASFTQQAGTELEKHDICPGLSIAGTKYIATDNDIVLSLSSILPGDRNTFKLDLPCGDGIGRLPSKRSIQNLREPGLVM  2080
ARO69854.1   2001  AFASFVQQAGTELERHDICPGLSIAGTKYIATENEIVLTLSSILPGDRNVFKLDLPCGDGIGRLPSKCSIQNLRKPGLVR  2080
ARO69860.1   2001  AFASFVQQAGTELERHDICPGLSIAGTKYIATENEIVLTLSSILPGDRNVFKLDLPCGDGIGRLPSKCSIQNLRKPGLVR  2080
ARO69863.1   2001  AFASFVQQAGTELERHDICPGLSIAGTKYIATENEIVLTLSSILPGDRNVFKLDLPCGDGIGRLPSKCSIQNLRKPGLVR  2080
DAZ85707.1   2001  AFASFTQQAGTELEKHDICPGLSIAGTKYIATEDEIVLSLSSILPGDRNVFKLDLPCGDGIGRLPSKSSILNLRRPGLVM  2080
AWO67781.1   2001  AFASFTQQAGTELEKHAICPGLSIAGTKYIATEDEIVLSLSSILPGDRNVHKLDLPCGDGIGRLPSKGSILNLRRPGLVM  2080
QBZ78367.1   2001  AFASFTQQAGTELEKHDICPGLSIAGTKYVATENEIVLSLSSILPGDRNVFKLDLPCGDGIGRLPSKGSILNLRKPGLVM  2080
ARO69873.1   2001  AFASFVQQAGTELERHDICPGLSIAGTKYIATENEIVLTLSSILPGDRNVFKLDLPCGDGIGRLPSKCSIQNLRKPGLVR  2080
AND76471.1   2001  AFASFVQQAGTELERHDICPGLSIAGTKYIATENEIVLTLSSILPGDRNVFKLDLPCGDGIGRLPSKCSIQNLRKPGLVR  2080
ARO69849.1   2001  AFASFVQQAGTELERHDICPGLSIAGTKYIATENEIVLTLSSILPGDRNVFKLDLPCGDGIGRLPSKCSIQNLRKPGLVR  2080
ARO69889.1   2001  AFASFVQQAGTELERHDICPGLSIAGTKYIATENEIVLTLSSILPGDRNVFKLDLPCGDGIGRLPSKCSIQNLRKPGLVR  2080
ARO69867.1   2001  AFASFVQQAGTELERHDICPGLSIAGTKYIATENEIVLTLSSILPGDRNVFKLDLPCGDGIGRLPSKCSIQNLRKPGLVR  2080
ARO69904.1   2001  AFASFVQQAGTELERHDICPGLSIAGTKYIATENEIVLTLSSILPGDRNVFKLDLPCGDGIGRLPSKCSIQNLRKPGLVR  2080
ARO69869.1   2001  AFASFVQQAGTELERHDICPGLSIAGTKYIATENEIVLTLSSILPGDRNVFKLDLPCGDGIGRLPSKCSIQNLRKPGLVR  2080
ARO69884.1   2001  AFASFVQQAGTELERHDICPGLSIAGTKYIATENEIVLTLSSILPGDRNVFKLDLPCGDGIGRLPSKCSIQNLRKPGLVR  2080
ARO69878.1   2001  AFASFVQQAGTELERHDICPGLSIAGTKYIATENEIVLTLSSILPGDRNVFKLDLPCGDGIGRLPSKCSIQNLRKPGLVR  2080
ARO69885.1   2001  AFASFVQQAGTELERHDICPGLSIAGTKYIATENEIVLTLSSILPGDRNVFKLDLPCGDGIGRLPSKCSIQNLRKPGLVR  2080
ARO69841.1   2001  AFASFVQQAGTELERHDICPGLSIAGTKYIATENEIVLTLSSILPGDRNVFKLDLPCGDGIGRLPSKCSIQNLRKPGLVR  2080
ARO69891.1   2001  AFASFVQQAGTELERHDICPGLSIAGTKYIATENEIVLTLSSILPGDRNVFKLDLPCGDGIGRLPSKCSIQNLRKPGLVR  2080
ARO69868.1   2001  AFASFVQQAGTELERHDICPGLSIAGTKYIATENEIVLSLSSILPGDRNVFKLDLPCGDGIGRLPSKCSIQNLRKPGLVR  2080
ARO69900.1   2001  AFASFVQQAGTELERHDICPGLSIAGTKYIATENEIVLTLSSILPGDRNVFKLDLPCGDGIGRLPSKCSIQNLRKPGLVR  2080
DAZ85706.1   2001  AFASFTQQAGTELEKHDICPGLSIAGTKYIATEDEIVLSLSSILPGDRNVFKLDLPCGEGIGRLPSKCSILNLRKPGLVM  2080
ARO69899.1   2001  AFASFVQQAGTELERHDICPGLSIAGTKYIATENEIVLSLSSILPGDRNVFKLDLPCGDGIGRLPSKCSIQNLRKPGLVR  2080
ARO69898.1   2001  AFASFVQQAGTELERHDICPGLSIAGTKYIATENEIVLTLSSILPGDRNVFKLDLPCGDGIGRLPSKCSIQNLRKPGLVR  2080
AYA22297.1   2001  AFASFTQQAGTELEKHDICPGLSIAGTRYIATENEIVLSLSSILPGDRNAIKLDLPCGDGIGRLPSKGSILNLRSPGLVM  2080
QYU59222.1   2001  AFASFTQQAGTELEKHDICPGLSIAGTKYIATEDEIVLSLSSVLPGDRNVFKLDLPCGDGIGRLPSKGSILKLREPGLVM  2080
ARO69845.1   2001  AFASFVQQAGTELERHDICPGLSIAGTKYIATENEIVLTLSSILPGDRNVFKLDLPCGDGIGRLPSKCSIQNLRKPGLVR  2080
AND76473.1   2001  AFASFVQQAGTELERHDICPGLSIAGTKYIATENEIVLSLSSILPGDRNVFKLDLPCGDGIGRLPSKCSIQNLRKPGLVR  2080
ARO69857.1   2001  AFASFVQQAGTELERHDICPGLSIAGTKYIATENEIVLTLSSILPGDRNVFKLDLPCGDGIGRLPSKCSIQNLRKPGLVR  2080
DAZ85708.1   2001  AFASFAQQAGTELEKHDICPGLSIAGTKYIATENEIVLSLSSILPGDKNVFKLDLPCGDGIGRLPSKSSIQNLRNPGLVS  2080
ARO69881.1   2001  AFASFTQQAGTELEKHDICPGLSIAGTKYIATEDEIVLSLSSILPGDKNVFKLDLPCGDGIGRLPSKGSILGLRKPGLVR  2080
ARO69893.1   2001  AFASFTQQAGTELEKHDICPGLSIAGTKYIATEDEIVLSLSSILPGDKNVFKLDLPCGDGIGRLPSKGSILGLRKPGLVR  2080
ARO69897.1   2001  AFASFTQQAGTELEKHDICPGLSIAGTKYIATEDEIVLSLSSILPGDKNVFKLDLPCGDGIGRLPSKGSILGLRKPGLVR  2080
ARO69872.1   2001  AFASFTQQAGTELEKHDICPGLSIAGTKYIATEDEIVLSLSSILPGDKNVFKLDLPCGDGIGRLPSKGSILGLRKPGLVR  2080
ARO69843.1   2001  AFASFTQQAGTELEKHDICPGLSIAGTKYIATEDEIVLSLSSILPGDKNVFKLDLPCGDGIGRLPSKGSILGLRKPGLVR  2080
ARO69886.1   2001  AFASFTQQAGTELEKHDICPGLSIAGARYIATEDEIVLSLSSILPGDKNVFKLDLPCGDGIGRLPSKGSILGLRKPGLVR  2080
ARO69864.1   2001  AFASFTQQAGTELEKHDICPGLSIAGTKYIATEDEIVLSLSSILPGDKNVFKLDLPCGDGIGRLPSKGSILGLRKPGLVR  2080
ARO69876.1   2001  AFASFTQQAGTELEKHDICPGLSIAGTKYIATEDEIVLSLSSILPGDKNVFKLDLPCGDGIGRLPSKGSILGLRKPGLVR  2080
ARO69888.1   2001  AFASFTQQAGTELEKHDICPGLSIAGTKYIATEDEIVLSLSSILPGDKNVFKLDLPCGDGIGRLPSKGSILGLRKPGLVR  2080
ARO69853.1   2001  AFASFTQQAGTELEKHDICPGLSIAGTKYIATEDEIVLSLSSILPGDKNVFKLDLPCGDGIGRLPSKGSILGLRKPGLVR  2080
ARO69903.1   2001  AFASFTQQAGTELEKHDICPGLSIAGTKYIATEDEIVLSLSSILPGDKNVFKLDLPCGDGIGRLPSKGSILGLRKPGLVR  2080
ARO69865.1   2001  AFASFTQQAGTELEKHDICPGLSIAGTKYIATEDEIVLSLSSILPGDKNVFKLDLPCGDGIGRLPSKGSILGLRKPGLVR  2080
ARO69902.1   2001  AFASFTQQAGTELEKHDICPGLSIAGTKYIATEDEIVLSLSSILPGDKNVFKLDLPCGDGIGRLPSKGSILGLRKPGLVR  2080
ARO69861.1   2001  AFASFTQQAGTELEKHDICPGLSIAGTKYIATEDEIVLSLSSILPGDKNVFKLDLPCGDGIGRLPSKGSILGLRKPGLVR  2080
ARO69850.1   2001  AFASFTQQAGTELEKHDICPGLSIAGTKYIATEDEIVLSLSSILPGDKNVFKLDLPCGDGIGRLPSKGSILGLRKPGLVR  2080
QYU59227.1   2001  AFASFVQQAGTELEKHDICPGLSIVGTKYIATENEIVLSLSSTLPGDRNVFKLDLPCGDGIGRLPSKSSILHLRKPGLVM  2080
AFM91094.1   2001  AFASFTQQAGTELEKHAICPGLSIAGTKYIAAEGEIVLSLSSILPGDKNVFKLDLPCGDGIGRLPSKGSILNLRKPGLVM  2080
ARO69883.1   2001  TFASFTQQAGTELEKHDICPGLSIAGTKYIATEDEIVLSLSSILPGDKNVFKLDLPCGDGIGRLPSKGSILNLRKPGLVR  2080
ARO69879.1   2001  AFASFTQQAGTELEKHDICPGLSIAGTKYIATEDEIVLSLSSILPGDKNVFKLDLPCGDGIGRLPSKGSILGLRKPGLVR  2080
AND76472.1   2001  AFASFTQQAGTELEKHDICPGLSIAGTKYIATEDEIVLSLSSILPGDKNVFKLDLPCGDGIGRLPSKGSILSLRKPGLVR  2080
ARO69846.1   2001  AFASFTQQAGTELEKHDICPGLSIAGTKYIANEDEIVLSLSSILPGDKNVFKLDLPCGDGIGRLPSKGSILDLRKPGLVR  2080
ARO69870.1   2001  TFASFTQQAGTELEKHDICPGLSIAGTKYIATEDEIVLSLSSILPGDKNVFKLDLPCGDGIGRLPSKGSILNLRKPGLVR  2080
ARO69856.1   2001  TFASFTQQAGTELEKHDICPGLSLAGTKYIATEDEIVLSLSSILPGDKNVFKLDLPCGDGIGRLPSKGSILNLRKPGLVR  2080
ARO69847.1   2001  AFASFVQQAGTELERHDICPGLSIAGTKYIATENEIVLTLSSILPGDRNVFKLDLPCGDGIGRLPSKCSIQNLRKPGLVR  2080
ARO69875.1   2001  AFASFVQQAGTELERHDICPGLSIAGTKYIATENEIVLTLSSILPGDRNVFKLDLPCGDGIGRLPSKCSIQNLRKPGLVR  2080
ARO69895.1   2001  AFASFVQQAGTELERHDICPGLSIAGTKYIATENEIVLTLSSILPGDRNVFKLDLPCGDGIGRLPSKCSIQNLRKPGLVR  2080
ARO69880.1   2001  AFASFVQQAGTELERHDICPGLSIAGTKYIATENEIVLTLSSILPGDRNVFKLDLPCGDGIGRLPSKCSIQNLRKPGLVR  2080
BCT87061.1   2001  KFASFVQQAGTELEKHDICPGLSIAGARYIATKDEIVLSLSSILPGDVNAFKLELPCGDGIGRLPSKSSILMLRRPGLVK  2080
QYU59225.1   2001  AFASFVQQAGTELEKHDICPGLSVAGAKYVAKGGEIVLSLSSRLPGDVDTFKLELPCGDGIGRLPSKASILALRKPGLVM  2080
APD26306.1   2001  AFAGFVRQSGTELEKHDICPGLAVAGAKYIAAENEIILSLSSMLPGDVNASKLELPCGDGIGRLPSKTSILALRKPGLVT  2080
```

```
NP_619689.1  2081  RLCKRAQDEKKTLVIRDERPYIGAWAVACICGESFGFGQQSVLALYANLLGPNQRNGLASYFSDFESPIHIKKVHAKTNS  2160
AWO68042.1   2081  RLCKRAQDEKKTLVIRDERPYIGAWAVACICGESFGFGQQSVLALYANLLGPNQRNGLASYFSDFESPIHIKKVHAKTNS  2160
QYU59223.1   2081  RLCERAQVEKKTLVIRDERPYIGAWAVACICGESFGFGLQSVLTLYANLLGPNQRNGLASYFSDFESPIHIKRVHAKTNS  2160
ARO69887.1   2081  KLCELAQIEKKTLVIRDERPYIGAWAVACICGESFGFGLQSVLALYANLLGPNQRNGLASYFSDFESPIHIKRVHAKTNS  2160
ARO69840.1   2081  KLCELAQIEKKTLVIRDERPYIGAWAVACICGESFGFGLQSVLALYANLLGPNQRNGLASYFSDFESPIHIKRVHAKTNS  2160
ARO69892.1   2081  KLCELAQIEKKTLVIRDERPYIGAWAVACICGESFGFGLQSVLALYANLLGPNQRNGLASYFSDFESPIHIKRVHAKTNS  2160
ARO69862.1   2081  KLCELAQIEKKTLVIRDERPYIGAWAVACICGESFGFGLQSVLALYANLLGPNQRNGLASYFSDFESPIHIKRVHAKTNS  2160
ARO69844.1   2081  KLCELAQIEKKTLVIRDERPYIGAWAVACICGESFGFGLQSVLALYANLLGPNQRNGLASYFSDFESPIHIKRVHAKTNS  2160
ARO69896.1   2081  KLCELAQIEKKTLVIRDERPYIGAWAVACICGESFGFGLQSVLALYANLLGPNQRNGLASYFSDFESPIHIKRVHAKTNS  2160
QGP72541.1   2081  KLCERAQAEKKTLVIRDERPYIGAWAVACICGESFGFGLQSVLTLYANLLGPNQRNGLASYFSNFESPIHIKKVHAKTNS  2160
ARO69901.1   2081  KLCELAQIEKKTLVIRDERPYIGAWAVACICGESFGFGLQSVLALYANLLGPNQRNGLASYFSDFESPIHIKRVHAKTNS  2160
AYA22299.1   2081  TLCQLARVEKKTLVIRDERPYIGAWAVACICGESFGFGLQSVLTLYANLLGPNRKNGLASYFADFESPIHIKEVHAKTNS  2160
ARO69890.1   2081  KLCELAQIEKKTLVIRDERPYIGAWAVACICGESFGFGLQSVLALYANLLGPNQRNGLASYFSDFESPIHIKRVHAKTNS  2160
ARO69859.1   2081  KLCELAQIEKKTLVIRDERPYIGAWAVACICGESFGFGLQSVLALYANLLGPNQRNGLASYFSDFESPIHIKRVHAKTNS  2160
```

```
ARO69905.1   2081  KLCELAQIEKKTLVIRDERPYIGAWAVACICGESFGFGLQSVLALYANLLGPNQRNGLASYFSDFESPIHIKRVHAKTNS  2160
ARO69871.1   2081  KLCELAQIEKKTLVVRDERPYIGAWAVACICGESFGFGLQSVLTLYANLLGPNQRNGLASYFSDFESPIHIKRVHAKTNS  2160
ARO69848.1   2081  KLCELAQIEKKTLVIRDERPYIGAWAVACICGESFGFGLQSVLALYANLLGPNQRNGLASYFSDFESPIHIKRVHAKTNS  2160
ARO69874.1   2081  KLCELAQIEKKTLVIRDERPYIGAWAVACICGESFGFGLQSVLALYANLLGPNQRNGLASYFSDFESPIHIKRVHAKTNS  2160
ARO69882.1   2081  KLCELAQIEKKTLVIRDERPYIGAWAVACICGESFGFGLQSVLALYANLLGPNQRNGLASYFSDFESPIHIKRVHAKTNS  2160
QYU59221.1   2081  KLCERAQVEKKTLVIRDERPYIGAWAVACICGESFGFGLQSVLALYANLLGPNRRNGLASYFSDFESSIHIKKVHAKTNS  2160
ARO69842.1   2081  KLCELAQIEKKTLVIRDERPYIGAWAVACICGESFGFGLQSVLALYANLLGPNQRNGLASYFSDFESPIHIKRVHAKTNS  2160
AFV34731.1   2081  KLCKRAQVEKKTLVIRDERPYIGAWAVACICGESFGFGLQSVLTLYANLLGPNQKNGLASYFSDFESPICIKKVHAKTNS  2160
AND76470.1   2081  KLCELAQIEKKTLVIRDERPYIGAWAVACICGESFGFGLQSVLALYANLLGPNQRNGLASYFSDFESPIHIKRVHAKTNS  2160
ACZ58632.1   2081  RLCKRAQDEKKTLVIRDERPYIGAWAVACICGESFGFGQQSVLTLYANLLGPNQKNGLASYFSDFESPIHIKRVHAKTNS  2160
QBZ78374.1   2081  RLCERAQVEKKTLVIRDERPYIGAWAVACICGESFGFGLQSVLTLYANLLGPNRRNGLASYFSDFESPIHIQRIHAKTNS  2160
QGP72540.1   2081  QLCKRAQNEKKTLVVRDERPYIGAWAVACICGESFGFGLQSVLALYANLLGPNQRNGLASYFSDFESPIHIKKVHAKTNS  2160
QBZ78372.1   2081  RLCERAQVEKKTLVIRDERPYIGAWAVACICGESFGFGLQSVLTLYANLLGPNRRNGLASYFSDFESPIHIQRIHAKTNS  2160
QYU59224.1   2081  KLCERAQVEKKTLVIRDERPYIGAWAVACICGESFGFGLQSVLALYANLLGPNRRNGLASYFTDFESPVHIKRVHAKTNA  2160
ARO69877.1   2081  KLCELAQIEKKTLVIRDERPYIGAWAVACICGESFGFGLQSVLALYANLLGPNQRNGLASYFSDFESPIHIKRVHAKTNS  2160
ARO69858.1   2081  KLCELAQIEKKTLVIRDERPYIGAWAVACICGESFGFGLQSVLALYANLLGPNQRNGLASYFSDFESPIHIKRVHAKTNS  2160
ARO69855.1   2081  KLCELAQIEKKTLVIRDERPYIGAWAVACICGESFGFGLQSVLALYANLLGPNQRNGLASYFSDFESPIHIKRVHAKTNS  2160
QGP72542.1   2081  RLCKRAQNEKKTLVVRDERPYIGAWAVACICGESFGFGLQSVLALYANLLGPNQRNGLASYFSDFESPIHIKKVHAKTNS  2160
AYQ93055.1   2081  KLCELAQLEKKTLVIRDERPYIGAWAVACICGESFGFGLQSVLALYANLLGPNRRNGLASYFSDFESPTHIRKVHAKTNS  2160
AYQ93053.1   2081  KLCELAQLEKKTLVIRDERPYIGAWAVACICGESFGFGLQSVLALYANLLGPDRRNGLASYFSDFESPTHIRKVHAKTNS  2160
AYQ93051.1   2081  SLCKRAQIEKKTLVIRDERPYIGAWAIACICGESFGFGLQSVLTLYANLLGPNQRNGLASYFSDFESPIHIKKVHAKTNS  2160
AFI71813.1   2081  RLCERAQVERKTLVIRDERPYIGAWAVACICSESFGFGLQSVLALYANLLGPNRKNGLASYFSNFESPTHIKRVHATTNS  2160
QGP72539.1   2081  QLCKRAQNEKKTLVVRDERPYIGAWAVACICGESFGFGLQSVLALYANLLGPNQRNGLASYFSDFESPIHIKKVHAKTNS  2160
QYU59226.1   2081  ELCKRAQVEKKTLVIRGERPYIGAWAVACVCGESFGFGLQSVLTLYANLLGPNQRNGLASYFSDFESPIHIKKVHAKTNS  2160
ARO69852.1   2081  KLCELAQIEKKTLVIRDERPYIGAWAVACICGESFGFGLQSVLALYANLLGPNQRNGLASYFSDFESPIHIKRVHAKTNS  2160
QGP72543.1   2081  SLCKRAQDEKKTLVIRDERPYIGAWAVACICGESFGFGLQSVLTLYANLLGPNQRNGLASYFSDFESPIHIKKVHAKTNS  2160
QYU59219.1   2081  SLCERAQIEKKTLVIRDERPYIGAWAVACICGESFGFGLQSVLTLYANLLGPDRRNGLAGYFSDFESPIHIKKVHAKTNS  2160
AGT42200.1   2081  RLCERAQVERKTLVIRDERPYIGAWAVACICSESFGFGLQSVLALYANLLGPNRKNGLASYFSNFESPTHIKRVHATTNS  2160
ACZ58633.1   2081  KLCERAQVEKKTLVIRDERPYIGAWAVACICGESFGFGLQSVLALYANLLGPDRRNGLASYFSDFESPIHIKRVYAKTNS  2160
QBZ78369.1   2081  KLCERAQVEKKTLVIRDERPYIGAWAVACICGESFGFGLQSVLALYANLLGPNRRNGLASYFSDFESPIHIRKVHARTNS  2160
ARO69851.1   2081  KLCKLAQIEKKTLVIRDDRPYIGAWAVACICGESFGFGIQSVLTLYANLLGPNRKNGLASYFSDFESPTCVKKVYAKTNS  2160
QYU59220.1   2081  RLCKRAQVEGKTLVIRDERPYIGAWAVACICGESFGFGLQSVLTLYANLLGPNRRNGLASYFSDFESPIHIAKIHAKTNS  2160
AFV34755.1   2081  RLCKRAQAEKKTLVIRDERPYIGAWAVACICGESFGFGLQSVLTLYANLLGPNRRNGLASYFSDYESPIHVKRIHAKTNS  2160
QGP72544.1   2081  GLCKRAQVEKKTLVIRDERPYIGAWAVACICGESFGFGLQSVLTLYANLLGPNRRNGLASYFSDFESPIHVKRVHAKTNS  2160
QYA72352.1   2081  RLCKRAQVEKKTLVIRDERPYIGAWAVACICGESFGFGLQSVLTLYANLLGPDRRNGLASYFSDFESPIHIKRVHAKTNS  2160
ARO69854.1   2081  KLCELAQIEKKTLVIRDDRPYIGAWAVACICGESFGFGLQSVLTLYANLLGPNRKNGLASYFSDFESPTCVKRVYAKTNS  2160
ARO69860.1   2081  KLCELAQIEKKTLVIRDDRPYIGAWAVACICGESFGFGLQSVLTLYANLLGPNRKNGLASYFSDFESPTCVKKVYAKTNS  2160
ARO69863.1   2081  KLCELAQIEKKTLVIRDDRPYIGAWAVACICGESFGFGLQSVLTLYANLLGPNRKNGLASYFSDFESPTCVKKVYAKTNS  2160
DAZ85707.1   2081  RLCKIAQEAKRTLVIRDERPYIGAWAVACICGESFGFGLQSVLTLYANLLGPNQRNGLASYFSDFESPIHIKKVHAATNS  2160
AWO67781.1   2081  TLCKLAQDEKKTLVIRDERPYIGAWAVACICGESFGFGSQSVLTLYANLLGPNQRNGLASYFSDFESPICIKKIHAKTNS  2160
QBZ78367.1   2081  KLCERAQVEKKTLVIRDERPYIGAWAVACVCGESFGFGLQSVLTLYANLLGPDRRNGLASYFSDFESPIHIKRVYAKTNS  2160
ARO69873.1   2081  KLCELAQIEKKTLVIRDDRPYIGAWAVACICGESFGFGLQSVLTLYANLLGPNRKNGLASYFSDFESPTCVKKVYAKTNS  2160
AND76471.1   2081  KLCELAQIEKKTLVIRDDRPYIGAWAVACICGESFGFGLQSVLTLYANLLGPNRKNGLASYFSDFESPTCVKKVYAKTNS  2160
ARO69849.1   2081  KLCELAQIEKKTLVIRDDRPYIGAWAVACICGESFGFGLQSVLTLYANLLGPNRKNGLASYFSDFESPTCVKKVYAKTNS  2160
ARO69889.1   2081  KLCELAQIEKKTLVIRDDRPYIGAWAVACICGESFGFGLQSVLTLYANLLGPNRKNGLASYFSDFESPTCVKRVYAKTNS  2160
ARO69867.1   2081  KLCELAQIEKKTLVIRDDRPYIGAWAVACICGESFGFGLQSVLTLYANLLGPNRKNGLASYFSDFESPTCVKRVYAKTNS  2160
ARO69904.1   2081  KLCELAQIEKKTLVIRDDRPYIGAWAVACICGESFGFGLQSVLTLYANLLGPNRKNGLASYFSDFESPTCVKRVYAKTNS  2160
ARO69869.1   2081  KLCELAQIEKKTLVVRDDRPYIGAWAVACICGESFGFGLQSVLTLYANLLGPNRKNGLASYFSDFESPTCVKKVYAKTNS  2160
ARO69884.1   2081  KLCELAQIEKKTLVIRDDRPYIGAWAVACICGESFGFGLQSVLTLYANLLGPNRKNGLASYFSDFESPTCVKRVYAKTNS  2160
ARO69878.1   2081  KLCELAQIEKKTLVIRDDRPYIGAWAVACICGESFGFGLQNVLTLYANLLGPNRKNGLASYFSDFESPTCVKKVYAKTNS  2160
ARO69885.1   2081  KLCELAQIEKKTLVIRDDRPYIGAWAVACICGESFGFGLQSVLTLYANLLGPNRKNGLASYFSDFESPTCVKRVYAKTNS  2160
ARO69841.1   2081  KLCELAQIEKKTLVIRDDRPYIGAWAVACICGESFGFGLQSVLTLYANLLGPNRKNGLASYFSDFESPTCVKRVYAKTNS  2160
ARO69891.1   2081  KLCELAQIEKRTLVIRDDRPYIGAWAVACICGESFGFGLQSVLTLYANLLGPNRKNGLASYFSDFDSPTCIKKVYAKTNS  2160
ARO69868.1   2081  KLCELAQIEKRTLVIRDDRPYIGAWAVACICGESFGFGLQSVLTLYANLLGPNRKNGLASYFSDFDSPTCIKKVYAKTNS  2160
ARO69900.1   2081  KLCELAQIEKKTLVIRDDRPYIGAWAVACICGESFGFGIQSVLTLYANLLGPNRKNGLASYFSDFESPTCVKKVYAKTNS  2160
DAZ85706.1   2081  RLCKIAQETKKTLVIRDERPYIGAWAVACICGESFGFGLQSVLTLYANLLGPNQRNGLASYFSDFESPIHIKRVHASTNS  2160
ARO69899.1   2081  KLCELAQIEKRTLVIRDDRPYIGAWAVACICGESFGFGLQSVLTLYANLLGPNRKNGLASYFSDFDSPTCIKKVYAKTNS  2160
ARO69898.1   2081  KLCELAQIEKKTLVIRDDRPYIGAWAVACICGESFGFGLQSVLTLYANLLGPNRKNGLASYFSDFESPTCVKRVYAKTNS  2160
AYA22297.1   2081  NLCKRAQNEKKTLVIRDERPYIGAWAVACICGESFGFGLQSVLTLYANLLGPDRRNGLASYFTDFESPIHVKKVYAKTNS  2160
QYU59222.1   2081  KLCKRALVEKKTLVIRDERPYIGAWAVACICGESFGFGLQSVLALYANLLGPDRRNGLASYFSDFESPTHIKKIHAKTNS  2160
ARO69845.1   2081  KLCELAQIEKKTLVIRDDRPYIGAWAVACICGESFGFGLQSVLTLYANLLGPNRKNGLASYFSDFESPTCVKRVYAKTNS  2160
AND76473.1   2081  KLCELAQIEKRTLVIRDDRPYIGAWAVACICGESFGFGLQSVLTLYANLLGPNRKNGLASYFSDFDSPTCIKKVYAKTNS  2160
ARO69857.1   2081  KLCELAQIEKRTLVIRDDRPYIGAWAVACICGESFGFGLQSVLTLYANLLGPNRKNGLASYFSDFDSPTCIKKVYAKTNS  2160
DAZ85708.1   2081  GLCKRAQVEKKTLVIRDERPYIGAWAVACICGESFGFGLQSVLTLYANLLGPNRKNGLASYFSDFESPIHVKKVHAKTNS  2160
ARO69881.1   2081  KLCELAQIEKKTLVVRDERPYIGAWAVACICGESFGFGLQSVLTLYANLLGPNQRNGLASYFSDFESPIHIKRVHAKTNS  2160
ARO69893.1   2081  KLCELAQIEKKTLVVRDERPYIGAWAVACICGESFGFGLQSVLTLYANLLGPNQRNGLASYFSDFESPIHIKRVHAKTNS  2160
ARO69897.1   2081  KLCELAQIEKKTLVVRDERPYIGAWAVACICGESFGFGLQSVLTLYANLLGPNQRNGLASYFSDFESPIHIKRVHAKTNS  2160
ARO69872.1   2081  KLCELAQIEKKTLVVRDERPYIGAWAVACICGESFGFGLQSVLTLYANLLGPNQRNGLASYFSDFESPIHIKRVHAKTNS  2160
ARO69843.1   2081  KLCELAQIEKKTLVVRDERPYIGAWAVACICGESFGFGLQSVLTLYANLLGPNQRNGLASYFSDFESPIHIKRVHAKTNS  2160
ARO69886.1   2081  KLCELAQIEKKTLVVRDERPYIGAWAVACICGESFGFGLQSVLTLYANLLGPNQRNGLASYFSDFESPIHIKRVHAKTNS  2160
ARO69864.1   2081  KLCELAQIEKKTLVVRDERPYIGAWAVACICGESFGFGLQSVLTLYANLLGPNQRNGLASYFSDFESPIHIKRVHAKTNS  2160
ARO69876.1   2081  KLCELAQIEKKTLVVRDERPYIGAWAVACICGESFGFGLQSVLTLYANLLGPNQRNGLASYFSDFESPIHIKRVHAKTNS  2160
ARO69888.1   2081  KLCELAQIEKKTLVVRDERPYIGAWAVACICGESFGFGLQSVLTLYANLLGPNQRNGLASYFSDFESPIHIKRVHAKTNS  2160
ARO69853.1   2081  KLCELAQIEKKTLVVRDERPYIGAWAVACICGESFGFGLQSVLTLYANLLGPNQRNGLASYFSDFESPIHIKRVHAKTNS  2160
ARO69903.1   2081  KLCELAQIEKKTLVVRDERPYIGAWAVACICGESFGFGLQSVLTLYANLLGPNQRNGLASYFSDFESPIHIKRVHAKTNS  2160
ARO69865.1   2081  KLCELAQIEKKTLVVRDERPYIGAWAVACICGESFGFGLQSVLTLYANLLGPNQRNGLASYFSDFESPIHIKRVHAKTNS  2160
ARO69902.1   2081  KLCELAQIEKKTLVVRDERPYIGAWAVACICGESFGFGLQSVLTLYANLLGPNQRNGLASYFSDFESPIHIKRVHAKTNS  2160
ARO69861.1   2081  KLCELAQIEKKTLVVRDERPYIGAWAVACICGESFGFGLQSVLTLYANLLGPNQRNGLASYFSDFESPIHIKRVHAKTNS  2160
ARO69850.1   2081  KLCELAQIEKKTLVVRDERPYIGAWAVACICGESFGFGLQSVLTLYANLLGPNQRNGLASYFSDFESPIHIKRVHAKTNS  2160
QYU59227.1   2081  TLCKLAQIEKKTLVIRDERPYIGAWAVACICGESFGFGLQSVLALYANLLGPNRKNGLASYFSDFESPVHIKRVHAKTNS  2160
AFM91094.1   2081  NLCKLVQDEKKTLVIRDERPYIGAWAVACICGESFGFGLQSVLTLYANLLGPNQRNGLACYFSDFESPVYIQKIHAKTNS  2160
ARO69883.1   2081  KLCELAQIEKKTLVVRDERPYIGAWAVACICGESFGFGLQSVLTLYANLLGPNQRNGLASYFSDFESPIHIKRVHAKTNS  2160
ARO69879.1   2081  KLCELAQIEKKTLVVRDERPYIGAWAVACICGESFGFGLQSVLTLYANLLGPNQRNGLASYFSDFESPIHIKRVHAKTNS  2160
AND76472.1   2081  KLCELAQIEKKTLVVRDERPYIGAWAVACICGESFGFGLQSVLTLYANLLGPNQRNGLASYFSDFESPIHIKRVHAKTNS  2160
ARO69846.1   2081  KLCELAQIEKKTLVVRDERPYIGAWAVACICGESFGFGLQSVLTLYANLLGPNQRNGLASYFSDFESPIHIKRVHAKTNS  2160
ARO69870.1   2081  KLCELAQIEKKTLVVRDERPYIGAWAVACICGESFGFGLQSVLTLYANLLGPNQRNGLASYFSDFESPIHIKRVHAKTNS  2160
ARO69856.1   2081  KLCELAQIEKKTLVVRDERPYIGAWAVACICGESFGFGLQSVLTLYANLLGPNQRNGLASYFSDFESPIHIKRVHAKTNS  2160
ARO69847.1   2081  KLCELAQIEKKTLIIRDDRPYIGAWAVACICGESFGFGLQSVLTLYANLLGPNRKNGLASYFSDFESPTCVKKVYAKTNS  2160
```

```
ARO69875.1   2081  KLCELAQIEKKTLIIRDDRPYIGAWAVACICGESFGFGLQSVLTLYANLLGPNRKNGLASYFSDFESPTCVKKVYAKTNS  2160
ARO69895.1   2081  KLCELAQIEKKTLIIRDDRPYIGAWAVACICGESFGFGLQSVLTLYANLLGPNRKNGLASYFSDFESPTCVKKVYAKTNS  2160
ARO69880.1   2081  KLCELAQIEKKTLIIRDDRPYIGAWAVACICGESFGFGLQSVLTLYANLLGPNRKNGLASYFSDFESPTCVKKVYAKTNS  2160
BCT87061.1   2081  NLCFQAQSEKKTLVIRDKRPYIGAWAVACICGESFGFGLQSVLALYANLMGPDQKNRLASYFSDFSSPIHIKKVYAATNS  2160
QYU59225.1   2081  GLCQQAILEKRTLVIRDERPYIGAWAVACICGDSFGFGLQSVLALYANLLGPDRKNGLAHYFTAFGSPTHIRKVFARTNC  2160
APD26306.1   2081  KLCHRAQLERKTLVIRDERPYIGAWAVACICGESFGFGLQSVLTLYANLLGPNQKNGLASYFSDFDNPIHIKKIHAATNS  2160


NP_619689.1  2161  YEGGEALKEIFTFCETIFYEATEMDTRKVMLQNQPDVYPSISLVGGVCFPNEGGEPGAMYSETDVTMAREVQGVYVSEAC  2240
AWO68042.1   2161  YEGGEALKEIFTFCETIFYEATEMDTRKVMLQNQPDVYPSISLVGGVCFPNEGGEPGAMYSETDVTMARQVQGVYVSEAC  2240
QYU59223.1   2161  REGGEALKEIFTFCETTLYSATDMDTRKVMIQNQPDVYPSISLVGGVCFPNEGGEPGAMYSENDMLRAKQTPGVYVSEAC  2240
ARO69887.1   2161  NEGGEALKEVFTFCETTIYSATDTDTRKVMIQNQPDIYPSISLVGGICFPTEGKEPGALFSEDDVLKAKRAPAVYVSEAC  2240
ARO69840.1   2161  NEGGEALKEVFTFCETTIYSATDTDTRKVMIHNQPDIYPSISLVGGICFPTEGKEPGALFSGDDVLKAKRAPAVYVSEAC  2240
ARO69892.1   2161  NEGGEALKEVFTFCETTIYSATDTDTRKVMIHNQPDIYPSISLVGGICFPTEGKEPGALFSGDDVLKAKRAPAVYVSEAC  2240
ARO69862.1   2161  NEGGEALKEVFTFCETTIYSATDPDTRKVMIHNQPDIYPSISLVGGICFPTEGKEPGALFSDDDVLKAKRAPAVYVSEAC  2240
ARO69844.1   2161  NEGGEALKEVFTFCETTIYSATDTDTRKVMIHNQPDIYPSISLVGGICFPTEGKEPGALFSGDDVLKAKRAPAVYVSEAC  2240
ARO69896.1   2161  NEGGEALKEVFTFCETTIYSATDTDTRKVMIHNQPDIYPSISLVGGICFPTEGKEPGALFSGDDVLKAKRAPAVYVSEAC  2240
QGP72541.1   2161  HEGGEALKEVFTFCETIFYDATSFDTRKVMVQNQPDIYPSISLVGGICFPKEGGEPGAMFSRDDVLGAEQIPGVYVSEAC  2240
ARO69901.1   2161  NEGGEALKEVFTFCETTIYSATDTDTRKVMIHNQPDIYPSISLVGGICFPTEGKEPGALFSGDDVLKAKRAPAVYVSEAC  2240
AYA22299.1   2161  REGGEALREIFRFCEITSYDATDKNTREMMVQNQPDVYPSVSLVGGICFPNEGGEPGAMYSETDVAMANQVPGVYVSEAC  2240
ARO69890.1   2161  NEGGEALKEVFTFCETTIYSATDTDTRKVMIHNQPDIYPSISLVGGICFPTEGKEPGALFSGDDVLKAKRAPAVYVSEAC  2240
ARO69859.1   2161  NEGGEALKEVFTFCETTIYSATDTDTRKVMIHNQPDIYPSISLVGGICFPTEGKEPGALFSGDDVLKAKRAPAVYVSEAC  2240
ARO69905.1   2161  NEGGEALKEVFTFCETTIYSATDTDTRKVMIHNQPDIYPSISLVGGICFPTEGKEPGALFSGDDVLKAKRAPAVYVSEAC  2240
ARO69871.1   2161  NEGGEALKEVFTFCETTIYSATDPDTRKVMIHNQPDIYPSISLVGGICFPTEGKEPGALFSDDDVLKAKRAPAVYVSEAC  2240
ARO69848.1   2161  NEGGEALKEVFTFCETTIYSATDPDTRKVMIHNQPDIYPSISLVGGICFPTEGKEPGALFSDDDVLKAKRAPAVYVSEAC  2240
ARO69874.1   2161  NEGGEALKEVFTFCETTIYSATDTDTRKVMIHNQPDIYPSISLVGGICFPTEGKEPGALFSDDDVLKAKRAPAVYVSEAC  2240
ARO69882.1   2161  NEGGEALKEVFTFCETTIYSATDPDTRKVMIHNQPDIYPSISLVGGICFPTEGKEPGALFTDDDVLKAKRAPAVYVSEAC  2240
QYU59221.1   2161  LEGGEALKEVFTFCETTFYNATEVDTRKVMIQNQPDVYPSISLVGGICFPHVGEKPGALFAGDDVIKAEQMPGVYVSEAC  2240
ARO69842.1   2161  NEGGEALKEVFTFCETTIYSATDPDTRKVMIHNQPDIYPSISLVGGICFPIEGKEPGALFSDDDVLKAKRAPAVYVSEAC  2240
AFV34731.1   2161  HEGDEALKEVFAFCENTLYSATDTDTRKVMIQNQPDAYPSISLVGGVCFPNDGGEPGALFSSDDVLKAGHTPGVYVSEAC  2240
AND76470.1   2161  NEGGEALKEVFTFCETTIYSATDPDTRKVMIHNQPDIYPSISLVGGICFPTEGKEPGALFSDDDVLKAKRAPAVYVSEAC  2240
ACZ58632.1   2161  HEGGEALKEVFNFCETIVYNATDADTRKEMLRNLPDVYPSISLVGGVCFPNEGGEPGALFSDNDALIASQTPGVYVSEAC  2240
QBZ78374.1   2161  REGSEALKEVFTFCETTLYDASDINTRKVMIQNQPEAYPSISLVGGICFPNEGEIPGALFSDEDAHRAARTQGVYVSEAC  2240
QGP72540.1   2161  REGDEALREVFTFCETTLYNANDSDTRKVMFQNQPDVFPSISLVGGVCFPNEGAKPGALFSDDDVLEVEQTPGVYVSEAC  2240
QBZ78372.1   2161  REGSEALKEVFTFCETTLYDASDINTRKVMIQNQPEAYPSISLVGGICFPNEGEIPGALFSDEDAHRAARTQGVYVSEAC  2240
QYU59224.1   2161  REGGEALKEIFPFCEITFYDATDMDTRRVMIQNQPDAYPSISLVGGICFPNEGGEPGAIYSETDVMMARQVRGVYVSEAC  2240
ARO69877.1   2161  NEGGEALKEVFTFCETTIYSATDTDTRKVMIHNQPDIYPSISLVGGICFPTEGKEPGALFSDDDVLKAKRAPAVYVSEAC  2240
ARO69858.1   2161  NEGGEALKEVFTFCETTIYSATDTDTRKVMIHNQPDIYPSISLVGGICFPTEGKEPGALFSDDDVLKAKRAPAVYVSEAC  2240
ARO69855.1   2161  NEGGEALKEVFTFCETTIYSATDTDTRKVMIHNQPDIYPSISLVGGICFPTEGKEPGALFSDDDVHKAKRAPAVYVSEAC  2240
QGP72542.1   2161  REGDEALREVFTFCETTLYNANDSDTRKVMVQNQPDVFPSVSLVGGVCFPNEGAKPGALFSDDDVLEVERTPGVYVSEAC  2240
AYQ93055.1   2161  CEGGEALKEIFTFCETTFYNATDIDTRGAMIQNQPDVYPSVSLVGGISFPKEGGEPGAMYSETDVTMAKQVQGVYVSEVC  2240
AYQ93053.1   2161  CEGGEALKEIFTFCETIFYNATDIDTRGAMIQNQPDVYPSVSLVGGISFPKEGGEPGAMYSETDVTMAKQVQGVYVSEVC  2240
AYQ93051.1   2161  REGSEALEEVFSFCETTLYSATDIDTRRVMLQNQPDAYPSISLVGGVCFPKEGSNPGALFSDVDVLKAGQISGVYVSEAC  2240
AFI71813.1   2161  HVGGKALKEVFTFCETTLYNATCMDTRKEMIRNQPDAYPSISLVGGICFPNEGGEPGAMYSETDVTMAGQVQGVYVSEAC  2240
QGP72539.1   2161  REGDEALREVFTFCETTLYNANDSDTRKVMFQNQPDVFPSISLVGGVCFPNEGAKPGALFSDDDVLEVEQTPGVYVSEAC  2240
QYU59226.1   2161  HVGGEALKEAFPFCETTFYNATDADTRKVMIQHQPDVYPSVSLVGGICFPHVGEKPGALFTDDDVLKAERVPGVYVSEAC  2240
ARO69852.1   2161  NEGGEALKEVFTFCETTIYSATDTDTRKVMIHNQPDIYPSISLVGGICFPTEGKEPGALFSGDDVLKAKRAPAVYVSEAC  2240
QGP72543.1   2161  REGGEALKEVFSFCETTLYSATDVDTRKVMIQNQLDVYPSISLVGGVCFPYEGGEPGALFSDDDALKAKQTPGVYVSEAC  2240
QYU59219.1   2161  REGSEALKEIFTFCETTLYNATNMDTRRVMIQSQPDVFPSISLVGGICFPKEGGEPGAMYSETDITMAKQVPGVYVSEVC  2240
AGT42200.1   2161  HVGGKALKEVFTFCETTLYNATCMDTRKEMIRNQPDTYPSISLVGGICFPNEGGEPGAMYSETDVTMAGQVQGVYVSEAC  2240
ACZ58633.1   2161  REGGEALKEVFTSCETTHYDATDVDTRKVMIQNQPEVYPSISLVGGICFPSVGEKPGALFVDDDLLKVERVPGVYVSEAC  2240
QBZ78369.1   2161  LEGGEALKEVFTFCETTLYDATDVDTRKVMIQNQPDAYPSVSLVGGICFPHVGEEPGALFSNDDMLKAKQTPGVYVSEVC  2240
ARO69851.1   2161  REGGEALKDVFTFCETTFYEATDIDTRREMNQNQPDVYPSVSLVGGISFPHEGDKPGALFVDDDVLRAEQIPGVYVSEAC  2240
QYU59220.1   2161  CEGGEALKEVFTFCETTIYSATDTDTRKVMNQNQPDAYPSVSLVGGICFPNIGERPGALFSDDDVLRAERIPGVFVSEAC  2240
AFV34755.1   2161  LEGGEALKEVFTFCETTFYNATDMDTRKVMVQNQPDVYPSISLVGGICFPHAGEKPGALFVDDDMLKAEQTPGVYVSEAC  2240
QGP72544.1   2161  CEGGEALKEVFTFCETTLYNATDTDTRKVMIQNQPDVYPSISLVGGICFPIEGGEPGALFSGGDVLKAEQTSGVYVSEAC  2240
QYA72352.1   2161  HEGSEALKEIFSFCETTLYTATDTDTRKVMIQNQPDIYPSISLVGGICFPNEGGEPGATYSKTDATMANQVQGVYVSEAC  2240
ARO69854.1   2161  REGGEALKDVFTFCETTFYEATDTDTRREMNQNQPDVYPSVSLVGGISFPHEGDKPGALFVDDDVLRAEQIPGVYVSEAC  2240
ARO69860.1   2161  REGGEALKDVFTFCETTFYEATDIDTRREMNQNQPDVYPSVSLVGGISFPHEGDKPGALFVDDDVLRAEQIPGVYVSEAC  2240
ARO69863.1   2161  REGGEALKDVFTFCETTFYEATDIDTRREMNQNQPDVYPSVSLVGGISFPHEGDKPGALFVDDDVLRAEQIPGVYVSEAC  2240
DAZ85707.1   2161  HGGGDALKEVFTFCETTLFDATEKDTRRVMAQNQPDIYPSISLVGGICFPKEGEEPGALFTDDDALVATQTPGVYVSEAC  2240
AWO67781.1   2161  REGGEALKECFTFCETVFNNATESDTRKEMNRNLSDAYPSISLVGGICFPYDGEKPGALFSDDDVLKAEQTPGVYVSEAC  2240
QBZ78367.1   2161  REGGEALKEVFTFCETTHYDATDVDTRKVMIQNQPEVYPSISLVGGICFPSVGEKPGALFVDDDLLKVERVPGVYVSEAC  2240
ARO69873.1   2161  REGGEALKDVFTFCETTFYEATDIDTRREMNQNQPDVYPSVSLVGGISFPHEGDKPGALFVDDDVLRAEQIPGVYVSEAC  2240
AND76471.1   2161  REGGEALKEVFTFCETTFYEATDIDTRREMNQNQPDVYPSVSLVGGISFPHEGDKPGALFVDDDVLRAEQIPGVYVSEAC  2240
ARO69849.1   2161  REGGEALKDVFTFCETTFYEATDIDTRREMNQNQPDVYPSVSLVGGISFPHEGDKPGALFVDDDVLRAEQIPGVYVSEAC  2240
ARO69889.1   2161  REGGEALKDVFTFCETTFYEATDIDTRREMNQNQPDVYPSVSLVGGISFPHEGDKPGALFVDDDVLRAEQIPGVYVSEAC  2240
ARO69867.1   2161  REGGEALKDVFTFCETTFYEATDIDTRREMNQNQPDVYPSVSLVGGISFPHEGDKPGALFVDDDVLRAEQIPGVYVSEAC  2240
ARO69904.1   2161  REGGEALKDVFTFCETTFYEATDIDTRREMNQNQPDVYPSVSLVGGISFPHEGDKPGALFVDDDVLRAEQIPGVYVSEAC  2240
ARO69869.1   2161  REGGEALKDVFTFCETTFYEATDIDTRREMNQNQPDVYPSVSLVGGISFPHEGDKPGALFVDDDVLRAEQIPGVYVSEAC  2240
ARO69884.1   2161  REGGEALKDVFTFCETTFYEATDIDTRREMNQNQPDVYPSVSLVGGISFPHEGDKPGALFVDDDVLRAEQIPGVYVSEAC  2240
ARO69878.1   2161  REGGEALKDVFTFCETTFYEATDIDTRREMNQNQPDVYPSVSLVGGISFPHEGDKPGALFVDDDVLRAEQIPGVYVSEAC  2240
ARO69885.1   2161  REGGEALKDVFTFCGTTFYEATDIDTRREMNQNQPDVYPSVSLVGGISFPHEGDKPGALFVDDDVLRAEQIPGVYVSEAC  2240
ARO69841.1   2161  REGGEALKDVFTFCETTFYEATDIDTRREMNQNQPDVYPSVSLVGGISFPHEGDKPGALFVDDDVLRAEQIPGVYVSEAC  2240
ARO69891.1   2161  REGGEALKDVFTFCETTFYEATDINTRREMNRNQPDVYPSVSLVGGVSFPHEGEKPGALFVDDDVLRAERIPGVYVSEAC  2240
ARO69868.1   2161  REGGEALKDVFTFCETTFYEATDINTRREMNRNQPDVYPSVSLVGGVSFPHEGDKPGALFVDDDVLRAERIPGVYVSEAC  2240
ARO69900.1   2161  REGGEALKDVFTFCETTFYEATDIDTRREMNQNQPDVYPSVSLVGGISFPHEGDKPGALFVDDDVLRAEQIPGVYVSEAC  2240
DAZ85706.1   2161  LEGGDALKEVFTFCETTLYDATEVDTRRVMIQNQPDVYPSISLVGGICFPKEGEGPGALFTNGDTLVAEQTPGVYVSEAC  2240
ARO69899.1   2161  REGGEALKDVFTFCETTFYEATDINTRREMNRNQPDVYPSVSLVGGVSFPHEGEKPGALFVDDDVLRAERIPGVYVSEAC  2240
ARO69898.1   2161  REGGEALKDVFTFCETTFYEATDIDTRREMNQNQPDVYPSVSLVGGISFPHEGDKPGALFVDDDVLRAEQIPGVYVSEAC  2240
AYA22297.1   2161  VEGGEALKEIFTFCEVEQYKATHRETREEVHLNQPDIFPSVSLVGGICFPREGGEPGAMYSEADVVMAKQVPGIYVSEVC  2240
QYU59222.1   2161  HEGGEALKEVFTFCETTLYNATETDTRRVMIQNQPDVYPSVSLVGGICFPSVGAEPGALFVDDDVLKAEQIPGVYVSEAC  2240
ARO69845.1   2161  REGGEALKDVFTFCETTFYEATDIDTRREMNQNQPDVYPSVSLVGGISFPHEGDKPGALFVDDDVLRAEQIPGVYVSEAC  2240
AND76473.1   2161  REGGEALKDVFTFCETTFYEATDINTRREMNRNQPDVYPSVSLVGGVSFPHEGDKPGALFVDDDVLRAERIPGVYVSEAC  2240
ARO69857.1   2161  REGGEALKDVFTFCETTFYEATDINTRREMNRNQPDVYPSVSLVGGVSFPHEGDKPGALFVDDDVLRAERIPGVYVSEAC  2240
DAZ85708.1   2161  REGGEALREIFTFCEVEQYRAIHIDTREEVHLNQPDIYPSVSLVGGICFPNVGGEPGAMYSETDVVMAKKVSGVYVSEVC  2240
ARO69881.1   2161  NEGGEALKEVFTFCETTFYSATDADTRKVMNQNQPDIYPSISLVGGICFPTEGKEPGALFSEDDVLKAKRAPAVYVSEAC  2240
ARO69893.1   2161  NEGGEALKEVFTFCETTFYSATDADTRKVMNQNQPDIYPSISLVGGICFPTEGKEPGALFSEDDVLKAKRAPAVYVSEAC  2240
```

```
ARO69897.1   2161  NEGGEALKEVFTFCETTFYSATDADTRKVMNQNQPDIYPSISLVGGICFPTEGKEPGALFSEDDVLKAKRAPAVYVSEAC  2240
ARO69872.1   2161  NEGGEALKEVFTFCETTFYSATDADTRKVMNQNQPDIYPSISLVGGICFPTEGKEPGALFSEDDVLKAKRAPAVYVSEAC  2240
ARO69843.1   2161  NEGGEALKEVFTFCETTFYSATDADTRKVMNQNQPDIYPSISLVGGICFPTEGKEPGALFSEDDVLKAKRAPAVYVSEAC  2240
ARO69886.1   2161  NEGGEALKEVFTFCETTFYSATDADTRKVMNQNQPDIYPSISLVGGICFPTEGKEPGALFSEDDVLKAKRAPAVYVSEAC  2240
ARO69864.1   2161  NEGGEALKEVFTFCETTFYSATDADTRKVMNQNQPDIYPSVSLVGGICFPTEGKEPGALFSEDDVLKAKRAPAVYVSEAC  2240
ARO69876.1   2161  NEGGEALKEVFTFCETTFYSATDADTRKVMNQNQPDIYPSISLVGGICFPTEGKEPGALFSEDDVLKAKRAPAVYVSEAC  2240
ARO69888.1   2161  NEGGEALREVFTFCETTFYSATDADTRKVMNQNQPDIYPSISLVGGICFPTEGKEPGALFSEDDVLKAKRAPAVYVSEAC  2240
ARO69853.1   2161  NEGGEALKEVFTFCETTFYSATDADTRKVMNQNQPDIYPSISLVGGICFPTEGKEPGALFSEDDVLKAKRAPAVYVSEAC  2240
ARO69903.1   2161  NEGGEALREVFTFCETTFYSATDADTRKVMNQNQPDIYPSISLVGGICFPTEGKEPGALFSEDDVLKAKRAPAVYVSEAC  2240
ARO69865.1   2161  NEGGEALREVFTFCETTFYSATDADTRKVMNQNQPDIYPSISLVGGICFPTEGKEPGALFSEDDVLKAKRAPAVYVSEAC  2240
ARO69902.1   2161  NEGGEALREVFTFCETTFYSATDADTRKVMNQNQPDIYPSISLVGGICFPTEGKEPGALFSEDDVLKAKRAPAVYVSEAC  2240
ARO69861.1   2161  NEGGEALKEVFTFCETTFYSATDADTRKVMNQNQPDIYPSISLVGGICFPTEGKEPGALFSEDDVLKAKRAPAVYVSEAC  2240
ARO69850.1   2161  NEGGEALKEVFTFCETTFYSATDADTRKVMNQNQPDIYPSISLVGGICFPTEGKEPGALFSEDDVLKAKRAPAVYVSEAC  2240
QYU59227.1   2161  REGGEALKEIFTFCETALYSATDADTRREMLQNQPDVYPSISLVGGICFPKEGEEPGALFSDDDALKAEQTPGVYVSEAC  2240
AFM91094.1   2161  REGGEALLEVFSFCETAFYEATSSDTRKEMLQNQSDIYPSISLVGGICFPKEGEKPGALFSDEDVLKAEQTPGVYVSEVC  2240
ARO69883.1   2161  NEGGEALKEVFTFCETTFYSATDADTRKVMNLNQPDIYPSISLVGGICFPTEGKEPGALFSEDDVLKAKRAPAVYVSEAC  2240
ARO69879.1   2161  NEGGEALKEVFTFCETTFYSATDADTRKVMNQNQPDIYPSISLVGGICFPTEGKEPGALFSEDDVLKAKRAPAVYVSEAC  2240
AND76472.1   2161  NEGGEALREVFTFCETTFYSATDADTRKVMNQNQPDIYPSISLVGGICFPTEGKEPGALFSEDDVLKAKRAPAVYVSEAC  2240
ARO69846.1   2161  NEGGEALKEVFTFCETTFYSATDADTRKVMNQNQPDIYPSISLVGGICFPTEGKEPGALFSEDDVLKAKRVPAVYVSEAC  2240
ARO69870.1   2161  NEGGEALKEVFTFCETTFYSATDADTRKVMNLNQPDIYPSISLVGGICFPTEGKEPGALFSEDDVLKAKRAPAVYVSEAC  2240
ARO69856.1   2161  NEGGEALKEVFTFCETTFYSATDADTRKVMNLNQPDIYPSISLVGGICFPTEGKEPGALFSEDDVLKAKRAPAVYVSEAC  2240
ARO69847.1   2161  REGGEALKDVFAFCETTFYEATDIDTRREMNQNQPDVYPSVSLVGGVSFPHEGDKPGALFVDDDVLRAEQIPGVYVSEAC  2240
ARO69875.1   2161  REGGEALKDVFAFCETTFYEATDIDTRREMNQNQPDVYPSVSLVGGVSFPHEGDKPGALFVDDDVLRAEQIPGVYVSEAC  2240
ARO69895.1   2161  REGGEALKDVFAFCETTFYEATDIDTRREMNQNQPDVYPSVSLVGGVSFPHEGDKPGALFVDDDVLRAEQIPGVYVSEAC  2240
ARO69880.1   2161  REGGEALKDVFAFCETTFYEATDIDTRREMNQNQPDVYPSVSLVGGVSFPHEGDKPGALFVDDDVLRAEQIPGVYVSEAC  2240
BCT87061.1   2161  NEGSEALKEIFTFCEVELCTAANKDTRREVFLNQPDAYPSVSLIGGVCFPVEGEEPGAMYSEADVMAKQVPGVYVSEAC  2240
QYU59225.1   2161  ESGSEALKEVFPFREIERYKATHVNTREEVFLNQPEAYPSVSLVGGICFPNEGGGPGAAYSEADVAMAPTVPGVYVSEAC  2240
APD26306.1   2161  VEGSDALREVFTFCETKLYEATDVDVRKAVHLNQPNAYPSVSLVGGISFPVEGKEPGAMFSKADVERISQTPGVYVSEAC  2240


NP_619689.1  2241  VKCCRRCVGVATRVVTDTQLFGNNLLKTHLKALRKIQNHTCLRK  2284
AWO68042.1   2241  VKCCRRCAGVATRVVTDTQLFGNNLLKTHLKALRKIQNHTCLRK  2284
QYU59223.1   2241  LKCCRSCAGVATKTVTNTQLFGNSLLKTHLKTLRRIQTHSCSRK  2284
ARO69887.1   2241  LKCCPRCVGFTAKTVSNTATFGSNLLKTHLRTLRRVENHSCSRK  2284
ARO69840.1   2241  LKCCPRCVGFATKTVSSTATFGNSLLKTHLRTLRRVENHSCSRK  2284
ARO69892.1   2241  LKCCPRCVGFATKTVSSTATFGNSLLKTHLRTLRRVENHSCSRK  2284
ARO69862.1   2241  LKCCPRCVGFATKTVSSTATFGNSLLKTHLRTLRRVENHSCSRK  2284
ARO69844.1   2241  LKCCPRCVGFATKTVSSTATFGNSLLKTHLRTLRRVENHSCSRK  2284
ARO69896.1   2241  LKCCPRCVGFATKTVSSTATFGNSLLKTHLRTLRRVENHSCSRK  2284
QGP72541.1   2241  LKCCHRCMGFTAKTVSSTAVFKNSLLKTHLKTLRRIQTHSCSRK  2284
ARO69901.1   2241  LKCCPRCVGFATKTVSSTATFGNSLLKTHLRTLRRVENHSCSRK  2284
AYA22299.1   2241  LKCCRRCVGVATKVVTRTQLFGNSLLKTHLKALRKIETHSCSRK  2284
ARO69890.1   2241  LKCCPRCVGFATKTVSSTATFGNNLLKTHLRTLRRVENHSCSRK  2284
ARO69859.1   2241  LKCCPRCVGFATKTVSSTATFGNNLLKTHLRTLRRVENHSCSRK  2284
ARO69905.1   2241  LKCCPRCVGFATKTVSSTATFGNSLLKTHLRTLRRVENHSCSRK  2284
ARO69871.1   2241  LKCCPRCVGFATKTVSSTATFGNSLLKTHLRTLRRVENHSCSRK  2284
ARO69848.1   2241  LKCCPRCVGFATKTVSSTATFGNSLLKTHLRTLRRVENHSCSRK  2284
ARO69874.1   2241  LKCCPRCVGFATKTVSSTATFGNSLLKTHLRTLRRVENHSCSRK  2284
ARO69882.1   2241  LKCCPRCVGFATKTVSSTATFGNSLLKTHLRTLRRVENHSCSRK  2284
QYU59221.1   2241  LKCCRRCIGFTTRTVSSTAVFKNSLLKTHLKTLRRVQTHSCSRK  2284
ARO69842.1   2241  LKCCPRCVGFATKTVSSTATFGNSLLKTHLRTLRRVENHSCSRK  2284
AFV34731.1   2241  LRCCSRCVGYATKTVSSTTAFKSSLLKTHLKTLRRVQTHMCSRK  2284
AND76470.1   2241  LKCCPRCVGFATKTVSSTATFGNSLLKTHLRTLRRVENHSCSRK  2284
ACZ58632.1   2241  LKCCHRCVGFTTKTVSSTVAFKNSLLKTHLKTLRRIQTHSCARR  2284
QBZ78374.1   2241  LKCCRRCMGVTSKTVSSTASFKNSLLKTHLKTLRRIQTHSCLRK  2284
QGP72540.1   2241  LKCCHRCLGVTAKTVSSTTVFKSSLLKTHLKTLRRIQTHSCLRK  2284
QBZ78372.1   2241  LKCCRRCVGVTSKTVSSTASFKNSLLKTHLKTLRRIQTHSCLRK  2284
QYU59224.1   2241  LKCCRRCVGVATRVVTNTQLFGNNLLKTHLKALRKIQNHTCLRK  2284
ARO69877.1   2241  LKCCPRCVGFATKTVSSAATFGNSLLKTHLKTLRRVENHSCSRK  2284
ARO69858.1   2241  LKCCPRCVGFATKTVSSAATFGNSLLKTHLKTLRRVENHSCSRK  2284
ARO69855.1   2241  LKCCPRCVGFATKTVSSTATFGSSLLKTHLRTLRRVENHSCSRK  2284
QGP72542.1   2241  LKCCHRCLGVTAKTVSSTTVFKNSLLKTHLKTLRRIQTHSCLRK  2284
AYQ93055.1   2241  LKCCHRCAGVATKVVTSTQLFGNNLMKTHLKALRKVQNHKCLRK  2284
AYQ93053.1   2241  LKCCQRCAGVATKVVTSTQLFGNNLMKTHLKALRKVQNHKCLRK  2284
AYQ93051.1   2241  LKCCQRCIGYTAKTVSSTNIFGNNLLKTHLKTLRRVQIHSCSRK  2284
AFI71813.1   2241  IKCCRRCAGVATKVVTNTQLFGNNLLKTHLKALRKIQNHTCLRK  2284
QGP72539.1   2241  LKCCHRCLGVTAKTVSSTTVFKSSLLKTHLKTLRRIQTHSCLRK  2284
QYU59226.1   2241  LKCCRRCVGFTTRTVSSTSIFKNSLLKTHLKTLRRVQTHSCSRK  2284
ARO69852.1   2241  LKCCPRCVGFATKTVSSTATFGNSLLKTHLRTLRRVENHSCSRK  2284
QGP72543.1   2241  LKCCKRCIGVTAKTVSGSTVFKNSLLKTHLKTLRRIQTHSCSRK  2284
QYU59219.1   2241  LKCCRRCAGVATRVVTNTQLFGNSLLKTHLKALRRVQTHSCLRK  2284
AGT42200.1   2241  LKCCRRCAGVATKVVTNTQLFGNNLLKTHLKALRKIQNHTCLRK  2284
ACZ58633.1   2241  LKCCRRCVGVTTRTVTNTALFKNSLLKTHLKTLRRIQTHSCSRK  2284
QBZ78369.1   2241  LKCCRRCVGHTAKTVSSTGNFGNNLLKTHLKTLRRIQTHSCSRK  2284
ARO69851.1   2241  LKCCRRCVGVTAKTVTKTTLFKNSLLKAHLKTLRQIQNHSCPRK  2284
QYU59220.1   2241  LKCCSRCVGFTTRTVSSTTAFKNSLLKTHLKTLRRIQIHSCSRK  2284
AFV34755.1   2241  LKCCKRCLGFTTKTVSSTAAFKNSLLKTHLKTLRRVQTHSCSRR  2284
QGP72544.1   2241  LKCCRRCVGFTTKTVSSTTTFKNSLLKTHLKTLRRIQTHSCSRK  2284
QYA72352.1   2241  LKCCRRCAGVATKVVTKTQLFGNGLLKTHLKALRRIQTHSCSRK  2284
ARO69854.1   2241  LKCCRRCVGVTAKTVTKTALFKNSLLKAHLKTLRQIQNHSCQRK  2284
ARO69860.1   2241  LKCCRRCVGVTAKTVTKTTLFKNSLLKAHLKTLRQIQNHSCPRK  2284
ARO69863.1   2241  LKCCRRCVGVTAKTVTKTTLFKNSLLKAHLKTLRQIQNHSCPRK  2284
DAZ85707.1   2241  LKCCHRCVGVAAKTVSSTSTFGNSLLKTHLKTLRRIQNHSCSRR  2284
AWO67781.1   2241  LKCCQRCVGVTAKTVSSTTFFKNSLLKTHLKTLRRIQNHSCSRK  2284
QBZ78367.1   2241  LKCCRRCVGVTTRTVTNTALFKNSLLKTHLKTLRRIQTHSCSRK  2284
ARO69873.1   2241  LKCCRRCVGVTAKTVTKTTLFKNSLLKAHLKTLRQIQNHSCPRK  2284
AND76471.1   2241  LKCCRRCVGVTAKTVTKTTLFKNSLLKAHLKTLRQIQNHSCPRK  2284
ARO69849.1   2241  LKCCRRCVGVTAKTVTKTTLFKNSLLKAHLKTLRQIQNHSCLRK  2284
```

```
ARO69889.1   2241  LKCCRRCVGVTAKTVTKTTLFKNSLLKAHLKTLRQIQNHSCQRK  2284
ARO69867.1   2241  LKCCRRCVGVTAKTVTKTTLFKNSLLKAHLKTLRQIQNHSCQRK  2284
ARO69904.1   2241  LKCCRRCVGVTAKTVTKTTLFKNSLLKAHLKTLRQIQNHSCQRK  2284
ARO69869.1   2241  LKCCRRCVGVTAKTVTKTTLFKNSLLKAHLKTLRQIQNHSCPRK  2284
ARO69884.1   2241  LKCCRRCVGVTAKTVTKTTLFKNSLLKAHLKTLRQIQNHSCQRK  2284
ARO69878.1   2241  LKCCRRCVGVTAKTVTKTTLFKNSLLKAHLKTLRQIQNHSCPRK  2284
ARO69885.1   2241  LKCCRRCVGVTAKTVTKTTLFKNSLLKAHLKTLRQIQNHSCQRK  2284
ARO69841.1   2241  LKCCRRCVGVTAKTVTKTTLFKNSLLKAHLKTLRQIQNHSCQRK  2284
ARO69891.1   2241  LKCCHRCVGVTAKTVTSTALFKNSLLKTHLKTLRQIQNHSCPRK  2284
ARO69868.1   2241  LKCCHRCVGVTAKTVTSTALFKNSLLKTHLKTLRQIQNHSCPRK  2284
ARO69900.1   2241  LKCCRRCVGVTAKTVTKTTLFKNSLLKAHLKTLRQIQNHSCPRK  2284
DAZ85706.1   2241  LKCCSRCVGFATRTVSSTSIFKNSLLKTHLKTLRRIQAHSCSRK  2284
ARO69899.1   2241  LKCCHRCVGVTAKTVTSTALFKNSLLKTHLKTLRQIQNHSCPRK  2284
ARO69898.1   2241  LKCCRRCVGVTAKTVTKTTLFKNSLLKAHLKTLRQIQNHSCQRK  2284
AYA22297.1   2241  LKCCKRCLGSTTRIVHSTQYPGMNLVRTHLKALRKVQTHSCSRK  2284
QYU59222.1   2241  LKCCQRCTGVTTRTVSSTAAFKNSLLKTHLKTLRRIQAHSCPRK  2284
ARO69845.1   2241  LKCCRRCVGVTAKTVTKTTLFKNSLLKAHLKTLRQIQNHSCQRK  2284
AND76473.1   2241  LKCCHRCVGVTAKTVTSTALFKNSLLKTHLKTLRQIQNHSCPRK  2284
ARO69857.1   2241  LKCCHRCVGVTAKTVTSTALFKNSLLKTHLKTLRQIQNHSCPRK  2284
DAZ85708.1   2241  LKCCKRCLGSTARVVHSTQYPGMNLMRTHLKALRKVQNHSCCRK  2284
ARO69881.1   2241  LKCCPRCVGFTAKTVSNTATFGSNLLKTHLRTLRRVENHSCSRK  2284
ARO69893.1   2241  LKCCPRCVGFTAKTVSNTATFGSNLLKTHLRTLRRVENHSCSRK  2284
ARO69897.1   2241  LKCCPRCVGFTAKTVSNTATFGSNLLKTHLRTLRRVENHSCSRK  2284
ARO69872.1   2241  LKCCPRCVGFTAKTVSNTATFGSNLLKTHLRTLRRVENHSCSRK  2284
ARO69843.1   2241  LKCCPRCVGFTAKTVSNTATFGSNLLKTHLRTLRRVENHSCSRK  2284
ARO69886.1   2241  LKCCPRCVGFTAKTVSNTATFGSNLLKTHLRTLRRVENHSCSRK  2284
ARO69864.1   2241  LKCCPRCVGFTAKTVSNTATFGSNLLKTHLRTLRRVENHSCSRK  2284
ARO69876.1   2241  LKCCPRCVGFTAKTVSNTATFGSNLLKTHLRTLRRVENHSCSRK  2284
ARO69888.1   2241  LKCCPRCVGFTAKTVSNTATFGSNLLKTHLRTLRRVENHSCSRK  2284
ARO69853.1   2241  LKCCPRCVGFTAKTVSNTATFGSNLLKTHLRTLRRVENHSCSRK  2284
ARO69903.1   2241  LKCCPRCVGFTAKTVSNTATFGSNLLKTHLRTLRRVENHSCSRK  2284
ARO69865.1   2241  LKCCPRCVGFTAKTVSNTATFGSNLLKTHLRTLRRVENHSCSRK  2284
ARO69902.1   2241  LKCCPRCVGFTAKTVSNTATFGSNLLKTHLRTLRRVENHSCSRK  2284
ARO69861.1   2241  LKCCPRCVGFTAKTVSNTATFGSNLLKTHLRTLRRVENHSCSRK  2284
ARO69850.1   2241  LKCCPRCVGFTAKTVSNTATFGSNLLKTHLRTLRRVENHSCSRK  2284
QYU59227.1   2241  IKCCLRCVGITAKTVSSTNTFNNSLLKTHLKTLRRIQTHSCSRK  2284
AFM91094.1   2241  LKCCHRCLGVVAKTVSSTAHFKNSLLKTHLKTLRRVQAHSCPRK  2284
ARO69883.1   2241  LKCCPRCVGFTAKTVSNTATFGSNLLKTHLRTLRRVENHSCSRK  2284
ARO69879.1   2241  LKCCPRCVGFTAKTVSNTATFGSNLLKTHLRTLRRVENHSCSRK  2284
AND76472.1   2241  LKCCPRCVGFTAKTVSNTATFGSNLLKTHLRTLRRVENHSCSRK  2284
ARO69846.1   2241  LKCCPRCVGFTAKTVSNTATFGSNLLKTHLRTLRRVENHSCSRK  2284
ARO69870.1   2241  LKCCPRCVGFTAKTVSNTATFGSNLLKTHLRTLRRVENHSCSRK  2284
ARO69856.1   2241  LKCCPRCVGFTAKTVSNTATFGSNLLKTHLRTLRRVENHSCSRK  2284
ARO69847.1   2241  LKCCHRCVGVTAKTVTKTTLFKNSLLKAHLKTLRQIQNHSCLRK  2284
ARO69875.1   2241  LKCCHRCVGVTAKTVTKTTLFKNSLLKAHLKTLRQIQNHSCLRK  2284
ARO69895.1   2241  LKCCHRCVGVTAKTVTKTTLFKNSLLKAHLKTLRQIQNHSCLRK  2284
ARO69880.1   2241  LKCCHRCVGVTAKTVTKTTLFKNSLLKAHLKTLRQIQNHSCLRK  2284
BCT87061.1   2241  LKCCSRCVGVSTKTVVNTQTFGSNLVKAHLKSLRKLQNHTCLRK  2284
QYU59225.1   2241  LKCCPRCVGVTTKVVCNTRLFGTNLLKTHLKALRRIQSHTCLRK  2284
APD26306.1   2241  LKCCLRCPGVVTKTVSSTHTFGSTFLKTHLKTLRRLQTHTCRRR  2284
```

GFLV isolates P2 polyprotein

```
NP_619706.1  1     MGKFYYSNRRLACWAAGKNPHLGGSVEQWLAAINTDPSFRQTVKEDVQENREQPTAVRMFSWKVGSGPID---NPEKC--  75
AWO68043.1   1     MGKFYYSNRRLACWAAGKNPHLGGSVEQWLAAINTDPSFRQTVKEDVQENREQPTAVRMFSWKVGSGPID---NPEKC--  75
ADJ10937.1   1     MGKFYYSNRRLACWAAGKNPHLGGSVEQWLAAINTDPSFRQTVKEDVQENREQPTAVRMFSWKVGSGPID---NPEKC--  75
ADJ10931.1   1     MGKFYYSNRRLACWAAGKNPHLGGSVEQWLAAINTDPSFRQTVKEDVQENREQPTAVRMFSWKVGSGPID---NPEKC--  75
ADJ10938.1   1     MGKFYYSNRRLACWAAGKNPHLGGSVEQWLAAINTDPSFRQTVKEDVQENREQPTAVRMFSWKVGSGPID---NPEKC--  75
QYU59229.1   1     MGKFYYSNRRLACWAAGKNPHLGGSVEQWLAAINTDSSFRQTVKEDVQENRTEPTAIRMFSYKVGFGPID---NPEKC--  75
ACZ58630.1   1     MGKFYYSNRRLACWAAGKNPHLGGSVEQWLAAINTDPSFRQTVKEDVQENREQPTAVRMFSWKIGFGPID---NPERC--  75
QGP72535.1   1     MGKFYYSNRRLACWAAGKNPHLGGSVEQWLAAINTDPSFRQTVKEDVQDNREQPTAIRMFSWKIGFGPID---NPEKC--  75
Q91HK5.1     1     MGKFYYSNRRLACWAAGKNPHLGGSVEQWLAAINTDSSFRQTVKEDVQDNREQPTAIRMFSWKVGFGPID---NPEKC--  75
ARO69912.1   1     MGKFYYSSRRLACWAAGKNPHLGGSVEQWLAAIETDPSFRQTVKEDVQLNRMRPDAIRMFSWKVGFGPID---NPERC--  75
AFI71814.1   1     MGKFYYSNRRLACWAAGKNPHLGGSVEQWLAAISTDSSFRQTVKEDVQENREQPTAVRMFSWKVGFGPID---NPEKC--  75
ADJ10932.1   1     MGKFYYSNRRLACWAAGKNPHLGGSVEQWLAAINTDSSFRQTVKEDVQENREQPTAVRMFSWKIGFGPID---NPEKC--  75
ARO69925.1   1     MGKFYYSSRRLACWAAGKNPHLGGSVEQWLAAIETDPSFRQTVKEDVQLNRMRPDAIRMFSWKVGFGPID---NPAKC--  75
ARO69911.1   1     MGKFYYSSRRLACWAAGKNPHLGGSVEQWLAAIETDPSFRQTVKEDVQLNRMRPDAIRMFSWKVGFGPID---NPEKC--  75
ACZ58628.1   1     MGKFYYSNRRLACWAAGKNPHLGGSVEQWLAAINTDPSFRQTVKEDVQENREQPTAVRMFSWKIGFGPID---NPEKC--  75
ABI63932.1   1     MGKFYFSNRRLACWAAGKNPHLGGSVEQWLAAISTDPSFRQTVKEDVQDNKLQPTAVRMFSWKVGFGPID---NPEKC--  75
ARO69945.1   1     MGKFYYSSRRLACWAAGKNPHLGGSVEQWLAAIETDPSFRQTVKEDVQLNRMRPDAIRMFSWKVGFGPID---NPEKC--  75
ARO69930.1   1     MGKFYYSSRRLACWAAGKNPHLGGSVEQWLAAIETDPSFRQTVKEDVQLNRMRPDAIRMFSWKVGFGPID---NPEKC--  75
ABI63925.1   1     MGKFYYSNRRLACWAAGKYPHLGGSVEQWLMAISTDSSFRQTVKEDVQENRDEPSAIRMFSWKLGYGPID---NPEKC--  75
ARO69906.1   1     MGKFYYSSRRLACWAAGKNPHLGGSVEQWLAAIETDPSFRQTVKEDVQLNRMRPDAIRMFSWKVGFGPID---NPEKC--  75
ACZ58627.1   1     MGKFYYSNRRLACWAAGKNPHLGGSVEQWLAAINTDPSFRQTVKEDVQENREQPTAVRMFSWKIGFGPID---NPEKC--  75
ARO69920.1   1     MGKFYYSSRRLACWAAGKNPHLGGSVEQWLAAIETDPSFRQTVKEDVQLNRMRPDAIRMFSWKVGFGPID---NPEKC--  75
ACZ58629.1   1     MGKFYYSNRRLACWAAGKNPHLGGSVEQWLAAINTDPSFRQTVKEDVQENREQPTAVRMFSWKIGFGPID---NPEKC--  75
AGT42201.1   1     MGKFYYSNRRLACWAAGKNPHLGGSVEQWLAAINTDSSFRQTVKEDVQENREQPTAVRMFSWKVGFGPID---DPEKC--  75
ARO69914.1   1     MGKFYYSSRRLACWAAGKNPHLGGSVEQWLAAIETDPSFRQTVKEDVQLNRMRPDAIRMFSWKVGFGPID---NPEKC--  75
ABI63916.1   1     MGKFYFSNRRLACWAAGKNPHLGGSVEQWLAAISTDPSFRQTVKEDVQDNKLQPTAIRMFSWKVGFGPID---NPEKC--  75
ARO69918.1   1     MGKFYYSSRRLACWAAGKNPHLGGSVEQWLAAIETDPSFRQTVKEDVQLNRMRPDAIRMFSWKVGFGPID---NPEKC--  75
ARO69948.1   1     MGKFYYSSRRLACWAAGKNPHLGGSVEQWLAAIETDPSFRQTVKEDVQLNRMRPDAIRMFSWKVGFGPID---NPEKC--  75
QYA72353.1   1     MGKFYYSNRRLACWAAGKNPHLGGSVEQWLAAINTDSSFRQTVKEDVQENREQPTAIRIFSWKVGFGPID---NPEKC--  75
ABI63917.1   1     MGKFYHSNRRLACWAAGKNPHLGGSIEQWLMAISTDSSFRQTVKEDVQDNRDEPSAIRMFSWKLGYGPID---NPEKC--  75
ARO69941.1   1     MGKFYYSSRRLACWAAGKNPHLGGSVEQWLAAIETDPSFRQTVKEDVQLNRMRPDAIRMFSWKVGFGPID---NPEKC--  75
ABI63926.1   1     MGKFYFSNRRLACWAAGKNPHLGGSVEQWLAAISTDPSFRQTVKEDVQENKLQPTAIRMFSWKVGFGPID---NPEKS--  75
CDN67471.1   1     MGKFYYSNRRLACWAAAKNPHLGGSVEQWLAAITTDSSFRQTVKEDVQENREHPVVIRMFSWKIGSGPID---NPEKN--  75
ABI63920.1   1     MGKFYYSNRRLACWAAGKNPHLGGSIEQWLMAISTDSSFRQTVKEDVQDNRDEPSAIRMFSWKLGYGPID---NPEKC--  75
ABI63927.1   1     MGKFYFSNRRLACWAAGKNPHLGGSVEQWLAAISTDPSFRQTVKEDVQENKLQPTAIRMFSWKVGFGPID---NPEKS--  75
ARO69908.1   1     MGKFYYSSRRLACWAAGKNPHLGGSVEQWLAAIETDPSFRQTVKEDVQLNRMRPDAIRMFSWKVGFGPID---NPERC--  75
ARO69916.1   1     MGKFYYSSRRLACWAAGKNPHLGGSVEQWLAAIETDPSFRQTVKEDVQLNRMRPDAIRMFSWKVGFGPID---NPEKC--  75
ARO69910.1   1     MGKFYYSSRRLACWAAGKNPHLGGSVEQWLAAIETDPSFRQTVKEDVQLNRMRPDAIRMFSWKVGFGPID---NPEKC--  75
DAZ85720.1   1     MGKFYYSNRRLACWAAGKNPHLGGSVEQWLAAIETDSSFRQTVKEDVQDNRTHPTAVRMFSWKIGFGPID---NPEKC--  75
AAX18708.1   1     MGKFYYSSRRLACWAAGKNPHLGGSVEQWLAAIETDPSFRQTVKEDVQLNRKRPDAIRMFSWKVGFGPID---NPEKC--  75
AYA22298.1   1     MGKFYYSSRRLACWAAGKNPHLGGSVEQWLAAIETDPSFRQTVKEDVQLNRKRPDAIRMFSWKVGFGPID---NPEKC--  75
ABI63939.1   1     MGKFYFSNRRLACWAAGKNPHLGGSVEQWLAAISTDPSFRQTVKEDVQDNKLQPTAVRMFSWKVGFGPLD---NPEKS--  75
ARO69926.1   1     MGKFYYSSRRLACWAAGKNPHLGGSVEQWLAAIETDPSFRQTVKEDVQLNRMRPDAIRMFSWKVGFGPID---NPEKC--  75
QGP72537.1   1     MGKFYYSNRRLACWAAGKNPHLGGSVEQWLAAINTDSSFRQTVKEDVQLNRERPDAVRMFSWKIGFGPID---NPEKC--  75
ARO69947.1   1     MGKFYYSSRRLACWAAGKNPHLGGSVEQWLAAIETDPSFRQTVKEDVQLNRMRPDAIRMFSWKVGFGPID---NPEKC--  75
ARO69921.1   1     MGKFYYSSRRLACWAAGKNPHLGGSVEQWLAAIETDPSFRQTVKEDVQLNRMRPDAIRMFSWKVGFGPID---NPEKC--  75
ARO69946.1   1     MGKFYYSSRRLACWAAGKNPHLGGSVEQWLAAIETDPSFRQTVKEDVQLNRMRPDAIRMFSWKVGFGPID---NPEKC--  75
QGP72536.1   1     MGKFYYSNRRLACWAAGKNPHLGGSVEQWLAAINTDPSFRQTVKEDVQDNREQPTAIRMFSWKIGFGPID---NPEKC--  75
QGP72538.1   1     MGKFYYSNRRLACWAAGKNPHLGGSVEQWLAAINTDSSFRQTVKEDVQLNRERPDAVRMFSWKIGFGPID---NPEKC--  75
ARO69913.1   1     MGKFYYSSRRLACWAAGKNPHLGGSVEQWLAAIETDPSFRQTVKEDVQLNRMRPDAIRMFSWKVGFGPID---NPEKC--  75
ARO69924.1   1     MGKFYYSSRRLACWAAGKNPHLGGSVEQWLAAIETDPSFRQTVKEDVQLNRMRPDAIRMFSWKVGFGPID---NPEKC--  75
ABI63924.1   1     MGKFYYSNRRLACWAAGKNPHLGGSVEQWLMAISTDSSFRQTVKEDVQENRDEPSAIRMFSWKLGYGPID---NPEKC--  75
ARO69931.1   1     MGKFYYSSRRLACWAAGKNPHLGGSVEQWLAAIETDPSFRQTVKEDVQLNRMRPDAIRMFSWKVGFGPID---NPEKC--  75
AWO67711.1   1     MGKFYYSNRRLACWAAGKNPHLGGSVEQWLAAINSDSSFRQTVKEDVQENRLEPTAIRMFSWKIGFGPID---NPEKC--  75
ARO69943.1   1     MGKFYYSSRRLACWAAGKNPHLGGSVEQWLAAIETDPSFRQTVKEDVQLNRMRPDAIRMFSWKVGFGPID---NPEKC--  75
ADJ10928.1   1     MGKFYYSNTPLACWAAGKNPHLGGSVEQWLAAINADPSFRQTVKEDVQENRERPTAVRMFSWKIGSGPID---NPEKC--  75
ARO69939.1   1     MGKFYYSSRRLACWAAGKNPHLGGSVEQWLAAIETDPSFRQTVKEDVQLNRMRPDAIRMFSWKVGFGPID---NPERC--  75
ABI63938.1   1     MGKFYFSNRRLACWAAGKNPHLGGSVEQWLAAISTDPSFRQTVKEDVQDNKLQPTAVRMFSWKVGFGPLD---NPEKS--  75
ABI63940.1   1     MGKFYFSNRRLACWAAGKNPHLGGSVEQWLAAISTDPSFRQTVKEDVQDNKLQPTAVRMFSWKVGFGPLD---NPEKS--  75
ABI63929.1   1     MGKFYFSNRRLACWAAGKNPHLGGSVEQWLTAISTDPSFRQTVKEDVQENKLQPTAIRMFSWKVGFGPID---NPEKS--  75
ABI63919.1   1     MGKFYYSNRRLACWAAGKNPHLGGSIEQWLMAINTDSSFRQTVKEDVQDNRDEPSAIWMFSWKLGYGPID---NPEKC--  75
ADJ10934.1   1     MGKFYYSNRRLACWAAGKNPHLGGSVEQWLAAISTDSSFRQTVKEDVQLNRERPDAIRMFSWKVGFGPID---NPEKC--  75
ARO69937.1   1     MGKFYYSSRRLACWAAGKNPHLGGSVEQWLAAIETDPSFRQVVKEDVQANRERPDAIRMFSWKVGSGPID---NPDNC--  75
ARO69928.1   1     MGKFYYSSRRLACWAAGKNPHLGGSVEQWLAAIETDPSFRQTVKEDVQENRKRPDAIRMFSWKVGFGPID---DPNNC--  75
ARO69927.1   1     MGKFYYSSRRLACWAAGKNPHLGGSVEQWLAAIETDPSFRQTVKEDVQLNRMRPDAIRMFSWKVGFGPID---NPERC--  75
ABI63934.1   1     MGKFYFSNRRLACWAAGKNPHLGGSVGQWLAAISTDPSFRQTVKEDVQDNKLQPTAVRMFSWKVGFGPID---NPEKC--  75
ARO69935.1   1     MGKFYYSSRRLACWAAGKNPHLGGSVEQWLAAIETDPSFRQVVKEDVQANRERPDAIRMFSWKVGSGPID---NPDNC--  75
ARO69940.1   1     MGKFYYSSRRLACWAAGKNPHLGGSVEQWLAAIETDPSFRQTVKEDVQENRKRPDAIRMFSWKVGFGPID---DPNNC--  75
ARO69933.1   1     MGKFYYSSRRLACWAAGKNPHLGGSVEQWLAAIETDPSFRQTVKEDVQENRKRPDAIRMFSWKVGFGPID---DPNNC--  75
ABI63922.1   1     MGKFYYSNRRLACWAAGKNPHLGGSIEQWLMAISTDSSFRQTVKEDVQENRNEPSAIRMFSWKLGYGPID---NPEKC--  75
ABI63936.1   1     MGKFYFSNRRLACWAAGKNPHLGGSVEQWLAAISTDPSFRQTVKEDVQVNKLQPTAVRMFSWKVGFGPID---NPEKC--  75
ABI63918.1   1     MGKFYYSNRRLACWAAGKNPHLGGSIEQWLMAISTDSSFRQTVKEDVQDNRDEPSAIRMFTWKLGYGPID---NPEKC--  75
ABI63931.1   1     MGKFYYSNRRLACWAAGKNPHLGGSIEQWLLAISTDPSFRQTVKEDVQDNRDEPSAIRMFSWKLGYGPID---NPEKC--  75
QYU59230.1   1     MGKFYYSNRRLACWAAGKNPHLGGSVEQWLAAISTDSSFRQTVKEDVQENREQPTAIRMFSWKVGFGPID---NPEKC--  75
ARO69922.1   1     MGKFYYSSRRLACWAAGKNPHLGGSVEQWLAAIETDPSFRQTVKEDVQENRKRPDAIRMFSWKVGFGPID---DPNNC--  75
ABI63941.1   1     MGKFYFSNRRLACWAAGKNPHLGGSVEQWLAAISADPSFRQTVKEDVQDNKLQPTAVRMFSWKVGFGPLD---NPEKS--  75
ARO69949.1   1     MGKFYYSSRRLACWAAGKNPHLGGSVEQWLAAIETDPSFRQVVKEDVQANRERPDAIRMFSWKVGSGPID---NPDNC--  75
ARO69917.1   1     MGKFYYSSRRLACWAAGKNPHLGGSVEQWLAAIETDPSFRQTVKEDVQENRKRPDAIRMFSWKVGFGPID---DPNNC--  75
ARO69915.1   1     MGKFYYSSRRLACWAAGKNPHLGGSVEQWLAAIETDPSFRQTVKEDVQENRKRPDAIRMFSWKVGFGPID---DPNNC--  75
QYU59228.1   1     MGKFYYSNRRLACWAAGKNPHLGGSVEQWLAAISTDSSFRQTVKEDVQLNRERPDAVRMFSWKIGFGPID---NPEKC--  75
AGN29358.1   1     MGKFYYSNRRLACWAAGKNPHLGGSVEQWLAAINTDSSFRQTVKEDVQENRLQPTAVRMFSWKVGSGPID---NPEKC--  75
ARO69944.1   1     MGKFYYSSRRLACWAAGKNPHLGGSVEQWLAAIETDPSFRQTVKEDVQENRKRPDAIRMFSWKVGFGPID---DPNNC--  75
AGN29367.1   1     MGKFYYSNRRLACWAAGKNPHLGGSIEQWLAAISTDSSFRQTVKEDVQENRDEPTAIRMFSWKIGYGPID---DPEKC--  75
ABI63921.1   1     MGKFYYSNRRLACWAAGKNPHLGGSIEQWLMAINTDSSFRQTVKEDVQDNRDEPSAIRMFSWKLGYGPID---NPEKC--  75
ABI63923.1   1     MGKFYYSNRRLACWAAGKNPHLGGSIEQWLMAISTDSSFRQTVKEDVQENRDEPSAIRMFSWKLGYGPID---NPEKC--  75
CDN67470.1   1     MGKFYYSNRRLACWAAGKNPHLGGSVEQWLAAINTDSSFRQTVKEDVQENRTEPTAIRMFSYKVGFGPID---NPEKC--  75
CDN67474.1   1     MGKFYFSNRRLACWAAGKNTHLGGSVEQWLAAINTDSSFRQTVKEDVLENRDQPTAIRMFSWKVGFGPID---NPEKC--  75
```

```
AAX18710.1   1     MGKFYYSSRRLACWAAGKNPHLGGSVEQWLAAIETDPSFRQTVKEDVQLNRMRPDAIRMFSWKVGFGPID---NPENC--  75
QBZ78370.1   1     MGKFYYSNRRLACWAAGKNPHLGGSVEQWLAAINTDSSFRQTVKEDVQLNRERPDAVRMFSWKIGFGPID---NPEKC--  75
ARO69923.1   1     MGKFYYSSRRLACWAAGKNPHLGGSVEQWLAAIETDPSFRQVVKEDVQANRERPDAIRMFSWKVGFGPID---NPDNC--  75
ARO69907.1   1     MGKFYYSSRRLACWAAGKNPHLGGSVEQWLAAIETDPSFRQTVKEDVQENRKRPDAIRMFSWKVGFGPID---DPNNC--  75
ARO69942.1   1     MGKFYYSSRRLACWAAGKNPHLGGSVEQWLAAIETDPSFRQTVKEDVQENRKRPDAIRMFSWKVGFGPID---DPNNC--  75
ARO69909.1   1     MGKFYYSSRRLACWAAGKNPHLGGSVEQWLAAIETDPSFRQTVKEDVQENRKRPDAIRMFSWKVGFGPID---DPNNC--  75
AAX18711.1   1     MGKFYYSSRRLACWAAGKNPHLGGSVEQWLAAIETDPSFRQTVKEDVQLNRERLDAIRMFSWKVGSGPID---NPEKC--  75
ARO69936.1   1     MGKFYYSSRRLACWAAGKNPHLGGSVEQWLAAIETDPSFRQTVKEDVQENRKRPDAIRMFSWKVGFGPID---DPNNC--  75
AGN29357.1   1     MGKFYYSNRRLACWAAAKNTHLGGAVEQWLAAINTDPSFRQTVKEDVQENRTRPEVIRMFSWKLGFGPID---NPEKC--  75
ARO69919.1   1     MGKFYYSSRRLACWAAGKNPHLGGSVEQWLAAIETDPSFRQTVKEDVQENRKRPDAIRMFSWKVGFGPID---DPNNC--  75
ADJ10929.1   1     MGKFYYSNRRLACWAAAKNTHLGGAVEQWLAAINTDPSFRQTVKEDVQENRARPEAIRMFSWKVGFGPID---NPEKC--  75
ABI63943.1   1     MGKFYFSNRRLACWAAGKNPHLGGSVEQWLAAISTDPSFRQTVKEDVQDNKLQPTAVRMFSWKVGFGPLD---NPEKS--  75
AGN29365.1   1     MGKFYFSNRRLACWAAGKNPHLGGSVEQWLAAIDTDPSFRQTVKEDVQENREQPTAIRMFSWKVGYGPID---NPEKC--  75
ABI63930.1   1     MGKFYYSNRRLACWAAGKNPHLGGSIEQWLLAISTDPSFRQTVKGDVQDNRDEPSAIRMFSWKLGYGPID---NPEKC--  75
ABI63942.1   1     MGKFYYSNRRLACWAAGKNPHLGGSVEQWLLAISTDPSFRQTVKEDVQDNRDEPSAIRMFSWKLGYGPID---NPEKC--  75
ADJ10939.1   1     MGKFYYSNRRLACWAAGKNPHLGGSVEQWLAAIETDSSFRQTVKEDVQENRTQPTAIRMFSWKIGFGPID---NPEKC--  75
ABI63928.1   1     MGKFYYSNRRLACWAAGKNPHLGGSVEQWLLAISTDPSFRQTVKEDVQDNRDEPSAIRMFSWKLGYGPID---NPEKC--  75
CDN67476.1   1     MGRLYFSDRRLACWAAGKNPHLGGSVEQWLAAISTDPSFRQTVKEDVQENRNEPTAIRMFSYKVGFGPID---NPEKC--  75
ABI63933.1   1     MGKFYYSNRRLACWAAGKNPHLGGSIEQWLLAISTDPSFRQTVKGDVQDNRDEPSAIRMFSWKLGYGPTD---NPEKC--  75
AAX18707.1   1     MGKFYYSSRRLACWAAGKNPHLGGSVEQWLAAIETDPSFRQTVKEDVQLNRMRPDAIRMFSWKVGFGPID---NPENC--  75
ACZ58631.1   1     MGKFYYSNRRLACWAAGKNPHLGGSVEQWLAAINSDPSFRQTVKEDVQENRTEPTAIRMFSWKIGFGPID---NPEKC--  75
AGN29359.1   1     MGKFYYSNRRLACWAAGKNPHLGGSVEQWLAAINTDSSFRQTVKEDVQENRLQPTAVRMFSWKVGFGPID---NPEKS--  75
AGN29361.1   1     MGKFYYSNRRLACWAAGKNPHLGGSVEQWLAAINTDSSFRQTVKEDVQENRLQPTAIRMFSWKVGFGPID---NPEKC--  75
ABI63935.1   1     MGKFYYSNRRLACWAAGKNPHLGGSIEQWLLAISTDPSFRQTVKEDVQDNRDEPSAIRMFSWKLGYGPID---NPEKC--  75
AGN29360.1   1     MGKFYYSNRRLACWAAGKNPHLGGSVEQWLAAINTDPSFRQTVKEDVQDNRGQPTAVRMFSWKVGFGPID---NPEKC--  75
AWO67712.1   1     MGKFYYSNRRLACWAAGKNPHLGGSVEQWLAAINSDSSFRQTVKEDVQENRLEPTAIRMFSWKVGFGPID---NPEKC--  75
AYA22300.1   1     MGKFYYSNRRLACWAAGKNPHLGGSVEQWLAAIETDSSFRQTVKEDVQLNRERPDAIRMFSWKVGFGPID---NPEKC--  75
QBZ78373.1   1     MGKFYYSNRRLACWAAGKNPHLGGSVEQWLAAINTDSSFRQTVKEDVQLNRMRPDAIRMFSWKIGFGPID---DPERC--  75
AYQ93056.1   1     MGKFYYSNRRLACWAAAKNPHLGGSVEQWLAAINTDSSFRQTVKEDVQENRTEPTAIRVFSWKIGSGPID---NPDML--  75
AYQ93054.1   1     MGKFYYSNRRLACWAAAKNPHLGGSVEQWLAAINTDSSFRQTVKEDVQENRTEPTAIRVFSWKIGSGPID---NPDKP--  75
AAX18709.1   1     MGKFYYSNRRLACWAAGKNPHLGGSVEQWLAAIETDPSFRQTVKEDVQLNRERPDAIRMFSWKVGFGPID---NPEKC--  75
AYQ93052.1   1     MGKFYYSNRRLACWAAGKNPHLGGSVEQWLAAISTDSSFRQTVKEDVQLNRERPDAVRMFSWKIGFGPID---NPERC--  75
BCT87062.1   1     MGKFYYSNRRLACWAAGKNPHLGGSVEQWVAAISTDPSFPQTVKEDVQDNRLEPTAIRMFSWTLGSGPID---NPENC--  75
AGN29364.1   1     MGKFYYSNRRLACWAAAKNTHLGGSVEQWLAAINTDSSFRQTVKEDVQENRTEPAVIRMFSWKIGSGPID---NPDKL--  75
QYU59234.1   1     MGKFYYSNRRLACWAAGKNPHLGGSVEQWLAAINTDPSFRQTVKEDVQENRMLPDAIRMFSWKVGFGPID---DPEKC--  75
CDN67475.1   1     MGKFYYSNRRLACWAAGKNPHLGGSVESWLAAINFDSSFRQTVKEDVQENRTEPTAVRMFSWKIGCGPID---NPANC--  75
AGN29356.1   1     MGKFYYSNRRLACWAAGKNPHLGGSIEQWLAAISTDSSFRQTVKEDVQENRDEPTAIRMFSWKIGSGPID---NPEKC--  75
AGN29363.1   1     MGKFYYSNRRLACWAAAKNTHLGGSVEQWLAAINTDSSFRQTVKEDVQENRTEPTAIRMFSWKIGSGPID---NPDML--  75
AFV73369.1   1     MGKFYYSNRRLACWAAGKNPHLGGSVEQWLAAINSDPSFRQTVKEDVQENRTEPSAIRMFSWKIGSGPID---NPDML--  75
AFV34732.1   1     MGKFYYSNRRLACWAAGKNPHLGGSVESWLAAINSDPSFRQTVKEDVQENRTEPSAIRMFSWKIGSGPID---NPDML--  75
AFI43912.1   1     MGKFYYSNRRLACWAAGKNPHLGGSVEQWLAAINTDPSFRQTVKEDVQENRLEPTAIRMFSWKVGSGPID---NPEKP--  75
CDN67472.1   1     MGKFHYSNRRLACWAAAKNTHLGGSVEQWLAAITTDPSFRQTVKEDVQINRARPEAVRIFSWKVGFGPVD---NPETC--  75
AGN29355.1   1     MGKFYYSNRRLACWAAGKNPHLGGSVEQWLAAISNDSSFRQTVKEDVQENRNEPTAIRMFSWRIGSGPID---NPEEC--  75
AFI43911.1   1     MGKFYYSNRRLACWAAGKNPHLGGSVEQRLAAINTDPSFRQTVKEDVQENRLEPTAIRMFSWKVGSGPID---NPEKP--  75
ADJ10940.1   1     MGKFYYSNRRLACWAAAKNTHLGGAVEQWLAAINTDPSFRQTVKEDVQENRTRPEAIRMFSWKVGFGPID---NPEKC--  75
DAZ85718.1   1     MGKFYYSNRRLACWAAGKNPHLGGSVEQWLAAINTDSSFRQTVKEDVQENREQPTAIRMFSWKIGFGPID---NPEKC--  75
ADJ10930.1   1     MGKFYYSNRRLACWAAAKNTHLGGAVEQWLAAINTDPSFRQTVKEDVQENRMRPEAIRMFSWKVGFGPID---NPEKC--  75
ADJ10933.1   1     MGKFYYSNRRLACWAAAKNTHLGGAVEQWLAAINTDPSFRQTVKEDVQENRMRPEAIRMFSWKVGFGPID---NPEKC--  75
ADJ10935.1   1     MGKFYYSNRRLACWAAAKNTHLGGAVEQWLAAINTDPSFRQTVKEDVQENRMRPEAIRMFSWKVGFGPID---NPEKC--  75
ADJ10941.1   1     MGKFYYSNRRLACWAAAKNTHLGGAVEQWLAAIITDPSFRQTVKEDVQENRTRPEVIRMFSWKVGFGPID---NPEKC--  75
DAZ85719.1   1     MGKFYYSNRRLACWAAGKNPHLGGSVEQWLAAINTDSSFRQTVKEDVQENREHPTAIRMFSWKIGFGPID---NPEKC--  75
ADJ10936.1   1     MGKFYYSNRRLACWAAAKNTHLGGAVEQWLAAINTDPSFRQTVKEDVQENRMRPEAIRMFSWKVGFGPID---NPEKC--  75
ABR09917.1   1     MGKFYYSNRRLACWAAGKNPHLGGSVEQWLAAINTDSSFRQTVKEDVQENRNEPTAIRMFSWKIGFGPID---NPEKC--  75
CDN67473.1   1     MGKFYYSNRRLAVFAQAQSRHLGGSYEQWLACVSGDSAFRAEVKARVQKDRSLERVVRMFSYPVGQGPTEcpiTPGGI--  78
QYU59232.1   1     MGKFYYSNRRLACWAAAKNTHLGGSVEQWLAAIDTDPSFRQTVKEDVQVNRTRPEVVRIFSWKLGFGPID---NPERC--  75
AGN29362.1   1     MGKFNYSNWRITRRTAGKNPHLGGSVEQWLAAIINDSSFRQTVKEDVQENRNEPTAIRMFSWRIGSGPID---NPEHC--  75
ACZ58625.1   1     MGKFYYSNRRLAAYCLGKDGR--GTYQQWLQCME-DPSFRKEVKEDVQHNRAEPSVIRVFERPLGQGPVE---GSAGI--  72
ACZ58626.1   1     MGKFYYSNRRLAAYCLGKDGR--GTYQQWLQCME-DPSFRKEVKEDVQRNRAEPSVIRVFERPLGQGPVE---GSAGI--  72
ACZ58624.1   1     MGRFYYSNRRLAAYCLGKDGR--GTYQQWLQCME-DPSFRKEVKEDVQRNRAEPSVIRVFERPLGQGPVE---GSAGI--  72
AGN29366.1   1     MGKFYYSNRRLAAYCLGKDGH-GGTYQQWLQCME-DPSFRKEVKEDVQLNRAEPSVIRVFERPIGQGPVE---GPAGI--  73
QBZ78368.1   1     MGRFYYSNRRLAAYCLGKDGR--GTYQQWLQCME-DPSFRKEVKEDVQRNRAEPSVIRVFERPLGQGPVE---GSAGI--  72
AFI43914.1   1     MGKFYYSNRRLAVFAQAQSRHLGGSYEQWLACVSGDSASRAEVKARVQKDRSLERVVRMFSYPVGQGPIE---CPITPgg  77
AFI43913.1   1     MDKFYYSNRRLAVFAQAQSRHLGGSYEQWLACVSGDSAFRAEVKARVQKDRSLERVVRMFSYPVGQGPIE---CPITPgg  77
AFV34756.1   1     ------------------------------------------------------MFSYKIGSGPID---NPDML--  17
QYU59231.1   1     MGKFYYSNRRLAAYCLGKDGR--GTYQQWLQCME-DLSFRKEVKEDVQLNRAEPSVIRIFEYPVGRGPVE---GPAGI--  72
QYU59233.1   1     MGKFYYSNRRLAVFAQAQSRHLGGSYEQWLACVSGDSAFRAEVKKRVQKDRSLERVVRMFSYPVGCGPTEcpiTPGGI--  78
APD26307.1   1     MGKFYYSNRRLAVFAQAQSRHLGGSYEQWLACVSGDSAFRAEVKARVQKDRSLERVIRMFSYPVGQGPTEcpiTPGGI--  78
QBZ78371.1         --------------------------------------------------------------------------------


NP_619706.1  76    -DWHFVLTGERPA-----PSRPVKADEVVVVPQPKKV-VIPTPPPPPAPYFRAVGAFAPTRSEFVRAIVERLTRLREESR  148
AWO68043.1   76    -DWHFVLTGERPA-Q---PSRPVKADEVVVVPQPKKV-VIPTPPPPPAPYFRAVGAFAPTRSEFVRAIVERLTRLREESR  149
ADJ10937.1   76    -DWHFVLTGERPA-Q---PSRPVKADEVVVVPQPKKV-VIPTPPPPPAPYFRAVGAFAPTRSGFVRAIVERLTRLREESR  149
ADJ10931.1   76    -DWHFVLTGERPA-Q---PSRPVKADEVVVVPQPKKV-VIPTPPPPPAPYFRAVGAFAPTRSGFVRAIVERLTRLREESR  149
ADJ10938.1   76    -DWHFVLTGERPA-Q---PSRPVKADEVVVVPQPKKV-VIPTPPPPPAPYFRAVGAFAPTRSGFVRAIVERLTRLREESR  149
QYU59229.1   76    -NWHFVLTGERPA-Q---PSRPVKADEVVVVPQPKKV-VIPSPPPPPVPYFRAVGAFAPTRSGFIRATVERLTREREESR  149
ACZ58630.1   76    -DWHFVLTGERPT-Q---PSRPVKADEVVVVPQPKKV-VIPSPPPPPAPYFRAVGAFAPTRSGFIRATVERLTREREESR  149
QGP72535.1   76    -NWHFVLTGEKPL-Q---PSQPVKADEVVVVSQPKKV-VIPTPPPPPAPYFRAVGAFAPTRSGFIRATVERLTREREESR  149
Q91HK5.1     76    -DWHFVLTGERPA-Q---PTRPVKADEVVVVPQLKKV-VIPSPPPPPAVYFRAVGAFAPTRSGFIRATVERLSREREESR  149
ARO69912.1   76    -DWHFVLTGERPA-Q---PARPVKADEVAVVPQSKKA-VIPSPPPPPAPYFRAVGAFAPTRSGFVRATVERLTREREESR  149
AFI71814.1   76    -DWHFVLTGERPA-Q---PHRPAKVDEVVVVPQSRKV-VIPSPPPSPAPYFRAVGAFAPTRSGFIRATVERLTREREESR  149
ADJ10932.1   76    -DWHFVLTGERPA-Q---PSRPVKADEVVVVPQPKKV-VTPSPPPPPVPYFRAVGAFAPTRSGFIRATVERLTREREESR  149
ARO69925.1   76    -DWHFVLTGERPA-Q---PARPVKADEVAVVPQSKKV-VIPSPPPPPAPYFRAVGAFAPTRSGFVRATVERLTREREESR  149
ARO69911.1   76    -DWHFVLTGERPA-Q---PARPVKADEVAVVPQSKKA-VIPSPPPPPAPYFRAVGAFAPTRSGFVRATVERLTREREESR  149
ACZ58628.1   76    -NWHFVLTGERPA-Q---PSRPVKADEVVVVPQPKKV-VIPSPPSPPAPYFRAVGAFAPTRSGYIRATVERLTREREESR  149
ABI63932.1   76    -DWHFVLTGERPT-Q---PSRPVKADEVVVVPQSKKV-MTPSPPPPPTPYFRAVGAFAPTRSGFVRATVERLIREREESR  149
ARO69945.1   76    -DWHFVLTGERPA-Q---PARPVKADEVAVVPQSKKA-VIPSPPPPPAPYFRAVGAFAPTRSGFVRATVERLTREREESR  149
ARO69930.1   76    -DWHFVLTGERPA-Q---PARPVKADEVAVVPQSKKV-VIPSPPPPPAPYFRAVGAFAPTRSGFVRATVERLTREREESR  149
```

ABI63925.1	76	-DWHFVLTGERPT-Q---PSRPVKADEVVVVPQSKKV-TIPSPPPPPTPYFRAVGAFAPTRSGFVRATVERLTREREESR	149
ARO69906.1	76	-DWHFVLTGERPA-Q---PARPVKADEVAVVPQSKKA-VIPSPPPPPAPYFRAVGAFAPTRSGFVRATVERLTREREESR	149
ACZ58627.1	76	-NWHFVLTGERPA-Q---PSRPVKADEVVVVPQPKKV-VIPSPPSPPAPYFRAVGAFAPTRSGYIRATVERLTREREESR	149
ARO69920.1	76	-DWHFVLTGERPA-Q---PARPVKADEVAVVPQSKKA-VIPSPPPPPAPYFRAVGAFAPTRSGFVRATVERLTREREESR	149
ACZ58629.1	76	-DWHFVLTGERPA-Q---PSRPVKADEVVVVPQPKKV-VIPSPPSPPAPYFRAVGAFAPTRSGYIRATVERLTREREESR	149
AGT42201.1	76	-DWHFVLTGERPA-Q---PRRPAKVDEVVVVPQSRKV-VIPSPPPSPAPYFRAVGAFAPTRSGFIRATVERLTREREESR	149
ARO69914.1	76	-DWHFVLTGERPA-Q---PARPVKADEVAVVPQSKKA-VIPSPPPPPAPYFRAVGAFAPTRSGFVRATVERLTREREESR	149
ABI63916.1	76	-DWHFVLTGERPT-Q---PSRPAKADEVVVVPQSKKV-MIPSPPPPPTPYFRAVGAFAPTRSGFVRATVERLIREREESR	149
ARO69918.1	76	-DWHFVLTGERPA-Q---PARPVKADEVAVVPQSKKA-VIPSPPPPPAPYFRAVGAFAPTRSGFVRATVERLTREREESR	149
ARO69948.1	76	-DWHFVLTGERPA-Q---PTRPVKADEVAVVPQSKKA-VIPSPPPPPAPYFRAVGAFAPTRSGFIRATVERLTREREESR	149
QYA72353.1	76	-DWHFVLTGERPT-Q---PTRPVKADEVVVVPQPEKV-VIPSPPPPPAVYFRAVGAFAPTRSGFIRATVKRLSQEREESR	149
ABI63917.1	76	-DWHFVLTGERPT-Q---PSRPVKADEVVVVPQSKKV-TIPSPPPPPTPYFRAVGAFAPTRSGFVRATVERLTREREESR	149
ARO69941.1	76	-DWHFVLTGERPA-Q---PARPVKADEVAVVPQSKKV-VIPSPPPPPAPYFRAVGAFAPTRSGFVRATVERLTREREESR	149
ABI63926.1	76	-DWHFVLTGERPT-Q---PSRPVKADEVVVVPQSKKV-MIPSPPPPPTPYFRAVGAFAPTRSGFVRATVERLVREREESR	149
CDN67471.1	76	-DWHFVLTGERPV-Q---PTQPVKADEVVVVPQPKKV-MIPSPPPPPTSYFRAVGAFAPTRSGFIRATVERLTREREESR	149
ABI63920.1	76	-DWHFVLTGERPT-Q---PSRPVKADEVVVVPQSKKV-TIPSPPPPPTPYFRAVGAFAPTRSGFVRATVERLTREREESR	149
ABI63927.1	76	-DWHFVLTGERPT-Q---PSRPVKADEVVVVPQSKKV-MIPSPPPPPTPYFRAVGAFAPTRSGFVRATVERLIREREESR	149
ARO69908.1	76	-DWHFVLTGERPA-Q---PARPVKADEVAVVPQSKKA-VIPSPPPPPAPYFRAVGAFAPTRSGFVRATVERLTREREESR	149
ARO69916.1	76	-DWHFVLTGERPA-Q---PARPVKADEVAVVPQSKKV-VIPSPPPPPAPYFRAVGAFAPTRSGFVRATVERLTREREESR	149
ARO69910.1	76	-DWHFVLTGERPA-Q---PARPVKADEVAVVPQSKKA-VIPSPPPPPAPYFRAVGAFAPTRSGFIRATVERLTREREESR	149
DAZ85720.1	76	-DWLFVLTGERLA-Q---PSRPVKADEVVVVPQPKKV-VIPSPPPPPAPYFRAVGAFAPTRFGFIRATVERLTREREESR	149
AAX18708.1	76	-DWHFVLTGEKPV-Q---PSRPVKADEVVVVPQPKRV-VIPSPPPPPAPYFRAVGAFAPTRSGFIRATVERLTREREESR	149
AYA22298.1	76	-DWHFVLTGEKPV-Q---PSRPVKADEVVVVPQPKRV-VIPSPPPPPAPYFRAVGAFAPTRSGFIRATVERLTREREESR	149
ABI63939.1	76	-DWHFVLTGERPT-Q---PSRPVKADEVVVVPQSKKV-MIPSPPPPPTPYFRAVGAFAPTRSGFVRATVERLIREREESR	149
ARO69926.1	76	-DWHFVLTGERPA-Q---PARPVKADEVAVVPQSKKA-VIPSPPPPPAPYFRAVGAFAPTRSGFIRATVERLTREREESR	149
QGP72537.1	76	-DWHFVLTGERPS-Q---PTRPVKADEVVVVPQPKKV-VIPSPPSPPAPYFRAVGAFAPTRSGFIRATVERLTQEREESR	149
ARO69947.1	76	-DWHFVLTGERPA-Q---PTRPVKADEVAVVPQSKKA-VIPSPPPPPAPYFRAVGAFAPTRSGFIRATVERLTREREESR	149
ARO69921.1	76	-DWHFVLTGERPA-Q---PTRPVKADEVAVVPQSKKA-VIPSPPPPPAPYFRAVGAFAPTRSGFVRATVERLTREREESR	149
ARO69946.1	76	-DWHFVLTGERPA-Q---PARPVKADEVAVVPQSKKV-VIPSPPPPPAPYFRAVGAFAPTRSGFVRATVERLTREREESR	149
QGP72536.1	76	-NWHFVLTGERPL-Q---PSQPVKADEVVVVPQPKKV-VIPTPPPPPAPYFRAVGAFAPTRSGFIRATVERLTREREESR	149
QGP72538.1	76	-DWHFVLTGERPS-Q---PTRPVKADEVVVVPQPKKV-VIPSPPSLPAPYFRAVGAFAPTRSGFIRATVERLTQEREESR	149
ARO69913.1	76	-DWHFVLTGERPA-Q---PARPVKADEVAVVPQSKKA-VIPSPPPPPAPYFRAVGAFAPTRSGFIRATVERLTREREESR	149
ARO69924.1	76	-DWHFVLTGERPA-Q---PARPVKADEVAVVPQSKKA-VIPSPPPPPAPYFRAVGAFAPTRSGFIRATVERLTREREESR	149
ABI63924.1	76	-DWHFVLTGERPT-Q---PSRPVKADEVVVVPQSKKV-TIPSPPPPPTPYFRAVGAFAPTRSGFVRATVERLTREREESR	149
ARO69931.1	76	-DWHFVLTGERPA-Q---PARPVKADEVAVVPQSKKV-VIPSPPPPPAPYFRAVGAFAPTRSGFVRATVERLTREREESR	149
AWO67711.1	76	-NWDFVLTGKRPG-Q---PSEPVKAQEV-VVQQPRKV-VIPSPPPSPAPYFRAVGAFAPTRSGFIRATVERLTREREESR	148
ARO69943.1	76	-DWHFVLTGERPA-Q---PTRPVKADEVAVVPQSKKA-VIPSPPPPPAPYFRAVGAFAPTRSGFIRATVERLTREREESR	149
ADJ10928.1	76	-NWHFVFTGERPA-Q---PSRPAKVDEVVVVPQPKKV-VIPAPPPPPAPYFRAVGAFAPTRSGFIRATVERLTREREESR	149
ARO69939.1	76	-DWHFVLTGERPA-Q---PARPVKADEVAVVPQSKKV-VIPSPPPPPAPYFRAVGAFAPTRSGFVRATVERLTREREESR	149
ABI63938.1	76	-DWHFVLTGERPT-Q---PSRPVKADEVVVVPQSKKV-MIPSPPPPPTPYFRAVGAFAPTRSGFVRATVERLIREREESR	149
ABI63940.1	76	-DWHFVLTGERPT-Q---PSRPVKADEVVVAPQSKKV-MIPSPPPPPTPYFRAVGAFAPTRSGFVRATVERLIREREESR	149
ABI63929.1	76	-DWHFVLTGERPT-Q---PSRPVKADEVVVVPQSKKV-MIPSPPPPPTPYFRAVGAFAPTRSGFVRATVERLIREREESR	149
ABI63919.1	76	-DWHFVLTGERPT-Q---PSRPVKADEVVVVPQSKKV-TIPSPPPPPTPYFRAVGAFAPTRSGFVRATVERLTREREESR	149
ADJ10934.1	76	-DWHFVLTGERPS-Q---PTEPVKAQEVEVPPV--KV-VIPSPPPVLRPYFRPIGAFAPTRSGFIRATVERLTREREESR	147
ARO69937.1	76	-NWHFVLTGERPV-Q---PTRPVKADEVVVVPQPKKV-VIPSPPPPPAPYFRAVGAFAPTRSGFIRATVERLTREREESR	149
ARO69928.1	76	-NWHFVLTGERPA-Q---PTRPVKADEVVVVPQPKKV-VIPSPPPPPAPYFRAVGAFAPTRSGFIRATVERLTREREESR	149
ARO69927.1	76	-DWHFVLTGERPA-Q---PARPVKADEVAVVPQSKKV-VIPSPPPPPAPYFRAVGAFAPTRSGFVRATVERLTREREESR	149
ABI63934.1	76	-DWHFVLTGERPT-Q---PSRPVKADEVVVVPQSKKV-MIPSP-PPPTPYFRAVGAFAPTRSGFVRATVERLIREREESR	148
ARO69935.1	76	-NWHFVLTGERPV-Q---PTRPVKADEVVVVPQPKKV-VIPSPPPPPAPYFRAVGAFAPTRSGFIRATVERLTREREESR	149
ARO69940.1	76	-NWHFVLTGERPA-Q---PTRPVKADEVVVVPQPKKV-VIPSPPPPPAPYFRAVGAFAPTRSGFIRATVERLTREREESR	149
ARO69933.1	76	-NWHFVLTGEKPA-Q---PTRPVKADEVVVVPQPKKV-VIPSPPPPPAPYFRAVGAFAPTRSGFIRATVERLTREREESR	149
ABI63922.1	76	-DWHFVLTGERPT-Q---PSRPVKADEVVVVPQSKKV-TIPSPPPPPTPYFRAVGAFAPTRSGFVRATVERLTREREESR	149
ABI63936.1	76	-DWHFVLTGERPT-Q---PSRPVKADEVVVVPQSKKV-MIPSPPPPPTPYFRAVGAFASTRSGFVRATVERLIREREESR	149
ABI63918.1	76	-DWHFVLTGERPT-Q---PSRPVKADEVVVVPQSKKV-TIPSPPPPPTPYFRAVGAFAPTRSGFVRATVERLTREREESR	149
ABI63931.1	76	-DWHFVLTGERPQ-Q---PTEPVKAHEV-VVPQPKKV-VIPSPPPPPTPYFRAVGAFAPTRSGFIRATVERLTREREESR	148
QYU59230.1	76	-NWHYVLTGERPA-Q---PTQPVKADEVVVVPQPKKM-VIPSPPPPPASYFRAVGAFAPTRSGFVRATVERLTREREESR	149
ARO69922.1	76	-NWQFVLTGERPA-Q---PTRPVKADEVVVVPQPKKV-VIPSPPPPPAPYFRAVGAFAPTRSGFIRATVERLTREREESR	149
ABI63941.1	76	-DWHFVLTGERPT-Q---PSRPVKADEVVVVPQSKKV-MIPSPPPPPTPYFRAVGAFAPTRSGFVRATVERLIREREESR	149
ARO69949.1	76	-NWHFVLTGERSV-Q---PTRPVKADEVVVVPQPKKV-VIPSPPPPPAPYFRVVGAFAPTRSGFIRATVERLTREREESR	149
ARO69917.1	76	-NWHFVLTGEKPA-Q---PTRPVKADEVVVVPQPKKV-VIPSPPPPPAPYFRAVGAFAPTRSGFIRATVERLTREREESR	149
ARO69915.1	76	-NWHFVLTGERPA-Q---PTRPVKADEVVVVPQPKKV-VIPSPPPPPAPYFRAVGAFAPTRSGFIRATVERLTREREESR	149
QYU59228.1	76	-DWHFVLTGERPS-Q---PAGPVKAQEVGGEVPPVKV-VIPSPPPFLRPYFRAVGAFAPTRSGFIRATVERLTREREESR	149
AGN29358.1	76	-DWHFVLTGERPV-Q---PTRPVKADEVVVVPQPKKV-VIPTPPPPPAPYFRAVGAFAPTRSGFIRATVERLTREREESR	149
ARO69944.1	76	-NWHFVLTGERSA-Q---PTRPVKADEVVVVPQPKKV-VIPSPPPPPAPYFRAVGAFAPTRSGFIRATVERLTREREESR	149
AGN29367.1	76	-NWHFVLTGERPA-Q---PTEPVEAQEVVVPPVK----VIPSPPPVPGPYFRPIGAFAPTRSGFIRATVERLTREREESR	146
ABI63921.1	76	-DWHFVLTGERPT-Q---PSRPVKADEVVVVPQSKKV-TIPSPPPPPTPYFRAVGAFAPTRSGFVRATVERLTRERDESR	149
ABI63923.1	76	-DWHFVLTGERPT-Q---PSRPVKADEVVVVPQSKKV-TIPSPPPPPTPYFRAVGAFAPTRSGFVRATVERLTREREESR	149
CDN67470.1	76	-NWHFVLTGEGPA-Q---PTEPVRAQEVVVPPVK----VIPSPPPMPRSYFRPVGAFAPTRSGFIRATVERLTREREESR	146
CDN67474.1	76	-NWHFVITGERPA-Q---PTQPAKADEVVVVPQSKKV-VIPLPPPPPAVYFRAVGAFAPTRSGFIRATVERLTREREESR	149
AAX18710.1	76	-NWHFVLTGERPA-Q---PTRPVKADEVVVVPQPKKV-VIPSPPSPPAPYFRAVGAFAPTRSGFIRATVERLTQEREESR	149
QBZ78370.1	76	-DWHFVLTGERPS-Q---PTEPVKAQEVEV--PPVKV-VVPSPPPVPGPYFRPIGAFAPTRSGFIRATVERLTREREESR	147
ARO69923.1	76	-NWHFVLTGERSV-Q---PTRPVKADEVVVVPQPKKV-VIPSPPPPPAPYFRAVGAFAPTRSGFIRATVERLTREREESR	149
ARO69907.1	76	-NWHFVLTGERLA-Q---PTRPVKADEVVVVPQPKKV-VIPSPPPPPAPYFRAVGAFAPTRSGFIRATVERLTREREESR	149
ARO69942.1	76	-NWHFVLTGERLA-Q---PTRPVKADEVVVVPQPKKV-VIPSPPPPPAPYFRAVGAFAPTRSGFIRATVERLTREREESR	149
ARO69909.1	76	-NWHFVLTGEKPA-Q---PTRPVKADEVVVVPQPKKV-VIPSPPPPPAPYFRAVGAFAPTRSGFIRATVERLTREREESR	149
AAX18711.1	76	-DWHFVLTGERPA-Q---PIRPVKADEVVVVPQPKKV-VIPPPPSPPAPYFRAVGAFAPTRSGFVRATVERLTREREESR	149
ARO69936.1	76	-NWHFVLTGERLA-Q---PTRPVKADEVVVVPQPKKV-VIPSPPPPPAPYFRAVGAFAPTRSGFIRATVERLTREREESR	149
AGN29357.1	76	-NWHFVLTGERPE-Q---PTRPVKADEVVVAPQLKKV-VIPSPPPPPAPYFRAVGAFAPTRSGFIRATVERLSREREESR	149
ARO69919.1	76	-NWHFVLTGERLA-Q---PTRPVKADEVVVVPQPKKV-VIPSPPPPPAPYFRAVGAFAPTRSGFIRATVERLTREREESR	149
ADJ10929.1	76	-NWHFVLTGERPE-Q---PTRPVKADEVVVVPQPKKV-VIPSPPPPPAPYFRAAGAFAPTRSGFIRATVERLTREREESR	149
ABI63943.1	76	-DWHFVLTGERPT-Q---PSRPVKADEVVVVPQSKKV-MIPSPSPPPTPYFRAVGAFAPTRSGFVRATVERLIREREESR	149
AGN29365.1	76	-DWHFVLTGERPS-Q---PNRPAKASEVVVVPQPKKA-VIPSPPPVPTPYFKAVGAFAPTRSGFIRATVERLSREREELR	149
ABI63930.1	76	-DWHFVLTGERPQ-Q---PTEPVKAHEV-VVPQPKKV-VIPSPPPPPTPYFRAVGAFAPTRSGFIRATVERLTREREESR	148
ABI63942.1	76	-DWHFVLTGERPQ-Q---PTEPVKAHEV-VVPQPKKV-VIPSPPPPPTPYFRAVGAFAPTRSGFIRATVERLTREREESR	148
ADJ10939.1	76	-DWRFVLTGKGPL-Q---PTQPVKADEVVVVPQPKKV-VIPSPPPPPAAYFRAVGAFAPTRSGFIRATVERLTREREESR	149
ABI63928.1	76	-DWHFVLTGERPQ-Q---PTEPVKAHEV-VVPQPKKV-VIPSPPPPPTPYFRAVGAFAPTRSGFIRATVERLTREREESR	148
CDN67476.1	76	-NWHFVLTGERPA-Q---PTEPVKAQEVVVPPVK----VIPSPPPMPRSYFRPIGAFAPTRSGYIRATVERLTREREELR	146
ABI63933.1	76	-DWHFVLTGERPQ-Q---PTEPVKAHEV-VVPQPKKV-VIPSPPPPPTPYFRAVGAFAPTRSGFIRATVERLTREREESR	148

```
AAX18707.1   76    -NWHLVLTGERPA-Q---PTRPVKADEVVVVPQPKKV-VIPSPPSPPAPYFRAVGAFAPTRSGFIRATVERLTQEREESR  149
ACZ58631.1   76    -NWHFVLTGERPL-Q---PTEPAKAQEVEV--PPVKA-VVPSPPPVPRPYFRPIGAFAPTRSGFIRATVERLTREREESR  147
AGN29359.1   76    -DWHFVLTGERPV-Q---PTRPVKADEIVVVPQSKKV-VIPTPPPPPAPYFRAVGAFAPTRSGFIRATVERLTREREESR  149
AGN29361.1   76    -DWHFVLTGERPV-Q---PTRPVKADEVVVVPQSKKV-VIPTPPPPPAPYFRAVGAFAPTRSGFIRATVERLTREREESR  149
ABI63935.1   76    -DWHFVLTGERPQ-Q---PTEPVKVHEV-VVPQPKKV-VIPSPPPPPTPYFRAVGAFAPTRSGFIRATVGRLTREREESG  148
AGN29360.1   76    -DWHYVFTGKVST-Q---PAQPVKADEVMAVPHPKKGwLIPSPPPPPAPYFRAVGAFAPTRSGFIRATVERLSREREESR  150
AWO67712.1   76    -NWDFVLTGKRPG-Q---PSEPVKAQEV-VAPQPKKV-VIPSPPPLPAPYFRVVGAFAPTRSGFIRATVERLTREREESR  148
AYA22300.1   76    -NWHFVLTGEMPT-Q---PSEPVKA-QVVVVPPVK---VIPSPPPMPRPYFRPIGAFAPTRSGFIRATVERLSREREESR  146
QBZ78373.1   76    -NWHFVLTGERPA-Q---PNEPAKAQEVEVPPVK----VIPSPPPMPRSYFRPVGAFAPTRSGFIRASVERLTREREESR  146
AYQ93056.1   76    -DWQFVLTGKVSR-Q---PTEPVKAQEVVVPPVK----VIPSPPPMPRSYFRPIGAFAPTRSGFIRATVERLTREREESR  146
AYQ93054.1   76    -DWQFVLTGKVSR-Q---PTEPVKAQEVVVPPVK----VIPSPPPMPRSYFRPIGAFAPTRSGFIRATVERLTREREESR  146
AAX18709.1   76    -NWHFVLTGERPT-Q---PSEPVKA-QVVVVPPVK---VIPSPPPMPRPYFRPIGAFAPTRSGFIRATVERLSREREGSR  146
AYQ93052.1   76    -DWHFVLTGERPS-Q---PTEPADAQEVEV--PPVKV-VIPSPPPVPRPYFRPVGAFAPTRSGFIRATVERLSLEREESR  147
BCT87062.1   76    -DWRYVLTGVPT--R---PSQPVKADEVVVVPQPKKV-VIPSPPPPPAPYFRAVGAFAPTRSGFIRATVERLTREREESR  148
AGN29364.1   76    -DWQFVLTGKVSR-Q---PTEPAKAQEVVVPPVK----VIPSPPPMPRSYFRPIGAFAPTRSGFIRATVERLTRERGESR  146
QYU59234.1   76    -NWHFVLTGERPV-Q---PTGLVKVREVEVPPV--KV--IPSPPPEPRPYFRAVGAFAPTRSGFIRATVERLTREREELR  146
CDN67475.1   76    -NWHFVLTGERPT-Q---PTEPVKAQEVVVLPVR----VIPPPPPMPRPYFRAVGAFAPTRSGFVRATVERLTREREELR  146
AGN29356.1   76    -NWHFVLTGERPA-Q---PIEPVEAQEVVVPPVK----VIPSPPPVPGPYFRPIGAFAPTRSGFIRATVERLTREREESR  146
AGN29363.1   76    -DWQFVLTGKVSR-Q---PTEPAKAQEVVVPPVK----VIPSPPPMPRSYFRPIGAFAPTRSGFIRATVERLTREREESR  146
AFV73369.1   76    -DWQFVLTGKVSR-Q---PTGPAKAQEVVVPPQPQKV-EIPSPPPPPAPYFRPIGAFAPTRSGFIRATVERLTREREESR  149
AFV34732.1   76    -DWQFVLTGKVSR-Q---PTGPAKAQEVVVPPV--KV--IPSPPPVPGPYFRPIGAFAPTRSGFIRATVERLTREREESR  146
AFI43912.1   76    -DWRYVLTGIPA--G---PSQPVKADEVVVVSQPKKK-VIPPPPSPPAPYFRAVGAFAPTRSGFIRATVERLTREREESR  148
CDN67472.1   76    -NWHFVLTGERPA-Q---PTGPVKAQEVVVPPV--KV--IPSPPPVPRPYFRPIGAFAPTRSGFIRATVERLSREREESR  146
AGN29355.1   76    -NWHFVLTGERPA-Q---PTEPVEAQEVVVPPVK----VIPSPPPVPGPYFRSIGAFAPTRSGFIRATVERLTREREESR  146
AFI43911.1   76    -DWRYVLTGIPA--G---PSQPVKADEVVVVSQPKKK-VIPPPPSPPAPYFRAVGAFAPTRSGFIRATVERLTREREESR  148
ADJ10940.1   76    -NWHFVLTGERPE-Q---PIRPVKADEVVVVPQPKKV-VIPSPPPPPAPYFRAAGAFAPTRSGFIRATVERLTRERDESR  149
DAZ85718.1   76    -DWHFVITGERPT-Q---PSRPAKVDEVVVAPQPKKV-VIPSPPSPPTPYFRAVGAFAPTRSGFIRATVERLTREREESR  149
ADJ10930.1   76    -NWHFVLTGERPE-Q---PTRPVKADEVVVVPQPKKV-VIPSPPPPPAPYFRAAGAFAPTRSGFIRATVERLTREREESR  149
ADJ10933.1   76    -NWHFVLTGERPE-Q---PTRPVKADEVVVVPQPKKV-VIPSPPPPPAPYFRAAGAFAPTRPGFIRATVERLAREREESR  149
ADJ10935.1   76    -NWHFVLTGERPE-Q---PTRPVKADEVVVVPQPKKV-VIPSPPPPPAPYFRAAGAFAPTWSGFIRATVERLTREREESR  149
ADJ10941.1   76    -DWHFVLTGERPE-Q---PTRPVKADEVVVVPQPKKV-VIPSPPPPPAPYFRAAGAFAPTRSGFIRATVERLAREREESR  149
DAZ85719.1   76    -NWHFVLTGERPT-Q---PSRPAKVDEVVVVPQPKKV-VIPSPPSPPTPYFRAVGAFAPTRSGFIRATVERLTREREESR  149
ADJ10936.1   76    -NWHFVLTGERPE-Q---PTRPVKADEVVVVPQPKKV-VIPSPPPPPAPYFRTAGAFAPTRSGFIRATVERLTREREESR  149
ABR09917.1   76    -DWHFVLTGERPM-Q---PTEPVKAHEVVVVPQPKRV-VIPSPPPPPTPYFRAVGAFAPTRSGFIRATVERLSQEREESR  149
CDN67473.1   79    -NWEFITTGTTPT-SrvvPTEPVKAHEVVAL-QPKKK-VVPPPPPPPLPYFRAVGAFAPTRSGFIRATVERLSREREESR  154
QYU59232.1   76    -NWHFVLTGERPA----qPTEPVKAQEVVVPPVK----VIPSPPPMPRPYFRPIGAFAPTRSGFIRATVERLTREREESR  146
AGN29362.1   76    -NWHFVLTGERPT-Q---PTQPVESHEVVVPPV--KV--IPSPPPVPGPYFRSIGAFAPTWSGFIRATVDRLTRQREESR  146
ACZ58625.1   73    -AWHFIVHGPSLG-L-vpSPRPTKADEVVVVPQPKKV-VIPSPPPPPVPYFRVIGAFAPTRSGFIRATVERLTREREESR  148
ACZ58626.1   73    -AWHFIVHGPSLG-L-vpSPRPTKADEVVVVPQPKKV-VIPSPPPPPVPYFRAIGAFAPTRSGFIRATVERLTREREESR  148
ACZ58624.1   73    -AWHFIVHGPSLG-L-vpPPRPAKADEVVVVPQPKKV-VIPPPPPSPVPYFRAVGAFAPTRSGFIRATVERLTREREESR  148
AGN29366.1   74    -AWHYIVHGPSLG--lvpPPRPVKADEVVVVPQLKKV-VIPLPPPPPAPYFRAVGAFAPTRSGFIRATVERLIQERDELR  149
QBZ78368.1   73    -AWHFIVHGPSLG-L-vpSPRPTKADEVVVVPQPKKV-VIPSPPPPPVPYFRVVGAFAPTRSGFIRATVERLIREREESR  148
AFI43914.1   78    iNWEFITTGTTPT-SrvvPTEPVKAHEVVVVPQPKKA-AIPSPPPPPKPYSRAVGAFAPTRSGFIRATVERLTREREESR  155
AFI43913.1   78    iNWEFITTGTTPT-SrvvLTEPVKAHEVVVVPQPKKA-AIPSPPPPPKPYFRAVGAFAPTRSGFIRATVERLTREREESR  155
AFV34756.1   18    -DWQFVLTGKVSR-R---PPQPVKADEVVVVPHP----VIPSPPPVPGPYFRPVGAFAPTRSGFIRATVERLSREREESR  88
QYU59231.1   73    -AWHYIVHG--PAlGlvpPPRPVKADEVVVVPQPRKV-VIPPPPPPPTPYFRAVGAFAPTRSGFIRATVERLTREREESR  148
QYU59233.1   79    -NWEFITTGTTPT-ShvaPTGPAKVHEVVVVPQPKKV-VIPSPPSPPKPYYRAIGAFAPTRSGFIRATVERLTREREEAR  155
APD26307.1   79    -NWEFITTGTTPT-SrvvPTGPAKVHEVVVVPQPKKV-VIPSPPPPPAPYFRAVGAFAPTRSGFIRATVERLTREREESR  155
QBZ78371.1         --------------------------------------------------------------------------------


NP_619706.1  149   AAALFAELPLEYPQGAPLKLSLAAKFAMLKHTTWRKWYDTSDERLLEAHPGGPCLPPP-PP-IQ-NP-PSFQERVREFCR  224
AWO68043.1   150   AAALFAELPLEYPQGAPLKLSLAAKFAMLKHTTWRKWYDTSDERLLEAHPGGPCLPPP-PP-IQ-NP-PSFQERVREFCR  225
ADJ10937.1   150   AAALFAELPLEYPQGAPLKLSLAAKFAMLKHTTWRKWYDTSDERLLEAHPGGPCLPPP-PP-IQ-NP-PSFQERVREFCR  225
ADJ10931.1   150   AAALFAELPLEYPQGAPLKLSLAAKFAMLKHTTWRKWYDTRDERLLEAHPGGPCLPPP-PP-IQ-NP-PSFQERVREFCR  225
ADJ10938.1   150   AAALFAELPLEYPQGAPLKLSLAAKFAMLKHTTWRKWYDTSDERLLEAHPGGPCLPPP-PP-IQ-NP-PSFQERVREFCR  225
QYU59229.1   150   AAALFAELPLEYPQGAPLKLSLAMKFAMLKHTTWKKWYDTSDERLLVAHPGGPCLPPP-PP-IQ-SP-PSFQEKVREFCR  225
ACZ58630.1   150   AAALFAELPLEYPQGNPLKLSLAMRFAMLKHTTWRKWYDASDERLLKAHPGGPCLPPP-PP-VQ-SP-PTFRERVREYCR  225
QGP72535.1   150   AAALFAELPLEYPQGAPLKLSLAMKFAMLKHTTWRKWYDTSDERLLVAHPGGPCLPPP-PP-IQ-SP-PSFQERVREFCR  225
Q91HK5.1     150   AAALFAELPLEYPQGAPLKLSLAMKFAMLKHTTWRKWYDTSDERLSEAHPGGPCLPPP-PP-IQ-NP-PFFQERVREFCR  225
ARO69912.1   150   AAALFAELPLEYPQGTPLKLSLAMKFAMLKHTTWRKWYDTSDERLLEAHPGGPRLPPP-LP-IQ-SP-PSFQERVREFCR  225
AFI71814.1   150   AAALFAELPLEYPQGAPLKLSLAMKFAMLKHTTWRKWYDASDERLSEAHPGGPCLPPP-PP-IQ-SP-PSFRERVREFCR  225
ADJ10932.1   150   AAALFAELPLEYPQGAPLKLSLAAKFAMLKHTTWRKWYDTSDERLLEAHPGGPCLPPP-PP-IQ-NP-PSFQERVREFCR  225
ARO69925.1   150   AAALFAELPLEYPQGTPLKLSLAMKFAMLKHTTWRKWYDTSDERLLEAHPGGPCLPPP-LL-IQ-SP-PSFQERVREFCR  225
ARO69911.1   150   AAALFAELPLEYPQGTPLKLSLAMKFAMLKHTTWRKWYDTSDERLLQAHPGGPRLPPP-LP-IQ-SP-PSFQERVREFCR  225
ACZ58628.1   150   AAALFAELPLEYPQGSPLKLSMAMKFAMVKHTTWRKWYDTSDERLLAAHPGGPCLPPP-PP-IQ-SP-PSFYEKVREFCR  225
ABI63932.1   150   AAALFAELPLEYPQGAPLKLSLAMKFAMLKHTTWRKWYDTSDERLLKAHPGGPCLPSP-PP-IQ-SP-PSFQERVREFCR  225
ARO69945.1   150   AAALFAELPLEYPQGTPLKLSLAMKFAMLKHTTWRKWYDTSDERLLQAHPGGPRLPPP-LP-IQ-SP-PSFQERVREFCR  225
ARO69930.1   150   AAALFAELPLEYPQGTPLKLSLAMKFAMLKHTTWRKWYDTSDERLLEAHPGGPRLPPP-LP-IQ-SP-PSFQERVREFCR  225
ABI63925.1   150   AAALFAELPLEYPQGAPLKLSLAMKFAMLKHTTWRKWYDTSDERLLKAHPGGPCFPPP-PP-IQ-SP-PSFQERVREFCR  225
ARO69906.1   150   AAALFAELPLEYPQGTPLKLSLAMKFAMLKHTTWRKWYDTSDERLLEAHPGGPCLPPP-PL-IQ-SP-PSFQERVREFCR  225
ACZ58627.1   150   AAALFAELPLEYPQGSPLKLSMAMKFAMVKHTTWRKWYDTSDERLLAAHPGGPCLPPP-PP-IQ-SP-PSFCEKVREFCR  225
ARO69920.1   150   AAALFAELPLEYPQGTPLKLSLAMKFAMLKHTTWRKWYDTSDERLLQAHPGGPRLPPP-LP-IQ-SP-PSFQERVREFCR  225
ACZ58629.1   150   AAALFAELPLEYPQGSPLKLSMAMKFAMVKHTTWRKWYDTSDERLLAAHPGGPCLPPP-PP-IQ-SP-PSFYEKVGEFCR  225
AGT42201.1   150   AAALFAELPLEYPQGAPLKLSLAMKFAMLKHTTWRKWYDASDERLLEAHPGGPCLPPP-PP-IQ-SP-PSFRERVREFCR  225
ARO69914.1   150   AAALFAELPLEYPQGTPLKLSLAMKFAMLKHTTWRKWYDTSDERLLQAHPGGPHLPPP-LP-IQ-SP-PSFQERVREFCR  225
ABI63916.1   150   AAALFAELPLEYPQGAPLKLSLAMKFAMLKHTTWRKWYDTSDERLLKAHPGGPCLPPP-PP-IQ-SP-PSFQERVREFCR  225
ARO69918.1   150   AAALFAELPLEYPQGTPLKLSLAMKFAMLKHTTWRKWYDTSDERLLQAHPGGPRLPPP-LP-IQ-SP-PSFQERVREFCR  225
ARO69948.1   150   AAALFAELPLEYPQGIPLKLSLAMKFAMLKHTTWRKWYDTSDERLTEAHPGGPCLPPP-PP-IQ-KP-PSFQERVREFCR  225
QYA72353.1   150   AAALFAELPLEYPQGAPLKLSLAMKFAMLKHTTWRKWYDTSDERLSEAHPGGPCLPPS-PP-IQ-NP-PSFQERVREFCR  225
ABI63917.1   150   AAALFAELPLEYPQGAPLKLSLAMKFAMLKHTTWRKWYDTSDERLLKAHPGGPCFPPP-PP-IQ-SP-PSFQERVREFCR  225
ARO69941.1   150   AAALFAELPLEYPQGTPLKLSLAMKFAMLKHTTWRKWYDTSDERLLEAHPGGPRLPPP-PP-IQ-KP-PSFQERVREFCR  225
ABI63926.1   150   AAALFAELPLEYPQGAPLKLSLAMKFAMLKHTTWRKWYDTSDERLLKAHPGGPCLPPP-PP-IQ-SP-PSFQERVREFCR  225
CDN67471.1   150   AAALFAELPLEYPQGAPLKLSLAMKFAMLKHTTWRKWYDTSDERLLEAHPGGPCLPPP-PP-IQ-SP-PSFYERVREFCR  225
ABI63920.1   150   AAALFAELPLEYPQGAPLKLSLAMKFAMLKHTTWRKWYDTSDERLLKAHPGGPCFPPP-PP-IQ-SP-PSFQERVREFCR  225
ABI63927.1   150   AAALFAELPLEYPQGAPLKLSLAMKFAMLKHTTWRKWYDTSDERLLKAHPGGPCLPPP-PP-IQ-SP-PSFQERVREFCR  225
ARO69908.1   150   AAALFAELPLEYPQGTPLKLSLAMKFAMLKHTTWRKWYDTSDERLLEAHPGGPRLPPP-LP-IQ-KP-PSFQERVREFCR  225
ARO69916.1   150   AAALFAELPLEYPQGTPLKLSLAMKFAMLKHTTWRKWYDTSDERLLEAHPGGPRLPPP-PP-IQ-SP-PSFQERVREFCR  225
```

ARO69910.1	150	AAALFAELPLEYPQGAPLKLSLAMKFAMLKHTTWRKWYDASDERLMEAHPGGPCLPPP-PP-IQ-KP-PSFQERVRESCR	225
DAZ85720.1	150	AAALFAELPLEYPQGAPLKLSLAMKFAMLKHTTWRKWYDTSDERLFEAHPGGPCFPPP-PP-IQ-SP-PSFQERVREFCR	225
AAX18708.1	150	AAALFAELPLEYPQGAPLKLSLAMKFAMLKHTTWRKWYDTSDERLLEAHPGGPCLPPP-PL-IQ-NP-PSFQERVRKFCR	225
AYA22298.1	150	AAALFAELPLEYPQGAPLKLSLAMKFAMLKHTTWRKWYDTSDERLLEAHPGGPCLPPP-PP-IQ-NP-PSFQERVRKFCR	225
ABI63939.1	150	AAALFAELPLEYPQGAPLKLSLAMKFAMLKHTTWRKWYDTSDERLLKAHPGGPCLPPP-PP-IQ-SP-PSFQERVREFCR	225
ARO69926.1	150	AAALFAELPLEYPQGIPLKLSLAMKFAMLKHTTWRKWYDTSDERLTEAHPGGPCLPPP-PP-IQ-KP-PSFQERVREFCR	225
QGP72537.1	150	AAALFAELPLEYPQGTPLKLGLAMKFAMLKHATWRKWYDTSDERLLVAHPGGPCLPPP-PP-IQ-SP-LSFQERIRNFCR	225
ARO69947.1	150	AAALFAELPLEYPQGIPLKLSLAMKFAMLKHTTWRKWYDTSDERLTEAHPGGPCLPPP-PP-IQ-KP-PSFQERVREFCR	225
ARO69921.1	150	AAALFAELPLEYPQGIPLKLSLAMKFAMLKHTTWRKWYDTSDERLMEAHPGGPCLPPP-PP-IQ-KP-PSFQERVREFCR	225
ARO69946.1	150	AAALFAELPLEYPQGTPLKLSLAMKLAMLKHTTWRKWYDTSDERLLEAHPGGPRLPPP-PP-IQ-KP-PSFQERVREFCR	225
QGP72536.1	150	AAALFAELPLEYPQGAPLKLSLAMKFAMLKHTTWRKWYDTSDERLLVAHPGGPCLPPP-PP-IQ-SP-PSFQERVREFCR	225
QGP72538.1	150	AAALFAELPLEYPQGTPLKLGLAMKFAMLKHATWRKWYDTSDERLLVAHPGGPCLPPP-PP-IQ-SP-LSFQERIRNFCR	225
ARO69913.1	150	AAALFAELPLEYPQGIPLKLSLAMKFAMLKHTTWRKWYDTSDERLTEAHPGGPCLPPP-PP-IQ-KP-PSFQERVREFCR	225
ARO69924.1	150	AAALFAELPLEYPQGIPLKLSLAMKFAMLKHTTWRKWYDTSDERLTEAHPGGPCLPPP-PP-IQ-KP-PSFQERVREFCR	225
ABI63924.1	150	AAALFAELPLEYPQGAPLKLSLAMKFAMLKHTTWRKWYDTSDERLLKAHPGGPCFPPP-PP-IQ-SP-PSFQERVREFCR	225
ARO69931.1	150	AAALFAELPLEYPQGTPLKLSLAMKFAMLKHTTWRKWYDTSDERLLEAHPGGPRLPPL-LP-IQ-KP-PSFQERVREFCR	225
AWO67711.1	149	AAALFAELPLEYPQGAPLKLSLAMKFAMLKHTTWRKWYDTSDERLLEAHPGGPCLPPP-PP-IQ-NP-PSFQERVREFCR	224
ARO69943.1	150	AAALFAELPLEYPQGIPLKLSLAMKFAMLKHTTWRKWYDTSDERLMEAHPGGPCLPPP-PP-IQ-KP-PSFQERVREFCR	225
ADJ10928.1	150	AAALFAELPLEYPQGAPLKLSLVMKFAMLKHATWRKWYDTSDERLLEAHPGGPCLPSP-PP-IQ-SP-PSFREKVREFCR	225
ARO69939.1	150	AAALFAELPLEYPQGTPLKLSLAMKFAMLKHTTWRKWYDTSDERLLEAHPGGPCLPPL-LP-IQ-KP-PSFQERVREFCR	225
ABI63938.1	150	AAALFAELPLEYPQGAPLKLSLAMKFAMLKHTTWRKWYDTSDERLLKAHPGGPCLPPP-PP-IQ-SP-PSFQERVREFCR	225
ABI63940.1	150	AAALFAELPLEYPQGAPLKLSLAMKFAMLKHTTWRKWYDTSDERLLKAHPGGPCLPPP-PP-IQ-SP-PSFQERVREFCR	225
ABI63929.1	150	AAALFAELPLEYPQGAPLKLSLAMKFAMLKHTTWRKWYDTSDERLLKAHPGGPCLPPP-PP-IQ-SP-PSFQERVREFCR	225
ABI63919.1	150	AAALFAELPLEYPQGAPLKLSLAMKFAMLKHTTWRRWYDTSDERLLKAHPGGPCFPPP-PP-IQ-SP-PSFQERVREFCR	225
ADJ10934.1	148	AAALFAELPLEYPQGAPLKLSLAAKFAMLRHTTWRKWYDTSDERLLEAHPGGPCLPPP-PP-IQ-NP-PSFQERVRKFCR	223
ARO69937.1	150	AAALFAELPLEYPQGVPLKLSLAMKFAMLKHTTWRKWYDTSDERLTEAHPGGPCLPPP-PP-IQ-KP-PSFQERVREFCR	225
ARO69928.1	150	AAALFAELPLEYPQGAPLKLSLAMKFAMLKHTTWRKWYDASDERLMEAHPGGPCLPPP-PP-IQ-KP-PSFQERVRESCR	225
ARO69927.1	150	AAALFAELPLEYPQGTPLKLSLAMKFAMLKHTTWRKWYDTSDERLLEAHPGGPRLPPL-LP-IQ-KP-PSFQERVREFCR	225
ABI63934.1	149	AAALFAELPLEYPQGAPLKLSLAMKFAMLKHTTWRKWYDTSDERLLKAHPGGPCLPPP-PP-IQ-SP-PSFQERVREFCR	224
ARO69935.1	150	AAALFAELPLEYPQGVPLKLSLAMKFAMLKHTTWRKWYDTSDERLTKAHPGGPCLPPP-PP-IQ-KP-PSFQERVREFCR	225
ARO69940.1	150	AAALFAELPLEYPQGAPLKLSLAMKFAMLKHTTWRKWYDASDERLMEAHPGGPCLPPP-PP-IQ-KP-PSFQERVRESCR	225
ARO69933.1	150	AAALFAELPLEYPQGAPLKLSLAMKFAMLKHTTWRKWYDASDERLMEAHPGGPCLPPP-PP-IQ-KP-PSFQERVRESCR	225
ABI63922.1	150	AAALFAELPLEYPQGAPLKLSLAMKFAMLKHTTWRKWYDTSDERLLKAHPGGPCFPPP-PP-IQ-SP-PSFQERVREFRR	225
ABI63936.1	150	AAALFAELPLEYPQGAPLKLSLAMKFAMLKHTTWRKWYDTSDERLLKAHSGGPCLPPP-PP-IQ-SP-PSFQERVREFCR	225
ABI63918.1	150	AAALFAELPLEYPQGAPLKLSLAMKFAMLKHTTWRKWYDTSDERLLKAHPGGPCFPPP-PP-IQ-SP-PSFQERVREFCR	225
ABI63931.1	149	AAALFAELPLEYPQGAPLKLSLAMKFAMLKHTTWRKWYDTSDERLFEAHPGGPCFPPP-PP-IQ-SP-PSCQERIREFCR	224
QYU59230.1	150	AAALFAELPLEYPQGDPLKLSLAMKFAMLKHTTWRKWYDTSDERLLGAHPGGPCLPPS-PP-IQ-NP-PSFCERIREFCR	225
ARO69922.1	150	AAALFAELPLEYPQGAPLKLSLAMKFAMLKHTTWRKWYDTSDERLMEAHPGGPCLPPP-PP-IQ-KP-PSFQERVRESCR	225
ABI63941.1	150	AAALFAELPLEYPQGAPLKLSLAMKFAMLKHTTWRKWYDTSDERLLKAHPGGPCLPPP-PP-IQ-SP-PSFQERVREFCR	225
ARO69949.1	150	AAALFAELPLEYPQGVPLKLSLAMKFAMLKHTTWRKWYDTSDERLTEAHPGGPCLPPP-PP-IQ-KP-PSFQERVREFCR	225
ARO69917.1	150	AAALFAELPLEYPQGAPLKLSLAMKFAMLKHTTWRKWYDASDERLMEAHPGGPCLPPP-PP-IQ-KP-PSFQERVRESCR	225
ARO69915.1	150	AAALFAELPLEYPQGAPLKLSLAMKFAMLKHTTWRKWYDASDERLMEAHPGGPCLPPP-PP-IQ-KP-PSFQERVRESCR	225
QYU59228.1	150	AAALFAELPLEYPQGTPLKLSMAMKFAMLKHTTWRKWYDTSDERLFEAHPGGPCLPPP-PP-IQ-NP-PSFSERVREFCR	225
AGN29358.1	150	AAALFAELPLEYPQGAPLKLSLAMKFAMLKHTTWRKWYDTSDERLEKAHPGGPCLPPP-LP-IQ-SP-PSFSERIRELCR	225
ARO69944.1	150	AAALFAELPLEYPQGAPLKLSLAMKFAMLKHTTWRKWYDASDERLMEAHPGGPCLPPP-PP-IQ-KP-PSFQERVRESCR	225
AGN29367.1	147	AAALFAELPLEYPQGAPLKLSLAIKFAMLKHTTWRKWYDTSDERLSKAHPGGPCLPPP-PP-IQ-SP-PSFQERVREFCR	222
ABI63921.1	150	AAALFAELPLEYPQGAPLKLSLAMKFAMLKHTTWRKWYDTSDERLLKAHPGGPCFPPP-PP-IQ-SP-PSFQERVREFCR	225
ABI63923.1	150	AAALFAELPLEYPQGVPLMLSLAMKFAMLKHTTWRKWYDTSDERLLKAHPGGPCFPPP-PP-IQ-SP-PSFQERVREFRR	225
CDN67470.1	147	AAALFAELPLEYPQGAPMKLSLAMKFAMLKHTTWRKWYDASDERLMEAHPGGPCLPPP-PP-IQ-SP-PSFQERVREFCR	222
CDN67474.1	150	AAALFAELPSEYPQGAPLKLSLAMKFAMLKHATWRKWYDASDERLLAAHPGGPCLPPP-PP-IQ-SP-PSFQERVREFCR	225
AAX18710.1	150	AAALFAELPLEYPQGAPSKLSLAMKFAMLKHTTWRKWYDTSDERLLEAHPGGPCLLPP-PP-IQ-SP-PSFRERVREFCR	225
QBZ78370.1	148	AAALFAELPLEYPQGAPLKLSLAMKFAMLKHTTWRKWYDTSDERLLGAHPGGPCLPPP-PP-IQ-NP-PSFQERVREFCR	223
ARO69923.1	150	AAALFAELPLEYPQGVPLKLSLAMKFAMLKHTTWRKWYDTSDERLTEAHPGGPCLPPP-PP-IQ-KP-PSFQERVREFCR	225
ARO69907.1	150	AAALFAELPLEYPQGAPLKLSLAMKFAMLKHTTWRKWYDASDERLMEAHPGGPCLPPP-PP-IQ-KP-PSFQERVRESCR	225
ARO69942.1	150	AAALFAELPLEYPQGAPLKLSLAMKFAMLKHTTWRKWYDASDERLMEAHPGGPCLPPP-PP-IQ-KP-PSFQERVRESCR	225
ARO69909.1	150	AAALFAELPLEYPQGAPLKLSLAMKFAMLKHTTWRKWYDASDERLMEAHPGGPCLPPP-PP-IQ-KP-PSFQERVRESCR	225
AAX18711.1	150	AAALFAELPLEYPQGAPLKLSLAMKFAMLKHTTWRKWYDTSDERLLEAHPGGPCLLPP-PP-IQ-SP-PSFQERVREFCR	225
ARO69936.1	150	AAALFAELPLEYPQGAPLKLSLAMKFAMLKHTTWRKWYDASDERLMEAHPGGPCLPPP-PP-IQ-KP-PSFQERVRESCR	225
AGN29357.1	150	AAALFAELPLEYPQGAPLKLSLAMKFAMLKHTTWRKWYDTSDERLLGPHPGGPCLPPP-PP-IQ-SP-LSFQERVREFCR	225
ARO69919.1	150	AAALFAELPLEYPQGAPLKLSLAMKFAMLKHTTWRKWYDASDERLMEAHPGGPCLPPP-PP-IQ-KP-PSFQERVRESCR	225
ADJ10929.1	150	AAALFAELPLEYPQGAPLKLSLAMKFAMLKHTTWRKWYDTSDERLFEAHPGGPCLPPP-PP-IQ-SP-PSFRERLRETCR	225
ABI63943.1	150	AAALFAELPLEYPQGAPLKLSLAMKFAMLKHTTWRKWYDTSDERLLKAHPGGPCLPPP-PP-IQ-SP-PSFQERVREFCR	225
AGN29365.1	150	AAALFAELPLEYPQGTPLKLSLAMKFAMLKHTTWRKWYDTSDERLMEAHPGGPCLPPP-PP-IQ-SP-PSFHERVRELCR	225
ABI63930.1	149	AAALFAELPLEYPQGAPLKLSLAMKSAMLKHTTWRKWYDTSDERLFEAHPGGPCFPPP-PP-IQ-SP-PSFQERIREFCR	224
ABI63942.1	149	AAALFAELPSEYPQGAPLKLSLAMKFAMLKHTTWRKWYDTSDERLFEAHPGGPCFPPP-PP-IQ-SP-PSFQERIREFCR	224
ADJ10939.1	150	VAALFAELPLEYPQGAPLKLSLAAKFAMLKHTTWRKWYDTSDERLLEAHPGGPCLPPP-PP-IQ-NP-PSFQERVREFCR	225
ABI63928.1	149	AAALFAELPLEYPQGAPLKLSLAMKFAMLKHTTWRKWYDTSDEHLFEAHPGGPCFPPP-PP-IQ-SP-PSFQERIREFCR	224
CDN67476.1	147	AAALFAELPLEYPQGAPLKLSLAMKFAMLKHATWRKWYDASDERLFEAHPGGPCLPPP-PS-IQ-SP-PSFHERVRELCR	222
ABI63933.1	149	AAALFAELPLEYPQGAPLKLSLAMKFAMLKHTTWRKWYDTSDERLFEAHPGGPCFPPP-PL-IQ-SP-PSLQERIREFCR	224
AAX18707.1	150	AAALFAELPLEYPQGAPLKLSLAMKFAMLKHTTWRKWYDTSDERLLEAHPGGPCLLPP-PP-IQ-SP-PSFRERVREFCR	225
ACZ58631.1	148	AAALFAELPLEYPQGTPLKLSLVMKFAMLKHTTWRKWYDTSDERLLAAHPGGPCLPPP-PP-IQ-SP-PSFRERLRETCR	223
AGN29359.1	150	AAALFAELPLEYPQGAPLKLSLAMKFAMLKHTTWRKWYDTSDERLEKAHPGGPCLPPP-LP-IQ-SP-PSFSERVRELCR	225
AGN29361.1	150	AAALFAELPLEYPQGAPLKLSLAMKFAMLKHTTWRKWYDTSDERLEKAHPGGPCLPPP-PP-IQ-SS-PSFSERVREFCR	225
ABI63935.1	149	AAALFAELPLEYPQGAPLKLSLAMKFAMLKHTTWRKWYDTSDERLFEAHPGGPCFPPP-PP-IQ-SP-PSFQERIREFCR	224
AGN29360.1	151	AAALFAELPLEYPQGAPLKLSLAMKFAMLKHTTWRKWYDTSDERLLATHPGGPCLPPP-PP-IQ-SP-PSFQERVREFCR	226
AWO67712.1	149	AAALFAELPLEYPQGAPLKLGLAEKFAMLKHTTWRKWYDASDERLLVAHPGGPCLPPP-PP-IQ-SP-PSFQERVRESCR	224
AYA22300.1	147	AAALFAELPLEYPQGAPLKLSLAIKFAMLKHTTWRKWYDTSDERLMVVHPGGPCLPPP-PP-IQ-NP-PSFQERVRKFCR	222
QBZ78373.1	147	AAALFAELPLEYPQGAPLKLSLAMKFAMLKHTTWRKWYDTSDERLLEAHPGGPCLPPP-PP-IQ-SP-PSFQERVREFCR	222
AYQ93056.1	147	AAALFAELPLEYPQGTPLKLSLVMKFAMLKHTTWRKWYDTSDERLLEAHPGGPCLPPP-PP-IQ-NP-PSLRERVKEACR	222
AYQ93054.1	147	AAALFAELPLEYPQGTPLKLSLVMKFAMLKHTTWRKWYDTSDERLLEAHPGGPCLPPP-PP-IQ-NP-PSLRERVKEACR	222
AAX18709.1	147	AAALFAELPLEYPQGAPLKLSLAIKFAMLKHTTWRKWYDTSDERLMVVHPGGPCLPPP-PP-IQ-NP-PSFQERVREFCR	222
AYQ93052.1	148	AAALFAELPLEYPQGAPLKLGLAMKFAMLKHATWRKWYDTSDERLFEAHPGGPCLPPP-PS-IQ-SP-PSFHERVREFCR	223
BCT87062.1	149	AAALFAELPLEYPQGAPLKLSLAMKFALLKHTTWRKWYDASDERLLAIHPGGPSLPPS-PP-IQ-NP-PSFYERVKEFCR	224
AGN29364.1	147	AAALFAELPLGYPQGAPLKLSLAMKFAMLKHTTWRKWYDTSDERLLEAHPGGPCLPPP-PP-IQ-SP-PSFQERVREFCR	222
QYU59234.1	147	AAALFAELPLEYPQGAPLKLSLAMKFAMLKHTTWRKWYDTSDERLLVTHPGGPCLPPS-PP-IQ-NP-PSFQERVREFCR	222
CDN67475.1	147	AAALFAELPLEYPQGTPLKLSLVMKFVMLKHTTWRKWYDTSDERLLEAHPGGPCLPPP-PP-IQ-SP-PSFHERVREFCR	222
AGN29356.1	147	AAALFAELPLEYPQGAPLKLSLAMKFAMLKHTTWRKWYDTSDERLSKAHPGGPCLPPP-PP-IQ-SP-PSFQERVREFCR	222
AGN29363.1	147	AAALFAEPPLEYPQGAPLKLSLAMKFAMLKHTTWRKWYDTSDERLLEVHPGGPCLPPP-PP-IQ-SP-PSFQERVREFCR	222

```
AFV73369.1   150   AAALFAELPMEYPQGAPLKLSLAMKFAMLKHTTWRKWYDASDERLLEAHPGGPCLPPP-PP-IQ-SP-PSFQERVREFCR  225
AFV34732.1   147   AAALFAELPLEYPQGAPLKLSLAMKFAMLKHTTWRKWYDASDERLLEAHPGGPCLPPP-PP-IQ-SP-PSFCERVREFCR  222
AFI43912.1   149   AAALFAELPLEYPQGAPLKMSLAMKFAMLKHATWRKWYDASDERLLNVHPGGPSLPPPhPQ-IQ-TTlPSFKERVRVFLE  226
CDN67472.1   147   AAALFAELPLEYPQGAPLKLGLAMKFAMLKHTTWRKWYDTSDERLLEAHPGGPCLPPP-PP-IQ-SP-PSFQERVKDFCR  222
AGN29355.1   147   AAALFAELPLEYPQGAPLKLSLAMKFAMLKHTTWRKWYDTSDERLLKAHPGGPCLPPP-PP-IQ-SP-PSFQERVREFCR  222
AFI43911.1   149   AAALFAELPLEYPQGAPLKLSMDMKFAALKHATWRKWYDTSDERLQNAHPGGPSLPPPlLQ-IQ-STlPSFKERVRVFLE  226
ADJ10940.1   150   AAALFAELPLEYPQGTPLKLSLAVKFAMLKHTTWRKWYDTSDERLYGAHPGGPCLPPP-PP-IQ-KP-PSFEERLHAAVE  225
DAZ85718.1   150   AAALFAELPLEYPQGAPLKLGLAMKFAMLKHTTWRKWYDTSDERLYGVHPGGPCLPPP-PP-IQ-KP-LSFEERLQAALS  225
ADJ10930.1   150   AAALFAELPLEYPQGTPLKLSLAMKFAMLKHTTWRKWYDTSDKRLYGAHPGGPCLPPP-PP-IQ-KP-PSFEERLHAAVE  225
ADJ10933.1   150   AAALFAELPLEYPQGTPLKLSLAMKFAMLKHTTWRKWYDTSDKRLYGAHPGGPCLPPP-PP-IQ-KP-PSFEERLHAAVE  225
ADJ10935.1   150   AAALFAELPLEYPQGAPLKLSLAMKFALLKHTTWRKWYDTSDKRLYGAHPGGPCLPPP-PP-IQ-KP-PSFEERLHAAVE  225
ADJ10941.1   150   AAALFAELPLEYPQGTPLKLSLAMKFAMLKHTTWRKWYDTSDERLYGAHPGGPCLPPP-PP-IQ-KP-PSFEERLHAAVK  225
DAZ85719.1   150   AAALFAELPLEYPQGAPLKLGLAMKFAMLKHTTWRKWYDTSDERLYGVHPGGPCLPPP-PP-IQ-KP-LSFEERLQAALS  225
ADJ10936.1   150   AAALFAELPLEYPQGTPLKLSLAMKFAMLKHTTWRKWYDTSDKRLYGAHPGGPCLPPP-PP-IQ-KP-PSFEERLHAAVE  225
ABR09917.1   150   AAALFAELPLEYPQGAPLKLSLAMKFATLKHTTWRKWYDTSDERLFGTHPGGPCLPPP-PS-IQ-KP-LSFGERLQAALS  225
CDN67473.1   155   AAALFAELPLEYPQGAPLKLSLAMKFAMLKHTTWRKWYDTSDERLLEAHPGGPCLPPP-PP-IQ-SP-PSFYERVGEFCR  230
QYU59232.1   147   AAALFAELPLEYPQGAPLKLSLAMKFAMLKHATWRKWYDTSDERLCGAHPGGPCLPPP-PE-IQ-KP-SSFVERLHAAIE  222
AGN29362.1   147   ATTLFAELPLEYPQAAPLKLSLPMKFAMLKHTTCKRWYDTVDERLLDAHPCGPCHPPP-PP-IQ-SP-HFFQESVREFCR  222
ACZ58625.1   149   AAALFAELPLEYPQGAPLKLSLAMKFAMLKHTTWRKWYDASDERLLKAHPGGPCLPPP-SP-IQ-SL-PSFQERVREFCR  224
ACZ58626.1   149   AAALFAELPLEYPQGAPLKLSLAMKFAMLKHTTWRKWYDTSDERLLIGHPGGPCLPPP-PP-IQ-SL-PSFQERVREFCR  224
ACZ58624.1   149   AAALFAELPLEYPQGAPLKLSLAMKFAMLKHTTWRKWYDTSDERLLKAHPGGPCLPPP-PP-IQ-SL-PSFQERVREFCR  224
AGN29366.1   150   AAALFAELPLEYPQGAPLKLSLAMKFAMLKHATWRKWYDTSDERLLEAHPGGPCLPSP-PL-IQ-SP-PSFYERVRAFCR  225
QBZ78368.1   149   AAALFAELPLEYPQGAPLKLSLAMKFAMLKHTTWRKWYDASDERLLKAHPGGPCLPPP-PP-IQ-SL-PSFQERVREFCR  224
AFI43914.1   156   AAALFADLPLEYPQGAPLKLSMAMKFAALKHATWRKWYDTSDERLQNAHPGGPSLPPPlLQ-IQ-STlPSFKERVRVFLE  233
AFI43913.1   156   AAALFAELPLEYPQGAPLKLSMDMKFAALKHATWRKWYDTSDERLQNAHPGGPSLPPPlLQ-IQ-STlPSFKERVRVFLE  233
AFV34756.1   89    AAALFAELPLEYPQGAPLKLSLAMKFAMLKHTTWRKWYDTSDERLLEAHPGGPCLPPP-PP-IQ-SP-PSFHERVREFCR  164
QYU59231.1   149   AAALFAELPLEYPQGTPLKLSLAMKFAMLKHTTWRKWYDTSDERLCGAHPGGPCLPPP-PE-IQ-KP-PSFVERLHAAIG  224
QYU59233.1   156   AAALFAELPLEYPQGAPLKLSLAMKFAMLKHTTWRKWYDTSDERLYGAHPGGPCLPPP-PK-IQ-KP-PSFAERLHAAIG  231
APD26307.1   156   AAALFAELPLEYPQGAPLKLSLAMKFAMLKHTTWRKWYDTSDERLLVAHPGGPCLPPS-PPeIQiTL-PSFCERLRVFLE  233
QBZ78371.1   1     -----------------------MKFAMLKHTTWRKWYDTSDERLLGVHPGGPCLPPP-PP-IQ-NP-PSFHEKVREFHR  53
```

2a-MP cleavage site

```
NP_619706.1  225   MKSCTKAFALETSLGLNKAWVGLVDIPSTSVCCADGKTTGGQTIAQEADPLQHRISTSVAPGRAQWISERRQALRRREQA  304
AWO68043.1   226   MKSCAKAFALETSLGLNKAWVGLVDIPSTSVCCADGKTTGGQTIAQEADPLQHRISTSVAPGRAQWISERRQALRRREQA  305
ADJ10937.1   226   MKSCAKAFALETSLGLNKAWVGLVDIPSTSVCCADGKTTGGQTIAQEADPLQHRISTSVAPGRAQWISERRQALRRREQA  305
ADJ10931.1   226   MKSCAKAFALETSLGLNKAWVGLVDISSTSVCCADGKTTGGQTIAQEADPLQHRISTSVAPGRAQWISERRQALRRREQA  305
ADJ10938.1   226   MKSCAKAFALETSLGLNKAWVGLVDIPSTSVCCADGKTTGGQTIAQEADPLQHRISTSVAPGRAQWISERRQALRRREQA  305
QYU59229.1   226   MKSCARAFALETSLGFNRAWVGLVDIPSTSVCCADGRTTGGQTIAQEADPLQHRVSTSVAPGRAQWISERRQALRRREQA  305
ACZ58630.1   226   MKSCARVFALETSLGLNKAWVGLVDIPSTSVCCADGRTTGGQTIAQEADPLQHRVSTSVAPGRAQWISERRQALRRREQA  305
QGP72535.1   226   IKSCAKAFALETSLGLNKAWVGLVNIPSTSVCCADGRTTGGQTIAQEADPLQHRVSTSVAPGRAQWISERRQALRRREQA  305
Q91HK5.1     226   MKSCARAFALETSLGLNRAWVGLVDIPSTSVCCADGRTTGGQTIAQEADPLQHRVSTSVAPGRAQWISERRQALRRREQA  305
ARO69912.1   226   VKSCARAFALETSLGLNKAWVGLVDIPSTSVCCADGRTTGGQTIAQEADPLQHRVSTSVAPGRAQWISERRQALRRREQA  305
AFI71814.1   226   MKSCAKAFSLETSLGLNKAWVGSIDIPSTSVCCADGRTTGGQTIAQEADPLQHRVSTSVAPGRAQWISERRQALRRREQA  305
ADJ10932.1   226   MKSCAKAFALETSLGLNKAWVGLVDIPSTSVCCADGKTTGGQTIAQEADPLQHRISTSVAPGRAQWISERRQALRRREQA  305
ARO69925.1   226   VKSCARAFALETSLGLNKAWVGLVDIPSTSVCCADGRTTGGQTIAQEADPLQHRISTSVAPGRAQWISERRQALRRREQA  305
ARO69911.1   226   VKSCARAFALETSLGLNKAWVGLVDIPSTSVCCADGRTTGGQTLAQEADPLQHRVSTSVAPGRAQWISERRQALRRREQA  305
ACZ58628.1   226   KKSCAKAFALETSLGLNKAWVGIVDIPSTSVCCADGRTTGGQTIAQEADPLQHRVSTSVAPGRAQWISERRQALRRREQA  305
ABI63932.1   226   MKSCAKAFALETSLGLNKAWVGLVDIPSTSVCCADGRTTGGQTIAQEADPLQHRVSISVAPGRAQWISERRQALRRREQA  305
ARO69945.1   226   VKSCARAFALETSLGLNKAWVGLVDIPSTSVCCADGRTTGGQTLAQEADPLQHRVSTSVAPGRAQWISERRQALRRREQA  305
ARO69930.1   226   VKSCARAFALETSLGLNKAWVGLVDIPSTSVCCADGRTTGGQTLAQEADPLQHRVSTSVAPGRAQWISERRQALRRREQA  305
ABI63925.1   226   MKSCAKAFALETSLGLNKAWVGLVDIPSTSVCCADGRTTGGQTIAQEADPLQHRVSTSVAPGRAQWISERRQALRRREQA  305
ARO69906.1   226   VKSCARAFALETSLGLNKAWVGLVDIPSTSVCCADGRTTGGQTIAQEADPLQHRISTSVAPGRAQWISERRQALRRREQA  305
ACZ58627.1   226   KKSCAKAFALETSLGLNKAWVGIVDIPSTSVCCADGRTTGGQTIAQEADPLQHRVSTSVAPGRAQWISERRQALRRREQA  305
ARO69920.1   226   VKSCARAFALETSLGLNKAWVGLVDIPSTSVCCADGRTTGGQTLAQEADPLQHRVSTSVAPGRAQWISERRQALRRREQA  305
ACZ58629.1   226   KKSCAKAFALETSLGLNKAWVGIVDIPSTSVCCADGRTTGGQTIAQEADPLQHRVSTSVAPGRAQWISERRQALRRREQA  305
AGT42201.1   226   MKSCAKAFSLETSLGLNKAWVGSIDIPSTSVCCADGRTTGGQTIAQEADPLQHRVSTSVAPGRAQWISERRQALRRREQA  305
ARO69914.1   226   IKSCARAFALETSLGLNKAWVGLVDIPSTSVCCADGRTTGGQTLAQEADPLQHRVSTSVAPGRAQWISERRQALRRREQA  305
ABI63916.1   226   MKSCAKAFALETSLGLNKAWVGLVDIPSTSVCCADGRTTGGQTIAQETDPLQHRVSTSVAPGRAQWISERRQALRRREQA  305
ARO69918.1   226   VKSCARAFALETSLGLNKAWVGLVDIPSTSVCCADGRTTGGQTLAQEADPLQHRVSTSVAPGRAQWISERRQALRRREQA  305
ARO69948.1   226   IKSCARAFALETSLGLNRAWVGLVDIPSTSVCCADGRTTGGQTIAQEADPLQHRVSTSVAPGRAQWISERRQALRRREQA  305
QYA72353.1   226   MKSCARAFALETSLGLNRAWVGLVDIPSTSVCCADGRTTGGQTIAQEADPLQHRVSTSVAPGRAQWISERRQALRRREQA  305
ABI63917.1   226   MKSCAKAFALETSLGLNKAWVGLVDIPSTSVCCADGRTTGGQTIAQEADPLQHRVSTSVAPGRAQWISERRQALRRREQA  305
ARO69941.1   226   IKSCARAFALETSLGLNRAWVGLVDIPSTSVCCADGRTTGGQTIAQEADPLQHRVSTSVAPGRAQWISERRQALRRREQA  305
ABI63926.1   226   MKSCAKAFALETSLGLNKAWVGLVDIPSTSVCCADGRTTGGQTIAQEADPLQHGVSISVAPGRAQWISERRQALRRREQA  305
CDN67471.1   226   MKSCARAFALETSLGLNGAWIGLVDIPSTSVCCADGRTTGGQTIAQEADPLQHRVSTSVAPGRAQWISERRQALRRREQA  305
ABI63920.1   226   MKSCAKAFALETSLGLNKAWVGLVDIPSTSVCCADGRTTGGQTIAQEADPLQHRVSTSVAPGRAQWISERRQALRRREQA  305
ABI63927.1   226   MKSCAKAFALETSLGLNKAWVGLVDIPSTSVCCADGRTTGGQTIAQEADPLQHRVSISVAPGRAQWISERRQALRRREQA  305
ARO69908.1   226   IKSCARAFALETSLGLNRAWVGLVDIPSTSVCCADGRTTGGQTIAQEADPLQHRVSTSVAPGRAQWISERRQALRRREQA  305
ARO69916.1   226   VKSCARAFALETSLGLNKAWVGLVDIPSTSVCCADGRTTGGQTIAQEADPLQHRVSTSVAPGRAQWISERRQALRRREQA  305
ARO69910.1   226   IKSCARAFALETSLGLNRAWVGLVDIPSTSVCCADGRTTGGQTIAQEADPLQHRVSTSVAPGRAQWISERRQALRRREQA  305
DAZ85720.1   226   MKSCARAFALETSLGLNKAWVGLVDIPSTSVCCADGRTTGGQTIAQEADPLQHRVSTSVAPGRAQWISERRQALRRREQA  305
AAX18708.1   226   MKSCSRAFALETSLGFNRAWVGLVDIPSTSVCCADGRTTGGQTIAQEADPLQHRVSTSVAPGRAQWISERRQALRRREQA  305
AYA22298.1   226   MKSCSRAFALETSLGFNRAWVGLVDIPSTSVCCADGRTTGGQTIAQEADPLQHRVSTSVAPGRAQWISERRQALRRREQA  305
ABI63939.1   226   MKSCAKAFALETSLGLNKAWVGLVDIPSTSVCCADGRTTGGQTIAQEADPLQHRVSISVAPGRAQWISERRQALRRREQA  305
ARO69926.1   226   IKSCARAFALETSLGLNRAWVGLVDIPSTSVCCADGRTTGGQTIAQEADPLQHRVSTSVAPGRAQWISERRQALRRREQA  305
QGP72537.1   226   VKSYARAFALETSLGLNKAWVGLVNIPSTSVCCADGRTTGGQTIAQEADPLQHRVSTSVAPGRAQWISERRQALRRREQA  305
ARO69947.1   226   IKSCARAFALETSLGLNRAWVGLVDIPSTSVCCADGRTTGGQTIAQEADPLQHRVSTSVAPGRAQWISERRQALRRREQA  305
ARO69921.1   226   IKSCARAFALETSLGLNRAWVGLVDIPSTSVCCADGRTTGGQTIAQEADPLQHRVSTSVAPGRAQWISERRQALRRREQA  305
ARO69946.1   226   IKSCARAFALETSLGLDRAWVGLVDIPSTSVCCADGRTTGGQTIAQEADPLQHRVSTSVAPGRAQWISERRQALRRREQA  305
QGP72536.1   226   IKSCAKAFALETSLGLNRAWVGLVNIPSTSVCCADGRTTGGQTIAQEADPLQHRVSASVAPGRAQWISERRQALRRREQA  305
QGP72538.1   226   VKSYARAFALETSLGLNKAWVGLVNIPSTSVCCADGRTTGGQTIAQEADPLQHRVSTSVAPGRAQWISERRQALRRREQA  305
ARO69913.1   226   IKSCSRAFALETSLGLNRAWVGLVDIPSTSVCCADGRTTGGQTIAQEADPLQHRVSTSVAPGRAQWISERRQALRRREQA  305
ARO69924.1   226   IKSCARAFALETSLGLNRAWVGLVDIPSTSVCCADGRTTGGQTIAQEADPLQHRVSTSVAPGRAQWISERRQALRRREQA  305
ABI63924.1   226   MKSCAKAFALETSLGLNKAWVGLVDIPSTSVCCADGRTTGGQTIAQEADPLQHRVSTSVAPGRAQWISERRQALRRREQA  305
ARO69931.1   226   IKSCARAFALETSLGLNRAWVGLVDIPSTSVCCADGRTTGGQTIAQEADPLQHRVSTSVAPGRAQWISERRQALRRREQA  305
```

```
AWO67711.1   225   MKSCARAFALETSLGLNMAWVGLVDIPSTSVCCADGRTTGGQTIAQEADPLQHRVSTSVAPGRAQWISERRQALRRREQA  304
ARO69943.1   226   IKSCARAFALETSLGLNRAWVGLVDIPSTSVCCADGRTTGGQTIAQEADPLQHRVSTSVAPGRAQWISERRQALRRREQA  305
ADJ10928.1   226   MKSCARAFALETSLGLNRAWMGSVDIPSTSVCCADGRTTGGQTIAQEADPLQHRVSTSVAPGRAQWISERRQALRRREQA  305
ARO69939.1   226   IKSCARAFALETSLGLNRAWVGLVDIPSTSVCCADGRTTGGQTIAQEADPLQHRVSTSVAPGRAQWISERRQALRRREQA  305
ABI63938.1   226   MKSCAKAFALETSLGLNKAWVGLVDIPSTSVCCADGRTTGGRTIAQEADPLQHRVSISVAPGRAQWISERRQALRRREQA  305
ABI63940.1   226   MKSCAKAFALETSLGLNKAWVGLVDIPSTSVCCADGRTTGGQTIAQEADPLQHRVSISVAPGRAQWISERRQALRRREQA  305
ABI63929.1   226   MKSCAKAFALETSLGLNKAWVGLVDIPSTSVCCADGRTTGGQTIAQEADPLQHRVSISVAPGRAQWISERRQALRRREQA  305
ABI63919.1   226   MKSCAKAFALETSLGLNKAWVGLVDIPSTSVCCADGRTTGGQTIAQEADPLQHRVSTSVAPGRAQWISERRQALRRREQA  305
ADJ10934.1   224   MKSCAKAFALETSLGLNKAWVGLVDIPSTSVCCADGKTTGGQTIAQEADPLQHRISTSVAPGRAQWISERRQALRRREQA  303
ARO69937.1   226   IKSCARAFALETSLGLNRAWVGLVDIPSTSVCCADGRTTGGQTIAQEADPLQHRVSTSVAPGRAQWISERRQALRRREQA  305
ARO69928.1   226   IKSCARAFALETSLGLNRAWVGLVDIPSTSVCCADGRTTGGQTIAQEADPLQHRVSTSVAPGRAQWISERRQALRRREQA  305
ARO69927.1   226   IKSCARAFALETSLGLNRAWVGLVDIPSTSVCCADGRTTGGQTIAQEADPLQHRVSTSVAPGRAQWISERRQALRRREQA  305
ABI63934.1   225   MKSCAKAFALETSLGLNKARVGLVDIPSTSVCCADGRTTGGQTIAQEADPLQHRVSMSVAPGRAQWISERRQALRRREQA  304
ARO69935.1   226   IKSCARAFALETSLGLNRAWVGLVDIPSTSVCCADGRTTGGQTIAQEADPLQHRVSTSVAPGRAQWISERRQALRRREQA  305
ARO69940.1   226   IKSCARAFALETSLGLNRAWVGLVDIPSTSVCCADGRTTGGQTIAQEADPLQHRVSTSVAPGRAQWISERRQALRRREQA  305
ARO69933.1   226   IKSCARAFALETSLGLNRAWVGLVDIPSTSVCCADGRTTGGQTIAQEADPLQHRVSTSVAPGRAQWISERRQALRKREQA  305
ABI63922.1   226   MKSCAKAFALETSLGLNKAWVGLVDIPSTSVCCADGRTTGGQTIAQEADPLQHRASTSVAPGRAQWISERRQALRRREQA  305
ABI63936.1   226   MKSCAKAFALETSLGLNKAWVGLVDIPSTSVCCADGRTTGGQTIAQEADPLQHRVSISVAPGRAQWISERRQALRRREQA  305
ABI63918.1   226   MKSCAKAFALGTSLGLNKAWVGLVDIPSTSVCCADGRTTGGQTIAQEADPLQHRVSTSVAPGRAQWISERRQALRRREQA  305
ABI63931.1   225   MKSCARAFALETSLGLNKAWVGLVDIPSTSVCCADGRTTGGQTIAQEADPLQHRVSTSVAPGRAQWVSERRQALRRREQA  304
QYU59230.1   226   MKSCARAFALETSLGLNKAWTGLVDIPSTSVCCADGRTTGGQTIAQEADPLLHRVSSSVAPGRAQWISERRQALRRREQA  305
ARO69922.1   226   IKSCARAFALETSLGLNRAWVGLVDIPSTSVCCADGRTTGGQTIAQEADPLQHRVSTSVAPGRAQWISERRQALRRREQA  305
ABI63941.1   226   MKSCAKAFALETSLGLNKAWVGLVDIPSTSVCCADGRTTGGQTIAQEADPLQHRVSISVAPGRAQWISERRQALRRREQA  305
ARO69949.1   226   IKSCARAFALETSLGLNRAWVGLVDIPSTSVCCADGRTTGGQTIAQEADPLQHRVSTSVAPGRAQWISDRRQALRRREQA  305
ARO69917.1   226   IKSCARAFALETSLGLNRAWVGLVDIPSTSVCCADGRTTGGQTIAQEADPLQHRVSTSVAPGRAQWISERRQALRRREQA  305
ARO69915.1   226   IKSCARAFALETSLGLNRAWVGLVDIPSTSVCCADGRTTGGQTIAQEADPLQHRVSTSVAPGRAQWISERRQALRRREQA  305
QYU59228.1   226   KKSCARAFALETSLGLNMAWVGLVDIPSTSVCCADGRTTGGQTIAQEADPLQHRVSTSVAPGRAQWISERRQALRRREQA  305
AGN29358.1   226   TKSCARAFALETSLGLNRAWVGLVDIPSTSVCCADGRTTGGQTIAQEADPLQHRVSTSVAPGRAQWISERRQALRRREQA  305
ARO69944.1   226   IKSCARAFALETSLGLNRAWVGLVDIPSTSVCCADGRTTGGQTIAQEADPLQHRVSTSVAPGRAQWISERRQALRRREQA  305
AGN29367.1   223   MKSCSRAFALETSLGLNRAWVGLVDIPSTSVCCADGRTTGGQTIAQEADPLQHRVSTSVAPGRAQWISERRQALRRREQA  302
ABI63921.1   226   MKSCAKAFALETSLGLNKAWVGLVDIPSTSVCCADGRTTGGQTIAQEADPLQHRVSTSVAPGRAQWISERRQALRRREQA  305
ABI63923.1   226   MKSCAKAFALETSLGLNKAWVGLVDIPSTSVCCADGRTTGGQTIAQEADPLQHRVSTSVAPGGAQWISERRQALRRREQA  305
CDN67470.1   223   MKSCAKAFALETSLGLTRAWIGLVDIPSTSVCCADGRTTGGQTIAQEADPLQHRVSTSVAPGRAQWISERRQALRRREQA  302
CDN67474.1   226   KKSCARAFALETSLGLNKAWVGLVDIPSTSVCCADGRTTGGQTIAQEADPLQHRVSTSVAPGRAQWISERRQALRRREQA  305
AAX18710.1   226   ARSCARAFALETSLGLNRAWVGSIDIPSTSVCCADGRTTGGQTIAQEADPLQHRVSTSVAPGRAQWISERRQALRRREQA  305
QBZ78370.1   224   MKSCAKAFALETSLGLNRAWVGLVDIPSTSVCCADGRTTGGQTIAQEADPLQHRVSTSVAPGRAQWISERRQALRRREQA  303
ARO69923.1   226   IKSCARAFALETSLGLNRAWVGLVDIPSTSVCCADGRTTGGQTIAQEADPLQHRVSTSVAPGRAQWISERRQALRRREQA  305
ARO69907.1   226   IKSCARAFALETSLGLNRAWVGLVDIPSTSVCCADGRTTGGQTIAQEADPLQHRVSTSVAPGRAQWISERRQALRRREQA  305
ARO69942.1   226   IKSCARAFALETSLGLNRAWVGLVDIPSTSVCCADGRTTGGQTIAQEADPLQHRVSTSVAPGRAQWISERRQALRRREQA  305
ARO69909.1   226   IKSCARAFALETSLGLNRAWVGLVDIPSTSVCCADGRTTGGQTIAQEADPLQHRVSTSVAPGRAQWISERRQALRRREQA  305
AAX18711.1   226   ARSCARAFAWETSLGLNRAWVGSIDIPSTSVCCADGRTTGGQTIAQEADPLQHRISTSVAPGRAQWISERRQALRRREQA  305
ARO69936.1   226   IKSCARAFALETSLGLNRAWVGLVDIPSTSVCCADGRTTGGQTIAQEADPLQHRVSTSVAPGRAQWISERRQALRRREQA  305
AGN29357.1   226   MRSCARAFALETSLGFNKAWVGLVDIPGTSVCCADGRTTGGQTIAQEADPLHIRVSTSVAPGRAQWISERRQALRRREQA  305
ARO69919.1   226   IKSCARAFALETSLGLNRAWVGLVDIPSTSVCCADGRTTGGQTIAQEADPLQHRVSTSVAPGRAQWISERRQALRRREQA  305
ADJ10929.1   226   VKSCARAFALETSLGLNKAWVGLVNIPSTSVCCADGRTTGGQTIAQEADPLQHRVSTSVAPGRAQWISERRQALRRREQA  305
ABI63943.1   226   MKSCAKAFALETSLGLNKAWVGLVDIPSTSVCCADGRTTGGQTIAQEADPLQHRVSISVAPGRAQWISERRQALRRREQA  305
AGN29365.1   226   TKSCARAFALETSLGLNRAWVGLVDIPSTSVCCADGRTTGGQTIAQEADPLQHRVSTSVAPGRAQWISERRQALRRREQA  305
ABI63930.1   225   MKSCARAFALETSLGLNKAWVGLVDIPSTSVCCADGRTTGGQTIAQEADPLQHRVSTSVAPGRAQWVSERRQALRRREQA  304
ABI63942.1   225   MKSCARAFALETSLGLNKAWVGLVDIPSTSVCCADGRTTGGQTIAQEADPLQHRVSTSVAPGRAQWVSERRQALRRREQA  304
ADJ10939.1   226   MKSCAKAFALETSLGLNKAWVGLVDIPSTSVCCADGKTTGGQTIAQEADSLQHRISTSVAPGRAQWISERRQALRRREQA  305
ABI63928.1   225   MKSCAWAFALETSLGLNKAWVGLVDIPSTSVCCADGRTTGGQTIAQEADPLQHRVSTSVAPGGAQWVSERRQALRRREQA  304
CDN67476.1   223   MKSCAKAFALETSLGLNRAWIGLVDIPSTSVCCADGRTTGGQTIAQEADPLQHRVSTSVAPGRAQWVSERRQALRRREQA  302
ABI63933.1   225   MKSCARAFALETSLGLNKAWVGLVDIPSTAVCCADGRTTGGQTIAQEADPLQHRVSTSVAPGRAQWVSERRQALRRREQA  304
AAX18707.1   226   ARSCARAFALETSLGLNRAWVGSIDIPSTSVCCADGRTTGGQTIAQEADPLQHRVSTSVAPGRAQWISERRQALRRREQA  305
ACZ58631.1   224   VKSCARAFALETSLGLNKAWVGLVNIPSTSVCCADGRTTGGQTIAQEADPLQHRVSTSVAPGRAQWISERRQALRRREQA  303
AGN29359.1   226   TKSCARAFALETSLGLNRAWVGLVDIPSTSVCCADGRTTGGQTIAQEADPLQHRVSTSVAPGRAQWISERRQALRRREQA  305
AGN29361.1   226   TKSCARAFALETSLGLNRAWVGLVDIPSTSVCCADGRTTGGQTIAQEADPLQHRVSTSVAPGRAQWISERRQALRRREQA  305
ABI63935.1   225   MKSCARALALETSLGLNKAWVGLVDIPSTSVCCADGRTTGGQTIAQEADPLQHRVSTSVAPGRAQWVSERRQALRRREQA  304
AGN29360.1   227   MKSCAKAFALETSLGLNKAWVGLVDIPSTSVCRPDGRTTGGQTIAQEADPLQHRVSTSVAPGTAQWISERRQALRRTEQA  306
AWO67712.1   225   VKSCARAFALETSLGLNRAWVGLVNIPSTSVCCADGRTTGGQTIAQEADPLQHRVSASVAPGRAQWISERRQALRRREQA  304
AYA22300.1   223   LKSCARAFALETSLGSNKAWVGLVDIPSTSVCCADGRTTGGQTIAQEADPLQHRVSTSVAPGRAQWISERRQALRRREQA  302
QBZ78373.1   223   MKSCARAFALETSLGLNKAWMGLVDIPSTSVCCADGRTTGGQTIAQEADPLQHRVSSSVAPGRAQWISERRQALRRREQA  302
AYQ93056.1   223   IKSCARAFALETSLGLNRAWIGLVDIPSTSVCCADGRTTGGQTIAQEADPLQHRVSTSVAPGRAQWISERRQALRRREQA  302
AYQ93054.1   223   IKSCARAFALETSLGLNRAWIGLVDIPSTSVCCADGRTTGGQTIAQEADPLQHRVSTSVAPGRAQWISERRQALRRREQA  302
AAX18709.1   223   LKSCARAFALETSLGFNKAWVGLVDIPSTSVCCADGRTTGGQTIAQEADPLQHRVSTSVAPGRAQWISERRQALRRREQA  302
AYQ93052.1   224   AKSCARAFALETSLGLNRAWVGFVDIPSTSVCCADGRTTGGQTIAQEADPLQHRVSTSVAPGRAQWISERRQALRRREQA  303
BCT87062.1   225   VKSCARAFALETSLGLNRAWVGIVDIPSTSVCCADGRTTGGQTIAQEADPLQHRVSTSVAPGRAQWISERRQALRRREQA  304
AGN29364.1   223   VKSCAKAFALETSLGLNKAWVGLVDIPSTSVCCADGRTTGGQTIAQEADPLQHRVSTSVAPGRAQWISERRQALRRREQA  302
QYU59234.1   223   VKTCARAFALETSLGLNRAWVGSVNIPSTSVCCADGRTTGGQTIAQEADPLQHRVSTSVAPGRAQWISERRQALRRREQA  302
CDN67475.1   223   AKSCARAFALETSLGLNKVWVGLVDIPSTSVCCADGRTTGGQTIAQEADPLQHRVSTSVAPGRAQWISERRQALRRREQA  302
AGN29356.1   223   MKSCSRAFALETSLGLNKAWVGLVDIPSTSVCCADGRTTGGQTIAQEADPLQHRVSTSVAPGRAQWISERRQALRRREQA  302
AGN29363.1   223   AKSCAKAFALETSLGLNKAWVGLVDIPSTSVCCVDGRTTGGQTIAQEADPLQHRVSTSVAPGRAQWISERRQALRRREQA  302
AFV73369.1   226   MKSCARVFALETSSGLNKAWIGSINIPSTSVCCADGRTTGGQTIAQEADPLQHRVSTSVAPGRAQWISERRQALRRREQA  305
AFV34732.1   223   MKSCARVFALETSSGLNKAWIGSINIPSTSVCCADGRTTGGQTIAQEADPLQHRVSTSVAPGRAQWISERRQALRRREQA  302
AFI43912.1   227   RKSCSRTFSLETSLGLNRAWVGTIDLPSTSVCCADGRTTGGQTIAQEADPLQHRVSSSVAPGRAEWISERRQALRRREQA  306
CDN67472.1   223   RKSCARAFALETSLGLNKAWVGLVDIPSTSVCCADGRTTGGQTIAQEADPLQHRVSTSVAPGRAQWISERRQALRRREQA  302
AGN29355.1   223   MKSCSRAFALETSLGLNRAWVGLVDIPSTSVCCADGRTTGGQTIAQEADPLQHRVSTSVAPGRAQWISERRQALRRREQA  302
AFI43911.1   227   RKSCSRAFSLETSLGLNRAWVGTVDIPSTSVCCADGRTTGGQTIAQEADPLQHRVSSSVAPGRAEWISERRQALRRREQA  306
ADJ10940.1   226   RQQCARAFALETSLGLNKAWVGIIDIPSTSVCCADGRTTGGQTIAQEADPLNHRVSSNTAPGRAAWISERRSALRRREQA  305
DAZ85718.1   226   RQSCARAFALETSLGLNMAWLGTVTIPSTSVCCADGRTTGGQTIAQEADPLNHRVSSNTAPGRAAWISERRSALRRREQA  305
ADJ10930.1   226   RQQCARAFALETSLGLNKAWVGIFDIPSTSVCCADGRTTGGQTIAQEADPLNHRVSSNTAPGRAAWISERRSALRRREQA  305
ADJ10933.1   226   RQQCARAFALETSLGLNKAWVGIFDIPSTSVCCADGRTTGGQTIAQEADPLNHRVSSNTAPGRAAWISERRSALRRREQA  305
ADJ10935.1   226   RQQCARAFALETSLGLNKAWVGIFDIPSTSVCCADGRTTGGQTIAQEADPLNHRVSSNTAPGRAAWISERRSALRRREQA  305
ADJ10941.1   226   RQQCARAFAQETSLGLNKAWVGIIDIPSTSVCCADGRTTGGQTIAQEADPLNHRVSSNTAPGRAAWISERRSALRRREQA  305
DAZ85719.1   226   RQSCARAFALETSLGLNMAWLGTVTIPSTSVCCADGRTTGGQTIAQEADPLNHRVSSNTAPGRAAWISERRSALRRREQA  305
ADJ10936.1   226   RQQCARAFALETSLGLNKAWVGIFDIPSTSVCCADGRTTGGQTIAQEADPLNHRVSSNTAPGRAAWISERRSALRRREQA  305
ABR09917.1   226   RQSCARAFALETSLGLNMAWVGTAAIPSTSVCCADGRTTGGQTIAQEADPLNHRVSSNTAPGRAAWISERRSALRRREQA  305
CDN67473.1   231   MKSCARAFALETSLGLNRAWIGLVDIPSTSVCCADGRTTGGQTIAQEADPLQHRVSTSVAPGRAQWISERRQALRRREQA  310
```

```
QYU59232.1   223   RQQCARAFALETSLGLNRAWVGTVDIPSTSVCCADGRTTGGQTIAQEADPLSHRVSSNVAPGRAEWISERRSALRRREQA  302
AGN29362.1   223   MKSCSRAFALETSLALNRAWVGLKEITSRRVCCADGRNIGGQTIAQEADPLQHRVSTSVAPGRAQWISERRQALRRREQA  302
ACZ58625.1   225   MKSCAEAFALETSLGLNRAWVGLVDIPSTSVCCADGRTTGGQTIAQEADPLQHRVSTSVAPGRAQWISERRQALRRREQA  304
ACZ58626.1   225   MKSCAEAFALETSLGLNRAWVGLVDIPSTSVCCADGRTTGGQTIAQEADPLQHRVSTSVAPGRAQWISERRQALRRREQA  304
ACZ58624.1   225   MKSCAEAFALETSLGLNRAWIGLVDIPSTSVCCADGRTTGGQTIAQEADPLQHRVSTSVAPGRAQWISERRQALRRREQA  304
AGN29366.1   226   MKSCSKAFALETSLGLIKAWVGLVDIPSTSVCCADGRTTGGQTIAQEADPLQHRVSTSVAPGRAQWISERRQALRRREQA  305
QBZ78368.1   225   MKSCAEAFALETSLGLNRAWIGLMDIPSTSVCCADGRTTGGQTIAQEADPLQHRVSTSVAPGRAQWISERRQALRRREQA  304
AFI43914.1   234   RKSCSRAFSLETSLGLNKAWVGTIDIPSTSVCCADGRTTGGQTIAQEADPLQHRVSSNVAPGRAEWISERRQALRRREQA  313
AFI43913.1   234   RKSCSRAFSLETSLGLNKAWVSTIDIPSTSVCCADGRTAGGQTIAQEADPLQHRVSSNVAPSRAEWISERRQALKRREQA  313
AFV34756.1   165   MKSCARVFALETSSGLNKAWIGSINIPNTSVCCADGRTTGGQTIAQEADPLQHRVSTSVAPGRAQWISERRQALRRREQA  244
QYU59231.1   225   RQQCARAFALETSLGLNKAWVGTVDIPSTSVCCADGRTTGGQTIAQEADPLSHRVSSNVAPGRAEWISERRSALRRREQA  304
QYU59233.1   232   RQQCVRAFALETSLGLNKAWVGIVDIPNTSVCCADGRTTGGQTIAQEADPLSHRVSSNVAPGRAEWISERRSALRRREQA  311
APD26307.1   234   KKSCARAFSNEISLGLNRAWVGLVDIPSTSVCCADGRTTGGQTIAQEADPLQHRVSSNTAPGRAQWISERRQALRRREQA  313
QBZ78371.1   54    MKSCARVFALETSLGLNQAWVGTFDIPSTSVCCADGRTTGGQTIAQEADPLQHRVSSNVAPGRAEWISERRQALRRREQA  133


NP_619706.1  305   NSFEGLAAQTDMTFEQARNAYLGAADMIEQGLPLLPPLRSAYAPRGLWRGPSTRANYTLDFRLNGIPTGTNTLEILYNPV  384
AWO68043.1   306   NSLQGLAAQTDMTFEQARNAYLGAADMIEQGLPLLPPLRSAYAPRGLWRGPSTRANYTLDFRLNGIPTGTNTLEILYNPV  385
ADJ10937.1   306   NSLQGLAAQTDMTFEQARNAYLGAADMIEQGLPLLPPLRSAYAPRGLWRGPSTRANYTLDFRLNGIPTGTNTLEILYNPV  385
ADJ10931.1   306   NSLQGLAAQTDMTFEQARNAYLGAADMIEQGLPLLPPLRSAYAPRGLWRGPSTRANYTLDFRLNGIPTGTNTLEILYNPV  385
ADJ10938.1   306   NSLQGLAAQTDMTFEQARNAYLGAADMIEQGLPLLPPLRSAYAPRGLWRGPSTRANYTLDFRLNGIPTGTNTLEILYNPV  385
QYU59229.1   306   NSLQGLAAQTDMTFEQARNAYLGAADMIEQGLPLLPPLRSAYAPRGLWRGPSTRANYTLDFRLNGIPTGTNTLEILYNPV  385
ACZ58630.1   306   NSLQGLAAQTDMTFEQARNAYLGAADMIEQGLPLLPPLRSAYAPRGLWRGPSTRANYTLDFRLNGIPTGTNTLEILYNPV  385
QGP72535.1   306   NSLQGLAAQTDMTFEQARNAYLGAADMIEQGLPLLPPLRSAYAPRGLWRGPSTRANYTLDFRLNGIPTGTNTLEILYNPV  385
Q91HK5.1     306   NSLQGLAAQTDMTFEQARNAYLGAADMIEQGLPLLPPLRSAYAPRGLWRGPSTRANYTLDFRLNGIPTGTNTLEILYNPV  385
ARO69912.1   306   NSLQGLAAQTDMTFEQARNAYLGAADMIEQGLPLLPPLRSAYAPRGLWRGPSTRANYTLDFRLNGIPTGTNTLEILYNPV  385
AFI71814.1   306   NSLQGLAAQTDMTFEQARNAYLGAADMIEQGLPLLPPLRSAYAPRGLWRGPSTRANYTLDFRLNGIPTGTNTLEILYNPV  385
ADJ10932.1   306   NSLQGLAAQTDMTFEQARNAYLGAADMIEQGLPLLPPLRSAYAPRGLWRGPSTRANYTLDFRLNGIPTGTNTLEILYNPV  385
ARO69925.1   306   NSLQGLAVQTDMTFEQARNAYLGAADMIEQGLPLLPPLRSAYAPRGLWRGPSTRANYTLDFRLNGIPTGTNTLEILYNPV  385
ARO69911.1   306   NSLQGLAAQTDMTFEQARNAYLGAADMIEQGLPLLPPLRSAYAPRGLWRGPSTRANYTLDFRLNGIPTGTNTLEILYNPV  385
ACZ58628.1   306   NSLQGLAAQTDMTFEQARNAYLGAADMIEQGLPLLPPLRSAYAPRGLWRGPSTRANYTLDFRLNGIPTGTNTLEILYNPV  385
ABI63932.1   306   NSLQGLAAQTDMTFEQARNAYLGAADMIEQGLPLLPPLRSAYAPRGLWRGPSTRANYTLDFRLNGIPTGTNTLEILYNPV  385
ARO69945.1   306   NSLQGLAAQTDMTFEQARNAYLGAADMIEQGLPLLPPLRSAYAPRGLWRGPSTRANYTLDFRLNGIPTGTNTLEILYNPV  385
ARO69930.1   306   NSLQGLAAQTDMTFEQARNAYLGAADMIEQGLPLLPPLRSAYAPRGLWRGPSTRANYTLDFRLNGIPTGTNTLEILYNPV  385
ABI63925.1   306   NSLQGLAAQTDMTFEQARNAYLGAADMIEQGLPLLPPLRSAYAPRGLWRGPSTRANYTLDFRLNGIPTGTNTLEILYNPV  385
ARO69906.1   306   NSLQGLAVQTDMTFEQARNAYLGAADMIEQGLPLLPPLRSAYAPRGLWRGPSTRANYTLDFRLNGIPTGTNTLEILYNPV  385
ACZ58627.1   306   NSLQGLAAQTDMTFEQARNAYLGAADMIEQGLPLLPPLRSAYAPRGLWRGPSTRANYTLDFRLNGIPTGTNTLEILYNPV  385
ARO69920.1   306   NSLQGLAVQTDMTFEQARNAYLGAADMIEQGLPLLPPLRSAYAPRGLWRGPSTRANYTLDFRLNGIPTGTNTLEILYNPV  385
ACZ58629.1   306   NSLQGLAAQTDMTFEQARNAYLGAADMIEQGLPLLPPLRSAYAPRGLWRGPSTRANYTLDFRLNGIPTGTNTLEILYNPV  385
AGT42201.1   306   NSLQGLAAQTDMTFEQARNAYLGAADMIEQGLPLLPPLRSAYAPRGLWRGPSTRANYTLDFRLNGIPTGTNTLEILYNPV  385
ARO69914.1   306   NSLQGLAAQTDMTFEQARNAYLGAADMIEQGLPLLPPLRSAYAPRGLWRGPSTRANYTLDFRLNGIPTGTNTLEILYNPV  385
ABI63916.1   306   NSLQGLAAQTDMTFEQARNAYLGAADMIEQGLPLLPPLRNAYAPRGLWRGPSTRANYTLDFRLNGIPTGTNTLEILYNPV  385
ARO69918.1   306   NSLQGLAAQTDMTFEQARNAYLGAADMIEQGLPLLPPLRSAYAPRGLWRGPSTRANYTLDFRLNGIPTGTNTLEILYNPV  385
ARO69948.1   306   NSLQGLAAQTDMTFEQARNAYLGAADMIEQGLPLLPPLRSAYAPRGLWRGPSTRANYTLDFRLNGIPTGTNTLEILYNPV  385
QYA72353.1   306   NSLQGLAAQTDMTFEQARNAYLGAADMIEQGLPLLPPLRNAYAPRGLWRGPSTRANYTLDFRLNGIPTGTNTLEILYNPV  385
ABI63917.1   306   NSLQGLAAQTDMTFEQARNAYLGAADMIEQGLPLLPPLRSAYAPRGLWRGPSTRANYTLDFRLNGIPTGTNTLEILYNPV  385
ARO69941.1   306   NSLQGLAAQTDMTFEQARNAYLGAADMIEQGLPLLPPLRSAYAPRGLWRGPSTRANYTLDFRLNGIPTGTNTLEILYNPV  385
ABI63926.1   306   NSLQGLAAQTDMTFEQARNAYLGAADMIEQGLPLLPPLRSAYAPRGLWRGPSTRANYTLDFRLNGIPTGTNTLEILYNPV  385
CDN67471.1   306   NSLQGLAAQTDMTFEQARNAYLGAADMIEQGLPLLPPLRSAYAPRGLWRGPSTRANYTLDFRLNGIPTGTNTLEILYNPV  385
ABI63920.1   306   NSLQGLAAQTDMTFEQARNAYLGAADMIEQGLPLLPPLRSAYAPRGLWRGPSTRANYTLDFRLNGIPTGTNTLEILYNPV  385
ABI63927.1   306   NSLQGLAAQTDMTFEQARNAYLGAADMIEQGLPLLPPLRSAYAPRGLWRGPSTRANYTLDFRLNGIPTGTNTLEILYNPV  385
ARO69908.1   306   NSLQGLAAQTDMTFEQARNAYLGAADMIEQGLPLLPPLRSAYAPRGLWRGPSTRANYTLDFRLNGIPTGTNTLEILYNPV  385
ARO69916.1   306   NSLQGLAAQTDMTFEQARNAYLGAADMIEQGLPLLPPLRSAYAPRGLWRGPSTRANYTLDFRLNGIPTGTNTLEILYNPV  385
ARO69910.1   306   NSLQGLAAQTDMTFEQARNAYLGAADMIEQGLPLLPPLRNAYAPRGLWRGPSTRANYTLDFRLNGIPTGTNTLEILYNPV  385
DAZ85720.1   306   NSLQGLAAQTDMTFEQARNAYLGAADMIEQGLPLLPPLRSAYAPRGLWRGPSTRANYTLDFRLNGIPTGTNTLEILYNPV  385
AAX18708.1   306   NSLQGLAAQTDMTFEQARNAYLGAADMIEQGLPLLPPLRSAYAPRGLWRGPSTRANYTLDFRLNGIPTGTNTLEILYNPV  385
AYA22298.1   306   NSLQGLAAQTDMTFEQARNAYLGAADMIEQGLPLLPPLRSAYAPRGLWRGPSTRANYTLDFRLNGIPTGTNTLEILYNPV  385
ABI63939.1   306   NSLQGLAAQTDMTFEQARNAYLGAADMIEQGLPLLPPLRSAYAPRGLWRGPSTRANYTLDFRLNGIPTGTNTLEILYNPV  385
ARO69926.1   306   NSLQGLAAQTDMTFEQARNAYLGAADMIEQGLPLLPPLRSAYAPRGLWRGPSTRANYTLDFRLNGIPTGTNTLEILYNPV  385
QGP72537.1   306   NSLQGLAAQTDMTFEQARNAYLGAADMIEQGLPLLPPLRSAYAPRGLWRGPSTRANYTLDFRLNGIPTGTNTLEILYNPV  385
ARO69947.1   306   NSLQGLAAQTDMTFEQARNAYLGAADMIEQGLPLLPPLRSAYAPRGLWRGPSTRANYTLDFRLNGIPTGTNTLEILYNPV  385
ARO69921.1   306   NSLQGLAAQTDMTFEQARNAYLGAADMIEQGLPLLPPLRSAYAPRGLWRGPSTRANYTLDFRLNGIPTGTNTLEILYNPV  385
ARO69946.1   306   NSLQGLAAQTDMTFEQARNAYLGAADMIEQGLPLLPPLRSAYAPRGLWRGPSTRANYTLDFRLNGIPTGTNTLEILYNPV  385
QGP72536.1   306   NSLQGLAAQTDMTFEQARNAYLGAADMIEQGLPLLPPLRNAYAPRGLWRGPSTRANYTLDFRLNGIPTGTNTLEILYNPV  385
QGP72538.1   306   NSLQGLAAQTDMTFEQARNAYLGAADMIEQGLPLLPPLRSAYAPRGLWRGPSTRANYTLDFRLNGIPTGTNTLEILYNPV  385
ARO69913.1   306   NSLQGLAAQTDMTFEQARNAYLGAADMIEQGLPLLPPLRSAYAPRGLWRGPSTRANYTLDFRLNGIPTGTNTLEILYNPV  385
ARO69924.1   306   NSLQGLAAQTDMTFEQARNAYLGAADMIEQGLPLLPPLRSAYAPRGLWRGPSTRANYTLDFRLNGIPTGTNTLEILYNPV  385
ABI63924.1   306   NSLQGPAAQTDMTFEQARNAYLGAADMIEQGLPLLPPLRSAYAPRGLWRGPSTRANYTLDFRLNGIPTGTNTLEILYNPV  385
ARO69931.1   306   NSLQGLAAQTDMTFEQARNAYLGAADMIEQGLPLLPPLRSAYAPRGLWRGPSTRANYTLDFRLNGIPTGTNTLEILYNPV  385
AWO67711.1   305   NSLQGLAAQTDMTFEQARNAYLGAADMIEQGLPLLPPLRSAYAPRGLWRGPSTRANYTLDFRLNGIPTGTNTLEILYNPV  384
ARO69943.1   306   NSLQGLAAQTDMTFEQARNAYLGAADMIEQGLPLLPPLRSAYAPRGLWRGPSTRANYTLDFRLNGIPTGTNTLEILYNPV  385
ADJ10928.1   306   NSLQGPAAQTDMTFEQARNAYLGAADMIEQGLPLLPPLRSAYAPRGLWRGPSTRANYTLDFRLNGIPTGTNTLEILYNPV  385
ARO69939.1   306   NSLQGLAAQTDMTFEQARNAYLGAADMIEQGLPLLPPLRSAYAPRGLWRGPSTRANYTLDFRLNGIPTGTNTLEILYNPV  385
ABI63938.1   306   NSLQGLAAQTDMTFEQARNAYLGAADMIEQGLPLLPPLRSAYAPRGLWRGPSTRANYTLDFRLNGIPTGTNTLEILYNPV  385
ABI63940.1   306   NSLQGLAAQTDMTFEQARNAYLGAADMIEQGLPLLPPLRSAYAPRGLWRGPSTRANYTLDFRLNGIPTGTNTLEILYNPV  385
ABI63929.1   306   NSLQGLAAQTDMTFEQARNAYLGAADMIEQGIPLLPPLRSAYAPRGLWRGPSTRANYTLDFRLSGIPTGTNTLEILYNPV  385
ABI63919.1   306   NSLQGLAAQTDMTFEQARNAYLGAADMIEQGLPLLPPLRSAYAPRGLWRGPSTRANYTLDFRLNGIPTGTNTLEILYDPV  385
ADJ10934.1   304   NSLQGLAAQTDMTFEQARNAYLGAADMIEQGLPLLPPLRSAYAPRGLWRGPSTRANYTLDFRLNGIPTGTNTLEILYNPV  383
ARO69937.1   306   NSLQGLAAQTDMTFEQARNAYLGAADMIEQGLPLLPPLRSAYAPRGLWRGPSTRANYTLDFRLNGIPTGTNTLEILYNPV  385
ARO69928.1   306   NSLQGLAAQTDMTFEQARNAYLGAADMIEQGLPLLPPLRNAYAPRGLWRGPSTRANYTLDFRLNGIPTGTNTLEILYNPV  385
ARO69927.1   306   NSLQGLAAQTDMTFEQARNAYLGAADMIEQGLPLLPPLRSAYAPRGLWRGPSTRANYTLDFRLNGIPTGTNTLEILYNPV  385
ABI63934.1   305   NSLQGLAAQTDMTFEQARNAYLGAADMIEQGLPLLPPLRSAYAPRGLWRGPSTRANYTLDFRLNGIPTGTNTLEILYNPV  384
ARO69935.1   306   NSLQGLAAQTDMTFEQARNAYLGAADMIEQGLPLLPPLRSAYAPRGLWRGPSTRANYTLDFRLNGIPTGTNTLEILYNPV  385
ARO69940.1   306   NSLQGLAAQTDMTFEQARNAYLGAADMIEQGLPLLPPLRNAYAPRGLWRGPSTRANYTLDFRLNGIPTGTNTLEILYNPV  385
ARO69933.1   306   NSLQGLAAQTDMTFEQARNAYLGAADMIEQGLPLLPPLRNAYAPRGLWRGPSTRANYTLDFRLNGIPTGTNTLEILYNPV  385
ABI63922.1   306   NSLQGLAAQTDMTFEQARNAYLGAADMIEQGLPLLPPLRSAYAPRGLWRGPSTRTNYTLDFRLNGIPTGTNTLEILYNPV  385
ABI63936.1   306   NSLQGLAAQTDMTFEQARNAYLGAADMIEQGLPLLPPLRSAYAPRGLWRGPSTRANYTLDFRLNGIPTGTNTLEILYNPV  385
ABI63918.1   306   NSLQGLAAQTDMTFEQARNAYLGAADMIEQGLPLLPPLRSAYAPRGLWRGPSTRANYTLDFRLNGIPTGTNTLEILYNPV  385
```

```
ABI63931.1   305   NSLQGLAAQTDMTFEQARNAYLGAADMIEQGLPLLPPLRSAYAPRGLWRGPSTRANYTLDFRLNGIPTGTNTLEILYNPV  384
QYU59230.1   306   NSLQGLAAQTDMTFEQARNAYLGAADMIEQGLPLLPPLRSAYAPRGLWRGPSTRANYTLDFRLNGIPTGTNTLEILYNPV  385
ARO69922.1   306   NSLQGLAAQTDMTFEQARNAYLGAADMIEQGLPLLPPLRNAYAPRGLWRGPSTRANYTLDFRLNGIPTGTNTLEILYNPV  385
ABI63941.1   306   NSLQGLAAQTDMTFEQARNAYLGAADMIEQGLPLLPPLRSAYAPRGLWRGPSTRANYTLDFRLNGIPTGTNTLEILYNPV  385
ARO69949.1   306   NSLQGLAAQTDMTFEQARNAYLGAADMIEQGLPLLPPLRSAYAPRGLWRGPSTRANYTLDFRLNGIPTGTNTLEILYNPV  385
ARO69917.1   306   NSLQGLAAQTDMTFEQARNAYLGAADMIEQGLPLLPPLRNAYAPRGLWRGPSTRANYTLDFRLNGIPTGTNTLEILYNPV  385
ARO69915.1   306   NSLQGLAAQTDMTFEQARNAYLGAADMIEQGLPLLPPLRNAYAPRGLWRGPSTRANYTLDFRLNGIPTGTNTLEILYNPV  385
QYU59228.1   306   NSLQGLAAQTDMTFEQARNAYLGAADMIEQGLPLLPPLRSAYAPRGLWRGPSTRANYTLDFRLDGIPTGTNTLEILYNPV  385
AGN29358.1   306   NSLQGLAAQTDMTFEQARNAYLGAADMIEQGLPLLPPLRSAYAPRGLWRGPSTRANYTLDFRLNGIPTGTNTLEILYNPV  385
ARO69944.1   306   NSLQGLAAQTDMTFEQARNAYLGAADMIEQGLPLLPPLRNAYAPRGLWRGPSTRANYTLDFRLNGIPTGTNTLEILYNPV  385
AGN29367.1   303   NSLQGLAAQTDMTFEQARNAYLGAADMIEQGLPLLPPLRSAYAPRGLWRGPSTRANYTLDFRLNGIPTGTNTLEILYNPV  382
ABI63921.1   306   NSLQGLAAQTDMTFEQARNAYLGAADMIEQGLPLLPPLRSAYAPRGLWRGPSTRANYTLDFRLNGIPTGTNTLEILYNPV  385
ABI63923.1   306   NSLQGLAAQTDMTFEQARNAYLGAADMIEQGLPLLPPLRSAYAPRGLWRGPSTRANYTLDFRLNGIPTGTNTLEILYNPV  385
CDN67470.1   303   NSLQGLAAQTDMTFEQARNAYLGAADMIEQGLPLLPPLRSAYAPRGLWRGPSTRANYTLDFRLNGIPTGTNTLEILYNPV  382
CDN67474.1   306   NSLQGLAAQTDMTFEQARNAYLGAADMIEQGLPLLPPLRSAYAPRGLWRGLSTRANYALDFRLNGIPTGTNTLEILYNPV  385
AAX18710.1   306   NSLQGLAAQTDMTFEQARNAYLGAADMIEQGLPLLPPLRSAYAPRGLWRGPSTRANYTLDFRLNGIPTGTNTLEILYNPV  385
QBZ78370.1   304   NSLQGLAAQTDMTFEQARNAYLGAADMIEQGLPLLPPLRSAYAPRGLWRGPSTRANYTLDFRLNGIPTGTNTLEILYNPV  383
ARO69923.1   306   NSLQGLAAQTDMTFEQARNAYLGAADMIEQGLPLLPPLRSAYAPRGLWRGPSTRANYTLDFRLNGIPTGTNTLEILYNPV  385
ARO69907.1   306   NSLQGLAAQTDMTFEQARNAYLGAADMIEQGLPLLPPLRNAYAPRGLWRGPSTRANYTLDFRLNGIPTGTNTLEILYNPV  385
ARO69942.1   306   NSLQGLAAQTDMTFEQARNAYLGAADMIEQGLPLLPPLRNAYAPRGLWRGPSTRANYTLDFRLNGIPTGTNTLEILYNPV  385
ARO69909.1   306   NSLQGLATQTDMTFEQARNAYLGAADMIEQGLPLLPPLRNAYAPRGLWRGPSTRANYTLDFRLNGIPTGTNTLEILYNPV  385
AAX18711.1   306   NSLQGLAAQTDMTFEQARNAYLGAADMIEQGLPLLPPLRSAYAPRGLWRGPSTRANYTLDFRLNGIPTGTNTLEILYNPV  385
ARO69936.1   306   NSLQGLAAQTDMTFEQARNAYLGAADMIEQGLPLLPPLRSAYAPRGLWRGPSTRANYTLDFRLNGIPTGTNTLEILYNPV  385
AGN29357.1   306   NSLQSLAAQTDMTFEQARNAYLGAADMIEQGLPLLPPLRSAYAPRGLWRGPSTRANYTLDFRLNGIPTGTNTLEILYNPV  385
ARO69919.1   306   NSLQGLAAQTDMTFEQARNAYLGAADMIEQGLPLLPPLRNAYAPRGLWRGPSTRANYTLDFRLNGIPTGTNTLEILYNPV  385
ADJ10929.1   306   NSLQGLAAQTDVTFEQARNAYLGAADMIEQGLPLLPPLRNAYAPRGLWRGPSTRANYTLDFRLNGIPTGTNTLEILYNPV  385
ABI63943.1   306   NSLQGLAAQTDMTFEQARNAYLGAADMIEQGLPLLPPLRSAYAPRGLWRGPSTRANYTLDFRLNGIPTGTNTLEILYNPV  385
AGN29365.1   306   NSLQGLAAQTDMTFEQARNAYLGAADMIEQGLPLLPPLRNAYAPRGLWRGPSTRANYTLDFRLNGIPTGTNTLEILYNPV  385
ABI63930.1   305   NSLQGLAAQTDMTFEQARNAYLGAADMIEQGLPLLPPLRSAYAPRGLWRGPSTRANYTLDFRLNGIPTGTNTLEILYNPV  384
ABI63942.1   305   NSLQGLAAQTDMTFEQARNAYLGAADMIEQGLPLLPPLRSAYAPRGLWRGPSTRANYTLDFRLNGIPTGTNTLEILYNPV  384
ADJ10939.1   306   NSLQGLAAQTDMTFEQARNAYLGAADMIEQGLPLLPPLRSAYAPRGLWRGPSTRANYTLDFRLNGIPTGTNTLEILYNPV  385
ABI63928.1   305   NSLQGLAAQTDMTFEQARNAYLGAADMIEQGLPLLPPLRSAYAPRGLWRGPSTRANYTLDFRLNGIPTGTNTLEILYNPV  384
CDN67476.1   303   NSLQGLAAQTDMTFEQARNAYLGAADMIEQGLPLLPPLRSAYAPRGLWRGPSTRANYTLDFRLNGIPTGTNTLEILYNPV  382
ABI63933.1   305   NSLQGLAAQTDMTFEQARNAYLGAADMIEQGLPLLPPLRSAYAPRGLWRGPSTRANYTLDFRLNGIPTGTNTLEILYNPV  384
AAX18707.1   306   NSLQGLAAQTDMTFEQARNAYLGAADMIEQGLPLLPPLRSAYAPRGLWRGPSTRANYTLDFRLNGIPTGTNTLEILYNPV  385
ACZ58631.1   304   NSLQGLAAQTDMTFEQARNAYLGAADMIEQGLPLLPPLRNAYAPRGLWRGPSTRANYTLDFRLNGIPTGTNTLEILYNPV  383
AGN29359.1   306   NSLQGLAAQTDMTFEQARNAYLGAADMIEQGLPLLPPLRSAYAPRGLWRGPSTRANYTLDFRLNGIPTGTNTLEILYNPV  385
AGN29361.1   306   NSLQGLAAQTDMTFEQARNAYLGAADMIEQGLPLLPPLRSAYAPRGLWRGPSTRANYTLDFRLNGIPTGTNTLEILYNPV  385
ABI63935.1   305   NSLQGLAAQTDMTFEQARNAYLGAADMIEQGLPLLPPLRSAYAPRGLWRGPSTRANYTLDFRLNGIPTGTNTLEILYNPV  384
AGN29360.1   307   NSLQGLAAHTDMTFEQARNAYLGAADMIEQGLPLLPPLRSAYAPRGLWRGPSTRANYTLDFRLNGIPTGTNTLEILYNPV  386
AWO67712.1   305   NSLQGLAAQTDMTFEQARNAYLGAADMIEQGLPLLPPLRNAYAPRGLWRGPSTRANYTLDFRLNGIPTGTNTLEILYNPV  384
AYA22300.1   303   NSLQGLAAQTDMTFEQARNAYLGAADMIEQGLPLLPPLRSAYAPRGLWRGPSTRANYTLDFRLNGIPTGTNTLEILYNPV  382
QBZ78373.1   303   NSLQGLAAQTDMTFEQARNAYLGAADMIEQGLPLLPPLRNAYAPRGLWRGPSTRANFTLDFRLNGIPTGTNTLEIMYNPV  382
AYQ93056.1   303   NSLQGLAAQTDMTFEQARNAYLGAADMIEQGLPLLPPLRSAYAPRGLWRGPSTRANYTLDFRLNGIPTGTNTLEILYNPV  382
AYQ93054.1   303   NSLQGLAAQTDMTFEQARNAYLGAADMIEQGLPLLPPLRSAYAPRGLWRGPSTRANYTLDFRLNGIPTGTNTLEILYNPV  382
AAX18709.1   303   NSLQGLAAQTDMTFEQARNAYLGAADMIEQGLPLLPPLRSAYAPRGLWRGPSTRANYTLDFRLNGIPTGTNTLEILYNPV  382
AYQ93052.1   304   NSLQGLAAQTDMTFEQARNAYLGAADMIEQGLPLLPPLRSAYAPRGLWRGPSTRANYTLDFRLNGIPTGTNTLEILYNPV  383
BCT87062.1   305   NSLQGLAAQTDMTFEQARNAYLGAADMIEQGLPLLPPLRSAYAPRGLWRGPSTRANYTLDFRLNGIPTGTNTLEIMYNPV  384
AGN29364.1   303   NSLRGLAAQTDMTFEQARNAYLGAADMIEQGLPLLPPLRGAYAPRGLWRGPSTRANYTLDFRLNGIPTGTNTLEILYNPV  382
QYU59234.1   303   NSLQGLAAQTDMTFEQARNAYLGAADMIEQGLPLLPPLRSAYAPRGLWRGPSTRANYTLDFRLNGIPTGTNTLEILYNPV  382
CDN67475.1   303   NSLQGLAAQTDMTFEQARNAYLGAADMIEQGLPLLPPLRSAYAPRGLWRGPSTRANYTLDLRLNGIPTGTNTLEILYNPV  382
AGN29356.1   303   NSLQGLAAQTDMTFEQARNAYLGAADMIEQGLPLLPPLRNAYAPRGLWRGPSTRANYTLDFRLNGIPTGTNTLEILYNPV  382
AGN29363.1   303   NSLRGLAAQTDMTFEQARNAYLGAADMIEQGLPLLPPLRSAYAPRGLWRGPSTRANYTLDFRLNGIPTGTNTLEILYNPV  382
AFV73369.1   306   NSLQGLAAQTDMTFEQARNAYLGAADMIEQGLPLLPPLRNAYAPRGLWRGPSTRANYTLDFRLNGIPTGTNTLEILYNPV  385
AFV34732.1   303   NSLQGLAAQTDMTFEQARNAYLGAADMIEQGLPLLPPLRSAYAPRGLWRGPSTRANYTLDFRLNGIPTGTNTLEILYNPV  382
AFI43912.1   307   NSLQGLAAQTDMTFEQARNAYLGAADMIEQGLPLLPPLRSAYAPRGLWRGPSTRANYTLDFRLNGIPTGTNTLEIMYNPV  386
CDN67472.1   303   NSLQGLAAQTDMTFEQARNAYLGAADMIEQGLPLLPPLRSTYAPRGLWRGPSTRANYTLDFRLNGIPTGTNTLEILYNPV  382
AGN29355.1   303   NSLQGLAAQTDMTFEQARNAYLGAADMIEQGLPLLPPLRSAYAPRGLWRGPSTRANYTLDFRLNGIPTGTNTLEILYNPV  382
AFI43911.1   307   NSLQGLAAQTDMTFEQARNAYLGAADMIEQGLPLLPPLRSAYAPRGLWRGPSTRANYTLDFRLNGIPTGANTLEIMYNPV  386
ADJ10940.1   306   QSLQGLAAQTDMTFEQARNAYLGAADMIEQGLPLLPPLRNAYMPRGLWRGPSTRANYTLDFRLNGIPTGENTLEILYNPV  385
DAZ85718.1   306   QSLQGLAAQTDMTFEQARNAYLGAADMIEQGLPLLPPLRNAYMPRGLWRGPSTRANYTLDFRLNGIPTGENTLEILYNPV  385
ADJ10930.1   306   QSLQGLAAQTDMTFEQARNAYLGAADMIEQGLPLLPPLRNAYMPRGLWRGPSTRANYTLDLRLNGIPTGENTLEILYNPV  385
ADJ10933.1   306   QSLQGLAAQTDMTFEQARNAYLGAADMIEQGLPLLPPLRNAYMPRGLWRGPSTRANYTLDFRLNGIPTGENTLEILYNPV  385
ADJ10935.1   306   QSLQGLAAQTDMTFEQARNAYLGAADMIEQGLPLLPPLRNAYMPRGLWRGPSTRANYTLDFRLNGIPTGENTLEILYNPV  385
ADJ10941.1   306   QSLQGLAAQTDMTFEQARNAYLGAADMIEQGLPLLPPLRNAYTPRGLWRGPSTRANYTLDFRLNGIPTGENTLEILYNPV  385
DAZ85719.1   306   QSLQGLAAQTDMTFEQARNAYLGAADMIEQGLPLLPPLRNAYMPRGLWRGPSTRANYTLDFRLNGIPTGENTLEILYNPV  385
ADJ10936.1   306   QSLRGLAAQTDMTFEQARNAYLGAADMIEQGLPLLPPLGNAYMPRGLWRGPSTRANYTLDFRLNGIPTGENTLEILYNPV  385
ABR09917.1   306   QSLQGLAAQTDMTFEQARNAYLGAADMIEQGLPLLPPLRNAYMPRGLWRGPSTRANYTLDFRLNGIPTGENTLEILYNPV  385
CDN67473.1   311   NSLQGPAAQTDMTFEQARNAYLGAADMIEQGLPLLPPLRSAYAPRGLWRGPSTRANYTLDFRLNGIPTGTNTLEILYNPV  390
QYU59232.1   303   NSLQNLAVHTDMTFEQARNAYLGAADMIEQGLPLLPPLRNAYAPRGLWRGPSTRANFTLDFRLNGIPTGENTLEILYNPV  382
AGN29362.1   303   NSLQGLAAQTDMTFEQARNAYLGAADMIEQGLPLLPPLRSAYAPRGLWRGPSTRANYTLDFRLNGIPTGTNTLEILYNPV  382
ACZ58625.1   305   NSLQGLAAQTEMTFEQARNAYLGAADMVEQGLPLLPPLRSAYAPRGLWRGPSTRANYTLDFRLNGIPTGTNTLEILYNPV  384
ACZ58626.1   305   NSLQGLAAQTEMTFEQARNAYLGAADMIEQGLPLLPPLRSAYAPRGLWRGPSTRANYTLDFRLNGIPTGTNTLEILYNPV  384
ACZ58624.1   305   NSLQSLAAQTEMTFEQTRNAYLGAADMVEQGLPLLPPLRSAYAPRGLWRGPSTRANYTLDFRLNGIPTGTNTLEILYNPV  384
AGN29366.1   306   NSLQSLAAQTDMTFEQARNAYLGAADMIEQGLPLLPPLRSAYAPRGLWRGPSTRANYTLDFRLNGIPTGTNTLEILYNPV  385
QBZ78368.1   305   NSLQGLAAQTEMTFEQARNAYLGAADMVEQGLPLLPPLRSAYAPRGLWRGPSTRANYTLDFRLNGIPTGTNTLEILYNPV  384
AFI43914.1   314   NSLQGLAAQTDMSFEQARNAYLGAADMIEQGLPLLPPLRSAYAPRGLWRGPSTRANYTLDFRLNGTPTGTNTLEILYNPV  393
AFI43913.1   314   NSLQGLAAQTDMTFEQARNAYLGAADMIEQGLLLLPPLRSAYAPRGLWRGPSTRANYTLDFRLNGIPTGTNTLEILYNPV  393
AFV34756.1   245   NSLQGLAAQTDMTFEQARNAYLGAADMIEQGLPLLPPLRNAYAPRGLWRGPSTRANYTLDFRLNGIPTGINTLEILYNPV  324
QYU59231.1   305   NSLQNLAVNTDMTFEQARNAYLGAADMIEQGLPLLPPLRNAYAPRGLWRGPSTRANFTLDFRLNGIPTGENTLEILYNPV  384
QYU59233.1   312   NSLQSLAVNTDMTFEQARNAYLGAADMIEQGLPLLPPLRNAYAPRGLWRGPSTRANFTLDFRLNGIPTGENTLEILYNPV  391
APD26307.1   314   NSLQGLAAQTDMTFEQARNAYLGAADMIEQGLPLLPPLSSAYAPRGLWRGPSTRANYTLDFRLSGIPTGTNTLEILYNPV  393
QBZ78371.1   134   NSLQGLAAQTDMTFEQARNAYLGAADMIEQGLPLLPPLRNAYAPRGLWRGPSTRANYTLDFRLNGIPTGTNTLEILYNPV  213


NP_619706.1  385   SEEEMEEYRDRGMSAVVIDALEIAINPFGMPGNPTDLTVVATYGHERDMTRAFIGSASTFLGNGLARAIFFPGLQYSQEE  464
AWO68043.1   386   SEEEMEEYRDRGMSAVVIDALEIAINPFGMPGNPTDLTVVATYGHERDMTRAFIGSASTFLGNGLARAIFFPGLQYSQEE  465
ADJ10937.1   386   SEEEMEEYRDRGMSAVVIDALEIAINPFGMPGNPTDLTVVATYGHERDMTRAFIGSASTFLGNGLARAIFFPGLQYSQEE  465
```

```
ADJ10931.1   386   SEEEMEEYRDRGMSAVVIDALEIAINPFGMPGNPTDLTVVATYGHERDMTRAFIGSASTFLGNGLARAIFFPGLQYSQEE  465
ADJ10938.1   386   SEEEMEEYRDRGMSAVVIDALEIAINPFGMPGNPTDLTVVATYGHERDMTRAFIGSASIFLGNGLARAIFFPGLQYSQEE  465
QYU59229.1   386   SEEEMEEYRDRGMSAVVIDALEIAINPFGMPGNPTDLTVVATYGHERDMTRAFIGSASTFLGNGLARAIFFPGLQYSQEE  465
ACZ58630.1   386   SEEEMEEYRDRGMSAVVIDALEIAINPFGMPGNPTDLTVVATYGHERDMTRAFIGSASTFLGNGLARAIFFPGLQYSQEE  465
QGP72535.1   386   SEEEMEEYRDRGMSAVVIDALEIAINPFGMPGNPTDLTVVATYGHERDMTRAFIGSASTFLGNGLARAIFFPGLQYSQEE  465
Q91HK5.1     386   SEEEMEEYRDRGMSAVVIDALEIAINPFGMPGNPTDLTVVATYGHERDMTRAFIGSASTFLGNGLARAIFFPGLQYSQEE  465
ARO69912.1   386   SEEEMEEYRDRGMSAVVVDALEIAINPFGMPGNPTDLTVVATYGHERDMTRAFIGSASTFLGNGLARAIFFPGLQYSQEE  465
AFI71814.1   386   SEEEMEEYRDRGMSAVVIDALEIAINPFGMPGNPTDLTVVATYGHERDMTRAFIGSASTFLGNGLARAIFFPGLQYSQEE  465
ADJ10932.1   386   SEEEMEEYRDRGMSAVVIDALEIAINPFGMPGNPTDLTVVATYGHERDMTRASIGSASTFLGNGLARAIFFPGLQYSQEE  465
ARO69925.1   386   SEEEMEEYRDRGMSAVVIDALEIAINPFGMPGNPTDLTVVATYGHERDMTRAFIGSASTFLGNGLARAIFFPGLQYSQEE  465
ARO69911.1   386   SEEEMEEYRDRGMSAVVVDALEIAINPFGMPGNPTDLTVVATYGHERDMTRAFIGSASTFLGNGLARAIFFPGLQYSQEE  465
ACZ58628.1   386   SEEEMEEYRDRGMSAVVIDALEIAINPFGMPGNPTDLTVVATYGHERDMTRAFIGSASTFLGNGLARAIFFPGLQYSQEE  465
ABI63932.1   386   SEEEMEEYRDRGMSAVVVDALEIAINPFGMPGNPTDLTVVATYGHERDMARAFIGSASTFLGNGLARAIFFPGLQYSQEE  465
ARO69945.1   386   SEEEMEEYRDRGMSAVVVDALEIAINPFGMPGNPTDLTVVATYGHERDMTRAFIGSASTFLGNGLARAIFFPGLQYSQEE  465
ARO69930.1   386   SEEEMEEYRDRGMSAVVVDALEIAINPFGMPGNPTDLTVVATYGHERDMTRAFIGSASTFLGNGLARAIFFPGLQYSQEE  465
ABI63925.1   386   SEEEMEEYRDRGMSAVVVDALEIAINPFGMPGNPTDLTVVATYGHERDMARAFIGSASTFLGNGLARAIFFPGLQYSQEE  465
ARO69906.1   386   SEEEMEEYRDRGMSAVVIDALEIAINPFGMPGNPTDLTVVATYGHERDMTRAFIGSASTFLGNGLARAIFFPGLQYSQEE  465
ACZ58627.1   386   SEEEMEEYRDRGMSAVVIDALEIAINPFGMPGNPTDLTVVATYGHERDMTRAFIGSASTFLGNGLARAIFFPGLQYSQEE  465
ARO69920.1   386   SEEEMEEYRDRGMSAVVVDALEIAINPFGMPGNPTDLTVVATYGHERDMTRAFIGSASTFLGNGLARAIFFPGLQYSQEE  465
ACZ58629.1   386   SEEEMEEYRDRGMSAVVIDALEIAINPFGMPGNPTDLTVVATYGHERDMTRAFIGSASTFLGNGLARAIFFPGLQYSQEE  465
AGT42201.1   386   SEEEMEEYRDRGMSAVVIDALEIAINPFGMPGNPTDLTVVATYGHERDMTRAFIGSASTFLGNGLARAIFFPGLQYSQEE  465
ARO69914.1   386   SEEEMEEYRDRGMSAVVVDALEIAINPFGMPGNPTDLTVVATYGHERDMTRAFIGSASTFLGNGLARAIFFPGLQYSQEE  465
ABI63916.1   386   SEEEMEEYRDRGMSAVVVDALEIAINPFGMPGNPTDLTVVATYGHERDMARAFIGSASTFLGNGLARAIFFPGLQYSQEE  465
ARO69918.1   386   SEEEMEEYRDRGMSAVVVDALEIAINPFGMPGNPTDLTVVATYGHERDMTRAFIGSASTFLGNGLARAIFFPGLQYSQEE  465
ARO69948.1   386   SEEEMEEYRDRGMSAVVIDALEIAINPFGMPGNPTDLTVVATYGHERDMTRAFIGSASTFLGNGLARAIFFPGLQYSQEE  465
QYA72353.1   386   SEEEMEEYRDRGMSAVVVDALEIAINPFGMPGNPTDLTVVATYGHERDMTRAFIGSASTFLGNGLARAIFFPGLQYSQEE  465
ABI63917.1   386   SEEEMEEYRDRGMSAVVVDALEIAINPFGMPGNPTDLTVVATYGHERDMARAFIGSASTFLGNGLARAIFFPGLQYSQEE  465
ARO69941.1   386   SEEEMEEYRDRGMSAVVIDALEIAINPFGMPGNPTDLTVVATYGHERDMTRAFIGSASTFLGNGLARAIFFPGLQYSQEE  465
ABI63926.1   386   SEEEMEEYRDRGMSAVVVDALEIAINPFGMPGNPTDLTVVATYGHERDMARAFIGSASTFLGNGLARAIFFPGLQYSQEE  465
CDN67471.1   386   SEEEMEEYRDRGMSAVVIDALEIAINPFGMPGNPTDLTVVATYGHERDMTRAFIGSASTFLGNGLARAIFFPGLQYSQEE  465
ABI63920.1   386   SEEEMEEYRDRGMSAVVVDALEIAINPFGMPGNPTDLTVVATYGHERDMARAFIGSASTFLGNGLARAIFFPGLQYSQEE  465
ABI63927.1   386   SEEEMEEYRDRGMSAVVVDALEIAINPFGMPGNPTDLTVVATYGHERDMARAFIGSASTFLGNGLARAIFFPGLQYSQEE  465
ARO69908.1   386   SEEEMEEYRDRGMSAVVIDALEIAINPFGMPGNPTDLTVVATYGHERDMTRAFIGSASTFLGNGLARAIFFPGLQYSQEE  465
ARO69916.1   386   SEEEMEEYRDRGMSAVVIDALEIAINPFGMPGNPTDLTVVATYGHERDMTRAFIGSASTFLGNGLARAIFFPGLQYSQEE  465
ARO69910.1   386   SEEEMEEYRDRGMSAVVIDALEIAINPFGMPGNPTDLTVVATYGHERDMTRAFIGSASTFLGNGLARAIFFPGLQYSQEE  465
DAZ85720.1   386   SEDEMEEYRDRGMSAVVIDALEIAINPFGMPGNPTDLTVVATYGHERDMTRAFIGSASTFLGNGLARAIFFPGLQYSQEE  465
AAX18708.1   386   SEEEMEEYRDRGMSAVVIDALEIAINPFGMPGNPTDLTVVATYGHERDMTRAFIGSASTFLGNGLARAIFFPGLQYSQEE  465
AYA22298.1   386   SEEEMEEYRDRGMSAVVIDALEIAINPFGMPGNPTDLTVVATYGHERDMTRAFIGSASTFLGNGLARAIFFPGLQYSQEE  465
ABI63939.1   386   SEEEMEEYRDRGMSAVVVDALEIAINPFGMPGNPTDLTVVATYGHERDMARAFIGSASTFLGNGLARAIFFPGLQYSQEE  465
ARO69926.1   386   SEEEMEEYRDRGMSAVVIDALEIAINPFGMPGNPTDLTVVATYGHERDMTRAFIGSASTFLGNGLARAIFFPGLQYSQEE  465
QGP72537.1   386   SEEEMEEYRDRGMSAVVIDALEIAINPFGMPGNPTDLTVVATYGHERDMTRAFIGSASTFLGNGLARAIFFPGLQYSQEE  465
ARO69947.1   386   SEEEMEEYRDRGMSAVVIDALEIAINPFGMPGNPTDLTVVATYGHERDMTRAFIGSASTFLGNGLARAIFFPGLQYSQEE  465
ARO69921.1   386   SEEEMEEYRDRGMSAVVIDALEIAINPFGMPGNPTDLTVVATYGHERDMTRAFIGSASTFLGNGLARAIFFPGLQYSQEE  465
ARO69946.1   386   SEEEMEEYRDRGMSAVVIDALEIAINPFGMPGNPTDLTVVATYGHERDMTRAFIGSASTFLGNGLARAIFFPGLQYSQEE  465
QGP72536.1   386   SEEEMEEYRDRGMSAVVIDALELAINPFGMPGNPTDLTVVATYGHERDMTRAFIGSASTFLGNGLARAIFFPGLQYSQEE  465
QGP72538.1   386   SEEEMEEYRDRGMSAVVIDALEIAINPFGMPGNPTDLTVVATYGHERDMTRAFIGSASTFLGNGLARAIFFPGLQYSQEE  465
ARO69913.1   386   SEEEMEEYRDRGMSAVVIDALEIAINPFGMPGNPTDLTVVATYGHERDMTRAFIGSASTFLGNGLARAIFFPGLQYSQEE  465
ARO69924.1   386   SEEEMEEYRDRGMSAVVIDALEIAINPFGMPGNPTDLTVVATYGHERDMTRAFIGSASTFLGNGLARAIFFPGLQYSQEE  465
ABI63924.1   386   SEEEMEEYRDRGMSAVVVDALEIAINPFGMPGNPTDLTVVATYGHERDMARAFIGSASTFLGNGLARAIFFPGLQYSQEE  465
ARO69931.1   386   SEEEMEEYRDRGMSAVVIDALEIAINPFGMPGNPTDLTVVATYGHERDMTRAFIGSASTFLGNGLARAIFFPGLQYSQEE  465
AWO67711.1   385   SEEEMEEYRDRGMSAVVIDALEIAINPFGMPGNPTDLTVVATYGHERDMTRAFIGSASTFLGNGLARAIFFPGLQYSQEE  464
ARO69943.1   386   SEEEMEEYRDRGMSAVVIDALEIAINPFGMPGNPTDLTVVATYGHERDMTRAFIGSASTFLGNGLARAIFFPGLQYSQEE  465
ADJ10928.1   386   SEEEMDEYRDRGMSAVVIDALEIAINPFGMPGNPTDLTVVATYGHERDMTRAFIGSASTFLGNGLARAIFFPGLQYSQEE  465
ARO69939.1   386   SEEEMEEYRDRGMSAVVIDALEIAINPFGMPGNPTDLTVVATYGHERDMTRAFIGSASTFLGNGLARAIFFPGLQYSQEE  465
ABI63938.1   386   SEEEMEEYRDRGMSAVVVDALEIAINPFGMPGNPTDLTVVATYGHERDMARAFIGSASTFLGNGLARAIFFPGLQYSQEE  465
ABI63940.1   386   SEEEMEEYRDRGMSAVVVDALEIAINPFGMPGNPTDLTVVATYGHERDMARAFIGSASTFLGNGLARAIFFPGLQYSQEE  465
ABI63929.1   386   SEEEMEEYRDRGMSAVVVDALEIAINPFGMPGNPTDLTVVATYGHERDMARAFIGSASTFLGNGLARAIFFPGLQYSQEE  465
ABI63919.1   386   SEEEMEEYRDRGMSAVVVDALEIAINPFGMPGNPTDLTVVATYGHERDMVRAFIGSASTFLGNGLARAIFFPGLQYGQGE  465
ADJ10934.1   384   SEEEMEEYRDRGMSAVVIDALEIAINPFGMPGNPTDLTVVATYGHERDMTRAFIGSASTFLGNGLARAIFFPGLQYSQEE  463
ARO69937.1   386   SEEEMEEYRDRGMSAVVIDALEIAINPFGMPGNPTDLTVVATYGHERDMTRAFIGSASTFLGNGLARAIFFPGLQYSQEE  465
ARO69928.1   386   SEEEMEEYRDRGMSAVVIDALEIAINPFGMPGNPTDLTVVATYGHERDMTRAFIGSASTFLGNGLARAIFFPGLQYSQEE  465
ARO69927.1   386   SEEEMEEYRDRGMSAVVIDALEIAINPFGMPGNPTDLTVVATYGHERDMTRAFIGSASTFLGNGLARAIFFPGLQYSQEE  465
ABI63934.1   385   SEEEMEEYRDRGMSAVVVDALEIAINPFGMPGNPTDLTVVATYGHERDMARAFIGSASTFLGNGLARAIFFPGLQYSQEE  464
ARO69935.1   386   SEEEMEEYRDRGMSAVVIDALEIAINPFGMPGNPTDLTVVATYGHERDMKRAFIGSASTFLGNGLARAIFFPGLQYSQEE  465
ARO69940.1   386   SEEEMEEYRDRGMSAVVIDALEIAINPFGMPGNPTDLTVVATYGHERDMTRAFIGSASTFLGNGLARAIFFPGLQYSQEE  465
ARO69933.1   386   SEEEMEEYRDRGMSAVVIDALEIAINPFGMPGNPTDLTVVATYGHERDMTRAFIGSASTFLGNGLARAIFFPGLQYSQEE  465
ABI63922.1   386   SEEEMEEYRDRGMSAVVVDALEIAINPFGMPGNPTDLTVVATYGHERDMARAFIGSASTFLGNGLARAIFFPGLQYSQEE  465
ABI63936.1   386   SEEEMEEYRDRGMSAVVVDALEIAINPFGMPGNPTDLTVVATYGHERDMARAFIGSASTFLGNGLARAIFFPGLQYSQEE  465
ABI63918.1   386   SEEEMEEYRDRGMSAVVVDALEIAINPFGMPGNPTDLTVVATYGHERDMARAFIGSASTFLGNGLARAIFFPGLQYSQEE  465
ABI63931.1   385   SEEEMEEYRDRGMSAVVIDALEIAINPFGMPGNPTDLTVVATYGHERDMTRAFIGSASTFLGNGLARAIFFPGLQYSQEE  464
QYU59230.1   386   SEEEMEEYRDRGMSAVVIDALEIAINPFGMPGNPTDLTVVATYGHERDMTRAFIGSASTFLGNGLARAIFFPGLQYSQEE  465
ARO69922.1   386   SEEEMEEYRDRGMSAVVIDALEIAINPFGMPGNPTDLTVVATYGHERDMTRAFIGSASTFLGNGLARAIFFPGLQYSQEE  465
ABI63941.1   386   SEEEMEEYRDRGMSAVVVDAPEIAINPFGMPGNPTDLTVVATYGHERDMARAFIGSASTFLGNGLARAIFFPGLQYSQEE  465
ARO69949.1   386   SEEEMEEYRDRGMSAVVIDALEIAINPFGMPGNPTDLTVVATYGHERDMTRAFIGSASTFLGNGLARAIFFPGLQYSQEE  465
ARO69917.1   386   SEEEMEEYRDRGMSAVVIDALEIAINPFGMPGNPTDLTVVATYGHERDMTRAFIGSASTFLGNGLARAIFFPGLQYSQEE  465
ARO69915.1   386   SEEEMEEYRDRGMSAVVIDALEIAINPFGMPGNPTDLTVVATYGHERDMTRAFIGSASTFLGNGLARAIFFPGLQYSQEE  465
QYU59228.1   386   SEEEMEEYRDRGMSAVVIDALEIAINPFGMPGNPTDLTVVATYGHERDMTRAFIGSASTFLGNGLARAIFFPGLQYSQEE  465
AGN29358.1   386   SEEEMDEYRDRGMSAVVIDALEIAINPFGMPGNPTDLTVVATYGHERDMTRAFIGSASTFLGNGLARAIFFPGLQYSQEE  465
ARO69944.1   386   SEEEMEEYRDRGMSAVVIDALEIAINPFGMPGNPTDLTVVATYGHERDMTRAFIGSASTFLGNGLARAIFFPGLQYSQEE  465
AGN29367.1   383   SEEEMEEYRDRGMSAVVIDALEIAINPFGMPGNPTDLTVVATYGHERDMTRAFIGSASTFLGNGLARAIFFPGLQYSQEE  462
ABI63921.1   386   SEEEMEEYRDRGMSAVVVDALEIAINPFGMPGNPTDLTVVATYGHERDMARAFIGSASTFLGNGLARAIFFPGLQYSQEE  465
ABI63923.1   386   SEEEMEEYRDRGMSAVVVDALEIAINPFGMPGNPTDLTVVATYGHERDMARAFIGSAATFLGNGLARAIFFPGSQYSQEE  465
CDN67470.1   383   SEEEMEEYRDRGMSAVVIDALEIAINPFGMPGNPTDLTVVATYGHERDMTRALIGSASTFLGNGLARAIFFPGLQYSQEE  462
CDN67474.1   386   SEEEMEEYRDRGMSAVVIDALEIAINPFGMPGNPTDLTVVATYGHERDMTRAFIGSASTFLGNGLARAIFFPGLQYSQEE  465
AAX18710.1   386   SEEEMEEYRDRGMSAVVIDALEIAINPFGMPGNPTDLTVVATYGHERDVTRAFIGSASTFLGNGLARAIFFPGLQYSQEE  465
QBZ78370.1   384   SEEEMEEYRDRGMSAVVIDALEIAINPFGMPGNPTDLTVVATYGHERDMTRAFIGSASTFLGNGLARAIFFPGLQYSQEE  463
ARO69923.1   386   SEEEMEEYRDRGMSAVVIDALEIAINPFGMPGNPTDLTVVATYGHERDMTRAFIGSASTFLGNGLARAIFFPGLQYSQEE  465
ARO69907.1   386   SEEEMEEYRDRGMSAVVIDALEIAINPFGMPGNPTDLTVVATYGHERDMTRAFIGSASTFLGNGLARAIFFPGLQYSQEE  465
```

```
ARO69942.1   386   SEEEMEEYRDRGMSAVVIDALEIAINPFGMPGNPTDLTVVATYGHERDMTRAFIGSASTFLGNGLARAIFFPGLQYSQEE  465
ARO69909.1   386   SEEEMEEYRDRGMSAVVIDALEIAINPFGMPGNPTDLTVVATYGHERDMTRAFIGSASTFLGNGLARAIFFPGLQYSQEE  465
AAX18711.1   386   SEEEMEEYRDRGMSAVVIDALEIAINPFGMPGNPTDLTVVATYGHERDMTRAFIGSASTFLGNGLARAIFFPGLQYSQEE  465
ARO69936.1   386   SEEEMEEYRDRGMSAVVIDALEIAINPFGMPGNPTDLTVVATYGHERDMTRAFIGSASTFLGNGLARAIFFPGLQYSQEE  465
AGN29357.1   386   SEEEMEEYRDRGMSAVVIDALEIAINPFGMPGNPTDLTVVATYGHERDMTRAFIGSASTFLGNGLARAIFFPGLQYSQEE  465
ARO69919.1   386   SEEEMEEYRDRGMSAVVIDALEIAINPFGMPGNPTDLTVVATYGHERDMTRAFIGSASTFLGNGLARAIFFPGLQYSQEE  465
ADJ10929.1   386   SEEEMEEYRDRGMSAVVIDALEIAINPFGMPGNPTDLTVVATYGHERDMTRAFIGSASTFLGNGLARAIFFPGLQYSQEE  465
ABI63943.1   386   SEEEMEEYRDRGMSAVVVDALEIAINPFGMPGNPTDLTVVATYGHERDMARAFIGSASTFLGNGLARAIFFPGLQYSQEE  465
AGN29365.1   386   SEEEMEEYRDRGMSAVVIDALEIAINPFGMPGNPTDLTVVATYGHERDMTRAFIGSASTFLGNGLARAIFFPGLQYSQEE  465
ABI63930.1   385   SEEEMEEYRDRGMSAVVIDALEIAINPFGMPGNPTDLTVVATYGHERDMTRAFIGSASTFLGNGLARAIFFPGLQYSQEE  464
ABI63942.1   385   SEEEMEEYRDRGMSAVVIDALEIAINPFGMPGNPTDLTVVATYGHERDMTRAFIGSASTFLGNGLARAIFFPGLQYSQEE  464
ADJ10939.1   386   SEEEMEEYRDRGMSAVVIDALEIAINPFGMPGNPTDLTVVATYGHERDTTRAFIGSASTFLGNGLARAIFFPGLQYSQEE  465
ABI63928.1   385   SEEEMEEYRDRGMSAVVIDALEIAINPFGMPGNPTDLTVVATYGHERDMTRAFIGSASTFLGNGLARAIFFPGLQYSQEE  464
CDN67476.1   383   SEEEMEEYRDRGMSAVVIDALEIAINPFGMPGNPTDLTVVATYGHERDMTRAFIGSASTFLGNGLARAIFFPGLQYSQEE  462
ABI63933.1   385   SEEEMEEYWDRGMSAVVIDALEIAINPFGMPGNPTDLTVVATYGHERDMTRAFIGSASTFLGNGLARAIFFPGLQYSQEE  464
AAX18707.1   386   SEEEMEEYRDRGMSAVVIDALEIAINPFGMPGNPTDLTVVATYGHERDMTRASIGSASTFLGNGLARAIFFPGLQYSQEE  465
ACZ58631.1   384   SEEEMEEYRDRGMSAVVIDALEIAINPFGMPGNPTDLTVVATYGHERDMTRAFIGSASTFLGNGLARAIFFPGLQYSQEE  463
AGN29359.1   386   SEEEMDEYRDRGMSAVVIDALEIAINPFGMPGNPTDLTVVATYGHERDMTRAFIGSASTFLGNGLARAIFFPGLQYSQEE  465
AGN29361.1   386   SEEEMDEYRDRGMSAVVIDALEIAINPFGMPGNPTDLTVVATYGHERDMTRAFIGSASTFLGNGLARAIFFPGLQYSQEE  465
ABI63935.1   385   SEEEMEEYRDRGMSAVVIDALEIAINPFGMPGNPTDLTVVATYGHERDMTRAFIGSASTFLGNGLARAIFFPGLQYSQEE  464
AGN29360.1   387   SEEEMEEYRDRGMSAVVIDALEIAINTFGMPGIPTGLTVVATYGHERDMTRAFIGSASTFLGNGLARAIFFPGLQYSQEE  466
AWO67712.1   385   SEEEMEEYRDRGMSAVVIDALELAINPFGMPGNPTDLTVVATYGHERDMTRAFIGSASTFLGNGLARAIFFPGLQYSQEE  464
AYA22300.1   383   SEEEMEEYRDRGMSAVVIDALEIAINPFGMPGNPTDLTVVATYGHERDMTRAFIGSASTFLGNGLARAIFFPGLQYSQEE  462
QBZ78373.1   383   SEEEMEEYRDRGMSAVVIDALELAINPFGMPGNPTDLTVVATYGHERDMTRAFIGSASTFLGNGLARAIFFPGLQYSQEE  462
AYQ93056.1   383   SEEEMEEYRDRGMSAVVIDALEIAINPFGMPGNPTDLTVVATYGHERDMTRAFIGSASTFLGNGLARAIFFPGLQYSQEE  462
AYQ93054.1   383   SEEEMEEYRDRGMSAVVIDALEIAINPFGMPGNPTDLTVVATYGHERDMTRAFIGSASTFLGNGLARAIFFPGLQYSQEE  462
AAX18709.1   383   SEEEMEEYRDRGMSAVVIDALEIAINPFGMPGNPTDLTVVATYGHERDMTRAFIGSASTFLGNGLARAIFFPGLQYSQEE  462
AYQ93052.1   384   SEEEMEEYRDRGMSAVVIDALEIAINPFGMPGNPTDLTVVATYGHERDMTRAFIGSASTFLGNGLARAIFFPGLQYSQEE  463
BCT87062.1   385   SEEEMDEYRDRGMSAVVIDALEIAINPFGMPGNPTDLTVVATYGHERDMTRAFIGSASTFLGNGLARAIFFPGLQYSQEE  464
AGN29364.1   383   SEEEMEEYRDRGMSAVVIDALEIAINPFGMPGNPTDLTVVATYGHERDMARAFIGSASTFLGNGLARAIFFPGLQYSQEE  462
QYU59234.1   383   SEEEMEEYRDRGMSAVVIDALEIAINPFGMPGNPTDLTVVATYGHERDMTRAFIGSASTFLGNGLARAIFFPGLQYSQEE  462
CDN67475.1   383   SEEEMEEYRDRGMSAVVIDALEIAINPFGMPGNPTDLTVVATYGHERDMTRAFIGSASTFLGNGLARAIFFPGLQYSQEE  462
AGN29356.1   383   SEEEMEEYRDRGMSAVVIDALEIAINPFGMPGNPTDLTVVATYGHERDMARAFIGSASTFLGNGLARAIFFPGLQYSQEE  462
AGN29363.1   383   SEEEMEEYRDRGMSAVVIDALEIAINPFGMPGNPTDLTVVATYGHERDMARAFIGSASTFLGNGLARAIFFPGLQYSQEE  462
AFV73369.1   386   SEEEMEEYRDRGMSAVVIDALEIAINPFGMPGNPTDLTVVATYGHERNMTRAFIGSASTFLGNGLARAIFFPGLQYSQEE  465
AFV34732.1   383   SEEEMEEYRDRGMSAVVIDALEIAINPFGMPGNPTDLTVVATYGHERNMTRAFIGSASTFLGNGLARAIFFPGLQYSQEE  462
AFI43912.1   387   SEEEMDEYRDRGMSAVVIDALEIAINPFGMPGNPTDLTVVATYGHERDMTRAFIGSASTFLGNGLARAIFSPGLQYSQEE  466
CDN67472.1   383   SEEEMEEYRDRGMSAVVIDALEIAINPFGMPGNPTDLTVVATYGHERDMTRAFIGSASTFLGNGLARAIFFPGLQYSQEE  462
AGN29355.1   383   SEEEMEEYRDRGMSAVVIDALEIAINPFGMPGNPTDLTVVATYGHERDMTRAFIGSASTFLGNGFTRAYFLPGLQYSQEE  462
AFI43911.1   387   SEEEMDEYRDRGMSAVVIDALEIAINPFGMPGNPTDLTVVATYGHERDMTRAFIGSASTFLGNGLARAIFFPGLQYSQEE  466
ADJ10940.1   386   SDEEMNDYRDRGMSAVVIDALEIAINPLGMPGNPTDLTVVATYGHERNMERAFIGSSSTFLGNGLARAIFFPGLQYSQEE  465
DAZ85718.1   386   ADEEMDDYRDRGMSAVVIDALEIAINPFGMPGNPTDLTVVATYGHERNMERAFIGSSSTFLGNGLARAIFFPGLQYSQEE  465
ADJ10930.1   386   SDEEMDDYRDRGMSAVVIDALEIAINPFGMPGNPTDLTVVATYGHERNMERAFIGSSSTFLGNGLARAIFFPGLQYSQEE  465
ADJ10933.1   386   SDEEMDDYRDRGMSAVVIDALEIAINPFGMPGNPTDLTVVATYGHERNMERAFIGSSSTFLGNGLARAIFFPGLQYSQEE  465
ADJ10935.1   386   ADEEMDDYRDRGMSAVVIDALEIAINPFGMPGNPTDLTVVATYGHERNMERAFIGSSSTFLGNGLARAIFFPGLQYSQEE  465
ADJ10941.1   386   SDEEMDDYRDRGMSAVVIDALEIAINPFGMPGNPTDLTVVATYGHERNMERAFIGSSSTFLGNGLARAIFFPGLQYSQEE  465
DAZ85719.1   386   ADEEMDDYRDRGMSAVVIDALEIAINPFGMPGNPTDLTVVATYGHERNMERAFIGSSSTFLGNGLARAIFFPGLQYSQEE  465
ADJ10936.1   386   SDEEMDDYRDRGMSAVVIDALEIAINPFGMPGNPTDLTVVATYGHERNMERAFIGSSSTFLGNGLARAIFFPGLQYSQEE  465
ABR09917.1   386   ADEEMDDYRDRGMSAVVIDALEIAINPFGMPGNPTDLTVVATYGHERNMERAFIGSSSTFLGNGLARAIFFPGLQYSQEE  465
CDN67473.1   391   SEEEMEEYRDRGMSAVVIDALEIAINPFGMPGNPTDLTVVATYGHERDMTRAFIGSASTFLGNGLARAIFFPGLQYSQEE  470
QYU59232.1   383   SDEEMDDYRDRGMSAVVIDALEIAINPFGMPGNPTDLTVVATYGHERNMERAFIGSSSTFLGNGLSRAIFFPGLQYSQEE  462
AGN29362.1   383   SEEEMDEYRDRGMSAVVIDALEIAINPFGMPGNPTDLTVVATYGHERDMARAFIGSASTFLGNGLARAIFFPGLQYSQEE  462
ACZ58625.1   385   SEEEMDEYRDRGMSAVVIDALELAINPFGMPGNPTDLTVVATYGHERDMTRAFIGSASTFLGNGLARAIFFPGLQYSQEE  464
ACZ58626.1   385   SEEEMDEYRDRGMSAVVIDALELAINPFGMPGNPTDLTVVATYGHERDMTRAFIGSASTFLGNGLARAIFFPGLQYSQEE  464
ACZ58624.1   385   SEEEMDEYRDRGMSAVVIDALELAINPFGMPGNPTDLTVVATYGHERDMTRAFIGSASTFLGNGLARAIFFPGLQYSQEE  464
AGN29366.1   386   SEEEMEEYRDRGMSAVVIDALELAINPFGMPGNPTDLTVVATYGHERDMTRAFIGSASTFLGNGLARAIFFPGLQYSQEE  465
QBZ78368.1   385   SEEEMDEYRDRGMSAVVIDALELAINPFGMPGNPTDLTVVATYGHERDMTRAFIGSASTFLGNGLARAIFFPGLQYSQEE  464
AFI43914.1   394   SEEEMDEYRDRGMSAVVIDVLEIAINPFGMPGNPTDLTVVATYGHERDMTRAFIGSASTFLGNVLARAIFFPGLQYSQEE  473
AFI43913.1   394   SEEEMDEYRDRGMSAVVIDALEIAINPFGVPGNPTDLTVVATYGHERDMTRAFIGSASTFLGNVLARAIFFPGLQYSQEE  473
AFV34756.1   325   SEEEMEEYRDRGMSAVVIDALEIAINPFGMPGNPTDLTVVATYGHERNMSRAFIGSASTFLGNGLARAIFFPGLQYSQEE  404
QYU59231.1   385   SDEEMDDYRDRGMSAVVIDALEIAINPFGMPGNPTDLTVVATYGHERNMERAFIGSSSTFLGNGLSRAIFFPGLQYSQEE  464
QYU59233.1   392   SDEEMDDYRDRGMSAVVIDALEIAINPFGMPGNPTDLTVVATYGHERNMERAFIGSSSTFLGNGLSRAIFFPGLQYSQEE  471
APD26307.1   394   SEEEMDEYRDRGMSAVVIDALEIAINPFGMPGNPTDLTVVATYGHERDMTRAFIGSASTFLGNGLARAIFFPGLQYSQEE  473
QBZ78371.1   214   SDEEMDEYRDRGMSAVVIDALEIAINPFGMPGNPTDLTVVATYGHERDMTRAFIGSASTFLGNGLARAIFFPGLQYSQEE  293


NP_619706.1  465   PRRESIIRLYVASTNATVDTDSVLAAISVGTLRQHVGSMHYRTVASTVHQAQVQGTTLRATMMGNTVVVSPEGSLVTGTP  544
AWO68043.1   466   PRRESIIRLYVASTNATVDTDSVLAAISVGTLRQHVGSMHYRTVASTVHQAQVQGTTLRATMMGNTVVVSPEGSLVTGTP  545
ADJ10937.1   466   PRRESIIRLYVASTNATVDTDSVLAAISVGTLRQHVGSMHYRTVASAVHQAQVQGTTLRATMMGNTVVVSPEGSLVTGTP  545
ADJ10931.1   466   PRRESIIRLYVASTNATVDTDSVLAAISVGTLRQHVGSMHYRTVASTVHQAQVQGTTLRATMMGNTVVVSPEGSLVTGTP  545
ADJ10938.1   466   PRRESIIRLYVASTNATVDTDSVLAAISVGTLRQHVGSMHYRTVASVVHQAQVQGTTLRATMMGNTVVVSPEGSLVTGTP  545
QYU59229.1   466   PRRESIIRLYVASTNATVDTDSVLAAISVGTLRQHVGSMHYRTVANTVHQAQVQGTTLRATMMGNTVVVSPEGSLVTGTP  545
ACZ58630.1   466   PRRESIIRLYVASTNATVDTDSVLAAISVGTLRQHVGSMHYRTVASTVHQAQVQGTTLRATMMGNTVVVSPEGSLVTGTP  545
QGP72535.1   466   PRRESIIRLYVASTNATVDTDSVLAAISVGTLRQHVGSTHYRTVASTVHQAQVQGTTLRATMMGNTVVVSPEGSLVTGTP  545
Q91HK5.1     466   PRRESIIRLYVASTNATVDTDSVLAAISVGTLRQHVGSMHYRTVASTVHQAQVQGTTLRATMMGNTVVVSPEGSLVTGTP  545
ARO69912.1   466   PRRESIIRLYVASTNATVDTDSVLAAISIGTLRQHVGSMHYRTVASTVHQAQVQGTTLRATMMGNTVVVSPEGSLVTGTP  545
AFI71814.1   466   PRRESIIRLYVASTNATVDTDSVLAAISVGTLRQHVGSMHYRTVASTVHQAQVQGTTLRATMMGNTVVVSPEGSLVAGTP  545
ADJ10932.1   466   PRREFIIRLYVASTNATVDTDSVLAAISVGTLRQHVGSMHYRTVASAVHQAQVQGTTLRATMMGNTVVVSPEGSLVTGTP  545
ARO69925.1   466   PRRESIIRLYVASTNATVDTDSVLAAISVGTLRQHVGSMHYRTVASTVHQAQVQGTTLRATMMGNTVVVSPEGSLVTGTP  545
ARO69911.1   466   PRRESIIRLYVASTNATVDTDSVLAAISVGTLRQHVGSMHYRTVASTVHQAQVQGTTLRATMMGNTVVVSPEGSLVTGTP  545
ACZ58628.1   466   PRRESIIRLYVASTNATVDTDSVLAAISVGTLRQHVGSMHYRTVASTVHQAQVQGTTLRATMMGNTVVVSPEGSLVTGTP  545
ABI63932.1   466   PRRESIIRLYVASTNATVDTDSILAAISVGTLRQHVGSMHYRTVANTVHQAQVQGTTLRATMMGNTVVVSPEGSLVTGTP  545
ARO69945.1   466   PRRESIIRLYVASTNATVDTDSVLAAISVGTLRQHVGSMHYRTVASTVHQAQVQGTTLRATMMGNTVVVSPEGSLVTGTP  545
ARO69930.1   466   PRRESIIRLYVASTNATVDTDSVLAAISVGTLRQHVGSMHYRTVASTVHQAQVQGTTLRATMMGNTVVVSPEGSLVTGTP  545
ABI63925.1   466   PRRESIIRLYVASTNATVDTDSVLAAISVGTLRQHVGSMHYRTVANTVHQAQVQGTTLRATMMGNTVVVSPEGSLVTGTP  545
ARO69906.1   466   PRRESIIRLYVASTNATVDTDSVLAAISVGTLRQHVGSMHYRTVASTVHQAQVQGTTLRATMMGNTVVVSPEGSLVAGTP  545
ACZ58627.1   466   PRRESIIRLYVASTNATVDTDSVLAAISVGTLRQHVGSMHYRTVASTVHQAQVQGTTLRATMMGNTVVVSPEGSLVTGTP  545
ARO69920.1   466   PRRESIIRLYVASTNATVDTDSVLAAISVGTLRQHVGSMHYRTVASTVHQAQVQGTTLRATMMGNTVVVSPEGSLVTGTP  545
```

```
ACZ58629.1   466   PRRESIIRLYVASTNATVDTDSVLAAISVGTLRQHVGSMHYRTVASTVHQAQVQGTTLRATMMGNTVVVSPEGSLVTGTP  545
AGT42201.1   466   PRRESIIRLYVASTNATVDTDSVLAAISVGTLRQHVGSMHYRTVASTVHQAQVQGTTLRATMMGNTVVVSPEGSLVAGTP  545
ARO69914.1   466   PRRESIIRLYVASTNATVDTDSVLAAISVGTLRQHVGSMHYRTVASTVHQAQVQGTTLRATMMGNTVVVSPEGSLVTGTP  545
ABI63916.1   466   PRRESIIRLYVASTNATVDTDSILAAISVGTLRQHVGSMHYRTVANTVHQAQVQGTTLRATMMGNTVVVSPEGSLVTGTP  545
ARO69918.1   466   PRRESIIRLYVASTNATVDTDSVLAAISVGTLRQHVGSMHYRTVASTVHQAQVQGTTLRATMMGNTVVVSPEGSLVTGTP  545
ARO69948.1   466   PRRESIIRLYVASTNATVDTDSVLAAISVGTLRQHVGSMHYRTVASTVHQAQVQGTTLRATMMGNTVVVSPEGSLVTGTP  545
QYA72353.1   466   PRRESIIRLYVASTNATVDTDSVLAAISVGTLRQHVGSMHYRTVASTVHQAQVQGTTLRATMMGNTVVVSPEGSLVTGTP  545
ABI63917.1   466   PRRESIIRLYVASTNATVDTDSVLAAISVGTLRQHVGSMHYRTVANTVHQAQVQGTTLRATMMGNTVVVSPEGSLVTGTP  545
ARO69941.1   466   PRRESIIRLYVASTNATVDTDSVLAAISVGTLRQHVGSMHYRTVASTVHQAQVQGTTLRATMMGNTVVVSPEGSLVTGTP  545
ABI63926.1   466   PRRESIIRLYVASTNATVDTDSILAAISVGTLRQHVGSMHYRTVANTVHQAQVQGTTLRATMMGNTVVVSPEGSLVTGTP  545
CDN67471.1   466   PRRESIIRLYVASTNATVDTDSVLAAISVGTLRQHVGSMHYRTVASTVHQAQVQGTTLRATMMGNTVVVSPEGSLVTGTP  545
ABI63920.1   466   PRRESIIRLYVASTNATVDTDSVLAAISVGTLRQHVGSMHYRTVANTVHQAQVQGTTLRATMMGNTVVVSPEGSLVTGTP  545
ABI63927.1   466   PRRESIIRLYVASTNATVDTDSILAAISVGTLRQHVGSMHYRTVANTVHQAQVQGTTLRATMMGNTVVVSPEGSLVTGTP  545
ARO69908.1   466   PRRESIIRLYVASTNATVDTDSVLAAISVGTLRQHVGSMHYRTVASTVHQAQVQGTTLRATMMGNTVVVSPEGSLVTGTP  545
ARO69916.1   466   PRRESIIRLYVASTNATVDTDSVLAAISVGTLRQHVGSMHYRTVASTVHQAQVQGTTLRATMMGNTVVVSPEGSLVTGTP  545
ARO69910.1   466   PRRESIIRLYVASTNATVDTDSVLAAISVGTLRQHVGSMHYRTVASTVHQAQVQGTTLRATMMGNTVVVSPEGSLVTGTP  545
DAZ85720.1   466   PRRESIIRLYVASTNATVDTDSVLAAISVGTLRQHVGSMHYRTVASAVHQAQVQGTTLRATMMGNTVVVSPEGSLVTGTP  545
AAX18708.1   466   PRRESIIRLYVASTNATVDTDSVLAAISVGTLRQHVGSMHYRTVANTVHQAQVQGTTLRATMMGNTVVVSPEGSLVTGTP  545
AYA22298.1   466   PRRESIIRLYVASTNATVDTDSVLAAISVGTLRQHVGSMHYRTVANTVHQAQVQGTTLRATMMGNTVVVSPEGSLVTGTP  545
ABI63939.1   466   PRRESIIRLYVASTNATVDTDSILAAISVGTLRQHVGSMHYRTVANTVHQAQVQGTTLRATMMGNTVVVSPEGSLVTGTP  545
ARO69926.1   466   PRRESIIRLYVASTNATVDTDSVLAAISVGTLRQHVGSMHYRTVASTVHQAQVQGTTLRATMMGNTVVVSPEGSLVTGTP  545
QGP72537.1   466   PRRESIIRLYVASTNATVDTDSVLAAISVGTLRQHVGSMHYRTVASTVHQAQVQGTTLRATMMGNTVVVSPEGSLVTGTP  545
ARO69947.1   466   PRRESIIRLYVASTNATVDTDSVLAAISVGTLRQHVGSMHYRTVASTVHQAQVQGTTLRATMMGNTVVVSPEGSLVTGTP  545
ARO69921.1   466   PRRESIIRLYVASTNATVDTDSVLAAISVGTLRQHVGSMHYRTVASTVHQAQVQGTTLRATMMGNTVVVSPEGSLVTGTP  545
ARO69946.1   466   PRRESIIRLYVASTNATVDTDSVLAAISVGTLRQHVGSMHYRTVASTVHQAQVQGTTLRATMMGNTVVVSPEGSLVTGTP  545
QGP72536.1   466   PRRESIIRLYVASTNATVDIDSVLAAISVGTLRQHVGSMHYRTVASTVHQAQVQGTTLRATMMGNTVVVSPEGSLVTGTP  545
QGP72538.1   466   PRRESIIRLYVASTNATVDTDSVLAAISVGTLRQHVGSMHYRTVASTVHQAQVQGTTLRATMMGNTVVVSPEGSLVTGTP  545
ARO69913.1   466   PRRESIIRLYVASTNATVDTDSVLAAISVGTLRQHVGSMHYRTVASTVHQAQVQGTTLRATMMGNTVVVSPEGSLVTGTP  545
ARO69924.1   466   PRRESIIRLYVASTNATVDTDSVLAAISVGTLRQHVGSMHYRTVASTVHQAQVQGTTLRATMMGNTVVVSPEGSLVTGTP  545
ABI63924.1   466   PRRESIIRLYVASTNATVDTDSVLAAISVGTLRQHVGSMHYRTVANTVHQAQVQGTTLRATMMGNTVVVSPEGSLVTGTP  545
ARO69931.1   466   PRRESIIRLYVASTNATVDTDSVLAAISVGTLRQHVGSMHYRTVASTVHQAQVQGTTLRATMMGNTVVVSPEGSLVTGTP  545
AWO67711.1   465   PRRESIIRLYVASTNATVDTDSVLAAISVGTLRQHVGSMHYRTVASTVHQAQVQGTTLRATMMGNTVVVSPEGSLVTGTP  544
ARO69943.1   466   PRRESIIRLYVASTNATVDTDSVLAAISVGTLRQHVGSMHYRTVASTVHQAQVQGTTLRATMMGNTVVVSPEGSLVTGTP  545
ADJ10928.1   466   PRRESIIRLYVASTNATVDTDSVLAAISVGTLRQHVGSMHYRTVASTVHQAQVQGTTLRATMMGNTVVVSPEGSLVTGTP  545
ARO69939.1   466   PRRESIIRLYVASTNATVDTDSVLAAISVGTLRQHVGSMHYRTVASTVHQAQVQGTTLRATMMGNTVVVSPEGSLVTGTP  545
ABI63938.1   466   PRRESIIRLYVASTNATVDTDSILAAISVGTLRQHVGSMHYRTVANTVHQAQVQGTTLRATMMGNTVVVSPEGSLVTGTP  545
ABI63940.1   466   PRRESIIRLYVASTNATVDTDSILAAISVGTLRQHVGSMHYRTVANTVHQAQVQGTTLRATMMGNTVVVSPEGSLVTGTP  545
ABI63929.1   466   PRRESIIRLYVASTNATVDTDSILAAISVGTLRQHVGSMRYRTVANTVHQAQVQGTTLRATMMGNTVVVSPEGSLVTGTP  545
ABI63919.1   466   PRRESIIRLYVASTNATVDTDSVLAAISVGTLRQHVGSMHYRTVANTVHQAQVQGTTLRATMMGNTVVVSPEGSLVTGTP  545
ADJ10934.1   464   PRRESIIRLYVASTNATVDTDSVLAAISVGTLRQHVGSMHYRTVASAVHQAQVQGTTLRATMMGNTVVVSPEGSLVTGTP  543
ARO69937.1   466   PRRESIIRLYVASTNATVDTDSVLAAISVGTLRQHVGSMHYRTVASTVHQAQVQGTTLRATMMGNTVVVSPEGSLVTGTP  545
ARO69928.1   466   PRRESIIRLYVASTNATVDTDSVLAAISVGTLRQHVGSMHYRTVASTVHQAQVQGTTLRATMMGNTVVVSPEGSLVTGTP  545
ARO69927.1   466   PRRESIIRLYVASTNATVDTDSVLAAISVGTLRQHVGSMHYRTVASTVHQAQVQGTTLRATMMGNTVVVSPEGSLVTGTP  545
ABI63934.1   465   PRRESIIRLYVASTNATVDTDSILAAISVGTLRQHVGSMHYRTVANTVHQAQVQGTTLRATMMGNTVVVSPEGSLVTGTP  544
ARO69935.1   466   PRRESIIRLYVASTNATVDTDSVLAAISVGTLRQHVGSMHYRTVASTVHQAQVQGTTLRATMMGNTVVVSPEGSLVTGTP  545
ARO69940.1   466   PRRESIIRLYVASTNATVDTDSVLAAISVGTLRQHVGSMHYRTVASTVHQAQVQGTTLRATMMGNTVVVSPEGSLVTGTP  545
ARO69933.1   466   PRRESIIRLYVASTNATVDTDSVLAAISVGTLRQHVGSMHYRTVASTVHQAQVQGTTLRATMMGNTVVVSPEGSLVTGTP  545
ABI63922.1   466   PRRESIIRLYVASTNATVDTDSVLAAISVGTLRQHVGSMHYRTVANTVHQAQVQGTTLRATMMGNTVVVSPEGSLVTGTP  545
ABI63936.1   466   PRRESIIRLYVASTNATVDTDSILAAISVGTLRQHVGSMHYRTVANTVHQAQVQGTTLRATMMGNTVVVSPEGSLVTGTP  545
ABI63918.1   466   PRRESIIRLYVASTNATVDTGSVLAAISVGTLRQHVGSMHYRTVANTVHQAQVQGTTLRATMMGNTVVVSPEGSLVTGTP  545
ABI63931.1   465   PRRESIIRLYVASTNATVDTDSVLAAISVGTLRQHVGSMHYRTVASTVHQAQVQGTALRATMMGNTVVVSPEGSLVTGTP  544
QYU59230.1   466   PRRESIIRLYVASTNATVDTDSVLAAISVGTLRQHVGSMHYRTVASTVHQAQVQGTTLRATMMGNTVVVSPEGSLVTGTP  545
ARO69922.1   466   PRRESIIRLYVASTNATVDTDSVLAAISVGTLRQHVGSMHYRTVASTVHQAQVQGTTLRATMMGNTVVVSPEGSLVTGTP  545
ABI63941.1   466   PRRESIIRLYVASTNATVDTDSILAAISVGTLRQHVGSMHYRTVANTVHQAQVQGTTLRATMMGNTVVVSPEGSLVTGTP  545
ARO69949.1   466   PRRESIIRLYVASTNATVDTDSVLAAISVGTLRQHVGSMHYRTVASTVHQAQVQGTTLRATMMGNTVVVSPEGSLVTGTP  545
ARO69917.1   466   PRRESIIRLYVASTNATVDTDSVLAAISVGTLRQHVGSMHYRTVASTVHQAQVQGTTLRATMMGNTVVVSPEGSLVTGTP  545
ARO69915.1   466   PRRESIIRLYVASTNATVDTDSVLAAISVGTLRQHVGSMHYRTVASTVHQAQVQGTTLRATMMGNTAVVSPEGSLVTGTP  545
QYU59228.1   466   PRRESIIRLYVASTNATVDTDSVLAAISVGTLRQHVGSMHYRTVANTVHQAQVQGTTLRATMMGNTVVVSPEGSLVTGTP  545
AGN29358.1   466   PRRESIIRLYVASTNATVDTDSVLAAISVGTLRQHVGSMHYRTVASTVHQAQVQGTTLRATMMGNTVVVSPEGSLVTGTP  545
ARO69944.1   466   PRRESIIRLYVASTNATVDTDSVLAAISVGTLRQHVGSMHYRTVASTVHQAQVQGTTLRATMMGNTVVVSPEGSLVTGTP  545
AGN29367.1   463   PRRESIIRLYVASTNATVDTDSVLAAISVGTLRQHVGSMHYRTVASTVHQAQVQGTTLRATMMGNTVVVSPEGSLVTGTP  542
ABI63921.1   466   PRRESIIRLYVASTNATVDTDSVLAAISVGTLRQHVGSMHYRTVANTVHQAQVQGTTLRATMMGNTVVVSPEGSLVTGTP  545
ABI63923.1   466   PRRESIIRLYVASTNATVDTDSVLAAISVGTLRQHVGSMHYRTVANTVHQAQVQGTTLRATMMGNTVVVSPEGSLVTGTP  545
CDN67470.1   463   PRRESIIRLYVASTNATVDTDSVLAAISVGTLRQHVGSMHYRTVASTVHQAQVQGTTLRATMMGNTVVVSPEGSLVTGTP  542
CDN67474.1   466   PRRESIIRLYVASTNATVDTDSVLAAISVGTLRQHVGSMHYRTVASTVHQAQVQGTTLRATMMGNTVVVSPEGSLVAGTP  545
AAX18710.1   466   PRRESIIRLYVASTNATVDTDSVLAAISVGTLRQHVGSMHYRTVANTVHQAQVQGTTLRATMMGNTVVVSPEGSLVTGTP  545
QBZ78370.1   464   PRRESIIRLYVASTNATVDTDSVLAAISVGTLRQHVGSMHYRTVASTVHQAQVQGTTLRATMMGNTVVVSPEGSLVTGTP  543
ARO69923.1   466   PRRESIIRLYVASTNATVDTDSVLAAISVGTLRQHVGSMHYRTVASTVHQAQVQGTTLRATMMGNTVVVSPEGSLVTGTP  545
ARO69907.1   466   PRRESIIRLYVASTNATVDTDSVLAAISVGTLRQHVGSMHYRTVASTVHQAQVQGTTLRATMMGNTVVVSPEGSLVTGTP  545
ARO69942.1   466   PRRESIIRLYVASTNATVDTDSVLAAISVGTLRQHVGSMHYRTVASTVHQAQVQGTTLRATMMGNTVVVSPEGSLVTGTP  545
ARO69909.1   466   PRRESIIRLYVASTNATVDTDSVLAAISVGTLRQHVGSMHYRTVASTVHQAQVQGTTLRATMMGNTVVVSPEGSLVTGTP  545
AAX18711.1   466   PRRESIIRLYVASTNATVDTDSVLAAISVGTLRQHVGSMHYRTVASTVHQAQVQGTTLRAAMMGNTVVVSPEGSLVTGTP  545
ARO69936.1   466   PRRESIIRLYVASTNATVDTDSVLAAISVGTLRQHVGSMHYRTVASTVHQAQVQGTTLRATMMGNTVVVSPEGSLVTGTP  545
AGN29357.1   466   PRRESIIRLYVASTNATVDTDSVLAAISVGTLRQHVGSMHYRTVASTVHQAQVQGTTLRATMMGNTVVVSPEGSLVTGTP  545
ARO69919.1   466   PRRESIIRLYVASTNATVDTDSVLAAISVGTLRQHVGSMHYRTVASTVHQAQVQGTTLRATMMGNTVVVSPEGSLVTGTP  545
ADJ10929.1   466   PRRESIIRLYVASTNATVGTDSVLAAISVGTLRQHVGSMHYRTVASTVHQAQVQGTTLRATMMGNTVVVSPEGSLVTGTP  545
ABI63943.1   466   PRCESIIRLYVASTNATVDTDSIVAAISVGTLRQHVGSMHYRTVANTVHQAQVQGTTLRATMMGNTVVVSPEGSLVTGTP  545
AGN29365.1   466   PRRESIIRLYVASANATVDTDSVLAAISVGTVRQHVGSLHYRTVASAVHQAQVQGTTLRATMMGNTVVVSPEGSLVTGTP  545
ABI63930.1   465   PRRESIIRLYVASTNATVDTDSVLAAISVGTLRQHVGSMHYRTVASTVHQAQVQSTALRATMMGNTVVVSPEGSLVTGTP  544
ABI63942.1   465   PRRESIIRLYVASTNATVDTDSVLAAISVGTLRQHVGSMHYRTVASTVHQAQVQGTVLRATMMGNTVVVSPEGGLVTGTP  544
ADJ10939.1   466   PRRESIIRLYVASTNATVDTDSVLAAISVGTLRQHVGSMHYRTVASTVHQAQVQGTTLRATMMGNTVVVSPEGSLVTGTP  545
ABI63928.1   465   PRRESIIRLYVASTNATVDTDSVLAAISVGTLRQHVGSMHYRTVASTVHQAQVQGTALRATMMGNTVVVSPEGSLVTGTP  544
CDN67476.1   463   PRRESIIRLYVASTNATVDTDSVLAAISVGTLRQHVGSMHYRTVASTVHQAQVQGTTLRATMMGNTVVVSPEGSLVTGTP  542
ABI63933.1   465   PRRESIIRLYVASTNATVDTDSVLAAISVGTLRQHVGSMHYRTVASTVHQAQAQGTVLRATMMGNTVVVSPEGSLVTGTP  544
AAX18707.1   466   PRRESIIRLYVASTNATVDTDSVLAAISVGTLRQHVGSMHYRTVANTVHQAQVQGTTLRATMMGNTVVVSPEGSLVTGTP  545
ACZ58631.1   464   PRRESIIRLYVASTNATVDTDSVLAAISVGTLRQHVGSLHYRTVASTVHQAQVQGTTLRATMMGNTVVVSPEGSLVTGTP  543
AGN29359.1   466   PRRESIIRLYVASTNATVDTDSVLAAISVGTLRQHVGSMHYRTVASTVHQAQVQGTTLRATMMGNTVVVSPEGSLVTGTP  545
AGN29361.1   466   PRRESIIRLYVASTNATVDTDSVLAAISVGTLRQHVGSMHYRTVASTVHQAQVQGTTLRATMMGNTVVVSPEGSLVTGTP  545
```

```
ABI63935.1   465   PRRESIIRLYVASTNATVDTDSVLAAISVGTLRQHVGSMHYRTVASTVHQAQVQGTALRATMMGNTVVVSPEGSLVTGTP  544
AGN29360.1   467   PRRESIIRLYVASTNATVDTDSVLAAISVGTLRQHVGSMHYRTVASTVHQAQVQGTTLRATMMGNTVVVSPEGSLVTGTP  546
AWO67712.1   465   PRRESIIRLYVASTNATVDTDSVLAAISVGTLRQHVGSMHYRTVASTVHQAQVQGTTLRATMMGNTVVVSPEGSLVTGTP  544
AYA22300.1   463   PRRESIIRLYVASTNATVDTDSVLAAISVGTLRQHVGSMHYRTVASTVHQAQVQGTTLRATMMGNTVVVSPEGSLVTGTP  542
QBZ78373.1   463   PRRESIIRLYVASTNATVDADSVLAAISVGTLRQHVGSMHYRTVASAMHQAQVQGTTLRATMMGNTVVVSPEGSLVTGTP  542
AYQ93056.1   463   PRRESIIRLYVASTNATVDTDSVLAAISVGTLRQHVGSMHYRTVASTVHQAQVQGTTLRATMMGNTVVVSPEGSLVTGTP  542
AYQ93054.1   463   PRRESIIRLYVASTNATVDTDSVLAAISVGTLRQHVGSMHYRTVASTVHQAQVQGTTLRATMMGNTVVVSPEGSLVTGTP  542
AAX18709.1   463   PRRESIIRLYVASTNATVDTDSVLAAISVGTLRQHVGSMHYRTVASTVHQAQVQGTTLRATMMGNTVVVSPEGSLVTGTP  542
AYQ93052.1   464   PRRESIIRLYVASTNATVDTDSVLAAISVGTLRQHVGSMHYRTVASTVHQAQVQGTTLRATMMGNTVVVSPEGSLVTGTP  543
BCT87062.1   465   PRRESIIRLYVASTNATVDTDSVLAAISVGTLRQHVGSMHYRTVASTVHQAQVQGTTLRATMMGNTVVVSPEGSLVAGTP  544
AGN29364.1   463   PRRESIIRLYVASTNATVDNDSVLAAISVGTLRQHIGSMHYRTVASAVHQAQVQGTTLRATMMGNTVVVSPEGSLVTGTP  542
QYU59234.1   463   PRRESIIRLYVASTNATVDTDSVLAAISVGTLRQHVGSMHYRTVASTVHQAQVQGTTLRATMMGNTVVVSPEGSLVTGTP  542
CDN67475.1   463   PRRESIIRLYVASTNATVDTDSVLAAISVGTLRQHVGSMHYRTVASTVHQAQVQGTTLRATMMGNTVVVSPEGSLVTGTP  542
AGN29356.1   463   PRRESIIRLYVASTNATVDTDSVLAAISVGTLRQHVGSMHYRTVASSVHQAQVHGTTFRVIMMGNTVVVSPEGSLVTGIP  542
AGN29363.1   463   PRRESIIRLYVASTNATVDTDSVLAAISVGTLRQHVGSMHYRTVASTVHQAQVQGMTLRATMMGNTVVVSPEGSLVTGTP  542
AFV73369.1   466   PRRESIVRLYVASTNATVDADSILAAISVGTLRQHIGSMHYRTVVNTVHQAQVQGTTLRATMMGNTVVVSPEGSLVTGTP  545
AFV34732.1   463   PRRESIVRLYVASTNATVDADSILAAISVGTLRQHVGSMHYRTVANTVHQAQVQGTTLRATMMGNTVVVSPEGSLVTGIP  542
AFI43912.1   467   PRRESIIRLYVASTNATVDTDSVLAAISVGTLRQHVGSMHYRTVASTVHQAQVQGTTLRATMMGNTVVVSPEGSLVAGTP  546
CDN67472.1   463   PRRESIIRLYVASTNATVDTDSVLAAISVGTLRQHVGSMHYRTVASTVHQAQVQGTTLRATMMGNAVVVSPEGGLVTGVP  542
AGN29355.1   463   PRRESLILLYVASTHATVNTDSVLAAISVGTLRQHVGSMHYRTVASTVHQAQVQGTTLRATMMGNTVVVSPEGSLVTGTP  542
AFI43911.1   467   PRRESIIRLYVASTNATVDTDSVLAAISVGTLRQHVGSMHYRTVASTVHQAQVQGTTLRATMMGNTVVVSPEGSLVVGTP  546
ADJ10940.1   466   PRRESLIRLYVASTNATVDPDSVLAAISVGTLRQHIGSLHYRTVASSVHTAQVQGTTLRATMMGNAVVVSPEGGLVTGVP  545
DAZ85718.1   466   PRRESLIRLYVASTNATVDPDSVLAAISVGTLRQHIGSLHYRTVASSVHTAQVQGTTLRATMMGNAVVVSPEGGLVTGIP  545
ADJ10930.1   466   PRRESLIRLYVASTNATVDPDSVLAAISVGTLRQHIGSLHYRTVASSVHTAQVQGTTLRATMMGNAVVVSPEGGLVTGVP  545
ADJ10933.1   466   PRRESLIRLYVASTNATVDPDSVLAAISVGTLRQHIGSLHYRTVASSVHTAQVQGTTLRATMMGNAVVVSPEGGLVTGVP  545
ADJ10935.1   466   PRRESLIRLYVASTNATVDPDSVLAAISVGTLRQHIGSLHYRTVASSVHTAQVQGTTLRATMMGNAVVVSPEGGLVTGVP  545
ADJ10941.1   466   PRRESLIRLYVASTNATVDPDSVLAAISVGTLRQHIGSLHYRTVASSVHTAQVQGTTLRATMMGNAVVVSPEGGLVTGVP  545
DAZ85719.1   466   PRRESLIRLYVASTNATVDPDSVLAAISVGILRQHIGSLHYRTVASSVHTAQVQGTTLRATMMGNAVVVSPEGGLVTGIP  545
ADJ10936.1   466   PRRESLIRLYVASTNATVDPDSVLAAISVGTLRQHIGSLHYRTVASSVHTAQVQGTTPRATMMGNAVVVSPEGGLVTGVP  545
ABR09917.1   466   PRRESLIRLYVASTNATVDPDSVLAAISVGTLRQHIGSLHYRTVASSVHTAQVQGTTLRATMMGNAVVVSPEGGLVTGVP  545
CDN67473.1   471   PRRESIIRLYVASTNATVDTDSVLAAISVGTLRQHVGSMHYRTVASTVHQAQVQGTTLRATMMGNTVVVSPEGSLVTGTP  550
QYU59232.1   463   PRRESIIRLYVASTNATVDPDSVLAAISVGTLRQHIGSLHNRTVASSVHAAQVQGTTLRATMMGNSVVVSPEGGLVTGIP  542
AGN29362.1   463   PRRESIIRLYVASTNATVDTDSFLAAISVGTLRQHVGSMHYRTVASTVHQAQVQGTTLRATMMGNTVVVSPEGSLVTGTP  542
ACZ58625.1   465   PRRESLIRLYVASTNATVDTDSVLAAISVGTLRQHVGSMHYRTVANAVHQAQVQGTTLRATMMGNTVVVSPEGSLVTGTP  544
ACZ58626.1   465   PRRESLIRLYVASTNATVDTDSVLAAISVGTLRQHVGSMHYRTVANAVHQAQVQGTTLRATMMGNTVVVSPEGSLVTGTP  544
ACZ58624.1   465   PRRESLIRLYVASTNATVDTDSVLAAISVGTLRQHVGSMHYRTVANAVHQAQVQGTTLRATMMGNTVVVSPEGSLVTGTP  544
AGN29366.1   466   PRRESIIRLYVASTNATVDTDSVLAAISVGTLRQHVGSMHYRTVASTVHQAQVQGTTLRATMMGNTVVVSPEGSLVAGTP  545
QBZ78368.1   465   PRRESLIRLYVASTNATVDTDSVLAAISVGTLRQHVGSMHYRTVANAVHQAQVQGTTLRATMMGNTVVVSPEGSLVTGTP  544
AFI43914.1   474   PRHESIIRLYVASTNATVDTDSVLAAISVGTLRQHVGSMHYRTVASTVHQAQVQGTTLRATMMGNTVVVSPEGSLVAGTP  553
AFI43913.1   474   PRHESIIRLYVASTNATVDTDSVLAAISVGTLRQHVGSMHYRTVASTVHQAQVQGTTLRATMMGNTVVVSPEGSLVAGTP  553
AFV34756.1   405   PRRESIVRLYVASTNATVDADSILAAISVGTLRQHVGSMHYRTVASTVHQAQVQGTTLRATMMGNTVVVSPEGSLVTGIP  484
QYU59231.1   465   PRRESIIRLYVASTNATVDPDSVLAAISVGTLRQHIGSLHNRTVASSVHAAQVQGTTLRATMMGNSIVVSPEGGLVAGIP  544
QYU59233.1   472   PRRESIIRLYVASTNATVDPDSVLAAISVGTLRQHIGSLHNRTVASSVHAAQVQGTTLRATMMGNSVVVSPEGGLVTGVP  551
APD26307.1   474   PRRESIIRLYVASTNATVDPDSVLAAISVGTLRQHIGSLHNRTVARTVHQAQVQGTTLRATMMGNTVVVSPEGSLVTGTP  553
QBZ78371.1   294   PRRESIIRLYVASTNATVDTDSVLAAISVGTLRQHVGSMHYRTVASTVHQAQVQGTTLRATMMGNTVVVSPEGSLVTGTP  373
```

MP-CP cleavage site

```
NP_619706.1  545   EARVEIGGGSSIRMVGPLQWESVEEPGQTFSIRSRSRSVRIDRNVDLPQLEAEPRLSSTVRGLAGRGVIYIPKDCQANRY  624
AWO68043.1   546   EARVEIGGGSSIRMVGPLQWESVEEPGQTFSIRSRSRSVRIDRNVDLPQLEAEPRLSSTVRGLAGRGVIYIPKDCQANRY  625
ADJ10937.1   546   EARVEIGGGSSIRMVGPLQWESVEEPGQTFSIRSRSRSVRIDRNVDLPQLEAEPRLSSTVRGLAGRGVIYIPKDCQANRY  625
ADJ10931.1   546   EARVEIGGGSSIRMVGPLQWESVEEPGQTFSIRSRSRSVRIDRNVDLPQLEAEPRLSSTVRGLAGRGVIYIPKDCQANRY  625
ADJ10938.1   546   EARVEIGGGSSIRMVGPLQWESVEEPGQTFSIRSRSRSVRIDRNVDLPQLEAEPRLSSTVRGLAGRGVVYIPKDCQANRY  625
QYU59229.1   546   EARVEIGGGSSIRMVGPLQWESVEEPGQTFSIRSRSRSVRIDRNADLPQLEAEPRLSSTVRGLAGRGVVYIPKDCQANRY  625
ACZ58630.1   546   EARVEIGGGSSIRMVGPLQWESVEEPGQTFSIRSRSRSVRIDRNVDLPQLEAEPRLSSTVRGLAGRGVVYIPKDCQANRY  625
QGP72535.1   546   EARVEIGGGSSIRMVGPLQWESVEEPGQTFSIRSRSRSVRIDRNVDLPQLEAEPRLSSTVRGLAGRGVVYIPKDCQANRY  625
Q91HK5.1     546   EARVEIGGGSSIRMVGPLQWESVEEPGQTFSIRSRSRSVRIDRNVDLPQLEAEPRLSSTVRGLAGRGVVYIPKDCQANRY  625
ARO69912.1   546   EARVEIGGGSSIRMVGPLQWESVEEPGQTFSIRSRSRSVRIDRNVDLPQLEAEPRLSSTVRGLAGRGVVYIPKDCQANRY  625
AFI71814.1   546   EARVEIGGGSSIRMVGPLQWESVEEPGQTFSIRSRSRSVRIDRNVDLPQLEAEPRLSSTVRGLAGRGVIYIPKDCQANRY  625
ADJ10932.1   546   EARVEIGGGSSIRMVGPLQWESVEEPGQTFSIRSRSRSVRIDRNVDLPQLEAEPRLSSTVRGLAGRGVIYIPKDCQANRY  625
ARO69925.1   546   EARVEIGGGSSIRMVGPLQWESVEEPGQTFSIRSRSRSVRIDRNVDLPQLEAEPRLSSTVRGLAGRGVVYIPKDCQANRY  625
ARO69911.1   546   EARVEIGGGSSIRMVGPLQWESVEEPGQTFSIRSRSRSVRIDRNVDLPQLEAEPRLSSTVRGLAGRGVVYIPKDCQANRY  625
ACZ58628.1   546   EARVEIGGGSSIRMVGPLQWESVEEPGQTFSIRSRSRSVRIDRNVDLPQLEAEPRLSSTVRGLAGRGVIYIPKDCQANRY  625
ABI63932.1   546   EAKVEIGGGSSIRMVGPLQWESVEEPGQTFSIRSRSRSVRIDRNVDLPQLEAEPRLSSTVRGLAGRGVIYIPKDCQANRY  625
ARO69945.1   546   EARVEIGGGSSIRMVGPLQWESVEEPGQTFSIRSRSRSVRIDRNVDLPQLEAEPRLSSTVRGLAGRGVVYIPKDCQANRY  625
ARO69930.1   546   EARVEIGGGSSIRMVGPLQWESVEEPGQTFSIRSRSRSVRIDRNVDLPQLEAEPRLSSTVRGLAGRGVVYIPKDCQANRY  625
ABI63925.1   546   EAKVEIGGGSSIRMVGPLQWESVEEPGQTFSIRSRSRSVRIDRNVDLPQLEAEPRLSSTVRGLAGRGVIYIPKDCQANRY  625
ARO69906.1   546   EARVEIGGGSSIRMVGPLQWESVEEPGQTFSIRSRSRSVRIDRNVDLPQLEAEPRLSSTVRGLAGRGVVYIPKDCQANRY  625
ACZ58627.1   546   EARVEIGGGSSIRMVGPLQWESVEEPGQTFSIRSRSRSVRIDRNVDLPQLEAEPRLSSTVRGLAGRGVIYIPKDCQANRY  625
ARO69920.1   546   EARVEIGGGSSIRMVGPLQWESVEEPGQTFSIRSRSRSVRIDRNVDLPQLEAEPRLSSTVRGLAGRGVVYIPKDCQANRY  625
ACZ58629.1   546   EARVEIGGGSSIRMVGPLQWESVEEPGQTFSIRSRSRSVRIDRNVDLPQLEAEPRLSSTVRGLAGRGVIYIPKDCQANRY  625
AGT42201.1   546   EARVEIGGGSSIRMVGPLQWESVEEPGQTFSIRSRSRSVRIDRNVDLPQLEAEPRLSSTVRGLAGRGVIYIPKDCQANRY  625
ARO69914.1   546   EARVEIGGGSSIRMVGPLQWESVEEPGQTFSIRSRSRSVRIDRNVDLPQLEAEPRLSSTVRGLAGRGVVYIPKDCQANRY  625
ABI63916.1   546   EAKVEIGGGSSIRMVGPLQWESVEEPGQTFSIRSRSRSVRIDRNVDLPQLEAEPRLSSTVRGLAGRGVIYIPKDCQANRY  625
ARO69918.1   546   EARVEIGGGSSIRMVGPLQWESVEEPGQTFSIRSRSRSVRIDRNVDLPQLEAEPRLSSTVRGLAGRGVVYIPKDCQANRY  625
ARO69948.1   546   EARVEIGGGSSIRMVGPLQWESVEEPGQTFSIRSRSRSVRIDRNVDLPQLEAEPRLSSTVRGLAGRGVVYIPKDCQANRY  625
QYA72353.1   546   EARVEIGGGSSIRMVGPLQWESVEEPGQTFSIRSRSRSVRIDRNVDLPQLEAEPRLSSTVRGLAGRGVIYIPKDCQANRY  625
ABI63917.1   546   EAKVEIGGGSSIRMVGPLQWESVEEPGQTFSIRSRSRSVRIDRNVDLPQLEAEPRLSSTVRGLAGRGVIYIPKDCQANRY  625
ARO69941.1   546   EARVEIGGGSSIRMVGPLQWESVEEPGQTFSIRSRSRSVRIDRNVDLPQLEAEPRLSSTVRGLAGRGVVYIPKDCQANRY  625
ABI63926.1   546   EAKVEIGGGSSIRMVGPLQWESVEEPGQTFSIRSRSRSVRIDRNVDLPQLEAEPRLSSTVRGLAGRGVIYIPKDCQANRY  625
CDN67471.1   546   EARVEIGGGSSIRMVGPLQWESVEEPGQTFSIRSRSRSVRVDRNVDLPQLEAEPRLSSTVRGLAGRGVVYIPKDCQANRY  625
ABI63920.1   546   EAKVEIGGGSSIRMVGPLQWESVEEPGQTFSIRSRSRSVRIDRNVDLPQLEAEPRLSSTVRGLAGRGVIYIPKDCQANRY  625
ABI63927.1   546   EAKVEIGGGSSIRMVGPLQWESVEEPGQTFSIRSRSRSVRIDRNVDLPQLEAEPRLSSTVRGLAGRGVIYIPKDCQANRY  625
ARO69908.1   546   EARVEIGGGSSIRMVGPLQWESVEEPGQTFSIRSRSRSVRIDRNVDLPQLEAEPRLSSTVRGLAGRGVVYIPKDCQANRY  625
ARO69916.1   546   EARVEIGGGSSIRMVGPLQWESVEEPGQTFSIRSRSRSVRIDRNVDLPQLEAEPRLSSTVRGLAGRGVVYIPKDCQANRY  625
ARO69910.1   546   EARVEIGGGSSIRMVGPLQWESVEEPGQTFSIRSRSRSVRIDRNVDLPQLEAEPRLSSTVRGLAGRGVVYIPKDCQANRY  625
```

DAZ85720.1	546	EARVEIGGGSSIRMVGPLQWESVEEPGQTFSIRSRSRSVRIDRNVDLPQLEAEPRLSSTVRGLAGRGVIYIPKDCQANRY	625
AAX18708.1	546	EARVEIGGGSSIRMVGPLQWESVEEPGQTFSIRSRSRSVRIDRNVDLPQLEAEPRLSSTVRGLAGRGVVYIPKDCQANRY	625
AYA22298.1	546	EARVEIGGGSSIRMVGPLQWESVEEPGQTFSIRSRSRSVRIDRNVDLPQLEAEPRLSSTVRGLAGRGVVYIPKDCQANRY	625
ABI63939.1	546	EARVEIGGGSSIRMVGPLQWESVEEPGQTFSIRSRSRSVRIDRNVDLPQLEAEPRLSSTVRGLAGRGVIYIPKDCQANRY	625
ARO69926.1	546	EARVEIGGGSSIRMVGPLQWESVEEPGQTFSIRSRSRSVRIDRNVDLPQLEAEPRLSSTVRGLAGRGVVYIPKDCQANRY	625
QGP72537.1	546	EARVEIGGGSSIRMVGPLQWESVEEPGQTFSIRSRSRSVRIDRNVDLPQLEAEPRLSSTVRGLAGRGVIYIPKDCQANRY	625
ARO69947.1	546	EARVEIGGGSSIRMVGPLQWESVEEPGQTFSIRSRSRSVRIDRNVDLPQLEAEPRLSSTVRGLAGRGVVYIPKDCQANRY	625
ARO69921.1	546	EARVEIGGGSSIRMVGPLQWESVEEPGQTFSIRSRSRSVRIDRNVDLPQLEAEPRLSSTVRGLAGRGVVYIPKDCQANRY	625
ARO69946.1	546	EARVEIGGGSSIRMVGPLQWESVEEPGQTFSIRSRSRSVRIDRNVDLPQLEAEPRLSSTVRGLAGRGVVYIPKDCQANRY	625
QGP72536.1	546	EARVEMGGGSSIRMVGPLQWESVEEPGQTFSIRSRSRSVRIDRNVDLLQPEAEPRLSSTVRGLAGRGVVYIPKDCQANRY	625
QGP72538.1	546	EARVEIGGGSSIRMVGPLQWESVEEPGQTFSIRSRSRSVRIDRNVDLPQLEAEPRLSSTVRGLAGRGVIYIPKDCQANRY	625
ARO69913.1	546	EARVEIGGGSSIRMVGPLQWESVEEPGQTFSIRSRSRSVRIDRNVDLPQLEAEPRLSSTVRGLAGRGVVYIPKDCQANRY	625
ARO69924.1	546	EARVEIGGGSSIRMVGPLQWESVEEPGQTFSIRSRSRSVRIDRNVDLPQLEAEPRLSSTVRGLAGRGVVYIPKDCQANRY	625
ABI63924.1	546	EAKVEIGGGSSIRMVGPLQWESVEEPGQTFSIRSRSRSVRIDRNVDLPQLEAEPRLSSTVRGLAGRGVIYIPKDCQANRY	625
ARO69931.1	546	EARVEIGGGSSIRMVGPLQWESVEEPGQTFSIRSRSRSVRIDRNVDLPQLEAEPRLSSTVRGLAGRGVVYIPKDCQANRY	625
AWO67711.1	545	EARVELGGGSSIRMVGPLQWESVEEPGQTFSIRSRSRFVRIDRNVDLPQLEAEPRLSSTVRGLAGRGVVYIPKDCQANRY	624
ARO69943.1	546	EARVEIGGGSSIRMVGPLQWESVEEPGQTFSIRSRSRSVRIDRNVDLPQLEAEPRLSSTVRGLAGRGVVYIPKDCQANRY	625
ADJ10928.1	546	EARVEIGGGSSIRMVGPLQWESVEEPGQTFSIRSRSRSVRIDRNVDLPQLEAEPRLSSTVRGLAGRGVIYIPKDCQANRY	625
ARO69939.1	546	EARVEIGGGSSIRMVGPLQWESVEEPGQTFSIRSRSRSVRIDRNVDLPQLEAEPRLSSTVRGLAGRGVVYIPKDCQANRY	625
ABI63938.1	546	EARVEIGGGSSIRMVGPLQWESVEEPGQTFSIRSRSRSVRIDRNVDLPQLEAEPRLSSTVRGLAGRGVIYIPKDCQANRY	625
ABI63940.1	546	EARVEIGGGSSIRMVGPLQWESVEEPGQTFSIRSRSRSVRIDRNVDLPQLEAEPRLSSTVRGLAGRGVIYIPKDCQANRY	625
ABI63929.1	546	EAKVEIGGDSSIRMVGPLQWESVEEPGQTFSIRSRSRSVRIDRNVDLPQLEAEPRLSSTVRGLAGRGVIYIPKDCQANRY	625
ABI63919.1	546	EAKVEIGGGSSIRMVGPLQWESVEEPGQTFSIRSRSRSVRIDRNVDLPQLEAEPRLSSTVRGLAGRGVIYIPKDCQANRY	625
ADJ10934.1	544	EARVEIGGGSSIRMVGPLQWESVEEPGQTFSIRSRSRSVRIDRNVDLPQLEAEPRLSSTVRGLAGRGVIYIPKDCQANRY	623
ARO69937.1	546	EARVEIGGGSSIRMVGPLQWESVEEPGQTFSIRSRSRSVRIDRNVDLPQLEAEPRLSSTVRGLAGRGVVYIPKDCQANRY	625
ARO69928.1	546	EARVEIGGGSSIRMVGPLQWESVEEPGQTFSIRSRSRSVRIDRNVDLPQLEAEPRLSSTVRGLAGRGVVYIPKDCQANRY	625
ARO69927.1	546	EARVEIGGGSSIRMVGPLQWESVEEPGQTFSIRSRSRSVRIDRNVDLPQLEAEPRLSSTVRGLAGRGVVYIPKDCQANRY	625
ABI63934.1	545	EAKVEIGGGSSIRMVGPLQWESVEEPGRTFSIRSRSRSVRIDRNVDLPQLEAEPRLSSTVRGLAGRGVIYIPKDCQANRY	624
ARO69935.1	546	EARVEIGGGSSIRMVGPLQWESVEEPGQTFSIRSRSRAVRIDRNVDLPQLEAEPRLSSTVRGLAGRGVVYIPKDCQANRY	625
ARO69940.1	546	EARVEIGGGSSIRMVGPLQWESVEEPGQTFSIRSRSRSVRIDRNVDLPQLEAEPRLSSTVRGLAGRGVVYIPKDCQANRY	625
ARO69933.1	546	EARVEIGGGSSIRMVGPLQWESVEEPGQTFSIRSRSRSVRIDRNVDLPQLEAEPRLSSTVRGLAGRGVVYIPKDCQANRY	625
ABI63922.1	546	EAKVEIGGGSSIRMVGPLQWESVEEPGQTFSIRSRSRSVRIDRNVDLPQLEAEPKLSSTVRGLAGRGVIYIPKDCQANRY	625
ABI63936.1	546	EAKVEIGGGSSIRMVGPLQWESVEEPGQTFSIRSRSRSVRIDRNVDLPQLEAEPRLSSTVRGLAGRGVIYIPKDCQANRY	625
ABI63918.1	546	EAKVEIGGGSSIRMVGPLQWESVEEPGQTFSIRSRSRSVRIDRNVDLPQLEAEPRLSSTVRGLAGRGVIYIPKDCQANRY	625
ABI63931.1	545	EARVEIGGGSSIRMVGPLQWESVEEPGQIFSIKSRSRSVRVDRNVDLPQLEAEPRLSSTVRGLAGRGVVYIPKDCQANRY	624
QYU59230.1	546	EARVEMGGGSSIRMVGPLQWESVEEPGQTFSIRSRSRSVRIDRNVDLPQLEAEPRLSSTVRGLAGRGVVYIPKDCQANRY	625
ARO69922.1	546	EARVEIGGGSSIRMVGPLQWESVEEPGQTFSIRSRSRSVRIDRNVDLPQLEAEPRLSSTVRGLAGRGVVYIPKDCQANRY	625
ABI63941.1	546	EARVEIGGGSSIRMVGPLQWESVEEPGQTFSIRSRSRSVRIDRNVDLPQLEAEPRLSSTVRGLAGRGVIYIPKDCQANRH	625
ARO69949.1	546	EARVEIGGGSSIRMVGPLQWESVEEPGQTFSIRSRSRSVRIDRNVDLPQLEAEPRLSSTVRGLAGRGVVYIPKDCQANRY	625
ARO69917.1	546	EARVEIGGGSSIRMVGPLQWESVEEPGQTFSIRSRSRSVRIDRNVDLPQLEAEPRLSSTVRGLAGRGVVYIPKDCQANRY	625
ARO69915.1	546	EARVEIGGGSSIRMVGPLQWESVEEPGQTFSIRSRSRSVRIDRNVDLPQLEAEPRLSSTVRGLAGRGVVYIPKDCQANRY	625
QYU59228.1	546	EARVEIGGGSSIRMVGPLQWESVEEPGQTFSIRSRSRSVRIDRNADLPQLEAEPRLSSTVRGLAGRGVVYIPKDCQANRY	625
AGN29358.1	546	EARVEIGGGSSIRMVGPLQWESVEEPGQTFSIRSRSRSVRIDRNVDLPQLEAEPRLSSTVRGLAGRGVIYIPKDCQANRY	625
ARO69944.1	546	EARVEIGGGSSIRMVGPLQWESVEEPGQTFSIRSRSRSVRIDRNVDLPQLEAEPRLSSTVRGLAGRGVVYIPKDCQANRY	625
AGN29367.1	543	EARVEIGGGSSIRMVGPLQWESVEEPGQTFSIRSRSRFVRIDRNVDLPQLEAEPRLSSTVRGLAGRGVIYIPKDCQANRY	622
ABI63921.1	546	EAKVEIGGGSSIRMVGPLQWESVEEPGQTFSIRSRSRSVRIDRNVDLPQLGAEPRLSSTVRGLAGRGVIYIPKDCQANRY	625
ABI63923.1	546	EAKAEIGGGSSIRMVGPLQWESVEEPGQTFSIRSRSRSVRIDRNVDLPQLEAEPRLSSTVRGLAGRGVIYIPKDCQANRY	625
CDN67470.1	543	EARVEIGGGSSIRMVGPLQWESVEEPGQTFSIRSRSRSVRIDRNVDLPQLEAEPRLSSTVRGLAGRGVVYIPKDCQANRY	622
CDN67474.1	546	EARVEIGGGSSIRMVGPLQWESVEEPGQTFSIRSRSRSVRIDRNVDLPQLEAEPRLSSTVRGLAGRGVVYIPKDCQANRY	625
AAX18710.1	546	EARVEIGGGSSIRMVGPLQWESVEEPGQTFSIRSRSRSVRIDRNVDLPQLEAEPRLSSTVRGLAGRGVVYIPKDCQANRY	625
QBZ78370.1	544	EARVEIGGGSSIRMVGPLQWESVEEPGQTFSIKSRSRSVRIDRNVDLPQLEAEPRLSSTVRGLAGRGVIYIPKDCQANRY	623
ARO69923.1	546	EARVEIGGGSSIRMVGPLQWESVEEPGQTFSIRSRSRSVRIDRNVDLPQLEAEPRLSSTVRGLAGRGVVYIPKDCQANRY	625
ARO69907.1	546	EARVEIGGGSSIRMVGPLQWESVEEPGQTFSIRSRSRSVRIDRNVDLPQLEAEPRLSSTVRGLAGRGVVYIPKDCQANRY	625
ARO69942.1	546	EARVEIGGGSSIRMVGPLQWESVEEPGQTFSIRSRSRSVRIDRNVDLPQLEAEPRLSSTVRGLAGRGVVYIPKDCQANRY	625
ARO69909.1	546	EARVEIGGGSSIRMVGPLQWESVEEPGQTFSIRSRSRSVRIDRNVDLPQLEAEPRLSSTVRGLAGRGVVYIPKDCQANRY	625
AAX18711.1	546	EARVEIGGGSSIRMVGPLQWESVEEPGQTFSIRSRSRSMRVDRNVDLPQLEAEPRLSSTVRGLAGRGVIYIPKDCQANRY	625
ARO69936.1	546	EARVEIGGGSSIRMVGPLQWESVEEPGQTFSIRSRSRSVRIDRNVDLPQLEAEPRLSSTVRGLAGRGVVYIPKDCQANRY	625
AGN29357.1	546	EARVEIGGGSSIRMVGPLQWESVEEPGQTFSIRSRSRSVRIDRNVDLPQLEAEPRLSSTVRGLAGRGVVYIPKDCQANRY	625
ARO69919.1	546	EARVEIGGGSSIRMVGPLQWESVEEPGQTFSIRSRSRSVRIDRNVDLPQLEAEPRLSSTVRGLAGRGVVYIPKDCQANRY	625
ADJ10929.1	546	EARVEIGGGSSIRMVGPLQWESVEEPGHTFSIRSRSRSVRIDRNVDLPQLEAEPRLSSTVRGLAGRGVIYIPKDCQANRY	625
ABI63943.1	546	EARVEIGGGSSIRMVGPLQWESVEEPGQTFSIRSRSRSVRIDRNVDLPQLEAEPRLSSTVRGLAGRGVIYIPKDCQANRY	625
AGN29365.1	546	EARVEIGGGSSIRMVGPLQWESVEEPGQTFSIRSRSRSVRIDRNVDLPQLEAEPRLSSTVRGLAGRGVVYIPKDCQANRY	625
ABI63930.1	545	EARVEIGGGSSIRMVGPLQWESVEEPGQIFSIRSRSRSVRVDRNVDLPQLEAEPRLSSTVRGLAGRGVVYIPKDCQANRY	624
ABI63942.1	545	EARVEIGGGSSIRMVGPLQWESVEEPGQIFSIRSRSRSVRVDRNVDLPQLEAEPRLSSTVRGLAGRGVVYIPKDCQANRY	624
ADJ10939.1	546	EARVEIGGGSSIRMVGPLQWESVEEPGQTFSIRSRSRSVRIDRNVDLPQLEAEPRLSSTVRGLAGRGVIYIPKDCQANRY	625
ABI63928.1	545	EARVEIGGGSSIRMVGPLQWESVEEPGQIFSIRSRSRSVRVDRNVDLPQLEAEPRLSSTVRGLAGRGVVYIPKDCQANRY	624
CDN67476.1	543	EARVEIGGGSSIRMVGPLQWESVEEPGQTFSIRSRSRSVRIDRNVDLPQLEAEPRLSSTVRGLAGRGVIYIPKDCQANRY	622
ABI63933.1	545	EARVEIGGGSSIRMVGPLQWESVEEPGQIFSIRSRSRSVRIDRNVDLPQLEAEPRLSSTVRGLAGRGVVYIPKDCQANRY	624
AAX18707.1	546	EARVEIGGGSSIRMVGPLQWESVEEPGQTFSIRSRSRPVRIDRNVDLPQLEAEPRLSSTVRGLAGRGVVYIPKDCQANRY	625
ACZ58631.1	544	EARVDIGGGSSIRMVGPLQWESVEEPGQTFSIRSRSRSVRIDRNVDLPQLEAEPRLSSTVRGLAGRGVVYIPKDCQANRY	623
AGN29359.1	546	EARVEIGGGSSIRMVGPLQWESVEEPGQTFSIRSRSRSVRIDRNVDLPQLEAEPRLSSTVRGLAGRGVIYIPKDCQANRY	625
AGN29361.1	546	EARVEIGGGSSIRMVGPLQWESVEEPGQTFSIRSRSRSVRIDRNVDLPQLEAEPRLSSTVRGLAGRGVIYIPKDCQANRY	625
ABI63935.1	545	EARVEIGGGSSIRMVGPLQWESVEEPGQIFSIRSRSRSVRVDRNVDLPQLEAEPRLSSTVRGLAGRGVVYIPKDCQANRY	624
AGN29360.1	547	EARVEIGGGSSIRMVGPLQWESVEEPGQTFSIRSRSRSVRIDRNVDLPQLEAEPRLSSTVRGLAGRGTIYIPKDCQANRY	626
AWO67712.1	545	EARVEIGGGSSIRMIGPLQWESVEEPGQTFSIRSRSRSVRIDRNVDLPQLEAEPRLSSTVRGLAGRGVVYIPKDCQANKY	624
AYA22300.1	543	EARVEIGGGSSIRMVGPLQWESVEEPGQTFSIRSRSRSVRIDRNVDLPQLEAEPRLSSTVRGLAGRGVVYIPKDCQANRY	622
QBZ78373.1	543	EARVEIGGGSSIRMVGPLQWESVEEPGQTFSIRSRSRSVRIDRNVDLPQLEAEPRMSSTVRGLAGRGVIYIPKDCQANKY	622
AYQ93056.1	543	EARVEIGGGSSIRMVGPLQWESVEEPGQTFSIRSRSRSVRIDRNVDLPQLEAEPRLSSTVRGLAGRGVVYIPKDCQANRY	622
AYQ93054.1	543	EARVEIGGGSSIRMVGPLQWESVEEPGQTFSIRSRSRSVRIDRNVDLPQLEAEPRLSSTVRGLAGRGVVYIPKDCQANRY	622
AAX18709.1	543	EARVEIGGGSSIRMVGPLQWESVEEPGQTFSIRSRSRSVRIDRNVDLPQLEAEPRLSSTVRGLAGRGVVYIPKDCQANRY	622
AYQ93052.1	544	EARVEIGGGSSIRMVGPLQWESVEEPGQTFSIRSRSRSVRIDRNVDLPQPEAEPRLSSTVRGLAGRGVVYIPKDCQANRY	623
BCT87062.1	545	EARVEIGGGSSIRMVGPLQWESVEEPGQTFSIRSRSRSVRIDRNVDLPQLEAEPRLSSTVRGLAGRGVIYIPKDCQANRY	624
AGN29364.1	543	EARVEIGGGSSIRMVGPLQWESVEEPGQTFSIRSRSRSVRIDRNVDLPQLEAEPRLSSTVRGLAGRGVVYIPKDCQANRY	622
QYU59234.1	543	EARVEIGGGSSIRMVGPLQWESVEEPGQTFSIRSRSRSVRIDRNVDLPQLEVEPRLSSTVRGLAGRGVIYIPKDCQANRY	622
CDN67475.1	543	EARVEIGGGSSIRMVGPLQWESVEEPGQTFSIRSRSRSVRIDRNVDLPQLEAEPRLSSTVRGLAGRGVIYIPKDCQANRY	622
AGN29356.1	543	EARVDIGGGSSIRMVGPLQWESVEEPGQTFSIRSRSRSVRIDRNVDLPQLEAEPRLSSTVRGLAGRGVVYIPKDCQANRY	622
AGN29363.1	543	EARVEIGGGSSIRMVGPLQWESVEEPGQTFSIRSRSRSVRIDRNVDLPQLEAEPRLSSTVRGLAGRGVVYIPKDCQANRY	622
AFV73369.1	546	EARVEIGGGSSIRMVGPLQWESVEEPGQTFSIRSRSRSVRIDRNVDLPQLEAEPRLSSTVRGLAGRGVVYIPKDCQANRY	625

AFV34732.1	543	EARVEIGGGSSIRMVGPLQWESVEEPGQTFSIRSRSRSVRIDRNVDLPQLEAEPRLSSTVRGLAGRGVVYIPKDCQANRY	622
AFI43912.1	547	EARVEIGGGSSVRMVGPLQWESVEEPGHTFSIRSRSRSVRIDRNVDLPQLEAEPRLSSTVRGLAGRGVIYIPKDCQANRY	626
CDN67472.1	543	EANVHIGGSSSMRMVGPQAWENVEEPGQTFSIRSRSRSVRIDRNVDLPQLEAEPRMSSTVRGLAGRGVVYIPKDCQEKRY	622
AGN29355.1	543	EARVEIGGGSSIRMVGPLQWESVEEPGQTFSIRSRSRFVRIDRNVDLPQLEAEPRLSSTVRGLAGRGVIYIPKDCQANRY	622
AFI43911.1	547	EARVEIGGGSSVRMVVPLQWESVEEPGHTFSIRSRSRSVRIDRNVDLPQLEAEPRLSSTVRGLAGRGVIYIPKDCQANRY	626
ADJ10940.1	546	EANVHIGGSSSMRMVGPLAWENVEEPGQTFSIRSRSRSVRIDRNVDLPQLEAEPRLSSTVRGLAGRGVIYIPKDCQANRY	625
DAZ85718.1	546	EANVHIGGNSSMRMVGPLAWENIEEPGQTFSIRSRSRSVRIDRNVDLPQLEAEPRLSSTVRGLAGRGVIYIPKDCQANRY	625
ADJ10930.1	546	EANVHIGGSSSMRMVGPLAWENVEEPGQTFSIRSRSRSVRIDRNVDLPQLEAEPRLSSTVRGLAGRGVIYIPKDCQANRY	625
ADJ10933.1	546	EANVHIGGSSSMRMVGPLAWENVEEPGQTFSIRSRSRSVRIDRNVDLPQLEAEPRLSSTVRGLAGRGVIYIPKDCQANRY	625
ADJ10935.1	546	EANVHIGGSSSMRMVGPLAWENVEEPGQTFSIRSRSRSVRIDRNVDLPQLEAEPRLSSTVRGLAGRGVIYIPKDCQANRY	625
ADJ10941.1	546	EANVHIGGSSSMRMVGPLAWENVEEPGQTFSIRSRSRSVRIDRNVDLPQLEAEPRLSSTVRGLAGRGVIYIPKDCQANRY	625
DAZ85719.1	546	EANVHIGGNSSMRMVGPLAWENIEEPGQTFSIRSRSRSVRIDRNVDLPQLEAEPRLSSTVRGLAGRGVIYIPKDCQANRY	625
ADJ10936.1	546	EANVHIGGSSSMRMVGPLAWENVEEPGQTFSIRSRSRSVRIDRNVDLPQLEAEPRLSSTVRGLAGRGVIYIPKDCQANRY	625
ABR09917.1	546	EANVHIGGNSSMRMVGPLAWENIEEPGQTFSIRSRSRSVRVDRNVDLPQLEAEPRLSSTVRGLAGRGVVYIPKDCQANRY	625
CDN67473.1	551	KARVEIGGGSSIRMVGPLQWESVEEPGHTFSIRSRSRSVRVDRDVDLPQLESEPRLSSTVRGLAGRGVVYIPKDCQANRY	630
QYU59232.1	543	EANVTIGGGSSMRMVGPLAWENVEEPGRTFSIRSRSRSVRIDRNVDLPQLEAEPRLSSTVRGLAGRGVIYIPKDCQANRY	622
AGN29362.1	543	EARVEIGGGSSIRMVGPLQWESVEEPGQTFSIRSRSRSVRIDRNVDLPQLEAEPRLSSTVRGLAGRGVIYIPKDCQANRY	622
ACZ58625.1	545	DARVEIGGGSSMRMVGPLQWESVEEPGQIFSIRSRSRSVRIDRNVDLPQLEAEPRLSSTVRGLAGRGVVYIPKDCQANRY	624
ACZ58626.1	545	DARVEMGGGSSMRMVGPLQWESVEEPGQIFSIRSRSRSVRIDRNVDLPQLEAEPRLSSTVRGLAGRGVVHIPKDCQANRY	624
ACZ58624.1	545	DARVEIRGGSSMRMVGPLQWESVEEPGQIFSIRSRSRSVRIDRNVDLPQLEAEPRLSSTVRGLAGRGVVYIPKDCQANRY	624
AGN29366.1	546	EARVEIGGGSSIRMVGPLQWESVEEPGQTFSIRSRSRSVRIDRNVDLPQLEAEPRLSSTVRGLAGRGVIYIPKDCQANRY	625
QBZ78368.1	545	DARVEIGGGSSMRMVGPLQWESVEEPGQIFSIRSRSRSVRIDRNVDLPQLEAEPRLSSTVRGLAGRGVVYIPKDCQANRY	624
AFI43914.1	554	EACVEIGGGSSIRMVGPLQWESVEEPGQTFSIRSRSRSVRIDRNVDLPQLEAEPRLSSTVRGLAGRGVIYIPKDCQANRY	633
AFI43913.1	554	EACVEIGGGSSIRMVGPLQWESVEEPGQTFSIRSRSRSVRIDRNVDLPQLEAEPRLSSTVRGLAGRGVIYIPKDCQANRY	633
AFV34756.1	485	EARVEIGGGSSIRMVGPLQWESVEEPGQTFSIRSRSRSVRIDRNVDLPQLEAEPRLSSTVRGLAGRGVVYIPKDCQANRY	564
QYU59231.1	545	EANVTIGGGSSMRMVGPLAWENVEEPGQTFSIRSRSRSVRIDRNVDLPQLEAEPRLSSTVRGLAGRGVVYIPKDCQANRY	624
QYU59233.1	552	EANVTIGGGSSMRMVGPLAWENVEEPGQTFSIRSRSRSVRVDRNVDLPQLEAEPRLSSTVRGLAGRGVVYIPKDCQANRY	631
APD26307.1	554	EAHVEIGGGSSIRMVGPLQWESVEEPGQTFSIRSRSRSVRVDRNVDLSQLDTQPRLSSTTRGLAGRGVISIPKDCQANRY	633
QBZ78371.1	374	EAQVEIGGGSSIRMVGPLQWERVEEPGQTFSIRSRSRSVRIDRNVDLPQLEAEPRLSSTVRGLAGRGVVYIPKDCQANRY	453

NP_619706.1	625	LGTLNIRDMISDFKGVQYEKWITAGLVMPTFKIVIRLPANAFTGLTWVMSFDAYNRITSRITASADPVYTLSVPHWLIHH	704
AWO68043.1	626	LGTLNIRDMISDFKGVQYEKWITAGLVMPTFKIVIRLPANAFTGLTWVMSFDAYNRITSRITASADPVYTLSVPHWLIHH	705
ADJ10937.1	626	LGTLNIRDMISDFKGVQYEKWITAGLVMPTFKIVIRLPANAFTGLTWVMSFDAYNRITSRIIASADPVYTLSVPHWLIHH	705
ADJ10931.1	626	LGTLNIRDMISDFKGVQYEKWITAGLVMPTFKIVIRLPANAFTGLTWVMSFDAYNRITSRITASADPVYTLSVPHWLIHH	705
ADJ10938.1	626	LGTLNIRDMIIDFKGVQYEKWIAAGLVMPTFKIIIRLPANAFTGLTWVMSFDAYNRITSRITTSADPVYTLSVPHWLIHH	705
QYU59229.1	626	LGTLNIRDMISDFKGVQYEKWITAGLVMPTFKIVVRLPANAFTGLTWVMSFDAYNRITSRITTSADPVYTLSVPHWLIHH	705
ACZ58630.1	626	LGTLNIRDMISDFKGVQYEKWITAGLVMPTFKIVVRLPANAFTGLTWVMSFDAYNRITSRITASADPVYTLSVPHWLIHH	705
QGP72535.1	626	LGTLNIRDMISDFKGVQYEKWITAGLVMPTFKIVIRLPANAFTGLTWVMSFDAYNRITSRITTSADPVYTLSVPHWLIHH	705
Q91HK5.1	626	LGTLNIRDMISDFKGVQYEKWITAGLVMPTFKIVIRLPANAFTGLTWVMSFDAYNRITSRITTSADPVYTLSVPHWLIHH	705
ARO69912.1	626	LGTLNIRDMISDFKGVQYEKWITAGLVMPIFKIVIRLPANAFTGLTWVMSFDAYNRITSKITASADPVYTLSVPHWLIHH	705
AFI71814.1	626	LGTLNIRDMISDFKGVQYEKWITAGLVMPTFKIVIRLPANAFTGLTWVMSFDAYNRITSRITASADPVYTLSVPHWLIHH	705
ADJ10932.1	626	LGTLNIRDTISDFKGVQYERWITAGLVMPTFKIVVRLPANAFTGLTWVMSFDAYNRITSRITISADPVYTLSVPHWLIHH	705
ARO69925.1	626	LGTLNIRDMISDFKGVQYEKWITAGLVMPIFKIVIRLPANAFTGLTWVMSFDAYNRITSRITASADPVYTLSVPHWLIHH	705
ARO69911.1	626	LGTLNIRDMISDFKGVQYEKWITAGLVMPIFKIVIRLPANAFTGLTWVMSFDAYNRITSRITTSADPVYTLSVPHWLIHH	705
ACZ58628.1	626	LGTLNIRDMISDFKGVQYEKWITAGLVMPIFKIIIRLPANAFTGLTWVMSFDAYNRITSRITASADPVYTLSVPHWLIHH	705
ABI63932.1	626	LGTLNIRDMISDFKGVQYEKWITAGLVMPTFKIVIRLPANAFTGLTWVMSFDAYNRITSRITTSADPVYTLSVPHWLIHH	705
ARO69945.1	626	LGTLNIRDMISDFKGVQYEKWITAGLVMPIFKIIIRLPANAFTGLTWVMSFDAYNRITSRITTSADPVYTLSVPHWLIHH	705
ARO69930.1	626	LGTLNIRDMISDFKGVQYEKWITAGLVMPIFKIVIRLPANAFTGLTWVMSFDAYNRITSRITTSADPVYTLSVPHWLIHH	705
ABI63925.1	626	LGTLNIRDMISDFKGVQYEKWITAGLVMPTFKIVIRLPANAFTGLTWVMSFDAYNRITSRITTSADPVYTLSVPHWLIHH	705
ARO69906.1	626	LGTLNIRDMISDFKGVQYEKWITAGLVMPIFKIVIRLPANAFTGLTWVMSFDAYNRITSRITASADPVYTLSVPHWLIHH	705
ACZ58627.1	626	LGTLNIRDMISDFKGVQYEKWITAGLVMPIFKIIIRLPANAFTGLTWVMSFDAYSRITSKITASADPVYTLSVPHWLIHH	705
ARO69920.1	626	LGTLNIRDMISDFKGVQYEKWITAGLVMPIFKIVIRLPANAFTGLTWVMSFDAYNRITSRITTSADPVYTLSVPHWLIHH	705
ACZ58629.1	626	LGTLNIRDMISDFKGVQYEKWITAGLVMPIFKIIIRLPANAFTGLTWVMSFDAYNRITSRITASADPVYTLSVPHWLIHH	705
AGT42201.1	626	LGTLNIRDMISDFKGVQYEKWITAGLVMPTFKIVIRLPANAFTGLTWVMSFDAYNRITSRITASADPVYTLSVPHWLIHH	705
ARO69914.1	626	LGTLNIRDMISDFKGVQYEKWITAGLVMPIFKIVIRLPANAFTGLTWVMSFDAYNRITSRITTSADPVYTLSVPHWLIHH	705
ABI63916.1	626	LGTLNIRDMISDFKGVQYEKWITAGLVMPTFKIVIRLPANAFTGLTWVMSFDAYNRITSRITTSADPAYTLSVPHWLIHH	705
ARO69918.1	626	LGTLNIRDMISDFKGVQYEKWITAGLVMPIFKIIIRLPANAFTGLTWVMSFDAYNRITSRITTSADPVYTLSVPHWLIHH	705
ARO69948.1	626	LGTLNIRDMISDFKGVQYEKWITAGLVMPIFKIVIRLPANAFTGLTWVMSFDAYNRITSKITASADPVYTLSVPHWLIHH	705
QYA72353.1	626	LGTLNIRDMISDFKGVQYEKWITAGLVMPTFKIVIRLPANAFTGLTWVMSFDAYNRITSRITTSADPVYTLSVPHWLIHH	705
ABI63917.1	626	LGTLNIRDMISDFKGVQYEKWITAGLVMPTFKIVIRLPANAFTGLTWVMSFDAYNRITSRITTSADPVYTLSVPHWLIHH	705
ARO69941.1	626	LGTLNIRDMISDFKGVQYEKWITAGLVMPTFKIVVRLPANAFTGLTWVMSFDAYNRITSRITASADPVYTLSVPHWLIHH	705
ABI63926.1	626	LGTLNIRDMISDFKGVQYEEWITAGLVMPTFKIVIRLPANAFTGLTWVMSFDAYNRITSRITTSADPVYTLSVPHWLIHH	705
CDN67471.1	626	LGTLNIRDMISDFKGVQYEKWITAGLVMPTFKIVIRLPANAFTGLTWVMSFDAYNRITSRITTSADPVYTLSVPHWLIHH	705
ABI63920.1	626	LGTLNIRDMISDFKGVQYEKWITAGLVMPTFKIVIRLPANAFTGLTWVMSFDAYNRITSRITTSADPVYTLSVPHWLIHH	705
ABI63927.1	626	LGTLNIRDMISDFKGVQYEKWITAGLVMPTFKIVIRLPANAFTGLTWVMSFDAYNRITSRITTSADPVYTLSVPHWLIHH	705
ARO69908.1	626	LGTLNIRDMISDFKGVQYEKWITAGLVMPTFKIVVRLPANAFTGLTWVMSFDAYNRITSRITASADPVYTLSVPHWLIHH	705
ARO69916.1	626	LGTLNIRDMISDFKGVQYEKWITAGLVMPTFKIVVRLPANAFTGLTWVMSFDAYNRITSRITASADPVYTLSVPHWLIHH	705
ARO69910.1	626	LGTLNIRDMISDFKGVQYEKWITAGLVMPTFKIVVRLPANAFTGLTWVMSFDAYNRITSRITASADPVYTLSVPHWLIHH	705
DAZ85720.1	626	LGTLNIRDMISDFKGVQYEKWITAGLVMPIFKIIVRLPANAFTGLTWVMSFDAYNRITSRITASADPVYTLSVPHWLIHH	705
AAX18708.1	626	LGTLNIRDMISDFKGVQYEKWITAGLVMPTFKIVVRLPANAFTGLTWVMSFDAYNRITSRITASADPIYTLSVPHWLIHH	705
AYA22298.1	626	LGTLNIRDMISDFKGVQYEKWITAGLVMPTFKIVVRLPANAFTGLTWVMSFDAYNRITSRITASADPIYTLSVPHWLIHH	705
ABI63939.1	626	LGTLNIRDMISDFKGVQYEKWITAGLVMPTFKIVIRLPANAFTGLTWVMSFDAYNRITSRITTSADPVYTLSVPHWLIHH	705
ARO69926.1	626	LGTLNIRDMISDFKGVQYEKWITAGLVMPTFKIVVRLPANAFTGLTWVMSFDAYNRITSRITASADPVYTLSVPHWLIHH	705
QGP72537.1	626	LGTLNIRDMISDFKGVQYEKWITAGLVMPTFKIVIRLPANAFTGLTWVMSFDAYNRITSRITASADPVYTLSVPHWLIHH	705
ARO69947.1	626	LGTLNIRDMISDFKGVQYEKWITAGLVMPTFKIVVRLPANAFTGLTWVMSFDAYNRITSRITASADPVYTLSVPHWLIHH	705
ARO69921.1	626	LGTLNIRDMISDFKGVQYEKWITAGLVMPTFKIVVRLPANAFTGLTWVMSFDAYNRITSRITASADPVYTLSVPHWLIHH	705
ARO69946.1	626	LGTLNIRDMISDFKGVQYEKWITAGLVMPTFKIVVRLPANAFTGLTWVMSFDAYNRITSRITASADPVYTLSVPHWLIHH	705
QGP72536.1	626	LGTLNIRDMISDFKGVQYEKWITAGLVMPTFKIVVRLPANAFTGLTWVMSFDAYNRITSRITASADPVYTLSVPHWLIHH	705
QGP72538.1	626	LGTLNIRDMISDFKGVQYEKWITAGLVMPTFKIVVRLPANAFTGLTWVMSFDAYNRITSRITASADPVYTLSVPHWLIHH	705
ARO69913.1	626	LGTLNIRDMISDFKGVQYEKWITAGLVMPTFKIVVRLPANAFTGLTWVMSFDAYNRITSRITASADPVYTLSVPHWLIHH	705
ARO69924.1	626	LGTLNIRDMISDFKGVQYEKWITAGLVMPTFKIVVRLPANAFTGLTWVMSFDAYNRITSRITASADPVYTLSVPHWLIHH	705
ABI63924.1	626	LGTLNIRDMISDFKGVQYGKWITAGLVMPTFKIVIRLPANAFTGLTWVMSFDAYNRITSRITTSADPVYTLSVPHWLIHH	705
ARO69931.1	626	LGTLNIRDMISDFKGVQYEKWITAGLVMPTFKIVVRLPANAFTGLTWVMSFDAYNRITSRITASADPVYTLSVPHWLIHH	705
AWO67711.1	625	LGTLDIRDMILDFKGVQYEKWITAGLVMPTFKIVVRLPANAFTGLTWVMSFDAYNRITSRITASADPVYTLSVPHWLIHH	704
ARO69943.1	626	LGTLNIRDMISDFKGVQYEKWITAGLVMPTFKIVVRLPANAFTGLTWVMSFDAYNRITSRITASADPVYTLSVPHWLIHH	705
ADJ10928.1	626	LGTLNIRDMISDFKGVQYEKWITAGLVMPTFKIVIRLPANAFTGLTWVMSFDAYNRITSRITASADPVYTLSVPHWLIHH	705
ARO69939.1	626	LGTLNIRDMISDFKGVQYEKWITAGLVMPTFKIVVRLPANAFTGLTWVMSFDAYNRITSRITASADPVYTLSVPHWLIHH	705

```
ABI63938.1   626   LGTLNIRDMISDFKGVQYEKWITAGLVMPTFKIVIRLPANAFTGLTWVMSFDAYNRITSRITTSADPVYTLSVPHWLIHH  705
ABI63940.1   626   LGTLNIRDMISDFKGGQYEKWITAGLVMPTFKIVIRLPANAFTGLTWVMSFDAYNRITSRITTSADPVYTLSVPHWLIHH  705
ABI63929.1   626   LGTLNMRDMISDFKGVQYEKWITAGLVMPTFKIVIRLPANAFTGLTWVMSFDAYNRITSRITTSADPVYTLSVPHWLIHH  705
ABI63919.1   626   LGTLNIRDMISDFKGVQYEKWITAGLVMPTFKIVIRLPANAFTGLTWVMSFDAYNRITSRITTSADPVYTLSVPHWLIHH  705
ADJ10934.1   624   LGTLNIRDMISDFKGVQYEKWITAGLVMPTFKIVVRLPANAFTGLTWVMSFDAYNRITSRIIASADPVYTLSVPHWLIHH  703
ARO69937.1   626   LGTLNIRDMIADFKGVQYEKWITAGLVMPTFKIVVRLPANAFTGLTWVMSFDAYNRITSRITASADPVYTLSVPHWLIHH  705
ARO69928.1   626   LGTLNIRDMISDFKGVQYEKWITAGLVMPTFKIVVRLPANAFTGLTWVMSFDAYNRITSRITASADPVYTLSVPHWLIHH  705
ARO69927.1   626   LGTLNIRDMISDFKGVQYEKWITAGLVMPTFKIVVRLPANAFTGLTWVMSFDAYNRITSRITASADPVYTLSVPHWLIHH  705
ABI63934.1   625   LGTLNIRDMISDFKGVQYEKWITAGLVMPTFKIVIRLPANAFTGLTWVMSFDAYNRITSRITTSADPVYTLSVPHWLIHH  704
ARO69935.1   626   LGTLNIRDMISDFKGVQYEKWITAGLVMPTFKIVVRLPANAFTGLTWVMSFDAYNRITSRITASADPVYTLSVPHWLIHH  705
ARO69940.1   626   LGTLNIRDMISDFKGVQYEKWITAGLVMPTFKIVVRLPANAFTGLTWVMSFDAYNRITSKITASADPVYTLSVPHWLIHH  705
ARO69933.1   626   LGTLNIRDMISDFKGVQYEKWITAGLVMPTFKIVVRLPANAFTGLTWVMSFDAYNRITSRITASADPVYTLSVPHWLIHH  705
ABI63922.1   626   LGTLNIRDMISDFKGVQYEKWITAGLVMPTFKIVIRLPANAFTGLTWVMSFDAYNRITSRITTSADPVYTLSVPHWLIHH  705
ABI63936.1   626   LGTLNIRDMISDFKGVQYEKWITAGLVMPTFKIVIRLPANAFTGLTWVMSFDAYNRITSRITTSADPVYTLSVPHWLIHH  705
ABI63918.1   626   LGTLNIRDMISDFKGVQYEKWITAGLVMPTFKIVIRLPANAFTGLTWVMSFDAYNRITSRITTSADPVYTLSVPHWLIHH  705
ABI63931.1   625   LGTLNIRDMISDFKGVQYEKWITAGLVMPTFKIVVRLPANAFTGLTWVMSFDAYDRITSRITTSADPVYTLSVPHWLIHH  704
QYU59230.1   626   LGTLNIRDMISDFKGVQYEKWITAGLVMPTFKIVVRLPANAFTGLTWVMSFDAYNRITSRITTSADPVYTLSVPHWLIHH  705
ARO69922.1   626   LGTLNIRDMISDFKGVQYEKWITAGLVMPTFKIVVRLPANAFTGLTWVMSFDAYNRITSKITASADPVYTLSVPHWLIHH  705
ABI63941.1   626   LGTLNIRDMISDFKGVQYEKWITAGLVMPTFKIVIRLPANAFTGLTWVMSFDAYNRITSRITTSADPVYTLSVPHWLIHH  705
ARO69949.1   626   LGTLNIRDMISDFKGVQYEKWITAGLVMPTFKIVVRLPANAFTGLTWVMSFDAYNRITSRITASADPVYTLSVPHWLIHH  705
ARO69917.1   626   LGTLNIRDMISDFKGVQYEKWITAGLVMPTFKIVVRLPANAFTGLTWVMSFDAYNRITSRITASADPVYTLSVPHWLIHH  705
ARO69915.1   626   LGTLNIRDMISDFKGVQYEKWITAGLVMPTFKIVVRLPANAFTGLTWVMSFDAYNRITSKITASADPVYTLSVPHWLIHH  705
QYU59228.1   626   LGTLNIRDMISDFKGVQYEKWITAGLVMPTFKIVVRLPANAFTGLTWVMSFDAYNRITSRITTSADPVYTLSVPHWLIHH  705
AGN29358.1   626   LGTLNIRDMISDFKGVQYEKWITAGLVMPTSKIVIRLPAMAFTGLTWVMSFDAYNRITSRITTSADPVYTLSVPHWLIHH  705
ARO69944.1   626   LGTLNIRDMISDFKGVQYEKWITAGLVMPTFKIVVRLPANAFTGLTWVMSFDAYNRITSKITASADPVYTLSVPHWLIHH  705
AGN29367.1   623   LGTLNIRDMISDFKGVQYEKWITAGLVMPTFKIVIRLPASAFTGLTWVMSFDAYNRITSRITASADPVYTLSVPHWLIHH  702
ABI63921.1   626   LGTLNIRDMISDFKGVQYEKWITAGLVMPTFKIVIRLPANAFTGLTWVMSFDAYNRITSRITTSADPVYTLSVPHWLIHH  705
ABI63923.1   626   LGTLNIRDMISDFKGVQYEKWITAGLVMPTFKIVIRLPANAFTGLTWVMSFDAYNRITSRITTSADPVYTLSVPHWLIHH  705
CDN67470.1   623   LGTLNIRDMISDFKGVQYEKWITAGLVMPIFKIIIRLPANAFTGLTWVMSFDAYNRITSRITTSADPVYILSVPHWLIHH  702
CDN67474.1   626   LGTLNIRDMISDFKGVQYEKWITAGLVMPVFKIVIRLPANAFTGLTWVMSFDAYNRITSRITASADPVYTLSVPHWLIHH  705
AAX18710.1   626   LGTLNIRDMISDFKGVQYEKWITAGLVMPTFKIVVRLPANAFTGLTWVVSFDAYNRITSRITASADPIYTLSVPHWLIHH  705
QBZ78370.1   624   LGTLNIRDMISDFKGVQYEKWITAGLVMPTFKIVVRLPANAFTGLTWVMSFDAYNRITSRITVSADPVYTLSVPHWLIHH  703
ARO69923.1   626   LGTLNIRDMIADFKGVQYEKWITAGLVMPTFKIVVRLPANAFTGLTWVMSFDAYNRITSRITASADPVYTLSVPHWLIHH  705
ARO69907.1   626   LGTLNIRDMISDFKGVQYEKWITAGLVMPTFKIVVRLPANAFTGLTWVMSFDAYNRITSRITASADPVYTLSVPHWLIHH  705
ARO69942.1   626   LGTLNIRDMISDFKGVQYEKWITAGLVMPTFKIVVRLPANAFTGLTWVMSFDAYNRITSKITASADPVYTLSVPHWLIHH  705
ARO69909.1   626   LGTLNIRDMISDFKGVQYEKWITAGLVMPTFKIVVRLPANAFTGLTWVMSFDAYNRITSRITASADPVYTLSVPHWLIHH  705
AAX18711.1   626   LGTLNIRDMIADFKGVQYEKWITAGLVMPIFKIVVRLPANAFTGLTWVMSFDAYNRITSRITVSADPVYTLSVPHWLIHH  705
ARO69936.1   626   LGTLNIRDMISDFKGVQYEKWITAGLVMPTFKIVVRLPANAFTGLTWVMSFDAYNRITSRITASADPVYTLSVPHWLIHH  705
AGN29357.1   626   LGTLNIRDMISDFKGVQYEKWITAGLVMPTFKIVIRLPAIAFTGLTWVMSFDAYNRITSRITASADPIYTLSVPHWLIHH  705
ARO69919.1   626   LGTLNIRDMISDFKGVQYEKWINAGLVMPIFKIVVRLPANAFTGLTWVMSFDAYNRITSKITASADPVYTLSVPHWLIHH  705
ADJ10929.1   626   LGTLNIRDMISDFRGVQYEKWITAGLVMPTFKIVVRLPANAFTGLTWVMSFDAYNRITSRITTSADPVYTLSVPHWLIHH  705
ABI63943.1   626   LGTLNIRDMISDFKGVQYEKWITAGLVMPTFKIVIRLPANAFTGLTWVMSFDAYNRITSRITTSADPVYTLSVPHWLIHH  705
AGN29365.1   626   LGTLNIRDMISDFKGVQYEKWITAGLVMPTFKIVTRLPANAFTGLTWVMSFDAYNRITSKITASADPVYTLSVPHWLIHH  705
ABI63930.1   625   LGTLNIRDMISDFKGVQYEKWITAGLVMPTIKIVVRLPANAFTGLTWVMSFDAYNRITSRITTSADPVYTLSVPHWLIHH  704
ABI63942.1   625   LGTLNIRDMISDFKGVQYEKWITAGLVMPTFKIVVRLPANAFTGLTWVMSFDAYNRITSRITTSADPVYTLSVPHWLIHH  704
ADJ10939.1   626   LGTLNIRDMISDFKGVQYEKWITAGLVMLTFKIVVRLPANAFTGLTWVMSFDAYNRITSRITTSADPVYTLSVPHWLIHH  705
ABI63928.1   625   LGTLNIRDMISDFKGVQYEKWITAGLVMPTFKIVVRLPANAFTGLTWVMSFDAYNRITSRITTSADPVYTLSVPHWLIHH  704
CDN67476.1   623   LGTLNIRDMISDFKGVQYEKWITAGLVMPVFEIVIRLPANAFTGLTWVMSFDAYNRITSRITASADPIYTLSVPHWLIHH  702
ABI63933.1   625   LGTLNIRDMISDFKGVQYEKWITAGLVMPTFKIVVRLPANAFTGLTWVMSFDAYNRITSRITTSADPVYTLSVPHWLIHH  704
AAX18707.1   626   LGTLNIRDMISDFKGVQYEKWITAGLVMPTFKIVVRLPADAFTGLTWVMSFDAYNRITSRITASADPIYTLSVPHWLIHH  705
ACZ58631.1   624   LGTLNIRDMISDFKGVQYEKWITAGLVMPIFKIVVRLPANAFTGLTWVMSFDAYNRITSRITASADPVYTLSVPHWLIHH  703
AGN29359.1   626   LGTLNIRDMISDFKGVQYEKWITAGLVMPTSKIVIRLPAIAFTGLTWVMSFDAYNRITSRITTSADPVYTLSVPHWLIHH  705
AGN29361.1   626   LGTLNIRDMISDFKGVQYEKWITAGLVMPTFKIVIRLPAIAFTGLTWVMSFDAYNRITSRITTSADPVYTLSVPHWLIHH  705
ABI63935.1   625   LGTLNIRDMISDFKGVQYEKWITAGLVMPTFKIVVRLPANAFTGLTWVMSFDAYNRITSRITTSADPVYTLSVPRWLIHH  704
AGN29360.1   627   LGTLNMRDMISDFKGVQYEKWITAGLVMPTFKIIIRLPANAFTGLTWVMSFDAYNRITSRITASADPVYTLSIPHWLIHH  706
AWO67712.1   625   LGTLNIRDMISDFKGVQYEKWITAGLVMPTFKIVVRLPANAFTGLTWVMSFDAYNRITSRITTSADPVYTLSVPHWLIHH  704
AYA22300.1   623   LGTLNIRDMITDFKGVQYEKWITAGLVMPIFKIVVRLPANAFTGLTWVMSFDAYNRITSRITASADPIYTLSVPHWLIHH  702
QBZ78373.1   623   LGTLNIRDMISDFKGVQYEKWITAGLVMPIFKIIIRLPANAFTGLTWVMSFDAYNRITSRITASADPVYTLSVPHWLIHH  702
AYQ93056.1   623   LGTLNIRDMISDFKGVQYEKWITAGLVMPTFKIVVRLPANAFTGLTWVMSFDAYNRITSRIAASADPVYTLSVPHWLIHH  702
AYQ93054.1   623   LGTLNIRDMILDFKGVQYEKWITAGLVMPTFKIVVRLPANAFTGLTWVMSFDAYNRITSRIAASADPVYTLSVPHWLIHH  702
AAX18709.1   623   LGTLNIRDMITDFKGVQYEKWITAGLVMPIFKIVVRLPANAFTGLTWVMSFDAYNRITSRITASADPIYTLSVPHWLIHH  702
AYQ93052.1   624   LGTLNIRDMISDFKGVQYEKWITAGLVMPIFKIVVRLPANAFTGLTWVMSFDAYNRITSRITTSADPIYTLSVPHWLIHH  703
BCT87062.1   625   LGTLNIRDMITDFKGVQYEKWITAGLVMPIFKIVVRLPANAFTGLTWVMSFDAYNRITSRITTSADPVYTLSVPHWLIHH  704
AGN29364.1   623   LGTLNIRDMISDFKGVQYGKWITAGLVMPTFKIVIRLPANAFTGLTWVMSFDAYNRITSRITASADPVYTLSVPHWLIHH  702
QYU59234.1   623   LGTLNMRDMISDFKGVQYEKWITAGLVMPIFKIVIRLPANAFTGLTWVMSFDAYNRITSRITTSADPVYTLSVPHWLIHH  702
CDN67475.1   623   LGTLNIRDMISDFKGVQYEKWITAGLVMPTFKVVVRLPANAFTGLTWVMSFDAYNRITSRITVSADPVYTLSVPHWLIHH  702
AGN29356.1   623   LGTLNIRDMISDFKGVQYEKWITAGLVMPTFKIVIRLPANAFTGLTWVMSFDAYNRITSRITASADPVYTLSVPHWLIHH  702
AGN29363.1   623   LGTLNIRDMISDFKGVQYEKWITAGLVMPTFKIVIRLPANAFTGLTWVMSFDAYNRITSRITASADPVYTLSVPHWLIHH  702
AFV73369.1   626   LGTLNIRDMISDFKGVQYEKWITAGLVMPTFKIVVRLPANAFTGLTWVMSFDAYNRITSRITASADPVYTLSVPHWLIHH  705
AFV34732.1   623   LGTLNIRDMILDFKGVQYEKWITAGLVMPTFKIVVRLPANAFTGLTWVMSFDAYNRITGRIAASADPIYTLSVPHWLIHH  702
AFI43912.1   627   LGTLNIRDMIIDFKGVQYEKWITAGLVMPIFKIVVRLPANAFTGLTWVMSFDAYNRITSRITASADPVYTLSVPHWLIHH  706
CDN67472.1   623   LGTLNIRDMVSDFKGVQYEKWITAGLVMPIFKVVVRLPANAFTGLTWVMSFDAYNRITSRITTSADPVYTLSVPHWLIHH  702
AGN29355.1   623   LGTLNIRDMISDFKGVQYEKWITAGLVMPTFKVVIRLPASAFTGLTWVMSFDAYNRITSRITASADPVYTLSVPHWLIHH  702
AFI43911.1   627   LGTLNIRDMITDFKGVQYEKWITAGLVMPIFKIVVRLPANAFTGLTWVMSFDAHNRITSRITASADPVYTLSVPHWLIHH  706
ADJ10940.1   626   LGTLNIRDMISDFKGVQYEKWITAGLVMPTFKIVIRLPANAFTGLTWVMSFDAYNRITSRITASADPVYTLSVPHWLIHH  705
DAZ85718.1   626   LGTLNIRDMISDFKGVQYEKWITAGLVMPIFKIVVRLPANAFTGLTWVMSFDAYNRITSRITTSADPVYTLSVPHWLIHH  705
ADJ10930.1   626   LGTLNIRDTISDFKGVQYERWITAGLVMPTFKIVVRLPANAFTGLTWVMSFDAYNRITSRITISADPVYTLSVPHWLIHH  705
ADJ10933.1   626   LGTLNIRDTISDFKGVQYERWITAGLVMPTFKIVVRLPANAFTGLTWVMSFDAYNRITSRITISADPVYTLSVPHWLIHH  705
ADJ10935.1   626   LGTLNIRDMISDFKGVQYEKWITAGLVMPTFKIVIRLPANAFTGLTWVMSFDAYNRITSRITASADPVYTLSVPHWLIHH  705
ADJ10941.1   626   LGTLNIRDTISDFKGVQYERWITAGLVMPTFKIVVRLPANAFTGLTWVMSFDAYNRITSRITISADPVYTLSVPHWLIHH  705
DAZ85719.1   626   LGTLNIRDMISDFKGVQYEKWITAGLVMPTFKIIVRLPANAFTGLTWVMSFDAYNRITSRITASADPIYTLSVPHWLIHH  705
ADJ10936.1   626   LGTLNIRDTISDFKGVQYERWITAGLVMPTFKIVVRLPANAFTGLTWVMSFDAYNRITSRITISADPVYTLSVPHWLIHH  705
ABR09917.1   626   LGTLDIRDMIADFKGVQYEKWITAGLVMPTFKIVVRLPANAFTGLTWVMSFDAYNRITSRITTSADPVYTLSVPHWLIHH  705
CDN67473.1   631   LGTLNIRDMISDFKGVQYEKWITAGLVMPTFKIVVRLPANAFTGLTWVMSFDAYNRITSRITASADPVYTLSVPHWLIHH  710
QYU59232.1   623   LGTLNIRDMISDFKGVQYEKWITAGLVMPTFKIVVRLPANAFTGLTWVMSFDAYSRITSRITVSADPIYTLSVPHWLIHH  702
AGN29362.1   623   LGTLNIRDMISDFKGVQYEKWITAGLVMPTSKIVIRLPANAFTGLTWVMSFDAYNRITSRITTSADPVYTLSVPHWLIHH  702
ACZ58625.1   625   LGTLNLRDMISDFKGVQYEKWITAGLVMPTFKIVIRLPANAFTGLTWVMSFDAYNRITSRITASADPIYTLSVPHWLIHH  704
ACZ58626.1   625   LGTLNIRDMISDFKGVQYEKWITAGLVMPIFKIVIRLPANAFTGLTWVMSFDAYNRITSRITASADPIYTLSVPHWLIHH  704
```

```
ACZ58624.1   625   LGTLNIRDMISDFKGVQYEKWITAGLVMPIFKIVIRLPANAFTGLTWVMSFDAYNRITSRITASADPIYTLSVPHWLIHH  704
AGN29366.1   626   LGTLNIRDMISDFKGVQYEKWITAGLVMPTFKIVIRLPANAFTGLTWVMSFDAYNRITSRITASADPVYTLSVPHWLIHH  705
QBZ78368.1   625   LGTLNIRDMISDFKGVQYEKWITAGLVMPIFKIVIRLPANAFTGLTWVMSFDAYNRITSRITASADPIYTLSVPHWLIHH  704
AFI43914.1   634   LGTLNIRDMIIDFKGVQYEKWITAGLVMPVFKIVIRLPANAFTGLTWVMSFDAYNRITSRITTSADPVYTLSVPHWLIHH  713
AFI43913.1   634   LGTLNIRDMITDFKGVQYERWITAGLVMPIFKIVIRLPANAFTGLTWVMSFDAYNRITSRITTSADPVYTLSVPHWLIHH  713
AFV34756.1   565   LGTLNIRDMISDFKGVQYEKWITAGLVMPTFKIVVRLPANAFTGLTWVMSFDAYNRITSRITASADPVYTLSVPHWLIHH  644
QYU59231.1   625   LGTLNIRDMISDFKGVQYEKWITAGLVMPIFKIVVRLPANAFTGLTWVMSFDAYNRITSRITTSADPVYTLSVPHWLIHH  704
QYU59233.1   632   LGTLNIRDMISDFKGVQYEKWITAGLVMPVFKIIVRLPANAFTGLTWVMSFDAYNRITSRISASADPVYTLSVPHWLIHH  711
APD26307.1   634   LGTLNIRDMIIDFKGIQYEKWITAGLVMPTFKIVVRLPANAFTGLTWVMSFDAYNRITSRITTSADPIYTLSVPHWLIHH  713
QBZ78371.1   454   LGTLNIRDMISDFKGVQYEKWITAGLVMPIFKIVVRLPANAFTGLTWVMSFDAYNRITSRITTSADPIYTLSVPHWLIHH  533


NP_619706.1  705   KLGTFSCEIDYGELCGHAMWFKSTTFESPRLHFTCLTGNNKELAADWQAVVELYAELEEATSFLGKPTLVFDPGVFNGKF  784
AWO68043.1   706   KLGTFSCEIDYGELCGHAMWFKSTTFESPRLHFTCLTGNNKELAADWQAVVELYAELEEATSFLGKPTLVFDPGVFNGKF  785
ADJ10937.1   706   KLGTFSCEIDYGELCGHAMWFKSTTFESPRLHFTCLTGNNKELAADWQAVAELYAELEEATSFLGKPTLVFDPGVFNGKF  785
ADJ10931.1   706   KLGTFSREIDYGELCGHAMWFKSTTFESPRLHFTCLTGNNKELAADWQAVVELYAELEEATSFLGKPTLVFDPGVFNGKF  785
ADJ10938.1   706   KLGTFSCEIDYGELCGHAMWFKSTTFESPRLHFTCLTGNNKELAADWQAVVELYAELEEATSFLGKPTLVFDPGVFNGKF  785
QYU59229.1   706   KLGTFSCEIDYGELCGHAMWFKSTTFESPRLHFTCLTGNNKELAADWQAVVELYAELEEATSFLGKPTLVFDPGVFNGKF  785
ACZ58630.1   706   KLGTFSCEIDYGELCGHAMWFKSTTFESPRLHFTCLTGNNKELAADWQAVVELYAELEEATSFLGKPTLVFDPGVFDGKF  785
QGP72535.1   706   KLGTFSCEIDYGELCGHAMWFKSTTFESPRLHFTCLTGNNKELAADWQAVVELYAELEEATSFLGKPTLIFDPGVFNGKF  785
Q91HK5.1     706   KLGTFSCEIDYGELCGHAMWFKSTTFESPKLHFTCLTGNNKELAADWQAVVELYAELEEASSFLGKPTLVFDPGVFNGKF  785
ARO69912.1   706   KLGTFSCEIDYGELCGHAMWFKSTTFESPRLHFTCLTGNNKELAADWQAVVELYAELEEAITFLGKPTLVFDPGVFNGKF  785
AFI71814.1   706   KLGTFSCEVNYGELCGHAMWFKSTTFESPRLHFTCLTGNNKELAADWQAVVELYAELEEATSFLGKPTLVFDPGVFNGKF  785
ADJ10932.1   706   KLGTFSCEIDYGELCGHAMWFKSTTFESPRLHFTCLTGNNKELAADWQAVVELYAELEEATSFLGKPTLVFDPGVFDGKF  785
ARO69925.1   706   KLGTFSCEIDYGELCGHAMWFKSTTFESPRLHFTCLTGNNKELAADWQAVVELYAELEEATTFLGKPTLVFDPGVFNGKF  785
ARO69911.1   706   KLGTFSCEIDYGELCGHAMWFKSTTFESPRLHFTCLTGNNKELAADWQAVVELYAELEEAITFLGKPTLVFDPGVFNGKF  785
ACZ58628.1   706   KLGTFSCEIDYGELCGHAMWFKSTTFESPRLHFTCLTGNNKELAADWQAVVELYTELEEATSFLGKPTLVFDPGVFNGRF  785
ABI63932.1   706   KLGTFSCEVDYGELCGHAMWFKSTTFESPRLHFTCLTGNNKELAADWQAVVELYAELEEATSFLGKPTLVFDPGVFNGKF  785
ARO69945.1   706   KLGTFSCEIDYGELCGHAMWFKSTTFESPRLHFTCLTGNNKELAADWQAVVELYAELEEAITFLGKPTLVFDPGVFNGKF  785
ARO69930.1   706   KLGTFSCEIDYGELCGHAMWFKSTTFESPRLHFTCLTGNNKELAADWQAVVELYAELEEAITFLGKPTLVFDPGVFNGKF  785
ABI63925.1   706   KLGTFSCEVDYGELCGHAMWFKSTTFESPRLHFTCLTGNNKELAADWQAVVELYAELEEATSFLGKPTLVFDPGVFNGKF  785
ARO69906.1   706   KLGTFSCEIDYGELCGHAMWFKSTTFESPRLHFTCLTGNNKELAADWQAVVELYAELEEATTFLGKPTLVFDPGVFNGKF  785
ACZ58627.1   706   KLGTFSCEIDYGELCGHAMWFKSTTFESPRLHFTCLTGNNKELAADWQAVVELYTELEEATSFLGKPTLVFDPGVFNGRF  785
ARO69920.1   706   KLGTFSCEIDYGELCGHAMWFKSTTFESPRLHFTCLTGNNKELAADWQAVVELYAELEEAITFLGKPTLVFDPGVFNGKF  785
ACZ58629.1   706   KLGTFSCEIDYGELCGHAMWFKSTTFESPRLHFTCLTGNNKELAADWQAVVELYTELEEATSFLGKPTLVFDPGVFNGRF  785
AGT42201.1   706   KLGTFSCEVDYGELCGHAMWFKSTTFESPRLHFTCLTGNNKELAADWQAVVELYAELEEATSFLGKPTLVFDPGVFNGKF  785
ARO69914.1   706   KLGTFSCEIDYGELCGHAMWFKSTTFESPRLHFTCLTGNNKELAADWQAVVELYAELEEAITFLGKPTLVFDPGVFNGKF  785
ABI63916.1   706   KLGTFSCEVDYGELCGHAMWFKSTTFESPRLHFTCLTGNNKELAADWQAVVELYAELEEATSFLGKPTLVFDPGVFNGKF  785
ARO69918.1   706   KLGTFSCEIDYGELCGHAMWFKSTTFESPRLHFTCLTGNNKELAADWQAVVELYAELEEAITFLGKPTLVFDPGVFNGKF  785
ARO69948.1   706   KKGTFSCEIDYGELCGHAMWFKSTTFESPRLHFTCLTGNNKELAADWQAVVELYAELEEATSFLGKPTLVFDPGVFNGKF  785
QYA72353.1   706   KLGTFSCEIDYGELCGHAMWFKSTTFESPRLHFTCLTGNNKELAADWQAVVELYAELEEASSFLGKPTLVFDPGVFNGKF  785
ABI63917.1   706   KLGTFSCEVDYGELCGHAMWFKSTTFESPRLHFTCLTGNNKELAADWQAVVELYAELEEATSFLGKPTLVFDPGVFNGKF  785
ARO69941.1   706   KKGTFTCEIDYGELCGHAMWFKSTTFESPRLHFTCLTGNNKELAADWQAVVELYAELEEATSFLGKPTLVFDPGVFNGKF  785
ABI63926.1   706   KLGTFSCEVDYGELCGHAMWFKSTTFESPRLHFTCLTGNNKELAADWQAVVELYAELEEATSFLGKPTLVFDPGVFNGKF  785
CDN67471.1   706   KLGTFTCEMDYGELCGHAMWFKSTTFESPRLHFTCLTGNNKELAADWQAVVELYAELEEATSFLGKPTLVFDPGVFNGKF  785
ABI63920.1   706   KLGTFSCEVDYGELCGHAMWFKSTTFESPRLHFTCLTGNNKELAADWQAVVELYAELEEATSFLGKPTLVFDPGVFNGKF  785
ABI63927.1   706   KLGTFSCEVDYGELCGHAMWFKSTTFESPRLHFTCLTGNNKELAADWQAVVELYAELEEATSFLGKPTLDFDPGVFNGKF  785
ARO69908.1   706   KKGTFTCEIDYGELCGHAMWFKSTTFESPRLHFTCLTGNNKELAADWQAVVELYAELEEATSFLGKPTLVFDPGVFNGKF  785
ARO69916.1   706   KKGTFTCEIDYGELCGHAMWFKSTTFESPRLHFTCLTGNNKELAADWQAVVELYAELEEATSFLGKPTLVFDPGVFNGKF  785
ARO69910.1   706   KKGTFSCEIDYGELCGHAMWFKSTTFESPRLHFTCLTGNNKELAADWQAVVELYAELEEATSFLGKPTLVFDPGVFNGKF  785
DAZ85720.1   706   KLGTFSCEIDYGELCGHAMWFKSTTFESPRLHFTCLTGNNKELAADWQAVVELYAELEEATSFLGKPTLVFDPGVFNGKF  785
AAX18708.1   706   KLGTFTCEIDYGELCGHAMWFKSTTFESPRLHFTCLTGNNKELAADWQAVVELYAELEEAASFLGKPTLVFDPGVFDGKF  785
AYA22298.1   706   KLGTFTCEIDYGELCGHAMWFKSTTFESPRLHFTCLTGNNKELAADWQAVVELYAELEEAASFLGKPTLVFDPGVFDGKF  785
ABI63939.1   706   KLGTFSCEVDYGELCGHAMWFKSTTFESPRLHFTCLTGNNKELAADWQAVVELYAELEEATSFLGKPTLVFDPGVFNGKF  785
ARO69926.1   706   KKGTFTCEIDYGELCGHAMWFKSTTFESPRLHFTCLTGNNKELAADWQAVVELYAELEEATSFLGKPTLVFDPGVFNGKF  785
QGP72537.1   706   KLGTFSCEIDYGELCGHAMWFKTTTFESPRLHFTCLTGNNKELAADWQAVVELYAELEEATSFLGKPTLVFDPGVFNGKF  785
ARO69947.1   706   KKGTFTCEIDYGELCGHAMWFKSTTFESPRLHFTCLTGNNKELAADWQVVVELYAELEEATSFLGKPTLVFDPGVFNGKF  785
ARO69921.1   706   KKGTFTCEIDYGELCGHAMWFKSTTFESPRLHFTCLTGNNKELAADWQAVVELYAELEEATSFLGKPTLVFDPGVFNGKF  785
ARO69946.1   706   KKGTFTCEIDYGELCGHAMWFKSTTFESPRLHFTCLTGNNKELAADWQAVVELYAELEEATSFLGKPTLVFDPGVFNGKF  785
QGP72536.1   706   KLGTFSCEIDYGELCGHAMWFKSTTFESPRLHFTCLTGNNKELAADWQAVVELYAELEEAASFLGKPTLVFDPGVFNGKF  785
QGP72538.1   706   KLGTFSCEIDYGELCGHAMWFKTTTFESPRLHFTCLTGNNKELAADWQAVVELYAELEEATSFLGKPTLVFDPGVFNGKF  785
ARO69913.1   706   KKGTFTCEIDYGELCGHAMWFKSTTFESPRLHFTCLTGNNKELAADWQAVVELYAELEEATSFLGKPTLVFDPGVFNGKF  785
ARO69924.1   706   KKGTFTCEIDYGELCGHAMWFKSTTFESPRLHFTCLTGNNKELAADWQAVVELYAELEEATSFLGKPTLVFDPGVFNGKF  785
ABI63924.1   706   KLGTFSCEVDYGELCGHAMWFKSTTFESPRLHFTCLTGNNKELAADWQAVVELYAELEEATSFLGKPTLVFDPGVFNGKF  785
ARO69931.1   706   KKGTFTCEIDYGELCGHAMWFKSTTFESPRLHFTCLTGNNKELAADWQAVVELYAELEEATSFLGKPTLVFDPGVFNGKF  785
AWO67711.1   705   KLGTFSCEIDYGELCGHAMWFRSTTFESPRLHFTCLTGNNKELAADWQAVVELYAELEEATSFLGKPTLVFDPGVFNGKF  784
ARO69943.1   706   KKGTFTCEIDYGELCGHAMWFKSTTFESPRLHFTCLTGNNKELAADWQGVVELYAELEEATSFLGKPTLVFDPGVFNGKF  785
ADJ10928.1   706   KLGTFSCEIDYGELCGHAMWFKSTTFESPRLHFTCLTGNNKELAADWQAVVELYAELEEATSFLGKPTLVFDPGVFNGKF  785
ARO69939.1   706   KKGTFTCEIDYGELCGHAMWFKSTTFESPRLHFTCLTGNNKELAADWQAVVELYAELEEATSFLGKPTLVFDPGVFNGKF  785
ABI63938.1   706   KLGTFSCEVDYGELCGHAMWFKSTTFESPRLHFTCLTGNNKELAADWQAVVELYAELEEAISFLGKPTLVFDPGVFNGKF  785
ABI63940.1   706   KLGTFSCEVDYGELCGHAMWFKSTTFESPRLHFTCLTGNNKELAADWQAVVELYAELEEATSFLGKPTLVFDPGVFNGKF  785
ABI63929.1   706   KLGTFSCEVDYGELCGHAMWFKSTTFESPRLHFTCLTGNNKELAADWQAVVELYAELEEATSFLGKPTLVFDPGVFNGKF  785
ABI63919.1   706   KLGTFSCEVDYGELCGHAMWFKSTTFESPRLHFTCLTGNNKELAADWQAVVELYAELEEATSFLGKPTLVFDPGVFNGKF  785
ADJ10934.1   704   KLGTFSCEIDYGALCGHAMWFKSTTFESPRLHFTCLTGNNKELAADWQAVVELYAELEEATSFLGKPTLVFDPGVFNGKF  783
ARO69937.1   706   KKGTFTCEIDYGELCGHAMWFKSTTFESPRLHFTCLTGNNKELAADWQAVVELYAELEEATSFLGKPTLVFDPGVFNGKF  785
ARO69928.1   706   KKGTFTCEIDYGELCGHAMWFKSTTFESPRLHFTCLTGNNKELAADWQAVVELYAELEEATSFLGKPTLVFDPGVFNGKF  785
ARO69927.1   706   KKGTFTCEIDYGELCGHAMWFKSTTFESPRLHFTCLTGNNKELAADWQAVVELYAELEEATSFLGKPTLVFDPGVFNGKF  785
ABI63934.1   705   KLGTFSCEVDYGELCGHAMWFKSTTFESPRLHFTCLTGNNKELAADWQAVVELYAELEEATSFLGKPTLVFDPGVFNGKF  784
ARO69935.1   706   KKGTFTCEIDYGELCGHAMWFKSTTFESPRLHFTCLTGNNKELAADWQAVVELYAELEEATSFLGKPTLVFDPGVFNGKF  785
ARO69940.1   706   KKGTFSCEIDYGELCGHAMWFKSTTFESPRLHFTCLTGNNKELAADWQAVVELYAELEEATSFLGKPTLVFDPGVFNGKF  785
ARO69933.1   706   KKGTFTCEIDYGELCGHAMWFKSTTFESPRLHFTCLTGNNKELAADWQAVVELYAELEEATSFLGKPTLVFDPGVFNGKF  785
ABI63922.1   706   KLGTFSCEVDYGELCGHAMWFKSTTFESPRLHFTCLTGNNKELAADWQAVVELYAELEEATSFLGKLTLVFDPGVFSGKF  785
ABI63936.1   706   KLGTLSCEVDYGELCGHAMWFKSTTFESPRLHFTCLTGNNKELAADWQAVVELYAELEEATSFLGKPTLVFDPGVFNGKF  785
ABI63918.1   706   KLGTFSCEVDYGELCGHAMWFKSTTFESPRLHFTRLTGNNKELAADWQAVVELYAELEEATSFLGKPTLVFDPGVFNGKF  785
ABI63931.1   705   KLGTFSCEIDYGELCGHAMWFKSTTFESPRLHFTCLTGNNKELAADWQAVVELYAELEEATSFLGKPTLVFDPGVFDGKF  784
QYU59230.1   706   KLGTFTCEVDYGELCGHAMWFKSTTFESPRLHFTCLTGNNKELAADWQAVVELYAELEEATSFLGKPTLVFDPGVFSGKF  785
ARO69922.1   706   KKGTFSCEIDYGELCGHAMWFKSTTFESPRLHFTCLTGNNKELAADWQAVVELYAELEEATSFLGKPTLVFDPGVFNGKF  785
ABI63941.1   706   KLGTFSCEVDYGELCGHAMWFKSTTFESPRLHFTCLTGNNKELAAGWQAVVELYAELEEATSFLGKPTLVFDPGVFNGKF  785
```

```
ARO69949.1   706   KKGTFTCEIDYGELCGHAMWFKSTTFESPRLHFTCLTGNNKELAADWQAVVELYAELEEATSFLGKPTLVFDPGVFNGKF  785
ARO69917.1   706   KKGTFTCEIDYGELCGHAMWFKSTTFESPRLHFTCLTGNNKELAADWQAVVELYAELEEATSFLGKPTLVFDPGVFNGKF  785
ARO69915.1   706   KKGTFSCEIDYGELCGHAMWFKSTTFESPRLHFTCLTGNNKELAADWQAVVELYAELEEATSFLGKPTLVFDPGVFNGKF  785
QYU59228.1   706   KLGTFSCEIDYGELCGHAMWFKSTTFESPRLHFTCLTGNNKELAADWQAVVELYAELEEATSFLGKPTLVFDPGVFNGKF  785
AGN29358.1   706   KLGTFSCEIDYGELCGHAMWFKSTTFESPRLHFTCLTGNNKELAADWQAVVELYAELEEATSFLGKPTLVFDPGVFTGQF  785
ARO69944.1   706   KKGTFSCEIDYGELCGHAMWFKSTTFESPRLHFTCLTGNNKELAADWQAVVELYAELEEATSFLGKPTLVFDPGVFNGKF  785
AGN29367.1   703   KLGTFSCEIDYGELCGHAMWFKSTTFESPRLHFTCLTGNNKELAADWQAVVELYAELEEATTFLGKPTLVFDPGVFNGKF  782
ABI63921.1   706   KLGTFSCEVDYGELCGHAMWFKSTTFESPRLHFTRLTGNNKELAADWQAVVEPYAELEEATSFLGKPTLVFDPGVFNGKF  785
ABI63923.1   706   KLGTFSCEVDYGELCGHAMWFKSTTFESPRLHFTCLTGNNKELAADWQAVVELYAELEEATSFLGKPTLVFDPGVFNGKF  785
CDN67470.1   703   KLGTFSCEIDYGELCGHAMWFKSTTFESPRLHFTCLTGNNKELAADWQAVVELYAELEEATSFLGRPTLVFDPGVFNGKF  782
CDN67474.1   706   KLGTFACEIDYGELCGHAMWFKSTTFESPRLHFTCLTGNNKELAADWQAVVELYAELEEATSFLGKPTLVFDPGVFNGKF  785
AAX18710.1   706   KLGTFTCEIDYGELCGHAMWFKSTTFESPRLHFTCLTGNNKELAADWQAVVELYAELEEATAFLGKPTLVFDPGVFNGKF  785
QBZ78370.1   704   KLGTFSCEIDYGELCGHAMWFKSTTFESPRLHFTCLTGNNKELAADWQAVVELYAELEEASSFLGRPTLVFDPGVFDGKF  783
ARO69923.1   706   KKGTFTCEIDYGELCGHAMWFKSTTFESPRLHFTCLTGNNKELAADWQAVVELYAELEEATSFLGKPTLVFDPGVFNGKF  785
ARO69907.1   706   KKGTFTCEIDYGELCGHAMWFKSTTFESPRLHFTCLTGNNKELAADWQAVVELYAELEEATSFLGKPTLVFDPGVFNGKF  785
ARO69942.1   706   KKGTFSCEIDYGELCGHAMWFKSTTFESPRLHFTCLTGNNKELAADWQAVVELYAELEEATSFLGKPTLVFDPGVFNGKF  785
ARO69909.1   706   KKGTFSCEIDYGELCGHAMWFKSTTFESPRLHFTCLTGNNKELAADWQAVVELYAELEEATSFLGKPTLVFDPGVFNGKF  785
AAX18711.1   706   KLGTFSCEIDYGELCGHAMWFKSTTFESPRLHFTCLTGNNKELAADWQAVVELYAELEEAASFLGKPTLVFDPGVFDGKF  785
ARO69936.1   706   KKGTFTCEIDYGELCGHAMWFKSTTFESPRLHFTCLTGNNKELAADWQAVVELYAELEEATSFLGKPTLVFDPGVFNGKF  785
AGN29357.1   706   KLGTFSCEIDYGELCGHAMWFKSTTFESPRLHFTCLTGNNKELAADWQAVVELYAELEEATSFLGKPTLVFDPGVFNGKF  785
ARO69919.1   706   KKGTFSCEIDYGELCGHAMWFKSTTFESPRLHFTCLTGNNKELAADWQAVVELYAELEEATSFLGKPTLVFDPGVFNGKF  785
ADJ10929.1   706   KLGTFSCEVDYGELCGHAMWFKATTFESPRLHFTCLTGNNKELAADWQAVVELYAELEEATSFLGKPTLVFDPGVFDGKF  785
ABI63943.1   706   KLGTFSCEVDYGELCGHAMWFKSTTFESPRLHFTCLTGNNKELAADWQAVVELYAELEEASSFLGKPTLVSDLGVFNGKF  785
AGN29365.1   706   KSGTFSCEIDYGELCGHAMWFKSTTFESPRLHFTCLTGNNRELAADWQAVVELYAELEEAVSFLGEPTLVFDPGAFNGKF  785
ABI63930.1   705   KLGTFSCEIDYGELCGHAMWFKSTTFESPRLHFTCLTGNNKELAADWQAVVELYAELEEATSFLGKPTLVFDPGVFDGKF  784
ABI63942.1   705   KLGTFSCEIDYGELCGHAMWFKSTTFESPRLHFTCLTGNNKELAADWLAVVELYAELEEATSFLGKPTLVFDPGVFDGKF  784
ADJ10939.1   706   KLGTFSCEVDYGELCGHAMWFKATTFESPRLHFTCLTGNNKELAADWQAVVELYAELEEATSFLGKPTLVFDPGAFDGKF  785
ABI63928.1   705   KLGTFSCEIDYGELCGHAMWFKSTTFESPRLHFTCLTGNNKELAADWQAVVELYAELEEATSFLGKPTLVFDPGVFDGKF  784
CDN67476.1   703   KLGTFSCEIDYGELCGHAMWFKSTTFESPRLHFTCLTGNNKELAADWQAVVELYAELEEATSFLGKPTLVFDPGVFNGKF  782
ABI63933.1   705   KLGTFSCEIDYGELCGHAMWFKSTTFESPRLHFTCLTGNNKELAADWQAVVELYAELEEATSFLGKPTLVFDPGVFDGKF  784
AAX18707.1   706   KLGTFTCEIDYGELCGHAMWFKSTTFESPRLHFTCLTGNNKELAADWQAVVELYAELEEATAFLGKSTLVFDPGVFNGKF  785
ACZ58631.1   704   KLGTFSCEIDYGKLCGHAMWFKSTTFESPRLHFTCLTGNNKELAADWQAVVELYAELEEATSFLGKPTLVFDPGVFNGKF  783
AGN29359.1   706   KLGTFSCEIDYGELCGHAMWFKSTTFESPRLHFTCLTGNNKELAADWQAVVELYAELEEATSFLGKPTLVFDPGVFTGQF  785
AGN29361.1   706   KLGTFSCEIDYGELCGHAMWFKSTTLESPRLHLTCLTGNNKEFAADWQTVVESYAELEEATSFLGKPTLVFDPGVFTGQF  785
ABI63935.1   705   KLGTFSCEIDYGELCGHAMWFKSTTFESPRLHFTCLTGNDKELAADWQAVVELYAELEEATSFLGKPTLVFDPGVFDGKF  784
AGN29360.1   707   KLGTFSCEIDYGELCGHAMWFKSTTFESPRLHFTCLTGNNKELAADWQVVVELYAELEEATSFLGKPTLVFDPGVFDGKF  786
AWO67712.1   705   KLGTFSCEIDYGELCGHAMWFRSTTFESPRLHFTCLTGNNKELAADWQAVVELYAELEEATSFLGKPTLVFDPGVFNGKF  784
AYA22300.1   703   KLGTFSCEIDYGELCGHAMWFKSTTFESPRLHFTCLTGNNKELAADWQAVVELYAELEEATSFLGKPTLVFDPGVFNGKF  782
QBZ78373.1   703   KLGTFSCEVDYGELCGHAMWFKSTTFESPRLHFTCLTGNNKELAADWQAVVELYAELEEASSFLGKPTLVFDPGVFDGKF  782
AYQ93056.1   703   KLGTFSCEIDYGELCGHAMWFKSTTFESPRLHFTCLTGNNKELAADWQAVVELYAELEEATTFLGKPTLVFDPGVFNGKF  782
AYQ93054.1   703   KLGTFSCEIDYGELCGHAMWFKSTTFESPRLHFTCLTGNNKELAADWQAVVELYAELEEATTFLGKPTLVFDPGVFNGKF  782
AAX18709.1   703   KLGTFSCEIDYGELCGHAMWFKSTTFESPRLHFTCLTGNNKELAADWQAVVELYAELEEAASFLGKPTLVFDPGVFNGKF  782
AYQ93052.1   704   KLGTFSCEIDYGELCGHAMWFKSTTFESPRLHFTCLTGNNKELAADWQAVVELYAELEEATSFLGKPTLVFDPGVFSGKF  783
BCT87062.1   705   KLGTFSCEIDYGELCGHAMWFKSTTFESPRLHFTCLTGNNKELAADWQAVVELYAELEEATSFLGKPTLVFDPGVFDGKF  784
AGN29364.1   703   KLGTFSCEIDYGELCGHAMWFKSTTFESPRLHFTCLTGNNKELAADWQAVVELYAELEEATSFLGKPTLVFDPGVFNGKF  782
QYU59234.1   703   KLGTFTCEVDYGELCGHAMWFKSTTFESPRLHFTCLTGNNKELAADWQAVVELYAELEEATSFLGKPTLVFDPGVFSGKF  782
CDN67475.1   703   KLGTFSCEIDYGELCGHAMWFKSTTFESPRLHFTCLTGNNKELAADWQAVVELYAELEEATSFLGKPTLVFDPGVFNGQF  782
AGN29356.1   703   KLGTFSCKIDYGELCGHAMWFKSTTFESPRLHFTRLTGNNKQLAAYWQAVAELYAELEEASSFLGKPTLVFDPGVFKGKF  782
AGN29363.1   703   KLGTFSCEIDYGELCGHAMWFKSTTFESPRLHFTCLTGNNKELAADWQAVVELYAELEEATAFLGKPTLVFDPGVFNGKF  782
AFV73369.1   706   KLGTFSCEIDYGELCGHAMWFKSTTFESPRLHFTCLTGNNKELAADWQAVVELYAELEEATSFLGKPTLVFDPGVFDGKF  785
AFV34732.1   703   KLGTFTCEIDYGELCGHAMWFKSTTFESPRLHFTCLTGNNKELAADWQAVVELYAELEEATSFLGKPTLVFDPGVFDGKF  782
AFI43912.1   707   KLGTFSCEVDYGELCGHAMWFKSTTFESPRLHFTCLTGNNKELAADWQAVVELYTELEEATSFLGKPTLVFDPGVFDGKF  786
CDN67472.1   703   KLGTFSCEIDYGELCGHAMWFRSTTFESPRLHFTCLTGNNKELAADWQAVVELYAELEEATSFLGKPTLVFDPGVFDGKF  782
AGN29355.1   703   KLGTFSCEIDYGELCGHAMWFKSTTFESPRLHFTCLTGNNRELAADWQAVVSLYAELEEATTFLGKPTLVFDPGVFNGKF  782
AFI43911.1   707   KLGTFSCEVDYGELCGHAMWFKSTTFESPRLHFTCLTGNNKELAADWQAVVELYTELEEATSFLGKPTLVFDPGVFDGKF  786
ADJ10940.1   706   KLGTFSCEIDYGELCGHAMWFKSTTFESPRLHFTCLTGNNKELAADWQAVVELYAELEEATSFLGKPTLVFDPGVFNGKF  785
DAZ85718.1   706   KLGTFSCEIDYGELCGHAMWFKSTTFESPRLHFTCLTGNNKELAADWQAVVELYAELEEATSFLGQPTMIFDPGVFDGKF  785
ADJ10930.1   706   KLGTFSCEIDYGELCGHAMWFKSTTFESPRLHFTCLTGNNKELAADWQAVVELYAELEEATSFLGKPTLVFDPGVFDGKF  785
ADJ10933.1   706   KLGTFSCEIDYGELCGHAMWFKSTTFESPRLHFTCLTGNNKELAADWQAVVELYAELEEATSFLGKPTLVFDPGVFDGKF  785
ADJ10935.1   706   KLGTFSCEIDYGELCGHAMWFKSTTFESPRLHFTCLTGNNKELAADWQAVVELYAELEEATSFLGKPTLVFDPGVFDGKF  785
ADJ10941.1   706   KLGTFSCEIDYGELCGHAMWFKSTTFESPRLHFTCLTGNNKELAADWQAVVELYAELEEATSFLGKPTLVFDPGVFDGKF  785
DAZ85719.1   706   KLGTFTCEVDYGELCGHAMWFKSTTFESPRLHFTCLTGNNKELAADWQAVVELYAELEEATSFLGKPTLVFDPGVFNGKF  785
ADJ10936.1   706   KLGTFSCEIDYGELCGHAMWFKSTTFESPRLHFTCLTGNNKELAADWQAVVELYAELEEATSFLGKPTLVFDPGVFDGKF  785
ABR09917.1   706   KSGTFSCEIDYGELCGHAMWFKTTTFESPRLHFTCLTGNNKELAADWQAVVELYAELEEATSFLGKPTLVFDPGVFNGKF  785
CDN67473.1   711   KLGTFSCEIDYGELCGHAMWFKSTTFESPRLHFTCLTGNNKELAADWQAVVELYAELEEATSFLGKPTLVFDPGVFNGKF  790
QYU59232.1   703   KLGTFTCEIDYGELCGHAMWFKSTTFESPRLHFTCLTGNNKELAADWQAVVELYAELEEATSFLGKPTLVFDPGVFNGKF  782
AGN29362.1   703   KLGTFSCEIDYGELCGHAMWFKSTTFESPRLHFTCLTGNNKELAADWQADVELYAELEIATSFLRKPTLVFDPGVFTGQF  782
ACZ58625.1   705   KLGTFSCEIDYGELCGHAMWFKSTTFESPRLHFTCLTGNNKELAADWQAVVELYAELEEANSFLGKPTLVFDPGLFNGRF  784
ACZ58626.1   705   KLGTFSCEIDYGELCGHAMWFKSTTFESPRLHFTCLTGNNKELAADWQAVVELYAELEEANSFLGKPTLVFDPGLFNGRF  784
ACZ58624.1   705   KLGTFSCEIDYGELCGHAMWFKSTTFESPRLHFTCLTGNNKELAADWQAVVELYAELEEATSFLGKPTLVFDPGLFNGRF  784
AGN29366.1   706   KLGTFTCEVDYGELCGHAMWFKSTTFESPRLHFTCLTGNNKELAADWQAVVELYAELEEATTFLGKPTLVFDPGVFNGKF  785
QBZ78368.1   705   KLGTFSCEIDYGELCGHAMWFKSTTFESPRLHFTCLTGNNKELAADWQAVVELYAELEEANSFLGKPTLVFDPGFFNGRF  784
AFI43914.1   714   KLGTFSCEVDYGELCGHAMWFKSTTFESPRLHFTCLTGNNKELAADWQAVVELYAELEEAASFLGKPTLVFDPGVFDGKF  793
AFI43913.1   714   KLGTFSCEVDYGELCGHAMWFKSTTFESPRLHFTCLTGNNKELAADWQAVVELYAELEEATSFLGKPTLVFDPGVFDGKF  793
AFV34756.1   645   KLGTFSCEIDYGELCGHAMWFKSTTFESPRLHFTCLTGNNKELAADWQAVVELYAELEEATSFLGKPTLVFDPGVFNGKF  724
QYU59231.1   705   KLGTFSCEIDYGELCGHAMWFKSTTFESPRLHFTCLTGNNKELAADWQAVVELYAELEEATSFLGMPTLVFDPGVFNGKF  784
QYU59233.1   712   KLGTFSCEIDYGELCGHAMWFKSTTFESPRLHFTCLTGNNKELAADWQVVVELYAELEEATSFLGKPTLVFDPGVFNGKF  791
APD26307.1   714   KLGTFSCEIDYGELCGHAMWFRSTTFESPRLHFTCLTGNNKELAAEWQAVVELYAELEETTTFLGKPTLVFDPSCFSGKF  793
QBZ78371.1   534   KLGTFSCEIDYGELCGHAMWFKSTTFESPRLHFTCLTGNNKELAADWQAVVELYAELEEATSFLGKPTLVFDPGVFDGKF  613


NP_619706.1  785   QFLTCPPIFFDLTAVTALRSAGLTLGQVPMVGTTKVYNLNSTLVSCVLGMGGTVRGRVHICAPIFYSIVLWVVSEWNGTT  864
AWO68043.1   786   QFLTCPPIFFDLTAVTALRSAGLTLGQVPMVGTTKVYNLNSTLVSCVLGMGGTVRGRVHICAPIFYSIVLWVVSEWNGTT  865
ADJ10937.1   786   QFLTCPPIFFDLTAVTALRSAGLTLGQVPMVGTTKVYNLNSTLVSCVLGMGGTVRGRVHICAPIFYSIVLWVVSEWNGTT  865
ADJ10931.1   786   QFLTCPPIFFDLTAVTALRSAGLTLGQVPMVGTTEVYNLNSTLVSCVLGMGGTVRGRVHICAPIFYSIVLWVVSEWNGTT  865
ADJ10938.1   786   QFLTCPPIFFDLTAVTALRSAGLTLGQVPMVGTTKVYNLNSALVSCVLGMGGTIKGRVHICAPIFYSIVLWVVSEWNGTT  865
QYU59229.1   786   QFLTCPPIFFDLTAVTALRSAGLTLGQVPMVGTTKVYNLNSTLVSCVLGMGGTIRGKVHICAPIFYSIVLWVVSEWNGTT  865
ACZ58630.1   786   QFLTCPPIFFDLTAVTALKSAGLTLGQVPMVGTTKVYNLNSALVSCVLGMGGTIRGRVHICAPIFYSIVLWVVSEWNGTT  865
```

QGP72535.1	786	QFLTCPPIFFDLTAVTALKSAGLTLGQVPMVGTTKVYNLNSTLVSCVLGMGGTIRGRVHICAPIFYSIVLWVVSEWNGTT	865
Q91HK5.1	786	QFLTCPPIFFDLTAVTALKSAGLTLGQVPMVGTTKVYNLNSALVSCVLGMGGTIRGRVHICAPIFYSIVLWVVSEWNGTT	865
ARO69912.1	786	QFLTCPPIFFDLTAVTALRSAGLTLGQVPMVGTTKVYNLNSTLVSCVLGMGGTIRGRVHICAPIFYSIVLWVVSEWNGTT	865
AFI71814.1	786	QFLTCPPIFFDLTAVTALRSAGLTLGQVPMVGTTKVYNLNSTLVSCVLGMGGTIRGRVHICAPIFYSIVLWVVSEWNGTT	865
ADJ10932.1	786	QFLTCPPIFFDLTAVTALRSAGLTLGQVPMVGTTKVYNLNSTLVSCVLGMGGTIRGRVHICAPIFYSIILWVVSEWNGTT	865
ARO69925.1	786	QFLTCPPIFFDLTAVTALRSAGLTLGQVPMVGTTKVYNLNSTLVSCVLGMGGTIRGRVYICAPIFYSIVLWVVSEWNGTT	865
ARO69911.1	786	QFLTCPPIFFDLTAVTALRSAGLTLGQVPMVGTTKVYNLNSTLVSCVLGMGGTIRGRVHICAPIFYSIVLWVVSEWNGTT	865
ACZ58628.1	786	QFFTCPPIFFDLTAVTALKSAGLTLGQVPMVGTTKVYNLNSTLVSCVLGMGGTIRGKVHICAPIFYSIVLWVVSEWNGTT	865
ABI63932.1	786	QFLTCPPIFFDLTAVTALKSAGLTLGQVPMVGTTKVYNLNSTLVSCVLGMGGTIRGRVHICAPIFYSIVLWVVSEWNGTT	865
ARO69945.1	786	QFLTCPPIFFDLTAVTALRSAGLTLGQVPMVGTTKVYNLNSTLVSCVLGMGGTIRGRVHICAPIFYSIVLWVVSEWNGTT	865
ARO69930.1	786	RFLTCPPIFFDLTAVTALRSAGLTLGQVPMVGTTKVYNLNSTLVSCVLGMGGTIRGRVHICAPIFYSIVLWVVSEWNGTT	865
ABI63925.1	786	QFLTCPPIFFDLTAVTALKSAGLTLGQVPMVGTTKVYNLNSTLVSCVLGMGGTIRGRVHICAPVFYSIVLWVVSEWNGTT	865
ARO69906.1	786	QFLTCPPIFFDLTAVTALRSAGLTLGQVPMVGTTKVYNLNSTLVSCVLGMGGTIRGRVYICAPIFYSIVLWVVSEWNGTT	865
ACZ58627.1	786	QFFTCPPMFFDLTAVTALKSAGLTLGQVPMVGTTKVYNLNSTLVSCVLGMGGTIRGKVHICAPIFYSIVLWVVSEWNGTT	865
ARO69920.1	786	QFLTCPPIFFDLTAVTALRSAGLTLGQVPMVGTTKVYNLNSTLVSCVLGMGGTIRGRVHICAPIFYSIVLWVVSEWNGTT	865
ACZ58629.1	786	QFFTCPPIFFDLTAVTALKSAGLTLGQVPMVGTTKVYNLNSTLVSCVLGMGGTIRGKVHICAPIFYSIVLWVVSEWNGTT	865
AGT42201.1	786	QFLTCPPIFFDLTAVTALRSAGLTLGQVPMVGTTKVYNLNSALVSCVLGMGGTIRGRVHICAPIFYSIVLWVVSEWNGTT	865
ARO69914.1	786	QFLTCPPIFFDLTAVTALRSAGLTLGQVPMVGTTKVYNLNSTLVSCVLGMGGTIRGRVHICAPIFYSIVLWVVSEWNGTT	865
ABI63916.1	786	QFLTCPPIFFDLTAVTALKSAGLTLGQVPMVGTTKIYNLNSTLVSCVLGMGGTIRGRVHICAPIFYSIVLWVVSEWNGTT	865
ARO69918.1	786	QFLTCPPIFFDLTAVTALRSAGLTLGQVPMVGTTKVYNLNSTLVSCVVGIGGTIRGRVHICAPIFYSIVLWVVSEWNGTT	865
ARO69948.1	786	QFLTCPPIFFDLTAVTALKSAGLTLGQVPMVGTTKVYNLNSTLVSCVLGMGGTIRGRVHICAPIFYSIVLWVVSEWNGTT	865
QYA72353.1	786	QFLTCPPIFFDLTAVTALKSAGLTLGQVPMVGTTKVYNLNSALASCVLGMGGTIRGRVHICAPIFYSIVLWVVSEWNGTT	865
ABI63917.1	786	QFLTCPPIFFDLTAVTALKSAGLTLGQVPMVGTTKVYNLNSTLVSCVLGMGGTIRGRVHICAPVFYSIVLWVVSEWNGTT	865
ARO69941.1	786	QFLTCPPIFFDLTAVTALKSAGLTLGQVPMVGTTKVYNLNSTLVSCVLGMGGTIRGRVHICAPIFYSIVLWVVSEWNGTT	865
ABI63926.1	786	QFLTCPPIFFDLTAVTALKSAGLTLGQVPMVGTTKVYNLNSTLVSCVLGMGGTIRGRVHICAPIFYSIVLWVVSEWNGTT	865
CDN67471.1	786	QFLTCPPIFFDLTAVTALRSAGLTLGQVPMVGTTKVYNLNSALVSCVLGMGGTIKGRVHICAPIFYSIVLWVVSVWNGTT	865
ABI63920.1	786	QFLTCPPIFFDLTAVTALKSAGLTLGQVPMVGTTKVYNLNSTLVSCVLGMGGTIRGRVHICAPVFYSIVLWVVSEWNGTT	865
ABI63927.1	786	QFLTCPPIFFDLTAVTALKSAGLTLGQVPMVGTTKVYNLNSTLVSCVLGMGGTIRGRVHICAPIFYSIVLWVVSEWNGTT	865
ARO69908.1	786	QFLTCPPIFFDLTAVTALKSAGLTLGQVPMVGTTKVYNLNSTLVSCVLGMGGTIRGRVHICAPIFYSIVLWVVSEWNGTT	865
ARO69916.1	786	QFLTCPPIFFDLTAVTALKSAGLTLGQVPMVGTTKVYNLNSTLVSCVLGMGGTIRGRVHICAPIFYSIVLWVVSEWNGTT	865
ARO69910.1	786	QFLTCPPIFFDLTAVTALKSAGLTLGQVPMVGTTKVYNLNSTLVSCVLGMGGTIRGRVHICAPIFYSIVLWVVSEWNGTT	865
DAZ85720.1	786	QFLTCPPIFFDLTAVTALRSAGLTLGQVPMVGTTRVYNLNSTLVSCVLGMGGTIRGKVHICAPIFYSIVLWVVSEWNGTT	865
AAX18708.1	786	QFLTCPPIFFDLTAVTALRSAGLTLGQVPMVGTTKVYNLNSTLVSCVLGMGGTIRGRVHVCAPIFYSIVLWVVSEWNGTT	865
AYA22298.1	786	QFLTCPPIFFDLTAVTALRSAGLTLGQVPMVGTTKVYNLNSTLVSCVLGMGGTIRGRVHVCAPIFYSIVLWVVSEWNGTT	865
ABI63939.1	786	QFLTCPPIFFDLTTVTALKSAGLTLGQVPMVGTTKVYNLNSTLVSCVLGMGGIIRGRVHICAPIFYSIVLWVVSEWNGTT	865
ARO69926.1	786	QFLTCPPIFFDLTAVTALKSAGLTLGQVPMVGTTKVYNLNSTLVSCVLGMGGTIRGRVHICAPIFYSIVLWVVSEWNGTT	865
QGP72537.1	786	QFLTCPPIFFDLTAVTALRSAGLTLGQVPMVGTTKVYNLNSTLVSCVLGMGGTIRGKVHICAPIFYSIVLWVVSEWNGTT	865
ARO69947.1	786	QFLTCPPIFFDLTAVTALKSAGLTLGQVPMVGTTKVYNLNSTLVSCVLGMGGTIRGRVHICAPIFYSIVLWVVSEWNGTT	865
ARO69921.1	786	QFLTCPPIFFDLTAVTALKSAGLTLGQVPMVGTTKVYNLNSTLVSCVLGMGGTIRGRVHICAPIFYSIVLWVVSEWNGTT	865
ARO69946.1	786	QFLTCPPIFFDLTAVTALKSAGLTLGQVPMVGTTKVYNLNSTLASCVLGMGGTIRGRVHICAPIFYSIVLWVVSEWNGTT	865
QGP72536.1	786	QFLTCPPIFFDLTAVTALKSAGLTLGQVPMVGTTKVYNLNSALVSCVLGMGGTIKGRVHICAPIFYSIVLWVVSEWNGTT	865
QGP72538.1	786	QFLTCPPIFFDLTAVTALRSAGLTLGQVPMVGTTKVYNLNSTLVSCVLGMGGTIRGKVHICAPIFYSIVLWVVSEWNGTT	865
ARO69913.1	786	QFLTCPPIFFDLTAVTALKSAGLTLGQVPMVGTTKVYNLNSTLVSCVLGMGGTIRGKVHICAPIFYSIVLWVVSEWNGTT	865
ARO69924.1	786	QFLTCPPIFFDLTAVTALKSAGLTLGQVPMVGTTKVYNLNSTLVSCVLGMGGTIRGKVHICAPIFYSIVLWVVSEWNGTT	865
ABI63924.1	786	QFLSCPPIFFDLTAVTALKSAGLTLGQVPMVGTTKVYNLNSTLVSCVLGMGGTIRGRVHICAPVFYSIVLWVVSEWNGAT	865
ARO69931.1	786	QFLTCPPIFFDLTAVTALKSAGLTLGQVPMVGTTKVYNLNSTLVSCVLGMGGTIRGRVHICAPIFYSIVLWVVSEWNGTT	865
AWO67711.1	785	QFLTCPPIFFDLTAVTALRSAGLTLGQVPMVGTTKVYNLNSTLVSCVLGMGGTIRGKVHICAPIFYSIVLWVVSEWNGTT	864
ARO69943.1	786	QFLTCPPIFFDLTAVTALKSAGLTLGQVPMVGTTKVYNLNSTLVSCVLGMGGTIRGRVHICAPIFYSIVLWVVSEWNGTT	865
ADJ10928.1	786	QFLTCPPIFFDLTAVTALRSAGLTLGQVPMVGTTKVYNLNSTLVSCVLGMGGTVRGRVHICAPIFYSIVLWVVSEWNGTT	865
ARO69939.1	786	QFLTCPPIFFDLTAVTALKSAGLTLGQVPMVGTTKVYNLNSTLVSCVLGMGGTIRGRVHICAPIFYSIVLWVVSEWNGTT	865
ABI63938.1	786	QFLTCPPIFFDLTTVTALKSAGLTLGQVPMVGTTKVYNLNSTLVSCVLGMGGIIRGRVHICAPIFYSIVLWVVSEWNGTT	865
ABI63940.1	786	QFLTCPPIFFDLTTVTALKSAGLTLGQVPMVGTTKVYNLNSTLVSCVLGMGGIIRGRVHICAPIFYSIVLWVVSGWNGTT	865
ABI63929.1	786	QFLTCPPIFFDLTAVTALKSAGLTLGQVPMVGTTKVYNLNSTLVSCVLGMGGTIRGRVHICAPIFYSIVLWVVSEWNGTT	865
ABI63919.1	786	QFLTCPPIFFDLTAVTALKSAGLTLGQVPMVGTTKVYNLNSTLVSCVLGMGGTIRGRVHICAPVFYSIVLWVVSEWNGTT	865
ADJ10934.1	784	QFLTCPPIFFDLTAVTALKSAGLTLGQVPMVGTTKVYNLNSALVSCVLGMGGTIRGKVHICAPIFYSIVLWVVSEWNGTT	863
ARO69937.1	786	QFLTCPPIFFDLTAVTALKSAGLTLGQVPMVGTTKVYNLNSTLVSCVLGMGGTIRGRVHICAPIFYSIVLWVVSEWNGTT	865
ARO69928.1	786	QFLTCPPIFFDLTAVTALKSAGLTLGQVPMVGTTKVYNLNSTLVSCVLGMGGTIRGRVHICAPIFYSIVLWVVSEWNGTT	865
ARO69927.1	786	QFLTCPPIFFDLTAVTALKSAGLTLGQVPMVGTTKVYNLNSTLVSCVLGMGGTIRGRVHICAPIFYSIVLWVVSEWNGTT	865
ABI63934.1	785	QFLTCPPIFFDLTAVTALKSAGLTLGQVPMVGTTKVYNLNSTLVSCVLGMGGTIRGRVHICAPIFYSIVLWVVSEWNGTT	864
ARO69935.1	786	QFLTCPPIFFDLTAVTALKSAGLTLGQVPVVGTTKVYNLNSTLVSCVLGMGGTIRGRVHICAPIFYSIVLWVVSEWNGTT	865
ARO69940.1	786	QFLTCPPIFFDLTAVTALKSAGLTLGQVPMVGTTKVYNLNSTLVSCVLGMGGTIRGRVHICAPIFYSIVLWVVSEWNGTT	865
ARO69933.1	786	QFLTCPPIFFDLTAVTALKSAGLTLGQVPMVGTTKVYNLNSTLVSCVLGMGGTIRGRVHICAPIFYSIVLWVVSEWNGTT	865
ABI63922.1	786	QFLTCPPIFFDLTAVTALKSAGLTLGQVPMVGTTKVYNLNSTLVSCILGMGGTIRGRVHICAPVFYSIVLWVVSEWNGTT	865
ABI63936.1	786	QFLTCPPIFFDLTAVTALKSAGLTLGQVPMVGTTKVYNLNSTLVSCVLGMGGTIRGRVHICAPIFYSIVLWVVSEWNGTT	865
ABI63918.1	786	QFLTCPPLFFDLTAVTALKSAGLTLGQVPMVGTTKVYNLNSTLVSCVLGMGGTIRGRVHICAPVFYSIVLWVVSEWNGTT	865
ABI63931.1	785	QYLTCPPIFFDLTAVTALKSAGLTLGQVPMVGTTRVYNLNSTLVSCVLGMGGTIKGKVHICAPIFYSIVLWVVSEWNGTT	864
QYU59230.1	786	QFLTCPPIFFDLTAVTALKSAGLTLGQVPMVGTTKVYNLNSALVSCVLGMGGTIRGKVHICAPIFYSIVLWVVSEWNGTT	865
ARO69922.1	786	QFLTCPPIFFDLTAVTALKSAGLTLGQVPMVGTTKVYNLNSTLVSCVLGMGGTIRGRVHICAPIFYSIVLWVVSEWNGTT	865
ABI63941.1	786	QFLTCPPIFFDLTTVTALKSAGLTLGQVPMVGTTKVYNLNSTLVSCVLGMGGIIRGRVHICAPIFYSIVLWVVSEWNGTT	865
ARO69949.1	786	QFLTCPPIFFDLTAVTALKSAGLTLGQVPMVGTTKVYNLNSTLVSCVLGMGGTIRGRVHICAPIFYSIVLWVVSEWNGTT	865
ARO69917.1	786	QFLTCPPIFFDLTAVTALKSAGLTLGQVPMVGTTKVYNLNSTLVSCVLGMGGTIRGRVHICAPIFYSIVLWVVSEWNGTT	865
ARO69915.1	786	QFLTCPPIFFDLTAVTALKSAGLTLGQVPMVGTTKVYNLNSTLVSCVLGMGGTIRGRVHICAPIFYSIVLWVVSEWNGTT	865
QYU59228.1	786	QFLTCPPIFFDLTAVTALRSAGLTLGQVPMVGTTRVYNLNSTLVSCVLGMGGTIRGKVHICAPIFYSVVLWVVSEWNGTT	865
AGN29358.1	786	QFLTCPPIFFDLTAVTALRSAGLTLGQVPMVGTTKVYNLNSTLVSCVLGMGGTIRGKVHICAPIFYSIVLWVVSEWNGTT	865
ARO69944.1	786	QFLTCPPIFFDLTAVTALKSAGLTLGQVPMVGTTKVYNLNSTLVSCVLGMGGTIRGRVHICAPIFYSIVLWVVSEWNGTT	865
AGN29367.1	783	QFLTCPPIFFDLTAVTALKSAGLTLGQVPMVGTTRVYNLNSTLVSCVWGMGGTIRGKVHICAPIFNSIVLWVVSEWNGTT	862
ABI63921.1	786	QFLTCPPIFFDLTAVTALKSAGLTLGQVPMVGTTKVYNLNSTLVSCVLGMGGTIRGRVHICAPVFYSIVLWVVSERNGTT	865
ABI63923.1	786	QFLTCPPIFFDLTAVTALKSAGLTLGQVPMVGTTKVYNLNSTLVSCVLGMGGTIRGRVHICAPVFYSIVLWVVSEWNGTT	865
CDN67470.1	783	QFLTCPPIFFDLTAVTALKSAGLTLGQVPMVGTTRVYNLNSALVSCVLGMGGTIKGRVHICAPIFYSIVLWVVSEWDGTT	862
CDN67474.1	786	QFLTCPPIFFDLTAVTALKSAGLTLGQVPMVGTTKVYNLNSTLVSCVLGIGGTIRGKVHICAPIFYSIVLWVVSEWDGTT	865
AAX18710.1	786	QFLTCPPIFFDLTAVTALRSAGLTLGQVPMVGTTKVYNLNSTLVSCVLGMGGTIRGKVHICAPIFYSIVLWVVSEWNGTT	865
QBZ78370.1	784	QFLTCPPIFFDLTATTALRSAGLTLGQVPMVGTTKVYNLNSTLVSCVLGMGGTIRGKVHICAPIFYSIVLWVVSEWNGTT	863
ARO69923.1	786	QFLTCPPIFFDLTAVTALKSAGLTLGQVPMVGTTKVYNLNSTLVSCVLGMGGTIRGRVHICAPIFYSIVLWVVSEWNGTT	865
ARO69907.1	786	QFLTCPPIFFDLTAVTALKSAGLTLGQVPMVGTTKVYNLNSTLVSCVLGMGGTIRGRVHICAPIFYSIVLWVVSEWNGTT	865
ARO69942.1	786	QFLTCPPIFFDLTAVTALKSAGLTLGQVPMVGTTKVYNLNSTLVSCVLGMGGTIRGRVHICAPIFYSIVLWVVSEWNGTT	865
ARO69909.1	786	QFLTCPPIFFDLTAVTALKSAGLTLGQVPMVGTTKVYNLNSTLVSCVLGMGGTIRGRVHICAPIFYSIVLWVVSEWNGTT	865
AAX18711.1	786	QFLTCPPIFFDLTAVTALRSAGLTLGQVPMVGTTKAYNLNSTLVSCVLGMGGTIRGRVHICAPIFYSIVLWVVSEWNGTT	865
ARO69936.1	786	QFLTCPPIFFDLTAVTALKSAGLTLGQVPVVGTTKVYNLNSTLVSCVLGMGGTIRGRVHICAPIFYSIVLWVVSEWNGTT	865

```
AGN29357.1   786   QFLTCPPIFFDLTAVTALRSAGLTLGQVPMVGTTKVYNLNSTLVSCVLGMGGTIKGKVHICAPIFYSIVLWVVSEWNGTP  865
ARO69919.1   786   QFLTCPPIFFDLTAVTALKSAGLTLGQVPMVGTTKVYNLNSTLVSCVLGMGGTIRGRVHICAPIFYSIVLWVVSEWNGTT  865
ADJ10929.1   786   QFLTCPPIFFDLTAVTALRSAGLTLGQVPVVGTTKVYNLNSTLVSCVLGMGGTIRGKVHICAPIFYSIVLWVVSEWNGTT  865
ABI63943.1   786   QFLTCPPIFFDLTTVTALKSAGLTLGQVPMVGTTKVYNLNSTLVSCVLGMGGIIRRRVHICAPIFYSIVLWVVSEWNGTT  865
AGN29365.1   786   QFLTCPPIFFDLTAVTALKSAGLTLGQVPMVGTTKVYNLNSALVSCVLGMGGTIRGRVHICAPIFYSMVLWVVSEWNGTT  865
ABI63930.1   785   QYLTCPPIFFDLTAVTALKSAGLTLGQVPMVGTTRVYSLNNTLVSCVLGMGGTIKGKVHICAPIFYSIVLWVVSEWNGTT  864
ABI63942.1   785   QYLTCPPIFFDLTAVTALKSAGLTLGQVPMVGTIRVYNLNNTLVSCVLGMGGTIKGKVHICAPIFYSIVLWVVSEWNGTT  864
ADJ10939.1   786   QFLTCPPIFFDLTAVTALRSAGLTLGQVPVVGTTKVYNLNSTLVSCVLGMGGTIRGKVHICAPIFYSIVLWVVSEWNGTT  865
ABI63928.1   785   QYLTCPPIFFDLTAVTALKSAGLTLGQVPMVGTTRVYNLNNTLVSCVLGMGGTIKGKVHICAPIFYSIVLWVVSEWNGTT  864
CDN67476.1   783   QFLTCPPIFFDLTAVTALRSAGLTLGQVPMVGTTRVYNLNSTLVSCVLGMGGTIKGRVHICAPIFYSIVLWVVSVWNGTT  862
ABI63933.1   785   QYLTCPPIFFDLTAVTALKSAGLTLGQVPMVGTTRVYNLNSTLVSCVLGMGGTNKGKVHICAPIFYSIVLWVVSEWNGTT  864
AAX18707.1   786   QFLTCPPIFFDLTAVTALKSAGLTLGQVPMVGTTKVYNLNSTLVSCVLGMGGTIRGKVHICVPIFYSIVLWVVSVWNGTT  865
ACZ58631.1   784   QFLTCPPIFFDLTAVTALKSAGLTLGQVPMVGTTKVYNLNSALVSCVLGMGGTIRGKVHICAPIFYSIVLWVVSEWNGTT  863
AGN29359.1   786   QFLTCPPIFFDLTAVTALRSAGLTLGQVPMVGTTKVYNLNSTLVSCVLGMGGTIRGKVHICAPIFYSIVLWVVSEWTGTP  865
AGN29361.1   786   QFLTCPPIFFDLTAVTALRSAGLTLGQVPMVGTTKVYNLNSTLVSCVLGMGGTIRGKVHICAPIFYSMFVRVVSEWNGTT  865
ABI63935.1   785   QYLTCPPIFFDLTAVTALKSAGLTLGQVPMVGTTRVYNLNNTLVSCVLGMGGTIKGKVHICAPIFYSIVLWVVSEWNGTT  864
AGN29360.1   787   QFLTCPPIFFDLTAVTALKSAGLTLGQVPMVGTTKVYNLNSALVSCVLGMGGTIKGRVHICAPIFYSIVLWVVSEWNGTT  866
AWO67712.1   785   QFLTCPPIFFDLTAVTALKSAGLTLGQVPMVGTTKVYNLNSALVSCVLGMGGTIRGRVHICAPIFYSIVLWVVSEWNGTT  864
AYA22300.1   783   QFLTCPPMFFDLTAVTALKSAGLTLGQVPMVGTTKVYNLNSTLVSCVLGMGGTIRGKVHICAPIFYSIVLWVVSEWNGTT  862
QBZ78373.1   783   RFLTCPPIFFDLTAVTALKSAGLTLGQVPMVGTTKVYNLNSTLVSCVLGMGGTIRGKVHICAPIFYSIVLWVVSEWNGTT  862
AYQ93056.1   783   QFLTCPPIYFDLTAVTALRSAGLTLGQVPMVGTTKVYNLNSALVSCVLGMGGTIRGRVHICAPIFYSIVLWVVSEWNGTT  862
AYQ93054.1   783   QFLTCPPIFFDLTAVTALRSAGLTLGQVPMVGTTKVYNLNSALVSCVLGMGGTIRGRVHICAPIFYSIVLWVVSEWNGTT  862
AAX18709.1   783   QFLTCPPMFFDLTAVTALKSAGLTLGQVPMVGTTKVYNLNSTLVSCVLGMGGTIRGKVHICAPIFYSIVLWVVSEWNGTT  862
AYQ93052.1   784   QFLTCPPIFFDLTAVTALKSAGLTLGQVPMVGTTRVYNLNSTLVSCVLGMGGTIRGRVHICAPIFYSIVLWVVSEWNGTT  863
BCT87062.1   785   KFLTCPPIFFDLTAVTALKSAGLTLGQVPMVGTTKVYNLNSTLVSCVLGMGGTIKGKVHICAPIFYSVVLWVVSEWNGTT  864
AGN29364.1   783   QFLTCPPIFFDLTAVTALKSAGLTLGQVPMVGTTKVYNLNSTLVSCVLGMGGTIRGRVHICAPIFYSIVLWVVSEWNGTT  862
QYU59234.1   783   QFLTCPPIFFDLTAVTALRSAGLTLGQVPMVGTTKVYNLNSALVSCVLGMGGTIKGKVHICAPIFYSVVLWVVSEWNGTT  862
CDN67475.1   783   QFLTCPPIFFDLTAVTALKSAGLTLGQVPMVGTTKVYNLNSALVSCVLGMGGTIRGRVHICAPIFYSIVLWVVSVWNGTT  862
AGN29356.1   783   QFLTCPPIFFDLTAVTALKSAGLTLGQVPMVGTTKVYNLNSTLVSCVLGMGGTIRGRVHICAPIFYSIVLWVVSVWNGTT  862
AGN29363.1   783   QFLTCPPIFFDLTAVTALKSAGLTLGQVPMVGTTKVYNLNSTLVSCVLGMGGTIRGRVHICAPIFYSIVLWVVSEWNGTT  862
AFV73369.1   786   QFLTCPPIFFDLTAVTALKSAGLTLGQVPMVGTTKVYNLNSTLVSCVLGMGGTIRGKVHICAPIFYSIVLWVVSEWNGTT  865
AFV34732.1   783   QFLTCPPIFFDLTAVTALKSAGLTLGQVPMVGTTKVYNLNSTLVSCVLGMGGTIRGRVHICAPIFYSIVLWVVSEWNGTT  862
AFI43912.1   787   KFLTCPPIFFDLTAVTALKSAGLTLGQVPMVGTTKVYNLNSTLVSCVLGMGGTIKGKVHICAPIFYSIVLWVVSEWNGTT  866
CDN67472.1   783   KFLTCPPIFFDLTAVTALKSAGLTLGQVPMVGTTKVYNLNSTLVSCVLGMGGTIKGRVHICAPIFYSIVLWVVSEWDGTT  862
AGN29355.1   783   QFLTCPPIFFDLTAVTALKSAGLTLGQVPMVGTTRVYNLNSTLVSCVLGMGGTIRGKVHICAPIFYSIVLWVFSEWNGTT  862
AFI43911.1   787   KFLTCPPIFFDLTAVTALKSAGLTLGQVPMVGTTKVYNLNSTLVSCVLGMGGTIKGKVHICAPIFYSIVLWVVSEWNGTT  866
ADJ10940.1   786   QFLTCPPIFFDLTAVTALRSAGLTLGQVPMVGTTKVYNLNSTLVSCVLGMGGTVRGRVHICAPIFYSIVLWVVSEWNGTT  865
DAZ85718.1   786   QFLTCPPIFFDLTAVTALKSAGLTLGQVPMVGTTKVYNLNSTLASCVLGMGGTIRGKVHICAPIFYSIVLWVVSEWNGTT  865
ADJ10930.1   786   QFLTCPPIFFDLTAVTALRSAGLTLGQVPMVGTTKVYNLNSTLVSCVLGMGGTIRGRVHICAPIFYSIILWVVSEWNGTT  865
ADJ10933.1   786   QFLTCPPIFFDLTAVTALRSAGLTLGQVPMVGTTKVYNLNSTLVSCVLGMGGTIRGRVHICAPIFYSIILWVVSEWNGTT  865
ADJ10935.1   786   KLLTCPPISFDLTAVTALRSAGLTLGQVPMVGTTKVYNLNSTLVSCVLGMGGTVRGRVHICAPIFYSIVLWVVSEWNGTT  865
ADJ10941.1   786   QFLTCPPIFFDLTAVTALRSAGLTLGQVPMVGTTKVYNLNSTLVSCVLGMGGTIRGRVHICAPIFYSIILWVVSEWNGTT  865
DAZ85719.1   786   QFLTCPPIFFDLTAVTALRSAGLTLGQVPMVGTTKVYNLNSTLVSCVLGMGGTIKGKVHICAPIFYSIVLWVVSEWNGTT  865
ADJ10936.1   786   QFLTCPPIFFDLTAVTALRSAGLTLGQVPMVGTTKVYNLNSTLVSCVLGMGGTIRGRVHICAPIFYSIILWVVSEWNGTT  865
ABR09917.1   786   QFLTCPPIFFDLTAVTALRSAGLTLGQVPMVGTTKVYNLNSALVSCVLGMGGTIKGKVHICAPIFYSIVLWVVSEWNGTT  865
CDN67473.1   791   QFLTCPPIFFDLTAVTALKSAGLTLGQVPMVGTTRVYNLNSTLVSCVLGMGGTIKGKVHICAPIFYSIVLWVVSEWDGTT  870
QYU59232.1   783   QFLTCPPIFFDLTAVTALRSAGLTLGQVPMVGTTKVYNLNSALVSCVLGMGGTIRGRVHICAPIFYSIVLWVVSEWNGTT  862
AGN29362.1   783   QFLTCPPIFFDLTAVTALRSAGLTLGHVPMVGTTKVYNLNSTLVSCVLGMGGTIRGKVHICAPIFYSIVVRVVSERNGTT  862
ACZ58625.1   785   QFLTCPPIFFDLTAVTAFKSAGLTLGQVPMVGTTKVYNLNSALVSCVLGLGGTIRGRVHICAPIFYSIVLWVVSEWNGTT  864
ACZ58626.1   785   QFLTCPPIFFDLTAVTAFKSAGLTLGQVPMVGTTKVYNLNSALVSCVLGLGGTIRGKVHICAPIFYSIVLWVVSEWNGTT  864
ACZ58624.1   785   QFLTYPPIFFDLTAVTALKSAGLTLGQVPMVGTTRVYNLNSALVSCVLGLGGTIRGRVHICAPIFYSIVLWVVSEWNGTT  864
AGN29366.1   786   QFLTCPPIFFDLTAVTALKSAGLTLGQVPMVGTTKVYNLNSTLVSCVLGMGGTIRGRVHICAPIFYNIILWVLSHCNGTH  865
QBZ78368.1   785   QFLTYPPIFFDLTAVTAFKSAGLTLGQVPMVGTTKVYNLNSALVSCVLGLGGTIRGRVHICAPIFYSVVLWVVSEWNGAT  864
AFI43914.1   794   KFLTCPPIFFDLTAVTALRSAGLTLGQVPMVGTTKVYNLNSTLVSCVLGMGGTIKGKVHICAPIFYSIVLWVVSEWNGTT  873
AFI43913.1   794   KFLTCPPIFFDLTAVTALRSAGLTLGQVPMVGTTKVYNLNSTLVSCVLGMGGTIKGKVHVCAPIFYSIVLWVVSEWNGTT  873
AFV34756.1   725   QFLTCPPIFFDLTAVTALKSAGLTLGQVPMVGTTKVYNLNSTLVSCVLGMGGTIRGKVHICAPIFYSIVLWVVSEWNGTT  804
QYU59231.1   785   QFLTCPPIFFDLTAVTALKSAGLTLGQVPMVGTTKVYNLNSTLVSCVLGMGGTIRGKVHICAPIFYSIVLWVVSEWNGTT  864
QYU59233.1   792   QFLTCPPIFFDLTAVTALRSAGLTLGQVPMVGTTKVYNLNSTLVSCVLGMGGIIRGKVHICAPIFYSIILWVVSEWNGTT  871
APD26307.1   794   KYLTCPPIFFDLTATTALRSAGLTLGQVPMVGTTKVYNLNSTLVSCVLGIGGTIKGKVHICAPIFYSIVLWVVSEWNGTT  873
QBZ78371.1   614   KFLTCPPIFFDLTAVTALRSAGLTLGQVPMVGTTKVYNLNSTLVSCVLGMGGTIKGKVHICAPIFYSIVLWVVSEWNGTT  693


NP_619706.1  865   MDWNELFKYPGVYVEEDGSFEVKIRSPYHRTPARLLAGQSQRDMSSLNFYAIAGPIAPSGETAQLPIVVQIDEIVRPDLS  944
AWO68043.1   866   MDWNELFKYPGVYVEEDGSFEVKIRSPYHRTPARLLAGQSQRDMSSLNFYAIAGPIAPSGETAQLPIVVQIDEIVRPDLS  945
ADJ10937.1   866   MDWNELFKYPGVYVEEDGSFEVKIRSPYHRTPARLLADQSQRDMSSLNFYAIAGPIAPSGETAQLPIVVQIDEIVRPDLS  945
ADJ10931.1   866   MDWNELFKYPGVYVEEDGSFEVKIRSPYHRTPARLLAGQSQRDMSSLNFYAIAGPIAPSGETARLPIVVQIDEIVRPDFS  945
ADJ10938.1   866   MDWNELFKYPGVYVEEDGSFEVKIRSPYHRTPARLLAGQSQRDMSSLNFYAIAGPIAPSGETARLPIVVQIDEIVRPDFS  945
QYU59229.1   866   MDWNELFKYPGVYVEEDGSFEVKIRSPYHRTPARLLAGQSQRDMSSLNFYAIAGPIAPSGETARLPIVVQIDEIARPDLS  945
ACZ58630.1   866   MDWNELFKYPGVYVEEDGSFEVKIRSPYHRTPARLLAGQSQRDMSSLNFYAIAGPIAPSGETARLPIVVQIDEIVRPDFS  945
QGP72535.1   866   MDWNELFKYPGVYVEEDGSFEVKIRSPYHRAPARLFAGQSQREMSSLNFYAIAGPIAPSGETARLPIVVQIEEIVHPDLS  945
Q91HK5.1     866   MDWNELFKYPGVYVEEDGSFEVKIRSPYHRTPARLLAGQSQRDMSSLNFYAIAGPIAPSGETARLPIVVQIDEIVRPDLA  945
ARO69912.1   866   MDWNELFKYPGVYVEEDGSFEVKIRSPYHRTPARLLAGQSQRDMSSLNFYAIAGPIAPSGETARLPIVVQIDEIVRPDLS  945
AFI71814.1   866   MDWNELFKYPGVYVEEDGFFEVKIRSPYHRTPAKLLAGQSQRDMSSLNFYAIAGPIAPSGETARLPIVVQIDEIVRPDLS  945
ADJ10932.1   866   MDWNELFKYPGVYVEEDGSFEVKIRSPYHRTPARLLAGQSQRDMSSLNFYAIAGPIAPSGETARLPIVVQIDEIVRPDLS  945
ARO69925.1   866   MDWNELFKYPGVYVEEDGSFEVKIRSPYHRTPARLLAGQSQRDMSSLNFYAIAGPIAPSGETARLPIVVQIDEIVRPDLS  945
ARO69911.1   866   MDWNELFKYPGVYVEEDGSFEVKIRSPYHRTPARLLAGQSQRDMSSLNFYAIAGPIAPSGETARLPIVVQIDEIVRPDLS  945
ACZ58628.1   866   MDWNELFKYPGVYVEEDGNFEVKIRSPYHRTPARLLAGQSQRDMSSLNFYAIAGPIAPSGETARLPIVVQIDEIVRPDLS  945
ABI63932.1   866   MDWNELFKYPGVYVEEDGSFEVKIRSPYHRTPARLLVGQSQRDMSSLNFYAIAGPIAPSGETARLPIVVQIDEIVRPDLS  945
ARO69945.1   866   MDWNELFKYPGVYVEEDGSFEVKIRSPYHRTPARLLAGQSQRDMSSLNFYAIAGPIAPSGETARLPIVVQIDEIVRPDLS  945
ARO69930.1   866   MDWNELFKYPGVYVEEDGSFEVKIRSPYHRTPARLLAGQSQRDMSSLNFYAIAGPIAPSGETARLPIVVQIDEIVRPDLS  945
ABI63925.1   866   MDWNELFKYPGVYVEEDGSFEVKIRSPYHRTPARLLVGQSQRDMSSLNFYAIAGPIAPSGETARLPIVVQIDEIVRPDLS  945
ARO69906.1   866   MDWNELFKYPGVYVEEDGSFEVKIRSPYHRTPARLLAGQSQRDMSSLNFYAIAGPIAPSGETARLPIVVQIDEIVRPDLS  945
ACZ58627.1   866   MDWNELFKYPGVYVEEDGSFEVKIRSPYHRTPARLLAGQSQRDMSSLNFYAIAGPIAPSGETARLPIVVQIDEIVRPDLS  945
ARO69920.1   866   MDWNELFKYPGVYVEEDGSFEVKIRSPYHRTPARLLAGQSQRDMSSLNFYAIAGPIAPSGETARLPIVVQIDEIVRPDLS  945
ACZ58629.1   866   MDWNELFKYPGVYVEEDGNFEVKIRSPYHRTPARLLAGQSQRDMSSLNFYAIAGPIAPSGETARLPIVVQIDEIVRPDLS  945
AGT42201.1   866   MDWNELFKYPGVYVEEDGFFEVKIRSPYHRTPAKLLAGQSQRDMSSLNFYAIAGPIAPSGETARLPIVVQIDEIVRPDLS  945
ARO69914.1   866   MDWNELFKYPGVYVEEDGSFEVKIRSPYHRTPARLLAGQSQRDMSSLNFYAIAGPIAPSGETARLPIVVQIDEIVRPDLS  945
ABI63916.1   866   MDWNELFKYPGVYVEEDGSFEVKIRSPYHRTPARLLVGQSQRDMSSLNFYAIAGPIAPSGETARLPIVVQIDEIVRPDLS  945
```

ARO69918.1	866	MDWNELFKYPGVYVEEDGSFEVKIRSPYHRTPARLLAGQSQRDMSSLNFYAIAGPIAPSGETARLPIVVQIDEIVRPDLS	945
ARO69948.1	866	MDWNELFKYPGVYVEEDGSFEVKIRSPYHRTPARLLAGQSQRDMSSLNFYAIAGPIAPSGETARLPIVVQIDEIVRPDLS	945
QYA72353.1	866	MDWNELFKYPGVYVEEDGSFEVKIRSPYHRTPARLLAGQSQRDMSSLNFYAIAGPIAPSGETARLPIVVQIDEIVRPDLA	945
ABI63917.1	866	MDWNELFKYPGVYVEEDGSFEVKIRSPYHRTPARLLVGQSQRDMSSLNFYAIAGPIAPSGETARLPIVVQIDGIVRPDLS	945
ARO69941.1	866	MDWNELFKYPGVYVEEDGNFEVKIRSPYHRTPARLLAGQSQRDMSSLNFYAIAGPIAPSGETARLPIVVQIDEIVRPDLS	945
ABI63926.1	866	MDWNELFKYPGVYVEEDGSFEVKIRSPYHRTPARLLVGQSQRDMSSLNFYAIAGPIAPSGETARLPIVVQIDEIVRPDLS	945
CDN67471.1	866	MDWNELFKYPGVYVEEDGSFEVKIRSPYHRTPARLLAGQSQRDMSSLNFYAIAGPIAPSGETARLPIVVQIDEIVRPDLS	945
ABI63920.1	866	MDWNELFKYPGVYVEEDGSFEVKIRSPYHRTPARLLVGQSQRDMSSLNFYAIAGPIAPSGETARLPIVVQIDEIVRPDLS	945
ABI63927.1	866	MDWNGLFKYPGVYVEEDGSFEVKIRSPYHRTPARLLVGQSQRDMSSLNFYAIAGPIAPSGETARLPIVVQIDEIVRPDLS	945
ARO69908.1	866	MDWNELFKYPGVYVEEDGNFEVKIRSPYHRTPARLLAGQSQRDMSSLNFYAIAGPIAPSGETARLPIVVQIDEIVRPDLS	945
ARO69916.1	866	MDWNELFKYPGVYVEEDGNFEVKIRSPYHRTPARLLAGQSQRDMSSLNFYAIAGPIAPSGETARLPIVVQIDEIVRPDLS	945
ARO69910.1	866	MDWNELFKYPGVYVEEDGSFEVKIRSPYHRTPARLLAGQSQRDMSSLNFYAIAGPIAPSGETARLPIVVQIDEIVRPDLS	945
DAZ85720.1	866	MDWNELFKYPGVYVEEDGSFEVKIRSPYHRTPARLLAGQSQRDMSSLNFYAIAGPIAPSGETARLPIVVQIDEIVRPDLS	945
AAX18708.1	866	MDWNELFKYPGVYVEEDGSFEVKIRSPYHRTPARLLAGQSQRDMSSLNFYAIAGPIAPSGETARLPIVVQIDEIVRPDLA	945
AYA22298.1	866	MDWNELFKYPGVYVEEDGSFEVKIRSPYHRTPARLLAGQSQRDMSSLNFYAIAGPIAPSGETARLPIVVQIDEIVRPDLA	945
ABI63939.1	866	MDWNELFKYPGVYVEEDGSFEVKIRSPYHRTPARLLVGQSQRDMSSLNFYAIAGPIAPSGETARLPIVVQIDEIVRPDLS	945
ARO69926.1	866	MDWNELFKYPGVYVEEDGNFEVKIRSPYHRTPARLLAGQSQRDMSSLNFYAIAGPIAPSGETARLPIVVQIDEIVRPDLS	945
QGP72537.1	866	MDWNELFKYPGVYVEEDGSFEVKIRSPYHRTPARLLAGQSQRDMSSLNFYAIAGPIAPSGETARLPIVVQIEEIVRPDLS	945
ARO69947.1	866	MDWNELFKYPGVYVEEDGNFEVKIRSPYHRTPARLLAGQSQRDMSSLNFYAIAGPIAPSGETARLPIVVQIDEIVRPDLS	945
ARO69921.1	866	MDWNELFKYPGVYVEEDGNFEVKIRSPYHRTPARLLAGQSQRDMSSLNFYAIAGPIAPSGETARLPIVVQIDEIVRPDLS	945
ARO69946.1	866	MDWNELFKYPGVYVEEDGNFEVKIRSPYHRTPARLLAGQSQRDMSSLNFYAIAGPIAPSGETARLPIVVQIDEIVRPDLS	945
QGP72536.1	866	MDWNELFKYPGVYVEEDGSFEVKIRSPYHRTPARLLSGQSQRDMSSLNFYAIAGPIAPSGETARLPIVVQIDEIVRPDLS	945
QGP72538.1	866	MDWNELFKYPGVYVEEDGSFEVKIRSPYHRTPARLLAGQSQRDMSSLNFYAIAGPIAPSGETARLPIVVQIEEIVRPDLS	945
ARO69913.1	866	MDWNELFKYPGVYVEEDGNFEVKIRSPYHRTPARLLAGQSQRDMSSLNFYAIAGPIAPSGETARLPIVVQIDEIVRPDLS	945
ARO69924.1	866	MDWNELFKYPGVYVEEDGNFEVKIRSPYHRTPARLLAGQSQRDMSSLNFYAIAGPIAPSGETARLPIVVQIDEIVRPDLS	945
ABI63924.1	866	MDWNELFKYPGVYVEEDGSFEVKIRSPYHRTPARLLVGQSQRDMSSLNFYAIAGPIAPSGETARLPIVVQIDEIVRPDLS	945
ARO69931.1	866	MDWNELFKYPGVYVEEDGNFEVKIRSPYHRTPARLLAGQSQRDMSSLNFYAIAGPIAPSGETARLPIVVQIDEIVRPDLS	945
AWO67711.1	865	MDWNELFKYPGVYVEEDGSFEVKIRSPYHRTPARLLSGQSQRDMSSLNFYAIAGPIAPSGETARLPIVVQIDEIVRPDLS	944
ARO69943.1	866	MDWNELFKYPGVYVEEDGNFEVKIRSPYHRTPARLLAGQSQRDMSSLNFYAIAGPIAPSGETARLPIVVQIDEIARPDLS	945
ADJ10928.1	866	MDWNELFKYPGVYVEEDGSFEVKIRSPYHRTPARLLAGQSQRDMSSLNFYAIAGPIAPSGETARLPIVVQIDEIVRPDLS	945
ARO69939.1	866	MDWNELFKYPGVYVEEDGNFEVKIRSPYHRTPARLLAGQSQRDMSSLNFYAIAGPIAPSGETARLPIVVQIDEIVRPDLS	945
ABI63938.1	866	MDWNELFKYPGVYVEEDGSFEVKIRSPYHRTPARLLVGQSQRDMSSLNFYAIAGPIAPSGETARLPIVVQIDEIVRPDLS	945
ABI63940.1	866	MDWNELFKYPGVYVEEDGSFEVKIRSPYHRTPARLLVGQSQRDMSSLNFYAIAGPIAPSGETARLPIVVQIDEIVRPDLS	945
ABI63929.1	866	MDWNELFKYPGVYVEEDGSFEVKIRSPYHRTPARLLVGQSQRDMSSLNFYAIAGPIAPSGETARLPIVVQIDEIVRPDLS	945
ABI63919.1	866	MDWNELFKYPGVYVEEDGSFEVKIRSPYHRTPARLLVGQSQRDMSSLNFYAIAGPIAPSGETARLPIVVQIDEIVRPDLS	945
ADJ10934.1	864	MDWNELFKYPGVYVEEDGSFEVKIRSPYHRTPARLLTGQNQRDMSSLNFYAIAGHIAPSGETARLPIVVQIEEIVRPDLS	943
ARO69937.1	866	MDWNELFKYPGVYVEEDGNFEVKIRSPYHRTPARLLAGQSQRDMSSLNFYAIAGPIAPSGETARLPIVVQIDEIVRPDLS	945
ARO69928.1	866	MDWNELFKYPGVYVEEDGNFEVKIRSPYHRTPARLLAGQSQRDMSSLNFYAIAGPIAPSGETARLPIVVQIDEIVRPDLS	945
ARO69927.1	866	MDWNELFKYPGVYVEEDGNFEVKIRSPYHRTPARLLAGQSQRDMSSLNFYAIAGPIAPSGETARLPIVVQIDEIVRPDLS	945
ABI63934.1	865	MDWNELFKYPGVYVEEDGSFDVKIRSPYHRTPARLLVGQSQRDMSSLNFYAIAGPIAPSGETARLPIVVQIDEIVRPDLS	944
ARO69935.1	866	MDWNELFKYPGVYVEEDGNFEVKIRSPYHRTPARLLAGQSQRDMSSLNFYAIAGPIAPSGETARLPIVVQIDEIVRPDLS	945
ARO69940.1	866	MDWNELFKYPGVYVEEDGSFEVKIRSPYHRTPARLLAGQSQRDMSSLNFYAIAGPIAPSGETARLPIVVQIDEIVRPDLS	945
ARO69933.1	866	MDWNELFKYPGVYVEEDGSFEVKIRSPYHRTPARLLAGQSQRDMSSLNFYAIAGPIAPSGETARLPIVVQIDEIVRPDLS	945
ABI63922.1	866	MDWNELFKYPGVYVEEDGSFEVKIRSPYHRTPARLLVGQSQRDMSSLNFYAIAGPIAPSGETARLPIVVQIDEIVRPDLS	945
ABI63936.1	866	MDWNELFKYPGGYVEEDGSFEVKIRSPYHRTPARLLVGQSQRDMSSLNFYVIAGPIAPSGETARLPIVVQIDEIVRPDLS	945
ABI63918.1	866	MDWNELFKYPGVYVEEDGSFEVKIRSPYHRTPARLLVGQSQRDMSSLNFYAIAGPIAPSGETARLPIVVQIDEIVRPDLS	945
ABI63931.1	865	MDWNELFKYPGVYVEEDGSFEVKIRSPYHRTPARLLAGQSQRDMSSLNFYAIAGPIAPAGETARLPVVVQIEEIVRPDLS	944
QYU59230.1	866	MDWNELFKYPGVYVEEDGSFEVKIRSPYHRTPARLLAGQSQRDMSSLNFYAIAGPIAPSGETARLPIVVQIDEIVRPDLS	945
ARO69922.1	866	MDWNELFKYPGVYVEEDGSFEVKIRSPYHRTPARLLAGQSQRDMSSLNFYAIAGPIAPSGETARLPIVVQIDEIVRPDLS	945
ABI63941.1	866	MDWNELFKYPGVYVEEDGSFEVKIRSPYHRTPARLLVGQSQRDMSSLNFYAIAGPIAPSGETARLPIVVQIDEIVRPDLS	945
ARO69949.1	866	MDWNELFKYPGVYVEEDGNFEVKIRSPYHRTPARLLAGQSQRDMSSLNFYAIAGPIAPSGETARLPIVVQIDEIVRPDLS	945
ARO69917.1	866	MDWNELFKYPGVYVEEDGNFEVKIRSPYHRTPARLLAGQSQRDMSSLNFYAIAGPIAPSGETARLPIVVQIDEIVRPDLS	945
ARO69915.1	866	MDWNELFKYPGVYVEEDGSFEVKIRSPYHRTPARLLAGQSQRDMSSLNFYAIAGPIAPSGETARLPIVVQIDEIVRPDLS	945
QYU59228.1	866	MDWNELFKYPGVYVEEDGSFEVKIRSPYHRTPARLLAGQSQRDMSSLNFYAIAGPIAPSGETARLPIVVQIDEIVHPDLS	945
AGN29358.1	866	MDWNELFKYPGVNVEEDGSFEVKIRSPYHRTPARLLAGQSQRDMSSLNFYANAGPIAPSGETARLPIVVQIDEIVRPDLS	945
ARO69944.1	866	MDWNELFKYPGVYVEEDGSFEVKIRSPYHRTPARLLAGQSQRDMSSLNFYAIAGPIAPSGETARLPIVVQIDEIVRPDLS	945
AGN29367.1	863	MDWNELFKYPGVYVEEDGSFEVKIRSPYHRTPARLLAGQSQRDMSSLNFYAIAGPIAPSGETAQLPIVVQIDEIVRPDLS	942
ABI63921.1	866	MDWNELFKYPGVYVEEDGSFEVKIRSPYHRTPARLLVGQSQRDMSSLNFYAIAGPIAPSGETARLPIVVQIDEIVRPDLS	945
ABI63923.1	866	MDWNELFKYPGVYVEEDGSFEVKIRSPYHRTPARLLVGQSQRDMSSLNFYAIAGPIAPSGETARLPIVVQIDEIVRPDLS	945
CDN67470.1	863	MDWNELFKYPGVYVEEDGSFEVKIRSPYHRTPARLLAGQSQRDMSSLNFYAIAGPIAPSGETARLPIVVQIDEIVRPDLS	942
CDN67474.1	866	MDWNELFKYPGVYVEEDGSFEVKIRSPYHRTPARLLASQSQRDMSSLNFYAIAGPIAPSGETARLPIVVQIDEIVRPDLS	945
AAX18710.1	866	MDWNELFKYPGVYVEEDGSFEVKIRSPYHRTPARLLAGQSQRDMSSLNFYAIAGPIAPSGETARLPIVVQIDEIVRPDLS	945
QBZ78370.1	864	MDWNELFKYPGVYVEEDGSFEVKIRSPYHRTPARLLAGQSQRDMSSLNFYAIAGPIAPSGETARLPIVVQIDEIVHPDLS	943
ARO69923.1	866	MDWNELFKYPGVYVEEDGNFEVKIRSPYHRTPARLLAGQSQRDMSSLNFYAIAGPIAPSGETARLPIVVQIDEIVRPDLS	945
ARO69907.1	866	MDWNELFKYPGVYVEEDGNFEVKIRSPYHRTPARLLAGQSQRDMSSLNFYAIAGPIAPSGETARLPIVVQIDEIVRPDLS	945
ARO69942.1	866	MDWNELFKYPGVYVEEDGNFEVKIRSPYHRTPARLLAGQSQRDMSSLNFYAIAGPIAPSGETARLPIVVQIDEIVRPDLS	945
ARO69909.1	866	MDWNELFKYPGVYVEEDGSFEVKIRSPYHRTPARLLAGQSQRDMSSLNFYAIAGPIAPSGETARLPIVVQIDEIVRPDLS	945
AAX18711.1	866	MDWNELFKYPGVYMEEDGSFEVKIRSPYHRTPARLLAGQSQRDMSSLNFYAIAGPIAPSGETARLPIVIQIDEIVRPDLA	945
ARO69936.1	866	MDWNELFKYPGVYVEEDGNFEVKIRSPYHRTPARLLAGQSQRDMSSLNFYAIAGPIAPSGETARLPIVVQIDEIVRPDLS	945
AGN29357.1	866	MDWNELFKYPGVYVEEDGSFEVKIRSPYHRTPARLLAGQSQRDMSSLNFYAIAGPIAPSGETARLPIVVQIDEIVRPDLS	945
ARO69919.1	866	MDWNELFKYPGVYVEEDGSFEVKIRSPYHRTPARLLAGQSQRDMSSLNFYAIAGPIAPSGETARLPIVVQIDEIVRPDLS	945
ADJ10929.1	866	MDWNELFKYPGVYVEEDGSFEVKIRSPYHRTPARLLAGQSQRDMSSLNFYAIAGPIAPSGETARLPIVVQIEEIVRPDLS	945
ABI63943.1	866	MDWNELFKYPGVYVEEDGSFEVKIRSPYHRTPARLLVGQSQRDMSSLNFYAIAGPIAPSGETARLPIVVQIDEIVRPDLS	945
AGN29365.1	866	MDWNELFKYPGVCVEEDGSFEVKIRSPYHRTPARLLAGQSQRDMSSLNFYAIAGPIAPSGETARLPIVVQIDEIVRPDFS	945
ABI63930.1	865	MDWNELFKYPGVYVEEDGSFEVKIRSPYHRTPARLLAGQSQRDMSSLNFYAIAGPITPAGETARLPVVVQIEEIVRPDLS	944
ABI63942.1	865	MDWNELFRYPGVYVEEDGSFEVKIRSPYHRTPARLLAGQSQRDMSSLNFYAIAGPIAPAGETARLPVVVQIEEIIRPDLS	944
ADJ10939.1	866	MDWNELFKYPGVYVEEDGSFEVKIRSPYHRTPARLLAGQSQRDMSSLNFYAIAGPIAPSGETARLPIVVQIGEIVRPDFS	945
ABI63928.1	865	MDWNELFKYPGVYVEEDGSFEVKIRSPYHRTPARLLAGQSQRDMSSLNFYAIAGPIAPAGETARLPVVVQIEEIVRPDLS	944
CDN67476.1	863	MDWNELFKYPGVYVEEDGSFEVKIRSPYHRTPARLLAGQSQRDMSSLNFYAIAGPIAPSGETARLPIVVQIDEIVRPDFS	942
ABI63933.1	865	MDWNELFKYPGVYVEEDGSFEVKIRSPYHRTPARLLAGQSQRDMSSLNFYAIAGPIAPAGETARLPVVVQIEEIVRPDLS	944
AAX18707.1	866	MDWNELFKYPGVYVEEDGSFEVKVRSPYHRTPARLLAGQSQRDMSSLNFYAIAGPIAPSGETARLPIVVQIDEIVRPDLS	945
ACZ58631.1	864	MDWNELFKYPGVYVEEDGSFEVKIRSPYHRTPARLLAGQSQRDMSSLNFYAIAGPIAPSGETARLPVVVQIDEIVRPDLS	943
AGN29359.1	866	MDWNELYKYPGVYVEEDGSFEVKIRSPYHRTPARLLAGQSQRDMSSLNIYAIAGPIAPSGETARLPIVVQIDEIVRPDLS	945
AGN29361.1	866	MDWNELFKYPGVYVEEDGSFEVKIRSPYHRTPARLLAGQSQGNMSSLNFYAIAGPIAPSGETARLPIVVQIDEIVRPDLS	945
ABI63935.1	865	MDWNELFKYPGVYVEEDGSFEVKIRSPYHRTPARLLAGQSQRDMSSLNFYAIAGPIAPAGETARLPVVVQIEEIVRPDLS	944
AGN29360.1	867	MDWNELFKYPGVYVEEDGSFEVKIRSPYHRTPARLLAGQSQRDMSSLNFYAIAGPIAPSGETAQLPIVVQIDEIVRPDLS	946
AWO67712.1	865	MDWNELFKYPGVYVEEDGSFEVKIRSPYHRTPARLLTGQSQRDMSSLNFYAIAGPIAPSGETARLPIVVQIDEIARPDLS	944
AYA22300.1	863	MDWNELFKYPGVYVEEDGSFEVKIRSPYHRTPARLLAGQSQRDMSSLNFYAIAGPIAPSGETARLPIVVQIDEIVRPDLS	942

QBZ78373.1	863	MDWNELFKYPGVYVEEDGAFEVKIRSPYHRTPARLLANQSQRDMSSLNFYAIAGPIAPSGETARLPIVVQIDEIVRPDLS	942
AYQ93056.1	863	MDWNELFKYPGVYVEEDGSFEVKIRSPYHRTPARLLAGQSQRDMSSLNFYAIAGPIAPSGETARLPIVVQIEEIVRPDLS	942
AYQ93054.1	863	MDWNELFKYPGVYVEEDGSFEVKIRSPYHRTPARLLAGQSQRDMSSLNFYAIAGPIAPSGETARLPIVVQIEEIVRPDLS	942
AAX18709.1	863	MDWNELFKCPGVYVEEDGSFEVKIRSPYHRTPARLLAGQSQRDMSSLNFYAIAGPIAPSGETARLPIVVQIDEIVRPDLS	942
AYQ93052.1	864	MDWNELFKYPGVYVEEDGSFEVKIRSPYHRTPARLLAGQNQRDMSSLNFYAIAGPIAPSGETARLPIVVQIDEIMRPDLS	943
BCT87062.1	865	MDWNELFKYPGVYVEADGSFEVKIRSPYHRTPARLLAGQSQRDMSSLNFYAIAGPIAPSGETARLPIVVQIEEIVRPDLS	944
AGN29364.1	863	MDWNELFKYPGVYVEEDGSFEVKIRSPYNRTPARLLVGQSQRDMSSLNFYAIAGPIAPSGETARLPIVVQIDEIVRPDLS	942
QYU59234.1	863	MDWNELFKYPGVYVEEDGSFEVKIRSPYHRTPARLLAGQSQRDMSSLNFYAIAGPIAPTGETARLPIVVQIEEIVRPDLS	942
CDN67475.1	863	MDWNELFKYPGVYVEEDGSFEVKIRSPYHRTPARLLAGQSQRDMSSLNFYAIAGPIAPSGETARLPIVVQIDEIVRPDLS	942
AGN29356.1	863	MDWNELFNYPGVYVEEDGSFEVKIRSPYHRTPARLLAGQSQRDMSSLNFYAIAGPIAPSGETARLPVVVQIDEIVRPDLS	942
AGN29363.1	863	MDWNELFKYPGVYVEEDGSFEVKIRSPYNRTPARLLAGQSQRDMSSLNFYAIAGPIAPSGETARLPIVVQIDEIVRPDLS	942
AFV73369.1	866	MDWNELFKYPGVYVEEDGSFEVKIRSPYHRTPARLLAGQSQRDMSSLNFYAIAGPIAPSGETARLPIVVQIEEIVRPDLS	945
AFV34732.1	863	MDWNELFKYPGVYVEEDGSFEVKIRSPYHRTPAKLLAGQSQRDMSSLNFYAIAGPIAPSGETARLPIVVQIDEIVRPDFS	942
AFI43912.1	867	MDWNELFKYPGVYVEADGSFEVKIRSPYHRTRARLLAGQNQRDMSSLNFYAIAGPIAPSGETARLPIVVQIEEIVRPDLS	946
CDN67472.1	863	MDWNELFKYPGVYVEEDGSFEVKIRSPYHRTPARLLAGQSQRDLSSLNFYAIAGPIAPSGETARLPIVVQIDEIVRPDLS	942
AGN29355.1	863	MDWNELFKYPGVYVEEDGSFEVKIRSPYHRTPARLLAGQSQRDMSSLNFYAIAGPIAPSGETAQLPIVVQIDEIVRPDLS	942
AFI43911.1	867	MDWNELFKYPGVYVVADGSFEVKIRSPYHRTPARLLAGQNQRDMSSLNFYAIAGPIAPSGETARLPIVVQIEEIVRPDLS	946
ADJ10940.1	866	MDWNELFKYPGVYVEEDGSFEVKIRSPYHRTPARLLAGQSQRDMSSLNFYAIAGPIAPSGETARLPIVVQIEEIVRPDLS	945
DAZ85718.1	866	MDWNELFKYPGVYVEEDGSFEVKIRSPYHRTPARLLAGQSQRDMSSLNFYAIAGPIAPSGETARLPIVVQIEEIVRPDLS	945
ADJ10930.1	866	MDWNELFKYPGVYVEEDGSFEVKIRSPYHRTPARLLAGQSQRDMSSLNFYAIAGPIAPSGETARLPIVVQIEEIVRPDLS	945
ADJ10933.1	866	MDWNELFKYPGVYVEEDGSFEVKIRSPYHRTPARLLAGQSQRDMSSLNFYAIAGPIAPSGETARLPIVVQIEEIVRPDLS	945
ADJ10935.1	866	MDWNELFKYPGVYVEEDGSFEVKIRSPYHRTPARLLAGQSQRDMSSLNFYAIAGPIAPSGETARLPIVVQIEEIVRPDLS	945
ADJ10941.1	866	MDWNELFKYPGVYVEEDGSFEVKIRSPYHRTPARLLAGQSQRDMSSLNFYAIAGPIAPSGETARLPIVVQIEEIVRPDLS	945
DAZ85719.1	866	MDWNELFKYPGVYVEEDGSFEVKIRSPYHRTPARLLAGQSQRDMSSLNFYAIAGPIAPSGETARLPIVVQIDEIVRPDLS	945
ADJ10936.1	866	MDWNELFKYPGVYVEEDGSFEVKIRSPYHRTPARLLAGQSQRDMSSLNFYAIAGPIAPSGETARLPIVVQIEEIVRPDLS	945
ABR09917.1	866	MDWNELFKYPGVYVEEDGSFEVKIRSPYHRTPARLLAGQSQRDMSSLNFYAVAGPIAPSGETARLPIVVQIEEIVRPDLS	945
CDN67473.1	871	MDWNELFKYPGVYVEEDGSFEVKIRSPYHRTPARLLAGQSQRDMSSLNFYAIAGPIAPAGETARLPVVVQIEEIIRPDLS	950
QYU59232.1	863	MDWNELFKYPGVYVEEDGSFEVKIRSPYHRTPARLLTGQSQRDMSSLNFYAIAGPIAPSGETARLPIVVQIDEIVRPDLS	942
AGN29362.1	863	MDWNELFKYPGVYVEEDGSFEVKIRSPYHRTPARLLAGQSQGNMSSLNFYAIAGPIAPSGETARLPIVVQIDEIVRPDLS	942
ACZ58625.1	865	MDWNELFKYPGVYVEEDGSFEVKIRSPYHRTPARLLAGQSQRDMSSLNFYAIAGPIAPSGETARLPVVVQIDEIVRPDLS	944
ACZ58626.1	865	MDWNELFKYPGVYVEEDGSFEVKIRSPYHRTPARLLAGQSQRDMSSLNFYAIAGPIAPSGETARLPVVVQIDEIVRPDLS	944
ACZ58624.1	865	MDWNELFKYPGVYVEEDGSFEVKIRSPYHRTPARLLAGQSQRDMSSLNFYAIAGPIAPSGETARLPVVVQIDEIVRPDLS	944
AGN29366.1	866	MDWNELFKYPGVYVEEDGSFEVKIRSPYHRTPARLLAGQSQRDMSSLNFYAIAGPIAPSGETARLPIVVQIDEIVRPDFS	945
QBZ78368.1	865	MDWNELFKYPGVYVEEDGSFEVKIRSPYHRTPAKLLAGQSQRDMSSLNFYAIAGPIAPSGETARLPIVVQIDEIVRPDLS	944
AFI43914.1	874	MDWNELFKYPGVYVEADGSFEVKIRSPYHRTPARLLAGQSQRDMSSLNFYAIAGPIAPSGETARLPIVVQIEEIVRPDLS	953
AFI43913.1	874	MDWNELFKYPGVYVEADGSFEVKIRSPYHRTPARLLAGQSQRDMSSLNFYAIAGHIAPSGETARLPIVVQIEEIVRPDLS	953
AFV34756.1	805	MDWNELFKYPGVYVEEDGSFEVKIRSPYHRTPAKLLAGQSQRDMSSLNFYAIAGPIAPSGETARLPIVVQIDEIVRPDLS	884
QYU59231.1	865	MDWNELFKYPGVYVEEDGSFEVKIRSPYHRTPARLLAGQSQRDMSSLNFYAIAGPIAPSGETARLPIVVQIDEIVRPDLS	944
QYU59233.1	872	MDWNELFKYPGVYVEEDGSFEVKIRSPYHRTPARLLAGQSQRDMSSLNFYAIAGPIAPSGETARLPIVVQIDEIVRPDLS	951
APD26307.1	874	TDWNELFKYPGVYVEADGSFEVKIRSPYHRTPARLLAGQNQRDMSSLNFYAIAGPIAPSGETARLPIVVQIEEIVRPDLS	953
QBZ78371.1	694	MDWNELFKYPGVYVEADGSFEVKIRSPYHRTPARLLAGQSQRDMSSLNFYAIAGPIAPSGETARLPIVVQIEEIVRPDLS	773

NP_619706.1	945	LPSFEDDYFVWVDFSEFTLDKEEIEIGSRFFDFTSNTCRVSMGENPFAAMIACHGLHSGVLDLKLQWSLNTEFGKSSGSV	1024
AWO68043.1	946	LPSFEDDYFVWVDFSEFTLDKEEIEIGSRFFDFTSNTCRVSMGENPFAAMIACHGLHSGVLDLKLQWSLNTEFGKSSGSV	1025
ADJ10937.1	946	LPSFEDDYFVWVDFSEFTLDKEEIEIGSRFFDFTSNTCRVSMGENPFAAMIACHGLHSGVLDLKLQWSLNTEFGKSSGSV	1025
ADJ10931.1	946	LPSFEDDYFVWVDFSEFTLDKEEIEIGSRFFDFTSSTCKVSMGENPFAAMIACHGLHSGVLDLKLQWSLNTEFGKSSGSV	1025
ADJ10938.1	946	LLSLEDDYFVWVDFSEFTLDKEEIEIGSRFSDFTSSTCRVSMGENPFAAMIACHGLHSGVLDLKLQWSLNTEFGKSSGSV	1025
QYU59229.1	946	LPSFEDDYFVWVDFSEFTLDKEEIEIGSRFFDFTSNTCRVSMGENPFAAMIACHGLHSGVLDLKLQWSLNTEFGKSSGSV	1025
ACZ58630.1	946	LPSFGDDYFVWVDFSEFTLDKEEIEIGSRFFDFTSNTCRVSMGENPFAAMIACHGLHSGVLDLKLQWSLNTEFGKSSGSV	1025
QGP72535.1	946	LPSFEDDYFVWVDFSEFTLDKEEIEIGSRFFDFTSNTCRVSMGENPFAAMIACHGLHSGVLDLKLQWSLNTEYGKSSGSV	1025
Q91HK5.1	946	LPSFEDDYFVWVDFSEFTLDKEELEIGSRFFDFTSSTCRVIMGENPFAAMIACHGLHSGVLDLKLQWSLNTDFGKSSGSV	1025
ARO69912.1	946	LPSFEDDYFVWVDFSEFTLDKEEIEIGSRFFDFTSNTCRVSMGENPFAAMIACHGLHSGVLDLKLQWSLNTEFGKSSGSV	1025
AFI71814.1	946	LPSFEDDYFVWVDFSEFTLDKEEIEIGSRFFDFTSSTCRVSMGENPFAAMIACHGLHSGVLDLKLQWSLNTEFGKSSGSV	1025
ADJ10932.1	946	LPSFEGGYFVWVDFSEFTLDKEEIEIGSRFFDFTSSTCRVSMGENPFAAMIACHGLHSGVLDLKLQWSLNTEFGKSSGSV	1025
ARO69925.1	946	LPSFEDDYFVWVDFSEFTLDKEEIEIGSRFFDFTSNTCRVSMGENPFAAMIACHGLHSGVLDLKLQWSLNTEFGKSSGSV	1025
ARO69911.1	946	LPSFEDDYFVWVDFSEFTLDKEEIEIGSRFFDFTSNTCRVSMGENPFAAMIACHGLHSGVLDLKLQWSLNTEFGKSSGSV	1025
ACZ58628.1	946	LPSFEDDYFVWVDFSEFTLDKEELEIGSRFFDFTSNTCRVSMGENPFAAMIACHGLHSGVLDLKLQWSLNTEFGKSSGSV	1025
ABI63932.1	946	LPSFEDDYFVWVDFSEFTLDKEEIEIGSRFFDFTSRTCRVSMGENPFAAMIACHGLHSGVLDLKFQWSLNTEFGKSSGSV	1025
ARO69945.1	946	LPSFEDDYFVWVDFSEFTLDKEEIEIGSRFFDFTSNTCRVSMGENPFAAMIACHGLHSGVLDLKLQWSLNTEFGKSSGSV	1025
ARO69930.1	946	LPSFEDDYFVWVDFSEFTLDKEEIEIGSRFFDFTSNTCRVSMGENPFAAMIACHGLHSGVLDLKLQWSLNTEFGKSSGSV	1025
ABI63925.1	946	LPSFEDDYFVWVDFSEFTLDKEEIEIGSRFFDFTSRTCRVSMGENPFAAMIACHGLHSGVLDLKFQWSLNTEFGKSSGSV	1025
ARO69906.1	946	LPSLEDDYFVWVDFSEFTLDKEEIEIGSRFFDFTSNTCRVSMGENPFAAMIACHGLHSGVLDLKLQWSLNTEFGKSSGSV	1025
ACZ58627.1	946	LPSFEDDYFVWVDFSEFTLDKEELEIGSRFFDFTSNTCRVSMGENPFAAMIACHGLHSGVLDLKLQWSLNTEFGKSSGSV	1025
ARO69920.1	946	LPSFEDDYFVWVDFSEFTLDKEEIEIGSRFFDFTSNTCRVSMGENPFAAMIACHGLHSGVLDLKLQWSLNTEFGKSSGSV	1025
ACZ58629.1	946	LPSFEDDYFVWVDFSEFTLDKEELEIGSRFFDFTSNTCRVSMGENPFAAMIACHGLHSGVLDLKLQWSLNTEFGKSSGSV	1025
AGT42201.1	946	LPSFEDDYFVWVDFSEFTLDKEEIEIGSRFFDFTSSTCRVSMGENPFAAMIACHGLHSGVLDLKLQWSLNTEFGKSSGSV	1025
ARO69914.1	946	LPSFEDDYFVWVDFSEFTLDKEEIEIGSRFFDFTSNTCRVSMGENPFAAMIACHGLHSGVLDLKLQWSLNTEFGKSSGSV	1025
ABI63916.1	946	LPSFEDDYFVWVDFSEFTLDKEEIEIGSRFFDFTSRTCRVSMGENPFAAMIACHGLHSGVLDLKFQWSLNTEFGKSSGSV	1025
ARO69918.1	946	LPSFEDDYFVWVDFSEFTLDKEEIEIGSRFFDFTSNTCRVSMGENPFAAMIACHGLHSGVLDLKLQWSLNTEFGKSSGSV	1025
ARO69948.1	946	LPSFEDDYFVWVDFSEFTLDKEEIEIGSRFFDFTSNTCRVSMGENPFAAMIACHGLHSGVLDLKFQWSLNTEFGKSSGSV	1025
QYA72353.1	946	LPSFEDDYFVWVDFSEFTLDKEEIEIGSRFFDFTSNTCRVIMGENPFAAMIACHGLHSGVLDLKLQWSLNTDFGKSSGSV	1025
ABI63917.1	946	LPSFEDDYFVWVDFSEFTLDKEEIEIGSRFFDFTSRTCRVSMGENPFAAMIACHGLHSGVLDLKFQWSLNTEFGKSSGSV	1025
ARO69941.1	946	LPSFEDDYFVWVDFSEFTLDKEEIEIGSRFFDFTSNTCRVSLGENPFAAMIACHGLHSGVLDLKFQWSLNTEFGKSSGSV	1025
ABI63926.1	946	LPSFEDDYFVWVDFSEFTLDKEEIEIGSRFFDFTSRTCRVSMGENPFAAMIACHGLHSGVLDLKFQWSLNTEFGKSSGSV	1025
CDN67471.1	946	LPSFEDDFFVWVDFSEFTLDKEEIEIGSRFFDFTSSTCRVSMGENPFAAMIACHGLHSGVLDLKLQWSLNTDFGKSSGSV	1025
ABI63920.1	946	FPSFEDDYFVWVDFSEFTLDKEEIEIGSRFFDFTSRTCRVSMGENPFAAMIACHGLHSGVLDLKFQWSLNTEFGKSSGSV	1025
ABI63927.1	946	LPSFEDDYFVWVDFSEFTLDKEEIEIGSRFFDFTSRTCRVSMGENPFAAMIACHGLHSGVLDLKFQWSLNTEFGKSSGSV	1025
ARO69908.1	946	LPSFEDDYFVWVDFSEFTLDKEEIEIGSRFFDFTSNTCRVSLGENPFAAMIACHGLHSGVLDLKFQWSLNTEFGKSSGSV	1025
ARO69916.1	946	LPSFEDDYFVWVDFSEFTLDKEEIEIGSRFFDFTSNTCRVSLGENPFAAMIACHGLHSGVLDLKFQWSLNTEFGKSSGSV	1025
ARO69910.1	946	LPSFEDDYFVWVDFSEFTLDKEEIEIGSRFFDFTSNTCRVSLGENPFAAMIACHGLHSGVLDLKFQWSLNTEFGKSSGSV	1025
DAZ85720.1	946	LPSFEDDYFVWVDFSEFTLDKEEIEIGSRFFDFTSNTCRVSLGENPFAAMIACHGLHSGVLDLKFEWSLNTEFGKSSGSV	1025
AAX18708.1	946	LPSFEDDYFVWVDFSEFTLDKEEIEIGSRFFDFTSSTCRVSMGENPFAAMIACHGLHSGVLDLKFQWSLNTEFGKSSGSV	1025
AYA22298.1	946	LPSFEDDYFVWVDFSEFTLDKEEIEIGSRFFDFTSSTCRVSMGENPFAAMIACHGLHSGVLDLKFQWSLNTEFGKSSGSV	1025
ABI63939.1	946	LPSFEDDYFVWVDFSEFTLDKEEIEIGSRFFDFTSRTCRVSMGENPFAAMIACHGLHSGVLDLKFQWSLNTEFGKSSGSV	1025
ARO69926.1	946	LPSFEDDYFVWVDFSEFTLDKEEIEIGSRFFDFTSNTCRVSLGENPFAAMIACHGLHSGVLDLKFQWSLNTEFGKSSGSV	1025
QGP72537.1	946	LPSLEDDYFVWVDFSEFTLDKEEIEIGSRFFDFTSNTCKVSMGENPFAAMIACHGLHSGVLDLKLQWSLNTEFGKSSGSI	1025
ARO69947.1	946	LPSFEDDYFVWVDFSEFTLDKEEIEIGSRFFDFTSNTCRVSLGENPFAAMIACHGLHSGVLDLKFQWSLNTEFGKSSGSV	1025

```
ARO69921.1   946   LPSFEDDYFVWVDFSEFTLDKEEIEIGSRFFDFTSNTCRVSLGENPFAAMIACHGLHSGVLDLKFQWSLNTEFGKSSGSV  1025
ARO69946.1   946   LPSFEDDYFVWVDFSEFTLDKEEIEIGSRFFDFTSNTCRVSLGENPFAAMIACHGLHSGVLDLKFQWSLNTEFGKSSGSV  1025
QGP72536.1   946   LPSFEDDYFVWVDFSEFTLDKEEIEIGSRFFDFTSNTCRVIMGENPFAAMIACHGLHSGVLDLKLQWSLSTEFGKSSGSV  1025
QGP72538.1   946   LPSLEDDYFVWVDFSEFTLDKEEIEIGSRFFDFTSNTCKVSMGENPFAAMIACHGLHSGVLDLKLQWSLNTEFGKSSGSI  1025
ARO69913.1   946   LPSFEDDYFVWVDFSEFTLDKEEIEIGSRFFDFTSNTCRVSLGENPFAAMIACHGLHSGVLDLKFQWSLNTEFGKSSGSV  1025
ARO69924.1   946   LPSFEDDYFVWVDFSEFTLDKEEIEIGSRFFDFTSNTCRVSLGENPFAAMIACHGLHSGVLDLKFQWSLNTEFGKSSGSV  1025
ABI63924.1   946   LPSFEDDYFVWVDFSEFTLDKEEIEIGSRFFDFTSRTCRVSMGENPFAAMIACHGLHSGVLDLKFQWSLNTEFGKSSGSV  1025
ARO69931.1   946   LPSFEDDYFVWVDFSEFTLDKEEIEIGSRFFDFTSNTCRVSLGENPFAAMIACHGLHSGVLDLKFQWSLNTEFGKSSGSV  1025
AWO67711.1   945   LQSFEDDYFVWVDFSEFTLDKEEIEIGSRFFDFTSNTCKVSMGENPFAAMIACHGLHSGVLDLKLQWSLNTEFGKSSGSV  1024
ARO69943.1   946   LPSFEDDYFVWVDFSEFTLDKEEIEIGSRFFDFTSNTCRVSLGENPFAAMIACHGLHSGVLDLKFQWSLNTEFGKSSGSV  1025
ADJ10928.1   946   LPSFEDDYFVWVDFSEFTLDKEEIEIGSRFSDFTSSTCRVSMGENPFAAMVACHGLHSGILDLKLQWSLNTEFGKSSGSV  1025
ARO69939.1   946   LPSFEDDYFVWVDFSEFTLDKEEIEIGSRFFDFTSNTCRVSLGENPFAAMIACHGLHSGVLDLKFQWSLNTEFGKSSGSV  1025
ABI63938.1   946   LPSFEDDYFVWVDFSEFTLDKEEIEIGSRFFDFTSRTCRVSMGENPFAAMIACHGLHSGVLDLKFQWSLNTEFGKSSGSV  1025
ABI63940.1   946   LPSFEDDYFVWVDFSEFTLDKEEIEIGSRFFDFTSRTCRVSMGENPFAAMIACHGLHSGVLDLKFQWSLNTEFGKSSGSV  1025
ABI63929.1   946   LPSFEDDYFVWVDFSEFTLDKEEIEIGSRFFDFTSRTCRVSMGENPFAAMIACHGLHSGVLDLKFQWSLNTEFGKSSGSV  1025
ABI63919.1   946   LPSFEDDYFVWVDFSEFTLDKEEIEIGSRFFDFTSRTCRVSMGENPSAAMIACHGLHSGVLDLKFQWSLNTEFGKSSGSV  1025
ADJ10934.1   944   LPSFEEDYFVWVDFSEFTLDKEEIEIGSRFFDFTSNTCKVSMGENPFAAMIACHGLHSGILDLKLQWSLNTEFGKSSGSV  1023
ARO69937.1   946   LPSFEDDYFVWVDFSEFTLDKEEIEIGSRFFDFTSNTCRVSLGENPFAAMIACHGLHSGVLDLKFQWSLNTEFGKSSGSV  1025
ARO69928.1   946   LPSFEDDYFVWVDFSEFTLDKEEIEIGSRFFDFTSNTCRVSLGENPFAAMIACHGLHSGVLDLKFQWSLNTEFGKSSGSV  1025
ARO69927.1   946   LPSFEDDYFVWVDFSEFTLDKEEIEIGSRFFDFTSNTCRVSLGENPFAAMIACHGLHSGVLDLKFQWSLNTEFGKSSGSV  1025
ABI63934.1   945   LPSFEDDYFVWVDFSEFTLDKKEIEIGSRFFDFTSRTCRVSMGENPFAAMIACHGLHSGVLDLKFQWSLNTEFGKSSGSV  1024
ARO69935.1   946   LPSFEDDYFVWVDFSEFTLDKEEIEIGSRFFDFTSNTCRVSLGENPFAAMIACHGLHSGVLDLKFQWSLNTEFGKSSGSV  1025
ARO69940.1   946   LPSFEDDYFVWVDFSEFTLDKEEIEIGSRFFDFTSNTCRVSMGENPFAAMIACHGLHSGVLDLKFQWSLNTEFGKSSGSV  1025
ARO69933.1   946   LPSFEDDYFVWVDFSEFTLDKEEIEIGSRFFDFTSNTCRVSMGENPFAAMIACHGLHSGVLDLKFQWSLNTEFGKSSGSV  1025
ABI63922.1   946   LPSFEDDYFVWVDFSEFTLDKEEIEIGSRFFDFTSRTCRVSMGENPFAAMIACHGLHSGVLDLKFQWSLNTEFGKSSGSV  1025
ABI63936.1   946   LPSFEDDYFVWVDFSEFTLDKEEIEIGSRFSDFTSRTCRVSMGENPFAAMIACHGLHSGVLDLKFQWSLNTEFGKSSGSV  1025
ABI63918.1   946   LPSFEDDYFVWVDFSEFTLDKEEIEIGSRFFDFTSRTCRVSMGENPFAAMIACHGLHSGVLDLKFQWSLNTEFGKSSGSV  1025
ABI63931.1   945   LPSFEDDYFVWVDFSEFTLDKEEIEIGSRFFDFTSNTCRVSMGENPFAAMIACHGLHSGILDLKLQWSLNTEFGKSSGSV  1024
QYU59230.1   946   LPSFEDEYFVWVDFSEFTLDKEEIEIGSRFFDFTSNTCKVFMGENPFAAMIACHGLHSGVLDLKFQWSLNTEFGKSSGSI  1025
ARO69922.1   946   LPSFEDDYFVWVDFSEFTLDKEEIEIGSRFFDFTSKTCRVSMGENPFAAMIACHGLHSGVLDLKFQWSLNTEFGKSSGSV  1025
ABI63941.1   946   LPSFEDDYFVWVDFSEFTLDKEEIEIGSRFFDFTSRTCRVSMGENPFAAMIACHGLHSGVLDLKFQWSLNTEFGKSSGSV  1025
ARO69949.1   946   LPSFEDDYFVWVDFSEFTLDKEEIEIGSRFFDFTSNTCRVSLGENPFAAMIACHGLHSGVLDLKFQWSLNTEFGKSSGSV  1025
ARO69917.1   946   LPSFEDDYFVWVDFSEFTLDKEEIEIGSRFFDFTSNTCRVSLGENPFAAMIACHGLHSGVLDLKFQWSLNTEFGKSSGSV  1025
ARO69915.1   946   LPSFEDDYFVWVDFSEFTLDKEEIEIGSRFFDFTSNTCRVSMGENPFAAMIACHGLHSGVLDLKFQWSLNTEFGKSSGSV  1025
QYU59228.1   946   LPSFEDDYFVWVDFSEFTLDKEEIEIGSRFFDFTSSTCRVSMGENPFAAMIACHGLHSGVLDLKFQWSLNTEFGKSSGSI  1025
AGN29358.1   946   LPSFEDDYFVWVDFSEFTLDKEEIEIGSRFFDSTSSTCRVIKGENPFAAMIACHGLHSGVLDLKLQWSLNTEFGKSSGSV  1025
ARO69944.1   946   LPSFEDDYFVWVDFSEFTLDKEEIEIGSRFFDFTSNTCRVSMGENPFAAMIACHGLHSGVLDLKFQWSLNTEFGKSSGSV  1025
AGN29367.1   943   LPSFEDDYFVWVDFSEFTLDREEIEIGSRFFDFTSNTCRVSMGENPFAAMIACHGLHSGVLDLKLQWSLNTEFGKSSGSV  1022
ABI63921.1   946   LPSFEDDYFVWVDFSEFTLDKEEIEIGSRFFDFTSRTCRVSMGENPFAAMIACHGLHSGVLDLKFQWSLNTEFGKSSGSV  1025
ABI63923.1   946   LPSFEDDYFVWVDFSEFTLDKEEIEIGSRFFDFTSRSCRVSMGENPFAAMIACHGLHSGVLDLKFQWSLNTEFGKSSGSV  1025
CDN67470.1   943   LPSFEDDYFVWVDFSEFTLDKEEIEIGSRFFDFTSNTCRVSMGENPFAAMIACHGLHSGVLDLKLQWSLNTEFGKSSGSV  1022
CDN67474.1   946   LPSFEDDYFVWVDFSEFTLDKEEIEIGSRFFDFTSNTCRVSLGENPFAAMIACHGLHSGVLDLKFQWSLNTEFGKSSGSV  1025
AAX18710.1   946   LPSFEDDYFVWVDFSEFTLDKEEIEIGSRFFDFTSNTCRVSMGENPFAAMIACHGLHSGVLDLKLQWSLNTEFGKSSGSV  1025
QBZ78370.1   944   LPSLEDDYFVWVDFSAFTLDREEIEIGSRFFDFTSSTCKVSMGENPFAAMIACHGLHSGVLDLKFQWSLNTEFGKSSGSI  1023
ARO69923.1   946   LPSFEDDYFVWVDFSEFTLDKEEIEIGSRFFDFTSNTCRVSLGENPFAAMIACHGLHSGVLDLKFQWSLNTEFGKSSGSV  1025
ARO69907.1   946   LPSFEDDYFVWVDFSEFTLDKEEIEIGSRFFDFTSNTCRVSLGENPFAAMIACHGLHSGVLDLKFQWSLNTEFGKSSGSV  1025
ARO69942.1   946   LPSFEDDYFVWVDFSEFTLDKEEIEIGSRFFDFTSNTCRVSLGENPFAAMIACHGLHSGVLDLKFQWSLNTEFGKSSGSV  1025
ARO69909.1   946   LPSFEDDYFVWVDFSEFTLDKEEIEIGSRFFDFTSNTCRVSMGENPFAAMIACHGLHSGVLDLKFQWSLNTEFGKSSGSV  1025
AAX18711.1   946   LPSFEDDYFVWVDFSEFTLDKEEIEIGSRFFDFTSSTCRVSMGGNPFAAMIACHGLHSGVLDLKFQWSLNTEFGKSSGSV  1025
ARO69936.1   946   LPSFEDDYFVWVDFSEFTLDKEEIEIGSRFFDFTSNTCRVSLGENPFAAMIACHGLHSGVLDLKFQWSLNTEFGKSSGSV  1025
AGN29357.1   946   LPSFEDDYFVWVDFSEFTLDKEEIEIGSRFFDFTSSTCRVIMGENPFAAMIACHGLHSGVLDLKLQWSLNTEFGKSSGSV  1025
ARO69919.1   946   LPSFEDDYFVWVDFSEFTLDKEEIEIGSRFFDFTSNTCRVSMGENPFAAMIACHGLHSGVLDLKFQWSLNTEFGKSSGSV  1025
ADJ10929.1   946   LPSFEEDYFVWVDFSEFTLDKEEIEIGSRFFDFTSNTCRVSMGENPFAAMIACHGLHSGILDLKLQWSLNTEFGKSSGSV  1025
ABI63943.1   946   LPSFEDDYFVWVDFSEFTLDKEEIEVGSRFFDFTSRTCRVSMGENPFAAMIACHGLHSGVLDLKFQWSLNTEFGKSSGSV  1025
AGN29365.1   946   LPSFEDDYFVWVDFSEFTLDREEIEIGSRFFDFTSNTCRVSMGENPFAAMIACHGLHSGVLDLKLQWSLNTEFGKSSGSI  1025
ABI63930.1   945   LPSFEDDYFVWVDFSEFTLDKEEIEIGSRFFDFTSNTCRVSMGENPFAAMIACHGLHSGILDLKLQWSLNTEFGKSSGSV  1024
ABI63942.1   945   LPSFEDDYFVWVDFSEFTLDKEEIEIGSRFFDFTSNTCRVSMGENPFAAMIACHGLHSGILDLKLQWSLNTEFGRSSGSV  1024
ADJ10939.1   946   LLSLEDDYFVWVDFSEFTLDKEEIEIGSRFSDFTSSTCSVSMGENPFAAVIACHGLHSGVLGLKLQWSLNTEFGKSSGSV  1025
ABI63928.1   945   LPSFEDDYFVWVDFSEFTLDKEEIEIGSRFFDFTSNTCRVSMGENPFAAMIACHGLHSGILDLKLQWSLNTEFGKSSGSV  1024
CDN67476.1   943   LSSFEDDYFVWVDFSEFTLDKEEIEIGSRFFDFTSNTCRVSMGENPFAAMIACHGLHSGVLDLKLQWSLNTEFGKSSGSV  1022
ABI63933.1   945   LPSFEDDYFVWVDFSEFTLDKEEIEIGSRFFDFTSNTCRVSMGENPFAAMIACHGLHSGILDLKLQWSLNTEFGKSSGSV  1024
AAX18707.1   946   LPSFEDDYFVWVDFSEFTLDKEEIEIGSRFFDFTSNTCRVSMGENPFAAMIACHGLHSGVLDLKFQWSLNTEFGKSSGSV  1025
ACZ58631.1   944   LPSFEDDYFVWVDFSEFTLDKEEIEIGSRFFDFTSSTCRVSMGENPFAAMIACHGLHSGVLDLKFQWSLNTEYGKSSGSV  1023
AGN29359.1   946   LPSFEDDYFVWVDFSEFTLDQEEIEIGSRFIDFTSSTCRVIMGENPFAAMIACHGLHSGVLDLKLQWRLNTEFGKSSGSV  1025
AGN29361.1   946   LPSFGDDYFVWVDFSEFTLDREEIEIGSRFFDFTSNTCRVSMGENPFAAMIACHGLHSGVLDLKLQWSLNTEFGKSSGSV  1025
ABI63935.1   945   LPSFEDDYFVWVDFSEFTLDKEEIEIGSRFFDSTSNTCRVSMGENPFAAMIACHGLHSGILDLKLQWSLNTEFGKSSGSV  1024
AGN29360.1   947   LPSFEDDYFVWVDFSEFTLDREEIEIGSRFFDFTSNTCRVSMGENPFAAMIACHGLHSGVLDLKLQWSLNTEFGKSSGSV  1026
AWO67712.1   945   LPSFEDDYFVWVDFSEFTLDKEEIEIGSRFFDFTSSTCRVSMGENPFAAMVACHGLHSGVLDLKLQWSLNTEFGKSSGSV  1024
AYA22300.1   943   LPSFEDDYFVWVDFSEFTLDREEIEIGSRFFDFTSNTCKVSLGENPFAAMIACHGLHSGVLDLKFQWSLNTEFGKSSGSV  1022
QBZ78373.1   943   LPSFEDDYFVWVDFSEFTLDKEEIEIGSRFFDFTSNTCRVSMGENPFAAMIACHGLHSGVLDLKFQWSLNTEFGKSSGSV  1022
AYQ93056.1   943   LPSFEDDYFVWVDFSEFTLDKEEIEIGSRFFDFTSNTCKVSMGENPFAAMIACHGLHSGVLDLKLQWSLNTEFGKSSGSV  1022
AYQ93054.1   943   LPSFEDDYFVWVDFSEFTLDKEEIEIGSRFFDFTSNTCKVSMGENPFAAMIACHGLHSGVLDLKLQWSLNTEFGKSSGSV  1022
AAX18709.1   943   LPSFEDDYFVWVDFSEFTLDREEIEIGSRFFDFTSNTCKVSMGENPFAAMIACHGLHSGVLDLKFQWSLNTEFGKSSGSV  1022
AYQ93052.1   944   LPSFEDDYFVWVDFSEFTLDKEEIEIGSRFFDFTSSTCRVSMGENPFAAMIACHGLHSGVLDLKLQWSLNTEFGKSSGSV  1023
BCT87062.1   945   LPSFEDDYFVWVDFSEFTLDKEEIEIGSRFFDFTSNTCKVSMGENPFAAMIACHGLHSGILDLKFQWSLNTEFGKSSGSV  1024
AGN29364.1   943   LPSFEDDYFVWVDFSEFTLDKEEIEIGSRFFDFTSSTCRVSMGENPFAAMIACHGLHSGVLDLKFQWSLNTEFGKSSGSV  1022
QYU59234.1   943   LPSFEDDYFVWVDFSEFTLDKEEIEIGSRFFDFTSNTCKVAMGENPFAAMIACHGLHSGILDLKLQWGLKTEFGKSSGSI  1022
CDN67475.1   943   LPSFEDDYFVWVDFSEFTLDKEEIEIGSRFFDFTSSTCRVSMGENPFAAMIACHGLHSGVLDLKLQWSLSTEFGKSSGSI  1022
AGN29356.1   943   LPSFEDDYFVWVDFSEFTLDKEELEIGSRFFDFTSSTCRVSMGENPFAAMIACHGLHNGVLDLKLQWSLNTEFGQSSGSV  1022
AGN29363.1   943   LSSFEDDYFVWVDFSEFTLDKEEIEIGSRFFDFTSSTCRVSMGENPFAAMIACHGLHSGVLDLKFQWSLNTEFGKSSGSV  1022
AFV73369.1   946   IPSFEDDYFVWVDFSEFTLDKEEIEIGSRFFDFTSSTCRVSMGENPFAAMIACHGLHSGVLDLKLQWSLNTEFGKSSGSV  1025
AFV34732.1   943   LPSFEDDYFVWVDFSEFTLDKEEIEIGSRFFDFTSSTCRVSMGENPFAAMIACHGLHSGVLDLKLQWSLNTEFGKSSGSV  1022
AFI43912.1   947   LPSFEDDYFVWVDFSEFTHDKEEIEIGSRFFDFTSSTCRVSMGENPFAAMIACHGLHSGILDLKFQWSLNTEFGKSSGSV  1026
CDN67472.1   943   LPSFEDDYFVWVDFSEFTLDKEEIEIGSRFFDFTSNTCRVSMGENPFAAMIACHGLHSGVLDLKFQWSLNTEFGKSSGSV  1022
AGN29355.1   943   LPSFEDDYFVWVDFSEFTLDREEIDIGSRFFDFTSNTCRVSMGENPFAAMIACHGLHSGVLDPKLSCSLITLFGKSSGFV  1022
AFI43911.1   947   LPSFEDDYFVWVDFSEFTHDKEEIEIGSRFFDFTSSTCRVSMGENPFAAMIACHGLHSGILDLKFQWSLNTEFGKSSGSV  1026
ADJ10940.1   946   LPSFEEDYFVWVDFSEFTLDKEEIEIGSRFFDFTSNTCRVSMGENPFAAMIACHGLHSGILDLKLQWSLNTEFGKSSGSV  1025
DAZ85718.1   946   LPSFEDDYFVWVDFSEFTLDKEEIEIGSRFFDFTSSTCRVSMGENPFAAMIACHGLHSGILDLKFQWSLNTEFGKSSGSV  1025
```

```
ADJ10930.1   946   LPSFEEDYFVWVDFSEFTLDKEEIEIGSRFFDFTSNTCRVSMGENPFAAMIACHGLHSGILDLKLQWSLNTEFGKSSGSV  1025
ADJ10933.1   946   LPSFEEDYFVWVDFSEFTLDKEEIEIGSRFFDFTSNTCRVSMGENPFAAMIACHGLHSGILDLKLQWSLNTEFGKSSGSV  1025
ADJ10935.1   946   LPSFEEDYFVWVDFSEFTLDKEEIEIGSRFFDFTSNTCRVSMGENPFAAMIACHGLHSGILDLKLQWSLNTEFGKSSGSV  1025
ADJ10941.1   946   LPSFEEDYFVWVDFSEFTLDKEEIEIGSRFFDFTSNTCRVSMGENPFAAMIACHGLHSGILDLKLQWSLNTEFGKSSGSV  1025
DAZ85719.1   946   LPSFGDDYFVWVDFSEFTLDKEEIEIGSRFFDFTSNTCRVSMGENPFAAMIACHGLHSGVLDLKLQWSLTTEFGKSSGSV  1025
ADJ10936.1   946   LPSFEEDYFVWVDFSEFTLDKEEIEIGSRFFDFTSNTCRVSMGENPFAAMIACHGLHSGILDLKLQWSLNTEFGKSSGSV  1025
ABR09917.1   946   MPSFEDDYFVWVDFSEFTLDKEEIEIGSRFFDFTSSTCRVSMGENPFAAMIACHGLHSGILDLKLQWSLSAEFGKSSGSV  1025
CDN67473.1   951   LPSFEDDYFVWVDFSEFTLDKEEIEIGSRFFDFTSNTCRVSMGENPFAAMIACHGLHSGILDLKLQWSLNTEFGRSSGSV  1030
QYU59232.1   943   LPSFEDDYFVWVDFSEFTLDKEEIEIGSRFFDFTSNTCRVSMGENPFAAMIACHGLHSGVLDLKLQWSLYTEFGKSSGSV  1022
AGN29362.1   943   LPSFGDDYFVWVDFSEFTLDKEEIEIGSRFFDFTSNTCRVSMGENPFAAMIACHGLHSGVLDLKFQWSLNTEFGKSSGSV  1022
ACZ58625.1   945   LPSFEDDYFVWVDFSEFTLDKEEIEIGSRFFDFTSSTCRVSMGENPFAVMIACHGLHSGILDLKLQWSLNTEFGKSSGSV  1024
ACZ58626.1   945   LPSFEDDYFVWVDFSEFTLDKEEIEIGSRFFDFTSNTCRVSMGENPFAVMIACHGLHSGVLDLKLQWSLNTEFGKSSGSI  1024
ACZ58624.1   945   LPSFEDDYFVWVDFSEFTLDKEEIEIGSRFFDFTSSTCRVSMGENPFATMIACHGLHSGVLDLKFQWSLNTEFGKSSGSV  1024
AGN29366.1   946   LPSFEDDYFVWVDFSELTLDKEEIEIGSRFFDFTSNTCRVTMGENLFAAMIAFHGLHSSVLDLKLQWSLNTEFGKSSGSV  1025
QBZ78368.1   945   LPSFEDDYFVWVDFSEFTLDKEEIEIGSRFFDFTSSTCRVSMGENPFAAMIACHGLHSGVLDLKLQWSLNTEFGKSSGSV  1024
AFI43914.1   954   LPSFEDDYFVWVDFSEFTLDKEEIEIGSRFFDFTSSTCRVSMGENPFAAMIACHGLHSGILDLKFQWSLNAEFGKSSGSV  1033
AFI43913.1   954   LPSFEDDYFVWVDFSEFTLDKEEIEIGSRFFDFTSSTCRVSMGENPFAAMIACHGLHSGILDLKFQWSLNTEFGKSSGSV  1033
AFV34756.1   885   IPSFEDDYFVWVDFSEFTLDKEEIEIGSRFFDFTSSTCRVSMGENPLAAMIACHGLHSGVLDLKFQWSLNTEFGKSSGSV  964
QYU59231.1   945   LPSFEDDYFVWVDFSEFTLDKEEIEIGSRFFDFTSNTCRVSMGENPFAAMIACHGLHSGILDLKFQWSLSAEFGKSSGSV  1024
QYU59233.1   952   LPSFEDDYFVWVDFSEFTLDKEEIEIGSRFFDFTSNTCRVSMGENPFAAMIACHGLHSGVLDLKFQWSLSTEFGKSSGSV  1031
APD26307.1   954   MPSFEDDYFVWVDFSEFTLDNEEIEIGSRFFDFTSRTCRVSMGENPFAAMIACHGLHSGTLDLKLQWSLSDDYGKSTGSV  1033
QBZ78371.1   774   LPSFEDDYFVWVDFSEFTLDKEEIEIGSRFFDFTSNTCRVMMGENPFAAMIACHGLHSGILDLKLQWSLNTEFGKSSGSV  853


NP_619706.1  1025  TITKLVGDKAMGLDGPSHVFAIQKLEGTTELLVGNFAGANPNTRFSLYSRWMAIKLDQAKSIKVLRVLCKPRPGFSFYGR  1104
AWO68043.1   1026  TITKLVGDKAMGLDGPSHVFAIQKLEGTTELLVGNFAGANPNTRFSLYSRWMAIKLDQAKSIKVLRVLCKPRPGFSFYGR  1105
ADJ10937.1   1026  TITKLVGDKAMGLDGPSHVFAIQKLEGTTELLVGNFAGANPNTRFSLYSRWMAIKLDQAKSIKVLRVLCKPRPGFSFYGR  1105
ADJ10931.1   1026  TVTKLVGDKATGLDGPSQIFAIQKLEGVTDLLIGNFAGANPNTHFSLFSRWMAIKLDQAKSIKVLRVLCKPRPGFSFYGR  1105
ADJ10938.1   1026  TITKLVGDKATGLDGPSQIFAIQKLEGTTELLVGNFAGANPNTRFSLYSRWLAIKLDQAKSIKVLRVLCKPRPGFSFYGR  1105
QYU59229.1   1026  TITKLVGDKAMGLDGPSQVFAIQKLEGITELLIGNFAGANPNTHFSLYSRWMAIKLDQAKSIKVLRVLCKPRPGFSFYGR  1105
ACZ58630.1   1026  TVTKLVGDKAMGLDGPSQVFAIQKLEGVTDLLIGNFAGANPNTHFSLYSRWMAIKLDQAKSIKVLRVLCKPRPGFSFYGR  1105
QGP72535.1   1026  TVTKLVGDKATGLDGPSNVFAIQKLEGTTELLVGNFAGANPNTHFSLYSRWMAIKLDQAKSIKVLRVLCKPRPGFSFYGR  1105
Q91HK5.1     1026  TVTKLVGDKATGLDGPSQVFAIQKLEGVTDLLIGNFAGANPNTHFSLYSRWMAIKLDQAKSIKVLRVLCKPRPGFSFYGR  1105
ARO69912.1   1026  TVTKLVGDKATGLDGPSQIFAMQKLEGVTELLIGNFAGANPNTHFSLYSRWMAIKLDQAKSIKVLRVLCKPRPGFSFYGR  1105
AFI71814.1   1026  TITKLVGDKAMGLDGPSQVFAIQKLEGVTDLLIGNFAGANPNSHFSLYSRWMAIKLDQAKSIKVLRVLCKPRPGFSFYGR  1105
ADJ10932.1   1026  TITKLVGDKATGLVGPSQVFAIQKLEGVTELLVGNFAGANPNTHFSLYSRWMAIKLDQAKSTKVLRVLCKPRSGFSFYGR  1105
ARO69925.1   1026  TVTKLVGDKATGLDGPSQIFAMQKLEGVTDLLIGNFAGANPNTHFSLYSRWMAIKLDQAKSIKVLRVLCKPRPGFSFYGR  1105
ARO69911.1   1026  TVTKLVGDKATGLDGPSQIFAMQKLEGVTELLIGNFAGANPNTHFSLYSRWMAIKLDQAKSIKVLRVLCKPRPGFSFYGR  1105
ACZ58628.1   1026  TITKLVGDKATGLDGPSQVIAMQKLEGVTELLIGNFAGANPNTHVSLYSRWMAIKLDQAKSIKVLRVLCKPRPGFSFYGR  1105
ABI63932.1   1026  TITKLVGDKATGLDGPSQIFALQKLEGVSELLVGNFAGANPNTHFSLYSRWMAIKLDQAKSIKVLRVLCKPRPGFSFYGR  1105
ARO69945.1   1026  TVTKLVGDKATGLDGPSQIFAMQKLEGVTELLIGNFAGANPNTHFSLYSRWMAIKLDQAKSIKVLRVLCKPRPGFSFYGR  1105
ARO69930.1   1026  TVTKLVGDKATGLDGPSQIFAMQKLEGVTELLIGNFAGANPNTHFSLYSRWMAIKLDQAKSIKVLRVLCKPRPGFSFYGR  1105
ABI63925.1   1026  TITKLVGDKATGLDGPSQIFALQKLEGVSELLVGNFAGANPNTHFSLYSRWMAIKLDQAKSIKVLRVLCKPRPGFSFYGR  1105
ARO69906.1   1026  TVTKLVGDKATGLDGPSQIFAMQKLEGVTDLLIGNFAGANPNTHFSLYSRWMAIKLDQAKSIKVLRVLCKPRPGFSFYGR  1105
ACZ58627.1   1026  TITKLVGDKATGLDGPSQVIAMQKLEGVTELLIGNFAGANPNTHVSLYSRWMAIKLDQAKSIKVLRVLCKPRPGFSFYGR  1105
ARO69920.1   1026  TVTKLVGDKATGLDGPSQIFAMQKLEGVTELLIGNFAGANPNTHFSLYSRWMAIKLDQAKSIKVLRVLCKPRPGFSFYGR  1105
ACZ58629.1   1026  TITKLVGDKATGLDGPSQVIAMQKLEGVTELLIGNFAGANPNTHVSLYSRWMAIKLDQAKSIKVLRVLCKPRPGFSFYGR  1105
AGT42201.1   1026  TITKLVGDKATGLDGPSQVFAIQKLEGVTDLLIGNSAGANPNSHFSLYSRWMAIKLDQAKSIKVLRVLCKPRPGFSFYGR  1105
ARO69914.1   1026  TVTKLVGDKATGLDGPSQIFAMQKLEGVTELLIGNFAGANPNTHFSLYSRWMAIKLDQAKSIKVLRVLCKPRPGFSFYGR  1105
ABI63916.1   1026  TITKLVGDKATGLDGPSQIFALQKLEGVSELLVGNFAGANPNTHFSLYSRWMAIKLDQAKSIKVLRVLCKPRPGFSFYGR  1105
ARO69918.1   1026  TVTKLVGDKATGLDGPSQIFAMQKLEGVTELLIGNFAGANPNTHFSLYSRWMAIKLDQAKSIKVLRVLCKPRPGFSFYGR  1105
ARO69948.1   1026  TITKLVGDKATGLDGPSQVIALQRLEGVAELLIGNFAGANPNSHFSLYSRWMAIKLDQAKSIKVLRVLCKPRPGFSFYGR  1105
QYA72353.1   1026  TVTKLVGDKATGLDGPSQVFAIQKLEGTTELLIGNFAGANPNTHFSLYSRWMAIKLDRAMSIKVLRVLCKPRPGFNFYGR  1105
ABI63917.1   1026  TITKLVGDKATGLDGPSQIFALQKLEGVSELLVGNFAGANPNTHFSLYSRWMAIKLDQAKSIKVLRVLCKPRPGFSFYGR  1105
ARO69941.1   1026  TITKLVGDKVTGLDGPSQVIALQRLEGAAELLIGNFAGANPNSHFSLYSRWMAIKLDQAKSIKVLRVLCKPRPGFSFYGR  1105
ABI63926.1   1026  TITKLVGDKATGLDGPSQIFALQKLEGVSELLVGNFAGANPNTHFSLYSRWMAIKLDQAKSIKVLRVLCKPRPGFSFYGR  1105
CDN67471.1   1026  TVTKLVGDKAMGLDGPSQVFAIQKLEGSTELLIGNFAGANPNTHFSLYSRWMAIKLDQAKSIKVLRVLCKPRPGFSFYGR  1105
ABI63920.1   1026  TITKLVGDKATGLDGPSQIFALQKLEGVSELLVGNFAGANPNTHFSLYSRWMAIKLDQAKSIKVLRVLCKSRPGFSFYGR  1105
ABI63927.1   1026  TITKLVGDKATGLDGPSQIFALQKLEGVSELLVGNFAGANPNTHFSLYSRWMAIKLDQAKSIKVLRVLCKPRPGFSFYGR  1105
ARO69908.1   1026  TITKLVGDKVTGLDGPSQVIALQRLEGAAELLIGNFAGANPNSHFSLYSRWMAIKLDQAKSIKVLRVLCKPRPGFSFYGR  1105
ARO69916.1   1026  TITKLVGDKVTGLDGPSQVIALQRLEGAAELLIGNFAGANPNSHFSLYSRWMAIKLDQAKSIKVLRVLCKPRPGFSFYGR  1105
ARO69910.1   1026  TITKLVGDKATGLDGPSQVIALQRLEGVAELLIGNFAGANPNSHFSLYSRWMAIKLDQAKSIKVLRVLCKPRPGFSFYGR  1105
DAZ85720.1   1026  TITKLVGDKTTGLDGPSHVFAIQKLEGTAELLVGNFAGANPNSHFSLYSRWMAIKLDQAKSIKVLRVLCKPRPGFSFYGR  1105
AAX18708.1   1026  TVTKLVGDKATGLDGSSQIFAIQKLEGVAELLIGNFAGANPNTHFSLYSRWMAIKLDQAKSIKVLRVLCKPRPGFSFYGR  1105
AYA22298.1   1026  TVTKLVGDKATGLDGSSQIFAIQKLEGVAELLIGNFAGANPNTHFSLYSRWMAIKLDQAKSIKVLRVLCKPRPGFSFYGR  1105
ABI63939.1   1026  TITKLVGDKATGLDGPSQIFALQKLEGVSELLVGNFAGANPNTHFSLYSRWMAIKLDQAKSIKVLRVLCKPRPGFSFYGR  1105
ARO69926.1   1026  TITKLVGDKVTGLDGPSQVIALQRLEGAAELLIGNFAGANPNSHFSLYSRWMAIKLDQAKSIKVLRVLCKPRPGFSFYGR  1105
QGP72537.1   1026  TITKLVGDKATGLDGPSQVFAIQKLEGVTELLVGNFAGANPNTRFSLYSRWMAIKMDQAKSIKVLRVLCRPRPGFSFYGR  1105
ARO69947.1   1026  TITKLVGDKVTGLDGPSQVIALQRLEGAAELLIGNFAGANPNSHFSLYSRWMAIKLDQAKSIKVLRVLCKPRPGFSFYGR  1105
ARO69921.1   1026  TITKLVGDRATGLDGPSQVIALQRLEGAAELLIGNFAGANPNSHFSLYSRWMAIKLDQAKSIKVLRVLCKPRSGFSFYGR  1105
ARO69946.1   1026  TITKLVGDKVTGLDGPSQVIALQRLEGAAELLVGNFAGANPNSHFSLYSRWMAIKLDQAKSIKVLRVLCKPRPGFSFYGR  1105
QGP72536.1   1026  TITKLVGDKAMGLDGPSQVFAIQKLEGVTELLIGNFAGANPNSHFSLYSRWMAIKLDQAKSIKVLRVLCKPRPGFSFYGR  1105
QGP72538.1   1026  TITKLVGDKATGLDGPSQVFAIQKLEGVTELLVGNFAGANPNTRFSLYSRWMAIKMDQAKSIKVLRVLCRPRPGFSFYGR  1105
ARO69913.1   1026  TITKLVGDKATGLDGPSQVIALQRLEGAAELLIGNFAGANPNSHFSLYSRWMAIKLDQAKSIKVLRVLCKPRSGFSFYGR  1105
ARO69924.1   1026  TITKLVGDKATGLDGPSQVIALQRLEGAAELLIGNFAGANPNSHFSLYSRWMAIKLDQAKSIKVLRVLCKPRSGFSFYGR  1105
ABI63924.1   1026  TITKLVGDKAAGLDGPSQIFALQKLEGVSELLVGNFAGANPNTHFSLYSRWTAIKLDQAKSIKVLRVLCKPRPGFSFYGR  1105
ARO69931.1   1026  TITKLVGDKVTGLDGPSQVIALQRLEGAAELLIGNFAGANPNSHFSLYSRWMAIKLDQAKSIKVLRVLCKPRPGFSFYGR  1105
AWO67711.1   1025  TVTKLVGDKAMGLDGPSQIFAMQKLEGVTDLLIGNFAGANPNSHFSLYSRWMAIKLDQAKSIKVLRVLCKPRPGFSFYGR  1104
ARO69943.1   1026  TITKLAGDRATGLDGPSQVIALQRLEGAAELLIGNFAGANPNSHFSLYSRWMAIKLDQAKSIKVLRVLCKPRSGFSFYGR  1105
ADJ10928.1   1026  TITKLVGDKATGLDGPSQVFAIQKLEGVTELLIGNFAGANPNTHFSLYSRWMAIKLDQAKSIKVLRVLCKPRSGFSFYGR  1105
ARO69939.1   1026  TITKLVGDKVTGLDGPSQVIALQRLEGAAELLIGNFAGANPNSHFSLYSRWMAIKLDQAKSIKVLRVLCKPRPGFSFYGR  1105
ABI63938.1   1026  TITKLVGDKATGLDGPSQIFALQKLEGVSELLVGNFAGANPNTHFSLYSRWMAIKLDQAKSIKVLRVLCKPRPGFSFYGR  1105
ABI63940.1   1026  TITKLVGDKATGLDGPSQIFALQKLEGVSELLVGNFAGANPNTHFSLYSRWMAIKLDQAKSIKVLRVLCKPRPGFSFYGR  1105
ABI63929.1   1026  TITKLVGDKATGLDGPSQIFALQKLEGVSELLVGNFAGANPNTHFSLYSRWMAIKLDQAKSIKVLRVLCKPRPGFSFYGR  1105
ABI63919.1   1026  TITKLVGDKATGLDGPSQIFALQKLEGVSELLVGNFAGANPNTHFSLYSRWMAIKLDQAKSIKVLRVLCKPRPGFSFYGR  1105
ADJ10934.1   1024  TITKLVGDKAMGLDGPSQVFALQKLEGTTELLIGNFAGANPNTHFSLYSRWMAIKLDQAKSIKVLRVLCKPRPGFNFYGR  1103
ARO69937.1   1026  TITKLVGDKVTGLDGPSQVIALQRLEGAAELLIGNFAGANPNSHFSLYSRWMAIKLDQAKSIKVLRVLCKPRPGFSFYGR  1105
ARO69928.1   1026  TITKLVGDKATGLDGPSQVIALQRLEGAAELLIGNFAGANPNSHFSLYSRWMAIKLDQAKSIKVLRVLCKPRPGFSFYGR  1105
```

ARO69927.1	1026	TITKLVGDKVTGLDGPSQVIALQRLEGAAELLIGNFAGANPNSHFSLYSRWMAIKFAQAKSIKVLRVLCKPRPGFSFYGR	1105
ABI63934.1	1025	TITKLVGDKATGLDGPSQIFALQKLEGVSELLVGNFAGANPNTHFSLYSRWMAIKLDQAKSIKVLRVLCKPRPGFSFYGR	1104
ARO69935.1	1026	TITKLVGDKVTGLDGPSQVIALQRLEGAAELLIGNFAGANPNSHFSLYSRWMAIKLDQAKSIKVLRVLCKPRPGFSFYGR	1105
ARO69940.1	1026	TITKLAGDKVTGLDGPSQVIALQRLEGVAELLIGNFAGANPNSHFSLYSRWMAIKLDQAKSIKVLRVLCKPRPGFSFYGR	1105
ARO69933.1	1026	TITKLVGDKVTGLDGPSQVIALQRLEGVAELLIGNFAGANPNSHFSLYSRWMAIKLDQAKSIKVLRVLCKPRPGFSFYGR	1105
ABI63922.1	1026	TITKLVGDKATGLDGPSQIFALQKLEGVSELLVGNFAGANPNTHFSLYSRWMAIKLDQAKSIKVLRVLCKPRPGFSFYGR	1105
ABI63936.1	1026	TITKLVGDKATGLDGPSQIFALQKLEGVSELLVGNFAGANPNTHFSLYSRWMAIKLDQAKSIKVLRVLCKPRPGFSFYGR	1105
ABI63918.1	1026	TITKLVGDKATGLDGPSQIFALQKLEGVSELLVWNFAGANPNTHFSLYSRWMAIKLDQAKSIKVLRVLCKPRPGFSFYGR	1105
ABI63931.1	1025	TITKLVGDKAMGLDGPSQVFAIQKLEGTTELLIGNFAGANPNTHFSLYSRWMAIKLDQAKSIMVLRVLCKPRPGFSFYGR	1104
QYU59230.1	1026	TITKLVGDKAMGLDGPSQVFAIQKLEGATELLIGNFAGANPNTHVSLYSRWMAIKLDQAKSIKVLRVLCKPRPGFSFYGR	1105
ARO69922.1	1026	TITKLVGDKVTGLDGPSQVIALQRLEGVAELLIGNFAGANPNSHFSLYSRWMAIKLDQAKSIKVLRVLCKPRPGFSFYGR	1105
ABI63941.1	1026	TITKLVGDKATGLDGPSQIFALQKLEGVSELLVGNFAGANPNTHFSLYSRWMAIKLDQAKSIKVLRVLCKPRPGFSFYGR	1105
ARO69949.1	1026	TITKLVGDKVTGLDGPSQVIALQRLEGAAELLIGNFAGANPNSHFSLYSRWMAIKLDQAKSIKVLRVLCKPRPGFSFYGR	1105
ARO69917.1	1026	TITKLVGDKVTGLDGPSQVIALQRLEGAAELLIGNFAGANPNSHFSLYSRWMAIKLDQAKSIKVLRVLCKPRPGFSFYGR	1105
ARO69915.1	1026	TITKLVGDKVTGLDGPSQVIALQRLEGVAELLIGNFAGANPNSHFSLYSRWMAIKLDRAKSIKVLRVLCKPRPGFSFYGR	1105
QYU59228.1	1026	TITKLVGDKAMGLDGPSQVFAIQKLEGVTELLIGNFAGANPNTHFSLYSRWMAIKLDQAKSIKVLRVLCKPRPGFSFYGR	1105
AGN29358.1	1026	TITKLVGDKAMGLDGPSHVFAIQKLEGVIELLIGNFAGANPNSHFSLYSRWMAIKLDQAKSIKVLRVLCKPRPGFSFYGR	1105
ARO69944.1	1026	TITKLVGDKVTGLDGPSQVIALQRLEGVAELLIGNFAGANPNSHFSLYSRWMAIKLDQAKSIKVLRVLCKPRPGFSFYGR	1105
AGN29367.1	1023	TITKLVGDKARGLDGPSQVFAMQKLEGITDLLIGNFAGANPNTHFSLYSRWMAIKLDQAKSIKVLRVLCKPRPGFSFYGR	1102
ABI63921.1	1026	TITKLVGDKATGLDGPSQIFALQKLEGVSELLVGNFAGANPNTHFSLYGRWMAIKLVQAKSIKVLRVLCKPRPGFSFYGR	1105
ABI63923.1	1026	TITKLVGDKATGLDGPSQIFALQKLEGVSELLVGNFAGANPNTHFSLYSRWMAIKLDQAKSIKVLRVLCKPRPGFSFYGR	1105
CDN67470.1	1023	TITKLVGDKAMGLDGPSQVFAIQKLEGTTELLIGNFAGANPNTHSSLYSRWMAIKLDQAKSIKVLRILCKPRPGFSFYGR	1102
CDN67474.1	1026	TVTKLVGDKATGLDGPSQVFAIQKLEGTAELLVGNFAGANPNSHFSLYSRWMAIKLDQAKSIKVLRVLCKPRPGFSFYGR	1105
AAX18710.1	1026	TITKLAGDKAMGLDGPSQVFAIQKLEGVTDLLIGNFAGANPNTHVSLYSRWMAIKLDQAKSIKILRVLCKPRPGFSFYGR	1105
QBZ78370.1	1024	TITKLVGDKATGLDGPSQVFAIQKLEGVAELLVGNFAGANPNTHFSLYSRWMAIKMDQAKSIKLLRVLCKPRPGFSFYGR	1103
ARO69923.1	1026	TITKLVGDKVTGLDGPSQVIALQRLEGAAELLIGNFAGANPNSHFSLYSRWMAIKLDQAKSIKVLRVLCKPRPGFSFYGR	1105
ARO69907.1	1026	TITKLVGDKVTGLDGPSQVIALQRLEGAAELLIGNFAGANPNSHFSLYSRWMAIKLDQAKSIKVLRVLCKPRPGFSFYGR	1105
ARO69942.1	1026	TITKLVGDKVTGLDGPSQVIALQRLEGAAELLIGNFAGANPNSHFSLYSRWMAIKLDQAKSIKVLRVLCKPRPGFSFYGR	1105
ARO69909.1	1026	TITKLVGDKATGLDGLSQVIALQRLEGVAELLIGNFAGANPNSHFPLYSRWMAIKLDQAKSIKVLRVLCKPRPGFSFYGR	1105
AAX18711.1	1026	TVTKLVGDKATGLDGSSQVFAIQKLEGVTELLIGNFAGANPNTHFSLYSRWMAIKLDQAKSIKVLRVLCKPRPGFSFYGR	1105
ARO69936.1	1026	TITKLVGDKVTGLDGPSQVIALQRLEGAAELLIGNFAGANPNSHFSLYSRWMAIKLDQAKSIKVLRVLCKPRPGFSFYGR	1105
AGN29357.1	1026	TITKLVGDKAMGLDGPSQVFAIQKLEGVTELLIGNFAGANPNSHFSLYSRWMAIKLDQAKSIKVLRVLCKPRPGFSFYGR	1105
ARO69919.1	1026	TITKLVGDKVTGLDGPSQVIALQRLEGVAELLIGNFAGANPNSHFSLYSRWMAIKLDQAKSIKVLRVLCKPRPGFSFYGR	1105
ADJ10929.1	1026	TIAKLVGDKAMGLDGPSQVFALQKLEGTTELLIGNFAGANPNTHFSLYSRWMAIKLDQAKSIKVLRVLCKPRPGFNFYGR	1105
ABI63943.1	1026	TITKLVGDKATGLDGPSQIFALQKLEGVSELLVGNFAGANPNTHFSLYSRWMAIKLDQAKSIKVLRVLCKPRPGFSFYGR	1105
AGN29365.1	1026	TVTKLVGDKAMGLDGPSQVFAIQKLEGTTDLLIGNFAGANPNTHFSLYSRWMAIKLDQAKSIKVLRVLCKPRPGFSFYGR	1105
ABI63930.1	1025	TITKLVGDKAMGLDGPSQVFAIQKLEGTTELLIGNFAGANPNTHFSLYSRWMAIKLDQAKSIKVLRVLCKPRPGFSFYGR	1104
ABI63942.1	1025	TITKLVGDKAMGLDGPSQVFAIQKLEGTTELLIGNFAGANPNTHFSLYSRWMAIKLDQAKSIKVLRVLCKPRPGFSFYGR	1104
ADJ10939.1	1026	TVTKLVGDKATGLDGPSQIFAIQKLEGTTELLIGNFAGANPNTRFSLYSRWLAIKLDQAKSIKVLRVLCKPRPGFSFYGR	1105
ABI63928.1	1025	TITKLVGDKAMGLDGPSQVFAIQKLEGTTELLIGNFAGANPNTHFSHYSRWMAIKLDQAKSIKVLRVLCKPRPGFSFCGR	1104
CDN67476.1	1023	TITKLVGDKAMGLDGPSQVFAIQKLEGVTDLLVGNFAGANPNTHISLYSRWMAIKLDQAKSIKVLRVLCKPRPGFSFYGR	1102
ABI63933.1	1025	TITKLVGDKAMGLDGPSQVFAIRKLEGTTELLIGNFAGANPNTHFSLYSRWMAIKLDQAKSIKVLRVLCKPRPGFSFYGR	1104
AAX18707.1	1026	TITKLVGDKAMGLDGPSQVFAIQKLEGAADLLIGNFAGANPNTHFSLYSRWMAIKLDQAKSIKVLRVSCKPRPGFSFYGR	1105
ACZ58631.1	1024	TITKLVGDKATGLDGPSQIFAIQKLEGVSELLVGNFAGANPNTHFSLYSRWMAIKLDQAKSIKVLRVLCKPRPGFSFYGR	1103
AGN29359.1	1026	TITKLVGDKAMGLDGPSQVFAIQKLEGVTELLIGNFAGANPNSHFSLYSRWMAIKLHQAKSIKVLRVLCKPRPGFCFYGR	1105
AGN29361.1	1026	TITELVGDKARGLDGPSQVFAMQKLEGITDLLIGNFAGANPNTHFSLYSRWMAIKLDQAKSIKVLRVLCKPRPGFSFYGR	1105
ABI63935.1	1025	TITKLVGDKAMGLDGPSQVFAIQKLEGTTELLIGNFAGANPNTHFSLYSRWMAIKLDQAKSIKVLRVLCKPRPGFSFYGR	1104
AGN29360.1	1027	TITELVGDKARGLDGPSQVFAMQKLEGITDLLIGNFAGANPNTHFSLYSRWMAIKLDQAKSIKVLRVLCKPRPGFSFYGR	1106
AWO67712.1	1025	TITKLVGDKALGLDGPSQIFAMQKLEGVTELLIGNFAGANPNTHFSLYSRWMAIKLDQAKSIRVLRVLCKPRPGFSFYGR	1104
AYA22300.1	1023	TITKLVGDKAMGLDGPSQVFAIQKLEGAADLLIGNFAGANPNTHFSLYSRWMAIKLDQAKSIKVLRVLCKPRPGFSFYGR	1102
QBZ78373.1	1023	TITKLVGDKATGLDGPSQVFALQKLEGTAELLVGNFAGANPNTHFSLYSRWMAIKLDQAKSIKVLRVLCKPRPGFSFYGR	1102
AYQ93056.1	1023	IVTKLVGDKAMGLDGPSQIFAIQKLEGVTELLIGNFAGANPNSHFSLYSRWMAIKLDQAKSIKVLRVLCKPRPGFSFYGR	1102
AYQ93054.1	1023	IVTKLVGDKAMGLDGPSQIFAIQKLEGVTELLIGNFAGANPNSHFSLYSRWMAIKLDQAKSIKVLRVLCKPRPGFSFYGR	1102
AAX18709.1	1023	TITKLVVDKAMGLDGPSQVFAIQKLEGAADLLIGNFAGANPNTHFSLYSRWMAIKLDQAKSIKVLRVLCKPRPGFSFYGR	1102
AYQ93052.1	1024	TITKLVGDKAMGLDGPSQVFAMQKLEGVTDLLIGNFAGANPNNHFSLYSRWMAIKLDQAKSIKVLRVLCKPRPGFSFYGR	1103
BCT87062.1	1025	TITRLVGDKALGLDGPSQVFALQRLEGTAELLIGNFAGANPNSHFSLYSRWMAIKLDQAKSIKVLRVLCKPRPGFSFYGR	1104
AGN29364.1	1023	TITKLVGDKAMGLDGPSQVFAIQKLEGATDLLIGNFAGANPNSHFSLYSRWMAIKLDQAKSIKVLRVLCKPRPGFSFYGR	1102
QYU59234.1	1023	TITKLVGDKAMGLDGPSQVFAIQKLEGTTELLIGNFAGANPNTHFSLYGRWMAIKMDQAKSISVLRILCKPRPGFSFYGR	1102
CDN67475.1	1023	TITKLVGDKAMGLDGPSQVFAIQKLEGATELLIGNFAGANPNTHFSLSSGGRAIKLDQAKSIKVLRVLCKPLPGFSFYGR	1102
AGN29356.1	1023	TVTKLVGDKATGMDGPSQIFAMQKLEGATELLIGNFAGANPNTHFSLYSRWMAIKLDQAKSIKVLRVLCKPRPGFSFYGR	1102
AGN29363.1	1023	TITKLVGDKATGLDGPSQVFAMQKLEGITDLLIGNFAGANPNTHFSLYSRWMAIKLDQAKSIKVLRVLCKPRPGFSFYGR	1102
AFV73369.1	1026	TVTKLVGDKAMGLDGPSQIFAIQKLEGVTDLLIGNFAGANPNSHFSLYSRWMAIKLDQAKSIKVLRVLCKPRPGFSFYGR	1105
AFV34732.1	1023	TVTKLVGDKAMGLDGPSQIFAIQKLEGVTDLLIGNFAGANPNSHFSLYSRWMAIKLDQAKSIKVLRVLCKPRPGFSFYGR	1102
AFI43912.1	1027	TITRLVGDKALGLDGPSQVFALQRLEGTAELLIGNFAGANPNTHFSLYSRWMAIKLDQAKSMKVLRVLCKPRPGFSFYGR	1106
CDN67472.1	1023	TITKLVGDKAMGLDGPSQVFAIQKLEGAADLLIGNFAGANPNTHFSLYSRWMAIKLDQAKSIKVLRVSCKPRPGFSFYGR	1102
AGN29355.1	1023	TIKKLARDKATDLNLPSQIFAIQKLERITDLLIWNFAGANPNAHFSLYSRWMAIKLDQAKSIKVLRVLCKPRPGFSFYGR	1102
AFI43911.1	1027	TITRLVGDKALGLDGPSQVFALQRLEGTAELLIGNFAGANPNSHFSLYSRWMAIKLDQARSMKVLRVLCKPRPGFSFYGR	1106
ADJ10940.1	1026	TITKLVGDKATGLDGPSQVFALQKLEGTTELLIGNFAGANPNTHFSLYSRWMAIKLDQAKSIKVLRVLCKPRPGFNFYGR	1105
DAZ85718.1	1026	TVTKLVGDKAMGLDGPSQVFAIQKLEGATELLIGNFAGANPNTHFSLYSRWMAIKLDQAKSIKVLRVLCKPRPGFSFYGR	1105
ADJ10930.1	1026	TITKLVGDKAMGLDGPSQVFALQKLEGTTELLIGNFAGANPNTHFSLYSRWMAIKLDQAKSIKVLRVLCKPRPGFNFYGR	1105
ADJ10933.1	1026	TITKLVGDKAMGLDGPSQVFALQKLEGTTELLIGNFAGANPNTHFSLYSRWMAIKLDQAKSIKVLRVLCKPRPGFNFYGR	1105
ADJ10935.1	1026	TITKLVGDKAMGLDGPSQVFALQKLEGTTELLIGNYAGANPNTHFSLYSRWMAIKLDQAKSIKVLRVLCKPRPGFNFYGR	1105
ADJ10941.1	1026	TITKLVGDKATGLDGPSQVFALQKLEGTTELLIGNFAGANPNTHFSLYSRWMAIKLDQAKSIKVLRVLCKPRPGFNFYGR	1105
DAZ85719.1	1026	TVTQLVGDKATGLDGPSNVFAIQKLEGTTELLIGNFAGANPNSHFSLYSRWMAIKLDQAKSIKVLRVLCKPRPGFSFYGR	1105
ADJ10936.1	1026	TITKLVGDKAMGLDGPSQVFALQKLEGTTELLIGNFAGANPNTHFSLYSRWMAIKLDQAKSIKVLRVLCKPRPGFNFYGR	1105
ABR09917.1	1026	TVTKLVGDKAMGLDGPSQVFAIQKLEGTTELLIGNFAGANPNTHFSLYSRWMAIKLDQAKSIKVLRVLCKPRPGFSFYGR	1105
CDN67473.1	1031	TITKLVGDKAMGLDGPSQVFAIQKLEGTTELLIGNFAGANPNTHFSLYSRWMAIKLDQAKSIKVLRVLCKPRPGFSFYGR	1110
QYU59232.1	1023	TITKLVGDKAMGLDGPSQIFALQKLEGATELLIGNFAGANPNTHFSLYSRWMAIKLDQAKSIKVLRVLCKPRPGFSFYGR	1102
AGN29362.1	1023	TITKLEGDKATGLDGPSCVFAIQKLEGTAELLIGNFAGANPNTHFSLYSRWMAIKLDQAKSIKVLRVLCKPRPGFSFYGR	1102
ACZ58625.1	1025	IITQLVGDKATGMDGPSQIFAMQKLEGVAELLVGNFAGANPNTHFSLYSRWMAIKLDQAKSIKVLRVLCKPRPGFSFYGR	1104
ACZ58626.1	1025	TITQLVGDKATGMDGPSQIFAMQKLEGVAELLVGNFAGANPNTHCSLYSRWMAIKLDQAKSIKVLRVLCKPRPGFSFYGR	1104
ACZ58624.1	1025	TITQLVGDIATGMDGPSQIFAMQKLEGVAELLVGNFAGANPNTHFSLYSRWMAIKLDQAKSIKVLRVLCKPRPGFSFYGR	1104
AGN29366.1	1026	TITKMVGDKAMGLVGPCQVFAIQKLEGATELLIGNFAGANPNTYVSLYSRWMAIKLDQAKSIKVLRVLCKPRPGFSFYGR	1105
QBZ78368.1	1025	TITQLVGDKATGMDGPSQIFAMQKLEGVAELLVGNFAGANPNTHFSLYSRWMAIKLDQAKSIKVLRVLCKPRPGFSFYGR	1104
AFI43914.1	1034	TITRLVGDKALGLDGPSQVFALQRLEGTAELLIGNFAGANPNSHFSLYSRWMAIKLDQARSMKVLRVLCKPRPGFSFYGR	1113
AFI43913.1	1034	TITRLVGDKALGLDGPSQVFALQRLEGTAELLIGNFAGANPNSHFSLYSRWMAIKLDQARSMKVLRVLCKPRPGFSFYGR	1113
AFV34756.1	965	IVTKLVGDKAMGLDGPSQIFAIQKLEGVTDLLIGNFAGANPNSHFSLYSRWMAIKLDQAKSIKVLRVLCKPRPGFSFYGR	1044
QYU59231.1	1025	TVTKLVGDKAMGLDGPSQIFALQKLEGTAELLIGNFAGANPNTHFTLYSRWMAIKLDQAKSIKVLRVLCKPRPGFSFYGR	1104

```
QYU59233.1   1032  TITKLVGDKVMGLDGPSQIFALQKLEGTAELLVGNFAGANPNTHFSLYSRWMAIKLDQAKSIKVLRVLCKPRPGFSFYGR  1111
APD26307.1   1034  TVTKLVGDKALGLDGPSQVFAIQKLEGATELLIGNFAGANPNTYASLYSRWMAIKLDRAKSMKILRVLCKPRPGFSFYGR  1113
QBZ78371.1   854   VVTRLVGDKALGLDGPSHVFALQKLEGTAELLVGNFAGANPNTHSSLYSRWMAIKLDQAKSMKVLRVLCKPRPGFSFYGR  933


NP_619706.1  1105  TSFPV  1109
AWO68043.1   1106  TSFPV  1110
ADJ10937.1   1106  TSFPV  1110
ADJ10931.1   1106  TSFPV  1110
ADJ10938.1   1106  TSFPV  1110
QYU59229.1   1106  TSFPV  1110
ACZ58630.1   1106  TSFPV  1110
QGP72535.1   1106  TSFPV  1110
Q91HK5.1     1106  TSFPV  1110
ARO69912.1   1106  TSFPV  1110
AFI71814.1   1106  TSFPV  1110
ADJ10932.1   1106  TSFPV  1110
ARO69925.1   1106  TSFPV  1110
ARO69911.1   1106  TSFPV  1110
ACZ58628.1   1106  TSFPV  1110
ABI63932.1   1106  TSFPV  1110
ARO69945.1   1106  TSFPV  1110
ARO69930.1   1106  TSFPV  1110
ABI63925.1   1106  TSFPV  1110
ARO69906.1   1106  TSFPV  1110
ACZ58627.1   1106  TSFPV  1110
ARO69920.1   1106  TSFPV  1110
ACZ58629.1   1106  TSFPV  1110
AGT42201.1   1106  TSLPV  1110
ARO69914.1   1106  TSFPV  1110
ABI63916.1   1106  TSFPV  1110
ARO69918.1   1106  TSFPV  1110
ARO69948.1   1106  TSFPV  1110
QYA72353.1   1106  TSFPV  1110
ABI63917.1   1106  TSFPV  1110
ARO69941.1   1106  TSFPV  1110
ABI63926.1   1106  TSFPV  1110
CDN67471.1   1106  TSFPV  1110
ABI63920.1   1106  TSFPV  1110
ABI63927.1   1106  TSFPV  1110
ARO69908.1   1106  TSFPV  1110
ARO69916.1   1106  TSFPV  1110
ARO69910.1   1106  TSFPV  1110
DAZ85720.1   1106  TSFPV  1110
AAX18708.1   1106  TSFPV  1110
AYA22298.1   1106  TSFPV  1110
ABI63939.1   1106  TSFPV  1110
ARO69926.1   1106  TSFPV  1110
QGP72537.1   1106  TSFPV  1110
ARO69947.1   1106  TSFPV  1110
ARO69921.1   1106  TSFPV  1110
ARO69946.1   1106  TSFPV  1110
QGP72536.1   1106  TSFPV  1110
QGP72538.1   1106  TSFPV  1110
ARO69913.1   1106  TSFPV  1110
ARO69924.1   1106  TSFPV  1110
ABI63924.1   1106  TSFPV  1110
ARO69931.1   1106  TSFPV  1110
AWO67711.1   1105  TSFPV  1109
ARO69943.1   1106  TSFPV  1110
ADJ10928.1   1106  TSFPV  1110
ARO69939.1   1106  TSFPV  1110
ABI63938.1   1106  TSFPV  1110
ABI63940.1   1106  TSFPV  1110
ABI63929.1   1106  TSFPV  1110
ABI63919.1   1106  TSFPV  1110
ADJ10934.1   1104  TSFPV  1108
ARO69937.1   1106  TSFPV  1110
ARO69928.1   1106  TSFPV  1110
ARO69927.1   1106  TSFPV  1110
ABI63934.1   1105  TSFPV  1109
ARO69935.1   1106  TSFPV  1110
ARO69940.1   1106  TSFPV  1110
ARO69933.1   1106  TSFPV  1110
ABI63922.1   1106  TSFPV  1110
ABI63936.1   1106  TSFPV  1110
ABI63918.1   1106  TSFPV  1110
ABI63931.1   1105  TSFPV  1109
QYU59230.1   1106  TSFPV  1110
ARO69922.1   1106  TSFPV  1110
ABI63941.1   1106  TSFPV  1110
ARO69949.1   1106  TSFPV  1110
ARO69917.1   1106  TSFPV  1110
ARO69915.1   1106  TSFPV  1110
QYU59228.1   1106  TSFPV  1110
AGN29358.1   1106  TSFPF  1110
ARO69944.1   1106  TSFPV  1110
AGN29367.1   1103  TSFPV  1107
```

ABI63921.1	1106	TSFPV	1110
ABI63923.1	1106	TSFPV	1110
CDN67470.1	1103	TSFPV	1107
CDN67474.1	1106	TSFPV	1110
AAX18710.1	1106	TSFPV	1110
QBZ78370.1	1104	TSFPV	1108
ARO69923.1	1106	TSFPV	1110
ARO69907.1	1106	TSFPV	1110
ARO69942.1	1106	TSFPV	1110
ARO69909.1	1106	TSFPV	1110
AAX18711.1	1106	TSFPV	1110
ARO69936.1	1106	TSFPV	1110
AGN29357.1	1106	TSFPV	1110
ARO69919.1	1106	TSFPV	1110
ADJ10929.1	1106	TSFPV	1110
ABI63943.1	1106	TSFPV	1110
AGN29365.1	1106	TSFPV	1110
ABI63930.1	1105	TSFPV	1109
ABI63942.1	1105	TSFPV	1109
ADJ10939.1	1106	TSFPV	1110
ABI63928.1	1105	TSFPV	1109
CDN67476.1	1103	TSFPV	1107
ABI63933.1	1105	TSFPV	1109
AAX18707.1	1106	TSFPV	1110
ACZ58631.1	1104	TSFPV	1108
AGN29359.1	1106	TSFPV	1110
AGN29361.1	1106	TSFPV	1110
ABI63935.1	1105	TSFPV	1109
AGN29360.1	1107	TSFPV	1111
AWO67712.1	1105	TSFPV	1109
AYA22300.1	1103	TSFPV	1107
QBZ78373.1	1103	TSFPV	1107
AYQ93056.1	1103	TSFPV	1107
AYQ93054.1	1103	TSFPV	1107
AAX18709.1	1103	TSFPV	1107
AYQ93052.1	1104	TSFPV	1108
BCT87062.1	1105	TSFPV	1109
AGN29364.1	1103	TSFPV	1107
QYU59234.1	1103	TSFPV	1107
CDN67475.1	1103	TSFPV	1107
AGN29356.1	1103	TSFPV	1107
AGN29363.1	1103	TSLPS	1107
AFV73369.1	1106	TSFPV	1110
AFV34732.1	1103	TSFPV	1107
AFI43912.1	1107	TSFPV	1111
CDN67472.1	1103	TSFPV	1107
AGN29355.1	1103	TSFPV	1107
AFI43911.1	1107	TSFPV	1111
ADJ10940.1	1106	TSFPV	1110
DAZ85718.1	1106	TSFPV	1110
ADJ10930.1	1106	TSFPV	1110
ADJ10933.1	1106	TSFPV	1110
ADJ10935.1	1106	TSFPV	1110
ADJ10941.1	1106	TSFPV	1110
DAZ85719.1	1106	TSFLV	1110
ADJ10936.1	1106	TSFPV	1110
ABR09917.1	1106	TSFPV	1110
CDN67473.1	1111	TSFPV	1115
QYU59232.1	1103	TSFPV	1107
AGN29362.1	1103	TSFPV	1107
ACZ58625.1	1105	TSFPV	1109
ACZ58626.1	1105	TSFPV	1109
ACZ58624.1	1105	TSFPV	1109
AGN29366.1	1106	TSFPV	1110
QBZ78368.1	1105	TSFPV	1109
AFI43914.1	1114	TSFPA	1118
AFI43913.1	1114	TSFPV	1118
AFV34756.1	1045	TSFPV	1049
QYU59231.1	1105	TSFPV	1109
QYU59233.1	1112	TSFPV	1116
APD26307.1	1114	TSFPV	1118
QBZ78371.1	934	TSFPV	938
